# Identification and analysis of hub genes of hypoxia-immunity in type 2 diabetes mellitus

**DOI:** 10.3389/fgene.2023.1154839

**Published:** 2023-04-21

**Authors:** Jing Li, Ni Yan, Xiaofeng Li, Shenglin He, Xiangyou Yu

**Affiliations:** ^1^ Department of Endocrinology Diabetes, Shaanxi Provincial People’s Hospital, Xi’an, China; ^2^ Department of Rheumatology and Immunology, Shaanxi Provincial People’s Hospital, Xi’an, China

**Keywords:** type 2 diabetes, immune infiltration, WGCNA, hypoxia, hub genes

## Abstract

The chronic metabolic disease named type 2 diabetes (T2D) accounts for over 90% of diabetes mellitus. An increasing number of evidences have revealed that hypoxia has a significantly suppressive effect on cell-mediated immunity, as well as the utilization of glucose in diabetics. Therefore, we aimed to screen and identify hypoxia-immune-related hub genes in T2D through bioinformatic analysis. The Gene Expression Omnibus (GEO) database was used to get T2D gene expression profile data in the peripheral blood samples (GSE184050), and hypoxia-related genes were acquired from Molecular Signatures Database (MSigDB). Differentially expressed mRNAs (DEGs) and lncRNAs (DELs) between T2D and normal samples were identified by DeSeq2 package. The clusterProfiler package was used to perform enrichment analyses for the overlapped genes of DEGs and hypoxia-related genes. Further, Hypoxia-related hub genes were discovered using two machine learning algorithms. Next, the compositional patterns of immune and stromal cells in T2D and healthy groups were estimated by using xCell algorithm. Moreover, we used the weighted correlation network analysis (WGCNA) to examine the connection between genes and immune cells to screen immune-related genes. Gene Set Enrichment Analysis (GSEA) was used to investigate the functions of the hypoxia-immune-related hub genes. Finally, two peripheral blood cohorts of T2D (GSE184050 and GSE95849) as well as the quantitative real-time PCR (qRT-PCR) experiments for clicinal peripheral blood samples with T2D were used for verification analyses of hub genes. And meanwhile, a lncRNA-TF-mRNA network was constructed. Following the differentially expressed analysis, 38 out of 3822 DEGs were screened as hypoxia-related DEGs, and 493 DELs were found. These hypoxia-related DEGs were mainly enriched in the GO terms of pyruvate metabolic process, cytoplasmic vesicle lumen and monosaccharide binding, and the KEGG pathways of glycolysis/gluconeogenesis, pentose phosphate pathway and biosynthesis of nucleotide sugars. Moreover, 7 out of hypoxia-related DEGs were identified as hub genes. There were six differentially expressed immune cell types between T2D and healthy samples, which were further used as the clinical traits for WGCNA to identify *AMPD3* and *IER3* as the hypoxia-immune-related hub genes. The results of the KEGG pathways of genes in high-expression groups of *AMPD3* and *IER3* were mainly concentrated in glycosaminoglycan degradation and vasopressin-regulated water reabsorption, while the low-expression groups of *AMPD3* and *IER3* were mainly associated with RNA degradation and nucleotide excision repair. Finally, when compared to normal samples, both the *AMPD3* and *IER3* were highly expressed in the T2D groups in the GSE184050 and GSE95849 datasets. The result of lncRNA-TF-mRNA regulatory network showed that lncRNAs such as BACH1-IT1 and SNHG15 might induce the expression of the corresponding TFs such as TFAM and THAP12 and upregulate the expression of *AMPD3*. This study identified *AMPD3* and *IER3* as hypoxia-immune-related hub genes and potential regulatory mechanism for T2D, which provided a new perspective for elucidating the upstream molecular regulatory mechanism of diabetes mellitus.

## 1 Introduction

The World Health Organization (WHO) defines diabetes as a multietiological metabolic disorder characterized by chronic hyperglycemia and impaired metabolism of carbohydrates, fats and proteins due to defects in insulin secretion or insulin action or both. The incidence of diabetes in mainland China has increased in the past 20 years (Peng et al., 2022), and meanwhile, it is expected that the number of people with diabetes will continue to increase from 141.65 million in 2020 to 202.84 million in 2050 (Li et al., 2022a). Effective treatment of diabetes could greatly reduce deaths caused by non-communicable causes (NCD Countdown, 2030 collaborators., 2020). T2D is characterized by fasting and postprandial hyperglycemia. There are a number of treatments that can reduce hyperglycemia in T2D patients by improving insulin secretion or reducing insulin resistance in peripheral tissues. Nevertheless, complications of diabetes are prevalent worldwide, and diabetes remains a leading cause of blindness, end-stage renal disease, lower limb amputation and cardiovascular disease. Therefore, it is urgent to develop and implement new prevention and treatment strategies to address the rising incidence of type 2 diabetes worldwide.

The pathogenesis of diabetes has not been fully elucidated and hypoxia may be involved in the occurrence and development of diabetes (Wang et al., 2017). Hypoxia is inadequate or reduced oxygen supply caused by a decrease in arterial oxygen saturation. Patients with obstructive sleep apnea hypopnea syndrome (OSAHS) experience repeated hypopnea and respiratory disruption during sleep, resulting in intermittent blood oxygen partial pressure and decreased oxygen saturation. Clinically, OSAHS and type 2 diabetes often exist in the same patient. The prevalence of OSAHS in T2D patients is 24%–26% (Tahrani, 2015; Tahrani, 2017), and the risk of OSAHS is 50% higher than that of non-T2D patients (Subramanian et al., 2019). Additionally, the prevalence of diabetes in patients with OSAHS is also significantly higher than that in normal people (Foster et al., 2009). Animal studies about intermittent hypoxia (CHI) have shown that CHI-exposed mice exhibit elevated basal plasma insulin levels and insulin resistance, leading to islet beta cell dysfunction (Wang et al., 2013). Therefore, we hypothesize that hypoxia is closely related to the development of type 2 diabetes.

Current studies suggest that glucotoxicity, lipid toxicity, oxidative stress, and endoplasmic reticulum stress can induce chronic inflammation of islets (Zhou et al., 2010), contributing to impaired insulin secretion and even apoptosis of islet *β* cells, which is associated with the occurrence and development of T2D. Further, literature has shown that hypoxia plays an important role in inflammatory processes, including the regulation of neutrophil production, macrophage production and differentiation, T cell differentiation, and dendritic cell function (Hafner et al., 2017). Hypoxia can also alter transcription of inflammatory cells. Promoting the expression of inflammation-related genes, including cytokines adhesion molecules chemokines and enzymes, leads to the development of inflammation (Wang et al., 2015). The reprogramming of adipocytes metabolism in obese patients causes hypoxia of adipocytes and functional impairment of adipocytes, which contributes to chronic inflammation, lipolysis and insulin resistance. Consequently, the inflammation may be relevant to the development of type 2 diabetes (Oates and Antoniewicz, 2023).

In this study, bioinformatic methods were used to analysed the hypoxic-related genes of T2D and identify the immune genes associated with T2D, so as to screen the key hypoxic-immune genes of T2D, and provide ideas for the diagnosis and treatment of diabetes.

## 2 Materials and methods

### 2.1 Data source

The gene expression profile data of the GSE184050 and GSE95849 datasets related to T2D were downloaded from the Gene Expression Omnibus (GEO) database (https://www.ncbi.nlm.nih.gov/geo/). GSE184050 dataset including 50 peripheral blood samples obtained from T2D patients and 66 non-diabetic controls, was sequenced by Illumina HiSeq 2000 at the Baylor Sequencing Center following standard protocols. The GSE95849 dataset that includes peripheral blood mononuclear cells (PBMCs) samples from 6 T2D patients and 6 non-diabetic controls, was obtained from GPL22448 and used as a validation set. In addition, Molecular Signatures Database (MSigDB, https://www.gsea-msigdb. org/gsea/msigdb) provided 196 hypoxia-related genes.

### 2.2 Identification of hypoxia-related DEGs

The DeSeq2 R package was used to analysed the differentially expressed lncRNAs (DELs) and mRNAs (DEGs) between T2D and matched healthy samples in the GSE184050 dataset, with the screening criteria of |log_2_FC| > 0.1 and *p <* 0.05 (Love et al., 2014; Leirer et al., 2019). R package “ggvenn” was used to intersect the 196 hypoxia-related genes with the DEGs, and the overlapped genes were regarded as hypoxia-related DEGs.

### 2.3 Functional enrichment analysis of hypoxia-related DEGs

Gene Ontology (GO) and Kyoto Encyclopedia of Genes and Genomes (KEGG) enrichment analyses were taken to investigate the potential functions of the hypoxia-related DEGs by using the clusterProfiler package (Wu et al., 2021), and *p <* 0.05 was set as the screening threshold values of the results.

### 2.4 Screening of hypoxia-related hub genes

To identify hypoxia-related hub genes in T2D, we used two machine learning algorithms in the study. The Least Absolute Shrinkage and Selector Operation (LASSO) algorithm in R package “glmnet” and the Support Vector Machine-Recursive Feature Elimination (SVM-RFE) algorithm in R package “e1071” with five-fold cross-validation (nfolds = 5) were used to screen the hub genes among the hypoxia-related DEGs in T2D (Sanz et al., 2018; Zhang et al., 2019). For the LASSO analysis, following the penalty regularization parameter lambda was selected, that is, binomial deviance reached a minimum value, the genes with non-zero coefficients were chosen as candidate genes. Simultaneously, the SVM-RFE algorithms could identify the most relevant predictors by visualizing each one the RFE iteration, where the functionality is removed backward. With the weights for all genes were ranked and arranged, the feature genes with the lowest error rate point were chosen. The genes identified by two algorithms were intersected as hypoxia-related hub genes for further analysis.

### 2.5 Evaluation of immune cell infiltration

The scores for 64 immune and stromal cells infiltration of T2D and control samples in the GSE184050 dataset were calculated using the xCell algorithm based on the cell gene markers (https://xcell.ucsf.edu/) (Aran et al., 2017), where the immune cells and stromal cells with differentially infiltrated scores between T2D and healthy groups were identified by Wilcoxon test, with the screening criteria of *p <* 0.05.

### 2.6 Construction of weighted correlation network analysis (WGCNA)

In order to find the genes associated with differentially infiltrated immune cells, the WGCNA package in R was utilized to construct a weighted correlation network based on all genes of all samples in the GSE184050 dataset (Langfelder and Horvath, 2008). First, to cluster the samples in the GSE184050 dataset, we employed the Pearson’s correlation coefficient and constructed a sample clustering tree after removing outliers. Then, in order to ensure the gene interactions accord with the scale-free distribution to the maximum extent possible, we set the soft threshold (power) as 7 to construct a scale-free network by using the “pickSoftThreshold” function provided by WGCNA R package. The genes were clustered after determining the power value of the network. For each gene module, the minimum number of genes was set to 100, then modules were obtained by using dynamic shear tree algorithm. Next, we used the “mergeCloseModules” function to set the cut height to 0.3, and similar modules were analysed using the merged dynamic shear tree algorithm. Ultimately, we calculated the correlation between the modules and the differentially infiltrated immune cells to identify key modules (correlation coefficient > 0.7).

### 2.7 Identification and GSEA of hypoxia-immune-related hub genes

Firstly, the immune-related genes identified in WGCNA were intersected with hypoxia-related hub genes to further identify hypoxia-immune-related hub genes in T2D. Moreover, the functional enrichment analyses were carried for all genes in the high- and low-expression groups of hypoxia-immune-related hub genes by GSEA (https://www.gsea-msigdb.org/gsea/index.jsp) (Yu et al., 2019), the top ten most important GO and KEGG terms were screened and visualized by “gseaplot2” function in R package.

### 2.8 Validation of the hypoxia-immune-related hub genes

We used the GSE184050 and GSE95849 datasets to confirm the expression of hypoxia-immune-related hub genes. Significance in gene expression was tested using limma package (Ritchie et al., 2015).

In addition, quantitative real-time PCR (qRT-PCR) was used to validate the expressions of hypoxia-immune-related hub genes with 10 normal blood samples (Con group), and 10 T2D blood samples (T2D gropup). All participants were aware of the study and signed informed consent. An equal volume of human peripheral blood lymphocyte separation medium (Human) (Beijing Solarbio Science and Technology, Beijing, China) was added to 4 mL blood sample for a centrifugation of 20 min at 2,000 g. The buffy coats was collected and placed within a new tube for another 10 min at 1,000 g to extract peripheral blood mononuclear cells (PBMC), which were furher lysed by TRIzol Reagent (Life Technologies, CA, United States), and the total RNA was isolated following the manufacturer’s instructions. After detecting the concentration and the purity of RNA, 1.5 μg qualified RNA was reverse-transcribed to cDNA using the SureScript-First-strand-cDNA-synthesis-kit (Genecopoeia, Guangzhou, China). The resulting cDNA was 5-fold diluted and used for the qRT-PCR. The qRT-PCR reaction consisted of 3 µL of cDNA, 5 µL of 2xUniversal Blue SYBR Green qPCR Master Mix (Servicebio, Wuhan, China), and 1 µL each of forward and reverse primer. PCR was performed in a BIO-RAD CFX96 Touch TM PCR detection system (Bio-Rad Laboratories, Inc., United States) under the thermal cycling conditions: 40 cycles at 95°C for 60 s, 95°C for 20 s, 55°C for 20 s, and 72°C for 30 s. The 2^−△△Ct^ method was used to compute gene expressions, Statistical differences were compared using Unpaired *t*-test and the results of statistic analysis were conducted by Graphpad Prism 5 (*p* < 0.05). The primer sequences used in the current study were given in following Table 1.

**TABLE 1:** Primers for qRT-PCR used in the current study.

Primer	Sequence
*IER3*-F	FCAGCCGCAGGGTTCTCTAC
*IER3*-R	RGATCTGGCAGAAGACGATGGT
*AMPD3*-F	FCCACCGGGACTTCTATAACGT
*AMPD3*-R	RGTCAGGCTCCGTCTGGTATGT
Internal reference GAPDH-F	ACA​ACT​TTG​GTA​TCG​TGG​AAG​G
Internal reference GAPDH-R	GCC​ATC​ACG​CCA​CAG​TTT​C

### 2.9 Construction of the lncRNA-TF-mRNA network

To find out whether the hypoxia-immune-related hub genes exist regulatory network mediated by lncRNAs and human transcription factors (TFs). We obtained the human TFs from the AnimalTFDB3.0 database (http://bioinfo.life.hust.edu.cn/AnimalTFDB#!/), and then used the DELs, TFs and hypoxia-immune-related hub genes to establish a lncRNA-TF-mRNA regulatory network, with the screening criteria of the correlation coefficient > 0.7 and *p* < 0.05.

### 2.10 Statistical analysis

All analyses were conducted using R language (https://www.r-project.org/). Differences between T2D and healthy groups were compared by Wilcoxon test. Statistical data of the qRT-PCR experiment was analysed using Unpaired *t*-test and shown as mean ± standard deviation (SD) using GraphPad 5. If not specified above, *p <* 0.05 was regarded as statistically significant.

## 3 Results

### 3.1 Results for the hypoxia-related DEGs in GSE184050 cohorts

**FIGURE 1:**
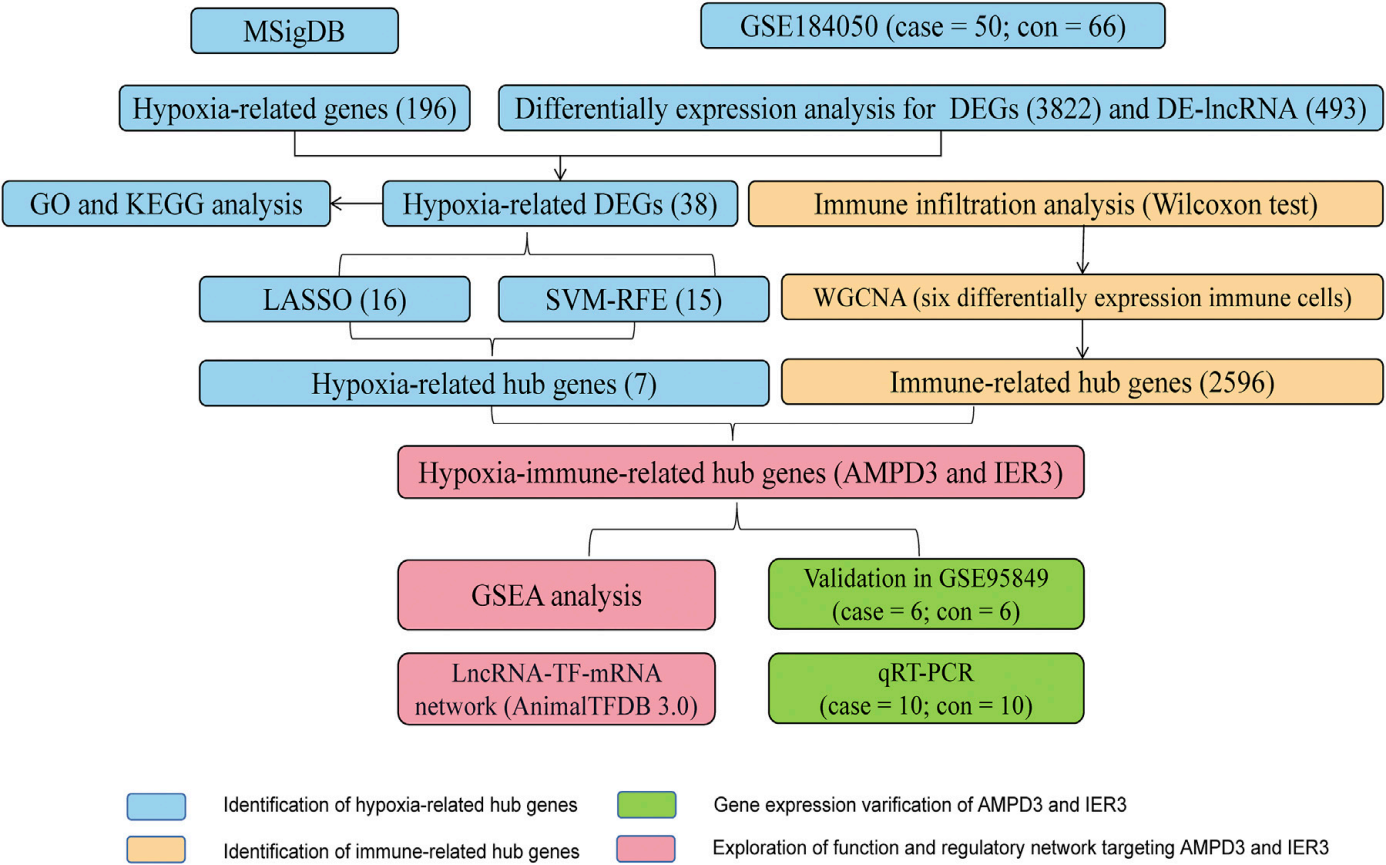
The workflow diagram of the current study.

**FIGURE 2:**
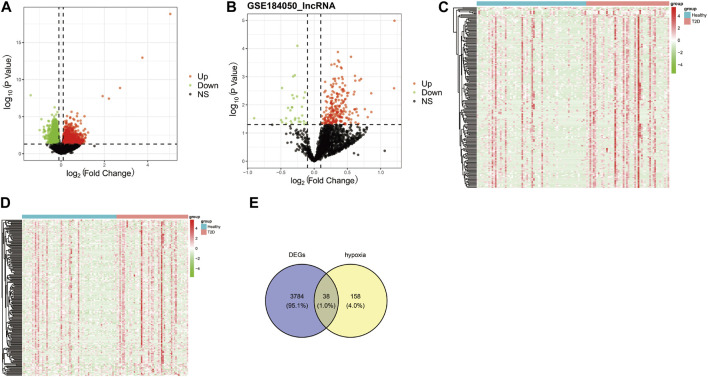
Identification of the hypoxia-related differentially expressed mRNAs (DEGs). **(A)** Volcano plot of DEGs between type 2 diabetes and control subjects in the GSE184050 dataset, with the screening criteria of |log_2_FC| > 0.1 and *p <* 0.05. **(B)** Volcano plot of differentially expressed lncRNAs (DELs) between type 2 diabetes and control subjects in the GSE184050 dataset, with the screening criteria of |log_2_FC| > 0.1 and *p <* 0.05. **(C)** Heatmap of top 200 DEGs in the GSE184050 dataset. **(D)** Heatmap of top 200 DELs in the GSE184050 dataset. **(E)** Venn diagram of the intersection of the hypoxia-related genes and the DEGs.

The workflow diagram of the current study was displayed in Figure 1. There were 3822 DEGs and 493 DELs between T2D and control samples identified in the analysis of the GSE184050 dataset. Among 3822 DEGs, 1950 genes were upregulated, but 1872 genes were downregulated in T2D samples compared to the control samples (Figure 2A; Supplementary Table S1). In addition, among 493 DELs, 461 lncRNAs were upregulated, while 32 lncRNAs were downregulated in T2D samples (Figure 2B; Supplementary Table S2). We plotted the heatmaps of top 200 DEGs and DELs in the GSE184050 dataset, respectively (Figures 2C, D). Then, we obtained 38 hypoxia-related DEGs through the intersection of the 196 hypoxia-related genes and the 3822 DEGs (Figure 2E; Supplementary Table S3).

### 3.2 Hypoxia-related DEGs were associated with the activation of “response to oxygen levels” and “glycolysis/gluconeogenesis”

**FIGURE 3:**
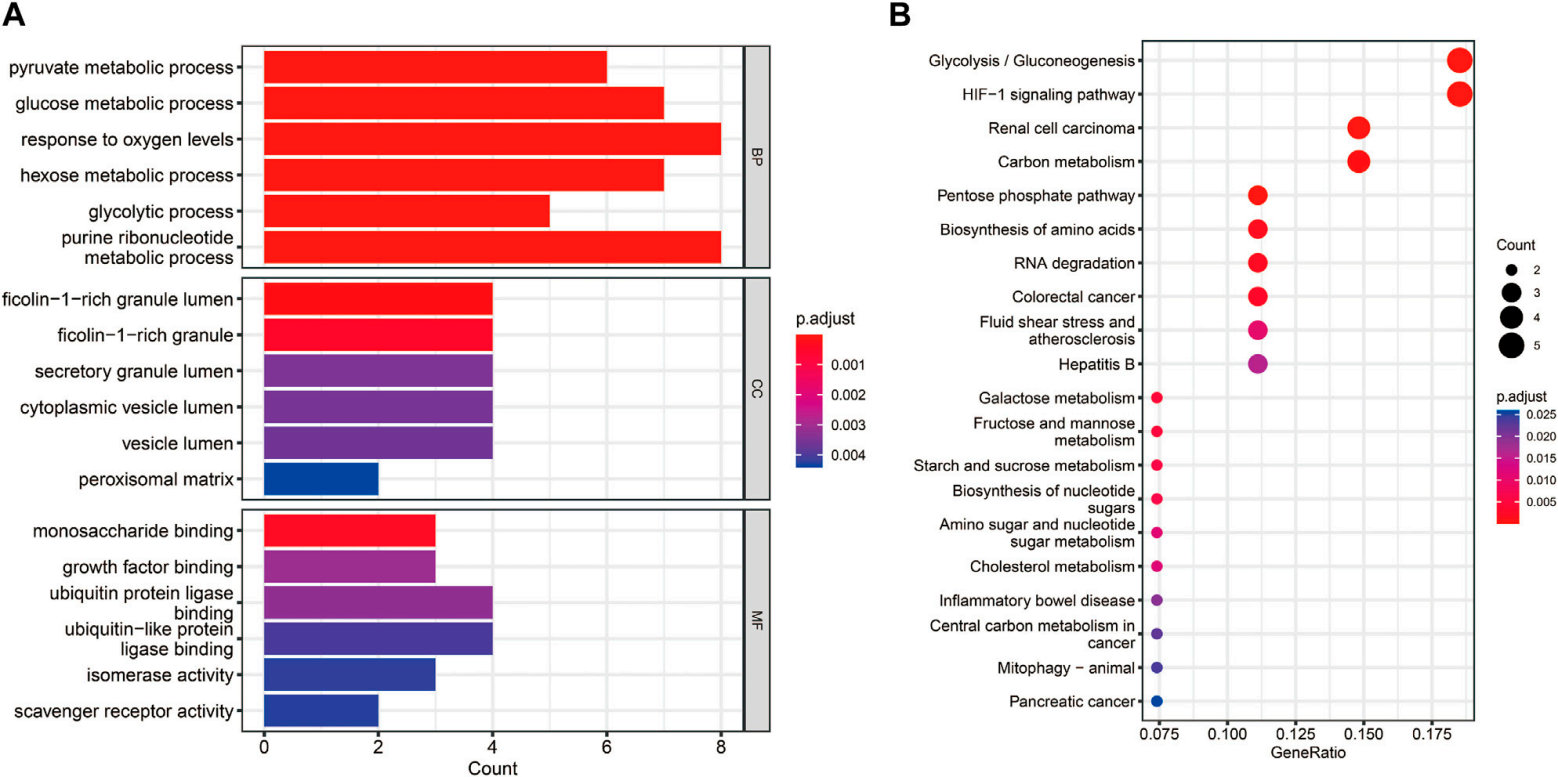
Functional enrichment analyses for the hypoxia-related DEGs. **(A)** Bar chart of the enriched Gene Ontology (GO) terms. **(B)** Bubble chart of the activated Kyoto Encyclopedia of Genes and Genomes (KEGG) terms.

The functional enrichment analyses of GO and KEGG were conducted to investigate the possible biological functions of these 38 hypoxia-related DEGs. We obtained 59 KEGG pathways and 1230 GO entries [including 1,145 biological processes (BP), 63 cell components (CC) and 22 molecular functions (MF)]. The results revealed that in the BP category, the hypoxia-related DEGs were mainly associated with “pyruvate metabolic process,” “glucose metabolic process,” and “response to oxygen levels.” As for CC, these hypoxia-related DEGs were mainly correlated with “cytoplasmic vesicle lumen,” “peroxisomal matrix,” and “vesicle lumen.” From the MF, these hypoxia-related DEGs were significantly related to “monosaccharide binding,” “growth factor binding,” and “ubiquitin protein ligase binding” (Figure 3A). According to the KEGG analysis, these hypoxia-related DEGs were significantly enriched in “glycolysis/gluconeogenesis,” “pentose phosphate pathway,” and “biosynthesis of nucleotide sugars” (Figure 3B).

### 3.3 Seven hypoxia-related hub genes were identified through the LASSO and SVM-RFE algorithms

**FIGURE 4:**
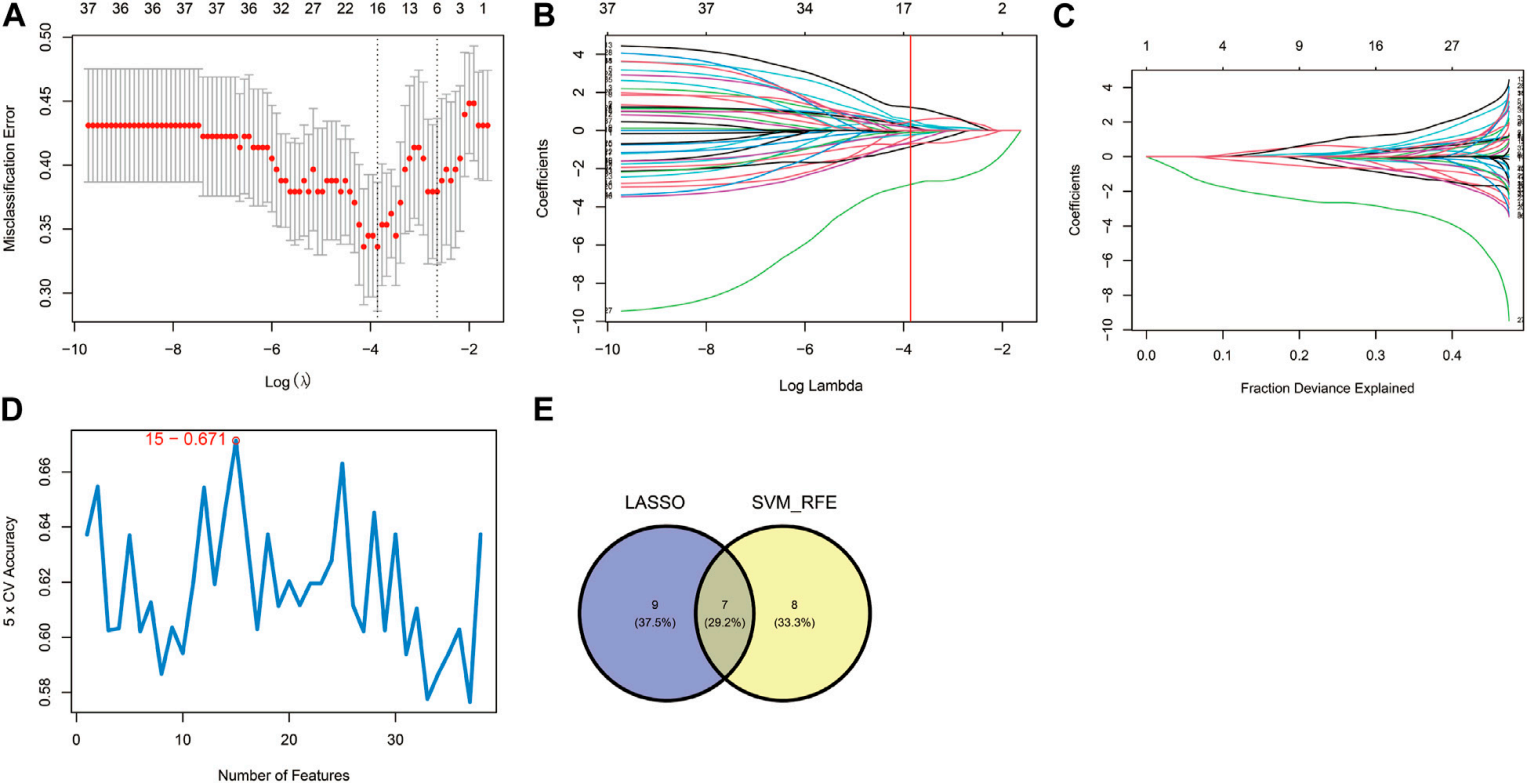
Identification of hypoxia-related hub genes. **(A–C)** Result of the Least Absolute Shrinkage and Selector Operation (LASSO) algorithm (*λ* = 0.02110191). **(D)** Result of the Support Vector Machine-Recursive Feature Elimination (SVM-RFE) algorithm. **(E)** The overlap of genes from the two algorithms.

These 38 hypoxia-related DEGs were analysed by using the LASSO and SVM-RFE algorithms to explore the hub genes. The LASSO algorithm revealed that 16 genes have been identified as signature genes (Figures 4A–C). Besides, we evaluated these 38 hypoxia-related DEGs and 15 signature genes were obtained by using the SVM-RFE algorithm (Figure 4D). There were 7 overlapping genes (*IER3*, CDKN1A, KLF6, *AMPD3*, SCARB1, VHL and PRDX5) obtained through the intersection of these two algorithms (Figure 4E).

### 3.4 Six differentially infiltrated immune cells were identified in GSE184050

**FIGURE 5:**
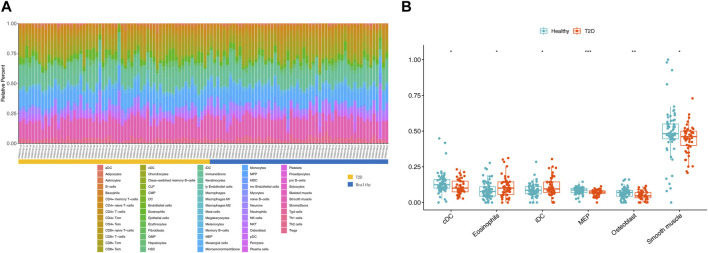
Immune infiltration analysis. **(A)** Histogram of 64 immune and stromal cells score through the xCell algorithm. **(B)** Boxplot for the differentially infiltrated immune cells and stromal cells between T2D and healthy groups using Wilcoxon test, *p < 0.05, **p < 0.01, ***p < 0.001.

To evaluate abundance of immune infiltrates, xCell algorithm was used to depict the immune and stromal cell landscapes of T2D and healthy samples in the GSE184050 dataset. Figure 5A showed the proportions of immune cells and stromal cells for each samples. It can be found that cDC, eosinophils, iDC, MEP, osteoblast and smooth muscle expressed differently between T2D and healthy samples through Wilcoxon test (Figure 5B).

### 3.5 Identification of immune-related genes through WGCNA

**FIGURE 6:**
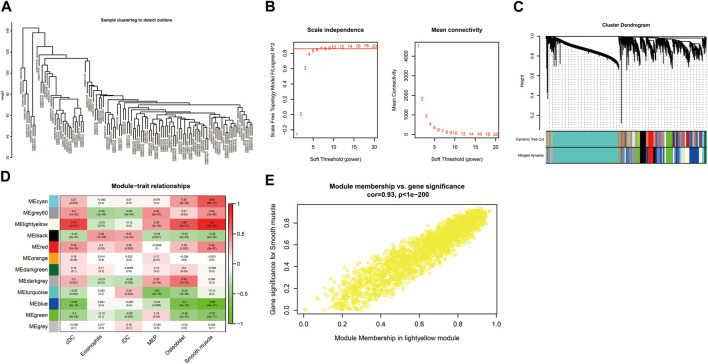
Construction of weighted correlation network analysis (WGCNA) for key modules. **(A)** The sample clustering tree of all samples in the GSE184050 dataset.**(B)** Analysis of network topology for various soft-thresholding powers through the “pickSoftThreshold” function, including the scale-free fit index (left) and the mean connectivity (right). **(C)** Result of clustering dendrogram of genes with different module colors. **(D)** The correlation heatmap between the modules and the differentially infiltrated immune cells. **(E)** Correlation scatter plots of module membership (MM) and gene significance (GS) for the lightyellow module.

The sample clustering tree of all samples in the GSE184050 dataset showed there were no outliers (Figure 6A). The scale independence reached 0.8 when the soft threshold was set at 7 (Figure 6B). After determining the soft threshold, we set the minimum module size at 100, the cut height at 0.3 and then 12 gene co-expression modules were established (Figure 6C). We correlated modules with differentially expressed immune and stromal cells and the results showed that lightyellow module was significantly correlated with cDC and the correlation coefficient was 0.74. It was also significantly correlated with smooth muscle and the correlation coefficient was 0.9. Therefore, we regarded the 2,596 mRNAs in the lightyellow module as the immune-related genes (Figures 6D, E).

### 3.6 *AMPD3* and *IER3* were identified as the hypoxia-immune-related hub genes

**FIGURE 7:**
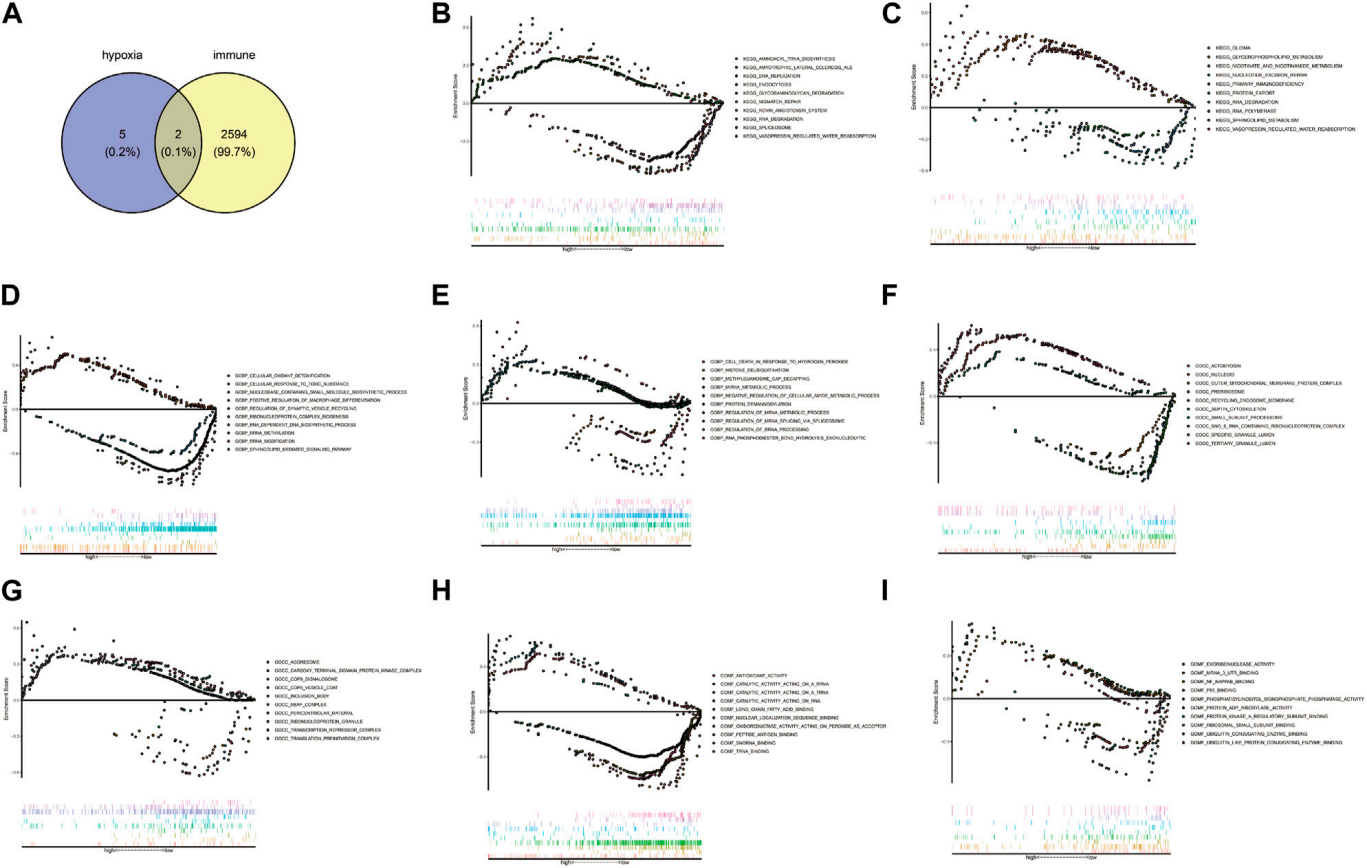
Gene Set Enrichment Analysis (GSEA) of hypoxia-immune-related hub genes. **(A)** Venn diagram by intersecting 2,596 immune-related genes and 7 hypoxia-related hub genes. **(B,C)** The results of GSEA analysis between all genes in the high- and low-expression groups of hypoxia-immune-related hub genes. **(D,E)** The results of GSEA analysis in high-expression groups of *AMPD3* and *IER3*. **(F,G)** The significantly enriched biological processes (BP) terms in high-expression groups of *AMPD3* and *IER3*. **(H,I)**. The significantly enriched molecular functions (MF) terms in high-expression groups of *AMPD3* and *IER3*.

Venn diagram showed *AMPD3* and *IER3* were hypoxia-immune-related hub genes in T2D, through the intersection of 2,596 immune-related genes and 7 hypoxia-related hub genes (Figure 7A). Moreover, the results of GSEA analysis between these genes in the high- and low-expression groups of hypoxia-immune-related hub genes showed that the KEGG pathways of genes in high-expression groups of *AMPD3* and *IER3* were mainly enriched in “glycosaminoglycan degradation,” “amyotrophic lateral sclerosis,” “nicotinate and nicotinamide metabolism” and “vasopressin-regulated water reabsorption,” while the genes in low-expression groups of *AMPD3* and *IER3* were mainly in “RNA degradation” and “nucleotide excision repair” (Figures 7B, C). Meanwhile, the results of genes in high-expression groups of *AMPD3* and *IER3* revealed that the BP were mainly enriched in “positive regulation of macrophage differentiation,” “regulation of synaptic vesicle recycling,” “miRNA metabolic process” and “cell death in response to hydrogen peroxide,” the genes in low-expression groups were significantly associated with “rRNA methylation” and “methylguanosine cap decapping” (Figures 7D, E). As for CC, the significantly enriched terms in high-expression groups of *AMPD3* and *IER3* were “septin cytoskeleton,” “actomyosin,” “ribonucleoprotein granule” and “transcription repressor complex,” but the genes in low-expression groups were closely associated with “small subunit processome” and “COP9 Signalosome” (Figures 7F, G). The significantly enriched MF terms in high-expression groups of *AMPD3* and *IER3* included “nuclear localization sequence binding,” “antioxidant activity,” “ubiquitin-like protein conjugating enzyme binding” and “ubiquitin conjugating enzyme binding,” while the low-expression groups were closely correlated with “snoRNA binding” and “ribosomal small subunit binding” (Figures 7H, I).

### 3.7 Validation of *AMPD3* and *IER3* in different online cohorts and clinical samples

**FIGURE 8:**
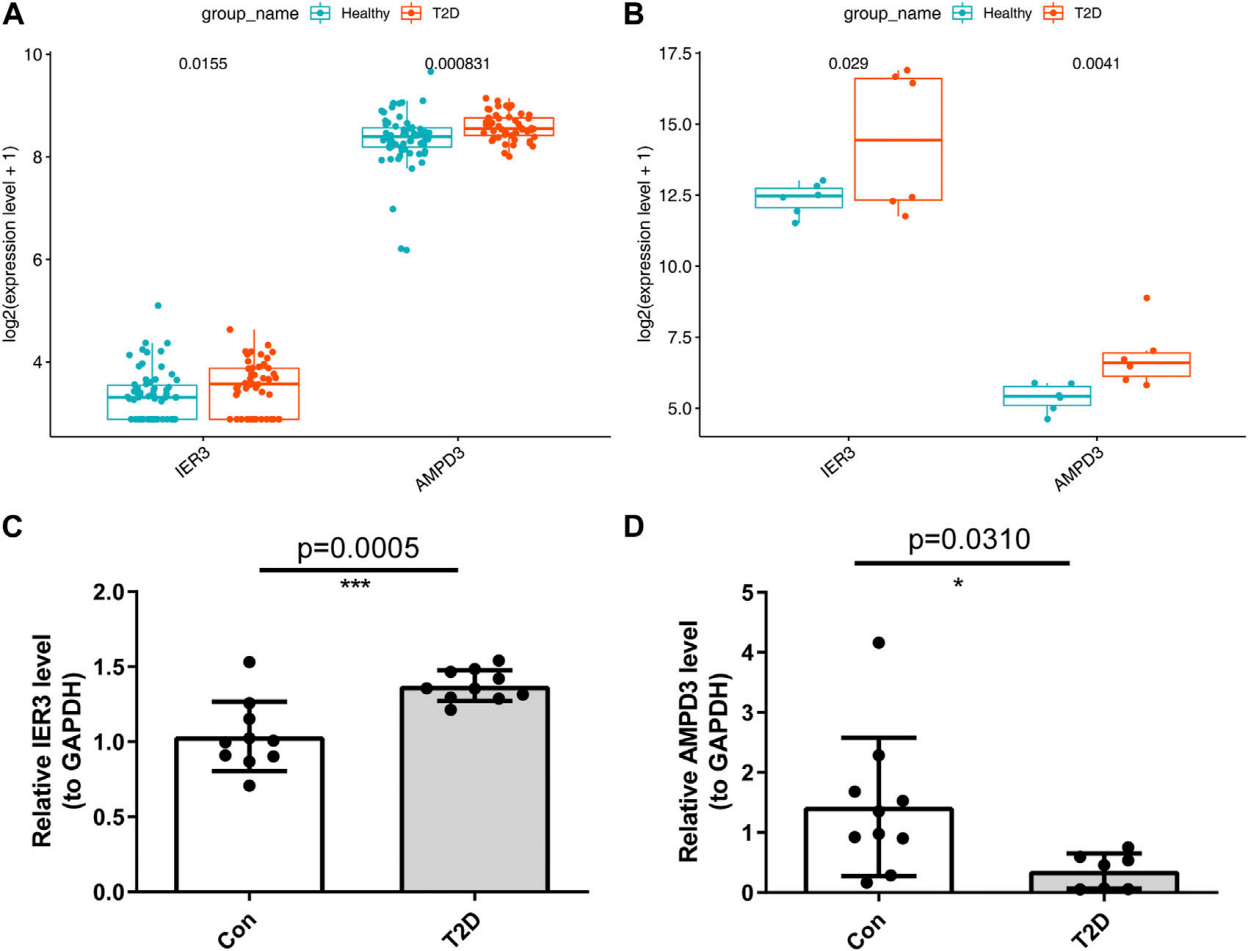
Validation of hypoxia-immune-related hub genes in T2D and healthy groups. **(A)** The expression patterns of *AMPD3* and *IER3* in the GSE184050. **(B)** The expression patterns of *AMPD3* and *IER3* in the GSE95849. **(C, D)** The qRT-PCR was used to varify the expression of two hub genes in blood lymphocytes that were extracted from 10 normal samples and 10 T2D samples. Unpaired *t*-test was performed to analysed the difference significance, and the results were presented as means ± standard deviation (SD), *p < 0.05, **p < 0.01, ***p < 0.001.

In addition, the GSE184050 and GSE95849 datasets were utilized to verify the expression of *AMPD3* and *IER3* between T2D and healthy groups. The expression results of these two hub genes were demonstrated in Figures 8A, B, respectively. Both the *IER3* and *AMPD3* were highly expressed in the T2D groups compared with normal samples. And meanwhile, the qRT-PCR results for 10 normal and 10 T2D blood samples indicated the expression of *IER3* was consistent with the bioinformatic results (Figure 8C; Table 2), but the expression of *AMPD3* was lower in T2D samples than that in controls (Figure 8D; Table 2).

**TABLE 2:** Statistics result of hub genes by qRT-PCR.

	Con	T2D	t, df	p
*IER3*	1.0355 ± 0.2313	1.3730 ± 0.1027	t = 4.217 df = 18	0.0005
*AMPD3*	1.4265 ± 1.1501	0.3590 ± 0.2950	t = 2.380 df = 15	0.031

### 3.8 The key lncRNAs and TFs targeting *AMPD3* were predicted

**FIGURE 9:**
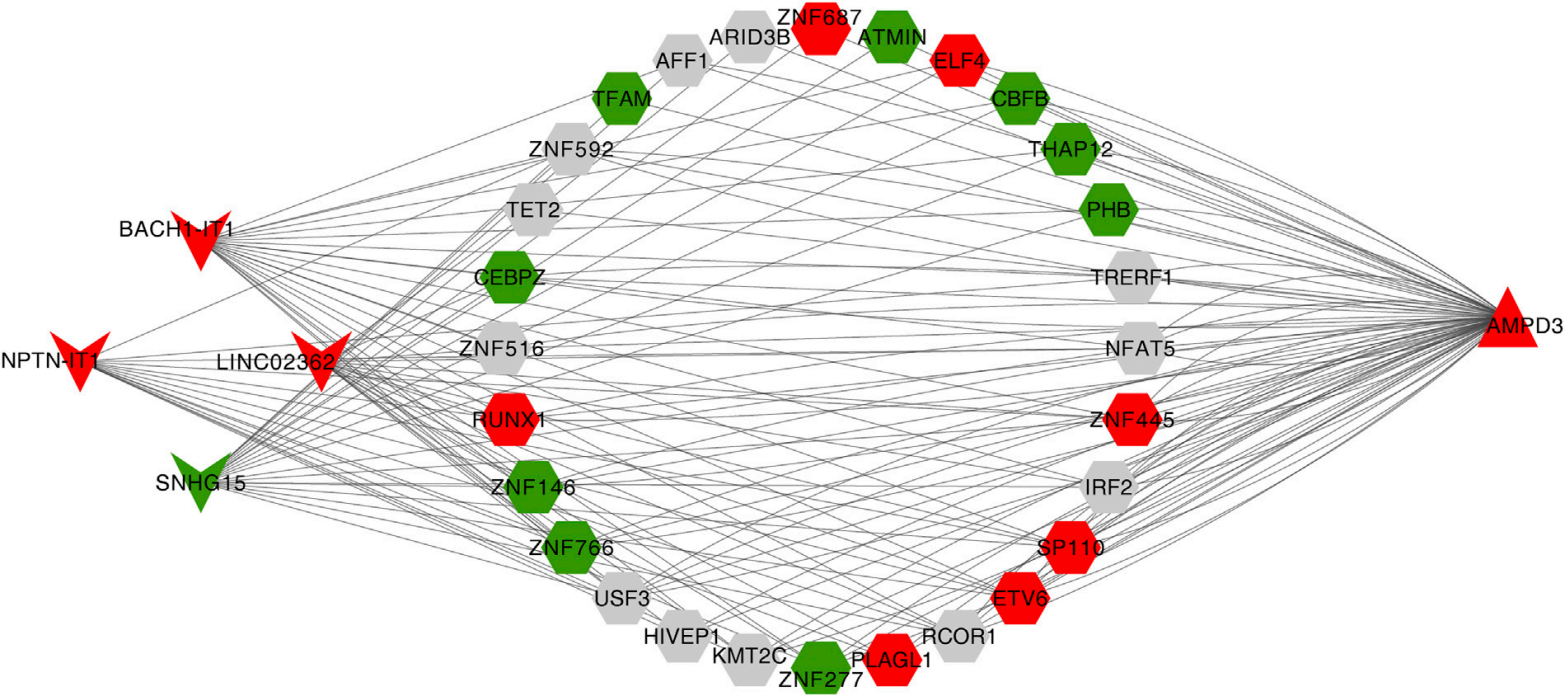
Construction of the lncRNA-TF-mRNA network. Red indicates upregulation and green indicates downregulation. LncRNA, TF, and mRNA were indicated by V shanpe, hexagon, and triangle, respectively.

Eventually, a lncRNA-TF-mRNA network was constructed to explore the molecular mechanism of T2D-related lncRNA. 1,665 human TFs were obtained from the database, 493 DELs, 1,665 human TFs and 2 hypoxia-immune-related hub genes (*AMPD3* and *IER3*) were used to establish the regulatory network. It can be found from the lncRNA-TF-mRNA network (Figure 9) that 3 lncRNAs (BACH1-IT1, NPTN-IT1 and LINC02362) were upregulated while 1 lncRNA (SNHG15) was downregulated, and they might make the *AMPD3* upregulated through inducing the expression of the corresponding TFs such as TFAM, CBFB and THAP12.

## 4 Discussion

Type 2 diabetes is a metabolic disease characterized by abnormal glucose metabolism. Insulin resistance is the core link of its development, and obesity is the main inducement. Adipose hypoxia is one of the early manifestations of obesity, accompanied by secretion changes of cytokines and fatty acids (Lolmède et al., 2003). In addition to causing adipose tissue inflammation and insulin resistance, these factors can also affect the insulin sensitivity of skeletal muscle and liver through blood circulation, leading to systematic insulin resistance (Sears et al., 2009), and further aggravating glucose metabolism disorder.

We used bioinformatic methods to preliminarily explore and screen the hypoxia-immune-related hub genes associated with diabetes and validated them in human subjects. Adenosine monophosphate deaminase 3 (*AMPD3*) and immediate early response 3 (*IER3*) genes were found to be hub genes of hypoxia-immunity in T2D. *AMPD3* encodes AMP deaminase 3, which catalyzes the first and rate-limiting-step of the purine nucleotide cycle (PNC). Purine nucleotide cycling deaminates amino acids to produce α-ketoglutaric acid, which further participates in metabolism. Previous publications have shown that overexpression of *AMPD3* predisposes skeletal muscles to use lipids instead of glucose for energy, causing insulin resistance and glucose intolerance and insulin resistance process could be aggravated by systemic and tissue-specific alterations of lymphocyte (Hong et al., 2009; Ip et al., 2015). In addition, *AMPD3* in erythrocytes catalyzes AMP into IMP if *AMPD3* is overexpressed, and it accelerates these biochemical reactions and lowers AMP levels. Previous studies of the obese rat model of T2D have shown that the upregulated protein levels of *AMPD3* could impair cardiac energy by targeting ATP depletion and systolic dysfunction (Tatekoshi et al., 2018; Igaki et al., 2021; Ogawa et al., 2023). In this study, the results in the online datasets were consistent with previous studies that the *AMPD3* gene was upregulated in T2D samples. Similarly, it was also reported that the activation of the amyotrophic lateral sclerosis and glycosaminoglycan degradation signal pathways in the GSEA results of *AMPD3* could be due to the increased expression of *AMPD3* (Miller et al., 2021), indicating that the crutial significance of the over-expression of *AMPD3* in T2D progression. However, the results of qRT-PCR for peripheral blood samples with T2D and found that *AMPD3* expression was downregulated in T2D samples. Considering the consistency of sample sources both in online databases and the qRT-PCR experiments, the paradox of *AMPD3* expression might be attributed to the small sample size in clinical experiments. For this, we will further explore the expression changes and underlying mechanism of AMPD3 in T2D progression by collecting more clinical samples in the future. Moreover, the decrease of AMP level leads to the decrease of ATP level, which reduces the P50 value of erythrocytes and increases the affinity of erythrocytes for oxygen, thus reducing oxygen delivery to tissues and leading to the occurrence of tissue hypoxia (O'Brien et al., 2015). Previous researches have shown that hypoxia can increase the expression of hypoxia-inducible factor-1α (HIF-1α) in many human tissues (Lemus-Varela et al., 2010), and intermittent hypoxia can increase the production of reactive oxygen species (ROS) in mitochondria (Wang et al., 2013). The level of ROS in cells depends on the balance of oxidases and antioxidant enzymes. NADPH oxidase (NOx4) is the major oxidase of pancreatic β cells (Mahadev et al., 2004), and HIF-1A increases ROS production by activating NOx4 (Diebold et al., 2010). Continuous ROS production may contribute to excessive insulin secretion, further resulting in *β* cell dysfunction (Anvari et al., 2015). Mitochondrial ROS may also trigger the activation of pro-inflammatory signaling pathway, especially, it induces the activation of redox sensitive transcription factors, such as nuclear factor κB (NF-κB). NF-κB contributes to the production of pro-inflammatory cytokines, including IL-1 and IL-8 (Patergnani et al., 2021), which further influence glucose metabolism.

*IER3* is an early stress-inducing gene and a direct downstream transcription target of NF-κB (De Keulenaer et al., 2002). It plays an important role in regulating apoptosis and controlling the heterogeneity of immune cells (Zhang et al., 2003; Shen et al., 2006). Macrophages are a major cause of obesity-related inflammation. Adipose macrophages, which switch from anti-inflammatory alternative activated macrophages (AAM) to pro-inflammatory classical activated macrophages (CAM), is important in obesity-related inflammation (Arkan et al., 2005; Lumeng et al., 2007). The high expression of *IER3* in macrophages can promote the transformation of macrophages from AAM to CAM, and promote the occurrence of obesity-related inflammation. Hong et al. (2017), report that increased AAMs and reduced inflammation in *Ier3* ^−/−^ mice may contribute to improved insulin sensitivity in these mice. In conclusion, *IER3* may affect glucose metabolism through obesity-related inflammation.

In this study, 38 hypoxia-related DEGs were functionally enriched by GO and KEGG. Pyruvate is the final product of glucose anaerobic glycolysis. Peroxisomes are ubiquitous in all types of cells in eukaryotes, and its signature enzyme is catalase, a type of ROS scavengers, which hydrolyzes hydrogen peroxide to protect cells. If hydrogen peroxide exceeds the catalase catalytic capacity, cell damage can be caused. Insulin, which is synthesized by the islet *β* cells, is packaged in vesicles and shipped out of the cell, and instability of this system can cause diabetes. The intermediate products of glycolysis can enter the pentose phosphate pathway to generate pentose, which provides raw materials for the synthesis of ribonucleotide. The abnormality of these pathways will affect glucose metabolism.

Our final lncRNA-TF-mRNA network contains four lncRNAs, which are BACH1-IT1, NPTN-IT1, LINC02362 and SNHG15. So far, no relevant studies have been found on lncRNA BACH1-IT1 in any diseases. NPTN-IT1 is a recently identified lncRNA, located on chromosome 15q24.1, which has a low expression level in tumor tissues and can inhibit the growth and metastasis of hepatocellular carcinoma, lung adenocarcinoma (Yang et al., 2013; Liu et al., 2016), nasopharyngeal carcinoma and cervical cancer (Jiang et al., 2015; Sun et al., 2015). LINC02362 is associated with better prognosis of HCC patients, and inhibits the survival, migration and invasion of HCC cells and epithelial interstitial transformation (EMT) (Li et al., 2022b). Although there is no relevant study on the above lncRNANs in diabetes, it is well known that hypoxia is an important feature of tumor microenvironment, and the above lncRNAN regulates tumor growth through hypoxia related pathways, and may also affect glucose metabolism through hypoxia related pathways. However, further laboratory studies are needed to verify this. In addition, SNHG15 is an IL-4-induced macrophage LncRNA that uniquely inhibits K63 linked TRAF2 ubiquitination, thereby promoting M2 macrophage polarization and alleviating inflammatory response after stroke (Sun et al., 2022). In conclusion, SNHG15 is a negative regulator of inflammation, and its expression is downregulated in patients with type 2 diabetes, which may be related to the high level of inflammation in type 2 diabetes.

Our findings provide a new perspective and lay the foundation for future studies on the potential role of hypoxia-immune genes in T2D. Altogether, this study provides a novel reference for the follow-up exploration of the molecular mechanism of lncRNA in the progression of T2D and provides a new reference for potential immunotherapy targets. We will continue to focus on the role of these genes in T2D based on more basic experiments that target potential pathways.

## Data Availability

Publicly available datasets were analyzed in this study. This data can be found here: https://www.ncbi.nlm.nih.gov/geo/.
