# Supplementary material for: Identification and analysis of hub genes of hypoxia-immunity in type 2 diabetes mellitus
Source: Front Genet. 2023 Apr 21;14:1154839. doi: 10.3389/fgene.2023.1154839 (PMC10160629; doi:10.3389/fgene.2023.1154839)
Supplement: Supplementary file 1 [file Table1.DOCX]

***Supplementary Material***

**Identification and analysis of hub genes of hypoxia-immunity in type 2 diabetes mellitus**

Jing Li^1^, Ni Yan^2^, Xiaofeng Li^1^, Shenglin He^1^, Xiangyou Yu^1*^

^1^Department of Endocrinology Diabetes, Shaanxi Provincial People's Hospital, Xi 'an, China.

^2^Department of Rheumatology and Immunology, Shaanxi Provincial People's Hospital, Xi 'an, China.

*** Correspondence:**

Xiangyou Yu

yxy74998@sina.com

1 **Supplementary Tables**

**Supplementary Table 1.** Lists of the differentially expressed mRNAs (DEGs) between type 2 diabetes (T2D) and normal samples in GSE184050.

| symbol | logfc | p.value | adj.p.val | direction | log10pvalue |
| --- | --- | --- | --- | --- | --- |
| SAMD11 | 0.685428655 | 0.008826711 | 0.119262139 | Up | 2.054201115 |
| NOC2L | -0.043682281 | 0.604922831 | 0.757685768 | NS | 0.218300024 |
| KLHL17 | 0.072379091 | 0.459249031 | 0.650647776 | NS | 0.337951752 |
| PLEKHN1 | 0.703198666 | 0.012812739 | 0.136078062 | Up | 1.892358034 |
| PERM1 | 0.917456801 | 0.061196204 | NA | NS | 1.213275516 |
| HES4 | 0.489199354 | 0.015711532 | 0.148749691 | Up | 1.803781469 |
| ISG15 | 0.456118037 | 0.007357352 | 0.112409264 | Up | 2.133278454 |
| AGRN | 0.598891404 | 0.004151538 | 0.091333846 | Up | 2.381790937 |
| RNF223 | 0.839173521 | 0.10048365 | NA | NS | 0.997904599 |
| C1orf159 | 0.168404891 | 0.128240035 | 0.350012309 | NS | 0.891976372 |
| TTLL10 | 0.774181508 | 0.049347706 | NA | Up | 1.306733033 |
| TNFRSF18 | -0.026582028 | 0.846208186 | 0.913405762 | NS | 0.072522778 |
| TNFRSF4 | 0.279840335 | 0.02722119 | 0.18581809 | Up | 1.565092895 |
| SDF4 | -0.106479674 | 0.20634221 | 0.436307756 | NS | 0.685411921 |
| B3GALT6 | -0.129937533 | 0.068962617 | 0.269014112 | NS | 1.161386267 |
| C1QTNF12 | 0.699542577 | 0.040418191 | NA | Up | 1.393423126 |
| UBE2J2 | -0.062475773 | 0.239770099 | 0.467834086 | NS | 0.620204978 |
| SCNN1D | 0.250741472 | 0.125735279 | 0.347428564 | NS | 0.90054285 |
| ACAP3 | 0.047568855 | 0.658607479 | 0.794659316 | NS | 0.181373342 |
| PUSL1 | 0.160129761 | 0.071455719 | 0.272521361 | NS | 1.145963006 |
| INTS11 | -0.122986148 | 0.027707893 | 0.186639939 | Down | 1.557396503 |
| CPTP | 0.482351417 | 2.26E-06 | 8.93E-04 | Up | 5.645755803 |
| TAS1R3 | 0.165124989 | 0.198237419 | 0.428143472 | NS | 0.702814364 |
| DVL1 | -0.058651345 | 0.39607857 | 0.601442813 | NS | 0.402218655 |
| MXRA8 | 0.108877264 | 0.421418669 | 0.621298082 | NS | 0.375286229 |
| AURKAIP1 | -0.09471314 | 0.319934702 | 0.539024418 | NS | 0.494938652 |
| CCNL2 | -0.110763055 | 0.030737182 | 0.194647058 | Down | 1.512335957 |
| MRPL20 | -0.124004912 | 0.110678579 | 0.329185788 | NS | 0.955936426 |
| ANKRD65 | 0.698288299 | 0.095781335 | NA | NS | 1.018719115 |
| TMEM88B | 1.098497999 | 0.038113124 | NA | Up | 1.418925456 |
| VWA1 | 1.242088758 | 0.009530923 | NA | Up | 2.020865022 |
| ATAD3C | 0.604569056 | 0.051548843 | 0.239738115 | NS | 1.287781075 |
| ATAD3B | -0.084010535 | 0.295990597 | 0.517745305 | NS | 0.528722085 |
| ATAD3A | -0.038996647 | 0.678315719 | 0.808135389 | NS | 0.168568119 |
| TMEM240 | 0.682746197 | 0.043385913 | NA | Up | 1.362651258 |
| SSU72 | -0.03554954 | 0.378501011 | 0.588455293 | NS | 0.421932957 |
| FNDC10 | 0.769854915 | 0.002130445 | NA | Up | 2.671529634 |
| MIB2 | 0.070986128 | 0.513513071 | 0.691711349 | NS | 0.289448497 |
| MMP23B | 0.721849695 | 0.013678233 | NA | Up | 1.863970005 |
| CDK11B | -0.287120019 | 0.002928881 | 0.08091344 | Down | 2.533298214 |
| SLC35E2B | 0.087952723 | 0.34700415 | 0.561433348 | NS | 0.459665332 |
| CDK11A | -0.020630149 | 0.843067293 | 0.911847772 | NS | 0.074137759 |
| NADK | 0.007384959 | 0.935237624 | 0.964029708 | NS | 0.02907803 |
| GNB1 | -0.021143325 | 0.693219007 | 0.817765618 | NS | 0.159129538 |
| CALML6 | 0.279340336 | 0.393880882 | NA | NS | 0.404635098 |
| TMEM52 | 1.232389687 | 0.013685529 | NA | Up | 1.863738415 |
| CFAP74 | 0.881447108 | 0.031863977 | 0.197978516 | Up | 1.496700015 |
| GABRD | 0.976746353 | 0.01647216 | 0.15144336 | Up | 1.783249447 |
| PRKCZ | 0.023442423 | 0.772997717 | 0.867838133 | NS | 0.111821789 |
| FAAP20 | 0.179342105 | 0.044533116 | 0.225674429 | Up | 1.351316919 |
| SKI | -0.007149481 | 0.964791733 | 0.980397915 | NS | 0.015566427 |
| MORN1 | 0.129412538 | 0.235779357 | 0.463583348 | NS | 0.627494221 |
| RER1 | -0.034803979 | 0.350905129 | 0.56491456 | NS | 0.454810284 |
| PEX10 | -0.094506838 | 0.234607399 | 0.46270915 | NS | 0.629658295 |
| PLCH2 | -0.038172472 | 0.809414029 | 0.890440969 | NS | 0.091829273 |
| PANK4 | -0.170390509 | 0.006485127 | 0.1080547 | Down | 2.188081519 |
| TNFRSF14 | -0.062542172 | 0.391605777 | 0.598382407 | NS | 0.40715091 |
| PRXL2B | -0.077424524 | 0.616406055 | 0.765518761 | NS | 0.210133104 |
| MMEL1 | -0.075368221 | 0.828257994 | NA | NS | 0.081834364 |
| TTC34 | 0.89666124 | 0.041265231 | NA | Up | 1.384415725 |
| ACTRT2 | 0.969549815 | 0.113690725 | NA | NS | 0.944274962 |
| PRDM16 | 0.985463741 | 0.007945087 | 0.11472857 | Up | 2.099901328 |
| ARHGEF16 | 0.860660393 | 0.033538608 | 0.201690575 | Up | 1.47445497 |
| MEGF6 | -0.065526812 | 0.52934523 | 0.703928955 | NS | 0.276260996 |
| TPRG1L | 0.033257385 | 0.693206602 | 0.817765618 | NS | 0.15913731 |
| WRAP73 | -0.100067869 | 0.019958955 | 0.163807837 | Down | 1.699862202 |
| TP73 | 0.463354028 | 0.045372071 | 0.227426386 | Up | 1.343211402 |
| CCDC27 | 0.651789646 | 0.120055367 | 0.340707314 | NS | 0.920618419 |
| SMIM1 | 0.689998348 | 0.033906382 | 0.202615011 | Up | 1.46971855 |
| LRRC47 | -0.121640949 | 0.075562995 | 0.278850098 | NS | 1.121690838 |
| CEP104 | 0.062570183 | 0.389127569 | 0.596625801 | NS | 0.409907999 |
| DFFB | -0.080572416 | 0.096572392 | 0.310675787 | NS | 1.01514701 |
| C1orf174 | -0.092723621 | 0.109189126 | 0.327173527 | NS | 0.961820611 |
| AJAP1 | 0.444099097 | 0.061375822 | 0.257629963 | NS | 1.212002677 |
| NPHP4 | 0.211985198 | 0.104855768 | 0.32076633 | NS | 0.979407675 |
| KCNAB2 | -0.015299399 | 0.847103039 | 0.913947039 | NS | 0.07206376 |
| CHD5 | 0.693238716 | 0.032441996 | 0.198993631 | Up | 1.48889243 |
| RPL22 | -0.18102578 | 0.056717548 | 0.250037591 | NS | 1.246282553 |
| RNF207 | 0.217850197 | 0.125398493 | 0.346938269 | NS | 0.901707681 |
| ICMT | -0.184079064 | 0.020036063 | 0.164173855 | Down | 1.698187603 |
| HES3 | 0.986086129 | 0.066882784 | NA | NS | 1.174685657 |
| GPR153 | 0.533728704 | 0.002588924 | 0.077810428 | Up | 2.586880633 |
| ACOT7 | 0.326213302 | 0.010879621 | 0.12856202 | Up | 1.963386235 |
| HES2 | 1.035650421 | 0.047932964 | NA | Up | 1.319365713 |
| ESPN | 0.622682958 | 0.008355333 | 0.117538314 | Up | 2.078036259 |
| TNFRSF25 | -0.023701965 | 0.809243352 | 0.890338742 | NS | 0.09192086 |
| PLEKHG5 | 0.201735134 | 0.256780048 | 0.483775638 | NS | 0.590438725 |
| NOL9 | 0.022763078 | 0.654171905 | 0.792178995 | NS | 0.184308112 |
| TAS1R1 | 0.146362819 | 0.689516859 | NA | NS | 0.161455111 |
| ZBTB48 | 0.041840316 | 0.503190043 | 0.684244941 | NS | 0.298267961 |
| KLHL21 | 0.170681228 | 0.068230223 | 0.268476763 | NS | 1.166023208 |
| PHF13 | 0.006581797 | 0.912058242 | 0.951225368 | NS | 0.039977428 |
| THAP3 | -0.171969213 | 0.008740504 | 0.118782974 | Down | 2.058463543 |
| DNAJC11 | -0.047097692 | 0.506134321 | 0.68631843 | NS | 0.295734212 |
| CAMTA1 | 0.01718877 | 0.78072091 | 0.873306158 | NS | 0.107504189 |
| VAMP3 | -0.081900357 | 0.290862681 | 0.513652722 | NS | 0.536311998 |
| PER3 | 0.031134975 | 0.721545614 | 0.835727388 | NS | 0.141736209 |
| UTS2 | -0.4089119 | 0.13517915 | 0.358941784 | NS | 0.86909029 |
| TNFRSF9 | 0.068521445 | 0.560171636 | 0.724761145 | NS | 0.251678885 |
| PARK7 | -0.240621434 | 0.011961776 | 0.133960191 | Down | 1.922204341 |
| ERRFI1 | 0.060383512 | 0.600502409 | 0.754648056 | NS | 0.221485246 |
| SLC45A1 | 0.812955087 | 0.015318629 | NA | Up | 1.814780102 |
| RERE | 0.006479615 | 0.954298419 | 0.975336593 | NS | 0.020315796 |
| ENO1 | -0.239020172 | 0.015763033 | 0.148929448 | Down | 1.802360214 |
| CA6 | -0.111981768 | 0.506901814 | 0.686967593 | NS | 0.295076154 |
| SLC2A7 | 0.755372646 | 0.108934385 | NA | NS | 0.962835016 |
| SLC2A5 | -0.030303591 | 0.819957629 | 0.897279394 | NS | 0.086208589 |
| GPR157 | 0.004420323 | 0.958631885 | 0.977149164 | NS | 0.01834813 |
| H6PD | 0.226154633 | 0.011602779 | 0.132180121 | Up | 1.935437984 |
| SPSB1 | 0.097377801 | 0.392410961 | 0.599053182 | NS | 0.40625887 |
| SLC25A33 | -0.17652677 | 0.098003172 | 0.312744522 | NS | 1.008759866 |
| TMEM201 | 0.45265975 | 0.035016509 | 0.20515428 | Up | 1.455727158 |
| PIK3CD | 0.007110364 | 0.939182647 | 0.966553041 | NS | 0.027249941 |
| CLSTN1 | 0.059700917 | 0.56172461 | 0.726076138 | NS | 0.250476549 |
| CTNNBIP1 | 0.190568258 | 0.017197885 | 0.154054413 | Up | 1.764524968 |
| LZIC | -0.201681287 | 0.001556638 | 0.062836479 | Down | 2.807812246 |
| NMNAT1 | -0.05228375 | 0.372114815 | 0.583052983 | NS | 0.429323039 |
| RBP7 | -0.095832811 | 0.512623377 | 0.691068933 | NS | 0.290201592 |
| UBE4B | 0.028412777 | 0.762833601 | 0.861842787 | NS | 0.117570185 |
| KIF1B | 0.1505045 | 0.123877681 | 0.344887752 | NS | 0.907006933 |
| PGD | 0.03685989 | 0.73748614 | 0.845467666 | NS | 0.132246137 |
| CENPS-CORT | -0.442991489 | 0.228798438 | NA | NS | 0.640546945 |
| CENPS | 0.160314945 | 0.514802138 | NA | NS | 0.288359658 |
| CORT | 0.301127771 | 0.309367335 | NA | NS | 0.509525543 |
| DFFA | -0.14197764 | 0.010456454 | 0.127471706 | Down | 1.980615585 |
| PEX14 | 0.085753332 | 0.401264774 | 0.605820044 | NS | 0.396568964 |
| CASZ1 | 0.351778268 | 0.007370424 | 0.112456655 | Up | 2.132507556 |
| C1orf127 | 0.307941202 | 0.336841231 | 0.55308503 | NS | 0.472574754 |
| TARDBP | 0.137960912 | 4.13E-04 | 0.034593867 | Up | 3.383886214 |
| MASP2 | -6.34E-04 | 0.992699141 | 0.995749068 | NS | 0.003182354 |
| SRM | 0.18889576 | 0.046535143 | 0.229687036 | Up | 1.332218947 |
| EXOSC10 | -0.193189326 | 5.66E-04 | 0.040211156 | Down | 3.247515455 |
| MTOR | 0.017326611 | 0.763438312 | 0.862228238 | NS | 0.117226049 |
| ANGPTL7 | 0.174458813 | 0.217054267 | 0.446641045 | NS | 0.663431672 |
| UBIAD1 | -0.1241911 | 0.054487069 | 0.246759511 | NS | 1.263706556 |
| DISP3 | 0.571698979 | 0.117516282 | 0.337510592 | NS | 0.929901958 |
| FBXO2 | -0.105694349 | 0.544167581 | 0.713285597 | NS | 0.264267335 |
| FBXO44 | -0.129386346 | 0.117265817 | 0.337267611 | NS | 0.930828567 |
| FBXO6 | 0.010612066 | 0.926126938 | 0.959397324 | NS | 0.033329484 |
| MAD2L2 | -0.147512078 | 0.074107301 | 0.276552943 | NS | 1.130139003 |
| DRAXIN | -0.002722483 | 0.981958679 | 0.989345725 | NS | 0.007906787 |
| AGTRAP | 0.164949314 | 0.032904035 | 0.200078102 | Up | 1.482750844 |
| C1orf167 | 0.975338081 | 0.019983118 | NA | Up | 1.699336736 |
| MTHFR | 0.146789797 | 0.061800797 | 0.258321151 | NS | 1.209005925 |
| CLCN6 | 0.049933848 | 0.643221762 | 0.784127485 | NS | 0.191639271 |
| NPPA | 0.280461682 | 0.475651558 | NA | NS | 0.322711076 |
| NPPB | 1.228234171 | 0.053760516 | NA | NS | 1.269536574 |
| KIAA2013 | 0.040311224 | 0.55609155 | 0.722126301 | NS | 0.254853704 |
| PLOD1 | -0.173592665 | 0.101778996 | 0.317523848 | NS | 0.992341837 |
| MFN2 | -0.10039584 | 0.292907265 | 0.515298984 | NS | 0.533269857 |
| MIIP | -0.019801442 | 0.829605635 | 0.903191142 | NS | 0.081128307 |
| TNFRSF8 | -0.152764323 | 0.044299163 | 0.225202979 | Down | 1.353604483 |
| TNFRSF1B | -0.038010413 | 0.631429138 | 0.775637745 | NS | 0.199675381 |
| VPS13D | 0.091770732 | 0.263831476 | 0.490085852 | NS | 0.578673393 |
| DHRS3 | -0.091856397 | 0.286824767 | 0.509965128 | NS | 0.542383351 |
| AADACL4 | 0.905256231 | 0.078437639 | NA | NS | 1.105475489 |
| AADACL3 | 0.376225457 | 0.387936266 | NA | NS | 0.411239619 |
| C1orf158 | 0.741854433 | 0.103942273 | NA | NS | 0.983207789 |
| PRAMEF12 | 0.684980631 | 0.159846924 | NA | NS | 0.796295717 |
| PRAMEF1 | 0.515338282 | 0.285762893 | NA | NS | 0.543994166 |
| PRAMEF15 | 0.504712041 | 0.368296376 | NA | NS | 0.433802554 |
| PRAMEF14 | 0.788863088 | 0.121589858 | NA | NS | 0.915102649 |
| PRAMEF19 | 0.789467914 | 0.17782474 | NA | NS | 0.750007818 |
| PRAMEF17 | 0.684923524 | 0.238509971 | NA | NS | 0.62249346 |
| PRAMEF20 | 0.805407437 | 0.184898856 | NA | NS | 0.733065776 |
| LRRC38 | 0.681018707 | 0.269974679 | NA | NS | 0.568676966 |
| PDPN | 0.746220611 | 0.084458305 | NA | NS | 1.073357639 |
| PRDM2 | 0.060143609 | 0.44231968 | 0.636786856 | NS | 0.354263738 |
| KAZN | 0.43490152 | 0.027287026 | 0.185919029 | Up | 1.564043797 |
| TMEM51 | 0.561197151 | 0.021463917 | 0.168494559 | Up | 1.668291029 |
| FHAD1 | 0.525489936 | 0.00696483 | 0.110157823 | Up | 2.157089503 |
| EFHD2 | 0.091248279 | 0.227846577 | 0.456610283 | NS | 0.642357491 |
| CTRC | 0.348221851 | 0.278723658 | NA | NS | 0.554826167 |
| CELA2A | 0.982031723 | 0.020447923 | NA | Up | 1.689350791 |
| CELA2B | 0.241956381 | 0.255178091 | NA | NS | 0.593156616 |
| CASP9 | 0.003715068 | 0.952635146 | 0.974466869 | NS | 0.0210734 |
| DNAJC16 | -0.108088654 | 0.083810221 | 0.291572488 | NS | 1.076703013 |
| AGMAT | -0.156515796 | 0.127449093 | 0.348830209 | NS | 0.894663251 |
| DDI2 | -0.127814419 | 0.229364843 | 0.457791831 | NS | 0.639473151 |
| RSC1A1 | 0.086204184 | 0.574064796 | 0.735427195 | NS | 0.241039085 |
| PLEKHM2 | 0.025389201 | 0.723787847 | 0.837096722 | NS | 0.140388713 |
| SLC25A34 | 0.379697973 | 0.035363442 | 0.205857644 | Up | 1.451445472 |
| TMEM82 | 1.084229986 | 0.032862361 | NA | Up | 1.483301237 |
| FBLIM1 | 0.383189054 | 0.345085586 | NA | NS | 0.46207318 |
| UQCRHL | -0.471416392 | 0.002329256 | 0.074444948 | Down | 2.632782833 |
| SPEN | -0.008651924 | 0.916143824 | 0.953437582 | NS | 0.038036342 |
| ZBTB17 | -0.015485244 | 0.79810392 | 0.883857639 | NS | 0.097940556 |
| SRARP | 0.720905862 | 0.202354882 | NA | NS | 0.693886314 |
| HSPB7 | 0.657597749 | 0.09534164 | NA | NS | 1.020717384 |
| CLCNKA | 0.657258326 | 0.145202738 | NA | NS | 0.838025195 |
| CLCNKB | 0.546567227 | 0.239563603 | 0.46765135 | NS | 0.620579164 |
| FAM131C | 0.724643617 | 0.192329686 | NA | NS | 0.715953678 |
| EPHA2 | 0.376318207 | 0.24648533 | 0.474646517 | NS | 0.608208923 |
| ARHGEF19 | 0.154205282 | 0.087115798 | 0.296673013 | NS | 1.059903079 |
| CPLANE2 | 0.056129607 | 0.599905803 | 0.75439633 | NS | 0.221916937 |
| FBXO42 | 0.057365023 | 0.119048591 | 0.339617693 | NS | 0.924275739 |
| SZRD1 | -0.007560584 | 0.914346038 | 0.95220598 | NS | 0.038889412 |
| SPATA21 | 0.104066678 | 0.523845304 | 0.699504346 | NS | 0.280796945 |
| NECAP2 | -0.017248256 | 0.7065762 | 0.826424395 | NS | 0.150840995 |
| NBPF1 | 0.092814493 | 0.340327044 | 0.556026263 | NS | 0.468103539 |
| CROCC | -0.007630279 | 0.954766723 | 0.97562483 | NS | 0.020102726 |
| MFAP2 | 0.327634005 | 0.565102859 | NA | NS | 0.247872496 |
| ATP13A2 | 0.139240345 | 0.209149673 | 0.439122277 | NS | 0.679542811 |
| SDHB | -0.123713685 | 0.096638567 | 0.310804402 | NS | 1.014849518 |
| PADI2 | -0.103454369 | 0.499774205 | 0.681502164 | NS | 0.301226163 |
| PADI4 | 0.080041217 | 0.541667323 | 0.711759999 | NS | 0.266267363 |
| PADI6 | -0.396209302 | 0.405632489 | NA | NS | 0.391867267 |
| RCC2 | -0.220567424 | 0.031576358 | 0.19723047 | Down | 1.500637968 |
| ARHGEF10L | -0.039527685 | 0.749661075 | 0.852977839 | NS | 0.125135039 |
| ACTL8 | 0.529730449 | 0.347755771 | NA | NS | 0.458725654 |
| IGSF21 | 0.827245585 | 0.06401999 | NA | NS | 1.1936844 |
| KLHDC7A | 0.357709081 | 0.285477287 | NA | NS | 0.544428439 |
| PAX7 | 0.614716015 | 0.167768343 | 0.395942971 | NS | 0.775289985 |
| TAS1R2 | 0.565439315 | 0.274498816 | NA | NS | 0.561459525 |
| ALDH4A1 | -0.118758385 | 0.213988417 | 0.44326725 | NS | 0.669609735 |
| IFFO2 | -0.227306009 | 0.004447837 | 0.09355616 | Down | 2.351851131 |
| UBR4 | 0.052252718 | 0.587223851 | 0.745735868 | NS | 0.231196313 |
| EMC1 | 0.110359582 | 0.128178706 | 0.350012309 | NS | 0.892184116 |
| MRTO4 | -0.233467641 | 0.007217148 | 0.11179811 | Down | 2.141634404 |
| AKR7A3 | 0.409897235 | 0.155861341 | NA | NS | 0.807261591 |
| AKR7A2 | -0.177283876 | 0.064497277 | 0.263488519 | NS | 1.190458621 |
| SLC66A1 | 0.159830008 | 0.074202149 | 0.276681476 | NS | 1.129583514 |
| CAPZB | 0.205845617 | 0.001321615 | 0.059001229 | Up | 2.878894985 |
| MICOS10 | -0.015929552 | 0.762833532 | 0.861842787 | NS | 0.117570225 |
| NBL1 | -0.043644199 | 0.788831324 | 0.878303028 | NS | 0.103015852 |
| MICOS10-NBL1 | 0.791905925 | 0.216154343 | NA | NS | 0.665236034 |
| HTR6 | -0.066978226 | 0.681038194 | 0.809988404 | NS | 0.166828532 |
| TMCO4 | -0.103593522 | 0.133127985 | 0.356387477 | NS | 0.875730643 |
| RNF186 | 1.218061643 | 0.019739097 | NA | Up | 1.704672711 |
| OTUD3 | 0.135757727 | 0.009470195 | 0.122534699 | Up | 2.023641056 |
| PLA2G2E | 0.43095034 | 0.37359891 | NA | NS | 0.4275944 |
| PLA2G2A | 0.942738816 | 0.087952695 | NA | NS | 1.05575085 |
| PLA2G5 | 0.491570977 | 0.246359892 | NA | NS | 0.608429994 |
| PLA2G2D | 0.476364615 | 0.247958119 | NA | NS | 0.605621667 |
| PLA2G2F | 0.687784597 | 0.066267563 | 0.2655108 | NS | 1.178698998 |
| PLA2G2C | 0.203057808 | 0.39083313 | 0.597761105 | NS | 0.408008629 |
| UBXN10 | -0.119969714 | 0.229814962 | 0.458066753 | NS | 0.638621701 |
| VWA5B1 | 0.453861282 | 0.180474063 | 0.410276616 | NS | 0.743585205 |
| CAMK2N1 | 0.172197457 | 0.439254978 | 0.634448632 | NS | 0.357283308 |
| MUL1 | -0.152012759 | 0.01553777 | 0.148281257 | Down | 1.808611309 |
| FAM43B | 1.414826442 | 0.00515101 | NA | Up | 2.288107641 |
| CDA | 0.311591573 | 0.038340433 | 0.213464789 | Up | 1.416342988 |
| PINK1 | 0.071490633 | 0.608411805 | 0.759898494 | NS | 0.215802368 |
| DDOST | -0.26208616 | 0.002368266 | 0.074605254 | Down | 2.625569588 |
| KIF17 | 1.046592542 | 0.012619535 | 0.135545097 | Up | 1.898956658 |
| SH2D5 | 1.377126825 | 8.67E-04 | NA | Up | 3.061872063 |
| HP1BP3 | -0.250697498 | 0.004476437 | 0.093796308 | Down | 2.349067505 |
| EIF4G3 | 0.044193964 | 0.535081244 | 0.70726901 | NS | 0.271580272 |
| ECE1 | 0.061666368 | 0.569071641 | 0.731710119 | NS | 0.244833056 |
| NBPF3 | -0.04312672 | 0.698386651 | 0.821449904 | NS | 0.155904071 |
| ALPL | 0.34675672 | 0.072665807 | 0.274345075 | NS | 1.138669897 |
| RAP1GAP | 0.709762756 | 0.030291922 | 0.193469884 | Up | 1.518673163 |
| USP48 | -0.002412846 | 0.959344413 | 0.977353459 | NS | 0.018025449 |
| LDLRAD2 | 0.594748595 | 0.144044232 | NA | NS | 0.841504128 |
| HSPG2 | 0.554062601 | 0.059338095 | 0.25415179 | NS | 1.226666398 |
| CELA3B | -0.036071571 | 0.954671253 | NA | NS | 0.020146155 |
| CELA3A | 0.576181802 | 0.325652261 | NA | NS | 0.487245903 |
| CDC42 | -0.086257294 | 0.204577377 | 0.434761171 | NS | 0.689142395 |
| WNT4 | 0.305753699 | 0.003444926 | 0.085194315 | Up | 2.462820149 |
| ZBTB40 | -0.085261681 | 0.205615711 | 0.435575897 | NS | 0.686943704 |
| EPHA8 | 0.937320262 | 0.034941569 | 0.20492492 | Up | 1.456657599 |
| C1QA | -0.279329493 | 0.120167308 | 0.340734879 | NS | 0.920213669 |
| C1QC | 0.785751161 | 0.031628708 | NA | Up | 1.499918547 |
| C1QB | -0.005951298 | 0.978639503 | 0.987485532 | NS | 0.009377258 |
| EPHB2 | 0.468702406 | 0.038566554 | 0.213464789 | Up | 1.413789163 |
| LACTBL1 | 0.9249682 | 0.078543916 | NA | NS | 1.104887451 |
| TEX46 | 0.805502226 | 0.023355656 | NA | Up | 1.631607931 |
| KDM1A | -0.158628008 | 0.001054345 | 0.052878189 | Down | 2.977017217 |
| LUZP1 | -0.010682547 | 0.830377679 | 0.903626518 | NS | 0.080724333 |
| HTR1D | 0.497056012 | 0.334808955 | NA | NS | 0.475202935 |
| HNRNPR | -0.187226749 | 0.003582909 | 0.086556724 | Down | 2.44576426 |
| ZNF436 | -0.163638875 | 0.248339728 | 0.476110795 | NS | 0.604953798 |
| TCEA3 | -0.33495466 | 0.056826044 | 0.250275011 | NS | 1.245452578 |
| ASAP3 | -0.51098269 | 0.007883569 | NA | Down | 2.103277126 |
| E2F2 | -0.112405466 | 0.593268895 | 0.749606805 | NS | 0.226748421 |
| ID3 | -0.133919181 | 0.346841792 | 0.561433348 | NS | 0.459868579 |
| RPL11 | -0.49632156 | 0.009196822 | 0.121258266 | Down | 2.03636224 |
| ELOA | 0.00757513 | 0.851345317 | 0.916355684 | NS | 0.069894248 |
| PITHD1 | -0.447329806 | 0.032207698 | 0.198231845 | Down | 1.492040317 |
| LYPLA2 | -0.001003624 | 0.986014426 | 0.991599806 | NS | 0.006116731 |
| GALE | -0.052097055 | 0.487501695 | 0.672373612 | NS | 0.31202387 |
| HMGCL | -0.095648969 | 0.029807676 | 0.192297294 | NS | 1.525671884 |
| FUCA1 | -0.161239202 | 0.074375907 | 0.276923592 | NS | 1.128567727 |
| CNR2 | 0.119321396 | 0.195151847 | 0.424804449 | NS | 0.709627334 |
| PNRC2 | -0.212464121 | 0.013111016 | 0.137453511 | Down | 1.882363664 |
| SRSF10 | -0.076772813 | 0.187332679 | 0.416819945 | NS | 0.727386455 |
| MYOM3 | 0.599030428 | 0.159635209 | 0.387107395 | NS | 0.796871314 |
| IL22RA1 | 0.661790745 | 0.143269612 | NA | NS | 0.843845916 |
| IFNLR1 | -0.041571104 | 0.651004384 | 0.789620058 | NS | 0.186416087 |
| GRHL3 | 0.37014044 | 0.206308738 | 0.436307756 | NS | 0.685482377 |
| STPG1 | 0.118994407 | 0.205278572 | 0.435285994 | NS | 0.687656382 |
| NIPAL3 | 0.002033139 | 0.974701102 | 0.985403414 | NS | 0.011128543 |
| RCAN3 | 0.012089858 | 0.885439642 | 0.93539087 | NS | 0.052841038 |
| NCMAP | 0.389135081 | 0.442870563 | NA | NS | 0.353723186 |
| SRRM1 | 0.044324635 | 0.407838394 | 0.611112752 | NS | 0.389511892 |
| CLIC4 | -0.022113789 | 0.792681817 | 0.880521472 | NS | 0.100901104 |
| RUNX3 | 0.093239389 | 0.375249545 | 0.585819023 | NS | 0.425679826 |
| SYF2 | -0.182301829 | 0.028556971 | 0.188856987 | Down | 1.544287861 |
| RSRP1 | 0.045982913 | 0.476808848 | 0.664589876 | NS | 0.321655694 |
| RHD | 0.610129433 | 0.013778807 | 0.141119705 | Up | 1.860788372 |
| TMEM50A | 0.009450862 | 0.885207013 | 0.935231398 | NS | 0.052955154 |
| RHCE | 0.008339795 | 0.961942903 | 0.978825093 | NS | 0.016850705 |
| MACO1 | -0.077772831 | 0.32804144 | 0.545799372 | NS | 0.48407129 |
| LDLRAP1 | -0.040460061 | 0.631694137 | 0.775715902 | NS | 0.199493154 |
| MAN1C1 | 0.102031568 | 0.307564803 | 0.528460433 | NS | 0.512063366 |
| SELENON | 0.230244696 | 0.11474599 | 0.333576117 | NS | 0.940262484 |
| MTFR1L | -0.11949875 | 0.04487813 | 0.226545246 | Down | 1.347965247 |
| AUNIP | 0.254452046 | 0.084572697 | 0.29258065 | NS | 1.072769821 |
| PAQR7 | -0.102181315 | 0.265864948 | 0.491832837 | NS | 0.575338916 |
| STMN1 | -0.352100874 | 0.003373381 | 0.085084692 | Down | 2.471934635 |
| PAFAH2 | -0.067427254 | 0.222111533 | 0.450837396 | NS | 0.65342889 |
| EXTL1 | 0.694567757 | 0.03697373 | 0.209441592 | Up | 1.432106738 |
| SLC30A2 | 0.556566586 | 0.149178647 | NA | NS | 0.826293336 |
| TRIM63 | 0.479832175 | 0.381539589 | NA | NS | 0.418460392 |
| PDIK1L | -0.213468261 | 0.012885025 | 0.136134668 | Down | 1.889914731 |
| FAM110D | 0.784086678 | 0.157921273 | NA | NS | 0.801559365 |
| C1orf232 | 0.420476083 | 0.435072272 | NA | NS | 0.361438594 |
| ZNF593OS | 0.275186495 | 0.166356394 | NA | NS | 0.778960502 |
| CNKSR1 | 0.369162523 | 0.232571001 | 0.460995419 | NS | 0.633444437 |
| CATSPER4 | 0.581685704 | 0.271735356 | NA | NS | 0.565853852 |
| CEP85 | -0.177579423 | 0.006989862 | 0.110477441 | Down | 2.155531417 |
| SH3BGRL3 | 0.240063214 | 0.208964795 | 0.438906623 | NS | 0.679926875 |
| UBXN11 | 0.037171779 | 0.748943634 | 0.852500353 | NS | 0.125550866 |
| CD52 | -0.186410807 | 0.261315564 | 0.487309587 | NS | 0.582834724 |
| CRYBG2 | 0.201902713 | 0.082021107 | 0.288503668 | NS | 1.086074375 |
| ZNF683 | 0.195700372 | 0.317185757 | 0.536617286 | NS | 0.498686323 |
| LIN28A | -0.007621864 | 0.982767521 | NA | NS | 0.007549205 |
| DHDDS | 0.128169127 | 0.004904621 | 0.096834757 | Up | 2.309394542 |
| HMGN2 | -0.123464201 | 0.034223489 | 0.203152353 | Down | 1.465675714 |
| RPS6KA1 | -0.042454951 | 0.558683395 | 0.72406582 | NS | 0.252834237 |
| ARID1A | 0.213101771 | 0.133112335 | 0.356387477 | NS | 0.875781699 |
| PIGV | -0.030811649 | 0.408991723 | 0.612044462 | NS | 0.388285481 |
| ZDHHC18 | 0.076898642 | 0.48609045 | 0.671572908 | NS | 0.313282912 |
| SFN | -0.472998435 | 0.15006336 | 0.376217962 | NS | 0.823725332 |
| GPN2 | 0.030518181 | 0.483454699 | 0.669452779 | NS | 0.315644215 |
| GPATCH3 | 0.071423936 | 0.196388199 | 0.426159043 | NS | 0.706884612 |
| NUDC | -0.145568067 | 0.038846681 | 0.21411954 | Down | 1.410646081 |
| NR0B2 | 0.264050775 | 0.422383215 | NA | NS | 0.374293348 |
| KDF1 | 0.185976451 | 0.309551749 | 0.530005545 | NS | 0.509266737 |
| TRNP1 | 0.202620981 | 0.320819534 | 0.539490663 | NS | 0.493739197 |
| TENT5B | 0.491212414 | 0.209292876 | NA | NS | 0.679245554 |
| SLC9A1 | -0.037162076 | 0.847843487 | 0.914327833 | NS | 0.071684312 |
| WDTC1 | -0.191504964 | 0.220424684 | 0.449602156 | NS | 0.656739774 |
| TMEM222 | 0.094338258 | 0.079056095 | 0.283948594 | NS | 1.10206464 |
| SYTL1 | -0.120836004 | 0.069479705 | 0.269939848 | NS | 1.158142033 |
| MAP3K6 | 0.066942324 | 0.560721195 | 0.72527035 | NS | 0.251253027 |
| FCN3 | 0.495297965 | 0.375008564 | NA | NS | 0.425958815 |
| CD164L2 | 0.805559348 | 0.101991649 | NA | NS | 0.991435387 |
| GPR3 | 0.395646218 | 0.220091364 | NA | NS | 0.657396999 |
| WASF2 | 0.210806504 | 0.156463846 | 0.383636723 | NS | 0.805585998 |
| AHDC1 | 0.210896527 | 0.320337394 | 0.539082004 | NS | 0.494392362 |
| FGR | -0.103581997 | 0.176121889 | 0.405585069 | NS | 0.754186665 |
| IFI6 | 0.694648876 | 1.45E-04 | 0.019296974 | Up | 3.838855749 |
| FAM76A | -0.184831791 | 0.006176844 | 0.105138876 | Down | 2.209233361 |
| STX12 | -0.108653763 | 0.049682652 | 0.235917914 | Down | 1.303795229 |
| PPP1R8 | -0.24381981 | 0.001805355 | 0.066048296 | Down | 2.743437457 |
| THEMIS2 | -0.113199151 | 0.182111275 | 0.412166461 | NS | 0.739663166 |
| RPA2 | -0.175872926 | 0.022898469 | 0.17233675 | Down | 1.640193559 |
| SMPDL3B | 0.050054327 | 0.788959158 | 0.878322434 | NS | 0.102945479 |
| XKR8 | 0.080214326 | 0.434557873 | 0.630833536 | NS | 0.361952377 |
| EYA3 | -0.117378439 | 0.161996436 | 0.389682175 | NS | 0.790494539 |
| PTAFR | -0.016174483 | 0.884879994 | 0.935058442 | NS | 0.053115624 |
| DNAJC8 | -0.254877759 | 0.01396611 | 0.141412627 | Down | 1.854924529 |
| ATP5IF1 | -0.187315458 | 0.213846709 | 0.443234232 | NS | 0.669897428 |
| SESN2 | 0.24436496 | 0.046389688 | 0.229315052 | Up | 1.333578547 |
| MED18 | -0.113061289 | 0.219122914 | 0.448551211 | NS | 0.659312205 |
| PHACTR4 | -0.048249071 | 0.70056814 | 0.822549366 | NS | 0.154549617 |
| RCC1 | -0.107965066 | 0.13841767 | 0.363014451 | NS | 0.858808465 |
| TRNAU1AP | -0.100656852 | 0.040859228 | 0.218105862 | Down | 1.388709845 |
| TAF12 | -0.045677255 | 0.455981693 | 0.647822129 | NS | 0.341052594 |
| RAB42 | 0.382006093 | 0.037204518 | 0.209820891 | Up | 1.429404313 |
| GMEB1 | -0.068082793 | 0.039933359 | 0.215796828 | NS | 1.398664157 |
| YTHDF2 | -0.156666787 | 0.019919643 | 0.163747187 | Down | 1.70071845 |
| OPRD1 | 0.614868442 | 0.117829661 | 0.337950518 | NS | 0.928745373 |
| EPB41 | -0.261527958 | 0.126483183 | 0.348451623 | NS | 0.897967213 |
| TMEM200B | 0.495924403 | 0.002227445 | 0.072832159 | Up | 2.652192985 |
| SRSF4 | -0.054505641 | 0.117093377 | 0.33690942 | NS | 0.931467668 |
| MECR | 0.128100593 | 0.167071527 | 0.395112774 | NS | 0.777097559 |
| PTPRU | 0.671125825 | 0.038450306 | 0.213464789 | Up | 1.415100194 |
| MATN1 | 0.330418003 | 0.30871812 | 0.529171648 | NS | 0.510437879 |
| LAPTM5 | 0.261181421 | 5.09E-04 | 0.038504109 | Up | 3.292923705 |
| SDC3 | 0.299756203 | 0.295724436 | 0.517512114 | NS | 0.529112788 |
| PUM1 | -0.003364821 | 0.936287516 | 0.964721136 | NS | 0.028590767 |
| NKAIN1 | 0.70805734 | 0.131318391 | NA | NS | 0.881674449 |
| SNRNP40 | -0.222145983 | 0.00441073 | 0.093388262 | Down | 2.355489564 |
| ZCCHC17 | -0.216124276 | 0.011326714 | 0.130616522 | Down | 1.945896051 |
| FABP3 | 0.122449744 | 0.21948239 | 0.448847618 | NS | 0.65860032 |
| SERINC2 | 0.297958601 | 0.257202047 | 0.484200085 | NS | 0.589725579 |
| TINAGL1 | 0.619039564 | 0.153786098 | NA | NS | 0.813082922 |
| HCRTR1 | 0.212964667 | 0.132723889 | 0.355930037 | NS | 0.8770509 |
| PEF1 | -0.124387285 | 0.063001316 | 0.261116791 | NS | 1.200650382 |
| COL16A1 | 0.560847441 | 0.149750124 | 0.375926488 | NS | 0.824632808 |
| ADGRB2 | 0.545863557 | 0.023457621 | 0.173939239 | Up | 1.629716037 |
| SPOCD1 | 0.375414113 | 0.076044682 | 0.279636371 | NS | 1.118931149 |
| PTP4A2 | -0.158903314 | 0.050415415 | 0.237623758 | NS | 1.297436651 |
| KHDRBS1 | 0.11244927 | 0.002879043 | 0.080409563 | Up | 2.540751854 |
| TMEM39B | 0.059363517 | 0.4526331 | 0.645312359 | NS | 0.34425369 |
| KPNA6 | 0.022158089 | 0.773936434 | 0.868209356 | NS | 0.111294708 |
| TXLNA | -0.028914216 | 0.552541211 | 0.719689405 | NS | 0.257635325 |
| CCDC28B | -0.049534114 | 0.547752854 | 0.7160589 | NS | 0.261415351 |
| IQCC | -0.090439053 | 0.421438274 | 0.621298082 | NS | 0.375266026 |
| DCDC2B | 0.273144471 | 0.054651001 | 0.24676996 | NS | 1.262401875 |
| TMEM234 | 0.106433757 | 0.22080745 | 0.449641695 | NS | 0.655986278 |
| EIF3I | -0.301176894 | 0.00282917 | 0.079855432 | Down | 2.548340921 |
| FAM167B | 0.397744091 | 0.194781433 | NA | NS | 0.710452443 |
| LCK | -0.31602612 | 7.34E-04 | 0.04469432 | Down | 3.134433228 |
| HDAC1 | -0.194678642 | 0.001008781 | 0.052507031 | Down | 2.996203273 |
| MARCKSL1 | 0.208540283 | 0.025006847 | 0.179125158 | Up | 1.601941057 |
| TSSK3 | -0.024623907 | 0.916763731 | NA | NS | 0.037742577 |
| FAM229A | 0.218941661 | 0.155284509 | 0.382558975 | NS | 0.808871866 |
| BSDC1 | -0.11516711 | 0.028823192 | 0.189577468 | Down | 1.540257924 |
| ZBTB8B | 0.682528668 | 0.124565692 | 0.346130003 | NS | 0.904601555 |
| ZBTB8A | 0.270507006 | 0.178074311 | 0.408089396 | NS | 0.749398726 |
| ZBTB8OS | -0.127946254 | 0.212524881 | 0.442396366 | NS | 0.672590219 |
| RBBP4 | -0.019117592 | 0.604258061 | 0.757278886 | NS | 0.218777547 |
| SYNC | 0.249760937 | 0.431341655 | NA | NS | 0.365178599 |
| KIAA1522 | 0.138347252 | 0.331991115 | 0.548813376 | NS | 0.478873539 |
| YARS1 | -0.181567798 | 0.011537709 | 0.131824706 | Down | 1.93788043 |
| S100PBP | 0.012167202 | 0.682417554 | 0.810825369 | NS | 0.16594981 |
| FNDC5 | 0.110388239 | 0.345492966 | 0.560413621 | NS | 0.46156079 |
| HPCA | 0.661626879 | 0.072299277 | NA | NS | 1.140866046 |
| TMEM54 | 0.619360677 | 0.176173916 | NA | NS | 0.754058391 |
| RNF19B | 0.171534984 | 0.048765184 | 0.234036095 | Up | 1.311890132 |
| AK2 | -0.140465426 | 0.006829177 | 0.109755654 | Down | 2.165631644 |
| AZIN2 | 1.50E-04 | 0.998903376 | 0.999558051 | NS | 4.77E-04 |
| TRIM62 | 0.094991713 | 0.48619949 | 0.671629213 | NS | 0.313185501 |
| ZNF362 | 0.070605225 | 0.446220587 | 0.640149328 | NS | 0.350450397 |
| A3GALT2 | 0.243523879 | 0.321054771 | NA | NS | 0.493420871 |
| PHC2 | 0.173659534 | 0.175592921 | 0.40496105 | NS | 0.755492997 |
| ZSCAN20 | -0.038411305 | 0.620037411 | 0.767572799 | NS | 0.207582106 |
| CSMD2 | 0.752660176 | 0.042044084 | 0.220751111 | Up | 1.376295103 |
| HMGB4 | 0.844694571 | 0.110510845 | NA | NS | 0.956595101 |
| C1orf94 | 0.585379011 | 0.211635761 | NA | NS | 0.674410946 |
| SMIM12 | -0.108771725 | 0.047454589 | 0.231481365 | Down | 1.323721779 |
| GJB5 | 0.46352178 | 0.206736772 | NA | NS | 0.68458227 |
| GJB4 | 0.814778813 | 0.091241405 | NA | NS | 1.039808033 |
| GJB3 | 0.928890878 | 0.008923099 | NA | Up | 2.049484276 |
| GJA4 | 0.066344819 | 0.737087073 | NA | NS | 0.132481205 |
| DLGAP3 | 0.470031477 | 0.071376299 | 0.272521361 | NS | 1.146445976 |
| TMEM35B | 0.109422768 | 0.657011289 | NA | NS | 0.182427168 |
| ZMYM6 | 0.016164068 | 0.745994945 | 0.850708372 | NS | 0.127264115 |
| ZMYM1 | -0.159173183 | 0.017396222 | 0.154727789 | Down | 1.759545065 |
| SFPQ | -0.037595775 | 0.317798059 | 0.537121802 | NS | 0.49784876 |
| ZMYM4 | -0.049351442 | 0.462443737 | 0.653153652 | NS | 0.334941098 |
| KIAA0319L | 0.208597451 | 0.011529092 | 0.131821896 | Up | 1.938204907 |
| NCDN | 0.223585537 | 0.01945873 | 0.161936818 | Up | 1.710885509 |
| TFAP2E | 0.250404444 | 0.012661015 | 0.135623278 | Up | 1.897531472 |
| PSMB2 | -0.081262169 | 0.163085426 | 0.390811178 | NS | 0.787584847 |
| C1orf216 | -0.113899561 | 0.18571953 | 0.415463304 | NS | 0.731142424 |
| CLSPN | -0.181083519 | 0.291720167 | 0.514514845 | NS | 0.535033546 |
| AGO4 | 0.052166672 | 0.532274313 | 0.70520342 | NS | 0.273864492 |
| AGO1 | 0.01411731 | 0.83209378 | 0.904614627 | NS | 0.079827725 |
| AGO3 | 0.070194019 | 0.169829311 | 0.398309642 | NS | 0.769987353 |
| TEKT2 | 0.375795573 | 0.273143981 | NA | NS | 0.563608365 |
| ADPRS | -0.216730194 | 0.001365616 | 0.060029453 | Down | 2.864671299 |
| COL8A2 | 0.128948549 | 0.203033555 | 0.432986484 | NS | 0.692432181 |
| TRAPPC3 | -0.185138065 | 0.002838281 | 0.079855432 | Down | 2.546944541 |
| MAP7D1 | 0.055437838 | 0.641862278 | 0.783197059 | NS | 0.192558147 |
| THRAP3 | -0.215833327 | 5.53E-04 | 0.040103819 | Down | 3.257000593 |
| SH3D21 | -0.086004628 | 0.546478227 | 0.714923132 | NS | 0.262427137 |
| STK40 | -0.006292686 | 0.956249157 | 0.976161497 | NS | 0.019428935 |
| LSM10 | 0.126655087 | 0.112046053 | 0.330785464 | NS | 0.950603437 |
| OSCP1 | 0.025617626 | 0.877743176 | 0.931341466 | NS | 0.056632538 |
| MRPS15 | -0.246664701 | 0.017761967 | 0.155915893 | Down | 1.750508944 |
| CSF3R | 0.180328059 | 0.161025959 | 0.388764128 | NS | 0.793104106 |
| GRIK3 | 0.72629433 | 0.063352277 | 0.261443316 | NS | 1.198237772 |
| ZC3H12A | -0.031364917 | 0.559623145 | 0.724472223 | NS | 0.252104332 |
| MEAF6 | -0.101512568 | 0.088878575 | 0.299209951 | NS | 1.051202917 |
| SNIP1 | -0.075007986 | 0.156470132 | 0.383636723 | NS | 0.80556855 |
| DNALI1 | 0.187659877 | 0.182296198 | 0.412292544 | NS | 0.739222388 |
| GNL2 | -0.156372309 | 0.015736767 | 0.148841337 | Down | 1.803084476 |
| RSPO1 | 0.872740331 | 0.083285247 | NA | NS | 1.079431923 |
| C1orf109 | -0.199233305 | 0.001303498 | 0.058839026 | Down | 2.884889554 |
| CDCA8 | -0.135538149 | 0.421834799 | 0.621413862 | NS | 0.374857596 |
| EPHA10 | 0.56480716 | 0.165484507 | 0.393472401 | NS | 0.781242661 |
| MANEAL | 0.134580408 | 0.303209164 | 0.524458861 | NS | 0.518257678 |
| YRDC | -0.200033107 | 0.012822306 | 0.136078062 | Down | 1.892033855 |
| C1orf122 | -0.056858265 | 0.461510925 | 0.652569236 | NS | 0.335818014 |
| MTF1 | 0.219698613 | 8.33E-04 | 0.048073047 | Up | 3.079181257 |
| INPP5B | -0.104589466 | 0.016942963 | 0.153154431 | Down | 1.771010645 |
| SF3A3 | -0.218040149 | 4.19E-04 | 0.034853289 | Down | 3.378296951 |
| FHL3 | -0.066412445 | 0.607298807 | 0.759189807 | NS | 0.216597572 |
| UTP11 | -0.170039991 | 0.033492864 | 0.201567051 | Down | 1.475047713 |
| POU3F1 | 0.991298154 | 0.017386767 | NA | Up | 1.759781176 |
| RRAGC | 0.018757602 | 0.762903972 | 0.861879774 | NS | 0.117530124 |
| MYCBP | -0.09324603 | 0.195049114 | 0.424782693 | NS | 0.709856019 |
| GJA9 | 0.313657538 | 0.327041213 | NA | NS | 0.485397515 |
| RHBDL2 | 0.410513856 | 0.100409038 | 0.315614575 | NS | 0.998227193 |
| AKIRIN1 | -0.12506157 | 0.03264092 | 0.19959403 | Down | 1.486237603 |
| NDUFS5 | -0.422744919 | 0.018179435 | 0.156913557 | Down | 1.740419624 |
| MACF1 | -0.002574557 | 0.969351642 | 0.982823994 | NS | 0.01351865 |
| BMP8A | 0.172340727 | 0.347692837 | 0.562229849 | NS | 0.458804256 |
| PABPC4 | -0.014173747 | 0.7933766 | 0.880651596 | NS | 0.100520613 |
| HEYL | 0.370587807 | 0.365255864 | 0.577579907 | NS | 0.437402803 |
| NT5C1A | 0.364775246 | 0.263634544 | 0.489958478 | NS | 0.578997685 |
| HPCAL4 | 0.213907694 | 0.082584648 | 0.289685039 | NS | 1.083100676 |
| PPIE | -0.056056565 | 0.396506549 | 0.601735669 | NS | 0.401749635 |
| BMP8B | -0.016558376 | 0.85163696 | 0.916355684 | NS | 0.069745499 |
| OXCT2 | 0.190226041 | 0.506289535 | NA | NS | 0.29560105 |
| TRIT1 | -0.120778247 | 0.034415409 | 0.203549976 | Down | 1.463247069 |
| MYCL | -0.152054284 | 0.10942077 | 0.327490129 | NS | 0.960900234 |
| MFSD2A | -0.251034614 | 0.0290615 | 0.190037745 | Down | 1.53668197 |
| CAP1 | 0.111933047 | 0.20333162 | 0.433423376 | NS | 0.69179508 |
| PPT1 | -0.126479505 | 0.279560421 | 0.503380199 | NS | 0.553524315 |
| RLF | -0.13054409 | 0.025866052 | 0.182170348 | Down | 1.587269853 |
| TMCO2 | 0.278014327 | 0.40509073 | NA | NS | 0.392447695 |
| ZMPSTE24 | -0.085170202 | 0.240663059 | 0.468519668 | NS | 0.618590568 |
| COL9A2 | 0.334250837 | 0.036760732 | 0.209023715 | Up | 1.434615853 |
| SMAP2 | 0.260616765 | 0.019466075 | 0.161936818 | Up | 1.710721598 |
| ZFP69B | 0.08482067 | 0.264174747 | 0.490365542 | NS | 0.5781087 |
| ZFP69 | -0.094774845 | 0.104871361 | 0.32076633 | NS | 0.979343095 |
| EXO5 | -0.053057811 | 0.421280053 | 0.621298082 | NS | 0.375429103 |
| ZNF684 | -0.13987018 | 0.099858592 | 0.314828493 | NS | 1.000614562 |
| RIMS3 | -0.028716844 | 0.782667604 | 0.874453587 | NS | 0.106422642 |
| NFYC | 0.091861307 | 0.080303334 | 0.285933942 | NS | 1.095266422 |
| KCNQ4 | 0.752799521 | 0.022704245 | 0.171834971 | Up | 1.643892934 |
| CITED4 | 0.528236123 | 0.017710196 | NA | Up | 1.751776628 |
| CTPS1 | 0.019728742 | 0.67671726 | 0.806968386 | NS | 0.169592747 |
| SLFNL1 | 0.13018678 | 0.36099774 | 0.573615336 | NS | 0.442495517 |
| SCMH1 | -0.078065141 | 0.329249202 | 0.546767111 | NS | 0.482475269 |
| EDN2 | 0.877640561 | 0.113913114 | NA | NS | 0.943426275 |
| HIVEP3 | 0.171949481 | 0.217506881 | 0.447145363 | NS | 0.662527 |
| GUCA2B | 0.573883742 | 0.351395031 | NA | NS | 0.454204384 |
| GUCA2A | 1.213647483 | 0.0536261 | NA | NS | 1.270623784 |
| FOXJ3 | 0.066976617 | 0.244230602 | 0.472173665 | NS | 0.612199921 |
| RIMKLA | 0.407736384 | 0.110246098 | 0.328790965 | NS | 0.957636772 |
| ZMYND12 | 0.153628641 | 0.450419249 | 0.643681777 | NS | 0.346383058 |
| PPCS | -0.074093781 | 0.292027243 | 0.514739316 | NS | 0.534576631 |
| CCDC30 | 0.14586822 | 0.12675678 | 0.348680896 | NS | 0.897028801 |
| PPIH | -0.074185856 | 0.282252118 | 0.505287111 | NS | 0.54936279 |
| YBX1 | -0.231779479 | 0.089808167 | 0.30028957 | NS | 1.046684169 |
| CLDN19 | 0.493558065 | 0.251139448 | NA | NS | 0.600085065 |
| P3H1 | -0.098189238 | 0.049833911 | 0.236440071 | NS | 1.30247503 |
| C1orf50 | -0.023802498 | 0.726505623 | 0.838611255 | NS | 0.13876102 |
| TMEM269 | 0.277388107 | 0.078384636 | 0.28303577 | NS | 1.105769055 |
| SVBP | -0.27379042 | 0.077902178 | 0.282355958 | NS | 1.1084504 |
| ERMAP | 0.078079695 | 0.494137102 | 0.677463766 | NS | 0.306152536 |
| ZNF691 | -0.235921939 | 6.05E-05 | 0.011445412 | Down | 4.218456602 |
| SLC2A1 | -0.133997449 | 0.222868427 | 0.451584405 | NS | 0.651951453 |
| FAM183A | 0.845753351 | 0.13414288 | NA | NS | 0.872432374 |
| EBNA1BP2 | -0.208454664 | 0.011553765 | 0.131906447 | Down | 1.937276459 |
| CFAP57 | 0.317211346 | 0.306646697 | 0.52763506 | NS | 0.51336171 |
| TMEM125 | 0.290661989 | 0.543422266 | NA | NS | 0.264862571 |
| C1orf210 | 0.635166421 | 0.209296917 | NA | NS | 0.67923717 |
| TIE1 | 0.28252949 | 0.357118197 | 0.570303392 | NS | 0.44718802 |
| MPL | 0.244509588 | 0.143580898 | 0.368733913 | NS | 0.842903334 |
| CDC20 | 0.157950649 | 0.15696451 | 0.384159619 | NS | 0.804198531 |
| ELOVL1 | -0.120716735 | 0.041537811 | 0.219609623 | Down | 1.381556397 |
| MED8 | -0.182316516 | 0.018404733 | 0.157572037 | Down | 1.735070475 |
| SZT2 | 0.025482291 | 0.818216599 | 0.896550239 | NS | 0.087131715 |
| PTPRF | -0.009733133 | 0.960327557 | 0.97787656 | NS | 0.017580609 |
| KDM4A | -0.161105039 | 0.001311621 | 0.058899483 | Down | 2.882191752 |
| ST3GAL3 | 0.193679923 | 0.108145759 | 0.325834237 | NS | 0.965990508 |
| ARTN | 0.968930517 | 0.00521798 | NA | Up | 2.282497579 |
| IPO13 | 0.063970218 | 0.365167986 | 0.577480818 | NS | 0.437507304 |
| DPH2 | -0.042944748 | 0.558544635 | 0.724008899 | NS | 0.252942115 |
| ATP6V0B | -0.041039303 | 0.679075741 | 0.808796118 | NS | 0.168081784 |
| B4GALT2 | 0.186885997 | 0.282957999 | 0.505888876 | NS | 0.548278024 |
| CCDC24 | 0.086343341 | 0.527988433 | 0.702828416 | NS | 0.277375592 |
| SLC6A9 | -0.201464513 | 0.402437775 | 0.606636604 | NS | 0.395301261 |
| KLF17 | 0.544396847 | 0.288422805 | NA | NS | 0.539970403 |
| KLF18 | 0.620818792 | 0.277580584 | NA | NS | 0.556610916 |
| DMAP1 | 0.038821663 | 0.469653456 | 0.658751023 | NS | 0.328222478 |
| ERI3 | 0.06311921 | 0.324887588 | 0.543425032 | NS | 0.48826688 |
| RNF220 | -0.126044215 | 0.024281406 | 0.176550027 | Down | 1.614726172 |
| TMEM53 | -0.095789758 | 0.254577309 | 0.48140404 | NS | 0.594180308 |
| ARMH1 | 0.152231724 | 0.123700029 | 0.344659912 | NS | 0.9076302 |
| KIF2C | 0.12252872 | 0.452556174 | 0.645277145 | NS | 0.344327506 |
| RPS8 | -0.391567548 | 0.01605483 | 0.150123175 | Down | 1.794394287 |
| BEST4 | 0.177048901 | 0.292353013 | 0.515175671 | NS | 0.534092425 |
| PLK3 | 0.008522248 | 0.942162542 | 0.968378356 | NS | 0.025874166 |
| DYNLT4 | 0.250884789 | 0.054340248 | 0.246476974 | NS | 1.264878383 |
| BTBD19 | 0.010791383 | 0.955917534 | 0.976076658 | NS | 0.019579572 |
| PTCH2 | 0.200701453 | 0.353934071 | 0.567156318 | NS | 0.451077628 |
| EIF2B3 | -0.107874843 | 0.14710098 | 0.372664177 | NS | 0.832384435 |
| HECTD3 | -0.18160729 | 0.124896235 | 0.346400199 | NS | 0.903450654 |
| UROD | -0.167176275 | 0.027844537 | 0.187062363 | Down | 1.555259992 |
| ZSWIM5 | 0.075547421 | 0.427795715 | 0.625790589 | NS | 0.36876357 |
| HPDL | 0.260083121 | 0.175617476 | 0.40496105 | NS | 0.755432269 |
| MUTYH | 0.137513743 | 0.204662556 | 0.434843849 | NS | 0.688961607 |
| TOE1 | -0.074356367 | 0.188275599 | 0.417680291 | NS | 0.725205962 |
| TESK2 | -0.054190919 | 0.355391686 | 0.568577045 | NS | 0.449292736 |
| CCDC163 | 0.507238574 | 0.024595672 | 0.17769403 | Up | 1.609141312 |
| MMACHC | 0.01797751 | 0.824506741 | 0.900078142 | NS | 0.083805789 |
| PRDX1 | -0.211140389 | 0.021130405 | 0.168202484 | Down | 1.675092182 |
| AKR1A1 | -0.094202259 | 0.187673404 | 0.417152261 | NS | 0.726597269 |
| NASP | -0.047048847 | 0.202405317 | 0.43255039 | NS | 0.693778084 |
| CCDC17 | 0.17924928 | 0.104104122 | 0.32021733 | NS | 0.982532073 |
| GPBP1L1 | -0.067943015 | 0.383645138 | 0.592463989 | NS | 0.416070301 |
| TMEM69 | -0.078504929 | 0.190578836 | 0.420149522 | NS | 0.719925331 |
| IPP | 0.047305646 | 0.274516227 | 0.499328217 | NS | 0.561431979 |
| MAST2 | 0.134190544 | 0.124989719 | 0.346425578 | NS | 0.903125708 |
| PIK3R3 | 0.169794706 | 0.09389786 | 0.307206971 | NS | 1.027344304 |
| TSPAN1 | 0.443288974 | 0.18818678 | 0.417621476 | NS | 0.725410889 |
| POMGNT1 | -0.194665231 | 6.14E-04 | 0.041332441 | Down | 3.212103436 |
| LURAP1 | 0.545843554 | 0.134558587 | NA | NS | 0.871088582 |
| RAD54L | 0.067314219 | 0.689499224 | 0.81541388 | NS | 0.161466219 |
| LRRC41 | -0.01267956 | 0.79297738 | 0.880637817 | NS | 0.100739201 |
| UQCRH | -0.306512074 | 0.043732428 | 0.224236694 | Down | 1.359196413 |
| NSUN4 | 0.066476732 | 0.19664806 | 0.426359356 | NS | 0.706310333 |
| FAAH | 0.148901821 | 0.196587857 | 0.426336336 | NS | 0.706443312 |
| DMBX1 | 0.781238054 | 0.116488365 | NA | NS | 0.93371745 |
| TMEM275 | 0.902150774 | 0.088326054 | NA | NS | 1.053911171 |
| KNCN | 0.724080087 | 0.12377169 | NA | NS | 0.907378681 |
| MKNK1 | 0.152722431 | 0.115210548 | 0.334078498 | NS | 0.938507757 |
| MOB3C | -0.002757994 | 0.957571885 | 0.976655159 | NS | 0.018828614 |
| ATPAF1 | -0.109428952 | 0.062015012 | 0.258700409 | NS | 1.207503169 |
| TEX38 | 0.412899746 | 0.070267012 | 0.271077991 | NS | 1.153248513 |
| EFCAB14 | 0.080902053 | 0.033198814 | 0.200900063 | NS | 1.478877427 |
| CYP4B1 | 0.354516369 | 0.292114422 | NA | NS | 0.534447001 |
| CYP4A11 | 0.135920735 | 0.757066129 | NA | NS | 0.120866183 |
| CYP4X1 | 0.312588343 | 0.461806404 | NA | NS | 0.335540049 |
| CYP4Z1 | 0.53880494 | 0.271421747 | NA | NS | 0.566355359 |
| CYP4A22 | 0.627966529 | 0.181364727 | 0.411330706 | NS | 0.741447175 |
| PDZK1IP1 | -0.276540757 | 0.221151974 | 0.449806618 | NS | 0.655309179 |
| TAL1 | -0.166561465 | 0.284929275 | 0.507935726 | NS | 0.545262926 |
| STIL | -0.145967127 | 0.430927309 | 0.627925 | NS | 0.365595983 |
| CMPK1 | -0.393110201 | 2.21E-04 | 0.024537741 | Down | 3.655745034 |
| FOXE3 | -0.048998998 | 0.913433016 | NA | NS | 0.039323295 |
| FOXD2 | 0.114054954 | 0.559755152 | NA | NS | 0.2520019 |
| TRABD2B | 0.658062159 | 0.121609104 | 0.34233565 | NS | 0.91503391 |
| SLC5A9 | 0.319902431 | 0.055437728 | 0.248604264 | NS | 1.256194574 |
| SPATA6 | 0.219152346 | 0.037223923 | 0.209820891 | Up | 1.429177862 |
| AGBL4 | 0.222740431 | 0.537237402 | 0.708744872 | NS | 0.26983376 |
| BEND5 | 0.017938127 | 0.88719336 | 0.936336513 | NS | 0.051981717 |
| ELAVL4 | 0.454452857 | 0.205899673 | 0.435885316 | NS | 0.686344342 |
| DMRTA2 | 1.022056977 | 0.037052722 | NA | Up | 1.431179881 |
| FAF1 | -0.147964347 | 0.02173112 | 0.169115127 | Down | 1.662917885 |
| CDKN2C | -0.112581783 | 0.37324742 | 0.583828339 | NS | 0.428003186 |
| C1orf185 | 1.02215183 | 0.065427181 | NA | NS | 1.18424179 |
| RNF11 | -0.218583814 | 0.183367287 | 0.413619384 | NS | 0.73667814 |
| TTC39A | 0.149184239 | 0.369208728 | 0.580703131 | NS | 0.432728041 |
| EPS15 | -0.112735366 | 0.023539127 | 0.17423553 | Down | 1.628209643 |
| OSBPL9 | -0.007882369 | 0.875640041 | 0.930187311 | NS | 0.057674387 |
| NRDC | -0.015167137 | 0.817252462 | 0.896051125 | NS | 0.087643762 |
| RAB3B | 0.486874519 | 0.188783707 | 0.418176271 | NS | 0.724035489 |
| TXNDC12 | 0.144774294 | 0.036381433 | 0.208301893 | Up | 1.439120203 |
| KTI12 | -0.05498477 | 0.503171716 | 0.684244941 | NS | 0.298283779 |
| BTF3L4 | -0.073522712 | 0.346580953 | 0.56126774 | NS | 0.460195308 |
| ZFYVE9 | 0.015601789 | 0.84759167 | 0.914246619 | NS | 0.07181332 |
| CC2D1B | 0.015224551 | 0.837765255 | 0.908709855 | NS | 0.076877655 |
| ORC1 | 0.103412875 | 0.40615644 | 0.609654875 | NS | 0.391306656 |
| PRPF38A | -0.057412114 | 0.127244259 | 0.348830209 | NS | 0.895361803 |
| TUT4 | 0.0051046 | 0.918450535 | 0.954891691 | NS | 0.036944228 |
| GPX7 | -0.146109161 | 0.052019293 | 0.240919284 | NS | 1.283835551 |
| SHISAL2A | 0.113097377 | 0.230913289 | 0.459299647 | NS | 0.636551073 |
| COA7 | -0.13113561 | 0.04464027 | 0.225889772 | Down | 1.35027319 |
| ZYG11B | -0.076710946 | 0.275044829 | 0.499728415 | NS | 0.560596516 |
| ZYG11A | 0.415458976 | 0.073227188 | 0.275328789 | NS | 1.135327645 |
| ECHDC2 | -0.012764899 | 0.87865144 | 0.931684267 | NS | 0.056183375 |
| SCP2 | -0.177471883 | 0.042890994 | 0.222136939 | Down | 1.367633888 |
| PODN | 0.374592641 | 0.093276228 | 0.305740971 | NS | 1.030229027 |
| SLC1A7 | 0.383473369 | 0.02359737 | 0.174388254 | Up | 1.627136403 |
| CPT2 | 0.066836606 | 0.14633147 | 0.371811513 | NS | 0.834662266 |
| CZIB | -0.137060213 | 0.021872777 | 0.169347779 | Down | 1.660096078 |
| MAGOH | -0.089780509 | 0.372034367 | 0.583021911 | NS | 0.429416939 |
| LRP8 | 0.070339889 | 0.280007535 | 0.50348007 | NS | 0.552830282 |
| DMRTB1 | 0.574571294 | 0.291886481 | NA | NS | 0.53478602 |
| GLIS1 | 0.826059705 | 0.048253551 | NA | Up | 1.316470719 |
| NDC1 | -0.197605666 | 0.008251374 | 0.116794174 | Down | 2.083473713 |
| YIPF1 | 0.090148464 | 0.268119704 | 0.493891886 | NS | 0.571671269 |
| DIO1 | 0.106803113 | 0.455568615 | 0.647513534 | NS | 0.341446203 |
| HSPB11 | 0.027598438 | 0.668898494 | 0.802299848 | NS | 0.174639782 |
| LRRC42 | 0.075408336 | 0.262325527 | 0.488556494 | NS | 0.581159447 |
| LDLRAD1 | 0.552862011 | 0.310811667 | NA | NS | 0.507502688 |
| TMEM59 | -0.15806795 | 0.055463492 | 0.248604264 | NS | 1.255992792 |
| TCEANC2 | 0.066480373 | 0.053826729 | 0.245223741 | NS | 1.269002012 |
| CDCP2 | 0.755051117 | 0.25327878 | NA | NS | 0.596401195 |
| CYB5RL | 0.201273634 | 0.076943491 | 0.281244486 | NS | 1.113828111 |
| MRPL37 | -0.166014728 | 0.033622767 | 0.201786342 | Down | 1.473366543 |
| SSBP3 | -0.1288814 | 0.374384965 | 0.585067517 | NS | 0.426681601 |
| ACOT11 | 0.405829644 | 0.024514237 | 0.177561993 | Up | 1.610581627 |
| FAM151A | 0.546248353 | 0.098896458 | NA | NS | 1.004819262 |
| MROH7 | 0.736569721 | 0.034089807 | 0.202909595 | Up | 1.467375462 |
| MROH7-TTC4 | 1.162995429 | 0.099116562 | NA | NS | 1.003853769 |
| TTC4 | 0.014791603 | 0.880071843 | 0.932281518 | NS | 0.055481874 |
| PARS2 | 0.237317259 | 0.028703659 | 0.189026296 | Up | 1.542062744 |
| TTC22 | -0.036404131 | 0.576610898 | 0.737358732 | NS | 0.239117153 |
| LEXM | -0.042778802 | 0.781568593 | 0.873812361 | NS | 0.107032901 |
| DHCR24 | 0.257094474 | 0.009248939 | 0.121396148 | Up | 2.033908082 |
| TMEM61 | 1.481223263 | 0.036154397 | NA | Up | 1.441838872 |
| BSND | 0.398386861 | 0.202334162 | 0.432475007 | NS | 0.693930786 |
| PCSK9 | 0.691440475 | 0.047565863 | 0.231629471 | Up | 1.322704616 |
| USP24 | -0.028992049 | 0.591994798 | 0.748757762 | NS | 0.227682109 |
| PLPP3 | 0.192307908 | 0.327198152 | 0.545298507 | NS | 0.485189158 |
| PRKAA2 | 0.606401695 | 0.236674134 | 0.46462634 | NS | 0.625849203 |
| FYB2 | 0.459445732 | 0.283508139 | 0.506387725 | NS | 0.547434468 |
| C8A | 0.470083189 | 0.323812104 | NA | NS | 0.489706921 |
| C8B | 0.835040257 | 0.053665211 | NA | NS | 1.270307162 |
| DAB1 | 0.253947915 | 0.186547679 | 0.415999509 | NS | 0.729210149 |
| OMA1 | -0.223893228 | 0.007508868 | 0.113019428 | Down | 2.124425555 |
| TACSTD2 | 0.259594158 | 0.339147619 | 0.554994713 | NS | 0.469611228 |
| MYSM1 | 0.048889093 | 0.294596118 | 0.516682516 | NS | 0.530772981 |
| JUN | -0.565990171 | 6.27E-04 | 0.041832319 | Down | 3.203067351 |
| FGGY | -0.050002857 | 0.577268991 | 0.737786069 | NS | 0.238621771 |
| HOOK1 | -0.093647229 | 0.383602325 | 0.592463989 | NS | 0.41611877 |
| CYP2J2 | 0.290046484 | 0.186376341 | 0.41588454 | NS | 0.729609219 |
| C1orf87 | 0.50291138 | 0.188307755 | 0.417687574 | NS | 0.725131794 |
| NFIA | 0.040294773 | 0.742317623 | 0.84888192 | NS | 0.129410229 |
| TM2D1 | 0.026265653 | 0.471845341 | 0.660546067 | NS | 0.326200328 |
| PATJ | 0.011135401 | 0.865379534 | 0.923872929 | NS | 0.06279338 |
| L1TD1 | 0.332176106 | 0.11731195 | 0.337268176 | NS | 0.930657745 |
| KANK4 | 0.581356433 | 0.158632831 | NA | NS | 0.799606925 |
| USP1 | -0.308788865 | 0.001611332 | 0.063905938 | Down | 2.792815059 |
| DOCK7 | 0.021981439 | 0.754946181 | 0.85656564 | NS | 0.122084007 |
| ANGPTL3 | 0.224731113 | 0.064323555 | 0.263154352 | NS | 1.19162996 |
| ATG4C | -0.145320585 | 0.035855219 | 0.207148636 | Down | 1.445447624 |
| FOXD3 | 0.84634425 | 0.103487007 | NA | NS | 0.985114174 |
| ALG6 | -0.048008935 | 0.244265891 | 0.472202029 | NS | 0.612137173 |
| ITGB3BP | -0.148649897 | 0.067008898 | 0.266824786 | NS | 1.173867522 |
| EFCAB7 | -0.11142311 | 0.08543364 | 0.293695772 | NS | 1.068371087 |
| PGM1 | -0.158001244 | 0.041974917 | 0.220736981 | Down | 1.377010152 |
| ROR1 | 0.405427259 | 0.147661293 | 0.373548982 | NS | 0.830733333 |
| UBE2U | 0.488585494 | 0.318088996 | NA | NS | 0.497451355 |
| CACHD1 | 0.237377614 | 0.291145033 | 0.513896227 | NS | 0.535890615 |
| RAVER2 | 0.056512111 | 0.330218687 | 0.547441347 | NS | 0.481198354 |
| JAK1 | -0.167801224 | 0.01612419 | 0.150269448 | Down | 1.792522103 |
| AK4 | 0.354620289 | 0.0924839 | 0.304670061 | NS | 1.033933867 |
| DNAJC6 | -0.513314516 | 0.009162721 | 0.121087499 | Down | 2.037975539 |
| LEPROT | 0.152043578 | 0.04240769 | 0.220932874 | Up | 1.372555384 |
| LEPR | -0.138515861 | 0.330049361 | 0.54741342 | NS | 0.481421103 |
| PDE4B | -0.02128254 | 0.758317805 | 0.858906555 | NS | 0.120148747 |
| SGIP1 | -0.032375227 | 0.913656984 | 0.951982359 | NS | 0.039216822 |
| DYNLT5 | 0.272348795 | 0.223499083 | 0.452250929 | NS | 0.650724253 |
| INSL5 | 0.467380044 | 0.538712082 | NA | NS | 0.268643284 |
| DNAI4 | -0.290124009 | 0.159748594 | 0.387272393 | NS | 0.796562956 |
| MIER1 | -0.012930588 | 0.81055513 | 0.891225388 | NS | 0.091217441 |
| SLC35D1 | 0.02808931 | 0.566179904 | 0.729939888 | NS | 0.247045549 |
| C1orf141 | 0.50153451 | 0.297959148 | NA | NS | 0.525843276 |
| IL23R | -0.090054215 | 0.64736333 | 0.787105276 | NS | 0.188851905 |
| IL12RB2 | -0.029602799 | 0.79615216 | 0.882506982 | NS | 0.099003922 |
| SERBP1 | -0.165059335 | 0.003423633 | 0.085194315 | Down | 2.465512824 |
| GADD45A | -0.108895915 | 0.450781025 | 0.643924649 | NS | 0.346034373 |
| GNG12 | 0.303083992 | 0.355738508 | NA | NS | 0.448869121 |
| DIRAS3 | 0.339616775 | 0.410962266 | NA | NS | 0.386198052 |
| WLS | 0.160700665 | 0.263941868 | 0.490237234 | NS | 0.578491713 |
| RPE65 | 0.872887711 | 0.110404356 | NA | NS | 0.957013792 |
| DEPDC1 | -0.038595784 | 0.863974723 | 0.923147761 | NS | 0.063498963 |
| LRRC7 | 0.246088339 | 0.076509847 | 0.280401425 | NS | 1.116282669 |
| LRRC40 | -0.143780016 | 0.085861815 | 0.294548726 | NS | 1.066199933 |
| SRSF11 | -0.049411397 | 0.340802611 | 0.556312287 | NS | 0.467497087 |
| ANKRD13C | -0.263909279 | 0.011278682 | 0.130167764 | Down | 1.947741654 |
| CTH | -0.26714197 | 0.105314701 | 0.32124631 | NS | 0.977510999 |
| PTGER3 | 0.56326095 | 0.023228177 | 0.173280689 | Up | 1.633984868 |
| ZRANB2 | 0.023647311 | 0.735584608 | 0.843921385 | NS | 0.133367367 |
| NEGR1 | -0.046543536 | 0.817722373 | 0.896284948 | NS | 0.08739412 |
| LRRIQ3 | -8.47E-04 | 0.995447811 | 0.997330139 | NS | 0.001981504 |
| FPGT-TNNI3K | 0.16788777 | 0.21380738 | 0.443234232 | NS | 0.669977309 |
| FPGT | 0.007032448 | 0.926947298 | 0.959843008 | NS | 0.032944957 |
| TNNI3K | 0.506619874 | 0.103073007 | NA | NS | 0.986855054 |
| LRRC53 | 0.654305091 | 0.172654356 | NA | NS | 0.76282246 |
| ERICH3 | 0.299081594 | 0.35169349 | 0.565262486 | NS | 0.45383567 |
| CRYZ | -0.033184711 | 0.746572682 | 0.851155294 | NS | 0.126927906 |
| TYW3 | -0.177490304 | 0.029737737 | 0.19220312 | Down | 1.52669209 |
| LHX8 | 0.232322445 | 0.638713049 | NA | NS | 0.194694211 |
| SLC44A5 | -0.207255506 | 0.441304982 | 0.635925671 | NS | 0.35526117 |
| ACADM | -0.060800904 | 0.412147994 | 0.614423458 | NS | 0.38494681 |
| RABGGTB | -0.091284555 | 0.141524979 | 0.365923729 | NS | 0.849166901 |
| MSH4 | 0.292454973 | 0.048022383 | 0.232763728 | Up | 1.318556293 |
| ASB17 | 0.748509373 | 0.271804639 | NA | NS | 0.565743135 |
| ST6GALNAC3 | 0.163516116 | 0.148742852 | 0.374763795 | NS | 0.827563894 |
| ST6GALNAC5 | 0.462487241 | 0.178508466 | 0.408452483 | NS | 0.748341181 |
| PIGK | -0.220338645 | 0.022231731 | 0.170639329 | Down | 1.653026724 |
| AK5 | -0.200654687 | 0.117391716 | 0.337318365 | NS | 0.93036255 |
| ZZZ3 | -0.166571822 | 0.004268605 | 0.092751043 | Down | 2.369713998 |
| USP33 | -0.165430485 | 0.010775544 | 0.12826482 | Down | 1.967560786 |
| MIGA1 | -0.052927324 | 0.332983096 | 0.549738961 | NS | 0.477577813 |
| NEXN | 0.008581522 | 0.86291326 | 0.922704331 | NS | 0.064032857 |
| FUBP1 | -0.078147401 | 0.122796879 | 0.343633202 | NS | 0.91081267 |
| DNAJB4 | -0.39582316 | 0.001698177 | 0.065122985 | Down | 2.770017038 |
| GIPC2 | 0.014002682 | 0.961471827 | 0.978517825 | NS | 0.017063437 |
| PTGFR | 0.13998804 | 0.569858732 | 0.732121898 | NS | 0.244232792 |
| IFI44L | 0.776234307 | 2.06E-04 | 0.023784694 | Up | 3.686910653 |
| IFI44 | 0.376028459 | 0.029225739 | 0.190430641 | Up | 1.534234501 |
| ADGRL4 | 0.488997104 | 0.267115431 | 0.493076543 | NS | 0.573301022 |
| ADGRL2 | 0.515951176 | 0.249816799 | 0.476907072 | NS | 0.60237836 |
| TTLL7 | 0.318631807 | 0.043253627 | 0.223075426 | Up | 1.363977469 |
| PRKACB | -0.193463866 | 0.05312131 | 0.243367273 | NS | 1.274731223 |
| SAMD13 | 0.013084339 | 0.962972049 | 0.979263171 | NS | 0.016386318 |
| DNASE2B | 0.425232097 | 0.349335245 | NA | NS | 0.456757595 |
| RPF1 | -0.216981497 | 0.028630844 | 0.189018044 | Down | 1.543165846 |
| GNG5 | 0.216805111 | 2.29E-04 | 0.025062694 | Up | 3.640699372 |
| SPATA1 | 0.2999514 | 0.005814016 | 0.102762132 | Up | 2.23552381 |
| CTBS | 0.179123273 | 0.097689356 | 0.312338632 | NS | 1.010152755 |
| SSX2IP | -0.276778131 | 0.002273792 | 0.07365544 | Down | 2.643249171 |
| LPAR3 | -0.18066481 | 0.42377229 | 0.622769058 | NS | 0.372867444 |
| MCOLN2 | 0.070469224 | 0.402413855 | 0.606636604 | NS | 0.395327075 |
| DNAI3 | 0.188557285 | 0.311789964 | 0.531453838 | NS | 0.506137868 |
| MCOLN3 | 0.179182639 | 0.152778887 | 0.37932805 | NS | 0.815936658 |
| SYDE2 | 0.068445425 | 0.549919987 | 0.717566978 | NS | 0.259700496 |
| C1orf52 | -0.155070502 | 0.013544315 | 0.139789052 | Down | 1.868242949 |
| BCL10 | -0.066281535 | 0.312103432 | 0.531854011 | NS | 0.505701455 |
| DDAH1 | 0.235354523 | 0.230185007 | 0.458531536 | NS | 0.637922968 |
| CCN1 | 0.362898007 | 0.221426624 | 0.450227637 | NS | 0.654770161 |
| ZNHIT6 | -0.02642909 | 0.615494646 | 0.765009138 | NS | 0.21077572 |
| COL24A1 | 0.183924483 | 0.330068637 | 0.54741342 | NS | 0.481395741 |
| ODF2L | -1.08E-04 | 0.998594809 | 0.999423952 | NS | 6.11E-04 |
| CLCA2 | 0.506499532 | 0.259307865 | 0.485568555 | NS | 0.586184311 |
| CLCA1 | 0.628881258 | 0.149914482 | NA | NS | 0.824156411 |
| CLCA4 | 0.178673988 | 0.602672167 | NA | NS | 0.219918865 |
| SH3GLB1 | 0.090985716 | 0.207558312 | 0.437401068 | NS | 0.68285987 |
| SELENOF | -0.193480365 | 0.067038739 | 0.266826765 | NS | 1.173674162 |
| HS2ST1 | 0.027652142 | 0.664347647 | 0.799059388 | NS | 0.177604599 |
| LMO4 | -0.021289482 | 0.700134629 | 0.822281193 | NS | 0.154818442 |
| PKN2 | -0.144911091 | 0.167907466 | 0.39601577 | NS | 0.774929991 |
| GTF2B | -0.158768215 | 0.075454451 | 0.278643389 | NS | 1.122315139 |
| KYAT3 | 0.076565373 | 0.105679918 | 0.321835351 | NS | 0.976007532 |
| RBMXL1 | -0.00168855 | 0.971330781 | 0.983614147 | NS | 0.012632848 |
| GBP3 | -0.030426231 | 0.702460793 | 0.823782045 | NS | 0.15337791 |
| GBP1 | 0.180691157 | 0.21359805 | 0.443180155 | NS | 0.670402715 |
| GBP2 | 0.014020844 | 0.886398284 | 0.935950794 | NS | 0.052371094 |
| GBP7 | 0.005475998 | 0.981475774 | NA | NS | 0.008120416 |
| GBP4 | 0.092543314 | 0.340355307 | 0.556026263 | NS | 0.468067474 |
| GBP5 | 0.161689888 | 0.252451631 | 0.479406904 | NS | 0.597821819 |
| GBP6 | 0.15608319 | 0.395090706 | 0.600626238 | NS | 0.403303186 |
| LRRC8B | 0.113876379 | 0.069766292 | 0.270202877 | NS | 1.156354361 |
| LRRC8C | 0.068837498 | 0.198393308 | 0.428291956 | NS | 0.70247298 |
| LRRC8D | -0.170419573 | 0.022033546 | 0.170172208 | Down | 1.656915595 |
| ZNF326 | -0.002019366 | 0.965847343 | 0.980793502 | NS | 0.015091511 |
| BARHL2 | 0.431194 | 0.450113915 | NA | NS | 0.346677561 |
| ZNF644 | -0.168623931 | 0.010176547 | 0.126233693 | Down | 1.992399552 |
| HFM1 | 0.217899357 | 0.415605341 | 0.61690127 | NS | 0.381318879 |
| CDC7 | -0.071339927 | 0.32959444 | 0.546983469 | NS | 0.482020123 |
| TGFBR3 | -0.091266377 | 0.376593748 | 0.586757604 | NS | 0.424126894 |
| BRDT | 0.44887354 | 0.281230473 | 0.504325448 | NS | 0.550937623 |
| EPHX4 | 0.248460344 | 0.330959301 | NA | NS | 0.480225409 |
| SETSIP | 0.326839493 | 0.514724269 | NA | NS | 0.288425354 |
| BTBD8 | -0.029507176 | 0.61991422 | 0.767514358 | NS | 0.207668402 |
| C1orf146 | 0.220480321 | 0.102895467 | 0.318705975 | NS | 0.987603758 |
| GLMN | -0.097210937 | 0.160818752 | 0.388591588 | NS | 0.793663311 |
| RPAP2 | -0.053273681 | 0.323598442 | 0.54228177 | NS | 0.489993578 |
| GFI1 | -0.054200237 | 0.548865748 | 0.716899929 | NS | 0.260533871 |
| EVI5 | -0.052821982 | 0.462089412 | 0.652975363 | NS | 0.335273983 |
| RPL5 | -0.360230518 | 0.020187273 | 0.164578764 | Down | 1.694922348 |
| DIPK1A | -0.021865598 | 0.726915984 | 0.838611255 | NS | 0.138515781 |
| MTF2 | -0.05964245 | 0.19171225 | 0.420934764 | NS | 0.717350135 |
| TMED5 | -0.072322218 | 0.283688468 | 0.506512301 | NS | 0.547158319 |
| CCDC18 | -0.558835624 | 3.29E-04 | 0.030549091 | Down | 3.48217817 |
| DR1 | 0.019627673 | 0.69844752 | 0.821479309 | NS | 0.15586622 |
| FNBP1L | -0.194725768 | 0.116699722 | 0.336518862 | NS | 0.932930178 |
| BCAR3 | 0.277677318 | 0.026796031 | 0.185022315 | Up | 1.571929525 |
| DNTTIP2 | -0.261121591 | 0.007595374 | 0.11324821 | Down | 2.119450849 |
| GCLM | -0.113647124 | 0.285529752 | 0.508649842 | NS | 0.544348632 |
| ABCA4 | 0.126765517 | 0.754146516 | 0.856082454 | NS | 0.122544271 |
| ARHGAP29 | 0.349293718 | 0.075076737 | 0.27811629 | NS | 1.12449461 |
| ABCD3 | -0.166537684 | 0.003908407 | 0.089796534 | Down | 2.408000241 |
| F3 | 0.316911144 | 0.344544909 | NA | NS | 0.462754163 |
| SLC44A3 | 0.355842911 | 0.190866896 | 0.420152441 | NS | 0.719269389 |
| CNN3 | -0.02819285 | 0.847721174 | 0.914312439 | NS | 0.071746969 |
| ALG14 | 0.006431638 | 0.953428866 | 0.974839407 | NS | 0.020711704 |
| TLCD4 | -0.361687692 | 0.061862563 | 0.258394384 | NS | 1.208572094 |
| TLCD4-RWDD3 | -0.132542711 | 0.331959205 | 0.548813376 | NS | 0.478915284 |
| RWDD3 | -0.100376151 | 0.144395084 | 0.36967266 | NS | 0.840447593 |
| PTBP2 | 0.063361393 | 0.261258132 | 0.487278404 | NS | 0.582930182 |
| DPYD | 0.049167172 | 0.473162901 | 0.66144032 | NS | 0.324989314 |
| SNX7 | 0.066684989 | 0.819273735 | NA | NS | 0.086570968 |
| PLPPR5 | 0.219976155 | 0.506813198 | NA | NS | 0.295152084 |
| PLPPR4 | 0.162744856 | 0.632077139 | NA | NS | 0.199229917 |
| PALMD | 0.418629255 | 0.152618583 | 0.379261154 | NS | 0.816392582 |
| FRRS1 | 0.19534892 | 0.130480003 | 0.35238833 | NS | 0.884456041 |
| AGL | 0.010405313 | 0.844407471 | 0.91272351 | NS | 0.073447933 |
| SLC35A3 | -0.015418039 | 0.767153143 | 0.864377708 | NS | 0.115117931 |
| MFSD14A | 0.041881229 | 0.578446412 | 0.738355798 | NS | 0.237736869 |
| SASS6 | -0.128576613 | 0.112684723 | 0.331213846 | NS | 0.948134959 |
| TRMT13 | -0.02974778 | 0.658862063 | 0.794880839 | NS | 0.181205498 |
| LRRC39 | 0.073103718 | 0.484421902 | 0.670346247 | NS | 0.31477623 |
| DBT | -0.050717043 | 0.231529809 | 0.45979109 | NS | 0.635393087 |
| RTCA | -0.058751765 | 0.198976568 | 0.428784342 | NS | 0.701198064 |
| CDC14A | 0.010163247 | 0.844543688 | 0.912766905 | NS | 0.07337788 |
| GPR88 | 0.239517076 | 0.347441212 | NA | NS | 0.459118669 |
| VCAM1 | 0.169166625 | 0.559960811 | 0.724761145 | NS | 0.251842366 |
| EXTL2 | -0.194678584 | 0.016421558 | 0.151221722 | Down | 1.784585636 |
| SLC30A7 | 0.058283169 | 0.164564831 | 0.39234476 | NS | 0.783662971 |
| DPH5 | -0.036924591 | 0.642563413 | 0.783765833 | NS | 0.192084006 |
| S1PR1 | -0.189835859 | 0.013619307 | 0.140154175 | Down | 1.865844976 |
| OLFM3 | 0.487649064 | 0.313940849 | NA | NS | 0.503152171 |
| COL11A1 | 0.398381112 | 0.371930625 | 0.582902564 | NS | 0.42953806 |
| RNPC3 | 0.103935675 | 0.035965206 | 0.207422602 | Up | 1.444117446 |
| AMY2B | -0.023093088 | 0.599951176 | 0.75439633 | NS | 0.221884091 |
| AMY2A | 0.040714107 | 0.945311483 | NA | NS | 0.024425066 |
| PRMT6 | -0.136167443 | 0.08911541 | 0.299430914 | NS | 1.050047193 |
| NTNG1 | 0.357334189 | 0.183492868 | 0.413733592 | NS | 0.736380811 |
| VAV3 | 0.058563955 | 0.352486263 | 0.566064118 | NS | 0.452857804 |
| SLC25A24 | -0.146265121 | 0.173843266 | 0.402960258 | NS | 0.759842127 |
| NBPF6 | 0.618499798 | 0.363929788 | NA | NS | 0.438982395 |
| FAM102B | -0.062689851 | 0.263103011 | 0.489264288 | NS | 0.579874182 |
| HENMT1 | -0.208770768 | 0.015683449 | 0.148636863 | Down | 1.804558426 |
| PRPF38B | 0.069554619 | 0.193902113 | 0.423210349 | NS | 0.712417458 |
| FNDC7 | 0.434625326 | 0.144008755 | NA | NS | 0.841611103 |
| STXBP3 | -0.03056089 | 0.633434352 | 0.776709642 | NS | 0.198298388 |
| AKNAD1 | 0.411185036 | 0.24957527 | NA | NS | 0.602798451 |
| GPSM2 | -0.113125866 | 0.340578252 | 0.556086414 | NS | 0.467783088 |
| CLCC1 | 0.011673312 | 0.851694491 | 0.916355684 | NS | 0.069716162 |
| WDR47 | -0.023365184 | 0.632613797 | 0.77617707 | NS | 0.19886134 |
| TAF13 | -0.106153241 | 0.245838017 | 0.47408069 | NS | 0.609350955 |
| TMEM167B | -0.05927237 | 0.468404416 | 0.657909015 | NS | 0.329379019 |
| C1orf194 | 0.2911506 | 0.096762212 | 0.310921075 | NS | 1.014294211 |
| ELAPOR1 | -0.026552666 | 0.878034458 | 0.931434766 | NS | 0.05648844 |
| SARS1 | -0.159769923 | 0.007001956 | 0.110516053 | Down | 2.154780633 |
| CELSR2 | 0.127228597 | 0.485438788 | 0.671360296 | NS | 0.313865525 |
| PSRC1 | -0.081212347 | 0.405477667 | 0.609064684 | NS | 0.392033061 |
| MYBPHL | 0.245751965 | 0.577700221 | NA | NS | 0.238297466 |
| SORT1 | 0.307484909 | 0.00663402 | 0.108543277 | Up | 2.178223205 |
| PSMA5 | -0.119945941 | 0.137889725 | 0.362607036 | NS | 0.860468094 |
| SYPL2 | 0.196692649 | 0.623561411 | NA | NS | 0.205120769 |
| ATXN7L2 | 0.163733532 | 0.224386792 | 0.45280697 | NS | 0.649002711 |
| CYB561D1 | 0.15227539 | 0.012990247 | 0.136762107 | Up | 1.886382603 |
| AMIGO1 | 0.146516774 | 0.133637585 | 0.356834322 | NS | 0.87407138 |
| GPR61 | 0.156006583 | 0.184153276 | 0.414362912 | NS | 0.734820551 |
| GNAI3 | -0.050221953 | 0.460270638 | 0.651552088 | NS | 0.336986729 |
| GNAT2 | 0.104105765 | 0.352513198 | 0.56606768 | NS | 0.452824619 |
| AMPD2 | -0.10676056 | 0.233724109 | 0.462121173 | NS | 0.631296487 |
| GSTM4 | 0.047917897 | 0.770492665 | 0.866095171 | NS | 0.113231491 |
| GSTM2 | 0.250988462 | 0.07114438 | 0.2723297 | NS | 1.1478594 |
| GSTM1 | -0.11944511 | 0.765741723 | 0.863717375 | NS | 0.115917689 |
| GSTM5 | 0.167618923 | 0.638142958 | 0.779952504 | NS | 0.195082019 |
| GSTM3 | 0.132373615 | 0.482281979 | 0.66853282 | NS | 0.316698965 |
| EPS8L3 | 0.488749224 | 0.280422671 | 0.503699249 | NS | 0.552186879 |
| CSF1 | 0.505622733 | 4.45E-04 | 0.035816595 | Up | 3.352007558 |
| AHCYL1 | -0.127511152 | 0.027635088 | 0.186639939 | Down | 1.558539143 |
| STRIP1 | -0.003289044 | 0.945302899 | 0.970256218 | NS | 0.02442901 |
| ALX3 | 0.441575835 | 0.287814173 | NA | NS | 0.540887823 |
| UBL4B | 0.366344799 | 0.580735364 | NA | NS | 0.236021727 |
| SLC6A17 | 0.674418745 | 0.128768993 | 0.350578636 | NS | 0.890188699 |
| KCNC4 | 0.198750732 | 0.261957355 | 0.488148686 | NS | 0.581769403 |
| RBM15 | -0.08882315 | 0.030490319 | 0.193861542 | NS | 1.515838039 |
| SLC16A4 | 0.059643287 | 0.461493584 | 0.652569236 | NS | 0.335834333 |
| LAMTOR5 | 0.02657285 | 0.768134378 | 0.864804079 | NS | 0.114562797 |
| PROK1 | 0.533160326 | 0.366187902 | NA | NS | 0.436296008 |
| KCNA10 | 0.466386584 | 0.409663367 | NA | NS | 0.38757287 |
| KCNA2 | 0.182214873 | 0.259800958 | 0.485843002 | NS | 0.585359252 |
| KCNA3 | -0.009394109 | 0.865108765 | 0.92378711 | NS | 0.062929288 |
| CD53 | 0.012879527 | 0.851282641 | 0.916355684 | NS | 0.069926222 |
| LRIF1 | -0.192566566 | 0.006471629 | 0.1080547 | Down | 2.188986367 |
| DRAM2 | -0.14899888 | 0.026184987 | 0.182943431 | Down | 1.581947641 |
| CEPT1 | -0.00261623 | 0.949221669 | 0.972534991 | NS | 0.022632356 |
| DENND2D | 0.024687744 | 0.633938623 | 0.776736637 | NS | 0.197952788 |
| CHI3L2 | -0.174865614 | 0.108166381 | 0.325834237 | NS | 0.9659077 |
| CHIA | 0.320130905 | 0.509167822 | 0.688449726 | NS | 0.29313905 |
| PIFO | 0.443501989 | 0.190621787 | 0.420149522 | NS | 0.719827464 |
| OVGP1 | 0.086480633 | 0.424782372 | 0.623453338 | NS | 0.371833514 |
| WDR77 | -0.015694042 | 0.786895214 | 0.877042739 | NS | 0.104083096 |
| ATP5PB | -0.231322057 | 0.043651258 | 0.224086881 | Down | 1.360003237 |
| C1orf162 | -0.045177795 | 0.52080236 | 0.696717554 | NS | 0.283327057 |
| TMIGD3 | 0.518423416 | 0.015740709 | 0.148841337 | Up | 1.802975708 |
| ADORA3 | 0.547428212 | 0.004608867 | 0.094750689 | Up | 2.336405843 |
| RAP1A | 0.057919502 | 0.210091509 | 0.440111431 | NS | 0.677591499 |
| INKA2 | 0.226507648 | 0.069607208 | 0.270056629 | NS | 1.157345785 |
| DDX20 | -0.175869874 | 0.004517838 | 0.094211252 | Down | 2.345069362 |
| KCND3 | 0.137806855 | 0.293846537 | 0.515962844 | NS | 0.531879423 |
| CTTNBP2NL | 0.060516838 | 0.576000792 | 0.737041577 | NS | 0.239576919 |
| WNT2B | 0.163081346 | 0.164599905 | 0.392382726 | NS | 0.783570421 |
| ST7L | 0.054749085 | 0.310303575 | 0.530362297 | NS | 0.508213221 |
| CAPZA1 | -0.069050352 | 0.493873012 | 0.677407901 | NS | 0.306384706 |
| MOV10 | 0.180938213 | 0.015163132 | 0.147333918 | Up | 1.81921109 |
| RHOC | -0.179749866 | 0.090283346 | 0.300982388 | NS | 1.044392354 |
| PPM1J | 0.135662474 | 0.572911842 | 0.734484271 | NS | 0.241912201 |
| TAFA3 | 0.852152203 | 0.136883261 | NA | NS | 0.863649656 |
| SLC16A1 | -0.361307307 | 0.002482773 | 0.076573431 | Down | 2.605062915 |
| LRIG2 | 0.010540363 | 0.866092279 | 0.92428916 | NS | 0.062435833 |
| MAGI3 | 0.230160577 | 0.003224106 | 0.084483388 | Up | 2.491590656 |
| PHTF1 | 0.202584408 | 0.009722469 | 0.124070824 | Up | 2.012223437 |
| RSBN1 | 0.078308872 | 0.138926883 | 0.363222438 | NS | 0.857213708 |
| PTPN22 | -0.184758226 | 0.043328627 | 0.223141939 | Down | 1.363225078 |
| BCL2L15 | 0.190377969 | 0.087485977 | 0.297087647 | NS | 1.058061556 |
| AP4B1 | -0.074170818 | 0.049636291 | 0.235795549 | NS | 1.304200676 |
| DCLRE1B | -0.175456276 | 0.143836907 | 0.368968732 | NS | 0.842129665 |
| HIPK1 | 0.034546353 | 0.727686957 | 0.838894931 | NS | 0.138055409 |
| OLFML3 | 0.567907066 | 0.006136492 | 0.104722751 | Up | 2.21207985 |
| SYT6 | 0.65103376 | 0.09109375 | NA | NS | 1.040511421 |
| TRIM33 | 0.00221381 | 0.957850747 | 0.976662809 | NS | 0.018702158 |
| BCAS2 | -0.310270784 | 0.004022381 | 0.090296145 | Down | 2.39551679 |
| DENND2C | -0.046969853 | 0.721418364 | 0.835695173 | NS | 0.141812807 |
| AMPD1 | 0.406983862 | 0.352431161 | NA | NS | 0.452925699 |
| NRAS | -0.192974998 | 0.012595285 | 0.135545097 | Down | 1.899791988 |
| CSDE1 | -0.213125267 | 0.005882995 | 0.103006385 | Down | 2.230401519 |
| SIKE1 | -0.107904034 | 0.112433221 | 0.330929727 | NS | 0.949105349 |
| SYCP1 | 0.538513134 | 0.2206026 | 0.449602156 | NS | 0.656389373 |
| TSHB | 0.580215986 | 0.011158113 | NA | Up | 1.952409258 |
| TSPAN2 | 0.101095108 | 0.387827345 | 0.595868901 | NS | 0.411361573 |
| NGF | 0.454589865 | 0.301204666 | NA | NS | 0.521138305 |
| VANGL1 | -0.030167386 | 0.70968401 | 0.828229882 | NS | 0.14893498 |
| CASQ2 | 0.443010946 | 0.323516194 | NA | NS | 0.490103975 |
| NHLH2 | 0.46352864 | 0.255105508 | 0.481968846 | NS | 0.593280164 |
| SLC22A15 | 0.135418229 | 0.16250633 | 0.390076509 | NS | 0.789129717 |
| MAB21L3 | 0.19110321 | 0.327267302 | 0.545334383 | NS | 0.485097384 |
| ATP1A1 | -0.105778007 | 0.14153185 | 0.365923729 | NS | 0.849145818 |
| CD58 | 0.014756786 | 0.840491531 | 0.910073372 | NS | 0.075466658 |
| IGSF3 | 0.506776489 | 0.086668086 | 0.295984568 | NS | 1.062140792 |
| CD2 | -0.010940529 | 0.870512432 | 0.92688994 | NS | 0.060225022 |
| PTGFRN | 0.266676351 | 0.082948164 | 0.290260272 | NS | 1.081193221 |
| CD101 | -0.032439002 | 0.7414113 | 0.848481332 | NS | 0.129940799 |
| TTF2 | 0.24215948 | 6.78E-04 | 0.042911362 | Up | 3.168592539 |
| TRIM45 | 0.28107835 | 0.062878606 | 0.260925136 | NS | 1.201497098 |
| VTCN1 | 0.632158967 | 0.207206418 | NA | NS | 0.683596797 |
| MAN1A2 | -0.281126061 | 0.017254408 | 0.154219421 | Down | 1.763099935 |
| TENT5C | -0.364109309 | 0.134486776 | 0.358023145 | NS | 0.871320419 |
| GDAP2 | 0.151527236 | 0.009077205 | 0.120653595 | Up | 2.042047868 |
| WDR3 | -0.190757121 | 0.003968108 | 0.090040171 | Down | 2.401416533 |
| SPAG17 | 0.079963484 | 0.685941456 | 0.813328047 | NS | 0.163712949 |
| TBX15 | 0.341090987 | 0.395122331 | NA | NS | 0.403268424 |
| WARS2 | -0.09230856 | 0.149953243 | 0.376107992 | NS | 0.824044138 |
| HAO2 | 0.943169913 | 0.066101316 | NA | NS | 1.179789893 |
| HSD3B2 | 0.242261487 | 0.575386503 | NA | NS | 0.24004033 |
| HSD3B1 | 0.827496459 | 0.090045152 | NA | NS | 1.045539667 |
| ZNF697 | 0.105719671 | 0.252162897 | 0.47907912 | NS | 0.598318815 |
| PHGDH | 0.249300323 | 0.151504479 | 0.377971312 | NS | 0.819574529 |
| HMGCS2 | 0.41918865 | 0.436449985 | NA | NS | 0.360065517 |
| REG4 | 0.718366213 | 0.098525739 | NA | NS | 1.006450301 |
| ADAM30 | 0.86176401 | 0.111930683 | NA | NS | 0.951050845 |
| NOTCH2 | 0.193115687 | 0.037266105 | 0.209852065 | Up | 1.428685991 |
| SEC22B | 0.016495034 | 0.727010978 | 0.838611255 | NS | 0.138459031 |
| NOTCH2NLR | -0.046626017 | 0.878301217 | 0.931631442 | NS | 0.056356516 |
| NBPF26 | -0.080127995 | 0.595931251 | 0.751211884 | NS | 0.224803839 |
| PPIAL4A | 0.432805043 | 0.36614766 | NA | NS | 0.436343738 |
| FCGR1B | 0.468248261 | 0.002617931 | 0.078024785 | Up | 2.582041858 |
| FAM72B | 0.017311447 | 0.939066201 | NA | NS | 0.02730379 |
| SRGAP2C | -0.043715222 | 0.460841971 | 0.652105895 | NS | 0.336447975 |
| H3-2 | 0.320634086 | 0.110225978 | 0.328790965 | NS | 0.957716041 |
| FAM72C | 0.421057332 | 0.28845094 | NA | NS | 0.539928042 |
| NBPF15 | 0.02553748 | 0.710708199 | 0.828971135 | NS | 0.148308674 |
| SRGAP2B | 0.043220462 | 0.621345957 | 0.768528035 | NS | 0.206666523 |
| FAM72D | 0.414521484 | 0.047308187 | NA | Up | 1.325063697 |
| NBPF20 | -0.486381052 | 0.010246635 | 0.126233693 | Down | 1.989418731 |
| GPR89A | -0.012778299 | 0.845096825 | 0.913061552 | NS | 0.07309353 |
| PDZK1 | 0.124503243 | 0.310135601 | 0.530296194 | NS | 0.508448377 |
| CD160 | -0.020976729 | 0.859579728 | 0.92064233 | NS | 0.065713835 |
| RNF115 | -0.09228823 | 0.053229765 | 0.243667415 | NS | 1.273845452 |
| POLR3C | -0.161773263 | 0.053552061 | 0.244494302 | NS | 1.271223808 |
| NUDT17 | -0.00335333 | 0.971123599 | 0.98353481 | NS | 0.012725492 |
| PIAS3 | 0.223733525 | 0.100186438 | 0.315304357 | NS | 0.999191064 |
| ANKRD35 | 0.285842283 | 0.215991291 | 0.445361714 | NS | 0.665563759 |
| ITGA10 | 0.052819935 | 0.752169281 | 0.85466151 | NS | 0.123684408 |
| PEX11B | -0.144918655 | 0.023989365 | 0.175491741 | Down | 1.619981254 |
| RBM8A | -0.260192231 | 4.28E-04 | 0.035144156 | Down | 3.368416089 |
| LIX1L | 0.010669094 | 0.835453511 | 0.90719057 | NS | 0.078077711 |
| ANKRD34A | 0.038524686 | 0.704033672 | 0.824734454 | NS | 0.152406569 |
| POLR3GL | -0.237069916 | 0.0211782 | 0.168202484 | Down | 1.674110957 |
| TXNIP | -0.066796387 | 0.414576722 | 0.616134093 | NS | 0.382395087 |
| HJV | 0.348044361 | 0.159317533 | 0.387046796 | NS | 0.797736426 |
| NBPF10 | 0.086963035 | 0.40209466 | 0.606438716 | NS | 0.395671695 |
| NOTCH2NLA | 0.199884615 | 0.21114426 | 0.44112624 | NS | 0.67542072 |
| PPIAL4H | 0.290059256 | 0.108776981 | 0.326758974 | NS | 0.963462999 |
| NBPF12 | 0.106184405 | 0.147132542 | 0.372664177 | NS | 0.832291261 |
| PRKAB2 | 0.05575271 | 0.229361889 | 0.457791831 | NS | 0.639478744 |
| FMO5 | 0.006344515 | 0.902631652 | 0.945728256 | NS | 0.044489442 |
| CHD1L | 0.150742486 | 0.143546435 | 0.368719207 | NS | 0.843007589 |
| BCL9 | 0.186032489 | 0.101081393 | 0.316641506 | NS | 0.995328784 |
| ACP6 | 0.075137026 | 0.324143385 | 0.54273518 | NS | 0.489262837 |
| GJA5 | 0.457998416 | 0.318202088 | NA | NS | 0.497296975 |
| GJA8 | 0.932478742 | 0.06214609 | NA | NS | 1.20658619 |
| GPR89B | 0.010976872 | 0.862614149 | 0.922641007 | NS | 0.064183422 |
| NBPF11 | 0.024428335 | 0.680653084 | 0.809824766 | NS | 0.167074184 |
| PPIAL4G | 0.146843608 | 0.772603972 | NA | NS | 0.112043063 |
| NBPF14 | 0.164973757 | 0.087802979 | 0.297553096 | NS | 1.05649075 |
| PDE4DIP | 0.045170381 | 0.366787527 | 0.579141844 | NS | 0.435585441 |
| NBPF9 | 0.06738318 | 0.397145612 | 0.602346279 | NS | 0.401050231 |
| NOTCH2NLC | 0.171533016 | 0.175879391 | 0.405352704 | NS | 0.754785046 |
| NBPF19 | 0.110553423 | 0.40367792 | 0.607466438 | NS | 0.393965004 |
| PPIAL4C | -0.176152541 | 0.365145565 | 0.577480818 | NS | 0.43753397 |
| FCGR1A | 0.51161749 | 0.005423939 | 0.100338483 | Up | 2.265685234 |
| H2BC18 | 0.001805879 | 0.982658311 | 0.989708157 | NS | 0.007597468 |
| H3C13 | 0.073920583 | 0.529957118 | 0.70413711 | NS | 0.275759271 |
| H4C14 | 0.187566765 | 0.356436029 | NA | NS | 0.448018404 |
| H2AC18 | 0.01834795 | 0.9831796 | NA | NS | 0.007367141 |
| H2BC21 | -0.049230955 | 0.570604289 | 0.732633258 | NS | 0.243664968 |
| H2AC20 | -0.043597516 | 0.707044428 | 0.826665892 | NS | 0.150553296 |
| H2AC21 | -0.162996826 | 0.225963823 | 0.454435762 | NS | 0.645961087 |
| BOLA1 | -0.122934684 | 0.167033755 | 0.395096697 | NS | 0.777195755 |
| SV2A | 0.295472661 | 0.175174473 | 0.404501491 | NS | 0.75652918 |
| SF3B4 | 0.194490922 | 0.028035871 | 0.187577424 | Up | 1.552285944 |
| MTMR11 | -0.147876674 | 0.066183729 | 0.2655108 | NS | 1.179248764 |
| OTUD7B | -0.070414178 | 0.322944918 | 0.541599628 | NS | 0.490871545 |
| VPS45 | -0.059336092 | 0.223994302 | 0.452443504 | NS | 0.649763029 |
| PLEKHO1 | -0.162809283 | 0.008326535 | 0.117263361 | Down | 2.079535666 |
| ANP32E | -0.299278613 | 0.003806004 | 0.088772476 | Down | 2.419530744 |
| CA14 | 0.124773271 | 0.206299721 | 0.436307756 | NS | 0.68550136 |
| APH1A | -0.198660424 | 0.012844404 | 0.136123342 | Down | 1.891286039 |
| C1orf54 | -0.012004605 | 0.956961174 | 0.976356639 | NS | 0.019105682 |
| CIART | 0.314843468 | 0.362520241 | 0.575120433 | NS | 0.440667739 |
| MRPS21 | -0.153620849 | 0.146625129 | 0.372132189 | NS | 0.833791593 |
| PRPF3 | -0.131563899 | 0.010109351 | 0.126203819 | Down | 1.995276739 |
| RPRD2 | 0.052176917 | 0.450374597 | 0.643681777 | NS | 0.346426114 |
| TARS2 | -0.230676242 | 5.52E-04 | 0.040103819 | Down | 3.257827701 |
| ECM1 | 0.375838086 | 0.244931406 | NA | NS | 0.610955525 |
| ADAMTSL4 | 0.012718432 | 0.938985515 | 0.966414088 | NS | 0.027341107 |
| MCL1 | 0.032935286 | 0.698827767 | 0.82172696 | NS | 0.155629847 |
| ENSA | 0.194701385 | 0.002902186 | 0.080662453 | Up | 2.537274775 |
| GOLPH3L | -0.132410412 | 0.041894913 | 0.220569495 | Down | 1.377838705 |
| HORMAD1 | 0.276243007 | 0.015951213 | 0.149719131 | Up | 1.797206287 |
| CTSS | -0.237630254 | 0.047362663 | 0.23126872 | Down | 1.324563884 |
| CTSK | 0.244063745 | 0.009558473 | 0.122775181 | Up | 2.019611483 |
| ARNT | -0.031506278 | 0.524249506 | 0.699759296 | NS | 0.28046197 |
| CTXND2 | 0.474918532 | 0.296297225 | NA | NS | 0.528272417 |
| SETDB1 | 0.091607293 | 0.14322551 | 0.368154582 | NS | 0.843979622 |
| CERS2 | -0.160533902 | 0.025787569 | 0.181999045 | Down | 1.5885896 |
| ANXA9 | -0.005573518 | 0.950697036 | 0.973306371 | NS | 0.02195786 |
| MINDY1 | -0.026839299 | 0.793032807 | 0.880637817 | NS | 0.100708846 |
| PRUNE1 | -0.127229173 | 0.076550764 | 0.280416761 | NS | 1.116050473 |
| BNIPL | 0.084034643 | 0.541972055 | 0.711997018 | NS | 0.266023106 |
| C1orf56 | 0.132291302 | 0.056099644 | 0.249087638 | NS | 1.251039898 |
| CDC42SE1 | -0.065456167 | 0.267725403 | 0.493675297 | NS | 0.572310418 |
| MLLT11 | -0.047905264 | 0.568459243 | 0.731396269 | NS | 0.245300668 |
| GABPB2 | -0.103737658 | 0.047928682 | 0.232603517 | Down | 1.319404513 |
| SEMA6C | 0.365553074 | 0.104730557 | 0.320720021 | NS | 0.979926585 |
| TNFAIP8L2 | -0.034839121 | 0.664641998 | 0.799245525 | NS | 0.17741222 |
| SCNM1 | -0.22545893 | 0.11012011 | 0.328638357 | NS | 0.958133365 |
| LYSMD1 | 0.172347717 | 0.081105565 | 0.28717991 | NS | 1.090949344 |
| TMOD4 | 0.161355891 | 0.133536712 | 0.356765072 | NS | 0.874399322 |
| VPS72 | -0.24690352 | 9.00E-04 | 0.050282353 | Down | 3.045560443 |
| PIP5K1A | -0.077477849 | 0.054143822 | 0.246032104 | NS | 1.266451093 |
| PSMD4 | -0.161255728 | 0.011060922 | 0.129467323 | Down | 1.956208686 |
| ZNF687 | 0.220032342 | 0.014767886 | 0.145781952 | Up | 1.830681663 |
| PI4KB | 0.016773108 | 0.775512289 | 0.86926304 | NS | 0.110411316 |
| RFX5 | -0.082135095 | 0.155399573 | 0.382714082 | NS | 0.80855018 |
| SELENBP1 | 0.057181793 | 0.787258003 | 0.87719095 | NS | 0.103882916 |
| PSMB4 | -0.162594219 | 0.069084349 | 0.269259661 | NS | 1.160620328 |
| POGZ | 0.182973082 | 0.010592269 | 0.127927347 | Up | 1.975011017 |
| CGN | 0.293564214 | 0.383112935 | 0.592321099 | NS | 0.416673184 |
| TUFT1 | -0.054410384 | 0.588803276 | 0.746871926 | NS | 0.230029782 |
| SNX27 | 0.159559088 | 0.05884003 | 0.253109387 | NS | 1.230327112 |
| CELF3 | 0.226561545 | 0.158434653 | 0.385884336 | NS | 0.800149824 |
| RIIAD1 | 0.343889204 | 0.459744946 | NA | NS | 0.337483036 |
| MRPL9 | -0.207952904 | 0.012909142 | 0.136242016 | Down | 1.889102616 |
| OAZ3 | 0.265869127 | 0.18322602 | 0.413453601 | NS | 0.737012852 |
| TDRKH | -0.118333271 | 0.096307953 | 0.310402489 | NS | 1.01633785 |
| LINGO4 | 0.468147047 | 0.177422835 | NA | NS | 0.750990485 |
| RORC | 0.056555877 | 0.648799794 | 0.788174637 | NS | 0.187889296 |
| C2CD4D | 0.484968156 | 5.94E-04 | 0.040636454 | Up | 3.226139798 |
| THEM5 | -0.186060052 | 0.066510424 | 0.265925581 | NS | 1.177110285 |
| THEM4 | -0.021626528 | 0.784532961 | 0.875595735 | NS | 0.105388805 |
| S100A10 | 0.053247698 | 0.515463665 | 0.693278651 | NS | 0.287801942 |
| S100A11 | 0.525554073 | 3.73E-04 | 0.033152046 | Up | 3.427713044 |
| TCHHL1 | 0.339686118 | 0.448745645 | NA | NS | 0.347999753 |
| TCHH | 0.837447108 | 0.06048215 | 0.256368522 | NS | 1.218372782 |
| RPTN | 1.12785171 | 0.030123292 | NA | Up | 1.521097574 |
| HRNR | 0.468283166 | 0.036535236 | 0.208452907 | Up | 1.437288087 |
| FLG | 0.554628255 | 0.172306868 | NA | NS | 0.763697412 |
| FLG2 | 0.593604369 | 0.128431676 | 0.350159792 | NS | 0.891327849 |
| CRNN | 0.031375339 | 0.956107803 | NA | NS | 0.019493137 |
| LCE5A | 0.751937593 | 0.112579538 | NA | NS | 0.948540536 |
| CRCT1 | 0.882400215 | 0.106524864 | NA | NS | 0.972549013 |
| LCE3E | 0.696470489 | 0.284279034 | NA | NS | 0.546255169 |
| LCE3D | 0.49823412 | 0.380242279 | NA | NS | 0.419939596 |
| LCE3A | 0.422160137 | 0.676080306 | NA | NS | 0.170001715 |
| LCE2D | 0.634701618 | 0.282633914 | NA | NS | 0.548775728 |
| LCE2C | 0.698049373 | 0.244736589 | NA | NS | 0.611301096 |
| LCE2B | 0.806340101 | 0.167735995 | NA | NS | 0.775373731 |
| LCE2A | 0.621221793 | 0.231134701 | NA | NS | 0.636134847 |
| LCE4A | 0.407615141 | 0.574098165 | NA | NS | 0.241013841 |
| C1orf68 | 0.187040242 | 0.770794942 | NA | NS | 0.113061144 |
| KPRP | 0.759385948 | 0.094726804 | NA | NS | 1.023527113 |
| LCE1F | 0.124754913 | 0.831848752 | NA | NS | 0.07995563 |
| LCE1E | 0.481079005 | 0.405661338 | NA | NS | 0.391836381 |
| LCE1D | 0.94558758 | 0.177665721 | NA | NS | 0.750396358 |
| LCE1C | 0.899701888 | 0.127318822 | NA | NS | 0.89510739 |
| LCE1B | 0.17280683 | 0.750232711 | NA | NS | 0.124804004 |
| LCE1A | 1.079512352 | 0.058768566 | NA | NS | 1.230854906 |
| LCE6A | 1.535061077 | 0.01767157 | NA | Up | 1.752724858 |
| SMCP | 0.467748098 | 0.455510416 | NA | NS | 0.341501688 |
| IVL | 1.053488757 | 0.049256518 | NA | Up | 1.30753629 |
| SPRR4 | 0.445324729 | 0.496601383 | NA | NS | 0.303992075 |
| SPRR1A | 0.3715768 | 0.633986338 | NA | NS | 0.197920101 |
| SPRR3 | 0.879123343 | 0.144370996 | NA | NS | 0.840520047 |
| SPRR1B | 0.611366886 | 0.396735062 | NA | NS | 0.401499416 |
| SPRR2D | 1.081293441 | 0.046279327 | NA | Up | 1.334612965 |
| SPRR2A | 0.46818738 | 0.448654665 | NA | NS | 0.348087812 |
| SPRR2B | 0.815899884 | 0.215964468 | NA | NS | 0.665617696 |
| SPRR2E | 1.179991557 | 0.034207627 | NA | Up | 1.465877049 |
| SPRR2F | 0.207609402 | 0.685496847 | NA | NS | 0.163994539 |
| SPRR2G | -0.180770351 | 0.785870891 | NA | NS | 0.104648797 |
| LELP1 | 0.425941592 | 0.531734937 | NA | NS | 0.274304804 |
| PRR9 | 1.106777927 | 0.070319142 | NA | NS | 1.15292644 |
| LORICRIN | 1.175319691 | 0.067629987 | NA | NS | 1.169860698 |
| PGLYRP3 | 0.620212277 | 0.172849177 | NA | NS | 0.762332684 |
| PGLYRP4 | 0.56394512 | 0.199084582 | NA | NS | 0.700962373 |
| S100A9 | 0.105087659 | 0.516741729 | 0.694202442 | NS | 0.286726466 |
| S100A12 | 0.191370724 | 0.351174512 | 0.565113052 | NS | 0.454477013 |
| S100A8 | -0.016919846 | 0.938541618 | 0.966130894 | NS | 0.027546465 |
| S100A7A | 0.594241235 | 0.225615048 | NA | NS | 0.646631938 |
| S100A7L2 | 0.456505254 | 0.658726406 | NA | NS | 0.181294927 |
| S100A7 | 1.356482137 | 0.016787926 | NA | Up | 1.775002954 |
| S100A6 | 0.227289272 | 0.043472613 | 0.223613314 | Up | 1.361784252 |
| S100A5 | 0.057182604 | 0.758116827 | 0.858801581 | NS | 0.120263864 |
| S100A4 | -0.163944005 | 0.100742323 | 0.316153692 | NS | 0.996788041 |
| S100A3 | 1.010197995 | 0.004588418 | NA | Up | 2.338337018 |
| S100A2 | 0.174936907 | 0.585639534 | NA | NS | 0.232369613 |
| S100A16 | 1.106814834 | 0.036063393 | NA | Up | 1.442933412 |
| S100A14 | 0.635851224 | 0.273287121 | NA | NS | 0.563380835 |
| S100A13 | 0.10639322 | 0.513820172 | 0.691921536 | NS | 0.28918885 |
| S100A1 | -0.056938566 | 0.813820229 | 0.893571334 | NS | 0.089471519 |
| CHTOP | 0.081236092 | 0.037387881 | 0.210115856 | NS | 1.427269143 |
| SNAPIN | -0.132958802 | 0.164510603 | 0.392308942 | NS | 0.783806106 |
| ILF2 | -0.209045653 | 0.017906781 | 0.156228986 | Down | 1.746982488 |
| NPR1 | 0.393175173 | 0.419552747 | 0.619739833 | NS | 0.377213431 |
| INTS3 | 0.042261681 | 0.703128926 | 0.824107404 | NS | 0.152965035 |
| SLC27A3 | -0.171662147 | 0.115447844 | 0.334470149 | NS | 0.937614172 |
| GATAD2B | -0.003906821 | 0.979714208 | 0.987953626 | NS | 0.008900594 |
| DENND4B | -0.016354646 | 0.888561018 | 0.937172484 | NS | 0.051312743 |
| CRTC2 | 0.03141977 | 0.824408424 | 0.900078142 | NS | 0.083857579 |
| SLC39A1 | 0.207611742 | 0.063624142 | 0.262071962 | NS | 1.196378059 |
| CREB3L4 | -0.119615129 | 0.137580657 | 0.362460855 | NS | 0.861442621 |
| JTB | -0.163167136 | 0.027366247 | 0.186060433 | Down | 1.562784751 |
| RAB13 | 0.096742358 | 0.708100646 | 0.82706671 | NS | 0.149905009 |
| RPS27 | -0.478962909 | 0.03636435 | 0.208256151 | Down | 1.439324169 |
| NUP210L | 0.241177731 | 0.068893442 | 0.268916491 | NS | 1.161822114 |
| TPM3 | -0.126715507 | 0.046930237 | 0.230411397 | Down | 1.328547253 |
| C1orf189 | -0.008275999 | 0.970067374 | NA | NS | 0.013198102 |
| C1orf43 | -0.074357932 | 0.222381372 | 0.451089986 | NS | 0.652901594 |
| UBAP2L | 0.047717668 | 0.617951011 | 0.766202615 | NS | 0.209045953 |
| HAX1 | -0.254451871 | 0.001804101 | 0.066048296 | Down | 2.743739207 |
| AQP10 | 0.256907082 | 0.223779002 | 0.452337751 | NS | 0.650180666 |
| ATP8B2 | -0.140934941 | 0.04671295 | 0.2299699 | Down | 1.330562708 |
| IL6R | 0.002530426 | 0.977990386 | 0.987153917 | NS | 0.009665414 |
| SHE | 0.324549662 | 0.06322721 | 0.261404175 | NS | 1.199095983 |
| TDRD10 | 0.336587695 | 0.349871301 | NA | NS | 0.45609168 |
| UBE2Q1 | -0.266842332 | 2.32E-04 | 0.025213462 | Down | 3.633720295 |
| CHRNB2 | 0.189248745 | 0.141385424 | 0.365829154 | NS | 0.849595362 |
| ADAR | 0.092508043 | 0.235926994 | 0.463687081 | NS | 0.627222366 |
| KCNN3 | 0.455639564 | 0.169677488 | 0.398151008 | NS | 0.770375773 |
| PMVK | -0.083224571 | 0.272538006 | 0.497939004 | NS | 0.564572926 |
| PBXIP1 | -0.206229943 | 0.012721328 | 0.135824642 | Down | 1.895467546 |
| PYGO2 | 0.155249789 | 0.031863173 | 0.197978516 | Up | 1.496710974 |
| SHC1 | 0.107997942 | 0.074778902 | 0.277611674 | NS | 1.126220916 |
| CKS1B | -0.122672561 | 0.350828731 | 0.56491456 | NS | 0.454904847 |
| FLAD1 | -0.143542247 | 0.007257129 | 0.111845738 | Down | 2.139235162 |
| LENEP | 0.375428646 | 0.043661681 | NA | Up | 1.359899543 |
| ZBTB7B | 0.195317562 | 0.011144929 | 0.129586317 | Up | 1.952922689 |
| DCST2 | 0.297284841 | 0.19902094 | 0.428784342 | NS | 0.701101228 |
| DCST1 | 0.302740969 | 0.28955728 | 0.512711385 | NS | 0.538265512 |
| ADAM15 | -0.140848801 | 0.13164099 | 0.354417312 | NS | 0.880608861 |
| EFNA4 | -0.183946606 | 0.023336372 | 0.173578953 | Down | 1.631966658 |
| EFNA3 | 0.732029058 | 0.053548599 | NA | NS | 1.271251885 |
| EFNA1 | 0.250741621 | 0.113848661 | 0.332423088 | NS | 0.943672073 |
| SLC50A1 | 0.014298591 | 0.868424564 | 0.925872137 | NS | 0.061267901 |
| DPM3 | 0.132026956 | 0.267940933 | 0.493873903 | NS | 0.571960934 |
| KRTCAP2 | 0.160354855 | 0.059333782 | 0.25415179 | NS | 1.226697971 |
| TRIM46 | 0.156427549 | 0.402069902 | 0.606438716 | NS | 0.395698436 |
| MUC1 | 0.338436549 | 0.292702647 | NA | NS | 0.53357335 |
| THBS3 | 0.019097511 | 0.807246082 | 0.888995469 | NS | 0.092994054 |
| MTX1 | 0.011764374 | 0.901090508 | 0.94499381 | NS | 0.045231585 |
| GBA | 0.063731569 | 0.460721968 | 0.65200856 | NS | 0.336561079 |
| FAM189B | 0.33533672 | 0.31362073 | NA | NS | 0.503595239 |
| SCAMP3 | -0.292443318 | 0.020668241 | 0.166806057 | Down | 1.684696475 |
| CLK2 | -0.217575909 | 5.59E-04 | 0.040125454 | Down | 3.252662769 |
| FDPS | -0.131165496 | 0.010541305 | 0.127927347 | Down | 1.977105637 |
| RUSC1 | 0.026212732 | 0.707962775 | 0.82706671 | NS | 0.149989577 |
| ASH1L | -0.018330252 | 0.727040839 | 0.838611255 | NS | 0.138441193 |
| MSTO1 | -0.034147331 | 0.6405315 | 0.782168094 | NS | 0.193459508 |
| YY1AP1 | -0.129123202 | 0.266201195 | 0.492079345 | NS | 0.574789999 |
| DAP3 | -0.277002591 | 8.67E-05 | 0.014340402 | Down | 4.061779581 |
| GON4L | -0.154095795 | 0.071916811 | 0.273508834 | NS | 1.14316958 |
| SYT11 | -0.152619245 | 0.078150695 | 0.28258922 | NS | 1.107067153 |
| RIT1 | -0.031534198 | 0.669801567 | 0.803046565 | NS | 0.174053841 |
| KHDC4 | 0.033837616 | 0.572909271 | 0.734484271 | NS | 0.24191415 |
| RXFP4 | -0.014550166 | 0.942728836 | 0.968743025 | NS | 0.025613209 |
| ARHGEF2 | -0.07870295 | 0.318996065 | 0.538089997 | NS | 0.496214674 |
| SSR2 | -0.182273892 | 0.07340127 | 0.275579653 | NS | 1.134296426 |
| UBQLN4 | 0.116481111 | 0.306795107 | 0.527638781 | NS | 0.513151572 |
| LAMTOR2 | -0.242654396 | 0.007316879 | 0.112308813 | Down | 2.135674153 |
| RAB25 | 0.134929808 | 0.732892449 | NA | NS | 0.134959753 |
| MEX3A | 0.586500388 | 0.101583017 | 0.317326231 | NS | 0.993178891 |
| LMNA | -0.097179735 | 0.487863567 | 0.672694188 | NS | 0.311701613 |
| SEMA4A | 0.053960117 | 0.623477154 | 0.770308731 | NS | 0.205179456 |
| SLC25A44 | 0.205604825 | 0.03685721 | 0.209195494 | Up | 1.433477546 |
| PMF1 | -0.099660988 | 0.172371078 | 0.401144432 | NS | 0.763535602 |
| BGLAP | 0.126451789 | 0.680335361 | NA | NS | 0.167276956 |
| PAQR6 | 0.365024539 | 0.027419565 | 0.186246285 | Up | 1.561939438 |
| SMG5 | -0.027727502 | 0.675060248 | 0.806240246 | NS | 0.170657465 |
| TMEM79 | 0.090175964 | 0.259760578 | 0.485843002 | NS | 0.585426759 |
| GLMP | 0.25326378 | 0.012545687 | 0.135545097 | Up | 1.90150554 |
| VHLL | 0.505587922 | 0.175140972 | NA | NS | 0.756612246 |
| CCT3 | -0.237661758 | 0.002519505 | 0.077093963 | Down | 2.598684861 |
| TSACC | 0.308028209 | 0.214779779 | NA | NS | 0.668006609 |
| RHBG | 0.834880926 | 0.100120357 | NA | NS | 0.999477611 |
| MEF2D | 0.023337801 | 0.851884429 | 0.916433852 | NS | 0.06961932 |
| IQGAP3 | 0.480836242 | 0.124420414 | 0.34606147 | NS | 0.905108359 |
| TTC24 | 0.03970987 | 0.763132686 | 0.862095634 | NS | 0.117399944 |
| NAXE | -0.174473157 | 0.022854543 | 0.172237653 | Down | 1.641027456 |
| HAPLN2 | 0.966603038 | 0.039970803 | NA | Up | 1.398257131 |
| BCAN | 0.510012453 | 0.229810937 | 0.458066753 | NS | 0.638629306 |
| NES | 0.39405862 | 0.407887686 | 0.611112752 | NS | 0.389459406 |
| CRABP2 | 0.43823435 | 0.148261934 | NA | NS | 0.828970339 |
| ISG20L2 | 0.092096587 | 0.048442783 | 0.233418183 | NS | 1.314770912 |
| METTL25B | -0.024890483 | 0.686706503 | 0.813982316 | NS | 0.16322884 |
| MRPL24 | -0.228437695 | 0.010166089 | 0.126233693 | Down | 1.992846086 |
| HDGF | -0.282973705 | 0.065472328 | 0.264686187 | NS | 1.183942219 |
| PRCC | 0.124547089 | 0.138371341 | 0.363014451 | NS | 0.858953849 |
| SH2D2A | 0.030564906 | 0.819392566 | 0.897114813 | NS | 0.086507981 |
| NTRK1 | 0.357162118 | 0.101820626 | 0.317610458 | NS | 0.992164236 |
| INSRR | 0.666950345 | 0.126931768 | 0.348807379 | NS | 0.896429672 |
| PEAR1 | 0.371906594 | 0.156420438 | 0.383636723 | NS | 0.805706503 |
| LRRC71 | 0.464195563 | 0.098070697 | 0.312859047 | NS | 1.008460739 |
| ARHGEF11 | 0.05680008 | 0.615631002 | 0.765059537 | NS | 0.210679518 |
| ETV3L | 0.514332247 | 0.210509348 | NA | NS | 0.676728613 |
| ETV3 | -0.034562556 | 0.393482266 | 0.5998058 | NS | 0.405074836 |
| FCRL5 | -0.068056798 | 0.6766124 | 0.806945644 | NS | 0.169660047 |
| FCRL4 | 0.373174786 | 0.326673183 | 0.54485998 | NS | 0.485886516 |
| FCRL3 | -0.08656966 | 0.351280438 | 0.565122842 | NS | 0.454346034 |
| FCRL2 | -0.010644693 | 0.935826524 | 0.964419702 | NS | 0.02880465 |
| FCRL1 | 0.100408085 | 0.414273216 | 0.616044456 | NS | 0.382713145 |
| CD5L | 0.299493939 | 0.409045053 | NA | NS | 0.388228855 |
| KIRREL1 | 0.657489886 | 0.119780336 | 0.340379194 | NS | 0.921614473 |
| SMIM42 | -0.070799405 | 0.913106049 | NA | NS | 0.03947878 |
| CD1D | -0.326189146 | 0.002021116 | 0.070790133 | Down | 2.694408784 |
| CD1A | -0.159401874 | 0.27455483 | 0.499328217 | NS | 0.561370912 |
| CD1C | -0.346308361 | 0.001513567 | 0.062591307 | Down | 2.819998425 |
| CD1B | 0.109140614 | 0.684900478 | NA | NS | 0.164372531 |
| CD1E | -0.083405646 | 0.702420127 | 0.823782045 | NS | 0.153403053 |
| OR10T2 | 0.514724471 | 0.494429064 | NA | NS | 0.305896008 |
| OR10K2 | 0.135960986 | 0.791276663 | NA | NS | 0.101671642 |
| OR10K1 | 0.52803178 | 0.209024973 | NA | NS | 0.679801824 |
| OR6Y1 | 0.302206814 | 0.483745356 | 0.669693293 | NS | 0.315383191 |
| OR6P1 | 0.710219671 | 0.171912445 | NA | NS | 0.764692682 |
| OR10X1 | 0.334989848 | 0.616015486 | NA | NS | 0.21040837 |
| OR10Z1 | 0.13290493 | 0.713260685 | 0.830478881 | NS | 0.146751714 |
| SPTA1 | -0.373594153 | 0.047715883 | 0.23201468 | Down | 1.321337038 |
| OR6K2 | 1.135291251 | 0.059542328 | NA | NS | 1.225174193 |
| OR6K3 | -0.192586925 | 0.53985647 | 0.710280503 | NS | 0.267721689 |
| OR6N1 | 0.199781174 | 0.439573338 | 0.634788359 | NS | 0.356968657 |
| OR6K6 | 0.70329166 | 0.055413632 | NA | NS | 1.256383384 |
| OR6N2 | 0.693434837 | 0.019934416 | 0.163751071 | Up | 1.700396493 |
| MNDA | -0.030791555 | 0.826407155 | 0.901127299 | NS | 0.082805932 |
| PYHIN1 | 0.04045367 | 0.600288037 | 0.754559383 | NS | 0.221640311 |
| IFI16 | 0.096204862 | 0.280234468 | 0.503488416 | NS | 0.552478449 |
| AIM2 | 0.140981913 | 0.227575411 | 0.456346385 | NS | 0.642874664 |
| CADM3 | 0.615404353 | 0.189919992 | NA | NS | 0.721429318 |
| ACKR1 | -0.10218309 | 0.669105498 | 0.802422061 | NS | 0.174505402 |
| FCER1A | -0.366119289 | 0.079927194 | 0.285340058 | NS | 1.097305432 |
| OR10J1 | 0.221199633 | 0.669414763 | NA | NS | 0.174304714 |
| OR10J5 | 0.89070815 | 0.147763525 | NA | NS | 0.830432756 |
| APCS | -0.188779183 | 0.790127378 | NA | NS | 0.102302889 |
| CRP | 0.788899736 | 0.175803079 | NA | NS | 0.754973523 |
| DUSP23 | -0.076087925 | 0.421681282 | 0.621413862 | NS | 0.375015676 |
| FCRL6 | -0.280558324 | 0.033448684 | 0.201567051 | Down | 1.475620967 |
| SLAMF8 | -0.177710998 | 0.299136253 | 0.520433287 | NS | 0.52413095 |
| VSIG8 | 0.565252581 | 0.118042147 | NA | NS | 0.927962901 |
| CFAP45 | -0.07457483 | 0.647277688 | 0.787042932 | NS | 0.188909363 |
| TAGLN2 | -0.013297711 | 0.888928081 | 0.93735026 | NS | 0.051133374 |
| IGSF9 | 0.554110483 | 0.03109806 | 0.195930608 | Up | 1.507266709 |
| SLAMF9 | 0.239409507 | 0.631594756 | NA | NS | 0.199561484 |
| KCNJ10 | 0.260373406 | 0.212185539 | 0.441890807 | NS | 0.673284218 |
| PIGM | 0.05997212 | 0.240927442 | 0.468835099 | NS | 0.61811373 |
| KCNJ9 | 0.451483281 | 0.21469391 | 0.443925599 | NS | 0.668180274 |
| IGSF8 | 0.036984342 | 0.691614854 | 0.81688906 | NS | 0.160135688 |
| ATP1A2 | 0.50131325 | 0.232542958 | 0.46097973 | NS | 0.633496808 |
| ATP1A4 | 0.515377982 | 0.135144601 | 0.35893236 | NS | 0.869201301 |
| CASQ1 | 0.099342941 | 0.773496581 | NA | NS | 0.111541601 |
| PEA15 | 0.025877746 | 0.711650081 | 0.82967879 | NS | 0.147733497 |
| DCAF8 | 0.041482075 | 0.34940765 | 0.563490881 | NS | 0.45666759 |
| PEX19 | -0.07092593 | 0.207025604 | 0.436958106 | NS | 0.68397594 |
| COPA | -0.0951542 | 0.096492995 | 0.310542373 | NS | 1.015504214 |
| NCSTN | 0.205710488 | 0.008504758 | 0.118000168 | Up | 2.070338027 |
| NHLH1 | 0.355149152 | 0.462320745 | NA | NS | 0.335056619 |
| VANGL2 | 0.458234899 | 0.279754369 | 0.503452347 | NS | 0.553223122 |
| SLAMF6 | -0.025264343 | 0.706266496 | 0.826364601 | NS | 0.151031396 |
| CD84 | 0.155830666 | 0.00220294 | 0.072832159 | Up | 2.656997391 |
| SLAMF1 | 0.07583992 | 0.264823959 | 0.491058839 | NS | 0.577042726 |
| CD48 | -0.269279328 | 0.034698853 | 0.204367256 | Down | 1.459684884 |
| SLAMF7 | -0.147105247 | 0.117075207 | 0.33690942 | NS | 0.931535065 |
| LY9 | 0.081420195 | 0.217408624 | 0.44703648 | NS | 0.662723232 |
| CD244 | -0.273149174 | 0.00214206 | 0.072389226 | Down | 2.669168435 |
| ITLN1 | -0.046456355 | 0.817977234 | 0.896459521 | NS | 0.087258783 |
| ITLN2 | 0.420234876 | 0.300615247 | NA | NS | 0.521988996 |
| F11R | 0.135439313 | 0.109116802 | 0.327136142 | NS | 0.962108372 |
| TSTD1 | -0.165570505 | 0.039975323 | 0.215862456 | Down | 1.398208016 |
| USF1 | -0.084386629 | 0.191931909 | 0.420934764 | NS | 0.716852818 |
| ARHGAP30 | -0.118763517 | 0.05835623 | 0.252212565 | NS | 1.233912772 |
| NECTIN4 | 0.703302789 | 0.096086184 | 0.310157264 | NS | 1.017339054 |
| KLHDC9 | 0.213942805 | 0.270047442 | 0.495546752 | NS | 0.568559932 |
| PFDN2 | -0.246583733 | 0.033598681 | 0.201786342 | Down | 1.473677767 |
| NIT1 | -0.05750733 | 0.26443353 | 0.490607316 | NS | 0.577683477 |
| DEDD | 0.169679878 | 0.031855588 | 0.197978516 | Up | 1.496814368 |
| UFC1 | -0.20381222 | 0.027154842 | 0.185717716 | Down | 1.566152713 |
| USP21 | 0.052210873 | 0.440939454 | 0.635758963 | NS | 0.35562104 |
| PPOX | -0.043173913 | 0.505763844 | 0.6861156 | NS | 0.296052221 |
| B4GALT3 | -0.079112115 | 0.483618718 | 0.669598929 | NS | 0.315496898 |
| ADAMTS4 | 0.352082315 | 0.06826632 | 0.268476763 | NS | 1.165793506 |
| NDUFS2 | -0.08384758 | 0.067314057 | 0.267225956 | NS | 1.171894232 |
| FCER1G | 0.261894581 | 0.028153533 | 0.187761274 | Up | 1.550467095 |
| APOA2 | 0.317040612 | 0.0903774 | 0.301065195 | NS | 1.043940157 |
| TOMM40L | -0.0474473 | 0.422517424 | 0.622069963 | NS | 0.374155377 |
| NR1I3 | 0.250220825 | 0.038298764 | 0.213410777 | Up | 1.416815241 |
| PCP4L1 | 0.290420686 | 0.162256533 | 0.389928636 | NS | 0.789797809 |
| MPZ | 0.038839998 | 0.717806059 | 0.833461487 | NS | 0.14399288 |
| SDHC | -0.029712313 | 0.631702595 | 0.775715902 | NS | 0.199487339 |
| CFAP126 | 0.030186316 | 0.634988391 | 0.777424576 | NS | 0.197234215 |
| FCGR2A | 0.223859952 | 0.075695284 | 0.279014573 | NS | 1.120931177 |
| HSPA6 | 0.018345776 | 0.896251664 | 0.941860029 | NS | 0.047570025 |
| FCGR3A | -0.028596322 | 0.820550227 | 0.897689944 | NS | 0.08589483 |
| FCGR3B | 0.211762183 | 0.321984957 | 0.540464635 | NS | 0.492164418 |
| FCGR2B | -0.385939341 | 1.19E-04 | 0.017390253 | Down | 3.924964323 |
| FCRLA | -0.262101602 | 0.073277524 | 0.275354648 | NS | 1.135029212 |
| FCRLB | 0.121877772 | 0.252641541 | 0.479607512 | NS | 0.597495237 |
| DUSP12 | -0.1717711 | 0.037855939 | 0.211715925 | Down | 1.421865978 |
| ATF6 | 0.128562794 | 0.091111851 | 0.302283613 | NS | 1.040425132 |
| OLFML2B | 0.156028259 | 0.425319702 | 0.62368582 | NS | 0.371284499 |
| NOS1AP | 0.453442252 | 0.233108792 | 0.461424095 | NS | 0.632441347 |
| SPATA46 | 0.351536577 | 0.389797469 | NA | NS | 0.409160985 |
| C1orf226 | 0.273479431 | 0.365937833 | NA | NS | 0.436592688 |
| SH2D1B | -0.082186524 | 0.529604923 | 0.704071643 | NS | 0.276047987 |
| UHMK1 | -0.234917222 | 0.001249233 | 0.05733452 | Down | 2.903356685 |
| UAP1 | -0.152756737 | 0.054650806 | 0.24676996 | NS | 1.262403431 |
| DDR2 | 0.292075937 | 0.128639489 | 0.350434349 | NS | 0.890625694 |
| HSD17B7 | -0.065087144 | 0.174217099 | 0.403422181 | NS | 0.758909221 |
| CCDC190 | 0.427120078 | 0.326372912 | 0.544632642 | NS | 0.486285894 |
| RGS4 | 0.608110033 | 0.200944084 | NA | NS | 0.696924776 |
| RGS5 | 0.065512884 | 0.494737492 | 0.677822328 | NS | 0.305625177 |
| NUF2 | -0.115468731 | 0.398177967 | 0.603175118 | NS | 0.399922775 |
| PBX1 | 0.053337559 | 0.749947622 | 0.85313978 | NS | 0.124969067 |
| LMX1A | 0.472302419 | 0.336296083 | NA | NS | 0.473278191 |
| RXRG | 0.781120179 | 0.11075689 | NA | NS | 0.955629246 |
| LRRC52 | 0.645133027 | 0.253809201 | NA | NS | 0.595492639 |
| MGST3 | -0.130042184 | 0.074813688 | 0.277636607 | NS | 1.126018938 |
| ALDH9A1 | -0.116489728 | 0.099731453 | 0.314784866 | NS | 1.001167855 |
| TMCO1 | -0.177826271 | 0.098106707 | 0.312866385 | NS | 1.0083013 |
| UCK2 | -0.146609455 | 0.063684534 | 0.262179256 | NS | 1.195966024 |
| FAM78B | 0.30145598 | 0.36563926 | 0.577986632 | NS | 0.436947179 |
| POGK | -0.032775856 | 0.479581713 | 0.666669523 | NS | 0.319137385 |
| TADA1 | -0.114760678 | 0.05328636 | 0.243707535 | NS | 1.273383948 |
| ILDR2 | 0.203028958 | 0.461601691 | 0.652608305 | NS | 0.335732609 |
| MAEL | 0.364830077 | 0.27456293 | 0.499328217 | NS | 0.561358099 |
| GPA33 | 0.102840165 | 0.393898633 | 0.600038083 | NS | 0.404615527 |
| STYXL2 | 0.429645458 | 0.34497024 | NA | NS | 0.46221837 |
| POU2F1 | 0.144872036 | 0.039586097 | 0.215363569 | Up | 1.402457319 |
| CD247 | -0.264936429 | 0.003455834 | 0.085194315 | Down | 2.461447083 |
| CREG1 | 0.147401974 | 0.307109914 | 0.527906189 | NS | 0.512706164 |
| RCSD1 | -0.134146062 | 0.040212179 | 0.216404071 | Down | 1.395642388 |
| MPZL1 | -0.013645951 | 0.916241849 | 0.953437582 | NS | 0.037989876 |
| ADCY10 | -0.043077078 | 0.796511512 | 0.882819855 | NS | 0.098807943 |
| MPC2 | -0.198749513 | 0.044879739 | 0.226545246 | Down | 1.347949678 |
| DCAF6 | -0.00306288 | 0.978689486 | 0.987485532 | NS | 0.009355077 |
| GPR161 | 0.3797933 | 0.04164108 | 0.22001662 | Up | 1.380478016 |
| TIPRL | -0.140878776 | 0.085670341 | 0.294200351 | NS | 1.067169507 |
| SFT2D2 | 0.122048391 | 0.050827989 | 0.238684151 | NS | 1.293897072 |
| TBX19 | 0.198899695 | 0.047741138 | 0.232088207 | Up | 1.321107233 |
| XCL2 | -0.187462902 | 0.234098501 | 0.46228643 | NS | 0.630601367 |
| XCL1 | -0.072041087 | 0.688727377 | 0.815257617 | NS | 0.161952654 |
| DPT | 0.287651234 | 0.571203608 | NA | NS | 0.243209058 |
| ATP1B1 | 0.080692698 | 0.380448229 | 0.590393369 | NS | 0.419704433 |
| NME7 | 0.069069354 | 0.258526989 | 0.485086532 | NS | 0.587494112 |
| BLZF1 | -0.039385291 | 0.520718718 | 0.696705035 | NS | 0.283396811 |
| CCDC181 | 0.262647592 | 0.036959168 | 0.209410899 | Up | 1.43227782 |
| SLC19A2 | 0.051387464 | 0.410916542 | 0.613681816 | NS | 0.386246375 |
| F5 | 0.203849856 | 0.103497915 | 0.319326135 | NS | 0.9850684 |
| SELP | 0.111693149 | 0.408322885 | 0.61140312 | NS | 0.388996279 |
| C1orf112 | 0.021993692 | 0.713387766 | 0.830478881 | NS | 0.146674343 |
| SELL | 0.085380276 | 0.467993722 | 0.657666578 | NS | 0.329759973 |
| SELE | 0.557532686 | 0.039527398 | NA | Up | 1.403101768 |
| METTL18 | -0.32806648 | 0.02079881 | 0.167428594 | Down | 1.681961509 |
| SCYL3 | 0.065269292 | 0.170895732 | 0.399291375 | NS | 0.767268783 |
| KIFAP3 | -0.093037735 | 0.086975241 | 0.29656937 | NS | 1.06060436 |
| NTMT2 | -0.186727264 | 0.749300792 | NA | NS | 0.125343808 |
| GORAB | -0.025102763 | 0.660431339 | 0.795979293 | NS | 0.180172326 |
| PRRX1 | 0.584564342 | 0.146679354 | 0.372132189 | NS | 0.833631013 |
| MROH9 | 0.558394024 | 0.069517594 | 0.26998337 | NS | 1.157905269 |
| FMO3 | 0.207009658 | 0.518172224 | NA | NS | 0.285525871 |
| FMO2 | 0.28816152 | 0.32325644 | 0.541975306 | NS | 0.490452814 |
| FMO1 | 0.283354702 | 0.417791622 | NA | NS | 0.379040273 |
| FMO4 | -0.004466104 | 0.94770029 | 0.97158694 | NS | 0.023328986 |
| PRRC2C | 0.057097261 | 0.48047657 | 0.667265443 | NS | 0.318327785 |
| MYOCOS | 0.235508286 | 0.239484632 | 0.46763242 | NS | 0.62072235 |
| MYOC | 0.794124613 | 0.212366018 | NA | NS | 0.672914977 |
| VAMP4 | -0.012459362 | 0.830632497 | 0.903626518 | NS | 0.080591082 |
| METTL13 | -0.029344062 | 0.558854878 | 0.724151228 | NS | 0.252700954 |
| DNM3 | -0.025667296 | 0.748248468 | 0.85200549 | NS | 0.125954163 |
| PIGC | -0.472857157 | 0.003074978 | 0.083174295 | Down | 2.51215804 |
| C1orf105 | -0.14142594 | 0.437032061 | 0.632874377 | NS | 0.359486701 |
| SUCO | -0.131668486 | 0.17391646 | 0.403014115 | NS | 0.759659312 |
| FASLG | 0.05085305 | 0.552500986 | 0.719677943 | NS | 0.257666942 |
| TNFSF18 | 0.546193617 | 0.054946786 | NA | NS | 1.260057708 |
| TNFSF4 | -0.138298246 | 0.198558537 | 0.428432978 | NS | 0.702111435 |
| PRDX6 | -0.352087189 | 0.009853224 | 0.125018575 | Down | 2.006421659 |
| SLC9C2 | 0.302422919 | 0.442929494 | 0.637332127 | NS | 0.353665399 |
| ANKRD45 | 0.4777256 | 0.321392621 | NA | NS | 0.492964099 |
| TEX50 | 0.759964029 | 0.222130235 | NA | NS | 0.653392324 |
| KLHL20 | -0.144486503 | 2.57E-04 | 0.026605481 | Down | 3.590561579 |
| CENPL | -0.090104427 | 0.23862466 | 0.466720558 | NS | 0.622284677 |
| DARS2 | -0.051208163 | 0.19359316 | 0.422794379 | NS | 0.713109992 |
| ZBTB37 | 0.142453653 | 0.064340483 | 0.263166811 | NS | 1.191515685 |
| SERPINC1 | 0.08510277 | 0.674428847 | 0.806015792 | NS | 0.171063862 |
| RC3H1 | 0.126585483 | 0.037063973 | 0.209518227 | Up | 1.431048028 |
| RABGAP1L | -0.117178293 | 0.174288365 | 0.403422181 | NS | 0.758731605 |
| GPR52 | 0.350000822 | 0.006415209 | 0.107862277 | Up | 2.192789159 |
| CACYBP | -0.18295133 | 0.036116651 | 0.207877243 | Down | 1.442292524 |
| MRPS14 | -0.088384519 | 0.209714004 | 0.439792418 | NS | 0.678372567 |
| TNN | 0.347519973 | 0.429121097 | 0.626769724 | NS | 0.367420133 |
| KIAA0040 | 0.087232588 | 0.273628064 | 0.498725191 | NS | 0.562839362 |
| TNR | 0.464752381 | 0.113326971 | 0.332115589 | NS | 0.945666721 |
| COP1 | 0.066929613 | 0.341715799 | 0.557088214 | NS | 0.466334941 |
| PAPPA2 | 0.46815639 | 0.204065075 | 0.434170494 | NS | 0.690231316 |
| ASTN1 | 0.554346189 | 0.205275842 | 0.435285994 | NS | 0.687662158 |
| BRINP2 | 0.894770357 | 0.074464369 | NA | NS | 1.128051485 |
| SEC16B | 0.082435538 | 0.830507289 | NA | NS | 0.080656552 |
| RASAL2 | 0.342803734 | 0.056349828 | 0.249336874 | NS | 1.249107407 |
| CLEC20A | 0.820884398 | 0.039025037 | NA | Up | 1.408656673 |
| TEX35 | 0.503001014 | 0.2354097 | NA | NS | 0.628175647 |
| RALGPS2 | -0.006587752 | 0.940960242 | 0.96744653 | NS | 0.026428726 |
| ANGPTL1 | 0.172379245 | 0.143095106 | 0.36803457 | NS | 0.844375219 |
| FAM20B | -0.112215847 | 0.141452171 | 0.365923729 | NS | 0.849390382 |
| TOR3A | -0.104901957 | 0.128213882 | 0.350012309 | NS | 0.892064949 |
| ABL2 | 0.102487714 | 0.060670754 | 0.256526216 | NS | 1.217020608 |
| SOAT1 | -0.077907958 | 0.16492523 | 0.392552916 | NS | 0.782712902 |
| AXDND1 | 0.397963503 | 0.195843365 | 0.425651579 | NS | 0.708091138 |
| NPHS2 | 1.047319692 | 0.041279199 | NA | Up | 1.384268742 |
| TDRD5 | 0.385330231 | 0.384943137 | 0.59332226 | NS | 0.414603418 |
| FAM163A | 0.785902541 | 0.069971292 | NA | NS | 1.155080107 |
| TOR1AIP2 | 0.029453849 | 0.46726295 | 0.656962127 | NS | 0.330438654 |
| TOR1AIP1 | -0.085960597 | 0.264610573 | 0.49081905 | NS | 0.577392806 |
| CEP350 | -0.045800484 | 0.287353767 | 0.510520606 | NS | 0.541583106 |
| QSOX1 | 0.029900871 | 0.760843416 | 0.860315785 | NS | 0.118704713 |
| LHX4 | 0.191663023 | 0.050744602 | 0.238451278 | NS | 1.294610146 |
| ACBD6 | -0.052141831 | 0.286089126 | 0.509186875 | NS | 0.543498649 |
| XPR1 | -0.090234596 | 0.179909605 | 0.409996481 | NS | 0.74494565 |
| KIAA1614 | 0.56300496 | 0.01334282 | 0.138543685 | Up | 1.874752359 |
| STX6 | -0.058154656 | 0.228149304 | 0.456877344 | NS | 0.641780852 |
| MR1 | 0.03097025 | 0.567845321 | 0.731017062 | NS | 0.245769949 |
| IER5 | -0.177708166 | 0.076293313 | 0.279989714 | NS | 1.117513523 |
| CACNA1E | 0.322704607 | 0.06364757 | 0.26212132 | NS | 1.196218169 |
| ZNF648 | 0.689339044 | 0.117116799 | NA | NS | 0.931380804 |
| GLUL | -0.246568363 | 0.043235194 | 0.223062043 | Down | 1.364162589 |
| TEDDM1 | 0.098551754 | 0.492819684 | 0.676692631 | NS | 0.307311954 |
| RGSL1 | 0.341061071 | 0.290751469 | 0.513652722 | NS | 0.536478082 |
| RNASEL | 0.030862111 | 0.713032136 | 0.830448491 | NS | 0.146890896 |
| RGS16 | 0.371997272 | 0.03555626 | 0.206049964 | Up | 1.449083932 |
| RGS8 | 0.589209931 | 0.213433651 | 0.443180155 | NS | 0.670737106 |
| NPL | -0.117902432 | 0.262210126 | 0.488460737 | NS | 0.581350541 |
| DHX9 | -0.142881326 | 0.003359887 | 0.085084692 | Down | 2.473675287 |
| SHCBP1L | 0.628764417 | 0.182833833 | NA | NS | 0.737943435 |
| LAMC1 | 0.015845305 | 0.895615378 | 0.941415199 | NS | 0.047878458 |
| LAMC2 | 0.566500603 | 0.203207931 | 0.433200041 | NS | 0.692059346 |
| NMNAT2 | 0.511104579 | 0.194944941 | 0.424620048 | NS | 0.710088032 |
| SMG7 | 0.110422962 | 0.280515761 | 0.503714282 | NS | 0.552042733 |
| NCF2 | 0.051806562 | 0.613210776 | 0.763371918 | NS | 0.212390222 |
| ARPC5 | -0.054554172 | 0.587969082 | 0.746351175 | NS | 0.230645511 |
| RGL1 | 0.173750476 | 0.0887953 | 0.29914533 | NS | 1.051610022 |
| APOBEC4 | 0.045107687 | 0.799184236 | 0.884455271 | NS | 0.097353091 |
| COLGALT2 | 0.07235825 | 0.569886345 | 0.732121898 | NS | 0.244211749 |
| TSEN15 | -0.124140296 | 0.073528738 | 0.275712399 | NS | 1.133542887 |
| C1orf21 | -0.203971267 | 0.07030301 | 0.271077991 | NS | 1.15302608 |
| EDEM3 | -0.025505982 | 0.588295537 | 0.746703583 | NS | 0.230404446 |
| NIBAN1 | -0.005795389 | 0.965454023 | 0.980611497 | NS | 0.015268403 |
| RNF2 | -0.184541783 | 0.007415033 | 0.112612127 | Down | 2.129886908 |
| TRMT1L | 0.034712002 | 0.579888347 | 0.73941889 | NS | 0.236655618 |
| SWT1 | -0.491676511 | 0.001802257 | 0.066048296 | Down | 2.744183283 |
| IVNS1ABP | 0.01092164 | 0.851479311 | 0.916355684 | NS | 0.0698259 |
| HMCN1 | 0.349665795 | 0.199352188 | 0.428932451 | NS | 0.700378994 |
| PRG4 | 0.108002922 | 0.364259997 | 0.576482789 | NS | 0.438588521 |
| TPR | -0.07423736 | 0.192298906 | 0.421075584 | NS | 0.716023187 |
| ODR4 | -0.114315587 | 0.032940523 | 0.200078102 | Down | 1.482269506 |
| PDC | 0.291756862 | 0.065767545 | 0.265127437 | NS | 1.181988367 |
| PTGS2 | -0.076588965 | 0.459090904 | 0.650509495 | NS | 0.338101312 |
| PLA2G4A | -0.040644751 | 0.646503998 | 0.786436212 | NS | 0.189428785 |
| BRINP3 | 0.604570246 | 0.223790194 | NA | NS | 0.650158948 |
| RGS18 | -0.205666616 | 0.076199237 | 0.279935021 | NS | 1.118049379 |
| RGS21 | 0.61249999 | 0.161384291 | NA | NS | 0.792138743 |
| RGS1 | -0.239305388 | 0.162768499 | 0.390501169 | NS | 0.788429642 |
| RGS13 | 0.202450023 | 0.389499685 | 0.59694612 | NS | 0.409492889 |
| RPS27AP5 | -0.010753929 | 0.968274427 | NA | NS | 0.014001538 |
| RGS2 | -4.47E-04 | 0.997339528 | 0.998604025 | NS | 0.001156968 |
| UCHL5 | -0.131334142 | 0.025012761 | 0.179125158 | Down | 1.601838364 |
| RO60 | -0.166576681 | 0.004300929 | 0.092915457 | Down | 2.366437741 |
| GLRX2 | -0.086309046 | 0.14863059 | 0.374763795 | NS | 0.827891799 |
| CDC73 | -0.011971184 | 0.782874924 | 0.874453587 | NS | 0.106307617 |
| B3GALT2 | 0.066188575 | 0.358971474 | 0.571841543 | NS | 0.444940062 |
| KCNT2 | 0.609636615 | 0.087935 | 0.297765891 | NS | 1.055838233 |
| CFH | -0.308216301 | 0.017782031 | 0.155923237 | Down | 1.750018631 |
| CFHR3 | 0.069956633 | 0.903350127 | NA | NS | 0.04414389 |
| CFHR1 | 0.471993816 | 0.385937002 | NA | NS | 0.413483581 |
| CFHR4 | 0.601706276 | 0.325591196 | NA | NS | 0.487327347 |
| CFHR2 | 0.612617623 | 0.337311951 | NA | NS | 0.471968271 |
| CFHR5 | 0.605258135 | 0.254334652 | NA | NS | 0.594594465 |
| F13B | 0.533791966 | 0.32623117 | NA | NS | 0.486474546 |
| ASPM | -0.258919123 | 0.065158143 | 0.264255675 | NS | 1.1860313 |
| ZBTB41 | -0.062949798 | 0.435136298 | 0.631046553 | NS | 0.361374687 |
| CRB1 | 0.10138811 | 0.708692591 | 0.827410161 | NS | 0.149542108 |
| DENND1B | -0.04323559 | 0.457704518 | 0.649222647 | NS | 0.339414801 |
| C1orf53 | 0.435574465 | 0.20159916 | NA | NS | 0.695511282 |
| LHX9 | 0.524575425 | 0.176444984 | 0.40604331 | NS | 0.753390684 |
| NEK7 | -0.119653191 | 0.078067139 | 0.282523416 | NS | 1.107531739 |
| PTPRC | -0.046461899 | 0.590562729 | 0.747833322 | NS | 0.228733966 |
| NR5A2 | 0.722714027 | 0.042095016 | 0.220751111 | Up | 1.375769321 |
| ZNF281 | -0.016239464 | 0.851238468 | 0.916355684 | NS | 0.069948759 |
| KIF14 | -0.793749079 | 2.33E-04 | 0.025213462 | Down | 3.633012113 |
| DDX59 | -0.030498261 | 0.578423129 | 0.738355798 | NS | 0.23775435 |
| CAMSAP2 | 0.084178904 | 0.315945753 | 0.535429157 | NS | 0.500387478 |
| GPR25 | 0.536758582 | 0.03336856 | NA | Up | 1.476662541 |
| INAVA | 0.943038142 | 0.014545069 | 0.144454103 | Up | 1.837284224 |
| KIF21B | -0.004035445 | 0.972378736 | 0.984309822 | NS | 0.012164547 |
| CACNA1S | 0.630763406 | 0.164472583 | 0.392288179 | NS | 0.783906488 |
| ASCL5 | 0.19451367 | 0.376151008 | NA | NS | 0.42463777 |
| TMEM9 | -0.141803867 | 0.068887591 | 0.268916491 | NS | 1.161858999 |
| IGFN1 | 0.529696833 | 0.201634769 | 0.431600737 | NS | 0.695434578 |
| PKP1 | 0.621215897 | 0.141968594 | 0.3663829 | NS | 0.847807717 |
| TNNT2 | 0.519047792 | 0.284776335 | NA | NS | 0.545496104 |
| LAD1 | 0.676380576 | 0.107926697 | NA | NS | 0.966871116 |
| TNNI1 | 0.683099761 | 0.128272255 | 0.350046063 | NS | 0.89186727 |
| PHLDA3 | -0.004551462 | 0.982664125 | 0.989708157 | NS | 0.007594899 |
| CSRP1 | 0.053922294 | 0.240100775 | 0.468184257 | NS | 0.619606439 |
| NAV1 | 0.230124064 | 0.121688917 | 0.34234916 | NS | 0.914748973 |
| IPO9 | 0.094334268 | 0.058073878 | 0.25208642 | NS | 1.236019173 |
| SHISA4 | -0.370654398 | 0.108910226 | 0.327019978 | NS | 0.962931339 |
| LMOD1 | -0.020394138 | 0.944292869 | 0.96956715 | NS | 0.02489329 |
| TIMM17A | -0.190721453 | 0.042506519 | 0.221095683 | Down | 1.371544461 |
| RNPEP | -0.053915848 | 0.506362941 | 0.686528045 | NS | 0.295538086 |
| ELF3 | 0.343274894 | 0.170918998 | 0.399304997 | NS | 0.767209662 |
| GPR37L1 | 0.416508245 | 0.009097634 | 0.120659125 | Up | 2.041071551 |
| ARL8A | 0.172481458 | 0.023196398 | 0.173122445 | Up | 1.634579441 |
| PTPN7 | 0.15967539 | 0.008798929 | 0.119193046 | Up | 2.055570169 |
| LGR6 | 0.050208883 | 0.765120532 | 0.86331923 | NS | 0.116270144 |
| UBE2T | -0.056542633 | 0.657708991 | 0.794202854 | NS | 0.181966221 |
| PPP1R12B | 0.145535496 | 0.180221065 | 0.41011753 | NS | 0.744194449 |
| SYT2 | -0.159726513 | 0.358768882 | 0.571703656 | NS | 0.445185233 |
| KDM5B | 0.125773354 | 0.03574425 | 0.206720913 | Up | 1.446793809 |
| RABIF | -0.007554697 | 0.886442275 | 0.935950794 | NS | 0.05234954 |
| KLHL12 | -0.111311968 | 0.014411152 | 0.143935548 | Down | 1.841301289 |
| ADIPOR1 | 0.021996167 | 0.89284081 | 0.939752341 | NS | 0.049225967 |
| CYB5R1 | -0.199146726 | 0.00649827 | 0.108077981 | Down | 2.187202247 |
| TMEM183A | -0.176243508 | 0.014569945 | 0.144575771 | Down | 1.836542088 |
| PPFIA4 | 0.135758669 | 0.531181389 | 0.704804549 | NS | 0.27475715 |
| MYOG | 0.989824915 | 0.029662251 | NA | Up | 1.527795901 |
| ADORA1 | 0.475561184 | 0.213680407 | 0.443230566 | NS | 0.670235298 |
| MYBPH | 0.171777384 | 0.531742902 | 0.704988765 | NS | 0.274298299 |
| CHI3L1 | -0.10044271 | 0.608262546 | 0.759851448 | NS | 0.215908924 |
| CHIT1 | 0.34986635 | 0.007733603 | 0.114126159 | Up | 2.111618098 |
| BTG2 | 0.174123881 | 0.104297487 | 0.320319302 | NS | 0.981726158 |
| FMOD | 0.530850131 | 0.292115573 | NA | NS | 0.53444529 |
| PRELP | 0.611972379 | 0.202793781 | 0.432901167 | NS | 0.692945367 |
| OPTC | 0.436522831 | 0.429953207 | NA | NS | 0.366578807 |
| ATP2B4 | 0.099909721 | 0.234930782 | 0.462908732 | NS | 0.629060075 |
| LAX1 | -0.08101869 | 0.153018454 | 0.379636943 | NS | 0.815256191 |
| ZC3H11A | 0.179777711 | 0.020193272 | 0.164578764 | Up | 1.694793294 |
| ZBED6 | 0.28942576 | 0.016641016 | 0.152056591 | Up | 1.77882016 |
| SNRPE | -0.222263314 | 0.13656031 | 0.361024479 | NS | 0.864675507 |
| SOX13 | 0.234961321 | 0.186205874 | 0.415835484 | NS | 0.730006622 |
| ETNK2 | 0.595171299 | 0.012007958 | 0.134014426 | Up | 1.92053084 |
| REN | 0.656038273 | 0.182578137 | NA | NS | 0.738551228 |
| KISS1 | 1.453492771 | 0.010890288 | NA | Up | 1.962960642 |
| GOLT1A | 0.508179714 | 0.309477114 | NA | NS | 0.509371462 |
| PLEKHA6 | 0.567251447 | 0.067740287 | 0.267671219 | NS | 1.16915297 |
| PPP1R15B | -0.025826687 | 0.686080917 | 0.813367075 | NS | 0.16362466 |
| PIK3C2B | 0.068962458 | 0.495416205 | 0.67814368 | NS | 0.305029792 |
| MDM4 | 0.105310986 | 0.089723774 | 0.30015394 | NS | 1.047092467 |
| LRRN2 | 0.065678568 | 0.623009812 | 0.769862318 | NS | 0.205505114 |
| NFASC | 0.320565779 | 0.338315726 | 0.554290685 | NS | 0.470677814 |
| CNTN2 | 0.350464006 | 0.126991351 | 0.348807379 | NS | 0.896225856 |
| TMEM81 | -0.098290192 | 0.202867823 | 0.432918271 | NS | 0.692786831 |
| RBBP5 | -0.008513204 | 0.851696231 | 0.916355684 | NS | 0.069715274 |
| DSTYK | -0.064522636 | 0.089408142 | 0.299846059 | NS | 1.048622929 |
| TMCC2 | -0.296455101 | 0.174285443 | 0.403422181 | NS | 0.758738885 |
| NUAK2 | 0.134955635 | 0.262409271 | 0.488672721 | NS | 0.581020825 |
| KLHDC8A | 0.112415933 | 0.622845587 | 0.769743797 | NS | 0.205619608 |
| LEMD1 | 0.812615725 | 0.153656494 | NA | NS | 0.813449081 |
| BLACAT1 | 0.480588406 | 0.190187408 | 0.419662012 | NS | 0.72081824 |
| CDK18 | 0.507284294 | 0.121967628 | 0.342773783 | NS | 0.913755422 |
| MFSD4A | 0.225802471 | 0.025819992 | 0.182115635 | Up | 1.588043889 |
| ELK4 | 0.017004064 | 0.740601455 | 0.847850763 | NS | 0.130415439 |
| SLC45A3 | -0.283399322 | 0.104197845 | 0.320245132 | NS | 0.982141265 |
| NUCKS1 | -0.236856936 | 7.91E-04 | 0.046828766 | Down | 3.101649732 |
| RAB29 | -0.104541766 | 0.155887095 | 0.383185333 | NS | 0.807189835 |
| SLC41A1 | 0.167781143 | 0.130167961 | 0.35213444 | NS | 0.885495898 |
| PM20D1 | 0.162209155 | 0.411396018 | 0.614050321 | NS | 0.385739916 |
| SLC26A9 | 0.587790436 | 0.193353843 | 0.422666547 | NS | 0.713647193 |
| RAB7B | 0.353568904 | 0.276511871 | NA | NS | 0.558286219 |
| CTSE | -0.34895334 | 0.145308737 | 0.37054456 | NS | 0.837708273 |
| RHEX | -0.05638464 | 0.803189947 | 0.886916883 | NS | 0.095181736 |
| AVPR1B | 0.418000811 | 0.265956822 | 0.491940819 | NS | 0.575188865 |
| FAM72A | 0.291308272 | 0.157354481 | NA | NS | 0.803120885 |
| SRGAP2 | 7.27E-04 | 0.994796057 | 0.996972664 | NS | 0.002265945 |
| IKBKE | -0.065305091 | 0.338331046 | 0.554290685 | NS | 0.470658149 |
| RASSF5 | 0.126601643 | 0.01549258 | 0.14796125 | Up | 1.809876239 |
| EIF2D | -0.083715471 | 0.0468903 | 0.230347845 | NS | 1.328916987 |
| DYRK3 | -0.191147161 | 0.289459652 | 0.51262024 | NS | 0.538411965 |
| MAPKAPK2 | -0.024412405 | 0.728063782 | 0.839150968 | NS | 0.137830573 |
| IL10 | 0.054170623 | 0.79267466 | 0.880521472 | NS | 0.100905025 |
| IL19 | 0.0999873 | 0.745236309 | 0.850563239 | NS | 0.127705994 |
| IL20 | 0.424910361 | 0.275034943 | NA | NS | 0.560612125 |
| IL24 | 0.041549798 | 0.727537992 | 0.838765421 | NS | 0.138144323 |
| FCMR | 0.213500282 | 0.026217218 | 0.183071016 | Up | 1.581413399 |
| PIGR | 0.604868374 | 0.112583204 | NA | NS | 0.948526396 |
| FCAMR | 0.069102477 | 0.899823156 | NA | NS | 0.045842835 |
| C1orf116 | 0.030103716 | 0.887082171 | 0.936262311 | NS | 0.052036149 |
| PFKFB2 | -0.148380376 | 0.040968193 | 0.218105862 | Down | 1.387553194 |
| YOD1 | -0.379604323 | 0.076942632 | 0.281244486 | NS | 1.113832962 |
| C4BPB | 0.14580143 | 0.651056743 | NA | NS | 0.186381159 |
| C4BPA | -0.165918356 | 0.613574047 | 0.763691556 | NS | 0.212133018 |
| CD55 | 0.104437549 | 0.291890548 | 0.514593347 | NS | 0.534779969 |
| CR2 | -0.072746062 | 0.566568689 | 0.730235585 | NS | 0.24674743 |
| CR1 | 0.153223673 | 0.2223563 | 0.451089986 | NS | 0.652950561 |
| CR1L | -0.279341134 | 0.135926048 | 0.360104507 | NS | 0.866697308 |
| CD46 | 0.024360276 | 0.804315786 | 0.887603726 | NS | 0.094573407 |
| CD34 | 0.414795088 | 0.248402762 | 0.476110795 | NS | 0.604843579 |
| PLXNA2 | 0.060049596 | 0.645673054 | 0.786072297 | NS | 0.189987338 |
| CAMK1G | 0.402077136 | 0.37005469 | NA | NS | 0.431734087 |
| LAMB3 | 0.124079956 | 0.539796446 | 0.710280503 | NS | 0.267769979 |
| G0S2 | -0.989610941 | 6.63E-04 | 0.042665485 | Down | 3.17834672 |
| HSD11B1 | 0.236158198 | 0.113933746 | 0.332423088 | NS | 0.943347622 |
| TRAF3IP3 | 0.026337855 | 0.58558643 | 0.744358129 | NS | 0.232408996 |
| C1orf74 | 0.040709481 | 0.555582213 | 0.721924582 | NS | 0.255251667 |
| IRF6 | 0.0147202 | 0.947205673 | 0.971541457 | NS | 0.02355571 |
| UTP25 | 0.049983897 | 0.350913044 | 0.56491456 | NS | 0.454800488 |
| SYT14 | 0.425652061 | 0.287100803 | 0.510257845 | NS | 0.541965592 |
| HHAT | 0.123595224 | 0.279245447 | 0.503278325 | NS | 0.5540139 |
| KCNH1 | 0.100784943 | 0.673833618 | 0.805843649 | NS | 0.171447325 |
| RCOR3 | 0.048916648 | 0.544086051 | 0.713285597 | NS | 0.264332408 |
| TRAF5 | -0.062152509 | 0.259063783 | 0.485568555 | NS | 0.586593297 |
| RD3 | 0.688105688 | 0.148897612 | NA | NS | 0.827112267 |
| SLC30A1 | -0.071678371 | 0.328046823 | 0.545799372 | NS | 0.484064163 |
| NEK2 | 0.114266932 | 0.583070451 | 0.742186934 | NS | 0.234278967 |
| LPGAT1 | 0.025763776 | 0.767532876 | 0.86472048 | NS | 0.114903013 |
| INTS7 | -0.032139864 | 0.467676681 | 0.657422745 | NS | 0.330054284 |
| DTL | -0.292994239 | 0.149071589 | 0.375188026 | NS | 0.82660512 |
| PPP2R5A | -0.014971182 | 0.826969435 | 0.901525799 | NS | 0.082510542 |
| PACC1 | -0.01797822 | 0.704089829 | 0.824734454 | NS | 0.152371929 |
| NENF | -0.202689759 | 0.047378769 | 0.23126872 | Down | 1.324416232 |
| ATF3 | 0.252481684 | 0.151605009 | 0.378055086 | NS | 0.81928645 |
| BATF3 | -0.289423789 | 0.073736285 | 0.275940596 | NS | 1.132318745 |
| NSL1 | -0.06069747 | 0.190705123 | 0.420149522 | NS | 0.719637641 |
| TATDN3 | -0.099516697 | 0.138961221 | 0.363222438 | NS | 0.85710638 |
| SPATA45 | 0.592621438 | 0.054495382 | NA | NS | 1.263640299 |
| FLVCR1 | -0.112495796 | 0.168906211 | 0.397032746 | NS | 0.77235438 |
| VASH2 | 0.11773104 | 0.239703366 | 0.467804559 | NS | 0.620325867 |
| ANGEL2 | -0.012105906 | 0.807144279 | 0.888975046 | NS | 0.093048827 |
| RPS6KC1 | -0.059722958 | 0.251460919 | 0.478279044 | NS | 0.599529502 |
| PROX1 | 0.34779976 | 0.305302903 | 0.526578834 | NS | 0.515269067 |
| SMYD2 | -0.089141787 | 0.151041987 | 0.377609248 | NS | 0.820902309 |
| PTPN14 | 0.214741024 | 0.198418376 | 0.428291956 | NS | 0.702418109 |
| CENPF | -0.315783173 | 0.038450129 | 0.213464789 | Down | 1.415102201 |
| KCNK2 | 0.726078468 | 0.129377514 | NA | NS | 0.888141198 |
| KCTD3 | -0.149218955 | 0.055999029 | 0.249087638 | NS | 1.251819505 |
| USH2A | 0.25630334 | 0.351442109 | 0.565259301 | NS | 0.454146203 |
| ESRRG | 0.422106984 | 0.320227631 | 0.539074418 | NS | 0.494541198 |
| GPATCH2 | -0.048685459 | 0.330144003 | 0.547441347 | NS | 0.481296587 |
| SPATA17 | 0.233128235 | 0.576323493 | 0.737285229 | NS | 0.239333677 |
| RRP15 | -0.127621097 | 0.090455401 | 0.301169683 | NS | 1.043565494 |
| TGFB2 | 0.249165757 | 0.311474442 | 0.531192767 | NS | 0.506577584 |
| LYPLAL1 | -0.089683578 | 0.136139048 | 0.36040417 | NS | 0.866017291 |
| ZC3H11B | 0.095270776 | 0.769308796 | NA | NS | 0.113899302 |
| SLC30A10 | 0.171902956 | 0.682010944 | 0.810483683 | NS | 0.166208656 |
| EPRS1 | -0.209032613 | 0.002699757 | 0.078862345 | Down | 2.56867535 |
| BPNT1 | -0.161737511 | 0.034431605 | 0.203549976 | Down | 1.463042739 |
| IARS2 | -0.158737041 | 0.038484974 | 0.213464789 | Down | 1.414708803 |
| RAB3GAP2 | -0.007223232 | 0.840746217 | 0.910175838 | NS | 0.075335078 |
| MARK1 | 0.355479895 | 0.291546973 | 0.514367828 | NS | 0.535291463 |
| C1orf115 | -0.293535734 | 0.010554196 | 0.127927347 | Down | 1.976574864 |
| MTARC2 | 0.085324 | 0.70658528 | 0.826424395 | NS | 0.150835415 |
| MTARC1 | -0.078673906 | 0.526112671 | 0.701218204 | NS | 0.278921238 |
| HLX | 0.162338063 | 0.135517422 | 0.359514684 | NS | 0.868004867 |
| DUSP10 | -0.092882129 | 0.286195146 | 0.509280592 | NS | 0.543337737 |
| HHIPL2 | 0.404659358 | 0.153621031 | NA | NS | 0.813549325 |
| TAF1A | -0.035246167 | 0.568778925 | 0.731587525 | NS | 0.245056504 |
| MIA3 | -0.075958714 | 0.038569266 | 0.213464789 | NS | 1.413758626 |
| AIDA | -0.104549419 | 0.393830395 | 0.600038083 | NS | 0.404690769 |
| BROX | -0.081115906 | 0.190072126 | 0.419488468 | NS | 0.721081567 |
| FAM177B | -0.050217984 | 0.693386096 | 0.817836349 | NS | 0.159024871 |
| DISP1 | 0.037969588 | 0.59557485 | 0.750945369 | NS | 0.22506365 |
| TLR5 | 0.296809209 | 0.015042662 | 0.146973997 | Up | 1.822675298 |
| SUSD4 | 0.225061324 | 0.156439326 | 0.383636723 | NS | 0.805654064 |
| CCDC185 | 1.458261968 | 0.006261539 | NA | Up | 2.2033189 |
| CAPN8 | 0.392618529 | 0.205541474 | 0.435492143 | NS | 0.687100533 |
| CAPN2 | -0.223198218 | 0.003274427 | 0.084967003 | Down | 2.484864719 |
| TP53BP2 | -0.122228328 | 0.011587184 | 0.132156218 | Down | 1.936022101 |
| FBXO28 | -0.086642278 | 0.133320383 | 0.35644448 | NS | 0.875103446 |
| DEGS1 | -0.16249387 | 0.064992239 | 0.264064926 | NS | 1.187138498 |
| NVL | -0.09747999 | 0.045020898 | 0.226907704 | NS | 1.346585847 |
| CNIH4 | 0.114288121 | 0.139848112 | 0.364064028 | NS | 0.854343392 |
| WDR26 | -0.049423403 | 0.698206454 | 0.821297286 | NS | 0.156016141 |
| CNIH3 | -0.092282102 | 0.554430275 | 0.72131755 | NS | 0.256153063 |
| DNAH14 | 0.046099241 | 0.752446587 | 0.854745882 | NS | 0.123524324 |
| LBR | -0.072500543 | 0.404936669 | 0.60860084 | NS | 0.392612894 |
| ENAH | 0.447616491 | 0.066026237 | 0.265193419 | NS | 1.180283454 |
| SRP9 | -0.268092118 | 0.023485734 | 0.174068049 | Down | 1.629195869 |
| EPHX1 | -0.030664371 | 0.858458905 | 0.92008732 | NS | 0.06628049 |
| TMEM63A | 6.44E-05 | 0.999585465 | 0.999716421 | NS | 1.80E-04 |
| LEFTY1 | -0.030172695 | 0.882039092 | 0.93320019 | NS | 0.054512167 |
| PYCR2 | -0.005232749 | 0.918408752 | 0.954891598 | NS | 0.036963986 |
| LEFTY2 | 1.40474481 | 0.001585763 | NA | Up | 2.799761659 |
| SDE2 | -0.155428001 | 0.031597349 | 0.197250269 | Down | 1.500349353 |
| H3-3A | 0.149270516 | 0.129361442 | 0.351284745 | NS | 0.888195151 |
| ACBD3 | -0.177326614 | 0.004712417 | 0.095487183 | Down | 2.326756257 |
| MIXL1 | 0.406499333 | 0.174015408 | NA | NS | 0.759412297 |
| LIN9 | -0.199387601 | 0.129734275 | 0.351821639 | NS | 0.886945272 |
| PARP1 | -0.179194264 | 0.005983171 | 0.103408357 | Down | 2.223068556 |
| STUM | 0.442173294 | 0.14547506 | 0.37059965 | NS | 0.837211455 |
| ITPKB | 0.147978037 | 0.039844721 | 0.215796828 | Up | 1.399629215 |
| PSEN2 | 0.242595621 | 0.191608062 | 0.420934764 | NS | 0.717586223 |
| COQ8A | -0.119137942 | 0.304247752 | 0.52547007 | NS | 0.516772622 |
| CDC42BPA | -0.455937418 | 0.01085507 | 0.12856202 | Down | 1.964367378 |
| ZNF678 | -0.125303376 | 0.105517953 | 0.321700573 | NS | 0.976673641 |
| SNAP47 | -0.094897632 | 0.070613076 | 0.271500817 | NS | 1.151114871 |
| JMJD4 | -0.022997584 | 0.753476535 | 0.85557636 | NS | 0.122930268 |
| PRSS38 | 0.677478275 | 0.179072713 | NA | NS | 0.746970587 |
| WNT9A | 0.527262344 | 0.148371243 | NA | NS | 0.828650266 |
| WNT3A | 0.966744793 | 0.076603036 | NA | NS | 1.115754017 |
| ARF1 | -0.043294641 | 0.670277301 | 0.803363573 | NS | 0.173745488 |
| C1orf35 | -0.127139497 | 0.074133309 | 0.276559869 | NS | 1.129986617 |
| MRPL55 | -0.1163007 | 0.144154053 | 0.369451222 | NS | 0.841173143 |
| GUK1 | -0.128276909 | 0.38649823 | 0.594562322 | NS | 0.412852491 |
| GJC2 | 0.319256558 | 0.065306707 | 0.264481527 | NS | 1.185042212 |
| IBA57 | -0.103562253 | 0.290853727 | 0.513652722 | NS | 0.536325367 |
| OBSCN | -0.080190175 | 0.560389637 | 0.724964326 | NS | 0.251509904 |
| TRIM11 | 0.087733227 | 0.096679209 | 0.310822251 | NS | 1.014666912 |
| TRIM17 | 0.082522927 | 0.430131228 | 0.627557816 | NS | 0.366399026 |
| RNF187 | -0.088083563 | 0.609899785 | 0.760957709 | NS | 0.21474152 |
| RHOU | -0.197663152 | 0.105736078 | 0.321845781 | NS | 0.975776804 |
| RAB4A | -0.109416467 | 0.078856135 | 0.283688849 | NS | 1.103164511 |
| CCSAP | -0.113332023 | 0.06590589 | 0.265127437 | NS | 1.181075771 |
| ACTA1 | 0.993234748 | 0.02430887 | NA | Up | 1.614235225 |
| NUP133 | -0.117723031 | 0.013664739 | 0.14033626 | Down | 1.864398649 |
| ABCB10 | -0.212754354 | 0.041927187 | 0.220688679 | Down | 1.377504271 |
| TAF5L | 0.021938373 | 0.594202792 | 0.750147861 | NS | 0.226065312 |
| URB2 | -0.08704697 | 0.18596924 | 0.415622384 | NS | 0.730558883 |
| GALNT2 | 0.004668982 | 0.939974593 | 0.966780353 | NS | 0.026883885 |
| PGBD5 | 0.629209808 | 0.08956478 | 0.299928733 | NS | 1.047862734 |
| COG2 | -0.133085824 | 0.006005289 | 0.103564104 | Down | 2.221466082 |
| AGT | 0.257023904 | 0.044231098 | 0.224956829 | Up | 1.354272283 |
| CAPN9 | 0.726421393 | 0.13707387 | NA | NS | 0.863045325 |
| C1orf198 | 0.060876104 | 0.655632322 | 0.793158916 | NS | 0.183339644 |
| TTC13 | -0.114959518 | 0.015058047 | 0.147015649 | Down | 1.82223136 |
| ARV1 | -0.179329255 | 0.099332589 | 0.314301595 | NS | 1.002908246 |
| FAM89A | -0.12959034 | 0.206959951 | 0.436943551 | NS | 0.684113688 |
| TRIM67 | 0.41285446 | 0.162542861 | 0.390120462 | NS | 0.789032101 |
| C1orf131 | -0.144885555 | 0.032148798 | 0.198223945 | Down | 1.492835255 |
| GNPAT | -0.090839453 | 0.081518905 | 0.287842422 | NS | 1.088741665 |
| EXOC8 | -0.047522218 | 0.464032115 | 0.654670332 | NS | 0.333451961 |
| SPRTN | -0.014242077 | 0.799162562 | 0.884455271 | NS | 0.09736487 |
| EGLN1 | 0.173711702 | 0.061688654 | 0.258215532 | NS | 1.209794704 |
| TSNAX | -0.156492967 | 0.063676171 | 0.262179256 | NS | 1.19602306 |
| TSNAX-DISC1 | 0.189420187 | 0.265290853 | 0.491489111 | NS | 0.576277725 |
| DISC1 | 0.148314762 | 0.081033489 | 0.287057145 | NS | 1.09133546 |
| SIPA1L2 | 0.122351345 | 0.403572496 | 0.60743385 | NS | 0.394078439 |
| MAP10 | -0.21345647 | 0.091966136 | 0.303838495 | NS | 1.03637206 |
| NTPCR | 0.129571112 | 0.095081631 | 0.308611041 | NS | 1.021903378 |
| PCNX2 | 0.004198779 | 0.94822662 | 0.971776348 | NS | 0.023087857 |
| MAP3K21 | 0.286844159 | 0.00757606 | 0.113107511 | Up | 2.120556604 |
| KCNK1 | -0.092299545 | 0.794187324 | 0.881041916 | NS | 0.100077049 |
| SLC35F3 | 0.29121689 | 0.160980497 | 0.388764128 | NS | 0.793226737 |
| COA6 | -0.229219133 | 0.060274874 | 0.256083601 | NS | 1.219863689 |
| TARBP1 | -0.110695928 | 0.053035208 | 0.243311765 | NS | 1.275435724 |
| IRF2BP2 | -0.096887852 | 0.071369878 | 0.272521361 | NS | 1.146485048 |
| TOMM20 | -0.361527739 | 2.53E-04 | 0.026605481 | Down | 3.597246724 |
| RBM34 | 0.108075763 | 0.245622373 | 0.473904262 | NS | 0.609732077 |
| ARID4B | -0.018663062 | 0.711866745 | 0.829804662 | NS | 0.147601295 |
| GGPS1 | -0.240762474 | 0.007726224 | 0.114126159 | Down | 2.112032689 |
| TBCE | -0.035616244 | 0.628423696 | 0.773688823 | NS | 0.201747447 |
| B3GALNT2 | -0.14406312 | 0.130530941 | 0.352442775 | NS | 0.88428653 |
| GNG4 | 1.126807752 | 0.039629245 | NA | Up | 1.401984202 |
| LYST | 0.036033681 | 0.670141087 | 0.803285417 | NS | 0.173833754 |
| NID1 | 0.253409272 | 0.127803824 | 0.349398501 | NS | 0.893456152 |
| GPR137B | 0.160000742 | 0.021958702 | 0.169726019 | Up | 1.658393335 |
| ERO1B | 0.075395846 | 0.159819841 | 0.38738272 | NS | 0.796369305 |
| EDARADD | 0.243535386 | 0.109603735 | 0.327780719 | NS | 0.960174645 |
| LGALS8 | 0.013108174 | 0.750681113 | 0.853671923 | NS | 0.124544511 |
| HEATR1 | -0.032732302 | 0.544149134 | 0.713285597 | NS | 0.264282058 |
| ACTN2 | 0.387529903 | 0.334899138 | 0.551352172 | NS | 0.47508597 |
| MTR | -0.014227962 | 0.782869283 | 0.874453587 | NS | 0.106310747 |
| RYR2 | 0.340231981 | 0.229931707 | 0.458158433 | NS | 0.638401137 |
| ZP4 | 0.500027274 | 0.347298031 | NA | NS | 0.459297679 |
| MTRNR2L11 | -0.159335889 | 0.766272093 | NA | NS | 0.115616991 |
| CHRM3 | 0.271127627 | 0.269706928 | 0.495176211 | NS | 0.569107898 |
| FMN2 | 0.519676252 | 0.221773593 | 0.450609639 | NS | 0.654090167 |
| GREM2 | 0.492030968 | 0.017727737 | 0.155687895 | Up | 1.751346711 |
| RGS7 | 0.295411698 | 0.258385341 | 0.485044351 | NS | 0.587732128 |
| FH | -0.154147627 | 0.020874528 | 0.167612586 | Down | 1.68038334 |
| KMO | 0.048149513 | 0.620289257 | 0.76776007 | NS | 0.20740574 |
| OPN3 | -0.102022815 | 0.210480278 | 0.440341584 | NS | 0.676788591 |
| CHML | -0.155108103 | 0.065333993 | 0.264481527 | NS | 1.184860798 |
| WDR64 | 0.243616778 | 0.346720418 | 0.561331191 | NS | 0.460020582 |
| EXO1 | 0.100585192 | 0.574999656 | 0.736180663 | NS | 0.240332415 |
| BECN2 | 0.605450941 | 0.273972182 | NA | NS | 0.562293532 |
| MAP1LC3C | 0.323385512 | 0.542662818 | NA | NS | 0.265469935 |
| PLD5 | 0.553630509 | 0.168503837 | 0.396697663 | NS | 0.773390206 |
| CEP170 | 0.031667408 | 0.536391861 | 0.708295535 | NS | 0.270517821 |
| SDCCAG8 | -0.230262664 | 0.020626022 | 0.166711709 | Down | 1.685584529 |
| AKT3 | -0.301757629 | 0.012828596 | 0.136081756 | Down | 1.891820863 |
| ZBTB18 | 0.041373359 | 0.533068674 | 0.705888336 | NS | 0.273216838 |
| C1orf100 | 0.126163301 | 0.415340248 | 0.616572671 | NS | 0.381595982 |
| ADSS2 | -0.121337962 | 0.003666205 | 0.087098982 | Down | 2.435783296 |
| CATSPERE | 0.23608587 | 0.118218596 | 0.338599586 | NS | 0.927314203 |
| DESI2 | -0.135945056 | 0.040152631 | 0.216267417 | Down | 1.396285987 |
| COX20 | 0.129540674 | 0.081668296 | 0.288059034 | NS | 1.087946508 |
| HNRNPU | -0.017046797 | 0.6606929 | 0.796210734 | NS | 0.18000036 |
| EFCAB2 | 0.023659654 | 0.871589421 | 0.927476114 | NS | 0.05968805 |
| KIF26B | 0.360896888 | 0.331163765 | NA | NS | 0.479957189 |
| SMYD3 | 0.023069314 | 0.72399507 | 0.837251823 | NS | 0.140264391 |
| TFB2M | -0.216988252 | 0.046947161 | 0.230429463 | Down | 1.328390669 |
| CNST | -0.136514158 | 0.013858017 | 0.141394243 | Down | 1.858298896 |
| SCCPDH | -0.364099919 | 0.00100081 | 0.052382393 | Down | 2.99964843 |
| AHCTF1 | 0.028753467 | 0.698934783 | 0.821799178 | NS | 0.155563346 |
| ZNF695 | 0.088675453 | 0.761680296 | NA | NS | 0.118227279 |
| ZNF670-ZNF695 | 0.526650824 | 0.057961388 | NA | NS | 1.236861223 |
| ZNF670 | 0.018887639 | 0.840644452 | 0.910108691 | NS | 0.075387649 |
| ZNF669 | -0.230116656 | 0.005082958 | 0.09830662 | Down | 2.293883446 |
| ZNF124 | -0.256365978 | 0.021373312 | 0.168379028 | Down | 1.670128174 |
| ZNF496 | 0.215811891 | 0.028197198 | 0.187888344 | Up | 1.54979404 |
| NLRP3 | -0.043886083 | 0.70275767 | 0.82383594 | NS | 0.153194406 |
| OR2B11 | 0.19300771 | 0.063842547 | 0.262358156 | NS | 1.194889798 |
| GCSAML | -0.016571605 | 0.928982767 | 0.961212294 | NS | 0.031992342 |
| OR2C3 | 0.12434912 | 0.72594833 | 0.838611255 | NS | 0.13909429 |
| OR2G2 | 0.676526291 | 0.244261209 | NA | NS | 0.612145497 |
| OR2G3 | 0.600802336 | 0.279392371 | NA | NS | 0.553785457 |
| OR14L1P | 0.351542386 | 0.279183588 | NA | NS | 0.554110116 |
| OR13G1 | -0.101821234 | 0.815192115 | NA | NS | 0.08874003 |
| OR6F1 | 1.284526674 | 0.023236423 | NA | Up | 1.633830723 |
| OR14A2 | 0.967034258 | 0.113997601 | NA | NS | 0.94310429 |
| OR14K1 | 0.056854608 | 0.923360571 | NA | NS | 0.034628674 |
| OR1C1 | 0.949547849 | 0.059644352 | NA | NS | 1.224430677 |
| OR14A16 | 0.700124695 | 0.204191169 | NA | NS | 0.689963044 |
| OR11L1 | 0.359687217 | 0.53587067 | NA | NS | 0.270940013 |
| TRIM58 | -0.149558223 | 0.530390584 | 0.704519993 | NS | 0.275404195 |
| OR2W3 | -0.28274445 | 0.228371205 | 0.457089362 | NS | 0.641358657 |
| OR2T8 | 0.124728581 | 0.621518997 | 0.768658144 | NS | 0.206545593 |
| OR2AJ1 | 0.155191347 | 0.730164561 | NA | NS | 0.13657925 |
| OR2L8 | 0.407475168 | 0.119399129 | NA | NS | 0.922998841 |
| OR2AK2 | 0.415138031 | 0.103652951 | NA | NS | 0.984418328 |
| OR2L5 | -0.012014224 | 0.961504974 | 0.978517825 | NS | 0.017048465 |
| OR2L2 | 0.209579498 | 0.471640014 | 0.660440209 | NS | 0.326389356 |
| OR2L3 | -0.147541545 | 0.595852487 | 0.751153942 | NS | 0.224861244 |
| OR2L13 | 0.091055463 | 0.755337825 | NA | NS | 0.121858766 |
| OR2M5 | 0.173338394 | 0.766702659 | NA | NS | 0.11537303 |
| OR2M2 | 0.067234685 | 0.909532186 | NA | NS | 0.041181928 |
| OR2M3 | 0.31476538 | 0.443770308 | 0.637917821 | NS | 0.352841759 |
| OR2M4 | 0.18014883 | 0.678892622 | NA | NS | 0.168198911 |
| OR2T33 | 0.326242418 | 0.355992623 | NA | NS | 0.448559001 |
| OR2T12 | 0.623669394 | 0.201640935 | NA | NS | 0.695421298 |
| OR2M7 | 0.743447069 | 0.2291272 | NA | NS | 0.639923351 |
| OR14C36 | 0.851133692 | 0.21158825 | NA | NS | 0.674508454 |
| OR2G6 | 0.27786754 | 0.351572745 | 0.565262486 | NS | 0.4539848 |
| OR2T29 | 0.545776488 | 0.215627431 | NA | NS | 0.666295991 |
| OR2T34 | 0.085079566 | 0.80784784 | NA | NS | 0.092670432 |
| OR2T10 | -0.041146685 | 0.873747111 | 0.928779593 | NS | 0.058614247 |
| OR2T11 | 0.455378342 | 0.169927835 | 0.398309642 | NS | 0.769735475 |
| OR2T27 | 0.992559932 | 0.071308806 | NA | NS | 1.146856838 |
| LYPD8 | -0.079733397 | 0.702739377 | 0.82383594 | NS | 0.15320571 |
| SH3BP5L | -0.036680635 | 0.589670714 | 0.747377833 | NS | 0.229390441 |
| ZNF672 | 0.040481263 | 0.392347704 | 0.599004293 | NS | 0.406328884 |
| ZNF692 | -0.109519861 | 0.176421679 | 0.406030478 | NS | 0.753448049 |
| PGBD2 | -0.149526234 | 0.002717976 | 0.078993761 | Down | 2.565754401 |
| FAM110C | 0.072199647 | 0.653797684 | 0.791973775 | NS | 0.184556622 |
| SH3YL1 | -0.162773488 | 0.029204762 | 0.1903921 | Down | 1.534546335 |
| ACP1 | -0.112529543 | 0.348310451 | 0.562612118 | NS | 0.458033495 |
| ALKAL2 | -0.017372201 | 0.854447636 | 0.917929393 | NS | 0.068314548 |
| TMEM18 | -0.069623602 | 0.277929258 | 0.502231925 | NS | 0.556065733 |
| SNTG2 | 0.547896135 | 0.008330604 | 0.117263361 | Up | 2.079323519 |
| TPO | 0.232692462 | 0.420562772 | NA | NS | 0.376169173 |
| PXDN | 0.509558032 | 0.113911833 | 0.332423088 | NS | 0.943431158 |
| MYT1L | 0.497015376 | 0.167702163 | 0.395909177 | NS | 0.775461337 |
| EIPR1 | -0.181342715 | 0.003487781 | 0.085797167 | Down | 2.457450784 |
| TRAPPC12 | 0.063778792 | 0.319975753 | 0.539024418 | NS | 0.49488293 |
| ADI1 | -0.100552066 | 0.158489327 | 0.385976454 | NS | 0.799999978 |
| RNASEH1 | 0.001659846 | 0.977259968 | 0.986738726 | NS | 0.009989891 |
| RPS7 | -0.541260683 | 0.025534938 | 0.180884983 | Down | 1.592865193 |
| COLEC11 | 0.929030374 | 0.035510051 | NA | Up | 1.449648704 |
| ALLC | 0.504649126 | 0.28429492 | NA | NS | 0.5462309 |
| DCDC2C | 0.388097848 | 0.406039478 | 0.609618207 | NS | 0.391431739 |
| SOX11 | 0.638214835 | 0.138612397 | 0.363039337 | NS | 0.858197925 |
| CMPK2 | 0.24239882 | 0.18890805 | 0.418330318 | NS | 0.723749536 |
| RSAD2 | 0.20943109 | 0.279102424 | 0.503208948 | NS | 0.554236391 |
| RNF144A | 0.111010456 | 0.150903677 | 0.37740073 | NS | 0.821300179 |
| ID2 | -0.132217435 | 0.174854895 | 0.404252655 | NS | 0.757322206 |
| KIDINS220 | 0.037475112 | 0.530806214 | 0.704673243 | NS | 0.275064002 |
| MBOAT2 | -0.050469989 | 0.760717332 | 0.860215688 | NS | 0.118776689 |
| ASAP2 | 0.003716652 | 0.981554394 | 0.989243035 | NS | 0.008085629 |
| ITGB1BP1 | -0.07374991 | 0.196571958 | 0.426336336 | NS | 0.706478436 |
| CPSF3 | -0.257489565 | 1.23E-04 | 0.017674589 | Down | 3.908404102 |
| IAH1 | 0.079123839 | 0.193351258 | 0.422666547 | NS | 0.713652998 |
| ADAM17 | 0.144483874 | 0.005971537 | 0.103371228 | Up | 2.223913889 |
| YWHAQ | -0.187929569 | 0.027734104 | 0.186639939 | Down | 1.556985862 |
| TAF1B | -0.025137266 | 0.633951673 | 0.776736637 | NS | 0.197943848 |
| GRHL1 | 0.007061416 | 0.956256362 | 0.976161497 | NS | 0.019425663 |
| KLF11 | -0.108191546 | 0.238638448 | 0.466720558 | NS | 0.622259585 |
| CYS1 | 1.045182227 | 0.004123409 | NA | Up | 2.384743567 |
| RRM2 | 0.182839478 | 0.219447676 | 0.448847618 | NS | 0.658669014 |
| HPCAL1 | -0.00347181 | 0.979299192 | 0.987728138 | NS | 0.009084604 |
| ODC1 | -0.399169927 | 4.78E-05 | 0.009869119 | Down | 4.320272066 |
| NOL10 | -0.199009293 | 0.001550702 | 0.062836479 | Down | 2.809471601 |
| ATP6V1C2 | 0.061882316 | 0.500354619 | 0.682049966 | NS | 0.300722087 |
| PDIA6 | -0.227395289 | 0.009661851 | 0.123479748 | Down | 2.01493967 |
| KCNF1 | 1.298144312 | 0.019937123 | NA | Up | 1.700337522 |
| C2orf50 | 0.311426398 | 0.218618206 | 0.44811547 | NS | 0.660313674 |
| SLC66A3 | -0.150095128 | 0.118014755 | 0.338396633 | NS | 0.928063692 |
| ROCK2 | -0.087832484 | 0.078485806 | 0.283183216 | NS | 1.105208878 |
| E2F6 | -0.110343729 | 0.095117923 | 0.308685088 | NS | 1.021737643 |
| GREB1 | 0.737729308 | 0.022285149 | 0.170667658 | Up | 1.651984451 |
| NTSR2 | 0.951262792 | 0.075309217 | NA | NS | 1.12315187 |
| LPIN1 | -0.01979776 | 0.687032731 | 0.814158315 | NS | 0.163022572 |
| TRIB2 | -0.118128496 | 0.119558406 | 0.34007656 | NS | 0.922419885 |
| LRATD1 | 0.408398257 | 0.341317589 | 0.55695443 | NS | 0.46684133 |
| NBAS | -0.20862377 | 0.001793602 | 0.066048296 | Down | 2.746274037 |
| DDX1 | -0.051280513 | 0.372247352 | 0.583052983 | NS | 0.429168383 |
| MYCN | 0.600871777 | 0.087223667 | NA | NS | 1.059365658 |
| CYRIA | 0.065621618 | 0.399036792 | 0.604094435 | NS | 0.39898706 |
| RAD51AP2 | 0.537605958 | 0.263113668 | NA | NS | 0.579856592 |
| VSNL1 | 0.338888037 | 0.023773008 | 0.174926344 | Up | 1.623915872 |
| SMC6 | -0.181916683 | 0.007381241 | 0.112471844 | Down | 2.131870616 |
| GEN1 | 0.056671126 | 0.42561597 | 0.623837924 | NS | 0.370982085 |
| MSGN1 | 1.437594176 | 0.006170041 | NA | Up | 2.209711919 |
| KCNS3 | 0.680790983 | 0.115509528 | NA | NS | 0.937382191 |
| RDH14 | -0.155965431 | 0.173468683 | 0.402469839 | NS | 0.760778918 |
| NT5C1B | 0.11442924 | 0.29750059 | 0.519231711 | NS | 0.526512169 |
| OSR1 | 1.008927642 | 0.035499257 | NA | Up | 1.449780741 |
| TTC32 | 0.011292559 | 0.872785374 | 0.928144996 | NS | 0.05909254 |
| WDR35 | -0.025663615 | 0.620545786 | 0.767922494 | NS | 0.207226169 |
| MATN3 | 0.712042081 | 0.088336602 | NA | NS | 1.053859309 |
| LAPTM4A | 0.013507817 | 0.882163463 | 0.933221287 | NS | 0.054450933 |
| SDC1 | 0.827406102 | 0.019735137 | NA | Up | 1.70475985 |
| PUM2 | 0.047724008 | 0.258924817 | 0.485499932 | NS | 0.586826322 |
| RHOB | -0.044548738 | 0.597080403 | 0.752080925 | NS | 0.223967183 |
| HS1BP3 | 0.118752916 | 0.092749629 | 0.305193805 | NS | 1.032687817 |
| GDF7 | 0.134234172 | 0.270657994 | 0.495997715 | NS | 0.567579141 |
| LDAH | -0.087814235 | 0.113343854 | 0.332115589 | NS | 0.945602025 |
| APOB | 0.500600103 | 0.250809818 | 0.47774934 | NS | 0.600655467 |
| TDRD15 | -0.141222356 | 0.507546239 | 0.687393493 | NS | 0.294524386 |
| KLHL29 | 0.36792148 | 0.004200182 | 0.091962298 | Up | 2.376731913 |
| ATAD2B | -0.133555588 | 0.06350224 | 0.261830379 | NS | 1.197210952 |
| UBXN2A | -0.421587323 | 2.56E-04 | 0.026605481 | Down | 3.591188847 |
| MFSD2B | -0.036961391 | 0.838855486 | 0.909109618 | NS | 0.076312851 |
| WDCP | -0.126669967 | 0.031991916 | 0.198142899 | Down | 1.494959752 |
| FKBP1B | -0.472641816 | 0.003069629 | 0.083174295 | Down | 2.512914134 |
| SF3B6 | -0.232843954 | 0.119469303 | 0.340007095 | NS | 0.922743671 |
| FAM228B | -0.147064028 | 0.132469709 | 0.355399001 | NS | 0.877883419 |
| TP53I3 | 0.2545696 | 0.003703845 | 0.087629596 | Up | 2.431347242 |
| PFN4 | 0.300875943 | 0.008315678 | 0.117263361 | Up | 2.080102329 |
| FAM228A | 0.152909065 | 0.460624881 | NA | NS | 0.336652607 |
| ITSN2 | -0.020417525 | 0.629696763 | 0.774589928 | NS | 0.200868539 |
| NCOA1 | 0.080122615 | 0.271311979 | 0.496530841 | NS | 0.56653103 |
| PTRHD1 | -0.334755832 | 0.021021623 | 0.167865133 | Down | 1.677333763 |
| CENPO | 0.055136557 | 0.537925758 | 0.709095159 | NS | 0.26927766 |
| ADCY3 | 0.044544766 | 0.600487491 | 0.754648056 | NS | 0.221496035 |
| DNAJC27 | 0.156623449 | 0.012631657 | 0.135545097 | Up | 1.898539681 |
| EFR3B | 0.417958574 | 0.127312346 | 0.348830209 | NS | 0.895129481 |
| POMC | 0.133626579 | 0.477194383 | 0.664987747 | NS | 0.321304677 |
| DNMT3A | 0.032303752 | 0.650029706 | 0.788976303 | NS | 0.187066796 |
| DTNB | 0.16211615 | 0.027709147 | 0.186639939 | Up | 1.55737685 |
| ASXL2 | 0.063093836 | 0.342668412 | 0.557886683 | NS | 0.465125928 |
| KIF3C | 0.100246786 | 0.384254967 | 0.593034804 | NS | 0.41538051 |
| RAB10 | -0.076507013 | 0.186833094 | 0.416322648 | NS | 0.728546194 |
| GAREM2 | 0.402633076 | 0.005794056 | 0.10258884 | Up | 2.2370173 |
| HADHA | -0.166257616 | 0.007309288 | 0.112271836 | Down | 2.136124942 |
| HADHB | -0.060381738 | 0.229949732 | 0.458158433 | NS | 0.638367092 |
| ADGRF3 | 0.182593824 | 0.120640206 | 0.341313216 | NS | 0.918507931 |
| SELENOI | 0.073928437 | 0.109369403 | 0.327424668 | NS | 0.96110416 |
| DRC1 | 0.339994303 | 0.253481445 | 0.48036674 | NS | 0.596053826 |
| OTOF | 0.8037135 | 0.00395638 | 0.090040171 | Up | 2.402701965 |
| FAM166C | 0.722719449 | 0.232662195 | NA | NS | 0.63327418 |
| CIB4 | 0.580921653 | 0.360933622 | NA | NS | 0.442572661 |
| KCNK3 | 0.591866439 | 0.197763492 | NA | NS | 0.703853878 |
| SLC35F6 | 0.266587076 | 2.08E-04 | 0.023858165 | Up | 3.681842249 |
| CENPA | -0.023406335 | 0.924844559 | 0.95895218 | NS | 0.033931254 |
| DPYSL5 | 0.668152808 | 0.137950201 | 0.362607036 | NS | 0.860277663 |
| MAPRE3 | 0.105032868 | 0.450993408 | 0.644091483 | NS | 0.345829806 |
| TMEM214 | 0.085768316 | 0.357566306 | 0.570700644 | NS | 0.446643412 |
| AGBL5 | 0.039819805 | 0.408108821 | 0.611282421 | NS | 0.389224018 |
| OST4 | -0.186980198 | 0.046835991 | 0.230265507 | Down | 1.329420288 |
| EMILIN1 | 0.144631905 | 0.268479422 | 0.494168679 | NS | 0.571088995 |
| KHK | 0.112156301 | 0.279808891 | 0.503478405 | NS | 0.55313849 |
| CGREF1 | 0.750769996 | 0.010885098 | NA | Up | 1.963167643 |
| ABHD1 | 0.104378951 | 0.314194323 | 0.53361135 | NS | 0.502801667 |
| PREB | 0.160945271 | 0.001690183 | 0.065056412 | Up | 2.772066337 |
| PRR30 | 0.483184155 | 0.34857322 | NA | NS | 0.457705982 |
| TCF23 | 0.711542028 | 0.139098993 | NA | NS | 0.856676013 |
| SLC5A6 | 0.052229624 | 0.452413535 | 0.645113927 | NS | 0.34446441 |
| ATRAID | -0.189285266 | 0.039097754 | 0.214419722 | Down | 1.407848192 |
| CAD | 0.015146754 | 0.880325266 | 0.932334579 | NS | 0.055356833 |
| SLC30A3 | 0.845189624 | 0.065376413 | 0.264481527 | NS | 1.184578911 |
| DNAJC5G | 0.454216632 | 0.349418435 | NA | NS | 0.456654186 |
| TRIM54 | 0.419836124 | 0.319853157 | NA | NS | 0.495049358 |
| UCN | 0.167084763 | 0.400034981 | NA | NS | 0.397902031 |
| MPV17 | -0.004331744 | 0.917272213 | 0.954273109 | NS | 0.037501763 |
| GTF3C2 | 0.023050299 | 0.688948551 | 0.815257617 | NS | 0.161813209 |
| EIF2B4 | -0.081837397 | 0.091461361 | 0.303220626 | NS | 1.038762342 |
| SNX17 | -0.139885414 | 0.056647021 | 0.249967258 | NS | 1.246822925 |
| ZNF513 | -0.036986247 | 0.557712451 | 0.723503487 | NS | 0.253589659 |
| PPM1G | -0.169896578 | 0.015120195 | 0.147229037 | Down | 1.820442621 |
| NRBP1 | 0.012377742 | 0.819532237 | 0.897114813 | NS | 0.086433959 |
| KRTCAP3 | 0.054092035 | 0.75493704 | 0.85656564 | NS | 0.122089266 |
| IFT172 | -0.079575445 | 0.353295512 | 0.566794858 | NS | 0.451861879 |
| FNDC4 | 0.485266458 | 0.321494395 | NA | NS | 0.492826595 |
| GCKR | 0.816279746 | 0.098030162 | 0.312773304 | NS | 1.008640279 |
| C2orf16 | 0.151130341 | 0.27871631 | 0.503079817 | NS | 0.554837616 |
| ZNF512 | -0.012569205 | 0.789418247 | 0.878537356 | NS | 0.102692839 |
| CCDC121 | 0.022575484 | 0.783462779 | 0.874811457 | NS | 0.105981631 |
| GPN1 | -0.112844113 | 0.127805296 | 0.349398501 | NS | 0.893451149 |
| SUPT7L | -0.05046791 | 0.36315009 | 0.575561911 | NS | 0.439913844 |
| SLC4A1AP | -0.185884477 | 1.88E-04 | 0.022715356 | Down | 3.724800259 |
| MRPL33 | -0.085419931 | 0.414522315 | 0.616134093 | NS | 0.382452085 |
| RBKS | -0.060660482 | 0.475077813 | 0.663186156 | NS | 0.323235252 |
| BABAM2 | 0.004401717 | 0.922437127 | 0.95733572 | NS | 0.035063225 |
| FOSL2 | 0.105941844 | 0.359701604 | 0.572632152 | NS | 0.444057625 |
| PLB1 | 0.149870113 | 0.246504483 | 0.474646517 | NS | 0.608175178 |
| PPP1CB | -0.069790174 | 0.410746472 | 0.613517094 | NS | 0.386426158 |
| SPDYA | 0.006227326 | 0.939419454 | 0.966600069 | NS | 0.02714045 |
| TRMT61B | -0.248689049 | 5.29E-04 | 0.03934337 | Down | 3.276451136 |
| WDR43 | -0.16596951 | 0.023911937 | 0.175295276 | Down | 1.621385241 |
| TOGARAM2 | -0.035229112 | 0.75222304 | 0.85466151 | NS | 0.123653369 |
| PCARE | 0.213542395 | 0.521370979 | 0.697170442 | NS | 0.282853146 |
| CLIP4 | -0.039936 | 0.434349086 | 0.630686195 | NS | 0.362161088 |
| ALK | 0.248603788 | 0.09440199 | 0.307427263 | NS | 1.025018851 |
| YPEL5 | -0.092128853 | 0.229299366 | 0.457791831 | NS | 0.639597147 |
| LBH | -0.192414237 | 0.024846478 | 0.17860453 | Down | 1.604735168 |
| LCLAT1 | -0.004304043 | 0.959011607 | 0.977275243 | NS | 0.018176137 |
| CAPN13 | 0.351593367 | 0.291771241 | 0.514555379 | NS | 0.534957518 |
| GALNT14 | 0.159415971 | 0.394859819 | 0.600495212 | NS | 0.403557057 |
| CAPN14 | 0.111240845 | 0.429856281 | 0.627282309 | NS | 0.366676723 |
| EHD3 | 0.057590388 | 0.602031992 | 0.75578231 | NS | 0.22038043 |
| XDH | 0.419804738 | 0.339025025 | 0.554953265 | NS | 0.469768243 |
| SRD5A2 | 0.774880952 | 0.076986494 | NA | NS | 1.113585459 |
| MEMO1 | 0.22174019 | 0.002328048 | 0.074444948 | Up | 2.633008076 |
| DPY30 | -0.151930771 | 0.213372509 | 0.443180155 | NS | 0.670861536 |
| SPAST | -0.081512354 | 0.179943162 | 0.409996481 | NS | 0.744864652 |
| SLC30A6 | -0.078231165 | 0.102509331 | 0.3182848 | NS | 0.989236603 |
| NLRC4 | 0.031251567 | 0.749585642 | 0.852934386 | NS | 0.125178741 |
| YIPF4 | -0.032721374 | 0.508827375 | 0.688232771 | NS | 0.293429532 |
| BIRC6 | 0.013270038 | 0.799092327 | 0.884439033 | NS | 0.09740304 |
| TTC27 | -0.15707987 | 0.014704071 | 0.14533994 | Down | 1.832562399 |
| LTBP1 | -0.152798789 | 0.324787222 | 0.543296834 | NS | 0.488401066 |
| RASGRP3 | 0.108988879 | 0.449439633 | 0.642954993 | NS | 0.347328633 |
| FAM98A | 0.074349736 | 0.180488032 | 0.410276616 | NS | 0.743551591 |
| CRIM1 | 0.086175865 | 0.214897197 | 0.444145438 | NS | 0.667769249 |
| FEZ2 | 0.032891815 | 0.471752841 | 0.660508711 | NS | 0.326285476 |
| VIT | 0.456260699 | 0.095476675 | 0.309119979 | NS | 1.020102716 |
| STRN | 0.057495125 | 0.216124052 | 0.445475027 | NS | 0.6652969 |
| HEATR5B | 0.016360982 | 0.706389826 | 0.826364601 | NS | 0.150955564 |
| GPATCH11 | -0.124215295 | 0.126242718 | 0.348045109 | NS | 0.898793665 |
| EIF2AK2 | 0.375220475 | 1.00E-04 | 0.015913 | Up | 3.998640317 |
| SULT6B1 | 0.472868577 | 0.387447853 | NA | NS | 0.411786742 |
| CEBPZOS | 0.136227117 | 0.012809803 | 0.136078062 | Up | 1.892457554 |
| CEBPZ | -0.249982847 | 0.006872008 | 0.109855975 | Down | 2.162916372 |
| NDUFAF7 | -0.045994678 | 0.293932411 | 0.515962844 | NS | 0.531752523 |
| PRKD3 | -0.095890761 | 0.086074425 | 0.29485869 | NS | 1.065125868 |
| QPCT | 0.105907561 | 0.525602569 | 0.700742231 | NS | 0.279342521 |
| CDC42EP3 | 0.15832712 | 0.018772132 | 0.159116357 | Up | 1.72648639 |
| RMDN2 | -0.114130986 | 0.143524602 | 0.368714157 | NS | 0.84307365 |
| CYP1B1 | 0.008536195 | 0.950226568 | 0.973085712 | NS | 0.022172831 |
| ATL2 | -0.118144818 | 0.029197134 | 0.1903921 | Down | 1.534659778 |
| HNRNPLL | -0.024841807 | 0.604308582 | 0.757278886 | NS | 0.218741238 |
| GALM | -0.165988153 | 0.027481443 | 0.186246285 | Down | 1.560960464 |
| SRSF7 | -0.132217119 | 0.030544707 | 0.194040657 | Down | 1.515064032 |
| GEMIN6 | -0.16148599 | 0.148381764 | 0.374564243 | NS | 0.828619469 |
| DHX57 | 7.81E-04 | 0.988857271 | 0.993516162 | NS | 0.004866389 |
| MORN2 | -0.234248677 | 0.264087458 | 0.490257438 | NS | 0.578252224 |
| ARHGEF33 | 0.228019492 | 0.066498467 | 0.265925581 | NS | 1.177188369 |
| SOS1 | 0.120833745 | 0.041487421 | 0.21947395 | Up | 1.382083562 |
| CDKL4 | 0.290843158 | 0.348756565 | NA | NS | 0.457477608 |
| MAP4K3 | -0.073204011 | 0.273547126 | 0.498708509 | NS | 0.562967843 |
| TMEM178A | 0.467776976 | 0.247643208 | NA | NS | 0.606173578 |
| THUMPD2 | -0.061757257 | 0.317622906 | 0.537032996 | NS | 0.498088186 |
| SLC8A1 | 0.241300787 | 0.035485125 | 0.206049964 | Up | 1.449953659 |
| PKDCC | 0.483017053 | 0.162322254 | NA | NS | 0.789621936 |
| EML4 | -0.038978553 | 0.350630338 | 0.564808607 | NS | 0.45515051 |
| COX7A2L | -0.268219175 | 0.004819113 | 0.096288809 | Down | 2.317032913 |
| KCNG3 | 0.683463904 | 0.13766349 | NA | NS | 0.861181226 |
| MTA3 | 0.019787604 | 0.784175162 | 0.875331655 | NS | 0.105586918 |
| OXER1 | 0.387007223 | 0.020908484 | 0.167663201 | Up | 1.679677454 |
| HAAO | 0.301617493 | 0.029447721 | 0.191213981 | Up | 1.530948308 |
| ZFP36L2 | 0.116623266 | 0.220877308 | 0.449641695 | NS | 0.6558489 |
| THADA | 0.038458268 | 0.401138818 | 0.605820044 | NS | 0.396705309 |
| PLEKHH2 | 0.111110983 | 0.622806235 | 0.769743797 | NS | 0.205647048 |
| C1GALT1C1L | 0.47202617 | 0.346954759 | NA | NS | 0.459727151 |
| DYNC2LI1 | -0.209530917 | 0.081276499 | 0.287607291 | NS | 1.09003501 |
| ABCG5 | 0.631284564 | 0.091374951 | NA | NS | 1.039172841 |
| ABCG8 | 0.265700316 | 0.172801804 | 0.40169579 | NS | 0.762451728 |
| LRPPRC | -0.044719094 | 0.361616153 | 0.574243041 | NS | 0.441752178 |
| PPM1B | -0.021940109 | 0.690507174 | 0.816206209 | NS | 0.160831805 |
| SLC3A1 | 0.212501965 | 0.068521539 | 0.268602708 | NS | 1.164172892 |
| PREPL | -0.001901434 | 0.967839261 | 0.982114953 | NS | 0.014196765 |
| CAMKMT | 0.084831183 | 0.324960395 | 0.543463056 | NS | 0.488169566 |
| SIX3 | 0.873151767 | 0.076392293 | NA | NS | 1.116950452 |
| SIX2 | 0.761435273 | 0.113440377 | NA | NS | 0.94523234 |
| SRBD1 | -0.024439609 | 0.665208125 | 0.799541399 | NS | 0.177042455 |
| PRKCE | 0.168385274 | 0.029352579 | 0.190920976 | Up | 1.53235374 |
| EPAS1 | 0.351253154 | 0.021646236 | 0.16890702 | Up | 1.664617606 |
| TMEM247 | 0.612295703 | 0.263472925 | NA | NS | 0.579264007 |
| ATP6V1E2 | -0.183341496 | 0.00663375 | 0.108543277 | Down | 2.178240908 |
| RHOQ | 0.019124813 | 0.730844048 | 0.840657301 | NS | 0.136175286 |
| PIGF | 0.051621593 | 0.401201191 | 0.605820044 | NS | 0.396637786 |
| CRIPT | 0.080503018 | 0.211670338 | 0.441339592 | NS | 0.674339997 |
| SOCS5 | 0.053766303 | 0.354013755 | 0.567244316 | NS | 0.450979864 |
| MCFD2 | -0.127919966 | 0.050316473 | 0.237303925 | NS | 1.298289811 |
| TTC7A | -0.12307476 | 0.252288853 | 0.47918438 | NS | 0.598101938 |
| STPG4 | 0.119302656 | 0.476168156 | 0.664181931 | NS | 0.322239651 |
| CALM2 | -0.173612897 | 0.047891357 | 0.232522335 | Down | 1.319742859 |
| EPCAM | 0.469824646 | 0.191795394 | 0.420934764 | NS | 0.717161826 |
| MSH2 | -0.19970325 | 0.006957624 | 0.110119911 | Down | 2.157539029 |
| KCNK12 | 0.273711962 | 0.033254964 | 0.201101634 | Up | 1.47814352 |
| MSH6 | -0.065130169 | 0.14270879 | 0.367353179 | NS | 0.845549277 |
| FBXO11 | 0.096641805 | 0.064773413 | 0.26405139 | NS | 1.188603217 |
| FOXN2 | 0.003878521 | 0.956913843 | 0.976353954 | NS | 0.019127163 |
| PPP1R21 | 0.076256147 | 0.028107006 | 0.187669577 | NS | 1.551185415 |
| STON1 | 0.111666603 | 0.346938759 | 0.561433348 | NS | 0.459747179 |
| STON1-GTF2A1L | 0.547216517 | 0.298481852 | NA | NS | 0.525082069 |
| GTF2A1L | 0.150965066 | 0.813437483 | NA | NS | 0.089675819 |
| LHCGR | -0.018451809 | 0.966447284 | NA | NS | 0.01482183 |
| FSHR | 0.091636236 | 0.86277691 | NA | NS | 0.064101486 |
| NRXN1 | 0.211550643 | 0.374793044 | 0.585545419 | NS | 0.426208478 |
| ASB3 | -0.030790164 | 0.337722557 | 0.553771174 | NS | 0.471439932 |
| CHAC2 | -0.038752617 | 0.629983607 | 0.774734714 | NS | 0.200670751 |
| ERLEC1 | -0.093936906 | 0.196178609 | 0.425986774 | NS | 0.707348349 |
| GPR75 | 0.062250344 | 0.637114672 | 0.779069904 | NS | 0.195782393 |
| PSME4 | 0.030501239 | 0.803043245 | 0.886797647 | NS | 0.095261067 |
| ACYP2 | -0.103117729 | 0.270517425 | 0.495915885 | NS | 0.567804755 |
| TSPYL6 | 0.255809868 | 0.233543086 | 0.461882881 | NS | 0.631632985 |
| C2orf73 | 0.626326456 | 0.097970504 | NA | NS | 1.008904657 |
| SPTBN1 | -0.053671636 | 0.517981703 | 0.69521796 | NS | 0.285685581 |
| EML6 | 0.071614096 | 0.422934607 | 0.622376686 | NS | 0.373726777 |
| RTN4 | 0.084181518 | 0.061680673 | 0.258215532 | NS | 1.209850893 |
| CLHC1 | -0.190542393 | 0.219137041 | 0.448551211 | NS | 0.659284207 |
| RPS27A | -0.378199657 | 0.035083619 | 0.205352924 | Down | 1.454895612 |
| MTIF2 | -0.157310212 | 0.013212091 | 0.137913995 | Down | 1.879028449 |
| CCDC88A | -0.010535564 | 0.879207538 | 0.931881903 | NS | 0.055908597 |
| CFAP36 | -0.017837434 | 0.805135401 | 0.887901589 | NS | 0.094131077 |
| PPP4R3B | -0.092286791 | 0.040988893 | 0.218105862 | NS | 1.387333809 |
| PNPT1 | -0.14035765 | 0.079438684 | 0.284900136 | NS | 1.09996796 |
| EFEMP1 | 0.554121263 | 0.259091461 | NA | NS | 0.5865469 |
| CCDC85A | 1.250019261 | 0.009417116 | NA | Up | 2.026082088 |
| VRK2 | -0.110647099 | 0.177758096 | 0.407680235 | NS | 0.75017061 |
| FANCL | -0.440807192 | 4.99E-04 | 0.038126146 | Down | 3.301529251 |
| BCL11A | 0.089661136 | 0.37205769 | 0.583021911 | NS | 0.429389714 |
| PAPOLG | -0.017575255 | 0.622843703 | 0.769743797 | NS | 0.205620922 |
| REL | 0.106158245 | 0.033803372 | 0.20229026 | Up | 1.471039975 |
| PUS10 | 0.06867707 | 0.198856835 | 0.428784342 | NS | 0.701459477 |
| PEX13 | -0.160874218 | 0.005879751 | 0.103006385 | Down | 2.23064107 |
| SANBR | 0.048462044 | 0.459143433 | 0.650538413 | NS | 0.338051622 |
| C2orf74 | -0.148675208 | 0.285122695 | 0.508240968 | NS | 0.544968213 |
| USP34 | -0.016321803 | 0.811312015 | 0.891757908 | NS | 0.090812092 |
| XPO1 | -0.219153172 | 0.003279297 | 0.084967003 | Down | 2.484219184 |
| FAM161A | -0.059314493 | 0.640549868 | 0.782168094 | NS | 0.193447054 |
| CCT4 | -0.243031143 | 0.008612366 | 0.118495713 | Down | 2.064877527 |
| COMMD1 | -0.109848818 | 0.166702184 | 0.394802555 | NS | 0.778058711 |
| B3GNT2 | -0.071826848 | 0.339316914 | 0.554994713 | NS | 0.469394491 |
| TMEM17 | 0.168541379 | 0.431233576 | 0.62817783 | NS | 0.365287432 |
| EHBP1 | -0.047878519 | 0.421761146 | 0.621413862 | NS | 0.374933431 |
| OTX1 | 0.449846846 | 0.088408037 | 0.298422198 | NS | 1.053508252 |
| WDPCP | 0.009558969 | 0.853465788 | 0.917331246 | NS | 0.068813883 |
| MDH1 | -0.157519761 | 0.081554858 | 0.287924982 | NS | 1.088550163 |
| UGP2 | -0.067752931 | 0.150892094 | 0.37740073 | NS | 0.821333514 |
| VPS54 | -0.073894044 | 0.095565643 | 0.309119979 | NS | 1.019698212 |
| PELI1 | 0.069492134 | 0.490069559 | 0.674615197 | NS | 0.309742273 |
| LGALSL | -0.047291938 | 0.702601288 | 0.82383594 | NS | 0.153291058 |
| AFTPH | -0.141078068 | 0.006660578 | 0.108569793 | Down | 2.176488075 |
| SERTAD2 | 0.103280769 | 0.214215194 | 0.443419982 | NS | 0.669149729 |
| SLC1A4 | 0.1167413 | 0.108607452 | 0.326637903 | NS | 0.964140377 |
| CEP68 | 0.16597778 | 0.007530595 | 0.11304493 | Up | 2.123170681 |
| RAB1A | 0.103689558 | 0.055250407 | 0.24830006 | NS | 1.257664521 |
| ACTR2 | 0.044528888 | 0.538762491 | 0.709653107 | NS | 0.268602648 |
| SPRED2 | 0.17797713 | 0.279297806 | 0.503278325 | NS | 0.553932476 |
| MEIS1 | -0.142202751 | 0.265543853 | 0.491631796 | NS | 0.575863748 |
| ETAA1 | -0.196211006 | 0.038990619 | 0.214288889 | Down | 1.409039868 |
| C1D | 0.058396149 | 0.639622841 | 0.781260923 | NS | 0.194076036 |
| DNAAF10 | 0.017820345 | 0.822101742 | 0.898657538 | NS | 0.085074432 |
| PNO1 | -0.143928703 | 0.045469698 | 0.2276666 | Down | 1.342277928 |
| PPP3R1 | -0.106819202 | 0.42962905 | 0.627190497 | NS | 0.366906361 |
| CNRIP1 | 0.270864374 | 0.104711579 | 0.320720021 | NS | 0.98000529 |
| PLEK | 0.17197476 | 0.021272287 | 0.168379028 | Up | 1.67218582 |
| FBXO48 | -0.027271553 | 0.787253964 | 0.87719095 | NS | 0.103885144 |
| APLF | -0.234427092 | 0.134489264 | 0.358023145 | NS | 0.871312384 |
| PROKR1 | 0.569336532 | 0.122110165 | NA | NS | 0.913248181 |
| ARHGAP25 | -0.152374364 | 0.050154673 | 0.236833467 | NS | 1.299688599 |
| BMP10 | 0.233642332 | 0.434582286 | 0.630833536 | NS | 0.36192798 |
| GKN2 | 0.337046365 | 0.455839891 | NA | NS | 0.341187672 |
| GKN1 | 0.326178522 | 0.458966321 | NA | NS | 0.338219181 |
| ANTXR1 | 0.288420572 | 0.292417758 | 0.515229381 | NS | 0.533996257 |
| GFPT1 | -0.018066708 | 0.6731744 | 0.805319688 | NS | 0.171872408 |
| NFU1 | -0.163382872 | 0.155590819 | 0.38303138 | NS | 0.808016034 |
| AAK1 | 0.130407745 | 0.147834001 | 0.373699795 | NS | 0.83022567 |
| ANXA4 | -0.036801376 | 0.625866782 | 0.771788905 | NS | 0.203518098 |
| GMCL1 | -0.079898297 | 0.138934252 | 0.363222438 | NS | 0.857190672 |
| SNRNP27 | -0.006207305 | 0.931519161 | 0.962676894 | NS | 0.030808208 |
| MXD1 | 0.165576884 | 0.213836536 | 0.443234232 | NS | 0.66991809 |
| PCBP1 | -0.016494533 | 0.9268368 | 0.959776469 | NS | 0.032996731 |
| C2orf42 | -0.119812669 | 0.028785816 | 0.189386026 | Down | 1.540821457 |
| TIA1 | -0.063172322 | 0.235487882 | 0.463333673 | NS | 0.628031437 |
| PCYOX1 | -0.173154845 | 0.022781409 | 0.171982657 | Down | 1.642419416 |
| SNRPG | -0.235346675 | 0.144754008 | 0.369914478 | NS | 0.839369402 |
| FAM136A | -0.156730067 | 0.025875476 | 0.182170348 | Down | 1.587111659 |
| TGFA | 0.336667031 | 0.021242555 | 0.168379028 | Up | 1.672793242 |
| ADD2 | -0.108170612 | 0.385278665 | 0.593556772 | NS | 0.414225039 |
| FIGLA | 1.160509555 | 0.024131694 | NA | Up | 1.617412187 |
| CLEC4F | 0.17463525 | 0.454433789 | 0.646746373 | NS | 0.342529384 |
| CD207 | 0.587567406 | 0.145569509 | NA | NS | 0.836929581 |
| VAX2 | 0.87504527 | 0.069198916 | NA | NS | 1.159900712 |
| ATP6V1B1 | 0.754375209 | 0.113054898 | NA | NS | 0.946710617 |
| ANKRD53 | 0.50519771 | 0.011073794 | 0.129467323 | Up | 1.955703551 |
| TEX261 | 0.239075341 | 0.003909168 | 0.089796534 | Up | 2.407915635 |
| NAGK | 0.009104914 | 0.863921476 | 0.923140835 | NS | 0.06352573 |
| MCEE | -0.055373057 | 0.425586911 | 0.623837924 | NS | 0.371011738 |
| MPHOSPH10 | -0.127401486 | 0.066226887 | 0.2655108 | NS | 1.178965658 |
| PAIP2B | -0.095585446 | 0.228210216 | 0.456877344 | NS | 0.641664918 |
| ZNF638 | -0.130462914 | 0.012507117 | 0.135545097 | Down | 1.902842771 |
| DYSF | 0.018017476 | 0.917762988 | 0.954566872 | NS | 0.037269461 |
| CYP26B1 | 0.267889468 | 0.55326788 | 0.720308169 | NS | 0.257064542 |
| EXOC6B | -0.061631121 | 0.384268211 | 0.593034804 | NS | 0.415365542 |
| SPR | -0.150961099 | 0.355890661 | 0.568903557 | NS | 0.448683408 |
| EMX1 | 0.405294887 | 0.077563761 | 0.28177684 | NS | 1.110341143 |
| SFXN5 | 0.020464192 | 0.777016966 | 0.870268122 | NS | 0.109569498 |
| RAB11FIP5 | 0.014668562 | 0.89010007 | 0.93792961 | NS | 0.050561165 |
| NOTO | 0.924886559 | 0.043184448 | NA | Up | 1.36467263 |
| SMYD5 | 0.243050467 | 0.021366099 | 0.168379028 | Up | 1.670274768 |
| PRADC1 | -0.211429583 | 0.038621882 | 0.213650843 | Down | 1.413166564 |
| CCT7 | -0.173225382 | 0.023073668 | 0.172916604 | Down | 1.636883363 |
| FBXO41 | 0.224211807 | 0.094982016 | 0.308375125 | NS | 1.022358617 |
| EGR4 | 1.038129667 | 0.027668297 | NA | Up | 1.558017565 |
| ALMS1 | -0.037163352 | 0.60763987 | 0.759420118 | NS | 0.216353738 |
| NAT8 | 0.649240288 | 0.035398311 | NA | Up | 1.45101746 |
| TPRKB | -0.269855751 | 0.027323069 | 0.185919029 | Down | 1.563470517 |
| DUSP11 | -0.086862134 | 0.0875406 | 0.297103561 | NS | 1.05779048 |
| C2orf78 | 0.527526014 | 0.237531687 | NA | NS | 0.624278448 |
| STAMBP | 0.056994817 | 0.077037473 | 0.28141561 | NS | 1.113297972 |
| ACTG2 | 0.249495874 | 0.567327023 | 0.730611561 | NS | 0.246166529 |
| DGUOK | 0.054675458 | 0.277711405 | 0.502063988 | NS | 0.556406284 |
| TET3 | -0.097067067 | 0.386535604 | 0.594562322 | NS | 0.412810497 |
| BOLA3 | 0.08402406 | 0.557077666 | 0.722925697 | NS | 0.254084253 |
| MOB1A | 0.030359723 | 0.671812875 | 0.804363157 | NS | 0.172751677 |
| MTHFD2 | -0.103299821 | 0.241136128 | 0.469121611 | NS | 0.617737717 |
| SLC4A5 | 0.279119693 | 0.012114577 | 0.134107677 | Up | 1.916691751 |
| DCTN1 | 0.142701594 | 0.111655037 | 0.330121825 | NS | 0.95212168 |
| C2orf81 | 0.410373447 | 0.259337133 | NA | NS | 0.586135295 |
| WDR54 | -0.12156132 | 0.145434366 | 0.37059965 | NS | 0.837332958 |
| RTKN | 0.322975676 | 0.090992499 | 0.302059748 | NS | 1.040994409 |
| INO80B | 0.042377821 | 0.699044321 | 0.821855116 | NS | 0.155495288 |
| WBP1 | -0.118248034 | 0.118294571 | 0.338732467 | NS | 0.927035185 |
| MOGS | -0.112863753 | 0.103415784 | 0.31928753 | NS | 0.985413173 |
| CCDC142 | -0.066039975 | 0.185599159 | 0.415463304 | NS | 0.731423995 |
| TTC31 | -0.052616687 | 0.510285881 | 0.689437709 | NS | 0.292186448 |
| LBX2 | 0.043267084 | 0.814910964 | 0.89432791 | NS | 0.088889839 |
| PCGF1 | 0.03318861 | 0.618215498 | 0.766432243 | NS | 0.208860112 |
| TLX2 | 1.146426827 | 0.003705307 | NA | Up | 2.431175836 |
| DQX1 | -0.110082027 | 0.642655 | 0.783815879 | NS | 0.192022109 |
| AUP1 | -0.242660974 | 1.38E-04 | 0.018665732 | Down | 3.860941687 |
| HTRA2 | -0.118396451 | 0.042082135 | 0.220751111 | Down | 1.375902235 |
| LOXL3 | 0.01034408 | 0.906285341 | 0.94801213 | NS | 0.042735045 |
| DOK1 | -0.059654844 | 0.394876338 | 0.600495212 | NS | 0.403538889 |
| M1AP | 0.092843117 | 0.407464174 | 0.611075752 | NS | 0.38991057 |
| SEMA4F | -0.03576992 | 0.626562678 | 0.772227879 | NS | 0.203035478 |
| HK2 | 0.069715994 | 0.494547616 | 0.677764915 | NS | 0.305791888 |
| POLE4 | -0.109580722 | 0.300218065 | 0.52166875 | NS | 0.522563178 |
| TACR1 | 0.121480655 | 0.396177995 | 0.601442813 | NS | 0.40210965 |
| EVA1A | 0.343872591 | 0.372606111 | NA | NS | 0.428750027 |
| MRPL19 | 0.042882473 | 0.429091515 | 0.626769724 | NS | 0.367450073 |
| GCFC2 | -0.121397182 | 0.077148443 | 0.281480585 | NS | 1.112672836 |
| LRRTM4 | 0.495938211 | 0.338090146 | NA | NS | 0.470967487 |
| REG3G | 0.476271618 | 0.366452251 | NA | NS | 0.435982606 |
| REG1B | 0.202973888 | 0.676012129 | NA | NS | 0.170045512 |
| REG1A | 0.812829208 | 0.088872291 | NA | NS | 1.051233626 |
| REG3A | 0.788974687 | 0.189983342 | NA | NS | 0.721284478 |
| CTNNA2 | 0.32545222 | 0.367528521 | 0.579612876 | NS | 0.434708953 |
| LRRTM1 | 0.454637143 | 0.340234227 | NA | NS | 0.468222 |
| SUCLG1 | -0.038298087 | 0.586396203 | 0.745097972 | NS | 0.23180885 |
| DNAH6 | 0.139132521 | 0.345973464 | 0.560904975 | NS | 0.46095721 |
| TRABD2A | -0.059556044 | 0.577086354 | 0.737669138 | NS | 0.238759195 |
| TMSB10 | -0.210113271 | 0.087597945 | 0.29716607 | NS | 1.057506082 |
| KCMF1 | -0.011164883 | 0.821113635 | 0.898091813 | NS | 0.085596736 |
| TCF7L1 | 0.222736758 | 0.197002088 | 0.426763319 | NS | 0.705529172 |
| TGOLN2 | 0.04090801 | 0.558077423 | 0.723812954 | NS | 0.253305546 |
| RETSAT | -0.062616949 | 0.301033243 | 0.522450996 | NS | 0.521385542 |
| ELMOD3 | -0.009037231 | 0.844838579 | 0.912966221 | NS | 0.073226263 |
| CAPG | 0.090035369 | 0.31329403 | 0.532986174 | NS | 0.504047881 |
| SH2D6 | 0.219037574 | 0.618938687 | NA | NS | 0.208352371 |
| MAT2A | 0.089901977 | 0.019797899 | 0.163424633 | NS | 1.703380885 |
| GGCX | 0.083446256 | 0.084612314 | 0.292637999 | NS | 1.072566429 |
| VAMP8 | -0.254770899 | 0.032741946 | 0.199748547 | Down | 1.484895514 |
| VAMP5 | 0.119991498 | 0.273484709 | 0.498634409 | NS | 0.563066951 |
| RNF181 | -0.160304118 | 0.073955851 | 0.276212801 | NS | 1.131027463 |
| TMEM150A | 0.176542696 | 0.038006656 | 0.212212115 | Up | 1.42014034 |
| USP39 | -0.073697588 | 0.09557587 | 0.309119979 | NS | 1.019651741 |
| C2orf68 | -1.69E-04 | 0.996185655 | 0.997710407 | NS | 0.001659716 |
| SFTPB | 0.05273926 | 0.804682768 | 0.887737559 | NS | 0.094375299 |
| GNLY | -0.514941255 | 1.41E-04 | 0.019026652 | Down | 3.849438476 |
| ATOH8 | -0.222348863 | 0.366152237 | 0.57858521 | NS | 0.436338308 |
| ST3GAL5 | 0.007546742 | 0.895875044 | 0.941593862 | NS | 0.047752561 |
| POLR1A | 6.10E-04 | 0.993220255 | 0.996047216 | NS | 0.002954432 |
| PTCD3 | -0.112799854 | 0.019678149 | 0.162913934 | Down | 1.706015752 |
| IMMT | -0.114225173 | 0.026998541 | 0.185292358 | Down | 1.568659697 |
| MRPL35 | -0.15192063 | 0.052983818 | 0.24325135 | NS | 1.275856752 |
| REEP1 | 0.134415404 | 0.29465232 | 0.516741514 | NS | 0.530690135 |
| KDM3A | 0.049014422 | 0.270833287 | 0.496051178 | NS | 0.56729796 |
| CHMP3 | -0.065122414 | 0.190838381 | 0.420152441 | NS | 0.719334277 |
| RNF103-CHMP3 | 0.415698049 | 0.01145863 | 0.131355811 | Up | 1.940867293 |
| RNF103 | 0.148243763 | 0.017479891 | 0.155202892 | Up | 1.757461276 |
| RMND5A | -0.187731276 | 0.047534089 | 0.231617346 | Down | 1.322994829 |
| CD8A | -0.195398515 | 0.086832993 | 0.296188519 | NS | 1.061315231 |
| CD8B | -0.180407481 | 0.112020159 | 0.330785464 | NS | 0.950703816 |
| RGPD1 | 0.192055674 | 0.257224842 | 0.484200085 | NS | 0.58968709 |
| PLGLB1 | 0.26492217 | 0.028135939 | 0.18774505 | Up | 1.550738585 |
| PLGLB2 | 0.488450668 | 0.008995484 | 0.120055109 | Up | 2.045975482 |
| RGPD2 | 0.078431351 | 0.704082782 | 0.824734454 | NS | 0.152376276 |
| KRCC1 | -0.165442542 | 0.125253261 | 0.346840099 | NS | 0.902210959 |
| SMYD1 | 0.473998742 | 0.373463976 | 0.583986889 | NS | 0.427751283 |
| FABP1 | 0.249507458 | 0.64438354 | NA | NS | 0.190855562 |
| THNSL2 | -0.177228935 | 0.543047592 | 0.712919229 | NS | 0.265162108 |
| FOXI3 | 1.277602345 | 0.015056612 | NA | Up | 1.82227274 |
| TEX37 | 0.205376103 | 0.753548607 | NA | NS | 0.122888728 |
| EIF2AK3 | -0.0309522 | 0.543823827 | 0.713285597 | NS | 0.264541768 |
| RPIA | -0.389357814 | 0.018941812 | 0.159722155 | Down | 1.722578485 |
| TEKT4 | 0.966860807 | 0.078570025 | NA | NS | 1.10474311 |
| MAL | -0.126827772 | 0.303002662 | 0.524319378 | NS | 0.518553556 |
| MRPS5 | -0.066063334 | 0.094048764 | 0.30725936 | NS | 1.026646906 |
| ZNF514 | -0.0927299 | 0.111253011 | 0.329784684 | NS | 0.953688224 |
| ZNF2 | 0.091081058 | 0.090268661 | 0.300982388 | NS | 1.044462997 |
| PROM2 | 0.605642893 | 0.097083752 | 0.311358645 | NS | 1.012853448 |
| KCNIP3 | 0.498072858 | 0.270943887 | 0.496214067 | NS | 0.567120643 |
| FAHD2A | -0.066100147 | 0.241172635 | 0.469152781 | NS | 0.617671971 |
| TRIM43B | 0.362530457 | 0.616078511 | NA | NS | 0.210363939 |
| TRIM43 | -0.284215304 | 0.702839564 | NA | NS | 0.153143799 |
| ANKRD36C | -0.147693484 | 0.07223175 | 0.273882705 | NS | 1.141271863 |
| GPAT2 | -0.05633703 | 0.72388536 | 0.837167223 | NS | 0.140330206 |
| ADRA2B | 0.724805927 | 0.138670793 | NA | NS | 0.858015 |
| ASTL | 0.446616467 | 0.105697689 | 0.321835351 | NS | 0.975934509 |
| DUSP2 | -0.272008523 | 0.06646051 | 0.265911706 | NS | 1.177436329 |
| STARD7 | -0.194808719 | 0.017199652 | 0.154054413 | Down | 1.764480339 |
| TMEM127 | 0.189044644 | 0.026054257 | 0.182704458 | Up | 1.584121306 |
| CIAO1 | -0.045307718 | 0.22890962 | 0.457459696 | NS | 0.640335956 |
| SNRNP200 | -0.025603894 | 0.623518637 | 0.770308731 | NS | 0.205150561 |
| ITPRIPL1 | -0.123198878 | 0.155854585 | 0.383185333 | NS | 0.807280416 |
| NCAPH | -0.16289106 | 0.390834651 | 0.597761105 | NS | 0.408006939 |
| NEURL3 | 0.368452596 | 0.227880757 | NA | NS | 0.642292347 |
| ARID5A | 0.073871324 | 0.366219609 | 0.57858521 | NS | 0.436258405 |
| KANSL3 | 0.135493748 | 0.074475123 | 0.277060776 | NS | 1.127988771 |
| FER1L5 | 0.147898642 | 0.376846573 | 0.586981039 | NS | 0.42383543 |
| LMAN2L | -0.114017283 | 0.061923597 | 0.25855501 | NS | 1.208143822 |
| CNNM4 | 0.017035997 | 0.811958129 | 0.892253974 | NS | 0.090466366 |
| CNNM3 | -0.025115749 | 0.670524568 | 0.803450722 | NS | 0.173585305 |
| ANKRD23 | 0.151742136 | 0.140998328 | 0.365370413 | NS | 0.850786037 |
| ANKRD39 | 0.101964608 | 0.098864916 | 0.313732063 | NS | 1.004957799 |
| SEMA4C | -0.13134076 | 0.105527975 | 0.321700573 | NS | 0.976632396 |
| FAM178B | 0.721362603 | 0.031846781 | 0.197978516 | Up | 1.496934455 |
| FAHD2B | -0.002522393 | 0.977417422 | 0.986810695 | NS | 0.009919924 |
| ANKRD36 | -0.110387476 | 0.262124425 | 0.488365675 | NS | 0.581492509 |
| ANKRD36B | -0.158186149 | 0.113863376 | 0.332423088 | NS | 0.943615945 |
| COX5B | 0.004658716 | 0.963896888 | 0.979855587 | NS | 0.015969422 |
| ACTR1B | -0.164363007 | 0.003232158 | 0.084506638 | Down | 2.490507457 |
| C2orf92 | 0.221406099 | 0.040053801 | 0.215939772 | Up | 1.397356264 |
| ZAP70 | -0.075354985 | 0.299308629 | 0.520641517 | NS | 0.523880763 |
| TMEM131 | 0.114527368 | 0.080555162 | 0.286274601 | NS | 1.093906623 |
| VWA3B | 0.446560104 | 0.257776212 | 0.484730205 | NS | 0.588757162 |
| CNGA3 | 0.764379818 | 0.119642519 | NA | NS | 0.922114453 |
| INPP4A | 0.004403925 | 0.934424203 | 0.963841963 | NS | 0.029455921 |
| COA5 | -0.048460344 | 0.345639595 | 0.560549771 | NS | 0.461376513 |
| UNC50 | -0.103486112 | 0.046412779 | 0.229359492 | Down | 1.333362427 |
| MGAT4A | -0.031305209 | 0.590505296 | 0.7478297 | NS | 0.228776203 |
| CRACDL | 0.206349703 | 0.139746003 | 0.36397707 | NS | 0.854660604 |
| TSGA10 | 0.016546149 | 0.874422952 | 0.929282341 | NS | 0.058278451 |
| C2orf15 | -0.157992668 | 0.181452446 | 0.411407179 | NS | 0.741237173 |
| LIPT1 | -0.029785716 | 0.746215152 | 0.850874753 | NS | 0.127135937 |
| MITD1 | -0.140803971 | 0.04127606 | 0.218820448 | Down | 1.384301761 |
| MRPL30 | -0.098385076 | 0.189939521 | 0.419276618 | NS | 0.721384662 |
| LYG2 | 0.247850863 | 0.186219663 | 0.415835484 | NS | 0.729974464 |
| LYG1 | 0.050384905 | 0.619133716 | 0.767117527 | NS | 0.208215545 |
| TXNDC9 | -0.157022055 | 0.185243093 | 0.415416766 | NS | 0.732257975 |
| EIF5B | 0.063307704 | 0.317067047 | 0.5364679 | NS | 0.498848893 |
| REV1 | -0.084631608 | 0.072287712 | 0.273921771 | NS | 1.140935523 |
| AFF3 | 0.112849967 | 0.318665446 | 0.53773033 | NS | 0.496665027 |
| LONRF2 | 0.555687333 | 0.032226156 | 0.19828377 | Up | 1.491791496 |
| CHST10 | 0.030652007 | 0.751575931 | 0.854265322 | NS | 0.124027137 |
| NMS | 0.274627721 | 0.634947131 | NA | NS | 0.197262435 |
| PDCL3 | -0.168233129 | 0.113713664 | 0.332423088 | NS | 0.944187347 |
| NPAS2 | 0.601625683 | 0.022337579 | 0.170866811 | Up | 1.6509639 |
| RPL31 | -0.749008951 | 0.008525885 | 0.118160901 | Down | 2.069260545 |
| TBC1D8 | 0.002105825 | 0.976510379 | 0.986397233 | NS | 0.010323136 |
| CNOT11 | 0.107902486 | 0.008060673 | 0.115486104 | Up | 2.093628676 |
| RNF149 | 0.069058052 | 0.476316257 | 0.664231464 | NS | 0.322104595 |
| CREG2 | 0.248340125 | 0.268866367 | 0.494425689 | NS | 0.570463522 |
| RFX8 | -0.032905279 | 0.891280548 | 0.93866031 | NS | 0.049985572 |
| MAP4K4 | -0.013639076 | 0.849222708 | 0.915233404 | NS | 0.070978401 |
| IL1R2 | 0.294581069 | 0.10388332 | 0.319776317 | NS | 0.983454179 |
| IL1R1 | 0.288623613 | 0.0896514 | 0.299979972 | NS | 1.047442924 |
| IL1RL2 | 0.186659343 | 0.646424862 | NA | NS | 0.189481949 |
| IL1RL1 | 0.061015313 | 0.76714994 | 0.864377708 | NS | 0.115119744 |
| IL18R1 | -0.173761389 | 0.07048802 | 0.271366448 | NS | 1.151884686 |
| IL18RAP | 0.195354781 | 0.272144025 | 0.497465358 | NS | 0.565201196 |
| SLC9A4 | 0.321021113 | 0.465052123 | NA | NS | 0.332498368 |
| SLC9A2 | 0.542570176 | 0.028487173 | 0.188709674 | Up | 1.545350644 |
| MFSD9 | 0.281162468 | 0.00258178 | 0.077799913 | Up | 2.588080707 |
| TMEM182 | -0.052649502 | 0.569140542 | 0.731710119 | NS | 0.244780477 |
| POU3F3 | 0.997222619 | 0.021940665 | NA | Up | 1.658750211 |
| MRPS9 | -0.189589353 | 0.008528511 | 0.118160901 | Down | 2.069126761 |
| GPR45 | 0.211427402 | 0.437920243 | NA | NS | 0.358604979 |
| TGFBRAP1 | 0.070590264 | 0.126496673 | 0.348451623 | NS | 0.897920898 |
| C2orf49 | -0.109181671 | 0.034142593 | 0.202994201 | Down | 1.4667035 |
| FHL2 | 0.065205321 | 0.389728597 | 0.596947855 | NS | 0.409237726 |
| NCK2 | -0.074443613 | 0.322908293 | 0.541577863 | NS | 0.490920802 |
| ECRG4 | 0.394586218 | 0.27298678 | NA | NS | 0.563858384 |
| UXS1 | -0.140370733 | 0.056703449 | 0.250023562 | NS | 1.246390527 |
| RGPD3 | 0.375343087 | 0.055735776 | 0.248760687 | NS | 1.25386595 |
| CD8B2 | 0.171809255 | 0.496113457 | 0.678854657 | NS | 0.304418993 |
| ST6GAL2 | 0.379222374 | 0.202839615 | 0.432901167 | NS | 0.692847222 |
| RGPD4 | 0.082649879 | 0.558043399 | 0.723809817 | NS | 0.253332024 |
| SLC5A7 | 0.413038435 | 0.366324038 | NA | NS | 0.436134582 |
| SULT1C3 | 0.296964161 | 0.549057943 | NA | NS | 0.260381821 |
| SULT1C2 | 0.429149688 | 0.320766953 | 0.539487755 | NS | 0.493810381 |
| SULT1C4 | 0.565457039 | 0.117065309 | NA | NS | 0.931571783 |
| GCC2 | -0.090890269 | 0.148722209 | 0.374763795 | NS | 0.827624173 |
| LIMS1 | -0.086309342 | 0.305290273 | 0.526578834 | NS | 0.515287032 |
| RANBP2 | -0.135635972 | 0.010553997 | 0.127927347 | Down | 1.976583034 |
| CCDC138 | -0.32821283 | 0.009535881 | 0.122730647 | Down | 2.02063916 |
| EDAR | -0.059480019 | 0.676339512 | 0.806788244 | NS | 0.16983524 |
| SH3RF3 | 0.026811888 | 0.848854155 | 0.915060384 | NS | 0.071166921 |
| SEPTIN10 | 0.017749692 | 0.873118886 | 0.928370338 | NS | 0.058926618 |
| SOWAHC | -0.112786421 | 0.316241528 | 0.535840887 | NS | 0.4999811 |
| MALL | 1.373411483 | 0.003107592 | NA | Up | 2.507576005 |
| NPHP1 | 0.263065507 | 0.249440117 | 0.47657275 | NS | 0.603033699 |
| MTLN | 0.058422993 | 0.597907002 | 0.752708122 | NS | 0.223366361 |
| BUB1 | -0.174312331 | 0.253237442 | 0.480142719 | NS | 0.596472081 |
| ACOXL | 0.460029671 | 0.027714188 | 0.186639939 | Up | 1.557297841 |
| BCL2L11 | 0.122024515 | 0.083335395 | 0.290715494 | NS | 1.079170501 |
| ANAPC1 | -0.070667023 | 0.163436675 | 0.391129522 | NS | 0.786650483 |
| MERTK | -0.162947302 | 0.242079328 | 0.469918053 | NS | 0.616042294 |
| TMEM87B | -0.011670986 | 0.799844638 | 0.884702387 | NS | 0.096994362 |
| FBLN7 | 0.10196311 | 0.347833437 | 0.562298396 | NS | 0.458628672 |
| ZC3H8 | -0.056389343 | 0.320177995 | 0.539074418 | NS | 0.494608519 |
| ZC3H6 | -0.010477467 | 0.881500079 | 0.932950451 | NS | 0.054777645 |
| RGPD8 | -0.05150773 | 0.628194555 | 0.773528315 | NS | 0.201905832 |
| TTL | -0.003514708 | 0.961456789 | 0.978517825 | NS | 0.01707023 |
| POLR1B | -0.034948904 | 0.528572232 | 0.703278992 | NS | 0.276895656 |
| CHCHD5 | 0.392022235 | 0.004357864 | 0.093367359 | Up | 2.36072632 |
| SLC20A1 | 0.091298596 | 0.131749123 | 0.354444963 | NS | 0.880252268 |
| NT5DC4 | 0.268698588 | 0.161686536 | 0.38941477 | NS | 0.791326143 |
| CKAP2L | -0.084392895 | 0.657407143 | 0.794202854 | NS | 0.182165581 |
| IL1A | -0.400839302 | 0.314320145 | NA | NS | 0.502627784 |
| IL1B | -0.03710591 | 0.769865802 | 0.865858007 | NS | 0.113584972 |
| IL37 | 0.257773095 | 0.645074478 | NA | NS | 0.19039014 |
| IL36G | 0.262102378 | 0.675751568 | NA | NS | 0.170212938 |
| IL36A | 0.430270152 | 0.239102575 | NA | NS | 0.621415747 |
| IL36B | 0.533448758 | 0.319129732 | NA | NS | 0.496032733 |
| IL36RN | 0.164285904 | 0.760820089 | NA | NS | 0.118718029 |
| IL1F10 | 0.318120773 | 0.606266834 | NA | NS | 0.217336189 |
| IL1RN | 0.326191304 | 0.003377084 | 0.085084692 | Up | 2.471458095 |
| PSD4 | -0.022225342 | 0.740513331 | 0.847850763 | NS | 0.130467119 |
| PAX8 | 0.335098848 | 0.154836017 | 0.382060801 | NS | 0.81012801 |
| CBWD2 | -0.034007285 | 0.579260078 | 0.739023693 | NS | 0.237126401 |
| FOXD4L1 | 0.269374381 | 0.23990545 | NA | NS | 0.619959887 |
| RABL2A | 0.178050887 | 0.15278836 | 0.37932805 | NS | 0.815909732 |
| SLC35F5 | -0.027347987 | 0.508421197 | 0.687886942 | NS | 0.293776351 |
| ACTR3 | -0.10484588 | 0.096661182 | 0.310822251 | NS | 1.014747899 |
| DPP10 | 0.15155692 | 0.700709982 | 0.822549366 | NS | 0.154461696 |
| DDX18 | -0.210675681 | 0.006837821 | 0.109817511 | Down | 2.165082288 |
| CCDC93 | -0.01012044 | 0.80361573 | 0.887173172 | NS | 0.094951571 |
| INSIG2 | 0.078322772 | 0.080056169 | 0.285474818 | NS | 1.096605199 |
| EN1 | 0.804314358 | 0.109926356 | NA | NS | 0.95889817 |
| MARCO | 0.060912398 | 0.735006632 | 0.843680928 | NS | 0.133708742 |
| C1QL2 | 1.84298131 | 1.95E-04 | NA | Up | 3.709978511 |
| STEAP3 | 0.20640299 | 0.223641353 | 0.452317462 | NS | 0.650447889 |
| C2orf76 | 0.082130295 | 0.429512013 | 0.627120836 | NS | 0.367024685 |
| DBI | -0.211341851 | 0.175428937 | 0.404762595 | NS | 0.755898768 |
| TMEM37 | 0.728033017 | 0.146259279 | NA | NS | 0.834876571 |
| SCTR | 0.433359126 | 0.358396963 | NA | NS | 0.445635679 |
| CFAP221 | 0.273767195 | 0.551212924 | 0.718687588 | NS | 0.258680609 |
| TMEM177 | -0.128359441 | 0.141301185 | 0.365773968 | NS | 0.849854196 |
| PTPN4 | -0.248950357 | 5.26E-04 | 0.039237697 | Down | 3.27903153 |
| EPB41L5 | 0.111401674 | 0.037496475 | 0.210180429 | Up | 1.426009561 |
| TMEM185B | 0.071950379 | 0.220619554 | 0.449602156 | NS | 0.656355998 |
| RALB | 0.05145468 | 0.61481551 | 0.764372445 | NS | 0.211255185 |
| INHBB | 0.430907918 | 0.06883571 | 0.268885457 | NS | 1.162186203 |
| GLI2 | 0.816770275 | 0.059324679 | 0.25415179 | NS | 1.226764602 |
| TFCP2L1 | 0.415300639 | 0.057775639 | 0.251746134 | NS | 1.238255244 |
| CLASP1 | 0.10617397 | 0.099619473 | 0.314750917 | NS | 1.001655758 |
| NIFK | -0.214898156 | 0.044368595 | 0.225362854 | Down | 1.35292432 |
| TSN | -0.275743137 | 2.48E-04 | 0.026250964 | Down | 3.606274411 |
| CNTNAP5 | 0.376525051 | 0.375122548 | 0.585819023 | NS | 0.425826831 |
| GYPC | -0.218490733 | 0.234160941 | 0.462306368 | NS | 0.630485545 |
| TEX51 | 0.976676514 | 0.08293275 | NA | NS | 1.081273932 |
| BIN1 | -0.17135574 | 0.035311793 | 0.205674128 | Down | 1.452080236 |
| CYP27C1 | 0.702893539 | 0.111097288 | NA | NS | 0.954296544 |
| ERCC3 | -0.019340277 | 0.554829806 | 0.721521334 | NS | 0.255840216 |
| MAP3K2 | -0.002338312 | 0.978015997 | 0.987153917 | NS | 0.009654041 |
| PROC | 0.151435526 | 0.410241224 | 0.613073051 | NS | 0.386960701 |
| IWS1 | -0.06048489 | 0.100205178 | 0.315320004 | NS | 0.999109836 |
| MYO7B | 0.155568642 | 0.207843963 | 0.437584339 | NS | 0.682262585 |
| LIMS2 | 0.300672142 | 0.066298102 | 0.265540248 | NS | 1.178498902 |
| GPR17 | 0.973593311 | 0.011787453 | NA | Up | 1.928580033 |
| WDR33 | 0.029559326 | 0.473582174 | 0.661824442 | NS | 0.324604653 |
| POLR2D | -0.043388495 | 0.29890779 | 0.520339481 | NS | 0.524462766 |
| AMMECR1L | 0.133478142 | 0.012588902 | 0.135545097 | Up | 1.900012135 |
| SAP130 | 0.015657982 | 0.845697072 | 0.913247565 | NS | 0.072785173 |
| UGGT1 | -0.116614953 | 0.060319273 | 0.256161132 | NS | 1.2195439 |
| HS6ST1 | 0.100166671 | 0.176653254 | 0.40627765 | NS | 0.752878357 |
| RAB6C | 0.49804735 | 0.26072242 | NA | NS | 0.583821622 |
| POTEF | 0.729385844 | 0.15412892 | NA | NS | 0.812115866 |
| CCDC74B | 1.085473467 | 0.016241166 | NA | Up | 1.789382784 |
| SMPD4 | 0.224577002 | 0.011716246 | 0.132588561 | Up | 1.931211515 |
| MZT2B | -0.005478223 | 0.963787945 | 0.979831825 | NS | 0.01601851 |
| TUBA3E | 0.387570857 | 0.124262685 | NA | NS | 0.905659266 |
| CCDC115 | -0.133495229 | 0.072437069 | 0.27394225 | NS | 1.14003913 |
| IMP4 | -0.20636409 | 0.006504001 | 0.108094794 | Down | 2.186819416 |
| PTPN18 | 0.143306057 | 0.090135892 | 0.30078569 | NS | 1.04510224 |
| POTEI | 0.661178134 | 0.191722105 | NA | NS | 0.717327812 |
| CFC1 | 0.961130547 | 0.199572213 | NA | NS | 0.699899926 |
| POTEJ | 0.87467635 | 0.052100939 | NA | NS | 1.283154447 |
| GPR148 | 0.393426169 | 0.584497676 | NA | NS | 0.233217211 |
| AMER3 | 0.544660943 | 0.231254403 | NA | NS | 0.63590999 |
| ARHGEF4 | 0.101212066 | 0.422031906 | 0.621527536 | NS | 0.374654715 |
| FAM168B | -0.078914547 | 0.142135604 | 0.366657985 | NS | 0.847297122 |
| PLEKHB2 | -0.153556589 | 0.017443239 | 0.155019426 | Down | 1.758372866 |
| POTEE | 0.18970324 | 0.596039358 | NA | NS | 0.224725062 |
| RAB6D | 0.828680125 | 0.077661373 | NA | NS | 1.109794933 |
| MZT2A | -0.022300848 | 0.800268299 | 0.884970767 | NS | 0.096764386 |
| TUBA3D | 0.356348132 | 0.006796513 | 0.109684865 | Up | 2.167713834 |
| CCDC74A | 0.964900534 | 0.011782978 | NA | Up | 1.928744939 |
| ANKRD30BL | 0.691218105 | 0.104104021 | NA | NS | 0.982532495 |
| GPR39 | 0.417315119 | 0.350825663 | NA | NS | 0.454908645 |
| LYPD1 | 0.956082985 | 0.010274925 | NA | Up | 1.988221332 |
| NCKAP5 | 0.10997761 | 0.760535262 | 0.860168677 | NS | 0.118880645 |
| MGAT5 | -0.016885914 | 0.726920855 | 0.838611255 | NS | 0.138512871 |
| TMEM163 | -0.010500854 | 0.94016896 | 0.966870359 | NS | 0.026794091 |
| ACMSD | 0.104663735 | 0.487233852 | 0.672268351 | NS | 0.312262546 |
| CCNT2 | -0.002355188 | 0.965138722 | 0.980465179 | NS | 0.01541026 |
| MAP3K19 | 0.236822656 | 0.290110392 | 0.512990599 | NS | 0.537436713 |
| RAB3GAP1 | -0.00226856 | 0.950979686 | 0.973424162 | NS | 0.02182876 |
| ZRANB3 | -0.160193811 | 0.044710145 | 0.226127015 | Down | 1.349593921 |
| R3HDM1 | -0.02470547 | 0.806819859 | 0.888742142 | NS | 0.093223421 |
| UBXN4 | -0.031073657 | 0.510180286 | 0.689370991 | NS | 0.292276327 |
| LCT | 0.148412455 | 0.381936246 | 0.591380158 | NS | 0.418009125 |
| MCM6 | -0.00390902 | 0.953320602 | 0.974815753 | NS | 0.020761021 |
| DARS1 | -0.186928757 | 0.02678515 | 0.185022315 | Down | 1.572105921 |
| CXCR4 | -0.069623228 | 0.431209013 | 0.62817783 | NS | 0.36531217 |
| THSD7B | 0.297812604 | 0.489984857 | 0.674579693 | NS | 0.309817342 |
| HNMT | -0.257034569 | 0.072845574 | 0.274709259 | NS | 1.137596831 |
| SPOPL | 0.041989121 | 0.64969435 | 0.788808779 | NS | 0.18729091 |
| NXPH2 | 0.449775079 | 0.371253331 | NA | NS | 0.430329641 |
| LRP1B | 0.135709901 | 0.643238415 | 0.784127485 | NS | 0.191628027 |
| KYNU | 0.001825984 | 0.98419362 | 0.990467313 | NS | 0.006919455 |
| ARHGAP15 | 0.016699674 | 0.814681553 | 0.894216962 | NS | 0.089012118 |
| GTDC1 | -0.021609762 | 0.7219529 | 0.835903201 | NS | 0.141491135 |
| ZEB2 | 0.072170047 | 0.291090635 | 0.513839813 | NS | 0.535971766 |
| ACVR2A | 0.035170585 | 0.544160708 | 0.713285597 | NS | 0.26427282 |
| ORC4 | -0.048365856 | 0.358593628 | 0.571543689 | NS | 0.445397432 |
| MBD5 | 0.090773907 | 0.31008879 | 0.530296194 | NS | 0.508513933 |
| EPC2 | -0.098313525 | 0.029502748 | 0.191354273 | NS | 1.530137528 |
| KIF5C | -0.082043723 | 0.315059992 | 0.534442186 | NS | 0.501606742 |
| LYPD6B | 0.434121364 | 0.091521159 | 0.303287164 | NS | 1.038478487 |
| LYPD6 | 0.616732112 | 0.229565962 | NA | NS | 0.639092504 |
| MMADHC | -0.064368083 | 0.451328165 | 0.644288327 | NS | 0.345507564 |
| RND3 | 0.577267858 | 0.242575019 | NA | NS | 0.615153927 |
| RBM43 | 0.117841475 | 0.079307259 | 0.284596496 | NS | 1.100687061 |
| NMI | 0.015579403 | 0.858423157 | 0.92008732 | NS | 0.066298575 |
| TNFAIP6 | -0.019001051 | 0.92129717 | 0.956591911 | NS | 0.035600263 |
| RIF1 | -0.062143568 | 0.210466106 | 0.440341584 | NS | 0.676817833 |
| NEB | 0.19001349 | 0.055439942 | 0.248604264 | NS | 1.256177234 |
| ARL5A | -0.197391041 | 0.008111314 | 0.115845343 | Down | 2.09090876 |
| CACNB4 | 0.032693518 | 0.82443534 | 0.900078142 | NS | 0.0838434 |
| STAM2 | 0.004612705 | 0.935163824 | 0.963997025 | NS | 0.029112302 |
| FMNL2 | 0.140199522 | 0.037443102 | 0.210180429 | Up | 1.426628174 |
| PRPF40A | -0.067149352 | 0.189920735 | 0.419275561 | NS | 0.721427618 |
| ARL6IP6 | 0.146306507 | 0.005422263 | 0.100338483 | Up | 2.26581942 |
| RPRM | 0.907503447 | 0.050917134 | NA | NS | 1.293136048 |
| GALNT13 | 0.390377786 | 0.38793874 | 0.595953448 | NS | 0.411236849 |
| KCNJ3 | 0.243472141 | 0.599200479 | NA | NS | 0.222427848 |
| NR4A2 | 0.138084522 | 0.337178596 | 0.553354654 | NS | 0.472140002 |
| GPD2 | 0.004108121 | 0.939877822 | 0.966780353 | NS | 0.026928598 |
| GALNT5 | -0.12723774 | 0.47374143 | 0.662006604 | NS | 0.324458633 |
| ERMN | 0.221537381 | 0.013918792 | 0.141407346 | Up | 1.856398466 |
| CYTIP | 0.049169383 | 0.346572396 | 0.56126774 | NS | 0.460206031 |
| ACVR1C | 0.066189517 | 0.552005579 | 0.719196256 | NS | 0.258056533 |
| ACVR1 | 0.109535552 | 0.09413934 | 0.307260785 | NS | 1.026228853 |
| UPP2 | 0.423101575 | 0.264806309 | NA | NS | 0.577071672 |
| CCDC148 | 0.235032392 | 0.404925635 | 0.60860084 | NS | 0.392624728 |
| PKP4 | -0.261340063 | 2.93E-05 | 0.006982755 | Down | 4.533575355 |
| DAPL1 | 0.606008812 | 0.293673105 | NA | NS | 0.532135824 |
| TANC1 | 0.087148658 | 0.501165708 | 0.682824502 | NS | 0.300018653 |
| WDSUB1 | -0.010235912 | 0.888550708 | 0.937172484 | NS | 0.051317783 |
| BAZ2B | -0.010662678 | 0.901604142 | 0.94535931 | NS | 0.044984102 |
| MARCHF7 | -0.044882569 | 0.498295478 | 0.680143468 | NS | 0.302513054 |
| CD302 | 0.106524497 | 0.209031527 | 0.43899496 | NS | 0.679788206 |
| LY75 | -0.186044625 | 0.007824466 | 0.114722104 | Down | 2.106545277 |
| PLA2R1 | 0.438432597 | 0.278441903 | 0.502868581 | NS | 0.555265406 |
| ITGB6 | 0.39211987 | 0.401837585 | NA | NS | 0.395949445 |
| RBMS1 | 0.093751161 | 0.031918671 | 0.198125154 | NS | 1.4959552 |
| TANK | -0.02900807 | 0.581151652 | 0.740451328 | NS | 0.235710524 |
| PSMD14 | -0.134570844 | 0.101078223 | 0.316641506 | NS | 0.995342402 |
| TBR1 | 0.575329382 | 0.156497696 | 0.383636723 | NS | 0.805492051 |
| SLC4A10 | -0.314118008 | 0.090636429 | 0.30144576 | NS | 1.042697212 |
| DPP4 | -0.10193248 | 0.197629816 | 0.427410856 | NS | 0.704147533 |
| GCG | 0.627320267 | 0.282428476 | NA | NS | 0.549091517 |
| FAP | 0.417852115 | 0.191995658 | 0.420934764 | NS | 0.716708594 |
| IFIH1 | 0.049271561 | 0.588980056 | 0.746930686 | NS | 0.229899411 |
| GCA | 0.179298462 | 0.240563952 | 0.468406361 | NS | 0.618769451 |
| KCNH7 | 0.357541459 | 0.057585181 | 0.25144248 | NS | 1.239689266 |
| FIGN | 0.522422356 | 0.218357595 | 0.447821763 | NS | 0.660831698 |
| GRB14 | -0.048558484 | 0.800926278 | 0.885420215 | NS | 0.096407457 |
| COBLL1 | -0.187475494 | 0.110230263 | 0.328790965 | NS | 0.957699155 |
| SLC38A11 | 0.195026059 | 0.293415653 | 0.515424479 | NS | 0.532516722 |
| SCN3A | 0.038514309 | 0.797255334 | 0.883232725 | NS | 0.098402566 |
| SCN2A | 0.398960699 | 0.210608274 | 0.440477465 | NS | 0.676524572 |
| CSRNP3 | 0.352895028 | 0.366656819 | 0.578995688 | NS | 0.435740234 |
| GALNT3 | 0.025549549 | 0.761408672 | 0.860657491 | NS | 0.118382181 |
| TTC21B | 0.040884763 | 0.417712557 | 0.618692258 | NS | 0.379122469 |
| SCN1A | 0.458625998 | 0.300867323 | 0.522242188 | NS | 0.521624978 |
| SCN9A | 0.470681654 | 3.57E-04 | 0.032222893 | Up | 3.446875159 |
| SCN7A | 0.26608407 | 0.430471495 | 0.627698475 | NS | 0.366055601 |
| XIRP2 | 0.307429587 | 0.353118546 | 0.566682148 | NS | 0.452079473 |
| B3GALT1 | 0.580198309 | 0.142526352 | 0.36732856 | NS | 0.84610483 |
| STK39 | -0.051699816 | 0.431799991 | 0.628813708 | NS | 0.364717371 |
| CERS6 | -0.02313823 | 0.701488959 | 0.823069836 | NS | 0.15397916 |
| NOSTRIN | 0.366136271 | 0.359289668 | NA | NS | 0.444555272 |
| SPC25 | 0.070850292 | 0.854707509 | NA | NS | 0.06818248 |
| ABCB11 | 0.572410788 | 0.094711109 | NA | NS | 1.023599078 |
| DHRS9 | -0.020249264 | 0.897547087 | 0.942832005 | NS | 0.046942758 |
| LRP2 | 0.384845288 | 0.335106917 | 0.551502437 | NS | 0.474816608 |
| BBS5 | 0.421474693 | 0.055124215 | 0.247880381 | NS | 1.258657579 |
| KLHL41 | -0.16082911 | 0.387764358 | NA | NS | 0.411432112 |
| FASTKD1 | -0.196265106 | 0.010095076 | 0.126199476 | Down | 1.995890389 |
| PPIG | -0.119832364 | 0.079566553 | 0.284900136 | NS | 1.099269459 |
| CCDC173 | -0.056673864 | 0.543933239 | 0.713285597 | NS | 0.264454401 |
| PHOSPHO2 | 0.095287144 | 0.310353871 | 0.530362297 | NS | 0.508142833 |
| KLHL23 | -0.171055952 | 0.185799909 | 0.415463304 | NS | 0.730954504 |
| SSB | -0.212118794 | 0.045041921 | 0.226915537 | Down | 1.346383091 |
| METTL5 | -0.206804546 | 0.078065324 | 0.282523416 | NS | 1.107541834 |
| UBR3 | -0.020437061 | 0.550705586 | 0.718156208 | NS | 0.259080519 |
| MYO3B | 0.10550203 | 0.66320789 | 0.798317412 | NS | 0.178350316 |
| SP5 | 1.098097139 | 0.024370821 | NA | Up | 1.613129842 |
| ERICH2 | 0.682744903 | 0.163542937 | NA | NS | 0.786368207 |
| GAD1 | -0.255788406 | 0.465078818 | 0.655294755 | NS | 0.33247344 |
| GORASP2 | 0.097246962 | 0.047055976 | 0.230692912 | NS | 1.327385211 |
| TLK1 | 0.024651021 | 0.727454143 | 0.838753198 | NS | 0.138194379 |
| METTL8 | -0.04690623 | 0.487243179 | 0.672268351 | NS | 0.312254232 |
| DCAF17 | -0.051017506 | 0.42418501 | 0.623055741 | NS | 0.372444682 |
| CYBRD1 | -0.165088632 | 0.129778587 | 0.351821639 | NS | 0.886796958 |
| DYNC1I2 | 0.001518201 | 0.974682176 | 0.985403414 | NS | 0.011136976 |
| SLC25A12 | -0.051087689 | 0.217760642 | 0.447318524 | NS | 0.662020611 |
| HAT1 | -0.267993515 | 0.032183567 | 0.198223945 | Down | 1.492365817 |
| METAP1D | 0.131465378 | 0.205782239 | 0.435807734 | NS | 0.686592112 |
| DLX1 | 0.919639977 | 0.042234673 | NA | Up | 1.374330866 |
| DLX2 | 0.773810394 | 0.054047695 | NA | NS | 1.267222819 |
| ITGA6 | -0.093447184 | 0.255433639 | 0.482310264 | NS | 0.59272191 |
| PDK1 | 0.143772379 | 0.022567653 | 0.171443155 | Up | 1.646513597 |
| RAPGEF4 | 0.420241836 | 0.151718376 | 0.378214243 | NS | 0.818961814 |
| MAP3K20 | 0.01367288 | 0.890208368 | 0.93792961 | NS | 0.050508328 |
| CDCA7 | 0.111395264 | 0.29569056 | 0.517512114 | NS | 0.52916254 |
| SP3 | -0.005162799 | 0.930276593 | 0.961856154 | NS | 0.031387907 |
| OLA1 | -0.129408319 | 0.112766968 | 0.331356055 | NS | 0.947818097 |
| SP9 | 1.695135127 | 0.004504371 | NA | Up | 2.346365868 |
| CIR1 | 0.078138678 | 0.242781621 | 0.470460443 | NS | 0.614784193 |
| SCRN3 | 0.155532582 | 0.001667103 | 0.064977566 | Up | 2.77803744 |
| GPR155 | 0.185245178 | 0.004343713 | 0.093367359 | Up | 2.362138833 |
| WIPF1 | 0.154044603 | 0.035074109 | 0.205352924 | Up | 1.455013347 |
| CHRNA1 | 0.60379321 | 0.189222229 | NA | NS | 0.723027846 |
| CHN1 | 0.165486348 | 0.188825123 | 0.41822756 | NS | 0.723940223 |
| ATF2 | -0.097141328 | 0.072106098 | 0.273763446 | NS | 1.142028005 |
| ATP5MC3 | -0.19495262 | 0.075780094 | 0.279091018 | NS | 1.120444859 |
| LNPK | -0.211386506 | 0.063604336 | 0.262056846 | NS | 1.196513278 |
| EVX2 | 0.919814913 | 0.083783691 | NA | NS | 1.076840512 |
| HOXD13 | 0.256951424 | 0.601795128 | NA | NS | 0.220551333 |
| HOXD12 | 0.52984987 | 0.256507863 | NA | NS | 0.590899318 |
| HOXD11 | 1.168978442 | 0.052091591 | NA | NS | 1.283232375 |
| HOXD10 | 1.370167722 | 0.016090697 | NA | Up | 1.793425138 |
| HOXD9 | 0.631731882 | 0.220917177 | NA | NS | 0.655770515 |
| HOXD8 | 1.186939194 | 0.019683133 | NA | Up | 1.70590577 |
| HOXD3 | 1.014901739 | 0.044849105 | NA | Up | 1.348246217 |
| HOXD4 | 0.616393063 | 0.220420246 | NA | NS | 0.656748518 |
| HOXD1 | 1.421789684 | 0.06013301 | NA | NS | 1.220887058 |
| MTX2 | -0.080957727 | 0.310431691 | 0.530362297 | NS | 0.508033949 |
| HNRNPA3 | 0.154473634 | 0.002826724 | 0.079855432 | Up | 2.5487166 |
| NFE2L2 | -0.074231182 | 0.36537827 | 0.577693693 | NS | 0.437257285 |
| AGPS | 0.041864086 | 0.365363632 | 0.577693693 | NS | 0.437274684 |
| TTC30B | -0.016804332 | 0.831894446 | 0.90456968 | NS | 0.079931775 |
| TTC30A | -0.11031221 | 0.256626024 | 0.483565015 | NS | 0.590699305 |
| PDE11A | 0.572603151 | 0.158678554 | 0.386214013 | NS | 0.799481765 |
| RBM45 | -0.057765141 | 0.279481868 | 0.503278325 | NS | 0.553646363 |
| OSBPL6 | 0.304659469 | 0.279122909 | 0.503208948 | NS | 0.554204518 |
| PRKRA | 0.025946391 | 0.616966467 | 0.765799471 | NS | 0.20973844 |
| PJVK | 0.009588731 | 0.939286047 | 0.966553041 | NS | 0.027202129 |
| FKBP7 | 0.126178367 | 0.408600649 | 0.611677799 | NS | 0.388700948 |
| PLEKHA3 | 0.020447163 | 0.562693911 | 0.72691877 | NS | 0.249727784 |
| TTN | 0.086400273 | 0.341470128 | 0.55696544 | NS | 0.466647282 |
| CCDC141 | 0.060799561 | 0.547628496 | 0.7160589 | NS | 0.261513961 |
| SESTD1 | 0.201590025 | 0.004582322 | 0.094750689 | Up | 2.338914404 |
| ZNF385B | 0.470828339 | 0.143980712 | 0.369130894 | NS | 0.841695682 |
| CWC22 | -0.081508276 | 0.125770129 | 0.347453256 | NS | 0.900422494 |
| UBE2E3 | 0.017084486 | 0.839095309 | 0.909248203 | NS | 0.076188707 |
| ITGA4 | -0.041995812 | 0.441577718 | 0.636118562 | NS | 0.354992849 |
| CERKL | 0.086159394 | 0.32392602 | 0.542570549 | NS | 0.489554165 |
| NEUROD1 | 0.422042417 | 0.300138559 | 0.521612923 | NS | 0.522678207 |
| ITPRID2 | 0.007741313 | 0.909137575 | 0.949563038 | NS | 0.041370393 |
| PPP1R1C | 0.325722857 | 0.498892769 | 0.680705522 | NS | 0.301992791 |
| PDE1A | 0.424482686 | 0.261837168 | 0.488123307 | NS | 0.581968705 |
| DNAJC10 | -0.110410883 | 0.080291057 | 0.285933942 | NS | 1.095332822 |
| FRZB | 0.408176751 | 0.378697991 | NA | NS | 0.421706999 |
| NCKAP1 | -0.013647612 | 0.852104003 | 0.916579112 | NS | 0.069507395 |
| DUSP19 | -0.118139004 | 0.150028404 | 0.376171504 | NS | 0.82382651 |
| NUP35 | -0.142118615 | 0.123940832 | 0.344979695 | NS | 0.906785594 |
| ZNF804A | -0.089393405 | 0.646768843 | 0.786674813 | NS | 0.189250909 |
| FSIP2 | 0.192979844 | 0.15031459 | 0.376600453 | NS | 0.822998863 |
| ZC3H15 | -0.213305187 | 0.039425575 | 0.214828581 | Down | 1.40422196 |
| ITGAV | -0.075257773 | 0.264006767 | 0.490237234 | NS | 0.578384941 |
| FAM171B | 0.077643419 | 0.613709677 | 0.763762238 | NS | 0.212037029 |
| ZSWIM2 | 0.601713224 | 0.208578128 | NA | NS | 0.680731234 |
| CALCRL | -0.147941197 | 0.221196713 | 0.449857661 | NS | 0.655221331 |
| TFPI | -0.184232059 | 0.288168263 | 0.511321729 | NS | 0.540353852 |
| GULP1 | 0.454202217 | 0.254487963 | 0.48140404 | NS | 0.594332755 |
| COL3A1 | 0.503267937 | 0.280595541 | 0.503778541 | NS | 0.551919235 |
| COL5A2 | 0.213713249 | 0.252801811 | 0.47963442 | NS | 0.597219818 |
| WDR75 | -0.035530983 | 0.491798604 | 0.675891158 | NS | 0.308212708 |
| SLC40A1 | 0.102600905 | 0.279024962 | 0.503208948 | NS | 0.554356942 |
| ASNSD1 | -0.152436012 | 0.120086011 | 0.340734879 | NS | 0.920507581 |
| ANKAR | 0.027268204 | 0.646283007 | 0.786334454 | NS | 0.189577263 |
| OSGEPL1 | 0.011360994 | 0.844099231 | 0.912554786 | NS | 0.073606495 |
| ORMDL1 | -0.047471153 | 0.40665891 | 0.610228172 | NS | 0.390769708 |
| PMS1 | -0.096429842 | 0.095026692 | 0.308476443 | NS | 1.022154388 |
| C2orf88 | -0.103928304 | 0.27128014 | 0.496530841 | NS | 0.566581999 |
| MSTN | 0.604965587 | 0.076228279 | NA | NS | 1.117883882 |
| HIBCH | 0.040412269 | 0.386616059 | 0.594646138 | NS | 0.41272011 |
| INPP1 | 0.134476384 | 0.072145267 | 0.273763446 | NS | 1.141792156 |
| MFSD6 | -3.80E-04 | 0.99338433 | 0.996124521 | NS | 0.002882695 |
| NEMP2 | 0.030269464 | 0.637001124 | 0.779022011 | NS | 0.195859801 |
| NAB1 | -0.068581164 | 0.31154844 | 0.531239846 | NS | 0.506474419 |
| GLS | -0.046009652 | 0.422998459 | 0.622430654 | NS | 0.373661215 |
| STAT1 | 0.070409873 | 0.498302648 | 0.680143468 | NS | 0.302506805 |
| STAT4 | -0.147227738 | 0.025123606 | 0.179190542 | Down | 1.599918023 |
| MYO1B | -0.01031823 | 0.947683299 | 0.97158694 | NS | 0.023336773 |
| NABP1 | 0.253294983 | 0.045930657 | 0.228673646 | Up | 1.337897343 |
| CAVIN2 | -0.084609363 | 0.514610444 | 0.692659911 | NS | 0.288521404 |
| TMEFF2 | 0.114479877 | 0.788878553 | 0.878312913 | NS | 0.102989851 |
| SLC39A10 | -0.037459772 | 0.557637389 | 0.72344709 | NS | 0.253648115 |
| DNAH7 | 0.221785552 | 0.146245473 | 0.371713762 | NS | 0.834917569 |
| STK17B | -0.129890399 | 0.106055527 | 0.322346872 | NS | 0.974466693 |
| HECW2 | 0.196792812 | 0.218181146 | 0.447660331 | NS | 0.661182781 |
| CCDC150 | 0.140587433 | 0.494209551 | 0.677463766 | NS | 0.306088866 |
| GTF3C3 | -0.081345039 | 0.122303593 | 0.34308586 | NS | 0.912560782 |
| C2orf66 | 0.621832674 | 0.122717549 | NA | NS | 0.911093328 |
| PGAP1 | 0.184192706 | 0.019809046 | 0.163424633 | Up | 1.703136431 |
| ANKRD44 | 0.022518683 | 0.665020813 | 0.799449198 | NS | 0.177164762 |
| SF3B1 | -0.086471818 | 0.152454807 | 0.37908978 | NS | 0.816858877 |
| COQ10B | -0.053918626 | 0.437803939 | 0.633391396 | NS | 0.358720336 |
| HSPD1 | 0.009656521 | 0.862702279 | 0.922649089 | NS | 0.064139055 |
| HSPE1 | -0.140056144 | 0.327344044 | 0.545360849 | NS | 0.484995557 |
| MOB4 | -0.124251389 | 0.144333283 | 0.36967266 | NS | 0.840633509 |
| RFTN2 | 0.231668854 | 0.038410845 | 0.213464789 | Up | 1.415546136 |
| MARS2 | -0.130999751 | 0.070263388 | 0.271077991 | NS | 1.153270911 |
| BOLL | 0.247739075 | 0.282393208 | 0.5053027 | NS | 0.549145753 |
| PLCL1 | 0.088498001 | 0.267569997 | 0.493587441 | NS | 0.572562586 |
| SATB2 | 0.370075754 | 0.116978952 | 0.336807435 | NS | 0.931892274 |
| FTCDNL1 | 0.172928577 | 0.048532571 | 0.233703312 | Up | 1.313966697 |
| C2orf69 | -0.080828724 | 0.259798912 | 0.485843002 | NS | 0.585362672 |
| TYW5 | 0.059453975 | 0.238995239 | 0.467218862 | NS | 0.621610751 |
| MAIP1 | -0.011672433 | 0.868757772 | 0.925923112 | NS | 0.061101297 |
| SPATS2L | 0.201515052 | 0.109642832 | 0.327854831 | NS | 0.960019754 |
| KCTD18 | -0.124346805 | 0.021870345 | 0.169347779 | Down | 1.66014436 |
| SGO2 | -0.285631408 | 0.023167913 | 0.173122445 | Down | 1.635113095 |
| AOX1 | 0.29977538 | 0.444198361 | 0.6383679 | NS | 0.352423049 |
| BZW1 | -0.184289098 | 0.033797906 | 0.20229026 | Down | 1.471110209 |
| CLK1 | -0.056128698 | 0.411338451 | 0.614050321 | NS | 0.385800692 |
| PPIL3 | -0.218601915 | 0.063258065 | 0.261404175 | NS | 1.198884097 |
| NIF3L1 | -0.079162623 | 0.238529692 | 0.466667264 | NS | 0.622457553 |
| ORC2 | -0.051976112 | 0.199208779 | 0.428926322 | NS | 0.700691526 |
| FAM126B | 0.204198445 | 0.078588907 | 0.283350622 | NS | 1.104638751 |
| NDUFB3 | -0.07843892 | 0.636309803 | 0.778584555 | NS | 0.196331386 |
| CFLAR | 0.076282606 | 0.315988932 | 0.535462709 | NS | 0.500328129 |
| CASP10 | 0.022173276 | 0.608709992 | 0.760022153 | NS | 0.215589569 |
| CASP8 | -0.061785316 | 0.29278472 | 0.515290877 | NS | 0.533451592 |
| FLACC1 | -0.051493011 | 0.715055885 | 0.831406299 | NS | 0.145660015 |
| TRAK2 | -0.137363413 | 0.348364807 | 0.562612118 | NS | 0.457965725 |
| STRADB | -0.384490135 | 0.042352931 | 0.220932874 | Down | 1.373116528 |
| C2CD6 | 0.194273743 | 0.218902267 | 0.448376685 | NS | 0.659749742 |
| TMEM237 | -0.006129863 | 0.943792113 | 0.969292462 | NS | 0.025123656 |
| MPP4 | 0.483297825 | 0.147563659 | 0.37334323 | NS | 0.831020585 |
| ALS2 | 0.02975975 | 0.590314153 | 0.747720896 | NS | 0.228916804 |
| CDK15 | 0.256018412 | 0.326627748 | 0.54485998 | NS | 0.485946924 |
| FZD7 | 0.720094403 | 0.015661939 | NA | Up | 1.805154483 |
| KIAA2012 | 0.398715119 | 0.11216104 | 0.330787614 | NS | 0.950157974 |
| SUMO1 | -0.21197673 | 0.028033366 | 0.187577424 | Down | 1.552324758 |
| NOP58 | -0.163044217 | 0.028706378 | 0.189026296 | Down | 1.542021607 |
| BMPR2 | 0.134625064 | 0.030487265 | 0.193861542 | Up | 1.515881529 |
| FAM117B | 0.056044923 | 0.378090861 | 0.588050044 | NS | 0.42240382 |
| ICA1L | 0.144191711 | 0.075926122 | 0.279334948 | NS | 1.11960878 |
| WDR12 | -0.068770354 | 0.25725957 | 0.484200085 | NS | 0.589628461 |
| CARF | -0.008037193 | 0.910024164 | 0.950185712 | NS | 0.040947076 |
| NBEAL1 | -0.006988502 | 0.868684897 | 0.925923112 | NS | 0.061137729 |
| CYP20A1 | -0.128088714 | 0.003162707 | 0.084307443 | Down | 2.499940999 |
| ABI2 | 0.106612948 | 0.107677312 | 0.32500608 | NS | 0.967875796 |
| RAPH1 | 0.049792024 | 0.678008314 | 0.807899517 | NS | 0.16876498 |
| CD28 | -0.043881859 | 0.599944334 | 0.75439633 | NS | 0.221889044 |
| CTLA4 | -0.001540059 | 0.982896916 | 0.989855554 | NS | 0.007492028 |
| ICOS | -0.134024825 | 0.169371869 | 0.397719116 | NS | 0.771158721 |
| PARD3B | 0.366074532 | 0.113719672 | 0.332423088 | NS | 0.944164403 |
| NRP2 | 0.32466288 | 0.159267706 | 0.386966794 | NS | 0.797872276 |
| INO80D | -0.063914416 | 0.461788774 | 0.652711628 | NS | 0.335556628 |
| NDUFS1 | -0.214021408 | 1.70E-04 | 0.021195909 | Down | 3.768803542 |
| EEF1B2 | -0.340394719 | 0.133565556 | 0.356765072 | NS | 0.874305522 |
| ZDBF2 | -0.157182197 | 0.266476539 | 0.492363509 | NS | 0.574341021 |
| ADAM23 | 0.409154468 | 0.001817935 | 0.066397006 | Up | 2.740421592 |
| FAM237A | 0.617063471 | 0.264918876 | NA | NS | 0.576887096 |
| DYTN | 0.086575638 | 0.696516704 | 0.820148803 | NS | 0.157068464 |
| MDH1B | 0.601197964 | 0.127432391 | NA | NS | 0.894720167 |
| FASTKD2 | -0.08974127 | 0.130855369 | 0.353027407 | NS | 0.883208454 |
| CPO | 0.229082464 | 0.517043593 | NA | NS | 0.286472839 |
| KLF7 | 0.163606735 | 0.060973721 | 0.256832544 | NS | 1.214857302 |
| CREB1 | -0.015970711 | 0.722386677 | 0.836151808 | NS | 0.141230272 |
| METTL21A | -0.024203548 | 0.63387108 | 0.776736637 | NS | 0.197999062 |
| CCNYL1 | -0.101618285 | 0.356253364 | 0.569279552 | NS | 0.448241026 |
| FZD5 | 0.061583536 | 0.679896796 | 0.809261287 | NS | 0.167557006 |
| PLEKHM3 | 0.097251047 | 0.122565887 | 0.343398823 | NS | 0.911630389 |
| CRYGB | 0.123692691 | 0.837994997 | NA | NS | 0.076758574 |
| CRYGA | 0.744380529 | 0.298501624 | NA | NS | 0.525053302 |
| C2orf80 | 0.508496407 | 0.309404983 | NA | NS | 0.509472697 |
| IDH1 | -0.073507395 | 0.384421915 | 0.593103658 | NS | 0.415191862 |
| PIKFYVE | 0.015001463 | 0.727933989 | 0.839053008 | NS | 0.137908002 |
| PTH2R | -0.00965799 | 0.957100973 | 0.976414383 | NS | 0.019042242 |
| MAP2 | 0.233327389 | 0.304715329 | 0.525971545 | NS | 0.516105698 |
| UNC80 | 0.341203331 | 0.347780782 | 0.562255787 | NS | 0.45869442 |
| RPE | -0.035644491 | 0.443706907 | 0.637917821 | NS | 0.352903811 |
| KANSL1L | 0.021353804 | 0.665288558 | 0.799541399 | NS | 0.176989946 |
| ACADL | 0.54787972 | 0.164321694 | 0.392074272 | NS | 0.784305096 |
| MYL1 | 0.784873651 | 0.137040017 | NA | NS | 0.863152596 |
| LANCL1 | -0.025722594 | 0.65958149 | 0.795335085 | NS | 0.18073154 |
| CPS1 | 0.289298216 | 0.287740105 | 0.510878664 | NS | 0.540999602 |
| ERBB4 | 0.400771157 | 0.358268535 | 0.571412376 | NS | 0.445791333 |
| IKZF2 | 0.03341012 | 0.745882051 | 0.850664346 | NS | 0.127329843 |
| SPAG16 | -0.067099601 | 0.543454427 | 0.713114152 | NS | 0.264836869 |
| VWC2L | 0.233051086 | 0.313785761 | 0.533347298 | NS | 0.503366768 |
| BARD1 | -0.126470478 | 0.197085248 | 0.426862716 | NS | 0.705345881 |
| ABCA12 | 0.125862251 | 0.69388811 | 0.818259898 | NS | 0.158710554 |
| ATIC | 0.078798372 | 0.279812817 | 0.503478405 | NS | 0.553132396 |
| FN1 | 0.203742269 | 0.118748069 | 0.339297335 | NS | 0.925373444 |
| MREG | -0.069641583 | 0.313662932 | 0.533319352 | NS | 0.503536803 |
| PECR | 0.011130533 | 0.821453528 | 0.898334863 | NS | 0.085417001 |
| TMEM169 | 0.044758045 | 0.609055166 | 0.760306411 | NS | 0.215343369 |
| XRCC5 | -0.150154293 | 0.045969704 | 0.228716549 | Down | 1.337528296 |
| MARCHF4 | 0.928408763 | 0.044861344 | 0.226545246 | Up | 1.348127715 |
| SMARCAL1 | 0.030162071 | 0.44579406 | 0.63977789 | NS | 0.350865723 |
| RPL37A | -0.300535861 | 0.006770185 | 0.109499136 | Down | 2.169399464 |
| IGFBP2 | 0.074298445 | 0.801442159 | 0.885670156 | NS | 0.096127816 |
| IGFBP5 | 0.34563993 | 0.415276284 | 0.616572671 | NS | 0.38166287 |
| TNP1 | 0.197099385 | 0.827590965 | NA | NS | 0.082184259 |
| TNS1 | -0.49986252 | 0.042102696 | 0.220751111 | Down | 1.375690097 |
| RUFY4 | 0.268011795 | 0.178364187 | 0.408326331 | NS | 0.748692341 |
| CXCR2 | 0.282920203 | 0.095381142 | 0.309058959 | NS | 1.020537484 |
| CXCR1 | -0.010593835 | 0.953260132 | 0.974797443 | NS | 0.02078857 |
| ARPC2 | -0.071686326 | 0.333290341 | 0.550016309 | NS | 0.477177272 |
| GPBAR1 | 0.047209822 | 0.62963545 | 0.774589928 | NS | 0.200910828 |
| AAMP | -0.325840814 | 2.87E-04 | 0.028091276 | Down | 3.542086085 |
| PNKD | 0.048036034 | 0.573377795 | 0.734910168 | NS | 0.24155913 |
| TMBIM1 | -0.107597119 | 0.094295788 | 0.30742606 | NS | 1.025507706 |
| CATIP | 0.116404223 | 0.468534079 | 0.657981817 | NS | 0.329258815 |
| SLC11A1 | 0.010493979 | 0.930684344 | 0.962064222 | NS | 0.031197592 |
| CTDSP1 | 0.206615693 | 0.010201759 | 0.126233693 | Up | 1.991324958 |
| VIL1 | -0.026730011 | 0.860156383 | 0.921008463 | NS | 0.065422584 |
| USP37 | -0.174229156 | 0.021862468 | 0.169347779 | Down | 1.660300811 |
| CNOT9 | 0.005408164 | 0.884994356 | 0.93507754 | NS | 0.053059499 |
| PLCD4 | 0.648644371 | 0.036528578 | 0.208452907 | Up | 1.437367238 |
| ZNF142 | 0.153705493 | 0.180151522 | 0.410020841 | NS | 0.744362065 |
| BCS1L | -0.166319011 | 0.005570563 | 0.101332034 | Down | 2.254100919 |
| RNF25 | -0.227822508 | 0.001001812 | 0.052382393 | Down | 2.999213962 |
| STK36 | -0.084287315 | 0.363641669 | 0.575822548 | NS | 0.439326357 |
| TTLL4 | 0.11184113 | 0.258073403 | 0.484816428 | NS | 0.588256752 |
| CYP27A1 | -0.15922877 | 0.213099439 | 0.442932136 | NS | 0.671417693 |
| PRKAG3 | 0.335164727 | 0.125309139 | 0.346840099 | NS | 0.902017255 |
| WNT6 | 0.937307206 | 0.005422433 | NA | Up | 2.265805809 |
| WNT10A | 0.062411163 | 0.676212362 | 0.806788244 | NS | 0.169916894 |
| CDK5R2 | 0.837606765 | 0.079211809 | NA | NS | 1.101210066 |
| FEV | 1.376562898 | 0.0261884 | NA | Up | 1.581891042 |
| CRYBA2 | 0.811011403 | 0.1572961 | NA | NS | 0.803282044 |
| CFAP65 | 0.52707821 | 0.217296313 | 0.446955397 | NS | 0.662947643 |
| IHH | 1.158006145 | 0.011547121 | NA | Up | 1.937526292 |
| NHEJ1 | 0.164577171 | 0.083766117 | 0.291497295 | NS | 1.076931614 |
| SLC23A3 | 0.310970667 | 0.053493532 | 0.24433763 | NS | 1.271698723 |
| CNPPD1 | -0.104247248 | 0.522526442 | 0.698185564 | NS | 0.281891728 |
| RETREG2 | -0.010334739 | 0.921757356 | 0.956852823 | NS | 0.035383388 |
| ZFAND2B | 0.018838447 | 0.696918719 | 0.820297213 | NS | 0.15681787 |
| ABCB6 | 0.061323807 | 0.674313223 | 0.805989661 | NS | 0.171138324 |
| ATG9A | 0.110588387 | 0.416946693 | 0.617989235 | NS | 0.379919466 |
| ANKZF1 | -0.163812784 | 0.013563386 | 0.13992282 | Down | 1.867631886 |
| GLB1L | 0.163090085 | 0.113358989 | 0.332115589 | NS | 0.945544037 |
| STK16 | -0.049714384 | 0.407711424 | 0.611085087 | NS | 0.38964712 |
| TUBA4A | -0.092918669 | 0.3555886 | 0.568772881 | NS | 0.449052171 |
| TUBA4B | 0.004142105 | 0.979477587 | 0.987845504 | NS | 0.009005497 |
| DNAJB2 | -0.334689795 | 0.026322669 | 0.183162888 | Down | 1.579670073 |
| PTPRN | 0.248686263 | 0.451608312 | 0.644366928 | NS | 0.345238073 |
| RESP18 | 0.800337018 | 0.142782457 | NA | NS | 0.84532515 |
| DNPEP | -0.213122191 | 3.96E-04 | 0.034192468 | Down | 3.402697092 |
| DES | 0.526777545 | 0.066023047 | NA | NS | 1.180304438 |
| SPEG | 0.530855722 | 0.055717058 | 0.248760687 | NS | 1.254011822 |
| GMPPA | 0.128094776 | 0.120708344 | 0.341313216 | NS | 0.918262707 |
| ASIC4 | 0.790280272 | 0.05710254 | 0.250603546 | NS | 1.243344575 |
| CHPF | 0.550079761 | 5.45E-04 | 0.040010004 | Up | 3.263550226 |
| TMEM198 | 0.719154183 | 0.046373604 | NA | Up | 1.333729148 |
| OBSL1 | 0.684942393 | 0.040395614 | 0.216812832 | Up | 1.393665786 |
| INHA | 0.378106029 | 0.461688388 | NA | NS | 0.335651048 |
| STK11IP | 0.131778553 | 0.213919672 | 0.443234232 | NS | 0.669749275 |
| SLC4A3 | 0.560866168 | 0.061958778 | 0.258654747 | NS | 1.207897154 |
| EPHA4 | -0.083312143 | 0.331983121 | 0.548813376 | NS | 0.478883996 |
| PAX3 | 0.414177096 | 0.376970738 | 0.587104103 | NS | 0.42369236 |
| SGPP2 | 0.263579147 | 0.005196248 | 0.098827133 | Up | 2.284310153 |
| FARSB | -0.068514817 | 0.297613283 | 0.519257109 | NS | 0.52634769 |
| MOGAT1 | -0.070854701 | 0.846580384 | NA | NS | 0.072331799 |
| ACSL3 | -0.044575736 | 0.42260837 | 0.622069963 | NS | 0.374061905 |
| KCNE4 | 0.90327392 | 0.062123767 | NA | NS | 1.206742218 |
| SCG2 | 0.132891714 | 0.764964259 | NA | NS | 0.116358855 |
| AP1S3 | 0.303084679 | 0.060651188 | 0.256526216 | NS | 1.217160691 |
| WDFY1 | -0.09113377 | 0.250375955 | 0.477425471 | NS | 0.60140738 |
| MRPL44 | -0.075648909 | 0.398462261 | 0.603464006 | NS | 0.399612805 |
| SERPINE2 | 0.086624136 | 0.569005671 | 0.731688258 | NS | 0.244883406 |
| FAM124B | 0.004526267 | 0.970653117 | 0.983371222 | NS | 0.012935946 |
| CUL3 | -0.030444879 | 0.441124035 | 0.635784938 | NS | 0.355439278 |
| CCDC195 | 0.026822274 | 0.962837056 | NA | NS | 0.016447204 |
| DOCK10 | -0.011704704 | 0.836316098 | 0.907955026 | NS | 0.077629544 |
| NYAP2 | 0.121146617 | 0.744152732 | 0.850016886 | NS | 0.12833792 |
| IRS1 | 0.138589582 | 0.349915555 | 0.564031957 | NS | 0.456036751 |
| RHBDD1 | -0.09019823 | 0.453510203 | 0.646034128 | NS | 0.343412938 |
| COL4A4 | 0.079140378 | 0.409658687 | 0.612802303 | NS | 0.387577831 |
| COL4A3 | -0.01835014 | 0.889435558 | 0.93753639 | NS | 0.050885512 |
| MFF | -0.01626815 | 0.729626695 | 0.840327411 | NS | 0.136899285 |
| TM4SF20 | 0.353639299 | 0.020736908 | 0.167165321 | Up | 1.683255991 |
| AGFG1 | 0.120876174 | 0.030589431 | 0.19406635 | Up | 1.514428605 |
| SCYGR4 | 0.53192361 | 0.436403502 | NA | NS | 0.360111773 |
| SCYGR5 | 0.43904588 | 0.644697444 | NA | NS | 0.190644052 |
| SLC19A3 | 0.29907222 | 0.534722151 | 0.707055814 | NS | 0.271871825 |
| SCYGR8 | 0.896349276 | 0.333507581 | NA | NS | 0.47689429 |
| CCL20 | -0.133273452 | 0.697772663 | NA | NS | 0.156286049 |
| DAW1 | 0.862454718 | 0.052113014 | NA | NS | 1.283053808 |
| SPHKAP | 0.356670607 | 0.427469203 | 0.625472763 | NS | 0.369095169 |
| PID1 | -0.057659273 | 0.617607386 | 0.766154761 | NS | 0.209287519 |
| DNER | 0.243818915 | 0.481977842 | NA | NS | 0.316972927 |
| TRIP12 | -0.001449976 | 0.983433453 | 0.990134653 | NS | 0.007255022 |
| FBXO36 | 0.312948812 | 0.01961941 | 0.162621692 | Up | 1.707314051 |
| SLC16A14 | 0.428863214 | 0.014929207 | 0.14642771 | Up | 1.825963256 |
| SP110 | 0.299872157 | 4.32E-04 | 0.03524659 | Up | 3.364049987 |
| SP140 | -0.05263819 | 0.353815169 | 0.567005458 | NS | 0.451223551 |
| SP140L | -0.024547085 | 0.644893576 | 0.785410134 | NS | 0.190511949 |
| SP100 | 0.019316082 | 0.67588187 | 0.806704534 | NS | 0.170129203 |
| CAB39 | -0.059698561 | 0.424386405 | 0.623190546 | NS | 0.372238537 |
| ITM2C | 0.116811583 | 0.290414561 | 0.513358619 | NS | 0.536981613 |
| GPR55 | -0.085069316 | 0.341382647 | 0.55696544 | NS | 0.466758558 |
| SPATA3 | 0.58563199 | 0.180265979 | NA | NS | 0.744086228 |
| C2orf72 | 0.320198573 | 0.545424586 | NA | NS | 0.263265289 |
| PSMD1 | -0.129185492 | 0.001979502 | 0.069745459 | Down | 2.703444119 |
| HTR2B | 0.174051142 | 0.099291906 | 0.314301595 | NS | 1.003086151 |
| ARMC9 | 0.546461168 | 0.059217796 | 0.253884625 | NS | 1.227547759 |
| B3GNT7 | 0.353497659 | 0.039803409 | 0.215730166 | Up | 1.400079729 |
| NCL | -0.169367471 | 0.005661481 | 0.101933358 | Down | 2.247069969 |
| NMUR1 | 0.143668782 | 0.188076671 | 0.417538962 | NS | 0.72566507 |
| TEX44 | 1.183569188 | 0.041177104 | NA | Up | 1.385344204 |
| PTMA | -0.230398129 | 0.014111514 | 0.14237088 | Down | 1.850426383 |
| PDE6D | -0.100146024 | 0.072196031 | 0.273837943 | NS | 1.141486678 |
| COPS7B | 0.028588394 | 0.726597371 | 0.838611255 | NS | 0.138706178 |
| NPPC | 1.558907714 | 0.004088007 | NA | Up | 2.388488381 |
| DIS3L2 | 0.019672794 | 0.748841355 | 0.8524263 | NS | 0.125610179 |
| ALPP | 0.799557659 | 0.162740511 | NA | NS | 0.788504325 |
| ALPG | 1.257736551 | 0.012986257 | NA | Up | 1.886516001 |
| ALPI | 0.643552225 | 0.193033272 | NA | NS | 0.714367827 |
| ECEL1 | 1.434746064 | 0.002204978 | NA | Up | 2.656595739 |
| PRSS56 | 1.036656692 | 0.061796774 | NA | NS | 1.209034194 |
| CHRND | 0.166550873 | 0.449991517 | 0.643342638 | NS | 0.346795673 |
| CHRNG | 0.119094065 | 0.303557162 | 0.524780583 | NS | 0.517759516 |
| TIGD1 | -0.034000973 | 0.623934575 | 0.770656501 | NS | 0.204860948 |
| EIF4E2 | -0.111977938 | 0.017018316 | 0.153426713 | Down | 1.769083422 |
| EFHD1 | 0.580027008 | 0.116908392 | NA | NS | 0.932154313 |
| GIGYF2 | -0.06223627 | 0.158410388 | 0.385866275 | NS | 0.800216341 |
| KCNJ13 | 0.111594048 | 0.206032713 | 0.43596473 | NS | 0.686063818 |
| SNORC | 0.354743675 | 0.050434133 | 0.237663067 | NS | 1.297275444 |
| NGEF | 0.849852372 | 0.018785977 | 0.159116357 | Up | 1.72616622 |
| NEU2 | 0.564969218 | 0.367877604 | NA | NS | 0.434296651 |
| INPP5D | 0.014838168 | 0.871231055 | 0.927222822 | NS | 0.059866652 |
| ATG16L1 | -0.199175237 | 7.46E-05 | 0.013247124 | Down | 4.127162114 |
| DGKD | 0.051318289 | 0.549439103 | 0.717263403 | NS | 0.260080436 |
| USP40 | 0.087281049 | 0.105286165 | 0.32124631 | NS | 0.977628693 |
| UGT1A8 | 0.365860335 | 0.516221885 | NA | NS | 0.287163587 |
| UGT1A10 | 0.414407317 | 0.42959985 | NA | NS | 0.366935879 |
| UGT1A9 | -0.15746827 | 0.79562836 | NA | NS | 0.099289745 |
| UGT1A7 | 0.768578102 | 0.165263277 | NA | NS | 0.78182364 |
| UGT1A6 | 0.666296446 | 0.212362463 | NA | NS | 0.672922247 |
| UGT1A5 | 0.759419461 | 0.191898153 | NA | NS | 0.716929204 |
| UGT1A4 | 0.544199715 | 0.349161041 | NA | NS | 0.456974221 |
| UGT1A3 | 0.831506824 | 0.326397573 | NA | NS | 0.486253079 |
| UGT1A1 | 0.857989041 | 0.132035724 | NA | NS | 0.879308548 |
| MROH2A | 0.552505701 | 0.228014515 | 0.456786951 | NS | 0.642037505 |
| HJURP | 0.227532167 | 0.190788312 | 0.420152441 | NS | 0.719448235 |
| TRPM8 | 0.401689479 | 0.333548402 | 0.550147567 | NS | 0.476841136 |
| SPP2 | 0.788169924 | 0.124320715 | NA | NS | 0.9054565 |
| ARL4C | -0.315763012 | 4.32E-04 | 0.03524659 | Down | 3.364891828 |
| SH3BP4 | 0.014604293 | 0.931527928 | 0.962676894 | NS | 0.03080412 |
| AGAP1 | -0.069160206 | 0.617763548 | 0.766154761 | NS | 0.209177722 |
| GBX2 | 0.764320981 | 0.228999535 | NA | NS | 0.640165399 |
| ASB18 | 0.3743405 | 0.391938045 | NA | NS | 0.406782578 |
| IQCA1 | 0.481246554 | 0.138031968 | 0.362607036 | NS | 0.860020318 |
| ACKR3 | -0.222870703 | 0.022048089 | 0.170172208 | Down | 1.656629052 |
| COPS8 | -0.101709237 | 0.129338021 | 0.351284745 | NS | 0.888273788 |
| COL6A3 | 0.156269787 | 0.335403535 | 0.551701439 | NS | 0.474432364 |
| MLPH | 0.722971606 | 0.082827266 | 0.290136134 | NS | 1.081826674 |
| PRLH | 1.168321314 | 0.059052099 | NA | NS | 1.228764658 |
| RAB17 | 0.63764167 | 0.19507851 | NA | NS | 0.709790571 |
| LRRFIP1 | 0.080552847 | 0.16345129 | 0.391129522 | NS | 0.786611648 |
| RBM44 | 0.099901306 | 0.252195349 | 0.47907912 | NS | 0.598262927 |
| RAMP1 | 0.460906712 | 0.03960713 | 0.215363569 | Up | 1.402226626 |
| UBE2F | -0.186985756 | 0.048567156 | 0.233728157 | Down | 1.313657327 |
| UBE2F-SCLY | 0.255910925 | 0.190498165 | NA | NS | 0.720109203 |
| SCLY | 0.164567389 | 0.138948065 | 0.363222438 | NS | 0.857147495 |
| ESPNL | 0.835382085 | 0.025094822 | 0.179152549 | Up | 1.600415886 |
| KLHL30 | 1.037944493 | 7.65E-04 | 0.045627194 | Up | 3.116318421 |
| ERFE | 0.382737817 | 0.014499596 | 0.144329895 | Up | 1.838644107 |
| ILKAP | -0.076874692 | 0.431902013 | 0.628863377 | NS | 0.364614771 |
| HES6 | 0.311032842 | 0.062810993 | 0.260739053 | NS | 1.201964342 |
| PER2 | 0.086210906 | 0.375799418 | 0.586170007 | NS | 0.425043896 |
| TRAF3IP1 | -0.084420922 | 0.256129499 | 0.483082784 | NS | 0.5915404 |
| ASB1 | -0.041901709 | 0.360486657 | 0.573284175 | NS | 0.443110806 |
| HDAC4 | 0.151949157 | 0.09170353 | 0.303537202 | NS | 1.037613947 |
| NDUFA10 | -0.047380206 | 0.183071512 | 0.413359339 | NS | 0.737379231 |
| OR6B2 | 0.499668508 | 0.509620864 | NA | NS | 0.2927528 |
| OR6B3 | 0.207590203 | 0.758579163 | NA | NS | 0.119999091 |
| COPS9 | -0.310099476 | 0.017201858 | 0.154054413 | Down | 1.764424646 |
| OTOS | 1.09268725 | 0.139600161 | NA | NS | 0.855114082 |
| GPC1 | 0.431227052 | 0.002330671 | 0.074444948 | Up | 2.632519023 |
| ANKMY1 | 0.00835361 | 0.905774664 | 0.947779517 | NS | 0.042979831 |
| DUSP28 | 0.102953561 | 0.13645704 | 0.360995625 | NS | 0.865004054 |
| RNPEPL1 | 0.231671456 | 0.027894729 | 0.187070304 | Up | 1.554477851 |
| CAPN10 | -0.098350776 | 0.10864565 | 0.326644764 | NS | 0.963987656 |
| GPR35 | 0.083672372 | 0.419093808 | 0.619391222 | NS | 0.377688756 |
| AQP12B | 1.378207127 | 0.010497991 | NA | Up | 1.978893787 |
| AQP12A | 1.417066009 | 0.043175673 | NA | Up | 1.364760883 |
| KIF1A | 0.829589976 | 0.038936128 | 0.214288889 | Up | 1.409647234 |
| AGXT | 0.743045877 | 0.072994997 | 0.274846078 | NS | 1.136706903 |
| MAB21L4 | 0.561315689 | 0.25402948 | NA | NS | 0.595115882 |
| CROCC2 | 0.469743022 | 0.08367101 | 0.291442354 | NS | 1.077424987 |
| SNED1 | 0.111104381 | 0.233149169 | 0.461424095 | NS | 0.632366128 |
| MTERF4 | 0.035370082 | 0.55537325 | 0.721922646 | NS | 0.255415043 |
| PASK | -0.007247878 | 0.938517419 | 0.966130894 | NS | 0.027557662 |
| PPP1R7 | -0.285815089 | 6.98E-05 | 0.012686464 | Down | 4.156162288 |
| ANO7 | 0.366843673 | 0.144058467 | 0.369286484 | NS | 0.841461212 |
| HDLBP | -0.229136948 | 0.011570311 | 0.13202953 | Down | 1.93665496 |
| SEPTIN2 | -0.110204444 | 0.04956215 | 0.235541056 | Down | 1.304849865 |
| FARP2 | 0.07820988 | 0.097940214 | 0.312744522 | NS | 1.009038953 |
| STK25 | -0.009568363 | 0.875872289 | 0.930331849 | NS | 0.057559214 |
| BOK | 0.402084544 | 0.039440169 | 0.21485698 | Up | 1.404061237 |
| THAP4 | -0.105394467 | 0.132170711 | 0.355111874 | NS | 0.878864775 |
| ATG4B | -0.073177959 | 0.250084266 | 0.477107181 | NS | 0.601913632 |
| DTYMK | -0.011379387 | 0.870460431 | 0.92688994 | NS | 0.060250966 |
| ING5 | -0.134544628 | 0.015191692 | 0.147378943 | Down | 1.818393848 |
| D2HGDH | 0.08531001 | 0.520061416 | 0.696688569 | NS | 0.283945366 |
| PDCD1 | 0.299276844 | 0.161019569 | NA | NS | 0.793121339 |
| CHL1 | 0.163423204 | 0.435110748 | 0.631046553 | NS | 0.361400189 |
| CNTN6 | 0.425323528 | 0.364718851 | 0.577049473 | NS | 0.438041789 |
| CNTN4 | 0.682403492 | 0.015406653 | 0.147618407 | Up | 1.812291699 |
| IL5RA | 0.440134544 | 0.025873012 | 0.182170348 | Up | 1.58715301 |
| TRNT1 | -0.030641064 | 0.566145103 | 0.729939888 | NS | 0.247072245 |
| CRBN | -0.088982643 | 0.146244467 | 0.371713762 | NS | 0.834920555 |
| SUMF1 | -0.03461118 | 0.545489056 | 0.714200227 | NS | 0.263213958 |
| LRRN1 | 0.475905257 | 8.05E-04 | 0.047377025 | Up | 3.094356021 |
| SETMAR | 0.133965023 | 0.043836206 | 0.224412931 | Up | 1.358167038 |
| ITPR1 | 0.01243897 | 0.843511812 | 0.912135017 | NS | 0.073908831 |
| BHLHE40 | 0.072251723 | 0.337696521 | 0.553768131 | NS | 0.471473414 |
| ARL8B | -0.100218112 | 0.102220156 | 0.318112331 | NS | 0.990463459 |
| EDEM1 | 0.139448493 | 0.005032269 | 0.097887596 | Up | 2.298236162 |
| GRM7 | 0.471825889 | 0.1370738 | 0.361583064 | NS | 0.863045549 |
| LMCD1 | 0.264391792 | 0.392826318 | 0.599318562 | NS | 0.405799424 |
| SSUH2 | 0.309238621 | 0.46875559 | 0.6580408 | NS | 0.32905354 |
| CAV3 | 0.231444418 | 0.49427958 | NA | NS | 0.306027331 |
| OXTR | 0.350898545 | 0.050853542 | 0.238708444 | NS | 1.293678792 |
| RAD18 | -0.258465338 | 0.016594846 | 0.15196128 | Down | 1.780026777 |
| SRGAP3 | 0.303253148 | 0.058351797 | 0.252212565 | NS | 1.233945762 |
| THUMPD3 | -0.034147749 | 0.537424766 | 0.708869549 | NS | 0.269682324 |
| SETD5 | 0.149433532 | 0.077469969 | 0.281666493 | NS | 1.110866616 |
| LHFPL4 | 0.231031578 | 0.51496387 | 0.69301343 | NS | 0.28822324 |
| MTMR14 | -0.027810931 | 0.548240322 | 0.716409897 | NS | 0.261029026 |
| CPNE9 | 0.681762697 | 0.039262993 | 0.214616748 | Up | 1.4060166 |
| BRPF1 | -0.058996572 | 0.252679685 | 0.479607512 | NS | 0.597429673 |
| OGG1 | 0.020982289 | 0.742476333 | 0.848936245 | NS | 0.129317385 |
| CAMK1 | -0.079822706 | 0.508312407 | 0.687821019 | NS | 0.293869289 |
| TADA3 | 0.016095458 | 0.859073825 | 0.920338791 | NS | 0.065969513 |
| ARPC4 | -0.14035735 | 0.041252392 | 0.218796265 | Down | 1.384550868 |
| ARPC4-TTLL3 | -0.215906279 | 0.471444707 | NA | NS | 0.326569236 |
| TTLL3 | 0.046499058 | 0.683586092 | 0.811627802 | NS | 0.165206782 |
| RPUSD3 | -0.016624979 | 0.753024176 | 0.855274731 | NS | 0.123191081 |
| CIDEC | 0.169151075 | 0.736939267 | NA | NS | 0.132568302 |
| JAGN1 | -0.080389968 | 0.306263475 | 0.527408052 | NS | 0.513904795 |
| IL17RE | 0.278445598 | 0.210471566 | 0.440341584 | NS | 0.676806567 |
| IL17RC | 0.273715345 | 0.06529693 | 0.264481527 | NS | 1.185107235 |
| CRELD1 | 0.216896524 | 0.016897151 | 0.153016426 | Up | 1.772186527 |
| PRRT3 | 0.363116844 | 8.28E-04 | 0.048073047 | Up | 3.081982243 |
| EMC3 | -0.257370901 | 0.064183525 | 0.262840183 | NS | 1.192576437 |
| FANCD2 | -0.043924202 | 0.308872616 | 0.529238414 | NS | 0.510220594 |
| FANCD2OS | 0.069142224 | 0.842454602 | NA | NS | 0.074453493 |
| BRK1 | -0.246347278 | 0.002609002 | 0.078024785 | Down | 2.583525665 |
| VHL | -0.168626492 | 2.45E-06 | 9.51E-04 | Down | 5.610978638 |
| IRAK2 | -0.066303872 | 0.337801378 | 0.553781471 | NS | 0.471338583 |
| TATDN2 | 0.029946826 | 0.548944246 | 0.71696157 | NS | 0.260471762 |
| GHRL | -0.05366063 | 0.633253843 | 0.776665018 | NS | 0.198422166 |
| SEC13 | -0.189715252 | 0.001342741 | 0.059711551 | Down | 2.872007828 |
| ATP2B2 | 0.4793542 | 0.116095892 | 0.335456487 | NS | 0.935183148 |
| SLC6A11 | 0.645304375 | 0.133491774 | 0.356694506 | NS | 0.874545495 |
| SLC6A1 | 0.499086261 | 0.219144158 | 0.448551211 | NS | 0.659270103 |
| HRH1 | 0.477652884 | 0.05849167 | 0.252512011 | NS | 1.232905982 |
| ATG7 | 0.116773386 | 0.059657906 | 0.254856439 | NS | 1.224331993 |
| VGLL4 | 0.091760715 | 0.122682266 | 0.343564349 | NS | 0.911218212 |
| TAMM41 | 0.102607142 | 0.359896397 | 0.572764191 | NS | 0.443822501 |
| SYN2 | 0.592654921 | 0.059207063 | 0.253884625 | NS | 1.227626482 |
| TIMP4 | 0.379682295 | 0.519697294 | NA | NS | 0.284249544 |
| PPARG | 0.303245099 | 0.274061244 | 0.498866181 | NS | 0.562152375 |
| TSEN2 | -0.003123858 | 0.966049642 | 0.980911852 | NS | 0.015000556 |
| MKRN2OS | 0.226390471 | 0.069750127 | 0.270202877 | NS | 1.156454995 |
| MKRN2 | -0.035479826 | 0.351082902 | 0.565080739 | NS | 0.45459032 |
| RAF1 | 0.155964972 | 0.036273338 | 0.208236145 | Up | 1.440412474 |
| TMEM40 | -0.003358885 | 0.981338639 | 0.989156177 | NS | 0.008181101 |
| CAND2 | 0.253313964 | 0.114978384 | 0.333658762 | NS | 0.9393838 |
| RPL32 | -0.314461114 | 0.016131325 | 0.150269448 | Down | 1.792329964 |
| IQSEC1 | 0.067194812 | 0.539584187 | 0.710206067 | NS | 0.267940786 |
| NUP210 | 0.045426534 | 0.637474405 | 0.779366984 | NS | 0.195537248 |
| HDAC11 | -0.111937861 | 0.186238063 | 0.415835484 | NS | 0.729931553 |
| FBLN2 | 0.169888413 | 0.368654311 | 0.580406285 | NS | 0.433380684 |
| WNT7A | -0.008159664 | 0.94120573 | 0.96761205 | NS | 0.026315437 |
| CHCHD4 | -0.070206367 | 0.404915068 | 0.60860084 | NS | 0.392636062 |
| TMEM43 | -0.030441671 | 0.667297269 | 0.800958882 | NS | 0.175680653 |
| XPC | -0.181991304 | 0.006131149 | 0.104709593 | Down | 2.212458161 |
| LSM3 | -0.344473019 | 0.039264875 | 0.214616748 | Down | 1.405995778 |
| SLC6A6 | 0.118204771 | 0.279135729 | 0.503208948 | NS | 0.554184571 |
| GRIP2 | 0.272073748 | 0.157481836 | 0.3849556 | NS | 0.80276953 |
| CCDC174 | -0.175000347 | 0.005908895 | 0.10308115 | Down | 2.228493758 |
| C3orf20 | 0.334549741 | 0.175105412 | 0.404464362 | NS | 0.756700431 |
| FGD5 | 0.502099438 | 0.025957164 | 0.182465 | Up | 1.585742758 |
| NR2C2 | 0.090415689 | 0.093432506 | 0.306166324 | NS | 1.029502002 |
| MRPS25 | -0.029917995 | 0.600300059 | 0.754559383 | NS | 0.221631614 |
| RBSN | -0.001732833 | 0.95643068 | 0.976165401 | NS | 0.019346501 |
| CAPN7 | -0.00575985 | 0.881206722 | 0.932827018 | NS | 0.054922199 |
| SH3BP5 | -0.125639517 | 0.065387058 | 0.264481527 | NS | 1.184508201 |
| METTL6 | -0.056516957 | 0.315142426 | 0.534542427 | NS | 0.501493125 |
| EAF1 | -0.141192813 | 0.009648878 | 0.123395617 | Down | 2.015523189 |
| COLQ | 0.046800709 | 0.706022131 | 0.826187668 | NS | 0.151181685 |
| HACL1 | -0.092361587 | 0.112330458 | 0.330922582 | NS | 0.94950247 |
| BTD | 0.10632305 | 0.01207403 | 0.13408728 | Up | 1.918147756 |
| ANKRD28 | 0.009542365 | 0.866357308 | 0.924442765 | NS | 0.062302957 |
| GALNT15 | 0.496718487 | 0.21367096 | 0.443230566 | NS | 0.670254498 |
| DPH3 | -0.08737792 | 0.265454189 | 0.491585012 | NS | 0.576010416 |
| OXNAD1 | -0.015196572 | 0.859083899 | 0.920338791 | NS | 0.065964421 |
| RFTN1 | 0.098589498 | 0.118452179 | 0.338929511 | NS | 0.926456945 |
| DAZL | 0.309879558 | 0.299103407 | 0.520433287 | NS | 0.524178641 |
| PLCL2 | -0.105775185 | 0.124629384 | 0.346180975 | NS | 0.904379553 |
| TBC1D5 | -0.028352497 | 0.536406671 | 0.708295535 | NS | 0.27050583 |
| SATB1 | 0.115271368 | 0.107076006 | 0.323901028 | NS | 0.970307838 |
| KCNH8 | 0.169623836 | 0.21788357 | 0.447490765 | NS | 0.661775518 |
| EFHB | 0.265694772 | 0.367418214 | 0.579539855 | NS | 0.434839319 |
| RAB5A | -0.16780924 | 0.014862791 | 0.146214626 | Down | 1.827899617 |
| PP2D1 | 0.197071913 | 0.01385484 | 0.141394243 | Up | 1.858398488 |
| KAT2B | -0.147555654 | 0.284702349 | 0.507649736 | NS | 0.545608949 |
| SGO1 | 0.03512948 | 0.70908415 | 0.827740721 | NS | 0.149302222 |
| ZNF385D | 0.359779748 | 0.12697263 | 0.348807379 | NS | 0.896289885 |
| UBE2E2 | 0.124608121 | 0.135678161 | 0.3596977 | NS | 0.867490051 |
| UBE2E1 | -0.095968767 | 0.212778235 | 0.442579534 | NS | 0.672072798 |
| NKIRAS1 | 0.003184094 | 0.957172735 | 0.976444097 | NS | 0.019009681 |
| RPL15 | -0.228387791 | 0.064468148 | 0.263488519 | NS | 1.190654809 |
| NR1D2 | -0.157883487 | 0.030265259 | 0.193469884 | Down | 1.5190556 |
| THRB | 0.104431883 | 0.484289746 | 0.670203865 | NS | 0.314894726 |
| RARB | 0.122814156 | 0.099773965 | 0.314784866 | NS | 1.000982769 |
| TOP2B | -0.12920183 | 0.014914844 | 0.146349507 | Down | 1.826381295 |
| NGLY1 | 0.036438012 | 0.370839969 | 0.581904895 | NS | 0.430813464 |
| OXSM | -0.133205939 | 0.138917345 | 0.363222438 | NS | 0.857243525 |
| LRRC3B | 0.540391303 | 0.22112215 | 0.449806618 | NS | 0.655367751 |
| NEK10 | 0.139673643 | 0.385182255 | 0.593477126 | NS | 0.414333728 |
| SLC4A7 | -0.036952275 | 0.53719336 | 0.708735809 | NS | 0.269869364 |
| EOMES | -0.245680516 | 0.011979213 | 0.134014426 | Down | 1.921571707 |
| CMC1 | -0.012140951 | 0.895622843 | 0.941415199 | NS | 0.047874838 |
| AZI2 | 0.024293018 | 0.698837482 | 0.82172696 | NS | 0.15562381 |
| ZCWPW2 | 0.007515269 | 0.96259467 | 0.979032249 | NS | 0.016556547 |
| RBMS3 | 0.176563509 | 0.518775182 | 0.695811961 | NS | 0.285020808 |
| TGFBR2 | 0.100768474 | 0.07971929 | 0.285136837 | NS | 1.098436581 |
| GADL1 | 0.534463762 | 0.177555388 | 0.407521884 | NS | 0.750666145 |
| STT3B | -0.132884009 | 0.047959906 | 0.232647738 | Down | 1.319121674 |
| OSBPL10 | -0.007615103 | 0.944122302 | 0.969522418 | NS | 0.024971743 |
| ZNF860 | -0.076560021 | 0.529196893 | 0.70382202 | NS | 0.276382714 |
| GPD1L | -0.170104973 | 0.002759214 | 0.079323886 | Down | 2.559214557 |
| CMTM8 | 0.119158241 | 0.475317338 | 0.66338027 | NS | 0.323016344 |
| CMTM7 | -0.025579293 | 0.548728394 | 0.716765283 | NS | 0.260642567 |
| CMTM6 | -0.024716772 | 0.827541444 | 0.901763056 | NS | 0.082210247 |
| DYNC1LI1 | -0.100036634 | 0.098281504 | 0.313029559 | NS | 1.007528206 |
| CNOT10 | -0.152866358 | 9.40E-04 | 0.051014532 | Down | 3.026867053 |
| TRIM71 | 0.536423962 | 0.051468495 | 0.239579569 | NS | 1.288458529 |
| CCR4 | -0.168571448 | 0.038018789 | 0.212212115 | Down | 1.42000172 |
| GLB1 | -0.192178562 | 0.017694262 | 0.155670827 | Down | 1.752167547 |
| TMPPE | -0.037474132 | 0.680298082 | 0.809570625 | NS | 0.167300753 |
| CRTAP | -0.188213522 | 0.01295424 | 0.136466422 | Down | 1.887588064 |
| SUSD5 | 0.360177675 | 0.048455863 | 0.233432093 | Up | 1.314653669 |
| FBXL2 | 0.321589143 | 0.007909223 | 0.11472857 | Up | 2.101866173 |
| UBP1 | 0.054788795 | 0.2815158 | 0.504639547 | NS | 0.550497226 |
| CLASP2 | -0.107487214 | 0.0107178 | 0.12826482 | Down | 1.969894354 |
| PDCD6IP | 0.092844354 | 0.016727222 | 0.152381397 | NS | 1.776576186 |
| ARPP21 | 0.30185858 | 0.382056098 | 0.591485754 | NS | 0.417872864 |
| STAC | 0.215986128 | 0.474639756 | 0.662857472 | NS | 0.323635888 |
| DCLK3 | 0.627961534 | 0.104951858 | 0.320907849 | NS | 0.979009868 |
| TRANK1 | 0.02335708 | 0.799756411 | 0.884702387 | NS | 0.09704227 |
| EPM2AIP1 | 0.03120946 | 0.547702467 | 0.7160589 | NS | 0.261455303 |
| MLH1 | -0.084904152 | 0.037616333 | 0.210633069 | NS | 1.424623546 |
| LRRFIP2 | 0.045316985 | 0.486100083 | 0.671572908 | NS | 0.313274305 |
| GOLGA4 | -0.216005376 | 0.003298281 | 0.084985048 | Down | 2.481712403 |
| ITGA9 | 0.273395732 | 0.104406993 | 0.320470766 | NS | 0.981270412 |
| CTDSPL | 0.135810669 | 0.358910648 | 0.571832571 | NS | 0.445013658 |
| VILL | -0.109909557 | 0.0939481 | 0.30723623 | NS | 1.027111999 |
| PLCD1 | -0.140302139 | 0.020517705 | 0.166276176 | Down | 1.687871226 |
| DLEC1 | 0.154694973 | 0.129997326 | 0.352095778 | NS | 0.88606558 |
| ACAA1 | 0.049792978 | 0.362729346 | 0.575276172 | NS | 0.440417307 |
| MYD88 | 0.294769916 | 0.001417097 | 0.061004445 | Up | 2.848600294 |
| OXSR1 | -0.177069373 | 0.002628815 | 0.078188473 | Down | 2.580239954 |
| SLC22A13 | -0.0337032 | 0.904324918 | 0.946998137 | NS | 0.043675503 |
| SLC22A14 | 0.594403479 | 0.096265044 | 0.310402489 | NS | 1.016531388 |
| XYLB | 0.245876614 | 0.120411144 | 0.341125189 | NS | 0.919333316 |
| ACVR2B | -0.060152854 | 0.464842736 | 0.655127898 | NS | 0.332693951 |
| EXOG | 0.104804778 | 0.136718288 | 0.361144434 | NS | 0.864173387 |
| SCN5A | 0.501713556 | 0.091884955 | 0.303701724 | NS | 1.036755592 |
| SCN10A | 0.362458269 | 0.433241852 | 0.629744678 | NS | 0.363269596 |
| SCN11A | 0.138122685 | 0.62745022 | 0.77307214 | NS | 0.202420724 |
| WDR48 | -0.066633303 | 0.078605413 | 0.283365525 | NS | 1.104547544 |
| GORASP1 | -0.048089217 | 0.356456806 | 0.569525168 | NS | 0.447993089 |
| TTC21A | 0.205015789 | 0.067105781 | 0.266883237 | NS | 1.173240063 |
| CSRNP1 | -0.035872185 | 0.708019019 | 0.82706671 | NS | 0.149955076 |
| XIRP1 | 0.295377068 | 0.530728907 | NA | NS | 0.275127257 |
| CX3CR1 | -0.126434367 | 0.242090064 | 0.469918053 | NS | 0.616023034 |
| CCR8 | -0.184510913 | 0.109362172 | 0.327424668 | NS | 0.961132873 |
| SLC25A38 | -0.199403276 | 0.058873121 | 0.253156633 | NS | 1.230082938 |
| RPSA | -0.224353236 | 0.087672424 | 0.297286622 | NS | 1.057136987 |
| MOBP | 0.424500798 | 0.288915779 | 0.512011775 | NS | 0.539228739 |
| MYRIP | 0.376561919 | 0.006751058 | 0.109344229 | Up | 2.170628136 |
| EIF1B | -0.184524222 | 0.171387072 | 0.399868249 | NS | 0.76602194 |
| ENTPD3 | 0.217760213 | 0.208030153 | 0.437734891 | NS | 0.681873712 |
| RPL14 | -0.248173058 | 0.031850538 | 0.197978516 | Down | 1.496883227 |
| ZNF619 | 0.095922026 | 0.116728052 | 0.336518862 | NS | 0.932824764 |
| ZNF620 | 0.051778897 | 0.452369346 | 0.645091081 | NS | 0.344506832 |
| ZNF621 | 0.183634722 | 0.006652936 | 0.108543277 | Up | 2.176986644 |
| CTNNB1 | -0.102452871 | 0.058424279 | 0.252404602 | NS | 1.233406639 |
| ULK4 | -0.06163092 | 0.25912207 | 0.485568555 | NS | 0.586495595 |
| TRAK1 | 0.116109486 | 0.219039732 | 0.448497803 | NS | 0.659477101 |
| CCK | 0.786661134 | 0.1338081 | NA | NS | 0.873517597 |
| LYZL4 | 0.495372899 | 0.363003545 | NA | NS | 0.440089134 |
| VIPR1 | 0.168319897 | 0.120815052 | 0.341402767 | NS | 0.917878956 |
| SEC22C | -0.017674695 | 0.632066573 | 0.775886882 | NS | 0.199237177 |
| SS18L2 | -0.132249212 | 0.259829058 | 0.485843002 | NS | 0.585312281 |
| NKTR | 1.75E-04 | 0.997993409 | 0.999116173 | NS | 8.72E-04 |
| ZBTB47 | 0.121059436 | 0.180920399 | 0.410892401 | NS | 0.742512464 |
| KLHL40 | 0.711178988 | 0.159907128 | NA | NS | 0.796132176 |
| HHATL | 0.529195843 | 0.225451096 | NA | NS | 0.646947649 |
| CCDC13 | 0.420018964 | 0.052506475 | NA | NS | 1.279787135 |
| HIGD1A | -0.20294625 | 0.090205746 | 0.300887271 | NS | 1.044765796 |
| ACKR2 | -0.328051656 | 0.276540303 | 0.500969068 | NS | 0.558241565 |
| KRBOX1 | -0.374186389 | 0.448528652 | NA | NS | 0.348209809 |
| CYP8B1 | 0.670035184 | 0.119771495 | NA | NS | 0.921646531 |
| ZNF662 | 0.1417798 | 0.368851628 | 0.580544833 | NS | 0.433148296 |
| GASK1A | 0.367545351 | 0.118041683 | 0.338399285 | NS | 0.927964608 |
| POMGNT2 | -0.036202402 | 0.709072049 | 0.827740721 | NS | 0.149309634 |
| SNRK | -0.059323734 | 0.320421245 | 0.539143881 | NS | 0.494278696 |
| ANO10 | 0.074733667 | 0.252919774 | 0.479818478 | NS | 0.597017215 |
| ABHD5 | -0.015308917 | 0.898919043 | 0.943840268 | NS | 0.046279419 |
| TOPAZ1 | 0.499110797 | 0.265968528 | 0.491940819 | NS | 0.575169751 |
| TCAIM | 0.107639448 | 0.110358712 | 0.328879014 | NS | 0.957193377 |
| ZNF445 | 0.202156049 | 0.018419893 | 0.157584735 | Up | 1.734712892 |
| ZNF852 | -0.096195039 | 0.432948628 | 0.629508569 | NS | 0.363563633 |
| ZKSCAN7 | -0.006545369 | 0.960777966 | 0.978291703 | NS | 0.017376965 |
| ZNF197 | -0.079894718 | 0.345490428 | 0.560413621 | NS | 0.461563981 |
| ZNF35 | -0.042921838 | 0.731707272 | 0.841158516 | NS | 0.135662629 |
| ZNF502 | 0.343489694 | 0.192373342 | NA | NS | 0.71585511 |
| ZNF501 | -0.219823606 | 0.309814635 | NA | NS | 0.508898071 |
| KIAA1143 | -0.320836006 | 0.00475446 | 0.095617668 | Down | 2.322898783 |
| KIF15 | -0.559673415 | 0.00421753 | 0.0921659 | Down | 2.374941861 |
| TMEM42 | 0.002068797 | 0.990032969 | 0.994124998 | NS | 0.004350343 |
| ZDHHC3 | -0.047547007 | 0.451382878 | 0.644308389 | NS | 0.345454918 |
| EXOSC7 | -0.165667447 | 0.002188205 | 0.072832159 | Down | 2.65991203 |
| CLEC3B | 1.102875473 | 0.005892526 | NA | Up | 2.229698495 |
| CDCP1 | 0.347104572 | 0.110409639 | 0.328945174 | NS | 0.95699301 |
| TMEM158 | -0.414099903 | 0.186522955 | 0.415984879 | NS | 0.729267713 |
| LARS2 | -0.03898733 | 0.388330212 | 0.596161585 | NS | 0.41079882 |
| LIMD1 | 0.17683575 | 0.011074425 | 0.129467323 | Up | 1.955678806 |
| SACM1L | -0.090119135 | 0.059356041 | 0.25415179 | NS | 1.226535072 |
| SLC6A20 | 0.431123105 | 0.115445944 | 0.334470149 | NS | 0.937621322 |
| LZTFL1 | -0.136504677 | 0.05233588 | 0.241847979 | NS | 1.281200468 |
| CCR9 | -0.066960522 | 0.523144027 | 0.698847673 | NS | 0.281378729 |
| FYCO1 | 0.120137716 | 0.104344094 | 0.320354348 | NS | 0.981532129 |
| CXCR6 | -0.084121694 | 0.317000882 | 0.53644682 | NS | 0.498939529 |
| XCR1 | 0.12565176 | 0.607896166 | 0.759604867 | NS | 0.216170595 |
| CCR3 | 0.215983172 | 0.131450405 | 0.354132123 | NS | 0.881238071 |
| CCR1 | 0.28055021 | 0.026437031 | 0.183306356 | Up | 1.577787312 |
| CCR2 | -0.134183 | 0.246287124 | 0.474507252 | NS | 0.608558293 |
| CCR5 | -0.123964263 | 0.15445122 | 0.381395414 | NS | 0.811208657 |
| CCRL2 | -0.097221321 | 0.286706736 | 0.509874023 | NS | 0.542562103 |
| LTF | 0.439354962 | 0.062580213 | 0.260158294 | NS | 1.203562962 |
| RTP3 | 0.674221463 | 0.228746715 | NA | NS | 0.640645134 |
| LRRC2 | -0.082220684 | 0.733368011 | 0.842396864 | NS | 0.134678038 |
| TDGF1 | 0.972403635 | 0.079802114 | NA | NS | 1.097985603 |
| FAM240A | 0.367506156 | 0.53487541 | NA | NS | 0.271747368 |
| ALS2CL | -0.084653826 | 0.450392518 | 0.643681777 | NS | 0.346408832 |
| TMIE | -0.017600043 | 0.921403344 | 0.956615413 | NS | 0.035550216 |
| PRSS45P | 1.051189355 | 0.069633712 | NA | NS | 1.15718045 |
| MYL3 | 0.439717848 | 0.281196627 | NA | NS | 0.550989893 |
| PTH1R | 0.749013393 | 0.121145129 | NA | NS | 0.916694043 |
| CCDC12 | -0.106557292 | 0.075071046 | 0.27811629 | NS | 1.124527535 |
| NBEAL2 | -0.060984594 | 0.721624458 | 0.835776441 | NS | 0.141688756 |
| SETD2 | -0.051985064 | 0.581093589 | 0.740451328 | NS | 0.235753916 |
| KIF9 | 0.034746916 | 0.749029366 | 0.852555565 | NS | 0.125501155 |
| KLHL18 | 0.00251801 | 0.960246757 | 0.97783776 | NS | 0.017617151 |
| PTPN23 | -0.133395953 | 0.34221593 | 0.557467049 | NS | 0.465699778 |
| SCAP | 0.030748479 | 0.715571086 | 0.831794184 | NS | 0.145347216 |
| ELP6 | -0.100905033 | 0.14788474 | 0.373742695 | NS | 0.830076639 |
| CSPG5 | 0.869981982 | 0.012084366 | 0.13408728 | Up | 1.917776135 |
| SMARCC1 | -0.094020278 | 0.13656253 | 0.361024479 | NS | 0.864668446 |
| DHX30 | -0.149444947 | 0.010325747 | 0.126799065 | Down | 1.986078524 |
| MAP4 | 0.054618652 | 0.596657277 | 0.75175978 | NS | 0.224275058 |
| CDC25A | 0.224069379 | 0.300166913 | 0.521619442 | NS | 0.522637182 |
| CAMP | 0.592001205 | 0.003907706 | 0.089796534 | Up | 2.40807811 |
| ZNF589 | -0.134213989 | 0.023957423 | 0.175350879 | Down | 1.6205599 |
| NME6 | -0.003849882 | 0.936071159 | 0.964541604 | NS | 0.028691135 |
| SPINK8 | 0.393339744 | 0.072697818 | 0.274345075 | NS | 1.138478624 |
| FBXW12 | 0.808355095 | 0.086002393 | NA | NS | 1.065489466 |
| PLXNB1 | 0.289482577 | 0.231029116 | 0.459450227 | NS | 0.636333284 |
| CCDC51 | -0.131407714 | 0.138956598 | 0.363222438 | NS | 0.857120828 |
| TMA7 | -0.405205136 | 0.047636136 | 0.23187307 | Down | 1.32206347 |
| ATRIP | -0.146874508 | 0.184721794 | 0.41487678 | NS | 0.733481863 |
| SHISA5 | 0.126172007 | 0.085907529 | 0.294615999 | NS | 1.065968772 |
| PFKFB4 | 0.069461585 | 0.480206081 | 0.667051596 | NS | 0.318572345 |
| UCN2 | 0.948943571 | 0.08638192 | NA | NS | 1.063577148 |
| COL7A1 | 0.304975717 | 0.219548453 | 0.448876282 | NS | 0.658469619 |
| UQCRC1 | -0.145283674 | 0.066537518 | 0.265987475 | NS | 1.176933402 |
| TMEM89 | 1.492141706 | 0.012520635 | NA | Up | 1.902373658 |
| SLC26A6 | 0.024694271 | 0.75096055 | 0.853862502 | NS | 0.124382877 |
| CELSR3 | 0.208239868 | 0.215132247 | 0.444591115 | NS | 0.667294486 |
| NCKIPSD | 0.109822038 | 0.276495109 | 0.500969068 | NS | 0.558312546 |
| IP6K2 | 0.060432797 | 0.037511081 | 0.21019789 | NS | 1.42584042 |
| PRKAR2A | -0.030507621 | 0.579777321 | 0.739395267 | NS | 0.236738777 |
| SLC25A20 | -0.098017012 | 0.229406165 | 0.457791831 | NS | 0.639394915 |
| ARIH2 | -0.084527518 | 0.027055441 | 0.185571641 | NS | 1.567745384 |
| P4HTM | -0.024793478 | 0.626399574 | 0.772158843 | NS | 0.203148546 |
| WDR6 | -0.084149254 | 0.229523924 | 0.457848351 | NS | 0.639172039 |
| DALRD3 | 0.039860183 | 0.52001606 | 0.696688569 | NS | 0.283983243 |
| NDUFAF3 | 0.014298469 | 0.827436867 | 0.901734909 | NS | 0.082265133 |
| IMPDH2 | -0.161473493 | 0.01890198 | 0.15956253 | Down | 1.723492702 |
| QRICH1 | -0.125157199 | 0.006957317 | 0.110119911 | Down | 2.157558203 |
| QARS1 | -0.163978295 | 0.021165925 | 0.168202484 | Down | 1.674362745 |
| USP19 | -0.164734429 | 0.01794743 | 0.15641156 | Down | 1.745997729 |
| LAMB2 | 0.203690766 | 0.292516046 | 0.515264643 | NS | 0.533850306 |
| CCDC71 | 0.047553941 | 0.369251045 | 0.580729806 | NS | 0.432678267 |
| KLHDC8B | 0.060475603 | 0.612575187 | 0.762995428 | NS | 0.212840599 |
| C3orf84 | 0.18073728 | 0.665903833 | NA | NS | 0.176588485 |
| IHO1 | 0.383707241 | 0.010384759 | 0.127182759 | Up | 1.983603563 |
| C3orf62 | 0.148480541 | 0.123165539 | 0.343908458 | NS | 0.909510789 |
| USP4 | 0.130953611 | 0.055494104 | 0.248604264 | NS | 1.255753155 |
| GPX1 | -0.293970839 | 0.031039183 | 0.195775095 | Down | 1.508089717 |
| RHOA | -0.032952228 | 0.682796532 | 0.811110855 | NS | 0.165708693 |
| TCTA | 0.008522718 | 0.861702508 | 0.92205349 | NS | 0.064642643 |
| AMT | 0.317699642 | 0.0624728 | 0.259806076 | NS | 1.20430903 |
| DAG1 | 0.145323765 | 0.036187855 | 0.208162128 | Up | 1.441437157 |
| BSN | 0.301540186 | 0.054185144 | 0.246122206 | NS | 1.26611977 |
| APEH | -0.159575414 | 0.002778599 | 0.079430699 | Down | 2.556174071 |
| MST1 | 0.034475641 | 0.683855914 | 0.811840864 | NS | 0.165035393 |
| RNF123 | -0.08918378 | 0.60286362 | 0.756163132 | NS | 0.219780923 |
| GMPPB | -0.02852388 | 0.625622173 | 0.771733981 | NS | 0.203687868 |
| IP6K1 | 0.136735427 | 0.146677899 | 0.372132189 | NS | 0.833635321 |
| CDHR4 | 0.34136624 | 0.498203921 | NA | NS | 0.302592858 |
| INKA1 | 0.063204249 | 0.553755989 | 0.720722774 | NS | 0.256681564 |
| UBA7 | -0.019534137 | 0.72792754 | 0.839053008 | NS | 0.13791185 |
| TRAIP | 0.088039609 | 0.551731049 | 0.719031683 | NS | 0.258272575 |
| CAMKV | 0.901169387 | 0.11070071 | NA | NS | 0.955849592 |
| MST1R | 0.59848904 | 0.07081819 | 0.27176243 | NS | 1.149855179 |
| MON1A | -0.118124988 | 0.086198308 | 0.29504015 | NS | 1.064501261 |
| RBM6 | -0.134534217 | 0.082980445 | 0.290260272 | NS | 1.081024241 |
| RBM5 | 0.001660729 | 0.967355671 | 0.981846285 | NS | 0.014413818 |
| SEMA3F | 0.754961791 | 0.121384166 | 0.342066258 | NS | 0.91583796 |
| GNAT1 | 0.805417019 | 0.100909421 | NA | NS | 0.996068287 |
| SLC38A3 | 0.583978352 | 0.245994155 | NA | NS | 0.609075212 |
| GNAI2 | 0.077954012 | 0.2734233 | 0.498634409 | NS | 0.563164479 |
| SEMA3B | 0.438852725 | 0.077947402 | 0.28242971 | NS | 1.108198356 |
| LSMEM2 | 0.310739103 | 0.25868543 | NA | NS | 0.587228031 |
| IFRD2 | -0.096865426 | 0.37494885 | 0.585708926 | NS | 0.426027974 |
| HYAL3 | 0.02980317 | 0.793232603 | 0.880637817 | NS | 0.100599444 |
| NAA80 | 0.314429878 | 0.031545221 | 0.197174853 | Up | 1.50106642 |
| HYAL1 | 0.240200121 | 0.021124783 | 0.168202484 | Up | 1.675207747 |
| HYAL2 | 0.059852223 | 0.478559872 | 0.666071166 | NS | 0.320063721 |
| TUSC2 | 0.08669571 | 0.158955681 | 0.386625937 | NS | 0.798723947 |
| RASSF1 | -0.111431781 | 0.049935832 | 0.236635186 | Down | 1.301587712 |
| ZMYND10 | 0.094870616 | 0.342517127 | 0.557798994 | NS | 0.465317707 |
| NPRL2 | -0.127115404 | 0.013056381 | 0.137118535 | Down | 1.884177172 |
| CYB561D2 | -0.087482318 | 0.127247825 | 0.348830209 | NS | 0.895349632 |
| TMEM115 | 0.124412026 | 0.047703719 | 0.232004796 | Up | 1.321447758 |
| CACNA2D2 | 0.109458792 | 0.365136014 | 0.577480818 | NS | 0.437545329 |
| C3orf18 | -0.236251398 | 5.98E-04 | 0.040636454 | Down | 3.223530716 |
| HEMK1 | -0.082769339 | 0.153416767 | 0.380087378 | NS | 0.814127174 |
| CISH | 0.212265694 | 0.050968539 | 0.238791409 | NS | 1.292697815 |
| MAPKAPK3 | -0.126308488 | 0.06591753 | 0.265127437 | NS | 1.180999073 |
| DOCK3 | 0.262302574 | 0.056582969 | 0.249925391 | NS | 1.247314269 |
| MANF | -0.042803331 | 0.634618838 | 0.777257365 | NS | 0.19748704 |
| RBM15B | -0.092841276 | 0.046208 | 0.229024017 | NS | 1.335282827 |
| DCAF1 | -0.016927895 | 0.736124534 | 0.844413929 | NS | 0.133048707 |
| RAD54L2 | 0.024943574 | 0.674775186 | 0.806117594 | NS | 0.170840896 |
| TEX264 | -0.146640692 | 0.012108089 | 0.134107677 | Down | 1.916924396 |
| GRM2 | 0.33369627 | 0.247977577 | 0.476082024 | NS | 0.605587588 |
| IQCF6 | 1.295197698 | 0.016423908 | NA | Up | 1.784523507 |
| IQCF2 | 0.476417125 | 0.397753431 | NA | NS | 0.400386066 |
| IQCF1 | 0.021254761 | 0.969990064 | NA | NS | 0.013232714 |
| RRP9 | -0.354698176 | 3.38E-05 | 0.007962653 | Down | 4.471268684 |
| PARP3 | 0.201253393 | 0.087801267 | 0.297553096 | NS | 1.056499215 |
| GPR62 | 0.810705692 | 0.083493654 | NA | NS | 1.078346532 |
| PCBP4 | -0.102368273 | 0.222675501 | 0.451412976 | NS | 0.652327562 |
| ABHD14B | 0.128022313 | 0.071411726 | 0.272521361 | NS | 1.146230467 |
| ABHD14A | -0.237821687 | 0.017372631 | 0.154692513 | Down | 1.760134397 |
| ACY1 | 0.313708268 | 0.261993212 | NA | NS | 0.58170996 |
| RPL29 | -0.194628048 | 0.092941812 | 0.305431681 | NS | 1.031788864 |
| DUSP7 | 0.13965289 | 0.021594359 | 0.168842295 | Up | 1.665659685 |
| POC1A | -0.048746196 | 0.642839348 | 0.783893655 | NS | 0.191897548 |
| ALAS1 | 0.079942391 | 0.152320145 | 0.378947167 | NS | 0.817242656 |
| TWF2 | -0.028885039 | 0.681447601 | 0.810207571 | NS | 0.166567533 |
| PPM1M | -0.036311029 | 0.46263626 | 0.6532805 | NS | 0.334760331 |
| WDR82 | -0.110889495 | 0.072139469 | 0.273763446 | NS | 1.141827058 |
| GLYCTK | 0.00694575 | 0.928418163 | 0.960718993 | NS | 0.032256372 |
| DNAH1 | -0.005396561 | 0.960972882 | 0.97844667 | NS | 0.017288868 |
| BAP1 | -0.11585858 | 0.053358946 | 0.243966175 | NS | 1.272792761 |
| PHF7 | -0.365821205 | 0.004857575 | 0.096653512 | Down | 2.313580448 |
| SEMA3G | 0.34216492 | 0.068416248 | 0.268575405 | NS | 1.164840746 |
| TNNC1 | 0.031531059 | 0.866074776 | NA | NS | 0.06244461 |
| NISCH | 0.029648483 | 0.648836619 | 0.788174637 | NS | 0.187864647 |
| STAB1 | -0.017761571 | 0.900485719 | 0.944718083 | NS | 0.04552317 |
| NT5DC2 | 0.421687551 | 0.010643671 | 0.127927347 | Up | 1.972908566 |
| SMIM4 | 0.160389442 | 0.036302349 | 0.208236145 | Up | 1.440065273 |
| PBRM1 | -0.074558825 | 0.042261157 | 0.220751111 | NS | 1.374058617 |
| GNL3 | -0.24807008 | 0.015869862 | 0.149445555 | Down | 1.799426838 |
| GLT8D1 | -0.158560376 | 0.005353561 | 0.099924411 | Down | 2.271357249 |
| SPCS1 | 0.157189854 | 0.059577019 | 0.254791869 | NS | 1.224921229 |
| NEK4 | -0.339262466 | 2.13E-04 | 0.023858165 | Down | 3.672606208 |
| ITIH1 | 0.137570596 | 0.580527894 | 0.739981623 | NS | 0.236176908 |
| ITIH3 | 0.247418264 | 0.355883242 | 0.568903557 | NS | 0.448692462 |
| ITIH4 | 0.215382009 | 0.356784504 | 0.569969217 | NS | 0.447594017 |
| MUSTN1 | 0.327578449 | 0.400018424 | NA | NS | 0.397920006 |
| STIMATE-MUSTN1 | 0.257209805 | 0.15421555 | 0.381161723 | NS | 0.811871833 |
| STIMATE | 0.017067684 | 0.870336659 | 0.926875152 | NS | 0.060312724 |
| SFMBT1 | 0.067630852 | 0.185817423 | 0.415463304 | NS | 0.730913567 |
| RFT1 | 0.010382402 | 0.816736049 | 0.895742218 | NS | 0.087918275 |
| PRKCD | 0.099822762 | 0.343449805 | 0.558502793 | NS | 0.464136726 |
| TKT | 0.016522932 | 0.850497972 | 0.916056274 | NS | 0.070326718 |
| DCP1A | 0.130802271 | 0.012328056 | 0.134659948 | Up | 1.909105412 |
| CACNA1D | 0.304721764 | 0.046873666 | 0.230315532 | Up | 1.329071081 |
| CHDH | 0.620543031 | 0.00460376 | 0.094750689 | Up | 2.336887299 |
| IL17RB | 0.168067424 | 0.007007807 | 0.110532234 | Up | 2.154417845 |
| ACTR8 | -0.116392224 | 0.022459068 | 0.171252981 | Down | 1.648608273 |
| SELENOK | -0.243434916 | 0.055740422 | 0.248760687 | NS | 1.253829751 |
| CACNA2D3 | -0.299658533 | 0.010832798 | 0.12856202 | Down | 1.965259362 |
| LRTM1 | 0.383825831 | 0.476611923 | NA | NS | 0.321835098 |
| WNT5A | 0.701439328 | 0.061514886 | 0.257977278 | NS | 1.211019777 |
| ERC2 | 0.192865421 | 0.594603769 | 0.750384214 | NS | 0.225772343 |
| CCDC66 | -0.22311365 | 0.073617873 | 0.27580509 | NS | 1.133016737 |
| TASOR | -0.129420577 | 0.040973192 | 0.218105862 | Down | 1.3875002 |
| ARHGEF3 | 0.011889364 | 0.782930347 | 0.874453587 | NS | 0.106276873 |
| SPATA12 | 0.766983341 | 0.080393007 | NA | NS | 1.094781725 |
| IL17RD | 0.541781255 | 0.151504234 | 0.377971312 | NS | 0.81957523 |
| HESX1 | 0.58129652 | 0.005079554 | NA | Up | 2.294174411 |
| APPL1 | -0.064653499 | 0.293079248 | 0.515386052 | NS | 0.533014932 |
| ASB14 | 0.154049613 | 0.028496375 | 0.188709674 | Up | 1.545210381 |
| DNAH12 | 0.335919512 | 0.177492112 | 0.407470364 | NS | 0.750820943 |
| PDE12 | -0.07696707 | 0.138512271 | 0.363014451 | NS | 0.858511751 |
| ARF4 | -0.107736219 | 0.096536695 | 0.310604578 | NS | 1.015307574 |
| DENND6A | -0.117683118 | 0.0226621 | 0.171572699 | Down | 1.644699847 |
| SLMAP | 0.107416798 | 0.02131789 | 0.168379028 | Up | 1.671255779 |
| FLNB | 0.050919102 | 0.507849518 | 0.687641579 | NS | 0.294264955 |
| DNASE1L3 | -0.197782877 | 0.332388602 | 0.549152659 | NS | 0.478353878 |
| ABHD6 | -0.148092672 | 0.025841589 | 0.182170348 | Down | 1.58768078 |
| RPP14 | -0.029449992 | 0.54511138 | 0.713828167 | NS | 0.263514752 |
| HTD2 | 0.255005711 | 0.175727845 | NA | NS | 0.755159416 |
| PXK | 0.22306552 | 8.13E-04 | 0.047636432 | Up | 3.089757432 |
| PDHB | -0.197244985 | 0.006281756 | 0.106173271 | Down | 2.201918918 |
| KCTD6 | -0.078502094 | 0.229439033 | 0.457791831 | NS | 0.639332697 |
| ACOX2 | 0.29301652 | 0.157828475 | 0.385290564 | NS | 0.801814639 |
| FAM107A | 0.850033508 | 0.023051445 | 0.172863191 | Up | 1.637301852 |
| FAM3D | 0.143923639 | 0.657738948 | NA | NS | 0.18194644 |
| CFAP20DC | 0.381820902 | 0.209952816 | 0.440001773 | NS | 0.677878297 |
| FHIT | 0.273457418 | 0.057288728 | 0.250991981 | NS | 1.241930822 |
| PTPRG | 0.500558644 | 0.058757759 | 0.253109387 | NS | 1.230934779 |
| C3orf14 | -0.097975238 | 0.567338922 | 0.730611561 | NS | 0.246157421 |
| FEZF2 | 0.604683581 | 0.241604517 | NA | NS | 0.61689495 |
| CADPS | 0.492103589 | 0.222621611 | 0.451412976 | NS | 0.652432678 |
| SYNPR | 0.656129068 | 0.161911857 | NA | NS | 0.790721345 |
| SNTN | 0.487466087 | 0.343572533 | 0.558604157 | NS | 0.463981563 |
| C3orf49 | 0.285090985 | 0.022188136 | 0.170578278 | Up | 1.653879181 |
| THOC7 | -0.312160077 | 0.015062488 | 0.147015649 | Down | 1.822103278 |
| ATXN7 | 0.115881861 | 0.077113714 | 0.281454506 | NS | 1.112868381 |
| PSMD6 | 0.003174441 | 0.936937887 | 0.965131299 | NS | 0.028289199 |
| PRICKLE2 | 0.465663283 | 0.079909288 | 0.285326242 | NS | 1.097402739 |
| ADAMTS9 | 0.620928966 | 0.144724129 | 0.369914478 | NS | 0.839459054 |
| MAGI1 | 0.45078511 | 0.195272655 | 0.424946251 | NS | 0.709358569 |
| SLC25A26 | -0.211470231 | 0.012321576 | 0.134659948 | Down | 1.909333739 |
| LRIG1 | -0.093517648 | 0.22816363 | 0.456877344 | NS | 0.641753581 |
| KBTBD8 | -0.092447624 | 0.404129882 | 0.607851206 | NS | 0.393479036 |
| SUCLG2 | -0.179508312 | 0.012772194 | 0.1360506 | Down | 1.893734489 |
| TAFA1 | -0.203912866 | 0.110220742 | 0.328790965 | NS | 0.95773667 |
| TAFA4 | 0.368317299 | 0.423601415 | NA | NS | 0.373042598 |
| EOGT | -0.020120969 | 0.700791267 | 0.822549366 | NS | 0.154411319 |
| TMF1 | -0.108351578 | 0.029042237 | 0.190037745 | Down | 1.536969933 |
| UBA3 | -0.028189614 | 0.67909611 | 0.808796118 | NS | 0.168068757 |
| ARL6IP5 | -0.358147986 | 0.002725446 | 0.079110476 | Down | 2.564562411 |
| LMOD3 | 0.030655706 | 0.729780112 | 0.840372591 | NS | 0.136807976 |
| FRMD4B | -0.164118342 | 0.027717046 | 0.186639939 | Down | 1.557253051 |
| MITF | 0.117056338 | 0.279635022 | 0.503419463 | NS | 0.553408438 |
| MDFIC2 | 0.620065566 | 0.235743402 | NA | NS | 0.627560454 |
| FOXP1 | 0.099005541 | 0.070026549 | 0.270674772 | NS | 1.154737276 |
| EIF4E3 | 0.16682426 | 0.040900061 | 0.218105862 | Up | 1.388276043 |
| GPR27 | 0.139827399 | 0.2734627 | 0.498634409 | NS | 0.563101903 |
| PROK2 | 0.257468528 | 0.126642455 | 0.348597283 | NS | 0.89742068 |
| RYBP | 0.032863423 | 0.719890725 | 0.834756433 | NS | 0.142733422 |
| SHQ1 | -0.135748613 | 0.029597928 | 0.191700154 | Down | 1.528738692 |
| GXYLT2 | 0.925779174 | 0.047592216 | NA | Up | 1.322464072 |
| PPP4R2 | -0.267239251 | 0.004106533 | 0.090779743 | Down | 2.386524704 |
| EBLN2 | 0.092927374 | 0.127922306 | 0.349506689 | NS | 0.893053721 |
| PDZRN3 | 0.462915147 | 0.247318918 | 0.475507879 | NS | 0.606742663 |
| CNTN3 | 0.611849999 | 0.239069932 | 0.467324991 | NS | 0.621475041 |
| FRG2C | 0.72761186 | 0.143501943 | NA | NS | 0.843142218 |
| ZNF717 | 0.123198717 | 0.04365888 | 0.224086881 | Up | 1.359927413 |
| ROBO2 | 0.474937362 | 0.288718522 | 0.511843768 | NS | 0.539525355 |
| ROBO1 | 0.236150698 | 0.433403585 | 0.629801069 | NS | 0.363107501 |
| GBE1 | -0.082054567 | 0.289668278 | 0.512751809 | NS | 0.538099062 |
| CADM2 | 0.237821765 | 0.476963399 | 0.664724382 | NS | 0.321514946 |
| VGLL3 | 0.515799983 | 0.264294586 | 0.490441204 | NS | 0.577911733 |
| CHMP2B | -0.022422791 | 0.759562305 | 0.85967363 | NS | 0.119436596 |
| POU1F1 | 0.164531185 | 0.312377071 | 0.532085935 | NS | 0.505320852 |
| HTR1F | -0.062028531 | 0.845375699 | 0.913147068 | NS | 0.07295024 |
| CGGBP1 | -0.111804255 | 0.040321023 | 0.216621104 | Down | 1.394468452 |
| ZNF654 | -0.069575756 | 0.297247126 | 0.519153789 | NS | 0.526882336 |
| C3orf38 | -0.077407326 | 0.32557148 | 0.544052393 | NS | 0.487353646 |
| CSNKA2IP | 0.178616316 | 0.705724628 | NA | NS | 0.151364726 |
| EPHA3 | 0.59073225 | 0.211604524 | 0.441282718 | NS | 0.674475052 |
| PROS1 | -0.188279183 | 0.271112725 | 0.496364509 | NS | 0.566850097 |
| ARL13B | -0.036491435 | 0.644301621 | 0.784889255 | NS | 0.190910776 |
| STX19 | 0.399170248 | 0.004532559 | 0.094211252 | Up | 2.34365651 |
| DHFR2 | -0.110192256 | 0.091742403 | 0.303537202 | NS | 1.037429889 |
| NSUN3 | -0.333222075 | 0.009581762 | 0.122936422 | Down | 2.018554621 |
| MTRNR2L12 | -0.404144806 | 0.614612572 | 0.764203112 | NS | 0.211398561 |
| EPHA6 | 0.426985254 | 0.280924763 | 0.504116551 | NS | 0.551409977 |
| ARL6 | -0.077333099 | 0.315363884 | 0.534759638 | NS | 0.501188044 |
| CRYBG3 | -0.11885987 | 0.080304214 | 0.285933942 | NS | 1.095261663 |
| RIOX2 | -0.118145223 | 0.040591517 | 0.21745659 | Down | 1.391564718 |
| GABRR3 | 0.121706324 | 0.772339412 | 0.867431116 | NS | 0.112191803 |
| OR5AC2 | 0.126402048 | 0.897823673 | NA | NS | 0.046808948 |
| OR5H1 | 0.43947316 | 0.421014914 | NA | NS | 0.375702519 |
| OR5H14 | 0.748460661 | 0.113513599 | 0.332228782 | NS | 0.944952108 |
| OR5H15 | 0.688265422 | 0.28110722 | NA | NS | 0.551128 |
| OR5H6 | 0.530665794 | 0.442859577 | NA | NS | 0.353733959 |
| OR5H2 | 0.575390424 | 0.282550266 | NA | NS | 0.548904279 |
| OR5K4 | 0.184984626 | 0.790637348 | NA | NS | 0.102022674 |
| OR5K3 | 0.915029139 | 0.162931721 | NA | NS | 0.787994354 |
| OR5K1 | 0.208924576 | 0.507938103 | NA | NS | 0.294189207 |
| OR5K2 | -0.090452901 | 0.587695695 | 0.746210944 | NS | 0.230847491 |
| CLDND1 | -0.09387 | 0.069774684 | 0.270202877 | NS | 1.156302121 |
| GPR15 | -0.120660145 | 0.433424795 | 0.629801069 | NS | 0.363086248 |
| CPOX | -0.251260258 | 7.06E-04 | 0.043826722 | Down | 3.151108053 |
| ST3GAL6 | 0.09132863 | 0.313703689 | 0.533319352 | NS | 0.503480374 |
| DCBLD2 | 0.09941021 | 0.321387141 | 0.53990329 | NS | 0.492971504 |
| COL8A1 | 0.24332118 | 0.532160991 | 0.705134865 | NS | 0.273956964 |
| CMSS1 | 0.047766854 | 0.524575082 | 0.699861268 | NS | 0.280192343 |
| FILIP1L | 0.249447928 | 0.011434031 | 0.131302419 | Up | 1.941800635 |
| TBC1D23 | -0.126784195 | 0.014605947 | 0.144746239 | Down | 1.835470293 |
| NIT2 | 0.028408666 | 0.543841679 | 0.713285597 | NS | 0.264527512 |
| TOMM70 | -0.185408562 | 0.013784158 | 0.141119705 | Down | 1.860619767 |
| LNP1 | -0.071611534 | 0.559574235 | 0.724472223 | NS | 0.25214229 |
| TMEM45A | 0.201492589 | 0.489201665 | 0.673825496 | NS | 0.310512074 |
| ADGRG7 | 0.150984768 | 0.779966826 | NA | NS | 0.107923869 |
| TFG | -1.54E-05 | 0.999748615 | 0.999802152 | NS | 1.09E-04 |
| ABI3BP | 0.190833256 | 0.077455205 | 0.281659932 | NS | 1.110949394 |
| IMPG2 | 0.090890094 | 0.243385654 | 0.471136696 | NS | 0.613705024 |
| SENP7 | -0.070262614 | 0.170767137 | 0.399072344 | NS | 0.767595703 |
| TRMT10C | -0.178365443 | 0.136041627 | 0.360187901 | NS | 0.866328182 |
| PCNP | -0.158819892 | 0.069683441 | 0.270202877 | NS | 1.156870411 |
| ZBTB11 | -0.100520777 | 0.016357394 | 0.151112662 | Down | 1.786285873 |
| RPL24 | -0.271836083 | 0.059124953 | 0.25366798 | NS | 1.228229191 |
| CEP97 | -0.073676375 | 0.18072996 | 0.41058204 | NS | 0.742969847 |
| NXPE3 | -0.069040517 | 0.339299425 | 0.554994713 | NS | 0.469416877 |
| NFKBIZ | -0.013385555 | 0.857658496 | 0.91967171 | NS | 0.066685606 |
| ZPLD1 | 0.313692388 | 0.422562828 | 0.622069963 | NS | 0.37410871 |
| ALCAM | -0.157402109 | 0.047924133 | 0.232603517 | Down | 1.319445733 |
| CBLB | 0.087705156 | 0.255497892 | 0.482391816 | NS | 0.592612678 |
| CCDC54 | 0.389446042 | 0.415947228 | NA | NS | 0.380961766 |
| BBX | -0.043425605 | 0.317675281 | 0.537032996 | NS | 0.498016577 |
| CD47 | 0.031208958 | 0.488292988 | 0.673019139 | NS | 0.311319512 |
| IFT57 | -0.164369207 | 0.070096756 | 0.270854716 | NS | 1.154302081 |
| HHLA2 | 0.378471363 | 0.109551894 | 0.327670599 | NS | 0.960380109 |
| MYH15 | 0.364959273 | 0.17531684 | 0.40462625 | NS | 0.756176365 |
| CIP2A | -0.027544688 | 0.713895926 | 0.830859056 | NS | 0.146365096 |
| DZIP3 | -0.171385371 | 0.114378577 | 0.332930626 | NS | 0.941655311 |
| RETNLB | 0.763247893 | 0.150246345 | NA | NS | 0.823196084 |
| TRAT1 | -0.254180239 | 0.038893026 | 0.214216116 | Down | 1.410128271 |
| GUCA1C | 0.869431116 | 0.148710317 | NA | NS | 0.827658899 |
| MORC1 | 0.065834316 | 0.805206013 | 0.887901589 | NS | 0.094092991 |
| C3orf85 | 0.373788613 | 0.468707716 | NA | NS | 0.329097897 |
| DPPA2 | 0.375521144 | 0.490985974 | NA | NS | 0.308930915 |
| DPPA4 | 0.01728017 | 0.934163478 | 0.963746654 | NS | 0.029577116 |
| NECTIN3 | -0.104848059 | 0.339603443 | 0.555293684 | NS | 0.469027915 |
| CD96 | -0.037167849 | 0.589500696 | 0.747377833 | NS | 0.229515678 |
| ZBED2 | 0.23719154 | 0.103132683 | 0.319138589 | NS | 0.986603686 |
| PLCXD2 | -0.039420481 | 0.541289664 | 0.711383242 | NS | 0.266570266 |
| PHLDB2 | -0.043270066 | 0.561013197 | 0.725489421 | NS | 0.251026923 |
| ABHD10 | -0.203003229 | 0.026728707 | 0.184769347 | Down | 1.573022049 |
| TAGLN3 | 0.018245985 | 0.969922924 | NA | NS | 0.013262776 |
| TMPRSS7 | 0.389239656 | 0.348243971 | 0.562612118 | NS | 0.458116393 |
| C3orf52 | 0.309686368 | 0.092835256 | 0.305256715 | NS | 1.032287063 |
| GCSAM | -0.131910651 | 0.317840328 | 0.537121802 | NS | 0.497791 |
| SLC9C1 | -0.389272975 | 0.188825383 | NA | NS | 0.723939625 |
| CD200 | 0.133063921 | 0.323591953 | 0.54228177 | NS | 0.490002287 |
| BTLA | 0.031592746 | 0.744163766 | 0.850016886 | NS | 0.12833148 |
| ATG3 | -0.078610127 | 0.310159906 | 0.530296194 | NS | 0.508414343 |
| SLC35A5 | -0.063788079 | 0.267819224 | 0.493768787 | NS | 0.572158252 |
| CCDC80 | 0.293046872 | 0.067346352 | 0.267261507 | NS | 1.171685923 |
| CD200R1L | 0.177123096 | 0.560935722 | NA | NS | 0.251086902 |
| CD200R1 | -0.032414039 | 0.71432053 | 0.831075885 | NS | 0.146106868 |
| GTPBP8 | -0.080393588 | 0.217379594 | 0.447016923 | NS | 0.662781226 |
| NEPRO | -0.101375204 | 0.045343554 | 0.227426386 | Down | 1.343484443 |
| BOC | 0.591200022 | 0.036566493 | 0.208527349 | Up | 1.436916684 |
| CFAP44 | 0.02395594 | 0.74165156 | 0.848546216 | NS | 0.129800086 |
| SPICE1 | 0.145818273 | 0.040843072 | 0.218105862 | Up | 1.388881595 |
| SIDT1 | 0.067648811 | 0.254605061 | 0.48140404 | NS | 0.594132968 |
| USF3 | 0.100932604 | 0.189883361 | 0.419275561 | NS | 0.72151309 |
| NAA50 | -0.115942344 | 0.02407266 | 0.175739247 | Down | 1.618475915 |
| ATP6V1A | -0.004687586 | 0.96422515 | 0.979940943 | NS | 0.015821545 |
| GRAMD1C | -0.002751541 | 0.983363172 | 0.99010742 | NS | 0.007286061 |
| ZDHHC23 | -0.010739093 | 0.868699744 | 0.925923112 | NS | 0.061130307 |
| CCDC191 | 0.006737125 | 0.906228663 | 0.94801213 | NS | 0.042762206 |
| QTRT2 | -0.037732861 | 0.330300609 | 0.547441347 | NS | 0.481090626 |
| DRD3 | -0.043217616 | 0.86063039 | 0.92129369 | NS | 0.065183322 |
| ZNF80 | 0.009822482 | 0.925349473 | 0.959164117 | NS | 0.033694218 |
| TIGIT | -0.127008377 | 0.163382078 | 0.391129522 | NS | 0.786795586 |
| ZBTB20 | 0.101557951 | 0.136324693 | 0.360770525 | NS | 0.865425472 |
| GAP43 | 0.338230515 | 0.486756317 | NA | NS | 0.312688404 |
| LSAMP | 0.4322033 | 0.326348479 | 0.544632642 | NS | 0.486318407 |
| IGSF11 | 0.496056222 | 0.052811803 | 0.242846705 | NS | 1.277269005 |
| TEX55 | 0.656475936 | 0.053819012 | NA | NS | 1.269064278 |
| UPK1B | 0.594120739 | 0.016001076 | 0.149941342 | Up | 1.795850814 |
| B4GALT4 | 0.153638285 | 0.014981917 | 0.146818936 | Up | 1.824432626 |
| ARHGAP31 | 0.094132607 | 0.249335284 | 0.476497659 | NS | 0.603216259 |
| TMEM39A | 0.011795264 | 0.73134152 | 0.84106463 | NS | 0.13587977 |
| POGLUT1 | -0.107252738 | 0.057998072 | 0.252025231 | NS | 1.236586444 |
| TIMMDC1 | -0.214690577 | 0.005115036 | 0.09835814 | Down | 2.291151341 |
| CD80 | -0.013841041 | 0.883811278 | 0.934370959 | NS | 0.053640461 |
| ADPRH | -0.144895933 | 0.138936417 | 0.363222438 | NS | 0.857183907 |
| PLA1A | 0.428485766 | 0.2528672 | NA | NS | 0.5971075 |
| POPDC2 | -0.093367071 | 0.377125253 | 0.587195697 | NS | 0.423514385 |
| COX17 | -0.065733666 | 0.441689599 | 0.636239712 | NS | 0.354882828 |
| CFAP91 | 0.582911047 | 0.11507384 | 0.333808878 | NS | 0.939023393 |
| NR1I2 | 0.311624268 | 0.215976538 | 0.445361714 | NS | 0.665593424 |
| GSK3B | 0.165608516 | 0.024936275 | 0.178924423 | Up | 1.603168424 |
| GPR156 | 0.457041505 | 0.234089102 | NA | NS | 0.630618804 |
| LRRC58 | -0.077800942 | 0.183303732 | 0.413557489 | NS | 0.736828693 |
| FSTL1 | -0.204423848 | 0.21994988 | 0.449237703 | NS | 0.657676271 |
| NDUFB4 | -0.14957947 | 0.050446537 | 0.237672619 | NS | 1.297168638 |
| HGD | -0.140622216 | 0.187982724 | 0.41741132 | NS | 0.725882061 |
| RABL3 | -0.030122302 | 0.656900061 | 0.79397469 | NS | 0.182500698 |
| GTF2E1 | -0.072644742 | 0.308859944 | 0.529238414 | NS | 0.510238412 |
| STXBP5L | 0.42480415 | 0.223617144 | 0.452317462 | NS | 0.650494903 |
| POLQ | -0.202157389 | 0.201289222 | 0.431035601 | NS | 0.696179479 |
| ARGFX | 0.830569758 | 0.091501049 | 0.303287164 | NS | 1.038573929 |
| FBXO40 | 0.302881766 | 0.004508816 | 0.094130269 | Up | 2.345937481 |
| HCLS1 | -0.119420943 | 0.089485187 | 0.299870767 | NS | 1.04824885 |
| GOLGB1 | -0.049961811 | 0.419075568 | 0.619391222 | NS | 0.377707658 |
| IQCB1 | -0.041935485 | 0.508291875 | 0.687821019 | NS | 0.293886832 |
| EAF2 | -0.237648109 | 0.017102583 | 0.153661571 | Down | 1.766938305 |
| SLC15A2 | 0.098828265 | 0.234219757 | 0.46236722 | NS | 0.630376474 |
| ILDR1 | 0.450502823 | 0.040924121 | 0.218105862 | Up | 1.388020645 |
| CD86 | -0.267054478 | 0.023136598 | 0.173122445 | Down | 1.635700502 |
| CASR | 0.646964785 | 0.070883626 | 0.271922411 | NS | 1.149454077 |
| CSTA | -0.200445694 | 0.338147559 | 0.554190311 | NS | 0.470893743 |
| MIX23 | 0.020126637 | 0.85548054 | 0.918743978 | NS | 0.067789865 |
| FAM162A | -0.038949537 | 0.574039711 | 0.735427195 | NS | 0.241058063 |
| WDR5B | 0.011510804 | 0.886578709 | 0.935950794 | NS | 0.052282702 |
| KPNA1 | -0.006576393 | 0.893919809 | 0.940563971 | NS | 0.048701439 |
| PARP9 | 0.163004504 | 0.107038965 | 0.323901028 | NS | 0.9704581 |
| DTX3L | 0.22753156 | 0.001753809 | 0.065523229 | Up | 2.756017647 |
| PARP15 | 0.083987577 | 0.378045042 | 0.588048799 | NS | 0.422456454 |
| PARP14 | 0.099667814 | 0.342338929 | 0.557588092 | NS | 0.465543712 |
| HSPBAP1 | 0.051960297 | 0.432086068 | 0.628973186 | NS | 0.364429737 |
| SLC49A4 | 0.227299035 | 0.009216214 | 0.121305834 | Up | 2.035447455 |
| SEMA5B | 0.754716145 | 0.066481097 | 0.265925581 | NS | 1.177301825 |
| PDIA5 | -0.217588374 | 0.02021942 | 0.164714539 | Down | 1.694231303 |
| SEC22A | -0.15087794 | 0.003433382 | 0.085194315 | Down | 2.464277839 |
| ADCY5 | 0.501054695 | 0.019236539 | 0.161080521 | Up | 1.715873053 |
| HACD2 | 0.008882591 | 0.88903292 | 0.93735026 | NS | 0.051082157 |
| MYLK | -0.039636716 | 0.689974908 | 0.815703352 | NS | 0.161166703 |
| CCDC14 | -0.15954789 | 0.03267329 | 0.199595539 | Down | 1.485807139 |
| ROPN1 | 0.394272963 | 0.415062495 | NA | NS | 0.381886508 |
| KALRN | 0.360219217 | 0.074820381 | 0.277636607 | NS | 1.125980083 |
| UMPS | -0.109663325 | 0.087862396 | 0.297622333 | NS | 1.056196957 |
| ITGB5 | 0.04961616 | 0.747654084 | 0.851783936 | NS | 0.12629929 |
| MUC13 | 0.61589559 | 0.170660248 | 0.399000229 | NS | 0.767867628 |
| HEG1 | 0.113502007 | 0.117383584 | 0.337318365 | NS | 0.930392636 |
| SLC12A8 | 0.526488133 | 0.074322668 | 0.276837611 | NS | 1.12887871 |
| ZNF148 | -0.025087295 | 0.657377032 | 0.794202854 | NS | 0.182185474 |
| SNX4 | -0.238987928 | 0.038056918 | 0.212321445 | Down | 1.419566389 |
| OSBPL11 | 0.091346895 | 0.157900186 | 0.385290564 | NS | 0.80161736 |
| ROPN1B | 0.355977568 | 0.03554592 | 0.206049964 | Up | 1.449210246 |
| SLC41A3 | -0.078192613 | 0.304694719 | 0.525971545 | NS | 0.516135073 |
| ALDH1L1 | 0.565533949 | 0.199626083 | 0.429199827 | NS | 0.699782715 |
| KLF15 | 0.722693626 | 0.071396917 | NA | NS | 1.146320542 |
| CFAP100 | 0.400684319 | 0.053142945 | 0.243367273 | NS | 1.27455438 |
| ZXDC | 0.030749226 | 0.591313708 | 0.748315552 | NS | 0.228182053 |
| UROC1 | 0.917573878 | 0.038068922 | NA | Up | 1.419429418 |
| CHST13 | 0.899362637 | 3.45E-05 | 0.007962653 | Up | 4.462131961 |
| C3orf22 | 0.783967945 | 0.001338594 | NA | Up | 2.873351044 |
| TXNRD3 | 0.169625302 | 0.262719435 | 0.488909438 | NS | 0.580507799 |
| CHCHD6 | -0.074089062 | 0.39753271 | 0.60261412 | NS | 0.40062713 |
| PLXNA1 | 0.250894269 | 0.020768853 | 0.167356721 | Up | 1.682587484 |
| C3orf56 | 0.537896167 | 0.28342049 | NA | NS | 0.547568756 |
| PRR20G | 0.798421509 | 0.130179749 | NA | NS | 0.88545657 |
| TPRA1 | -0.049150122 | 0.409089199 | 0.612150332 | NS | 0.388181987 |
| MCM2 | -0.107843146 | 0.288162692 | 0.511321729 | NS | 0.540362247 |
| PODXL2 | 0.291947898 | 0.011844531 | 0.133168118 | Up | 1.92648212 |
| ABTB1 | 0.185257031 | 0.184158616 | 0.414362912 | NS | 0.734807958 |
| MGLL | 0.218648011 | 0.144526394 | 0.369690386 | NS | 0.840052833 |
| KBTBD12 | 0.461063308 | 0.070342437 | 0.271115037 | NS | 1.152782588 |
| SEC61A1 | -0.059950461 | 0.257515941 | 0.484404935 | NS | 0.589195882 |
| RUVBL1 | -0.136080391 | 0.051219115 | 0.239227656 | NS | 1.290567929 |
| EEFSEC | 0.103078457 | 0.421415883 | 0.621298082 | NS | 0.3752891 |
| DNAJB8 | 0.368503723 | 0.440974433 | NA | NS | 0.355586589 |
| GATA2 | -0.159490543 | 0.449785585 | 0.643129524 | NS | 0.346994468 |
| RPN1 | -0.165279772 | 0.039469575 | 0.214966042 | Down | 1.403737549 |
| RAB7A | 0.381020074 | 1.20E-04 | 0.017390253 | Up | 3.920910432 |
| ACAD9 | -0.04295328 | 0.339620934 | 0.555293684 | NS | 0.469005548 |
| CFAP92 | 0.090661943 | 0.334876261 | 0.551352172 | NS | 0.475115638 |
| EFCC1 | 0.817151408 | 0.024440383 | NA | Up | 1.611891989 |
| GP9 | 0.32133459 | 0.032918119 | 0.200078102 | Up | 1.48256499 |
| RAB43 | 0.107092261 | 0.479887946 | 0.666842078 | NS | 0.318860159 |
| ISY1 | -0.069049306 | 0.319162325 | 0.538251515 | NS | 0.49598838 |
| CNBP | -0.177640772 | 0.015236258 | 0.147394235 | Down | 1.817121676 |
| COPG1 | -0.147885666 | 0.042502572 | 0.221095683 | Down | 1.371584787 |
| HMCES | -0.22136812 | 0.002750691 | 0.079323886 | Down | 2.560558139 |
| H1-10 | 0.197315124 | 0.061260172 | 0.257380382 | NS | 1.212821787 |
| EFCAB12 | 0.404442732 | 0.045525937 | 0.227848562 | Up | 1.341741103 |
| IFT122 | 0.009756419 | 0.925033552 | 0.959016206 | NS | 0.033842515 |
| MBD4 | -0.11668023 | 0.049578996 | 0.235572235 | Down | 1.30470227 |
| RHO | 0.195295196 | 0.693408481 | NA | NS | 0.159010851 |
| H1-8 | 1.104199676 | 0.034409977 | NA | Up | 1.463315618 |
| PLXND1 | 0.024110155 | 0.8765094 | 0.930722287 | NS | 0.057243422 |
| TMCC1 | 0.051376264 | 0.493371985 | 0.677207384 | NS | 0.306825515 |
| TRH | 0.90030758 | 0.092761033 | NA | NS | 1.032634424 |
| ALG1L2 | 0.45315717 | 0.019398252 | 0.161607408 | Up | 1.712237405 |
| COL6A5 | 0.645437653 | 0.099325948 | 0.314301595 | NS | 1.002937283 |
| COL6A6 | 0.251206437 | 0.057270918 | 0.250979442 | NS | 1.242065856 |
| PIK3R4 | -0.078553699 | 0.048804976 | 0.234117506 | NS | 1.311535897 |
| ATP2C1 | -0.047890067 | 0.166014375 | 0.393995982 | NS | 0.779854304 |
| ASTE1 | -0.04155562 | 0.473992678 | 0.662196078 | NS | 0.324228367 |
| NEK11 | 0.167564397 | 0.039401704 | 0.214800721 | Up | 1.404484995 |
| NUDT16 | 0.05833648 | 0.454300016 | 0.64659617 | NS | 0.342657248 |
| MRPL3 | -0.22930976 | 0.030554298 | 0.194040657 | Down | 1.514927694 |
| CPNE4 | 0.270887526 | 0.360551262 | 0.573327689 | NS | 0.44303298 |
| ACP3 | 0.181528112 | 0.060079888 | 0.255800259 | NS | 1.221270886 |
| DNAJC13 | 0.090801751 | 0.058252146 | 0.252197542 | NS | 1.23468807 |
| ACAD11 | 0.108283351 | 0.186405327 | 0.41588454 | NS | 0.729541681 |
| NPHP3-ACAD11 | 0.367905135 | 0.108231439 | NA | NS | 0.965646567 |
| ACKR4 | 0.289453804 | 0.008986983 | 0.120055109 | Up | 2.046386073 |
| UBA5 | -0.009567647 | 0.866581563 | 0.924552826 | NS | 0.062190555 |
| NPHP3 | -0.055372841 | 0.362730978 | 0.575276172 | NS | 0.440415353 |
| TMEM108 | 0.42209056 | 0.085496129 | 0.29386648 | NS | 1.068053546 |
| BFSP2 | 0.20058499 | 0.40362964 | 0.60743385 | NS | 0.394016949 |
| CDV3 | -0.194895074 | 0.001036286 | 0.052796552 | Down | 2.984520385 |
| TOPBP1 | -0.06618266 | 0.099625771 | 0.314750917 | NS | 1.001628306 |
| TF | 0.486021176 | 0.101392853 | 0.317114382 | NS | 0.993992656 |
| SRPRB | -0.188146226 | 0.022909505 | 0.172363169 | Down | 1.639984292 |
| RAB6B | 0.021031597 | 0.84136722 | 0.910589863 | NS | 0.075014412 |
| SLCO2A1 | 0.643031725 | 0.103535524 | 0.319328319 | NS | 0.984910615 |
| RYK | -0.064197033 | 0.191956336 | 0.420934764 | NS | 0.716797549 |
| AMOTL2 | 0.646786704 | 0.139017368 | 0.363272092 | NS | 0.856930939 |
| ANAPC13 | 0.029644273 | 0.735881659 | 0.844177607 | NS | 0.133192021 |
| CEP63 | 0.216184775 | 0.047380702 | 0.23126872 | Up | 1.324398509 |
| KY | -0.010558988 | 0.947702412 | 0.97158694 | NS | 0.023328014 |
| EPHB1 | 0.153428529 | 0.288721946 | 0.511843768 | NS | 0.539520204 |
| PPP2R3A | 0.142938603 | 0.053251035 | 0.243707535 | NS | 1.273671943 |
| MSL2 | 0.051496433 | 0.183056783 | 0.413359339 | NS | 0.737414175 |
| PCCB | -0.10616018 | 0.028688998 | 0.189026296 | Down | 1.542284625 |
| STAG1 | -0.080029084 | 0.035552845 | 0.206049964 | NS | 1.449125642 |
| SLC35G2 | 0.0944368 | 0.383478213 | 0.592439708 | NS | 0.416259305 |
| NCK1 | -0.111343284 | 0.081770617 | 0.288293278 | NS | 1.087402723 |
| IL20RB | 0.125730077 | 0.324541582 | 0.543083204 | NS | 0.488729651 |
| SOX14 | 1.097019565 | 0.049283078 | NA | Up | 1.307302173 |
| CLDN18 | 0.608107337 | 0.039951906 | 0.215796828 | Up | 1.398462497 |
| DZIP1L | 0.322624297 | 0.179780588 | 0.409922597 | NS | 0.745257203 |
| A4GNT | 0.212728453 | 0.653554373 | NA | NS | 0.184718275 |
| DBR1 | -0.139447683 | 0.035104213 | 0.205352924 | Down | 1.454640753 |
| ARMC8 | -0.093429113 | 0.055014613 | 0.247679315 | NS | 1.259521936 |
| NME9 | 0.233305664 | 0.01728793 | 0.154323671 | Up | 1.762257006 |
| MRAS | 0.096527937 | 0.469178725 | 0.658326224 | NS | 0.328661689 |
| ESYT3 | 0.402769255 | 0.055458411 | 0.248604264 | NS | 1.25603258 |
| CEP70 | -0.415830887 | 0.006523067 | 0.108176159 | Down | 2.185548174 |
| FAIM | 0.022782638 | 0.796894816 | 0.883073092 | NS | 0.098598998 |
| PIK3CB | 0.136274585 | 0.030541419 | 0.194040657 | Up | 1.515110795 |
| FOXL2 | 1.364690112 | 0.0078283 | NA | Up | 2.106332539 |
| FOXL2NB | 0.485445173 | 0.282798134 | NA | NS | 0.548523461 |
| PRR23A | 0.825772974 | 0.146134824 | NA | NS | 0.835246281 |
| MRPS22 | -0.107850685 | 0.104245847 | 0.320245132 | NS | 0.981941238 |
| PRR23B | 1.055149591 | 0.043754669 | NA | Up | 1.358975602 |
| PRR23C | 0.514776824 | 0.221504038 | NA | NS | 0.654618353 |
| COPB2 | -0.118830862 | 0.055454094 | 0.248604264 | NS | 1.25606639 |
| RBP2 | 0.614323474 | 0.004203865 | NA | Up | 2.376351215 |
| RBP1 | 0.204821059 | 0.516520381 | 0.694129196 | NS | 0.286912538 |
| NMNAT3 | 0.063581159 | 0.614304629 | 0.764059058 | NS | 0.211616212 |
| CLSTN2 | 0.493930412 | 0.226439448 | 0.45510454 | NS | 0.645047913 |
| TRIM42 | 0.257102725 | 0.644039913 | NA | NS | 0.191087218 |
| SLC25A36 | 0.031459039 | 0.602644968 | 0.756137351 | NS | 0.219938465 |
| SPSB4 | 0.687505036 | 0.124608959 | NA | NS | 0.904450732 |
| PXYLP1 | 0.112911422 | 0.155281995 | 0.382558975 | NS | 0.808878899 |
| ZBTB38 | -0.114260949 | 0.087838894 | 0.29758674 | NS | 1.056313139 |
| RASA2 | -0.061304009 | 0.239733182 | 0.467822882 | NS | 0.62027185 |
| RNF7 | -0.160590319 | 0.067029485 | 0.266826765 | NS | 1.173734117 |
| GRK7 | 0.62285497 | 0.072357733 | 0.27394225 | NS | 1.140515046 |
| ATP1B3 | -0.051239555 | 0.534436313 | 0.706800279 | NS | 0.272104041 |
| TFDP2 | -0.167372484 | 0.098849722 | 0.313727317 | NS | 1.00502455 |
| GK5 | 0.032606318 | 0.626405516 | 0.772158843 | NS | 0.203144426 |
| XRN1 | -0.013955069 | 0.862076232 | 0.922196766 | NS | 0.064454328 |
| ATR | -0.053812931 | 0.295970818 | 0.517745305 | NS | 0.528751108 |
| PLS1 | -0.085677536 | 0.491323027 | 0.675559048 | NS | 0.308632881 |
| TRPC1 | 0.006938177 | 0.931520778 | 0.962676894 | NS | 0.030807453 |
| PCOLCE2 | 0.436573578 | 0.175346127 | 0.404653064 | NS | 0.756103822 |
| PAQR9 | -0.562920919 | 0.030903412 | 0.195241361 | Down | 1.509993572 |
| U2SURP | -0.10410406 | 0.023390765 | 0.173829337 | Down | 1.630955575 |
| CHST2 | -0.157746027 | 0.151426362 | 0.377938812 | NS | 0.819798511 |
| SLC9A9 | -0.011106525 | 0.820966269 | 0.898016406 | NS | 0.085674687 |
| DIPK2A | -0.195691448 | 0.026294269 | 0.183092929 | Down | 1.580138892 |
| PLOD2 | 0.005474163 | 0.97585679 | 0.986116904 | NS | 0.010613912 |
| PLSCR4 | -0.066573566 | 0.762380788 | 0.861501199 | NS | 0.117828056 |
| PLSCR2 | 0.405230642 | 0.018928742 | 0.159670733 | Up | 1.722878257 |
| PLSCR1 | 0.289978774 | 0.023157712 | 0.173122445 | Up | 1.635304346 |
| PLSCR5 | 0.305779614 | 0.611574998 | NA | NS | 0.213550277 |
| ZIC4 | 0.400837042 | 0.382934655 | 0.59214603 | NS | 0.416875329 |
| ZIC1 | 0.745965877 | 0.121462932 | NA | NS | 0.91555624 |
| AGTR1 | 0.601182202 | 0.060737179 | NA | NS | 1.216545383 |
| CPB1 | 0.707392798 | 0.007696036 | 0.113974683 | Up | 2.113732893 |
| CPA3 | -0.394654065 | 0.10809961 | 0.325834237 | NS | 0.966175872 |
| GYG1 | 0.325489973 | 6.72E-04 | 0.042869495 | Up | 3.172630592 |
| HLTF | -0.286266579 | 0.00109541 | 0.05356245 | Down | 2.960423204 |
| HPS3 | -0.097095968 | 0.071319903 | 0.272521361 | NS | 1.146789255 |
| CP | 0.221140563 | 0.041396914 | 0.219308841 | Up | 1.383032034 |
| TM4SF18 | 0.447872465 | 0.350986668 | NA | NS | 0.45470938 |
| TM4SF1 | 0.57470594 | 0.0714248 | NA | NS | 1.14615097 |
| TM4SF4 | 0.360780217 | 0.503149331 | NA | NS | 0.298303101 |
| WWTR1 | 0.204009097 | 0.212282469 | 0.442052473 | NS | 0.67308587 |
| COMMD2 | -0.128504366 | 0.134722037 | 0.358293518 | NS | 0.870561359 |
| ANKUB1 | 0.092050491 | 0.390572807 | 0.59752734 | NS | 0.408297998 |
| RNF13 | -0.095120684 | 0.239779779 | 0.467834086 | NS | 0.620187444 |
| PFN2 | 0.029445395 | 0.6690304 | 0.802416036 | NS | 0.174554148 |
| TSC22D2 | 0.128834624 | 0.00574816 | 0.102208347 | Up | 2.240471182 |
| SERP1 | -0.237012139 | 0.01311396 | 0.137453511 | Down | 1.882266132 |
| EIF2A | -0.287106209 | 0.003588496 | 0.086559417 | Down | 2.445087579 |
| SELENOT | -0.045123546 | 0.651017637 | 0.789620058 | NS | 0.186407245 |
| ERICH6 | 0.311619469 | 0.37150589 | NA | NS | 0.430034296 |
| SIAH2 | -0.341380879 | 0.076548899 | 0.280416761 | NS | 1.116061053 |
| MINDY4B | 0.616050894 | 0.136598178 | NA | NS | 0.864555092 |
| CLRN1 | 0.308209623 | 0.232235875 | 0.460530436 | NS | 0.634070691 |
| MED12L | 0.090668175 | 0.346292632 | 0.561114608 | NS | 0.460556748 |
| GPR171 | -0.144012818 | 0.161024783 | 0.388764128 | NS | 0.793107277 |
| P2RY14 | 0.228090371 | 0.167870071 | 0.39601577 | NS | 0.775026726 |
| GPR87 | 0.18841843 | 0.630858925 | NA | NS | 0.200067748 |
| P2RY13 | 0.351816962 | 0.007688748 | 0.113974683 | Up | 2.114144358 |
| P2RY12 | -0.273853651 | 0.023937676 | 0.175295276 | Down | 1.620918008 |
| IGSF10 | 0.292197211 | 0.314885751 | 0.534344507 | NS | 0.501846992 |
| AADAC | 0.980560009 | 0.021463558 | NA | Up | 1.668298281 |
| SUCNR1 | -0.046377813 | 0.731398818 | 0.84106463 | NS | 0.135845746 |
| MBNL1 | -0.057254728 | 0.169039359 | 0.397264176 | NS | 0.772012163 |
| P2RY1 | -0.153906798 | 0.085384592 | 0.293571225 | NS | 1.068620492 |
| RAP2B | -0.181785042 | 0.002205605 | 0.072832159 | Down | 2.656472323 |
| DHX36 | -0.016645819 | 0.713158834 | 0.830448491 | NS | 0.146813734 |
| GPR149 | 0.444524884 | 0.367249105 | NA | NS | 0.435039253 |
| MME | -0.007437655 | 0.969320325 | 0.982823994 | NS | 0.013532681 |
| STRIT1 | 0.672997424 | 0.273161864 | NA | NS | 0.563579933 |
| PLCH1 | 0.331694056 | 0.105889923 | 0.322185601 | NS | 0.975145367 |
| C3orf33 | 0.045949941 | 0.82370779 | NA | NS | 0.084226827 |
| SLC33A1 | -0.086788972 | 0.086844202 | 0.296188519 | NS | 1.061259173 |
| GMPS | -0.005916501 | 0.928308961 | 0.960649398 | NS | 0.032307457 |
| KCNAB1 | 0.155810576 | 0.043631637 | 0.224086881 | Up | 1.360198496 |
| SSR3 | -0.142190785 | 0.104898866 | 0.320789668 | NS | 0.979229208 |
| TIPARP | -0.065160965 | 0.290228756 | 0.513109384 | NS | 0.53725956 |
| LEKR1 | 0.090707255 | 0.505406811 | 0.685915662 | NS | 0.296358909 |
| CCNL1 | 7.50E-04 | 0.989096848 | 0.993652498 | NS | 0.004761182 |
| VEPH1 | 0.047313867 | 0.691798772 | 0.816889686 | NS | 0.160020213 |
| PTX3 | -0.388772887 | 0.009538929 | 0.122730647 | Down | 2.020500383 |
| SHOX2 | 0.706032677 | 0.035893595 | 0.207166105 | Up | 1.444983038 |
| RSRC1 | -0.13973772 | 0.048985322 | 0.234600973 | Down | 1.30993403 |
| MLF1 | -0.077965539 | 0.654320386 | 0.792221749 | NS | 0.184209549 |
| GFM1 | -0.168326327 | 0.001773749 | 0.065838567 | Down | 2.751107878 |
| LXN | 0.028348516 | 0.693084575 | 0.817696744 | NS | 0.159213767 |
| RARRES1 | 0.199356406 | 0.091785369 | 0.303591642 | NS | 1.03722654 |
| MFSD1 | -0.123597407 | 0.082345805 | 0.289245956 | NS | 1.084358518 |
| IQCJ-SCHIP1 | 1.304429752 | 0.005495412 | NA | Up | 2.259999725 |
| IQCJ | -0.279003496 | 0.572315474 | NA | NS | 0.242364512 |
| SCHIP1 | 0.296497593 | 0.412004293 | NA | NS | 0.385098259 |
| IL12A | 0.244877179 | 0.147725218 | 0.373669421 | NS | 0.830545361 |
| C3orf80 | 0.7346724 | 0.057185748 | NA | NS | 1.242712191 |
| IFT80 | -0.149095394 | 0.075231535 | 0.278206739 | NS | 1.123600077 |
| SMC4 | -0.176314335 | 0.091598154 | 0.303366728 | NS | 1.038113279 |
| TRIM59 | -0.002069046 | 0.974591621 | 0.985403414 | NS | 0.011177327 |
| KPNA4 | -0.026090767 | 0.544415384 | 0.713447077 | NS | 0.264069611 |
| ARL14 | -0.09060898 | 0.830961236 | NA | NS | 0.080419235 |
| PPM1L | 0.098455033 | 0.201066292 | 0.430638757 | NS | 0.69666073 |
| B3GALNT1 | 0.334802493 | 0.04542956 | 0.227515368 | Up | 1.342661472 |
| NMD3 | -0.157020097 | 0.148741922 | 0.374763795 | NS | 0.82756661 |
| SPTSSB | -0.234977368 | 0.154753202 | 0.381995886 | NS | 0.810360357 |
| OTOL1 | 0.487264173 | 0.417120101 | NA | NS | 0.379738881 |
| SI | 0.341243962 | 0.468423072 | 0.657909015 | NS | 0.329361722 |
| SLITRK3 | 0.427328966 | 0.431806407 | NA | NS | 0.364710918 |
| BCHE | 0.474805429 | 0.376081005 | NA | NS | 0.424718602 |
| ZBBX | 0.413555663 | 0.381567916 | NA | NS | 0.41842815 |
| SERPINI2 | 0.255371808 | 0.086448664 | 0.295484168 | NS | 1.063241716 |
| WDR49 | -0.022888431 | 0.848310167 | 0.914646177 | NS | 0.071445328 |
| PDCD10 | -0.184527599 | 0.108217432 | 0.325834237 | NS | 0.965702775 |
| SERPINI1 | -0.121395395 | 0.25441871 | 0.481360925 | NS | 0.594450954 |
| GOLIM4 | -0.254525961 | 0.019890777 | 0.163677883 | Down | 1.701348252 |
| MECOM | 0.376502686 | 0.146961097 | 0.37264175 | NS | 0.832797616 |
| ACTRT3 | 0.594364574 | 0.018093849 | NA | Up | 1.742469036 |
| MYNN | -0.114900788 | 0.10537925 | 0.321400397 | NS | 0.977244898 |
| LRRC34 | 0.031238433 | 0.683360868 | 0.811486602 | NS | 0.165349894 |
| LRRIQ4 | 0.30494895 | 0.433102786 | NA | NS | 0.363409023 |
| LRRC31 | 0.381022623 | 0.474317314 | NA | NS | 0.323931022 |
| SAMD7 | 0.433805039 | 0.326857556 | NA | NS | 0.485641471 |
| SEC62 | -0.260087155 | 0.044073949 | 0.224627854 | Down | 1.355818037 |
| GPR160 | -0.126189499 | 0.20360542 | 0.433522809 | NS | 0.691210665 |
| PHC3 | -0.01984709 | 0.769647607 | 0.865739766 | NS | 0.113708077 |
| PRKCI | -0.183890651 | 0.006069808 | 0.104205953 | Down | 2.216825051 |
| SKIL | 0.001092657 | 0.988166785 | 0.993039952 | NS | 0.005169748 |
| CLDN11 | 0.689677636 | 0.100761634 | NA | NS | 0.996704798 |
| SLC7A14 | 0.515425553 | 0.227389591 | 0.45605363 | NS | 0.64322942 |
| RPL22L1 | -0.2084223 | 0.240055542 | 0.468184257 | NS | 0.619688264 |
| EIF5A2 | -0.169274746 | 0.057506291 | 0.251193798 | NS | 1.24028464 |
| SLC2A2 | 0.31798389 | 0.529122248 | NA | NS | 0.276443977 |
| TNIK | 0.069956256 | 0.27224596 | 0.497465358 | NS | 0.565038557 |
| PLD1 | 0.082329117 | 0.278782424 | 0.503126484 | NS | 0.554734609 |
| TMEM212 | 0.315621166 | 0.549461986 | NA | NS | 0.260062349 |
| FNDC3B | 0.096255605 | 0.254028533 | 0.480846473 | NS | 0.595117499 |
| GHSR | 0.545351423 | 0.096220938 | NA | NS | 1.016730412 |
| TNFSF10 | 0.065376136 | 0.576646884 | 0.737358732 | NS | 0.239090051 |
| NCEH1 | -0.308721931 | 0.002131771 | 0.072389226 | Down | 2.671259381 |
| ECT2 | -0.129522637 | 0.239617929 | 0.467680858 | NS | 0.62048069 |
| SPATA16 | 0.839179252 | 0.018387039 | NA | Up | 1.735488211 |
| NLGN1 | 0.386231161 | 0.269668698 | 0.495145716 | NS | 0.569169462 |
| NAALADL2 | 0.181879205 | 0.546912437 | 0.71536859 | NS | 0.2620822 |
| TBL1XR1 | -0.037604162 | 0.437122107 | 0.632879529 | NS | 0.359397229 |
| KCNMB2 | 0.40672161 | 0.267889186 | NA | NS | 0.572044818 |
| ZMAT3 | -0.007108117 | 0.902408707 | 0.945604168 | NS | 0.044596723 |
| PIK3CA | -0.007322663 | 0.886015288 | 0.935826321 | NS | 0.052558785 |
| KCNMB3 | 0.030028028 | 0.647400146 | 0.787108253 | NS | 0.188827207 |
| ZNF639 | -0.22446963 | 0.006681545 | 0.108679503 | Down | 2.175123112 |
| MFN1 | -0.057227551 | 0.15382732 | 0.380543257 | NS | 0.812966526 |
| GNB4 | -0.035662779 | 0.640319145 | 0.782028 | NS | 0.193603513 |
| ACTL6A | -0.124789508 | 0.09281756 | 0.305242354 | NS | 1.032369853 |
| MRPL47 | -0.293127381 | 0.051299152 | 0.23937514 | NS | 1.28988981 |
| NDUFB5 | -0.32082876 | 0.002788455 | 0.079430699 | Down | 2.554636301 |
| USP13 | 0.143436716 | 0.108034992 | 0.325769241 | NS | 0.966435556 |
| PEX5L | 0.24226912 | 0.22696661 | 0.455488754 | NS | 0.644038029 |
| TTC14 | -0.060381719 | 0.310395623 | 0.530362297 | NS | 0.508084411 |
| CCDC39 | 0.028426577 | 0.691375044 | 0.816810485 | NS | 0.160286301 |
| FXR1 | -0.07762702 | 0.069482814 | 0.269939848 | NS | 1.158122602 |
| DNAJC19 | -0.073096333 | 0.331020235 | 0.548094548 | NS | 0.480145458 |
| SOX2 | 0.413740332 | 0.371242252 | NA | NS | 0.430342602 |
| ATP11B | 0.053527708 | 0.424779676 | 0.623453338 | NS | 0.37183627 |
| DCUN1D1 | -0.431391597 | 0.003057378 | 0.083025772 | Down | 2.514650901 |
| MCCC1 | -0.147126912 | 0.001850715 | 0.067064999 | Down | 2.732660409 |
| LAMP3 | 0.494112459 | 0.012613736 | 0.135545097 | Up | 1.899156264 |
| MCF2L2 | 0.189093916 | 0.124531 | 0.346117593 | NS | 0.904722524 |
| B3GNT5 | 0.287717156 | 0.005746529 | 0.102208347 | Up | 2.240594365 |
| KLHL6 | 0.062022745 | 0.182302331 | 0.412292544 | NS | 0.739207779 |
| KLHL24 | -0.074820514 | 0.098002319 | 0.312744522 | NS | 1.008763647 |
| YEATS2 | -0.081836664 | 0.095172755 | 0.308775526 | NS | 1.021487358 |
| MAP6D1 | 0.168315069 | 0.566656194 | NA | NS | 0.246680359 |
| PARL | -0.155288802 | 0.0734444 | 0.275651204 | NS | 1.134041314 |
| ABCC5 | -0.021035142 | 0.794592979 | 0.881302161 | NS | 0.099855277 |
| HTR3D | 0.633221479 | 0.237263234 | NA | NS | 0.624769554 |
| HTR3C | 0.499052464 | 0.394375817 | NA | NS | 0.404089724 |
| HTR3E | 0.103938281 | 0.817793888 | NA | NS | 0.087356139 |
| EIF2B5 | -0.182837655 | 6.75E-04 | 0.042911362 | Down | 3.170870622 |
| DVL3 | 0.054336986 | 0.63068515 | 0.7751812 | NS | 0.200187395 |
| AP2M1 | 0.023770923 | 0.830939904 | 0.903832139 | NS | 0.080430384 |
| ABCF3 | -0.184932223 | 0.002442638 | 0.075698634 | Down | 2.612140931 |
| VWA5B2 | 0.428019545 | 0.050386454 | 0.237536143 | NS | 1.2976862 |
| ALG3 | 0.10172957 | 0.145424172 | 0.37059965 | NS | 0.837363401 |
| EEF1AKMT4 | 0.199229323 | 0.277927197 | 0.502231925 | NS | 0.556068952 |
| CAMK2N2 | 0.643002884 | 0.071902574 | NA | NS | 1.143255564 |
| ECE2 | 1.333002201 | 0.041780005 | NA | Up | 1.379031508 |
| PSMD2 | -0.090155171 | 0.125703776 | 0.347428564 | NS | 0.900651675 |
| EIF4G1 | -0.115834737 | 0.259533154 | 0.485843002 | NS | 0.585807155 |
| FAM131A | 0.140954353 | 0.084148701 | 0.292017244 | NS | 1.074952584 |
| CLCN2 | 0.270647124 | 0.15017958 | 0.376426917 | NS | 0.823389115 |
| POLR2H | -0.154640313 | 0.033010679 | 0.200267699 | Down | 1.481345547 |
| THPO | 0.457261771 | 0.224446002 | NA | NS | 0.648888126 |
| CHRD | 0.762196394 | 0.070672899 | 0.271568915 | NS | 1.150747093 |
| EPHB3 | 0.543607864 | 0.031917353 | 0.198125154 | Up | 1.495973128 |
| MAGEF1 | -0.203801784 | 0.086396998 | 0.295367076 | NS | 1.063501348 |
| VPS8 | 0.085727852 | 0.203486072 | 0.433444748 | NS | 0.691465312 |
| C3orf70 | 0.514528082 | 0.158094514 | 0.385419569 | NS | 0.8010832 |
| EHHADH | -0.088179957 | 0.483965738 | 0.669876909 | NS | 0.315185383 |
| MAP3K13 | 0.00562299 | 0.919824244 | 0.955625787 | NS | 0.036295148 |
| TMEM41A | -0.031847513 | 0.515414864 | 0.693278651 | NS | 0.287843061 |
| LIPH | 0.194503474 | 0.293463208 | 0.515445539 | NS | 0.53244634 |
| SENP2 | -0.138451096 | 0.002862505 | 0.080143153 | Down | 2.54325368 |
| IGF2BP2 | -0.128229317 | 0.549025546 | 0.717026862 | NS | 0.260407448 |
| TRA2B | -0.080125091 | 0.10345272 | 0.31928753 | NS | 0.985258084 |
| ETV5 | 0.404684916 | 0.133580495 | 0.356765072 | NS | 0.874256953 |
| DGKG | 0.083426974 | 0.395801352 | 0.601183351 | NS | 0.402522727 |
| CRYGS | -0.016771987 | 0.835450032 | 0.90719057 | NS | 0.07807952 |
| TBCCD1 | -0.146585158 | 0.001600173 | 0.063905938 | Down | 2.795833001 |
| DNAJB11 | -0.074006068 | 0.126762603 | 0.348680896 | NS | 0.89700885 |
| AHSG | 0.322677782 | 0.546898588 | NA | NS | 0.262093198 |
| FETUB | 0.491622688 | 0.376458568 | NA | NS | 0.424282814 |
| HRG | 0.553692581 | 0.242738406 | 0.470460443 | NS | 0.614861504 |
| KNG1 | 0.245493529 | 0.382240925 | 0.591611926 | NS | 0.417662816 |
| EIF4A2 | -0.194234773 | 0.018172999 | 0.156913557 | Down | 1.740573389 |
| RFC4 | -0.147295991 | 0.049530257 | 0.235468059 | Down | 1.305129417 |
| ADIPOQ | 0.687048791 | 0.215453736 | NA | NS | 0.66664597 |
| ST6GAL1 | 0.030221462 | 0.602438888 | 0.756016283 | NS | 0.220087002 |
| RPL39L | -0.040818765 | 0.788730146 | 0.87823307 | NS | 0.10307156 |
| RTP1 | 0.528430129 | 0.268716497 | NA | NS | 0.570705671 |
| MASP1 | 0.377859837 | 0.3911543 | 0.597971817 | NS | 0.407651891 |
| RTP4 | 0.154336187 | 0.168078847 | 0.396185854 | NS | 0.774486939 |
| SST | 1.226194335 | 0.055230866 | NA | NS | 1.257818149 |
| RTP2 | 0.799265389 | 0.123602836 | NA | NS | 0.907971563 |
| BCL6 | 0.25365431 | 0.067012681 | 0.266824786 | NS | 1.17384301 |
| LPP | 0.155073318 | 0.033616329 | 0.201786342 | Up | 1.473449714 |
| TPRG1 | -0.180862256 | 0.111061543 | 0.32960107 | NS | 0.954436298 |
| TP63 | 0.523606863 | 0.057752552 | 0.251741329 | NS | 1.238428822 |
| P3H2 | 0.543232307 | 0.060652424 | 0.256526216 | NS | 1.217151835 |
| CLDN1 | 0.826929457 | 0.099698134 | NA | NS | 1.001312968 |
| CLDN16 | 0.324763397 | 0.464315598 | NA | NS | 0.333186727 |
| TMEM207 | 0.75132678 | 0.130173982 | NA | NS | 0.885475808 |
| IL1RAP | 0.058464252 | 0.668527857 | 0.802023309 | NS | 0.174880491 |
| GMNC | 0.389538637 | 0.375829701 | NA | NS | 0.425008901 |
| OSTN | 0.48628516 | 0.098917423 | NA | NS | 1.004727208 |
| UTS2B | 0.157333273 | 0.385644919 | 0.593790502 | NS | 0.413812387 |
| CCDC50 | -0.070369912 | 0.342550042 | 0.557812931 | NS | 0.465275975 |
| FGF12 | 0.755690507 | 0.079767575 | 0.28517593 | NS | 1.09817361 |
| MB21D2 | -0.215101282 | 0.089230467 | 0.299597737 | NS | 1.049486832 |
| PLAAT1 | 0.269671055 | 0.231061077 | 0.459473889 | NS | 0.636273208 |
| ATP13A5 | 0.435233212 | 0.310363097 | 0.530362297 | NS | 0.508129924 |
| ATP13A4 | 0.128282066 | 0.352041923 | 0.565628183 | NS | 0.453405616 |
| OPA1 | -0.421279585 | 5.59E-06 | 0.001939873 | Down | 5.252556218 |
| HES1 | 0.392370488 | 0.006107924 | 0.104528345 | Up | 2.21410636 |
| CPN2 | 0.587074359 | 0.320947163 | NA | NS | 0.493566459 |
| LRRC15 | 0.404138692 | 0.418682169 | NA | NS | 0.378115535 |
| GP5 | 0.17520904 | 0.246614553 | 0.474778623 | NS | 0.607981298 |
| ATP13A3 | -0.016576417 | 0.700342273 | 0.822440711 | NS | 0.154689659 |
| TMEM44 | 0.256534418 | 0.064542091 | 0.263569594 | NS | 1.19015697 |
| LSG1 | -0.063744853 | 0.249984561 | 0.477055781 | NS | 0.602086812 |
| FAM43A | 0.02255675 | 0.783733927 | 0.874944277 | NS | 0.105831353 |
| XXYLT1 | -0.075068444 | 0.362990776 | 0.575468279 | NS | 0.440104411 |
| ACAP2 | -0.053759009 | 0.456330949 | 0.647996738 | NS | 0.340720076 |
| PPP1R2 | -0.14815478 | 0.04452488 | 0.225674429 | Down | 1.351397242 |
| APOD | 0.383507058 | 0.425365165 | NA | NS | 0.371238079 |
| TNK2 | -0.159249434 | 0.293925591 | 0.515962844 | NS | 0.5317626 |
| TFRC | -0.200338256 | 0.096685779 | 0.310822251 | NS | 1.014637398 |
| ZDHHC19 | 0.638691556 | 0.036815019 | 0.209163374 | Up | 1.433974974 |
| SLC51A | 0.010452736 | 0.904869318 | 0.947351639 | NS | 0.043414138 |
| PCYT1A | -0.140442546 | 0.011230675 | 0.13003023 | Down | 1.949594156 |
| DYNLT2B | 0.072079014 | 0.65641851 | 0.793648861 | NS | 0.182819181 |
| TM4SF19 | 0.027987098 | 0.866236659 | NA | NS | 0.062363441 |
| UBXN7 | 0.038712879 | 0.488439193 | 0.673099128 | NS | 0.311189495 |
| RNF168 | -0.077549618 | 0.045213936 | 0.227380227 | NS | 1.344727689 |
| SMCO1 | 0.284991402 | 0.628757977 | NA | NS | 0.201516492 |
| WDR53 | -0.015594792 | 0.789506253 | 0.878537356 | NS | 0.102644426 |
| FBXO45 | -0.195413845 | 0.007041638 | 0.110626998 | Down | 2.152326322 |
| NRROS | 0.208010544 | 0.1345576 | 0.358064534 | NS | 0.871091766 |
| PIGX | -0.003862512 | 0.964815659 | 0.980397915 | NS | 0.015555657 |
| CEP19 | 0.090979413 | 0.496371305 | 0.679085761 | NS | 0.304193332 |
| PAK2 | 0.177388761 | 0.042387346 | 0.220932874 | Up | 1.37276378 |
| SENP5 | -0.066320731 | 0.037251511 | 0.209852065 | NS | 1.428856112 |
| NCBP2 | -0.215725211 | 0.001304801 | 0.058839026 | Down | 2.884455709 |
| NCBP2AS2 | -0.208513045 | 0.085025624 | 0.293261573 | NS | 1.070450171 |
| PIGZ | 0.23748307 | 0.107626486 | 0.324922461 | NS | 0.968080838 |
| MELTF | 0.560986608 | 0.027266524 | 0.185919029 | Up | 1.564370225 |
| DLG1 | -0.081384537 | 0.164522427 | 0.392308942 | NS | 0.783774894 |
| BDH1 | 0.177584314 | 0.220193976 | 0.449374659 | NS | 0.657194567 |
| RUBCN | 0.021455015 | 0.85188893 | 0.916433852 | NS | 0.069617025 |
| FYTTD1 | -0.190611386 | 0.020679355 | 0.166818807 | Down | 1.684463007 |
| LRCH3 | -0.007616198 | 0.920492573 | 0.95614663 | NS | 0.035979711 |
| IQCG | -0.066772665 | 0.237955369 | 0.465981519 | NS | 0.623504491 |
| RPL35A | -0.539137239 | 0.00541136 | 0.100338483 | Down | 2.26669358 |
| LMLN | 0.095246496 | 0.475941327 | 0.663905973 | NS | 0.322446583 |
| ZNF595 | -0.146772129 | 0.171636622 | 0.400205856 | NS | 0.765390041 |
| ZNF718 | -0.19875156 | 0.001744919 | 0.065465219 | Down | 2.758224769 |
| ZNF732 | 0.503447443 | 0.070591784 | NA | NS | 1.151245845 |
| ZNF141 | -0.162061541 | 0.061708217 | 0.258215532 | NS | 1.209656999 |
| ZNF721 | -0.223656488 | 0.00949312 | 0.122561596 | Down | 2.02259103 |
| PIGG | 0.053273299 | 0.249008636 | 0.476376043 | NS | 0.603785591 |
| TMEM271 | 0.564256602 | 0.326277574 | NA | NS | 0.486412776 |
| PDE6B | -0.051912277 | 0.578251821 | 0.738275713 | NS | 0.237882991 |
| ATP5ME | -0.134034264 | 0.417873743 | 0.618692258 | NS | 0.378954917 |
| MYL5 | 0.082029774 | 0.403351859 | 0.60738002 | NS | 0.394315937 |
| SLC49A3 | 0.159931779 | 0.126671321 | 0.348597283 | NS | 0.897321701 |
| PCGF3 | 0.055234021 | 0.336893554 | 0.55308503 | NS | 0.472507299 |
| CPLX1 | 0.794663406 | 0.078132674 | NA | NS | 1.107167314 |
| GAK | -0.067654239 | 0.505581866 | 0.685949993 | NS | 0.296208512 |
| TMEM175 | 0.025443727 | 0.84157301 | 0.910726508 | NS | 0.074908201 |
| DGKQ | -0.025347832 | 0.804156853 | 0.887503427 | NS | 0.094659233 |
| SLC26A1 | 0.326972116 | 0.017155242 | 0.153970026 | Up | 1.76560315 |
| IDUA | 0.143874595 | 0.338030954 | 0.554078507 | NS | 0.471043529 |
| FGFRL1 | -0.090543206 | 0.371358715 | 0.58236492 | NS | 0.43020638 |
| RNF212 | 0.423431902 | 0.031357945 | 0.196726942 | Up | 1.503652412 |
| SPON2 | 0.060532005 | 0.626106525 | 0.771998042 | NS | 0.203351771 |
| CTBP1 | -0.185872826 | 0.005706346 | 0.102180893 | Down | 2.243641866 |
| MAEA | 2.38E-04 | 0.997104386 | 0.99845589 | NS | 0.001259373 |
| UVSSA | -6.71E-05 | 0.999500536 | 0.999687252 | NS | 2.17E-04 |
| NKX1-1 | 1.563767166 | 0.032435949 | NA | Up | 1.48897339 |
| FAM53A | 0.082570377 | 0.688592857 | NA | NS | 0.162037487 |
| SLBP | -0.229303729 | 0.001208989 | 0.056242293 | Down | 2.917577552 |
| TACC3 | 0.012889626 | 0.902198284 | 0.945506114 | NS | 0.044698003 |
| TMEM129 | 0.027270396 | 0.671329446 | 0.804120658 | NS | 0.173064303 |
| FGFR3 | 0.954587895 | 0.01210388 | 0.134107677 | Up | 1.917075386 |
| LETM1 | -0.151993648 | 0.027281991 | 0.185919029 | Down | 1.564123932 |
| NSD2 | -0.107715668 | 0.119482015 | 0.340007095 | NS | 0.922697464 |
| NELFA | 0.032023071 | 0.74739207 | 0.851665499 | NS | 0.126451514 |
| C4orf48 | 0.483191309 | 0.004523327 | 0.094211252 | Up | 2.344542025 |
| NAT8L | 0.993120892 | 0.004912289 | 0.096834757 | Up | 2.308716049 |
| POLN | 0.554909315 | 0.083033626 | 0.290260272 | NS | 1.080745999 |
| HAUS3 | -0.120990183 | 0.040613873 | 0.217525471 | Down | 1.391325596 |
| MXD4 | -6.64E-04 | 0.993734271 | 0.996257323 | NS | 0.002729732 |
| ZFYVE28 | 0.105225134 | 0.28719497 | 0.510345996 | NS | 0.541823171 |
| CFAP99 | 0.698478514 | 0.08361506 | 0.291291772 | NS | 1.077715494 |
| RNF4 | -0.099551764 | 0.068495538 | 0.268602708 | NS | 1.16433772 |
| FAM193A | 0.014995887 | 0.902415715 | 0.945604168 | NS | 0.044593351 |
| TNIP2 | -0.073327618 | 0.18338747 | 0.413624172 | NS | 0.736630341 |
| SH3BP2 | 0.113427988 | 0.290793672 | 0.513652722 | NS | 0.536415048 |
| ADD1 | -0.16052724 | 0.201763467 | 0.431728199 | NS | 0.695157468 |
| MFSD10 | -0.125740148 | 0.177938975 | 0.407964602 | NS | 0.749728915 |
| NOP14 | -0.204292121 | 0.001722309 | 0.065336585 | Down | 2.76388905 |
| GRK4 | 0.106298438 | 0.449738255 | 0.643129524 | NS | 0.34704017 |
| HTT | -0.011085207 | 0.89942942 | 0.944103767 | NS | 0.046032911 |
| MSANTD1 | 0.461903237 | 0.011270679 | 0.130166967 | Up | 1.948049911 |
| RGS12 | 0.105671349 | 0.310471667 | 0.530391028 | NS | 0.507978027 |
| HGFAC | 0.972791137 | 0.033606891 | 0.201786342 | Up | 1.473571658 |
| DOK7 | 0.388239558 | 0.03823325 | 0.213149437 | Up | 1.417558785 |
| LRPAP1 | -0.042189763 | 0.488259071 | 0.673012895 | NS | 0.311349679 |
| ADRA2C | 0.557333228 | 0.330699158 | NA | NS | 0.48056691 |
| OTOP1 | 0.998967332 | 0.102818342 | NA | NS | 0.987929403 |
| TMEM128 | -0.046988237 | 0.653099087 | 0.79126463 | NS | 0.185020923 |
| LYAR | -0.248477388 | 0.003372028 | 0.085084692 | Down | 2.472108836 |
| ZBTB49 | -0.011816167 | 0.806890849 | 0.888775213 | NS | 0.09318521 |
| NSG1 | 0.168888924 | 0.114850488 | 0.333655677 | NS | 0.939867154 |
| STX18 | -0.19113306 | 0.007733988 | 0.114126159 | Down | 2.111596493 |
| MSX1 | 0.553410861 | 0.209740684 | NA | NS | 0.678317319 |
| CYTL1 | 0.209229635 | 0.471558709 | NA | NS | 0.32646423 |
| STK32B | 0.399169164 | 0.134389201 | 0.357923187 | NS | 0.871635627 |
| EVC2 | 0.42227797 | 0.017084687 | 0.153632509 | Up | 1.767392976 |
| EVC | 0.369239944 | 0.065436849 | 0.264589463 | NS | 1.184177621 |
| CRMP1 | 0.401633675 | 0.162310854 | 0.389975155 | NS | 0.789652437 |
| C4orf50 | 0.03857849 | 0.819354995 | 0.897114813 | NS | 0.086527894 |
| JAKMIP1 | -0.107186226 | 0.489337555 | 0.673931606 | NS | 0.310391453 |
| WFS1 | 0.524598839 | 0.012487977 | 0.135480643 | Up | 1.903507927 |
| PPP2R2C | 0.639890692 | 0.108766737 | 0.326758974 | NS | 0.963503899 |
| MAN2B2 | 0.06021595 | 0.340709299 | 0.556206346 | NS | 0.467616013 |
| MRFAP1 | -0.169529145 | 0.013029772 | 0.137010024 | Down | 1.885063192 |
| S100P | 0.531778076 | 0.039608134 | 0.215363569 | Up | 1.402215615 |
| MRFAP1L1 | -0.267820114 | 0.003158094 | 0.084307443 | Down | 2.500574928 |
| BLOC1S4 | -0.182182256 | 0.051915171 | 0.240631705 | NS | 1.284705708 |
| KIAA0232 | 0.020040597 | 0.782718393 | 0.874453587 | NS | 0.10639446 |
| TBC1D14 | -0.073777299 | 0.332463447 | 0.549158158 | NS | 0.478256097 |
| CCDC96 | 0.339655226 | 0.040322058 | 0.216621104 | Up | 1.394457312 |
| TADA2B | 0.004158273 | 0.930579249 | 0.96204243 | NS | 0.031246636 |
| GRPEL1 | -0.089487471 | 0.155411796 | 0.382714082 | NS | 0.80851602 |
| SORCS2 | 0.591929705 | 0.038442724 | 0.213464789 | Up | 1.41518585 |
| PSAPL1 | 0.837859822 | 0.085394084 | NA | NS | 1.068572217 |
| AFAP1 | -0.158339064 | 0.303567075 | 0.524780583 | NS | 0.517745334 |
| ABLIM2 | 0.216924228 | 0.151505399 | 0.377971312 | NS | 0.819571891 |
| SH3TC1 | -0.10352519 | 0.242789799 | 0.470460443 | NS | 0.614769565 |
| HTRA3 | 0.581103945 | 0.092357862 | NA | NS | 1.034526131 |
| ACOX3 | -0.069082523 | 0.260834215 | 0.486967081 | NS | 0.583635441 |
| TRMT44 | 0.030281453 | 0.480322732 | 0.667102106 | NS | 0.318466859 |
| GPR78 | 0.723265547 | 0.090541447 | 0.301348674 | NS | 1.043152567 |
| CPZ | 0.816607748 | 0.123696127 | NA | NS | 0.907643899 |
| FAM90A26 | -0.271861213 | 0.805132238 | NA | NS | 0.094132783 |
| USP17L10 | 0.223539867 | 0.877013958 | NA | NS | 0.056993495 |
| USP17L18 | 0.551569095 | 0.406774736 | NA | NS | 0.390646028 |
| DEFB131A | 0.891666008 | 0.15028749 | NA | NS | 0.823077169 |
| SLC2A9 | 0.0337678 | 0.696528848 | 0.820148803 | NS | 0.157060892 |
| DRD5 | 0.617395644 | 0.12241731 | NA | NS | 0.912157169 |
| WDR1 | -0.002653364 | 0.957631594 | 0.976655159 | NS | 0.018801534 |
| ZNF518B | -0.035670212 | 0.573968176 | 0.735426831 | NS | 0.241112186 |
| CLNK | 0.154355075 | 0.343924781 | 0.558938783 | NS | 0.463536531 |
| HS3ST1 | 0.188805126 | 0.159076954 | 0.386749513 | NS | 0.798392732 |
| RAB28 | -0.073115444 | 0.137229278 | 0.361826493 | NS | 0.862553223 |
| NKX3-2 | 1.045467043 | 0.092575811 | NA | NS | 1.033502477 |
| BOD1L1 | -0.06170057 | 0.463102972 | 0.653681619 | NS | 0.334322432 |
| CPEB2 | 0.154528258 | 0.099915685 | 0.314860632 | NS | 1.00036633 |
| C1QTNF7 | 0.587067909 | 2.57E-04 | 0.026605481 | Up | 3.590510025 |
| CC2D2A | 0.381116989 | 0.001125201 | 0.054365705 | Up | 2.948770045 |
| FBXL5 | 0.068960812 | 0.498137434 | 0.680039551 | NS | 0.302650821 |
| FAM200B | 0.040165387 | 0.649836839 | 0.788856318 | NS | 0.187195672 |
| BST1 | 0.240682649 | 0.021575997 | 0.168827377 | Up | 1.666029129 |
| CD38 | 0.280802874 | 0.001143404 | 0.054782928 | Up | 2.94180027 |
| FGFBP1 | 0.723353455 | 0.094601123 | NA | NS | 1.024103706 |
| FGFBP2 | -0.242092618 | 0.137611965 | 0.362501636 | NS | 0.861343804 |
| PROM1 | 0.392989069 | 0.205068267 | 0.435100375 | NS | 0.688101539 |
| TAPT1 | -0.040612978 | 0.369358012 | 0.580818264 | NS | 0.432552476 |
| LDB2 | 0.113990852 | 0.631621453 | 0.775715902 | NS | 0.199543128 |
| QDPR | 0.291810203 | 6.62E-04 | 0.042665485 | Up | 3.179021266 |
| CLRN2 | -0.056255709 | 0.936564471 | NA | NS | 0.028462321 |
| LAP3 | 0.029904874 | 0.825844133 | 0.900867904 | NS | 0.083101912 |
| MED28 | -0.06962353 | 0.08625213 | 0.295172964 | NS | 1.064230172 |
| FAM184B | 0.375416367 | 0.008036077 | 0.115441239 | Up | 2.094955895 |
| DCAF16 | -0.109682592 | 0.007272234 | 0.111927889 | Down | 2.138332168 |
| NCAPG | 0.047483141 | 0.776023389 | 0.869623117 | NS | 0.110125189 |
| LCORL | -0.144936041 | 0.013426829 | 0.13912852 | Down | 1.872026542 |
| SLIT2 | 0.363797481 | 0.370222305 | 0.581378992 | NS | 0.43153742 |
| PACRGL | 0.036073142 | 0.536211115 | 0.70820686 | NS | 0.270664188 |
| KCNIP4 | 0.202715586 | 0.144358515 | 0.36967266 | NS | 0.840557595 |
| ADGRA3 | 0.044758579 | 0.765990421 | 0.863854529 | NS | 0.115776661 |
| PPARGC1A | 0.410437714 | 0.220677276 | 0.449641695 | NS | 0.656242385 |
| DHX15 | -0.068863199 | 0.197638874 | 0.427410856 | NS | 0.704127628 |
| SOD3 | 0.45152948 | 0.349345666 | NA | NS | 0.456744641 |
| CCDC149 | 0.093654909 | 0.416882442 | 0.61795739 | NS | 0.379986396 |
| LGI2 | 0.471374252 | 0.081663622 | 0.288059034 | NS | 1.087971364 |
| SEPSECS | -0.08591461 | 0.102307349 | 0.31812203 | NS | 0.990093168 |
| PI4K2B | -0.068104376 | 0.416371443 | 0.617519672 | NS | 0.380519065 |
| ZCCHC4 | -0.02604661 | 0.617537345 | 0.766154761 | NS | 0.209336773 |
| ANAPC4 | 0.021670475 | 0.612347764 | 0.762881338 | NS | 0.213001863 |
| SLC34A2 | 0.808134872 | 0.032903226 | 0.200078102 | Up | 1.482761524 |
| SEL1L3 | 0.001016708 | 0.986936149 | 0.992295615 | NS | 0.005710944 |
| SMIM20 | -0.258925596 | 0.0503492 | 0.237409384 | NS | 1.298007426 |
| RBPJ | 0.097956497 | 0.222849344 | 0.451584405 | NS | 0.65198864 |
| CCKAR | 0.581152792 | 0.230439466 | NA | NS | 0.637443139 |
| TBC1D19 | -0.035760256 | 0.602795057 | 0.756151929 | NS | 0.219830318 |
| STIM2 | 0.042387877 | 0.382092333 | 0.591501867 | NS | 0.417831676 |
| PCDH7 | 0.477965087 | 0.26880301 | 0.494425689 | NS | 0.570565873 |
| ARAP2 | -0.13348117 | 0.030424657 | 0.193712955 | Down | 1.516774307 |
| DTHD1 | -0.178109139 | 0.089017368 | 0.299357611 | NS | 1.05052525 |
| NWD2 | 0.611087734 | 0.100975668 | NA | NS | 0.995783264 |
| C4orf19 | 0.23224241 | 0.115809719 | 0.334925394 | NS | 0.93625499 |
| RELL1 | -0.043187558 | 0.618013161 | 0.766226582 | NS | 0.209002276 |
| PGM2 | -0.030971379 | 0.703723666 | 0.824593471 | NS | 0.152597844 |
| TBC1D1 | -0.057224558 | 0.401326147 | 0.605838206 | NS | 0.396502544 |
| PTTG2 | -0.119523625 | 0.481399452 | 0.668193506 | NS | 0.317494409 |
| KLF3 | -0.0401838 | 0.394037442 | 0.600042784 | NS | 0.404462509 |
| TLR10 | -0.004921768 | 0.958772643 | 0.977248837 | NS | 0.018284366 |
| TLR1 | 0.147166072 | 0.220691092 | 0.449641695 | NS | 0.656215196 |
| TLR6 | 0.176049013 | 0.13002449 | 0.352095778 | NS | 0.885974842 |
| FAM114A1 | 0.002228295 | 0.983332148 | 0.99010742 | NS | 0.007299762 |
| TMEM156 | -0.125782845 | 0.154117384 | 0.381018073 | NS | 0.812148372 |
| KLHL5 | 7.08E-04 | 0.989825472 | 0.994035648 | NS | 0.004441374 |
| WDR19 | -0.004558419 | 0.923684907 | 0.958245685 | NS | 0.034476153 |
| RFC1 | -0.178812988 | 5.64E-04 | 0.040211156 | Down | 3.248552059 |
| KLB | 0.129209351 | 0.152293099 | 0.378947167 | NS | 0.817319777 |
| RPL9 | -0.294553978 | 0.199242788 | 0.428926322 | NS | 0.700617389 |
| LIAS | -0.075221852 | 0.189311847 | 0.418800923 | NS | 0.722822208 |
| UGDH | -0.190952821 | 0.017835652 | 0.1561459 | Down | 1.748710999 |
| SMIM14 | -0.130907077 | 0.127256338 | 0.348830209 | NS | 0.895320577 |
| UBE2K | -0.072328184 | 0.308197836 | 0.528992493 | NS | 0.511170415 |
| PDS5A | -0.117508588 | 0.034402476 | 0.203549976 | Down | 1.463410297 |
| N4BP2 | -0.13822711 | 0.009036926 | 0.120388133 | Down | 2.043979292 |
| RHOH | -0.033192542 | 0.639361634 | 0.781025184 | NS | 0.194253428 |
| CHRNA9 | 0.225162913 | 0.472791481 | NA | NS | 0.325330358 |
| RBM47 | 0.190645865 | 0.091812273 | 0.303636778 | NS | 1.037099259 |
| NSUN7 | 0.355272783 | 0.027930408 | 0.187199945 | Up | 1.553922716 |
| APBB2 | 0.266546159 | 0.103125823 | 0.319138589 | NS | 0.986632571 |
| UCHL1 | 0.570261073 | 0.116449546 | NA | NS | 0.9338622 |
| LIMCH1 | 0.48028924 | 0.101943965 | 0.317905513 | NS | 0.991638479 |
| PHOX2B | 0.963106637 | 0.035734858 | NA | Up | 1.446907939 |
| TMEM33 | 0.016111981 | 0.815594663 | 0.894876106 | NS | 0.088525625 |
| DCAF4L1 | 0.228454604 | 0.026935702 | 0.185246086 | Up | 1.569671702 |
| SLC30A9 | -0.083300262 | 0.125845018 | 0.34757629 | NS | 0.900163973 |
| BEND4 | 0.089351008 | 0.428672359 | 0.626472744 | NS | 0.367874519 |
| SHISA3 | 0.546630082 | 0.056578683 | NA | NS | 1.247347168 |
| ATP8A1 | -0.029355551 | 0.607111556 | 0.759080027 | NS | 0.2167315 |
| GRXCR1 | 0.655543653 | 0.293633529 | NA | NS | 0.532194355 |
| KCTD8 | 0.580186216 | 0.268867943 | NA | NS | 0.570460976 |
| YIPF7 | 0.199978277 | 0.57379216 | NA | NS | 0.24124539 |
| GUF1 | -0.025720033 | 0.640962925 | 0.782522144 | NS | 0.193167091 |
| GNPDA2 | -0.043090165 | 0.536368263 | 0.708295535 | NS | 0.270536927 |
| GABRG1 | 0.635869527 | 0.192225556 | 0.42102173 | NS | 0.716188875 |
| GABRA2 | 0.442202024 | 0.358753569 | 0.571703656 | NS | 0.445203769 |
| COX7B2 | 0.520915386 | 0.394339537 | NA | NS | 0.404129677 |
| GABRA4 | 0.464389854 | 0.254183509 | 0.481079268 | NS | 0.59485263 |
| GABRB1 | 0.651861183 | 0.024283145 | 0.176550027 | Up | 1.614695067 |
| COMMD8 | -0.264526265 | 0.152235133 | 0.37892501 | NS | 0.817485109 |
| ATP10D | -0.036028029 | 0.545018587 | 0.713788281 | NS | 0.263588687 |
| CORIN | 0.147958737 | 0.414964502 | 0.616431019 | NS | 0.381989053 |
| NFXL1 | -0.026779697 | 0.847620228 | 0.914246619 | NS | 0.071798688 |
| NIPAL1 | 0.284656726 | 0.125587254 | 0.347286881 | NS | 0.901054437 |
| CNGA1 | 0.361837804 | 0.076395629 | 0.280162162 | NS | 1.116931488 |
| TXK | 0.029423612 | 0.718390681 | 0.833717613 | NS | 0.14363931 |
| TEC | 0.20916153 | 0.017528111 | 0.15536692 | Up | 1.756264881 |
| SLAIN2 | -0.112298142 | 0.088530649 | 0.298649128 | NS | 1.052906351 |
| SLC10A4 | 0.341163207 | 0.171990835 | NA | NS | 0.764494694 |
| ZAR1 | 0.453315567 | 0.072271768 | NA | NS | 1.141031319 |
| FRYL | -0.063151373 | 0.170012009 | 0.398309642 | NS | 0.7695204 |
| OCIAD1 | -0.176227833 | 0.0070773 | 0.110899425 | Down | 2.150132393 |
| OCIAD2 | -0.059571431 | 0.606205859 | 0.758568823 | NS | 0.21737987 |
| CWH43 | 0.682608938 | 0.132069898 | NA | NS | 0.879196158 |
| DCUN1D4 | -0.224483458 | 0.01264907 | 0.135558724 | Down | 1.897941417 |
| LRRC66 | 0.118755691 | 0.515848972 | 0.693487378 | NS | 0.287477431 |
| SGCB | -0.054868507 | 0.410830055 | 0.613592664 | NS | 0.386337792 |
| SPATA18 | 0.431392573 | 0.131198104 | NA | NS | 0.882072441 |
| USP46 | 0.115136066 | 0.033497694 | 0.201567051 | Up | 1.474985084 |
| ERVMER34-1 | 0.682367596 | 0.16828184 | NA | NS | 0.773962748 |
| RASL11B | 0.295129066 | 0.363549097 | NA | NS | 0.43943693 |
| SCFD2 | -0.183769756 | 0.009447472 | 0.122448223 | Down | 2.024684373 |
| FIP1L1 | -0.199227165 | 2.12E-04 | 0.023858165 | Down | 3.674064148 |
| LNX1 | -0.192140089 | 0.016380076 | 0.151203744 | Down | 1.7856841 |
| CHIC2 | 0.089076832 | 0.121776923 | 0.34249623 | NS | 0.914435002 |
| GSX2 | 1.013141382 | 0.036784422 | NA | Up | 1.434336059 |
| PDGFRA | 0.341329747 | 0.236856146 | 0.464700343 | NS | 0.625515342 |
| KIT | 0.197112201 | 0.175266291 | 0.40459113 | NS | 0.756301603 |
| KDR | 0.621205223 | 0.112548969 | 0.331021474 | NS | 0.94865848 |
| SRD5A3 | -0.100707658 | 0.378170863 | 0.588134532 | NS | 0.422311935 |
| TMEM165 | 0.005262703 | 0.92505576 | 0.959016206 | NS | 0.033832089 |
| CLOCK | 0.119415361 | 0.024401494 | 0.177017112 | Up | 1.612583577 |
| PDCL2 | 0.804478533 | 0.095848124 | NA | NS | 1.018416382 |
| NMU | 0.51172048 | 0.152255362 | NA | NS | 0.817427403 |
| EXOC1L | 0.69650106 | 0.163473626 | NA | NS | 0.786552305 |
| EXOC1 | -0.051269899 | 0.263746893 | 0.490023249 | NS | 0.578812648 |
| CEP135 | -0.108811727 | 0.148423183 | 0.374564243 | NS | 0.82849826 |
| CRACD | 0.135321153 | 0.533624752 | 0.70625717 | NS | 0.272764034 |
| AASDH | -0.137627198 | 0.021388042 | 0.168379028 | Down | 1.669828964 |
| PPAT | -0.157150966 | 0.030421684 | 0.193712955 | Down | 1.516816752 |
| PAICS | 0.009440541 | 0.859658184 | 0.920683302 | NS | 0.065674198 |
| SRP72 | -0.040004524 | 0.553775157 | 0.720722774 | NS | 0.256666531 |
| ARL9 | 0.427970127 | 0.151654892 | 0.378110567 | NS | 0.819143576 |
| THEGL | 0.198614867 | 0.378031928 | 0.588048799 | NS | 0.422471519 |
| HOPX | -0.343503693 | 0.01547885 | 0.147953512 | Down | 1.810261319 |
| SPINK2 | -0.434342874 | 0.066950521 | NA | NS | 1.174246036 |
| REST | 7.65E-04 | 0.983729152 | 0.99030176 | NS | 0.007124458 |
| NOA1 | 0.01422038 | 0.845418476 | 0.913147068 | NS | 0.072928265 |
| POLR2B | -0.221880472 | 0.0012107 | 0.056242293 | Down | 2.916963475 |
| IGFBP7 | -0.086185018 | 0.437840571 | 0.633404381 | NS | 0.358683998 |
| ADGRL3 | 0.386395469 | 0.351578871 | 0.565262486 | NS | 0.453977232 |
| TECRL | 0.474348983 | 0.400000606 | NA | NS | 0.397939351 |
| EPHA5 | 0.468092888 | 0.291660073 | 0.514510934 | NS | 0.535123019 |
| CENPC | -0.087095775 | 0.080620549 | 0.286282138 | NS | 1.093554248 |
| STAP1 | -0.140566409 | 0.277507654 | 0.501973011 | NS | 0.556725034 |
| UBA6 | -0.067332162 | 0.240070681 | 0.468184257 | NS | 0.619660875 |
| GNRHR | 0.037301456 | 0.770324552 | 0.866009611 | NS | 0.11332626 |
| TMPRSS11D | 0.069099747 | 0.712355575 | 0.830016761 | NS | 0.147303172 |
| TMPRSS11A | 0.586438012 | 0.101121831 | 0.316688091 | NS | 0.995155074 |
| TMPRSS11F | 0.652952511 | 0.175732426 | NA | NS | 0.755148095 |
| TMPRSS11B | 0.613486829 | 0.248735134 | NA | NS | 0.604262865 |
| YTHDC1 | -0.177615397 | 0.001496065 | 0.062296149 | Down | 2.825049518 |
| TMPRSS11E | 0.038850737 | 0.937840252 | NA | NS | 0.027871131 |
| UGT2B17 | -0.198844996 | 0.574989572 | 0.736180663 | NS | 0.240340032 |
| UGT2B15 | 0.055016691 | 0.941897391 | NA | NS | 0.025996406 |
| UGT2B7 | 0.545639028 | 0.301590604 | NA | NS | 0.520582193 |
| UGT2B11 | 0.416992814 | 0.078051949 | 0.282523416 | NS | 1.107616246 |
| UGT2B28 | 0.530485572 | 0.058539912 | 0.252625035 | NS | 1.232547936 |
| UGT2B4 | 0.553115652 | 0.364481757 | NA | NS | 0.438324204 |
| UGT2A1 | 0.536775653 | 0.260435081 | NA | NS | 0.584300517 |
| SULT1B1 | 0.361953928 | 0.015789742 | 0.149058805 | Up | 1.801624978 |
| SULT1E1 | 0.469957829 | 0.225879425 | NA | NS | 0.646123327 |
| CSN1S1 | 0.531303235 | 0.289931763 | NA | NS | 0.537704203 |
| CSN2 | 0.392856503 | 0.498875348 | NA | NS | 0.302007956 |
| ODAM | 0.129864016 | 0.83316791 | NA | NS | 0.079267465 |
| FDCSP | 0.897819771 | 0.159168714 | NA | NS | 0.798142294 |
| CSN3 | 0.851570797 | 0.136620592 | NA | NS | 0.864483837 |
| CABS1 | 0.748500181 | 0.267091409 | NA | NS | 0.57334008 |
| SMR3A | 0.310187256 | 0.596999376 | NA | NS | 0.224026123 |
| SMR3B | 0.526350137 | 0.338001282 | NA | NS | 0.471081653 |
| OPRPN | 0.042429579 | 0.93577334 | NA | NS | 0.028829332 |
| MUC7 | 0.340113806 | 0.505883838 | 0.686237734 | NS | 0.295949195 |
| AMTN | 0.74817592 | 0.221263661 | NA | NS | 0.655089907 |
| AMBN | 0.536988692 | 0.145528766 | NA | NS | 0.837051154 |
| ENAM | 0.055227734 | 0.712738491 | 0.830393544 | NS | 0.147069787 |
| JCHAIN | 0.02773077 | 0.913241866 | 0.951687128 | NS | 0.039414187 |
| UTP3 | -0.162636073 | 0.0284627 | 0.188709674 | Down | 1.545723898 |
| RUFY3 | -0.045436225 | 0.381457685 | 0.591038759 | NS | 0.418553632 |
| GRSF1 | -0.179449974 | 0.012925653 | 0.136295233 | Down | 1.888547494 |
| MOB1B | -0.020346306 | 0.793624431 | 0.880770824 | NS | 0.100384971 |
| DCK | -0.298728567 | 0.002287541 | 0.073891772 | Down | 2.640631091 |
| SLC4A4 | -0.106382176 | 0.382566374 | 0.591955618 | NS | 0.417293205 |
| GC | 0.44843512 | 0.409051933 | NA | NS | 0.388221551 |
| NPFFR2 | 0.503023476 | 0.380195892 | NA | NS | 0.419992581 |
| ADAMTS3 | 0.188886694 | 0.527449234 | 0.702304788 | NS | 0.277819334 |
| COX18 | -0.056418181 | 0.327773536 | 0.545611767 | NS | 0.484426113 |
| ANKRD17 | -0.041807987 | 0.675004692 | 0.806240246 | NS | 0.170693208 |
| ALB | 0.573628281 | 0.102211657 | NA | NS | 0.99049957 |
| AFP | 0.255071441 | 0.622456362 | NA | NS | 0.20589109 |
| AFM | 0.54540741 | 0.284850526 | NA | NS | 0.545382974 |
| RASSF6 | -0.017026652 | 0.912423892 | 0.951241312 | NS | 0.039803351 |
| CXCL8 | -0.413798879 | 0.034734441 | 0.204390587 | Down | 1.459239688 |
| CXCL6 | -0.040244288 | 0.80098402 | 0.885420215 | NS | 0.096376148 |
| PF4V1 | 0.336112294 | 0.095724664 | 0.309382762 | NS | 1.018976149 |
| CXCL1 | 0.049349366 | 0.71475279 | 0.831349335 | NS | 0.145844141 |
| PF4 | -0.063193677 | 0.694010243 | 0.818361785 | NS | 0.158634119 |
| PPBP | -0.223875483 | 0.202781279 | 0.432901167 | NS | 0.692972142 |
| CXCL5 | 0.102343727 | 0.551687904 | 0.719031683 | NS | 0.258306538 |
| CXCL3 | 0.074486162 | 0.765243375 | NA | NS | 0.116200421 |
| CXCL2 | -0.211793646 | 0.556242148 | NA | NS | 0.254736107 |
| MTHFD2L | -0.043445945 | 0.464579484 | 0.654918093 | NS | 0.332939973 |
| EPGN | 0.107917344 | 0.570326112 | 0.732392713 | NS | 0.243876744 |
| EREG | -0.493157086 | 0.038515934 | 0.213464789 | Down | 1.414359569 |
| AREG | -0.107029102 | 0.697371227 | NA | NS | 0.156535975 |
| BTC | 0.65164168 | 0.201037673 | NA | NS | 0.69672255 |
| PARM1 | 0.132255637 | 0.305342072 | 0.526578834 | NS | 0.515213351 |
| RCHY1 | -0.052364984 | 0.36259444 | 0.5751983 | NS | 0.44057886 |
| THAP6 | 0.009656141 | 0.858509034 | 0.920097988 | NS | 0.06625513 |
| ODAPH | 0.328049991 | 0.380760699 | NA | NS | 0.419347885 |
| CDKL2 | 0.170008303 | 0.514563688 | 0.692659911 | NS | 0.288560865 |
| G3BP2 | -0.135161555 | 0.010753571 | 0.12826482 | Down | 1.96844731 |
| USO1 | -0.103848899 | 0.064320111 | 0.263154352 | NS | 1.191653213 |
| PPEF2 | 0.40923382 | 0.199181619 | 0.428926322 | NS | 0.700750741 |
| NAAA | -0.292261921 | 1.13E-04 | 0.016924534 | Down | 3.948035916 |
| SDAD1 | -0.209879072 | 0.001120202 | 0.05424964 | Down | 2.950703542 |
| CXCL9 | 0.372815408 | 0.166982796 | 0.395025307 | NS | 0.777328271 |
| ART3 | 0.41609289 | 0.172926225 | 0.401822933 | NS | 0.762139139 |
| CXCL10 | -0.055123792 | 0.818138665 | 0.896550239 | NS | 0.087173082 |
| CXCL11 | -0.079592591 | 0.788794391 | NA | NS | 0.103036186 |
| NUP54 | -0.30710956 | 0.004597494 | 0.094750689 | Down | 2.337478858 |
| SCARB2 | -0.045333398 | 0.490140717 | 0.674632052 | NS | 0.309679218 |
| FAM47E | 0.535045759 | 0.06171827 | 0.258215532 | NS | 1.209586255 |
| STBD1 | -0.261860489 | 0.1999804 | NA | NS | 0.699012567 |
| CCDC158 | 0.205448045 | 0.600080905 | 0.754415898 | NS | 0.221790192 |
| SHROOM3 | 0.505537082 | 0.179053945 | 0.409087535 | NS | 0.747016106 |
| SOWAHB | 1.305059572 | 0.006759137 | NA | Up | 2.170108732 |
| SEPTIN11 | 0.028671794 | 0.578101084 | 0.738214279 | NS | 0.237996216 |
| CCNI | -0.004242909 | 0.979809489 | 0.988006205 | NS | 0.008858359 |
| CCNG2 | 0.122812025 | 0.072224874 | 0.273882705 | NS | 1.141313209 |
| CXCL13 | 0.882768818 | 0.014967952 | NA | Up | 1.824837627 |
| CNOT6L | -0.062099836 | 0.245985574 | 0.47416561 | NS | 0.609090362 |
| MRPL1 | -0.300044528 | 0.029722368 | 0.192180034 | Down | 1.526916587 |
| FRAS1 | 0.314012799 | 0.413502111 | 0.615486243 | NS | 0.383522269 |
| ANXA3 | 0.473796567 | 0.01828482 | 0.157273532 | Up | 1.737909301 |
| BMP2K | -0.095100991 | 0.282831358 | 0.505770575 | NS | 0.548472441 |
| PAQR3 | 0.008203533 | 0.88922389 | 0.937363658 | NS | 0.050988878 |
| NAA11 | 0.51753288 | 0.261997265 | NA | NS | 0.581703242 |
| GK2 | 1.60374407 | 0.006889932 | NA | Up | 2.161785058 |
| ANTXR2 | -0.058297893 | 0.393983221 | 0.600042784 | NS | 0.404522274 |
| PRDM8 | 0.082620541 | 0.274035823 | 0.498866181 | NS | 0.562192662 |
| FGF5 | 1.032430474 | 0.019887492 | NA | Up | 1.701419992 |
| CFAP299 | 0.521247787 | 0.255700671 | 0.482655301 | NS | 0.592268132 |
| BMP3 | 0.357653378 | 0.137956461 | 0.362607036 | NS | 0.860257955 |
| PRKG2 | -0.078942021 | 0.649113912 | 0.788396628 | NS | 0.187679083 |
| RASGEF1B | 0.032739867 | 0.748087678 | 0.851877572 | NS | 0.126047499 |
| HNRNPD | 0.057381506 | 0.261097699 | 0.487101622 | NS | 0.583196955 |
| HNRNPDL | -0.007585405 | 0.874849535 | 0.92952002 | NS | 0.058066635 |
| ENOPH1 | -0.206181071 | 0.020183166 | 0.164578764 | Down | 1.695010704 |
| TMEM150C | 0.056578156 | 0.801509511 | 0.885692448 | NS | 0.09609132 |
| SCD5 | 0.070551189 | 0.628703854 | 0.77391826 | NS | 0.201553878 |
| SEC31A | 0.015674583 | 0.744637935 | 0.850176877 | NS | 0.128054843 |
| THAP9 | -0.189425722 | 0.014813454 | 0.14588426 | Down | 1.829343652 |
| LIN54 | -0.180696326 | 0.010582555 | 0.127927347 | Down | 1.975409463 |
| COPS4 | -0.233329704 | 0.044917942 | 0.226679546 | Down | 1.347580149 |
| PLAC8 | -0.048126986 | 0.511176932 | 0.689903595 | NS | 0.291428753 |
| COQ2 | -0.023874733 | 0.702321324 | 0.823751048 | NS | 0.153464145 |
| HPSE | 0.243276343 | 0.009732985 | 0.12411182 | Up | 2.011753933 |
| HELQ | -0.183262831 | 0.006445313 | 0.1080547 | Down | 2.19075596 |
| MRPS18C | -0.031470808 | 0.652651302 | 0.790847626 | NS | 0.185318791 |
| ABRAXAS1 | -0.143326709 | 0.052134017 | 0.241262687 | NS | 1.282878807 |
| GPAT3 | -0.172040016 | 0.137921954 | 0.362607036 | NS | 0.8603666 |
| NKX6-1 | 1.005400239 | 0.013733661 | 0.140917694 | Up | 1.862213691 |
| CDS1 | 0.190099465 | 0.211376778 | 0.441234413 | NS | 0.674942727 |
| WDFY3 | 0.222822534 | 0.089562114 | 0.299928733 | NS | 1.047875665 |
| ARHGAP24 | 0.168272595 | 0.072570957 | 0.274170249 | NS | 1.139237152 |
| MAPK10 | 0.35789857 | 0.064008271 | 0.262600549 | NS | 1.193763904 |
| PTPN13 | 0.156263755 | 0.042488337 | 0.221095683 | Up | 1.37173027 |
| SLC10A6 | -0.185846012 | 0.650288909 | NA | NS | 0.186893653 |
| C4orf36 | 1.495202289 | 0.014446776 | NA | Up | 1.840229049 |
| AFF1 | -0.072383309 | 0.550084242 | 0.717672856 | NS | 0.259570796 |
| KLHL8 | 0.008523039 | 0.908963101 | 0.949563038 | NS | 0.041453747 |
| HSD17B13 | 0.231427168 | 0.079146652 | 0.28419828 | NS | 1.10156745 |
| HSD17B11 | -0.01960266 | 0.850868257 | 0.916239648 | NS | 0.070137678 |
| NUDT9 | -0.076044861 | 0.195711652 | 0.42550521 | NS | 0.708383316 |
| SPARCL1 | 0.415203586 | 0.134655228 | 0.358173522 | NS | 0.870776781 |
| DSPP | 0.588871132 | 0.214850327 | NA | NS | 0.66786398 |
| DMP1 | 0.501839633 | 0.349529885 | NA | NS | 0.456515687 |
| IBSP | 0.47383797 | 0.380302362 | NA | NS | 0.419870977 |
| MEPE | 0.470638845 | 0.362463623 | NA | NS | 0.440735573 |
| SPP1 | 0.099377214 | 0.677438945 | 0.807468862 | NS | 0.16912984 |
| PKD2 | -0.07002182 | 0.241280918 | 0.469262706 | NS | 0.617477023 |
| ABCG2 | 0.119445711 | 0.56487354 | 0.728912205 | NS | 0.248048768 |
| PPM1K | -0.098508041 | 0.133313191 | 0.35644448 | NS | 0.875126878 |
| HERC6 | 0.134096735 | 0.03422119 | 0.203152353 | Up | 1.46570489 |
| HERC5 | 0.365997206 | 0.025066255 | 0.179152549 | Up | 1.600910554 |
| HERC3 | 0.16206685 | 0.074260092 | 0.276762349 | NS | 1.129244515 |
| NAP1L5 | -0.10074116 | 0.344527465 | 0.559565981 | NS | 0.462776151 |
| FAM13A | 0.032322096 | 0.664903288 | 0.799433804 | NS | 0.17724152 |
| TIGD2 | -0.177926833 | 0.064203723 | 0.262851922 | NS | 1.192439791 |
| GPRIN3 | -0.02419323 | 0.703247276 | 0.824166467 | NS | 0.152891942 |
| SNCA | -0.401730678 | 0.03941598 | 0.214827411 | Down | 1.404327667 |
| MMRN1 | -0.06794462 | 0.67255148 | 0.804910848 | NS | 0.172274467 |
| CCSER1 | 0.007810086 | 0.924060424 | 0.958461587 | NS | 0.034299629 |
| GRID2 | 0.461662737 | 0.31502766 | 0.534426923 | NS | 0.501651313 |
| ATOH1 | 1.266173349 | 0.019290258 | NA | Up | 1.714661957 |
| SMARCAD1 | -0.151348152 | 0.017258946 | 0.154219421 | Down | 1.762985736 |
| HPGDS | -0.006327558 | 0.974668926 | 0.985403414 | NS | 0.01114288 |
| PDLIM5 | 0.054402284 | 0.287476554 | 0.510589731 | NS | 0.54139757 |
| BMPR1B | 0.256803368 | 0.485688119 | 0.671376842 | NS | 0.31364252 |
| UNC5C | 0.131565877 | 0.722768027 | 0.836344784 | NS | 0.141001068 |
| PDHA2 | 0.121008992 | 0.846964404 | NA | NS | 0.072134842 |
| STPG2 | 0.439834747 | 0.043695167 | 0.224222878 | Up | 1.359566601 |
| RAP1GDS1 | -0.079308287 | 0.135242677 | 0.359027215 | NS | 0.868886243 |
| TSPAN5 | -0.09838908 | 0.542472955 | 0.712450717 | NS | 0.265621909 |
| EIF4E | -0.153720193 | 0.044132537 | 0.224705062 | Down | 1.355241104 |
| METAP1 | -0.025834444 | 0.538735188 | 0.709653107 | NS | 0.268624657 |
| ADH5 | -0.221096146 | 0.021326964 | 0.168379028 | Down | 1.671070969 |
| ADH4 | 0.087142439 | 0.730110806 | 0.840420069 | NS | 0.136611224 |
| ADH6 | -0.024507743 | 0.898122396 | 0.943349803 | NS | 0.046664474 |
| ADH1A | 0.616084439 | 0.043492127 | NA | Up | 1.361589353 |
| ADH1B | 0.529325414 | 0.249718387 | 0.476865714 | NS | 0.602549478 |
| ADH1C | 0.533826234 | 0.331111191 | NA | NS | 0.480026141 |
| ADH7 | 0.221539735 | 0.70675331 | NA | NS | 0.150732149 |
| C4orf17 | 0.254710998 | 0.514739127 | NA | NS | 0.288412819 |
| TRMT10A | -0.198024742 | 0.034128267 | 0.202965138 | Down | 1.46688576 |
| MTTP | 0.705662813 | 0.094081026 | 0.30725936 | NS | 1.026497957 |
| C4orf54 | 0.87050456 | 0.035979887 | 0.207455025 | Up | 1.443940211 |
| DAPP1 | 0.061294694 | 0.436705573 | 0.632521568 | NS | 0.359811266 |
| LAMTOR3 | -0.123560775 | 0.139894911 | 0.364117884 | NS | 0.854198083 |
| DNAJB14 | -0.097099447 | 0.132031786 | 0.354863509 | NS | 0.879321502 |
| H2AZ1 | -0.091092795 | 0.391837516 | 0.598576761 | NS | 0.406893985 |
| DDIT4L | 0.080414283 | 0.781896258 | NA | NS | 0.106850865 |
| EMCN | 0.532821314 | 0.169170683 | 0.397409682 | NS | 0.771674897 |
| PPP3CA | 0.06232683 | 0.261210496 | 0.487232675 | NS | 0.583009376 |
| BANK1 | 0.020334095 | 0.838509592 | 0.908983201 | NS | 0.076491965 |
| SLC39A8 | -0.145810977 | 0.064130945 | 0.262789011 | NS | 1.192932358 |
| NFKB1 | -0.079253425 | 0.228760109 | 0.457360455 | NS | 0.640619705 |
| MANBA | -0.069996875 | 0.224009121 | 0.452443504 | NS | 0.649734298 |
| UBE2D3 | -0.146261248 | 0.084891878 | 0.293013352 | NS | 1.07113386 |
| CISD2 | -0.308940692 | 0.093765094 | 0.306991878 | NS | 1.027958806 |
| SLC9B1 | 0.041744793 | 0.688943003 | 0.815257617 | NS | 0.161816707 |
| SLC9B2 | 0.01159788 | 0.882926616 | 0.933787366 | NS | 0.054075391 |
| BDH2 | -0.122373251 | 0.234737015 | 0.462791997 | NS | 0.629418422 |
| CENPE | -0.599875933 | 6.99E-04 | 0.043826722 | Down | 3.15555709 |
| TACR3 | 0.672134812 | 0.171238822 | NA | NS | 0.766397768 |
| CXXC4 | 0.020613997 | 0.921075374 | 0.956459449 | NS | 0.035704829 |
| TET2 | 0.168590387 | 0.054665553 | 0.246775177 | NS | 1.262286252 |
| PPA2 | -0.142874377 | 0.035441033 | 0.206007753 | Down | 1.450493623 |
| ARHGEF38 | 0.033717388 | 0.810843123 | 0.891456445 | NS | 0.091063162 |
| INTS12 | -0.056004263 | 0.332163091 | 0.548939177 | NS | 0.478648627 |
| GSTCD | -0.092406665 | 0.185758921 | 0.415463304 | NS | 0.731050319 |
| NPNT | 0.519867905 | 0.225849504 | 0.454435762 | NS | 0.64618086 |
| TBCK | 0.005253779 | 0.92013957 | 0.955823306 | NS | 0.036146293 |
| AIMP1 | -0.097005471 | 0.177098574 | 0.406950818 | NS | 0.751784936 |
| GIMD1 | 0.255251583 | 0.553200209 | NA | NS | 0.257117665 |
| DKK2 | 0.268122053 | 0.354958732 | 0.568201921 | NS | 0.449822135 |
| PAPSS1 | 0.138985145 | 0.187916768 | 0.417308021 | NS | 0.726034465 |
| SGMS2 | -0.004969045 | 0.962591453 | 0.979032249 | NS | 0.016557999 |
| CYP2U1 | -0.144240799 | 0.078402713 | 0.28303577 | NS | 1.10566891 |
| HADH | -0.026682305 | 0.564306323 | 0.728337377 | NS | 0.248485083 |
| LEF1 | 0.016881383 | 0.878543647 | 0.931684267 | NS | 0.056236658 |
| RPL34 | -0.73850006 | 0.005319345 | 0.099529109 | Down | 2.274141878 |
| OSTC | -0.200501182 | 0.10159095 | 0.317326231 | NS | 0.99314498 |
| ETNPPL | 0.431939753 | 0.355329723 | NA | NS | 0.449368463 |
| COL25A1 | 0.450349032 | 0.130994649 | 0.353319922 | NS | 0.882746444 |
| SEC24B | -0.022262973 | 0.78446353 | 0.875577775 | NS | 0.105427242 |
| MCUB | -0.178881932 | 0.045581339 | 0.228075994 | Down | 1.341212922 |
| CASP6 | -0.116740476 | 0.085571501 | 0.293993175 | NS | 1.067670852 |
| PLA2G12A | 0.009859715 | 0.887312399 | 0.936375844 | NS | 0.05192345 |
| CFI | 0.409252028 | 0.447230947 | NA | NS | 0.349468152 |
| GAR1 | 0.060164731 | 0.463405886 | 0.654068877 | NS | 0.334038454 |
| RRH | 0.284508382 | 0.102490004 | 0.318267941 | NS | 0.989318491 |
| LRIT3 | 0.146580231 | 0.493292782 | 0.677207384 | NS | 0.306895239 |
| EGF | -0.013184909 | 0.931863947 | 0.962684504 | NS | 0.030647491 |
| ELOVL6 | -0.052420471 | 0.590886222 | 0.748022567 | NS | 0.228496137 |
| ENPEP | 0.187597142 | 0.578888669 | 0.738673295 | NS | 0.237404951 |
| PITX2 | 0.869736987 | 0.067395409 | 0.267270936 | NS | 1.171369687 |
| FAM241A | -0.063767522 | 0.402435979 | 0.606636604 | NS | 0.395303199 |
| AP1AR | -0.166241783 | 0.043135489 | 0.222949439 | Down | 1.365165276 |
| TIFA | 0.034260892 | 0.746863619 | 0.85131747 | NS | 0.126758695 |
| ALPK1 | 0.161828093 | 0.155144407 | 0.382552064 | NS | 0.809263875 |
| NEUROG2 | 1.498987627 | 0.005963757 | NA | Up | 2.224480059 |
| ZGRF1 | -0.152515759 | 0.097266996 | 0.311597251 | NS | 1.012034496 |
| LARP7 | -0.096407485 | 0.211462252 | 0.441234413 | NS | 0.674767146 |
| ANK2 | 0.320709731 | 0.349615045 | 0.563682195 | NS | 0.456409887 |
| CAMK2D | 0.04019477 | 0.478026237 | 0.665701757 | NS | 0.320548266 |
| ARSJ | 0.414071819 | 0.150768949 | 0.377320464 | NS | 0.821688092 |
| UGT8 | 0.182260564 | 0.126139874 | 0.347971019 | NS | 0.899147606 |
| NDST4 | 0.444003686 | 0.335488612 | NA | NS | 0.474322217 |
| MTRNR2L13 | 0.466676335 | 0.469517922 | NA | NS | 0.328347826 |
| TRAM1L1 | 1.339962649 | 0.023004679 | NA | Up | 1.638183827 |
| NDST3 | 0.339388417 | 0.241364117 | 0.469262706 | NS | 0.617327295 |
| PRSS12 | 0.519428608 | 0.191425401 | 0.420728886 | NS | 0.718000436 |
| METTL14 | -0.069417294 | 0.182659746 | 0.412849239 | NS | 0.73835715 |
| SEC24D | 0.175554349 | 0.003449539 | 0.085194315 | Up | 2.462238981 |
| SYNPO2 | 0.250121281 | 0.246498622 | 0.474646517 | NS | 0.608185504 |
| MYOZ2 | 0.069517327 | 0.778360669 | 0.871304366 | NS | 0.108819118 |
| USP53 | -0.187163607 | 0.018231705 | 0.157108372 | Down | 1.739172712 |
| C4orf3 | -0.082230155 | 0.390344386 | 0.59729185 | NS | 0.408552063 |
| FABP2 | 0.397469098 | 0.233048318 | NA | NS | 0.632554028 |
| PDE5A | 0.031196685 | 0.721911399 | 0.83589741 | NS | 0.1415161 |
| MAD2L1 | -0.087105528 | 0.470849403 | 0.659938374 | NS | 0.327117976 |
| PRDM5 | 0.181939376 | 0.291393058 | 0.51421512 | NS | 0.535520798 |
| NDNF | 0.400516769 | 0.355290233 | NA | NS | 0.449416732 |
| TNIP3 | -0.030051478 | 0.819861896 | 0.897279394 | NS | 0.086259297 |
| QRFPR | 0.700745314 | 0.139770168 | NA | NS | 0.854585514 |
| ANXA5 | -0.295151695 | 0.008248222 | 0.116794174 | Down | 2.083639665 |
| SMIM43 | 0.266925503 | 0.360580785 | NA | NS | 0.44299742 |
| EXOSC9 | -0.107818382 | 0.103103236 | 0.319138589 | NS | 0.986727704 |
| CCNA2 | 0.139794335 | 0.160807921 | 0.388591588 | NS | 0.793692564 |
| BBS7 | -0.144042582 | 0.033202851 | 0.200900063 | Down | 1.478824625 |
| TRPC3 | 0.290295764 | 0.169951008 | 0.398309642 | NS | 0.769676255 |
| KIAA1109 | 0.098803448 | 0.168757002 | 0.396950027 | NS | 0.772738197 |
| ADAD1 | 0.729323507 | 0.051225763 | 0.239227656 | NS | 1.290511564 |
| IL2 | 0.666652078 | 0.020457899 | NA | Up | 1.689138975 |
| IL21 | 0.42549883 | 0.254274151 | NA | NS | 0.594697788 |
| BBS12 | 0.068619369 | 0.384340716 | 0.593034804 | NS | 0.415283606 |
| FGF2 | 0.336501825 | 0.053881365 | 0.245326247 | NS | 1.26856141 |
| NUDT6 | 0.121646631 | 0.294290569 | 0.516383896 | NS | 0.531223655 |
| SPATA5 | -0.015930901 | 0.765943878 | 0.863854529 | NS | 0.115803051 |
| SPRY1 | -0.013549374 | 0.919287263 | 0.955284581 | NS | 0.036548757 |
| ANKRD50 | 0.099365895 | 0.181925322 | 0.412022664 | NS | 0.740106847 |
| FAT4 | 0.233224168 | 0.173155422 | 0.402191225 | NS | 0.761563905 |
| INTU | 0.18723006 | 0.432703046 | 0.629311308 | NS | 0.363810047 |
| SLC25A31 | 0.432190624 | 0.378857842 | NA | NS | 0.421523719 |
| HSPA4L | 0.173465874 | 0.261618275 | 0.487794653 | NS | 0.582331923 |
| PLK4 | -0.215004133 | 0.063373276 | 0.261443316 | NS | 1.198093843 |
| MFSD8 | 0.002182536 | 0.960003244 | 0.977720206 | NS | 0.0177273 |
| ABHD18 | -0.123756351 | 0.008476609 | 0.11782982 | Down | 2.071777849 |
| LARP1B | -0.589197242 | 3.72E-05 | 0.00829096 | Down | 4.42891052 |
| PGRMC2 | -0.067272968 | 0.126108486 | 0.34792634 | NS | 0.899255688 |
| JADE1 | -0.002243596 | 0.959056311 | 0.977277314 | NS | 0.018155892 |
| SCLT1 | -0.164524905 | 0.100722551 | 0.316153692 | NS | 0.996873282 |
| C4orf33 | 0.086022573 | 0.205914035 | 0.435885316 | NS | 0.686314051 |
| PCDH10 | 0.625868316 | 0.185167737 | 0.415384315 | NS | 0.732434682 |
| PABPC4L | 0.124669725 | 0.836158069 | NA | NS | 0.077711615 |
| PCDH18 | 0.498635191 | 0.129670445 | NA | NS | 0.887158998 |
| SLC7A11 | 0.462584298 | 0.004430079 | 0.093509368 | Up | 2.353588551 |
| NOCT | 0.090160625 | 0.230186755 | 0.458531536 | NS | 0.637919669 |
| ELF2 | -0.030027956 | 0.568682328 | 0.73151891 | NS | 0.245130267 |
| MGARP | 0.230040651 | 0.457255115 | NA | NS | 0.339841427 |
| NDUFC1 | -0.146628765 | 0.062541171 | 0.260043193 | NS | 1.203833989 |
| NAA15 | -0.060480294 | 0.189917489 | 0.419275561 | NS | 0.72143504 |
| RAB33B | 0.046057492 | 0.643763845 | 0.784561493 | NS | 0.191273418 |
| SETD7 | -0.040732198 | 0.539089437 | 0.709925767 | NS | 0.268339177 |
| MGST2 | -0.148239481 | 0.143632663 | 0.368733913 | NS | 0.842746789 |
| MAML3 | 0.016502767 | 0.87857972 | 0.931684267 | NS | 0.056218826 |
| SCOC | -0.261975702 | 0.066064601 | 0.265300982 | NS | 1.180031182 |
| CLGN | 0.188868354 | 0.484593834 | 0.670503141 | NS | 0.314622116 |
| MGAT4D | 0.649087325 | 0.142366142 | 0.3670873 | NS | 0.846593282 |
| ELMOD2 | -0.121008447 | 0.111430906 | 0.329942771 | NS | 0.952994337 |
| UCP1 | 0.47999134 | 0.038412938 | NA | Up | 1.415522478 |
| TBC1D9 | -0.128934144 | 0.133283833 | 0.35644448 | NS | 0.875222526 |
| RNF150 | 0.40530772 | 0.268020614 | 0.493891886 | NS | 0.571831802 |
| ZNF330 | -0.06792096 | 0.22522682 | 0.453643215 | NS | 0.647379896 |
| IL15 | 0.133967173 | 0.046983243 | 0.230557153 | Up | 1.328057008 |
| INPP4B | -0.10652463 | 0.20338188 | 0.433444748 | NS | 0.691687742 |
| USP38 | 0.026595685 | 0.569714061 | 0.732105905 | NS | 0.244343061 |
| GAB1 | -0.027310972 | 0.797729687 | 0.883614107 | NS | 0.098144246 |
| SMARCA5 | -0.310856723 | 0.003528993 | 0.086163115 | Down | 2.452349182 |
| FREM3 | 0.520301229 | 0.00686377 | 0.109849098 | Up | 2.16343728 |
| GYPE | -0.043837457 | 0.819255308 | 0.897114813 | NS | 0.086580736 |
| GYPB | -0.503839662 | 0.01780806 | 0.156031046 | Down | 1.74938339 |
| GYPA | -0.614937732 | 0.003258809 | 0.084906991 | Down | 2.486941087 |
| HHIP | 0.575606972 | 0.085232878 | 0.293490206 | NS | 1.069392849 |
| ANAPC10 | -0.005891438 | 0.944742185 | 0.969941522 | NS | 0.024686692 |
| ABCE1 | -0.253353039 | 0.007203834 | 0.111776563 | Down | 2.142436303 |
| OTUD4 | 0.026133016 | 0.682028956 | 0.810483683 | NS | 0.166197187 |
| SMAD1 | 0.223184843 | 0.14527748 | 0.37054456 | NS | 0.837801701 |
| MMAA | 0.014060572 | 0.831410295 | 0.90417202 | NS | 0.080184602 |
| C4orf51 | 0.199452927 | 0.577480926 | NA | NS | 0.238462356 |
| ZNF827 | 0.142641482 | 0.178891263 | 0.40892154 | NS | 0.74741087 |
| LSM6 | -0.054927637 | 0.451132516 | 0.644169642 | NS | 0.345695869 |
| REELD1 | 0.374437379 | 0.039253549 | 0.214616748 | Up | 1.406121068 |
| SLC10A7 | 0.157392657 | 0.005457162 | 0.100547004 | Up | 2.263033147 |
| POU4F2 | 0.938577643 | 0.047917908 | NA | Up | 1.319502147 |
| TTC29 | 0.246792188 | 0.579382393 | NA | NS | 0.237034707 |
| EDNRA | 0.541971416 | 0.199680018 | 0.429206416 | NS | 0.699665392 |
| TMEM184C | -0.116911979 | 0.034937133 | 0.20492492 | Down | 1.456712735 |
| PRMT9 | -0.041772619 | 0.36309721 | 0.57555733 | NS | 0.439977088 |
| ARHGAP10 | 0.10749837 | 0.190926184 | 0.420199064 | NS | 0.719134508 |
| NR3C2 | 0.059422565 | 0.45098139 | 0.644091483 | NS | 0.345841379 |
| IQCM | 0.415563501 | 0.389049651 | 0.596625801 | NS | 0.40999497 |
| DCLK2 | 0.191810481 | 0.385761977 | 0.593890885 | NS | 0.413680581 |
| LRBA | -0.0296084 | 0.593639886 | 0.749772128 | NS | 0.226476927 |
| MAB21L2 | 0.164957526 | 0.120641644 | 0.341313216 | NS | 0.918502753 |
| RPS3A | -0.739447852 | 0.00642406 | 0.107862277 | Down | 2.192190442 |
| SH3D19 | 0.185824291 | 0.343402486 | 0.558486277 | NS | 0.464196565 |
| PRSS48 | 0.903336643 | 0.075689063 | NA | NS | 1.120966873 |
| FHIP1A | 0.134930003 | 0.300658828 | 0.522157313 | NS | 0.52192604 |
| GATB | -0.054592888 | 0.422617525 | 0.622069963 | NS | 0.374052498 |
| FBXW7 | -0.201257452 | 0.039852856 | 0.215796828 | Down | 1.399540545 |
| TMEM154 | 0.011729944 | 0.921359782 | 0.956613552 | NS | 0.035570749 |
| TIGD4 | 0.043651887 | 0.78580298 | 0.876321892 | NS | 0.104686329 |
| ARFIP1 | -0.06250176 | 0.437588176 | 0.633279267 | NS | 0.358934422 |
| FHDC1 | 0.226159109 | 0.230559587 | 0.458835215 | NS | 0.637216815 |
| TRIM2 | -0.029965137 | 0.749952408 | 0.85313978 | NS | 0.124966296 |
| MND1 | 0.054563731 | 0.881472372 | NA | NS | 0.054791295 |
| TMEM131L | 0.012464165 | 0.813882708 | 0.893597093 | NS | 0.089438179 |
| TLR2 | 0.200332545 | 0.129146547 | 0.351110762 | NS | 0.888917201 |
| RNF175 | 0.240813099 | 0.057220805 | 0.250855835 | NS | 1.242446034 |
| SFRP2 | -0.33513536 | 0.164323222 | 0.392074272 | NS | 0.784301059 |
| DCHS2 | 0.285928657 | 0.580629899 | NA | NS | 0.236100604 |
| PLRG1 | -0.172355187 | 0.024889828 | 0.178845349 | Down | 1.603978104 |
| FGB | 0.728236648 | 0.140648143 | NA | NS | 0.851865998 |
| FGA | 0.406175124 | 0.402844195 | NA | NS | 0.394862891 |
| FGG | 0.44375964 | 0.373770914 | NA | NS | 0.427394498 |
| LRAT | 0.728179533 | 0.09984684 | 0.314828493 | NS | 1.000665674 |
| RBM46 | 0.769777784 | 0.094807869 | NA | NS | 1.023155617 |
| NPY2R | 0.558779065 | 0.295689381 | NA | NS | 0.529164272 |
| MAP9 | -0.081738431 | 0.350619727 | 0.564808607 | NS | 0.455163653 |
| GUCY1A1 | -0.039382863 | 0.722887031 | 0.836435041 | NS | 0.140929567 |
| GUCY1B1 | 0.011319249 | 0.927714485 | 0.960164368 | NS | 0.032585662 |
| ASIC5 | 0.428963291 | 0.590933448 | NA | NS | 0.228461427 |
| CTSO | -0.224820568 | 0.012361198 | 0.134807693 | Down | 1.90793944 |
| PDGFC | 0.163885471 | 0.095395261 | 0.309058959 | NS | 1.020473198 |
| GLRB | 0.515101888 | 0.379447917 | NA | NS | 0.420847827 |
| GRIA2 | 0.722001002 | 0.134731629 | 0.358293518 | NS | 0.870530438 |
| GASK1B | 0.16514503 | 0.130286527 | 0.352156503 | NS | 0.885100492 |
| TMEM144 | 0.341312885 | 0.003084634 | 0.083203163 | Up | 2.510796417 |
| RXFP1 | 0.321546349 | 0.38184744 | 0.591317506 | NS | 0.418110117 |
| C4orf46 | -0.066305098 | 0.41290515 | 0.614960916 | NS | 0.3841497 |
| ETFDH | 0.078925995 | 0.086633116 | 0.295984568 | NS | 1.062316065 |
| PPID | 0.199204497 | 5.75E-04 | 0.040235668 | Up | 3.240695106 |
| FNIP2 | -0.07127276 | 0.171331899 | 0.399868249 | NS | 0.766161771 |
| C4orf45 | 0.075376121 | 0.737811621 | 0.845697925 | NS | 0.132054509 |
| RAPGEF2 | -0.130034268 | 0.129923608 | 0.352006444 | NS | 0.886311927 |
| FSTL5 | 0.597949856 | 0.200331969 | NA | NS | 0.698249741 |
| NAF1 | 0.035790769 | 0.611506344 | 0.76220302 | NS | 0.213599033 |
| NPY1R | 0.185248473 | 0.719405241 | NA | NS | 0.143026403 |
| NPY5R | 1.013145016 | 0.028263783 | NA | Up | 1.548769713 |
| TKTL2 | 0.519929083 | 0.068582992 | NA | NS | 1.163783572 |
| TMA16 | -0.14999995 | 0.026115534 | 0.182828778 | Down | 1.583101089 |
| MARCHF1 | -0.021875135 | 0.778945333 | 0.871788399 | NS | 0.108493021 |
| SMIM31 | 0.645090687 | 0.20967755 | NA | NS | 0.678448067 |
| APELA | 0.544881914 | 0.281805205 | NA | NS | 0.550050989 |
| TRIM61 | -0.004556923 | 0.973411109 | 0.984936874 | NS | 0.011703702 |
| TRIM60 | 1.036634244 | 0.030846133 | NA | Up | 1.510799274 |
| TRIM75P | 0.392904161 | 0.25739719 | NA | NS | 0.589396199 |
| TMEM192 | -0.039464108 | 0.510421754 | 0.689473737 | NS | 0.292070824 |
| KLHL2 | 0.104887513 | 0.325389182 | 0.543906507 | NS | 0.487596889 |
| GK3P | 0.310247813 | 0.035526899 | 0.206049964 | Up | 1.4494427 |
| MSMO1 | -0.163981054 | 0.120125527 | 0.340734879 | NS | 0.920364693 |
| CPE | 0.603177177 | 0.118990363 | NA | NS | 0.924488209 |
| TLL1 | 0.35189724 | 0.472584428 | 0.661038751 | NS | 0.325520593 |
| SPOCK3 | 0.41804431 | 0.168032665 | 0.396158544 | NS | 0.774606284 |
| ANXA10 | 0.249691901 | 0.199974467 | NA | NS | 0.699025453 |
| DDX60 | 0.132159126 | 0.220103408 | 0.449270199 | NS | 0.657373234 |
| DDX60L | 0.23762468 | 0.02354637 | 0.17423553 | Up | 1.628076036 |
| PALLD | 0.13803883 | 0.106113685 | 0.322426185 | NS | 0.974228603 |
| CBR4 | -0.017501901 | 0.645963734 | 0.786192259 | NS | 0.189791863 |
| SH3RF1 | 0.128166888 | 0.280080453 | 0.50348007 | NS | 0.5527172 |
| NEK1 | -0.102298865 | 0.384351393 | 0.593034804 | NS | 0.415271541 |
| CLCN3 | -0.193980207 | 0.027080715 | 0.185578254 | Down | 1.567339881 |
| HPF1 | -0.18996222 | 0.052529294 | 0.242394626 | NS | 1.279598437 |
| MFAP3L | -0.156217773 | 0.202300392 | 0.432473031 | NS | 0.694003276 |
| AADAT | 0.655386832 | 0.135183337 | NA | NS | 0.869076838 |
| GALNTL6 | 0.206497936 | 0.471588953 | 0.660409087 | NS | 0.326436377 |
| GALNT7 | 0.045719911 | 0.509655079 | 0.688823879 | NS | 0.292723643 |
| HMGB2 | -0.153311194 | 0.201532738 | 0.431436042 | NS | 0.695654394 |
| SAP30 | -0.168391843 | 0.140852644 | 0.365265155 | NS | 0.851234995 |
| SCRG1 | 0.203642777 | 0.0834604 | 0.290969351 | NS | 1.078519538 |
| HAND2 | 1.087705453 | 0.047758917 | NA | Up | 1.320945532 |
| FBXO8 | -0.079566095 | 0.211644055 | 0.441324971 | NS | 0.674393925 |
| CEP44 | -0.152321097 | 0.013164959 | 0.137679829 | Down | 1.880580496 |
| HPGD | 0.06379335 | 0.587930614 | 0.746343713 | NS | 0.230673925 |
| GLRA3 | 0.452045083 | 0.1090363 | 0.327066056 | NS | 0.962428895 |
| ADAM29 | 0.116942264 | 0.640940422 | 0.782522144 | NS | 0.193182338 |
| GPM6A | -0.149394193 | 0.494943344 | 0.677861049 | NS | 0.305444512 |
| WDR17 | -0.036275637 | 0.795416092 | 0.881887737 | NS | 0.099405627 |
| SPATA4 | 1.177908775 | 0.007216827 | NA | Up | 2.141653696 |
| ASB5 | 0.305291911 | 0.54121851 | NA | NS | 0.266627359 |
| SPCS3 | -0.163987095 | 0.036073218 | 0.207679449 | Down | 1.442815109 |
| VEGFC | 0.432953229 | 0.143397679 | NA | NS | 0.843457879 |
| NEIL3 | -0.738863732 | 9.24E-04 | 0.050757919 | Down | 3.034386022 |
| AGA | -0.131863711 | 0.101208099 | 0.31690838 | NS | 0.994784731 |
| TENM3 | 0.640487313 | 0.121888026 | 0.342765174 | NS | 0.914038956 |
| DCTD | -0.090374041 | 0.179785133 | 0.409922597 | NS | 0.745246223 |
| WWC2 | 0.125777918 | 0.097203185 | 0.311565216 | NS | 1.012319506 |
| CLDN24 | -0.12431527 | 0.645850209 | NA | NS | 0.189868196 |
| CDKN2AIP | -0.156131176 | 0.071640476 | 0.27295712 | NS | 1.144841537 |
| ING2 | -0.132952605 | 0.067066842 | 0.266845867 | NS | 1.173492144 |
| RWDD4 | -0.17540276 | 0.028649615 | 0.189026296 | Down | 1.542881204 |
| TRAPPC11 | -0.110391105 | 0.016695276 | 0.152272089 | Down | 1.777406393 |
| STOX2 | 0.46448606 | 0.120518308 | 0.341246784 | NS | 0.918946975 |
| ENPP6 | 0.547203659 | 0.169504776 | NA | NS | 0.77081806 |
| IRF2 | 0.127410912 | 0.062063858 | 0.258716142 | NS | 1.207161229 |
| CASP3 | -0.044051823 | 0.646455055 | 0.786418447 | NS | 0.189461664 |
| PRIMPOL | -0.215251886 | 0.017684639 | 0.155670827 | Down | 1.752403802 |
| CENPU | -0.141457705 | 0.317420173 | 0.536894889 | NS | 0.498365476 |
| ACSL1 | 0.240012996 | 0.134789561 | 0.358364352 | NS | 0.870343741 |
| HELT | 1.307311354 | 0.016409982 | NA | Up | 1.784891903 |
| SLC25A4 | -0.023255518 | 0.714994813 | 0.831377499 | NS | 0.145697109 |
| CFAP97 | -0.194378408 | 0.012600957 | 0.135545097 | Down | 1.899596459 |
| SNX25 | -0.069441097 | 0.323579028 | 0.54228177 | NS | 0.490019634 |
| LRP2BP | 0.107259925 | 0.177779343 | 0.407680235 | NS | 0.750118704 |
| ANKRD37 | 0.137813444 | 0.170368977 | 0.39869447 | NS | 0.768609484 |
| UFSP2 | -0.243547081 | 0.001151768 | 0.055068459 | Down | 2.938634973 |
| C4orf47 | 0.562704415 | 0.109628519 | NA | NS | 0.960076453 |
| CCDC110 | 0.063272879 | 0.834507197 | NA | NS | 0.078569914 |
| PDLIM3 | 0.700742502 | 0.121212199 | 0.34187214 | NS | 0.91645367 |
| SORBS2 | 0.338859982 | 0.300060189 | 0.521592292 | NS | 0.522791622 |
| TLR3 | -0.068116409 | 0.570346379 | 0.732392713 | NS | 0.243861311 |
| FAM149A | 0.638548359 | 0.073259804 | 0.275354648 | NS | 1.135134246 |
| CYP4V2 | 0.041858951 | 0.504962707 | 0.685516059 | NS | 0.296740695 |
| KLKB1 | -0.144179233 | 0.26727662 | 0.493205152 | NS | 0.57303903 |
| F11 | 0.266744589 | 0.46886829 | NA | NS | 0.328949138 |
| MTNR1A | 0.414474537 | 0.501184644 | NA | NS | 0.300002244 |
| FAT1 | 0.203306298 | 0.331475448 | 0.54839717 | NS | 0.479548634 |
| ZFP42 | 0.684825488 | 0.235585389 | NA | NS | 0.627851648 |
| TRIML2 | 0.17629255 | 0.727624637 | NA | NS | 0.138092604 |
| TRIML1 | 0.290559566 | 0.585965728 | NA | NS | 0.232127784 |
| FRG1 | -0.269179144 | 0.004750584 | 0.095617668 | Down | 2.323252954 |
| DUX4 | 0.245372019 | 0.717338809 | NA | NS | 0.144275673 |
| PLEKHG4B | 0.85832574 | 0.026359762 | 0.183162888 | Up | 1.579058511 |
| LRRC14B | 0.730574649 | 0.084260503 | NA | NS | 1.074375951 |
| CCDC127 | -0.140490995 | 0.007781107 | 0.114529629 | Down | 2.108958595 |
| SDHA | -0.019431041 | 0.67959769 | 0.809073468 | NS | 0.167748106 |
| PDCD6 | 0.078649467 | 0.320248763 | 0.539074418 | NS | 0.494512539 |
| AHRR | 0.701693225 | 0.018597819 | NA | Up | 1.730537984 |
| EXOC3 | 0.022918566 | 0.744330099 | 0.85015041 | NS | 0.128234419 |
| SLC9A3 | 0.328187154 | 0.046316166 | 0.229298064 | Up | 1.334267394 |
| CEP72 | -0.125385947 | 0.181174905 | 0.411144241 | NS | 0.741901957 |
| TPPP | 0.118435684 | 0.396944051 | 0.602161483 | NS | 0.401270703 |
| ZDHHC11B | 0.394665115 | 0.03850337 | 0.213464789 | Up | 1.41450126 |
| ZDHHC11 | -0.128713252 | 0.480570615 | 0.667338513 | NS | 0.318242788 |
| BRD9 | -0.104563524 | 0.029131593 | 0.190232923 | Down | 1.535635765 |
| TRIP13 | 0.089081401 | 0.616626821 | 0.765626861 | NS | 0.209977589 |
| SLC12A7 | -0.095935242 | 0.56964948 | 0.73208388 | NS | 0.244392295 |
| SLC6A19 | 0.00678222 | 0.980964016 | 0.98882209 | NS | 0.008346923 |
| SLC6A18 | 0.727976435 | 0.165990079 | NA | NS | 0.779917868 |
| TERT | 0.877838941 | 0.014672516 | 0.145153331 | Up | 1.83349541 |
| CLPTM1L | -0.19296108 | 0.011236129 | 0.13003023 | Down | 1.949383285 |
| LPCAT1 | -0.082728465 | 0.276664332 | 0.500969068 | NS | 0.558046827 |
| MRPL36 | -0.234819377 | 0.055351722 | 0.248604264 | NS | 1.256868862 |
| NDUFS6 | -0.140113833 | 0.083275018 | 0.290593397 | NS | 1.079485264 |
| IRX4 | 1.009240329 | 0.050520709 | NA | NS | 1.296530561 |
| IRX2 | 1.320336236 | 0.016654694 | NA | Up | 1.778463346 |
| IRX1 | 1.40332749 | 0.003525356 | NA | Up | 2.45279699 |
| ADAMTS16 | 0.54006815 | 0.07143485 | 0.272521361 | NS | 1.146089862 |
| ICE1 | 0.048301195 | 0.290541843 | 0.513504345 | NS | 0.536791312 |
| MED10 | -0.026369614 | 0.641393038 | 0.782894954 | NS | 0.192875759 |
| UBE2QL1 | 0.883431885 | 0.058945917 | NA | NS | 1.229546273 |
| NSUN2 | -0.192198305 | 8.97E-04 | 0.050203348 | Down | 3.047417421 |
| SRD5A1 | 0.113250968 | 0.046352934 | 0.229315052 | Up | 1.333922768 |
| TENT4A | 0.049852356 | 0.496427343 | 0.679119341 | NS | 0.304144306 |
| ADCY2 | 0.514563656 | 0.224778052 | 0.453117414 | NS | 0.648246097 |
| C5orf49 | 1.296836926 | 0.008417429 | NA | Up | 2.074820515 |
| MTRR | -0.068106603 | 0.193227412 | 0.422583479 | NS | 0.713931263 |
| FASTKD3 | -0.042117087 | 0.515820986 | 0.693487378 | NS | 0.287500993 |
| SEMA5A | 0.389960738 | 0.014120587 | 0.142399682 | Up | 1.850147257 |
| TAS2R1 | 0.066359449 | 0.908320937 | NA | NS | 0.041760675 |
| ATPSCKMT | 0.006211149 | 0.908930292 | 0.949563038 | NS | 0.041469423 |
| CCT5 | -0.183721243 | 0.017370878 | 0.154692513 | Down | 1.760178218 |
| CMBL | 0.275919822 | 0.298888229 | 0.520339481 | NS | 0.524491189 |
| MARCHF6 | 0.05350819 | 0.586855079 | 0.745392188 | NS | 0.231469132 |
| ROPN1L | 0.274364319 | 0.048661465 | 0.233910643 | Up | 1.312814823 |
| ANKRD33B | 0.372778834 | 0.00104886 | 0.052878189 | Up | 2.97928242 |
| DAP | 0.044910348 | 0.742084777 | 0.848841757 | NS | 0.129546477 |
| CTNND2 | 0.33160711 | 0.328668012 | 0.546356595 | NS | 0.483242562 |
| DNAH5 | 0.461669702 | 0.241114725 | 0.469119823 | NS | 0.617776266 |
| TRIO | 0.062119439 | 0.471193956 | 0.660178881 | NS | 0.326800289 |
| OTULINL | -0.051044709 | 0.485996229 | 0.671550961 | NS | 0.313367101 |
| OTULIN | 0.01104695 | 0.827255165 | 0.901708524 | NS | 0.082360513 |
| ANKH | 0.122482034 | 0.275490008 | 0.500040883 | NS | 0.559894148 |
| FBXL7 | 0.963281966 | 0.053636601 | NA | NS | 1.270538754 |
| MARCHF11 | 0.494732593 | 0.439363833 | NA | NS | 0.357175695 |
| ZNF622 | -0.149756293 | 0.033148898 | 0.200787109 | Down | 1.479530906 |
| RETREG1 | -0.030256906 | 0.715239207 | 0.831519115 | NS | 0.145548687 |
| MYO10 | 0.324850012 | 0.081427713 | 0.287767953 | NS | 1.089227762 |
| BASP1 | 0.061365446 | 0.66253248 | 0.797755986 | NS | 0.178792826 |
| LINC02218 | 0.093147383 | 0.544596023 | 0.7135756 | NS | 0.263925535 |
| H3Y2 | 0.210318143 | 0.26399093 | 0.490237234 | NS | 0.578410994 |
| TAF11L2 | 0.205917351 | 0.434812624 | 0.630833536 | NS | 0.361697856 |
| TAF11L11 | 1.247006422 | 0.029615183 | NA | Up | 1.528485573 |
| TAF11L12 | 0.46629976 | 0.458818173 | NA | NS | 0.338359389 |
| TAF11L14 | 0.545482785 | 0.502915229 | NA | NS | 0.298505213 |
| H3Y1 | 1.234495645 | 0.073100259 | NA | NS | 1.136081084 |
| CDH18 | 0.386151706 | 0.449782018 | 0.643129524 | NS | 0.346997911 |
| CDH12 | 0.378043716 | 0.438502336 | 0.633805873 | NS | 0.358028089 |
| PRDM9 | 0.478024148 | 0.361428429 | NA | NS | 0.441977689 |
| CDH10 | 0.633291917 | 0.264205991 | NA | NS | 0.578057339 |
| CDH9 | 0.264299348 | 0.583271238 | NA | NS | 0.234129438 |
| CDH6 | 0.660354218 | 0.058244298 | 0.252197542 | NS | 1.234746582 |
| DROSHA | 0.062492101 | 0.423325058 | 0.622593337 | NS | 0.373326023 |
| C5orf22 | -0.055706574 | 0.391980632 | 0.598738083 | NS | 0.406735391 |
| PDZD2 | 0.163782983 | 0.114780774 | 0.333634889 | NS | 0.940130851 |
| GOLPH3 | -0.26129221 | 0.001507571 | 0.062591307 | Down | 2.821722155 |
| MTMR12 | -0.029451357 | 0.575784496 | 0.736805974 | NS | 0.239740034 |
| ZFR | -0.142504031 | 0.005119476 | 0.09836094 | Down | 2.290774486 |
| SUB1 | -0.188464447 | 0.129487241 | 0.351485827 | NS | 0.887773021 |
| NPR3 | 0.174602659 | 0.45157562 | 0.644360427 | NS | 0.345269513 |
| TARS1 | -0.118861677 | 0.158405902 | 0.385866275 | NS | 0.800228641 |
| ADAMTS12 | 0.389120714 | 0.343400529 | 0.558486277 | NS | 0.46419904 |
| SLC45A2 | 0.140358674 | 0.62278546 | NA | NS | 0.205661535 |
| AMACR | 0.096324882 | 0.307713778 | 0.528573145 | NS | 0.511853058 |
| C1QTNF3-AMACR | 0.038519321 | 0.862061383 | NA | NS | 0.064461809 |
| C1QTNF3 | 0.095248096 | 0.26514515 | 0.491370304 | NS | 0.576516313 |
| RAI14 | 0.298653188 | 0.226374596 | 0.455054069 | NS | 0.645172311 |
| TTC23L | 0.232996883 | 0.348743703 | 0.562790769 | NS | 0.457493626 |
| RAD1 | -0.039872098 | 0.482220835 | 0.66853282 | NS | 0.316754029 |
| BRIX1 | -0.27827591 | 0.015023811 | 0.146973997 | Down | 1.823219903 |
| DNAJC21 | -0.104652367 | 0.039934977 | 0.215796828 | Down | 1.398646558 |
| AGXT2 | 0.331151119 | 0.451695136 | NA | NS | 0.345154586 |
| PRLR | 0.184816728 | 0.185342411 | 0.41546259 | NS | 0.732025192 |
| SPEF2 | 0.05940589 | 0.457615524 | 0.64913662 | NS | 0.339499251 |
| IL7R | -0.084330899 | 0.283486316 | 0.506387725 | NS | 0.547467899 |
| CAPSL | 0.894936842 | 0.067800975 | NA | NS | 1.168764062 |
| UGT3A1 | 0.484887559 | 0.27525787 | 0.499918696 | NS | 0.560260255 |
| UGT3A2 | 0.873491102 | 0.023633727 | NA | Up | 1.626467785 |
| LMBRD2 | -0.028494068 | 0.591489364 | 0.748413779 | NS | 0.228053061 |
| SKP2 | 0.003156058 | 0.950355454 | 0.973174183 | NS | 0.022113928 |
| NADK2 | -0.086872935 | 0.115514131 | 0.334479283 | NS | 0.937364886 |
| RANBP3L | 0.371390947 | 0.04993703 | 0.236635186 | Up | 1.301577294 |
| SLC1A3 | 0.168596783 | 0.213536123 | 0.443180155 | NS | 0.670528646 |
| NIPBL | 0.025929285 | 0.715210463 | 0.831519115 | NS | 0.145566141 |
| CPLANE1 | -0.068877813 | 0.32795628 | 0.545728019 | NS | 0.484184048 |
| NUP155 | -0.11680451 | 0.015198369 | 0.147378943 | Down | 1.818203006 |
| WDR70 | -0.068255743 | 0.131963721 | 0.3547222 | NS | 0.879545446 |
| GDNF | 0.654114277 | 0.151972378 | NA | NS | 0.818235341 |
| EGFLAM | 0.478805977 | 0.297598415 | 0.519257109 | NS | 0.526369386 |
| LIFR | 0.516889437 | 0.146253047 | 0.371713762 | NS | 0.834895078 |
| OSMR | 0.238022725 | 0.017631892 | 0.155449116 | Up | 1.753701072 |
| RICTOR | 0.048905229 | 0.578100432 | 0.738214279 | NS | 0.237996706 |
| FYB1 | 0.181762701 | 0.016111062 | 0.150234341 | Up | 1.792875832 |
| C9 | 0.212709163 | 0.582207085 | NA | NS | 0.234922514 |
| DAB2 | -0.020801403 | 0.830596494 | 0.903626518 | NS | 0.080609906 |
| TTC33 | -0.111325866 | 0.098256785 | 0.313029559 | NS | 1.007637451 |
| PTGER4 | -0.166035847 | 0.014906123 | 0.146349507 | Down | 1.826635286 |
| PRKAA1 | -0.094887731 | 0.04492614 | 0.226679546 | NS | 1.347500893 |
| RPL37 | -0.260641938 | 0.06133783 | 0.257517688 | NS | 1.212271591 |
| CARD6 | 0.281776902 | 5.85E-04 | 0.040600896 | Up | 3.232823851 |
| C7 | 0.17164384 | 0.713460483 | 0.830478881 | NS | 0.146630077 |
| MROH2B | 0.613249423 | 0.17451066 | 0.403823699 | NS | 0.758178038 |
| C6 | 0.485854495 | 0.321763789 | 0.540212175 | NS | 0.492462833 |
| PLCXD3 | 0.530160642 | 0.22905553 | 0.457671413 | NS | 0.640059219 |
| OXCT1 | -0.059413227 | 0.311463836 | 0.531192767 | NS | 0.506592372 |
| C5orf51 | 0.106780782 | 0.05625005 | 0.249221499 | NS | 1.249877088 |
| FBXO4 | -0.141157294 | 0.143215583 | 0.368154582 | NS | 0.844009725 |
| GHR | 0.579379238 | 0.103584407 | 0.319328319 | NS | 0.984705617 |
| CCDC152 | -0.090001531 | 0.49470455 | 0.677817745 | NS | 0.305654095 |
| SELENOP | 0.067537087 | 0.523391797 | 0.699066057 | NS | 0.281173088 |
| ANXA2R | -0.147347267 | 0.099876482 | 0.314828493 | NS | 1.000536763 |
| ZNF131 | -0.187821225 | 0.019062136 | 0.160441392 | Down | 1.719828446 |
| NIM1K | 0.579922554 | 0.085811666 | 0.294509032 | NS | 1.066453667 |
| HMGCS1 | -0.046495773 | 0.416086182 | 0.617223125 | NS | 0.380816707 |
| CCL28 | -0.165454412 | 0.047099231 | 0.230730823 | Down | 1.32698618 |
| TMEM267 | -0.03420944 | 0.685051632 | 0.812777935 | NS | 0.164276695 |
| C5orf34 | 0.072290164 | 0.383592854 | 0.592463989 | NS | 0.416129492 |
| PAIP1 | -0.238505159 | 0.009990312 | 0.125713252 | Down | 2.00042096 |
| NNT | -0.069213661 | 0.264656594 | 0.490862108 | NS | 0.577317281 |
| FGF10 | 0.450174669 | 0.35996098 | NA | NS | 0.443744574 |
| MRPS30 | -0.095867679 | 0.156013118 | 0.383185333 | NS | 0.806838883 |
| HCN1 | 0.467427459 | 0.318982782 | 0.538089997 | NS | 0.496232758 |
| EMB | -0.085898531 | 0.263754981 | 0.490023249 | NS | 0.578799329 |
| PARP8 | 0.039133211 | 0.603608982 | 0.756766459 | NS | 0.219244306 |
| ISL1 | 0.790948225 | 0.09657162 | NA | NS | 1.015150483 |
| PELO | 0.198016886 | 0.022174304 | 0.170529186 | Up | 1.654149997 |
| ITGA1 | 0.093958762 | 0.495154542 | 0.678069201 | NS | 0.305259233 |
| ITGA2 | 0.265897858 | 0.009484907 | 0.122561596 | Up | 2.022966902 |
| MOCS2 | 0.005893949 | 0.934509894 | 0.96384353 | NS | 0.029416096 |
| FST | 0.463909277 | 0.284866667 | NA | NS | 0.545358366 |
| NDUFS4 | -0.369585149 | 0.020577847 | 0.166586724 | Down | 1.686600074 |
| ARL15 | 0.076831909 | 0.214231321 | 0.443419982 | NS | 0.669117035 |
| HSPB3 | 0.289750399 | 0.560284863 | NA | NS | 0.25159111 |
| SNX18 | -0.06131831 | 0.444704993 | 0.63886408 | NS | 0.351927994 |
| ESM1 | -0.326851062 | 0.056991 | 0.250513504 | NS | 1.244193725 |
| GZMK | -0.341936136 | 0.029401822 | 0.191078472 | Down | 1.531625751 |
| GZMA | -0.468741135 | 0.008744746 | 0.118782974 | Down | 2.058252793 |
| CDC20B | 0.424359288 | 0.103821887 | 0.319643728 | NS | 0.983711084 |
| GPX8 | 0.671944355 | 0.222917586 | NA | NS | 0.651855668 |
| MCIDAS | 1.481296049 | 0.002903667 | NA | Up | 2.537053257 |
| CCNO | 0.903501836 | 0.077380176 | NA | NS | 1.111370289 |
| DHX29 | -0.166531302 | 0.031971505 | 0.198142899 | Down | 1.495236913 |
| MTREX | -0.146847492 | 0.015269889 | 0.147395039 | Down | 1.816164134 |
| PLPP1 | -0.006023575 | 0.944220554 | 0.969563356 | NS | 0.02492655 |
| SLC38A9 | -0.044202657 | 0.404033303 | 0.607841471 | NS | 0.393582836 |
| DDX4 | 0.385658791 | 0.40301664 | 0.607189467 | NS | 0.394677022 |
| IL31RA | 0.324258926 | 0.150532668 | 0.377023092 | NS | 0.822369242 |
| IL6ST | 0.006790364 | 0.926984877 | 0.959843008 | NS | 0.032927351 |
| ANKRD55 | 0.136438885 | 0.268397187 | 0.494158082 | NS | 0.57122204 |
| MAP3K1 | -0.024920811 | 0.666242787 | 0.800288068 | NS | 0.17636748 |
| SETD9 | -0.154484527 | 0.221559495 | 0.450355512 | NS | 0.654509633 |
| MIER3 | -0.107563715 | 0.158615773 | 0.386161204 | NS | 0.799653627 |
| GPBP1 | -0.191432275 | 0.01362865 | 0.140154175 | Down | 1.865547153 |
| ACTBL2 | 0.43911583 | 0.407529133 | NA | NS | 0.38984134 |
| PLK2 | -0.035820299 | 0.817636077 | 0.896257188 | NS | 0.087439954 |
| GAPT | 0.013165523 | 0.90980281 | 0.950032257 | NS | 0.041052726 |
| RAB3C | 0.394394054 | 0.09068913 | 0.301577239 | NS | 1.042444766 |
| PDE4D | 0.048733057 | 0.314388526 | 0.533741944 | NS | 0.502533312 |
| DEPDC1B | -0.028590123 | 0.845688565 | 0.913247565 | NS | 0.072789541 |
| ELOVL7 | 0.03104247 | 0.822413828 | 0.898827081 | NS | 0.084909596 |
| ERCC8 | 0.01482571 | 0.76021489 | 0.860029708 | NS | 0.119063629 |
| NDUFAF2 | -0.040921252 | 0.566708237 | 0.730251058 | NS | 0.246640475 |
| SMIM15 | -0.232045514 | 0.01605981 | 0.150123175 | Down | 1.794259584 |
| ZSWIM6 | 0.180748281 | 0.002047693 | 0.071162764 | Up | 2.688735187 |
| KIF2A | -0.083736703 | 0.112657342 | 0.331203909 | NS | 0.948240499 |
| DIMT1 | -0.023604852 | 0.628969826 | 0.773986725 | NS | 0.201370189 |
| IPO11 | -0.018000185 | 0.701835952 | 0.82330825 | NS | 0.153764389 |
| LRRC70 | 0.036733636 | 0.761408417 | 0.860657491 | NS | 0.118382327 |
| HTR1A | 0.637559022 | 0.214740143 | NA | NS | 0.668086762 |
| RNF180 | 0.371691675 | 0.401620213 | NA | NS | 0.396184438 |
| RGS7BP | 0.817333311 | 0.07296373 | NA | NS | 1.136892972 |
| SHISAL2B | 0.660908044 | 0.050846901 | NA | NS | 1.293735514 |
| SREK1IP1 | -0.121461806 | 0.077182337 | 0.281514555 | NS | 1.112482073 |
| CWC27 | 0.060776466 | 0.287503816 | 0.510589731 | NS | 0.541356387 |
| ADAMTS6 | -0.105461432 | 0.312674365 | 0.532366984 | NS | 0.504907724 |
| CENPK | -0.45554642 | 0.029418128 | 0.191130203 | Down | 1.531384968 |
| PPWD1 | -0.079145936 | 0.11069621 | 0.329185788 | NS | 0.955867247 |
| TRIM23 | -0.167441603 | 0.059169849 | 0.253813051 | NS | 1.227899541 |
| TRAPPC13 | -0.03062281 | 0.62764868 | 0.773233462 | NS | 0.20228338 |
| SHLD3 | -0.141533297 | 0.183563983 | 0.413733592 | NS | 0.736212528 |
| SGTB | -0.085742224 | 0.163021889 | 0.390740691 | NS | 0.787754078 |
| NLN | -0.090498035 | 0.313956358 | 0.533518476 | NS | 0.503130718 |
| ERBIN | -0.155276283 | 0.009175652 | 0.121188457 | Down | 2.037363063 |
| SREK1 | -0.018842624 | 0.71433625 | 0.831075885 | NS | 0.14609731 |
| MAST4 | 0.064994303 | 0.362781473 | 0.575295755 | NS | 0.440354901 |
| CD180 | -0.036955192 | 0.687247037 | 0.814178911 | NS | 0.162887124 |
| PIK3R1 | -0.076963729 | 0.126661313 | 0.348597283 | NS | 0.897356014 |
| SLC30A5 | -0.164278685 | 0.017237813 | 0.15421109 | Down | 1.763517839 |
| CCNB1 | -0.461828921 | 0.006703364 | 0.108957018 | Down | 2.17370721 |
| CENPH | -0.480841107 | 0.004849692 | 0.096580558 | Down | 2.31428586 |
| MRPS36 | -0.258407861 | 0.024209838 | 0.176352962 | Down | 1.616008114 |
| CDK7 | -0.165069435 | 0.033141816 | 0.200787109 | Down | 1.479623702 |
| CCDC125 | -0.082206207 | 0.297930619 | 0.519509867 | NS | 0.525884861 |
| AK6 | -0.411999421 | 0.046618716 | 0.229855333 | Down | 1.331439691 |
| TAF9 | -0.388674941 | 0.023508064 | 0.174120857 | Down | 1.62878313 |
| RAD17 | -0.284125758 | 0.00406667 | 0.090508199 | Down | 2.390761049 |
| MARVELD2 | 0.287052025 | 0.444915735 | NA | NS | 0.351722234 |
| OCLN | 0.431021167 | 0.199397528 | NA | NS | 0.700280231 |
| GTF2H2C | -0.171708284 | 0.089864139 | 0.30028957 | NS | 1.046413585 |
| SMN2 | 0.177251613 | 0.460586264 | NA | NS | 0.336689018 |
| SERF1A | 0.250234136 | 0.666576761 | NA | NS | 0.176149831 |
| SMN1 | -0.112133511 | 0.352861944 | 0.566429119 | NS | 0.452395178 |
| NAIP | -0.010960993 | 0.946310507 | 0.971046941 | NS | 0.023966338 |
| GTF2H2 | -0.015358152 | 0.929588619 | 0.961626024 | NS | 0.031709202 |
| BDP1 | -0.115203737 | 0.052158181 | 0.241318517 | NS | 1.282677563 |
| MCCC2 | 0.07615469 | 0.131769064 | 0.354444963 | NS | 0.880186537 |
| CARTPT | 1.129036942 | 0.074129107 | NA | NS | 1.130011233 |
| MAP1B | 0.388839048 | 0.08430395 | 0.29207932 | NS | 1.074152077 |
| MRPS27 | -0.09944075 | 0.091112732 | 0.302283613 | NS | 1.040420933 |
| PTCD2 | 0.13059454 | 0.038929202 | 0.214288889 | Up | 1.409724499 |
| ZNF366 | 0.279414772 | 0.077243063 | 0.281620438 | NS | 1.112140514 |
| TNPO1 | -0.03788828 | 0.448944387 | 0.642504366 | NS | 0.347807454 |
| FCHO2 | -0.035956516 | 0.793969507 | 0.880983026 | NS | 0.100196177 |
| TMEM171 | 0.404994961 | 0.143125172 | NA | NS | 0.844283978 |
| TMEM174 | 1.431195106 | 0.009100115 | NA | Up | 2.040953097 |
| FOXD1 | 0.746775801 | 0.036651382 | NA | Up | 1.435909643 |
| BTF3 | -0.28218359 | 0.025926757 | 0.1823632 | Down | 1.586251811 |
| ANKRA2 | -0.073318311 | 0.155952804 | 0.383185333 | NS | 0.807006813 |
| UTP15 | -0.133670195 | 0.032773654 | 0.19983552 | Down | 1.484475142 |
| ARHGEF28 | 0.15239655 | 0.383200856 | 0.592417038 | NS | 0.41657353 |
| ENC1 | 0.09667869 | 0.417308795 | 0.618297974 | NS | 0.379542463 |
| HEXB | -0.138746145 | 0.102171274 | 0.31805444 | NS | 0.990671191 |
| GFM2 | -0.047067111 | 0.310662788 | 0.530519661 | NS | 0.507710764 |
| NSA2 | -0.256906626 | 0.046435051 | 0.229390756 | Down | 1.33315407 |
| FAM169A | -0.10530833 | 0.17069313 | 0.399000229 | NS | 0.767783958 |
| GCNT4 | -0.072680128 | 0.505442524 | 0.685923482 | NS | 0.296328223 |
| ANKRD31 | 0.162093663 | 0.232158452 | 0.460416771 | NS | 0.6342155 |
| HMGCR | 0.023253345 | 0.76131428 | 0.860657491 | NS | 0.118436024 |
| CERT1 | 0.06569297 | 0.178293709 | 0.408326093 | NS | 0.748863979 |
| POLK | -0.076989176 | 0.173517157 | 0.402525038 | NS | 0.760657576 |
| ANKDD1B | 0.228632309 | 0.015879338 | 0.149473321 | Up | 1.799167618 |
| POC5 | -0.030971606 | 0.529723168 | 0.704071643 | NS | 0.275951033 |
| SV2C | 0.251831259 | 0.257771757 | 0.484730205 | NS | 0.588764669 |
| IQGAP2 | -0.027206283 | 0.693277561 | 0.817788674 | NS | 0.159092856 |
| F2RL2 | 0.199092272 | 0.082949187 | 0.290260272 | NS | 1.081187868 |
| F2R | -0.062096677 | 0.57709739 | 0.737669138 | NS | 0.238750889 |
| F2RL1 | -0.019296362 | 0.89923318 | 0.943953719 | NS | 0.046127677 |
| S100Z | -0.078096501 | 0.434279739 | 0.630643243 | NS | 0.362230432 |
| CRHBP | 0.267011209 | 0.145069806 | 0.370347641 | NS | 0.838422971 |
| AGGF1 | -0.053687008 | 0.413552178 | 0.615486243 | NS | 0.383469688 |
| ZBED3 | 0.095383197 | 0.381555109 | 0.591109727 | NS | 0.418442727 |
| PDE8B | 0.019146566 | 0.867977828 | 0.9255682 | NS | 0.061491368 |
| WDR41 | -0.127499686 | 0.096130867 | 0.310224046 | NS | 1.017137141 |
| OTP | 1.102407003 | 0.019918571 | NA | Up | 1.700741832 |
| TBCA | -0.229030263 | 0.12495092 | 0.346400199 | NS | 0.903260542 |
| AP3B1 | -0.03115266 | 0.404832942 | 0.60860084 | NS | 0.392724155 |
| SCAMP1 | -0.137903311 | 0.063939452 | 0.262496805 | NS | 1.194231093 |
| LHFPL2 | 0.085785363 | 0.437916336 | 0.633431322 | NS | 0.358608854 |
| ARSB | 0.149583969 | 0.019756098 | 0.1632675 | Up | 1.704298834 |
| DMGDH | 0.478853843 | 0.073523862 | 0.275712399 | NS | 1.133571687 |
| BHMT2 | 0.355707299 | 0.362761749 | NA | NS | 0.440378513 |
| BHMT | 0.686010231 | 0.059081102 | NA | NS | 1.22855141 |
| JMY | 0.050713512 | 0.327142675 | 0.545260243 | NS | 0.4852628 |
| HOMER1 | -0.166385343 | 0.130821326 | 0.352977145 | NS | 0.883321453 |
| TENT2 | -0.021695942 | 0.632420732 | 0.77606492 | NS | 0.198993902 |
| CMYA5 | 0.278592952 | 0.17000686 | 0.398309642 | NS | 0.769533554 |
| MTX3 | 0.012785912 | 0.838215317 | 0.90890645 | NS | 0.076644407 |
| THBS4 | 0.220302701 | 0.05831761 | 0.252212565 | NS | 1.234200285 |
| SERINC5 | 0.152106385 | 0.033440307 | 0.201567051 | Up | 1.475729743 |
| SPZ1 | 0.423062566 | 0.449339981 | NA | NS | 0.347424937 |
| ZFYVE16 | 0.013518582 | 0.878028765 | 0.931434766 | NS | 0.056491256 |
| FAM151B | 0.168338442 | 0.05904087 | 0.253419931 | NS | 1.22884725 |
| ANKRD34B | 0.332367506 | 0.021152892 | 0.168202484 | Up | 1.674630251 |
| DHFR | -0.12997722 | 0.049444036 | 0.235321554 | Down | 1.305886084 |
| MSH3 | 0.194298803 | 0.001176656 | 0.055908237 | Up | 2.929350574 |
| RASGRF2 | 0.027074072 | 0.742564441 | 0.848994599 | NS | 0.129265852 |
| CKMT2 | 0.13328573 | 0.312906553 | 0.532524774 | NS | 0.504585341 |
| ZCCHC9 | -0.153630057 | 0.033956292 | 0.202672427 | Down | 1.469079746 |
| ACOT12 | 0.375323511 | 0.3182537 | NA | NS | 0.497226539 |
| SSBP2 | -0.049329968 | 0.314023788 | 0.533553882 | NS | 0.503037453 |
| ATG10 | 0.101368972 | 0.130051191 | 0.352095778 | NS | 0.885885667 |
| RPS23 | -0.271009109 | 0.089190072 | 0.299549938 | NS | 1.049683485 |
| TMEM167A | -0.030169546 | 0.701729211 | 0.823225202 | NS | 0.153830445 |
| XRCC4 | -0.124142824 | 0.236948271 | 0.464743416 | NS | 0.625346456 |
| VCAN | 0.131884334 | 0.203420642 | 0.433444748 | NS | 0.691604979 |
| HAPLN1 | 0.496525774 | 0.293411838 | NA | NS | 0.532522368 |
| EDIL3 | 0.408641632 | 0.254659416 | 0.48140404 | NS | 0.594040261 |
| COX7C | -0.353696668 | 0.065765081 | 0.265127437 | NS | 1.182004639 |
| RASA1 | 0.042405436 | 0.366871981 | 0.579215642 | NS | 0.435485456 |
| CCNH | 0.046573206 | 0.325534296 | 0.544029951 | NS | 0.487403251 |
| TMEM161B | 0.003308776 | 0.953189388 | 0.974797443 | NS | 0.020820801 |
| MEF2C | -0.109314554 | 0.146219601 | 0.371713762 | NS | 0.834994407 |
| CETN3 | -0.257178688 | 0.05415759 | 0.246045849 | NS | 1.26634067 |
| MBLAC2 | 0.018539505 | 0.805077839 | 0.887901589 | NS | 0.094162128 |
| POLR3G | -0.146287696 | 0.132442458 | 0.355399001 | NS | 0.877972768 |
| LYSMD3 | -0.263674472 | 0.007857031 | 0.11472857 | Down | 2.104741529 |
| ADGRV1 | 0.303242556 | 0.17184587 | 0.400530645 | NS | 0.7648609 |
| ARRDC3 | -0.184962812 | 0.062678421 | 0.260424746 | NS | 1.202881952 |
| NR2F1 | 0.70455656 | 0.106644977 | NA | NS | 0.972059595 |
| FAM172A | 0.039345744 | 0.392051256 | 0.598743523 | NS | 0.40665715 |
| POU5F2 | 0.273267038 | 0.024079546 | 0.175739247 | Up | 1.618351709 |
| KIAA0825 | 0.256515233 | 0.037019821 | 0.209468043 | Up | 1.431565689 |
| SLF1 | -0.119459017 | 0.200473526 | 0.430173773 | NS | 0.697942972 |
| MCTP1 | 0.098968807 | 0.174278061 | 0.403422181 | NS | 0.75875728 |
| FAM81B | 0.035295275 | 0.864318483 | 0.923382803 | NS | 0.0633262 |
| TTC37 | -0.13222605 | 0.040314738 | 0.216621104 | Down | 1.394536161 |
| ARSK | -0.104393033 | 0.14561654 | 0.370771051 | NS | 0.836789292 |
| GPR150 | 0.143136622 | 0.299539605 | 0.520845496 | NS | 0.523545747 |
| RFESD | -0.068672968 | 0.538008794 | 0.709108966 | NS | 0.269210626 |
| SPATA9 | 0.177322838 | 0.247758511 | 0.475860905 | NS | 0.605971417 |
| RHOBTB3 | -0.128488405 | 0.357594851 | 0.57070643 | NS | 0.446608743 |
| GLRX | 0.037334142 | 0.674729712 | 0.806117594 | NS | 0.170870165 |
| ELL2 | 0.061960005 | 0.460032422 | 0.651371229 | NS | 0.337211559 |
| PCSK1 | 0.293129099 | 0.157729089 | 0.385231054 | NS | 0.802088205 |
| CAST | 0.078076241 | 0.07767276 | 0.281911497 | NS | 1.109731264 |
| ERAP1 | 0.012827 | 0.769352981 | 0.865536277 | NS | 0.113874359 |
| ERAP2 | -0.346274385 | 0.004550959 | 0.094415114 | Down | 2.341897062 |
| LNPEP | -0.017224796 | 0.76607262 | 0.863854529 | NS | 0.115730059 |
| LIX1 | 0.183243057 | 0.241862879 | 0.469856956 | NS | 0.616430782 |
| RIOK2 | -0.103505827 | 0.172666815 | 0.401438949 | NS | 0.762791121 |
| RGMB | 0.257202278 | 0.022272277 | 0.170667658 | Up | 1.652235374 |
| CHD1 | -0.027256535 | 0.61863451 | 0.766747892 | NS | 0.208565857 |
| FAM174A | 0.068233604 | 0.455623116 | 0.647513534 | NS | 0.34139425 |
| ST8SIA4 | 0.020458844 | 0.833510831 | 0.905596331 | NS | 0.079088752 |
| SLCO4C1 | 0.060950061 | 0.564332667 | 0.728337377 | NS | 0.248464809 |
| SLCO6A1 | 0.566451778 | 0.24700194 | NA | NS | 0.607299636 |
| PAM | -0.159817753 | 0.152144566 | 0.378781917 | NS | 0.817743554 |
| GIN1 | -0.177786261 | 0.067014941 | 0.266824786 | NS | 1.173828362 |
| PPIP5K2 | -0.071516317 | 0.200313088 | 0.429960996 | NS | 0.698290673 |
| MACIR | -0.016143212 | 0.858047369 | 0.919794085 | NS | 0.066488736 |
| NUDT12 | -0.300548448 | 0.027596213 | 0.186639939 | Down | 1.559150513 |
| EFNA5 | 0.251394124 | 0.233781244 | 0.462194238 | NS | 0.631190335 |
| FBXL17 | 0.176696185 | 6.56E-04 | 0.04259149 | Up | 3.18285583 |
| FER | -0.06712328 | 0.16255871 | 0.390120462 | NS | 0.788989757 |
| PJA2 | -0.009273275 | 0.89734687 | 0.942664924 | NS | 0.047039648 |
| MAN2A1 | -0.131227349 | 0.009467005 | 0.122534699 | Down | 2.023787416 |
| TMEM232 | 0.152649457 | 0.41111356 | 0.613936017 | NS | 0.386038198 |
| SLC25A46 | -0.178503749 | 0.016764276 | 0.152541471 | Down | 1.775615202 |
| TSLP | 0.764924631 | 0.109991933 | NA | NS | 0.958639165 |
| WDR36 | -0.150857223 | 0.028364775 | 0.188347372 | Down | 1.547220652 |
| CAMK4 | 0.023717206 | 0.793897381 | 0.880945677 | NS | 0.100235631 |
| STARD4 | -0.032854733 | 0.642778785 | 0.783861541 | NS | 0.191938465 |
| NREP | 0.176354144 | 0.149287875 | 0.375271684 | NS | 0.825975463 |
| EPB41L4A | -0.11830298 | 0.412171251 | 0.614423458 | NS | 0.384922304 |
| APC | -0.026491435 | 0.656778945 | 0.793958901 | NS | 0.182580779 |
| SRP19 | -0.006383829 | 0.895392967 | 0.941303178 | NS | 0.047986321 |
| REEP5 | -0.111128507 | 0.169300175 | 0.397632305 | NS | 0.771342593 |
| DCP2 | -0.03358898 | 0.612549771 | 0.762995428 | NS | 0.212858618 |
| MCC | 0.13407969 | 0.121493499 | 0.342159876 | NS | 0.915446961 |
| TSSK1B | 0.384938389 | 0.68366135 | NA | NS | 0.165158972 |
| YTHDC2 | -0.016589263 | 0.756056474 | 0.857358159 | NS | 0.121445763 |
| KCNN2 | 0.68087621 | 0.134538795 | 0.358064534 | NS | 0.871152465 |
| TRIM36 | 0.128633334 | 0.298864969 | 0.520339481 | NS | 0.524524987 |
| PGGT1B | -0.006723865 | 0.904637451 | 0.947195487 | NS | 0.043525437 |
| CCDC112 | -0.124517877 | 0.127217505 | 0.348830209 | NS | 0.895453125 |
| FEM1C | 0.050680613 | 0.421544121 | 0.621325323 | NS | 0.375156963 |
| TICAM2 | 0.320845691 | 0.035761003 | NA | Up | 1.446590303 |
| TMED7-TICAM2 | 0.06720912 | 0.638832652 | 0.780587174 | NS | 0.194612894 |
| TMED7 | -0.150327188 | 0.098007486 | 0.312744522 | NS | 1.008740753 |
| CDO1 | 0.243834839 | 0.4922855 | 0.676245815 | NS | 0.307782956 |
| ATG12 | -0.078133225 | 0.127646251 | 0.349140624 | NS | 0.893991935 |
| AP3S1 | -0.142156184 | 0.084154825 | 0.292017244 | NS | 1.074920977 |
| LVRN | 0.405466235 | 0.188595617 | 0.417880884 | NS | 0.724468405 |
| ARL14EPL | -0.0092953 | 0.98466934 | NA | NS | 0.006709585 |
| COMMD10 | -0.10677881 | 0.141553956 | 0.365923729 | NS | 0.84907799 |
| SEMA6A | 0.157955299 | 0.154337026 | 0.381338502 | NS | 0.811529872 |
| DTWD2 | -0.034723462 | 0.606831756 | 0.758845143 | NS | 0.2169317 |
| DMXL1 | 0.016176293 | 0.720008371 | 0.834756433 | NS | 0.142662454 |
| TNFAIP8 | -0.177871261 | 0.039129913 | 0.214493363 | Down | 1.407491116 |
| HSD17B4 | -0.121451632 | 0.00961947 | 0.123151482 | Down | 2.01684886 |
| FAM170A | 0.477539969 | 0.402891942 | NA | NS | 0.394811418 |
| PRR16 | 0.553319909 | 0.128031462 | NA | NS | 0.892683295 |
| FTMT | 1.464714699 | 0.02250817 | NA | Up | 1.647659813 |
| SRFBP1 | -0.320358173 | 0.003953327 | 0.090040171 | Down | 2.403037219 |
| LOX | 0.154719704 | 0.18407654 | 0.414354362 | NS | 0.735001557 |
| ZNF474 | -0.32508763 | 0.287460357 | NA | NS | 0.541422039 |
| SNCAIP | 0.350125475 | 0.415631237 | 0.61690127 | NS | 0.38129182 |
| SNX2 | -0.142036058 | 0.079603352 | 0.284900136 | NS | 1.099068643 |
| SNX24 | -0.126536751 | 0.091571311 | 0.303343316 | NS | 1.038240566 |
| PPIC | 0.225389408 | 0.43597347 | NA | NS | 0.360539937 |
| PRDM6 | 0.609509278 | 0.180566368 | 0.410332502 | NS | 0.743363138 |
| CEP120 | -0.064174788 | 0.116831583 | 0.336690188 | NS | 0.932439737 |
| CSNK1G3 | -0.32229478 | 0.013224887 | 0.13792184 | Down | 1.878608033 |
| ZNF608 | 0.292084913 | 0.0635965 | 0.262056846 | NS | 1.196566786 |
| GRAMD2B | -0.105273204 | 0.239533487 | 0.46763242 | NS | 0.620633763 |
| ALDH7A1 | 0.330821594 | 0.235848464 | 0.463679416 | NS | 0.627366948 |
| PHAX | -0.192642916 | 0.010236251 | 0.126233693 | Down | 1.989859057 |
| TEX43 | -0.001522977 | 0.996611839 | NA | NS | 0.001473958 |
| LMNB1 | 0.173510141 | 0.138554743 | 0.363022621 | NS | 0.858378603 |
| MARCHF3 | 0.045864479 | 0.647260964 | 0.787042932 | NS | 0.188920585 |
| C5orf63 | 0.174242836 | 0.056011241 | 0.249087638 | NS | 1.251724801 |
| MEGF10 | 0.437976989 | 0.265847779 | 0.491832837 | NS | 0.575366963 |
| PRRC1 | -0.027582844 | 0.570598503 | 0.732633258 | NS | 0.243669372 |
| CTXN3 | 0.019174409 | 0.972024508 | NA | NS | 0.012322785 |
| CCDC192 | 0.417246862 | 0.08643585 | NA | NS | 1.063306095 |
| SLC12A2 | 0.019170522 | 0.712175985 | 0.829996153 | NS | 0.147412675 |
| FBN2 | -0.082897708 | 0.611438636 | 0.76220302 | NS | 0.213647122 |
| SLC27A6 | 0.177216465 | 0.694637122 | NA | NS | 0.158242012 |
| ISOC1 | -0.023738369 | 0.72932806 | 0.840152469 | NS | 0.137077077 |
| ADAMTS19 | 0.628597614 | 0.169436809 | NA | NS | 0.770992235 |
| MINAR2 | 0.808985817 | 0.12158472 | NA | NS | 0.915121003 |
| CHSY3 | 0.274979119 | 0.319589063 | NA | NS | 0.495408092 |
| HINT1 | -0.294743316 | 0.055072381 | 0.247775608 | NS | 1.259066146 |
| LYRM7 | -0.15489648 | 0.023460759 | 0.173939239 | Down | 1.629657937 |
| CDC42SE2 | -0.073397916 | 0.321064828 | 0.539671684 | NS | 0.493407268 |
| RAPGEF6 | 0.066535081 | 0.233198024 | 0.461479405 | NS | 0.632275133 |
| FNIP1 | 0.063200521 | 0.43017183 | 0.627557816 | NS | 0.366358033 |
| MEIKIN | 0.309822521 | 0.397530663 | NA | NS | 0.400629367 |
| ACSL6 | -0.143915676 | 0.142044376 | 0.36646393 | NS | 0.847575956 |
| IL3 | 0.141077597 | 0.845100856 | NA | NS | 0.073091458 |
| CSF2 | 0.126643096 | 0.760988016 | NA | NS | 0.118622182 |
| P4HA2 | 0.225506391 | 0.164148962 | 0.391978395 | NS | 0.784761859 |
| PDLIM4 | 0.459737017 | 0.068370656 | 0.268488472 | NS | 1.165130251 |
| SLC22A4 | 0.242128039 | 0.061820727 | 0.258321151 | NS | 1.208865893 |
| SLC22A5 | 0.140235282 | 0.096902255 | 0.311037904 | NS | 1.013666117 |
| IRF1 | 0.086105489 | 0.237848111 | 0.465890989 | NS | 0.623700293 |
| IL5 | 0.27607278 | 0.245217621 | 0.47328282 | NS | 0.610448326 |
| RAD50 | 0.167759098 | 0.030817536 | 0.19496774 | Up | 1.511202082 |
| IL13 | 0.500772145 | 0.271615543 | NA | NS | 0.566045381 |
| IL4 | 0.228198001 | 0.380760218 | NA | NS | 0.419348434 |
| KIF3A | -0.056117167 | 0.294208988 | 0.516303721 | NS | 0.531344064 |
| CCNI2 | 0.048106503 | 0.669674555 | 0.802936321 | NS | 0.174136202 |
| SEPTIN8 | 0.063897705 | 0.313840131 | 0.533400125 | NS | 0.503291523 |
| SOWAHA | 1.33489813 | 0.00338793 | NA | Up | 2.470065616 |
| SHROOM1 | 0.305462878 | 0.094259923 | 0.307415197 | NS | 1.025672919 |
| GDF9 | 0.231753926 | 0.136523101 | 0.361003586 | NS | 0.864793856 |
| UQCRQ | -0.172530414 | 0.206778892 | 0.436666684 | NS | 0.684493796 |
| LEAP2 | 0.135864872 | 0.117298429 | 0.337268176 | NS | 0.930707803 |
| AFF4 | 0.121346857 | 0.010615878 | 0.127927347 | Up | 1.974044064 |
| ZCCHC10 | -0.283644325 | 0.033506867 | 0.201569287 | Down | 1.474866173 |
| HSPA4 | -0.169032225 | 0.007439725 | 0.112720615 | Down | 2.128443127 |
| FSTL4 | 0.239997738 | 0.343130277 | 0.55833676 | NS | 0.464540959 |
| C5orf15 | -0.129020114 | 0.086080069 | 0.29485869 | NS | 1.065097393 |
| VDAC1 | -0.212987518 | 0.010754385 | 0.12826482 | Down | 1.968414435 |
| TCF7 | 0.108257264 | 0.366257586 | 0.57858521 | NS | 0.436213371 |
| SKP1 | -0.119035362 | 0.1980678 | 0.427972883 | NS | 0.703186123 |
| PPP2CA | -0.136481811 | 0.041433891 | 0.219327214 | Down | 1.382644276 |
| CDKL3 | -0.039995358 | 0.806112917 | 0.888482827 | NS | 0.09360412 |
| UBE2B | -0.023094106 | 0.862928023 | 0.922704331 | NS | 0.064025427 |
| CDKN2AIPNL | -0.135393078 | 0.121688445 | 0.34234916 | NS | 0.914750658 |
| JADE2 | 0.031572733 | 0.604726573 | 0.757628445 | NS | 0.218440947 |
| SAR1B | -0.218983609 | 0.003288755 | 0.084985048 | Down | 2.482968524 |
| SEC24A | 0.067590863 | 0.176717875 | 0.406385458 | NS | 0.752719519 |
| CAMLG | -0.218045195 | 0.019807791 | 0.163424633 | Down | 1.703163959 |
| DDX46 | -0.113826291 | 0.006172062 | 0.105138876 | Down | 2.209569689 |
| C5orf24 | 0.047300084 | 0.397201391 | 0.602351096 | NS | 0.40098924 |
| TXNDC15 | -0.18551996 | 0.001984707 | 0.06982144 | Down | 2.702303621 |
| PCBD2 | -0.006743585 | 0.919177873 | 0.955269172 | NS | 0.036600439 |
| CATSPER3 | 0.163713928 | 0.458912687 | NA | NS | 0.338269936 |
| PITX1 | 0.81029593 | 0.078958851 | NA | NS | 1.102599178 |
| MACROH2A1 | -0.013769358 | 0.833389407 | 0.905507363 | NS | 0.079152024 |
| TIFAB | -0.09165834 | 0.602595377 | 0.756116553 | NS | 0.219974205 |
| NEUROG1 | 0.647810508 | 0.22656198 | NA | NS | 0.644812969 |
| CXCL14 | 0.745402951 | 0.137521568 | NA | NS | 0.861629183 |
| SLC25A48 | 0.849801113 | 0.035546074 | 0.206049964 | Up | 1.449208363 |
| IL9 | 0.360627319 | 0.531000099 | NA | NS | 0.274905398 |
| LECT2 | 0.459116845 | 0.41036071 | NA | NS | 0.386834228 |
| TGFBI | -0.070773464 | 0.481555242 | 0.668358169 | NS | 0.317353885 |
| SMAD5 | -0.01472101 | 0.81719951 | 0.896035964 | NS | 0.087671902 |
| SMIM32 | 0.687104469 | 0.004875891 | NA | Up | 2.311945999 |
| TRPC7 | 0.661336233 | 0.153788308 | NA | NS | 0.813076682 |
| SPOCK1 | 0.226115748 | 0.281229344 | 0.504325448 | NS | 0.550939367 |
| KLHL3 | 0.102210288 | 0.279694767 | 0.503424202 | NS | 0.55331566 |
| HNRNPA0 | -0.151719818 | 0.051567121 | 0.239746285 | NS | 1.287627117 |
| MYOT | 0.139498348 | 0.29334246 | 0.515391563 | NS | 0.53262507 |
| PKD2L2 | 0.178991675 | 0.054503338 | 0.24676996 | NS | 1.263576899 |
| FAM13B | -0.078717648 | 0.284080237 | 0.506905224 | NS | 0.546558978 |
| WNT8A | 0.62629936 | 0.264444543 | NA | NS | 0.577665391 |
| NME5 | 0.825033799 | 0.103204542 | NA | NS | 0.98630119 |
| BRD8 | 0.080249785 | 0.17522918 | 0.404559472 | NS | 0.756393571 |
| KIF20A | 0.067688764 | 0.510491804 | 0.689496613 | NS | 0.292011226 |
| CDC23 | -0.187899433 | 0.001871203 | 0.067487067 | Down | 2.727879131 |
| GFRA3 | 0.210309546 | 0.672508679 | NA | NS | 0.172302107 |
| CDC25C | -0.01350036 | 0.955767438 | 0.976012104 | NS | 0.019647769 |
| FAM53C | 0.244427031 | 0.028965847 | 0.189964397 | Up | 1.538113768 |
| KDM3B | 0.092778452 | 0.111702765 | 0.330183522 | NS | 0.951936078 |
| REEP2 | 0.513524778 | 0.059559059 | NA | NS | 1.225052175 |
| EGR1 | 0.14977351 | 0.636954071 | 0.779022011 | NS | 0.195891882 |
| ETF1 | -0.232542467 | 1.06E-04 | 0.016409885 | Down | 3.974540267 |
| HSPA9 | -0.212859748 | 0.002182843 | 0.072832159 | Down | 2.660977567 |
| CTNNA1 | -0.166266739 | 0.076721636 | 0.280773235 | NS | 1.115082146 |
| LRRTM2 | 0.180774278 | 0.222190349 | 0.450931554 | NS | 0.653274809 |
| SIL1 | -0.131672144 | 0.037650493 | 0.210772814 | Down | 1.424229338 |
| MATR3 | -0.217277845 | 0.007111092 | 0.110938851 | Down | 2.14806369 |
| PAIP2 | -0.2636521 | 0.009293443 | 0.121621961 | Down | 2.03182336 |
| SLC23A1 | 0.218398413 | 0.010120786 | 0.126203819 | Up | 1.994785777 |
| MZB1 | 0.216814531 | 0.242600883 | 0.470389904 | NS | 0.615107623 |
| PROB1 | 0.380098534 | 0.033480455 | 0.201567051 | Up | 1.475208644 |
| SPATA24 | 0.087894206 | 0.467769776 | 0.657432551 | NS | 0.329967843 |
| DNAJC18 | 5.89E-04 | 0.996573917 | 0.998035641 | NS | 0.001490484 |
| STING1 | 0.079795876 | 0.29986875 | 0.521338656 | NS | 0.523068791 |
| UBE2D2 | -0.212950674 | 0.021145305 | 0.168202484 | Down | 1.674786046 |
| CXXC5 | -0.314509041 | 0.004503523 | 0.094105548 | Down | 2.346447628 |
| PSD2 | 0.640772991 | 0.088391246 | 0.298422198 | NS | 1.053590743 |
| NRG2 | 0.717640991 | 0.065987513 | NA | NS | 1.180538239 |
| PURA | 0.04872294 | 0.431237963 | 0.62817783 | NS | 0.365283014 |
| IGIP | 0.047687351 | 0.512185035 | 0.690734994 | NS | 0.290573115 |
| CYSTM1 | 0.282955843 | 0.040955572 | 0.218105862 | Up | 1.387687001 |
| PFDN1 | 0.113624994 | 0.089369736 | 0.299843258 | NS | 1.048809523 |
| HBEGF | 0.02277787 | 0.90623714 | 0.94801213 | NS | 0.042758143 |
| SLC4A9 | 0.8911832 | 0.003542094 | 0.08638519 | Up | 2.450739871 |
| ANKHD1 | 0.121089187 | 0.09683523 | 0.31095351 | NS | 1.013966613 |
| ANKHD1-EIF4EBP3 | 0.252580634 | 0.008768335 | 0.118894262 | Up | 2.057082866 |
| SRA1 | 0.057068912 | 0.248679632 | 0.476232204 | NS | 0.604359784 |
| EIF4EBP3 | -0.096269566 | 0.621585355 | NA | NS | 0.206499227 |
| APBB3 | 0.023767222 | 0.767380265 | 0.864591077 | NS | 0.114989374 |
| SLC35A4 | 0.220711863 | 0.014369127 | 0.143935548 | Up | 1.842569624 |
| CD14 | -0.115869343 | 0.373397882 | 0.583923407 | NS | 0.42782815 |
| NDUFA2 | -0.083622817 | 0.374944987 | 0.585708926 | NS | 0.426032448 |
| TMCO6 | 0.021310886 | 0.670722154 | 0.803625814 | NS | 0.173457349 |
| IK | -0.135712672 | 0.006364035 | 0.107168482 | Down | 2.196267423 |
| WDR55 | -0.012952379 | 0.673393344 | 0.805497459 | NS | 0.171731181 |
| HARS1 | -0.137839593 | 6.94E-04 | 0.043642779 | Down | 3.15885963 |
| HARS2 | 0.008469412 | 0.827424766 | 0.901734909 | NS | 0.082271484 |
| ZMAT2 | -0.383012788 | 0.001352093 | 0.059779209 | Down | 2.868993485 |
| PCDHA2 | 1.189735631 | 0.018302748 | NA | Up | 1.737483693 |
| PCDHA3 | 0.69696685 | 0.156293374 | NA | NS | 0.806059432 |
| PCDHA4 | 0.769216222 | 0.081396195 | 0.287719656 | NS | 1.089395898 |
| PCDHA5 | 0.524748899 | 0.233122573 | NA | NS | 0.632415673 |
| PCDHA6 | 1.482755414 | 0.002421824 | NA | Up | 2.615857496 |
| PCDHA7 | 1.178877576 | 0.046264432 | NA | Up | 1.334752767 |
| PCDHA8 | 0.929968917 | 0.122200371 | NA | NS | 0.912927475 |
| PCDHA9 | 0.416454821 | 0.381772246 | NA | NS | 0.418195647 |
| PCDHA10 | 0.782178703 | 0.083172491 | 0.290414939 | NS | 1.080020293 |
| PCDHA11 | 1.001321586 | 0.061255414 | NA | NS | 1.212855521 |
| PCDHA12 | 1.290089258 | 0.022129779 | NA | Up | 1.655022922 |
| PCDHA13 | 1.008800674 | 0.084981667 | NA | NS | 1.070674754 |
| PCDHAC1 | 0.924298377 | 0.057589482 | NA | NS | 1.23965683 |
| PCDHAC2 | 1.138989864 | 0.035399908 | NA | Up | 1.450997862 |
| PCDHB1 | 0.776433869 | 0.133126609 | NA | NS | 0.875735129 |
| PCDHB2 | 0.817828214 | 0.102125282 | NA | NS | 0.990866731 |
| PCDHB3 | 0.965160538 | 0.048801474 | NA | Up | 1.311567064 |
| PCDHB4 | 0.68356967 | 0.118644244 | NA | NS | 0.925753325 |
| PCDHB5 | 0.913313421 | 0.014733531 | NA | Up | 1.83169315 |
| PCDHB6 | 0.783704331 | 0.106153796 | NA | NS | 0.974064471 |
| PCDHB7 | 0.378643917 | 0.469244207 | NA | NS | 0.32860108 |
| PCDHB8 | 0.216306801 | 0.679570058 | NA | NS | 0.167765765 |
| PCDHB16 | 0.643437963 | 0.22927837 | NA | NS | 0.639636914 |
| PCDHB9 | 0.616935161 | 0.194018385 | NA | NS | 0.712157115 |
| PCDHB10 | 0.467346641 | 0.316130974 | NA | NS | 0.50013295 |
| PCDHB11 | 0.703018055 | 0.185068635 | NA | NS | 0.732667178 |
| PCDHB12 | 0.719741068 | 0.151407751 | NA | NS | 0.81985189 |
| PCDHB13 | 0.54091023 | 0.195853023 | NA | NS | 0.708069722 |
| PCDHB14 | 0.791567601 | 0.060063385 | NA | NS | 1.221390197 |
| PCDHB15 | 0.758650929 | 0.005284577 | NA | Up | 2.276989773 |
| TAF7 | -0.023850099 | 0.799965117 | 0.884720935 | NS | 0.09692895 |
| SLC25A2 | 0.354787702 | 0.053135584 | 0.243367273 | NS | 1.274614544 |
| PCDHGA1 | 0.012387001 | 0.953873595 | 0.975032942 | NS | 0.020509173 |
| PCDHGA2 | 0.491153338 | 0.152971448 | NA | NS | 0.815389622 |
| PCDHGA3 | 0.470916371 | 0.149690718 | NA | NS | 0.82480513 |
| PCDHGB1 | -0.087815086 | 0.766349826 | NA | NS | 0.115572937 |
| PCDHGA4 | 0.186339356 | 0.455658857 | 0.647524146 | NS | 0.341360184 |
| PCDHGB2 | 0.154167362 | 0.29908267 | 0.520433287 | NS | 0.52420875 |
| PCDHGA5 | 0.010680426 | 0.947308149 | 0.971541457 | NS | 0.023508727 |
| PCDHGB3 | -0.130860745 | 0.361455218 | 0.574067087 | NS | 0.441945502 |
| PCDHGA6 | 0.184286215 | 0.249599815 | 0.47668561 | NS | 0.602755742 |
| PCDHGA7 | 0.185747913 | 0.394187541 | 0.600085288 | NS | 0.404297106 |
| PCDHGB4 | 0.231121383 | 0.230508707 | 0.458820172 | NS | 0.637312665 |
| PCDHGA8 | 0.129478101 | 0.508888245 | 0.688234238 | NS | 0.293377581 |
| PCDHGB5 | 0.038200851 | 0.814190008 | 0.893829492 | NS | 0.089274232 |
| PCDHGA9 | 0.057596526 | 0.740537502 | 0.847850763 | NS | 0.130452943 |
| PCDHGB6 | 0.075650791 | 0.627005142 | 0.772690043 | NS | 0.202728897 |
| PCDHGA10 | 0.069036111 | 0.61949278 | 0.767330709 | NS | 0.207963751 |
| PCDHGB7 | 0.042870986 | 0.783011657 | 0.874459109 | NS | 0.106231773 |
| PCDHGA11 | 0.041652369 | 0.804014365 | 0.887442141 | NS | 0.094736192 |
| PCDHGA12 | 0.098540272 | 0.633796213 | 0.776736637 | NS | 0.19805036 |
| PCDHGC3 | 0.045878365 | 0.800811267 | 0.885400195 | NS | 0.096469825 |
| PCDHGC4 | -0.012997154 | 0.948969338 | NA | NS | 0.02274782 |
| PCDHGC5 | 0.475995132 | 0.071971063 | NA | NS | 1.142842082 |
| DIAPH1 | 0.082947525 | 0.446485203 | 0.640438462 | NS | 0.350192929 |
| HDAC3 | 0.009831846 | 0.796627137 | 0.882905279 | NS | 0.098744903 |
| RELL2 | 0.131976603 | 0.085314511 | 0.293565942 | NS | 1.068977093 |
| FCHSD1 | 0.013208283 | 0.863711937 | 0.92301679 | NS | 0.063631078 |
| ARAP3 | 0.048807195 | 0.768613627 | 0.865129468 | NS | 0.11429192 |
| PCDH1 | -0.052680587 | 0.773141603 | 0.86784941 | NS | 0.111740956 |
| DELE1 | 0.047325019 | 0.4082991 | 0.61140312 | NS | 0.389021578 |
| PCDH12 | 0.027156871 | 0.734922908 | 0.843669396 | NS | 0.133758215 |
| RNF14 | -0.252361631 | 0.068549863 | 0.268602708 | NS | 1.163993411 |
| GNPDA1 | -0.250834979 | 0.002836813 | 0.079855432 | Down | 2.54716929 |
| NDFIP1 | 0.031086708 | 0.556506617 | 0.722432319 | NS | 0.254529668 |
| SPRY4 | 0.826898143 | 0.051591653 | 0.239776024 | NS | 1.287420558 |
| FGF1 | 0.473074514 | 0.344684266 | 0.559617117 | NS | 0.462578541 |
| ARHGAP26 | 0.098006057 | 0.35300731 | 0.566622752 | NS | 0.452216301 |
| NR3C1 | -0.034656894 | 0.549457215 | 0.717263403 | NS | 0.260066119 |
| HMHB1 | 0.963380732 | 0.082316226 | NA | NS | 1.084514549 |
| YIPF5 | -0.163025356 | 0.088303599 | 0.298364885 | NS | 1.054021593 |
| KCTD16 | 0.581330346 | 0.142709861 | 0.367353179 | NS | 0.845546017 |
| PRELID2 | -0.433368591 | 0.01057797 | 0.127927347 | Down | 1.975597671 |
| GRXCR2 | -0.051850705 | 0.928458516 | NA | NS | 0.032237496 |
| SH3RF2 | 0.387855541 | 0.134631567 | 0.358152184 | NS | 0.870853099 |
| PLAC8L1 | 0.090520507 | 0.598167998 | 0.752953912 | NS | 0.223176826 |
| LARS1 | -0.11380193 | 0.028086506 | 0.187669577 | Down | 1.55150228 |
| RBM27 | -0.126467326 | 0.070586441 | 0.271500817 | NS | 1.151278713 |
| POU4F3 | 0.710627705 | 0.08539525 | NA | NS | 1.068566287 |
| TCERG1 | -0.082440136 | 0.100686463 | 0.316153692 | NS | 0.997028916 |
| GPR151 | 0.084039663 | 0.497701272 | 0.679732613 | NS | 0.30303125 |
| PPP2R2B | 0.069121384 | 0.464176473 | 0.654833657 | NS | 0.333316876 |
| STK32A | 0.241233207 | 0.590066096 | 0.747557481 | NS | 0.229099338 |
| DPYSL3 | 0.620600955 | 0.103756012 | 0.319642211 | NS | 0.983986728 |
| JAKMIP2 | -0.173736085 | 0.101997508 | 0.317905513 | NS | 0.991410441 |
| SPINK1 | 0.440788011 | 0.454175944 | NA | NS | 0.342775872 |
| SCGB3A2 | 0.425204651 | 0.235384899 | 0.46327275 | NS | 0.628221402 |
| C5orf46 | 0.576281144 | 0.192777645 | NA | NS | 0.714943329 |
| SPINK5 | 0.715712782 | 0.105931358 | 0.322198901 | NS | 0.974975462 |
| SPINK14 | 0.336132338 | 0.555659822 | NA | NS | 0.255191004 |
| SPINK6 | 0.117286706 | 0.868801788 | NA | NS | 0.061079294 |
| MARCOL | 0.21569592 | 0.722616382 | NA | NS | 0.141092197 |
| SPINK13 | 0.382963081 | 0.30801451 | NA | NS | 0.511428824 |
| SPINK7 | 0.109078817 | 0.514851421 | 0.692902817 | NS | 0.288318084 |
| SPINK9 | 0.237726257 | 0.169085741 | 0.397291642 | NS | 0.771893015 |
| FBXO38 | 0.024854387 | 0.705204271 | 0.825441491 | NS | 0.151685066 |
| HTR4 | 0.472471103 | 0.154788727 | 0.382001231 | NS | 0.810260672 |
| ADRB2 | -0.087954811 | 0.268946786 | 0.49444608 | NS | 0.570333641 |
| SH3TC2 | 0.20800297 | 0.218128632 | 0.447660331 | NS | 0.661287323 |
| ABLIM3 | 0.370126537 | 0.066256685 | 0.2655108 | NS | 1.178770294 |
| AFAP1L1 | 0.362155553 | 0.178390148 | 0.408344944 | NS | 0.748629135 |
| GRPEL2 | -0.046500441 | 0.517459737 | 0.6948615 | NS | 0.286123437 |
| PCYOX1L | 0.064122549 | 0.346607705 | 0.56126774 | NS | 0.460161788 |
| IL17B | 0.227443667 | 0.418592581 | NA | NS | 0.378208473 |
| CSNK1A1 | 0.153230674 | 2.67E-04 | 0.02707137 | Up | 3.573254731 |
| ARHGEF37 | -0.025621401 | 0.900674497 | 0.944854146 | NS | 0.045432134 |
| PPARGC1B | 0.123782536 | 0.245686003 | 0.473947173 | NS | 0.609619585 |
| PDE6A | 0.256528096 | 0.301996211 | 0.523527154 | NS | 0.519998505 |
| SLC26A2 | 0.046531486 | 0.346027414 | 0.560904975 | NS | 0.460889493 |
| TIGD6 | -0.088971918 | 0.280073226 | 0.50348007 | NS | 0.552728406 |
| HMGXB3 | -0.018136246 | 0.69593187 | 0.819766525 | NS | 0.157433275 |
| CSF1R | 0.017144714 | 0.886706492 | 0.935955238 | NS | 0.052220112 |
| PDGFRB | 0.299122647 | 0.057850568 | 0.251880934 | NS | 1.237692372 |
| CDX1 | 1.366246675 | 0.020828829 | NA | Up | 1.681335147 |
| SLC6A7 | 0.750690847 | 0.085177525 | 0.293490206 | NS | 1.069674983 |
| CAMK2A | 0.823666451 | 0.077505848 | 0.281707495 | NS | 1.110665529 |
| ARSI | 0.614667055 | 0.221622355 | NA | NS | 0.654386435 |
| TCOF1 | -0.097783209 | 0.306094353 | 0.527199584 | NS | 0.514144682 |
| CD74 | 0.377502635 | 0.003355999 | 0.085084692 | Up | 2.474178225 |
| RPS14 | -0.416962271 | 0.005453716 | 0.100547004 | Down | 2.263307485 |
| NDST1 | 0.017950695 | 0.88294321 | 0.933787366 | NS | 0.054067229 |
| SYNPO | 0.379052899 | 0.145372101 | 0.37059965 | NS | 0.837518931 |
| MYOZ3 | 0.545846826 | 0.057026488 | 0.250513504 | NS | 1.243923373 |
| RBM22 | 0.155583644 | 4.44E-05 | 0.009235457 | Up | 4.35302234 |
| DCTN4 | -0.02148933 | 0.708366534 | 0.827240343 | NS | 0.149741965 |
| SMIM3 | -0.029605528 | 0.786452971 | 0.876763177 | NS | 0.104327243 |
| IRGM | -0.250608427 | 0.058143652 | 0.252197542 | NS | 1.235497691 |
| ZNF300 | -0.236655883 | 0.031459433 | 0.196956206 | Down | 1.502249106 |
| GPX3 | 0.165026042 | 0.080631144 | 0.286282138 | NS | 1.09349718 |
| TNIP1 | 0.061192975 | 0.611227852 | 0.762141649 | NS | 0.213796864 |
| ANXA6 | -0.275906702 | 0.00148292 | 0.062253356 | Down | 2.828882164 |
| CCDC69 | 0.08268499 | 0.064896399 | 0.264064926 | NS | 1.187779399 |
| GM2A | -0.097520477 | 0.295722877 | 0.517512114 | NS | 0.529115078 |
| SLC36A3 | 0.35265765 | 0.257280187 | 0.484200085 | NS | 0.589593658 |
| SLC36A2 | 0.60253837 | 0.18892618 | NA | NS | 0.723707856 |
| SLC36A1 | 0.257294006 | 0.033211407 | 0.200900063 | Up | 1.478712728 |
| FAT2 | 0.310178474 | 0.079031157 | 0.283921514 | NS | 1.102201663 |
| SPARC | 0.265219084 | 0.089942644 | 0.300359644 | NS | 1.046034349 |
| ATOX1 | 0.085450528 | 0.074573623 | 0.277209074 | NS | 1.127414759 |
| G3BP1 | -0.010851431 | 0.799871086 | 0.884702387 | NS | 0.096980002 |
| GLRA1 | 0.329853125 | 0.139498938 | 0.36380609 | NS | 0.8554291 |
| NMUR2 | 0.534732404 | 0.323885946 | NA | NS | 0.489607897 |
| GRIA1 | 0.380153169 | 0.412954499 | 0.614960916 | NS | 0.384097798 |
| FAM114A2 | -0.158637626 | 0.022396846 | 0.171057734 | Down | 1.649813139 |
| MFAP3 | -0.024419119 | 0.600705538 | 0.754699919 | NS | 0.221338364 |
| GALNT10 | -0.050061983 | 0.468660857 | 0.658038805 | NS | 0.329141318 |
| SAP30L | 0.021771787 | 0.643960746 | 0.784676191 | NS | 0.191140605 |
| HAND1 | 0.844952907 | 0.126119053 | NA | NS | 0.8992193 |
| LARP1 | -0.032004461 | 0.78327343 | 0.874695991 | NS | 0.106086605 |
| FAXDC2 | -0.391443112 | 0.042117758 | 0.220751111 | Down | 1.375534751 |
| CNOT8 | -0.103939153 | 0.097884721 | 0.312701336 | NS | 1.009285093 |
| GEMIN5 | -0.167411265 | 0.013967137 | 0.141412627 | Down | 1.854892619 |
| MRPL22 | -0.053124299 | 0.539609525 | 0.710206067 | NS | 0.267920393 |
| KIF4B | 0.524989735 | 0.2164564 | NA | NS | 0.664629568 |
| SGCD | 0.554338055 | 0.01593326 | 0.149715225 | Up | 1.797695369 |
| PPP1R2B | 0.236002838 | 0.312780601 | NA | NS | 0.504760191 |
| TIMD4 | 0.18781159 | 0.267687606 | 0.493645346 | NS | 0.572371736 |
| HAVCR1 | 0.079593105 | 0.538065244 | 0.709142557 | NS | 0.26916506 |
| HAVCR2 | -0.146292967 | 0.165843215 | 0.393793812 | NS | 0.780302291 |
| MED7 | -0.088829686 | 0.28382022 | 0.506589562 | NS | 0.546956668 |
| ITK | -0.027790169 | 0.667848599 | 0.801418319 | NS | 0.175321981 |
| FAM71B | 0.604694327 | 0.280784145 | NA | NS | 0.55162742 |
| CYFIP2 | 0.039740612 | 0.493635066 | 0.67740743 | NS | 0.306593997 |
| FNDC9 | 0.520678594 | 0.028221936 | NA | Up | 1.549413201 |
| ADAM19 | 0.246442002 | 0.003920904 | 0.089796534 | Up | 2.406613836 |
| NIPAL4 | 0.217039519 | 0.098481509 | 0.313238455 | NS | 1.006645307 |
| SOX30 | 0.386731506 | 0.069050114 | 0.269217862 | NS | 1.160835597 |
| C5orf52 | 1.393300456 | 0.034295294 | NA | Up | 1.464765466 |
| THG1L | -0.295819893 | 0.004382583 | 0.093367359 | Down | 2.358269873 |
| LSM11 | -0.048738856 | 0.371468415 | 0.582497065 | NS | 0.430078107 |
| CLINT1 | -0.122351176 | 0.020775267 | 0.167356721 | Down | 1.682453394 |
| EBF1 | 0.113710525 | 0.340477123 | 0.556058409 | NS | 0.467912064 |
| RNF145 | -0.075469929 | 0.246240809 | 0.474504671 | NS | 0.60863997 |
| UBLCP1 | 0.114077399 | 0.106147069 | 0.322426185 | NS | 0.974091994 |
| IL12B | 0.502576435 | 0.120304299 | NA | NS | 0.919718853 |
| ADRA1B | 0.712199179 | 0.117279423 | NA | NS | 0.930778179 |
| TTC1 | -0.122806399 | 0.022243679 | 0.170661554 | Down | 1.652793378 |
| PWWP2A | -0.091340459 | 0.005917284 | 0.10308115 | NS | 2.227877574 |
| FABP6 | 0.011384468 | 0.96609057 | NA | NS | 0.014982157 |
| CCNJL | 0.451088857 | 0.003810294 | 0.088772476 | Up | 2.419041562 |
| C1QTNF2 | 0.98578608 | 0.065096284 | NA | NS | 1.186443803 |
| ZBED8 | -0.008613657 | 0.933039432 | 0.963078576 | NS | 0.030100002 |
| SLU7 | -0.142574428 | 0.040963819 | 0.218105862 | Down | 1.38759956 |
| PTTG1 | -0.226499381 | 0.029025993 | 0.190037745 | Down | 1.537212919 |
| ATP10B | 0.387688731 | 0.162054417 | 0.389682175 | NS | 0.790339127 |
| GABRB2 | 0.472909718 | 0.187874273 | 0.417308021 | NS | 0.726132686 |
| GABRA6 | 0.395498495 | 0.41680173 | NA | NS | 0.380070487 |
| GABRA1 | 0.42934487 | 0.354134004 | 0.567317918 | NS | 0.45083237 |
| GABRG2 | 0.358931021 | 0.438493115 | 0.633805873 | NS | 0.358037222 |
| CCNG1 | -0.24528853 | 0.006233524 | 0.105602826 | Down | 2.205266346 |
| NUDCD2 | -0.036947125 | 0.634984749 | 0.777424576 | NS | 0.197236705 |
| HMMR | -0.533007069 | 0.01545929 | 0.147951473 | Down | 1.810810447 |
| MAT2B | -0.251672752 | 0.004413634 | 0.093388262 | Down | 2.355203669 |
| TENM2 | 0.387907852 | 0.332064901 | 0.548846634 | NS | 0.478777027 |
| WWC1 | 0.462873201 | 0.057073194 | 0.250544431 | NS | 1.24356782 |
| RARS1 | -0.410942647 | 0.00148886 | 0.062253356 | Down | 2.827146073 |
| FBLL1 | 0.73532104 | 0.150089503 | NA | NS | 0.823649681 |
| PANK3 | -0.119346546 | 0.036508488 | 0.208452907 | Down | 1.43760615 |
| SLIT3 | 0.532726634 | 0.147018676 | 0.37264175 | NS | 0.832627492 |
| SPDL1 | -0.130121978 | 0.155766011 | 0.383185333 | NS | 0.807527301 |
| DOCK2 | -0.010151168 | 0.870115602 | 0.92673217 | NS | 0.060423044 |
| INSYN2B | 0.141065684 | 0.242106459 | 0.469918053 | NS | 0.615993625 |
| FOXI1 | 0.991823447 | 0.023758447 | NA | Up | 1.624181956 |
| C5orf58 | 0.157194752 | 0.070044688 | 0.270699199 | NS | 1.154624792 |
| LCP2 | 0.15144465 | 0.036071891 | 0.207679449 | Up | 1.442831095 |
| KCNIP1 | 0.685294522 | 0.116175881 | NA | NS | 0.934884026 |
| KCNMB1 | 0.245286343 | 0.082113742 | 0.288607877 | NS | 1.085584155 |
| GABRP | 0.455155057 | 0.29752868 | 0.519241167 | NS | 0.526471164 |
| RANBP17 | -0.23505644 | 0.279351664 | 0.503278325 | NS | 0.553848738 |
| TLX3 | 1.501430906 | 0.004665896 | NA | Up | 2.331064925 |
| NPM1 | -0.337617046 | 0.01009333 | 0.126199476 | Down | 1.995965509 |
| FGF18 | 0.521530241 | 0.230201434 | NA | NS | 0.637891975 |
| SMIM23 | 0.209970383 | 0.548973651 | NA | NS | 0.2604485 |
| FBXW11 | -0.086753401 | 0.059106467 | 0.253636182 | NS | 1.228365001 |
| STK10 | -0.094425145 | 0.352439021 | 0.566064118 | NS | 0.452916013 |
| EFCAB9 | 0.327003367 | 0.46479248 | NA | NS | 0.332740907 |
| UBTD2 | -0.083912019 | 0.288737102 | 0.511843768 | NS | 0.539497407 |
| SH3PXD2B | 0.411408855 | 0.099708752 | 0.314784866 | NS | 1.00126672 |
| NEURL1B | 0.548629739 | 0.030022168 | 0.193133954 | Up | 1.522557949 |
| DUSP1 | -0.186119519 | 0.190530234 | 0.420149522 | NS | 0.720036098 |
| ERGIC1 | 0.05388988 | 0.579308993 | 0.739044929 | NS | 0.237089729 |
| RPL26L1 | -0.367202804 | 0.074169341 | 0.276604177 | NS | 1.129775579 |
| ATP6V0E1 | -0.002659999 | 0.978675588 | 0.987485532 | NS | 0.009361245 |
| CREBRF | -0.05767605 | 0.429121879 | 0.626769724 | NS | 0.367419342 |
| BNIP1 | -0.207418653 | 0.054847379 | 0.247169359 | NS | 1.260844118 |
| NKX2-5 | 1.525924026 | 0.008845874 | NA | Up | 2.053259236 |
| STC2 | 0.82150054 | 0.063294526 | 0.261404175 | NS | 1.198633845 |
| BOD1 | -0.18330722 | 0.00769884 | 0.113974683 | Down | 2.113574703 |
| CPEB4 | 0.091165414 | 0.392698368 | 0.599318562 | NS | 0.405940904 |
| C5orf47 | 0.641580058 | 0.019579213 | 0.162444405 | Up | 1.708204759 |
| NSG2 | 0.603395178 | 0.104761142 | 0.320720021 | NS | 0.979799778 |
| MSX2 | 1.061833956 | 0.011096189 | NA | Up | 1.954826174 |
| DRD1 | 0.636129212 | 0.219916627 | NA | NS | 0.657741935 |
| SFXN1 | -0.071940201 | 0.196471537 | 0.426209538 | NS | 0.706700357 |
| HRH2 | 0.083934155 | 0.565011087 | 0.728925412 | NS | 0.24794303 |
| CPLX2 | 0.232104821 | 0.316742389 | 0.536302987 | NS | 0.499293812 |
| THOC3 | -0.042562458 | 0.605727049 | 0.758254148 | NS | 0.217723032 |
| FAM153B | 0.153487756 | 0.39229382 | 0.599004293 | NS | 0.406388534 |
| SIMC1 | 0.095090969 | 0.223090963 | 0.451824304 | NS | 0.651518022 |
| KIAA1191 | -0.194086744 | 0.004904856 | 0.096834757 | Down | 2.309373704 |
| ARL10 | 0.042527057 | 0.627116615 | 0.772725276 | NS | 0.202651693 |
| NOP16 | -0.112669496 | 0.151224399 | 0.377681699 | NS | 0.820378134 |
| HIGD2A | -0.120434941 | 0.197500094 | 0.427410856 | NS | 0.704432693 |
| CLTB | -0.260358887 | 0.001237025 | 0.057117621 | Down | 2.90762162 |
| FAF2 | -0.087239806 | 0.013891234 | 0.141394243 | NS | 1.857259166 |
| RNF44 | 0.013952264 | 0.921953048 | 0.957012587 | NS | 0.035291195 |
| CDHR2 | 0.162707835 | 0.199250533 | 0.428926322 | NS | 0.700600509 |
| GPRIN1 | 0.515337159 | 0.018786596 | 0.159116357 | Up | 1.726151895 |
| SNCB | 0.851269115 | 0.141506178 | NA | NS | 0.849224599 |
| EIF4E1B | 0.656536759 | 0.202648031 | NA | NS | 0.693257612 |
| TSPAN17 | -0.097750784 | 0.071845152 | 0.273458146 | NS | 1.143602534 |
| UNC5A | 0.379310614 | 0.009500346 | 0.122561596 | Up | 2.022260589 |
| HK3 | -0.04292781 | 0.671314196 | 0.804120658 | NS | 0.173074169 |
| UIMC1 | -0.096211036 | 0.284180206 | 0.507034518 | NS | 0.546406176 |
| ZNF346 | 0.038565266 | 0.719226212 | 0.834306778 | NS | 0.143134493 |
| FGFR4 | 0.762048214 | 0.023999656 | 0.175491741 | Up | 1.619794975 |
| NSD1 | 0.012562834 | 0.785468728 | 0.876214665 | NS | 0.104871101 |
| RAB24 | 0.167770422 | 0.098995372 | 0.313885091 | NS | 1.004385108 |
| MXD3 | 0.143297983 | 0.156473887 | 0.383636723 | NS | 0.805558129 |
| PRELID1 | -0.062906206 | 0.423408053 | 0.622593337 | NS | 0.373240886 |
| LMAN2 | -0.105951537 | 0.14883933 | 0.374840808 | NS | 0.827282294 |
| RGS14 | 0.109735587 | 0.218315995 | 0.447816652 | NS | 0.660914444 |
| SLC34A1 | 0.422418288 | 0.218741589 | 0.448245889 | NS | 0.660068638 |
| PFN3 | 1.758051514 | 0.016121223 | NA | Up | 1.792602021 |
| F12 | 0.183084259 | 0.262137771 | 0.488365675 | NS | 0.581470397 |
| GRK6 | 0.05861554 | 0.46895391 | 0.658139597 | NS | 0.328869839 |
| PRR7 | 0.083294398 | 0.569955415 | 0.732128494 | NS | 0.244159116 |
| DBN1 | 0.044017164 | 0.691681213 | 0.816889674 | NS | 0.16009402 |
| PDLIM7 | -0.072096861 | 0.691566372 | 0.81688906 | NS | 0.160166133 |
| DOK3 | 0.146670547 | 0.306849137 | 0.527666237 | NS | 0.513075093 |
| DDX41 | -0.148322713 | 0.017173803 | 0.154054413 | Down | 1.765133522 |
| FAM193B | 0.06110131 | 0.615735102 | 0.765059537 | NS | 0.210606087 |
| TMED9 | -0.129930767 | 0.099145436 | 0.314049068 | NS | 1.003727274 |
| B4GALT7 | -0.150046721 | 0.029098857 | 0.190161395 | Down | 1.536124067 |
| FAM153A | 0.12922186 | 0.363338232 | 0.57563916 | NS | 0.439688902 |
| PROP1 | 1.053593298 | 0.153551145 | NA | NS | 0.813746941 |
| N4BP3 | 0.103980025 | 0.426061069 | 0.624284262 | NS | 0.370528148 |
| RMND5B | -0.039420595 | 0.400502654 | 0.605341375 | NS | 0.397394602 |
| NHP2 | -0.186727667 | 0.072629297 | 0.274345075 | NS | 1.138888158 |
| GMCL2 | 0.522449086 | 0.254545911 | NA | NS | 0.594233876 |
| HNRNPAB | -0.192097164 | 0.007031852 | 0.110626998 | Down | 2.152930283 |
| PHYKPL | -0.026624079 | 0.728468914 | 0.839373872 | NS | 0.137588976 |
| COL23A1 | 0.543270948 | 0.072973327 | 0.274846078 | NS | 1.136835852 |
| CLK4 | 0.06296792 | 0.378457302 | 0.588455293 | NS | 0.421983111 |
| ZNF354A | -9.89E-04 | 0.992279801 | 0.995609559 | NS | 0.003365849 |
| ZNF354B | -0.003465349 | 0.960084504 | 0.977759486 | NS | 0.01769054 |
| ZFP2 | 0.042183619 | 0.783479949 | 0.874811457 | NS | 0.105972114 |
| ZNF454 | 0.316285741 | 0.433881166 | NA | NS | 0.362629201 |
| GRM6 | 0.60226574 | 0.140354729 | 0.36472336 | NS | 0.85277295 |
| ZNF879 | -0.128833213 | 0.149611658 | 0.375764013 | NS | 0.825034563 |
| ZNF354C | -0.110577384 | 0.178742122 | 0.408742094 | NS | 0.747773092 |
| ADAMTS2 | 0.979084961 | 0.004389516 | 0.093388262 | Up | 2.357583334 |
| RUFY1 | -0.057984786 | 0.535408984 | 0.707473838 | NS | 0.271314346 |
| HNRNPH1 | -0.201623327 | 9.91E-04 | 0.05219403 | Down | 3.003763319 |
| CANX | -0.315046938 | 2.09E-04 | 0.023858165 | Down | 3.680690886 |
| MAML1 | 0.191889633 | 0.196130213 | 0.425986774 | NS | 0.7074555 |
| LTC4S | 0.67179877 | 0.014868179 | NA | Up | 1.827742211 |
| MGAT4B | -0.058939028 | 0.544613606 | 0.7135756 | NS | 0.263911513 |
| SQSTM1 | -0.196685319 | 0.030462646 | 0.193793197 | Down | 1.516232382 |
| MRNIP | -0.310886395 | 0.015709274 | 0.148749691 | Down | 1.803843894 |
| TBC1D9B | -0.076252538 | 0.444710993 | 0.63886408 | NS | 0.351922135 |
| RNF130 | -0.034506915 | 0.738246375 | 0.846085096 | NS | 0.131798677 |
| RASGEF1C | 0.338641617 | 0.44266894 | NA | NS | 0.35392095 |
| MAPK9 | -0.045758108 | 0.351084352 | 0.565080739 | NS | 0.454588527 |
| GFPT2 | 0.120260713 | 0.631729862 | 0.775715902 | NS | 0.199468594 |
| CNOT6 | -0.065202089 | 0.445753031 | 0.639759098 | NS | 0.350905695 |
| SCGB3A1 | 0.475314127 | 0.286842111 | NA | NS | 0.54235709 |
| FLT4 | 0.192932505 | 0.306537332 | 0.52760544 | NS | 0.513516627 |
| OR2Y1 | 0.257888388 | 0.692005751 | NA | NS | 0.159890296 |
| MGAT1 | 0.002988964 | 0.959328702 | 0.977353459 | NS | 0.018032562 |
| ZFP62 | -0.071380516 | 0.31093908 | 0.530724994 | NS | 0.507324691 |
| BTNL8 | -0.020055937 | 0.907257031 | 0.948593888 | NS | 0.042269657 |
| BTNL3 | 0.125086311 | 0.714808856 | 0.831357536 | NS | 0.145810076 |
| BTNL9 | 0.165775752 | 0.53537637 | 0.707471561 | NS | 0.271340801 |
| OR2V1 | 0.876277513 | 0.092803176 | NA | NS | 1.03243716 |
| OR2V2 | 0.782047243 | 0.122098131 | NA | NS | 0.913290985 |
| TRIM7 | 0.02304783 | 0.82832277 | 0.902356851 | NS | 0.0818004 |
| TRIM41 | 0.019171729 | 0.696312947 | 0.820063721 | NS | 0.15719553 |
| RACK1 | -0.267766138 | 0.007798262 | 0.114557912 | Down | 2.108002205 |
| TRIM52 | -0.006911945 | 0.896516384 | 0.942008544 | NS | 0.047441769 |
| DUSP22 | -0.014758285 | 0.729143418 | 0.840103608 | NS | 0.13718704 |
| IRF4 | 0.04677751 | 0.517386068 | 0.694817724 | NS | 0.28618527 |
| EXOC2 | -0.070307332 | 0.211325997 | 0.441234413 | NS | 0.675047073 |
| HUS1B | 0.189632945 | 0.123930293 | 0.344979695 | NS | 0.906822525 |
| FOXQ1 | 0.885586281 | 0.041952227 | NA | Up | 1.377244977 |
| FOXF2 | 1.296100464 | 0.008810649 | NA | Up | 2.054992122 |
| FOXC1 | 0.674132433 | 0.041738438 | NA | Up | 1.37946381 |
| GMDS | 0.105731618 | 0.100500712 | 0.315859381 | NS | 0.997830862 |
| MYLK4 | -0.172707524 | 0.211525832 | 0.441234413 | NS | 0.674636587 |
| WRNIP1 | -0.090871816 | 0.028452241 | 0.188709674 | NS | 1.545883515 |
| SERPINB1 | 0.075165671 | 0.383487545 | 0.592439708 | NS | 0.416248737 |
| SERPINB9 | 0.130087539 | 0.015969615 | 0.149801122 | Up | 1.796705552 |
| SERPINB6 | -0.049298908 | 0.417091053 | 0.618106594 | NS | 0.379769126 |
| NQO2 | 0.175204737 | 0.100890305 | 0.316384536 | NS | 0.996150564 |
| RIPK1 | 0.082494516 | 0.051672144 | 0.240039644 | NS | 1.286743517 |
| BPHL | 0.105462865 | 0.234953266 | 0.462912912 | NS | 0.629018514 |
| TUBB2A | 0.482183891 | 0.108920985 | 0.327019978 | NS | 0.962888441 |
| TUBB2B | 0.849118352 | 0.022791344 | 0.171982657 | Up | 1.64223006 |
| PSMG4 | 0.039287067 | 0.503520765 | 0.684451126 | NS | 0.297982614 |
| SLC22A23 | 0.003530493 | 0.974311066 | 0.985403414 | NS | 0.011302365 |
| PXDC1 | 0.11235554 | 0.467297687 | 0.656970634 | NS | 0.330406368 |
| FAM50B | -0.023816735 | 0.802022148 | 0.88605457 | NS | 0.095813639 |
| PRPF4B | -0.034595934 | 0.363175495 | 0.575561911 | NS | 0.439883463 |
| FAM217A | 0.153251664 | 0.189997907 | 0.419365079 | NS | 0.721251184 |
| ECI2 | -0.219286031 | 0.002995993 | 0.082074431 | Down | 2.523459254 |
| CDYL | -0.54001496 | 1.90E-04 | 0.022816671 | Down | 3.720587757 |
| RPP40 | 0.004021456 | 0.969461839 | 0.982823994 | NS | 0.013469281 |
| PPP1R3G | 0.066309881 | 0.745823483 | 0.850639911 | NS | 0.127363946 |
| LYRM4 | -0.191650631 | 0.046933419 | 0.230411397 | Down | 1.328517804 |
| FARS2 | -0.060375774 | 0.26727041 | 0.493205152 | NS | 0.57304912 |
| NRN1 | 0.134278573 | 0.633535823 | 0.776709642 | NS | 0.198228823 |
| F13A1 | 0.06355977 | 0.66104311 | 0.796423237 | NS | 0.179770217 |
| LY86 | -0.280009327 | 0.03144783 | 0.196942357 | Down | 1.502409318 |
| RREB1 | 0.06720438 | 0.443721407 | 0.637917821 | NS | 0.352889619 |
| SSR1 | -0.078753242 | 0.190720218 | 0.420149522 | NS | 0.719603266 |
| CAGE1 | 0.272171547 | 0.32131501 | 0.539854476 | NS | 0.493068986 |
| RIOK1 | -0.085829064 | 0.116811885 | 0.336675787 | NS | 0.932512966 |
| DSP | 0.314009635 | 0.269143917 | 0.49444608 | NS | 0.570015431 |
| SNRNP48 | -0.088273968 | 0.060921937 | 0.25683088 | NS | 1.215226298 |
| BMP6 | 0.104801125 | 0.339546806 | 0.555251764 | NS | 0.46910035 |
| TXNDC5 | 0.123206226 | 0.420607955 | 0.620581631 | NS | 0.376122518 |
| BLOC1S5 | -0.263336023 | 3.63E-04 | 0.032535375 | Down | 3.440542946 |
| EEF1E1 | -0.142606057 | 0.256163935 | 0.483091769 | NS | 0.591482014 |
| SLC35B3 | -0.017063241 | 0.759019131 | 0.859313665 | NS | 0.119747278 |
| TFAP2A | 0.441780457 | 0.310711502 | 0.530523732 | NS | 0.50764267 |
| GCNT2 | -0.305431345 | 0.097983754 | 0.312744522 | NS | 1.008845925 |
| PAK1IP1 | -0.32238768 | 0.008068193 | 0.115486104 | Down | 2.093223699 |
| TMEM14C | -0.061227407 | 0.626446204 | 0.772167436 | NS | 0.203116218 |
| TMEM14B | -0.181586809 | 0.046788724 | 0.230265507 | Down | 1.329858797 |
| MAK | 0.213728842 | 0.094815904 | 0.30805417 | NS | 1.023118808 |
| GCM2 | 0.867910531 | 0.032491214 | NA | Up | 1.488234065 |
| SYCP2L | 0.458914043 | 0.060135101 | 0.255813968 | NS | 1.220871954 |
| ELOVL2 | 0.867760805 | 0.006424766 | NA | Up | 2.192142673 |
| SMIM13 | -0.076405254 | 0.20624993 | 0.436307756 | NS | 0.685606191 |
| ERVFRD-1 | 0.105241431 | 0.384492051 | 0.59312605 | NS | 0.415112635 |
| NEDD9 | 0.144443163 | 0.132460559 | 0.355399001 | NS | 0.877913418 |
| TMEM170B | 0.006398957 | 0.943359771 | 0.968956608 | NS | 0.025322648 |
| ADTRP | -0.139590295 | 0.37175257 | 0.582783036 | NS | 0.42974602 |
| HIVEP1 | 0.129621294 | 0.051637809 | 0.239928809 | NS | 1.287032194 |
| EDN1 | 0.358508239 | 0.036684181 | 0.208886406 | Up | 1.435521167 |
| PHACTR1 | 0.137403752 | 0.113433095 | 0.33220521 | NS | 0.945260218 |
| TBC1D7 | 0.162545646 | 0.006616112 | 0.108462559 | Up | 2.179397156 |
| GFOD1 | 0.191492411 | 0.022066977 | 0.170172208 | Up | 1.656257156 |
| SIRT5 | -0.236546654 | 0.020249262 | 0.164800499 | Down | 1.693590795 |
| NOL7 | -0.211250665 | 0.002435714 | 0.075642185 | Down | 2.613373634 |
| RANBP9 | -0.364901332 | 0.003418537 | 0.085194315 | Down | 2.466159696 |
| MCUR1 | -0.096515423 | 0.21070794 | 0.440576394 | NS | 0.676319098 |
| RNF182 | 0.474196435 | 0.189364515 | 0.418800923 | NS | 0.7227014 |
| CD83 | -0.281741037 | 0.018060223 | 0.156731802 | Down | 1.743276893 |
| JARID2 | -0.054089329 | 0.467160829 | 0.656899202 | NS | 0.33053358 |
| DTNBP1 | 0.203636666 | 2.59E-04 | 0.026702841 | Up | 3.586962964 |
| MYLIP | -0.122916002 | 0.089781719 | 0.30028957 | NS | 1.046812083 |
| GMPR | -0.174193484 | 0.402911707 | 0.607111252 | NS | 0.394790114 |
| ATXN1 | 0.186302663 | 0.026975622 | 0.185246086 | Up | 1.569028531 |
| STMND1 | 0.457421044 | 0.350268792 | NA | NS | 0.455598556 |
| RBM24 | 0.543248091 | 0.201313267 | NA | NS | 0.696127602 |
| CAP2 | 0.268520636 | 0.608711894 | NA | NS | 0.215588212 |
| FAM8A1 | 0.128989947 | 0.240474258 | 0.468406361 | NS | 0.618931407 |
| NUP153 | -0.107740936 | 0.096382048 | 0.3104996 | NS | 1.016003851 |
| KIF13A | 0.039747032 | 0.66930177 | 0.802573388 | NS | 0.174378026 |
| NHLRC1 | 0.072032461 | 0.655555557 | 0.793107943 | NS | 0.183390497 |
| TPMT | -0.307302787 | 0.006581537 | 0.108312793 | Down | 2.181672647 |
| KDM1B | 0.117210839 | 0.093229852 | 0.305676459 | NS | 1.030445006 |
| DEK | -0.312907779 | 0.004047252 | 0.090453197 | Down | 2.392839782 |
| RNF144B | -0.050216752 | 0.61144015 | 0.76220302 | NS | 0.213646047 |
| ID4 | 0.559347032 | 0.317364765 | NA | NS | 0.498441292 |
| MBOAT1 | 0.034772228 | 0.561383717 | 0.725799362 | NS | 0.250740188 |
| E2F3 | 0.120066232 | 0.158590827 | 0.386148484 | NS | 0.799721937 |
| CDKAL1 | -0.086030267 | 0.118388362 | 0.338814071 | NS | 0.92669099 |
| SOX4 | 0.032323386 | 0.779571459 | 0.872308102 | NS | 0.10814407 |
| PRL | 0.49804694 | 0.250723617 | NA | NS | 0.600804755 |
| HDGFL1 | 0.640522904 | 0.246177043 | NA | NS | 0.608752449 |
| NRSN1 | 0.401621777 | 0.410037168 | NA | NS | 0.387176774 |
| DCDC2 | 0.683624569 | 0.043737023 | 0.224236694 | Up | 1.359150777 |
| KAAG1 | 0.311045994 | 0.585271734 | NA | NS | 0.23264245 |
| MRS2 | 0.016445567 | 0.785080298 | 0.875872417 | NS | 0.105085921 |
| GPLD1 | -0.062840515 | 0.68884933 | 0.815257617 | NS | 0.161875759 |
| ALDH5A1 | -0.38691026 | 0.032397146 | 0.198810138 | Down | 1.489493248 |
| KIAA0319 | 0.354132364 | 0.022862209 | 0.172237653 | Up | 1.64088181 |
| TDP2 | 0.012002197 | 0.895051057 | 0.941159748 | NS | 0.04815219 |
| ACOT13 | 0.110482285 | 0.026947146 | 0.185246086 | Up | 1.569487222 |
| C6orf62 | -0.071134845 | 0.172915518 | 0.401822933 | NS | 0.76216603 |
| GMNN | -0.040592717 | 0.66530698 | 0.799541399 | NS | 0.17697792 |
| ARMH2 | 0.196359587 | 0.093953459 | 0.30723623 | NS | 1.027087225 |
| RIPOR2 | 0.194690359 | 0.007688508 | 0.113974683 | Up | 2.114157914 |
| CARMIL1 | 0.140238106 | 0.153432356 | 0.380087378 | NS | 0.814083047 |
| SCGN | 0.535716382 | 0.15226439 | NA | NS | 0.817401653 |
| H2AC1 | 0.628516875 | 0.399966631 | NA | NS | 0.39797624 |
| SLC17A4 | 0.623075581 | 0.171626314 | 0.400205856 | NS | 0.765416125 |
| SLC17A1 | 0.587870921 | 0.105169462 | NA | NS | 0.978110348 |
| SLC17A3 | 0.164027916 | 0.527000907 | 0.701952703 | NS | 0.278188637 |
| SLC17A2 | 0.712492074 | 0.12955022 | NA | NS | 0.887561844 |
| TRIM38 | 0.110416917 | 0.008483252 | 0.11782982 | Up | 2.071437644 |
| H1-1 | -0.001563269 | 0.995343417 | NA | NS | 0.002027051 |
| H3C1 | -0.453775016 | 0.008297542 | 0.117158014 | Down | 2.081050559 |
| H4C1 | -0.132238456 | 0.329949847 | 0.547302812 | NS | 0.481552068 |
| H4C2 | -0.250026984 | 0.058610162 | 0.252713796 | NS | 1.232027079 |
| H3C2 | 0.05668189 | 0.730406244 | 0.840464443 | NS | 0.136435523 |
| H2AC4 | 0.086420928 | 0.636353623 | 0.778587102 | NS | 0.196301479 |
| H2BC3 | -0.261548641 | 0.176273719 | 0.405730725 | NS | 0.753812433 |
| H3C3 | 0.143941955 | 0.402466336 | 0.606639727 | NS | 0.39527044 |
| H1-2 | -0.262624267 | 0.023677214 | 0.174639469 | Down | 1.625669404 |
| HFE | 0.067413459 | 0.377199055 | 0.587221315 | NS | 0.423429404 |
| H4C3 | -0.246013396 | 0.066792419 | 0.266334178 | NS | 1.175272827 |
| H1-6 | 0.199682151 | 0.185715297 | 0.415463304 | NS | 0.731152323 |
| H2BC4 | -0.005446077 | 0.957610904 | 0.976655159 | NS | 0.018810917 |
| H2AC6 | 0.018358997 | 0.837348721 | 0.908571347 | NS | 0.077093639 |
| H1-4 | -0.154743788 | 0.08721301 | 0.296703312 | NS | 1.059418727 |
| H2BC5 | -0.28850406 | 0.013702056 | 0.140656425 | Down | 1.863214266 |
| H2BC6 | -0.032631436 | 0.738966248 | 0.846651166 | NS | 0.131375397 |
| H4C4 | -0.110255889 | 0.417573775 | 0.618621812 | NS | 0.379266785 |
| H3C4 | -0.040300525 | 0.91396157 | NA | NS | 0.039072065 |
| H2AC7 | -0.294205448 | 0.114639886 | 0.33336367 | NS | 0.940664255 |
| H2BC7 | -0.152521678 | 0.162997127 | 0.390737932 | NS | 0.787820051 |
| H4C5 | -0.245577376 | 0.044495306 | 0.225674429 | Down | 1.351685798 |
| H2BC8 | -0.244012481 | 0.0686286 | 0.268602708 | NS | 1.163494862 |
| H2AC8 | -0.233768429 | 0.068274688 | 0.268476763 | NS | 1.165740278 |
| H3C6 | -0.139204315 | 0.248323588 | 0.476110795 | NS | 0.604982026 |
| H1-3 | -0.36325704 | 0.009226251 | 0.12133665 | Down | 2.034974733 |
| H4C6 | -0.194292265 | 0.223629045 | 0.452317462 | NS | 0.65047179 |
| H3C7 | 0.017799212 | 0.911153279 | 0.950817021 | NS | 0.040408557 |
| H2BC9 | -0.08922647 | 0.423223178 | 0.622593337 | NS | 0.373430556 |
| H3C8 | 0.280219456 | 0.16993913 | 0.398309642 | NS | 0.769706608 |
| H2BC10 | -0.282047516 | 0.059698397 | 0.25488678 | NS | 1.22403733 |
| H4C8 | -0.164172799 | 0.069544233 | 0.270000522 | NS | 1.157738878 |
| BTN3A2 | 0.04222609 | 0.41426068 | 0.616044456 | NS | 0.382726287 |
| BTN2A2 | 0.197620466 | 0.005389213 | 0.100338483 | Up | 2.268474676 |
| BTN3A1 | 0.022539105 | 0.691771428 | 0.816889686 | NS | 0.16003738 |
| BTN3A3 | -0.034833125 | 0.54403968 | 0.713285597 | NS | 0.264369424 |
| BTN2A1 | 0.233429631 | 0.011601571 | 0.132180121 | Up | 1.935483211 |
| BTN1A1 | 0.337105379 | 0.050757575 | 0.238451278 | NS | 1.294499136 |
| HMGN4 | -0.198643802 | 0.029138813 | 0.190232923 | Down | 1.535528144 |
| ABT1 | -0.119875118 | 0.104669043 | 0.32065922 | NS | 0.980181747 |
| ZNF322 | -0.078187539 | 0.317864474 | 0.537121802 | NS | 0.497758008 |
| H2BC11 | -0.096312264 | 0.367607139 | 0.57965703 | NS | 0.434616063 |
| H2AC11 | -0.014463066 | 0.912344839 | 0.951241312 | NS | 0.03984098 |
| H4C9 | 0.009089466 | 0.947776838 | 0.971619747 | NS | 0.023293909 |
| H2BC12 | -0.096700258 | 0.329113766 | 0.546661116 | NS | 0.482653952 |
| H2AC12 | -0.143125143 | 0.421746394 | 0.621413862 | NS | 0.374948622 |
| PRSS16 | 0.472601635 | 0.112793784 | 0.331392333 | NS | 0.947714834 |
| POM121L2 | 0.356353758 | 0.466942294 | NA | NS | 0.330736788 |
| ZNF391 | 0.363716329 | 0.036772184 | 0.209023715 | Up | 1.434480581 |
| ZNF184 | -0.237043408 | 0.005229495 | 0.098879942 | Down | 2.281540267 |
| H2BC13 | -0.183493145 | 0.237004113 | 0.46479433 | NS | 0.625244118 |
| H2AC13 | -0.071731786 | 0.555159104 | 0.721803766 | NS | 0.255582534 |
| H3C10 | 0.069228226 | 0.594743909 | 0.750384214 | NS | 0.225669997 |
| H2AC14 | 0.06275205 | 0.740459276 | 0.847850763 | NS | 0.130498822 |
| H2BC14 | 0.175515627 | 0.281930144 | 0.504917658 | NS | 0.549858487 |
| H4C11 | -0.456435265 | 0.001780164 | 0.065969754 | Down | 2.749540072 |
| H4C12 | -0.085798439 | 0.534931202 | 0.707211339 | NS | 0.27170207 |
| H2AC15 | -0.221260926 | 0.134022624 | 0.357528961 | NS | 0.872821885 |
| H2BC15 | -0.031511815 | 0.758664716 | 0.859039816 | NS | 0.119950114 |
| H2AC16 | -0.064647971 | 0.660881429 | 0.796312226 | NS | 0.179876452 |
| H1-5 | 0.006920273 | 0.965319949 | 0.980581176 | NS | 0.015328719 |
| H3C11 | 0.266053823 | 0.020968778 | 0.167842555 | Up | 1.678426887 |
| H4C13 | 0.062629703 | 0.642446836 | 0.783722102 | NS | 0.192162806 |
| H3C12 | 0.133449154 | 0.474771874 | 0.662880294 | NS | 0.323515017 |
| H2AC17 | 0.060889739 | 0.863681113 | NA | NS | 0.063646578 |
| H2BC17 | 0.036136244 | 0.817528404 | 0.896255005 | NS | 0.08749715 |
| OR2B2 | 0.161208175 | 0.439603442 | 0.634791805 | NS | 0.356938916 |
| OR2B6 | -0.07985975 | 0.719172105 | NA | NS | 0.143167166 |
| ZNF165 | -0.101931569 | 0.490640082 | 0.674954298 | NS | 0.309236976 |
| ZSCAN16 | -0.002035531 | 0.971653439 | 0.983723402 | NS | 0.012488608 |
| ZKSCAN8 | 0.11490824 | 0.075226596 | 0.278206739 | NS | 1.123628592 |
| ZKSCAN8P1 | 0.217220945 | 0.059016962 | 0.253419931 | NS | 1.22902315 |
| ZSCAN9 | 0.011124028 | 0.861362435 | 0.921775734 | NS | 0.064814072 |
| ZKSCAN4 | -0.040893677 | 0.405256677 | 0.609001865 | NS | 0.392269821 |
| NKAPL | -0.213630677 | 0.163650706 | 0.391382977 | NS | 0.786082118 |
| ZSCAN26 | -0.133608742 | 0.008410927 | 0.117652418 | Down | 2.075156149 |
| PGBD1 | 0.005668018 | 0.950655541 | 0.973306371 | NS | 0.021976816 |
| ZSCAN31 | 0.064156244 | 0.521031415 | 0.696811896 | NS | 0.28313609 |
| ZKSCAN3 | 0.076295542 | 0.191634906 | 0.420934764 | NS | 0.717525382 |
| ZSCAN12 | -0.032094936 | 0.595559638 | 0.750945369 | NS | 0.225074743 |
| ZSCAN23 | 0.150764422 | 0.388866203 | 0.596604696 | NS | 0.410199801 |
| GPX6 | 0.099908919 | 0.836204756 | NA | NS | 0.077687367 |
| ZBED9 | -0.04449276 | 0.924650973 | NA | NS | 0.034022169 |
| TRIM27 | 0.120031748 | 0.063236681 | 0.261404175 | NS | 1.199030936 |
| GABBR1 | -0.202656946 | 0.30929412 | 0.529643631 | NS | 0.509628337 |
| ZFP57 | -0.413419258 | 0.493299103 | NA | NS | 0.306889674 |
| HLA-F | -0.117856083 | 0.100244043 | 0.315357865 | NS | 0.998941427 |
| HLA-G | -0.654013451 | 0.00653576 | NA | Down | 2.184703923 |
| HLA-A | -0.077324542 | 0.566772026 | 0.730292165 | NS | 0.246591593 |
| POLR1H | -0.237793024 | 0.010289749 | 0.126492668 | Down | 1.987595202 |
| PPP1R11 | 0.06906929 | 0.596907658 | 0.751983842 | NS | 0.224092849 |
| TRIM40 | 0.210388503 | 0.479296711 | NA | NS | 0.319395552 |
| TRIM10 | 0.048624109 | 0.77410735 | 0.868209356 | NS | 0.111198809 |
| TRIM26 | -0.054942437 | 0.559230173 | 0.724299241 | NS | 0.252409405 |
| HLA-E | -0.038615233 | 0.660368239 | 0.795945129 | NS | 0.180213823 |
| GNL1 | -0.298051441 | 5.88E-04 | 0.04060164 | Down | 3.23057353 |
| PRR3 | 0.253036525 | 0.088663925 | 0.298878601 | NS | 1.052253046 |
| ABCF1 | -0.279445585 | 4.00E-04 | 0.034465889 | Down | 3.397602293 |
| PPP1R10 | -0.040242889 | 0.728391009 | 0.839326334 | NS | 0.137635423 |
| MRPS18B | -0.251221337 | 0.015940078 | 0.149715225 | Down | 1.797509548 |
| ATAT1 | 0.096278879 | 0.600607845 | 0.754697732 | NS | 0.221409 |
| C6orf136 | -0.022489683 | 0.869827315 | 0.926634346 | NS | 0.060566958 |
| DHX16 | -0.250017404 | 5.11E-05 | 0.010267543 | Down | 4.291502037 |
| PPP1R18 | 0.194946301 | 0.064960262 | 0.264064926 | NS | 1.187352232 |
| NRM | 0.813955907 | 0.002101459 | NA | Up | 2.677479104 |
| MDC1 | 0.005289198 | 0.965429834 | 0.980611497 | NS | 0.015279285 |
| TUBB | -0.190218511 | 0.023653883 | 0.174604313 | Down | 1.626097552 |
| FLOT1 | 0.080752536 | 0.341439574 | 0.55696544 | NS | 0.466686144 |
| IER3 | 0.720647794 | 0.04640942 | NA | Up | 1.333393859 |
| DDR1 | -0.153004687 | 0.376717809 | 0.586871047 | NS | 0.423983848 |
| PSORS1C1 | 0.274196572 | 0.774391915 | NA | NS | 0.11103919 |
| CCHCR1 | -0.285329488 | 0.003208838 | 0.084356647 | Down | 2.493652255 |
| TCF19 | -0.161656662 | 0.3758071 | 0.586170007 | NS | 0.425035019 |
| POU5F1 | 0.319652367 | 0.291313373 | NA | NS | 0.535639578 |
| HLA-C | 0.191323649 | 0.540783479 | 0.710965743 | NS | 0.266976585 |
| HLA-B | 0.038779139 | 0.766361996 | 0.863996378 | NS | 0.11556604 |
| MICA | -0.076736329 | 0.594566535 | 0.750384214 | NS | 0.225799539 |
| MICB | -0.068174124 | 0.40414594 | 0.607851206 | NS | 0.39346178 |
| DDX39B | 0.665428572 | 0.02576814 | NA | Up | 1.588916922 |
| NFKBIL1 | -0.042044758 | 0.773286741 | 0.867908648 | NS | 0.111659436 |
| TNF | 0.138006962 | 0.625640896 | NA | NS | 0.20367487 |
| LTB | -0.084084629 | 0.396182267 | 0.601442813 | NS | 0.402104967 |
| LST1 | -0.10681338 | 0.310552464 | 0.530428918 | NS | 0.50786502 |
| NCR3 | -0.165765056 | 0.303203243 | 0.524458861 | NS | 0.518266158 |
| AIF1 | -0.128682287 | 0.360687566 | 0.573444403 | NS | 0.442868829 |
| PRRC2A | -0.023335045 | 0.900751051 | 0.944854146 | NS | 0.045395222 |
| BAG6 | -0.190729592 | 0.322327735 | 0.540921067 | NS | 0.491702323 |
| APOM | -0.094755437 | 0.589037934 | 0.746962722 | NS | 0.229856736 |
| GPANK1 | -0.051987073 | 0.561017276 | 0.725489421 | NS | 0.251023765 |
| CSNK2B | 0.316713827 | 0.030198797 | 0.193469884 | Up | 1.520010353 |
| ABHD16A | 0.028405522 | 0.78809073 | 0.877819848 | NS | 0.103423781 |
| LY6G6F | 0.111085867 | 0.553489955 | 0.720495127 | NS | 0.256890257 |
| MPIG6B | 0.156348257 | 0.67926201 | 0.808797872 | NS | 0.167962674 |
| DDAH2 | -0.108660446 | 0.270263299 | 0.495760518 | NS | 0.568212927 |
| CLIC1 | -0.109038937 | 0.336643219 | 0.552913838 | NS | 0.472830129 |
| VWA7 | 0.438875608 | 0.171384532 | NA | NS | 0.766028377 |
| VARS1 | -0.117296624 | 0.277541131 | 0.501976536 | NS | 0.556672647 |
| LSM2 | 0.342818266 | 0.034297381 | 0.203333841 | Up | 1.464739037 |
| NEU1 | -0.163508336 | 0.095540352 | 0.309119979 | NS | 1.019813165 |
| SLC44A4 | -0.00437466 | 0.991705171 | NA | NS | 0.003617422 |
| EHMT2 | -0.191014899 | 0.062057437 | 0.258716142 | NS | 1.207206166 |
| C2 | -0.18917128 | 0.584017932 | NA | NS | 0.233573818 |
| NELFE | -0.024607134 | 0.676939884 | 0.807104056 | NS | 0.169449897 |
| SKIV2L | -0.013733552 | 0.831163424 | 0.90403233 | NS | 0.080313577 |
| DXO | -0.10820826 | 0.401359679 | 0.60584889 | NS | 0.39646626 |
| STK19 | -0.128139631 | 0.12031229 | 0.340929581 | NS | 0.919690007 |
| C4A | 0.480600874 | 0.065388425 | NA | NS | 1.184499122 |
| C4B | 0.596349175 | 0.140805356 | NA | NS | 0.851380826 |
| TNXB | 0.013205851 | 0.961097519 | 0.978517825 | NS | 0.017232544 |
| ATF6B | 0.054088615 | 0.636630885 | 0.778686207 | NS | 0.196112296 |
| FKBPL | 0.031474601 | 0.922996191 | NA | NS | 0.034800091 |
| AGPAT1 | 0.461742043 | 0.127104858 | NA | NS | 0.895837849 |
| RNF5 | -0.21462691 | 0.006042501 | 0.103993354 | Down | 2.218783241 |
| AGER | -0.334701236 | 0.077361771 | 0.281652078 | NS | 1.111473597 |
| PBX2 | -0.126075045 | 0.146239622 | 0.371713762 | NS | 0.834934945 |
| GPSM3 | 0.456089084 | 0.002229296 | 0.072832159 | Up | 2.651832206 |
| NOTCH4 | -0.171579694 | 0.537756257 | NA | NS | 0.269414528 |
| HLA-DRA | -0.135586009 | 0.31788267 | 0.537121802 | NS | 0.497733148 |
| HLA-DRB5 | 1.919159123 | 1.74E-08 | 1.21E-05 | Up | 7.759692012 |
| HLA-DRB1 | 0.535338829 | 0.003971684 | 0.090040171 | Up | 2.401025278 |
| HLA-DQA1 | 0.922720787 | 0.01392444 | 0.141407346 | Up | 1.85622225 |
| HLA-DQB1 | 1.061065215 | 0.040864761 | 0.218105862 | Up | 1.388651035 |
| HLA-DQA2 | -0.647687518 | 0.071068786 | 0.272222334 | NS | 1.148321103 |
| HLA-DOB | -0.102962873 | 0.440026222 | 0.635045158 | NS | 0.356521443 |
| TAP2 | 0.362556648 | 0.185270581 | 0.415416766 | NS | 0.732193537 |
| PSMB8 | -0.062147794 | 0.530605648 | 0.704623132 | NS | 0.275228132 |
| PSMB9 | -0.078735723 | 0.458102577 | 0.649541527 | NS | 0.339037265 |
| TAP1 | -0.078182166 | 0.492872645 | 0.676724779 | NS | 0.307265285 |
| HLA-DMB | -0.303130634 | 0.01280945 | 0.136078062 | Down | 1.892469512 |
| HLA-DMA | -0.175962628 | 0.104996277 | 0.320907849 | NS | 0.9788261 |
| BRD2 | 0.038552733 | 0.497598896 | 0.679732613 | NS | 0.303120592 |
| HLA-DOA | -0.307979581 | 0.020115502 | 0.164530441 | Down | 1.696469118 |
| HLA-DPA1 | -0.208989928 | 0.077297445 | 0.281620438 | NS | 1.111834863 |
| HLA-DPB1 | -0.267665158 | 0.035229295 | 0.205507211 | Down | 1.453096049 |
| COL11A2 | -0.33558181 | 0.226848151 | NA | NS | 0.644264757 |
| RXRB | -0.212566212 | 9.40E-04 | 0.051014532 | Down | 3.027002197 |
| SLC39A7 | -0.153803629 | 0.090828735 | 0.301866156 | NS | 1.041776736 |
| HSD17B8 | -0.222140009 | 0.055696336 | 0.248760687 | NS | 1.254173371 |
| RING1 | -0.251583137 | 0.00487868 | 0.096737261 | Down | 2.311697638 |
| VPS52 | -0.263670757 | 1.76E-04 | 0.021642631 | Down | 3.753984758 |
| RPS18 | -0.269872834 | 0.111545868 | 0.329942771 | NS | 0.952546513 |
| WDR46 | -0.332899902 | 3.24E-04 | 0.030244481 | Down | 3.49006119 |
| PFDN6 | -0.314567591 | 0.00964988 | 0.123395617 | Down | 2.015478091 |
| RGL2 | -0.029744746 | 0.726686905 | 0.838611255 | NS | 0.138652666 |
| TAPBP | 0.328204169 | 0.005188469 | 0.098827133 | Up | 2.284960801 |
| DAXX | 0.337302961 | 0.053166655 | 0.243427175 | NS | 1.274360663 |
| KIFC1 | -0.032925663 | 0.879298755 | NA | NS | 0.055863542 |
| PHF1 | -0.049678597 | 0.414552322 | 0.616134093 | NS | 0.382420648 |
| CUTA | -0.06336589 | 0.486681428 | 0.672011218 | NS | 0.312755226 |
| SYNGAP1 | -0.046051497 | 0.808238254 | 0.889703075 | NS | 0.092460598 |
| BAK1 | -0.021420017 | 0.761369939 | 0.860657491 | NS | 0.118404274 |
| ITPR3 | -0.144639855 | 0.185185385 | 0.415384315 | NS | 0.732393292 |
| UQCC2 | -0.067434877 | 0.349039067 | 0.563100091 | NS | 0.457125961 |
| IP6K3 | 0.5948925 | 0.195494802 | NA | NS | 0.708864785 |
| LEMD2 | 0.010231414 | 0.734914174 | 0.843669396 | NS | 0.133763377 |
| MLN | 0.824256453 | 0.035227242 | NA | Up | 1.453121353 |
| GRM4 | 0.582662631 | 0.135159928 | 0.35893236 | NS | 0.869152048 |
| HMGA1 | 0.261510315 | 0.02136738 | 0.168379028 | Up | 1.670248728 |
| SMIM29 | -0.034456635 | 0.615869253 | 0.765059537 | NS | 0.210511478 |
| NUDT3 | 0.202310061 | 0.084769338 | 0.292906969 | NS | 1.071761206 |
| RPS10 | -0.154038491 | 0.054286025 | 0.246433804 | NS | 1.265311959 |
| PACSIN1 | 0.517202941 | 0.067434308 | 0.267332615 | NS | 1.171119096 |
| SPDEF | 1.021594346 | 0.047859031 | NA | Up | 1.320036097 |
| ILRUN | -0.185045059 | 0.002225838 | 0.072832159 | Down | 2.652506492 |
| SNRPC | 0.438472775 | 1.95E-04 | 0.023041407 | Up | 3.710566354 |
| UHRF1BP1 | 0.064755077 | 0.297066332 | 0.51890879 | NS | 0.527146566 |
| TAF11 | -0.076958074 | 0.100380074 | 0.315566845 | NS | 0.998352486 |
| ANKS1A | 0.025388711 | 0.746958677 | 0.851383448 | NS | 0.126703423 |
| TCP11 | 0.421901208 | 0.286905013 | NA | NS | 0.542261864 |
| SCUBE3 | 0.119989749 | 0.27777972 | 0.502108221 | NS | 0.556299464 |
| ZNF76 | -0.095471018 | 0.153546762 | 0.380233538 | NS | 0.813759338 |
| DEF6 | -0.09762393 | 0.107682566 | 0.32500608 | NS | 0.967854602 |
| PPARD | -0.033252316 | 0.806611342 | 0.888681049 | NS | 0.093335676 |
| FANCE | -0.076077992 | 0.293492407 | 0.515457294 | NS | 0.53240313 |
| RPL10A | -0.304632648 | 0.024281564 | 0.176550027 | Down | 1.614723352 |
| TEAD3 | 0.5178251 | 0.036915026 | 0.209371187 | Up | 1.432796824 |
| TULP1 | 1.018131584 | 0.023137717 | 0.173122445 | Up | 1.635679495 |
| FKBP5 | 0.179043248 | 0.115540063 | 0.334479283 | NS | 0.9372674 |
| ARMC12 | 0.237251453 | 0.27149194 | NA | NS | 0.56624306 |
| CLPSL2 | 1.849370862 | 0.026593042 | NA | Up | 1.575231983 |
| CLPSL1 | 1.372083931 | 0.023435561 | NA | Up | 1.630124637 |
| CLPS | 0.323810986 | 0.6635229 | NA | NS | 0.178144084 |
| LHFPL5 | 0.29289257 | 0.024369115 | 0.176894283 | Up | 1.613160248 |
| SRPK1 | 0.157251387 | 0.114185323 | 0.332664071 | NS | 0.942389716 |
| SLC26A8 | 0.40371169 | 0.010528201 | 0.127913447 | Up | 1.977645852 |
| MAPK14 | 0.202196443 | 0.049988818 | 0.236733572 | Up | 1.301127131 |
| MAPK13 | 0.167125921 | 0.049155538 | 0.234974405 | Up | 1.308427543 |
| BRPF3 | -0.038744743 | 0.711509931 | 0.829642098 | NS | 0.147819034 |
| PNPLA1 | 0.238621368 | 0.119604879 | 0.34007656 | NS | 0.922251105 |
| BNIP5 | 0.398583607 | 0.323190006 | NA | NS | 0.490542077 |
| ETV7 | 0.14242967 | 0.566675708 | 0.730251058 | NS | 0.246665404 |
| PXT1 | 0.699701227 | 0.024738645 | NA | Up | 1.606624096 |
| KCTD20 | -0.117390483 | 0.022982428 | 0.172628257 | Down | 1.638604101 |
| STK38 | -0.031532628 | 0.348459238 | 0.56264736 | NS | 0.457848017 |
| SRSF3 | -0.161193334 | 0.015213719 | 0.147378943 | Down | 1.817764599 |
| CDKN1A | 0.265692707 | 0.047696054 | 0.232004796 | Up | 1.321517546 |
| RAB44 | 0.363351309 | 0.011608108 | 0.132180121 | Up | 1.935238558 |
| CPNE5 | 0.284671664 | 0.028695248 | 0.189026296 | Up | 1.542190017 |
| PPIL1 | -0.245307265 | 0.010061585 | 0.126199476 | Down | 1.997333583 |
| C6orf89 | -0.105845373 | 0.03666601 | 0.208886406 | Down | 1.435736343 |
| PI16 | -0.362560759 | 0.003352869 | 0.085084692 | Down | 2.474583478 |
| MTCH1 | 0.053644427 | 0.226718952 | 0.455304642 | NS | 0.644512175 |
| FGD2 | -0.037712775 | 0.741485695 | 0.848481332 | NS | 0.129897223 |
| PIM1 | -0.295179493 | 0.067737563 | 0.267671219 | NS | 1.169170433 |
| TMEM217 | 0.200802674 | 0.380292981 | 0.590232438 | NS | 0.41988169 |
| TBC1D22B | -0.233862101 | 0.125795641 | 0.34748182 | NS | 0.900334408 |
| RNF8 | -0.115492219 | 0.04113178 | 0.218408859 | Down | 1.385822501 |
| CMTR1 | -0.042683094 | 0.448036636 | 0.641635461 | NS | 0.348686472 |
| CCDC167 | 0.044902983 | 0.68540171 | 0.813023826 | NS | 0.164054816 |
| MDGA1 | 0.300861765 | 0.2521084 | 0.479033151 | NS | 0.598412685 |
| ZFAND3 | 0.094989517 | 0.274961669 | 0.499616959 | NS | 0.560727844 |
| BTBD9 | 0.033921607 | 0.480240957 | 0.667059582 | NS | 0.318540804 |
| GLO1 | -0.271894231 | 0.014239094 | 0.143336863 | Down | 1.84651763 |
| DNAH8 | 0.256777572 | 0.213866024 | 0.443234232 | NS | 0.669858204 |
| GLP1R | 0.558682401 | 0.059044375 | 0.253419931 | NS | 1.228821471 |
| SAYSD1 | -0.049295171 | 0.455970761 | 0.647822129 | NS | 0.341063006 |
| KCNK5 | 0.197675547 | 0.239522371 | 0.46763242 | NS | 0.620653918 |
| KCNK17 | 0.283359713 | 0.332322563 | NA | NS | 0.478440172 |
| KCNK16 | 0.466183426 | 0.34085269 | NA | NS | 0.467433275 |
| KIF6 | 0.498129586 | 0.206713765 | 0.43664994 | NS | 0.684630603 |
| DAAM2 | 0.352061436 | 0.228219231 | 0.456877344 | NS | 0.641647763 |
| MOCS1 | 0.102583977 | 0.710372318 | 0.828864216 | NS | 0.14851397 |
| LRFN2 | 0.229828693 | 0.353366478 | 0.566802009 | NS | 0.451774652 |
| UNC5CL | 0.107475942 | 0.207301463 | 0.437271578 | NS | 0.683397632 |
| OARD1 | -0.040512648 | 0.409986108 | 0.613001849 | NS | 0.387230859 |
| TSPO2 | 0.099864936 | 0.55681538 | 0.722631716 | NS | 0.254288777 |
| APOBEC2 | 0.13943357 | 0.098685994 | 0.313511809 | NS | 1.005744481 |
| NFYA | 0.111137548 | 0.035698784 | 0.206510117 | Up | 1.447346576 |
| TREML1 | 0.540170377 | 0.001067867 | 0.053165848 | Up | 2.971482837 |
| TREM2 | 0.417712005 | 0.114171979 | NA | NS | 0.942440471 |
| TREML2 | 0.119791764 | 0.283950203 | 0.506782072 | NS | 0.546757817 |
| TREML4 | 0.086706712 | 0.636628185 | 0.778686207 | NS | 0.196114138 |
| TREM1 | 0.203738167 | 0.096464326 | 0.310542373 | NS | 1.015633267 |
| NCR2 | 0.798437309 | 0.146703436 | NA | NS | 0.833559715 |
| FOXP4 | 0.177053255 | 0.468722925 | 0.6580408 | NS | 0.329083805 |
| MDFI | 0.672482417 | 0.196434123 | NA | NS | 0.706783069 |
| TFEB | 0.22407687 | 0.010433683 | 0.127457165 | Up | 1.981562373 |
| PGC | 0.183653702 | 0.619936765 | NA | NS | 0.207652608 |
| FRS3 | 0.206389723 | 0.152404873 | 0.379041408 | NS | 0.817001146 |
| PRICKLE4 | 0.213715969 | 0.39736885 | NA | NS | 0.40080618 |
| USP49 | 0.122256322 | 0.106970292 | 0.32383789 | NS | 0.970736817 |
| MED20 | -0.019776891 | 0.745767027 | 0.850639911 | NS | 0.127396822 |
| BYSL | 0.082686479 | 0.3357011 | 0.551832552 | NS | 0.474047236 |
| CCND3 | -0.00421483 | 0.9515515 | 0.973658853 | NS | 0.021567702 |
| TAF8 | -0.006377516 | 0.877194244 | 0.93114728 | NS | 0.056904227 |
| C6orf132 | 0.481418145 | 0.18439376 | 0.414505879 | NS | 0.734253779 |
| GUCA1ANB | 0.871377502 | 0.143183713 | NA | NS | 0.84410638 |
| GUCA1A | 0.414351216 | 0.436337506 | NA | NS | 0.360177455 |
| GUCA1B | -0.197966801 | 0.08817287 | 0.298122562 | NS | 1.054665022 |
| MRPS10 | -0.202067536 | 0.007570769 | 0.113104804 | Down | 2.120860023 |
| TRERF1 | 0.154111946 | 0.062022792 | 0.258700409 | NS | 1.207448685 |
| UBR2 | -0.093742541 | 0.327369176 | 0.545360849 | NS | 0.484962215 |
| PRPH2 | 0.205289788 | 0.050678624 | 0.238391305 | NS | 1.295175188 |
| TBCC | -0.115822333 | 0.147797338 | 0.373699795 | NS | 0.830333389 |
| BICRAL | 0.127180736 | 0.076366019 | 0.28014329 | NS | 1.117099851 |
| RPL7L1 | -0.200756629 | 0.001320848 | 0.059001229 | Down | 2.879147248 |
| C6orf226 | 0.038956967 | 0.74850026 | 0.852165091 | NS | 0.125808044 |
| PTCRA | 0.158586401 | 0.275928539 | 0.500539833 | NS | 0.559203379 |
| CNPY3 | 0.022393478 | 0.726758497 | 0.838611255 | NS | 0.138609882 |
| GNMT | -0.092904965 | 0.549834705 | 0.717551679 | NS | 0.259767852 |
| PEX6 | -0.09611707 | 0.195499206 | 0.425156472 | NS | 0.708855003 |
| PPP2R5D | 0.139900375 | 0.07842994 | 0.283089438 | NS | 1.105518117 |
| MEA1 | -0.181921952 | 0.010286916 | 0.126492668 | Down | 1.987714807 |
| KLHDC3 | -0.144675735 | 0.018082725 | 0.156740993 | Down | 1.742736122 |
| RRP36 | -0.185733823 | 0.001915298 | 0.068752597 | Down | 2.717763626 |
| CUL7 | 0.108851637 | 0.239619561 | 0.467680858 | NS | 0.620477731 |
| KLC4 | -0.060151825 | 0.304094554 | 0.525375187 | NS | 0.516991357 |
| MRPL2 | 0.083374521 | 0.507031942 | 0.687026733 | NS | 0.29496468 |
| PTK7 | 0.166039022 | 0.328164737 | 0.545886338 | NS | 0.483908088 |
| SRF | 0.078037487 | 0.136437714 | 0.360986197 | NS | 0.865065567 |
| CUL9 | -0.024152716 | 0.772667115 | 0.867602583 | NS | 0.112007571 |
| DNPH1 | -0.135455391 | 0.249381358 | 0.47650024 | NS | 0.603136014 |
| TTBK1 | 0.651724509 | 0.153647978 | 0.380291042 | NS | 0.813473151 |
| SLC22A7 | 0.252906295 | 0.488703976 | 0.673300418 | NS | 0.310954128 |
| CRIP3 | -0.002661107 | 0.985786232 | 0.991457393 | NS | 0.006217252 |
| ZNF318 | -0.007873112 | 0.910206161 | 0.950332414 | NS | 0.040860229 |
| ABCC10 | -0.051577244 | 0.537117874 | 0.708735809 | NS | 0.269930395 |
| DLK2 | 0.169546143 | 0.368387718 | 0.580230409 | NS | 0.433694858 |
| TJAP1 | 0.072537831 | 0.475162976 | 0.663224188 | NS | 0.323157406 |
| LRRC73 | 0.648062986 | 0.060298879 | NA | NS | 1.219690761 |
| POLR1C | -0.137727883 | 0.018253057 | 0.157108372 | Down | 1.738664398 |
| YIPF3 | 0.143780295 | 0.138642854 | 0.363045809 | NS | 0.85810251 |
| XPO5 | 0.016179309 | 0.756961074 | 0.858044076 | NS | 0.120926453 |
| POLH | -0.040498862 | 0.237904792 | 0.465922314 | NS | 0.62359681 |
| GTPBP2 | 0.179922704 | 0.021618687 | 0.168842295 | Up | 1.665170676 |
| MAD2L1BP | 0.01275364 | 0.841421827 | 0.91060593 | NS | 0.074986226 |
| RSPH9 | 0.1950966 | 0.040105321 | 0.216115778 | Up | 1.396798005 |
| MRPS18A | -0.198423166 | 0.005400694 | 0.100338483 | Down | 2.267550456 |
| VEGFA | 0.191485667 | 0.105543576 | 0.321700573 | NS | 0.976568196 |
| MRPL14 | -0.274315065 | 0.0122438 | 0.134364821 | Down | 1.912083785 |
| TMEM63B | -0.118656585 | 0.555585266 | 0.721924582 | NS | 0.25524928 |
| CAPN11 | 0.472374589 | 0.131637591 | 0.354417312 | NS | 0.880620075 |
| MYMX | 0.856333983 | 0.180205147 | NA | NS | 0.74423281 |
| SLC29A1 | 0.586924266 | 0.002295603 | 0.073928862 | Up | 2.639103207 |
| HSP90AB1 | -0.02224625 | 0.748623571 | 0.852220749 | NS | 0.125736503 |
| SLC35B2 | -0.160776102 | 0.012873487 | 0.136134668 | Down | 1.890303805 |
| NFKBIE | -0.060985824 | 0.359630547 | 0.572558831 | NS | 0.444143426 |
| TMEM151B | 0.473415844 | 0.254727671 | NA | NS | 0.593923874 |
| TCTE1 | 0.636835053 | 0.079917307 | NA | NS | 1.097359162 |
| AARS2 | -0.159160863 | 0.011158435 | 0.129586317 | Down | 1.9523967 |
| SPATS1 | 1.123798903 | 0.012110433 | NA | Up | 1.916840329 |
| CDC5L | -0.134160246 | 0.025207746 | 0.179511132 | Down | 1.598465984 |
| SUPT3H | 0.053787592 | 0.436344055 | 0.632117879 | NS | 0.360170937 |
| RUNX2 | 0.177796545 | 0.029295304 | 0.190765154 | Up | 1.533201994 |
| CLIC5 | 0.116085195 | 0.246772736 | 0.474843656 | NS | 0.607702825 |
| ENPP4 | -0.209787247 | 0.032105031 | 0.198223945 | Down | 1.493426912 |
| ENPP5 | -0.055716639 | 0.620015715 | 0.767572799 | NS | 0.207597303 |
| RCAN2 | 0.241298029 | 0.228398749 | 0.457089362 | NS | 0.641306279 |
| CYP39A1 | 0.478187528 | 0.325704509 | NA | NS | 0.487176229 |
| SLC25A27 | 0.093219164 | 0.632465884 | 0.776078744 | NS | 0.198962896 |
| TDRD6 | -0.067949227 | 0.61150621 | 0.76220302 | NS | 0.213599128 |
| PLA2G7 | -0.308348274 | 0.024191605 | 0.176346063 | Down | 1.616335321 |
| ANKRD66 | 0.589298647 | 0.20557662 | NA | NS | 0.687026279 |
| MEP1A | 0.646113514 | 0.117481532 | NA | NS | 0.930030397 |
| ADGRF5 | 0.437607797 | 0.32560537 | 0.544069326 | NS | 0.487308442 |
| ADGRF1 | 0.45980972 | 0.279391388 | 0.503278325 | NS | 0.553786984 |
| TNFRSF21 | -0.006265692 | 0.970057745 | 0.983124 | NS | 0.013202413 |
| CD2AP | -0.209244693 | 0.022510432 | 0.171387603 | Down | 1.647616173 |
| ADGRF4 | 0.713788831 | 0.216892194 | NA | NS | 0.663756078 |
| OPN5 | 0.322642601 | 0.455091102 | 0.647239423 | NS | 0.341901656 |
| PTCHD4 | 0.324468569 | 0.468103593 | 0.657722861 | NS | 0.329658026 |
| MMUT | -0.043392032 | 0.453050824 | 0.645697466 | NS | 0.343853076 |
| CENPQ | -0.281443666 | 0.051457724 | 0.239579569 | NS | 1.28854943 |
| GLYATL3 | 0.842834999 | 0.043152705 | NA | Up | 1.364991974 |
| C6orf141 | 0.352948695 | 0.525803194 | NA | NS | 0.27917678 |
| RHAG | -0.01290917 | 0.944114432 | 0.969522418 | NS | 0.024975363 |
| CRISP2 | -0.051746316 | 0.868565342 | NA | NS | 0.061197504 |
| CRISP3 | 0.326612781 | 0.139046509 | 0.363272092 | NS | 0.856839909 |
| PGK2 | 0.361284907 | 0.585086509 | NA | NS | 0.232779915 |
| CRISP1 | 0.574012373 | 0.248811259 | NA | NS | 0.604129971 |
| DEFB114 | 1.044620934 | 0.200557112 | NA | NS | 0.697761934 |
| DEFB113 | 0.414186631 | 0.684756857 | NA | NS | 0.16446361 |
| DEFB110 | 0.430721968 | 0.58339046 | NA | NS | 0.234040677 |
| DEFB112 | 0.700543562 | 0.251658017 | NA | NS | 0.59918923 |
| TFAP2D | 0.430343907 | 0.367703313 | NA | NS | 0.434502457 |
| TFAP2B | 0.706647201 | 0.095252218 | 0.308858318 | NS | 1.021124902 |
| PKHD1 | 0.46005071 | 0.243313482 | 0.471076623 | NS | 0.613833825 |
| IL17A | 0.692003711 | 0.198568385 | NA | NS | 0.702089896 |
| IL17F | 0.355589931 | 0.573597381 | NA | NS | 0.241392841 |
| MCM3 | -0.173459215 | 0.025938109 | 0.182387032 | Down | 1.586061695 |
| PAQR8 | 0.071313087 | 0.272726633 | 0.498012997 | NS | 0.56427245 |
| EFHC1 | 0.111650704 | 0.153680622 | 0.380291042 | NS | 0.813380892 |
| TRAM2 | 0.051108179 | 0.525117571 | 0.700299459 | NS | 0.279743449 |
| TMEM14A | -0.10402666 | 0.330501918 | 0.54753725 | NS | 0.480826015 |
| GSTA2 | 0.083888621 | 0.893087167 | NA | NS | 0.049106151 |
| GSTA1 | 0.284159317 | 0.479897832 | NA | NS | 0.318851212 |
| GSTA5 | 0.72493749 | 0.210770068 | NA | NS | 0.676191064 |
| GSTA3 | 1.116191634 | 0.075944763 | NA | NS | 1.119502171 |
| GSTA4 | 0.093359361 | 0.549870915 | 0.717558046 | NS | 0.259739251 |
| CILK1 | 0.023438282 | 0.543603209 | 0.713193861 | NS | 0.264717988 |
| FBXO9 | -0.151570337 | 0.19937062 | 0.428932451 | NS | 0.700338841 |
| GCM1 | 0.192923639 | 0.235933349 | 0.463687081 | NS | 0.627210668 |
| ELOVL5 | 0.077078044 | 0.287265491 | 0.510431707 | NS | 0.541716542 |
| GCLC | 0.073528418 | 0.608485834 | 0.759927068 | NS | 0.215749528 |
| KLHL31 | 0.242234779 | 0.268750383 | 0.494410898 | NS | 0.570650909 |
| LRRC1 | -0.186031986 | 0.032711242 | 0.199704044 | Down | 1.485302972 |
| MLIP | 0.40373293 | 0.372452027 | 0.583200897 | NS | 0.428929658 |
| TINAG | 0.345561567 | 0.495007505 | NA | NS | 0.305388217 |
| FAM83B | 0.55220504 | 0.303401578 | NA | NS | 0.517982165 |
| HCRTR2 | 1.243082349 | 0.036817539 | NA | Up | 1.433945249 |
| GFRAL | 0.388057378 | 0.477121257 | NA | NS | 0.321371234 |
| HMGCLL1 | 0.754687815 | 0.106756305 | 0.323446607 | NS | 0.971606466 |
| BMP5 | 0.354860146 | 0.522133792 | NA | NS | 0.282218199 |
| COL21A1 | 0.616621169 | 0.168819023 | 0.396950027 | NS | 0.772578617 |
| DST | 0.003438928 | 0.968138327 | 0.98232378 | NS | 0.014062586 |
| BEND6 | 0.288586273 | 0.425879283 | 0.624143299 | NS | 0.370713486 |
| KIAA1586 | -0.405091632 | 0.001467662 | 0.062253356 | Down | 2.833374073 |
| ZNF451 | -0.146523346 | 0.054360108 | 0.246476974 | NS | 1.264719688 |
| BAG2 | -0.041500229 | 0.624631421 | 0.77109254 | NS | 0.204376173 |
| RAB23 | -0.431506522 | 0.006288764 | 0.106213327 | Down | 2.201434707 |
| PRIM2 | -0.039667213 | 0.429804784 | 0.627282309 | NS | 0.366728754 |
| KHDRBS2 | 0.020075181 | 0.874121216 | 0.929029107 | NS | 0.058428339 |
| FKBP1C | 0.0369245 | 0.893642266 | NA | NS | 0.048836299 |
| LGSN | 0.752745076 | 0.009913945 | 0.125229427 | Up | 2.003753492 |
| PTP4A1 | 0.488983637 | 0.008199332 | NA | Up | 2.086221513 |
| PHF3 | -0.007255374 | 0.883450711 | 0.934194671 | NS | 0.053817675 |
| EYS | 0.233100162 | 0.043939865 | 0.224627854 | Up | 1.357141285 |
| ADGRB3 | 0.308326918 | 0.280856618 | 0.504053569 | NS | 0.551515338 |
| LMBRD1 | 0.023554751 | 0.710628067 | 0.828951459 | NS | 0.148357644 |
| COL19A1 | 0.055251703 | 0.649244464 | 0.788471562 | NS | 0.187591745 |
| COL9A1 | 0.387337353 | 0.154190971 | 0.381142105 | NS | 0.811941058 |
| FAM135A | -0.08718036 | 0.11264348 | 0.331203909 | NS | 0.94829394 |
| SDHAF4 | -0.17306122 | 0.070397419 | 0.271192883 | NS | 1.152443264 |
| SMAP1 | 0.09073241 | 0.032256258 | 0.19828377 | NS | 1.491386021 |
| B3GAT2 | 0.155926925 | 0.019320606 | 0.161365225 | Up | 1.713979267 |
| OGFRL1 | -0.07294077 | 0.48467233 | 0.670571238 | NS | 0.314551774 |
| RIMS1 | 0.621516945 | 0.144419517 | 0.36967266 | NS | 0.840374111 |
| KCNQ5 | 0.20100129 | 0.069366699 | 0.26985496 | NS | 1.158848969 |
| KHDC1L | 0.345055771 | 0.625860926 | NA | NS | 0.203522162 |
| KHDC1 | 0.163958041 | 0.266169078 | 0.492079345 | NS | 0.5748424 |
| DPPA5 | 1.324461226 | 0.036781638 | NA | Up | 1.43436894 |
| KHDC3L | 1.042764771 | 0.09534034 | NA | NS | 1.020723305 |
| OOEP | 0.851807655 | 0.058721922 | NA | NS | 1.231199741 |
| DDX43 | 0.32144702 | 0.010966912 | 0.129200737 | Up | 1.959915653 |
| CGAS | -0.032956986 | 0.619537158 | 0.767330709 | NS | 0.207932641 |
| MTO1 | -0.143963528 | 0.001757801 | 0.065565416 | Down | 2.755030219 |
| EEF1A1 | -0.37253874 | 0.009191624 | 0.121258266 | Down | 2.036607736 |
| SLC17A5 | -0.093837464 | 0.047874869 | 0.23249157 | NS | 1.319892404 |
| CD109 | 0.236301053 | 0.092530003 | 0.304765864 | NS | 1.033717423 |
| COL12A1 | 0.619783223 | 0.129440901 | 0.351447009 | NS | 0.887928472 |
| COX7A2 | -0.130304572 | 0.352473185 | 0.566064118 | NS | 0.452873917 |
| TMEM30A | -0.05038972 | 0.551106553 | 0.718597261 | NS | 0.258764425 |
| FILIP1 | 0.216376873 | 0.05011984 | 0.236833467 | NS | 1.299990326 |
| SENP6 | -0.212524694 | 0.0010556 | 0.052878189 | Down | 2.976500722 |
| MYO6 | 0.215241636 | 0.110557071 | 0.329041981 | NS | 0.956413475 |
| IMPG1 | 0.340818107 | 0.142410507 | 0.367119025 | NS | 0.846457969 |
| HTR1B | 0.209636065 | 0.553340736 | NA | NS | 0.257007357 |
| MEI4 | 0.546459109 | 0.154558467 | NA | NS | 0.810907198 |
| IRAK1BP1 | -0.081145054 | 0.194335276 | 0.423758145 | NS | 0.711448358 |
| PHIP | 0.009705736 | 0.855715408 | 0.918797854 | NS | 0.067670648 |
| HMGN3 | -0.120602634 | 0.168702765 | 0.396950027 | NS | 0.7728778 |
| LCA5 | -0.029818544 | 0.889098465 | 0.93735026 | NS | 0.051050139 |
| SH3BGRL2 | 0.085288889 | 0.563516566 | 0.727694186 | NS | 0.249093313 |
| ELOVL4 | -0.110807013 | 0.358323696 | 0.571412376 | NS | 0.445724471 |
| TTK | -0.326623521 | 0.106578525 | 0.32311031 | NS | 0.972330294 |
| BCKDHB | -0.030497147 | 0.625799108 | 0.771788905 | NS | 0.20356506 |
| TENT5A | 0.036644525 | 0.608592555 | 0.759936681 | NS | 0.215673364 |
| IBTK | -0.183208923 | 0.009207852 | 0.121305834 | Down | 2.035841689 |
| UBE3D | -0.009674422 | 0.878676469 | 0.931684267 | NS | 0.056171004 |
| DOP1A | -0.053860977 | 0.244875353 | 0.472764177 | NS | 0.611054924 |
| PGM3 | 0.002391225 | 0.961855701 | 0.97882334 | NS | 0.016890077 |
| RWDD2A | -0.110127106 | 0.255901819 | 0.482914828 | NS | 0.591926628 |
| ME1 | 0.226422511 | 0.108195635 | 0.325834237 | NS | 0.965790261 |
| PRSS35 | -0.388695697 | 0.13040907 | 0.352362967 | NS | 0.884692204 |
| SNAP91 | 0.707407564 | 0.098287282 | 0.313029559 | NS | 1.007502672 |
| RIPPLY2 | 0.271698992 | 0.564646474 | NA | NS | 0.24822338 |
| CYB5R4 | 0.079313845 | 0.327473752 | 0.545440282 | NS | 0.484823504 |
| MRAP2 | 0.489496251 | 0.272251214 | NA | NS | 0.565030176 |
| CEP162 | -0.066188684 | 0.474844767 | 0.662941651 | NS | 0.323448344 |
| TBX18 | 0.577963751 | 0.209592158 | 0.439690301 | NS | 0.678624972 |
| NT5E | -0.104786978 | 0.365082178 | 0.577464745 | NS | 0.437609367 |
| SNX14 | 0.025755315 | 0.641700582 | 0.783130487 | NS | 0.192667567 |
| SYNCRIP | 0.047081601 | 0.425616434 | 0.623837924 | NS | 0.370981612 |
| HTR1E | 1.047186396 | 0.038088487 | NA | Up | 1.419206278 |
| CGA | 0.390586834 | 0.457549394 | NA | NS | 0.339562015 |
| ZNF292 | -0.040747814 | 0.520657748 | 0.696688569 | NS | 0.283447664 |
| GJB7 | 0.137702626 | 0.180157407 | 0.410020841 | NS | 0.744347877 |
| SMIM8 | 0.0839011 | 0.124501599 | 0.346117593 | NS | 0.90482507 |
| C6orf163 | 0.001619031 | 0.986285376 | 0.991796234 | NS | 0.005997406 |
| CFAP206 | 0.516751288 | 0.191880571 | NA | NS | 0.716968998 |
| SLC35A1 | -0.118320579 | 0.288656017 | 0.511830295 | NS | 0.539619386 |
| RARS2 | 0.035974486 | 0.346413877 | 0.561209256 | NS | 0.460404719 |
| ORC3 | -0.137360485 | 0.019261923 | 0.161175218 | Down | 1.715300347 |
| AKIRIN2 | 0.153200744 | 0.005580067 | 0.101343921 | Up | 2.253360621 |
| SPACA1 | 0.81573371 | 0.119609278 | NA | NS | 0.922235133 |
| CNR1 | 0.039712206 | 0.799826683 | 0.884702387 | NS | 0.097004112 |
| RNGTT | -0.078844251 | 0.118068862 | 0.338399285 | NS | 0.927864623 |
| PNRC1 | -0.049844503 | 0.641815596 | 0.783187382 | NS | 0.192589734 |
| SRSF12 | 0.145441327 | 0.157955336 | 0.385290564 | NS | 0.801465699 |
| PM20D2 | -0.050777677 | 0.378991143 | 0.588979839 | NS | 0.42137094 |
| GABRR1 | 0.257694915 | 0.54613282 | NA | NS | 0.262701724 |
| GABRR2 | 0.083852652 | 0.548305685 | 0.716413556 | NS | 0.260977251 |
| UBE2J1 | 0.152144413 | 0.121014487 | 0.34169323 | NS | 0.917162634 |
| RRAGD | -0.071248047 | 0.474514073 | 0.662722363 | NS | 0.323750903 |
| ANKRD6 | 0.058393622 | 0.364694561 | 0.577049473 | NS | 0.438070713 |
| LYRM2 | -0.281923798 | 0.002860002 | 0.080143153 | Down | 2.543633723 |
| MDN1 | 0.035033569 | 0.626965671 | 0.772682979 | NS | 0.202756238 |
| CASP8AP2 | -0.403901232 | 0.004776644 | 0.095708406 | Down | 2.320877096 |
| BACH2 | 0.155202969 | 0.164733054 | 0.392382726 | NS | 0.78321925 |
| MAP3K7 | -0.058164074 | 0.113567735 | 0.332228782 | NS | 0.944745036 |
| EPHA7 | 0.576804946 | 0.22577645 | 0.454410076 | NS | 0.64632136 |
| MANEA | -0.164710776 | 0.056614186 | 0.249939307 | NS | 1.247074729 |
| FUT9 | 0.553607736 | 0.212611427 | 0.442496084 | NS | 0.672413397 |
| UFL1 | -0.184752044 | 0.012678867 | 0.135687571 | Down | 1.89691957 |
| FHL5 | 0.207186623 | 0.701035695 | NA | NS | 0.154259868 |
| GPR63 | 0.259022146 | 0.073478374 | 0.275712399 | NS | 1.13384046 |
| NDUFAF4 | -0.199592375 | 0.094012251 | 0.30725936 | NS | 1.026815548 |
| KLHL32 | 0.023028177 | 0.799419402 | 0.88458729 | NS | 0.097225316 |
| MMS22L | 0.036403477 | 0.5312815 | 0.704804549 | NS | 0.274675307 |
| POU3F2 | 0.474222124 | 0.165807229 | NA | NS | 0.780396537 |
| FBXL4 | 0.041764515 | 0.58549038 | 0.744318662 | NS | 0.232480236 |
| FAXC | 0.604335933 | 0.147246724 | 0.372870906 | NS | 0.831954358 |
| COQ3 | -0.123927588 | 0.239156831 | 0.467454957 | NS | 0.621317211 |
| PNISR | -0.057334927 | 0.234108412 | 0.46228643 | NS | 0.630582981 |
| USP45 | -0.013667437 | 0.856708489 | 0.919321075 | NS | 0.067166929 |
| TSTD3 | 0.089150146 | 0.19026706 | 0.419771632 | NS | 0.720636393 |
| CCNC | -0.152413758 | 0.073339867 | 0.275499766 | NS | 1.134659879 |
| PRDM13 | 0.935079711 | 0.040233661 | NA | Up | 1.395410449 |
| MCHR2 | 0.551417773 | 0.280620928 | NA | NS | 0.551879943 |
| SIM1 | 0.376004769 | 0.354603457 | 0.567871364 | NS | 0.450257034 |
| ASCC3 | -0.085073283 | 0.077729867 | 0.282029373 | NS | 1.109412078 |
| GRIK2 | 0.399351392 | 0.319529076 | 0.53867169 | NS | 0.495489616 |
| HACE1 | 0.024718196 | 0.606596401 | 0.758788758 | NS | 0.217100171 |
| LIN28B | 0.472258967 | 0.305719591 | NA | NS | 0.51467673 |
| BVES | 0.561635499 | 0.171794415 | NA | NS | 0.764990958 |
| POPDC3 | 1.603117238 | 0.00648856 | NA | Up | 2.187851706 |
| PREP | 0.139395091 | 0.025974545 | 0.182525004 | Up | 1.585452056 |
| PRDM1 | 0.161646453 | 0.018709642 | 0.158993777 | Up | 1.727934514 |
| ATG5 | -0.166295869 | 0.04200827 | 0.220751111 | Down | 1.376665205 |
| CRYBG1 | -0.052905127 | 0.232735892 | 0.461082819 | NS | 0.633136636 |
| RTN4IP1 | 0.052149095 | 0.476598474 | 0.664460977 | NS | 0.321847353 |
| QRSL1 | -0.113643478 | 0.149314275 | 0.375279334 | NS | 0.825898669 |
| CD24 | 0.026948285 | 0.839941358 | 0.909691525 | NS | 0.075751034 |
| MTRES1 | -0.142632905 | 0.069902666 | 0.270515523 | NS | 1.155506259 |
| BEND3 | 0.372397162 | 0.008215949 | 0.116653231 | Up | 2.085342269 |
| PDSS2 | 0.077434776 | 0.20492184 | 0.43495148 | NS | 0.688411753 |
| SOBP | 0.352263437 | 0.094128053 | 0.307260785 | NS | 1.026280923 |
| SCML4 | 0.104641578 | 0.084515196 | 0.292470085 | NS | 1.073065196 |
| SEC63 | 0.112842902 | 0.034128842 | 0.202965138 | Up | 1.466878443 |
| OSTM1 | -0.155681462 | 0.059790257 | 0.25508876 | NS | 1.223369581 |
| NR2E1 | 0.83030356 | 0.050824116 | NA | NS | 1.293930168 |
| SNX3 | -0.162815149 | 0.277066833 | 0.501373626 | NS | 0.557415459 |
| AFG1L | -0.074187195 | 0.300733693 | 0.522222199 | NS | 0.521817912 |
| FOXO3 | -0.166530381 | 0.353712253 | 0.567005458 | NS | 0.451349895 |
| ARMC2 | 0.144673469 | 0.027146163 | 0.185717716 | Up | 1.566291551 |
| SESN1 | -0.070808779 | 0.218459856 | 0.447886776 | NS | 0.660628357 |
| CEP57L1 | -0.207644211 | 0.12302081 | 0.343806623 | NS | 0.910021418 |
| CD164 | -0.063442221 | 0.37007132 | 0.581306195 | NS | 0.43171457 |
| PPIL6 | 0.18592965 | 0.444923888 | 0.639039227 | NS | 0.351714277 |
| SMPD2 | 0.029443943 | 0.58003487 | 0.73951782 | NS | 0.236545897 |
| MICAL1 | 0.043952152 | 0.686043338 | 0.813364629 | NS | 0.163648449 |
| ZBTB24 | -0.111353646 | 0.017019676 | 0.153426713 | Down | 1.76904872 |
| AK9 | 0.039076154 | 0.52640182 | 0.701317887 | NS | 0.278682618 |
| FIG4 | 0.082341105 | 0.077646605 | 0.281861236 | NS | 1.109877531 |
| GPR6 | 1.509683719 | 0.014904416 | NA | Up | 1.82668504 |
| WASF1 | 0.212877507 | 0.161493792 | 0.389156531 | NS | 0.791844169 |
| CDC40 | -0.031817076 | 0.519037488 | 0.69601515 | NS | 0.284801274 |
| METTL24 | 0.064267417 | 0.544939173 | 0.713725092 | NS | 0.263651971 |
| DDO | -0.326419991 | 0.068235724 | 0.268476763 | NS | 1.165988195 |
| SLC22A16 | -0.391461297 | 0.014348009 | 0.143932587 | Down | 1.843208368 |
| CDK19 | 0.057773488 | 0.415227082 | 0.616572671 | NS | 0.381714329 |
| AMD1 | -0.144352417 | 0.018865775 | 0.159320106 | Down | 1.724325341 |
| GTF3C6 | -0.254430183 | 0.010214166 | 0.126233693 | Down | 1.990797095 |
| RPF2 | -0.146362034 | 0.124552477 | 0.346130003 | NS | 0.904647632 |
| SLC16A10 | -0.062249472 | 0.598369069 | 0.753124226 | NS | 0.223030865 |
| MFSD4B | 0.135592997 | 0.019365935 | 0.161396883 | Up | 1.712961539 |
| REV3L | 0.009947251 | 0.829849833 | 0.903289966 | NS | 0.081000489 |
| TRAF3IP2 | 0.245458433 | 0.014435035 | 0.144048441 | Up | 1.840582158 |
| FYN | -0.0791042 | 0.109795904 | 0.328012756 | NS | 0.959413863 |
| CCN6 | 0.536936312 | 0.007454306 | 0.112759922 | Up | 2.127592769 |
| TUBE1 | 0.004387415 | 0.949697754 | 0.972805169 | NS | 0.022414589 |
| FAM229B | -0.072529592 | 0.653033672 | 0.791227233 | NS | 0.185064425 |
| LAMA4 | 0.478493664 | 0.218175707 | 0.447660331 | NS | 0.661193609 |
| RFPL4B | 0.288013527 | 0.600968517 | NA | NS | 0.221148279 |
| MARCKS | 0.036385572 | 0.720521205 | 0.835048159 | NS | 0.142353233 |
| HDAC2 | 0.120362169 | 0.073850268 | 0.276013938 | NS | 1.131647922 |
| HS3ST5 | 0.492137323 | 0.253170382 | NA | NS | 0.596587104 |
| FRK | 0.360366081 | 0.31589289 | 0.535418875 | NS | 0.500460149 |
| NT5DC1 | 0.068939074 | 0.213585067 | 0.443180155 | NS | 0.670429114 |
| COL10A1 | 0.143231626 | 0.153562701 | 0.380233538 | NS | 0.813714257 |
| TSPYL4 | -0.016433584 | 0.771111183 | 0.866450216 | NS | 0.112882999 |
| DSE | -0.051746821 | 0.511092824 | 0.689903595 | NS | 0.291500217 |
| TSPYL1 | -0.265162069 | 2.41E-05 | 0.006002076 | Down | 4.617784111 |
| CALHM6 | 0.455333091 | 0.008405713 | 0.117652418 | Up | 2.07542545 |
| TRAPPC3L | -0.114620757 | 0.54416677 | 0.713285597 | NS | 0.264267982 |
| CALHM5 | 0.227139397 | 0.241413553 | 0.469262706 | NS | 0.617238351 |
| CALHM4 | 0.778440516 | 0.065688967 | NA | NS | 1.182507569 |
| RWDD1 | -0.1492035 | 0.187355444 | 0.416819945 | NS | 0.727333682 |
| RSPH4A | 0.175381748 | 0.247579373 | 0.475796157 | NS | 0.606285541 |
| ZUP1 | -0.138502363 | 0.122824378 | 0.343668163 | NS | 0.910715425 |
| KPNA5 | -0.231255376 | 0.011634566 | 0.132235645 | Down | 1.934249823 |
| FAM162B | 0.648991926 | 0.209089092 | NA | NS | 0.679668623 |
| GPRC6A | 0.332455897 | 0.527827155 | NA | NS | 0.27750827 |
| RFX6 | 0.833975545 | 0.110533317 | NA | NS | 0.956506798 |
| VGLL2 | 0.680748463 | 0.214829479 | NA | NS | 0.667906125 |
| ROS1 | 0.531580403 | 0.242919023 | 0.470591394 | NS | 0.614538475 |
| DCBLD1 | -0.102379755 | 0.288753012 | 0.511843768 | NS | 0.539473477 |
| GOPC | -0.179933724 | 0.005697447 | 0.102180893 | Down | 2.244319671 |
| NUS1 | -0.010281926 | 0.876065129 | 0.930423093 | NS | 0.057463606 |
| SLC35F1 | 0.417807356 | 0.141617066 | 0.365979921 | NS | 0.848884406 |
| CEP85L | -0.09446798 | 0.058026768 | 0.252025231 | NS | 1.236371617 |
| PLN | 0.131285253 | 0.189195255 | 0.418800923 | NS | 0.723089759 |
| MCM9 | -0.02709458 | 0.485570892 | 0.671376828 | NS | 0.313747355 |
| ASF1A | -0.209325621 | 0.007042798 | 0.110626998 | Down | 2.152254758 |
| FAM184A | 0.100596996 | 0.323846096 | 0.54255474 | NS | 0.489661334 |
| MAN1A1 | -0.03836154 | 0.673295713 | 0.805422743 | NS | 0.171794151 |
| TBC1D32 | -0.052473247 | 0.434666988 | 0.630833536 | NS | 0.361843342 |
| GJA1 | 0.739646086 | 0.151241344 | NA | NS | 0.820329471 |
| HSF2 | -0.15795517 | 0.035271041 | 0.205555536 | Down | 1.452581722 |
| SERINC1 | -0.130000451 | 0.101973221 | 0.317905513 | NS | 0.991513861 |
| PKIB | 0.010632136 | 0.961208191 | 0.978517825 | NS | 0.017182537 |
| FABP7 | 0.558940129 | 0.28471521 | NA | NS | 0.545589332 |
| SMPDL3A | -0.109939176 | 0.295335277 | 0.517305432 | NS | 0.529684674 |
| CLVS2 | 0.56869855 | 0.229117873 | 0.457716113 | NS | 0.639941032 |
| TRDN | 0.315071292 | 0.506753797 | 0.686848284 | NS | 0.295202988 |
| NKAIN2 | 0.38332252 | 0.228988811 | NA | NS | 0.640185738 |
| RNF217 | 0.146270356 | 0.077227288 | 0.281620438 | NS | 1.112229217 |
| TPD52L1 | 0.342044729 | 0.042073684 | 0.220751111 | Up | 1.375989463 |
| HDDC2 | 0.01807537 | 0.820895056 | 0.897981399 | NS | 0.08571236 |
| HEY2 | 0.555478289 | 0.104156616 | NA | NS | 0.982313138 |
| NCOA7 | -0.045333357 | 0.411819902 | 0.61414986 | NS | 0.385292669 |
| HINT3 | -0.11672343 | 0.072326858 | 0.27394225 | NS | 1.1407004 |
| TRMT11 | -0.017403179 | 0.760129696 | 0.860025919 | NS | 0.119112301 |
| CENPW | 0.119943269 | 0.511141368 | 0.689903595 | NS | 0.291458969 |
| RSPO3 | 0.86571094 | 0.019239614 | NA | Up | 1.715803648 |
| RNF146 | 0.027493006 | 0.656309615 | 0.793578522 | NS | 0.182891233 |
| ECHDC1 | -0.097342244 | 0.234029269 | 0.46228643 | NS | 0.630729824 |
| KIAA0408 | -0.079830504 | 0.660304359 | NA | NS | 0.180255836 |
| SOGA3 | 0.091478529 | 0.739507565 | NA | NS | 0.131057379 |
| C6orf58 | 0.38077809 | 0.193241791 | NA | NS | 0.713898947 |
| THEMIS | -0.140775209 | 0.107253587 | 0.324266883 | NS | 0.969588176 |
| PTPRK | -0.238738367 | 0.070353535 | 0.271115037 | NS | 1.152714074 |
| LAMA2 | 0.175253738 | 0.202672472 | 0.432824035 | NS | 0.693205236 |
| ARHGAP18 | -0.094818522 | 0.251012637 | 0.47786296 | NS | 0.600304414 |
| TMEM244 | 0.085855493 | 0.639333068 | 0.781025184 | NS | 0.194272832 |
| L3MBTL3 | -0.093339902 | 0.023812994 | 0.175023449 | NS | 1.623186003 |
| SAMD3 | -0.184183282 | 0.025724652 | 0.181829487 | Down | 1.589650493 |
| TMEM200A | 0.079939618 | 0.50801608 | 0.687745124 | NS | 0.294122541 |
| SMLR1 | 0.167202172 | 0.47240527 | NA | NS | 0.325685266 |
| EPB41L2 | 0.076846771 | 0.258449616 | 0.485085487 | NS | 0.587624108 |
| AKAP7 | -0.360582577 | 0.007568906 | 0.113104804 | Down | 2.120966905 |
| ARG1 | 0.019032533 | 0.916063788 | 0.953437582 | NS | 0.038074284 |
| MED23 | -0.03323762 | 0.42063089 | 0.620581631 | NS | 0.376098837 |
| ENPP3 | 0.05573044 | 0.758022581 | 0.858762731 | NS | 0.120317857 |
| OR2A4 | 0.074430563 | 0.865331245 | NA | NS | 0.062817614 |
| CTAGE9 | 0.341486214 | 0.260124975 | NA | NS | 0.584817948 |
| ENPP1 | 0.135748419 | 0.585259477 | 0.744149041 | NS | 0.232651545 |
| CCN2 | 0.473147095 | 0.147158615 | NA | NS | 0.832214309 |
| MOXD1 | 0.30052761 | 0.061848002 | 0.258380691 | NS | 1.208674324 |
| STX7 | 0.113843129 | 0.039694963 | 0.215680673 | Up | 1.401264602 |
| TAAR9 | 0.50887891 | 0.414130089 | NA | NS | 0.382863214 |
| TAAR8 | 0.702640716 | 0.288772844 | NA | NS | 0.53944365 |
| TAAR6 | 1.115779409 | 0.054048919 | NA | NS | 1.267212989 |
| TAAR5 | 0.628560574 | 0.3644397 | NA | NS | 0.43837432 |
| TAAR2 | 0.733336513 | 0.240848452 | NA | NS | 0.618256141 |
| TAAR1 | 0.300351073 | 0.424462429 | NA | NS | 0.372160745 |
| VNN1 | -0.010051314 | 0.953950787 | 0.975068327 | NS | 0.020474029 |
| VNN3 | 0.067256596 | 0.676525052 | 0.806944066 | NS | 0.169716117 |
| VNN2 | 0.152607985 | 0.372795089 | 0.583458834 | NS | 0.428529818 |
| SLC18B1 | -0.105646416 | 0.342859432 | 0.558038995 | NS | 0.464883899 |
| RPS12 | -0.415312233 | 0.006492152 | 0.1080547 | Down | 2.187611313 |
| EYA4 | 0.345841851 | 0.442725632 | 0.637211063 | NS | 0.353865333 |
| TCF21 | 0.655004998 | 0.197850275 | NA | NS | 0.703663342 |
| TBPL1 | -0.093735253 | 0.333125843 | 0.549934985 | NS | 0.477391675 |
| SLC2A12 | -0.013146663 | 0.89042016 | 0.938099296 | NS | 0.050405016 |
| SGK1 | -0.186290678 | 0.040660933 | 0.217573993 | Down | 1.39082266 |
| ALDH8A1 | 0.170424942 | 0.236351119 | 0.464109863 | NS | 0.626442337 |
| HBS1L | -0.090240202 | 0.083169361 | 0.290414939 | NS | 1.080036636 |
| MYB | 0.126775315 | 0.187297089 | 0.416818378 | NS | 0.727468973 |
| AHI1 | 0.147440213 | 0.152077168 | 0.378696455 | NS | 0.817935984 |
| PDE7B | 0.241852917 | 0.046305398 | 0.229294319 | Up | 1.334368378 |
| MTFR2 | 0.290410971 | 0.02202374 | 0.170171284 | Up | 1.657108936 |
| BCLAF1 | -0.112311112 | 0.025439193 | 0.180429975 | Down | 1.594496676 |
| MAP7 | -0.173211801 | 0.110492315 | 0.329041981 | NS | 0.956667928 |
| MAP3K5 | 0.001154997 | 0.984862654 | 0.990789568 | NS | 0.006624331 |
| PEX7 | 0.003914484 | 0.955323881 | 0.975854235 | NS | 0.019849366 |
| SLC35D3 | 0.322251474 | 0.219927739 | NA | NS | 0.657719991 |
| IL20RA | 0.648290795 | 0.129635535 | NA | NS | 0.887275935 |
| IL22RA2 | 0.230230729 | 0.655207045 | NA | NS | 0.183621442 |
| IFNGR1 | -0.055020956 | 0.634767474 | 0.77728181 | NS | 0.197385335 |
| OLIG3 | 0.893221948 | 0.106413416 | NA | NS | 0.973003616 |
| TNFAIP3 | 0.103969802 | 0.119513304 | 0.340053882 | NS | 0.922583745 |
| PERP | -0.113106378 | 0.348525138 | 0.56266197 | NS | 0.457765893 |
| ARFGEF3 | 0.625683911 | 0.102577519 | 0.3184085 | NS | 0.98894781 |
| PBOV1 | 0.2002613 | 0.703100678 | NA | NS | 0.152982483 |
| SMIM28 | 0.46241478 | 0.363137934 | NA | NS | 0.439928382 |
| HEBP2 | 0.062704756 | 0.414698155 | 0.616155258 | NS | 0.382267897 |
| NHSL1 | 0.304451375 | 0.062731658 | 0.2605514 | NS | 1.202513237 |
| CCDC28A | -0.013786458 | 0.842452031 | 0.91154854 | NS | 0.074454819 |
| ECT2L | 0.130310483 | 0.199015803 | 0.428784342 | NS | 0.701112436 |
| REPS1 | 0.023639176 | 0.585340832 | 0.744211167 | NS | 0.232591179 |
| ABRACL | -0.183318032 | 0.112909824 | 0.331563123 | NS | 0.947268269 |
| HECA | 8.96E-04 | 0.985629045 | 0.991386376 | NS | 0.006286507 |
| TXLNB | -0.037104418 | 0.682449996 | 0.810825369 | NS | 0.165929164 |
| CITED2 | -0.03157014 | 0.597325263 | 0.752238586 | NS | 0.223789117 |
| NMBR | 0.503839756 | 0.385277219 | NA | NS | 0.41422667 |
| GJE1 | 0.379652857 | 0.432934687 | NA | NS | 0.363577617 |
| VTA1 | -0.094185283 | 0.166318653 | 0.394295091 | NS | 0.779059041 |
| ADGRG6 | 0.022956791 | 0.905674423 | 0.947761281 | NS | 0.043027897 |
| HIVEP2 | 0.029230712 | 0.614760915 | 0.764346063 | NS | 0.211293752 |
| AIG1 | -0.009927499 | 0.894735885 | 0.941068205 | NS | 0.048305144 |
| ADAT2 | 0.110205384 | 0.122377704 | 0.343177027 | NS | 0.912297698 |
| PEX3 | -0.121542265 | 0.098812816 | 0.313653655 | NS | 1.005186722 |
| FUCA2 | 0.012097303 | 0.872112625 | 0.927774217 | NS | 0.059427426 |
| PHACTR2 | 0.070500326 | 0.197617971 | 0.427410856 | NS | 0.704173565 |
| LTV1 | -0.098059561 | 0.168770988 | 0.396950027 | NS | 0.772702207 |
| ZC2HC1B | 0.329262097 | 0.138494682 | NA | NS | 0.858566904 |
| PLAGL1 | 0.216300169 | 0.048130113 | 0.232990033 | Up | 1.317583119 |
| SF3B5 | -0.243245346 | 0.020629682 | 0.166711709 | Down | 1.68550746 |
| STX11 | 0.215672938 | 0.019576836 | 0.162444405 | Up | 1.708257488 |
| UTRN | -0.009598956 | 0.863083869 | 0.922755556 | NS | 0.063947 |
| EPM2A | 0.050709994 | 0.528580093 | 0.703278992 | NS | 0.276889197 |
| FBXO30 | -0.007466132 | 0.902098974 | 0.9454453 | NS | 0.044745811 |
| SHPRH | -0.049247222 | 0.349186396 | 0.563253052 | NS | 0.456942685 |
| GRM1 | 0.610115368 | 0.121437501 | 0.342148251 | NS | 0.915647179 |
| RAB32 | -0.08950471 | 0.454735007 | 0.647080104 | NS | 0.342241611 |
| ADGB | 0.597792671 | 0.057313244 | 0.251020828 | NS | 1.241745011 |
| STXBP5 | 0.086221851 | 0.361372689 | 0.573975818 | NS | 0.442044673 |
| SAMD5 | 0.475890589 | 0.310182173 | NA | NS | 0.508383166 |
| SASH1 | 0.139214506 | 0.310153675 | 0.530296194 | NS | 0.508423068 |
| UST | 0.034369638 | 0.621056893 | 0.768211988 | NS | 0.206868614 |
| TAB2 | -0.038921832 | 0.581337812 | 0.740560605 | NS | 0.235571428 |
| SUMO4 | -0.061831581 | 0.447555125 | 0.641420994 | NS | 0.349153465 |
| ZC3H12D | 0.102924319 | 0.19041536 | 0.420003137 | NS | 0.720298022 |
| PPIL4 | -0.200780489 | 0.001239811 | 0.057131098 | Down | 2.906644448 |
| GINM1 | -0.196891802 | 0.00433798 | 0.093367359 | Down | 2.362712502 |
| KATNA1 | -0.128334633 | 0.044105595 | 0.224627854 | Down | 1.355506319 |
| LATS1 | 0.176893571 | 0.003590579 | 0.086559417 | Up | 2.444835484 |
| NUP43 | -0.099745775 | 0.057888827 | 0.251883322 | NS | 1.237405251 |
| PCMT1 | -0.15078373 | 0.084806026 | 0.292945341 | NS | 1.071573286 |
| LRP11 | 0.349187324 | 0.180003755 | NA | NS | 0.744718435 |
| RAET1E | 0.479427515 | 0.265678071 | 0.491781978 | NS | 0.575644291 |
| RAET1G | 0.837620048 | 0.144862961 | NA | NS | 0.839042642 |
| ULBP2 | 0.518649341 | 0.157775037 | NA | NS | 0.801961709 |
| ULBP1 | 0.549863078 | 0.274139194 | NA | NS | 0.562028868 |
| RAET1L | 0.088639121 | 0.898493737 | NA | NS | 0.046484946 |
| ULBP3 | 0.192427307 | 0.274698493 | 0.499348937 | NS | 0.561143723 |
| PPP1R14C | 0.044294193 | 0.867561922 | NA | NS | 0.061699518 |
| IYD | 0.533175216 | 0.23477065 | 0.462791997 | NS | 0.629356198 |
| PLEKHG1 | -0.035246193 | 0.711771719 | 0.829736125 | NS | 0.147659272 |
| MTHFD1L | 0.09757602 | 0.366110972 | 0.578572555 | NS | 0.436387256 |
| AKAP12 | -0.166016378 | 0.384345964 | 0.593034804 | NS | 0.415277676 |
| ZBTB2 | -0.23204037 | 6.55E-04 | 0.04259149 | Down | 3.183870357 |
| RMND1 | 0.04633344 | 0.286326314 | 0.509474418 | NS | 0.543138738 |
| ARMT1 | -0.153249612 | 0.153186106 | 0.379723802 | NS | 0.814780623 |
| CCDC170 | 0.037570741 | 0.754895806 | 0.85656564 | NS | 0.122112987 |
| ESR1 | 0.273072348 | 0.010829241 | 0.12856202 | Up | 1.965401966 |
| SYNE1 | 0.08142536 | 0.394269595 | 0.600130417 | NS | 0.404206714 |
| MYCT1 | 0.07663136 | 0.566973805 | 0.730511059 | NS | 0.246437006 |
| VIP | 0.633316661 | 0.125987916 | NA | NS | 0.899671109 |
| FBXO5 | -0.13151854 | 0.157948925 | 0.385290564 | NS | 0.801483324 |
| MTRF1L | -0.044588921 | 0.393116161 | 0.599370636 | NS | 0.405479102 |
| RGS17 | 0.012292312 | 0.919243491 | 0.955282441 | NS | 0.036569437 |
| OPRM1 | 0.329208682 | 0.375882302 | 0.586177342 | NS | 0.424948122 |
| IPCEF1 | 0.03521529 | 0.513166697 | 0.691488803 | NS | 0.289741535 |
| CNKSR3 | 0.316185542 | 0.282299246 | 0.505292484 | NS | 0.549290282 |
| SCAF8 | -0.09237664 | 0.047510989 | 0.23161048 | NS | 1.323205925 |
| TIAM2 | 0.066463168 | 0.463520261 | 0.654149681 | NS | 0.333931277 |
| TFB1M | 0.024290435 | 0.648507581 | 0.787998723 | NS | 0.188084943 |
| CLDN20 | 0.250482255 | 0.044022055 | 0.224627854 | Up | 1.35632969 |
| NOX3 | 0.361554749 | 0.473579818 | NA | NS | 0.324606813 |
| ARID1B | 0.100970191 | 0.185979311 | 0.415622384 | NS | 0.730535365 |
| TMEM242 | -0.112031302 | 0.081386581 | 0.287719656 | NS | 1.089447197 |
| ZDHHC14 | 0.231947823 | 0.071460896 | 0.272521361 | NS | 1.145931543 |
| SNX9 | -0.089422181 | 0.160343556 | 0.388015316 | NS | 0.794948489 |
| SYNJ2 | -0.023192997 | 0.743106533 | 0.849359975 | NS | 0.12894892 |
| SERAC1 | 0.005648007 | 0.914017408 | 0.952098002 | NS | 0.039045533 |
| GTF2H5 | -0.105657218 | 0.327628904 | 0.54554 | NS | 0.484617791 |
| TULP4 | -0.119688693 | 0.113357411 | 0.332115589 | NS | 0.945550082 |
| TMEM181 | -0.063750578 | 0.179734767 | 0.409908267 | NS | 0.745367907 |
| DYNLT1 | 0.06003088 | 0.420596705 | 0.620581631 | NS | 0.376134134 |
| SYTL3 | -0.097987847 | 0.138897834 | 0.363222438 | NS | 0.857304527 |
| EZR | -0.259959145 | 0.003557469 | 0.086397822 | Down | 2.448858922 |
| RSPH3 | 0.114688689 | 0.164703228 | 0.392382726 | NS | 0.783297888 |
| TAGAP | 0.031282522 | 0.546996289 | 0.715437408 | NS | 0.26201562 |
| FNDC1 | 0.755837246 | 0.081812411 | 0.28835916 | NS | 1.087180811 |
| SOD2 | -0.017700094 | 0.882566824 | 0.93355998 | NS | 0.054252402 |
| WTAP | -0.070016078 | 0.248489497 | 0.476119605 | NS | 0.604691963 |
| ACAT2 | -0.165572107 | 0.062955807 | 0.261103565 | NS | 1.200964206 |
| TCP1 | -0.178400655 | 0.003404661 | 0.085194315 | Down | 2.467926183 |
| MRPL18 | -0.201814743 | 0.009908348 | 0.125229427 | Down | 2.003998728 |
| PNLDC1 | 0.440150028 | 0.048572161 | 0.233728157 | Up | 1.313612577 |
| MAS1 | 0.424947891 | 0.351367946 | 0.565179709 | NS | 0.454237861 |
| IGF2R | -0.110132703 | 0.37606199 | 0.586177342 | NS | 0.42474056 |
| SLC22A1 | 0.003457685 | 0.982972453 | 0.989888098 | NS | 0.007458653 |
| SLC22A2 | 0.335183254 | 0.441467322 | 0.63607956 | NS | 0.355101438 |
| SLC22A3 | 0.055932852 | 0.770067566 | 0.865925463 | NS | 0.113471168 |
| LPA | 0.246544181 | 0.537159784 | NA | NS | 0.269896509 |
| PLG | 0.424139607 | 0.357515247 | 0.570658921 | NS | 0.446705432 |
| MAP3K4 | -0.065404843 | 0.180942446 | 0.410901724 | NS | 0.742459544 |
| AGPAT4 | -0.077202008 | 0.34329452 | 0.558389993 | NS | 0.464333129 |
| PRKN | 0.192881292 | 0.110653909 | 0.329159088 | NS | 0.956033241 |
| PACRG | 0.603803121 | 0.034869382 | 0.204782731 | Up | 1.457555751 |
| QKI | -0.00957467 | 0.894472785 | 0.940966849 | NS | 0.048432869 |
| C6orf118 | 0.259300333 | 0.629616085 | NA | NS | 0.200924186 |
| PDE10A | 0.436472644 | 0.258240142 | 0.484970539 | NS | 0.587976249 |
| TBXT | 1.34053121 | 0.002926318 | NA | Up | 2.533678492 |
| PRR18 | 0.784088709 | 0.166267948 | NA | NS | 0.779191462 |
| SFT2D1 | 0.031163594 | 0.633524551 | 0.776709642 | NS | 0.19823655 |
| MPC1 | -0.223989749 | 0.005420905 | 0.100338483 | Down | 2.265928193 |
| RPS6KA2 | 0.251979032 | 0.011083402 | 0.129489019 | Up | 1.955326927 |
| RAMACL | -0.257811714 | 0.17073091 | 0.3990284 | NS | 0.767687846 |
| RNASET2 | 0.168399398 | 0.041182476 | 0.218561587 | Up | 1.385287541 |
| CEP43 | 0.104987429 | 0.097168984 | 0.311544739 | NS | 1.012472338 |
| CCR6 | -0.128419967 | 0.213498725 | 0.443180155 | NS | 0.670604715 |
| TCP10L2 | 0.158879972 | 0.660917689 | NA | NS | 0.179852624 |
| GPR31 | 0.362604182 | 0.179395986 | NA | NS | 0.746187278 |
| UNC93A | 0.58757497 | 0.252596212 | NA | NS | 0.597573166 |
| TTLL2 | 0.345624634 | 0.123445112 | 0.344227438 | NS | 0.908526103 |
| AFDN | 0.041215361 | 0.737081652 | 0.84513087 | NS | 0.1324844 |
| KIF25 | 0.702485025 | 0.110841428 | 0.329289192 | NS | 0.955297888 |
| FRMD1 | 0.658528563 | 0.156664353 | 0.383817609 | NS | 0.805029811 |
| DACT2 | 0.852289026 | 0.072725078 | NA | NS | 1.138315807 |
| SMOC2 | 0.485485266 | 0.242609622 | 0.470389904 | NS | 0.61509198 |
| THBS2 | 0.779085982 | 0.030690817 | 0.194491094 | Up | 1.512991544 |
| WDR27 | 0.07733438 | 0.447947634 | 0.641635461 | NS | 0.348772753 |
| C6orf120 | -0.093482065 | 0.142981304 | 0.367845183 | NS | 0.844720747 |
| PHF10 | -0.043880233 | 0.429775398 | 0.627282309 | NS | 0.366758448 |
| ERMARD | -0.120049785 | 0.085346144 | 0.293565942 | NS | 1.068816096 |
| DLL1 | -0.069098594 | 0.798050697 | NA | NS | 0.097969519 |
| FAM120B | 0.03965095 | 0.501047228 | 0.682709639 | NS | 0.300121337 |
| PSMB1 | -0.146059456 | 0.126867542 | 0.348807379 | NS | 0.896649475 |
| TBP | 0.042908192 | 0.525112745 | 0.700299459 | NS | 0.279747441 |
| PDCD2 | -0.107675612 | 0.104166037 | 0.320245132 | NS | 0.982273858 |
| FAM20C | -0.049005192 | 0.843049889 | 0.911847772 | NS | 0.074146724 |
| FOXL3 | 1.166119935 | 0.029119364 | NA | Up | 1.535818112 |
| PDGFA | 0.111535418 | 0.435235046 | 0.631110107 | NS | 0.361276141 |
| PRKAR1B | 0.152069934 | 0.19213625 | 0.42102173 | NS | 0.71639069 |
| DNAAF5 | 0.087171123 | 0.377592031 | 0.587593442 | NS | 0.42297718 |
| SUN1 | -0.048031602 | 0.371291225 | 0.58236492 | NS | 0.430285315 |
| GET4 | 0.112245105 | 0.529234085 | 0.70382202 | NS | 0.276352193 |
| ADAP1 | 0.053059858 | 0.6087279 | 0.760022153 | NS | 0.215576793 |
| COX19 | -0.079912953 | 0.099683527 | 0.314777508 | NS | 1.001376604 |
| CYP2W1 | 0.78169205 | 0.04213561 | 0.220751111 | Up | 1.375350713 |
| C7orf50 | -0.01545812 | 0.845245741 | 0.913061552 | NS | 0.073017009 |
| GPR146 | -0.096258317 | 0.658774245 | 0.794818657 | NS | 0.181263388 |
| GPER1 | 0.106035621 | 0.495137713 | 0.678069201 | NS | 0.305273994 |
| ZFAND2A | -0.073416651 | 0.382804716 | 0.59214603 | NS | 0.41702272 |
| UNCX | 0.845204512 | 0.17572822 | NA | NS | 0.75515849 |
| MICALL2 | 0.302992713 | 0.194417063 | 0.423843588 | NS | 0.711265622 |
| INTS1 | -0.074215464 | 0.585688946 | 0.744447122 | NS | 0.232332972 |
| MAFK | -0.116532723 | 0.27755344 | 0.501976536 | NS | 0.556653386 |
| TMEM184A | 0.614156426 | 0.012319735 | 0.134659948 | Up | 1.90939865 |
| PSMG3 | -0.257054287 | 7.43E-04 | 0.044925371 | Down | 3.129066646 |
| ELFN1 | 1.202424492 | 0.006764254 | NA | Up | 2.169780105 |
| MAD1L1 | -0.243408543 | 0.008365534 | 0.117538314 | Down | 2.077506348 |
| MRM2 | 0.189545768 | 0.358595427 | NA | NS | 0.445395253 |
| NUDT1 | -0.358760759 | 6.29E-05 | 0.011713712 | Down | 4.201273771 |
| SNX8 | -0.104104471 | 0.19135039 | 0.42070534 | NS | 0.718170649 |
| EIF3B | -0.283585181 | 0.007814058 | 0.114642894 | Down | 2.107123368 |
| CHST12 | 0.103706576 | 0.255968654 | 0.482962112 | NS | 0.591813215 |
| GRIFIN | 0.565309517 | 0.107523533 | NA | NS | 0.968496475 |
| LFNG | -0.023986897 | 0.727408301 | 0.838753198 | NS | 0.138221747 |
| BRAT1 | -0.058099021 | 0.462647648 | 0.6532805 | NS | 0.334749641 |
| IQCE | -0.109016769 | 0.195433451 | 0.425134582 | NS | 0.7090011 |
| TTYH3 | 0.309479203 | 0.113586209 | 0.332228782 | NS | 0.944674394 |
| AMZ1 | 0.273248244 | 0.043655467 | 0.224086881 | Up | 1.359961363 |
| GNA12 | -0.024020575 | 0.88469524 | 0.934906348 | NS | 0.053206309 |
| CARD11 | -0.072744264 | 0.419969772 | 0.62016266 | NS | 0.376781967 |
| SDK1 | 0.582816512 | 0.024822057 | 0.178586374 | Up | 1.60516224 |
| FOXK1 | -0.009529109 | 0.92994503 | 0.961777506 | NS | 0.031542722 |
| AP5Z1 | -0.022333463 | 0.789616344 | 0.878539316 | NS | 0.102583871 |
| RADIL | 1.02716244 | 0.002485317 | 0.076573431 | Up | 2.604618243 |
| PAPOLB | 1.056681558 | 0.027243998 | NA | Up | 1.564729158 |
| MMD2 | 0.548497724 | 0.32455738 | NA | NS | 0.488708511 |
| RBAK | -0.070808502 | 0.36679995 | 0.579141844 | NS | 0.435570733 |
| WIPI2 | -0.136298659 | 0.12003813 | 0.340700614 | NS | 0.920680778 |
| SLC29A4 | 0.695138257 | 0.00357968 | 0.086556724 | Up | 2.446155746 |
| TNRC18 | 0.123969688 | 0.495200873 | 0.678080097 | NS | 0.305218599 |
| FBXL18 | 0.301294264 | 0.060837664 | 0.256782931 | NS | 1.215827468 |
| ACTB | 0.129925005 | 0.159446829 | 0.387098757 | NS | 0.797384112 |
| FSCN1 | 0.679592497 | 8.32E-04 | 0.048073047 | Up | 3.08001518 |
| RNF216 | 0.005648316 | 0.899139416 | 0.943941825 | NS | 0.046172963 |
| OCM | -2.37E-04 | 0.998880715 | 0.999558051 | NS | 4.86E-04 |
| CCZ1 | -0.13787172 | 0.086362054 | 0.295291694 | NS | 1.063677036 |
| RSPH10B | 0.174044246 | 0.467689313 | NA | NS | 0.330042554 |
| PMS2 | -0.039820471 | 0.401599291 | 0.606070359 | NS | 0.396207063 |
| AIMP2 | -0.073541029 | 0.26766983 | 0.493645346 | NS | 0.572400577 |
| EIF2AK1 | -0.027962499 | 0.880417467 | 0.932334579 | NS | 0.05531135 |
| ANKRD61 | 0.153752354 | 0.106049454 | 0.322346872 | NS | 0.974491565 |
| USP42 | 0.147765918 | 0.026424884 | 0.183277617 | Up | 1.577986913 |
| CYTH3 | -0.096230024 | 0.108542455 | 0.326591248 | NS | 0.96440036 |
| FAM220A | -0.095299482 | 0.247739559 | 0.475860905 | NS | 0.606004639 |
| RAC1 | 0.060595338 | 0.381431598 | 0.591038759 | NS | 0.418583333 |
| DAGLB | -0.090240409 | 0.219954892 | 0.449237703 | NS | 0.657666375 |
| KDELR2 | -0.033028348 | 0.526842636 | 0.701782705 | NS | 0.278319086 |
| GRID2IP | 0.40999728 | 0.008725846 | 0.118782974 | Up | 2.059192473 |
| ZDHHC4 | -0.13373988 | 0.039396652 | 0.214800721 | Down | 1.404540682 |
| C7orf26 | -0.065946937 | 0.152612627 | 0.379261154 | NS | 0.816409533 |
| ZNF853 | 0.011816874 | 0.884933095 | 0.93507141 | NS | 0.053089563 |
| ZNF316 | -0.127774591 | 0.185183807 | 0.415384315 | NS | 0.732396993 |
| ZNF12 | -0.112722115 | 0.064475009 | 0.263488519 | NS | 1.19060859 |
| RSPH10B2 | 0.345913264 | 0.239345665 | NA | NS | 0.620974434 |
| CCZ1B | -0.128282094 | 0.109002554 | 0.327029744 | NS | 0.962563327 |
| C1GALT1 | -0.02341473 | 0.745416285 | 0.850639911 | NS | 0.127601123 |
| COL28A1 | 0.365272869 | 0.206422099 | 0.436355477 | NS | 0.68524381 |
| MIOS | -0.158410111 | 0.003427556 | 0.085194315 | Down | 2.46501542 |
| RPA3 | -0.038400994 | 0.644143786 | 0.784773964 | NS | 0.191017179 |
| UMAD1 | 0.036674343 | 0.372892007 | 0.58357064 | NS | 0.428416925 |
| GLCCI1 | 0.096764492 | 0.080139943 | 0.28565992 | NS | 1.096150969 |
| ICA1 | 0.116617786 | 0.255585468 | 0.482477611 | NS | 0.592463843 |
| NXPH1 | 0.804695746 | 0.150873128 | NA | NS | 0.821388105 |
| NDUFA4 | -0.276143969 | 0.084247065 | 0.292034469 | NS | 1.074445221 |
| PHF14 | -0.075461335 | 0.155266139 | 0.382558975 | NS | 0.808923248 |
| THSD7A | 0.219905451 | 0.411780838 | 0.614131594 | NS | 0.385333867 |
| TMEM106B | -0.085198457 | 0.209805468 | 0.439792418 | NS | 0.678183197 |
| VWDE | 0.279835142 | 0.080894119 | 0.286854583 | NS | 1.092083049 |
| SCIN | 0.374073744 | 0.157945117 | 0.385290564 | NS | 0.801493797 |
| ARL4A | -0.049516211 | 0.705267228 | 0.825472993 | NS | 0.151646296 |
| ETV1 | 0.391939573 | 0.265498491 | 0.491622597 | NS | 0.575937943 |
| DGKB | 0.553229621 | 0.219643913 | 0.448972233 | NS | 0.658280827 |
| AGMO | 0.413413236 | 0.039360011 | 0.214726771 | Up | 1.404944783 |
| MEOX2 | 0.614309097 | 0.269617128 | NA | NS | 0.569252521 |
| CRPPA | -0.269084236 | 0.080957265 | 0.286965375 | NS | 1.091744171 |
| SOSTDC1 | 0.537091255 | 0.169360268 | NA | NS | 0.771188468 |
| LRRC72 | 0.358542602 | 0.445797687 | NA | NS | 0.350862189 |
| ANKMY2 | -0.154914112 | 0.022144264 | 0.170412614 | Down | 1.654738746 |
| BZW2 | -0.240435336 | 9.46E-04 | 0.051236698 | Down | 3.023951909 |
| TSPAN13 | -0.179650028 | 0.182190032 | 0.412275698 | NS | 0.739475388 |
| AGR2 | 0.317844407 | 0.121615704 | 0.34233565 | NS | 0.915010342 |
| AGR3 | 1.071735209 | 0.058609276 | NA | NS | 1.232033647 |
| AHR | -0.08413171 | 0.337567007 | 0.553636868 | NS | 0.471640006 |
| SNX13 | -0.004489851 | 0.932243607 | 0.962763487 | NS | 0.030470586 |
| PRPS1L1 | 1.008117705 | 0.085651712 | NA | NS | 1.067263951 |
| HDAC9 | -0.093246104 | 0.177621319 | 0.407521884 | NS | 0.750504909 |
| TWIST1 | 0.614687959 | 0.274420668 | NA | NS | 0.561583183 |
| POLR1F | -0.148270043 | 0.096782019 | 0.310921075 | NS | 1.01420532 |
| TMEM196 | 0.280141192 | 0.589557003 | NA | NS | 0.229474197 |
| MACC1 | 0.024408858 | 0.829158777 | 0.902881053 | NS | 0.081362298 |
| ITGB8 | -0.089015468 | 0.614560751 | 0.764203112 | NS | 0.21143518 |
| ABCB5 | -0.01262824 | 0.967877674 | 0.982114953 | NS | 0.014179528 |
| SP8 | 1.239894064 | 0.017305916 | NA | Up | 1.761805399 |
| SP4 | -0.063580872 | 0.27288788 | 0.498220521 | NS | 0.564015753 |
| DNAH11 | 0.296345229 | 0.074233218 | 0.276707218 | NS | 1.129401715 |
| CDCA7L | -0.076909812 | 0.455279518 | 0.64744052 | NS | 0.341721887 |
| RAPGEF5 | 0.083011081 | 0.579619709 | 0.739260992 | NS | 0.236856856 |
| STEAP1B | 0.096983587 | 0.542284993 | 0.712367266 | NS | 0.265772414 |
| IL6 | 0.293768254 | 0.20657418 | NA | NS | 0.684923962 |
| TOMM7 | -0.436690378 | 0.039793027 | 0.215730166 | Down | 1.400193026 |
| FAM126A | -0.058745295 | 0.281497571 | 0.504639547 | NS | 0.550525348 |
| KLHL7 | -0.024671899 | 0.655755265 | 0.793223847 | NS | 0.183258213 |
| NUP42 | -0.064811219 | 0.32183747 | 0.540296272 | NS | 0.492363395 |
| GPNMB | 0.229333149 | 0.359584681 | 0.572525609 | NS | 0.444198818 |
| MALSU1 | -0.053163259 | 0.335424454 | 0.551701439 | NS | 0.474405279 |
| IGF2BP3 | 0.147181384 | 0.159597081 | 0.387107395 | NS | 0.796975057 |
| TRA2A | -0.104751413 | 0.009249576 | 0.121396148 | Down | 2.033878164 |
| CCDC126 | -0.195474397 | 0.064963185 | 0.264064926 | NS | 1.187332691 |
| FAM221A | 0.179366161 | 0.024605055 | 0.177705761 | Up | 1.60897566 |
| STK31 | 0.275779794 | 0.182768931 | 0.412961135 | NS | 0.738097628 |
| NPY | 0.680069597 | 0.22839913 | NA | NS | 0.641305555 |
| PALS2 | -0.397744709 | 5.98E-04 | 0.040636454 | Down | 3.223327707 |
| GSDME | 0.175978041 | 0.106495815 | 0.322999226 | NS | 0.972667459 |
| OSBPL3 | 0.012597576 | 0.810965422 | 0.891548103 | NS | 0.090997663 |
| CYCS | -0.23088956 | 0.014504007 | 0.144329895 | Down | 1.838512006 |
| C7orf31 | 0.194040161 | 0.068575563 | 0.268602708 | NS | 1.163830616 |
| NPVF | 0.542998624 | 0.37047127 | NA | NS | 0.431245466 |
| NFE2L3 | 0.058801514 | 0.393697386 | 0.599977212 | NS | 0.404837468 |
| HNRNPA2B1 | 0.059786133 | 0.210748192 | 0.44059935 | NS | 0.676236143 |
| CBX3 | -0.193011098 | 0.065308639 | 0.264481527 | NS | 1.185029365 |
| SNX10 | 8.80E-04 | 0.993839703 | 0.996319408 | NS | 0.002683658 |
| SKAP2 | 0.005230329 | 0.956072055 | 0.976096862 | NS | 0.019509376 |
| HOXA1 | -0.01407475 | 0.938853765 | 0.966321915 | NS | 0.027402048 |
| HOXA2 | -0.009616633 | 0.941171929 | 0.96761205 | NS | 0.026331034 |
| HOXA3 | 0.5014185 | 0.025102852 | 0.179152549 | Up | 1.600276936 |
| HOXA4 | 0.515152156 | 0.268615622 | NA | NS | 0.570868734 |
| HOXA5 | 0.716844862 | 0.021955776 | NA | Up | 1.658451203 |
| HOXA6 | 1.255057979 | 0.030217925 | NA | Up | 1.519735356 |
| HOXA7 | 1.323828791 | 0.041310777 | NA | Up | 1.383936634 |
| HOXA9 | 0.103508064 | 0.667176223 | NA | NS | 0.175759439 |
| HOXA10 | 0.607790541 | 0.025999003 | NA | Up | 1.5850433 |
| HOXA11 | 0.406126154 | 0.491562639 | NA | NS | 0.308421133 |
| HOXA13 | 0.113364352 | 0.818298866 | NA | NS | 0.087088051 |
| EVX1 | 0.974348412 | 0.045782538 | NA | Up | 1.339300138 |
| HIBADH | -0.203680305 | 0.016833784 | 0.152894601 | Down | 1.773818256 |
| TAX1BP1 | -0.262537165 | 0.016895671 | 0.153016426 | Down | 1.772224561 |
| JAZF1 | -0.178423839 | 0.244689777 | 0.472582667 | NS | 0.611384175 |
| CREB5 | 0.260373903 | 0.077645101 | 0.281861236 | NS | 1.109885942 |
| TRIL | 0.864426368 | 0.032751339 | NA | Up | 1.484770943 |
| CPVL | -0.329865733 | 0.012028616 | 0.13403215 | Down | 1.919784331 |
| CHN2 | 0.092756864 | 0.457289171 | 0.648874634 | NS | 0.339809083 |
| PRR15 | 0.988378308 | 0.031879763 | NA | Up | 1.496484918 |
| WIPF3 | 0.441410192 | 0.103151141 | 0.319144166 | NS | 0.986525965 |
| SCRN1 | -0.149381537 | 0.073093849 | 0.275056743 | NS | 1.136119167 |
| FKBP14 | 0.020311281 | 0.770772803 | 0.86621231 | NS | 0.113073618 |
| PLEKHA8 | 0.0427769 | 0.346931617 | 0.561433348 | NS | 0.45975612 |
| MTURN | -0.031708068 | 0.726938867 | 0.838611255 | NS | 0.13850211 |
| ZNRF2 | 0.010421887 | 0.852710865 | 0.916973482 | NS | 0.069198203 |
| NOD1 | 0.149603326 | 0.011998651 | 0.134014426 | Up | 1.92086759 |
| GGCT | -0.180965867 | 0.090560983 | 0.301369896 | NS | 1.043058874 |
| GARS1 | 0.073013756 | 0.082298601 | 0.289124491 | NS | 1.084607549 |
| CRHR2 | 0.349455716 | 0.19899436 | 0.428784342 | NS | 0.701159233 |
| INMT | 0.745447196 | 0.014134716 | NA | Up | 1.849712921 |
| INMT-MINDY4 | 0.734248391 | 0.445099427 | NA | NS | 0.351542965 |
| MINDY4 | 0.112053495 | 0.698613981 | NA | NS | 0.155762727 |
| AQP1 | -0.266915278 | 0.211542828 | 0.441234413 | NS | 0.674601693 |
| GHRHR | 0.871502517 | 0.088895525 | NA | NS | 1.051120102 |
| ADCYAP1R1 | 0.506188781 | 0.247383589 | 0.475579532 | NS | 0.606629114 |
| NEUROD6 | 0.715967717 | 0.126838701 | NA | NS | 0.896748216 |
| ITPRID1 | 0.511167402 | 0.242120035 | 0.469918053 | NS | 0.615969273 |
| PPP1R17 | -0.190474522 | 0.39922043 | 0.604289507 | NS | 0.398787242 |
| PDE1C | 0.408394664 | 0.306198936 | 0.527340053 | NS | 0.513996323 |
| LSM5 | -0.163571543 | 0.21537845 | 0.444698636 | NS | 0.666797752 |
| AVL9 | 0.024233523 | 0.521883376 | 0.697642194 | NS | 0.282426537 |
| KBTBD2 | 0.015414412 | 0.805423049 | 0.888012645 | NS | 0.093975946 |
| FKBP9 | 0.182277104 | 0.13968521 | 0.363934068 | NS | 0.854849576 |
| NT5C3A | -0.239361176 | 0.040979338 | 0.218105862 | Down | 1.387435062 |
| RP9 | 0.048785629 | 0.489258827 | 0.673863703 | NS | 0.310461331 |
| BBS9 | 0.03082662 | 0.590497569 | 0.7478297 | NS | 0.228781886 |
| BMPER | 0.544197353 | 0.136275323 | 0.360681549 | NS | 0.86558278 |
| NPSR1 | 0.180098627 | 0.713530285 | NA | NS | 0.146587589 |
| DPY19L1 | -0.072305964 | 0.27102972 | 0.496290769 | NS | 0.566983084 |
| TBX20 | 0.93311662 | 0.004223788 | NA | Up | 2.374297924 |
| HERPUD2 | -0.067446682 | 0.083454934 | 0.290969351 | NS | 1.078547983 |
| SEPTIN7 | -0.10817432 | 0.07734102 | 0.281652078 | NS | 1.111590103 |
| EEPD1 | 0.021807381 | 0.822745989 | 0.899052571 | NS | 0.084734226 |
| KIAA0895 | 0.383330175 | 0.006558147 | 0.108312793 | Up | 2.183218839 |
| ANLN | -0.055571685 | 0.755422179 | 0.85689345 | NS | 0.121810268 |
| AOAH | -0.060753908 | 0.405958757 | 0.609560706 | NS | 0.391518085 |
| ELMO1 | 0.098391487 | 0.170018647 | 0.398309642 | NS | 0.769503444 |
| GPR141 | 0.379214629 | 0.002220837 | 0.072832159 | Up | 2.653483234 |
| EPDR1 | -0.163222551 | 0.287555076 | 0.510589731 | NS | 0.541278961 |
| NME8 | 0.105027357 | 0.353518498 | 0.566966431 | NS | 0.451587857 |
| SFRP4 | 0.319601998 | 0.155288274 | 0.382558975 | NS | 0.808861337 |
| STARD3NL | -0.026551153 | 0.74165355 | 0.848546216 | NS | 0.12979892 |
| AMPH | 0.093333792 | 0.433875321 | 0.630295756 | NS | 0.362635052 |
| VPS41 | -0.13735818 | 0.007933619 | 0.11472857 | Down | 2.10052868 |
| POU6F2 | 0.683201015 | 0.092794559 | 0.305242354 | NS | 1.032477487 |
| YAE1 | -0.113452869 | 0.185376397 | 0.415463304 | NS | 0.731945563 |
| RALA | 0.164683545 | 0.021003008 | 0.167842555 | Up | 1.677718492 |
| CDK13 | 0.032210434 | 0.270170782 | 0.495670211 | NS | 0.568361621 |
| MPLKIP | 0.113682255 | 0.057070546 | 0.250544431 | NS | 1.243587971 |
| SUGCT | 0.278648689 | 0.080277732 | 0.285933942 | NS | 1.095404905 |
| INHBA | 0.683183867 | 0.006646056 | 0.108543277 | Up | 2.177435982 |
| GLI3 | 0.609596847 | 0.075052906 | 0.27811629 | NS | 1.124632485 |
| C7orf25 | 0.225774254 | 0.128604588 | 0.350380951 | NS | 0.890743537 |
| PSMA2 | 0.108243163 | 0.081970488 | 0.28847931 | NS | 1.086342477 |
| MRPL32 | -0.262982578 | 0.008201399 | 0.116591208 | Down | 2.086112068 |
| HECW1 | 0.420978532 | 0.295436107 | 0.5174029 | NS | 0.529536428 |
| STK17A | -0.100569475 | 0.042147272 | 0.220751111 | Down | 1.375230531 |
| COA1 | 0.091355766 | 0.129040499 | 0.351109125 | NS | 0.889273966 |
| BLVRA | -0.112122774 | 0.312196507 | 0.53190927 | NS | 0.50557196 |
| MRPS24 | 0.121570725 | 0.461647761 | 0.65263315 | NS | 0.335689266 |
| URGCP | -0.143574026 | 0.023254637 | 0.173340472 | Down | 1.63349043 |
| UBE2D4 | 0.081199284 | 0.277587385 | 0.501982616 | NS | 0.556600275 |
| SPDYE1 | 0.230801592 | 0.087628857 | 0.297226909 | NS | 1.057352852 |
| DBNL | -0.051320182 | 0.286029704 | 0.509186875 | NS | 0.543588864 |
| POLM | 0.110543251 | 0.215319541 | 0.444697279 | NS | 0.666916555 |
| AEBP1 | 0.227856936 | 0.305627074 | 0.526741519 | NS | 0.514808177 |
| POLD2 | -0.036178331 | 0.624307557 | 0.770858943 | NS | 0.204601408 |
| MYL7 | 0.594756363 | 0.283056595 | NA | NS | 0.548126722 |
| GCK | 0.486752538 | 0.234930932 | 0.462908732 | NS | 0.629059799 |
| YKT6 | -0.024071527 | 0.601505993 | 0.755328997 | NS | 0.220760041 |
| CAMK2B | 0.704485254 | 0.068914107 | 0.268916491 | NS | 1.161691865 |
| NUDCD3 | -0.147988251 | 0.007094417 | 0.110917973 | Down | 2.149083294 |
| NPC1L1 | 0.812665392 | 0.057244255 | 0.250910606 | NS | 1.242268096 |
| DDX56 | -0.083608209 | 0.027370578 | 0.186060433 | NS | 1.562716029 |
| TMED4 | -0.104708286 | 0.01868392 | 0.158925385 | Down | 1.728531999 |
| OGDH | 0.024342283 | 0.789746889 | 0.878567629 | NS | 0.102512076 |
| ZMIZ2 | -0.049626403 | 0.745505761 | 0.850639911 | NS | 0.127548996 |
| PPIA | -0.185646363 | 0.095893771 | 0.309754462 | NS | 1.018209604 |
| H2AZ2 | -0.172623663 | 1.57E-04 | 0.020296551 | Down | 3.804477894 |
| PURB | 0.032094851 | 0.605031511 | 0.757720329 | NS | 0.218222006 |
| MYO1G | -0.076702084 | 0.234482988 | 0.462583289 | NS | 0.62988866 |
| CCM2 | -0.055760991 | 0.257069903 | 0.48410524 | NS | 0.589948767 |
| NACAD | 0.017403784 | 0.959732952 | NA | NS | 0.017849594 |
| TBRG4 | -0.014418655 | 0.809600618 | 0.89056068 | NS | 0.091729169 |
| RAMP3 | 0.149493373 | 0.361367969 | 0.573975818 | NS | 0.442050346 |
| ADCY1 | 0.321289559 | 0.111028649 | 0.32960107 | NS | 0.954564945 |
| CCDC201 | 0.521886914 | 0.330631397 | NA | NS | 0.480655908 |
| IGFBP1 | 1.012952819 | 0.036835278 | NA | Up | 1.433736054 |
| IGFBP3 | -0.039465958 | 0.773389717 | 0.867925187 | NS | 0.111601607 |
| TNS3 | 0.01862369 | 0.855708091 | 0.918797854 | NS | 0.067674361 |
| PKD1L1 | 0.389035015 | 0.048114254 | 0.232990033 | Up | 1.317726247 |
| HUS1 | 0.040263346 | 0.347647996 | 0.562229849 | NS | 0.45886027 |
| SUN3 | 0.677847864 | 0.032180513 | NA | Up | 1.492407036 |
| C7orf57 | 0.48609577 | 0.025780333 | 0.181999045 | Up | 1.588711481 |
| UPP1 | 0.213974983 | 0.001483373 | 0.062253356 | Up | 2.828749565 |
| ABCA13 | 0.48613862 | 0.026839878 | 0.185036235 | Up | 1.571219468 |
| CDC14C | 0.71395924 | 0.177188108 | NA | NS | 0.751565429 |
| VWC2 | 0.565061708 | 0.200027122 | 0.429790861 | NS | 0.698911114 |
| ZPBP | 0.180077058 | 0.6708395 | NA | NS | 0.173381374 |
| SPATA48 | 0.452218957 | 0.360064669 | NA | NS | 0.443619492 |
| IKZF1 | 0.138375382 | 0.002740867 | 0.079323886 | Up | 2.562112074 |
| FIGNL1 | -0.110019673 | 0.127323965 | 0.348830209 | NS | 0.895089845 |
| DDC | 0.693056675 | 0.132296567 | 0.355324965 | NS | 0.878451425 |
| GRB10 | 0.088360457 | 0.502884603 | 0.684073603 | NS | 0.298531661 |
| COBL | 0.495840458 | 0.191286726 | 0.42070534 | NS | 0.718315167 |
| POM121L12 | 1.08153487 | 0.02352696 | NA | Up | 1.628434178 |
| VSTM2A | 0.43568566 | 0.378460798 | 0.588455293 | NS | 0.421979099 |
| SEC61G | -0.086669785 | 0.407617868 | 0.611075752 | NS | 0.389746787 |
| EGFR | 0.557618625 | 0.181872514 | 0.412022664 | NS | 0.74023293 |
| LANCL2 | -0.034921241 | 0.537889585 | 0.709095159 | NS | 0.269306865 |
| VOPP1 | 0.117093745 | 0.005539472 | 0.101053331 | Up | 2.25653166 |
| SEPTIN14 | 0.479437117 | 0.310834045 | NA | NS | 0.507471419 |
| ZNF713 | 0.169770899 | 0.22283564 | 0.451584405 | NS | 0.652015348 |
| NIPSNAP2 | -0.114238991 | 0.137979938 | 0.362607036 | NS | 0.860184056 |
| MRPS17 | -0.181814403 | 0.082006712 | 0.288503668 | NS | 1.0861506 |
| PSPH | 0.285425758 | 0.010697 | 0.128196078 | Up | 1.970737985 |
| CCT6A | -0.244089248 | 0.002717153 | 0.078993761 | Down | 2.565885881 |
| SUMF2 | -0.324795939 | 4.84E-05 | 0.009896845 | Down | 4.31515863 |
| PHKG1 | 0.123415784 | 0.326158242 | 0.544532529 | NS | 0.486571642 |
| CHCHD2 | -0.146936235 | 0.186804551 | 0.416322648 | NS | 0.728612548 |
| NUPR2 | -0.010041037 | 0.985136248 | NA | NS | 0.006503701 |
| ZNF479 | 0.06023987 | 0.906839859 | NA | NS | 0.042469399 |
| ZNF716 | 0.612379996 | 0.250763192 | NA | NS | 0.600736211 |
| ZNF722P | 0.135108924 | 0.809886752 | NA | NS | 0.091575705 |
| ZNF727 | -0.244294219 | 0.161879748 | 0.38963426 | NS | 0.790807481 |
| ZNF735 | 0.959099557 | 0.157947537 | NA | NS | 0.801487141 |
| ZNF679 | 1.087288057 | 0.039802948 | NA | Up | 1.400084763 |
| ZNF736 | 0.001850132 | 0.974335298 | 0.985403414 | NS | 0.011291564 |
| ZNF680 | -0.243434378 | 0.006611761 | 0.108462559 | Down | 2.179682865 |
| ZNF107 | -0.135788656 | 0.088847303 | 0.299209951 | NS | 1.051355753 |
| ZNF138 | -0.112469492 | 0.133899807 | 0.35729133 | NS | 0.873220048 |
| ZNF273 | -0.177465683 | 0.032050372 | 0.198142899 | Down | 1.494166928 |
| ZNF117 | 0.158986087 | 0.206037166 | 0.43596473 | NS | 0.686054433 |
| ERV3-1 | 0.214004741 | 0.034650758 | 0.204304637 | Up | 1.460287259 |
| ZNF92 | -0.345006678 | 0.009392597 | 0.122151764 | Down | 2.027214311 |
| VKORC1L1 | -0.020834592 | 0.69279127 | 0.817597942 | NS | 0.159397594 |
| GUSB | -0.105059949 | 0.077381084 | 0.281652078 | NS | 1.111365188 |
| ASL | -0.144731586 | 0.054812445 | 0.247094287 | NS | 1.261120829 |
| CRCP | -0.198791436 | 7.27E-05 | 0.013008525 | Down | 4.138435378 |
| TPST1 | -0.127022784 | 0.385354978 | 0.593556772 | NS | 0.414139026 |
| KCTD7 | 0.033311326 | 0.569220831 | 0.731710119 | NS | 0.244719215 |
| RABGEF1 | 0.048497404 | 0.555944851 | 0.72202841 | NS | 0.254968287 |
| TMEM248 | -0.160275937 | 0.003212458 | 0.084356647 | Down | 2.493162511 |
| SBDS | -0.415353085 | 0.001795608 | 0.066048296 | Down | 2.745788443 |
| TYW1 | -0.029450266 | 0.545871083 | 0.71445534 | NS | 0.262909911 |
| SPDYE21 | 0.148102277 | 0.449174873 | 0.642736828 | NS | 0.347584547 |
| AUTS2 | 0.1245326 | 0.256540447 | 0.48352311 | NS | 0.590844153 |
| GALNT17 | 0.697708722 | 0.148750063 | NA | NS | 0.827542843 |
| CALN1 | 0.431333565 | 0.243681271 | 0.471587269 | NS | 0.613177849 |
| TYW1B | -0.065513907 | 0.355189127 | 0.568411807 | NS | 0.449540338 |
| POM121 | -0.153108082 | 0.244353313 | 0.47233116 | NS | 0.611981768 |
| TRIM74 | 0.031473605 | 0.921367293 | NA | NS | 0.035567208 |
| SPDYE10P | 1.32E-05 | 0.999965095 | NA | NS | 1.52E-05 |
| NSUN5 | -0.082302075 | 0.162260807 | 0.389928636 | NS | 0.789786369 |
| TRIM50 | 0.894349371 | 0.009401086 | NA | Up | 2.026821996 |
| FKBP6 | 0.685427191 | 0.131861895 | NA | NS | 0.879880688 |
| FZD9 | 0.761839289 | 0.099676906 | NA | NS | 1.001405453 |
| BAZ1B | -0.104693832 | 0.055653064 | 0.248695897 | NS | 1.25451092 |
| BCL7B | 0.011497817 | 0.855337959 | 0.918677013 | NS | 0.067862254 |
| TBL2 | -0.185570804 | 0.003554833 | 0.086397822 | Down | 2.449180827 |
| MLXIPL | 0.749193307 | 0.105081127 | 0.321069717 | NS | 0.978475278 |
| VPS37D | 0.636377966 | 0.059795388 | NA | NS | 1.223332309 |
| DNAJC30 | 0.37026266 | 6.29E-04 | 0.041844574 | Up | 3.201675815 |
| BUD23 | -0.289890961 | 3.51E-04 | 0.032169672 | Down | 3.454486764 |
| STX1A | 0.080035577 | 0.651805174 | 0.79024045 | NS | 0.185882196 |
| ABHD11 | -0.025364225 | 0.767831913 | 0.864743324 | NS | 0.114733841 |
| CLDN3 | 1.11223373 | 0.045611946 | NA | Up | 1.3409214 |
| CLDN4 | 0.551903556 | 0.12831354 | 0.350046063 | NS | 0.891727513 |
| METTL27 | 0.517525658 | 0.060960361 | NA | NS | 1.214952467 |
| TMEM270 | 0.878491277 | 0.128402218 | NA | NS | 0.891427473 |
| ELN | 0.61166922 | 0.153673511 | 0.380291042 | NS | 0.813400986 |
| LIMK1 | 0.220907317 | 0.04331949 | 0.223141939 | Up | 1.363316663 |
| EIF4H | -0.174955677 | 0.007357501 | 0.112409264 | Down | 2.133269674 |
| LAT2 | -0.074699103 | 0.390750018 | 0.597712859 | NS | 0.408100993 |
| RFC2 | -0.080730639 | 0.200520857 | 0.430188957 | NS | 0.697840448 |
| CLIP2 | 0.076438694 | 0.589724252 | 0.747377833 | NS | 0.229351012 |
| GTF2IRD1 | 0.754303755 | 0.040292831 | 0.216621104 | Up | 1.394772218 |
| GTF2I | -0.068900416 | 0.298481998 | 0.51995396 | NS | 0.525081857 |
| NCF1 | -0.046190472 | 0.638981239 | 0.780727077 | NS | 0.194511893 |
| GTF2IRD2 | 0.077743542 | 0.526284198 | 0.701283569 | NS | 0.27877967 |
| SPDYE12P | 0.469035472 | 0.037719289 | NA | Up | 1.423436506 |
| CASTOR2 | -0.036914811 | 0.821593311 | 0.898420735 | NS | 0.085343105 |
| RCC1L | -0.142524521 | 0.020594848 | 0.166606577 | Down | 1.686241399 |
| GTF2IRD2B | -0.072092855 | 0.308164222 | 0.528974443 | NS | 0.511217784 |
| TRIM73 | 0.350302376 | 0.134345474 | NA | NS | 0.871776961 |
| POM121C | -0.022308738 | 0.844574425 | 0.912766905 | NS | 0.073362074 |
| SPDYE5 | 0.215401392 | 0.18100231 | 0.410963814 | NS | 0.742315883 |
| HIP1 | 0.09624792 | 0.466272535 | 0.656254399 | NS | 0.331360165 |
| CCL26 | -0.193344952 | 0.781183877 | NA | NS | 0.107246729 |
| CCL24 | 0.745408836 | 0.192545821 | NA | NS | 0.715465904 |
| RHBDD2 | 0.299532258 | 0.001421306 | 0.061070841 | Up | 2.847312303 |
| POR | 0.039790439 | 0.636076622 | 0.77834082 | NS | 0.196490566 |
| TMEM120A | 0.040863431 | 0.588474105 | 0.746745381 | NS | 0.230272643 |
| STYXL1 | -0.114998734 | 0.111906186 | 0.330693609 | NS | 0.951145905 |
| MDH2 | 0.009098486 | 0.861568289 | 0.921952946 | NS | 0.064710294 |
| SRRM3 | 0.703059471 | 0.11457118 | 0.333321795 | NS | 0.940924613 |
| HSPB1 | 0.30109656 | 0.231361186 | 0.459789912 | NS | 0.635709498 |
| YWHAG | -0.053690323 | 0.341246869 | 0.556878708 | NS | 0.466931325 |
| SSC4D | 0.412565161 | 0.109234209 | NA | NS | 0.961641331 |
| ZP3 | -0.035603446 | 0.856336186 | 0.919130533 | NS | 0.067355704 |
| DTX2 | 0.031586737 | 0.83898862 | 0.909175612 | NS | 0.07624393 |
| UPK3B | 0.305962274 | 0.350638853 | NA | NS | 0.455139963 |
| SPDYE16 | 0.360476909 | 0.294733725 | NA | NS | 0.530570168 |
| POMZP3 | -0.072736756 | 0.689125439 | 0.815288294 | NS | 0.161701718 |
| SPDYE17 | -0.078330129 | 0.743256194 | NA | NS | 0.128861463 |
| SPDYE18 | 0.137222697 | 0.292697381 | 0.515290877 | NS | 0.533581164 |
| CCDC146 | -0.116255876 | 0.163003677 | 0.390737932 | NS | 0.787802599 |
| FGL2 | -0.219046132 | 0.089902845 | 0.300314316 | NS | 1.046226566 |
| GSAP | -0.082019086 | 0.104120142 | 0.32021733 | NS | 0.982465247 |
| PTPN12 | 0.115368229 | 0.073149324 | 0.275129876 | NS | 1.135789684 |
| RSBN1L | -0.135857941 | 0.00387051 | 0.089594751 | Down | 2.412231839 |
| TMEM60 | -0.123840582 | 0.174244624 | 0.403422181 | NS | 0.758840613 |
| PHTF2 | 0.100420116 | 0.040702951 | 0.217747953 | Up | 1.390374102 |
| MAGI2 | 0.232758436 | 0.447747351 | 0.641535901 | NS | 0.348966975 |
| GNAI1 | 0.210823862 | 0.391095816 | 0.597922322 | NS | 0.40771683 |
| CD36 | -0.166739632 | 0.121581779 | 0.342324306 | NS | 0.915131505 |
| GNAT3 | 1.186617326 | 0.031841183 | NA | Up | 1.497010804 |
| SEMA3C | -0.158024612 | 0.163783901 | 0.391542682 | NS | 0.78572879 |
| HGF | 0.036489235 | 0.717104684 | 0.832831159 | NS | 0.14441744 |
| CACNA2D1 | 0.447985856 | 0.299134384 | 0.520433287 | NS | 0.524133664 |
| PCLO | 0.498475219 | 0.216375163 | 0.445832118 | NS | 0.664792591 |
| SEMA3E | 0.608569423 | 0.209842163 | 0.439810123 | NS | 0.678107246 |
| SEMA3A | 0.07326043 | 0.758429536 | 0.858918632 | NS | 0.120084762 |
| SEMA3D | 0.43438125 | 0.158745383 | 0.386312482 | NS | 0.799298896 |
| GRM3 | 0.38511327 | 0.143314627 | 0.368289003 | NS | 0.843709482 |
| ELAPOR2 | -0.171369376 | 0.132590583 | 0.355614187 | NS | 0.877487318 |
| DMTF1 | 0.002136916 | 0.963100377 | 0.979350184 | NS | 0.016328447 |
| TMEM243 | -0.02001151 | 0.72489524 | 0.838061435 | NS | 0.139724752 |
| CROT | -0.095227226 | 0.10379156 | 0.319643728 | NS | 0.983837961 |
| ABCB4 | 0.047806539 | 0.682020162 | 0.810483683 | NS | 0.166202787 |
| ABCB1 | -0.137732678 | 0.10459138 | 0.320548581 | NS | 0.980504105 |
| RUNDC3B | 0.160568656 | 0.098904464 | 0.313746489 | NS | 1.004784107 |
| SLC25A40 | -0.021344969 | 0.705068759 | 0.825367242 | NS | 0.151768528 |
| DBF4 | -0.290864195 | 8.44E-04 | 0.048398623 | Down | 3.073907323 |
| ADAM22 | 0.034933269 | 0.649953172 | 0.788955719 | NS | 0.187117932 |
| SRI | -0.119270206 | 0.099676166 | 0.314777508 | NS | 1.001408674 |
| STEAP4 | 0.144991026 | 0.357687912 | 0.570815174 | NS | 0.446495736 |
| ZNF804B | 0.569243642 | 0.228471272 | 0.457089362 | NS | 0.6411684 |
| TEX47 | 1.138341583 | 0.082186339 | NA | NS | 1.085200367 |
| STEAP1 | 0.837070529 | 0.065660813 | NA | NS | 1.182693742 |
| STEAP2 | 0.544852688 | 0.140021021 | 0.364288325 | NS | 0.853806761 |
| CFAP69 | 0.46854586 | 0.173298514 | 0.40231957 | NS | 0.761205162 |
| FAM237B | 0.869996784 | 0.092748797 | NA | NS | 1.032691714 |
| GTPBP10 | -0.062230739 | 0.26522566 | 0.491479737 | NS | 0.576384461 |
| CLDN12 | 0.045947236 | 0.663655087 | 0.798687799 | NS | 0.178057572 |
| CDK14 | 0.136833185 | 0.080552828 | 0.286274601 | NS | 1.093919206 |
| FZD1 | 0.031170687 | 0.770969172 | 0.866375662 | NS | 0.112962987 |
| MTERF1 | -0.060412462 | 0.311999269 | 0.531731453 | NS | 0.505846423 |
| AKAP9 | -0.193263713 | 0.017620086 | 0.155449116 | Down | 1.753991984 |
| CYP51A1 | 0.01150989 | 0.924593265 | 0.958927405 | NS | 0.034049274 |
| LRRD1 | 0.636700176 | 0.088788321 | NA | NS | 1.051644158 |
| KRIT1 | 0.068103714 | 0.255061635 | 0.481965476 | NS | 0.593354861 |
| ANKIB1 | -0.194972379 | 0.002161097 | 0.072784472 | Down | 2.665325764 |
| GATAD1 | 0.009104243 | 0.847834948 | 0.914327833 | NS | 0.071688685 |
| ERVW-1 | 0.304234684 | 0.006315092 | 0.106579402 | Up | 2.199620285 |
| PEX1 | -0.091164534 | 0.05138417 | 0.239542615 | NS | 1.289170655 |
| RBM48 | -0.070223117 | 0.219394449 | 0.448822839 | NS | 0.658774364 |
| FAM133B | -0.141965218 | 0.155818887 | 0.383185333 | NS | 0.807379903 |
| CDK6 | -0.038546636 | 0.441063299 | 0.635777422 | NS | 0.355499078 |
| SAMD9 | -0.124327133 | 0.311646032 | 0.531287585 | NS | 0.506338399 |
| SAMD9L | 0.156442568 | 0.154461228 | 0.381395414 | NS | 0.811180517 |
| HEPACAM2 | 0.12995623 | 0.517114728 | 0.694601848 | NS | 0.286413093 |
| VPS50 | -0.256228054 | 0.001795962 | 0.066048296 | Down | 2.745702865 |
| CALCR | 0.494359029 | 0.237935025 | NA | NS | 0.623541623 |
| GNGT1 | 0.651943976 | 0.144447462 | NA | NS | 0.840290083 |
| TFPI2 | 0.726875659 | 0.079080812 | NA | NS | 1.101928878 |
| GNG11 | -0.188808814 | 0.268803941 | 0.494425689 | NS | 0.570564369 |
| BET1 | -0.088275323 | 0.220486592 | 0.449602156 | NS | 0.656617815 |
| COL1A2 | 0.480311684 | 0.325114337 | 0.543578482 | NS | 0.487963878 |
| CASD1 | -0.137463838 | 0.045865493 | 0.228598809 | Down | 1.338513931 |
| SGCE | 0.048889246 | 0.645268171 | 0.785767621 | NS | 0.190259757 |
| PEG10 | 0.147158684 | 0.441460354 | 0.63607956 | NS | 0.355108293 |
| PPP1R9A | 0.025251156 | 0.880421155 | 0.932334579 | NS | 0.055309531 |
| PON1 | 0.577550665 | 0.159487018 | NA | NS | 0.797274663 |
| PON3 | 0.728362925 | 0.146139072 | NA | NS | 0.835233654 |
| PON2 | -0.087642279 | 0.207786925 | 0.43757584 | NS | 0.682381784 |
| ASB4 | 0.500145411 | 0.269731946 | NA | NS | 0.569067614 |
| PDK4 | -0.042489172 | 0.69969271 | 0.822141627 | NS | 0.155092651 |
| DYNC1I1 | 0.266107133 | 0.381015637 | 0.590873519 | NS | 0.4190572 |
| SLC25A13 | 0.040199873 | 0.334805488 | 0.551277252 | NS | 0.475207431 |
| SEM1 | 0.047604953 | 0.478598686 | 0.666071166 | NS | 0.320028498 |
| DLX6 | 0.660049239 | 0.2387852 | NA | NS | 0.621992594 |
| DLX5 | 0.838058357 | 0.11351436 | NA | NS | 0.944949196 |
| SDHAF3 | -0.096776327 | 0.440529009 | 0.635367175 | NS | 0.356025487 |
| TAC1 | 1.171687061 | 0.024199863 | NA | Up | 1.616187094 |
| ASNS | 0.229983835 | 0.035671429 | 0.206431857 | Up | 1.447679493 |
| OCM2 | 0.713299026 | 0.243790128 | NA | NS | 0.612983885 |
| LMTK2 | 0.051090592 | 0.584229097 | 0.743210286 | NS | 0.233416817 |
| BHLHA15 | 0.07653964 | 0.533066891 | 0.705888336 | NS | 0.273218291 |
| TECPR1 | -0.184203963 | 0.033262291 | 0.201101634 | Down | 1.47804784 |
| BRI3 | 0.185781537 | 0.019917519 | 0.163747187 | Up | 1.700764763 |
| BAIAP2L1 | 0.16425553 | 0.110306465 | 0.328810103 | NS | 0.957399032 |
| NPTX2 | 0.804120493 | 0.022565189 | NA | Up | 1.646561018 |
| TMEM130 | 0.94114206 | 0.038141591 | NA | Up | 1.418601191 |
| TRRAP | 0.0888913 | 0.306359658 | 0.527497849 | NS | 0.513768424 |
| SMURF1 | 0.007366885 | 0.886580399 | 0.935950794 | NS | 0.052281874 |
| ARPC1A | -0.0017156 | 0.971304912 | 0.983614147 | NS | 0.012644415 |
| ARPC1B | -0.028713685 | 0.641757004 | 0.783157612 | NS | 0.192629383 |
| PDAP1 | -0.128616708 | 0.059583256 | 0.254791869 | NS | 1.224875769 |
| BUD31 | -0.209496435 | 0.011405257 | 0.131191965 | Down | 1.942894914 |
| PTCD1 | -0.043165862 | 0.53596964 | 0.708067984 | NS | 0.27085981 |
| CPSF4 | -0.142100893 | 0.005521224 | 0.100834985 | Down | 2.257964635 |
| ATP5MF | -0.020696039 | 0.807341661 | 0.889015228 | NS | 0.092942636 |
| ZNF789 | 0.111409076 | 0.065680875 | 0.265108108 | NS | 1.182561069 |
| ZNF394 | -0.160942891 | 0.005087277 | 0.09830662 | Down | 2.293514625 |
| ZKSCAN5 | -0.118509696 | 0.002851495 | 0.080030557 | Down | 2.544927414 |
| FAM200A | -0.031734998 | 0.723345777 | 0.836839008 | NS | 0.140654049 |
| ZNF655 | -0.032168737 | 0.323781958 | 0.542526661 | NS | 0.489747355 |
| TMEM225B | 0.144534826 | 0.572140846 | NA | NS | 0.242497046 |
| ZSCAN25 | -0.068246715 | 0.089216221 | 0.299593825 | NS | 1.049556177 |
| CYP3A5 | 0.026792888 | 0.630381678 | 0.775009952 | NS | 0.200396418 |
| CYP3A7-CYP3A51P | 0.448909599 | 0.247430955 | NA | NS | 0.606545969 |
| CYP3A7 | 0.304485463 | 0.135685722 | 0.3596977 | NS | 0.867465851 |
| CYP3A4 | 0.062304394 | 0.711098342 | 0.829246312 | NS | 0.148070334 |
| CYP3A43 | 0.379016026 | 0.273173716 | NA | NS | 0.563561089 |
| OR2AE1 | 0.154851103 | 0.616709465 | NA | NS | 0.209919386 |
| TRIM4 | -0.036393271 | 0.351913733 | 0.565501566 | NS | 0.453563785 |
| GJC3 | 0.213288548 | 0.377209603 | NA | NS | 0.423417259 |
| AZGP1 | 0.435863627 | 0.412063834 | NA | NS | 0.385035501 |
| ZKSCAN1 | -0.004278597 | 0.912564019 | 0.951241312 | NS | 0.039736659 |
| ZSCAN21 | -0.178203401 | 0.012252666 | 0.134364821 | Down | 1.9117694 |
| ZNF3 | -0.157340392 | 3.80E-05 | 0.00829096 | Down | 4.420079305 |
| COPS6 | -0.105113478 | 0.068300425 | 0.268476763 | NS | 1.165576592 |
| MCM7 | -0.11052031 | 0.054001407 | 0.245579871 | NS | 1.267594922 |
| AP4M1 | 0.102582915 | 0.246013828 | 0.474180162 | NS | 0.609040482 |
| TAF6 | -0.007605647 | 0.958378456 | 0.976977806 | NS | 0.018462958 |
| CNPY4 | -0.140156615 | 0.01042636 | 0.127457165 | Down | 1.981867285 |
| MBLAC1 | 0.117968853 | 0.360994413 | 0.573615336 | NS | 0.442499519 |
| LAMTOR4 | -0.134408333 | 0.047160069 | 0.230880699 | Down | 1.326425565 |
| TRAPPC14 | 0.118787086 | 0.203560731 | 0.433508262 | NS | 0.691305999 |
| GAL3ST4 | 0.115991398 | 0.502341648 | 0.683619254 | NS | 0.299000814 |
| GPC2 | 0.097048114 | 0.549564138 | 0.717321207 | NS | 0.259981615 |
| STAG3 | 0.201435841 | 0.081133864 | 0.2872357 | NS | 1.090797843 |
| PVRIG | 0.463996459 | 0.040968866 | 0.218105862 | Up | 1.387546057 |
| SPDYE3 | 0.122165666 | 0.49167368 | 0.675807863 | NS | 0.30832304 |
| PILRB | 0.060356078 | 0.535756266 | 0.707851045 | NS | 0.271032741 |
| PILRA | 0.427390512 | 0.002081363 | 0.071675497 | Up | 2.681652152 |
| ZCWPW1 | 0.060660266 | 0.513632728 | 0.691791152 | NS | 0.289347311 |
| MEPCE | -0.073724173 | 0.228402923 | 0.457089362 | NS | 0.641298342 |
| PPP1R35 | 0.092777321 | 0.316729766 | 0.536302987 | NS | 0.49931112 |
| C7orf61 | 0.203539552 | 0.390524777 | NA | NS | 0.408351407 |
| TSC22D4 | 0.048436815 | 0.754256544 | 0.856164918 | NS | 0.122480913 |
| NYAP1 | 0.790763971 | 0.01660815 | 0.152014927 | Up | 1.779678752 |
| AGFG2 | -0.029117758 | 0.667625379 | 0.80123442 | NS | 0.175467163 |
| LRCH4 | -0.135660777 | 0.118671875 | 0.339261425 | NS | 0.925652196 |
| FBXO24 | 0.271353796 | 0.201658387 | 0.431600737 | NS | 0.695383711 |
| PCOLCE | 0.238222712 | 0.333936682 | 0.550559204 | NS | 0.476335872 |
| MOSPD3 | 0.171382747 | 0.306615291 | 0.52763506 | NS | 0.513406191 |
| TFR2 | 0.057464701 | 0.742301455 | 0.84888192 | NS | 0.129419688 |
| ACTL6B | 0.602643418 | 0.204969588 | NA | NS | 0.688310571 |
| GNB2 | 0.0293125 | 0.748018428 | 0.851877572 | NS | 0.126087703 |
| GIGYF1 | -0.043307328 | 0.726155938 | 0.838611255 | NS | 0.138970107 |
| POP7 | -0.080186945 | 0.367203056 | 0.579396578 | NS | 0.435093713 |
| EPO | 0.867779432 | 0.070358408 | NA | NS | 1.152683997 |
| ZAN | 0.460404674 | 0.269496715 | 0.494869609 | NS | 0.569446525 |
| EPHB4 | 0.284027723 | 0.107560132 | 0.324850607 | NS | 0.968348672 |
| SLC12A9 | 0.108061023 | 0.22398523 | 0.452443504 | NS | 0.64978062 |
| TRIP6 | 0.001358221 | 0.98898184 | 0.993580544 | NS | 0.004811683 |
| SRRT | -0.026479907 | 0.825759249 | 0.900867904 | NS | 0.083146553 |
| UFSP1 | 0.098124344 | 0.449607714 | 0.643129524 | NS | 0.347166246 |
| ACHE | 0.182663602 | 0.481863801 | 0.668490967 | NS | 0.317075698 |
| MUC3A | 0.375994487 | 0.369505103 | 0.580929901 | NS | 0.432379559 |
| MUC12 | 0.313093012 | 0.294760112 | 0.516811827 | NS | 0.530531287 |
| MUC17 | 0.297726775 | 0.517967296 | 0.69521796 | NS | 0.28569766 |
| TRIM56 | 0.085195946 | 0.184586015 | 0.414734144 | NS | 0.733801206 |
| SERPINE1 | 0.16985582 | 0.482468161 | 0.668652698 | NS | 0.316531341 |
| AP1S1 | -0.07116025 | 0.476696297 | 0.664473439 | NS | 0.321758221 |
| VGF | 0.929560177 | 0.03915747 | NA | Up | 1.407185379 |
| NAT16 | 0.754752463 | 0.146874022 | NA | NS | 0.833055012 |
| MOGAT3 | 0.95566102 | 0.093193829 | NA | NS | 1.030612845 |
| PLOD3 | -0.098123437 | 0.265442489 | 0.491585012 | NS | 0.576029559 |
| ZNHIT1 | -0.023798711 | 0.739055723 | 0.846651166 | NS | 0.131322816 |
| CLDN15 | 0.114769878 | 0.304900944 | 0.526137408 | NS | 0.515841232 |
| FIS1 | -0.18507413 | 0.150650861 | 0.377154133 | NS | 0.822028383 |
| IFT22 | 0.055712251 | 0.680890951 | 0.809943926 | NS | 0.166922438 |
| COL26A1 | 0.628635331 | 0.046039273 | 0.228777969 | Up | 1.336871547 |
| MYL10 | 0.310294879 | 0.634349472 | NA | NS | 0.197671418 |
| CUX1 | 0.125541373 | 0.215771505 | 0.445189099 | NS | 0.66600591 |
| SH2B2 | 0.056615445 | 0.696952702 | 0.820297213 | NS | 0.156796694 |
| SPDYE6 | 0.242311573 | 0.276668272 | 0.500969068 | NS | 0.558040642 |
| PRKRIP1 | -0.151819877 | 0.001698802 | 0.065122985 | Down | 2.769857168 |
| ORAI2 | 0.053465565 | 0.518687995 | 0.695808062 | NS | 0.285093804 |
| ALKBH4 | -0.194026104 | 0.077636693 | 0.281861236 | NS | 1.109932971 |
| LRWD1 | 0.081414284 | 0.325482462 | 0.54398808 | NS | 0.487472407 |
| POLR2J | -0.044978791 | 0.504138828 | 0.684803526 | NS | 0.297449853 |
| RASA4B | 0.146083868 | 0.355978106 | 0.568958796 | NS | 0.448576712 |
| POLR2J3 | 0.017664784 | 0.904162953 | 0.946871825 | NS | 0.043753292 |
| SPDYE2 | 0.134082243 | 0.577148181 | 0.737685437 | NS | 0.238712668 |
| RASA4 | 0.024382214 | 0.915315648 | 0.95297354 | NS | 0.038429113 |
| SPDYE2B | 0.225282987 | 0.612795623 | NA | NS | 0.212684345 |
| POLR2J2 | -0.03003168 | 0.863429222 | 0.922909365 | NS | 0.063773257 |
| FAM185A | 0.050100646 | 0.471888665 | 0.660546067 | NS | 0.326160455 |
| FBXL13 | 0.129155371 | 0.343626529 | 0.558632857 | NS | 0.463913314 |
| LRRC17 | 0.214960555 | 0.174365632 | 0.40352887 | NS | 0.758539112 |
| ARMC10 | -0.213641394 | 0.006758242 | 0.109383228 | Down | 2.170166233 |
| NAPEPLD | -0.153749516 | 0.102794834 | 0.318649084 | NS | 0.988028712 |
| PMPCB | -0.095453655 | 0.109915774 | 0.328242412 | NS | 0.958939978 |
| DNAJC2 | -0.08312169 | 0.300459596 | 0.521930042 | NS | 0.52221392 |
| PSMC2 | -0.137099559 | 0.160511441 | 0.388258664 | NS | 0.794494005 |
| SLC26A5 | 0.395350774 | 0.231520545 | 0.45979109 | NS | 0.635410463 |
| RELN | 0.201431523 | 0.521041993 | 0.696811896 | NS | 0.283127274 |
| ORC5 | -0.204439913 | 0.006891736 | 0.109912638 | Down | 2.161671341 |
| LHFPL3 | 0.916469858 | 0.012026671 | NA | Up | 1.919854552 |
| KMT2E | -0.03944678 | 0.615156416 | 0.764630244 | NS | 0.211014442 |
| SRPK2 | 0.071104088 | 0.235902855 | 0.463687081 | NS | 0.627266803 |
| PUS7 | -0.328824493 | 0.009013078 | 0.120219864 | Down | 2.045126882 |
| RINT1 | -0.191151912 | 0.013165611 | 0.137679829 | Down | 1.880558978 |
| EFCAB10 | 0.078708646 | 0.630826608 | 0.775188655 | NS | 0.200089997 |
| ATXN7L1 | 0.076959454 | 0.331150091 | 0.548174874 | NS | 0.479975121 |
| CDHR3 | 0.198198107 | 0.041764354 | 0.220293088 | Up | 1.379194235 |
| SYPL1 | -0.018665298 | 0.793102163 | 0.880637817 | NS | 0.100670866 |
| NAMPT | 0.106281037 | 0.469973364 | 0.659019796 | NS | 0.327926755 |
| CCDC71L | 0.064075864 | 0.444521222 | 0.638698645 | NS | 0.3521075 |
| PIK3CG | 0.037650077 | 0.538520925 | 0.709538961 | NS | 0.268797417 |
| PRKAR2B | -0.125504613 | 0.398547904 | 0.603473989 | NS | 0.399519471 |
| HBP1 | -0.094335623 | 0.249114487 | 0.476376043 | NS | 0.603601015 |
| COG5 | -0.010943563 | 0.844985849 | 0.913039204 | NS | 0.073150564 |
| GPR22 | 0.098708817 | 0.790359673 | NA | NS | 0.102175227 |
| DUS4L | -0.148187078 | 0.712552356 | NA | NS | 0.14718322 |
| DUS4L-BCAP29 | 0.173046857 | 0.207801713 | 0.43757584 | NS | 0.682350876 |
| SLC26A4 | 0.293460032 | 0.278853223 | 0.503208948 | NS | 0.554624332 |
| CBLL1 | 0.055617042 | 0.328526372 | 0.546240087 | NS | 0.483429763 |
| SLC26A3 | 0.015645834 | 0.902052316 | 0.9454453 | NS | 0.044768274 |
| DLD | -0.045313976 | 0.370683588 | 0.581784233 | NS | 0.430996642 |
| LAMB1 | 0.223683856 | 0.004376061 | 0.093367359 | Up | 2.358916659 |
| LAMB4 | 0.44236787 | 0.217625 | 0.447248377 | NS | 0.662291215 |
| NRCAM | 0.309966458 | 0.133304561 | 0.35644448 | NS | 0.87515499 |
| PNPLA8 | -0.175591184 | 0.039717707 | 0.215730166 | Down | 1.401015831 |
| THAP5 | -0.141816207 | 0.11098645 | 0.329580794 | NS | 0.95473004 |
| DNAJB9 | -0.200850929 | 0.054473395 | 0.246746378 | NS | 1.263815559 |
| IMMP2L | 0.093609649 | 0.265003218 | 0.49119273 | NS | 0.576748853 |
| LRRN3 | -0.093701746 | 0.5616834 | 0.726076138 | NS | 0.250508411 |
| DOCK4 | 0.232133377 | 0.119080232 | 0.339629468 | NS | 0.924160328 |
| ZNF277 | -0.270713021 | 0.005681871 | 0.102180893 | Down | 2.245508632 |
| IFRD1 | 0.128172738 | 0.246939739 | 0.475085172 | NS | 0.607409016 |
| LSMEM1 | 0.225023728 | 0.167828039 | 0.396002241 | NS | 0.775135479 |
| TMEM168 | 0.037677469 | 0.4978808 | 0.679810772 | NS | 0.302874621 |
| BMT2 | -0.05396372 | 0.481811169 | 0.668470309 | NS | 0.317123137 |
| GPR85 | 0.208264076 | 0.479691061 | 0.666709632 | NS | 0.319038374 |
| SMIM30 | -0.28320816 | 0.07555433 | 0.278850098 | NS | 1.12174064 |
| PPP1R3A | 0.902640186 | 0.100135141 | NA | NS | 0.999413487 |
| FOXP2 | 0.544515978 | 0.182855375 | 0.413034205 | NS | 0.737892269 |
| MDFIC | -0.207661984 | 0.008805983 | 0.119193046 | Down | 2.055222134 |
| TFEC | -0.084739075 | 0.445892217 | 0.639795791 | NS | 0.350770109 |
| TES | -0.181927415 | 0.025316848 | 0.18002011 | Down | 1.596590359 |
| CAV2 | 0.131523778 | 0.530204798 | 0.70437672 | NS | 0.275556347 |
| CAV1 | 0.640437722 | 0.003872972 | 0.089594751 | Up | 2.411955651 |
| MET | 0.536672454 | 0.130776445 | 0.352897626 | NS | 0.883470471 |
| CAPZA2 | -0.163030759 | 0.205548326 | 0.435492143 | NS | 0.687086056 |
| ST7 | -0.147913509 | 0.185630853 | 0.415463304 | NS | 0.73134984 |
| WNT2 | 0.672964563 | 0.182826502 | NA | NS | 0.737960851 |
| CFTR | 0.159139678 | 0.707390973 | 0.826867416 | NS | 0.150340487 |
| ASZ1 | 0.539121148 | 0.303808791 | NA | NS | 0.517399664 |
| CTTNBP2 | 0.258816328 | 0.437648224 | 0.633299747 | NS | 0.35887483 |
| LSM8 | -0.103013506 | 0.130219729 | 0.352142193 | NS | 0.885323213 |
| ANKRD7 | 0.129891874 | 0.711038685 | NA | NS | 0.148106771 |
| KCND2 | 0.52997205 | 0.246476025 | NA | NS | 0.608225319 |
| TSPAN12 | 0.323259987 | 0.431907177 | NA | NS | 0.364609579 |
| ING3 | -0.120828042 | 0.032627775 | 0.199583679 | Down | 1.486412547 |
| CPED1 | -0.094422753 | 0.270060213 | 0.495546752 | NS | 0.568539393 |
| WNT16 | 0.014868666 | 0.889078104 | 0.93735026 | NS | 0.051060085 |
| FAM3C | -0.064110432 | 0.458827372 | 0.650291756 | NS | 0.338350681 |
| PTPRZ1 | 0.555314718 | 0.204072532 | 0.434170494 | NS | 0.690215447 |
| AASS | -0.031068949 | 0.751766131 | 0.854439104 | NS | 0.123917244 |
| FEZF1 | 0.731027496 | 0.170258844 | NA | NS | 0.768890319 |
| CADPS2 | 0.539506863 | 0.206338638 | 0.436307756 | NS | 0.685419441 |
| RNF133 | 0.14180384 | 0.828044181 | NA | NS | 0.08194649 |
| RNF148 | 1.064332807 | 0.096197905 | NA | NS | 1.016834386 |
| TAS2R16 | 0.296787337 | 0.608517986 | NA | NS | 0.215726581 |
| SLC13A1 | 0.281751011 | 0.573019446 | NA | NS | 0.24183064 |
| IQUB | 0.4586025 | 0.080431421 | 0.286164425 | NS | 1.094574258 |
| NDUFA5 | -0.277917511 | 0.017336061 | 0.154546701 | Down | 1.761049567 |
| ASB15 | 0.755067842 | 0.06436151 | 0.263168594 | NS | 1.191373777 |
| LMOD2 | 0.20566152 | 0.537345578 | NA | NS | 0.269746321 |
| WASL | 0.072604954 | 0.24769689 | 0.47582236 | NS | 0.606079446 |
| HYAL4 | 0.301821573 | 0.454634214 | NA | NS | 0.342337884 |
| SPAM1 | 0.7545206 | 0.128388014 | NA | NS | 0.891475518 |
| TMEM229A | 0.705536857 | 0.228821222 | NA | NS | 0.6405037 |
| SSU72P8 | 0.326750229 | 0.820171289 | NA | NS | 0.086095438 |
| GPR37 | 0.976135747 | 0.029966446 | NA | Up | 1.523364767 |
| POT1 | -0.068684225 | 0.315585476 | 0.535056158 | NS | 0.500882992 |
| GRM8 | 0.592494159 | 0.180851612 | 0.410776914 | NS | 0.742677615 |
| ZNF800 | -0.002002411 | 0.967426174 | 0.981874329 | NS | 0.014382167 |
| GCC1 | -0.162971972 | 0.008113524 | 0.115845343 | Down | 2.090790466 |
| ARF5 | 0.119578466 | 0.105292231 | 0.32124631 | NS | 0.977603672 |
| FSCN3 | 0.188505511 | 0.434208064 | 0.630585691 | NS | 0.362302115 |
| PAX4 | 0.750396176 | 0.135078897 | NA | NS | 0.869412494 |
| SND1 | -0.145403481 | 0.039807688 | 0.215730166 | Down | 1.40003304 |
| LRRC4 | 0.104750999 | 0.434654229 | 0.630833536 | NS | 0.361856091 |
| LEP | 0.174427413 | 0.592126476 | NA | NS | 0.22758552 |
| RBM28 | 0.060354999 | 0.403285134 | 0.60738002 | NS | 0.394387787 |
| PRRT4 | 0.834695971 | 0.011106737 | NA | Up | 1.95441351 |
| IMPDH1 | 0.261717703 | 0.019835989 | 0.163587977 | Up | 1.70254615 |
| HILPDA | 0.052369835 | 0.605493685 | 0.758108478 | NS | 0.217890382 |
| METTL2B | -0.090104529 | 0.10497252 | 0.320907849 | NS | 0.978924377 |
| FAM71F2 | 0.344328865 | 0.021231131 | 0.168379028 | Up | 1.673026874 |
| FAM71F1 | 0.184625788 | 0.692336028 | NA | NS | 0.159683068 |
| CALU | -0.107437582 | 0.078897432 | 0.283714663 | NS | 1.102937134 |
| OPN1SW | 0.495099849 | 0.055679583 | NA | NS | 1.254304027 |
| CCDC136 | 0.051503783 | 0.631499176 | 0.775682174 | NS | 0.199627212 |
| FLNC | 0.802362753 | 0.056958367 | 0.250424364 | NS | 1.244442473 |
| KCP | 0.43015682 | 0.05195568 | 0.240722026 | NS | 1.28436697 |
| ATP6V1F | 0.084460003 | 0.334157998 | 0.55076549 | NS | 0.476048139 |
| ATP6V1FNB | 0.317245365 | 0.08424905 | 0.292034469 | NS | 1.074434989 |
| IRF5 | 0.075257046 | 0.319776085 | 0.538985786 | NS | 0.495154019 |
| TNPO3 | -0.02621521 | 0.624205895 | 0.770816532 | NS | 0.204672134 |
| TSPAN33 | -0.002685679 | 0.979708213 | 0.987953626 | NS | 0.008903251 |
| SMO | 0.80108607 | 0.040334575 | 0.21663753 | Up | 1.394322518 |
| AHCYL2 | 0.031660379 | 0.515434221 | 0.693278651 | NS | 0.287826751 |
| STRIP2 | -0.172837058 | 0.394445202 | 0.60025103 | NS | 0.404013322 |
| SMKR1 | 0.026194083 | 0.868607337 | 0.925923112 | NS | 0.061176507 |
| NRF1 | 0.07238233 | 0.03471368 | 0.204367256 | NS | 1.459499343 |
| UBE2H | -0.16893731 | 0.279958711 | 0.50348007 | NS | 0.552906014 |
| ZC3HC1 | -0.069115533 | 0.263556781 | 0.489853708 | NS | 0.579125806 |
| KLHDC10 | -0.045631134 | 0.146815522 | 0.37239662 | NS | 0.833228026 |
| TMEM209 | 0.034628076 | 0.676540608 | 0.806944066 | NS | 0.16970613 |
| SSMEM1 | 1.146808217 | 0.094767142 | NA | NS | 1.023342218 |
| CPA2 | 0.614278216 | 0.263038449 | NA | NS | 0.579980764 |
| CPA4 | 0.469795726 | 0.3382704 | NA | NS | 0.470736003 |
| CPA5 | 0.583850481 | 0.055249092 | 0.24830006 | NS | 1.257674854 |
| CPA1 | 0.257792211 | 0.551924732 | NA | NS | 0.258120145 |
| CEP41 | 0.071062398 | 0.418156439 | 0.618901466 | NS | 0.378661211 |
| MEST | -0.064159444 | 0.569664956 | 0.73208388 | NS | 0.244380496 |
| COPG2 | -0.004607921 | 0.939306461 | 0.966553041 | NS | 0.027192691 |
| TSGA13 | 0.27517624 | 0.568229385 | NA | NS | 0.245476312 |
| KLF14 | 0.479849557 | 0.221635885 | NA | NS | 0.654359923 |
| MKLN1 | -0.022769309 | 0.766673405 | 0.864134767 | NS | 0.115389602 |
| PODXL | 0.181487012 | 0.048418967 | 0.233401637 | Up | 1.314984482 |
| PLXNA4 | 0.175174977 | 0.241706071 | 0.469632005 | NS | 0.616712441 |
| CHCHD3 | -0.135452929 | 0.007899264 | 0.11472857 | Down | 2.10241337 |
| EXOC4 | -0.114935762 | 0.010741358 | 0.12826482 | Down | 1.968940818 |
| LRGUK | 0.376917635 | 0.130736382 | 0.35283109 | NS | 0.883603537 |
| SLC35B4 | -0.025075564 | 0.539677824 | 0.710246037 | NS | 0.267865427 |
| AKR1B1 | -0.104666051 | 0.181382796 | 0.411330706 | NS | 0.741403907 |
| AKR1B10 | 0.722831477 | 0.1193444 | NA | NS | 0.923197954 |
| AKR1B15 | 0.242710217 | 0.571127573 | NA | NS | 0.243266872 |
| BPGM | -0.11264053 | 0.606046902 | 0.758494242 | NS | 0.217493765 |
| CALD1 | 0.188386099 | 0.184855279 | 0.415049925 | NS | 0.733168142 |
| AGBL3 | 0.128863594 | 0.111448486 | 0.329942771 | NS | 0.952925827 |
| CYREN | 0.003161882 | 0.966180646 | 0.98095798 | NS | 0.014941666 |
| TMEM140 | 0.040289153 | 0.685538628 | 0.813083944 | NS | 0.163968069 |
| WDR91 | 0.047923131 | 0.440034563 | 0.635045158 | NS | 0.35651321 |
| STRA8 | 0.427532849 | 0.410518624 | NA | NS | 0.386667136 |
| CNOT4 | 0.079603831 | 0.247648809 | 0.475813479 | NS | 0.606163757 |
| NUP205 | -0.051913067 | 0.306461679 | 0.527590736 | NS | 0.513623823 |
| STMP1 | 0.163323845 | 0.286906557 | 0.50999177 | NS | 0.542259527 |
| SLC13A4 | 0.090192027 | 0.486904653 | 0.67206682 | NS | 0.312556075 |
| FAM180A | 0.35228516 | 0.490567485 | NA | NS | 0.309301241 |
| MTPN | -0.01892112 | 0.811369988 | 0.891778829 | NS | 0.090781061 |
| CHRM2 | 0.739538265 | 0.142657372 | 0.367341931 | NS | 0.845705781 |
| PTN | 0.552267975 | 0.344285119 | NA | NS | 0.463081749 |
| DGKI | 0.417431802 | 0.22873444 | 0.45734906 | NS | 0.64066844 |
| CREB3L2 | 0.186664613 | 0.028270779 | 0.188091738 | Up | 1.548662224 |
| AKR1D1 | 0.378505487 | 0.423326736 | NA | NS | 0.373324302 |
| TRIM24 | -0.057905551 | 0.322124126 | 0.54065861 | NS | 0.491976747 |
| SVOPL | 0.446500677 | 0.192794464 | 0.421806458 | NS | 0.714905442 |
| ATP6V0A4 | 0.634911622 | 0.151153921 | 0.377654815 | NS | 0.820580582 |
| TMEM213 | 0.351086463 | 0.479200606 | NA | NS | 0.319482642 |
| KIAA1549 | 0.613357839 | 0.082625044 | 0.289737983 | NS | 1.082888295 |
| ZC3HAV1L | 0.207340387 | 0.164171547 | 0.391978395 | NS | 0.78470211 |
| ZC3HAV1 | 0.025899615 | 0.605398779 | 0.758055923 | NS | 0.21795846 |
| TTC26 | 0.113219545 | 0.466216889 | 0.656254399 | NS | 0.331411998 |
| UBN2 | 0.045434279 | 0.546230185 | 0.714753418 | NS | 0.262624304 |
| FMC1 | -0.043019319 | 0.74771486 | 0.851783936 | NS | 0.126263988 |
| LUC7L2 | 0.036362766 | 0.475648594 | 0.663630967 | NS | 0.322713782 |
| KLRG2 | 1.20666699 | 0.017936008 | NA | Up | 1.746274212 |
| CLEC2L | 0.500161581 | 0.0568407 | NA | NS | 1.24534058 |
| HIPK2 | 0.147645342 | 0.17621559 | 0.405682906 | NS | 0.753955673 |
| TBXAS1 | -0.035382599 | 0.662321722 | 0.797602358 | NS | 0.178931001 |
| PARP12 | 0.090518891 | 0.145689986 | 0.37085607 | NS | 0.836570298 |
| KDM7A | -0.003832562 | 0.962939279 | 0.979263171 | NS | 0.016401098 |
| SLC37A3 | 0.099320323 | 0.316899217 | 0.536370252 | NS | 0.499078833 |
| RAB19 | 0.092679201 | 0.515861103 | 0.693487378 | NS | 0.287467218 |
| MKRN1 | -0.28879092 | 0.095373541 | 0.309058959 | NS | 1.020572094 |
| DENND2A | 0.393810812 | 0.346589745 | 0.56126774 | NS | 0.460184291 |
| ADCK2 | -0.136286174 | 0.111049969 | 0.32960107 | NS | 0.954481558 |
| NDUFB2 | -0.225885052 | 0.068735165 | 0.268813653 | NS | 1.16282102 |
| BRAF | 0.104722069 | 0.133009328 | 0.356364174 | NS | 0.876117902 |
| MRPS33 | -0.193342427 | 0.149093016 | 0.375188026 | NS | 0.8265427 |
| TMEM178B | 0.528020834 | 0.038491215 | 0.213464789 | Up | 1.414638383 |
| AGK | -0.07105588 | 0.151334583 | 0.377750911 | NS | 0.820061817 |
| DENND11 | -0.113165821 | 0.112410105 | 0.330929727 | NS | 0.949194645 |
| SSBP1 | -0.269785011 | 0.005149892 | 0.0985614 | Down | 2.288201863 |
| TAS2R3 | 0.151115403 | 0.251647646 | 0.478554711 | NS | 0.599207128 |
| TAS2R4 | 0.179974585 | 0.123029996 | 0.343806623 | NS | 0.90998899 |
| TAS2R5 | 0.400906897 | 0.006234173 | 0.105602826 | Up | 2.20522118 |
| PRSS37 | 0.255249493 | 0.228484805 | 0.457089362 | NS | 0.641142676 |
| MGAM | 0.326726791 | 0.084375198 | 0.292162201 | NS | 1.073785193 |
| OR9A4 | 0.535067323 | 0.19675083 | NA | NS | 0.706083426 |
| CLEC5A | 0.021386788 | 0.865598064 | 0.924020083 | NS | 0.062683724 |
| TAS2R38 | 0.406600474 | 0.198098689 | NA | NS | 0.7031184 |
| MGAM2 | 0.38516513 | 0.063702316 | 0.262205328 | NS | 1.195844777 |
| PRSS1 | 0.1673303 | 0.609693069 | NA | NS | 0.214888742 |
| PRSS2 | 0.039986459 | 0.917425251 | NA | NS | 0.037429311 |
| EPHB6 | -0.022630478 | 0.82005019 | 0.897279394 | NS | 0.086159566 |
| TRPV6 | 0.281986928 | 0.420777403 | 0.620717816 | NS | 0.375947591 |
| TRPV5 | 0.599724766 | 0.139812912 | NA | NS | 0.854452718 |
| KEL | -0.292282234 | 0.166725265 | 0.394802555 | NS | 0.777998584 |
| PIP | -0.03544231 | 0.928861231 | NA | NS | 0.032049164 |
| TAS2R39 | 0.47478683 | 0.063188818 | 0.26136 | NS | 1.199359766 |
| TAS2R40 | 0.279667189 | 0.143277856 | 0.368235827 | NS | 0.843820926 |
| GSTK1 | 0.032650295 | 0.388234311 | 0.596096293 | NS | 0.410906086 |
| TMEM139 | 0.070726549 | 0.782281782 | NA | NS | 0.106636783 |
| CASP2 | 0.167955166 | 0.032713154 | 0.199704044 | Up | 1.485277588 |
| CLCN1 | 0.360829532 | 0.290055156 | 0.512990599 | NS | 0.53751941 |
| FAM131B | -0.175584062 | 0.312479063 | 0.53219272 | NS | 0.505179076 |
| ZYX | -0.175911847 | 0.483143859 | 0.669197484 | NS | 0.315923536 |
| EPHA1 | -0.231593941 | 0.094034869 | 0.30725936 | NS | 1.026711074 |
| TCAF2 | 0.528679669 | 0.017997861 | 0.15641156 | Up | 1.744779106 |
| TCAF1 | -0.267206243 | 0.016721409 | 0.152381397 | Down | 1.776727138 |
| OR2A25 | 0.421206818 | 0.637215239 | NA | NS | 0.195713847 |
| OR2A12 | 0.383522834 | 0.880596313 | NA | NS | 0.055223137 |
| OR2A14 | 0.190364058 | 0.842513201 | NA | NS | 0.074423286 |
| CTAGE4 | 0.006117472 | 0.987490301 | NA | NS | 0.005467161 |
| ARHGEF35 | 0.294416455 | 0.31220404 | NA | NS | 0.505561481 |
| OR2A42 | 0.176440969 | 0.492999085 | NA | NS | 0.307153886 |
| OR2A1 | -0.273806631 | 0.501401845 | NA | NS | 0.299814072 |
| ARHGEF5 | 0.084121995 | 0.662170701 | NA | NS | 0.179030039 |
| NOBOX | 0.04625199 | 0.968148981 | NA | NS | 0.014057808 |
| TPK1 | 0.034086743 | 0.606608524 | 0.758788758 | NS | 0.217091492 |
| CNTNAP2 | 0.245383609 | 0.395093628 | 0.600626238 | NS | 0.403299974 |
| C7orf33 | 0.851448379 | 0.184842984 | NA | NS | 0.73319703 |
| CUL1 | -0.090079771 | 0.106461757 | 0.322999226 | NS | 0.972806371 |
| EZH2 | -0.045876259 | 0.717505977 | 0.83319754 | NS | 0.144174477 |
| PDIA4 | -0.280205885 | 0.008579749 | 0.118335308 | Down | 2.066525392 |
| ZNF786 | 0.049454883 | 0.346449071 | 0.561209256 | NS | 0.460360599 |
| ZNF425 | 0.080201686 | 0.419176057 | 0.619472804 | NS | 0.377603532 |
| ZNF398 | 0.115723814 | 0.047417767 | 0.231351022 | Up | 1.324058902 |
| ZNF282 | 0.001184407 | 0.985345236 | 0.99114444 | NS | 0.006411579 |
| ZNF212 | -0.108903119 | 0.089050396 | 0.299388164 | NS | 1.050364144 |
| ZNF783 | -0.087212239 | 0.173679878 | 0.402796615 | NS | 0.760250494 |
| ZNF777 | 0.15566018 | 0.335724284 | 0.551832552 | NS | 0.474017244 |
| ZNF746 | 0.09435766 | 0.342068302 | 0.557385147 | NS | 0.465887169 |
| KRBA1 | 0.122193983 | 0.243726839 | 0.471597842 | NS | 0.613096644 |
| ZNF467 | 0.062431582 | 0.724914725 | 0.838061435 | NS | 0.139713079 |
| ZNF862 | 0.082185224 | 0.176005645 | 0.405398902 | NS | 0.754473403 |
| ATP6V0E2 | -0.044230883 | 0.532896218 | 0.705823214 | NS | 0.273357362 |
| ACTR3C | 0.155888302 | 0.069263 | 0.269588925 | NS | 1.1594987 |
| ZBED6CL | 0.164147576 | 0.001679951 | 0.064977566 | Up | 2.774703352 |
| LRRC61 | 0.616499038 | 0.006433608 | NA | Up | 2.191545374 |
| RARRES2 | 1.032143983 | 0.007360778 | NA | Up | 2.133076288 |
| REPIN1 | 0.192154517 | 0.005824175 | 0.102762132 | Up | 2.234765612 |
| ZNF775 | -0.085159341 | 0.38201897 | 0.591468256 | NS | 0.417915071 |
| GIMAP8 | -0.115527128 | 0.064909669 | 0.264064926 | NS | 1.187690605 |
| GIMAP7 | -0.192876988 | 0.096811553 | 0.310921075 | NS | 1.014072813 |
| GIMAP4 | -0.162300286 | 0.072461915 | 0.27394225 | NS | 1.139890195 |
| GIMAP6 | -0.214064419 | 0.005918772 | 0.10308115 | Down | 2.227768404 |
| GIMAP2 | -0.228853884 | 0.077951192 | 0.28242971 | NS | 1.108177239 |
| GIMAP1 | -0.135691175 | 0.028879852 | 0.189677769 | Down | 1.539405031 |
| GIMAP5 | -0.064449747 | 0.451909469 | 0.644595831 | NS | 0.344948558 |
| TMEM176B | 0.377088746 | 0.151046997 | 0.377609248 | NS | 0.820887905 |
| TMEM176A | -0.056219871 | 0.841871631 | 0.910963576 | NS | 0.074754125 |
| AOC1 | 0.885368335 | 0.002557373 | 0.077575819 | Up | 2.592205889 |
| KCNH2 | 0.138696571 | 0.444837367 | 0.638990315 | NS | 0.351798738 |
| NOS3 | 0.340516121 | 0.100910761 | 0.316384536 | NS | 0.996062517 |
| ATG9B | 0.27401359 | 0.185729795 | 0.415463304 | NS | 0.73111842 |
| ABCB8 | 0.051957406 | 0.513465549 | 0.691708199 | NS | 0.28948869 |
| ASIC3 | 0.127661092 | 0.610276479 | 0.761291569 | NS | 0.214473368 |
| CDK5 | -0.103685337 | 0.287542394 | 0.510589731 | NS | 0.541298116 |
| SLC4A2 | 0.079288788 | 0.410541043 | 0.613347918 | NS | 0.386643418 |
| FASTK | 0.026003935 | 0.689063911 | 0.815257617 | NS | 0.161740495 |
| TMUB1 | -0.063046536 | 0.367926269 | 0.579920676 | NS | 0.434239203 |
| AGAP3 | 0.121818074 | 0.302793075 | 0.524267164 | NS | 0.518854062 |
| GBX1 | 0.570124531 | 0.264462734 | NA | NS | 0.577635516 |
| ASB10 | 0.968701846 | 0.049024626 | NA | Up | 1.309585713 |
| IQCA1L | 0.765765713 | 0.079869763 | 0.285274066 | NS | 1.097617605 |
| H2BE1 | 0.412161002 | 0.091749396 | NA | NS | 1.037396786 |
| ABCF2 | 0.241116773 | 0.006107223 | 0.104528345 | Up | 2.214156226 |
| CHPF2 | 0.078306071 | 0.181672204 | 0.411702441 | NS | 0.740711516 |
| SMARCD3 | 0.054742821 | 0.472869103 | 0.661191049 | NS | 0.325259062 |
| NUB1 | -0.065957792 | 0.22698861 | 0.455488754 | NS | 0.643995934 |
| WDR86 | 0.161872708 | 0.101452749 | 0.317197386 | NS | 0.993736181 |
| CRYGN | 0.440906624 | 0.178817731 | 0.408874169 | NS | 0.74758942 |
| RHEB | -0.307608836 | 0.004017951 | 0.090296145 | Down | 2.395995319 |
| PRKAG2 | 0.123989753 | 0.006584492 | 0.108312793 | Up | 2.181477697 |
| GALNTL5 | 0.539607442 | 0.28333024 | NA | NS | 0.547707071 |
| GALNT11 | -0.006665195 | 0.856273417 | 0.919121381 | NS | 0.067387539 |
| KMT2C | 0.070608198 | 0.35222397 | 0.56588098 | NS | 0.453181092 |
| XRCC2 | -0.047823952 | 0.730069999 | 0.840420069 | NS | 0.136635498 |
| ACTR3B | 0.003335788 | 0.969094797 | 0.982782429 | NS | 0.013633738 |
| DPP6 | 0.784114241 | 0.063124213 | 0.261328765 | NS | 1.199804022 |
| PAXIP1 | -0.087569159 | 0.049986998 | 0.236733572 | NS | 1.301142948 |
| HTR5A | 0.830156086 | 0.064952765 | NA | NS | 1.187402359 |
| INSIG1 | -0.140290292 | 0.05003015 | 0.236733572 | NS | 1.300768198 |
| EN2 | 0.518932062 | 0.325411202 | NA | NS | 0.487567501 |
| CNPY1 | 0.670015763 | 0.174102618 | NA | NS | 0.759194699 |
| RBM33 | -0.052032147 | 0.612129841 | 0.762731099 | NS | 0.213156449 |
| SHH | 0.721792235 | 0.090359789 | NA | NS | 1.044024793 |
| RNF32 | 0.157852534 | 0.070161672 | 0.270968399 | NS | 1.153900072 |
| LMBR1 | 0.012598152 | 0.720534235 | 0.835048159 | NS | 0.14234538 |
| NOM1 | -0.098127449 | 0.0373525 | 0.210115856 | NS | 1.427680324 |
| MNX1 | 0.899644274 | 0.04719299 | NA | Up | 1.326122503 |
| UBE3C | -0.103257337 | 0.004191473 | 0.091951138 | Down | 2.377633281 |
| DNAJB6 | 0.05235921 | 0.294757739 | 0.516811827 | NS | 0.530534783 |
| PTPRN2 | 0.232639233 | 0.142682739 | 0.367353179 | NS | 0.845628562 |
| NCAPG2 | -0.108261357 | 0.25344985 | 0.480346613 | NS | 0.596107961 |
| ESYT2 | -0.122263566 | 0.108564281 | 0.326591248 | NS | 0.964313038 |
| DYNC2I1 | -0.053225708 | 0.604315297 | 0.757278886 | NS | 0.218736412 |
| VIPR2 | 0.436296436 | 0.083126782 | 0.290385838 | NS | 1.080259033 |
| ZNF596 | -0.029513324 | 0.739925732 | 0.847373693 | NS | 0.130811869 |
| FBXO25 | 0.043735518 | 0.39622484 | 0.601467573 | NS | 0.402058302 |
| TDRP | -0.044909498 | 0.762004167 | 0.86120309 | NS | 0.118042654 |
| ERICH1 | 0.135017097 | 0.092672704 | 0.305072627 | NS | 1.033048165 |
| CLN8 | -0.337395105 | 0.143054641 | NA | NS | 0.844498048 |
| MYOM2 | -0.123495963 | 0.739137087 | 0.846682217 | NS | 0.131275006 |
| CSMD1 | 0.435194448 | 0.09915679 | 0.314049068 | NS | 1.003677539 |
| MCPH1 | -0.04273392 | 0.326194821 | 0.544537778 | NS | 0.486522939 |
| ANGPT2 | 0.171283458 | 0.08889721 | 0.299209951 | NS | 1.051111867 |
| AGPAT5 | -0.266408404 | 0.008197438 | 0.116591208 | Down | 2.086321834 |
| XKR5 | 0.939621946 | 0.213860257 | NA | NS | 0.669869916 |
| DEFA4 | 0.348245946 | 0.217310643 | 0.446955397 | NS | 0.662919003 |
| DEFA3 | 0.533766662 | 0.090174536 | 0.300826981 | NS | 1.044916083 |
| USP17L1 | 0.216401123 | 0.53501882 | NA | NS | 0.271630941 |
| USP17L4 | 0.082249392 | 0.857361981 | NA | NS | 0.066835779 |
| ZNF705G | 1.248774154 | 0.163922355 | NA | NS | 0.785361815 |
| PRAG1 | 0.16065352 | 0.382809722 | 0.59214603 | NS | 0.417017041 |
| CLDN23 | 0.374212553 | 0.374212942 | NA | NS | 0.426881197 |
| MFHAS1 | -0.22088327 | 0.085316464 | 0.293565942 | NS | 1.06896715 |
| ERI1 | 0.061287762 | 0.423958887 | 0.622843442 | NS | 0.372676257 |
| PPP1R3B | 0.326345712 | 0.030688216 | 0.194491094 | Up | 1.513028364 |
| TNKS | 0.071691302 | 0.253222454 | 0.480142719 | NS | 0.596497786 |
| MSRA | 0.060153492 | 0.506047794 | 0.686297541 | NS | 0.295808464 |
| PRSS51 | 0.164070008 | 0.39558595 | 0.601055492 | NS | 0.402759141 |
| RP1L1 | 0.992254826 | 0.055658731 | NA | NS | 1.254466696 |
| PINX1 | -0.15709688 | 0.059614064 | 0.254791869 | NS | 1.224651274 |
| XKR6 | 0.254988495 | 0.045635277 | 0.228134524 | Up | 1.340699308 |
| MTMR9 | -0.032691313 | 0.541751984 | 0.711830403 | NS | 0.26619949 |
| FAM167A | -0.407529175 | 0.105424668 | NA | NS | 0.977057757 |
| BLK | -0.260329048 | 0.022923369 | 0.172380335 | Down | 1.639721547 |
| NEIL2 | -0.171924335 | 0.236145874 | 0.464025123 | NS | 0.626819638 |
| FDFT1 | -0.109782517 | 0.140952385 | 0.365370409 | NS | 0.850927571 |
| CTSB | -0.276235278 | 0.021788357 | 0.169280311 | Down | 1.661775526 |
| USP17L7 | 0.155914748 | 0.804720533 | NA | NS | 0.094354917 |
| USP17L2 | 1.195287537 | 0.00813391 | NA | Up | 2.089700625 |
| FAM86B1 | 0.131536213 | 0.706247963 | NA | NS | 0.151042792 |
| FAM86B2 | 0.817552073 | 0.012036582 | NA | Up | 1.919496833 |
| LONRF1 | 0.028100714 | 0.704006174 | 0.824734454 | NS | 0.152423532 |
| TRMT9B | 0.225419735 | 0.506828201 | NA | NS | 0.295139228 |
| DLC1 | 0.768607936 | 0.011859893 | 0.13321004 | Up | 1.925919226 |
| C8orf48 | 0.896747579 | 0.103597837 | NA | NS | 0.984649312 |
| SGCZ | 0.358132574 | 0.487225372 | NA | NS | 0.312270104 |
| TUSC3 | 0.591023 | 0.115991893 | 0.335282889 | NS | 0.935572364 |
| MSR1 | -0.158645368 | 0.37577041 | 0.586170007 | NS | 0.425077422 |
| FGF20 | 0.586289988 | 0.195663742 | NA | NS | 0.708489645 |
| MICU3 | 0.057780274 | 0.645820574 | 0.786105915 | NS | 0.189888124 |
| ZDHHC2 | -0.086574368 | 0.345201651 | 0.560099767 | NS | 0.461927136 |
| CNOT7 | -0.194534393 | 0.005609715 | 0.101640588 | Down | 2.251059166 |
| VPS37A | -0.08841229 | 0.092217813 | 0.304264722 | NS | 1.035185183 |
| MTMR7 | 0.177450963 | 0.024523823 | 0.177561993 | Up | 1.610411835 |
| SLC7A2 | 0.482463888 | 0.274315405 | 0.499195185 | NS | 0.561749803 |
| PDGFRL | 0.910073152 | 0.01221317 | NA | Up | 1.913171586 |
| MTUS1 | 0.355483501 | 0.038982112 | 0.214288889 | Up | 1.409134635 |
| FGL1 | 0.592157837 | 0.188904986 | NA | NS | 0.723756578 |
| PCM1 | -0.148503748 | 0.031719441 | 0.197517887 | Down | 1.49867448 |
| ASAH1 | -0.008289905 | 0.932719005 | 0.962907079 | NS | 0.030249174 |
| NAT1 | -0.050398526 | 0.60403974 | 0.757057851 | NS | 0.218934488 |
| NAT2 | 0.531356311 | 0.361323005 | NA | NS | 0.442104387 |
| PSD3 | 0.149771542 | 0.238226286 | 0.466312685 | NS | 0.623010319 |
| SH2D4A | 0.194284622 | 0.187370593 | 0.416819945 | NS | 0.727298568 |
| CSGALNACT1 | 0.058473557 | 0.534708926 | 0.707055814 | NS | 0.271882566 |
| INTS10 | -0.114178283 | 0.05200323 | 0.240893602 | NS | 1.283969682 |
| LPL | -0.149908039 | 0.572159136 | 0.733952836 | NS | 0.242483163 |
| SLC18A1 | 0.2973628 | 0.272251843 | 0.497465358 | NS | 0.565029171 |
| ATP6V1B2 | 0.060448456 | 0.50334017 | 0.68432749 | NS | 0.298138409 |
| LZTS1 | 0.520262789 | 0.068616995 | 0.268602708 | NS | 1.163568306 |
| GFRA2 | 0.171822694 | 0.354043863 | 0.567252872 | NS | 0.450942929 |
| DOK2 | 0.017139593 | 0.844229201 | 0.912609137 | NS | 0.07353963 |
| XPO7 | -0.310882374 | 0.026972717 | 0.185246086 | Down | 1.569075305 |
| NPM2 | 0.346112286 | 0.250756481 | NA | NS | 0.600747834 |
| FGF17 | 0.325058531 | 0.147340104 | 0.372983648 | NS | 0.831679028 |
| DMTN | 0.272519483 | 0.217991494 | 0.44751176 | NS | 0.661560452 |
| FHIP2B | 0.03813869 | 0.697189367 | 0.820505184 | NS | 0.156649245 |
| NUDT18 | 0.016699515 | 0.803413641 | 0.887035588 | NS | 0.095060799 |
| HR | 0.745857204 | 0.085226317 | 0.293490206 | NS | 1.069426277 |
| REEP4 | -0.108768268 | 0.537936394 | 0.709095159 | NS | 0.269269073 |
| LGI3 | 0.291048822 | 0.582223328 | NA | NS | 0.234910398 |
| SFTPC | 0.788378837 | 0.065543073 | NA | NS | 1.183473197 |
| BMP1 | 0.045892312 | 0.735278436 | 0.843792643 | NS | 0.13354817 |
| PHYHIP | 0.629101074 | 0.182585802 | NA | NS | 0.738532996 |
| POLR3D | 0.04823031 | 0.394262612 | 0.600130417 | NS | 0.404214405 |
| PIWIL2 | 0.280412498 | 0.190324915 | 0.419844077 | NS | 0.720504355 |
| SLC39A14 | -0.008977052 | 0.909741998 | 0.950021031 | NS | 0.041081756 |
| PPP3CC | -0.079012941 | 0.229441916 | 0.457791831 | NS | 0.639327239 |
| SORBS3 | 0.211111798 | 0.162263276 | 0.389928636 | NS | 0.78977976 |
| PDLIM2 | 0.149348786 | 0.026557786 | 0.184032195 | Up | 1.57580814 |
| C8orf58 | -0.063399572 | 0.453970152 | 0.646396397 | NS | 0.342972701 |
| CCAR2 | 0.015052792 | 0.854363859 | 0.91788906 | NS | 0.068357131 |
| BIN3 | 0.034610674 | 0.525463631 | 0.700679364 | NS | 0.279457338 |
| EGR3 | 0.188529346 | 0.617366666 | 0.76608861 | NS | 0.209456824 |
| PEBP4 | 0.049361919 | 0.897075384 | NA | NS | 0.04717106 |
| RHOBTB2 | 0.123669077 | 0.14157863 | 0.365923729 | NS | 0.849002293 |
| TNFRSF10B | -0.098242897 | 0.119572466 | 0.34007656 | NS | 0.922368814 |
| TNFRSF10C | 0.018722818 | 0.900859974 | 0.944881847 | NS | 0.045342709 |
| TNFRSF10D | -0.063121131 | 0.356892303 | 0.570101662 | NS | 0.447462818 |
| TNFRSF10A | -0.101752755 | 0.047335384 | 0.23126872 | Down | 1.324814096 |
| CHMP7 | -0.120785377 | 0.111358892 | 0.329942771 | NS | 0.953275097 |
| R3HCC1 | -0.217718105 | 0.003793169 | 0.088644036 | Down | 2.420997813 |
| LOXL2 | 0.089812279 | 0.407938875 | 0.611112752 | NS | 0.389404906 |
| ENTPD4 | 0.018534404 | 0.602783754 | 0.756151929 | NS | 0.219838461 |
| SLC25A37 | -0.024683954 | 0.888079715 | 0.936883395 | NS | 0.05154805 |
| NKX3-1 | 0.160267686 | 0.580740148 | 0.740128597 | NS | 0.236018149 |
| NKX2-6 | 1.313880797 | 0.009801749 | NA | Up | 2.008696444 |
| STC1 | 0.321490203 | 0.466382485 | 0.656328603 | NS | 0.331257768 |
| ADAM28 | 0.129667701 | 0.133142038 | 0.356387477 | NS | 0.875684801 |
| ADAMDEC1 | 0.05544554 | 0.689518574 | 0.81541388 | NS | 0.161454031 |
| ADAM7 | 0.461639799 | 0.262737175 | 0.488909438 | NS | 0.580478473 |
| NEFM | 0.511729679 | 0.119203739 | 0.339848628 | NS | 0.923710122 |
| NEFL | 0.013953425 | 0.926582946 | 0.959673247 | NS | 0.033115697 |
| DOCK5 | 0.096393706 | 0.416698018 | 0.617803976 | NS | 0.380178565 |
| GNRH1 | 0.079055848 | 0.500534211 | 0.682172965 | NS | 0.300566234 |
| KCTD9 | -0.028176833 | 0.697501693 | 0.820746225 | NS | 0.156454734 |
| CDCA2 | -0.193602613 | 0.353720815 | 0.567005458 | NS | 0.451339383 |
| EBF2 | 0.592529083 | 0.159839926 | 0.38738272 | NS | 0.79631473 |
| PPP2R2A | 0.054320007 | 0.37266545 | 0.583375539 | NS | 0.428680869 |
| BNIP3L | -0.588184051 | 0.00160261 | 0.063905938 | Down | 2.795172065 |
| PNMA2 | 0.562052773 | 0.086101167 | 0.29485869 | NS | 1.064990964 |
| DPYSL2 | -0.228487302 | 0.055394881 | 0.248604264 | NS | 1.256530363 |
| ADRA1A | 0.671114121 | 0.130141389 | 0.35213444 | NS | 0.885584561 |
| STMN4 | 0.462114834 | 0.27901341 | NA | NS | 0.554374923 |
| TRIM35 | -0.096648105 | 0.075408163 | 0.278548025 | NS | 1.122581637 |
| PTK2B | 0.083078101 | 0.458990485 | 0.650442663 | NS | 0.338196317 |
| CHRNA2 | 0.236160517 | 0.041264896 | 0.218811914 | Up | 1.384419242 |
| EPHX2 | 0.019273729 | 0.863546744 | 0.922951085 | NS | 0.063714149 |
| CLU | 0.31794052 | 0.062921236 | 0.261007452 | NS | 1.201202757 |
| SCARA3 | 0.996645816 | 0.024764143 | NA | Up | 1.606176695 |
| CCDC25 | -0.12963879 | 0.116915789 | 0.336721001 | NS | 0.932126834 |
| ESCO2 | -0.02687284 | 0.85396666 | 0.917634516 | NS | 0.068559085 |
| PBK | 0.178372044 | 0.418300273 | 0.618901466 | NS | 0.378511852 |
| SCARA5 | 0.401021258 | 0.200069879 | 0.429790861 | NS | 0.69881829 |
| NUGGC | -0.082522958 | 0.350868124 | 0.56491456 | NS | 0.454856086 |
| ELP3 | -0.159171596 | 0.010621975 | 0.127927347 | Down | 1.973794728 |
| PNOC | 0.251662979 | 0.057940214 | 0.251983816 | NS | 1.237019907 |
| ZNF395 | 0.232382954 | 0.016024592 | 0.149977606 | Up | 1.795213025 |
| FBXO16 | 0.808270927 | 0.052890826 | NA | NS | 1.27661965 |
| FZD3 | 0.012217649 | 0.860927261 | 0.921528262 | NS | 0.06503354 |
| EXTL3 | -0.053397923 | 0.524448673 | 0.699823783 | NS | 0.280297009 |
| INTS9 | -0.110012205 | 0.016496147 | 0.151603032 | Down | 1.782617485 |
| HMBOX1 | 0.156885876 | 0.034376049 | 0.203549976 | Up | 1.463744042 |
| KIF13B | -0.004025094 | 0.957826732 | 0.976662809 | NS | 0.018713046 |
| DUSP4 | 0.269418559 | 0.094356124 | 0.307427263 | NS | 1.025229908 |
| SARAF | -0.250194123 | 0.001556181 | 0.062836479 | Down | 2.807939789 |
| LEPROTL1 | -0.093143418 | 0.178949133 | 0.408934497 | NS | 0.7472704 |
| MBOAT4 | -0.070934007 | 0.576824777 | 0.73751904 | NS | 0.238956093 |
| DCTN6 | -0.23626794 | 0.054529185 | 0.24676996 | NS | 1.263370994 |
| RBPMS | 0.168177629 | 0.068355952 | 0.268476763 | NS | 1.165223667 |
| GTF2E2 | -0.093442955 | 0.113871166 | 0.332423088 | NS | 0.943586233 |
| SMIM18 | 0.248640486 | 0.007858222 | 0.11472857 | Up | 2.104675698 |
| GSR | -0.287475845 | 1.81E-04 | 0.02205531 | Down | 3.742202387 |
| UBXN8 | -0.196845342 | 0.041323547 | 0.218970818 | Down | 1.383802407 |
| PPP2CB | -0.041444712 | 0.569849826 | 0.732121898 | NS | 0.24423958 |
| TEX15 | 0.596924871 | 0.138972124 | 0.363222438 | NS | 0.857072304 |
| PURG | 0.330729269 | 0.405776365 | NA | NS | 0.391713253 |
| WRN | -0.107687644 | 0.200757007 | 0.430497005 | NS | 0.697329288 |
| NRG1 | 0.320416151 | 0.057875039 | 0.251883322 | NS | 1.237508703 |
| FUT10 | 0.138415663 | 0.208293172 | 0.438046853 | NS | 0.681324966 |
| TTI2 | -0.110396864 | 0.054455434 | 0.246713818 | NS | 1.263958776 |
| MAK16 | -0.127293456 | 0.066727281 | 0.266279415 | NS | 1.175696571 |
| RNF122 | 0.240924227 | 0.009814556 | 0.124735275 | Up | 2.008129336 |
| DUSP26 | 1.145432758 | 0.019203034 | NA | Up | 1.716630144 |
| UNC5D | 0.376242396 | 0.389985758 | 0.597102142 | NS | 0.408951253 |
| KCNU1 | 0.255894535 | 0.57539484 | 0.736492546 | NS | 0.240034037 |
| ZNF703 | 0.77745228 | 0.002619905 | 0.078024785 | Up | 2.58171449 |
| ERLIN2 | -9.56E-04 | 0.981913792 | 0.989344024 | NS | 0.00792664 |
| PLPBP | -0.223490831 | 0.001334513 | 0.059461129 | Down | 2.874677139 |
| ADGRA2 | 0.254818188 | 0.080900958 | 0.286854583 | NS | 1.092046333 |
| BRF2 | -0.073562785 | 0.313657472 | 0.533319352 | NS | 0.503544363 |
| RAB11FIP1 | -0.063963441 | 0.47928124 | 0.666575513 | NS | 0.319409569 |
| GOT1L1 | 0.475794941 | 0.356357628 | NA | NS | 0.448113941 |
| ADRB3 | 0.513447785 | 0.356684092 | NA | NS | 0.447716259 |
| EIF4EBP1 | 0.064076716 | 0.500491269 | 0.682155034 | NS | 0.300603495 |
| ASH2L | -0.084934495 | 0.494031201 | 0.677438836 | NS | 0.306245622 |
| STAR | 0.15670383 | 0.190695935 | 0.420149522 | NS | 0.719658564 |
| LSM1 | -0.33457964 | 0.004910689 | 0.096834757 | Down | 2.30885761 |
| BAG4 | 0.054639059 | 0.279118492 | 0.503208948 | NS | 0.554211391 |
| DDHD2 | -0.113605379 | 0.019802838 | 0.163424633 | Down | 1.703272555 |
| PLPP5 | 0.022935964 | 0.769284408 | 0.865536277 | NS | 0.11391307 |
| NSD3 | -0.002076251 | 0.970070369 | 0.983124 | NS | 0.013196761 |
| LETM2 | 0.124221623 | 0.220518562 | 0.449602156 | NS | 0.656554848 |
| FGFR1 | 0.337343504 | 0.03528168 | 0.205555536 | Up | 1.452450747 |
| TACC1 | 0.042030063 | 0.445918279 | 0.639795791 | NS | 0.350744725 |
| PLEKHA2 | 0.0873195 | 0.077761765 | 0.282100419 | NS | 1.109233889 |
| HTRA4 | 0.38019224 | 0.016581872 | 0.151907012 | Up | 1.780366445 |
| TM2D2 | -0.092639558 | 0.140044473 | 0.364288325 | NS | 0.853734027 |
| ADAM9 | 0.048063185 | 0.529945569 | 0.70413711 | NS | 0.275768735 |
| ADAM32 | -0.373831509 | 0.187553478 | 0.417008111 | NS | 0.726874877 |
| ADAM18 | -1.036645059 | 0.261213462 | NA | NS | 0.583004444 |
| IDO1 | 0.194756483 | 0.355251163 | 0.568471362 | NS | 0.449464492 |
| IDO2 | 0.263357143 | 0.324007653 | 0.542570549 | NS | 0.489444732 |
| TCIM | 0.910910147 | 0.13032715 | NA | NS | 0.884965102 |
| ZMAT4 | 0.085111228 | 0.734263475 | 0.843123696 | NS | 0.134148074 |
| SFRP1 | 0.570426807 | 0.153534154 | NA | NS | 0.813795 |
| GOLGA7 | -0.143532887 | 0.078079315 | 0.282523416 | NS | 1.107464005 |
| GINS4 | 0.163539834 | 0.162039471 | 0.389682175 | NS | 0.790379183 |
| GPAT4 | -0.128849223 | 0.007489424 | 0.112918226 | Down | 2.125551589 |
| NKX6-3 | 0.453926597 | 0.161427496 | NA | NS | 0.79202249 |
| ANK1 | -0.17729494 | 0.439092906 | 0.63425454 | NS | 0.357443579 |
| KAT6A | 0.072904253 | 0.224688885 | 0.453017417 | NS | 0.648418411 |
| AP3M2 | -0.068639384 | 0.271050779 | 0.496290769 | NS | 0.566949341 |
| PLAT | 0.217650615 | 0.271839804 | 0.497139279 | NS | 0.565686951 |
| IKBKB | -0.034291619 | 0.608169811 | 0.759851448 | NS | 0.215975141 |
| POLB | 0.022474888 | 0.7606382 | 0.860168677 | NS | 0.118821868 |
| DKK4 | 0.20602257 | 0.194297309 | 0.423758145 | NS | 0.711533214 |
| VDAC3 | -0.180092254 | 0.013764145 | 0.141040921 | Down | 1.861250746 |
| SLC20A2 | -0.076605198 | 0.08732073 | 0.296859543 | NS | 1.058882644 |
| SMIM19 | -0.245058501 | 0.030320561 | 0.193534415 | Down | 1.518262772 |
| CHRNB3 | 0.533320258 | 0.167754706 | NA | NS | 0.775325287 |
| CHRNA6 | 0.104198558 | 0.778053533 | NA | NS | 0.108990521 |
| THAP1 | -0.180995757 | 0.04510033 | 0.227108128 | Down | 1.345820277 |
| RNF170 | -0.090911053 | 0.116444028 | 0.335996111 | NS | 0.93388278 |
| HOOK3 | 0.049899534 | 0.306625625 | 0.52763506 | NS | 0.513391554 |
| FNTA | -0.101177876 | 0.057722401 | 0.251741329 | NS | 1.23865561 |
| POMK | 0.127242398 | 0.02453863 | 0.177561993 | Up | 1.610149684 |
| HGSNAT | 0.050637155 | 0.441288279 | 0.635925671 | NS | 0.355277608 |
| SPIDR | -0.095587552 | 0.138027278 | 0.362607036 | NS | 0.860035076 |
| CEBPD | 0.245548911 | 0.040643814 | 0.217533215 | Up | 1.391005546 |
| PRKDC | 0.068288291 | 0.406170382 | 0.609654875 | NS | 0.391291749 |
| MCM4 | -0.145324041 | 0.131726851 | 0.354444963 | NS | 0.88032569 |
| UBE2V2 | -0.25411094 | 0.009316815 | 0.121858195 | Down | 2.030732524 |
| EFCAB1 | 0.345562519 | 0.334988569 | 0.551420126 | NS | 0.474970012 |
| SNAI2 | 0.649268035 | 0.251776149 | NA | NS | 0.598985413 |
| PPDPFL | 0.393287364 | 0.438714248 | NA | NS | 0.35781826 |
| SNTG1 | 0.424507917 | 0.363851849 | 0.576035881 | NS | 0.439075413 |
| PXDNL | -0.357734453 | 0.11961037 | 0.34007656 | NS | 0.922231167 |
| PCMTD1 | -0.230964525 | 0.051554896 | 0.239738115 | NS | 1.287730089 |
| ST18 | 0.267136666 | 0.375598835 | 0.586154335 | NS | 0.425275763 |
| ALKAL1 | 1.071978897 | 0.045092579 | NA | Up | 1.34589493 |
| RB1CC1 | -0.080372983 | 0.18507001 | 0.415333011 | NS | 0.73266395 |
| NPBWR1 | 0.793365994 | 0.028051475 | NA | Up | 1.552044301 |
| OPRK1 | -0.086702435 | 0.854894202 | NA | NS | 0.068087629 |
| ATP6V1H | -0.008953551 | 0.802569276 | 0.886530727 | NS | 0.09551747 |
| RGS20 | 0.238931935 | 0.111211032 | 0.32972609 | NS | 0.953852128 |
| TCEA1 | -0.25200358 | 0.011627986 | 0.132226481 | Down | 1.934495505 |
| LYPLA1 | -0.107707875 | 0.281957459 | 0.504917658 | NS | 0.549816413 |
| MRPL15 | -0.315180292 | 0.010775147 | 0.12826482 | Down | 1.967576785 |
| SOX17 | 0.877711566 | 0.077664495 | NA | NS | 1.10977748 |
| RP1 | 0.184227857 | 0.673712954 | 0.805795597 | NS | 0.171525102 |
| XKR4 | 0.458788258 | 0.27660368 | 0.500969068 | NS | 0.558142046 |
| TMEM68 | -0.073034909 | 0.248705324 | 0.476241582 | NS | 0.604314919 |
| TGS1 | -0.139862382 | 0.030278427 | 0.193469884 | Down | 1.518866685 |
| LYN | 0.140227037 | 0.133842176 | 0.357214022 | NS | 0.873407011 |
| RPS20 | -0.418317261 | 0.012459394 | 0.135362928 | Down | 1.904503063 |
| MOS | 0.979749814 | 0.07548038 | NA | NS | 1.122165921 |
| PLAG1 | -0.062326041 | 0.528772225 | 0.70345289 | NS | 0.276731365 |
| CHCHD7 | -0.084346498 | 0.205991497 | 0.43596473 | NS | 0.686150707 |
| SDR16C5 | -0.024204323 | 0.930072543 | NA | NS | 0.031483177 |
| PENK | 0.663202311 | 0.143209379 | NA | NS | 0.844028538 |
| BPNT2 | 0.04155037 | 0.417831716 | 0.618692258 | NS | 0.378998598 |
| FAM110B | 0.524394156 | 0.012784506 | 0.136078062 | Up | 1.893316058 |
| UBXN2B | 0.261353433 | 0.008421952 | 0.117652418 | Up | 2.074587254 |
| CYP7A1 | 0.366807003 | 0.321736718 | NA | NS | 0.492499373 |
| SDCBP | 0.101438598 | 0.318104392 | 0.537417143 | NS | 0.497430334 |
| NSMAF | 0.018898988 | 0.714832442 | 0.831357536 | NS | 0.145795746 |
| TOX | 0.128458265 | 0.206065767 | 0.43596473 | NS | 0.68599415 |
| CA8 | 0.0665149 | 0.726923637 | 0.838611255 | NS | 0.138511209 |
| RAB2A | -0.062805963 | 0.336889972 | 0.55308503 | NS | 0.472511916 |
| CHD7 | 0.108746585 | 0.138979924 | 0.363222438 | NS | 0.857047929 |
| CLVS1 | 0.592706459 | 0.013646399 | 0.140273711 | Up | 1.864981937 |
| ASPH | 0.036217678 | 0.714422811 | 0.831115051 | NS | 0.146044687 |
| NKAIN3 | 0.373701464 | 0.351178454 | 0.565113052 | NS | 0.454472137 |
| GGH | 0.231963432 | 0.163848993 | 0.391619419 | NS | 0.785556225 |
| TTPA | 0.543065116 | 0.260282589 | NA | NS | 0.584554882 |
| YTHDF3 | 0.024924722 | 0.643240443 | 0.784127485 | NS | 0.191626658 |
| BHLHE22 | 0.809371878 | 0.100129049 | NA | NS | 0.999439907 |
| CYP7B1 | 0.394719928 | 0.048203447 | 0.23309868 | Up | 1.316921903 |
| ARMC1 | -0.165458585 | 0.021309879 | 0.168379028 | Down | 1.67141902 |
| MTFR1 | 0.09753244 | 0.175311517 | 0.40462625 | NS | 0.756189552 |
| PDE7A | 0.052523119 | 0.338430954 | 0.55429777 | NS | 0.470529921 |
| DNAJC5B | 0.262536585 | 0.30297968 | 0.524319378 | NS | 0.518586497 |
| TRIM55 | 0.263394194 | 0.577972124 | NA | NS | 0.238093108 |
| CRH | 0.892339895 | 0.064220129 | NA | NS | 1.192328824 |
| RRS1 | -0.052666177 | 0.485971028 | 0.671550961 | NS | 0.313389621 |
| ADHFE1 | -8.24E-05 | 0.999303023 | 0.999609713 | NS | 3.03E-04 |
| VXN | 0.828385841 | 0.048430143 | NA | Up | 1.31488425 |
| MYBL1 | -0.212005612 | 0.026651426 | 0.184430741 | Down | 1.574279551 |
| VCPIP1 | 0.048126634 | 0.490296131 | 0.674680296 | NS | 0.309541534 |
| C8orf44-SGK3 | 0.266884212 | 0.222977945 | NA | NS | 0.65173809 |
| SGK3 | -0.166897638 | 0.048578553 | 0.233728157 | Down | 1.313555424 |
| MCMDC2 | 0.26462131 | 0.050578072 | 0.238096402 | NS | 1.296037734 |
| TCF24 | 1.158331063 | 0.025409947 | NA | Up | 1.594996238 |
| PPP1R42 | 0.974894313 | 0.094590908 | NA | NS | 1.024150605 |
| COPS5 | -0.085501309 | 0.104852236 | 0.32076633 | NS | 0.979422303 |
| CSPP1 | 0.030213617 | 0.714943818 | 0.831377499 | NS | 0.145728085 |
| ARFGEF1 | 0.044531573 | 0.247885944 | 0.475985905 | NS | 0.605748099 |
| CPA6 | 0.203665755 | 0.633174811 | NA | NS | 0.198476371 |
| PREX2 | 0.576045737 | 0.118733083 | 0.339297335 | NS | 0.925428256 |
| C8orf34 | 0.400819755 | 0.357246625 | 0.570389167 | NS | 0.447031865 |
| SULF1 | 0.476523703 | 0.250350444 | 0.477425471 | NS | 0.601451634 |
| SLCO5A1 | 0.19306653 | 0.371549411 | 0.582584185 | NS | 0.429983423 |
| PRDM14 | 0.720868801 | 0.136745973 | NA | NS | 0.864085456 |
| NCOA2 | -0.039991792 | 0.605788846 | 0.758254148 | NS | 0.217678727 |
| TRAM1 | -0.16802256 | 0.066186608 | 0.2655108 | NS | 1.179229875 |
| LACTB2 | -0.200180236 | 0.020095655 | 0.164426826 | Down | 1.69689784 |
| XKR9 | 0.1012252 | 0.625358905 | 0.771491578 | NS | 0.203870661 |
| EYA1 | 0.607630409 | 0.156924657 | 0.384159619 | NS | 0.804308811 |
| MSC | 0.276241932 | 0.090252168 | 0.300982388 | NS | 1.044542359 |
| TRPA1 | 0.204651093 | 0.200307411 | 0.429960996 | NS | 0.698302983 |
| KCNB2 | 0.507857168 | 0.312142013 | NA | NS | 0.505647774 |
| TERF1 | 0.018681967 | 0.64935385 | 0.78854332 | NS | 0.18751858 |
| SBSPON | 0.245027386 | 0.016747776 | 0.152507982 | Up | 1.776042846 |
| C8orf89 | 0.777134335 | 0.059778678 | NA | NS | 1.223453697 |
| RPL7 | -0.647808481 | 0.007528737 | 0.11304493 | Down | 2.123277876 |
| RDH10 | -0.159711638 | 0.04990724 | 0.23659193 | Down | 1.301836445 |
| STAU2 | -0.040186845 | 0.328504312 | 0.546240087 | NS | 0.483458926 |
| UBE2W | 0.056797341 | 0.482988402 | 0.669171227 | NS | 0.316063298 |
| ELOC | -0.041259892 | 0.593036842 | 0.749460862 | NS | 0.226918326 |
| TMEM70 | -0.094487861 | 0.347234608 | 0.561735573 | NS | 0.459376996 |
| LY96 | 0.122608311 | 0.468043432 | 0.657696078 | NS | 0.329713845 |
| JPH1 | 0.736674555 | 0.095690178 | NA | NS | 1.019132636 |
| GDAP1 | 0.302117263 | 0.003770243 | 0.088644036 | Up | 2.423630671 |
| PI15 | 0.495337231 | 0.249334541 | 0.476497659 | NS | 0.603217553 |
| CRISPLD1 | 0.646910444 | 0.075983893 | NA | NS | 1.119278458 |
| HNF4G | 0.161609372 | 0.746608173 | NA | NS | 0.12690726 |
| ZFHX4 | 0.250637704 | 0.496455157 | 0.679119341 | NS | 0.304119973 |
| PEX2 | -0.05950889 | 0.395723654 | 0.601183351 | NS | 0.40260799 |
| PKIA | -0.204671026 | 0.067575928 | 0.267518048 | NS | 1.170207981 |
| ZC2HC1A | 0.058735528 | 0.556374961 | 0.722341233 | NS | 0.254632423 |
| IL7 | -0.074394395 | 0.439914921 | 0.635045158 | NS | 0.356631308 |
| STMN2 | 0.58194218 | 0.230496679 | NA | NS | 0.637335327 |
| HEY1 | 0.520986577 | 0.031111391 | 0.1959427 | Up | 1.507080567 |
| MRPS28 | -0.036187022 | 0.659015907 | 0.794900842 | NS | 0.181104103 |
| TPD52 | 0.031967835 | 0.672737181 | 0.805012822 | NS | 0.172154569 |
| ZBTB10 | -0.124405854 | 0.08908986 | 0.299430914 | NS | 1.050171725 |
| ZNF704 | 0.389748632 | 0.200459366 | 0.430173773 | NS | 0.697973648 |
| PAG1 | -0.011200199 | 0.83668303 | 0.908224228 | NS | 0.077439039 |
| FABP5 | 0.066781357 | 0.5806391 | 0.740040997 | NS | 0.236093722 |
| PMP2 | 0.382398624 | 0.467462503 | NA | NS | 0.33025322 |
| FABP9 | 0.819979688 | 0.161647125 | NA | NS | 0.791432015 |
| FABP4 | 0.019264913 | 0.970927928 | NA | NS | 0.012813007 |
| FABP12 | 0.632125975 | 0.30321479 | NA | NS | 0.518249619 |
| IMPA1 | -0.162189164 | 0.029762194 | 0.192220466 | Down | 1.526335052 |
| SLC10A5 | 0.062063085 | 0.743728861 | NA | NS | 0.128585365 |
| ZFAND1 | -0.308080834 | 0.004553209 | 0.094415114 | Down | 2.341682453 |
| CHMP4C | 0.548229708 | 0.150997819 | NA | NS | 0.821029326 |
| SNX16 | -0.126307414 | 0.204585675 | 0.434761171 | NS | 0.689124778 |
| RALYL | 0.598454353 | 0.255460984 | NA | NS | 0.592675419 |
| LRRCC1 | -0.236595761 | 0.119363988 | 0.340007095 | NS | 0.923126679 |
| E2F5 | -0.050723398 | 0.478328803 | 0.665963078 | NS | 0.320273467 |
| RBIS | -0.125835684 | 0.306533564 | 0.52760544 | NS | 0.513521965 |
| CA13 | 0.065601097 | 0.330672295 | 0.547740264 | NS | 0.480602191 |
| CA1 | -0.266127506 | 0.231433505 | 0.45979109 | NS | 0.635573767 |
| CA3 | -0.048769152 | 0.826837755 | 0.901425157 | NS | 0.082579701 |
| CA2 | -0.224235238 | 0.233323307 | 0.461567796 | NS | 0.632041877 |
| ATP6V0D2 | 0.852012655 | 0.088279128 | NA | NS | 1.054141964 |
| PSKH2 | 0.033577895 | 0.944733619 | NA | NS | 0.02469063 |
| SLC7A13 | 0.211585826 | 0.686546833 | NA | NS | 0.163329832 |
| WWP1 | 0.062050426 | 0.225748902 | 0.454394565 | NS | 0.646374353 |
| RMDN1 | 0.02515656 | 0.570014209 | 0.732162951 | NS | 0.244114318 |
| CPNE3 | -0.1083483 | 0.175025925 | 0.404321539 | NS | 0.756897618 |
| CNGB3 | 0.418304564 | 0.073388109 | 0.275575419 | NS | 1.1343743 |
| CNBD1 | 0.443855425 | 0.391766242 | NA | NS | 0.406972989 |
| DCAF4L2 | 0.320061191 | 0.538596549 | NA | NS | 0.268736434 |
| MMP16 | 0.413245678 | 0.352762604 | 0.566388752 | NS | 0.45251746 |
| RIPK2 | 0.054193531 | 0.457341257 | 0.648908337 | NS | 0.339759619 |
| OSGIN2 | 0.035653933 | 0.729927928 | 0.840372591 | NS | 0.136720019 |
| NBN | -0.099667098 | 0.242080594 | 0.469918053 | NS | 0.616040024 |
| DECR1 | 0.05670095 | 0.273643382 | 0.498725191 | NS | 0.56281505 |
| CALB1 | 0.13786329 | 0.174646281 | 0.404024697 | NS | 0.757840657 |
| TMEM64 | -0.103878577 | 0.150816153 | 0.377320464 | NS | 0.821552142 |
| NECAB1 | 0.126975875 | 0.356546642 | 0.569628963 | NS | 0.44788365 |
| C8orf88 | -0.230112436 | 0.202001282 | 0.43211595 | NS | 0.694645874 |
| PIP4P2 | 0.099785481 | 0.325767279 | 0.544181052 | NS | 0.48709254 |
| OTUD6B | 0.022024104 | 0.724953402 | 0.838063844 | NS | 0.139689908 |
| LRRC69 | 0.074019293 | 0.32253518 | 0.541229534 | NS | 0.491422909 |
| SLC26A7 | 0.321887336 | 0.187916915 | 0.417308021 | NS | 0.726034125 |
| RUNX1T1 | 0.3040299 | 0.39832199 | 0.603331363 | NS | 0.399765717 |
| TRIQK | -0.073916769 | 0.417993572 | 0.618803411 | NS | 0.378830397 |
| CIBAR1 | 0.118202189 | 0.139777239 | 0.36397707 | NS | 0.854563542 |
| RBM12B | -0.078893997 | 0.160723045 | 0.388565309 | NS | 0.793921849 |
| TMEM67 | 0.065965765 | 0.449289422 | 0.642780256 | NS | 0.347473807 |
| PDP1 | -0.164233644 | 0.039900339 | 0.215796828 | Down | 1.399023418 |
| CDH17 | 0.576202603 | 0.208514151 | 0.438346984 | NS | 0.680864465 |
| GEM | -0.092770382 | 0.766213505 | NA | NS | 0.115650197 |
| RAD54B | 0.05661215 | 0.680351316 | 0.809591907 | NS | 0.167266771 |
| FSBP | -0.044873557 | 0.703528116 | 0.824428133 | NS | 0.152718542 |
| VIRMA | 0.008594871 | 0.8327992 | 0.905027323 | NS | 0.0794597 |
| ESRP1 | 0.409424502 | 0.352825447 | 0.566410233 | NS | 0.4524401 |
| DPY19L4 | -0.149125696 | 0.020224316 | 0.164714539 | Down | 1.694126158 |
| INTS8 | -0.076291419 | 0.062371767 | 0.259501987 | NS | 1.20501195 |
| CCNE2 | -0.16404983 | 0.168538113 | 0.396737575 | NS | 0.773301872 |
| NDUFAF6 | 0.078542841 | 0.170611186 | 0.399000229 | NS | 0.767992499 |
| TP53INP1 | 0.137336915 | 0.122217198 | 0.342932892 | NS | 0.912867678 |
| PLEKHF2 | -0.246945656 | 0.017877403 | 0.156161405 | Down | 1.747695559 |
| CFAP418 | -0.09281194 | 0.305613733 | 0.526741519 | NS | 0.514827135 |
| GDF6 | 0.882116032 | 0.071418456 | NA | NS | 1.146189542 |
| UQCRB | -0.783005157 | 5.59E-04 | 0.040125454 | Down | 3.252843679 |
| MTERF3 | -0.10627961 | 0.160360011 | 0.388015316 | NS | 0.794903923 |
| PTDSS1 | -0.1414507 | 0.06578098 | 0.265127437 | NS | 1.181899659 |
| SDC2 | -0.186144374 | 0.279961054 | 0.50348007 | NS | 0.55290238 |
| CPQ | 0.010960527 | 0.898876358 | 0.943838721 | NS | 0.046300042 |
| TSPYL5 | -0.09776296 | 0.478703354 | 0.666135874 | NS | 0.31993353 |
| MTDH | -0.141466498 | 0.026398298 | 0.183204185 | Down | 1.578424079 |
| LAPTM4B | -0.018611755 | 0.869615437 | 0.926494824 | NS | 0.06067276 |
| MATN2 | 0.145432722 | 0.097188576 | 0.311563936 | NS | 1.012384782 |
| RPL30 | -0.395312634 | 0.014512312 | 0.144329895 | Down | 1.838263393 |
| ERICH5 | 0.502299939 | 0.234000287 | NA | NS | 0.63078361 |
| RIDA | -0.282742939 | 0.088738316 | 0.298997339 | NS | 1.051888817 |
| POP1 | -0.138926268 | 0.014215994 | 0.143172688 | Down | 1.847222772 |
| NIPAL2 | -0.16145465 | 0.035486373 | 0.206049964 | Down | 1.449938382 |
| STK3 | 0.045093837 | 0.578042996 | 0.738214279 | NS | 0.238039857 |
| KCNS2 | 0.492310969 | 0.018152195 | 0.156913557 | Up | 1.741070851 |
| OSR2 | 0.974761437 | 0.017612514 | NA | Up | 1.754178656 |
| VPS13B | 0.009656416 | 0.898337202 | 0.943418999 | NS | 0.046560615 |
| COX6C | -0.336827646 | 0.062232483 | 0.259136057 | NS | 1.205982874 |
| RGS22 | 0.324145872 | 0.435739383 | 0.631641456 | NS | 0.360773185 |
| FBXO43 | -0.029538519 | 0.911645376 | 0.951011314 | NS | 0.040174067 |
| POLR2K | -0.347518462 | 0.071677856 | 0.272957474 | NS | 1.144614996 |
| SPAG1 | 0.088104607 | 0.133169374 | 0.356407673 | NS | 0.875595643 |
| RNF19A | -0.154744145 | 0.040732219 | 0.217802775 | Down | 1.390061927 |
| ANKRD46 | -0.115785933 | 0.10318439 | 0.319144166 | NS | 0.986386 |
| SNX31 | 0.64612602 | 0.124673648 | NA | NS | 0.904225332 |
| PABPC1 | 0.053548545 | 0.548416764 | 0.716517812 | NS | 0.260889278 |
| YWHAZ | -0.003312388 | 0.956035692 | 0.976096862 | NS | 0.019525894 |
| ZNF706 | 0.112705327 | 0.045929004 | 0.228673646 | Up | 1.33791297 |
| GRHL2 | 0.335297028 | 0.1807522 | 0.410591835 | NS | 0.742916409 |
| NCALD | -0.207030083 | 0.014795497 | 0.145877179 | Down | 1.829870432 |
| RRM2B | -0.035274669 | 0.735144255 | 0.843792643 | NS | 0.133627432 |
| UBR5 | 0.058957904 | 0.202791436 | 0.432901167 | NS | 0.69295039 |
| ODF1 | 0.318362539 | 0.686971382 | NA | NS | 0.163061355 |
| KLF10 | 0.085745378 | 0.443768093 | 0.637917821 | NS | 0.352843927 |
| AZIN1 | -0.218961785 | 0.00149443 | 0.062296149 | Down | 2.82552441 |
| ATP6V1C1 | 0.051353719 | 0.380174655 | 0.590088781 | NS | 0.420016839 |
| BAALC | 0.351742491 | 0.141313775 | 0.365773968 | NS | 0.849815502 |
| FZD6 | -0.137795697 | 0.25259929 | 0.479568013 | NS | 0.597567874 |
| CTHRC1 | 0.341555812 | 0.007865113 | 0.11472857 | Up | 2.104295022 |
| SLC25A32 | 0.034578193 | 0.752396607 | 0.854731503 | NS | 0.123553171 |
| DCAF13 | -0.124368682 | 0.091770188 | 0.303585274 | NS | 1.037298378 |
| RIMS2 | 0.414471612 | 0.336269574 | 0.552487676 | NS | 0.473312427 |
| DPYS | 0.259865906 | 0.507019866 | NA | NS | 0.294975023 |
| DCSTAMP | -0.23196116 | 0.496886137 | 0.6793437 | NS | 0.30374312 |
| LRP12 | 0.124206348 | 0.195344603 | 0.425062433 | NS | 0.709198582 |
| ZFPM2 | 0.320391497 | 0.419958004 | 0.62016266 | NS | 0.376794137 |
| OXR1 | -0.099732255 | 0.078824239 | 0.283646978 | NS | 1.103340216 |
| ABRA | 0.228097997 | 0.07442958 | 0.277058381 | NS | 1.128254433 |
| ANGPT1 | -0.189369176 | 0.124910048 | 0.346400199 | NS | 0.903402626 |
| RSPO2 | 0.528453385 | 0.209814512 | 0.439792418 | NS | 0.678164476 |
| EIF3E | -0.289309457 | 0.016939224 | 0.153154431 | Down | 1.771106484 |
| EMC2 | -0.042082327 | 0.468424784 | 0.657909015 | NS | 0.329360135 |
| TMEM74 | 0.491610837 | 0.272913469 | NA | NS | 0.56397503 |
| TRHR | 0.134456738 | 0.633225885 | NA | NS | 0.19844134 |
| NUDCD1 | -0.14470069 | 0.09800591 | 0.312744522 | NS | 1.008747736 |
| ENY2 | -0.055991549 | 0.498935481 | 0.680723244 | NS | 0.301955611 |
| PKHD1L1 | -0.061149383 | 0.635186345 | 0.777500677 | NS | 0.197098847 |
| EBAG9 | -0.155676018 | 0.045310517 | 0.227416493 | Down | 1.343800984 |
| SYBU | 0.307719153 | 0.137400224 | 0.362193823 | NS | 0.862012559 |
| KCNV1 | 0.108880183 | 0.826124099 | 0.900947339 | NS | 0.082954709 |
| CSMD3 | 0.313703938 | 0.481629464 | 0.668380172 | NS | 0.317286953 |
| TRPS1 | 0.043522782 | 0.43717379 | 0.632879529 | NS | 0.359345883 |
| EIF3H | -0.224999164 | 0.009964893 | 0.12546233 | Down | 2.001527364 |
| UTP23 | -0.183038444 | 0.014752974 | 0.145697549 | Down | 1.831120417 |
| RAD21 | 0.058404035 | 0.250094826 | 0.477107181 | NS | 0.601895293 |
| AARD | 0.71762855 | 0.117128342 | NA | NS | 0.931338003 |
| SLC30A8 | 0.466005769 | 0.240825319 | 0.468716025 | NS | 0.618297855 |
| MED30 | -0.019126447 | 0.812425995 | 0.892507557 | NS | 0.090216189 |
| EXT1 | 0.036414337 | 0.618949189 | 0.766999754 | NS | 0.208345002 |
| SAMD12 | 0.096064808 | 0.419432588 | 0.619675889 | NS | 0.377337829 |
| TNFRSF11B | 0.310165732 | 0.483268827 | NA | NS | 0.315811218 |
| COLEC10 | 0.584963499 | 0.201558734 | NA | NS | 0.695598378 |
| MAL2 | 0.49417032 | 0.150361678 | NA | NS | 0.822862836 |
| CCN3 | -0.302350838 | 0.072963829 | 0.274846078 | NS | 1.136892384 |
| ENPP2 | 0.085574478 | 0.550865095 | 0.718323316 | NS | 0.258954746 |
| TAF2 | -0.129746581 | 0.011146474 | 0.129586317 | Down | 1.9528625 |
| DSCC1 | 0.052305893 | 0.688682394 | 0.815257617 | NS | 0.16198102 |
| DEPTOR | 0.001651945 | 0.990537126 | 0.994488679 | NS | 0.004129242 |
| COL14A1 | 0.448993918 | 0.237666599 | 0.465654928 | NS | 0.624031848 |
| MRPL13 | -0.193435315 | 0.108018721 | 0.3257678 | NS | 0.966500968 |
| MTBP | -0.12076834 | 0.163259767 | 0.391057496 | NS | 0.787120828 |
| SNTB1 | 0.087184001 | 0.065922053 | 0.265127437 | NS | 1.180969277 |
| HAS2 | 0.726550847 | 0.104277108 | NA | NS | 0.981811024 |
| ZHX2 | 0.200287017 | 0.075168898 | 0.278166277 | NS | 1.123961815 |
| DERL1 | -0.096172663 | 0.027873043 | 0.187065309 | NS | 1.554815614 |
| TBC1D31 | -0.346804507 | 6.81E-04 | 0.042942134 | Down | 3.167083161 |
| FAM83A | -0.201769118 | 0.574641635 | 0.735905724 | NS | 0.240602911 |
| C8orf76 | 8.04E-04 | 0.990080985 | 0.994124998 | NS | 0.00432928 |
| ZHX1-C8orf76 | 0.126147075 | 0.513362555 | NA | NS | 0.289575812 |
| ZHX1 | -0.198621301 | 0.003776594 | 0.088644036 | Down | 2.422899678 |
| ATAD2 | -0.21477275 | 0.0406426 | 0.217533215 | Down | 1.391018521 |
| NTAQ1 | 0.058177445 | 0.40066329 | 0.605476125 | NS | 0.397220447 |
| FBXO32 | 0.191698977 | 0.003915212 | 0.089796534 | Up | 2.407244701 |
| KLHL38 | 0.373934362 | 0.483242435 | NA | NS | 0.315834936 |
| ANXA13 | 0.42583121 | 0.376042296 | NA | NS | 0.424763304 |
| FAM91A1 | -0.051150219 | 0.420531962 | 0.620581631 | NS | 0.376200991 |
| FER1L6 | 0.262544803 | 0.553625368 | 0.720609728 | NS | 0.256784018 |
| TMEM65 | 0.079416076 | 0.080221595 | 0.285870705 | NS | 1.095708708 |
| TRMT12 | -0.100969382 | 0.221457267 | 0.450227637 | NS | 0.654710064 |
| RNF139 | -0.23276852 | 0.005015002 | 0.097847413 | Down | 2.299728881 |
| TATDN1 | -0.087602151 | 0.394155301 | 0.600085288 | NS | 0.404332628 |
| NDUFB9 | -0.036157248 | 0.49404018 | 0.677438836 | NS | 0.306237729 |
| MTSS1 | 0.102518294 | 0.19901033 | 0.428784342 | NS | 0.701124381 |
| ZNF572 | -0.143745207 | 0.120881802 | 0.341402767 | NS | 0.917639073 |
| SQLE | -0.048399104 | 0.554647148 | 0.721447662 | NS | 0.255983216 |
| WASHC5 | -0.013189555 | 0.78229607 | 0.874141176 | NS | 0.106628851 |
| NSMCE2 | -0.097573098 | 0.207409123 | 0.437312072 | NS | 0.683172146 |
| TRIB1 | 0.126140144 | 0.147126202 | 0.372664177 | NS | 0.832309977 |
| LRATD2 | 0.018728668 | 0.839855274 | 0.909641309 | NS | 0.075795546 |
| POU5F1B | 0.174509695 | 0.285505322 | 0.508645888 | NS | 0.544385791 |
| MYC | 0.012999865 | 0.882605916 | 0.93355998 | NS | 0.054233166 |
| CYRIB | 0.009818836 | 0.903750278 | 0.946526221 | NS | 0.043951556 |
| ASAP1 | -0.015828977 | 0.914469711 | 0.952222667 | NS | 0.038830674 |
| ADCY8 | 0.478064893 | 0.326032047 | NA | NS | 0.486739709 |
| EFR3A | -0.11709851 | 0.031986795 | 0.198142899 | Down | 1.495029274 |
| HHLA1 | 0.568006836 | 0.215872384 | 0.445318606 | NS | 0.665802913 |
| KCNQ3 | 0.254579173 | 0.434045413 | 0.63047823 | NS | 0.36246483 |
| DNAAF11 | 0.078931768 | 0.543406143 | 0.713114152 | NS | 0.264875457 |
| TMEM71 | 0.040710584 | 0.591771534 | 0.748596185 | NS | 0.22784593 |
| PHF20L1 | 0.022640682 | 0.758322004 | 0.858906555 | NS | 0.120146342 |
| TG | 0.182548915 | 0.165993228 | 0.393986621 | NS | 0.779909629 |
| SLA | 0.071594745 | 0.431519901 | 0.628468602 | NS | 0.364999171 |
| CCN4 | 0.463887009 | 0.081851936 | 0.288440228 | NS | 1.086971044 |
| NDRG1 | 0.039992857 | 0.421469173 | 0.621298082 | NS | 0.375234185 |
| ST3GAL1 | -0.162386713 | 0.156089474 | 0.383229102 | NS | 0.806626382 |
| ZFAT | -0.020609575 | 0.718203383 | 0.833717613 | NS | 0.143752554 |
| KHDRBS3 | 0.413531741 | 0.139697161 | 0.363934068 | NS | 0.854812418 |
| FAM135B | 0.363070516 | 0.384871569 | 0.593309885 | NS | 0.414684169 |
| COL22A1 | 0.41674041 | 0.27909161 | 0.503208948 | NS | 0.554253219 |
| KCNK9 | 0.890979884 | 0.039297736 | 0.214674614 | Up | 1.405632468 |
| TRAPPC9 | 0.075353879 | 0.363952909 | 0.576120873 | NS | 0.438954805 |
| CHRAC1 | -0.054243692 | 0.425922037 | 0.624166015 | NS | 0.370669889 |
| AGO2 | -0.124117875 | 0.380818741 | 0.590688169 | NS | 0.419281687 |
| PTK2 | -0.082739235 | 0.432807438 | 0.629383196 | NS | 0.363705284 |
| DENND3 | 0.053960151 | 0.640955991 | 0.782522144 | NS | 0.193171789 |
| SLC45A4 | -0.086809071 | 0.44798056 | 0.641635461 | NS | 0.348740832 |
| PTP4A3 | 0.047190631 | 0.687264311 | 0.814178911 | NS | 0.162876208 |
| TSNARE1 | 0.029676652 | 0.792664847 | 0.880521472 | NS | 0.100910401 |
| ADGRB1 | 0.467305469 | 0.080390488 | 0.286107685 | NS | 1.094795335 |
| ARC | 0.93410111 | 0.02586863 | NA | Up | 1.587226565 |
| JRK | 0.08919974 | 0.178575312 | 0.408483 | NS | 0.748178583 |
| PSCA | 0.573877358 | 0.094554536 | NA | NS | 1.02431763 |
| LY6K | 1.145493233 | 0.019058085 | NA | Up | 1.719920733 |
| THEM6 | -0.11021458 | 0.137115097 | 0.361608699 | NS | 0.862914726 |
| SLURP1 | 0.841195404 | 0.035454565 | NA | Up | 1.450327839 |
| LYPD2 | 0.073182922 | 0.755983132 | 0.857317439 | NS | 0.121487895 |
| LYNX1-SLURP2 | 0.679301947 | 0.435123843 | NA | NS | 0.361387118 |
| SLURP2 | 1.577477108 | 0.019805906 | NA | Up | 1.703205279 |
| LYNX1 | 0.195118863 | 0.278149034 | 0.502458526 | NS | 0.555722443 |
| LY6D | 1.367534606 | 0.026406324 | NA | Up | 1.578292047 |
| GML | 0.262020318 | 0.247913865 | 0.47599961 | NS | 0.605699183 |
| CYP11B1 | 0.475782222 | 0.027633967 | 0.186639939 | Up | 1.558556759 |
| CYP11B2 | 0.785774773 | 0.100711547 | NA | NS | 0.996920734 |
| LY6E | 0.319699877 | 0.01224479 | 0.134364821 | Up | 1.912048649 |
| LY6L | 0.102783253 | 0.888122153 | NA | NS | 0.051527297 |
| GPIHBP1 | 0.943355168 | 0.057965546 | NA | NS | 1.23683007 |
| ZFP41 | 0.158697879 | 0.2776401 | 0.50198456 | NS | 0.556517808 |
| GLI4 | 0.202570104 | 0.085557287 | 0.293993175 | NS | 1.067742995 |
| ZNF696 | 0.049650528 | 0.431125013 | 0.628173117 | NS | 0.36539678 |
| TOP1MT | -0.151911459 | 0.069573037 | 0.270000522 | NS | 1.157559036 |
| RHPN1 | 0.142021982 | 0.340327556 | 0.556026263 | NS | 0.468102886 |
| MAFA | 0.532973142 | 0.105192594 | NA | NS | 0.978014835 |
| ZC3H3 | 0.108122717 | 0.480204813 | 0.667051596 | NS | 0.318573492 |
| GSDMD | -0.165562073 | 0.059219792 | 0.253884625 | NS | 1.227533125 |
| MROH6 | -0.262790008 | 0.384045704 | NA | NS | 0.415617089 |
| NAPRT | -0.135291532 | 0.25626305 | 0.483238894 | NS | 0.59131401 |
| EEF1D | -0.293082721 | 0.016192815 | 0.150567543 | Down | 1.790677634 |
| PYCR3 | 0.092609114 | 0.630250975 | NA | NS | 0.200486474 |
| GFUS | -0.111514462 | 0.467508802 | 0.657227094 | NS | 0.330210208 |
| ZNF623 | 0.096456269 | 0.608284124 | 0.759851448 | NS | 0.215893518 |
| ZNF707 | -0.149451453 | 0.152343313 | 0.378947167 | NS | 0.817176603 |
| SCRIB | -0.142735222 | 0.324256664 | 0.542845477 | NS | 0.489111089 |
| PUF60 | -0.212487353 | 0.014407688 | 0.143935548 | Down | 1.841405707 |
| NRBP2 | 0.09446325 | 0.432278578 | 0.629114552 | NS | 0.364236286 |
| EPPK1 | 0.053384834 | 0.678011908 | 0.807899517 | NS | 0.168762678 |
| PLEC | -0.040085127 | 0.773707642 | 0.868056059 | NS | 0.111423113 |
| PARP10 | 0.014263636 | 0.881287181 | 0.932827018 | NS | 0.054882547 |
| GRINA | -0.054669522 | 0.821025049 | 0.89803781 | NS | 0.085643593 |
| SPATC1 | 0.490706895 | 0.175958849 | NA | NS | 0.754588888 |
| OPLAH | 0.198168771 | 0.186741318 | 0.416311011 | NS | 0.72875958 |
| EXOSC4 | -0.176471459 | 0.081976393 | 0.28847931 | NS | 1.086311195 |
| GPAA1 | -0.062338297 | 0.348490728 | 0.56264736 | NS | 0.457808772 |
| CYC1 | -0.206130542 | 0.012472228 | 0.135438108 | Down | 1.904055952 |
| SHARPIN | -0.054550676 | 0.755401019 | 0.85689345 | NS | 0.121822434 |
| MAF1 | -0.116115469 | 0.397971718 | 0.603040114 | NS | 0.40014779 |
| WDR97 | 0.283403516 | 0.134749878 | 0.358300443 | NS | 0.870471618 |
| HGH1 | -0.151387413 | 0.064922508 | 0.264064926 | NS | 1.187604715 |
| MROH1 | -0.007667338 | 0.957826014 | 0.976662809 | NS | 0.018713372 |
| BOP1 | -0.017473133 | 0.866221193 | 0.924353124 | NS | 0.062371195 |
| SCX | 0.594026014 | 0.082160451 | NA | NS | 1.085337184 |
| HSF1 | 0.180563411 | 0.238794369 | 0.466925155 | NS | 0.621975918 |
| DGAT1 | -0.170189791 | 0.010999141 | 0.129313304 | Down | 1.958641249 |
| SLC52A2 | -0.187293689 | 0.209269014 | 0.439211782 | NS | 0.679295072 |
| FBXL6 | -0.139931268 | 0.204651589 | 0.434843849 | NS | 0.688984878 |
| ADCK5 | -0.002097742 | 0.984472857 | 0.990614999 | NS | 0.006796253 |
| CPSF1 | 0.068872847 | 0.574984547 | 0.736180663 | NS | 0.240343827 |
| SLC39A4 | 0.466811827 | 0.0701932 | NA | NS | 1.153704956 |
| VPS28 | -0.081077368 | 0.452336909 | 0.645084992 | NS | 0.344537974 |
| TONSL | 0.222211082 | 0.18557386 | 0.415463304 | NS | 0.731483198 |
| CYHR1 | -0.031735032 | 0.65821467 | 0.794520233 | NS | 0.181632442 |
| KIFC2 | -0.127341206 | 0.372367595 | 0.583108558 | NS | 0.42902812 |
| FOXH1 | 0.583888967 | 0.224562658 | NA | NS | 0.64866246 |
| PPP1R16A | 0.39598075 | 0.031504674 | 0.197082776 | Up | 1.501625011 |
| GPT | 0.410013593 | 0.012083977 | 0.13408728 | Up | 1.917790119 |
| MFSD3 | 0.116176528 | 0.257925427 | 0.484730205 | NS | 0.588505842 |
| RECQL4 | 0.127801742 | 0.28012152 | 0.50348007 | NS | 0.552653526 |
| LRRC14 | 0.139864494 | 0.071324651 | 0.272521361 | NS | 1.146760346 |
| C8orf82 | 0.081521771 | 0.311760564 | 0.531443277 | NS | 0.506178822 |
| ARHGAP39 | 0.84287223 | 0.010828588 | 0.12856202 | Up | 1.965428186 |
| ZNF251 | -0.273732186 | 0.025336121 | 0.180089336 | Down | 1.596259878 |
| ZNF34 | -0.05287904 | 0.434786688 | 0.630833536 | NS | 0.361723761 |
| RPL8 | -0.145528267 | 0.202460583 | 0.432613573 | NS | 0.693659517 |
| ZNF517 | -0.049984563 | 0.478570051 | 0.666071166 | NS | 0.320054483 |
| ZNF7 | 0.007291228 | 0.848688914 | 0.914925321 | NS | 0.071251471 |
| COMMD5 | -0.117911782 | 0.060672951 | 0.256526216 | NS | 1.217004883 |
| ZNF250 | -0.103221517 | 0.008282669 | 0.117020166 | Down | 2.081829686 |
| ZNF16 | -0.096170668 | 0.159980996 | 0.387550465 | NS | 0.795931605 |
| C8orf33 | -0.077747455 | 0.290116769 | 0.512990599 | NS | 0.537427168 |
| WASHC1 | 0.009981415 | 0.942637225 | 0.968692351 | NS | 0.025655414 |
| FOXD4 | 0.234118895 | 0.348540022 | NA | NS | 0.457747346 |
| CBWD1 | -0.030392303 | 0.670082893 | 0.803257703 | NS | 0.173871469 |
| DOCK8 | 0.095749913 | 0.13426571 | 0.357885626 | NS | 0.872034886 |
| KANK1 | 0.113694038 | 0.360412961 | 0.57320678 | NS | 0.443199599 |
| DMRT1 | 0.84423122 | 0.095465383 | NA | NS | 1.02015408 |
| DMRT3 | 0.467601023 | 0.34942578 | NA | NS | 0.456645056 |
| DMRT2 | 0.674073391 | 0.085872967 | NA | NS | 1.066143531 |
| SMARCA2 | 0.057766883 | 0.215286192 | 0.444697279 | NS | 0.666983825 |
| VLDLR | 0.162851787 | 0.282351807 | 0.5053027 | NS | 0.549209428 |
| KCNV2 | 0.336379861 | 0.047767892 | 0.232168988 | Up | 1.320863921 |
| PUM3 | -0.105845031 | 0.092891411 | 0.30535169 | NS | 1.032024438 |
| RFX3 | 0.083475006 | 0.188113871 | 0.417570259 | NS | 0.72557918 |
| GLIS3 | 0.562876157 | 0.011024937 | 0.129467323 | Up | 1.9576239 |
| SLC1A1 | -0.044005282 | 0.829640732 | 0.903191142 | NS | 0.081109934 |
| SPATA6L | -0.09310703 | 0.517517633 | 0.694871679 | NS | 0.286074848 |
| PLPP6 | -0.083885792 | 0.270674153 | 0.495997715 | NS | 0.567553214 |
| CDC37L1 | -0.057826916 | 0.425596313 | 0.623837924 | NS | 0.371002143 |
| AK3 | -0.071222587 | 0.301236264 | 0.522647032 | NS | 0.521092748 |
| RCL1 | -0.255324339 | 0.028070432 | 0.187669577 | Down | 1.551750897 |
| JAK2 | 0.015059203 | 0.818467776 | 0.896653863 | NS | 0.086998414 |
| INSL6 | 0.303633275 | 0.001134714 | 0.054594994 | Up | 2.945113638 |
| INSL4 | 0.536605897 | 0.300928529 | NA | NS | 0.521536638 |
| RLN2 | 0.032708418 | 0.845063767 | 0.913061552 | NS | 0.073110519 |
| RLN1 | -0.03812402 | 0.742014651 | 0.848832144 | NS | 0.12958752 |
| PLGRKT | -0.115508366 | 0.242735438 | 0.470460443 | NS | 0.614866815 |
| CD274 | 0.286342474 | 0.060210819 | 0.256024541 | NS | 1.220325465 |
| PDCD1LG2 | 0.24783065 | 0.094435018 | 0.307427263 | NS | 1.024866931 |
| RIC1 | 0.044530085 | 0.270113727 | 0.495605238 | NS | 0.568453344 |
| ERMP1 | 0.025873063 | 0.574543221 | 0.735834294 | NS | 0.240677295 |
| KIAA2026 | -0.009224107 | 0.842713286 | 0.911681082 | NS | 0.074320159 |
| MLANA | 0.160370941 | 0.112320188 | 0.330922582 | NS | 0.949542178 |
| RANBP6 | -0.166498654 | 0.100601078 | 0.316044705 | NS | 0.997397364 |
| IL33 | 0.347977253 | 0.483998962 | NA | NS | 0.31515557 |
| TPD52L3 | 0.578443992 | 0.236197666 | NA | NS | 0.626724398 |
| UHRF2 | -0.056377738 | 0.178548318 | 0.408483 | NS | 0.748244238 |
| GLDC | 0.24888517 | 0.318265483 | 0.537481826 | NS | 0.49721046 |
| KDM4C | -0.038464701 | 0.42666079 | 0.624808838 | NS | 0.369917267 |
| DMAC1 | -0.172668279 | 0.101046872 | 0.316641506 | NS | 0.995477127 |
| PTPRD | 0.319639432 | 0.389583389 | 0.59694612 | NS | 0.409399569 |
| TYRP1 | 0.467765982 | 0.386577045 | NA | NS | 0.412763938 |
| LURAP1L | 0.143238925 | 0.584451018 | NA | NS | 0.23325188 |
| MPDZ | 0.488993731 | 0.228780459 | 0.457361215 | NS | 0.640581073 |
| NFIB | 0.528195706 | 0.043186613 | 0.222962086 | Up | 1.364650855 |
| ZDHHC21 | -0.090126336 | 0.204425265 | 0.434541248 | NS | 0.689465432 |
| CER1 | 0.582311562 | 0.367519439 | NA | NS | 0.434719685 |
| FREM1 | 0.363468303 | 0.27044573 | 0.495915885 | NS | 0.567919872 |
| TTC39B | 0.020674186 | 0.743873295 | 0.849897042 | NS | 0.128501032 |
| SNAPC3 | -0.015681775 | 0.71405558 | 0.830905915 | NS | 0.146267982 |
| PSIP1 | -0.065411218 | 0.146541559 | 0.372104233 | NS | 0.834039192 |
| CCDC171 | 0.048993865 | 0.628935036 | 0.773985503 | NS | 0.201394211 |
| BNC2 | 0.202857228 | 0.219999193 | 0.449257379 | NS | 0.657578913 |
| CNTLN | -0.62821228 | 1.62E-04 | 0.020530906 | Down | 3.790897566 |
| SH3GL2 | 0.192538274 | 0.71189727 | NA | NS | 0.147582672 |
| ADAMTSL1 | 0.67470204 | 0.052866027 | 0.243022432 | NS | 1.276823325 |
| SAXO1 | 0.237736629 | 0.414472872 | 0.616134093 | NS | 0.38250389 |
| RRAGA | -0.030922304 | 0.620648466 | 0.767922494 | NS | 0.207154314 |
| HAUS6 | -0.116629686 | 0.107053532 | 0.323901028 | NS | 0.970398998 |
| PLIN2 | -0.063657757 | 0.458584463 | 0.650068171 | NS | 0.338580663 |
| DENND4C | -0.118692028 | 0.018920605 | 0.159660905 | Down | 1.723064979 |
| RPS6 | -0.279838947 | 0.088971832 | 0.299299778 | NS | 1.050747469 |
| ACER2 | 0.113878643 | 0.300310185 | 0.521749648 | NS | 0.522429939 |
| SLC24A2 | 0.161436566 | 0.592879178 | 0.749346361 | NS | 0.227033802 |
| MLLT3 | -0.100996643 | 0.167887732 | 0.39601577 | NS | 0.774981038 |
| FOCAD | -0.009612408 | 0.859041575 | 0.920338791 | NS | 0.065985817 |
| HACD4 | 0.029402031 | 0.660657669 | 0.796210174 | NS | 0.180023519 |
| IFNB1 | 0.341161749 | 0.253269674 | NA | NS | 0.596416808 |
| IFNW1 | 0.438646377 | 0.171136323 | NA | NS | 0.766657803 |
| IFNA21 | 0.359993347 | 0.27075287 | NA | NS | 0.56742693 |
| IFNA4 | 1.15342419 | 0.047723272 | NA | Up | 1.321269785 |
| IFNA7 | 1.056379637 | 0.097122449 | NA | NS | 1.012680376 |
| IFNA10 | 1.289057174 | 0.031423546 | NA | Up | 1.502744813 |
| IFNA16 | -0.278896585 | 0.660067749 | NA | NS | 0.180411486 |
| IFNA14 | 0.762939608 | 0.198471183 | NA | NS | 0.702302541 |
| IFNA5 | 0.223571192 | 0.556017873 | NA | NS | 0.254911248 |
| KLHL9 | -0.060284733 | 0.417781636 | 0.618692258 | NS | 0.379050653 |
| IFNA6 | -0.235710271 | 0.713763747 | NA | NS | 0.146445514 |
| IFNA13 | 0.637964697 | 0.320321744 | NA | NS | 0.494413579 |
| IFNA2 | 0.036647007 | 0.956998631 | NA | NS | 0.019088683 |
| IFNA8 | 0.606673883 | 0.344760182 | NA | NS | 0.462482899 |
| IFNA1 | 0.328774353 | 0.612436341 | NA | NS | 0.212939047 |
| IFNE | -0.040937167 | 0.860154929 | NA | NS | 0.065423317 |
| MTAP | -0.022566384 | 0.612794612 | 0.763061234 | NS | 0.212685062 |
| CDKN2A | 0.007089599 | 0.946825729 | 0.971427419 | NS | 0.023729949 |
| CDKN2B | -0.047958906 | 0.557925675 | 0.723698109 | NS | 0.253423652 |
| DMRTA1 | 0.702322759 | 0.084992514 | NA | NS | 1.070619326 |
| ELAVL2 | 0.507561497 | 0.2719935 | 0.497340928 | NS | 0.565441475 |
| IZUMO3 | 0.405039887 | 0.526744739 | NA | NS | 0.278399793 |
| TUSC1 | 0.203577359 | 0.344787082 | NA | NS | 0.462449014 |
| CAAP1 | -0.177364329 | 0.015009194 | 0.146973997 | Down | 1.823642636 |
| PLAA | 0.04401019 | 0.181500471 | 0.411475329 | NS | 0.741122245 |
| IFT74 | -0.213617023 | 0.043963367 | 0.224627854 | Down | 1.356909057 |
| LRRC19 | 0.081261238 | 0.512801981 | 0.691200693 | NS | 0.290050305 |
| TEK | 0.295861807 | 0.171591147 | 0.400205856 | NS | 0.765505123 |
| EQTN | 0.518791741 | 0.226622307 | NA | NS | 0.644697343 |
| MOB3B | 0.193848117 | 0.022891206 | 0.17233675 | Up | 1.640331332 |
| IFNK | 0.408266201 | 0.002032069 | 0.070942758 | Up | 2.692061502 |
| C9orf72 | 0.149713902 | 0.105595332 | 0.321707479 | NS | 0.97635528 |
| LINGO2 | 0.031670996 | 0.886211737 | 0.935947485 | NS | 0.052462502 |
| ACO1 | 0.222798975 | 0.015145388 | 0.147327548 | Up | 1.819719586 |
| DDX58 | 0.170375048 | 0.055702018 | 0.248760687 | NS | 1.254129072 |
| TOPORS | -0.094004495 | 0.28246095 | 0.505328887 | NS | 0.549041584 |
| SMIM27 | 0.025293428 | 0.736741696 | 0.844910281 | NS | 0.132684751 |
| NDUFB6 | -0.064937225 | 0.574445865 | 0.735797513 | NS | 0.240750893 |
| TAF1L | 0.346362455 | 0.381371527 | NA | NS | 0.418651734 |
| TMEM215 | 0.482765592 | 0.2584118 | NA | NS | 0.587687658 |
| APTX | -0.014943349 | 0.732922883 | 0.842188847 | NS | 0.134941719 |
| DNAJA1 | -0.142571271 | 0.150025765 | 0.376171504 | NS | 0.823834151 |
| SMU1 | -0.117160057 | 0.057336836 | 0.251032686 | NS | 1.241566273 |
| B4GALT1 | 0.257619345 | 0.002227802 | 0.072832159 | Up | 2.65212337 |
| SPINK4 | 0.38874498 | 0.24288613 | NA | NS | 0.614597286 |
| BAG1 | -0.370856197 | 0.016872359 | 0.152976731 | Down | 1.772824184 |
| CHMP5 | -0.145904756 | 0.288504867 | 0.511822113 | NS | 0.539846856 |
| NFX1 | 0.032886243 | 0.49898889 | 0.680755559 | NS | 0.301909123 |
| AQP7 | 0.205410479 | 0.612350347 | 0.762881338 | NS | 0.213000032 |
| AQP3 | -0.196750717 | 0.049289528 | 0.235025767 | Down | 1.307245345 |
| NOL6 | 0.022592145 | 0.775416436 | 0.869240686 | NS | 0.110464998 |
| ANKRD18B | 0.395646347 | 0.268199816 | 0.493891886 | NS | 0.571541524 |
| PRSS3 | 0.371089173 | 0.272318424 | NA | NS | 0.564922975 |
| UBE2R2 | 0.18878136 | 0.015312655 | 0.147421994 | Up | 1.814949505 |
| UBAP2 | -0.374300267 | 0.008578655 | 0.118335308 | Down | 2.066580785 |
| DCAF12 | -0.068855236 | 0.714888448 | 0.831377499 | NS | 0.145761721 |
| UBAP1 | 0.094972584 | 0.361137156 | 0.573760885 | NS | 0.442327826 |
| KIF24 | -0.056501348 | 0.612906965 | 0.763118154 | NS | 0.212605443 |
| NUDT2 | -0.12728209 | 0.200852867 | 0.430584327 | NS | 0.697121964 |
| MYORG | 0.371192251 | 0.026750977 | 0.184867497 | Up | 1.572660345 |
| C9orf24 | 0.281635597 | 0.307404566 | NA | NS | 0.512289686 |
| FAM219A | 0.089385679 | 0.314437443 | 0.533741944 | NS | 0.502465744 |
| DNAI1 | 0.256876961 | 0.379115963 | 0.589025245 | NS | 0.421227929 |
| ENHO | -0.537615222 | 0.021189673 | NA | Down | 1.673875754 |
| CNTFR | 0.555395056 | 0.269663153 | NA | NS | 0.569178393 |
| RPP25L | -0.164173564 | 0.037498788 | 0.210180429 | Down | 1.425982774 |
| DCTN3 | -0.013641404 | 0.828844159 | 0.902682979 | NS | 0.081527119 |
| ARID3C | 0.739399736 | 0.115815459 | NA | NS | 0.936233468 |
| SIGMAR1 | -0.042414266 | 0.565211898 | 0.729102337 | NS | 0.247788704 |
| GALT | -0.077134136 | 0.418519134 | 0.618901614 | NS | 0.378284682 |
| IL11RA | 0.165566954 | 0.330238582 | 0.547441347 | NS | 0.48117219 |
| CCL19 | 0.550993766 | 0.31960424 | NA | NS | 0.495387467 |
| CCL21 | 0.922839805 | 0.091843971 | NA | NS | 1.036949345 |
| FAM205A | 0.401346919 | 0.338672186 | NA | NS | 0.470220469 |
| FAM205C | 0.544961984 | 0.327846691 | NA | NS | 0.484329195 |
| PHF24 | 0.327238477 | 0.152950517 | 0.379520766 | NS | 0.81544905 |
| DNAJB5 | 0.066161846 | 0.62627009 | 0.772075018 | NS | 0.203238329 |
| C9orf131 | 0.009055129 | 0.931769808 | 0.962684504 | NS | 0.030691366 |
| VCP | -0.165171369 | 0.024497868 | 0.177547525 | Down | 1.610871704 |
| FANCG | -0.114403533 | 0.0560634 | 0.249087638 | NS | 1.251320565 |
| PIGO | -0.125066242 | 0.080458157 | 0.286202881 | NS | 1.094429918 |
| STOML2 | -0.359069259 | 2.83E-04 | 0.028091276 | Down | 3.548946989 |
| FAM214B | -0.093742284 | 0.482246308 | 0.66853282 | NS | 0.316731089 |
| UNC13B | 0.383139916 | 0.046057033 | 0.228777969 | Up | 1.336704039 |
| RUSC2 | 0.131387905 | 0.330995286 | 0.548094548 | NS | 0.480178192 |
| FAM166B | 0.384760811 | 0.22895828 | NA | NS | 0.640243646 |
| TESK1 | 0.191291506 | 0.072132332 | 0.273763446 | NS | 1.141870025 |
| CD72 | -0.112268641 | 0.338118521 | 0.554182379 | NS | 0.470931039 |
| SIT1 | -0.168277281 | 0.088623788 | 0.298798608 | NS | 1.05244969 |
| CCDC107 | 0.502069649 | 4.83E-04 | 0.037229953 | Up | 3.316224472 |
| ARHGEF39 | -0.12364537 | 0.069032379 | 0.26919454 | NS | 1.160947162 |
| CA9 | 0.910873726 | 0.067993076 | NA | NS | 1.167535311 |
| TPM2 | -0.307246096 | 0.089791669 | 0.30028957 | NS | 1.046763957 |
| TLN1 | 0.028906642 | 0.810038749 | 0.890785913 | NS | 0.091494206 |
| CREB3 | -0.226171872 | 0.004097184 | 0.090695453 | Down | 2.387514513 |
| GBA2 | 0.028680985 | 0.597402646 | 0.752238586 | NS | 0.223732858 |
| RGP1 | 0.024239356 | 0.634033133 | 0.776753334 | NS | 0.197888046 |
| NPR2 | 0.014481192 | 0.916233131 | 0.953437582 | NS | 0.037994008 |
| SPAG8 | -0.158285487 | 0.204758434 | 0.434924698 | NS | 0.688758201 |
| HINT2 | 0.018736203 | 0.9065831 | 0.948235575 | NS | 0.042592381 |
| TMEM8B | 0.126223982 | 0.497488192 | 0.679709754 | NS | 0.303217223 |
| FAM221B | 0.394064304 | 0.293416088 | NA | NS | 0.532516077 |
| OR13J1 | 0.454841354 | 0.533806495 | NA | NS | 0.272616147 |
| HRCT1 | 0.739534927 | 0.160226146 | NA | NS | 0.795266614 |
| SPAAR | 0.816586664 | 0.035361312 | NA | Up | 1.451471626 |
| OR2S2 | 0.501581507 | 0.415712506 | NA | NS | 0.381206911 |
| RECK | 0.003238666 | 0.956107803 | 0.976096862 | NS | 0.019493138 |
| GLIPR2 | -0.034862669 | 0.722926639 | 0.836438612 | NS | 0.140905772 |
| CCIN | 0.313072929 | 0.366268064 | NA | NS | 0.436200947 |
| CLTA | -0.175643737 | 0.02275545 | 0.171982657 | Down | 1.642914565 |
| GNE | -0.104351061 | 0.032124448 | 0.198223945 | Down | 1.493164321 |
| RNF38 | 0.098645957 | 0.226632661 | 0.455213225 | NS | 0.644677502 |
| MELK | -0.365152645 | 0.058191014 | 0.252197542 | NS | 1.235144078 |
| PAX5 | 0.012740556 | 0.925440723 | 0.959164117 | NS | 0.033651394 |
| ZCCHC7 | -0.205258676 | 0.04610368 | 0.228839724 | Down | 1.336264404 |
| GRHPR | -0.168189126 | 0.018254536 | 0.157108372 | Down | 1.738629207 |
| ZBTB5 | 0.035370428 | 0.508808795 | 0.688232771 | NS | 0.293445391 |
| POLR1E | -0.24257191 | 0.004195627 | 0.091951138 | Down | 2.377203147 |
| FBXO10 | 0.163187824 | 0.144554074 | 0.369690386 | NS | 0.839969664 |
| TOMM5 | 0.068597833 | 0.343711298 | 0.558671124 | NS | 0.463806192 |
| FRMPD1 | 0.497130823 | 0.228396052 | NA | NS | 0.641311407 |
| TRMT10B | 0.038477006 | 0.425713216 | 0.623939849 | NS | 0.370882867 |
| EXOSC3 | 0.066407083 | 0.157976388 | 0.385300876 | NS | 0.801407821 |
| DCAF10 | -0.143575606 | 0.087595409 | 0.29716607 | NS | 1.057518657 |
| SLC25A51 | -0.122408802 | 0.07070291 | 0.271623362 | NS | 1.150562708 |
| SHB | 0.127718392 | 0.641891922 | 0.783197059 | NS | 0.19253809 |
| ALDH1B1 | -0.30040784 | 0.001945052 | 0.069602477 | Down | 2.711068738 |
| IGFBPL1 | 0.668142465 | 0.118184171 | NA | NS | 0.927440686 |
| FAM95C | -0.024658222 | 0.89884283 | NA | NS | 0.046316242 |
| ANKRD18A | 0.022749351 | 0.846005582 | 0.913313927 | NS | 0.072626771 |
| FAM240B | 1.834080554 | 0.001757729 | NA | Up | 2.755048117 |
| CNTNAP3 | 0.444392709 | 0.024895597 | 0.178845349 | Up | 1.603877448 |
| SPATA31A1 | -0.214003817 | 0.733287958 | NA | NS | 0.134725447 |
| FOXD4L6 | 0.654849675 | 0.271550889 | NA | NS | 0.566148772 |
| CBWD6 | 0.041817369 | 0.653778484 | 0.791973775 | NS | 0.184569376 |
| CNTNAP3B | 0.189899168 | 0.445105741 | 0.639053249 | NS | 0.351536804 |
| SPATA31A6 | 1.067350112 | 0.039362378 | NA | Up | 1.404918671 |
| SPATA31A7 | 0.103341236 | 0.858980663 | NA | NS | 0.066016613 |
| CNTNAP3C | 0.75907825 | 0.002349713 | 0.074602988 | Up | 2.628985163 |
| ANKRD20A4P | -0.654510636 | 0.031343287 | 0.196726942 | Down | 1.503855465 |
| FOXD4L5 | 0.418648541 | 0.333376436 | NA | NS | 0.477065101 |
| CBWD5 | 0.177091257 | 0.033367595 | 0.201419259 | Up | 1.47667509 |
| FOXD4L4 | 0.493989051 | 0.075524423 | NA | NS | 1.121912584 |
| ANKRD20A3P | 0.814795314 | 0.129220991 | NA | NS | 0.888666934 |
| ZNF658 | -0.081713221 | 0.428939024 | 0.626678972 | NS | 0.367604441 |
| SPATA31A3 | 0.614294533 | 0.378301071 | NA | NS | 0.422162429 |
| ANKRD20A1 | -0.020601681 | 0.954961926 | NA | NS | 0.020013943 |
| CBWD3 | 0.164647653 | 0.042142111 | 0.220751111 | Up | 1.375283717 |
| FOXD4L3 | 1.17180935 | 1.88E-04 | NA | Up | 3.726033662 |
| PGM5 | 0.203189011 | 0.261024362 | 0.487090765 | NS | 0.583318958 |
| TMEM252 | 0.262497572 | 0.14475668 | 0.369914478 | NS | 0.839361385 |
| PIP5K1B | 0.024077511 | 0.871705861 | 0.927542179 | NS | 0.059630034 |
| PABIR1 | -0.099749627 | 0.205440453 | 0.435426865 | NS | 0.687314036 |
| PRKACG | 0.762708788 | 0.081956925 | NA | NS | 1.086414347 |
| FXN | 0.026115049 | 0.673870895 | 0.805843649 | NS | 0.171423301 |
| TJP2 | 0.222225882 | 0.145031746 | 0.37031258 | NS | 0.838536924 |
| FAM189A2 | 0.556095344 | 0.139858323 | 0.364064028 | NS | 0.854311683 |
| APBA1 | 0.450694943 | 0.09615772 | NA | NS | 1.017015842 |
| PTAR1 | -0.017985466 | 0.780095092 | 0.872819278 | NS | 0.107852454 |
| C9orf135 | 0.704627056 | 0.097154091 | NA | NS | 1.012538907 |
| MAMDC2 | 0.339342998 | 0.045767071 | 0.228356747 | Up | 1.339446877 |
| SMC5 | -0.060875698 | 0.186217939 | 0.415835484 | NS | 0.729978485 |
| KLF9 | -0.056952918 | 0.454519626 | 0.646828339 | NS | 0.34244736 |
| TRPM3 | 0.562724851 | 0.142299797 | 0.366998868 | NS | 0.846795719 |
| CEMIP2 | 0.061367557 | 0.249564399 | 0.47668561 | NS | 0.602817368 |
| ABHD17B | -0.184763297 | 0.01087968 | 0.12856202 | Down | 1.96338389 |
| C9orf85 | -0.134222078 | 0.040487229 | 0.217010192 | Down | 1.392681941 |
| C9orf57 | 0.531633901 | 0.252759115 | NA | NS | 0.597293174 |
| GDA | 0.44879523 | 0.266856078 | 0.49286596 | NS | 0.573722901 |
| ZFAND5 | -0.196140032 | 0.02716594 | 0.185717716 | Down | 1.565975269 |
| TMC1 | 0.314577498 | 0.362744005 | 0.575276172 | NS | 0.440399757 |
| ALDH1A1 | -0.28310772 | 0.041495162 | 0.21947395 | Down | 1.382002535 |
| ANXA1 | -0.22045543 | 0.102535241 | 0.318322095 | NS | 0.989126843 |
| RORB | 0.326319505 | 0.263072757 | 0.489264288 | NS | 0.579924124 |
| TRPM6 | 0.04520785 | 0.757790927 | 0.858602276 | NS | 0.120450599 |
| C9orf40 | -0.387401069 | 0.02257013 | 0.171443155 | Down | 1.646465944 |
| CARNMT1 | -0.217624245 | 0.006561292 | 0.108312793 | Down | 2.183010611 |
| NMRK1 | -0.065831961 | 0.380801127 | 0.590688169 | NS | 0.419301775 |
| OSTF1 | -0.038854868 | 0.654499117 | 0.792290225 | NS | 0.184090935 |
| PCSK5 | 6.35E-05 | 0.999304182 | 0.999609713 | NS | 3.02E-04 |
| RFK | -0.28441366 | 0.005719046 | 0.102180893 | Down | 2.242676383 |
| GCNT1 | -0.084752442 | 0.230613125 | 0.458901885 | NS | 0.637115978 |
| PRUNE2 | 0.205722118 | 0.12705774 | 0.348830209 | NS | 0.895998873 |
| FOXB2 | 0.722794127 | 0.171689889 | NA | NS | 0.765255281 |
| VPS13A | -0.127491112 | 0.110548306 | 0.329041981 | NS | 0.956447908 |
| GNA14 | 0.369752442 | 0.077532852 | 0.281760929 | NS | 1.110514241 |
| GNAQ | 0.103722061 | 0.143666475 | 0.368744715 | NS | 0.842644565 |
| CEP78 | -0.16269715 | 0.075084841 | 0.27811629 | NS | 1.124447736 |
| PSAT1 | 0.101890443 | 0.332432934 | 0.549152659 | NS | 0.478295957 |
| TLE4 | 0.05281642 | 0.320624903 | 0.539353999 | NS | 0.494002749 |
| TLE1 | 0.139713542 | 0.130674929 | 0.352706804 | NS | 0.883807727 |
| SPATA31D3 | 0.280824937 | 0.652423482 | NA | NS | 0.185470416 |
| SPATA31D1 | 0.30459947 | 0.520612315 | 0.696688569 | NS | 0.283485563 |
| RASEF | 0.498321711 | 0.214604738 | 0.443861438 | NS | 0.668360695 |
| FRMD3 | 0.146488619 | 0.092425557 | 0.304565483 | NS | 1.034207923 |
| IDNK | 0.009321848 | 0.865720079 | 0.92406419 | NS | 0.062622509 |
| UBQLN1 | -0.075507139 | 0.338218663 | 0.55422752 | NS | 0.470802432 |
| GKAP1 | -0.351757482 | 0.011427127 | 0.131302419 | Down | 1.942062955 |
| KIF27 | 0.194848876 | 0.101403057 | 0.317114382 | NS | 0.993948952 |
| C9orf64 | -0.169572632 | 0.061234153 | 0.257380382 | NS | 1.213006288 |
| HNRNPK | -0.173723557 | 0.003195917 | 0.084356647 | Down | 2.495404457 |
| RMI1 | 0.215310056 | 0.00510254 | 0.09835814 | Up | 2.2922136 |
| SLC28A3 | 0.370288437 | 0.107839457 | 0.325350975 | NS | 0.967222306 |
| NTRK2 | 0.474066153 | 0.165122472 | 0.392897128 | NS | 0.782193818 |
| AGTPBP1 | -0.066253899 | 0.399464118 | 0.604541547 | NS | 0.398522225 |
| NAA35 | -0.065264033 | 0.099753764 | 0.314784866 | NS | 1.001070708 |
| GOLM1 | -0.020817867 | 0.755890028 | 0.857296263 | NS | 0.121541384 |
| C9orf153 | 0.046659841 | 0.789953296 | 0.87861252 | NS | 0.102398584 |
| ISCA1 | -0.311123017 | 0.038944958 | 0.214288889 | Down | 1.409548758 |
| TUT7 | 0.107306922 | 0.159561005 | 0.387098757 | NS | 0.797073237 |
| GAS1 | 0.138120618 | 0.540092498 | 0.710424351 | NS | 0.267531855 |
| DAPK1 | 0.004693632 | 0.965518446 | 0.980633441 | NS | 0.015239425 |
| CTSL | -0.198073233 | 0.124661371 | 0.346185853 | NS | 0.9042681 |
| SPATA31E1 | 0.58338196 | 0.181407786 | NA | NS | 0.741344078 |
| CDK20 | 0.143726504 | 0.207313544 | 0.437271578 | NS | 0.683372323 |
| SPATA31C2 | 0.437597042 | 0.27112973 | NA | NS | 0.566822858 |
| SPIN1 | 0.018850324 | 0.728226409 | 0.839178888 | NS | 0.137733576 |
| NXNL2 | 0.512201649 | 0.277648504 | NA | NS | 0.556504662 |
| S1PR3 | 0.156611057 | 0.086091804 | 0.29485869 | NS | 1.065038191 |
| SHC3 | 0.213772792 | 0.303237509 | 0.524458861 | NS | 0.51821708 |
| CKS2 | -0.087139228 | 0.597839192 | 0.752664129 | NS | 0.223415618 |
| SECISBP2 | -0.031960424 | 0.57942605 | 0.739153088 | NS | 0.237001984 |
| SEMA4D | 0.080905612 | 0.461517069 | 0.652569236 | NS | 0.335812232 |
| GADD45G | 0.426971372 | 0.010229306 | 0.126233693 | Up | 1.990153849 |
| DIRAS2 | 0.622983914 | 0.140182173 | NA | NS | 0.853307211 |
| SYK | -0.025552895 | 0.729383636 | 0.84017423 | NS | 0.137043984 |
| AUH | -0.045002132 | 0.418502799 | 0.618901614 | NS | 0.378301633 |
| NFIL3 | 0.172494902 | 0.120578763 | 0.341313216 | NS | 0.918729175 |
| ROR2 | 0.371864019 | 0.231503833 | 0.45979109 | NS | 0.635441813 |
| SPTLC1 | -0.099130695 | 0.037291012 | 0.2099407 | NS | 1.428395825 |
| IARS1 | -0.07384394 | 0.143745993 | 0.368818142 | NS | 0.842404252 |
| NOL8 | -0.106563916 | 0.084200978 | 0.292034469 | NS | 1.074682863 |
| CENPP | 0.175929404 | 0.019857026 | 0.163643616 | Up | 1.702085797 |
| OGN | 0.244081176 | 0.073866449 | 0.276013938 | NS | 1.131552776 |
| OMD | 0.247585418 | 0.111643923 | 0.330121825 | NS | 0.95216491 |
| ASPN | 0.213334291 | 0.189284036 | 0.418800923 | NS | 0.722886012 |
| ECM2 | 0.255642108 | 0.075705028 | 0.279014573 | NS | 1.120875274 |
| IPPK | -0.005662211 | 0.916302558 | 0.953437582 | NS | 0.037961101 |
| BICD2 | 0.001385878 | 0.981747398 | 0.989263416 | NS | 0.008000241 |
| ZNF484 | -0.069386795 | 0.329140241 | 0.546665444 | NS | 0.482619017 |
| FGD3 | 0.092856068 | 0.341667745 | 0.557049526 | NS | 0.466396019 |
| SUSD3 | -0.101446526 | 0.289411378 | 0.512601366 | NS | 0.538484398 |
| CARD19 | 0.030139715 | 0.683127376 | 0.811353013 | NS | 0.16549831 |
| NINJ1 | 0.278401357 | 0.007929565 | 0.11472857 | Up | 2.100750652 |
| WNK2 | 0.818015914 | 0.023147248 | 0.173122445 | Up | 1.635500643 |
| FAM120AOS | 0.003456771 | 0.927999774 | 0.960372834 | NS | 0.03245213 |
| FAM120A | 0.062060437 | 0.149136071 | 0.37524877 | NS | 0.826417302 |
| PHF2 | -0.06018485 | 0.458412913 | 0.649865214 | NS | 0.338743157 |
| BARX1 | 0.886791932 | 0.075204707 | NA | NS | 1.123754975 |
| PTPDC1 | -0.001786913 | 0.980175281 | 0.98824452 | NS | 0.008696254 |
| ZNF169 | 0.032557898 | 0.624602823 | 0.77109254 | NS | 0.204396057 |
| NUTM2F | 0.50748216 | 0.258089637 | NA | NS | 0.588229433 |
| MFSD14B | -0.166895323 | 0.08384121 | 0.291624423 | NS | 1.076542463 |
| FBP2 | 0.120425258 | 0.859475158 | NA | NS | 0.065766671 |
| FBP1 | -0.052359186 | 0.610722858 | 0.761682453 | NS | 0.214155825 |
| AOPEP | 0.010618566 | 0.885020555 | 0.93507754 | NS | 0.053046642 |
| FANCC | 0.080353098 | 0.266105423 | 0.492035072 | NS | 0.574946275 |
| PTCH1 | 0.131502153 | 0.276197342 | 0.500789448 | NS | 0.558780506 |
| ERCC6L2 | -0.022261159 | 0.64982085 | 0.788856318 | NS | 0.187206358 |
| HSD17B3 | 0.894838439 | 0.191579624 | NA | NS | 0.717650683 |
| SLC35D2 | -0.179016066 | 0.065299945 | 0.264481527 | NS | 1.185087182 |
| ZNF367 | -0.122854703 | 0.127560489 | 0.349031102 | NS | 0.894283823 |
| HABP4 | -0.200528229 | 0.007380918 | 0.112471844 | Down | 2.131889632 |
| CDC14B | 0.143853186 | 0.109791262 | 0.328012756 | NS | 0.959432224 |
| PRXL2C | -0.096032205 | 0.314925157 | 0.534371782 | NS | 0.501792646 |
| ZNF510 | -0.085585309 | 0.227265013 | 0.455855629 | NS | 0.643467419 |
| ZNF782 | -0.088940762 | 0.115567836 | 0.334479283 | NS | 0.937163021 |
| NUTM2G | 0.197050759 | 0.172011276 | 0.4006964 | NS | 0.764443081 |
| CTSV | 0.34898838 | 0.212685807 | 0.442550518 | NS | 0.67226149 |
| CCDC180 | 0.248049062 | 0.097394168 | 0.311830176 | NS | 1.011467046 |
| TDRD7 | 0.092246016 | 0.202762372 | 0.432901167 | NS | 0.693012637 |
| TMOD1 | -0.245013685 | 0.299522494 | 0.520845496 | NS | 0.523570556 |
| TSTD2 | -0.014539843 | 0.760628305 | 0.860168677 | NS | 0.118827517 |
| NCBP1 | -0.003888746 | 0.922439219 | 0.95733572 | NS | 0.03506224 |
| XPA | 0.020342715 | 0.686932396 | 0.814158315 | NS | 0.163086002 |
| FOXE1 | 0.663625981 | 0.011097966 | NA | Up | 1.954756602 |
| TRMO | 0.024824411 | 0.548112096 | 0.716324082 | NS | 0.261130614 |
| HEMGN | -0.367620714 | 0.018214879 | 0.157062186 | Down | 1.739573701 |
| ANP32B | -0.209246792 | 0.262827785 | 0.488909438 | NS | 0.580328726 |
| NANS | -0.046452864 | 0.301777597 | 0.523285452 | NS | 0.520313004 |
| TRIM14 | 0.200675177 | 0.01761187 | 0.155449116 | Up | 1.754194531 |
| CORO2A | 0.198094817 | 0.003322861 | 0.085084692 | Up | 2.478487863 |
| TBC1D2 | 0.055858451 | 0.529962945 | 0.70413711 | NS | 0.275754495 |
| GABBR2 | 0.820149128 | 0.058177448 | 0.252197542 | NS | 1.235245332 |
| ANKS6 | 0.211366295 | 0.010116968 | 0.126203819 | Up | 1.994949616 |
| GALNT12 | -0.050195284 | 0.565551711 | 0.729417429 | NS | 0.247527679 |
| COL15A1 | 0.682010852 | 0.048176899 | 0.233024927 | Up | 1.317161159 |
| TGFBR1 | -0.038547149 | 0.593715481 | 0.749772128 | NS | 0.226421627 |
| ALG2 | -0.114449578 | 0.078493016 | 0.283183216 | NS | 1.105168983 |
| SEC61B | -0.165174727 | 0.09540856 | 0.309058959 | NS | 1.020412659 |
| NR4A3 | 0.266745756 | 0.217039607 | 0.446641045 | NS | 0.663461006 |
| STX17 | 0.032270757 | 0.490778067 | 0.67506302 | NS | 0.309114855 |
| ERP44 | 0.098962912 | 0.011649687 | 0.132276213 | NS | 1.933685759 |
| INVS | -0.006755356 | 0.887567169 | 0.936558391 | NS | 0.05179877 |
| TEX10 | -0.03868243 | 0.384494421 | 0.59312605 | NS | 0.415109957 |
| MSANTD3 | -0.126186614 | 0.127456606 | 0.348830209 | NS | 0.89463765 |
| CAVIN4 | 0.044819263 | 0.854282183 | NA | NS | 0.068398651 |
| BAAT | 0.218079758 | 0.235398873 | 0.46327275 | NS | 0.62819562 |
| MRPL50 | -0.243003853 | 0.043640058 | 0.224086881 | Down | 1.360114683 |
| ZNF189 | -0.153736353 | 0.003960169 | 0.090040171 | Down | 2.402286296 |
| ALDOB | 0.323572457 | 0.112183539 | 0.330787614 | NS | 0.950070865 |
| PGAP4 | 0.453120416 | 0.160225946 | NA | NS | 0.795267155 |
| RNF20 | -0.172565958 | 3.09E-04 | 0.029354584 | Down | 3.510180433 |
| GRIN3A | 0.075931257 | 0.353325861 | 0.566794858 | NS | 0.451824574 |
| PPP3R2 | 0.344026913 | 0.177433839 | 0.407380051 | NS | 0.750963551 |
| CYLC2 | 0.996093383 | 0.046308483 | NA | Up | 1.334339442 |
| SMC2 | -0.158761606 | 0.017776737 | 0.155923237 | Down | 1.750147954 |
| OR13F1 | 0.370700157 | 0.457849169 | NA | NS | 0.339277569 |
| OR13C4 | 0.875172458 | 0.103844352 | NA | NS | 0.983617119 |
| OR13C8 | 0.089143888 | 0.884530281 | NA | NS | 0.053287295 |
| OR13C5 | 0.853512673 | 0.082528339 | NA | NS | 1.083396897 |
| OR13C9 | 0.464978038 | 0.373266622 | NA | NS | 0.427980844 |
| OR13D1 | 0.469186245 | 0.024105224 | NA | Up | 1.617888821 |
| NIPSNAP3A | -0.161077852 | 0.179964503 | 0.409996481 | NS | 0.744813148 |
| NIPSNAP3B | 0.174073736 | 0.12012086 | 0.340734879 | NS | 0.920381568 |
| ABCA1 | -0.075395668 | 0.548000125 | 0.716259494 | NS | 0.261219343 |
| SLC44A1 | -0.030808874 | 0.614094674 | 0.764054018 | NS | 0.211764669 |
| FSD1L | -0.009836784 | 0.886884653 | 0.936096982 | NS | 0.05213286 |
| FKTN | 0.055357958 | 0.368217641 | 0.580219196 | NS | 0.433895408 |
| TAL2 | 0.101731956 | 0.78617101 | NA | NS | 0.104482975 |
| TMEM38B | -0.113170943 | 0.21527712 | 0.444697279 | NS | 0.667002125 |
| ZNF462 | 0.410813395 | 0.122120486 | 0.342932892 | NS | 0.913211474 |
| RAD23B | -0.104341362 | 0.11486077 | 0.333655677 | NS | 0.939828276 |
| KLF4 | -0.121160593 | 0.284644642 | 0.507588489 | NS | 0.545696987 |
| ACTL7B | 1.176181568 | 0.049068519 | NA | Up | 1.309197052 |
| ACTL7A | 0.250188974 | 0.327118764 | NA | NS | 0.485294544 |
| ELP1 | -0.078668211 | 0.096803964 | 0.310921075 | NS | 1.01410686 |
| ABITRAM | -0.010807947 | 0.86178223 | 0.922095712 | NS | 0.064602465 |
| CTNNAL1 | 0.108409878 | 0.519078827 | 0.69601515 | NS | 0.284766686 |
| TMEM245 | 0.033980307 | 0.681286576 | 0.810199666 | NS | 0.166670168 |
| FRRS1L | 0.62917125 | 0.134225786 | 0.357862509 | NS | 0.872164044 |
| EPB41L4B | 0.263385375 | 0.518842273 | 0.695861193 | NS | 0.284964647 |
| PTPN3 | 0.291363357 | 0.374986034 | 0.585727059 | NS | 0.425984907 |
| PALM2AKAP2 | -0.089919885 | 0.258414539 | 0.485059403 | NS | 0.587683055 |
| C9orf152 | 0.671725965 | 0.104289256 | NA | NS | 0.981760432 |
| TXN | 0.044277958 | 0.716093872 | 0.832106239 | NS | 0.145030042 |
| TXNDC8 | 0.62309709 | 0.129499068 | NA | NS | 0.887733357 |
| SVEP1 | 0.408390828 | 0.265765098 | 0.491802866 | NS | 0.575502054 |
| MUSK | 0.493497104 | 0.044627558 | 0.225889772 | Up | 1.350396874 |
| LPAR1 | -0.037128259 | 0.751082318 | 0.853947238 | NS | 0.124312462 |
| OR2K2 | 0.861729556 | 0.092379429 | NA | NS | 1.034424725 |
| ECPAS | -0.028630042 | 0.483038584 | 0.669197484 | NS | 0.316018177 |
| ZNF483 | 0.094476435 | 0.198617731 | 0.428520327 | NS | 0.701981984 |
| PTGR1 | 0.19564425 | 0.522131583 | 0.697843221 | NS | 0.282220036 |
| DNAJC25 | -0.038169213 | 0.603842016 | 0.756851459 | NS | 0.219076671 |
| GNG10 | 0.315592031 | 0.027159859 | 0.185717716 | Up | 1.56607249 |
| SHOC1 | 0.337842369 | 0.048293123 | 0.233236844 | Up | 1.316114705 |
| UGCG | 0.008071575 | 0.918065215 | 0.954826686 | NS | 0.037126468 |
| SUSD1 | -0.199722129 | 0.028908756 | 0.189813165 | Down | 1.538970603 |
| PTBP3 | 0.07432821 | 0.255217611 | 0.482021584 | NS | 0.593089361 |
| HSDL2 | 0.0431421 | 0.662167959 | 0.797526721 | NS | 0.179031838 |
| KIAA1958 | 0.314138355 | 3.89E-04 | 0.033887464 | Up | 3.409878598 |
| INIP | -0.210285324 | 0.009699658 | 0.123893787 | Down | 2.013243592 |
| SNX30 | -0.020307967 | 0.681945478 | 0.810483683 | NS | 0.166250346 |
| SLC46A2 | 0.008286866 | 0.937791156 | 0.965732476 | NS | 0.027893867 |
| ZFP37 | -0.135426534 | 0.232654474 | 0.461081062 | NS | 0.633288592 |
| SLC31A2 | 0.144396976 | 0.181281971 | 0.411305697 | NS | 0.741645385 |
| FKBP15 | 0.099182341 | 0.102931176 | 0.318730365 | NS | 0.987453067 |
| SLC31A1 | -0.076224985 | 0.284077273 | 0.506905224 | NS | 0.546563509 |
| CDC26 | -0.209366408 | 0.010551979 | 0.127927347 | Down | 1.976666081 |
| PRPF4 | -0.25953293 | 1.13E-04 | 0.016924534 | Down | 3.945798596 |
| RNF183 | 0.441982458 | 0.343279733 | NA | NS | 0.464351836 |
| WDR31 | -0.143589877 | 0.524490999 | 0.699830625 | NS | 0.280261961 |
| BSPRY | 0.107243568 | 0.515039145 | 0.693074005 | NS | 0.288159762 |
| HDHD3 | -0.024250931 | 0.758263213 | 0.858906555 | NS | 0.120180013 |
| ALAD | -0.102043747 | 0.172539242 | 0.401370616 | NS | 0.763112114 |
| POLE3 | -0.300043447 | 7.59E-04 | 0.045387814 | Down | 3.119735374 |
| C9orf43 | 0.217493162 | 0.292550264 | 0.515264643 | NS | 0.533799505 |
| RGS3 | 0.150509718 | 0.009095468 | 0.120659125 | Up | 2.041174938 |
| ZNF618 | 0.280541323 | 0.131866403 | 0.354585459 | NS | 0.879865841 |
| AMBP | 0.691770461 | 0.064703543 | NA | NS | 1.189071935 |
| KIF12 | 0.929468374 | 0.065501914 | NA | NS | 1.18374601 |
| COL27A1 | 0.457319543 | 0.139848767 | 0.364064028 | NS | 0.854341359 |
| ORM1 | -0.039218887 | 0.86110972 | 0.921591421 | NS | 0.064941508 |
| ORM2 | 0.001106283 | 0.993089543 | 0.995959744 | NS | 0.003011591 |
| AKNA | 0.033182745 | 0.799355611 | 0.884559442 | NS | 0.097259972 |
| WHRN | 0.490444596 | 0.010927319 | 0.128865835 | Up | 1.961486394 |
| ATP6V1G1 | -0.166641994 | 0.151450093 | 0.377956852 | NS | 0.819730455 |
| TMEM268 | 0.16845899 | 0.016676712 | 0.152211073 | Up | 1.777889573 |
| TEX53 | 0.123242106 | 0.697041527 | NA | NS | 0.156741347 |
| TEX48 | 0.808865114 | 0.034374504 | NA | Up | 1.463763554 |
| TNFSF15 | 0.393918749 | 0.041189058 | 0.218561587 | Up | 1.38521814 |
| TNFSF8 | 0.035346811 | 0.49713617 | 0.679630648 | NS | 0.303524637 |
| TNC | 0.093685073 | 0.632581602 | 0.77617707 | NS | 0.198883443 |
| PAPPA | 0.251460485 | 0.207974702 | 0.437698643 | NS | 0.681989489 |
| ASTN2 | 0.048306409 | 0.592584642 | 0.749252632 | NS | 0.227249608 |
| TRIM32 | -0.039956745 | 0.531820776 | 0.705051196 | NS | 0.274234701 |
| TLR4 | 0.089777745 | 0.476361851 | 0.664249824 | NS | 0.322063026 |
| BRINP1 | 0.618199827 | 0.198121473 | NA | NS | 0.703068451 |
| CDK5RAP2 | 0.040753927 | 0.510364709 | 0.689457474 | NS | 0.292119364 |
| MEGF9 | 0.094959837 | 0.370611891 | 0.58171157 | NS | 0.431080651 |
| FBXW2 | -0.01342952 | 0.787595179 | 0.877481261 | NS | 0.103696951 |
| B3GALT9 | -0.060224353 | 0.308839243 | 0.529238414 | NS | 0.510267521 |
| PSMD5 | -0.002138826 | 0.953647508 | 0.97501943 | NS | 0.020612122 |
| PHF19 | -0.008827003 | 0.859095392 | 0.920338791 | NS | 0.06595861 |
| TRAF1 | 0.055910114 | 0.523604184 | 0.699258443 | NS | 0.280996891 |
| C5 | -0.068537498 | 0.52022471 | 0.696688569 | NS | 0.283809023 |
| CNTRL | 0.051610166 | 0.36627056 | 0.57858521 | NS | 0.436197987 |
| RAB14 | 0.164213198 | 0.002566682 | 0.077754172 | Up | 2.590627915 |
| GSN | 0.029707727 | 0.781597901 | 0.873812361 | NS | 0.107016616 |
| STOM | -0.246489406 | 0.064182126 | 0.262840183 | NS | 1.192585903 |
| GGTA1 | -0.257738263 | 0.066921372 | 0.266591281 | NS | 1.174435161 |
| DAB2IP | 0.69720683 | 0.046169898 | 0.229024017 | Up | 1.335641089 |
| TTLL11 | 0.04119189 | 0.73666911 | 0.844869345 | NS | 0.132727541 |
| NDUFA8 | -0.206520576 | 0.040878945 | 0.218105862 | Down | 1.388500324 |
| MORN5 | 0.280632558 | 0.658017629 | NA | NS | 0.181762471 |
| LHX6 | 0.748421219 | 0.082395991 | 0.289333484 | NS | 1.084093919 |
| RBM18 | -0.104107925 | 0.056555688 | 0.249901284 | NS | 1.247523713 |
| MRRF | 0.011437422 | 0.815941798 | 0.895042583 | NS | 0.088340819 |
| PTGS1 | 0.149798858 | 0.244415948 | 0.472332633 | NS | 0.611870461 |
| OR1J1 | 0.514056565 | 0.165728946 | NA | NS | 0.780601632 |
| OR1J2 | -0.511355745 | 0.288450168 | NA | NS | 0.539929204 |
| OR1J4 | 0.418531266 | 0.407127216 | NA | NS | 0.390269864 |
| OR1N1 | 0.423105408 | 0.374666349 | NA | NS | 0.426355312 |
| OR1N2 | -0.038128462 | 0.932559159 | NA | NS | 0.030323608 |
| OR1L8 | 0.79709039 | 0.018905434 | NA | Up | 1.723413349 |
| OR1Q1 | 0.07730772 | 0.808719149 | NA | NS | 0.092202274 |
| OR1B1 | 0.186057689 | 0.610972079 | NA | NS | 0.213978636 |
| OR1L1 | 0.319360694 | 0.487850637 | NA | NS | 0.311713124 |
| OR1L3 | 0.546096835 | 0.295194924 | NA | NS | 0.529891115 |
| OR1L4 | -0.443525963 | 0.315530155 | NA | NS | 0.500959129 |
| OR1L6 | 0.616923613 | 0.104514423 | NA | NS | 0.980823772 |
| OR5C1 | 0.412484905 | 0.31799621 | NA | NS | 0.497578056 |
| PDCL | -0.255670696 | 5.45E-04 | 0.040010004 | Down | 3.263793885 |
| OR1K1 | 0.402604993 | 0.115365065 | NA | NS | 0.937925683 |
| RC3H2 | -0.046741573 | 0.361353665 | 0.573975818 | NS | 0.442067536 |
| ZBTB6 | -0.123265523 | 0.171953463 | 0.400658716 | NS | 0.764589074 |
| ZBTB26 | 0.047049359 | 0.448108354 | 0.641635461 | NS | 0.34861696 |
| RABGAP1 | 0.017661773 | 0.63382626 | 0.776736637 | NS | 0.198029772 |
| GPR21 | 0.164573541 | 0.163961095 | 0.391802691 | NS | 0.785259191 |
| STRBP | 0.006276589 | 0.938292192 | 0.966009763 | NS | 0.027661898 |
| CRB2 | 0.400048969 | 0.119387593 | 0.340007095 | NS | 0.923040803 |
| DENND1A | -0.094630401 | 0.415044491 | 0.616445599 | NS | 0.381905346 |
| LHX2 | 1.518494594 | 0.004276305 | NA | Up | 2.368931278 |
| NEK6 | 0.185023838 | 0.02482863 | 0.178586374 | Up | 1.605047247 |
| PSMB7 | -0.116460089 | 0.027770304 | 0.186727984 | Down | 1.556419359 |
| ADGRD2 | 1.03167418 | 0.020461188 | 0.165935594 | Up | 1.689069164 |
| NR5A1 | 0.852241111 | 0.048822042 | NA | Up | 1.311384059 |
| NR6A1 | 0.277013918 | 0.045369817 | 0.227426386 | Up | 1.343232972 |
| OLFML2A | 0.830784853 | 0.009494387 | 0.122561596 | Up | 2.022533086 |
| WDR38 | 0.27937424 | 0.086008214 | 0.294830132 | NS | 1.065460072 |
| RPL35 | -0.478020108 | 0.012867049 | 0.136134668 | Down | 1.890521052 |
| ARPC5L | -0.049369188 | 0.431347925 | 0.628258088 | NS | 0.365172287 |
| GOLGA1 | -0.123910955 | 0.222946436 | 0.451651419 | NS | 0.651799465 |
| SCAI | 0.070757962 | 0.273238253 | 0.498621711 | NS | 0.5634585 |
| PPP6C | 0.011056613 | 0.855768092 | 0.918797854 | NS | 0.06764391 |
| RABEPK | -0.095593305 | 0.141863084 | 0.366333756 | NS | 0.848130603 |
| HSPA5 | 0.063367631 | 0.325193254 | 0.543658362 | NS | 0.487858472 |
| GAPVD1 | -0.093476692 | 0.129751206 | 0.351821639 | NS | 0.886888596 |
| MAPKAP1 | -0.115966259 | 0.009599095 | 0.123089851 | Down | 2.017769711 |
| PBX3 | 0.121591346 | 0.006489924 | 0.1080547 | Up | 2.187760366 |
| MVB12B | 0.205940759 | 0.069704584 | 0.270202877 | NS | 1.156738658 |
| LMX1B | 0.683033199 | 0.119435892 | 0.340007095 | NS | 0.922865142 |
| ZBTB43 | 0.007087036 | 0.836432312 | 0.908025084 | NS | 0.077569198 |
| ZBTB34 | 0.103837529 | 0.248672467 | 0.476232204 | NS | 0.604372298 |
| RALGPS1 | 0.134564555 | 0.070347497 | 0.271115037 | NS | 1.152751353 |
| ANGPTL2 | 0.032955849 | 0.828960384 | 0.902732643 | NS | 0.081466224 |
| GARNL3 | 0.096082781 | 0.488143792 | 0.672894495 | NS | 0.311452229 |
| SLC2A8 | 0.159000073 | 0.114965798 | 0.333658762 | NS | 0.939431341 |
| ZNF79 | -0.190026994 | 0.032273198 | 0.198315205 | Down | 1.491158004 |
| RPL12 | -0.284656072 | 0.02138402 | 0.168379028 | Down | 1.669910654 |
| LRSAM1 | 0.035405852 | 0.67551097 | 0.806509938 | NS | 0.170367594 |
| NIBAN2 | -0.136261629 | 0.239427948 | 0.46763242 | NS | 0.620825157 |
| STXBP1 | 0.239232672 | 0.136410616 | 0.360956202 | NS | 0.865151829 |
| PTRH1 | 0.388891776 | 0.247070499 | NA | NS | 0.607179108 |
| CFAP157 | 0.23924604 | 0.170179343 | 0.398511996 | NS | 0.769093156 |
| TTC16 | -0.114077691 | 0.331475942 | 0.54839717 | NS | 0.479547987 |
| TOR2A | 0.190263055 | 0.060870923 | 0.25683088 | NS | 1.215590111 |
| SH2D3C | -0.03070411 | 0.716841539 | 0.832679292 | NS | 0.144576836 |
| CDK9 | -0.012104883 | 0.80274356 | 0.886620118 | NS | 0.09542317 |
| FPGS | -0.11708913 | 0.098759044 | 0.313587396 | NS | 1.005423121 |
| ENG | 0.079458776 | 0.614222736 | 0.764054018 | NS | 0.211674112 |
| AK1 | 0.184253968 | 0.339762181 | 0.555418759 | NS | 0.468824964 |
| ST6GALNAC6 | 0.09052595 | 0.370754499 | 0.581855652 | NS | 0.43091357 |
| ST6GALNAC4 | -0.147258781 | 0.523040952 | 0.698750737 | NS | 0.281464306 |
| PIP5KL1 | 0.304076832 | 0.115399931 | 0.334437496 | NS | 0.93779445 |
| DPM2 | -0.117203337 | 0.512079638 | 0.690696017 | NS | 0.290662493 |
| FAM102A | 0.050797801 | 0.732952315 | 0.842188847 | NS | 0.134924279 |
| NAIF1 | -0.114067301 | 0.063342699 | 0.261443316 | NS | 1.198303435 |
| SLC25A25 | -0.183256571 | 0.00154189 | 0.062836479 | Down | 2.811946652 |
| PTGES2 | -0.033579569 | 0.629915104 | 0.774734714 | NS | 0.200717978 |
| LCN2 | 0.267431654 | 0.177517905 | 0.40748873 | NS | 0.750757836 |
| BBLN | 0.18813221 | 0.054391247 | 0.246520549 | NS | 1.264470985 |
| CIZ1 | -0.119541705 | 0.464353607 | 0.654918093 | NS | 0.333151177 |
| DNM1 | 0.418866174 | 0.093178138 | 0.305594403 | NS | 1.030685973 |
| GOLGA2 | -0.14738736 | 0.039277128 | 0.214632494 | Down | 1.405860277 |
| SWI5 | -0.091399331 | 0.202157069 | 0.432368435 | NS | 0.694311067 |
| TRUB2 | -0.317382622 | 9.45E-06 | 0.002886686 | Down | 5.024412564 |
| COQ4 | 0.162877394 | 0.019102536 | 0.160545426 | Up | 1.718908966 |
| SLC27A4 | -0.209027926 | 0.017036109 | 0.153485826 | Down | 1.768629587 |
| URM1 | 0.237914357 | 0.007021297 | 0.110626998 | Up | 2.153582634 |
| CERCAM | 0.599058518 | 0.015396898 | 0.147618407 | Up | 1.812566767 |
| ODF2 | -0.20843863 | 0.007341264 | 0.112311046 | Down | 2.134229139 |
| GLE1 | -0.067886047 | 0.088218348 | 0.298122562 | NS | 1.054441079 |
| SPTAN1 | 0.025151247 | 0.794107006 | 0.881041916 | NS | 0.100120972 |
| DYNC2I2 | 0.002107201 | 0.984196343 | 0.990467313 | NS | 0.006918253 |
| SET | -0.326119695 | 6.77E-04 | 0.042911362 | Down | 3.169276301 |
| PKN3 | 0.413953946 | 0.038971628 | 0.214288889 | Up | 1.409251457 |
| ZDHHC12 | 0.304429195 | 7.06E-04 | 0.043826722 | Up | 3.151196662 |
| ZER1 | -0.041874601 | 0.8217811 | 0.898521938 | NS | 0.085243851 |
| TBC1D13 | 0.06342423 | 0.491161116 | 0.675427637 | NS | 0.308776023 |
| ENDOG | 0.205119099 | 0.300124022 | 0.521612923 | NS | 0.522699243 |
| SPOUT1 | -0.078864641 | 0.541005207 | 0.71121642 | NS | 0.266798555 |
| KYAT1 | 0.282149124 | 0.161373242 | 0.389034501 | NS | 0.792168477 |
| LRRC8A | -0.175458736 | 0.193156191 | 0.422468062 | NS | 0.714091368 |
| PHYHD1 | 0.342859066 | 0.156300413 | 0.38354142 | NS | 0.806039873 |
| DOLK | -0.013661764 | 0.855897335 | 0.918889966 | NS | 0.067578326 |
| NUP188 | 4.79E-04 | 0.994750759 | 0.996970893 | NS | 0.002285721 |
| SH3GLB2 | -0.176190447 | 0.113930327 | 0.332423088 | NS | 0.943360655 |
| MIGA2 | -0.008754257 | 0.879160535 | 0.931881903 | NS | 0.055931816 |
| DOLPP1 | -0.145854568 | 0.04035308 | 0.216644936 | Down | 1.394123311 |
| CRAT | -0.10644751 | 0.520492057 | 0.696688569 | NS | 0.283585893 |
| PTPA | -0.155898601 | 0.07800817 | 0.282523416 | NS | 1.107859909 |
| IER5L | 0.383746694 | 0.0020415 | 0.071126352 | Up | 2.690050638 |
| NTMT1 | -0.098233843 | 0.186905564 | 0.416370589 | NS | 0.72837777 |
| C9orf50 | 0.452434517 | 0.142047461 | NA | NS | 0.847566526 |
| ASB6 | -0.041086901 | 0.531590641 | 0.704868508 | NS | 0.274422674 |
| PRRX2 | 1.476985913 | 0.010721492 | NA | Up | 1.969744789 |
| PTGES | 0.065207354 | 0.828729724 | 0.902628437 | NS | 0.081587084 |
| TOR1B | -0.110251068 | 0.172586705 | 0.401378778 | NS | 0.762992664 |
| TOR1A | 0.021467355 | 0.692154368 | 0.81722531 | NS | 0.159797036 |
| C9orf78 | -0.246856751 | 0.192037215 | 0.420985576 | NS | 0.7166146 |
| USP20 | -0.117606008 | 0.071584159 | 0.272827495 | NS | 1.145183071 |
| FNBP1 | 0.003069362 | 0.946291811 | 0.971046941 | NS | 0.023974918 |
| GPR107 | 0.009742694 | 0.866232938 | 0.924353124 | NS | 0.062365307 |
| NCS1 | 0.42041894 | 0.204788557 | 0.434924698 | NS | 0.688694315 |
| HMCN2 | 0.720530285 | 0.068926348 | 0.268918437 | NS | 1.161614731 |
| ASS1 | 0.609571725 | 0.12830753 | 0.350046063 | NS | 0.891747854 |
| FUBP3 | -0.111679841 | 0.022131864 | 0.170412614 | Down | 1.654981999 |
| PRDM12 | 0.819873575 | 0.079358994 | NA | NS | 1.100403845 |
| EXOSC2 | -0.127191588 | 0.020352055 | 0.165505627 | Down | 1.691391738 |
| ABL1 | 0.077867641 | 0.306763557 | 0.527638781 | NS | 0.513196236 |
| QRFP | 0.140174524 | 0.353304618 | 0.566794858 | NS | 0.451850686 |
| FIBCD1 | 0.926328674 | 0.012132468 | 0.134107677 | Up | 1.916050828 |
| LAMC3 | 0.703439877 | 0.030057638 | 0.193148154 | Up | 1.522045152 |
| AIF1L | 0.839528867 | 0.010649507 | 0.127927347 | Up | 1.972670492 |
| NUP214 | 0.073831016 | 0.167872457 | 0.39601577 | NS | 0.775020554 |
| FAM78A | -0.051383835 | 0.259314706 | 0.485568555 | NS | 0.586172853 |
| PLPP7 | 0.38545455 | 0.066174737 | 0.2655108 | NS | 1.179307778 |
| PRRC2B | -0.088994901 | 0.59006496 | 0.747557481 | NS | 0.229100175 |
| POMT1 | -0.109480772 | 0.127184518 | 0.348830209 | NS | 0.895565751 |
| UCK1 | -0.100946737 | 0.047661398 | 0.231897457 | Down | 1.321833225 |
| PRRT1B | -0.104726596 | 0.716235498 | 0.832144144 | NS | 0.144944158 |
| RAPGEF1 | 0.052296476 | 0.605658476 | 0.758215285 | NS | 0.2177722 |
| MED27 | 0.1032273 | 0.186977018 | 0.416429803 | NS | 0.728211771 |
| NTNG2 | 0.37337217 | 0.003092479 | 0.083224377 | Up | 2.509693297 |
| SETX | -0.059948091 | 0.303257939 | 0.524458861 | NS | 0.518187821 |
| TTF1 | -0.116869383 | 0.004470893 | 0.093796308 | Down | 2.349605764 |
| CFAP77 | 0.741340562 | 0.067030276 | NA | NS | 1.173728991 |
| BARHL1 | 0.161778621 | 0.663632022 | NA | NS | 0.178072666 |
| DDX31 | -0.050226454 | 0.403629741 | 0.60743385 | NS | 0.394016841 |
| GTF3C4 | -0.003456931 | 0.942323427 | 0.968413333 | NS | 0.025800012 |
| AK8 | 0.259639925 | 0.359139389 | 0.572055243 | NS | 0.444736961 |
| SPACA9 | 0.354501613 | 0.055260912 | 0.24830006 | NS | 1.257581952 |
| TSC1 | 0.03390337 | 0.630134197 | 0.774878296 | NS | 0.200566951 |
| GFI1B | 0.126001501 | 0.384577947 | 0.593184546 | NS | 0.415015624 |
| GTF3C5 | -0.129765988 | 0.008274702 | 0.117003754 | Down | 2.082247659 |
| CEL | 0.71844757 | 0.027342722 | NA | Up | 1.563158252 |
| RALGDS | 0.094396847 | 0.265280936 | 0.491489111 | NS | 0.576293958 |
| GBGT1 | 0.058970812 | 0.540349545 | 0.710599212 | NS | 0.26732521 |
| ABO | 0.005651275 | 0.982410173 | 0.98964515 | NS | 0.007707149 |
| SURF6 | -0.246457967 | 0.003129415 | 0.084119548 | Down | 2.504536885 |
| MED22 | -0.02402592 | 0.774171691 | 0.868227809 | NS | 0.111162713 |
| RPL7A | -0.252293105 | 0.019036315 | 0.160316339 | Down | 1.720417114 |
| SURF1 | -0.058369256 | 0.438775262 | 0.634035652 | NS | 0.357757866 |
| SURF2 | -0.266877673 | 0.011826619 | 0.133168118 | Down | 1.927139389 |
| SURF4 | -0.039887639 | 0.596602542 | 0.75175978 | NS | 0.2243149 |
| REXO4 | -0.265248089 | 0.00205437 | 0.071264622 | Down | 2.687321409 |
| ADAMTS13 | 0.131763084 | 0.593344337 | NA | NS | 0.226693198 |
| CACFD1 | -0.218632861 | 0.150590803 | 0.377054934 | NS | 0.822201552 |
| SLC2A6 | 0.067574465 | 0.589901437 | 0.747465395 | NS | 0.229220546 |
| MYMK | 0.702353532 | 0.185813181 | NA | NS | 0.730923481 |
| ADAMTSL2 | 0.848003251 | 0.017722547 | 0.155687895 | Up | 1.75147386 |
| FAM163B | 1.058677865 | 0.030403672 | NA | Up | 1.517073959 |
| DBH | 0.626800182 | 0.015686634 | 0.148636863 | Up | 1.804470229 |
| SARDH | 0.200490765 | 0.211578803 | 0.44126926 | NS | 0.674527845 |
| VAV2 | 0.141852483 | 0.197606336 | 0.427410856 | NS | 0.704199135 |
| BRD3OS | 0.039951425 | 0.62884991 | 0.773982446 | NS | 0.201452997 |
| BRD3 | 0.053502243 | 0.635044331 | 0.777451503 | NS | 0.197195957 |
| WDR5 | 0.00513065 | 0.916139489 | 0.953437582 | NS | 0.038038397 |
| RXRA | -0.139821977 | 0.211719268 | 0.441352326 | NS | 0.674239616 |
| COL5A1 | 0.59250234 | 0.071668016 | 0.272957474 | NS | 1.144674619 |
| FCN2 | 0.51323849 | 0.281489213 | NA | NS | 0.550538243 |
| FCN1 | -0.219839567 | 0.018394413 | 0.157572037 | Down | 1.735314062 |
| OLFM1 | 0.285017145 | 0.134297515 | 0.357887095 | NS | 0.871932022 |
| PPP1R26 | -0.006972551 | 0.938680802 | 0.966187314 | NS | 0.027482064 |
| C9orf116 | 0.630489601 | 0.014011984 | NA | Up | 1.85350037 |
| MRPS2 | -0.052880781 | 0.518259279 | 0.695486582 | NS | 0.285452914 |
| LCN1 | 0.693050688 | 0.1915628 | NA | NS | 0.717688824 |
| OBP2A | 1.274772449 | 0.010484798 | NA | Up | 1.979439918 |
| PAEP | 0.843541413 | 0.08929611 | NA | NS | 1.049167461 |
| GLT6D1 | 0.341006522 | 0.571966908 | NA | NS | 0.242629097 |
| LCN9 | 0.513372237 | 0.310283847 | NA | NS | 0.508240833 |
| SOHLH1 | 0.843947796 | 0.067372012 | NA | NS | 1.171520484 |
| KCNT1 | 0.768067747 | 0.005732882 | 0.102180893 | Up | 2.241626975 |
| CAMSAP1 | -0.086475144 | 0.089519656 | 0.299870767 | NS | 1.048081594 |
| UBAC1 | -0.21079233 | 0.013295094 | 0.138213457 | Down | 1.876308584 |
| NACC2 | 0.004283843 | 0.957678147 | 0.976655159 | NS | 0.018780423 |
| TMEM250 | -0.089903657 | 0.176966908 | 0.406740698 | NS | 0.752107938 |
| LHX3 | 1.126164963 | 0.040399229 | NA | Up | 1.393626923 |
| QSOX2 | -0.220868189 | 0.067858037 | 0.267853285 | NS | 1.168398708 |
| CCDC187 | 0.619597048 | 0.056215623 | 0.249221499 | NS | 1.250142974 |
| GPSM1 | 0.236288445 | 0.130439343 | 0.35238833 | NS | 0.884591398 |
| DNLZ | 0.353932231 | 0.025437002 | 0.180429975 | Up | 1.594534072 |
| CARD9 | -0.06572993 | 0.554074324 | 0.720989214 | NS | 0.256431975 |
| SNAPC4 | -0.025921469 | 0.850604196 | 0.916084519 | NS | 0.070272479 |
| ENTR1 | -0.128384194 | 0.028142903 | 0.18774505 | Down | 1.550631112 |
| PMPCA | -0.200485701 | 3.55E-04 | 0.032222893 | Down | 3.450348407 |
| INPP5E | 0.207080456 | 0.050652422 | 0.238391305 | NS | 1.295399781 |
| SEC16A | 0.024061898 | 0.845558514 | 0.913184055 | NS | 0.072856333 |
| NOTCH1 | -0.062674586 | 0.712374878 | 0.830016761 | NS | 0.147291404 |
| EGFL7 | 0.331481775 | 0.0470006 | 0.230592916 | Up | 1.327896601 |
| AGPAT2 | 0.012250388 | 0.877019261 | 0.931079927 | NS | 0.056990869 |
| DIPK1B | 0.238759185 | 0.038203114 | 0.213033289 | Up | 1.417901234 |
| LCN10 | 0.059054346 | 0.798187245 | 0.883857702 | NS | 0.097895216 |
| LCN6 | 1.220676944 | 0.044725257 | NA | Up | 1.349447154 |
| LCN8 | 0.110571 | 0.745117502 | 0.850494553 | NS | 0.127775236 |
| LCN15 | 0.78142274 | 0.10054662 | NA | NS | 0.997632524 |
| TMEM141 | 0.054751677 | 0.55637238 | 0.722341233 | NS | 0.254634438 |
| CCDC183 | -0.055915405 | 0.730292311 | 0.840464443 | NS | 0.136503272 |
| RABL6 | -0.009296754 | 0.912545472 | 0.951241312 | NS | 0.039745486 |
| AJM1 | 0.443493485 | 0.009761309 | 0.124334538 | Up | 2.010491937 |
| PHPT1 | -0.353066675 | 7.30E-04 | 0.044580868 | Down | 3.136693626 |
| MAMDC4 | 0.192344856 | 0.023003392 | 0.172649678 | Up | 1.638208126 |
| EDF1 | -0.165414444 | 0.031073273 | 0.19588222 | Down | 1.507612995 |
| TRAF2 | -0.048143391 | 0.422005915 | 0.621527536 | NS | 0.374681462 |
| FBXW5 | 0.013614379 | 0.834765758 | 0.906744706 | NS | 0.078435374 |
| C8G | 0.101722653 | 0.595412596 | 0.750888727 | NS | 0.225181982 |
| LCN12 | 0.970940108 | 1.82E-04 | NA | Up | 3.740136333 |
| PTGDS | 0.026086837 | 0.87556631 | 0.930152131 | NS | 0.057710957 |
| LCNL1 | 0.546803393 | 0.089955004 | NA | NS | 1.045974674 |
| PAXX | -0.104897849 | 0.170663591 | 0.399000229 | NS | 0.76785912 |
| CLIC3 | 0.482842476 | 0.001084859 | 0.053527283 | Up | 2.96462674 |
| ABCA2 | -0.016477159 | 0.906790644 | 0.948236123 | NS | 0.042492969 |
| FUT7 | 0.029636146 | 0.80114928 | 0.885517413 | NS | 0.096286554 |
| NPDC1 | 0.066985315 | 0.6322368 | 0.775917705 | NS | 0.199120229 |
| ENTPD2 | 0.214052113 | 0.489493885 | NA | NS | 0.310252729 |
| SAPCD2 | 0.256128099 | 0.135320295 | 0.359108389 | NS | 0.868637064 |
| UAP1L1 | -0.011935508 | 0.931052506 | 0.962357922 | NS | 0.031025827 |
| MAN1B1 | -0.125652862 | 0.014386097 | 0.143935548 | Down | 1.84205702 |
| DPP7 | -0.093879374 | 0.272199152 | 0.497465358 | NS | 0.565113232 |
| GRIN1 | 0.641576972 | 0.111437713 | 0.329942771 | NS | 0.952967811 |
| LRRC26 | 0.59362576 | 0.296973313 | NA | NS | 0.527282575 |
| TMEM210 | 0.298495981 | 0.597883982 | NA | NS | 0.223383082 |
| ANAPC2 | -0.077341163 | 0.420605592 | 0.620581631 | NS | 0.376124958 |
| SSNA1 | 0.019127644 | 0.770348741 | 0.866009611 | NS | 0.113312623 |
| TPRN | 0.26321742 | 0.009724353 | 0.124070824 | Up | 2.012139292 |
| TMEM203 | -0.284740432 | 0.001582498 | 0.063694842 | Down | 2.800656878 |
| NDOR1 | 0.002710755 | 0.984281623 | 0.990509611 | NS | 0.006880623 |
| RNF208 | 1.677960537 | 6.29E-04 | NA | Up | 3.201466391 |
| CYSRT1 | 0.839098696 | 0.004983819 | NA | Up | 2.302437739 |
| RNF224 | 0.924426649 | 0.010891397 | NA | Up | 1.962916402 |
| SLC34A3 | 0.446325058 | 0.047211844 | 0.230936707 | Up | 1.325949032 |
| TUBB4B | -0.046882453 | 0.559629644 | 0.724472223 | NS | 0.252099289 |
| FAM166A | -0.138998085 | 0.320018177 | 0.539024418 | NS | 0.494825353 |
| STPG3 | 0.871775233 | 0.016333129 | NA | Up | 1.786930601 |
| NELFB | -0.289804826 | 0.001201654 | 0.056242293 | Down | 2.920220488 |
| TOR4A | 0.315523324 | 0.003939469 | 0.090040171 | Up | 2.404562302 |
| NRARP | 0.926767085 | 0.008447867 | NA | Up | 2.073252947 |
| EXD3 | 0.194125091 | 0.102079906 | 0.317962583 | NS | 0.991059737 |
| NOXA1 | 0.238418574 | 0.144755342 | 0.369914478 | NS | 0.8393654 |
| ENTPD8 | 0.979591758 | 0.009038722 | NA | Up | 2.04389296 |
| NSMF | 0.033301413 | 0.714976032 | 0.831377499 | NS | 0.145708517 |
| PNPLA7 | 0.168292179 | 0.385347773 | 0.593556772 | NS | 0.414147147 |
| MRPL41 | 0.021592788 | 0.818760093 | 0.896830016 | NS | 0.086843333 |
| DPH7 | -0.112701343 | 0.03249346 | 0.198993631 | Down | 1.488204042 |
| ZMYND19 | -0.193063003 | 0.003630919 | 0.08688637 | Down | 2.439983445 |
| ARRDC1 | 0.050600393 | 0.494631661 | 0.677817745 | NS | 0.305718088 |
| EHMT1 | -0.080644955 | 0.242777647 | 0.470460443 | NS | 0.614791301 |
| CACNA1B | 0.680528825 | 0.072289895 | 0.273921771 | NS | 1.140922404 |
| TUBB8 | 0.597915733 | 0.159341121 | NA | NS | 0.797672132 |
| ZMYND11 | -0.115336599 | 0.063923709 | 0.262496805 | NS | 1.194338035 |
| DIP2C | 0.091711307 | 0.369343312 | 0.580818264 | NS | 0.43256976 |
| LARP4B | 0.09959041 | 0.148078759 | 0.374097398 | NS | 0.829507233 |
| GTPBP4 | -0.187998376 | 0.002210798 | 0.072832159 | Down | 2.655450856 |
| IDI2 | 0.355982795 | 0.231476523 | NA | NS | 0.63549305 |
| IDI1 | -0.042151745 | 0.699125901 | 0.821855116 | NS | 0.155444608 |
| WDR37 | -0.036163498 | 0.326095669 | 0.544491325 | NS | 0.486654969 |
| ADARB2 | 0.824128593 | 0.022403261 | 0.171057734 | Up | 1.64968877 |
| PFKP | -0.014121244 | 0.835333567 | 0.907146349 | NS | 0.078140067 |
| PITRM1 | -0.013964872 | 0.801539677 | 0.885692448 | NS | 0.096074975 |
| KLF6 | 0.187776965 | 0.001685297 | 0.064977566 | Up | 2.773323527 |
| AKR1E2 | -0.203187313 | 0.316801551 | 0.536323856 | NS | 0.499212701 |
| AKR1C1 | 0.096805261 | 0.560200934 | 0.724761145 | NS | 0.251656171 |
| AKR1C2 | 0.529775484 | 0.046294946 | 0.229292142 | Up | 1.334466414 |
| AKR1C3 | -0.430117529 | 0.00584608 | 0.102935441 | Down | 2.233135214 |
| AKR1C8P | 0.120597535 | 0.527645331 | 0.702525049 | NS | 0.277657901 |
| AKR1C4 | 0.184655016 | 0.34466293 | 0.559617117 | NS | 0.462605425 |
| UCN3 | 0.197188141 | 0.742712224 | NA | NS | 0.129179428 |
| TUBAL3 | 0.564360656 | 0.187677634 | NA | NS | 0.72658748 |
| NET1 | -0.22608763 | 0.01623885 | 0.150699117 | Down | 1.789444716 |
| CALML5 | 1.453680409 | 0.00830511 | NA | Up | 2.080654605 |
| CALML3 | 0.275272922 | 0.653177317 | NA | NS | 0.184968906 |
| ASB13 | -0.279378212 | 0.002955692 | 0.081359678 | Down | 2.529340864 |
| TASOR2 | -0.041754345 | 0.377097764 | 0.587195697 | NS | 0.423546043 |
| GDI2 | -0.154042621 | 0.042624428 | 0.221206584 | Down | 1.370341434 |
| ANKRD16 | 0.056380731 | 0.519217747 | 0.696038682 | NS | 0.284650472 |
| FBH1 | -0.073347479 | 0.119452115 | 0.340007095 | NS | 0.922806155 |
| IL15RA | 0.055640935 | 0.593664759 | 0.749772128 | NS | 0.22645873 |
| IL2RA | 0.001570095 | 0.982105432 | 0.989409763 | NS | 0.007841887 |
| RBM17 | -0.07692171 | 0.040626146 | 0.217533215 | NS | 1.39119437 |
| PFKFB3 | 0.092564437 | 0.477482418 | 0.665285778 | NS | 0.321042616 |
| PRKCQ | -0.067560079 | 0.171100998 | 0.399567153 | NS | 0.766747456 |
| SFMBT2 | 0.019688866 | 0.713431188 | 0.830478881 | NS | 0.146647909 |
| ITIH5 | 0.62070768 | 0.146254157 | 0.371713762 | NS | 0.83489178 |
| ITIH2 | 0.104673033 | 0.444854667 | 0.638990315 | NS | 0.351781849 |
| KIN | -0.056869877 | 0.159936864 | 0.387533163 | NS | 0.796051424 |
| ATP5F1C | -0.283563361 | 0.022558099 | 0.171443155 | Down | 1.646697506 |
| TAF3 | 0.133129197 | 0.070975274 | 0.27207252 | NS | 1.148892924 |
| GATA3 | 0.242652847 | 0.020441858 | 0.165837558 | Up | 1.689479639 |
| CELF2 | 0.224824934 | 0.003000276 | 0.082093562 | Up | 2.522838854 |
| USP6NL | -0.172503003 | 0.087780253 | 0.297553096 | NS | 1.05660317 |
| ECHDC3 | -0.121160622 | 0.425134468 | 0.623663211 | NS | 0.371473683 |
| PROSER2 | 0.182145121 | 0.274696117 | 0.499348937 | NS | 0.561147479 |
| UPF2 | -0.1282705 | 0.075242884 | 0.278206739 | NS | 1.123534564 |
| DHTKD1 | -0.02055931 | 0.696949213 | 0.820297213 | NS | 0.156798868 |
| SEC61A2 | 0.190673784 | 0.022424658 | 0.171132794 | Up | 1.649274174 |
| NUDT5 | 0.002601641 | 0.962440428 | 0.979032249 | NS | 0.016626142 |
| CDC123 | -0.059065336 | 0.427959667 | 0.625834892 | NS | 0.368597159 |
| CAMK1D | 0.140996964 | 0.129112101 | 0.351110762 | NS | 0.889033051 |
| CCDC3 | 0.424483865 | 0.070896659 | 0.27192686 | NS | 1.149374232 |
| OPTN | 0.152028353 | 0.258813956 | 0.485451041 | NS | 0.587012309 |
| MCM10 | -0.613837762 | 0.010633216 | 0.127927347 | Down | 1.973335365 |
| UCMA | 0.778347902 | 0.159485432 | NA | NS | 0.79727898 |
| PHYH | -0.334046138 | 0.002989943 | 0.082074431 | Down | 2.524337069 |
| SEPHS1 | -0.081900058 | 0.212957466 | 0.442773661 | NS | 0.67170713 |
| BEND7 | 0.233710547 | 0.207587777 | 0.437401068 | NS | 0.682798223 |
| PRPF18 | -0.104620045 | 0.031119238 | 0.1959427 | Down | 1.506971047 |
| FRMD4A | 0.059622367 | 0.709726469 | 0.828237227 | NS | 0.148908998 |
| FAM107B | -0.078258758 | 0.189892556 | 0.419275561 | NS | 0.72149206 |
| CDNF | 0.101870866 | 0.497510026 | 0.679709754 | NS | 0.303198163 |
| HSPA14 | -0.165543675 | 0.040489239 | 0.217010192 | Down | 1.392660385 |
| SUV39H2 | 0.019084853 | 0.766634699 | 0.864133669 | NS | 0.115411528 |
| DCLRE1C | -0.024404177 | 0.523399078 | 0.699066057 | NS | 0.281167047 |
| MEIG1 | 0.377359636 | 0.213733869 | NA | NS | 0.670126653 |
| OLAH | -0.421587876 | 0.176502 | NA | NS | 0.753250369 |
| ACBD7 | 0.433886424 | 0.115804822 | 0.334925394 | NS | 0.936273355 |
| RPP38 | -0.027358896 | 0.664733805 | 0.799313956 | NS | 0.177352235 |
| NMT2 | -0.152668486 | 0.028942706 | 0.189889462 | Down | 1.538460867 |
| FAM171A1 | 0.154175075 | 0.217751713 | 0.447318524 | NS | 0.662038419 |
| ITGA8 | 0.504916539 | 0.207161362 | 0.437044477 | NS | 0.683691243 |
| MINDY3 | -0.086455598 | 0.259283871 | 0.485568555 | NS | 0.586224497 |
| PTER | -0.038688094 | 0.589612071 | 0.747377833 | NS | 0.229433634 |
| C1QL3 | 0.277391986 | 0.049313303 | 0.235032736 | Up | 1.307035903 |
| RSU1 | -0.20478139 | 0.034268414 | 0.203267347 | Down | 1.465105991 |
| CUBN | 0.160347086 | 0.166852495 | 0.394803788 | NS | 0.777667294 |
| TRDMT1 | 0.093590606 | 0.105704546 | 0.321835351 | NS | 0.975906336 |
| VIM | -0.253055752 | 0.013220623 | 0.13792184 | Down | 1.878748073 |
| ST8SIA6 | -0.035841351 | 0.724660221 | 0.837894097 | NS | 0.139865578 |
| HACD1 | -0.216253594 | 0.240295848 | 0.468217208 | NS | 0.619253734 |
| STAM | -0.151989257 | 0.080020278 | 0.285455515 | NS | 1.096799944 |
| TMEM236 | -0.072667758 | 0.754418747 | 0.856264156 | NS | 0.122387528 |
| MRC1 | -0.016237579 | 0.930138675 | 0.961856154 | NS | 0.031452297 |
| SLC39A12 | 0.613341855 | 0.181279958 | NA | NS | 0.741650209 |
| CACNB2 | 0.14919288 | 0.102192381 | 0.318076912 | NS | 0.990581481 |
| NSUN6 | -0.09126952 | 0.140439486 | 0.364826818 | NS | 0.85251077 |
| ARL5B | -0.045303729 | 0.494347911 | 0.677612872 | NS | 0.305967297 |
| MALRD1 | 0.363803456 | 0.374283222 | 0.584970385 | NS | 0.42679964 |
| PLXDC2 | 0.117397489 | 0.236842344 | 0.464700343 | NS | 0.62554065 |
| NEBL | -0.512669198 | 0.12952389 | 0.351505645 | NS | 0.88765012 |
| SKIDA1 | 0.506645602 | 0.138624531 | 0.363039337 | NS | 0.858159911 |
| MLLT10 | -0.044562057 | 0.443272129 | 0.637603647 | NS | 0.353329574 |
| DNAJC1 | -0.215406229 | 0.007839039 | 0.11472857 | Down | 2.105737177 |
| EBLN1 | 0.605565701 | 0.234183242 | NA | NS | 0.630444186 |
| COMMD3 | -0.163068531 | 0.104995297 | 0.320907849 | NS | 0.978830153 |
| BMI1 | -0.254794105 | 0.008984759 | 0.120055109 | Down | 2.046493567 |
| SPAG6 | 0.30720148 | 0.217667907 | 0.447248377 | NS | 0.662205598 |
| PIP4K2A | -0.246667019 | 0.110623688 | 0.3291588 | NS | 0.956151867 |
| ARMC3 | -0.221084999 | 0.199568029 | 0.429155587 | NS | 0.699909032 |
| MSRB2 | 0.011057825 | 0.911135675 | 0.950817021 | NS | 0.040416948 |
| PTF1A | 0.946774077 | 0.072506456 | NA | NS | 1.139623323 |
| C10orf67 | 0.518352663 | 0.192693099 | 0.421737132 | NS | 0.715133839 |
| OTUD1 | -0.055153053 | 0.438986155 | 0.634216618 | NS | 0.357549177 |
| KIAA1217 | 0.642498908 | 0.100560175 | 0.316002898 | NS | 0.997573981 |
| ARHGAP21 | 0.024037337 | 0.73072897 | 0.840624617 | NS | 0.136243675 |
| PRTFDC1 | -0.018699363 | 0.905028495 | 0.947388363 | NS | 0.043337747 |
| ENKUR | -0.177000658 | 0.151958209 | 0.378507048 | NS | 0.818275835 |
| THNSL1 | -0.176812323 | 0.082910998 | 0.290260272 | NS | 1.081387859 |
| GPR158 | 0.457787941 | 0.268262821 | 0.493950404 | NS | 0.571439513 |
| MYO3A | 0.51677869 | 0.162940798 | 0.390737932 | NS | 0.787970161 |
| GAD2 | 0.702409809 | 0.123707329 | 0.344659912 | NS | 0.907604569 |
| APBB1IP | 0.229102408 | 0.004599495 | 0.094750689 | Up | 2.337289888 |
| PDSS1 | 0.159125122 | 0.008917768 | 0.11966441 | Up | 2.049743826 |
| ABI1 | -0.046604134 | 0.414545255 | 0.616134093 | NS | 0.382428052 |
| ANKRD26 | -0.207316869 | 0.033910721 | 0.202615011 | Down | 1.469662973 |
| YME1L1 | -0.181729822 | 6.02E-04 | 0.040785137 | Down | 3.220454781 |
| MASTL | -0.22033385 | 0.066313857 | 0.265556907 | NS | 1.178395712 |
| ACBD5 | -0.10434302 | 0.027604294 | 0.186639939 | Down | 1.55902336 |
| RAB18 | 0.027811037 | 0.61584485 | 0.765059537 | NS | 0.210528686 |
| MKX | 0.527045367 | 0.020143695 | 0.164578764 | Up | 1.695860859 |
| ODAD2 | 0.460428213 | 0.178640385 | 0.40855024 | NS | 0.748020354 |
| MPP7 | 0.163262462 | 0.005977716 | 0.103400032 | Up | 2.223464751 |
| WAC | -0.038252922 | 0.509218003 | 0.688476929 | NS | 0.293096251 |
| BAMBI | 0.286635121 | 0.105731556 | 0.321845781 | NS | 0.975795377 |
| LYZL1 | 0.37932464 | 0.452485177 | NA | NS | 0.344395643 |
| SVIL | 0.129263594 | 0.240252922 | 0.468217208 | NS | 0.619331321 |
| JCAD | 0.628405658 | 0.061046542 | 0.256954217 | NS | 1.214338934 |
| MTPAP | -0.265024084 | 0.004248432 | 0.092664376 | Down | 2.371771304 |
| MAP3K8 | -0.041931975 | 0.494196327 | 0.677463766 | NS | 0.306100487 |
| LYZL2 | 0.478452617 | 0.364253819 | NA | NS | 0.438595886 |
| ZNF438 | 0.144543124 | 0.155188188 | 0.382558975 | NS | 0.809141337 |
| ZEB1 | -0.026832709 | 0.619749649 | 0.767465473 | NS | 0.207783711 |
| ARHGAP12 | -0.126230783 | 0.054780109 | 0.247094287 | NS | 1.261377104 |
| KIF5B | -0.112043569 | 0.063874937 | 0.262397294 | NS | 1.194669516 |
| EPC1 | -0.039837645 | 0.194150676 | 0.423635716 | NS | 0.711861094 |
| CCDC7 | -0.256366667 | 0.122208133 | 0.342932892 | NS | 0.91289989 |
| ITGB1 | -0.15769465 | 0.033561752 | 0.201715468 | Down | 1.474155373 |
| NRP1 | 0.111245049 | 0.536596304 | 0.708403046 | NS | 0.270352323 |
| PARD3 | -0.177387283 | 0.242472277 | 0.470203225 | NS | 0.615337909 |
| CUL2 | 0.026268059 | 0.631932844 | 0.775881949 | NS | 0.199329072 |
| CREM | -0.085944803 | 0.229675561 | 0.458066753 | NS | 0.638885213 |
| CCNY | -0.090111999 | 0.141037486 | 0.365424845 | NS | 0.850665443 |
| GJD4 | 0.904662388 | 0.228600334 | NA | NS | 0.640923139 |
| FZD8 | 0.419626675 | 0.068463117 | NA | NS | 1.164543335 |
| ANKRD30A | 0.416966956 | 0.396657822 | NA | NS | 0.401583977 |
| MTRNR2L7 | 0.792009979 | 0.117826276 | NA | NS | 0.928757847 |
| ZNF248 | -0.001397116 | 0.98471467 | 0.990727732 | NS | 0.006689592 |
| ZNF25 | -0.120216674 | 0.079665932 | 0.285035021 | NS | 1.09872736 |
| ZNF33A | -0.017829649 | 0.780178447 | 0.872869897 | NS | 0.107806051 |
| ZNF37A | -0.1736743 | 0.005328685 | 0.099541221 | Down | 2.273379976 |
| PLD5P1 | -0.01593636 | 0.888787746 | 0.937284937 | NS | 0.051201942 |
| ZNF33B | -0.037292258 | 0.537283299 | 0.708764594 | NS | 0.269796659 |
| BMS1 | -0.150526248 | 0.005492852 | 0.100721984 | Down | 2.260202093 |
| RET | 0.566546167 | 0.022068442 | 0.170172208 | Up | 1.656228327 |
| CSGALNACT2 | 0.025178793 | 0.720687362 | 0.835096644 | NS | 0.142253094 |
| RASGEF1A | 0.105305871 | 0.299164838 | 0.520433287 | NS | 0.524089452 |
| FXYD4 | 0.999543941 | 0.080934011 | NA | NS | 1.091868935 |
| HNRNPF | -0.142802503 | 0.073747105 | 0.275940596 | NS | 1.132255023 |
| ZNF487 | 0.031680981 | 0.622375332 | 0.76946879 | NS | 0.205947629 |
| ZNF239 | -0.0565308 | 0.587815366 | 0.746280144 | NS | 0.230759065 |
| ZNF485 | -0.137438908 | 0.084333565 | 0.29207932 | NS | 1.073999543 |
| ZNF32 | -0.227349428 | 0.068634313 | 0.268602708 | NS | 1.163458706 |
| CXCL12 | 0.610531782 | 0.148748305 | NA | NS | 0.827547974 |
| TMEM72 | 0.401758919 | 0.061355715 | NA | NS | 1.212144978 |
| RASSF4 | -0.051007326 | 0.572113539 | 0.733952836 | NS | 0.242517775 |
| DEPP1 | -0.057237754 | 0.752684598 | 0.854945906 | NS | 0.123386971 |
| ZNF22 | -0.234525802 | 0.072721704 | 0.274345075 | NS | 1.138335953 |
| ALOX5 | 0.010448719 | 0.913201222 | 0.951687128 | NS | 0.039433516 |
| MARCHF8 | -0.326828229 | 0.092539596 | 0.304765864 | NS | 1.033672402 |
| ZFAND4 | -0.062940727 | 0.588939666 | 0.746923544 | NS | 0.229929194 |
| WASHC2C | 0.022021268 | 0.829568452 | 0.903191142 | NS | 0.081147772 |
| AGAP4 | -0.104864459 | 0.459094619 | 0.650509495 | NS | 0.338097797 |
| TIMM23 | -0.200274268 | 0.015586907 | 0.148490537 | Down | 1.807240047 |
| NCOA4 | 7.19E-04 | 0.995424902 | 0.997330139 | NS | 0.001991499 |
| MSMB | 0.203703082 | 0.720548363 | NA | NS | 0.142336864 |
| ANTXRL | 0.373284243 | 0.307885985 | 0.528756098 | NS | 0.51161008 |
| ANXA8L1 | 0.826106587 | 0.013043867 | NA | Up | 1.884593636 |
| NPY4R | 0.451441326 | 0.561578105 | NA | NS | 0.250589833 |
| GPRIN2 | 0.684640337 | 0.13970104 | NA | NS | 0.854800362 |
| SYT15 | 0.305478975 | 0.19693107 | 0.426649832 | NS | 0.705685758 |
| PTPN20 | 0.428911023 | 0.097316306 | 0.311668025 | NS | 1.011814383 |
| GDF10 | 0.888527901 | 0.003480523 | NA | Up | 2.458355522 |
| GDF2 | 0.63147894 | 0.17067943 | NA | NS | 0.767818816 |
| RBP3 | 1.071260613 | 0.029376792 | NA | Up | 1.531995629 |
| ZNF488 | 0.789418659 | 0.103230749 | NA | NS | 0.986190923 |
| ANXA8 | 0.538192989 | 0.150914776 | NA | NS | 0.821268236 |
| FAM25G | 0.995659264 | 0.145997941 | NA | NS | 0.83565327 |
| AGAP9 | 0.151183206 | 0.311628955 | 0.531287585 | NS | 0.506362197 |
| FRMPD2 | 0.285357904 | 0.510712737 | 0.689642871 | NS | 0.291823311 |
| MAPK8 | -0.048862742 | 0.315525367 | 0.534993851 | NS | 0.50096572 |
| ARHGAP22 | 0.100391047 | 0.423881161 | 0.622809158 | NS | 0.372755885 |
| WDFY4 | 0.122223603 | 0.207648245 | 0.437401068 | NS | 0.682671734 |
| LRRC18 | -0.075103503 | 0.569162511 | 0.731710119 | NS | 0.244763713 |
| VSTM4 | 0.705592585 | 0.043033576 | 0.222724285 | Up | 1.366192562 |
| FAM170B | 0.636975538 | 0.030217529 | NA | Up | 1.519741056 |
| TMEM273 | 0.20053702 | 0.07111308 | 0.272300912 | NS | 1.148050512 |
| C10orf71 | 0.632091371 | 0.159429876 | NA | NS | 0.797430291 |
| DRGX | 0.725484859 | 0.14215623 | NA | NS | 0.847234102 |
| ERCC6 | -0.052148798 | 0.312117628 | 0.531854011 | NS | 0.505681703 |
| CHAT | 0.539900888 | 0.214178282 | 0.443419982 | NS | 0.669224568 |
| SLC18A3 | 1.156304058 | 0.025249917 | NA | Up | 1.59774005 |
| C10orf53 | 0.652287909 | 0.15534063 | NA | NS | 0.808714937 |
| OGDHL | 1.006347616 | 0.022653329 | 0.171563006 | Up | 1.644867976 |
| PARG | -0.202614571 | 0.005250732 | 0.099054589 | Down | 2.279780123 |
| TIMM23B | 0.119022942 | 0.146631816 | 0.372132189 | NS | 0.833771788 |
| TIMM23B-AGAP6 | 0.301362944 | 0.067534451 | 0.267434379 | NS | 1.170474624 |
| AGAP6 | 0.184913398 | 0.123097022 | 0.343806623 | NS | 0.909752453 |
| WASHC2A | -0.020427048 | 0.744553232 | 0.850164936 | NS | 0.128104247 |
| ASAH2 | 0.066656002 | 0.763770839 | 0.862433673 | NS | 0.117036927 |
| SGMS1 | -0.179832905 | 0.01398483 | 0.141433221 | Down | 1.854342812 |
| ASAH2B | -0.058613036 | 0.558651002 | 0.72406582 | NS | 0.252859418 |
| A1CF | 0.609897966 | 0.052919841 | 0.243123408 | NS | 1.276381472 |
| PRKG1 | 0.18791706 | 0.318185204 | 0.537474372 | NS | 0.497320019 |
| CSTF2T | -0.154156477 | 0.040772575 | 0.217865963 | Down | 1.389631858 |
| DKK1 | 0.573734004 | 0.284721526 | NA | NS | 0.545579697 |
| MBL2 | 0.759230013 | 0.169574512 | NA | NS | 0.770639423 |
| PCDH15 | 0.373150223 | 0.401004173 | 0.605756874 | NS | 0.396851108 |
| MTRNR2L5 | 0.748955354 | 0.232119225 | NA | NS | 0.634288889 |
| ZWINT | 0.002367738 | 0.984653258 | 0.990727732 | NS | 0.006716678 |
| IPMK | -0.010014649 | 0.926510232 | 0.959655254 | NS | 0.03314978 |
| CISD1 | -0.262232178 | 0.008900648 | 0.11966441 | Down | 2.050578392 |
| UBE2D1 | 0.069254522 | 0.569244656 | 0.731710119 | NS | 0.244701038 |
| TFAM | -0.273225649 | 0.001256134 | 0.057535957 | Down | 2.900964068 |
| BICC1 | 0.397692326 | 0.125971363 | 0.347710491 | NS | 0.89972817 |
| PHYHIPL | 0.871312929 | 0.083985273 | NA | NS | 1.075796864 |
| FAM13C | 0.309199009 | 0.526615684 | NA | NS | 0.278506211 |
| SLC16A9 | 0.255976519 | 0.447299716 | NA | NS | 0.349401377 |
| MRLN | 0.182924194 | 0.698743148 | NA | NS | 0.155682438 |
| CCDC6 | 0.077553566 | 0.122900176 | 0.343701955 | NS | 0.910447496 |
| ANK3 | 0.067739519 | 0.471577337 | 0.660409087 | NS | 0.326447075 |
| CDK1 | 0.083942082 | 0.586855582 | 0.745392188 | NS | 0.23146876 |
| RHOBTB1 | -0.133225939 | 0.355918704 | 0.568903557 | NS | 0.448649188 |
| TMEM26 | 0.162493622 | 0.755856653 | NA | NS | 0.12156056 |
| CABCOCO1 | 0.459443185 | 0.240273369 | NA | NS | 0.619294362 |
| ARID5B | 0.059210274 | 0.30103261 | 0.522450996 | NS | 0.521386456 |
| RTKN2 | 0.110400628 | 0.325121738 | 0.543578482 | NS | 0.487953992 |
| ZNF365 | -0.31992553 | 0.035218408 | 0.205507211 | Down | 1.453230279 |
| ADO | -0.038988276 | 0.600086787 | 0.754415898 | NS | 0.221785936 |
| EGR2 | 0.451807059 | 0.012881524 | 0.136134668 | Up | 1.890032748 |
| NRBF2 | 0.184633172 | 0.110017298 | 0.328459933 | NS | 0.958539027 |
| JMJD1C | 0.114412852 | 0.139643831 | 0.363934068 | NS | 0.854978245 |
| REEP3 | -0.073724511 | 0.282042084 | 0.505014315 | NS | 0.549686084 |
| CTNNA3 | 0.494956073 | 0.143901693 | 0.369093579 | NS | 0.841934098 |
| LRRTM3 | 0.150641503 | 0.702970004 | NA | NS | 0.153063206 |
| DNAJC12 | 0.169894454 | 0.639480687 | NA | NS | 0.194172567 |
| SIRT1 | -0.158363505 | 0.002105413 | 0.071967411 | Down | 2.676662729 |
| HERC4 | 0.0419171 | 0.233043042 | 0.461411926 | NS | 0.632563859 |
| MYPN | 0.461553841 | 0.304569221 | 0.525838243 | NS | 0.516313988 |
| ATOH7 | 0.68004627 | 0.217557231 | NA | NS | 0.662426478 |
| PBLD | 0.036257096 | 0.603519126 | 0.756695228 | NS | 0.219308962 |
| HNRNPH3 | 0.132892926 | 0.034744557 | 0.204397599 | Up | 1.459113217 |
| RUFY2 | -0.163680668 | 0.096133964 | 0.310224046 | NS | 1.017123148 |
| DNA2 | -0.447792146 | 9.68E-04 | 0.051639949 | Down | 3.014220428 |
| SLC25A16 | -0.06340746 | 0.099106796 | 0.314049068 | NS | 1.003896563 |
| TET1 | 0.108477545 | 0.207146606 | 0.437044477 | NS | 0.683722177 |
| CCAR1 | -0.073662212 | 0.27333076 | 0.498634409 | NS | 0.563311492 |
| STOX1 | -0.130972326 | 0.630915874 | 0.775256753 | NS | 0.200028545 |
| DDX50 | -0.22737239 | 0.012373522 | 0.134877872 | Down | 1.907506654 |
| DDX21 | -0.226011429 | 7.15E-04 | 0.044011002 | Down | 3.145769212 |
| KIFBP | 0.056701101 | 0.171662267 | 0.400224904 | NS | 0.765325157 |
| SRGN | 0.038642874 | 0.793273869 | 0.880637817 | NS | 0.100576851 |
| VPS26A | -0.122434025 | 0.060101995 | 0.255813968 | NS | 1.221111114 |
| SUPV3L1 | -0.157870246 | 0.001288065 | 0.058646647 | Down | 2.890062241 |
| HKDC1 | -0.174286104 | 0.170821471 | 0.399158589 | NS | 0.767457543 |
| HK1 | -0.190846165 | 0.186741901 | 0.416311011 | NS | 0.728758224 |
| TACR2 | 0.232546914 | 0.145159631 | 0.370494358 | NS | 0.838154144 |
| TSPAN15 | -0.051275856 | 0.675868265 | 0.806704534 | NS | 0.170137945 |
| NEUROG3 | 1.234047398 | 0.009709412 | NA | Up | 2.012807064 |
| FAM241B | 0.156684435 | 0.302929714 | 0.524311995 | NS | 0.518658125 |
| COL13A1 | -0.024212507 | 0.929002029 | 0.961212294 | NS | 0.031983337 |
| MACROH2A2 | 0.269063014 | 0.004354094 | 0.093367359 | Up | 2.361102184 |
| AIFM2 | 0.193106892 | 0.04168547 | 0.220175424 | Up | 1.380015296 |
| TYSND1 | 0.05452095 | 0.320640324 | 0.539353999 | NS | 0.493981862 |
| SAR1A | -0.116432725 | 0.054345623 | 0.246476974 | NS | 1.26483543 |
| PPA1 | -0.279736622 | 0.058353681 | 0.252212565 | NS | 1.233931747 |
| NPFFR1 | 0.72840081 | 0.084391081 | 0.292173023 | NS | 1.073703448 |
| LRRC20 | -0.006449055 | 0.956417478 | 0.976165401 | NS | 0.019352496 |
| EIF4EBP2 | 0.00805653 | 0.925885828 | 0.959365989 | NS | 0.033442563 |
| NODAL | 0.27926154 | 0.075334874 | 0.278406106 | NS | 1.123003933 |
| PALD1 | 0.500718541 | 0.035136118 | 0.205472308 | Up | 1.454246226 |
| PRF1 | -0.144908623 | 0.285404379 | 0.50858474 | NS | 0.544539368 |
| ADAMTS14 | 0.575306137 | 0.102027788 | 0.317910259 | NS | 0.991281528 |
| TBATA | 0.526193825 | 0.240626968 | NA | NS | 0.618655701 |
| SGPL1 | 0.024091492 | 0.644375503 | 0.784889255 | NS | 0.190860978 |
| PCBD1 | -0.115874837 | 0.182259611 | 0.412292544 | NS | 0.73930956 |
| UNC5B | 0.773909399 | 0.052749627 | 0.242682194 | NS | 1.277780605 |
| SLC29A3 | 0.098380308 | 0.397381129 | 0.602512142 | NS | 0.40079276 |
| CDH23 | 0.040135379 | 0.685436383 | 0.813023826 | NS | 0.164032847 |
| C10orf105 | 0.211249808 | 0.170020777 | 0.398309642 | NS | 0.769498004 |
| VSIR | 0.154153867 | 0.100129555 | 0.315211969 | NS | 0.999437713 |
| PSAP | 0.185227934 | 0.040484186 | 0.217010192 | Up | 1.392714585 |
| CHST3 | 1.013063356 | 0.023561879 | NA | Up | 1.627790081 |
| SPOCK2 | 0.025394825 | 0.766187358 | 0.863884545 | NS | 0.115665018 |
| ASCC1 | 0.098101507 | 0.059015109 | 0.253419931 | NS | 1.229036784 |
| ANAPC16 | -0.233898647 | 9.20E-04 | 0.050757919 | Down | 3.036078981 |
| DDIT4 | -0.179344296 | 0.1594718 | 0.387098757 | NS | 0.797316105 |
| DNAJB12 | -0.04509507 | 0.397862627 | 0.602954599 | NS | 0.400266854 |
| MICU1 | 0.130458803 | 0.021252439 | 0.168379028 | Up | 1.672591224 |
| MCU | 0.041911021 | 0.427974038 | 0.625834892 | NS | 0.368582576 |
| OIT3 | 0.420573921 | 0.111117541 | 0.329681815 | NS | 0.954217376 |
| PLA2G12B | 0.648931112 | 0.149104071 | NA | NS | 0.8265105 |
| P4HA1 | -0.152888952 | 0.017878578 | 0.156161405 | Down | 1.747667017 |
| NUDT13 | 0.094771601 | 0.125910439 | 0.347631209 | NS | 0.899938261 |
| ECD | -0.012817092 | 0.67773938 | 0.807700853 | NS | 0.168937279 |
| FAM149B1 | -0.02181883 | 0.578535818 | 0.738428764 | NS | 0.237669748 |
| DNAJC9 | -0.144353876 | 0.018951547 | 0.159745428 | Down | 1.722355335 |
| MRPS16 | -0.360532778 | 2.50E-05 | 0.006155615 | Down | 4.60211903 |
| CFAP70 | 0.022409704 | 0.832546056 | 0.904848604 | NS | 0.079591732 |
| ANXA7 | -0.123248563 | 0.068234723 | 0.268476763 | NS | 1.165994565 |
| MSS51 | 0.074867415 | 0.353492919 | 0.566965111 | NS | 0.451619282 |
| PPP3CB | 0.001052086 | 0.990088074 | 0.994124998 | NS | 0.004326171 |
| USP54 | 0.080182415 | 0.392756098 | 0.599318562 | NS | 0.405877064 |
| MYOZ1 | 0.504468563 | 0.262641903 | NA | NS | 0.580635983 |
| SYNPO2L | 0.744208315 | 0.051729431 | 0.24015089 | NS | 1.286262297 |
| AGAP5 | -0.001946587 | 0.99152968 | NA | NS | 0.003694281 |
| SEC24C | -0.033380965 | 0.533378277 | 0.706053367 | NS | 0.272964676 |
| FUT11 | -0.052581364 | 0.398175927 | 0.603175118 | NS | 0.399925 |
| CHCHD1 | -0.126369537 | 0.223866486 | 0.452437558 | NS | 0.650010917 |
| ZSWIM8 | -0.082514081 | 0.418338628 | 0.618901466 | NS | 0.378472032 |
| NDST2 | 0.115609466 | 0.47100751 | 0.659998409 | NS | 0.326972168 |
| CAMK2G | 0.079548685 | 0.23878304 | 0.466925155 | NS | 0.621996524 |
| PLAU | 0.149358112 | 0.438167997 | 0.633591488 | NS | 0.358359346 |
| VCL | 0.05497607 | 0.542901226 | 0.712890538 | NS | 0.265279178 |
| AP3M1 | -0.023660821 | 0.632116505 | 0.775886882 | NS | 0.19920287 |
| ADK | -0.038978587 | 0.530570386 | 0.704623132 | NS | 0.275256994 |
| KAT6B | 0.06183375 | 0.373239947 | 0.583828339 | NS | 0.428011881 |
| DUSP13 | 0.333254721 | 0.25828317 | NA | NS | 0.587903892 |
| SAMD8 | 0.071794045 | 0.321493387 | 0.54003532 | NS | 0.492827956 |
| VDAC2 | -0.162954408 | 0.014391956 | 0.143935548 | Down | 1.841880175 |
| COMTD1 | 0.089071004 | 0.438505548 | 0.633805873 | NS | 0.358024908 |
| ZNF503 | 0.493375735 | 0.056427638 | NA | NS | 1.24850813 |
| LRMDA | 0.281651532 | 0.042480106 | 0.221095683 | Up | 1.371814408 |
| KCNMA1 | 0.637665561 | 0.023309882 | 0.173494612 | Up | 1.632459921 |
| DLG5 | -0.163452477 | 0.348409008 | 0.562633134 | NS | 0.457910624 |
| POLR3A | 0.009970494 | 0.825881576 | 0.900867904 | NS | 0.083082222 |
| RPS24 | -0.796508547 | 5.59E-04 | 0.040125454 | Down | 3.252939236 |
| ZMIZ1 | 0.086929115 | 0.5013995 | 0.682864613 | NS | 0.299816104 |
| PPIF | 0.092812512 | 0.189455488 | 0.418854096 | NS | 0.722492811 |
| ZCCHC24 | 0.253298686 | 0.015663967 | 0.148636863 | Up | 1.805098236 |
| EIF5AL1 | -0.249435448 | 0.348968531 | NA | NS | 0.457213734 |
| SFTPA2 | 0.415342518 | 0.458449292 | NA | NS | 0.338708694 |
| SFTPA1 | 0.630213097 | 0.218445104 | NA | NS | 0.660657685 |
| NUTM2B | 0.428345846 | 0.065630838 | 0.265046281 | NS | 1.182892049 |
| NUTM2E | -0.094738856 | 0.504045572 | 0.684785733 | NS | 0.297530196 |
| SFTPD | 0.27404576 | 0.24720449 | 0.475460066 | NS | 0.606943645 |
| TMEM254 | 0.12181645 | 0.071197093 | 0.272440404 | NS | 1.147537739 |
| PLAC9 | 0.206034563 | 0.053073778 | 0.243362019 | NS | 1.275119993 |
| ANXA11 | 0.101825456 | 0.132258081 | 0.355263262 | NS | 0.878577782 |
| MAT1A | 0.577308093 | 0.246591287 | NA | NS | 0.608022273 |
| DYDC1 | 1.32544503 | 0.018362441 | NA | Up | 1.736069577 |
| DYDC2 | 0.883284003 | 0.124103975 | NA | NS | 0.906214307 |
| PRXL2A | -0.137264921 | 0.197736903 | 0.427465598 | NS | 0.703912273 |
| TSPAN14 | 0.015557816 | 0.827233915 | 0.901708524 | NS | 0.082371669 |
| SH2D4B | 0.438365826 | 0.085153119 | 0.29348032 | NS | 1.069799442 |
| NRG3 | 0.721632787 | 0.15313961 | 0.379723802 | NS | 0.814912463 |
| GHITM | -0.126588481 | 0.08074584 | 0.286481987 | NS | 1.092879844 |
| C10orf99 | 0.037510397 | 0.961001675 | NA | NS | 0.017275855 |
| CDHR1 | 0.301412615 | 0.16407146 | 0.391953477 | NS | 0.784966958 |
| LRIT2 | 1.019317296 | 0.033291536 | NA | Up | 1.477666173 |
| RGR | 0.297201735 | 0.534937531 | NA | NS | 0.271696931 |
| LRIT1 | 1.072293986 | 0.070443195 | NA | NS | 1.152160953 |
| CCSER2 | -0.044348297 | 0.46285436 | 0.653532094 | NS | 0.334555641 |
| GRID1 | 0.743215021 | 0.023162285 | 0.173122445 | Up | 1.635218608 |
| WAPL | -0.160759487 | 0.001209273 | 0.056242293 | Down | 2.917475775 |
| OPN4 | 0.687769202 | 0.175841408 | NA | NS | 0.754878848 |
| LDB3 | 0.448863398 | 0.180273852 | 0.410149052 | NS | 0.74406726 |
| BMPR1A | -0.051567629 | 0.423677068 | 0.622710628 | NS | 0.372965042 |
| MMRN2 | 0.272398836 | 0.096256123 | 0.310402489 | NS | 1.016571633 |
| SNCG | 0.139600616 | 0.700591208 | NA | NS | 0.154535317 |
| GLUD1 | -0.176018883 | 0.016768439 | 0.152541471 | Down | 1.775507369 |
| SHLD2 | -0.249806642 | 0.008554876 | 0.118335308 | Down | 2.067786289 |
| NUTM2A | -0.082520163 | 0.660167173 | 0.795828435 | NS | 0.180346075 |
| NUTM2D | 0.215701255 | 0.133716223 | 0.356961061 | NS | 0.873815899 |
| MINPP1 | -0.515073444 | 4.65E-04 | 0.036600787 | Down | 3.332885215 |
| PAPSS2 | -0.051522312 | 0.425584321 | 0.623837924 | NS | 0.37101438 |
| ATAD1 | -0.071315887 | 0.294070591 | 0.516156091 | NS | 0.531548406 |
| PTEN | -0.035194061 | 0.683788694 | 0.811826265 | NS | 0.165078085 |
| RNLS | 0.03306974 | 0.665013449 | 0.799449198 | NS | 0.177169571 |
| LIPJ | 0.147208184 | 0.489732603 | 0.674313477 | NS | 0.310040983 |
| LIPF | 0.620908973 | 0.105965229 | NA | NS | 0.974836621 |
| LIPK | 0.464443438 | 0.138703679 | NA | NS | 0.857912019 |
| LIPN | 0.154383437 | 0.224422978 | 0.452840091 | NS | 0.64893268 |
| LIPM | 0.631981441 | 0.039718963 | NA | Up | 1.401002103 |
| ANKRD22 | 0.110284791 | 0.539859392 | 0.710280503 | NS | 0.267719339 |
| STAMBPL1 | 0.048211723 | 0.406935124 | 0.610522647 | NS | 0.390474824 |
| ACTA2 | 0.157317074 | 0.084060903 | 0.292007269 | NS | 1.075405951 |
| FAS | 0.145446128 | 0.109470135 | 0.327595066 | NS | 0.960704345 |
| CH25H | 0.459847446 | 0.437373777 | NA | NS | 0.359147259 |
| LIPA | -0.2002694 | 0.051125367 | 0.239125809 | NS | 1.291363562 |
| IFIT2 | 0.190943735 | 0.259809662 | 0.485843002 | NS | 0.585344702 |
| IFIT3 | 0.095403429 | 0.561246511 | 0.725674256 | NS | 0.250846346 |
| IFIT1B | 0.03940485 | 0.858040211 | 0.919794085 | NS | 0.066492359 |
| IFIT1 | 0.516982983 | 0.01276624 | 0.1360506 | Up | 1.893936992 |
| IFIT5 | 0.192195997 | 0.113315205 | 0.332115589 | NS | 0.945711809 |
| SLC16A12 | 0.189301938 | 0.613506741 | NA | NS | 0.212180661 |
| PANK1 | 1.48E-04 | 0.998751391 | 0.999521155 | NS | 5.43E-04 |
| KIF20B | -0.627293022 | 1.58E-04 | 0.02029709 | Down | 3.802019641 |
| HTR7 | 0.088382618 | 0.551940954 | 0.719152969 | NS | 0.25810738 |
| RPP30 | -0.095444821 | 0.090493119 | 0.301231602 | NS | 1.043384441 |
| ANKRD1 | 0.040293244 | 0.856667668 | 0.919321075 | NS | 0.067187624 |
| PCGF5 | -0.090501272 | 0.231192058 | 0.459654528 | NS | 0.636027089 |
| HECTD2 | 0.050922605 | 0.567263407 | 0.730596477 | NS | 0.246215231 |
| PPP1R3C | 0.415608856 | 0.37048335 | NA | NS | 0.431231305 |
| TNKS2 | -0.016538624 | 0.742438654 | 0.848935546 | NS | 0.129339425 |
| FGFBP3 | 0.035830954 | 0.733049818 | 0.842188847 | NS | 0.13486651 |
| BTAF1 | -0.070529455 | 0.105224543 | 0.32124631 | NS | 0.977882951 |
| CPEB3 | 0.248356773 | 3.48E-05 | 0.007962653 | Up | 4.458815641 |
| MARCHF5 | 0.178801918 | 0.002741045 | 0.079323886 | Up | 2.562083812 |
| IDE | -0.124814469 | 0.008039856 | 0.115441239 | Down | 2.094751743 |
| KIF11 | -0.142050703 | 0.279958904 | 0.50348007 | NS | 0.552905715 |
| HHEX | 0.144866252 | 0.064655875 | 0.263807027 | NS | 1.189392008 |
| EXOC6 | 0.036655278 | 0.615990643 | 0.765168829 | NS | 0.210425885 |
| CYP26C1 | 0.447839129 | 0.015657489 | 0.148636863 | Up | 1.80527788 |
| CYP26A1 | 0.914820889 | 0.036538417 | NA | Up | 1.43725027 |
| MYOF | -0.057972377 | 0.634318201 | 0.776977881 | NS | 0.197692827 |
| CEP55 | 0.027806669 | 0.873217016 | 0.928388473 | NS | 0.05887781 |
| FFAR4 | 0.329090815 | 0.242349568 | 0.470094569 | NS | 0.615557749 |
| RBP4 | 0.956260057 | 0.068517637 | NA | NS | 1.164197625 |
| PDE6C | 0.270825385 | 0.068823771 | 0.268884683 | NS | 1.162261533 |
| FRA10AC1 | -0.089616298 | 0.228409019 | 0.457089362 | NS | 0.641286752 |
| LGI1 | 0.466826933 | 0.276790853 | 0.501110356 | NS | 0.557848266 |
| SLC35G1 | 0.176478369 | 0.066735841 | 0.266279415 | NS | 1.175640859 |
| PLCE1 | 0.093943653 | 0.253916103 | 0.480746129 | NS | 0.595309755 |
| NOC3L | -0.156530586 | 0.040922145 | 0.218105862 | Down | 1.388041614 |
| TBC1D12 | -0.276361194 | 0.012190147 | 0.134220553 | Down | 1.913991052 |
| HELLS | 0.087151095 | 0.357113646 | 0.570303392 | NS | 0.447193554 |
| CYP2C18 | 0.310000627 | 0.600897757 | NA | NS | 0.221199417 |
| CYP2C19 | 0.428281828 | 0.387616092 | NA | NS | 0.411598201 |
| CYP2C9 | 0.386441966 | 0.46017124 | NA | NS | 0.337080527 |
| CYP2C8 | 0.259064059 | 0.309289798 | 0.529643631 | NS | 0.509634405 |
| ACSM6 | 0.327039365 | 0.093534494 | 0.306402213 | NS | 1.0290282 |
| PDLIM1 | 0.139080576 | 0.171378262 | 0.399868249 | NS | 0.766044265 |
| SORBS1 | -0.046248779 | 0.832656204 | 0.904925373 | NS | 0.079534278 |
| ALDH18A1 | -0.1279386 | 0.085223772 | 0.293490206 | NS | 1.069439249 |
| TCTN3 | 0.120833697 | 0.051254369 | 0.239263668 | NS | 1.290269111 |
| ENTPD1 | 0.215335319 | 0.017206915 | 0.154054413 | Up | 1.764296987 |
| CC2D2B | 0.246042285 | 0.010749898 | 0.12826482 | Up | 1.968595676 |
| CCNJ | 0.109973221 | 0.018740275 | 0.159116357 | Up | 1.727224051 |
| ZNF518A | -0.060777998 | 0.282423946 | 0.505318219 | NS | 0.549098484 |
| BLNK | 0.014245014 | 0.881144435 | 0.932827018 | NS | 0.054952898 |
| DNTT | 0.514465717 | 0.203612585 | NA | NS | 0.691195382 |
| OPALIN | 0.788648929 | 0.062140702 | NA | NS | 1.206623848 |
| TLL2 | 0.7027987 | 0.04600218 | 0.228732505 | Up | 1.33722159 |
| TM9SF3 | -0.207668195 | 0.001627561 | 0.064266221 | Down | 2.788462656 |
| PIK3AP1 | -0.068980856 | 0.457781946 | 0.64929226 | NS | 0.339341339 |
| LCOR | 0.045561599 | 0.380978523 | 0.590855972 | NS | 0.419099506 |
| SLIT1 | 0.412285651 | 0.002190639 | 0.072832159 | Up | 2.659429185 |
| ARHGAP19-SLIT1 | -0.009235178 | 0.97455066 | NA | NS | 0.01119558 |
| ARHGAP19 | 0.054688672 | 0.497384401 | 0.679709754 | NS | 0.303307839 |
| FRAT1 | 0.22787685 | 0.018709003 | 0.158993777 | Up | 1.727949346 |
| FRAT2 | 0.078925986 | 0.470268338 | 0.65932571 | NS | 0.32765426 |
| RRP12 | 0.131316431 | 0.269943669 | 0.495451988 | NS | 0.568726854 |
| PGAM1 | -0.004367567 | 0.948969958 | 0.972451116 | NS | 0.022747536 |
| EXOSC1 | -0.139583342 | 0.039564111 | 0.215363569 | Down | 1.402698584 |
| ZDHHC16 | 0.055774482 | 0.220446359 | 0.449602156 | NS | 0.656697071 |
| MMS19 | -0.068486867 | 0.13840197 | 0.363014451 | NS | 0.858857728 |
| UBTD1 | 0.253890222 | 0.047359126 | 0.23126872 | Up | 1.324596317 |
| ANKRD2 | 0.730180225 | 0.033006043 | NA | Up | 1.481406542 |
| HOGA1 | -0.038897008 | 0.941967684 | NA | NS | 0.025963996 |
| MORN4 | 0.121367783 | 0.540234461 | 0.710529468 | NS | 0.267417716 |
| PI4K2A | -0.145457027 | 0.058252217 | 0.252197542 | NS | 1.234687539 |
| AVPI1 | 0.086141871 | 0.494043465 | 0.677438836 | NS | 0.306234841 |
| MARVELD1 | 0.437240362 | 0.014696099 | 0.145323863 | Up | 1.83279792 |
| ZFYVE27 | -0.044744215 | 0.263003573 | 0.489182801 | NS | 0.580038351 |
| SFRP5 | -0.041712977 | 0.821610509 | 0.898420735 | NS | 0.085334014 |
| GOLGA7B | 0.071656985 | 0.40037373 | 0.605341375 | NS | 0.397534426 |
| CRTAC1 | 0.346430121 | 0.293890501 | NA | NS | 0.531814451 |
| R3HCC1L | -0.009937189 | 0.809944124 | 0.890785913 | NS | 0.091544941 |
| LOXL4 | 0.224823418 | 0.009186713 | 0.121258266 | Up | 2.036839851 |
| PYROXD2 | 0.002348732 | 0.976534122 | 0.986397233 | NS | 0.010312577 |
| HPS1 | 0.02756898 | 0.821878007 | 0.89852789 | NS | 0.085192641 |
| HPSE2 | 0.253722285 | 0.521512017 | 0.697236907 | NS | 0.28273568 |
| CNNM1 | 0.638541617 | 0.032000853 | 0.198142899 | Up | 1.494838448 |
| GOT1 | -0.187734838 | 0.012457966 | 0.135362928 | Down | 1.904552856 |
| NKX2-3 | 0.899137444 | 0.059587812 | NA | NS | 1.22484256 |
| SLC25A28 | -0.070447098 | 0.113034743 | 0.331759797 | NS | 0.946788047 |
| ENTPD7 | 0.123537818 | 0.01994915 | 0.163807837 | Up | 1.70007561 |
| CUTC | -0.0393432 | 0.572494917 | 0.734073901 | NS | 0.242228365 |
| COX15 | -0.082630801 | 0.148642406 | 0.374763795 | NS | 0.827857272 |
| ABCC2 | 0.206578679 | 0.030179916 | 0.193469884 | Up | 1.520281973 |
| DNMBP | 0.270306403 | 0.010968536 | 0.129200737 | Up | 1.959851316 |
| CPN1 | 0.148861651 | 0.779460314 | NA | NS | 0.108205992 |
| ERLIN1 | 0.132613267 | 0.13144237 | 0.354132123 | NS | 0.88126462 |
| CHUK | 0.030479228 | 0.65609491 | 0.793467056 | NS | 0.183033331 |
| CWF19L1 | -0.020611061 | 0.616793784 | 0.765715595 | NS | 0.209860012 |
| BLOC1S2 | -0.24402754 | 0.048708131 | 0.233958392 | Down | 1.312398536 |
| PKD2L1 | 0.429934398 | 0.251216026 | NA | NS | 0.599952659 |
| SCD | 0.332058473 | 0.001543559 | 0.062836479 | Up | 2.811476886 |
| WNT8B | 0.232159097 | 0.2277589 | 0.456594391 | NS | 0.642524644 |
| SEC31B | 0.071534055 | 0.628868641 | 0.773982446 | NS | 0.201440061 |
| NDUFB8 | -0.152228071 | 0.032872012 | 0.200078102 | Down | 1.483173715 |
| HIF1AN | 0.06441303 | 0.125892766 | 0.347624322 | NS | 0.899999224 |
| PAX2 | 0.563896468 | 0.139694338 | 0.363934068 | NS | 0.854821197 |
| SLF2 | 0.013288751 | 0.684196665 | 0.812184317 | NS | 0.164819047 |
| MRPL43 | -0.187389533 | 0.033922744 | 0.202633983 | Down | 1.469509021 |
| SEMA4G | -0.039359487 | 0.698716276 | 0.82172696 | NS | 0.15569914 |
| TWNK | -0.075463075 | 0.292903572 | 0.515298984 | NS | 0.533275332 |
| LZTS2 | 0.156110673 | 0.068621013 | 0.268602708 | NS | 1.163542877 |
| PDZD7 | 0.582715023 | 0.010586708 | 0.127927347 | Up | 1.975239083 |
| SFXN3 | -0.03756628 | 0.515911189 | 0.693514002 | NS | 0.287425053 |
| KAZALD1 | 0.29110822 | 0.359223857 | NA | NS | 0.444634829 |
| TLX1 | 0.727649093 | 0.162388421 | NA | NS | 0.789444941 |
| LBX1 | 1.214259943 | 0.013059568 | NA | Up | 1.884071188 |
| BTRC | 0.106158008 | 0.254632858 | 0.48140404 | NS | 0.594085555 |
| DPCD | -0.329103386 | 0.011146529 | 0.129586317 | Down | 1.952860358 |
| POLL | 0.060071302 | 0.680296312 | 0.809570625 | NS | 0.167301884 |
| FBXW4 | -0.178431664 | 0.039796853 | 0.215730166 | Down | 1.400151267 |
| FGF8 | 0.943252507 | 0.013192957 | NA | Up | 1.879657854 |
| NPM3 | -0.32082483 | 0.004092279 | 0.090695453 | Down | 2.388034734 |
| OGA | 0.013989299 | 0.767767404 | 0.864740339 | NS | 0.11477033 |
| KCNIP2 | 0.233396664 | 0.12218609 | 0.342932892 | NS | 0.912978231 |
| ARMH3 | -0.017627221 | 0.763941057 | 0.86258335 | NS | 0.116940149 |
| HPS6 | 0.051806774 | 0.455392945 | 0.64746767 | NS | 0.341613701 |
| LDB1 | 0.013545327 | 0.775475834 | 0.86926304 | NS | 0.110431731 |
| PPRC1 | 0.030095018 | 0.805366127 | 0.887992636 | NS | 0.09400664 |
| NOLC1 | -0.124168776 | 0.036790363 | 0.209075161 | Down | 1.434265924 |
| ELOVL3 | 0.358133357 | 0.145624208 | 0.370771051 | NS | 0.836766424 |
| PITX3 | 1.662830594 | 0.002952982 | NA | Up | 2.529739178 |
| GBF1 | 0.025615171 | 0.765600478 | 0.863648023 | NS | 0.115997804 |
| NFKB2 | -0.080451128 | 0.313365104 | 0.533027897 | NS | 0.503949368 |
| PSD | 0.311933058 | 0.079164657 | 0.284218368 | NS | 1.101468666 |
| FBXL15 | 0.181976365 | 0.093156457 | 0.305593248 | NS | 1.030787038 |
| CUEDC2 | -0.128638093 | 0.05554101 | 0.248604264 | NS | 1.25538623 |
| MFSD13A | 0.15793023 | 0.135931511 | 0.360104507 | NS | 0.866679856 |
| ACTR1A | -0.03763081 | 0.411500118 | 0.614058952 | NS | 0.385630036 |
| SUFU | 0.152677668 | 0.27714632 | 0.50147784 | NS | 0.557290884 |
| TRIM8 | 0.032683397 | 0.756952506 | 0.858044076 | NS | 0.120931369 |
| ARL3 | 0.021093369 | 0.793563666 | 0.880746066 | NS | 0.100418225 |
| SFXN2 | 0.110286559 | 0.251322037 | 0.478173738 | NS | 0.59976943 |
| WBP1L | -0.026452429 | 0.618734957 | 0.766830888 | NS | 0.208495347 |
| CYP17A1 | 0.163403103 | 0.581034217 | NA | NS | 0.235798291 |
| BORCS7 | -0.240021329 | 0.060956651 | 0.256832544 | NS | 1.214978903 |
| CNNM2 | 0.234479299 | 5.98E-04 | 0.040636454 | Up | 3.223641048 |
| NT5C2 | 0.103530218 | 0.193537707 | 0.422794379 | NS | 0.713234408 |
| RPEL1 | 0.395152896 | 0.419548221 | NA | NS | 0.377218116 |
| INA | 0.677365803 | 0.179339961 | NA | NS | 0.74632293 |
| PCGF6 | -0.380852443 | 0.002281577 | 0.07380322 | Down | 2.641764842 |
| TAF5 | -0.116170232 | 0.017620687 | 0.155449116 | Down | 1.753977174 |
| ATP5MK | -0.242848507 | 0.111348378 | 0.329942771 | NS | 0.953316106 |
| PDCD11 | -0.187160362 | 0.006861278 | 0.109849098 | Down | 2.163595015 |
| CALHM2 | 0.095472309 | 0.170317232 | 0.39869447 | NS | 0.76874141 |
| CALHM1 | 0.456531034 | 0.026078389 | 0.182704458 | Up | 1.583719246 |
| CALHM3 | 0.888416554 | 0.064092877 | NA | NS | 1.193190236 |
| NEURL1 | -0.072259963 | 0.547821506 | 0.71610777 | NS | 0.261360922 |
| SH3PXD2A | 0.086948853 | 0.388588176 | 0.596317771 | NS | 0.410510418 |
| STN1 | -0.054219763 | 0.246705672 | 0.47480291 | NS | 0.607820866 |
| SLK | -0.079104623 | 0.201020316 | 0.430601586 | NS | 0.696760049 |
| COL17A1 | 0.363815775 | 0.005110469 | 0.09835814 | Up | 2.291539251 |
| SFR1 | -0.289371384 | 0.024733613 | 0.178240783 | Down | 1.606712435 |
| CFAP43 | 0.306288482 | 0.194632354 | 0.424088309 | NS | 0.710784966 |
| GSTO1 | -0.146518933 | 0.155661797 | 0.383123748 | NS | 0.807817961 |
| GSTO2 | 0.267835963 | 0.011268147 | 0.130166967 | Up | 1.948147485 |
| ITPRIP | 0.170828905 | 0.085831239 | 0.294524696 | NS | 1.066354618 |
| CFAP58 | 0.086698405 | 0.542422685 | 0.712425552 | NS | 0.265662155 |
| SORCS3 | 0.226414438 | 0.259047785 | 0.485568555 | NS | 0.586620117 |
| SORCS1 | 0.379094365 | 0.394515275 | 0.600265002 | NS | 0.403936177 |
| XPNPEP1 | -0.084139092 | 0.063392561 | 0.261447225 | NS | 1.197961701 |
| ADD3 | -0.070676309 | 0.246498682 | 0.474646517 | NS | 0.608185398 |
| MXI1 | -0.105648641 | 0.591692559 | 0.748546815 | NS | 0.227903892 |
| SMNDC1 | 0.114813014 | 0.070260402 | 0.271077991 | NS | 1.153289369 |
| DUSP5 | -0.356059643 | 0.001481678 | 0.062253356 | Down | 2.829246214 |
| SMC3 | -0.143620754 | 0.00391851 | 0.089796534 | Down | 2.406878998 |
| RBM20 | 0.481438141 | 0.149841936 | 0.376033315 | NS | 0.824366623 |
| PDCD4 | -0.117420745 | 0.012862278 | 0.136134668 | Down | 1.890682117 |
| BBIP1 | 0.129761168 | 0.013253134 | 0.138004676 | Up | 1.877681424 |
| SHOC2 | -0.087110442 | 0.206659102 | 0.436615014 | NS | 0.684745462 |
| ADRA2A | 0.613496283 | 0.016236704 | 0.150699117 | Up | 1.789502136 |
| GPAM | -0.05467586 | 0.366939319 | 0.579259389 | NS | 0.435405749 |
| TECTB | 0.470366952 | 0.243469505 | NA | NS | 0.613555427 |
| ACSL5 | -0.185451141 | 0.005988163 | 0.103408357 | Down | 2.222706395 |
| ZDHHC6 | -0.027170592 | 0.395303673 | 0.600786059 | NS | 0.40306915 |
| VTI1A | -0.04884883 | 0.293279278 | 0.515386052 | NS | 0.532718621 |
| TCF7L2 | 0.275106277 | 0.015402407 | 0.147618407 | Up | 1.812411395 |
| HABP2 | 0.436576901 | 0.316009729 | NA | NS | 0.500299547 |
| NRAP | 0.560131758 | 0.225830496 | 0.454435762 | NS | 0.646217412 |
| CASP7 | -0.093303448 | 0.250568253 | 0.477493271 | NS | 0.601073955 |
| PLEKHS1 | 0.750108217 | 0.074460498 | 0.277058381 | NS | 1.128074061 |
| DCLRE1A | -0.170833854 | 0.026057832 | 0.182704458 | Down | 1.584061722 |
| NHLRC2 | 0.040666532 | 0.438127379 | 0.633591488 | NS | 0.358399606 |
| ADRB1 | 0.130969491 | 0.541154704 | 0.711320112 | NS | 0.266678562 |
| CCDC186 | -0.106653944 | 0.08717367 | 0.296703312 | NS | 1.059614669 |
| TDRD1 | 0.310380014 | 0.295251194 | 0.517197709 | NS | 0.529808337 |
| VWA2 | 0.791066207 | 0.010143347 | 0.126203819 | Up | 1.993818696 |
| AFAP1L2 | 0.168727016 | 0.120871545 | 0.341402767 | NS | 0.917675928 |
| ABLIM1 | 0.026604627 | 0.729261732 | 0.840152469 | NS | 0.137116575 |
| FHIP2A | 0.170474975 | 0.038029752 | 0.212221582 | Up | 1.419876511 |
| TRUB1 | -0.19584312 | 0.016333903 | 0.151094105 | Down | 1.786910039 |
| ATRNL1 | 0.327203583 | 0.256495008 | 0.483478946 | NS | 0.590921083 |
| GFRA1 | 0.483972954 | 0.264849363 | 0.491060566 | NS | 0.577001067 |
| CCDC172 | 1.212746549 | 0.028954482 | NA | Up | 1.538284204 |
| PNLIPRP3 | 0.268859513 | 0.596501643 | NA | NS | 0.224388356 |
| PNLIP | 0.670471336 | 0.246351107 | NA | NS | 0.608445482 |
| PNLIPRP1 | 0.396235852 | 0.406370538 | NA | NS | 0.391077786 |
| C10orf82 | 0.413823217 | 0.296688606 | NA | NS | 0.527699133 |
| HSPA12A | 0.316865741 | 0.1780825 | 0.408089396 | NS | 0.749378755 |
| ENO4 | 0.560252656 | 0.005733231 | 0.102180893 | Up | 2.24160059 |
| SHTN1 | 0.228394627 | 0.042276045 | 0.220751111 | Up | 1.373905647 |
| VAX1 | 0.916564269 | 0.035211859 | 0.205507211 | Up | 1.453311045 |
| KCNK18 | 0.535602961 | 0.358595759 | NA | NS | 0.445394852 |
| SLC18A2 | 0.032581728 | 0.765296868 | 0.863390585 | NS | 0.116170064 |
| PDZD8 | -0.002088772 | 0.976840528 | 0.986489187 | NS | 0.01017633 |
| EMX2 | 0.793050411 | 0.072149147 | NA | NS | 1.1417688 |
| RAB11FIP2 | 0.05899185 | 0.223925533 | 0.452443504 | NS | 0.649896384 |
| FAM204A | -0.126261574 | 0.010843202 | 0.12856202 | Down | 1.964842448 |
| PRLHR | 0.76551818 | 0.095350682 | NA | NS | 1.020676197 |
| CACUL1 | 0.086117955 | 0.198769289 | 0.428766531 | NS | 0.701650716 |
| NANOS1 | 0.154910218 | 0.048651711 | 0.233910643 | Up | 1.312901881 |
| EIF3A | -0.161371386 | 0.00120455 | 0.056242293 | Down | 2.919175313 |
| DENND10 | 0.028997787 | 0.696002776 | 0.819782739 | NS | 0.157389028 |
| SFXN4 | -0.217678708 | 0.008146581 | 0.116225959 | Down | 2.089024606 |
| PRDX3 | -0.062795669 | 0.533268752 | 0.705998557 | NS | 0.273053864 |
| GRK5 | -0.234877366 | 2.46E-04 | 0.026225737 | Down | 3.609855425 |
| RGS10 | -0.478365906 | 0.00172599 | 0.065336585 | Down | 2.762961605 |
| TIAL1 | 0.085873337 | 0.355903746 | 0.568903557 | NS | 0.448667441 |
| BAG3 | 0.265243598 | 0.02442432 | 0.177126593 | Up | 1.612177516 |
| INPP5F | 0.016897878 | 0.696976834 | 0.820297213 | NS | 0.156781656 |
| MCMBP | -0.083818332 | 0.276192483 | 0.500789448 | NS | 0.558788146 |
| SEC23IP | 0.031927199 | 0.401266554 | 0.605820044 | NS | 0.396567037 |
| PLPP4 | 0.442822516 | 0.321491893 | NA | NS | 0.492829974 |
| WDR11 | -0.115959343 | 0.042407673 | 0.220932874 | Down | 1.372555553 |
| FGFR2 | 0.249553646 | 0.19916904 | 0.428926322 | NS | 0.700778171 |
| ATE1 | 0.018419673 | 0.643854875 | 0.784594681 | NS | 0.191212011 |
| NSMCE4A | -0.117260685 | 0.097230719 | 0.3115682 | NS | 1.012196503 |
| TACC2 | 0.593076284 | 0.138724856 | 0.363177486 | NS | 0.857845718 |
| BTBD16 | 0.591899743 | 0.192194741 | NA | NS | 0.716258499 |
| PLEKHA1 | -0.108099293 | 0.088600827 | 0.298797842 | NS | 1.052562223 |
| ARMS2 | 0.527577819 | 0.406277116 | NA | NS | 0.391177639 |
| HTRA1 | 0.081249168 | 0.735397645 | 0.843833702 | NS | 0.133477765 |
| DMBT1 | 0.194512524 | 0.6310061 | 0.775295013 | NS | 0.199966443 |
| C10orf120 | 0.654246042 | 0.191413782 | NA | NS | 0.718026796 |
| CUZD1 | 0.182795674 | 0.280937889 | 0.504116551 | NS | 0.551389685 |
| FAM24B | -0.043273237 | 0.615594291 | 0.765059537 | NS | 0.210705416 |
| FAM24A | 0.078792458 | 0.879348206 | NA | NS | 0.055839118 |
| C10orf88 | -0.013393308 | 0.818022094 | 0.896465786 | NS | 0.087234966 |
| PSTK | 0.080392688 | 0.180068425 | 0.410006074 | NS | 0.744562433 |
| IKZF5 | 0.049161742 | 0.459869965 | 0.651234629 | NS | 0.337364954 |
| ACADSB | -0.088314961 | 0.087509967 | 0.297087647 | NS | 1.057942479 |
| HMX3 | 1.203768007 | 0.010208112 | NA | Up | 1.991054577 |
| HMX2 | 1.075284472 | 0.034356904 | NA | Up | 1.463985983 |
| BUB3 | -0.047777124 | 0.358559207 | 0.571528602 | NS | 0.445439122 |
| GPR26 | 0.461273297 | 0.263745951 | 0.490023249 | NS | 0.5788142 |
| CPXM2 | 0.729889474 | 0.055865813 | 0.249087638 | NS | 1.252853879 |
| CHST15 | 0.081397635 | 0.429908437 | 0.627284739 | NS | 0.366624032 |
| OAT | -0.130560543 | 0.166310592 | 0.394295091 | NS | 0.779080091 |
| NKX1-2 | 0.500447064 | 0.474005086 | NA | NS | 0.324216998 |
| LHPP | 0.133466026 | 0.371831559 | 0.582827074 | NS | 0.429653752 |
| FAM53B | -0.037077492 | 0.559727807 | 0.724558344 | NS | 0.252023117 |
| EEF1AKMT2 | 0.002067822 | 0.966779367 | 0.981478836 | NS | 0.014672627 |
| ABRAXAS2 | -0.01649084 | 0.784472114 | 0.875577775 | NS | 0.10542249 |
| ZRANB1 | -0.106720243 | 0.384690607 | 0.59325935 | NS | 0.414888417 |
| CTBP2 | 0.025750635 | 0.680906645 | 0.809943926 | NS | 0.166912427 |
| TEX36 | 0.470326253 | 0.432394192 | NA | NS | 0.364120148 |
| EDRF1 | -0.004934888 | 0.889230759 | 0.937363658 | NS | 0.050985523 |
| MMP21 | 0.334072007 | 0.005660934 | 0.101933358 | Up | 2.24711189 |
| UROS | -0.091689534 | 0.247258793 | 0.475499276 | NS | 0.606848255 |
| BCCIP | -0.114737014 | 0.108206122 | 0.325834237 | NS | 0.965748167 |
| DHX32 | -0.115661293 | 0.075811523 | 0.279137541 | NS | 1.120264777 |
| FANK1 | 0.258824146 | 0.134104852 | 0.357706654 | NS | 0.872555507 |
| ADAM12 | 0.159666142 | 0.31979094 | 0.538985786 | NS | 0.495133844 |
| C10orf90 | 0.596425724 | 0.12731827 | 0.348830209 | NS | 0.895109272 |
| DOCK1 | 0.230143506 | 0.29625957 | 0.517980183 | NS | 0.528327612 |
| INSYN2A | 0.334710721 | 0.280356323 | NA | NS | 0.552289645 |
| NPS | 0.526433893 | 0.358659247 | NA | NS | 0.445317968 |
| FOXI2 | 0.668955884 | 0.160947724 | NA | NS | 0.79331516 |
| CLRN3 | 0.691103342 | 0.184350316 | NA | NS | 0.734356115 |
| PTPRE | -0.074859863 | 0.32047082 | 0.539187682 | NS | 0.494211508 |
| MKI67 | 0.003281689 | 0.98415908 | 0.990467313 | NS | 0.006934696 |
| MGMT | -0.138402908 | 0.032737104 | 0.199748547 | Down | 1.484959745 |
| EBF3 | 0.677503196 | 0.045361467 | 0.227426386 | Up | 1.34331291 |
| C10orf143 | -0.021596017 | 0.830460274 | 0.903626518 | NS | 0.080681137 |
| GLRX3 | -0.123394045 | 0.07256575 | 0.274170249 | NS | 1.139268313 |
| TCERG1L | 0.506924588 | 0.117728378 | NA | NS | 0.92911884 |
| PPP2R2D | -0.144010918 | 5.50E-04 | 0.040103819 | Down | 3.259719728 |
| BNIP3 | -0.101678658 | 0.039878974 | 0.215796828 | Down | 1.399256022 |
| JAKMIP3 | 0.637890933 | 0.004811791 | 0.096244227 | Up | 2.317693235 |
| DPYSL4 | 0.463237526 | 0.023199405 | 0.173122445 | Up | 1.634523145 |
| STK32C | -0.142831168 | 0.056083999 | 0.249087638 | NS | 1.25116103 |
| LRRC27 | -0.026134683 | 0.750018698 | 0.853172812 | NS | 0.12492791 |
| PWWP2B | 0.029344986 | 0.740693112 | 0.847890045 | NS | 0.130361694 |
| INPP5A | 0.020843824 | 0.747537478 | 0.851746434 | NS | 0.126367029 |
| NKX6-2 | 0.385464647 | 0.026182151 | 0.182943431 | Up | 1.581994678 |
| CFAP46 | 0.773004137 | 0.024959032 | 0.178950571 | Up | 1.602772254 |
| ADGRA1 | 0.759824848 | 0.112923902 | NA | NS | 0.947214125 |
| KNDC1 | 0.378522798 | 0.083451741 | 0.290969351 | NS | 1.078564601 |
| UTF1 | 1.619794892 | 0.019888584 | NA | Up | 1.701396141 |
| VENTX | 0.309348352 | 0.074728181 | 0.277513347 | NS | 1.126515586 |
| ADAM8 | 0.152290572 | 0.168432173 | 0.396682192 | NS | 0.773574948 |
| TUBGCP2 | -0.154698314 | 0.025855764 | 0.182170348 | Down | 1.587442617 |
| ZNF511 | 0.043360616 | 0.61756396 | 0.766154761 | NS | 0.209318056 |
| CALY | 0.872915031 | 0.008931598 | NA | Up | 2.049070842 |
| PRAP1 | 0.216081945 | 0.324102498 | NA | NS | 0.489317622 |
| FUOM | -0.158855527 | 0.087179491 | 0.296703312 | NS | 1.059585672 |
| ECHS1 | -0.253976128 | 0.011128729 | 0.129586317 | Down | 1.953554425 |
| PAOX | 0.053909084 | 0.687197584 | 0.814178911 | NS | 0.162918376 |
| MTG1 | 0.246177254 | 0.012525328 | 0.135545097 | Up | 1.902210876 |
| SPRN | 0.286483049 | 0.039389108 | 0.214800721 | Up | 1.404623859 |
| SCART1 | 0.028066364 | 0.852030929 | 0.916543558 | NS | 0.06954464 |
| CYP2E1 | 0.317251477 | 0.186831783 | 0.416322648 | NS | 0.728549242 |
| SYCE1 | 0.783470835 | 0.069321135 | NA | NS | 1.159134334 |
| FRG2B | 0.401136763 | 0.548731858 | NA | NS | 0.260639825 |
| BET1L | 0.1719859 | 0.038967861 | 0.214288889 | Up | 1.40929343 |
| SCGB1C1 | -0.043839905 | 0.885121087 | NA | NS | 0.052997312 |
| ODF3 | -0.013681952 | 0.942147948 | 0.968378356 | NS | 0.025880893 |
| RIC8A | -0.146233324 | 0.015816993 | 0.149193072 | Down | 1.800876071 |
| SIRT3 | 0.036330645 | 0.54546247 | 0.714200227 | NS | 0.263235125 |
| PSMD13 | -0.191348336 | 0.005864133 | 0.102935441 | Down | 2.231796188 |
| NLRP6 | 0.142529892 | 0.416433922 | 0.61755717 | NS | 0.380453901 |
| PGGHG | 0.016821482 | 0.908756822 | 0.949511781 | NS | 0.041552316 |
| IFITM5 | 0.136029061 | 0.491538344 | 0.675742923 | NS | 0.308442598 |
| IFITM2 | 0.343180606 | 0.02604279 | 0.182704458 | Up | 1.584312483 |
| IFITM1 | 0.069798216 | 0.520675991 | 0.696688569 | NS | 0.283432448 |
| IFITM3 | 0.434955458 | 0.004533263 | 0.094211252 | Up | 2.343589042 |
| B4GALNT4 | 0.607538716 | 0.025621908 | 0.181332798 | Up | 1.591388535 |
| PKP3 | 0.853680073 | 0.028186962 | 0.187874794 | Up | 1.549951738 |
| SIGIRR | -0.052661479 | 0.580088641 | 0.739545204 | NS | 0.236505639 |
| ANO9 | -0.126088348 | 0.239215659 | 0.467477036 | NS | 0.621210394 |
| PTDSS2 | -0.035656269 | 0.701467438 | 0.823069836 | NS | 0.153992484 |
| RNH1 | -0.232105856 | 0.054351994 | 0.246476974 | NS | 1.264784517 |
| HRAS | 0.048735193 | 0.684355214 | 0.812288319 | NS | 0.164718419 |
| LRRC56 | 0.147495529 | 0.448032162 | 0.641635461 | NS | 0.348690809 |
| LMNTD2 | 0.06931239 | 0.813027225 | NA | NS | 0.089894912 |
| RASSF7 | -0.091778672 | 0.330749191 | 0.547828015 | NS | 0.48050121 |
| PHRF1 | -0.173161219 | 0.160381737 | 0.38802689 | NS | 0.794845087 |
| IRF7 | 0.377205179 | 7.95E-04 | 0.04690569 | Up | 3.099816159 |
| CDHR5 | 0.623034054 | 0.209276752 | NA | NS | 0.679279015 |
| DEAF1 | 0.076219095 | 0.275657951 | 0.5002471 | NS | 0.559629476 |
| EPS8L2 | 0.170031778 | 0.153347803 | 0.380042352 | NS | 0.814322443 |
| TMEM80 | -0.010838387 | 0.838877294 | 0.909109618 | NS | 0.076301561 |
| TALDO1 | -0.012769254 | 0.89493383 | 0.941136237 | NS | 0.048209074 |
| GATD1 | 0.008243449 | 0.901947054 | 0.9454453 | NS | 0.044818955 |
| CEND1 | 0.120353235 | 0.392925616 | 0.599318562 | NS | 0.405689657 |
| SLC25A22 | 0.303444831 | 0.004095893 | 0.090695453 | Up | 2.387651351 |
| PIDD1 | 0.194547344 | 0.184871378 | 0.415049925 | NS | 0.733130323 |
| RPLP2 | -0.05009768 | 0.676090342 | 0.806785925 | NS | 0.169995268 |
| PNPLA2 | 0.090211839 | 0.624837319 | 0.771138884 | NS | 0.20423304 |
| CRACR2B | 0.541219904 | 0.004206338 | 0.092009125 | Up | 2.376095826 |
| CD151 | 0.146944245 | 0.175597013 | 0.40496105 | NS | 0.755482877 |
| POLR2L | 0.003137738 | 0.973514071 | 0.984997537 | NS | 0.011657767 |
| TSPAN4 | -0.095815279 | 0.295530355 | 0.517431069 | NS | 0.529397904 |
| CHID1 | -0.176606013 | 0.120712414 | 0.341313216 | NS | 0.918248065 |
| AP2A2 | -0.037236759 | 0.674882311 | 0.806183741 | NS | 0.170771955 |
| MUC2 | 0.576876222 | 0.371472009 | NA | NS | 0.430073905 |
| MUC5B | 0.49703347 | 0.078046537 | 0.282523416 | NS | 1.107646359 |
| TOLLIP | -0.060899246 | 0.557910792 | 0.723698109 | NS | 0.253435237 |
| BRSK2 | 0.347175874 | 0.070307679 | 0.271077991 | NS | 1.152997236 |
| MOB2 | -0.175429396 | 0.007902577 | 0.11472857 | Down | 2.102231243 |
| IFITM10 | 0.261335303 | 0.315561839 | NA | NS | 0.500915522 |
| CTSD | 0.084704433 | 0.395515707 | 0.600988637 | NS | 0.402836264 |
| TNNI2 | 0.104905992 | 0.461217914 | 0.65234762 | NS | 0.336093833 |
| LSP1 | -0.242284556 | 0.051881364 | 0.240601654 | NS | 1.28498861 |
| TNNT3 | -0.235637872 | 0.290787497 | 0.513652722 | NS | 0.536424271 |
| MRPL23 | -0.178697836 | 0.120725857 | 0.341313216 | NS | 0.918199701 |
| IGF2 | 0.654257721 | 0.247829108 | NA | NS | 0.605847687 |
| INS | 1.154358002 | 0.097734325 | NA | NS | 1.00995288 |
| TH | 0.908752998 | 0.044593116 | NA | Up | 1.350732183 |
| ASCL2 | -0.113232095 | 0.396680432 | 0.601879903 | NS | 0.401559223 |
| C11orf21 | -0.046946958 | 0.425503816 | 0.623837924 | NS | 0.37109654 |
| TSPAN32 | -0.035200787 | 0.692267941 | 0.817275137 | NS | 0.15972578 |
| CD81 | -0.131831781 | 0.084420606 | 0.292231065 | NS | 1.073551533 |
| TSSC4 | -0.205512981 | 0.005009973 | 0.097847413 | Down | 2.30016458 |
| TRPM5 | 0.708632746 | 0.011104258 | 0.129551558 | Up | 1.95451047 |
| KCNQ1 | -0.03946543 | 0.658895199 | 0.794880839 | NS | 0.181183657 |
| CDKN1C | -0.135885377 | 0.55126997 | 0.718687588 | NS | 0.258635664 |
| SLC22A18AS | -0.018942815 | 0.961387401 | NA | NS | 0.017101573 |
| SLC22A18 | 0.186829052 | 0.15155292 | 0.378007512 | NS | 0.819435691 |
| NAP1L4 | -0.201723436 | 0.03779381 | 0.211420088 | Down | 1.422579322 |
| CARS1 | -0.133842288 | 0.036288584 | 0.208236145 | Down | 1.440229979 |
| OSBPL5 | -0.156950675 | 0.161254712 | 0.389034501 | NS | 0.792487587 |
| MRGPRG | 1.832945639 | 0.004517143 | NA | Up | 2.345136157 |
| MRGPRE | 0.432782788 | 0.205372136 | 0.435341785 | NS | 0.687458481 |
| ZNF195 | 0.021601439 | 0.689028619 | 0.815257617 | NS | 0.161762739 |
| ART5 | 0.986378602 | 0.057020288 | NA | NS | 1.243970593 |
| ART1 | 0.545019246 | 0.057686603 | NA | NS | 1.238925036 |
| CHRNA10 | 0.145738047 | 0.186846003 | 0.416322648 | NS | 0.728516188 |
| NUP98 | -0.174474259 | 0.017860069 | 0.156153671 | Down | 1.748116861 |
| PGAP2 | 0.113392017 | 0.117384201 | 0.337318365 | NS | 0.930390351 |
| RHOG | 0.480161679 | 7.89E-05 | 0.013591828 | Up | 4.102989234 |
| STIM1 | 0.078127094 | 0.204994007 | 0.43502342 | NS | 0.688258835 |
| RRM1 | -0.190437098 | 0.002194958 | 0.072832159 | Down | 2.658573688 |
| OR52B4 | 0.227511964 | 0.474736891 | NA | NS | 0.323547018 |
| TRIM21 | -0.005950284 | 0.942968543 | 0.968789986 | NS | 0.025502795 |
| OR52K2 | 0.085772941 | 0.643270771 | 0.784127485 | NS | 0.191606182 |
| OR52K1 | 0.283449286 | 0.072103907 | 0.273763446 | NS | 1.142041201 |
| OR52M1 | -0.209854105 | 0.702459437 | NA | NS | 0.153378749 |
| OR52I2 | 0.465382894 | 0.036928203 | 0.209371187 | Up | 1.432641828 |
| OR52I1 | 0.303731773 | 0.138485626 | NA | NS | 0.858595302 |
| TRIM68 | -0.097821386 | 0.100746208 | 0.316153692 | NS | 0.996771293 |
| OR51D1 | 0.536353145 | 0.279422119 | NA | NS | 0.553739218 |
| OR51E1 | 0.288422515 | 0.571903939 | NA | NS | 0.242676912 |
| OR51E2 | 0.494342072 | 0.327205739 | NA | NS | 0.485179087 |
| OR51C1P | -0.100765172 | 0.853161345 | NA | NS | 0.06896883 |
| MMP26 | 0.807142434 | 0.103717158 | NA | NS | 0.984149393 |
| OR52R1 | 1.053040646 | 0.078384877 | NA | NS | 1.105767721 |
| OR51F2 | 0.450096718 | 0.460689466 | NA | NS | 0.336591718 |
| OR51S1 | -0.300668209 | 0.618633531 | NA | NS | 0.208566544 |
| OR51T1 | 0.370836697 | 0.501994964 | NA | NS | 0.29930064 |
| OR51A7 | 0.516194677 | 0.356489376 | NA | NS | 0.447953409 |
| OR51G2 | 0.827235912 | 0.078960096 | NA | NS | 1.102592335 |
| OR51G1 | 0.06358741 | 0.911959693 | NA | NS | 0.040024356 |
| OR51A4 | 0.330000128 | 0.516747372 | NA | NS | 0.286721724 |
| OR51A2 | 0.575598365 | 0.546040494 | NA | NS | 0.262775149 |
| OR51L1 | 0.517710188 | 0.285975225 | 0.509166182 | NS | 0.54367159 |
| OR52J3 | 0.831223595 | 0.160279289 | NA | NS | 0.795122593 |
| OR52E2 | 0.222619595 | 0.729794664 | NA | NS | 0.136799316 |
| OR52A5 | 0.832609073 | 0.090148991 | NA | NS | 1.045039128 |
| OR52A1 | 0.600965621 | 0.183213881 | 0.413453601 | NS | 0.737041625 |
| OR51V1 | 1.313233748 | 0.018413424 | NA | Up | 1.734865442 |
| HBB | 5.041602052 | 1.43E-19 | 1.09E-15 | Up | 18.84606153 |
| HBD | -0.325098687 | 0.47824216 | 0.665917695 | NS | 0.32035214 |
| HBG1 | 0.138832431 | 0.847855217 | 0.914327833 | NS | 0.071678303 |
| HBG2 | 3.752292263 | 1.11E-13 | 5.09E-10 | Up | 12.95440996 |
| OR51B4 | 0.509884324 | 0.358227798 | NA | NS | 0.445840716 |
| OR51B5 | -0.103644439 | 0.767010684 | NA | NS | 0.115198587 |
| OR51B2 | 0.526359745 | 0.238112094 | NA | NS | 0.623218545 |
| OR51B6 | 0.997038402 | 0.063119408 | NA | NS | 1.199837082 |
| OR51M1 | 0.217035619 | 0.539125709 | NA | NS | 0.268309958 |
| OR51Q1 | 0.31342229 | 0.428368374 | NA | NS | 0.3681826 |
| OR51I1 | 0.287276798 | 0.498228364 | NA | NS | 0.302571552 |
| OR51I2 | 0.828034676 | 0.031074755 | NA | Up | 1.507592292 |
| OR52D1 | 0.217558139 | 0.582841232 | NA | NS | 0.234449733 |
| UBQLN3 | 0.166745547 | 0.60911515 | NA | NS | 0.215300599 |
| UBQLNL | 0.183805722 | 0.297035136 | 0.518893873 | NS | 0.527192176 |
| OR52H1 | 0.240255807 | 0.297613477 | NA | NS | 0.526347407 |
| OR52B6 | 0.339631417 | 0.147314952 | NA | NS | 0.831753173 |
| TRIM6 | 0.389245693 | 0.026263646 | 0.183092929 | Up | 1.580644977 |
| TRIM34 | 0.200724964 | 0.10382629 | 0.319643728 | NS | 0.983692665 |
| TRIM5 | 0.013534145 | 0.824242693 | 0.900052742 | NS | 0.083944894 |
| TRIM22 | 0.124598751 | 0.171616114 | 0.400205856 | NS | 0.765441936 |
| OR56B1 | 0.246412653 | 0.31323233 | 0.532960388 | NS | 0.504133419 |
| OR52N4 | 0.406362617 | 0.009769758 | 0.124347359 | Up | 2.010116179 |
| OR56B2P | 0.347611275 | 0.245624623 | NA | NS | 0.609728099 |
| OR52N5 | 0.4323355 | 0.330508762 | NA | NS | 0.480817023 |
| OR52N1 | 0.756862911 | 0.062988094 | NA | NS | 1.20074153 |
| OR52N2 | 1.069950265 | 0.041869702 | NA | Up | 1.378100134 |
| OR52E6 | 1.06765804 | 0.10902124 | NA | NS | 0.962488882 |
| OR52E8 | 1.348912738 | 0.049513847 | NA | Up | 1.305273329 |
| OR52E4 | 0.818582048 | 0.14817912 | NA | NS | 0.82921299 |
| OR52E5 | 0.47113737 | 0.414493423 | NA | NS | 0.382482356 |
| OR56A3 | 0.392548207 | 0.42432329 | NA | NS | 0.372303131 |
| OR56A5 | 0.939201261 | 0.099790588 | NA | NS | 1.000910417 |
| OR52L1 | 0.756130014 | 0.151532922 | NA | NS | 0.819493003 |
| OR56A4 | 0.488694272 | 0.323383395 | NA | NS | 0.490282285 |
| OR56A1 | 0.445267691 | 0.146307724 | 0.371808642 | NS | 0.834732747 |
| OR56B4 | 0.102578751 | 0.795165642 | NA | NS | 0.099542394 |
| OR52B2 | 0.672103204 | 0.05104877 | NA | NS | 1.292014719 |
| OR52W1 | 0.188059874 | 0.250845637 | 0.47774934 | NS | 0.600593448 |
| C11orf42 | 0.5674626 | 2.03E-05 | 0.00527564 | Up | 4.693115598 |
| FHIP1B | 0.096472905 | 0.204369958 | 0.43446401 | NS | 0.689582944 |
| CNGA4 | 0.483379838 | 0.048110717 | NA | Up | 1.317758166 |
| CCKBR | 0.635076008 | 0.182296768 | NA | NS | 0.739221031 |
| CAVIN3 | 0.228138441 | 0.309808594 | NA | NS | 0.508906539 |
| SMPD1 | 0.099481361 | 0.15678342 | 0.384027153 | NS | 0.804699867 |
| APBB1 | -0.203222408 | 0.024268167 | 0.176550027 | Down | 1.614963031 |
| HPX | 0.304682317 | 0.305255305 | 0.526578834 | NS | 0.515336779 |
| TRIM3 | 0.216249275 | 0.102097976 | 0.317962583 | NS | 0.990982866 |
| ARFIP2 | -0.207217978 | 0.001195767 | 0.056242293 | Down | 2.922353421 |
| TIMM10B | -0.085364214 | 0.11205719 | 0.330785464 | NS | 0.950560271 |
| DNHD1 | 0.123341228 | 0.288037237 | 0.511208059 | NS | 0.540551364 |
| RRP8 | 0.038197591 | 0.30413605 | 0.525407242 | NS | 0.516932099 |
| ILK | 0.272804548 | 0.022295637 | 0.170667658 | Up | 1.651780114 |
| TAF10 | 0.018345782 | 0.745497045 | 0.850639911 | NS | 0.127554074 |
| TPP1 | 0.203939498 | 0.057178472 | 0.250814283 | NS | 1.242767453 |
| DCHS1 | 0.154064359 | 0.324588372 | 0.543083204 | NS | 0.488667042 |
| MRPL17 | -0.11819475 | 0.167106394 | 0.395154428 | NS | 0.777006932 |
| OR2AG2 | 0.409400268 | 0.363881444 | NA | NS | 0.43904009 |
| OR2AG1 | 0.343891524 | 0.368879941 | 0.580544833 | NS | 0.43311496 |
| OR6A2 | 0.356595949 | 0.388818497 | NA | NS | 0.410253082 |
| OR10A5 | 0.223876224 | 0.650309147 | NA | NS | 0.186880137 |
| OR10A2 | 0.467694649 | 0.243778862 | 0.471635365 | NS | 0.613003954 |
| OR10A4 | 0.418850875 | 0.36631675 | NA | NS | 0.436143223 |
| OR2D2 | 0.478083267 | 0.432121844 | NA | NS | 0.36439378 |
| OR2D3 | 0.213335577 | 0.702791496 | NA | NS | 0.153173502 |
| ZNF215 | -0.196467222 | 0.37826418 | 0.588239713 | NS | 0.422204783 |
| ZNF214 | 0.226577531 | 0.545612014 | 0.714320063 | NS | 0.263116075 |
| NLRP14 | 0.500233235 | 0.228286271 | NA | NS | 0.641520206 |
| RBMXL2 | 1.439652832 | 0.004979233 | NA | Up | 2.302837515 |
| SYT9 | 0.605386486 | 0.014385304 | 0.143935548 | Up | 1.842080942 |
| OLFML1 | 0.279945754 | 0.040030035 | 0.215862456 | Up | 1.397614035 |
| PPFIBP2 | 0.073932141 | 0.305751409 | 0.526819451 | NS | 0.514631533 |
| CYB5R2 | -9.16E-04 | 0.994324494 | 0.99671815 | NS | 0.002471862 |
| OVCH2 | 0.435470338 | 0.227776035 | NA | NS | 0.642491971 |
| EIF3F | -0.163878098 | 0.031397868 | 0.196737067 | Down | 1.503099837 |
| TUB | 0.141233384 | 0.281308473 | 0.504425826 | NS | 0.550817187 |
| RIC3 | 0.05570032 | 0.550524777 | 0.718083975 | NS | 0.25922313 |
| LMO1 | 1.293088272 | 0.009379406 | NA | Up | 2.027824678 |
| STK33 | 0.144781338 | 0.415709632 | 0.616977642 | NS | 0.381209913 |
| TRIM66 | 0.251997173 | 0.035362501 | 0.205857644 | Up | 1.451457027 |
| RPL27A | -0.352683683 | 0.010223743 | 0.126233693 | Down | 1.990390064 |
| DENND2B | 0.259058533 | 0.248888566 | 0.476376043 | NS | 0.603995054 |
| AKIP1 | -0.124187876 | 0.185345441 | 0.41546259 | NS | 0.732018093 |
| C11orf16 | 0.279544177 | 0.25113046 | NA | NS | 0.600100608 |
| ASCL3 | 0.369806845 | 0.468400486 | NA | NS | 0.329382663 |
| TMEM9B | -0.236745212 | 0.003898864 | 0.089796534 | Down | 2.409061915 |
| NRIP3 | 0.169052168 | 0.227435201 | 0.456105163 | NS | 0.643142318 |
| SCUBE2 | 0.453003072 | 0.036519628 | 0.208452907 | Up | 1.437473656 |
| DENND5A | 0.096714808 | 0.210141863 | 0.440111431 | NS | 0.677487421 |
| TMEM41B | -0.072073635 | 0.173859096 | 0.402960258 | NS | 0.759802582 |
| IPO7 | -0.186068714 | 0.006817813 | 0.109684865 | Down | 2.166354918 |
| ZNF143 | -0.118309481 | 0.010621771 | 0.127927347 | Down | 1.973803052 |
| WEE1 | -0.039200161 | 0.573912215 | 0.735426831 | NS | 0.241154531 |
| SWAP70 | -0.045923449 | 0.58473341 | 0.743660436 | NS | 0.233042091 |
| SBF2 | 0.125259935 | 0.118758512 | 0.339297335 | NS | 0.925335253 |
| ADM | 0.101522264 | 0.520101084 | 0.696688569 | NS | 0.283912241 |
| AMPD3 | 0.189676227 | 0.020008011 | 0.164002673 | Up | 1.698796081 |
| MTRNR2L8 | 0.518668966 | 0.007593256 | 0.11324821 | Up | 2.119571949 |
| RNF141 | 0.028227087 | 0.717003614 | 0.83278308 | NS | 0.144478655 |
| LYVE1 | 0.216102455 | 0.125196803 | 0.346831641 | NS | 0.902406762 |
| IRAG1 | 0.150266852 | 0.236895315 | 0.464700343 | NS | 0.625443528 |
| CTR9 | -0.113694754 | 0.045824124 | 0.228442337 | Down | 1.338905826 |
| EIF4G2 | 0.101349962 | 0.004022065 | 0.090296145 | Up | 2.395550868 |
| ZBED5 | 0.03096812 | 0.678633397 | 0.808429756 | NS | 0.168364771 |
| GALNT18 | 0.667243258 | 0.059019628 | NA | NS | 1.229003534 |
| CSNK2A3 | -1.751843033 | 1.25E-07 | NA | Down | 6.904264283 |
| USP47 | -0.06047647 | 0.167228779 | 0.395348919 | NS | 0.776688982 |
| DKK3 | 0.087269414 | 0.600468082 | 0.754648056 | NS | 0.221510073 |
| MICAL2 | -0.076661717 | 0.658978938 | 0.794898116 | NS | 0.181128466 |
| PARVA | 0.545774635 | 0.046068702 | 0.228777969 | Up | 1.336594024 |
| TEAD1 | 0.284684959 | 0.355431651 | 0.568601262 | NS | 0.449243901 |
| RASSF10 | 0.882212688 | 0.039431913 | NA | Up | 1.404152148 |
| ARNTL | 0.053746775 | 0.353177985 | 0.566698115 | NS | 0.452006376 |
| BTBD10 | -0.02828133 | 0.638112219 | 0.779952504 | NS | 0.195102939 |
| PTH | 0.272432013 | 0.376577103 | NA | NS | 0.42414609 |
| FAR1 | 0.012280422 | 0.868330165 | 0.925814592 | NS | 0.061315112 |
| SPON1 | 0.104617025 | 0.221226037 | 0.449877348 | NS | 0.655163761 |
| RRAS2 | -0.151203283 | 0.064934952 | 0.264064926 | NS | 1.187521475 |
| COPB1 | -0.115751515 | 0.101874301 | 0.31773461 | NS | 0.991935358 |
| PSMA1 | -0.108498086 | 0.107497195 | 0.324789019 | NS | 0.968602867 |
| PDE3B | -0.00698648 | 0.909091404 | 0.949563038 | NS | 0.041392449 |
| CYP2R1 | 0.026252775 | 0.609975619 | 0.760957709 | NS | 0.214687524 |
| CALCB | 0.144913147 | 0.472708306 | 0.6610873 | NS | 0.325406766 |
| CALCA | 0.845414453 | 0.002096946 | NA | Up | 2.678412676 |
| INSC | 0.269634506 | 0.224639424 | 0.452997453 | NS | 0.648514024 |
| SOX6 | -0.551191023 | 0.003454019 | 0.085194315 | Down | 2.46167522 |
| C11orf58 | -0.149189852 | 0.031986588 | 0.198142899 | Down | 1.49503208 |
| PLEKHA7 | 0.290735575 | 0.091877487 | 0.303701724 | NS | 1.03679089 |
| RPS13 | -0.295864196 | 0.025306291 | 0.18002011 | Down | 1.596771494 |
| PIK3C2A | -0.042065341 | 0.202893665 | 0.432933078 | NS | 0.692731512 |
| NUCB2 | -0.23829401 | 0.050028463 | 0.236733572 | NS | 1.300782837 |
| NCR3LG1 | -0.053790957 | 0.578624968 | 0.738460241 | NS | 0.23760283 |
| KCNJ11 | 0.658161102 | 0.047071346 | 0.230692912 | Up | 1.327243379 |
| ABCC8 | 0.456505721 | 0.275200333 | 0.499918696 | NS | 0.560351045 |
| USH1C | 0.661876241 | 0.136760733 | 0.361173236 | NS | 0.864038581 |
| OTOG | 0.799400252 | 0.043790745 | 0.224344829 | Up | 1.358617671 |
| MYOD1 | 1.449077016 | 0.008009823 | NA | Up | 2.096377076 |
| KCNC1 | 0.174587332 | 0.198682512 | 0.428619714 | NS | 0.701840359 |
| SERGEF | -0.106739176 | 0.103677239 | 0.319528479 | NS | 0.984316577 |
| TPH1 | 0.109328779 | 0.442387343 | 0.636844235 | NS | 0.354197307 |
| SAAL1 | -0.081854486 | 0.191691408 | 0.420934764 | NS | 0.717397352 |
| MRGPRX3 | 0.493487931 | 0.354132098 | NA | NS | 0.450834708 |
| MRGPRX4 | 0.233839383 | 0.688075206 | NA | NS | 0.162364091 |
| SAA2 | 0.297393793 | 0.477230389 | NA | NS | 0.321271909 |
| HPS5 | -0.128101966 | 0.060345629 | 0.256205353 | NS | 1.219354181 |
| GTF2H1 | -0.248742973 | 1.25E-04 | 0.01784012 | Down | 3.901649773 |
| LDHA | -0.071619363 | 0.429398868 | 0.627054318 | NS | 0.367139105 |
| LDHC | -0.391689393 | 0.389079912 | NA | NS | 0.409961191 |
| LDHAL6A | 0.338652934 | 0.00170613 | 0.065122985 | Up | 2.767987861 |
| TSG101 | -0.113414506 | 0.056160346 | 0.249221499 | NS | 1.250570227 |
| UEVLD | -0.065312156 | 0.330392385 | 0.547523381 | NS | 0.480969971 |
| SPTY2D1OS | 0.09633806 | 0.501858932 | 0.68323853 | NS | 0.299418342 |
| SPTY2D1 | 0.064178453 | 0.174687374 | 0.404024697 | NS | 0.757738484 |
| TMEM86A | 0.204555641 | 0.061686951 | 0.258215532 | NS | 1.209806697 |
| IGSF22 | 0.372308356 | 0.107613597 | 0.324922461 | NS | 0.968132852 |
| PTPN5 | 0.503533189 | 0.280019995 | 0.50348007 | NS | 0.552810957 |
| MRGPRX1 | 0.413149565 | 0.440460753 | NA | NS | 0.356092783 |
| MRGPRX2 | 0.319597932 | 0.420964056 | NA | NS | 0.375754985 |
| ZDHHC13 | 0.10894713 | 0.023942172 | 0.175295276 | Up | 1.620836453 |
| CSRP3 | 0.439639007 | 0.135634084 | NA | NS | 0.867631163 |
| E2F8 | 0.468401584 | 0.061320704 | 0.257517688 | NS | 1.212392868 |
| NAV2 | 0.438514638 | 0.102716 | 0.318624114 | NS | 0.988361901 |
| DBX1 | 0.425252018 | 0.394552839 | NA | NS | 0.403894828 |
| HTATIP2 | -0.033048552 | 0.6847096 | 0.812571406 | NS | 0.164493583 |
| PRMT3 | -0.064400243 | 0.117282301 | 0.337267611 | NS | 0.930767523 |
| SLC6A5 | 0.44952425 | 0.31944158 | 0.53856383 | NS | 0.495608555 |
| NELL1 | 0.536114103 | 0.23921517 | 0.467477036 | NS | 0.621211282 |
| ANO5 | 0.14564351 | 0.323612128 | 0.54228177 | NS | 0.489975211 |
| SLC17A6 | 0.403781323 | 0.462664865 | NA | NS | 0.334733479 |
| FANCF | -0.06828938 | 0.398155397 | 0.603175118 | NS | 0.399947393 |
| GAS2 | 0.078059383 | 0.491024931 | 0.675362018 | NS | 0.308896457 |
| SVIP | -0.172424574 | 0.077872496 | 0.282355958 | NS | 1.108615903 |
| CCDC179 | 0.339125662 | 0.072158865 | NA | NS | 1.141710309 |
| LUZP2 | 0.427244066 | 0.360564086 | 0.573327689 | NS | 0.443017533 |
| ANO3 | 0.340940752 | 0.447456823 | 0.641420994 | NS | 0.349248865 |
| MUC15 | 0.442282638 | 0.382722793 | NA | NS | 0.417115673 |
| SLC5A12 | 0.705721699 | 0.131312997 | 0.354010326 | NS | 0.881692288 |
| FIBIN | 0.279747854 | 0.607031991 | NA | NS | 0.216788421 |
| BBOX1 | 0.569811262 | 0.31946129 | NA | NS | 0.495581759 |
| CCDC34 | -0.325350916 | 0.090360346 | 0.301065195 | NS | 1.044022114 |
| LGR4 | -0.172682654 | 0.127063611 | 0.348830209 | NS | 0.895978807 |
| LIN7C | -0.168741764 | 0.01621019 | 0.150606807 | Down | 1.790211887 |
| BDNF | 0.331304468 | 0.129882109 | 0.351977288 | NS | 0.886450668 |
| KIF18A | -0.483648419 | 9.57E-04 | 0.051584141 | Down | 3.018968274 |
| METTL15 | -0.071665196 | 0.136005762 | 0.360134579 | NS | 0.866442691 |
| KCNA4 | 0.499865709 | 0.341783337 | NA | NS | 0.466249114 |
| FSHB | 0.272799007 | 0.595337679 | NA | NS | 0.22523663 |
| ARL14EP | -0.157647796 | 0.080303631 | 0.285933942 | NS | 1.095264817 |
| MPPED2 | 0.318342829 | 0.260549771 | 0.486713772 | NS | 0.584109305 |
| DCDC1 | 0.374855696 | 0.226586721 | 0.455200797 | NS | 0.644765545 |
| DNAJC24 | -0.080181398 | 0.164911809 | 0.392552916 | NS | 0.782748244 |
| IMMP1L | -0.095202262 | 0.297856744 | 0.519457444 | NS | 0.525992562 |
| ELP4 | -0.029725934 | 0.619923177 | 0.767514358 | NS | 0.207662126 |
| PAX6 | 0.707375733 | 0.042269689 | 0.220751111 | Up | 1.373970947 |
| RCN1 | -0.047911051 | 0.584628926 | 0.743595005 | NS | 0.233119701 |
| WT1 | 0.545312189 | 0.201479975 | 0.431363409 | NS | 0.695768112 |
| EIF3M | -0.212836752 | 0.020985112 | 0.167842555 | Down | 1.67808871 |
| CCDC73 | 0.291474915 | 0.228006611 | 0.456786951 | NS | 0.64205256 |
| PRRG4 | 0.201511912 | 0.13949723 | 0.36380609 | NS | 0.855434416 |
| QSER1 | -0.058173043 | 0.44010499 | 0.635075576 | NS | 0.356443708 |
| DEPDC7 | 0.146630918 | 0.312726938 | 0.532416914 | NS | 0.504834708 |
| TCP11L1 | 0.115833784 | 0.073601024 | 0.27580509 | NS | 1.133116144 |
| CSTF3 | -0.002319555 | 0.953853582 | 0.975032942 | NS | 0.020518285 |
| HIPK3 | -0.004293038 | 0.944246838 | 0.969563356 | NS | 0.024914461 |
| KIAA1549L | 0.562895125 | 0.159719801 | 0.387243582 | NS | 0.79664124 |
| C11orf91 | 1.241925124 | 0.008630061 | NA | Up | 2.063986114 |
| CD59 | 0.155992817 | 0.119173102 | 0.339845894 | NS | 0.923821758 |
| FBXO3 | 0.034949708 | 0.430737495 | 0.627828924 | NS | 0.365787322 |
| LMO2 | -0.028187901 | 0.733891002 | 0.842822772 | NS | 0.134368437 |
| CAPRIN1 | -0.106364387 | 0.018815166 | 0.159240554 | Down | 1.725491938 |
| NAT10 | -0.145275356 | 0.026655565 | 0.184430741 | Down | 1.574212106 |
| ABTB2 | 0.414491469 | 0.002084354 | 0.071675497 | Up | 2.681028468 |
| CAT | 0.196005654 | 0.060725554 | 0.256526216 | NS | 1.216628517 |
| ELF5 | 0.722848923 | 0.079186526 | NA | NS | 1.101348708 |
| EHF | 0.081741397 | 0.727737081 | 0.838910486 | NS | 0.138025495 |
| APIP | -0.192908278 | 0.071216711 | 0.272469948 | NS | 1.147418089 |
| PDHX | -0.154029964 | 0.082565471 | 0.289685039 | NS | 1.08320154 |
| CD44 | 0.215964022 | 0.001099221 | 0.05356245 | Up | 2.958915121 |
| SLC1A2 | 0.42728869 | 0.1647188 | 0.392382726 | NS | 0.783256829 |
| PAMR1 | 0.480330024 | 0.256796045 | NA | NS | 0.590411669 |
| FJX1 | 0.900813889 | 0.130848272 | NA | NS | 0.883232009 |
| TRIM44 | 0.046299958 | 0.331710906 | 0.54864219 | NS | 0.479240249 |
| LDLRAD3 | 0.030419369 | 0.829946462 | 0.903352211 | NS | 0.080949922 |
| COMMD9 | -0.144794634 | 0.061906046 | 0.25852886 | NS | 1.208266932 |
| PRR5L | -0.171674665 | 0.048752917 | 0.234036095 | Down | 1.31199939 |
| TRAF6 | 0.067700372 | 0.202522543 | 0.432624874 | NS | 0.693526629 |
| RAG1 | 0.093511157 | 0.512095815 | 0.690696017 | NS | 0.290648773 |
| RAG2 | 0.35001759 | 0.27501663 | NA | NS | 0.560641044 |
| IFTAP | -0.04205102 | 0.811559369 | 0.891944173 | NS | 0.090679704 |
| LRRC4C | 0.556803293 | 0.210453007 | 0.440341584 | NS | 0.676844864 |
| API5 | -0.121851416 | 0.042853044 | 0.222091066 | Down | 1.36801832 |
| TTC17 | -0.050572195 | 0.119446689 | 0.340007095 | NS | 0.922825883 |
| HSD17B12 | -0.043387654 | 0.599046968 | 0.753683091 | NS | 0.222539126 |
| ALKBH3 | -0.055679893 | 0.33836881 | 0.554290685 | NS | 0.470609675 |
| C11orf96 | 0.650636671 | 0.001980125 | NA | Up | 2.703307467 |
| ACCSL | 0.136278768 | 0.658530993 | NA | NS | 0.181423781 |
| ACCS | -0.278763513 | 0.043657421 | 0.224086881 | Down | 1.359941926 |
| EXT2 | -0.146113056 | 0.008035361 | 0.115441239 | Down | 2.094994607 |
| ALX4 | 0.415772376 | 0.029855441 | 0.192425018 | Up | 1.524976508 |
| CD82 | 0.030735779 | 0.682038992 | 0.810483683 | NS | 0.166190796 |
| TSPAN18 | 0.118810563 | 0.331395256 | 0.548342905 | NS | 0.479653713 |
| TP53I11 | 0.074005169 | 0.625622788 | 0.771733981 | NS | 0.203687441 |
| PRDM11 | 0.183126679 | 0.079693983 | 0.285090847 | NS | 1.098574466 |
| SYT13 | 0.459369223 | 0.281938723 | NA | NS | 0.549845272 |
| CHST1 | 0.809403516 | 0.081177963 | NA | NS | 1.090561851 |
| SLC35C1 | 0.096861903 | 0.154493287 | 0.381395414 | NS | 0.811090388 |
| CRY2 | -0.129353237 | 0.008826463 | 0.119262139 | Down | 2.054213286 |
| MAPK8IP1 | 0.503413496 | 0.039997737 | 0.215862456 | Up | 1.39796458 |
| C11orf94 | 0.28200277 | 0.087363666 | 0.296859543 | NS | 1.058669148 |
| PEX16 | -0.114571489 | 0.022255032 | 0.170666732 | Down | 1.652571779 |
| LARGE2 | 0.22296209 | 0.079452943 | 0.284900136 | NS | 1.099890014 |
| PHF21A | 0.152371017 | 0.097640398 | 0.312312905 | NS | 1.010370458 |
| CREB3L1 | 0.653030542 | 0.17757864 | 0.407521884 | NS | 0.750609275 |
| DGKZ | 0.043576518 | 0.692469045 | 0.81738615 | NS | 0.159599636 |
| MDK | 0.557298107 | 0.019791648 | NA | Up | 1.703518046 |
| CHRM4 | 0.614446757 | 0.084466617 | NA | NS | 1.073314902 |
| AMBRA1 | 0.013486352 | 0.843110556 | 0.911847772 | NS | 0.074115473 |
| HARBI1 | -0.061238899 | 0.369719834 | 0.581122148 | NS | 0.43212725 |
| ATG13 | 0.11314003 | 0.084788619 | 0.292929394 | NS | 1.071662439 |
| ARHGAP1 | 0.183566244 | 0.121117063 | 0.341745092 | NS | 0.916794669 |
| ZNF408 | -0.020563394 | 0.758045034 | 0.858762731 | NS | 0.120304993 |
| F2 | 0.449167745 | 0.265006435 | 0.49119273 | NS | 0.57674358 |
| CKAP5 | -0.155338398 | 0.059952415 | 0.255495014 | NS | 1.222193319 |
| LRP4 | 0.381819188 | 0.362628738 | 0.575212866 | NS | 0.440537781 |
| C11orf49 | 0.101079454 | 0.146653111 | 0.372132189 | NS | 0.833708719 |
| ARFGAP2 | -0.139932496 | 0.001737103 | 0.065368236 | Down | 2.760174498 |
| PACSIN3 | 0.688554526 | 0.042176011 | NA | Up | 1.374934496 |
| DDB2 | 0.082551053 | 0.219719708 | 0.449082183 | NS | 0.658130987 |
| ACP2 | -0.004207769 | 0.954836037 | 0.97562483 | NS | 0.020071198 |
| NR1H3 | 0.003759455 | 0.970930335 | 0.983510672 | NS | 0.01281193 |
| MADD | -0.075296987 | 0.316362429 | 0.535897363 | NS | 0.499815099 |
| MYBPC3 | 0.09155049 | 0.595633028 | 0.750959956 | NS | 0.225021229 |
| SPI1 | 0.205539714 | 0.200719226 | 0.430459005 | NS | 0.697411026 |
| SLC39A13 | 0.080654268 | 0.280686227 | 0.503855726 | NS | 0.551778896 |
| PSMC3 | -0.241541148 | 0.004113361 | 0.090843006 | Down | 2.385803156 |
| RAPSN | 0.226439191 | 0.305486553 | 0.526667648 | NS | 0.515007902 |
| CELF1 | 0.005998101 | 0.911929729 | 0.951177969 | NS | 0.040038626 |
| NDUFS3 | -0.172474548 | 0.117726977 | 0.337782914 | NS | 0.929124009 |
| PTPMT1 | -0.111071957 | 0.187448838 | 0.416913012 | NS | 0.727117248 |
| KBTBD4 | -0.1788917 | 0.087020617 | 0.29656937 | NS | 1.060377842 |
| MTCH2 | -0.215438928 | 0.006819983 | 0.109684865 | Down | 2.166216717 |
| AGBL2 | -7.27E-04 | 0.995995656 | 0.997694638 | NS | 0.001742556 |
| FNBP4 | -0.067144813 | 0.241480129 | 0.46935228 | NS | 0.617118601 |
| NUP160 | -0.034965412 | 0.434823954 | 0.630833536 | NS | 0.361686539 |
| PTPRJ | -0.001867583 | 0.983306923 | 0.99010742 | NS | 0.007310903 |
| OR4B1 | 0.442861797 | 0.472277737 | NA | NS | 0.325802527 |
| OR4X2 | 0.948407086 | 0.102663902 | NA | NS | 0.988582235 |
| OR4X1 | 0.571773211 | 0.331984251 | NA | NS | 0.478882518 |
| OR4S1 | 0.490667495 | 0.391764162 | NA | NS | 0.406975295 |
| OR4C3 | 0.11679022 | 0.848553704 | NA | NS | 0.071320666 |
| OR4C5 | 0.465633411 | 0.400437787 | NA | NS | 0.397464947 |
| OR4A47 | 0.650909992 | 0.222437789 | NA | NS | 0.65279143 |
| TRIM51GP | 0.789400348 | 0.164571442 | NA | NS | 0.783645527 |
| TRIM49B | 0.793746051 | 0.19809783 | NA | NS | 0.703120281 |
| TRIM64C | 0.274907698 | 0.679087035 | NA | NS | 0.168074561 |
| FOLH1 | 0.425302254 | 0.349590408 | 0.563682195 | NS | 0.456440492 |
| OR4C13 | 1.039028414 | 0.073016707 | NA | NS | 1.136577759 |
| OR4C46 | 0.691119594 | 0.331107124 | NA | NS | 0.480031475 |
| OR4A5 | 0.040396896 | 0.953974706 | NA | NS | 0.02046314 |
| TRIM48 | 0.221140585 | 0.739204497 | NA | NS | 0.1312354 |
| OR4A16 | -0.531431498 | 0.464144985 | NA | NS | 0.333346338 |
| OR4A15 | 0.596573114 | 0.398808204 | NA | NS | 0.399235916 |
| OR4C15 | 0.271259586 | 0.68293205 | NA | NS | 0.165622505 |
| OR4C16 | 1.161896823 | 0.088012391 | NA | NS | 1.055456182 |
| OR4C11 | 0.499626339 | 0.493993495 | NA | NS | 0.306278769 |
| OR4P4 | 0.910799774 | 0.172932247 | NA | NS | 0.762124015 |
| OR4S2 | 0.481601912 | 0.416489238 | NA | NS | 0.380396217 |
| OR4C6 | 0.587166243 | 0.375928276 | NA | NS | 0.424895007 |
| OR5D3P | 0.114299537 | 0.835664645 | NA | NS | 0.077967972 |
| OR5D13 | 0.422464598 | 0.498748472 | NA | NS | 0.302118422 |
| OR5D14 | 0.408949231 | 0.606745344 | NA | NS | 0.216993547 |
| OR5L1 | 0.692679684 | 0.276375314 | NA | NS | 0.558500751 |
| OR5D18 | 0.241156587 | 0.728420983 | NA | NS | 0.137617553 |
| OR5L2 | 0.854525983 | 0.175849843 | NA | NS | 0.754858016 |
| OR5D16 | 0.649463458 | 0.342690693 | NA | NS | 0.46509769 |
| TRIM51 | 0.274480028 | 0.545495984 | NA | NS | 0.263208442 |
| OR5W2 | 0.583581218 | 0.425839771 | NA | NS | 0.37075378 |
| OR5I1 | 0.220890612 | 0.722855628 | NA | NS | 0.140948433 |
| OR10AG1 | 0.430623392 | 0.409158337 | NA | NS | 0.388108595 |
| OR5F1 | 0.417134913 | 0.549555158 | NA | NS | 0.259988711 |
| OR5AS1 | 0.273054413 | 0.577825096 | 0.738097515 | NS | 0.2382036 |
| OR8I2 | 0.357635339 | 0.639960175 | NA | NS | 0.193847052 |
| OR8H2 | 0.463960012 | 0.415134101 | NA | NS | 0.38181159 |
| OR8H3 | 0.291635563 | 0.664934996 | NA | NS | 0.177220809 |
| OR8J3 | 0.571674276 | 0.306028496 | NA | NS | 0.514238132 |
| OR8K5 | 0.350292693 | 0.707621771 | NA | NS | 0.150198814 |
| OR5J2 | 0.829760552 | 0.244562098 | NA | NS | 0.611610848 |
| OR8U3 | 0.406930898 | 0.53490781 | NA | NS | 0.271721061 |
| OR5M3 | 0.658190385 | 0.28875764 | NA | NS | 0.539466516 |
| OR5M8 | 0.276595819 | 0.723760722 | NA | NS | 0.14040499 |
| OR5M11 | 0.930365867 | 0.271061814 | NA | NS | 0.566931659 |
| OR5M10 | 0.566405722 | 0.373708734 | NA | NS | 0.427466753 |
| OR5M1 | 0.609796976 | 0.257811409 | NA | NS | 0.588697867 |
| OR5AP2 | 0.689168924 | 0.289574254 | NA | NS | 0.538240053 |
| OR5AR1 | 0.103007487 | 0.882593292 | NA | NS | 0.054239378 |
| OR9G1 | 0.134133708 | 0.821835236 | NA | NS | 0.085215242 |
| OR5AK2 | 0.043065617 | 0.948003467 | NA | NS | 0.023190074 |
| LRRC55 | 0.706142699 | 0.153955661 | NA | NS | 0.812604337 |
| APLNR | 0.788837019 | 0.132498049 | NA | NS | 0.877790516 |
| TNKS1BP1 | 0.247010192 | 0.050023555 | 0.236733572 | NS | 1.30082545 |
| SSRP1 | -0.131723448 | 0.015472176 | 0.147951473 | Down | 1.810448598 |
| P2RX3 | 0.517457523 | 0.343510794 | NA | NS | 0.464059612 |
| PRG3 | 0.995739299 | 0.041573399 | NA | Up | 1.381184469 |
| PRG2 | 0.475991556 | 0.423699827 | NA | NS | 0.372941713 |
| SLC43A3 | 0.046265542 | 0.538989374 | 0.7098703 | NS | 0.268419797 |
| RTN4RL2 | 0.788228399 | 0.060718154 | NA | NS | 1.216681438 |
| SLC43A1 | -0.00519402 | 0.963851823 | 0.979853269 | NS | 0.015989727 |
| TIMM10 | 0.104091878 | 0.389120726 | 0.596625801 | NS | 0.409915636 |
| SMTNL1 | 0.410034666 | 0.003791508 | 0.088644036 | Up | 2.421188003 |
| UBE2L6 | 0.133630801 | 0.155145558 | 0.382552064 | NS | 0.809260654 |
| SERPING1 | 0.654329823 | 0.002212835 | 0.072832159 | Up | 2.655050885 |
| YPEL4 | 0.187417216 | 0.411404599 | 0.614050321 | NS | 0.385730858 |
| CLP1 | -0.127023072 | 0.119402509 | 0.340007095 | NS | 0.922986546 |
| ZDHHC5 | 0.018848443 | 0.576628451 | 0.737358732 | NS | 0.239103934 |
| MED19 | 0.056376063 | 0.337790961 | 0.553781471 | NS | 0.471351976 |
| TMX2 | -0.083438521 | 0.170343824 | 0.39869447 | NS | 0.768673607 |
| SELENOH | 0.0565691 | 0.49251286 | 0.676474123 | NS | 0.307582425 |
| BTBD18 | 0.277534121 | 0.001590254 | 0.063782832 | Up | 2.798533506 |
| CTNND1 | 0.098661545 | 0.435020037 | 0.630958649 | NS | 0.361490739 |
| OR9Q1 | 0.047208769 | 0.940191678 | NA | NS | 0.026783597 |
| OR6Q1 | 0.227450582 | 0.778711397 | NA | NS | 0.108623469 |
| OR9I1 | 0.601949947 | 0.202365665 | NA | NS | 0.693863171 |
| OR9Q2 | 0.326942528 | 0.507906069 | NA | NS | 0.294216597 |
| OR1S2 | 0.3292162 | 0.536675462 | NA | NS | 0.270288261 |
| OR1S1 | 0.280015595 | 0.618942795 | NA | NS | 0.208349488 |
| OR10Q1 | 0.191632762 | 0.769731464 | NA | NS | 0.113660761 |
| OR10W1 | 0.443488828 | 0.435670152 | NA | NS | 0.360842193 |
| OR5B17 | 0.696683956 | 0.318466241 | NA | NS | 0.496936599 |
| OR5B3 | 0.730139708 | 0.17856354 | NA | NS | 0.748207212 |
| OR5B2 | 0.414128476 | 0.309204977 | NA | NS | 0.509753524 |
| OR5B12 | 0.645685463 | 0.14162369 | NA | NS | 0.848864093 |
| OR5B21 | 0.466745666 | 0.071068997 | NA | NS | 1.148319813 |
| LPXN | 0.045000404 | 0.319745694 | 0.538985786 | NS | 0.495195295 |
| ZFP91 | -0.099316715 | 0.064893165 | 0.264064926 | NS | 1.187801044 |
| CNTF | 0.065055157 | 0.423393754 | 0.622593337 | NS | 0.373255553 |
| GLYAT | 0.483212322 | 0.233632615 | NA | NS | 0.631466531 |
| GLYATL2 | -0.250252415 | 0.489678945 | NA | NS | 0.31008857 |
| GLYATL1 | 0.141889992 | 0.494860431 | 0.677832837 | NS | 0.305517271 |
| GLYATL1B | 0.015382917 | 0.923373747 | 0.958009674 | NS | 0.034622477 |
| FAM111B | -0.198946921 | 0.123282331 | 0.344057977 | NS | 0.909099162 |
| FAM111A | -0.057142306 | 0.191358289 | 0.42070534 | NS | 0.718152722 |
| DTX4 | 0.171217053 | 0.035228635 | 0.205507211 | Up | 1.453104182 |
| MPEG1 | 0.117114899 | 0.187219305 | 0.416766769 | NS | 0.727649371 |
| OR5AN1 | 0.239671045 | 0.418369463 | 0.618901466 | NS | 0.378440022 |
| OR5A2 | 0.385806738 | 0.367760729 | 0.579795963 | NS | 0.434434648 |
| OR5A1 | 0.349940414 | 0.447501912 | 0.641420994 | NS | 0.349205105 |
| OR4D6 | 0.52098077 | 0.36527753 | NA | NS | 0.437377043 |
| OR4D10 | 0.518580757 | 0.363880687 | NA | NS | 0.439040994 |
| OR4D11 | 0.692736655 | 0.283435657 | NA | NS | 0.547545515 |
| OR4D9 | 0.199460147 | 0.682753846 | NA | NS | 0.165735845 |
| OSBP | -0.029475021 | 0.45065019 | 0.643924649 | NS | 0.346160441 |
| PATL1 | 0.16351777 | 0.034846137 | 0.204782731 | Up | 1.457845364 |
| OR10V1 | -0.038721145 | 0.951699096 | NA | NS | 0.021500343 |
| STX3 | 0.205701406 | 0.076735384 | 0.280778681 | NS | 1.115004329 |
| MRPL16 | -0.027486378 | 0.689587635 | 0.81541388 | NS | 0.161410534 |
| CBLIF | 0.221655036 | 0.281779121 | 0.504863889 | NS | 0.55009119 |
| TCN1 | 0.171823322 | 0.331029769 | 0.548094548 | NS | 0.480132948 |
| OOSP3 | 0.409766689 | 0.079489292 | NA | NS | 1.099691372 |
| MS4A3 | -0.085751964 | 0.71893487 | 0.834077883 | NS | 0.143310451 |
| MS4A2 | -0.046433457 | 0.843462506 | 0.912124767 | NS | 0.073934218 |
| MS4A6A | -0.103888904 | 0.287967321 | 0.511141864 | NS | 0.540656793 |
| MS4A4A | 0.001898502 | 0.987964644 | 0.992994219 | NS | 0.005258597 |
| MS4A4E | -0.003696098 | 0.976046272 | 0.986208935 | NS | 0.010529593 |
| MS4A6E | 0.341306892 | 0.539890459 | NA | NS | 0.267694348 |
| MS4A7 | -0.082903325 | 0.474938775 | 0.663032479 | NS | 0.323362373 |
| MS4A14 | 0.201134214 | 0.049420117 | 0.235256601 | Up | 1.306096235 |
| MS4A5 | 0.564399611 | 0.211862875 | NA | NS | 0.673945138 |
| MS4A1 | -0.041781385 | 0.737051726 | 0.84513087 | NS | 0.132502033 |
| MS4A12 | 0.545316445 | 0.240477014 | NA | NS | 0.618926429 |
| MS4A13 | 0.389012617 | 0.491821871 | NA | NS | 0.308192162 |
| MS4A8 | 0.722476722 | 0.104303683 | NA | NS | 0.981700357 |
| MS4A18 | 0.479362234 | 0.366481524 | NA | NS | 0.435947916 |
| MS4A15 | 0.707910867 | 0.119998906 | NA | NS | 0.920822711 |
| MS4A10 | 0.512089331 | 0.336523372 | NA | NS | 0.472984768 |
| CCDC86 | 0.043071782 | 0.66674808 | 0.800727034 | NS | 0.176038226 |
| PTGDR2 | 0.214381576 | 0.188127235 | 0.417570259 | NS | 0.725548328 |
| ZP1 | 0.288733092 | 0.126929151 | 0.348807379 | NS | 0.896438624 |
| PRPF19 | -0.125158945 | 0.1124203 | 0.330929727 | NS | 0.949155262 |
| TMEM109 | -0.15252551 | 0.073997283 | 0.276267127 | NS | 1.130784227 |
| TMEM132A | 0.312780814 | 0.009128887 | 0.12077977 | Up | 2.039582146 |
| SLC15A3 | -0.005592769 | 0.943305662 | 0.968944487 | NS | 0.025347559 |
| CD6 | 0.013000337 | 0.884345531 | 0.934752451 | NS | 0.053378015 |
| CD5 | -0.045900906 | 0.630816204 | 0.775188655 | NS | 0.200097159 |
| VPS37C | 0.014975216 | 0.913638575 | 0.951982359 | NS | 0.039225572 |
| PGA3 | 0.438849738 | 0.279008123 | NA | NS | 0.554383153 |
| PGA5 | 0.053046372 | 0.900084578 | NA | NS | 0.045716679 |
| VWCE | -0.117873678 | 0.704157166 | 0.824734454 | NS | 0.152330396 |
| DDB1 | -0.230733467 | 0.00188067 | 0.067721854 | Down | 2.725687458 |
| TKFC | -0.033561728 | 0.671500865 | 0.804283919 | NS | 0.172953424 |
| CYB561A3 | -0.051345344 | 0.438381195 | 0.633791149 | NS | 0.358148084 |
| TMEM138 | -0.085904683 | 0.333223315 | 0.550016309 | NS | 0.47726462 |
| TMEM216 | 0.017775228 | 0.827319591 | 0.901734909 | NS | 0.082326691 |
| CPSF7 | 0.217979678 | 0.046908182 | 0.230386273 | Up | 1.328751396 |
| SDHAF2 | -0.070217125 | 0.192469934 | 0.421409791 | NS | 0.715637104 |
| PPP1R32 | 0.215232808 | 0.269784368 | 0.495199294 | NS | 0.568983218 |
| LRRC10B | 1.032406714 | 0.084695458 | NA | NS | 1.07213988 |
| SYT7 | 0.887166715 | 0.04890742 | 0.23437492 | Up | 1.310625244 |
| DAGLA | 0.398369727 | 0.101645928 | 0.317403374 | NS | 0.992910016 |
| MYRF | 0.272972839 | 0.027125147 | 0.185716026 | Up | 1.566627906 |
| TMEM258 | -0.156009486 | 0.096790759 | 0.310921075 | NS | 1.014166106 |
| FEN1 | -0.194700317 | 0.03963072 | 0.215433834 | Down | 1.401968041 |
| FADS2 | 0.132742165 | 0.379383648 | 0.589267593 | NS | 0.420921392 |
| FADS1 | 0.021381803 | 0.815770141 | 0.894940016 | NS | 0.088432195 |
| FADS3 | 0.08059991 | 0.421198561 | 0.621219053 | NS | 0.375513121 |
| RAB3IL1 | -0.403646635 | 0.12733964 | 0.348830209 | NS | 0.895036381 |
| BEST1 | 0.085226844 | 0.385046128 | 0.593353685 | NS | 0.414487239 |
| FTH1 | 0.262073547 | 0.193470766 | 0.422717401 | NS | 0.713384649 |
| INCENP | -0.089557457 | 0.304007213 | 0.525263915 | NS | 0.517116112 |
| SCGB1D2 | 0.316182283 | 0.65266092 | NA | NS | 0.185312391 |
| SCGB2A2 | 0.684698719 | 0.172140182 | NA | NS | 0.764117742 |
| SCGB1D4 | 0.459670585 | 0.572870515 | NA | NS | 0.24194353 |
| ASRGL1 | 0.160099542 | 0.067269063 | 0.267139948 | NS | 1.172184621 |
| SCGB1A1 | 0.84902019 | 0.065009108 | NA | NS | 1.187025793 |
| AHNAK | 9.14E-04 | 0.990819514 | 0.994641383 | NS | 0.004005449 |
| TUT1 | 0.026770555 | 0.770098753 | 0.865925463 | NS | 0.11345358 |
| MTA2 | -0.240658198 | 0.001964511 | 0.069745459 | Down | 2.706745526 |
| EML3 | -0.062708301 | 0.544310739 | 0.713350761 | NS | 0.264153097 |
| ROM1 | 0.124641235 | 0.376236879 | 0.58628135 | NS | 0.424538637 |
| B3GAT3 | -0.119452448 | 0.074119806 | 0.276554545 | NS | 1.130065726 |
| GANAB | 0.09474943 | 0.157751298 | 0.385244212 | NS | 0.80202706 |
| INTS5 | -0.009617569 | 0.884597326 | 0.934889154 | NS | 0.053254378 |
| C11orf98 | 0.324518325 | 0.298878615 | NA | NS | 0.524505158 |
| LBHD1 | 0.09161051 | 0.243588977 | 0.471490428 | NS | 0.613342369 |
| CSKMT | 0.076476248 | 0.345197169 | 0.560099767 | NS | 0.461932774 |
| UQCC3 | -0.180676085 | 0.061251195 | 0.257380382 | NS | 1.212885432 |
| UBXN1 | -0.200114281 | 0.014803011 | 0.145877179 | Down | 1.829649929 |
| LRRN4CL | 1.079067932 | 0.033710033 | NA | Up | 1.472240828 |
| BSCL2 | 0.003244177 | 0.976723469 | 0.986489187 | NS | 0.010228377 |
| GNG3 | 0.191264992 | 0.435194724 | NA | NS | 0.361316379 |
| HNRNPUL2 | 0.037523726 | 0.468809335 | 0.658045562 | NS | 0.329003749 |
| TTC9C | -0.070248491 | 0.114030079 | 0.33250813 | NS | 0.942980574 |
| ZBTB3 | 0.191221955 | 0.007033978 | 0.110626998 | Up | 2.152798978 |
| POLR2G | -0.177190502 | 0.018090896 | 0.156740993 | Down | 1.742539931 |
| TAF6L | -0.212597056 | 0.003323473 | 0.085084692 | Down | 2.47840791 |
| TMEM223 | -0.048933611 | 0.503107436 | 0.684214162 | NS | 0.298339264 |
| TMEM179B | 0.089827726 | 0.189668675 | 0.419082681 | NS | 0.722004391 |
| NXF1 | 0.046835273 | 0.506583432 | 0.686666569 | NS | 0.295349018 |
| STX5 | -0.02544172 | 0.609781597 | 0.760957709 | NS | 0.214825687 |
| TEX54 | 0.689378574 | 0.028534772 | NA | Up | 1.54462559 |
| WDR74 | -0.270269626 | 0.009919054 | 0.125229427 | Down | 2.003529734 |
| SLC3A2 | -0.187231542 | 0.0125926 | 0.135545097 | Down | 1.89988459 |
| CHRM1 | 0.801924046 | 0.127403554 | NA | NS | 0.894818458 |
| SLC22A6 | 1.022739005 | 0.033073841 | NA | Up | 1.480515364 |
| SLC22A8 | 0.323363967 | 0.475332732 | 0.66338027 | NS | 0.323002278 |
| SLC22A24 | 0.870439482 | 0.116418524 | NA | NS | 0.933977909 |
| SLC22A25 | 0.159847052 | 0.696919367 | 0.820297213 | NS | 0.156817467 |
| SLC22A10 | 0.491419395 | 0.310533951 | NA | NS | 0.507890911 |
| SLC22A9 | 1.102651668 | 0.020645195 | NA | Up | 1.685181016 |
| PLAAT5 | 0.68254905 | 0.001702247 | 0.065122985 | Up | 2.768977381 |
| LGALS12 | 0.258162068 | 0.062159847 | 0.258974863 | NS | 1.206490061 |
| PLAAT4 | -0.133827734 | 0.169973589 | 0.398309642 | NS | 0.769618556 |
| PLAAT2 | -0.040460606 | 0.889768206 | NA | NS | 0.050723117 |
| PLAAT3 | -0.137990469 | 0.196414381 | 0.426175477 | NS | 0.706826717 |
| ATL3 | 0.062064531 | 0.260386399 | 0.486523288 | NS | 0.584381705 |
| RTN3 | 0.157917759 | 0.063578453 | 0.262056846 | NS | 1.196690046 |
| ZFTA | 0.07318249 | 0.228141362 | 0.456877344 | NS | 0.641795969 |
| SPINDOC | 0.094321262 | 0.298118377 | 0.519715846 | NS | 0.525611252 |
| MARK2 | 0.110007701 | 0.388971073 | 0.596625801 | NS | 0.410082696 |
| RCOR2 | 0.724733344 | 0.004317042 | 0.093096899 | Up | 2.36481368 |
| NAA40 | 0.13576976 | 0.161353354 | 0.389034501 | NS | 0.792222004 |
| COX8A | 0.050675344 | 0.57900466 | 0.738738981 | NS | 0.237317941 |
| OTUB1 | 0.013522272 | 0.790624964 | 0.878932718 | NS | 0.102029477 |
| MACROD1 | 0.146638357 | 0.306482716 | 0.527590736 | NS | 0.513594013 |
| FLRT1 | -0.021718867 | 0.932012924 | 0.962698899 | NS | 0.030578065 |
| STIP1 | -0.293929142 | 7.05E-04 | 0.043826722 | Down | 3.152078761 |
| FERMT3 | 0.042368238 | 0.638605795 | 0.780393251 | NS | 0.194767145 |
| TRPT1 | -0.023030846 | 0.774076354 | 0.868209356 | NS | 0.111216199 |
| NUDT22 | 0.077646412 | 0.234410638 | 0.46252024 | NS | 0.630022683 |
| DNAJC4 | 0.05391462 | 0.540174481 | 0.710491383 | NS | 0.267465937 |
| VEGFB | 0.243611255 | 0.079829902 | 0.285263759 | NS | 1.097834402 |
| FKBP2 | 0.422507914 | 0.121865024 | NA | NS | 0.914120923 |
| PPP1R14B | -0.208161583 | 0.04216485 | 0.220751111 | Down | 1.375049439 |
| PLCB3 | -0.048864423 | 0.623831328 | 0.770603295 | NS | 0.204932819 |
| BAD | 0.142754505 | 0.124965437 | 0.346400199 | NS | 0.903210086 |
| GPR137 | 0.110733803 | 0.203434394 | 0.433444748 | NS | 0.691575621 |
| KCNK4 | 1.091354596 | 0.048944323 | NA | Up | 1.310297672 |
| CATSPERZ | 1.340569865 | 0.036494016 | NA | Up | 1.437778346 |
| ESRRA | 0.005035161 | 0.93152924 | 0.962676894 | NS | 0.030803508 |
| TRMT112 | -0.338206451 | 0.001155432 | 0.055128572 | Down | 2.937255429 |
| PRDX5 | -0.402300415 | 8.46E-05 | 0.014142511 | Down | 4.072626316 |
| CCDC88B | -0.363758724 | 0.006876232 | 0.109855975 | Down | 2.162649451 |
| RPS6KA4 | -0.022132223 | 0.800953037 | 0.885420215 | NS | 0.096392947 |
| SLC22A11 | 0.763672949 | 0.10287667 | 0.318690856 | NS | 0.987683102 |
| SLC22A12 | 0.713070093 | 0.143425844 | NA | NS | 0.843372587 |
| NRXN2 | 0.506678281 | 0.13801957 | 0.362607036 | NS | 0.86005933 |
| RASGRP2 | -0.058787943 | 0.333535805 | 0.550147567 | NS | 0.476857538 |
| PYGM | -0.024401856 | 0.870043791 | 0.92673217 | NS | 0.060458888 |
| SF1 | 0.13736254 | 0.148847513 | 0.374840808 | NS | 0.827258418 |
| MAP4K2 | 0.019425235 | 0.816376478 | 0.895390743 | NS | 0.088109517 |
| MEN1 | 0.083021756 | 0.371779396 | 0.582785196 | NS | 0.429714683 |
| CDC42BPG | 0.267249181 | 0.100113295 | 0.315204109 | NS | 0.999508243 |
| EHD1 | -0.035256053 | 0.65187746 | 0.790244421 | NS | 0.185834035 |
| ATG2A | -0.159372635 | 0.325745856 | 0.544181052 | NS | 0.4871211 |
| PPP2R5B | 0.056637688 | 0.73144198 | 0.84106463 | NS | 0.135820117 |
| GPHA2 | 0.061375238 | 0.848594913 | NA | NS | 0.071299576 |
| MAJIN | 0.399203712 | 0.189416754 | NA | NS | 0.722581609 |
| BATF2 | 0.660949693 | 0.003999111 | 0.090296145 | Up | 2.398036535 |
| ARL2 | -0.223205929 | 0.03386822 | 0.202466715 | Down | 1.470207623 |
| ARL2-SNX15 | 0.402503363 | 0.52983325 | NA | NS | 0.275860791 |
| SNX15 | 0.019833927 | 0.878008537 | 0.931434766 | NS | 0.056501262 |
| SAC3D1 | -0.025591602 | 0.827478119 | 0.901736957 | NS | 0.082243481 |
| NAALADL1 | -0.210387376 | 0.063380198 | 0.261443316 | NS | 1.198046409 |
| CDCA5 | 0.379380559 | 0.090975693 | 0.30205689 | NS | 1.041074629 |
| ZFPL1 | -0.011314761 | 0.914101074 | 0.952101191 | NS | 0.039005781 |
| TMEM262 | 0.286511552 | 0.137493576 | 0.362356488 | NS | 0.861717592 |
| VPS51 | -0.129555922 | 0.09811398 | 0.312866385 | NS | 1.008269105 |
| TM7SF2 | -0.1821427 | 0.04755326 | 0.231617346 | Down | 1.322819704 |
| ZNHIT2 | 0.025278354 | 0.810350139 | 0.891085556 | NS | 0.091327289 |
| FAU | -0.235482999 | 0.07618738 | 0.279935021 | NS | 1.118116962 |
| SYVN1 | -0.019422809 | 0.864561547 | 0.923387051 | NS | 0.063204085 |
| MRPL49 | -0.049140471 | 0.476891561 | 0.66466471 | NS | 0.321580363 |
| SPDYC | 0.583262632 | 0.030832331 | 0.195007468 | Up | 1.510993643 |
| CAPN1 | -0.0025478 | 0.979287767 | 0.987728138 | NS | 0.009089671 |
| POLA2 | -0.002554686 | 0.974300388 | 0.985403414 | NS | 0.011307125 |
| CDC42EP2 | 0.392302959 | 0.065401548 | 0.264493423 | NS | 1.184411971 |
| DPF2 | -0.040003533 | 0.454258385 | 0.64659617 | NS | 0.342697047 |
| TIGD3 | 0.141956555 | 0.274927339 | 0.499616959 | NS | 0.560782072 |
| SLC25A45 | 0.097999629 | 0.360009974 | 0.572765303 | NS | 0.443685467 |
| FRMD8 | -0.039176477 | 0.59236569 | 0.749026006 | NS | 0.227410104 |
| SCYL1 | -0.023232524 | 0.739745846 | 0.847252419 | NS | 0.130917465 |
| LTBP3 | -3.59E-04 | 0.998349135 | 0.999332818 | NS | 7.18E-04 |
| ZNRD2 | -0.204448725 | 0.027475637 | 0.186246285 | Down | 1.56105223 |
| FAM89B | 0.147245012 | 0.08831611 | 0.298364885 | NS | 1.053960067 |
| EHBP1L1 | -0.196914263 | 0.072994634 | 0.274846078 | NS | 1.136709063 |
| KCNK7 | -0.035744542 | 0.846928053 | 0.913844348 | NS | 0.072153481 |
| MAP3K11 | -0.030459517 | 0.765775099 | 0.863717375 | NS | 0.11589876 |
| PCNX3 | 0.003055234 | 0.982468006 | 0.98964515 | NS | 0.007681584 |
| SIPA1 | 0.0874253 | 0.365447563 | 0.577723483 | NS | 0.437174929 |
| RELA | 0.10958244 | 0.196106418 | 0.425986774 | NS | 0.707508194 |
| KAT5 | -0.232044307 | 5.80E-05 | 0.01126434 | Down | 4.236285654 |
| RNASEH2C | 0.011226878 | 0.863301237 | 0.922858706 | NS | 0.063837637 |
| AP5B1 | 0.258593371 | 0.030514504 | 0.193961468 | Up | 1.515493685 |
| OVOL1 | 0.921019515 | 0.074742346 | NA | NS | 1.126433273 |
| CFL1 | 0.192620056 | 0.002884591 | 0.080466389 | Up | 2.539915742 |
| SNX32 | 0.100429203 | 0.150856574 | 0.377380367 | NS | 0.821435759 |
| MUS81 | -0.038411081 | 0.491124939 | 0.675418444 | NS | 0.308808012 |
| EFEMP2 | 0.045727996 | 0.630634547 | 0.775160605 | NS | 0.200222242 |
| CTSW | 0.094245561 | 0.334251114 | 0.550791033 | NS | 0.475927137 |
| FIBP | -0.134961363 | 0.054793083 | 0.247094287 | NS | 1.261274262 |
| CCDC85B | 0.317584031 | 0.01833054 | 0.157466628 | Up | 1.736824737 |
| FOSL1 | 0.175532093 | 0.253405911 | 0.480307652 | NS | 0.596183259 |
| C11orf68 | 0.42622625 | 1.23E-05 | 0.003428602 | Up | 4.910942436 |
| DRAP1 | -0.098731938 | 0.159527174 | 0.387098757 | NS | 0.797165328 |
| TSGA10IP | 0.451175852 | 0.163256683 | NA | NS | 0.787129033 |
| SART1 | -0.307140197 | 0.008750787 | 0.118782974 | Down | 2.057952887 |
| EIF1AD | -0.112922968 | 0.064123232 | 0.262789011 | NS | 1.192984598 |
| BANF1 | -0.070647607 | 0.49660012 | 0.679155378 | NS | 0.30399318 |
| CST6 | 0.186983813 | 0.668366689 | NA | NS | 0.174985203 |
| CATSPER1 | -0.047175833 | 0.723460657 | 0.836887361 | NS | 0.140585082 |
| GAL3ST3 | 0.575371347 | 0.250081939 | NA | NS | 0.601917672 |
| SF3B2 | 0.081696686 | 0.26631585 | 0.492225453 | NS | 0.574602986 |
| PACS1 | 0.104318628 | 0.360035338 | 0.572765303 | NS | 0.443654871 |
| KLC2 | 0.159055256 | 0.187507335 | 0.41696213 | NS | 0.726981739 |
| RAB1B | 0.045293825 | 0.65173882 | 0.790201835 | NS | 0.18592641 |
| CNIH2 | 0.104287109 | 0.55004306 | 0.71766001 | NS | 0.25960331 |
| YIF1A | -0.232903888 | 0.017962238 | 0.15641156 | Down | 1.745639563 |
| TMEM151A | 0.913210132 | 0.113698855 | NA | NS | 0.944243908 |
| CD248 | 0.158963576 | 0.613822371 | 0.76384264 | NS | 0.211957287 |
| RIN1 | 0.061601115 | 0.597245801 | 0.752206519 | NS | 0.223846895 |
| BRMS1 | -0.169827094 | 0.006532304 | 0.108189554 | Down | 2.184933582 |
| B4GAT1 | -0.208235791 | 0.091639793 | 0.303460748 | NS | 1.037915902 |
| SLC29A2 | 0.18153415 | 0.246545734 | 0.474686037 | NS | 0.608102507 |
| NPAS4 | 0.519846543 | 0.343021301 | NA | NS | 0.464678911 |
| MRPL11 | -0.137623118 | 0.250376622 | 0.477425471 | NS | 0.601406223 |
| PELI3 | 0.072491101 | 0.333748532 | 0.550289298 | NS | 0.476580636 |
| DPP3 | -0.143565832 | 0.06160767 | 0.258215532 | NS | 1.210365212 |
| BBS1 | 0.219257819 | 0.101370768 | 0.317114382 | NS | 0.994087265 |
| ZDHHC24 | 0.044849629 | 0.575359542 | 0.736492546 | NS | 0.24006068 |
| ACTN3 | 0.620107633 | 0.067935319 | 0.267927444 | NS | 1.167904383 |
| CTSF | -0.143322656 | 0.18406382 | 0.414354362 | NS | 0.735031569 |
| CCDC87 | 0.236827724 | 0.57807941 | NA | NS | 0.238012499 |
| CCS | -0.266354538 | 0.008475525 | 0.11782982 | Down | 2.071833367 |
| RBM14 | 0.109528119 | 0.148821607 | 0.374840808 | NS | 0.827334009 |
| RBM14-RBM4 | 0.165630634 | 0.033040119 | 0.200340163 | Up | 1.480958403 |
| RBM4 | 0.033075245 | 0.437942169 | 0.633431322 | NS | 0.358583235 |
| RBM4B | -0.102746441 | 0.00757094 | 0.113104804 | Down | 2.120850204 |
| SPTBN2 | 0.563371184 | 0.037339425 | 0.210115856 | Up | 1.427832368 |
| C11orf80 | 0.015621479 | 0.848420451 | 0.914722021 | NS | 0.071388872 |
| RCE1 | -0.04129072 | 0.555497454 | 0.721922646 | NS | 0.255317927 |
| PC | 0.055648273 | 0.683095446 | 0.811353013 | NS | 0.16551861 |
| LRFN4 | 0.327890427 | 0.019338491 | 0.161365225 | Up | 1.713577427 |
| C11orf86 | 0.515020694 | 0.34697594 | NA | NS | 0.459700639 |
| SYT12 | 0.752111168 | 0.0826834 | 0.289831661 | NS | 1.082581671 |
| RHOD | 1.330414155 | 0.003963617 | NA | Up | 2.40190834 |
| KDM2A | 0.007230814 | 0.924568713 | 0.958927405 | NS | 0.034060807 |
| GRK2 | -0.008689342 | 0.902342328 | 0.945604168 | NS | 0.04462867 |
| ANKRD13D | 9.24E-04 | 0.991989313 | 0.995510242 | NS | 0.003493007 |
| SSH3 | 0.072018771 | 0.529136167 | 0.70382202 | NS | 0.276432553 |
| RAD9A | 0.138517324 | 0.170994078 | 0.399372923 | NS | 0.76701893 |
| POLD4 | 0.00511676 | 0.931758892 | 0.962684504 | NS | 0.030696454 |
| CLCF1 | -0.185367934 | 0.044377509 | 0.225362854 | Down | 1.352837084 |
| PPP1CA | 0.007373944 | 0.895933132 | 0.941611702 | NS | 0.047724403 |
| TBC1D10C | -0.060743523 | 0.304364537 | 0.525598354 | NS | 0.516605951 |
| CARNS1 | 0.162850454 | 0.211807476 | 0.44142459 | NS | 0.674058715 |
| RPS6KB2 | -0.101604761 | 0.038003464 | 0.212212115 | Down | 1.420176814 |
| CORO1B | 0.026880453 | 0.642054967 | 0.783312532 | NS | 0.19242779 |
| GPR152 | 0.659130129 | 0.093775051 | NA | NS | 1.02791269 |
| CABP4 | 0.099985056 | 0.589713731 | 0.747377833 | NS | 0.22935876 |
| TMEM134 | 0.134690836 | 0.086197629 | 0.29504015 | NS | 1.064504678 |
| AIP | 0.143716999 | 0.049890697 | 0.23659193 | Up | 1.30198043 |
| PITPNM1 | 0.018814731 | 0.86326849 | 0.922858706 | NS | 0.063854111 |
| CDK2AP2 | 0.420305674 | 1.54E-05 | 0.004208028 | Up | 4.811515539 |
| CABP2 | 0.624036151 | 0.188973406 | NA | NS | 0.723599309 |
| GSTP1 | -0.249292063 | 0.025061752 | 0.179152549 | Down | 1.600988564 |
| NDUFV1 | -0.243261499 | 4.05E-04 | 0.034514738 | Down | 3.392116546 |
| NUDT8 | 0.291372622 | 0.08469983 | 0.292761741 | NS | 1.072117463 |
| TBX10 | 0.668496657 | 0.109185279 | NA | NS | 0.96183591 |
| ACY3 | 0.338812576 | 0.24992494 | NA | NS | 0.602190404 |
| ALDH3B2 | 0.721468306 | 0.121670637 | NA | NS | 0.914814217 |
| UNC93B1 | 0.050231229 | 0.619572283 | 0.767330709 | NS | 0.207908019 |
| ALDH3B1 | -0.10831282 | 0.259113907 | 0.485568555 | NS | 0.586509278 |
| NDUFS8 | 0.123546369 | 0.043316151 | 0.223141939 | Up | 1.363350141 |
| TCIRG1 | 0.020326316 | 0.863731239 | 0.92301679 | NS | 0.063621373 |
| CHKA | 0.056532336 | 0.481407425 | 0.668193506 | NS | 0.317487216 |
| KMT5B | -0.031608599 | 0.464626211 | 0.65494365 | NS | 0.332896294 |
| C11orf24 | 0.053824472 | 0.490176472 | 0.674640719 | NS | 0.309647538 |
| LRP5 | 0.363595374 | 0.077404514 | 0.281652078 | NS | 1.111233712 |
| PPP6R3 | -0.141868007 | 0.002365163 | 0.074605254 | Down | 2.626138885 |
| GAL | 1.132242553 | 0.044182095 | NA | Up | 1.354753696 |
| TESMIN | 0.171846601 | 0.070140851 | 0.270968399 | NS | 1.154028972 |
| CPT1A | 0.181119189 | 0.011759733 | 0.132876014 | Up | 1.929602556 |
| MRPL21 | -0.314870363 | 0.005434123 | 0.100445755 | Down | 2.264870517 |
| IGHMBP2 | 0.028822056 | 0.758999092 | 0.859313665 | NS | 0.119758744 |
| MRGPRD | 0.382392792 | 0.157804658 | NA | NS | 0.801880183 |
| MRGPRF | 0.766904455 | 0.105539752 | NA | NS | 0.976583931 |
| TPCN2 | 0.083605723 | 0.490703663 | 0.675001219 | NS | 0.3091807 |
| SMIM38 | 0.200482943 | 0.339906124 | 0.55558404 | NS | 0.468641011 |
| MYEOV | 0.396206042 | 0.106028581 | 0.322346872 | NS | 0.974577049 |
| CCND1 | 0.263715004 | 0.060927525 | 0.25683088 | NS | 1.215186465 |
| LTO1 | 0.017247971 | 0.71967178 | 0.83461227 | NS | 0.142865526 |
| FGF19 | 1.082163572 | 0.074549018 | NA | NS | 1.127558072 |
| FGF4 | 0.746994717 | 0.095688737 | NA | NS | 1.019139179 |
| FGF3 | 1.347746874 | 0.003643325 | NA | Up | 2.438502056 |
| ANO1 | 0.604258847 | 0.180509012 | 0.410283585 | NS | 0.74350111 |
| FADD | 0.237091352 | 0.00635703 | 0.107129293 | Up | 2.196745714 |
| PPFIA1 | 0.224158178 | 0.008710471 | 0.118782974 | Up | 2.059958385 |
| CTTN | -0.095991975 | 0.55440876 | 0.72131755 | NS | 0.256169916 |
| SHANK2 | 0.571350077 | 0.104577475 | 0.320548581 | NS | 0.98056185 |
| DHCR7 | 0.007554716 | 0.935819022 | 0.964419702 | NS | 0.028808131 |
| NADSYN1 | 0.013534481 | 0.767777052 | 0.864740339 | NS | 0.114764873 |
| KRTAP5-7 | 0.553405144 | 0.269811479 | NA | NS | 0.568939578 |
| KRTAP5-8 | 0.90659226 | 0.071016472 | NA | NS | 1.148640904 |
| KRTAP5-9 | 0.746316332 | 0.064500349 | NA | NS | 1.190437935 |
| KRTAP5-10 | 0.547582743 | 0.285371608 | NA | NS | 0.544589238 |
| KRTAP5-11 | 0.725659225 | 0.210756789 | NA | NS | 0.676218426 |
| ZNF705E | 9.00E-04 | 0.995406482 | 0.997330139 | NS | 0.001999535 |
| DEFB108B | 0.14290043 | 0.570489677 | NA | NS | 0.243752209 |
| DEFB131B | -0.026258307 | 0.865921621 | 0.924223465 | NS | 0.062521416 |
| RNF121 | -0.093128955 | 0.084011298 | 0.29196157 | NS | 1.075662305 |
| IL18BP | -0.062017369 | 0.602476385 | 0.756016283 | NS | 0.220059971 |
| NUMA1 | -0.130407725 | 0.106867806 | 0.323655976 | NS | 0.971153107 |
| LRTOMT | -0.162732826 | 0.482534576 | NA | NS | 0.316471562 |
| LRRC51 | 0.179993917 | 0.013413903 | 0.139069808 | Up | 1.872444839 |
| LAMTOR1 | -0.040315808 | 0.573950695 | 0.735426831 | NS | 0.241125414 |
| ANAPC15 | -0.135249525 | 0.223437231 | 0.452165721 | NS | 0.650844459 |
| FOLR3 | 0.789882044 | 7.57E-04 | 0.045387814 | Up | 3.12078329 |
| FOLR1 | 0.051390569 | 0.916881497 | NA | NS | 0.037686791 |
| FOLR2 | -0.402161776 | 0.005221088 | 0.098879942 | Down | 2.282238966 |
| INPPL1 | 0.010699712 | 0.901907184 | 0.9454453 | NS | 0.044838154 |
| PHOX2A | 0.719857017 | 0.012581652 | NA | Up | 1.900262345 |
| CLPB | -0.027567685 | 0.711134292 | 0.829246312 | NS | 0.148048378 |
| PDE2A | 0.300947666 | 0.193599092 | 0.422794379 | NS | 0.713096685 |
| ARAP1 | -0.021852736 | 0.871018727 | 0.927170388 | NS | 0.059972507 |
| STARD10 | 0.27335842 | 0.01633099 | 0.151094105 | Up | 1.786987483 |
| ATG16L2 | -0.070156337 | 0.566476928 | 0.730158408 | NS | 0.246817774 |
| FCHSD2 | 0.207809544 | 0.004844412 | 0.096559376 | Up | 2.314758932 |
| P2RY2 | 0.364726637 | 0.008992042 | 0.120055109 | Up | 2.046141685 |
| P2RY6 | 0.353883281 | 0.153095458 | 0.379704589 | NS | 0.815037695 |
| ARHGEF17 | 0.533275593 | 0.060392008 | 0.256244335 | NS | 1.219020532 |
| RELT | 0.079125565 | 0.367914851 | 0.579920676 | NS | 0.434252681 |
| FAM168A | 0.162426104 | 0.089632964 | 0.299979972 | NS | 1.047532242 |
| PLEKHB1 | 0.168935133 | 0.112948319 | 0.331633642 | NS | 0.947120227 |
| RAB6A | -0.200585917 | 0.044099661 | 0.224627854 | Down | 1.35556475 |
| MRPL48 | -0.166578882 | 0.166329791 | 0.394295091 | NS | 0.779029958 |
| COA4 | -0.170905043 | 0.027146111 | 0.185717716 | Down | 1.566292381 |
| PAAF1 | -0.033096214 | 0.561074599 | 0.725522583 | NS | 0.250979392 |
| DNAJB13 | 0.079150416 | 0.644744938 | 0.785297483 | NS | 0.190612059 |
| UCP2 | 0.161801922 | 0.346882208 | 0.561433348 | NS | 0.459817975 |
| UCP3 | 0.009293472 | 0.925560076 | 0.959235083 | NS | 0.033595387 |
| C2CD3 | 0.07174209 | 0.37299291 | 0.583688666 | NS | 0.428299423 |
| PPME1 | -0.024923644 | 0.752393998 | 0.854731503 | NS | 0.123554678 |
| P4HA3 | 0.444338783 | 0.039766682 | 0.215730166 | Up | 1.400480645 |
| PGM2L1 | -0.214427807 | 0.102767137 | 0.318649084 | NS | 0.988145741 |
| KCNE3 | -0.009403927 | 0.931752741 | 0.962684504 | NS | 0.030699321 |
| LIPT2 | -0.221305807 | 0.039081821 | 0.214419722 | Down | 1.408025204 |
| POLD3 | 0.036579251 | 0.438384665 | 0.633791149 | NS | 0.358144646 |
| CHRDL2 | 0.597310345 | 0.177192752 | 0.407027924 | NS | 0.751554047 |
| RNF169 | 0.044461952 | 0.397931469 | 0.603019023 | NS | 0.400191715 |
| XRRA1 | -0.028892292 | 0.716147494 | 0.832126328 | NS | 0.144997523 |
| SPCS2 | 0.036969531 | 0.521995946 | 0.697720973 | NS | 0.28233287 |
| NEU3 | 0.142049057 | 0.016509502 | 0.151664904 | Up | 1.782266037 |
| OR2AT4 | 0.239146514 | 0.586500915 | 0.745189678 | NS | 0.231731306 |
| SLCO2B1 | 0.36257741 | 0.402315047 | 0.606611312 | NS | 0.395433724 |
| TPBGL | 0.73566334 | 0.227537337 | NA | NS | 0.642947329 |
| ARRB1 | 0.115941802 | 0.121460618 | 0.342148251 | NS | 0.915564513 |
| RPS3 | -0.21680213 | 0.077296469 | 0.281620438 | NS | 1.111840342 |
| KLHL35 | 0.251764262 | 0.165249826 | 0.393073717 | NS | 0.781858988 |
| GDPD5 | -0.010369903 | 0.94601328 | 0.970854819 | NS | 0.024102767 |
| SERPINH1 | 0.02941231 | 0.69944949 | 0.822044571 | NS | 0.155243642 |
| MAP6 | 0.845519743 | 0.014934491 | NA | Up | 1.825809577 |
| MOGAT2 | 0.578362716 | 0.241643828 | NA | NS | 0.616824294 |
| DGAT2 | 0.052984763 | 0.718269408 | 0.833717613 | NS | 0.14371263 |
| UVRAG | 0.030963128 | 0.466450543 | 0.656384045 | NS | 0.331194397 |
| WNT11 | 0.58997894 | 0.024523308 | NA | Up | 1.610420949 |
| THAP12 | -0.204652225 | 0.017625159 | 0.155449116 | Down | 1.753866957 |
| GVQW3 | 0.073231649 | 0.419448766 | 0.619675889 | NS | 0.377321079 |
| EMSY | 0.032794756 | 0.645762156 | 0.786076586 | NS | 0.18992741 |
| LRRC32 | 0.637144692 | 0.015099014 | 0.147147925 | Up | 1.821051406 |
| TSKU | 0.845582975 | 0.071064654 | NA | NS | 1.148346354 |
| ACER3 | 0.103133164 | 0.055631148 | 0.248695897 | NS | 1.254681977 |
| B3GNT6 | 0.937916497 | 0.05384898 | NA | NS | 1.26882252 |
| CAPN5 | -0.151419606 | 0.464570848 | 0.654918093 | NS | 0.332948046 |
| OMP | 0.511468278 | 0.199494397 | NA | NS | 0.700069298 |
| MYO7A | 0.303072806 | 0.134584253 | 0.358067909 | NS | 0.871005752 |
| GDPD4 | 0.36312097 | 0.33956299 | NA | NS | 0.469079651 |
| PAK1 | 0.063675702 | 0.428111007 | 0.625957236 | NS | 0.368443606 |
| CLNS1A | -0.341205464 | 0.003867775 | 0.089594751 | Down | 2.412538833 |
| AQP11 | 0.231417798 | 0.014323773 | 0.143756774 | Up | 1.843942558 |
| RSF1 | -0.115845064 | 0.094142386 | 0.307260785 | NS | 1.026214798 |
| AAMDC | 0.110656822 | 0.165652041 | 0.393591001 | NS | 0.780803209 |
| INTS4 | 0.00155905 | 0.962078989 | 0.978920074 | NS | 0.01678927 |
| KCTD14 | 0.706414368 | 0.187660565 | NA | NS | 0.726626981 |
| THRSP | 0.295543554 | 0.076026405 | 0.279614057 | NS | 1.119035544 |
| NDUFC2 | -0.198360776 | 0.028313989 | 0.188228443 | Down | 1.547998935 |
| ALG8 | -0.081281783 | 0.115163878 | 0.333985455 | NS | 0.938683718 |
| KCTD21 | 0.140169527 | 0.140738165 | 0.365211163 | NS | 0.851588116 |
| USP35 | 0.12049516 | 0.197586198 | 0.427410856 | NS | 0.704243396 |
| GAB2 | 0.209301397 | 0.085753087 | 0.294352097 | NS | 1.066750238 |
| NARS2 | -0.107343573 | 0.101655078 | 0.317403374 | NS | 0.992870921 |
| TENM4 | 0.592580212 | 0.053695696 | 0.244821585 | NS | 1.270060526 |
| FAM181B | 0.547530775 | 0.210041427 | NA | NS | 0.67769504 |
| PRCP | -0.037290121 | 0.630552229 | 0.775101023 | NS | 0.200278934 |
| DDIAS | -0.110907817 | 0.283006663 | 0.505924531 | NS | 0.54820334 |
| RAB30 | 0.088232815 | 0.272746268 | 0.498012997 | NS | 0.564241183 |
| PCF11 | -0.053651077 | 0.071468138 | 0.272521361 | NS | 1.145887533 |
| ANKRD42 | -0.042534943 | 0.438575061 | 0.633826342 | NS | 0.357956068 |
| CCDC90B | -0.133092981 | 0.066581219 | 0.266115722 | NS | 1.176648256 |
| DLG2 | 0.222472016 | 0.33709233 | 0.553332034 | NS | 0.47225113 |
| TMEM126B | -0.100617099 | 0.37259687 | 0.583308054 | NS | 0.428760797 |
| TMEM126A | -0.167997862 | 0.094794517 | 0.308053683 | NS | 1.02321678 |
| CREBZF | -0.101505828 | 0.103385021 | 0.31928753 | NS | 0.985542381 |
| CCDC89 | -0.065864287 | 0.644070945 | 0.784726966 | NS | 0.191066292 |
| SYTL2 | -0.247773402 | 0.010175631 | 0.126233693 | Down | 1.992438646 |
| CCDC83 | 0.490321043 | 0.125167013 | 0.346791064 | NS | 0.902510111 |
| PICALM | -0.157080276 | 0.106085388 | 0.322394846 | NS | 0.974344431 |
| EED | -0.057112106 | 0.270253121 | 0.495760518 | NS | 0.568229282 |
| HIKESHI | -0.093452135 | 0.234856062 | 0.462840852 | NS | 0.629198225 |
| CCDC81 | 0.099301711 | 0.615660414 | 0.765059537 | NS | 0.21065877 |
| ME3 | 0.201018177 | 0.087920551 | 0.297765891 | NS | 1.055909599 |
| PRSS23 | -0.25841635 | 0.097254706 | 0.311597251 | NS | 1.012089374 |
| FZD4 | 0.384037645 | 0.075186557 | 0.278178116 | NS | 1.1238598 |
| TMEM135 | 0.06757808 | 0.199309079 | 0.428926322 | NS | 0.700472919 |
| RAB38 | -0.080486694 | 0.66200414 | NA | NS | 0.179139295 |
| CTSC | -0.081481309 | 0.242761293 | 0.470460443 | NS | 0.614820558 |
| GRM5 | 0.795356106 | 0.129533508 | NA | NS | 0.887617872 |
| TYR | 0.23722946 | 0.684728776 | NA | NS | 0.16448142 |
| NOX4 | 0.141319906 | 0.760599922 | 0.860168677 | NS | 0.118843723 |
| TRIM77 | 0.656808213 | 0.275944023 | NA | NS | 0.559179009 |
| TRIM49 | 0.101034603 | 0.896047492 | NA | NS | 0.047668971 |
| TRIM64B | -0.141693239 | 0.618176343 | NA | NS | 0.208887619 |
| TRIM49D1 | -0.72719774 | 0.242400223 | NA | NS | 0.615466985 |
| TRIM49C | -0.013030012 | 0.984497051 | NA | NS | 0.00678558 |
| UBTFL1 | -0.231069355 | 0.818724575 | NA | NS | 0.086862174 |
| NAALAD2 | -0.287120617 | 0.523694633 | NA | NS | 0.280921877 |
| CHORDC1 | -0.083997988 | 0.159534827 | 0.387098757 | NS | 0.797144495 |
| FAT3 | 0.550163827 | 0.166127756 | 0.394142532 | NS | 0.779557802 |
| MTNR1B | 1.042016564 | 0.025287729 | NA | Up | 1.597090175 |
| SLC36A4 | 0.001442539 | 0.978662656 | 0.987485532 | NS | 0.009366983 |
| DEUP1 | 0.411874343 | 0.064016194 | 0.262600549 | NS | 1.193710152 |
| SMCO4 | -0.020687727 | 0.85122706 | 0.916355684 | NS | 0.069954579 |
| CEP295 | -0.113518339 | 0.056011843 | 0.249087638 | NS | 1.251720135 |
| TAF1D | -0.086488663 | 0.235141329 | 0.463084248 | NS | 0.628671031 |
| C11orf54 | -0.039567004 | 0.512643878 | 0.691068933 | NS | 0.290184224 |
| MED17 | 0.047256273 | 0.245315063 | 0.473430992 | NS | 0.610275784 |
| VSTM5 | 0.334067249 | 0.010884716 | 0.12856202 | Up | 1.963182913 |
| HEPHL1 | 0.210354641 | 0.159556615 | 0.387098757 | NS | 0.797085185 |
| PANX1 | -0.25803907 | 0.004628095 | 0.094890448 | Down | 2.33459774 |
| IZUMO1R | 0.985260673 | 0.095034295 | NA | NS | 1.022119642 |
| GPR83 | 0.235909771 | 0.436042068 | NA | NS | 0.36047161 |
| MRE11 | -0.08672924 | 0.110724007 | 0.329196574 | NS | 0.955758205 |
| ANKRD49 | -0.168121247 | 0.074320401 | 0.276837611 | NS | 1.128891956 |
| C11orf97 | 0.110961603 | 0.833054739 | NA | NS | 0.079326461 |
| PIWIL4 | 0.043846052 | 0.600708446 | 0.754699919 | NS | 0.221336262 |
| FUT4 | 0.006405414 | 0.912041989 | 0.951225368 | NS | 0.039985167 |
| AMOTL1 | 0.254859408 | 0.024141445 | 0.176078782 | Up | 1.617236736 |
| CWC15 | -0.156297601 | 0.113823013 | 0.332423088 | NS | 0.943769923 |
| KDM4D | 0.186918028 | 0.36638618 | NA | NS | 0.436060917 |
| KDM4E | 0.843093387 | 0.002312025 | NA | Up | 2.636007484 |
| KDM4F | 0.454442094 | 0.488016486 | NA | NS | 0.311565507 |
| SRSF8 | -0.219877167 | 0.032244941 | 0.19828377 | Down | 1.491538414 |
| ENDOD1 | -0.08820609 | 0.390969844 | 0.597849461 | NS | 0.407856739 |
| SESN3 | -0.116546479 | 0.413891527 | 0.615765954 | NS | 0.383113464 |
| FAM76B | -0.11283328 | 0.093026416 | 0.305431813 | NS | 1.03139371 |
| CEP57 | -0.157974077 | 0.038666367 | 0.213650843 | Down | 1.412666632 |
| MTMR2 | -0.001425948 | 0.974493414 | 0.985403414 | NS | 0.011221091 |
| MAML2 | 0.067420397 | 0.281599897 | 0.504671794 | NS | 0.550367508 |
| CCDC82 | -0.046072181 | 0.443040577 | 0.637383962 | NS | 0.353556496 |
| JRKL | -0.057845485 | 0.491112864 | 0.675418444 | NS | 0.30881869 |
| CNTN5 | 0.371867604 | 0.418411117 | 0.618901466 | NS | 0.378396785 |
| ARHGAP42 | -0.073204443 | 0.603792147 | 0.756851459 | NS | 0.21911254 |
| PGR | 0.394421582 | 0.366538663 | 0.578849018 | NS | 0.435880208 |
| TRPC6 | 0.226718358 | 0.222716074 | 0.451424332 | NS | 0.652248438 |
| ANGPTL5 | 0.331159284 | 0.247097338 | NA | NS | 0.607131933 |
| CEP126 | -0.021889042 | 0.834832028 | 0.906773683 | NS | 0.078400898 |
| CFAP300 | 0.683937283 | 0.153974278 | NA | NS | 0.812551824 |
| YAP1 | 0.187388596 | 0.589703809 | 0.747377833 | NS | 0.229366067 |
| BIRC3 | -0.131937527 | 0.184622068 | 0.414734144 | NS | 0.733716389 |
| BIRC2 | -0.165660786 | 0.200502607 | 0.430188957 | NS | 0.697879976 |
| TMEM123 | -0.130769508 | 0.249112896 | 0.476376043 | NS | 0.60360379 |
| MMP7 | 0.720418675 | 0.163907597 | NA | NS | 0.785400916 |
| MMP20 | -0.092723334 | 0.858653836 | NA | NS | 0.066181886 |
| MMP27 | 0.353321631 | 0.449233976 | NA | NS | 0.347527405 |
| MMP8 | 0.61006596 | 0.010975945 | 0.129200737 | Up | 1.959558061 |
| MMP10 | 0.623871759 | 0.22465796 | NA | NS | 0.64847819 |
| MMP1 | -0.012426162 | 0.969136249 | 0.982782429 | NS | 0.013615162 |
| MMP3 | 0.801193852 | 0.165876659 | NA | NS | 0.780214721 |
| MMP12 | 0.66508447 | 0.246643651 | NA | NS | 0.60793006 |
| MMP13 | 0.325986372 | 0.490564195 | NA | NS | 0.309304153 |
| DCUN1D5 | -0.001288725 | 0.981856352 | 0.989329675 | NS | 0.007952046 |
| DYNC2H1 | 0.029648676 | 0.72725549 | 0.838735282 | NS | 0.138312992 |
| PDGFD | -0.233708296 | 0.05153066 | 0.239738115 | NS | 1.287934295 |
| DDI1 | 0.155433744 | 0.36714946 | 0.579396578 | NS | 0.435157106 |
| CASP4 | 0.038581631 | 0.676675399 | 0.806968386 | NS | 0.169619613 |
| CASP5 | 0.273430851 | 0.094009331 | 0.30725936 | NS | 1.026829038 |
| CASP1 | -0.062623301 | 0.496595843 | 0.679155378 | NS | 0.303996921 |
| CARD16 | -0.009014519 | 0.934922641 | 0.96397808 | NS | 0.029224323 |
| CARD18 | 0.315590251 | 0.553056218 | NA | NS | 0.257230721 |
| GRIA4 | 0.485973623 | 0.109058007 | 0.327088329 | NS | 0.962342443 |
| MSANTD4 | -0.130404687 | 0.047511398 | 0.23161048 | Down | 1.323202191 |
| KBTBD3 | -0.112031173 | 0.15651551 | 0.383636723 | NS | 0.805442621 |
| AASDHPPT | -0.152607938 | 0.035518396 | 0.206049964 | Down | 1.449546651 |
| GUCY1A2 | 0.560470071 | 0.139044165 | 0.363272092 | NS | 0.856847231 |
| CWF19L2 | -0.139876148 | 0.012720805 | 0.135824642 | Down | 1.895485403 |
| ALKBH8 | -0.128647104 | 0.03741387 | 0.210115856 | Down | 1.426967363 |
| ELMOD1 | 0.562664654 | 0.200140972 | 0.429822631 | NS | 0.698663996 |
| SLN | 1.095812278 | 0.03805058 | NA | Up | 1.419638719 |
| SLC35F2 | -0.22162265 | 0.016518912 | 0.151690507 | Down | 1.78201856 |
| RAB39A | -0.120730436 | 0.260361323 | 0.486520481 | NS | 0.584423531 |
| CUL5 | -0.144499629 | 0.028610199 | 0.188990708 | Down | 1.543479116 |
| ACAT1 | -0.061494107 | 0.425048966 | 0.623662126 | NS | 0.371561036 |
| NPAT | -0.119840949 | 0.05480934 | 0.247094287 | NS | 1.261145427 |
| ATM | -0.06105323 | 0.22317285 | 0.451870269 | NS | 0.65135864 |
| C11orf65 | 0.127448233 | 0.137061765 | 0.361583064 | NS | 0.863083678 |
| POGLUT3 | 0.065218735 | 0.372322063 | 0.583077126 | NS | 0.429081227 |
| EXPH5 | 0.082530587 | 0.486448656 | 0.671762851 | NS | 0.312962992 |
| DDX10 | -0.041957167 | 0.408600356 | 0.611677799 | NS | 0.38870126 |
| C11orf87 | 0.674894271 | 0.173661067 | 0.402793777 | NS | 0.760297535 |
| RDX | -0.17173448 | 0.015320669 | 0.147421994 | Down | 1.814722276 |
| ZC3H12C | -0.065096501 | 0.535143516 | 0.707286288 | NS | 0.271529733 |
| FDX1 | -0.213186693 | 0.028468825 | 0.188709674 | Down | 1.545630464 |
| ARHGAP20 | 0.209159952 | 0.394963421 | 0.600587761 | NS | 0.403443124 |
| C11orf53 | 0.578880104 | 0.247438889 | NA | NS | 0.606532043 |
| COLCA2 | 0.174946425 | 0.487880991 | 0.672694188 | NS | 0.311686102 |
| POU2AF1 | 0.215659467 | 0.090592339 | 0.301422749 | NS | 1.042908529 |
| BTG4 | 0.563853895 | 0.257275702 | NA | NS | 0.589601229 |
| HOATZ | 0.596152573 | 0.24484744 | NA | NS | 0.611104433 |
| LAYN | 0.259495827 | 0.281912829 | 0.504917658 | NS | 0.54988516 |
| SIK2 | 0.047863509 | 0.488007953 | 0.672788234 | NS | 0.3115731 |
| PPP2R1B | -0.088744272 | 0.04096079 | 0.218105862 | NS | 1.387631677 |
| ALG9 | 0.069602088 | 0.267444057 | 0.493434609 | NS | 0.572767048 |
| FDXACB1 | -0.122447207 | 0.202520937 | 0.432624874 | NS | 0.693530073 |
| C11orf1 | -0.123022242 | 0.377902692 | 0.587917088 | NS | 0.422620014 |
| CRYAB | 0.622605409 | 0.122865133 | NA | NS | 0.910571344 |
| C11orf52 | 0.784361347 | 0.36356649 | NA | NS | 0.439416153 |
| DIXDC1 | 0.126915599 | 0.188907804 | 0.418330318 | NS | 0.723750101 |
| DLAT | -0.066987193 | 0.259943684 | 0.485926477 | NS | 0.585120731 |
| PIH1D2 | 0.520538894 | 0.048466235 | NA | Up | 1.314560714 |
| NKAPD1 | -0.122435601 | 0.075527354 | 0.278808425 | NS | 1.121895728 |
| TIMM8B | -0.297968345 | 0.048804863 | 0.234117506 | Down | 1.311536898 |
| SDHD | -0.219173018 | 0.036437632 | 0.208452907 | Down | 1.438449852 |
| IL18 | -0.106446657 | 0.310353622 | 0.530362297 | NS | 0.508143181 |
| TEX12 | 0.277063086 | 0.142585776 | NA | NS | 0.845923795 |
| BCO2 | 0.265119542 | 0.057828205 | 0.251831441 | NS | 1.237860289 |
| PTS | -0.028377884 | 0.617149201 | 0.765943262 | NS | 0.209609829 |
| PLET1 | 0.19097972 | 0.240122797 | 0.468184257 | NS | 0.619566607 |
| NCAM1 | -0.154022662 | 0.215455708 | 0.444767477 | NS | 0.666641996 |
| TTC12 | 0.090068181 | 0.21207896 | 0.441812042 | NS | 0.673502414 |
| ANKK1 | 0.526731671 | 0.021334315 | 0.168379028 | Up | 1.6709213 |
| DRD2 | 0.356142892 | 0.432186076 | NA | NS | 0.364329229 |
| TMPRSS5 | 0.111155595 | 0.571345607 | 0.733338401 | NS | 0.243101107 |
| ZW10 | -0.201523189 | 0.001408294 | 0.060860925 | Down | 2.851306681 |
| USP28 | -0.151520909 | 0.045155281 | 0.227234948 | Down | 1.34529145 |
| HTR3B | 0.951081669 | 0.002799834 | 0.079460125 | Up | 2.552867752 |
| HTR3A | 0.196027069 | 0.497304688 | NA | NS | 0.303377446 |
| ZBTB16 | 0.167650148 | 0.224294349 | 0.452740101 | NS | 0.649181668 |
| NNMT | 0.518411835 | 0.254124039 | NA | NS | 0.594954251 |
| C11orf71 | -0.139276076 | 0.086457052 | 0.295484168 | NS | 1.063199576 |
| RBM7 | 0.01200865 | 0.856567792 | 0.919265023 | NS | 0.067238259 |
| REXO2 | -0.188598399 | 0.058834736 | 0.253109387 | NS | 1.230366189 |
| NXPE1 | 0.150508372 | 0.547649751 | 0.7160589 | NS | 0.261497106 |
| NXPE4 | 0.134993636 | 0.664944588 | NA | NS | 0.177214544 |
| NXPE2 | 0.36372848 | 0.464170183 | NA | NS | 0.333322761 |
| CADM1 | 0.154628601 | 0.358980258 | 0.571841543 | NS | 0.444929435 |
| BUD13 | -0.068104059 | 0.086645953 | 0.295984568 | NS | 1.062251717 |
| ZPR1 | -0.16078091 | 0.00543963 | 0.100466463 | Down | 2.264430613 |
| APOA5 | 1.065318205 | 0.013813697 | NA | Up | 1.859690087 |
| APOA4 | 1.124174774 | 0.097060071 | NA | NS | 1.012959394 |
| APOC3 | 1.227873443 | 0.034653427 | NA | Up | 1.460253816 |
| APOA1 | 0.734577762 | 0.033559809 | NA | Up | 1.474180516 |
| SIK3 | 0.040046168 | 0.706489721 | 0.826397037 | NS | 0.150894152 |
| PAFAH1B2 | -0.018050386 | 0.683282528 | 0.811450496 | NS | 0.165399685 |
| SIDT2 | 0.053915769 | 0.679735254 | 0.809193979 | NS | 0.167660205 |
| TAGLN | 0.07127184 | 0.62467269 | 0.771101922 | NS | 0.20434748 |
| PCSK7 | -0.097819176 | 0.17013667 | 0.398485991 | NS | 0.769202072 |
| RNF214 | -0.032203141 | 0.808373302 | 0.889808967 | NS | 0.092388038 |
| BACE1 | 0.09585101 | 0.400753318 | 0.605572215 | NS | 0.397122873 |
| CEP164 | -0.036922101 | 0.626484733 | 0.772173369 | NS | 0.203089508 |
| DSCAML1 | 0.703121775 | 0.025447615 | 0.180433831 | Up | 1.594352918 |
| FXYD2 | 0.389331359 | 0.050307729 | 0.237303925 | NS | 1.298365288 |
| FXYD6 | 0.068447925 | 0.718788711 | 0.834077883 | NS | 0.143398752 |
| TMPRSS13 | 0.212492261 | 0.15593371 | 0.383185333 | NS | 0.807059989 |
| IL10RA | -0.118668253 | 0.018109371 | 0.156751663 | Down | 1.742096626 |
| SMIM35 | -0.060704023 | 0.507240699 | 0.687180693 | NS | 0.294785907 |
| TMPRSS4 | 0.515188776 | 0.194722142 | 0.424231973 | NS | 0.710584661 |
| SCN4B | 0.53409371 | 0.135474213 | 0.359475198 | NS | 0.868143362 |
| SCN2B | 0.539924996 | 0.168764843 | 0.396950027 | NS | 0.77271802 |
| JAML | -0.009581259 | 0.91137412 | 0.950858279 | NS | 0.040303308 |
| MPZL3 | 0.034852575 | 0.768066682 | 0.864804079 | NS | 0.114601074 |
| MPZL2 | -0.132721042 | 0.247543425 | 0.475766995 | NS | 0.606348605 |
| CD3E | 0.201022435 | 0.090094591 | 0.300691681 | NS | 1.045301283 |
| CD3D | -0.200752553 | 0.127440797 | 0.348830209 | NS | 0.89469152 |
| CD3G | 0.002489176 | 0.97432127 | 0.985403414 | NS | 0.011297816 |
| UBE4A | -0.014174666 | 0.726732874 | 0.838611255 | NS | 0.138625194 |
| ATP5MG | -0.213987905 | 0.061155866 | 0.257178045 | NS | 1.213561883 |
| KMT2A | 0.179772664 | 0.058822015 | 0.253109387 | NS | 1.230460105 |
| TTC36 | 0.229173225 | 0.17264572 | NA | NS | 0.762844185 |
| TMEM25 | 0.051249484 | 0.635434081 | 0.777679224 | NS | 0.196929496 |
| IFT46 | -0.136927861 | 0.040883416 | 0.218105862 | Down | 1.388452827 |
| ARCN1 | -0.058557788 | 0.212714876 | 0.442550518 | NS | 0.672202136 |
| PHLDB1 | 0.514626349 | 0.141725301 | 0.366135684 | NS | 0.848552613 |
| TREH | 0.887453841 | 0.077275289 | NA | NS | 1.111959364 |
| DDX6 | -0.172449348 | 0.007781334 | 0.114529629 | Down | 2.108945965 |
| CXCR5 | -0.09121685 | 0.502047418 | 0.683340661 | NS | 0.299255263 |
| BCL9L | -0.092227304 | 0.589857683 | 0.747465395 | NS | 0.229252759 |
| UPK2 | 0.308963988 | 0.212422973 | 0.442224427 | NS | 0.672798518 |
| FOXR1 | 0.743999618 | 0.347951007 | NA | NS | 0.458481902 |
| CENATAC | -0.019710644 | 0.79955376 | 0.884650476 | NS | 0.09715233 |
| RPS25 | -0.423948426 | 0.012180743 | 0.134186405 | Down | 1.914326237 |
| TRAPPC4 | -0.297079344 | 8.17E-04 | 0.047750337 | Down | 3.087610903 |
| SLC37A4 | -0.40999493 | 0.002362333 | 0.074605254 | Down | 2.626658799 |
| HYOU1 | -0.143962275 | 0.114604291 | 0.333333437 | NS | 0.94079912 |
| VPS11 | -0.151530441 | 0.037483859 | 0.210180429 | Down | 1.426155703 |
| HMBS | 0.17781518 | 0.283141162 | 0.506047986 | NS | 0.54799699 |
| H2AX | 0.330061395 | 0.004033401 | 0.090296145 | Up | 2.394328565 |
| DPAGT1 | -0.075410339 | 0.326936935 | 0.545101171 | NS | 0.485536014 |
| C2CD2L | 0.038427089 | 0.760101823 | 0.860025919 | NS | 0.119128226 |
| HINFP | 0.057478741 | 0.30536606 | 0.526578834 | NS | 0.515179235 |
| ABCG4 | 0.634691479 | 0.163741496 | 0.391482175 | NS | 0.785841245 |
| NLRX1 | 0.03686831 | 0.700068296 | 0.822245454 | NS | 0.15485959 |
| PDZD3 | 0.274076637 | 0.31822294 | 0.537481826 | NS | 0.497268517 |
| CCDC153 | 0.0770908 | 0.588842771 | 0.746880657 | NS | 0.230000652 |
| CBL | 0.229995305 | 0.031387012 | 0.196726942 | Up | 1.50325003 |
| MCAM | 0.133495413 | 0.383424499 | 0.592439708 | NS | 0.416320142 |
| RNF26 | 0.083522868 | 0.505928367 | 0.68625749 | NS | 0.295910969 |
| MFRP | 0.729433317 | 0.175841147 | NA | NS | 0.754879493 |
| USP2 | 0.448886423 | 0.39800555 | NA | NS | 0.400110872 |
| THY1 | 0.787796792 | 0.097482168 | 0.311937489 | NS | 1.011074822 |
| NECTIN1 | 0.1565365 | 0.393043305 | 0.599318562 | NS | 0.405559597 |
| TRIM29 | 0.875268448 | 0.044010999 | 0.224627854 | Up | 1.356438776 |
| OAF | 0.22617051 | 0.062793958 | 0.260716712 | NS | 1.202082143 |
| POU2F3 | 0.666313129 | 0.109099547 | 0.327136142 | NS | 0.962177051 |
| TLCD5 | 0.590628869 | 0.066844817 | NA | NS | 1.174932261 |
| ARHGEF12 | -0.207019101 | 0.240210812 | 0.468217208 | NS | 0.619407448 |
| GRIK4 | 0.155944614 | 0.568449596 | 0.731396269 | NS | 0.245308038 |
| TBCEL | -0.40225296 | 0.003695543 | 0.08761422 | Down | 2.432321686 |
| TBCEL-TECTA | 0.113323899 | 0.578254727 | 0.738275713 | NS | 0.237880809 |
| TECTA | -0.016115981 | 0.933183295 | 0.963088299 | NS | 0.030033044 |
| SC5D | 0.040523048 | 0.525517023 | 0.700709762 | NS | 0.279413211 |
| SORL1 | 0.009459712 | 0.932507108 | 0.962852453 | NS | 0.030347849 |
| BLID | 0.471605103 | 0.50703728 | NA | NS | 0.294960108 |
| UBASH3B | 0.203193285 | 1.94E-04 | 0.023041407 | Up | 3.711212598 |
| CRTAM | -0.139013601 | 0.203601148 | 0.433522809 | NS | 0.691219778 |
| JHY | 0.178134519 | 0.042181639 | 0.220751111 | Up | 1.374876546 |
| BSX | 1.160001999 | 0.031438539 | NA | Up | 1.50253764 |
| HSPA8 | -0.230904916 | 0.025756596 | 0.181900192 | Down | 1.589111542 |
| CLMP | 0.25321967 | 0.006803191 | 0.109684865 | Up | 2.167287329 |
| GRAMD1B | 0.316566947 | 0.002367217 | 0.074605254 | Up | 2.625761883 |
| SCN3B | 0.384929564 | 0.158993713 | 0.386629221 | NS | 0.798620049 |
| ZNF202 | -0.070553158 | 0.185777971 | 0.415463304 | NS | 0.731005785 |
| OR6X1 | -0.061384157 | 0.923217756 | NA | NS | 0.034695852 |
| OR6M1 | 0.729765857 | 0.350169447 | NA | NS | 0.45572175 |
| TMEM225 | 0.522020296 | 0.29141111 | NA | NS | 0.535493895 |
| OR8D4 | 0.56684657 | 0.264297506 | NA | NS | 0.577906935 |
| OR4D5 | 0.755426814 | 0.270671103 | NA | NS | 0.567558107 |
| OR6T1 | 0.297253485 | 0.690019705 | NA | NS | 0.161138507 |
| OR10S1 | 1.040284946 | 0.094207317 | NA | NS | 1.025915367 |
| OR10G6 | 0.057687184 | 0.925482318 | NA | NS | 0.033631874 |
| OR10G4 | 0.998057408 | 0.132279912 | NA | NS | 0.878506104 |
| OR10G8 | 0.389955486 | 0.509281925 | NA | NS | 0.293041737 |
| OR10G7 | 0.077964591 | 0.896240423 | NA | NS | 0.047575472 |
| VWA5A | -0.050383601 | 0.488478358 | 0.67310726 | NS | 0.311154673 |
| OR10D3 | 0.439968884 | 0.343442719 | NA | NS | 0.464145686 |
| OR8G1 | 0.494858956 | 0.331946917 | NA | NS | 0.47893136 |
| OR8G5 | 0.501190948 | 0.349613313 | NA | NS | 0.456412038 |
| OR8D1 | 0.507172571 | 0.266035341 | 0.49198493 | NS | 0.575060667 |
| OR8D2 | 0.611464262 | 0.348582142 | NA | NS | 0.457694866 |
| OR8B2 | 1.081106684 | 0.065801812 | NA | NS | 1.181762144 |
| OR8B3 | 0.834755243 | 0.097843895 | NA | NS | 1.009466268 |
| OR8B4 | -0.191086342 | 0.759834555 | NA | NS | 0.11928096 |
| OR8B8 | 0.633723315 | 0.160934125 | NA | NS | 0.793351857 |
| OR8B12 | 0.37676138 | 0.479282872 | NA | NS | 0.319408091 |
| OR8A1 | 0.715827189 | 0.124925841 | 0.346400199 | NS | 0.903347719 |
| PANX3 | 0.54744094 | 0.208847954 | NA | NS | 0.680169774 |
| TBRG1 | 0.068646632 | 0.034555941 | 0.204074304 | NS | 1.461477281 |
| SIAE | 0.2708291 | 0.003647633 | 0.08688637 | Up | 2.437988868 |
| SPA17 | 0.285880912 | 0.12275997 | 0.343571896 | NS | 0.910943227 |
| NRGN | 0.344634043 | 0.212608564 | 0.442496084 | NS | 0.672419246 |
| VSIG2 | 0.377047318 | 0.043272181 | 0.223075426 | Up | 1.363791218 |
| ESAM | 0.118026274 | 0.410183765 | 0.613073051 | NS | 0.387021533 |
| MSANTD2 | 0.041487362 | 0.464477573 | 0.654918093 | NS | 0.333035251 |
| ROBO3 | 0.395820229 | 0.050491573 | 0.23783587 | NS | 1.296781101 |
| ROBO4 | 0.428773294 | 0.252292606 | 0.47918438 | NS | 0.598095478 |
| HEPACAM | 0.715757611 | 0.045382507 | NA | Up | 1.343111517 |
| CCDC15 | -0.126685078 | 0.326031655 | 0.54446383 | NS | 0.486740232 |
| SLC37A2 | -0.190171707 | 0.073131212 | 0.275106935 | NS | 1.135897228 |
| TMEM218 | -0.026409113 | 0.561255266 | 0.725674256 | NS | 0.250839571 |
| PKNOX2 | 0.33322136 | 0.442084919 | 0.636699969 | NS | 0.3544943 |
| FEZ1 | -0.090181747 | 0.414680641 | 0.616155258 | NS | 0.382286239 |
| EI24 | -0.05352425 | 0.294222333 | 0.516303721 | NS | 0.531324366 |
| STT3A | -0.066832819 | 0.157726748 | 0.385231054 | NS | 0.802094652 |
| CHEK1 | 1.19E-04 | 0.99921569 | 0.999609713 | NS | 3.41E-04 |
| ACRV1 | 0.465576166 | 0.219439406 | NA | NS | 0.658685381 |
| PATE1 | 0.535944329 | 0.208377011 | NA | NS | 0.681150197 |
| PATE2 | 0.704453008 | 0.115165494 | NA | NS | 0.938677627 |
| PATE3 | 0.597277668 | 0.259505469 | NA | NS | 0.585853485 |
| PATE4 | 0.67900229 | 0.041842785 | 0.220492464 | Up | 1.378379418 |
| HYLS1 | -0.123219834 | 0.164331541 | 0.392074272 | NS | 0.784279073 |
| PUS3 | -0.156010363 | 0.073430578 | 0.2756445 | NS | 1.134123054 |
| DDX25 | 0.543866277 | 0.171280233 | 0.399855569 | NS | 0.766292754 |
| VSIG10L2 | 1.054635866 | 0.04011341 | NA | Up | 1.396710413 |
| CDON | 0.290307188 | 0.160190592 | 0.387974296 | NS | 0.795362994 |
| RPUSD4 | -0.098043999 | 0.104350862 | 0.320354348 | NS | 0.98150396 |
| FAM118B | -0.122576182 | 0.02064136 | 0.166747237 | Down | 1.685261701 |
| SRPRA | 0.084873174 | 0.087668553 | 0.297286622 | NS | 1.057156163 |
| FOXRED1 | 0.055469626 | 0.409519682 | 0.612634382 | NS | 0.387725221 |
| TIRAP | 0.286561093 | 0.007552382 | 0.113104804 | Up | 2.121916045 |
| DCPS | -0.026058434 | 0.720181731 | 0.834823202 | NS | 0.1425579 |
| ST3GAL4 | 0.078004274 | 0.475111263 | 0.663192426 | NS | 0.323204674 |
| KIRREL3 | 0.278698343 | 0.122163314 | 0.342932892 | NS | 0.913059193 |
| ETS1 | -0.066307653 | 0.355726302 | 0.568903557 | NS | 0.448884023 |
| FLI1 | -0.039832333 | 0.435850746 | 0.631722915 | NS | 0.360662206 |
| KCNJ1 | 0.073860017 | 0.61037653 | 0.76133344 | NS | 0.214402174 |
| KCNJ5 | 0.6730024 | 0.157779149 | 0.385271144 | NS | 0.801950392 |
| C11orf45 | 0.127799201 | 0.477403268 | 0.665215967 | NS | 0.321114613 |
| TP53AIP1 | 0.779342751 | 0.11562339 | NA | NS | 0.936954303 |
| ARHGAP32 | 0.087951895 | 0.333479114 | 0.550147567 | NS | 0.476931362 |
| BARX2 | 0.541569653 | 0.208628748 | NA | NS | 0.680625848 |
| TMEM45B | 0.143158636 | 0.236064152 | 0.463904342 | NS | 0.626969959 |
| NFRKB | 0.027822772 | 0.665354317 | 0.799556331 | NS | 0.176947021 |
| PRDM10 | 0.043261249 | 0.343847576 | 0.558852969 | NS | 0.463634033 |
| APLP2 | 0.075342772 | 0.320814756 | 0.539490663 | NS | 0.493745664 |
| ST14 | 0.092265114 | 0.430782286 | 0.627828924 | NS | 0.365742164 |
| ZBTB44 | -0.075024732 | 0.152164917 | 0.378791406 | NS | 0.817685466 |
| ADAMTS8 | 0.731881305 | 0.010138583 | NA | Up | 1.994022752 |
| ADAMTS15 | 0.893521064 | 0.050498443 | NA | NS | 1.296722009 |
| SNX19 | 0.006966691 | 0.885928601 | 0.93582108 | NS | 0.052601277 |
| NTM | 0.716092085 | 0.103192062 | 0.319144166 | NS | 0.986353711 |
| OPCML | 0.780350568 | 0.048178021 | 0.233024927 | Up | 1.317151047 |
| SPATA19 | 1.09013928 | 0.018385321 | NA | Up | 1.735528794 |
| IGSF9B | 0.159771072 | 0.279475206 | 0.503278325 | NS | 0.553656715 |
| JAM3 | -0.067413106 | 0.633590495 | 0.776709642 | NS | 0.198191346 |
| NCAPD3 | -0.051475718 | 0.240323696 | 0.468217208 | NS | 0.619203406 |
| VPS26B | 0.005460043 | 0.905965252 | 0.947892375 | NS | 0.042888459 |
| THYN1 | -0.156936205 | 0.028925853 | 0.189870991 | Down | 1.538713824 |
| ACAD8 | -0.083893293 | 0.039198606 | 0.214561777 | NS | 1.406729377 |
| GLB1L3 | 0.253263974 | 0.138418518 | 0.363014451 | NS | 0.858805806 |
| GLB1L2 | 0.063331021 | 0.692740073 | 0.817579653 | NS | 0.159429689 |
| B3GAT1 | 0.308927947 | 0.033754985 | 0.20229026 | Up | 1.471662078 |
| IQSEC3 | 0.501252948 | 0.161538211 | NA | NS | 0.791724731 |
| SLC6A12 | 0.219707576 | 0.071019955 | 0.272126319 | NS | 1.148619607 |
| SLC6A13 | 0.287343122 | 0.268091985 | 0.493891886 | NS | 0.57171617 |
| KDM5A | -0.015407504 | 0.750666186 | 0.853671923 | NS | 0.124553147 |
| CCDC77 | -0.191638065 | 0.028501736 | 0.188709674 | Down | 1.545128685 |
| B4GALNT3 | -0.211350378 | 0.355359891 | 0.568576726 | NS | 0.449331592 |
| NINJ2 | -0.350278803 | 0.002412295 | 0.075370241 | Down | 2.617569533 |
| WNK1 | -0.098194221 | 0.602368283 | 0.756016283 | NS | 0.220137903 |
| RAD52 | 0.111183612 | 0.108722992 | 0.326704032 | NS | 0.963678606 |
| ERC1 | 0.076675379 | 0.296341758 | 0.518054753 | NS | 0.528207147 |
| WNT5B | 0.13348368 | 0.458990651 | 0.650442663 | NS | 0.338196161 |
| FBXL14 | -0.077032401 | 0.274946985 | 0.499616959 | NS | 0.560751038 |
| ADIPOR2 | 0.123060666 | 0.200318043 | 0.429960996 | NS | 0.698279931 |
| CACNA2D4 | -0.03888866 | 0.743792562 | 0.849882793 | NS | 0.128548169 |
| DCP1B | 0.046729546 | 0.661855948 | 0.797234782 | NS | 0.179236524 |
| FKBP4 | 0.001872669 | 0.976482073 | 0.986397233 | NS | 0.010335725 |
| ITFG2 | -0.066290794 | 0.359323417 | 0.572255251 | NS | 0.444514479 |
| NRIP2 | 0.00881301 | 0.949461282 | 0.97264995 | NS | 0.022522741 |
| TEX52 | 0.115020433 | 0.6082363 | 0.759851448 | NS | 0.215927664 |
| FOXM1 | 0.118542293 | 0.469440165 | 0.658565871 | NS | 0.328419755 |
| RHNO1 | -0.16489827 | 0.036620528 | 0.20873154 | Down | 1.4362754 |
| TULP3 | -0.018391619 | 0.831975212 | 0.904571606 | NS | 0.079889613 |
| TEAD4 | 1.262333294 | 0.006261114 | NA | Up | 2.203348385 |
| TSPAN9 | 0.370734884 | 0.025095347 | 0.179152549 | Up | 1.600406794 |
| PRMT8 | 0.525831856 | 0.264244339 | 0.49043207 | NS | 0.577994307 |
| CRACR2A | 0.00517771 | 0.945469616 | 0.970383846 | NS | 0.024352423 |
| PARP11 | 0.063746149 | 0.207635971 | 0.437401068 | NS | 0.682697407 |
| CCND2 | 0.088843925 | 0.156139671 | 0.383229102 | NS | 0.806486739 |
| TIGAR | 0.091152731 | 0.204698033 | 0.434878882 | NS | 0.688886331 |
| FGF23 | 0.700066575 | 0.043221257 | 0.223040386 | Up | 1.364302605 |
| FGF6 | 0.474890075 | 0.408721712 | NA | NS | 0.388572291 |
| C12orf4 | -0.0561538 | 0.368722614 | 0.580416888 | NS | 0.433300226 |
| RAD51AP1 | -0.027439828 | 0.849955521 | 0.91573041 | NS | 0.070603801 |
| DYRK4 | -0.129335345 | 0.072019734 | 0.273667821 | NS | 1.142548484 |
| AKAP3 | 0.118692201 | 0.343148324 | 0.55833676 | NS | 0.464518118 |
| NDUFA9 | 0.02958764 | 0.546294715 | 0.714753418 | NS | 0.262573001 |
| GALNT8 | 0.161824155 | 0.395983145 | 0.601339831 | NS | 0.402323299 |
| KCNA6 | 0.15156533 | 0.286572287 | 0.509714081 | NS | 0.542765811 |
| KCNA1 | 0.436943646 | 0.313722745 | 0.533319352 | NS | 0.503453994 |
| KCNA5 | 0.118479248 | 0.695395429 | NA | NS | 0.157768168 |
| NTF3 | 0.988024675 | 0.06176581 | NA | NS | 1.209251861 |
| ANO2 | 0.304802186 | 0.328717631 | 0.546399419 | NS | 0.483177001 |
| VWF | 0.285189273 | 0.138752982 | 0.3632096 | NS | 0.857757675 |
| CD9 | 0.108959432 | 0.355694819 | 0.568903047 | NS | 0.448922461 |
| PLEKHG6 | 0.151421256 | 0.203647594 | 0.433564246 | NS | 0.691120716 |
| TNFRSF1A | 0.286279249 | 0.010846603 | 0.12856202 | Up | 1.964706238 |
| SCNN1A | 0.567038067 | 0.004728728 | 0.095559907 | Up | 2.325255626 |
| LTBR | 0.145759956 | 0.133079213 | 0.356387477 | NS | 0.875889778 |
| CD27 | 0.009176771 | 0.931898015 | 0.962684504 | NS | 0.030631613 |
| TAPBPL | 0.017667464 | 0.724132771 | 0.837368782 | NS | 0.140181798 |
| VAMP1 | 0.083402766 | 0.296472796 | 0.518137772 | NS | 0.528015151 |
| MRPL51 | -0.217520761 | 0.075159132 | 0.278166277 | NS | 1.124018243 |
| NCAPD2 | -0.171038234 | 0.025843801 | 0.182170348 | Down | 1.587643614 |
| GAPDH | -0.175518144 | 0.074948199 | 0.277924816 | NS | 1.125238796 |
| IFFO1 | -0.131118661 | 0.146456237 | 0.372020934 | NS | 0.834292129 |
| NOP2 | 0.082541058 | 0.413977685 | 0.615784606 | NS | 0.383023068 |
| CHD4 | 0.096659491 | 0.115560003 | 0.334479283 | NS | 0.937192457 |
| LPAR5 | 0.157886535 | 0.083497892 | 0.290972112 | NS | 1.07832449 |
| ACRBP | 0.134267453 | 0.259767078 | 0.485843002 | NS | 0.585415892 |
| ING4 | -0.148756217 | 0.038901617 | 0.214216116 | Down | 1.410032349 |
| ZNF384 | -0.042079956 | 0.688628192 | 0.815257617 | NS | 0.162015202 |
| PIANP | 0.254299016 | 0.111378037 | 0.329942771 | NS | 0.95320044 |
| COPS7A | -0.068091216 | 0.148994483 | 0.375057338 | NS | 0.826829811 |
| MLF2 | 0.115471496 | 0.067707382 | 0.267671219 | NS | 1.169363978 |
| PTMS | -0.113918056 | 0.572222935 | 0.733955912 | NS | 0.242434739 |
| LAG3 | 0.212167644 | 0.191163859 | 0.420492053 | NS | 0.71859421 |
| CD4 | 0.128016993 | 0.15897548 | 0.386625937 | NS | 0.798669855 |
| GPR162 | -0.163273308 | 0.255088334 | 0.481968846 | NS | 0.593309402 |
| P3H3 | 0.147073381 | 0.513819032 | 0.691921536 | NS | 0.289189814 |
| GNB3 | 0.482878174 | 0.190728003 | NA | NS | 0.719585539 |
| CDCA3 | 0.022847388 | 0.926170638 | 0.959397324 | NS | 0.033308992 |
| USP5 | -0.258844589 | 0.005869545 | 0.102935441 | Down | 2.231395535 |
| TPI1 | -0.211616258 | 0.016421237 | 0.151221722 | Down | 1.78459413 |
| SPSB2 | -0.120587598 | 0.115002782 | 0.333661624 | NS | 0.939291655 |
| LRRC23 | -0.140471295 | 0.164137713 | 0.391978395 | NS | 0.784791622 |
| ENO2 | -0.045212548 | 0.577291344 | 0.737786069 | NS | 0.238604954 |
| ATN1 | 0.073239611 | 0.657469755 | 0.794202854 | NS | 0.182124221 |
| C12orf57 | -0.10809675 | 0.447932128 | 0.641635461 | NS | 0.348787787 |
| PTPN6 | 0.080704618 | 0.386335576 | 0.594454271 | NS | 0.413035298 |
| PHB2 | -0.199614398 | 0.031855795 | 0.197978516 | Down | 1.496811553 |
| EMG1 | -0.177257526 | 0.028977493 | 0.189964397 | Down | 1.537939188 |
| LPCAT3 | 0.157315232 | 0.013653708 | 0.140285876 | Up | 1.864749395 |
| C1S | 0.261893042 | 0.367102981 | 0.57938064 | NS | 0.435212089 |
| C1R | 0.383350553 | 0.159812356 | NA | NS | 0.796389645 |
| C1RL | 0.062391254 | 0.548676884 | 0.716765283 | NS | 0.260683337 |
| CLSTN3 | -0.109148421 | 0.319095032 | 0.538217294 | NS | 0.496079958 |
| PEX5 | -0.189414576 | 0.004289611 | 0.092915457 | Down | 2.367582127 |
| ACSM4 | 0.45840398 | 0.21920996 | NA | NS | 0.659139717 |
| CD163L1 | 0.253026389 | 0.321230761 | 0.539828108 | NS | 0.493182874 |
| CD163 | 0.045403596 | 0.747874679 | 0.851876351 | NS | 0.12617117 |
| APOBEC1 | 0.750114028 | 0.168377747 | NA | NS | 0.773715307 |
| GDF3 | 0.030028016 | 0.963219095 | NA | NS | 0.016274916 |
| DPPA3 | 0.203714388 | 0.59918232 | NA | NS | 0.222441009 |
| CLEC4C | -0.25609724 | 0.250424996 | 0.477458646 | NS | 0.601322325 |
| NANOGNB | 0.728679751 | 0.15510072 | NA | NS | 0.809386186 |
| NANOG | 0.55488169 | 0.20482806 | 0.434924698 | NS | 0.688610549 |
| SLC2A14 | -0.141363933 | 0.580525291 | 0.739981623 | NS | 0.236178855 |
| SLC2A3 | 0.025766819 | 0.827981802 | 0.902071225 | NS | 0.081979208 |
| FOXJ2 | -0.103226389 | 0.472212896 | 0.66079798 | NS | 0.325862157 |
| C3AR1 | 0.108235161 | 0.408819152 | 0.611866175 | NS | 0.388468767 |
| NECAP1 | 0.005509455 | 0.886441891 | 0.935950794 | NS | 0.052349729 |
| CLEC4A | 0.066077455 | 0.502408095 | 0.683669055 | NS | 0.298943371 |
| ZNF705A | 0.256739473 | 0.533818665 | NA | NS | 0.272606245 |
| FAM90A1 | 0.645815092 | 0.007109705 | 0.110938851 | Up | 2.148148442 |
| CLEC6A | -0.143050237 | 0.294396923 | 0.516480516 | NS | 0.531066733 |
| CLEC4D | 0.274767953 | 0.059987224 | 0.255547275 | NS | 1.221941234 |
| CLEC4E | 0.070270783 | 0.633387498 | 0.776709642 | NS | 0.198330513 |
| AICDA | 0.451180814 | 0.123092666 | 0.343806623 | NS | 0.909767824 |
| MFAP5 | 0.198855633 | 0.632266885 | 0.775917705 | NS | 0.199099564 |
| RIMKLB | -0.047715094 | 0.438030626 | 0.633519255 | NS | 0.358495523 |
| A2ML1 | 0.333249223 | 0.316281939 | 0.535840887 | NS | 0.499925607 |
| PHC1 | 0.011145565 | 0.895250469 | 0.941286236 | NS | 0.048055443 |
| M6PR | -0.209598945 | 0.00386518 | 0.089594751 | Down | 2.412830304 |
| KLRG1 | -0.190802847 | 0.070308413 | 0.271077991 | NS | 1.152992707 |
| A2M | 0.00584281 | 0.971032365 | 0.983510672 | NS | 0.012766294 |
| PZP | 0.055493892 | 0.744631007 | 0.850176877 | NS | 0.128058883 |
| KLRB1 | -0.289758756 | 0.068502292 | 0.268602708 | NS | 1.164294899 |
| CLEC2D | -0.019002278 | 0.752003668 | 0.85466151 | NS | 0.123780041 |
| CD69 | -0.360403228 | 0.020687095 | 0.166822484 | Down | 1.684300487 |
| KLRF1 | -0.283357323 | 0.054673707 | 0.246775177 | NS | 1.262221476 |
| CLEC2B | -0.080582355 | 0.473400321 | 0.661651053 | NS | 0.324771452 |
| KLRF2 | 0.522215283 | 0.288996083 | NA | NS | 0.539108043 |
| CLEC2A | 0.00221776 | 0.996114043 | NA | NS | 0.001690937 |
| CLEC12A | -0.129975171 | 0.486162411 | 0.671618502 | NS | 0.313218623 |
| CLEC1B | -0.011630023 | 0.939909586 | 0.966780353 | NS | 0.026913921 |
| CLEC12B | 0.095255222 | 0.590816572 | 0.748017089 | NS | 0.228547332 |
| CLEC9A | -0.060604204 | 0.632140133 | 0.775886882 | NS | 0.199186637 |
| CLEC1A | 0.088925389 | 0.454995387 | 0.647163236 | NS | 0.341993007 |
| CLEC7A | 0.093613039 | 0.291788084 | 0.514555379 | NS | 0.534932447 |
| OLR1 | 0.421457063 | 0.055918049 | 0.249087638 | NS | 1.252447993 |
| TMEM52B | -0.123973859 | 0.739844306 | NA | NS | 0.130859665 |
| GABARAPL1 | -0.145883295 | 0.061735887 | 0.258242062 | NS | 1.209462306 |
| KLRD1 | 0.008994536 | 0.937740191 | 0.965732476 | NS | 0.02791747 |
| KLRK1 | 0.099586186 | 0.316890988 | 0.536370252 | NS | 0.499090111 |
| KLRC4-KLRK1 | -0.025842882 | 0.83448215 | 0.906479638 | NS | 0.078582949 |
| KLRC4 | 0.0302281 | 0.817883017 | 0.89639916 | NS | 0.08730881 |
| KLRC3 | -0.009974857 | 0.956615787 | 0.976310818 | NS | 0.019262456 |
| KLRC2 | -0.109795205 | 0.608275695 | 0.759851448 | NS | 0.215899537 |
| KLRC1 | -0.249288722 | 0.162057921 | 0.389682175 | NS | 0.790329738 |
| EIF2S3B | -1.404227364 | 1.32E-08 | 9.74E-06 | Down | 7.880059054 |
| MAGOHB | -4.02E-04 | 0.994976828 | 0.997066567 | NS | 0.002187033 |
| STYK1 | -0.013234238 | 0.925682872 | 0.959275526 | NS | 0.033537772 |
| YBX3 | -0.183781496 | 0.349307988 | 0.563369827 | NS | 0.456791483 |
| PRH1 | 0.055774158 | 0.838235346 | 0.90890645 | NS | 0.07663403 |
| TAS2R43 | -0.236232586 | 0.800484438 | NA | NS | 0.096647107 |
| PRB2 | -0.067596747 | 0.827914379 | NA | NS | 0.082014575 |
| ETV6 | 0.212738883 | 0.032469344 | 0.198993631 | Up | 1.48852648 |
| BCL2L14 | -0.138373491 | 0.743928267 | NA | NS | 0.128468939 |
| LRP6 | 0.160809341 | 0.262832632 | 0.488909438 | NS | 0.580320715 |
| MANSC1 | 0.417782978 | 0.024553685 | 0.177614809 | Up | 1.609883326 |
| BORCS5 | 0.139928654 | 0.138362353 | 0.363014451 | NS | 0.858982062 |
| DUSP16 | 0.248874855 | 0.01015053 | 0.126203819 | Up | 1.993511289 |
| CREBL2 | -0.350592667 | 1.62E-04 | 0.020530906 | Down | 3.789786717 |
| GPR19 | 0.019623221 | 0.852588236 | 0.916884663 | NS | 0.069260664 |
| CDKN1B | -0.114352452 | 0.085223195 | 0.293490206 | NS | 1.069442189 |
| APOLD1 | 0.230756549 | 0.005863399 | 0.102935441 | Up | 2.23185057 |
| DDX47 | 0.093075805 | 0.166795496 | 0.394802555 | NS | 0.77781568 |
| GPRC5A | 0.591529988 | 0.190551062 | 0.420149522 | NS | 0.719988626 |
| GPRC5D | 0.308396412 | 0.242850471 | NA | NS | 0.61466105 |
| HEBP1 | -0.062659275 | 0.732661165 | 0.84204376 | NS | 0.135096828 |
| FAM234B | -0.088572608 | 0.220284782 | 0.449439828 | NS | 0.657015505 |
| GSG1 | 0.46920485 | 0.104446763 | NA | NS | 0.981105016 |
| EMP1 | -0.111958777 | 0.473513332 | 0.661768617 | NS | 0.324667789 |
| GRIN2B | 0.435804559 | 0.266834445 | 0.492865751 | NS | 0.57375811 |
| ATF7IP | 0.05623601 | 0.388797705 | 0.596584367 | NS | 0.410276307 |
| PLBD1 | 0.085406419 | 0.514366772 | 0.692454021 | NS | 0.288727095 |
| GUCY2C | 0.284927097 | 0.112754483 | 0.331356055 | NS | 0.947866181 |
| H4-16 | -0.161854685 | 0.045702237 | 0.228303209 | Down | 1.34006254 |
| H2AJ | -0.184564406 | 0.072370629 | 0.27394225 | NS | 1.140437656 |
| WBP11 | 0.118179114 | 0.158407028 | 0.385866275 | NS | 0.800225553 |
| C12orf60 | 0.099174461 | 0.202600095 | 0.432750176 | NS | 0.693360354 |
| SMCO3 | 0.1336736 | 0.576412461 | NA | NS | 0.239266639 |
| ART4 | -0.107299272 | 0.629125858 | 0.774095547 | NS | 0.201262464 |
| MGP | 0.38366486 | 0.19909265 | NA | NS | 0.700944772 |
| ERP27 | 0.220448964 | 0.001301052 | 0.058839026 | Up | 2.885705338 |
| ARHGDIB | 0.162121826 | 0.045383915 | 0.227435977 | Up | 1.343098046 |
| PDE6H | -0.095967445 | 0.647776578 | 0.787398715 | NS | 0.188574759 |
| RERG | 0.68260462 | 0.117363519 | NA | NS | 0.930466877 |
| PTPRO | 0.104700847 | 0.188474442 | 0.417733637 | NS | 0.724747534 |
| EPS8 | 0.226528833 | 0.108981162 | 0.327029744 | NS | 0.962648566 |
| STRAP | -0.104145456 | 0.415898212 | 0.617097561 | NS | 0.381012947 |
| DERA | -0.077934886 | 0.266032271 | 0.49198493 | NS | 0.575065679 |
| SLC15A5 | 0.509642001 | 0.368482899 | NA | NS | 0.433582663 |
| MGST1 | -0.168556723 | 0.182810573 | 0.412993327 | NS | 0.737998691 |
| LMO3 | 0.487722736 | 0.168775563 | 0.396950027 | NS | 0.772690435 |
| RERGL | 0.319314347 | 0.541220795 | NA | NS | 0.266625525 |
| PIK3C2G | 0.692547231 | 0.148515899 | 0.374676263 | NS | 0.828227052 |
| PLCZ1 | 0.682406323 | 0.126752257 | 0.348680896 | NS | 0.897044298 |
| CAPZA3 | 0.034583281 | 0.969798106 | NA | NS | 0.013318668 |
| PLEKHA5 | -0.155655773 | 0.137728855 | 0.36251944 | NS | 0.860975063 |
| AEBP2 | 0.008523344 | 0.865154777 | 0.92378711 | NS | 0.06290619 |
| PDE3A | 0.028355776 | 0.865688758 | 0.92406419 | NS | 0.062638222 |
| SLCO1C1 | 0.404277372 | 0.433491466 | NA | NS | 0.363019448 |
| SLCO1B3 | 0.423696388 | 0.561916847 | NA | NS | 0.250327947 |
| SLCO1B1 | 0.034229857 | 0.954589536 | NA | NS | 0.020183331 |
| SLCO1A2 | 0.335007528 | 0.411339757 | 0.614050321 | NS | 0.385799312 |
| IAPP | 0.183685817 | 0.727493676 | NA | NS | 0.138170778 |
| PYROXD1 | -0.06234746 | 0.300140385 | 0.521612923 | NS | 0.522675565 |
| RECQL | -0.226930558 | 0.008674026 | 0.118708759 | Down | 2.061779273 |
| GOLT1B | -0.079836377 | 0.256495904 | 0.483478946 | NS | 0.590919566 |
| SPX | -0.029490652 | 0.856008365 | 0.918923014 | NS | 0.067521991 |
| GYS2 | -0.353830247 | 0.389062884 | NA | NS | 0.409980199 |
| LDHB | -0.153738642 | 0.206373434 | 0.436333491 | NS | 0.68534621 |
| KCNJ8 | 0.712110119 | 0.069012649 | NA | NS | 1.161071301 |
| ABCC9 | 0.254357491 | 0.528529991 | 0.703278992 | NS | 0.276930364 |
| CMAS | -0.274806325 | 0.04649054 | 0.229565837 | Down | 1.332635408 |
| ST8SIA1 | 0.105373667 | 0.249804681 | 0.476907072 | NS | 0.602399427 |
| C2CD5 | 0.039186385 | 0.321624362 | 0.540057273 | NS | 0.492651062 |
| ETNK1 | -0.057537754 | 0.294570269 | 0.516676747 | NS | 0.530811089 |
| SOX5 | 0.281021807 | 0.171000388 | 0.399372923 | NS | 0.767002905 |
| BCAT1 | 0.004108624 | 0.97405097 | 0.985366638 | NS | 0.011418317 |
| IRAG2 | 0.030754403 | 0.68084499 | 0.809943926 | NS | 0.166951754 |
| DNAI7 | 0.262098493 | 0.030075467 | 0.19315433 | Up | 1.521787624 |
| ETFRF1 | 0.022636576 | 0.776494856 | 0.869932305 | NS | 0.109861417 |
| KRAS | 0.009315571 | 0.857669565 | 0.91967171 | NS | 0.066680001 |
| LMNTD1 | 0.249000893 | 0.589315957 | NA | NS | 0.2296518 |
| RASSF8 | 0.084532229 | 0.730303819 | 0.840464443 | NS | 0.136496429 |
| BHLHE41 | 0.237222703 | 0.133289545 | 0.35644448 | NS | 0.875203913 |
| SSPN | 0.067317118 | 0.595221003 | 0.750729783 | NS | 0.225321753 |
| ITPR2 | 0.003572791 | 0.951066361 | 0.973466896 | NS | 0.021789179 |
| INTS13 | -0.139812275 | 0.024624411 | 0.177746811 | Down | 1.608634152 |
| FGFR1OP2 | -0.246127281 | 0.009915159 | 0.125229427 | Down | 2.00370032 |
| TM7SF3 | -0.070126886 | 0.100776666 | 0.316205947 | NS | 0.996640013 |
| MED21 | -0.001179168 | 0.985197029 | 0.991082419 | NS | 0.006476907 |
| C12orf71 | 0.460112073 | 0.103005409 | NA | NS | 0.987139971 |
| STK38L | -0.032936705 | 0.677438488 | 0.807468862 | NS | 0.169130133 |
| ARNTL2 | 0.096977903 | 0.494691684 | 0.677817745 | NS | 0.30566539 |
| SMCO2 | 0.469477231 | 0.29824792 | NA | NS | 0.525422576 |
| PPFIBP1 | 0.089657151 | 0.451487952 | 0.644308496 | NS | 0.345353835 |
| REP15 | 0.760383202 | 0.30469624 | NA | NS | 0.516132905 |
| MRPS35 | -0.323106239 | 0.001953453 | 0.069734836 | Down | 2.709197084 |
| MANSC4 | 0.233199142 | 0.306862533 | NA | NS | 0.513056135 |
| KLHL42 | -0.001829748 | 0.969956862 | 0.983124 | NS | 0.01324758 |
| PTHLH | 0.783166571 | 0.071001535 | NA | NS | 1.14873226 |
| CCDC91 | -0.27940963 | 0.020880216 | 0.167612586 | Down | 1.680265013 |
| FAR2 | 0.209012327 | 0.014517395 | 0.144329895 | Up | 1.8381113 |
| ERGIC2 | -0.01875314 | 0.793838362 | 0.880922868 | NS | 0.100267918 |
| OVCH1 | -0.43420693 | 0.160803744 | 0.388591588 | NS | 0.793703844 |
| TMTC1 | -0.333215807 | 0.085335935 | 0.293565942 | NS | 1.06886805 |
| IPO8 | -0.012437736 | 0.78456467 | 0.875595735 | NS | 0.105371252 |
| CAPRIN2 | -0.067649368 | 0.383271551 | 0.592439708 | NS | 0.416493416 |
| TSPAN11 | 0.446057445 | 0.199929034 | 0.429677949 | NS | 0.699124133 |
| DDX11 | -0.185638507 | 0.336796411 | 0.553084138 | NS | 0.472632546 |
| SINHCAF | -0.239703402 | 0.011618137 | 0.132180121 | Down | 1.934863489 |
| DENND5B | 0.079367928 | 0.400169441 | 0.605129122 | NS | 0.397756079 |
| AK4P3 | 0.477373786 | 0.220035822 | NA | NS | 0.65750661 |
| ETFBKMT | 0.150446687 | 0.08000546 | 0.285447117 | NS | 1.096880374 |
| AMN1 | 0.111503621 | 0.075837578 | 0.27918859 | NS | 1.120115543 |
| H3-5 | -0.090004571 | 0.71512513 | NA | NS | 0.14561796 |
| RESF1 | -0.048691783 | 0.576927118 | 0.737567537 | NS | 0.238879047 |
| BICD1 | 0.118690957 | 0.136178788 | 0.360467706 | NS | 0.865890537 |
| FGD4 | 0.09552169 | 0.304384376 | 0.525598354 | NS | 0.516577644 |
| DNM1L | -0.088201682 | 0.049321777 | 0.235032736 | NS | 1.306961288 |
| YARS2 | -0.066331789 | 0.294413289 | 0.516480516 | NS | 0.531042592 |
| PKP2 | 0.340603702 | 0.032307913 | 0.198422053 | Up | 1.490691089 |
| SYT10 | 0.34814778 | 0.498657386 | NA | NS | 0.302197744 |
| ALG10 | 0.088182747 | 0.14782858 | 0.373699795 | NS | 0.830241595 |
| ALG10B | -0.004957617 | 0.947056198 | 0.971536092 | NS | 0.023624249 |
| CPNE8 | 0.018605748 | 0.782928728 | 0.874453587 | NS | 0.106277771 |
| KIF21A | -0.078457501 | 0.327378436 | 0.545360849 | NS | 0.48494993 |
| ABCD2 | -0.112030171 | 0.218812456 | 0.448248137 | NS | 0.65992796 |
| C12orf40 | 0.110287741 | 0.434519001 | 0.630830691 | NS | 0.361991228 |
| SLC2A13 | -0.09046356 | 0.093164442 | 0.305593248 | NS | 1.030749811 |
| LRRK2 | 0.018192064 | 0.893051892 | 0.939877507 | NS | 0.049123305 |
| CNTN1 | 0.349977005 | 0.439904307 | 0.635045158 | NS | 0.356641786 |
| PDZRN4 | 0.550516576 | 0.200566432 | NA | NS | 0.697741751 |
| GXYLT1 | 0.057002193 | 0.177418763 | 0.407380051 | NS | 0.751000453 |
| YAF2 | 0.027391509 | 0.484063553 | 0.669931321 | NS | 0.315097615 |
| PPHLN1 | -0.036854269 | 0.438930504 | 0.634179951 | NS | 0.357604236 |
| ZCRB1 | -0.106799766 | 0.197655592 | 0.427410856 | NS | 0.704090893 |
| PRICKLE1 | 0.104465797 | 0.285652486 | 0.508789333 | NS | 0.544161991 |
| ADAMTS20 | 0.572723029 | 0.183983056 | 0.414320191 | NS | 0.735222172 |
| PUS7L | 0.012159757 | 0.831841097 | 0.904554618 | NS | 0.079959627 |
| IRAK4 | 0.083035306 | 0.067057805 | 0.266845867 | NS | 1.173550663 |
| TWF1 | -0.105704579 | 0.250739426 | 0.477700219 | NS | 0.600777373 |
| TMEM117 | -0.08241583 | 0.251857089 | 0.478833726 | NS | 0.59884582 |
| NELL2 | 0.056989795 | 0.654338774 | 0.792221749 | NS | 0.184197344 |
| DBX2 | 0.569049556 | 0.267361733 | NA | NS | 0.572900753 |
| ANO6 | -0.110264022 | 0.183560903 | 0.413733592 | NS | 0.736219814 |
| ARID2 | 0.091279659 | 0.080972114 | 0.286973593 | NS | 1.091664522 |
| SCAF11 | -0.03718359 | 0.321560395 | 0.540057273 | NS | 0.492737447 |
| SLC38A1 | -0.07829779 | 0.268582168 | 0.494180833 | NS | 0.570922824 |
| SLC38A2 | 0.016133533 | 0.820021785 | 0.897279394 | NS | 0.08617461 |
| SLC38A4 | 0.242582873 | 0.180330431 | 0.410166584 | NS | 0.74393098 |
| AMIGO2 | -0.114052403 | 0.12619171 | 0.347988266 | NS | 0.898969174 |
| PCED1B | 0.012870742 | 0.901762704 | 0.9454453 | NS | 0.044907731 |
| RPAP3 | -0.084416062 | 0.127624677 | 0.34912331 | NS | 0.894065345 |
| ENDOU | 0.314815249 | 0.016529092 | 0.151723149 | Up | 1.781751011 |
| RAPGEF3 | 0.213637119 | 0.250589337 | 0.477493717 | NS | 0.601037414 |
| SLC48A1 | 0.131194184 | 0.370182102 | 0.581355722 | NS | 0.431584583 |
| HDAC7 | 0.088358116 | 0.426700589 | 0.624815267 | NS | 0.369876757 |
| VDR | 0.098266545 | 0.423484691 | 0.622626077 | NS | 0.373162285 |
| TMEM106C | -0.083910588 | 0.230274513 | 0.458618178 | NS | 0.637754127 |
| COL2A1 | 0.597380219 | 0.131252932 | 0.35389153 | NS | 0.881890986 |
| SENP1 | -0.020001525 | 0.595054032 | 0.750601864 | NS | 0.225443598 |
| PFKM | -0.05165853 | 0.351618194 | 0.565262486 | NS | 0.453928661 |
| ASB8 | -0.070422276 | 0.223272214 | 0.451951586 | NS | 0.651165321 |
| CCDC184 | 1.274243559 | 0.003537061 | NA | Up | 2.451357399 |
| OR10AD1 | -0.012970839 | 0.922818983 | 0.957607628 | NS | 0.03488348 |
| H1-7 | 0.734397183 | 0.045718047 | NA | Up | 1.339912331 |
| ZNF641 | -0.013485605 | 0.88668589 | 0.935955238 | NS | 0.052230202 |
| ANP32D | 0.395826631 | 0.382229144 | NA | NS | 0.417676203 |
| C12orf54 | 0.397917299 | 0.392391842 | NA | NS | 0.406280031 |
| OR8S1 | 0.228056478 | 0.668725932 | NA | NS | 0.174751835 |
| OR5BS1P | 0.253787701 | 0.724839595 | NA | NS | 0.139758091 |
| LALBA | 0.468966958 | 0.430013816 | NA | NS | 0.366517591 |
| KANSL2 | -0.109393925 | 0.042635767 | 0.221215301 | Down | 1.370225919 |
| CCNT1 | 0.045312728 | 0.334255441 | 0.550791033 | NS | 0.475921515 |
| TEX49 | 0.522160522 | 0.381600626 | NA | NS | 0.418390922 |
| ADCY6 | 0.384866818 | 0.135662923 | 0.3596977 | NS | 0.867538829 |
| CACNB3 | 0.183980511 | 0.127009529 | 0.348815472 | NS | 0.896163696 |
| DDX23 | -0.217618507 | 3.78E-05 | 0.00829096 | Down | 4.422534901 |
| RND1 | 0.667352218 | 0.048299357 | NA | Up | 1.316058648 |
| CCDC65 | -0.20597957 | 0.031527759 | 0.197173328 | Down | 1.501306903 |
| FKBP11 | 0.133463726 | 0.1293658 | 0.351284745 | NS | 0.888180523 |
| ARF3 | 0.092463243 | 0.243930546 | 0.471820048 | NS | 0.612733812 |
| WNT10B | -0.088305695 | 0.423044549 | 0.622458481 | NS | 0.373613897 |
| WNT1 | 0.093391147 | 0.531982448 | 0.705114614 | NS | 0.274102696 |
| DDN | 0.870281854 | 0.053514514 | NA | NS | 1.271528418 |
| PRKAG1 | 0.128902624 | 0.132872137 | 0.356202467 | NS | 0.876566081 |
| KMT2D | -0.110630234 | 0.498234259 | 0.680131191 | NS | 0.302566414 |
| RHEBL1 | -0.171969423 | 0.164102936 | 0.39197804 | NS | 0.784883648 |
| DHH | 0.73833129 | 0.061002436 | NA | NS | 1.214652823 |
| LMBR1L | -0.046803545 | 0.282684106 | 0.505580821 | NS | 0.548698609 |
| TUBA1B | -0.197202062 | 0.023443784 | 0.173925996 | Down | 1.629972281 |
| TUBA1A | 0.033622919 | 0.769347863 | 0.865536277 | NS | 0.113877248 |
| TUBA1C | -0.249489371 | 0.004440749 | 0.09355616 | Down | 2.352543726 |
| PRPH | 0.498423149 | 0.432215449 | NA | NS | 0.364299714 |
| TROAP | -0.015065749 | 0.939919327 | 0.966780353 | NS | 0.02690942 |
| C1QL4 | 1.363434926 | 0.009364863 | NA | Up | 2.02849856 |
| DNAJC22 | 0.591814516 | 0.217701445 | 0.447277159 | NS | 0.662138689 |
| SPATS2 | 0.095599591 | 0.39295662 | 0.599318562 | NS | 0.405655391 |
| KCNH3 | 0.249633864 | 0.09150996 | 0.303287164 | NS | 1.038531633 |
| MCRS1 | 0.106432847 | 0.070453436 | 0.271317403 | NS | 1.152097821 |
| PRPF40B | 0.200194095 | 0.152414301 | 0.379041408 | NS | 0.816974281 |
| FAM186B | 0.016030988 | 0.916932082 | 0.95404482 | NS | 0.037662832 |
| FMNL3 | 0.101029566 | 0.165103044 | 0.39289172 | NS | 0.782244919 |
| TMBIM6 | -0.157805139 | 0.056154504 | 0.249221499 | NS | 1.250615405 |
| NCKAP5L | 0.140035541 | 0.384725916 | 0.59325935 | NS | 0.414848557 |
| BCDIN3D | -0.088299777 | 0.295115401 | 0.51711805 | NS | 0.530008126 |
| FAIM2 | 0.518696844 | 0.255558959 | 0.482467339 | NS | 0.592508889 |
| AQP2 | 0.719565226 | 0.139317235 | NA | NS | 0.855995152 |
| AQP5 | 1.31604952 | 0.010038009 | NA | Up | 1.99835242 |
| AQP6 | 0.668871829 | 0.161981962 | NA | NS | 0.790533344 |
| RACGAP1 | -0.102217762 | 0.092762819 | 0.305193805 | NS | 1.032626062 |
| ASIC1 | 0.428567878 | 0.112237268 | 0.330903438 | NS | 0.949862914 |
| SMARCD1 | 0.190352681 | 0.005725973 | 0.102180893 | Up | 2.242150736 |
| GPD1 | 0.273974529 | 0.303267678 | 0.524458861 | NS | 0.518173874 |
| COX14 | -0.223566044 | 0.097741833 | 0.312419185 | NS | 1.009919521 |
| CERS5 | 0.081573961 | 0.052778326 | 0.242765461 | NS | 1.277544386 |
| LIMA1 | -0.191889064 | 0.017955899 | 0.15641156 | Down | 1.745792855 |
| FAM186A | 0.252219865 | 0.337415215 | 0.5535843 | NS | 0.471835338 |
| LARP4 | -0.248720986 | 0.015027035 | 0.146973997 | Down | 1.823126716 |
| DIP2B | 0.04655557 | 0.531088427 | 0.704804549 | NS | 0.274833162 |
| ATF1 | -0.213507471 | 0.003956232 | 0.090040171 | Down | 2.40271823 |
| TMPRSS12 | 0.513843703 | 0.207757678 | NA | NS | 0.682442918 |
| METTL7A | -0.266279109 | 8.31E-04 | 0.048073047 | Down | 3.080326632 |
| HIGD1C | 0.210562525 | 0.40575917 | NA | NS | 0.391731656 |
| SLC11A2 | 0.072313196 | 0.180006751 | 0.409996481 | NS | 0.744711206 |
| LETMD1 | 0.011361263 | 0.809984237 | 0.890785913 | NS | 0.091523433 |
| CSRNP2 | 0.033164112 | 0.431811771 | 0.628813708 | NS | 0.364705523 |
| TFCP2 | 0.034457907 | 0.497246782 | 0.67963391 | NS | 0.303428019 |
| POU6F1 | -0.012628325 | 0.914112048 | 0.952101191 | NS | 0.039000567 |
| DAZAP2 | 0.024232417 | 0.719454354 | 0.834440814 | NS | 0.142996755 |
| SMAGP | 0.1140664 | 0.085090047 | 0.293307083 | NS | 1.070121236 |
| BIN2 | -0.005238264 | 0.911629377 | 0.951011314 | NS | 0.040181688 |
| CELA1 | 0.202177294 | 0.42351849 | NA | NS | 0.373127625 |
| GALNT6 | -0.271652695 | 0.006103047 | 0.104528345 | Down | 2.214453301 |
| SLC4A8 | 0.118620546 | 0.201698671 | 0.431629879 | NS | 0.695296963 |
| SCN8A | 0.319662065 | 0.119405788 | 0.340007095 | NS | 0.92297462 |
| TMDD1 | 1.160615235 | 0.002006487 | NA | Up | 2.697563651 |
| FIGNL2 | 0.380193655 | 0.002927376 | 0.08091344 | Up | 2.533521557 |
| ANKRD33 | 0.793460598 | 0.087774049 | NA | NS | 1.056633869 |
| ACVRL1 | 0.192695214 | 0.31915605 | 0.538251515 | NS | 0.495996919 |
| ACVR1B | 0.01873908 | 0.807149904 | 0.888975046 | NS | 0.0930458 |
| TAMALIN | -0.306729225 | 0.007393248 | 0.112579902 | Down | 2.13116471 |
| NR4A1 | 0.091848936 | 0.71235438 | 0.830016761 | NS | 0.147303901 |
| ATG101 | -0.22149652 | 0.01125885 | 0.130166967 | Down | 1.948505957 |
| SMIM41 | 0.825181218 | 0.094195338 | NA | NS | 1.025970592 |
| KRT80 | 0.268915153 | 0.537878926 | NA | NS | 0.269315471 |
| KRT7 | 0.828686083 | 0.023112716 | NA | Up | 1.636149012 |
| KRT86 | 0.626238838 | 0.079723598 | NA | NS | 1.09841311 |
| KRT81 | 0.730645311 | 0.075331127 | NA | NS | 1.123025536 |
| KRT83 | 1.033412286 | 0.034400864 | NA | Up | 1.463430646 |
| KRT85 | 0.958445053 | 0.036076434 | NA | Up | 1.442776394 |
| KRT84 | 0.394604201 | 0.483785324 | NA | NS | 0.31534731 |
| KRT82 | 0.567759115 | 0.1861968 | NA | NS | 0.730027788 |
| KRT75 | 0.477043388 | 0.275390786 | NA | NS | 0.560050594 |
| KRT6B | 0.615709623 | 0.22215646 | NA | NS | 0.653341053 |
| KRT6C | 0.736699175 | 0.123972311 | NA | NS | 0.906675305 |
| KRT6A | 0.541256855 | 0.280407843 | NA | NS | 0.552209844 |
| KRT5 | 0.348869036 | 0.204893002 | 0.43495148 | NS | 0.688472875 |
| KRT71 | 0.869252283 | 0.071417188 | NA | NS | 1.146197254 |
| KRT74 | 0.624773734 | 0.129600376 | NA | NS | 0.887393738 |
| KRT72 | -0.210436848 | 0.396175103 | 0.601442813 | NS | 0.402112821 |
| KRT73 | -0.190101951 | 0.480160613 | 0.667051596 | NS | 0.318613468 |
| KRT2 | 0.410120213 | 0.176576488 | 0.406141882 | NS | 0.753067126 |
| KRT1 | -0.224356359 | 0.317074033 | 0.5364679 | NS | 0.498839324 |
| KRT77 | 0.115530299 | 0.827053347 | NA | NS | 0.082466477 |
| KRT76 | 0.30508544 | 0.517961559 | NA | NS | 0.285702471 |
| KRT3 | 0.551818778 | 0.226861212 | NA | NS | 0.644239751 |
| KRT4 | 0.518768585 | 0.384897454 | NA | NS | 0.414654961 |
| KRT79 | 0.817399538 | 0.017022849 | 0.153426713 | Up | 1.768967764 |
| KRT78 | 0.678441812 | 0.1684172 | NA | NS | 0.773613557 |
| KRT8 | 0.589815351 | 0.093135262 | 0.305593248 | NS | 1.030885862 |
| KRT18 | -0.087474005 | 0.537132693 | 0.708735809 | NS | 0.269918413 |
| EIF4B | -0.289309115 | 0.00101697 | 0.05274695 | Down | 2.992691902 |
| TNS2 | 0.120699474 | 0.215873162 | 0.445318606 | NS | 0.665801346 |
| SPRYD3 | 0.272863889 | 0.018642335 | 0.158871289 | Up | 1.729499693 |
| IGFBP6 | 0.458637382 | 0.082675627 | NA | NS | 1.082622504 |
| SOAT2 | 0.195705645 | 0.495347253 | 0.67813036 | NS | 0.305090241 |
| CSAD | 0.005269965 | 0.965056428 | 0.980465179 | NS | 0.015447292 |
| ZNF740 | -0.074290248 | 0.11427236 | 0.332748328 | NS | 0.942058802 |
| ITGB7 | -0.175371667 | 0.050103282 | 0.236833467 | NS | 1.300133822 |
| RARG | 0.071599806 | 0.331612077 | 0.548582765 | NS | 0.479369661 |
| MFSD5 | -0.179654938 | 0.050310806 | 0.237303925 | NS | 1.298338727 |
| ESPL1 | 0.324878328 | 0.182478845 | 0.412549903 | NS | 0.738787475 |
| PFDN5 | -0.010543059 | 0.875892443 | 0.930331849 | NS | 0.057549221 |
| AAAS | 0.073353789 | 0.4347803 | 0.630833536 | NS | 0.361730142 |
| SP7 | 0.111534082 | 0.528613042 | 0.703281973 | NS | 0.276862126 |
| SP1 | 0.183020958 | 0.01185059 | 0.133170864 | Up | 1.926260013 |
| AMHR2 | 0.501441041 | 0.326750893 | NA | NS | 0.485783217 |
| PRR13 | 0.366917265 | 2.86E-04 | 0.028091276 | Up | 3.543511524 |
| PCBP2 | -0.018384538 | 0.877864619 | 0.93141706 | NS | 0.056572454 |
| MAP3K12 | 0.016680272 | 0.837408307 | 0.908580872 | NS | 0.077062735 |
| TARBP2 | -0.111677131 | 0.099199198 | 0.314139938 | NS | 1.003491838 |
| NPFF | 0.427680667 | 0.029926207 | NA | Up | 1.523948318 |
| ATF7 | 0.258931766 | 0.035220409 | 0.205507211 | Up | 1.4532056 |
| ATP5MC2 | -0.174922923 | 0.051144039 | 0.239138585 | NS | 1.291204974 |
| CALCOCO1 | -0.071752068 | 0.321252174 | 0.539828108 | NS | 0.493153925 |
| HOXC13 | 0.608491913 | 0.282469428 | NA | NS | 0.549028549 |
| HOXC12 | 0.733071507 | 0.142840931 | NA | NS | 0.845147328 |
| HOXC11 | 0.98989496 | 0.050959616 | NA | NS | 1.292773854 |
| HOXC10 | 0.650933391 | 0.258558358 | NA | NS | 0.587441419 |
| HOXC6 | 0.83490846 | 0.07246554 | NA | NS | 1.139868468 |
| HOXC9 | 1.326945483 | 0.00787283 | NA | Up | 2.103869135 |
| HOXC8 | 0.827486821 | 0.080240025 | NA | NS | 1.095608946 |
| HOXC4 | 0.685436664 | 0.021727207 | 0.169115127 | Up | 1.662996093 |
| HOXC5 | 0.383182369 | 0.528975309 | NA | NS | 0.276564599 |
| SMUG1 | -0.004451976 | 0.918364704 | 0.954891598 | NS | 0.036984816 |
| CBX5 | 0.191726453 | 0.021064063 | 0.168044994 | Up | 1.676457861 |
| HNRNPA1 | -0.174172075 | 0.044055769 | 0.224627854 | Down | 1.355997213 |
| NFE2 | -0.09795465 | 0.518566162 | 0.695694584 | NS | 0.285195826 |
| COPZ1 | -0.14530978 | 0.047066111 | 0.230692912 | Down | 1.327291689 |
| GPR84 | 0.102850326 | 0.612663549 | 0.763002143 | NS | 0.212777957 |
| ZNF385A | 0.138502053 | 0.487048379 | 0.672112677 | NS | 0.312427897 |
| ITGA5 | -0.018511995 | 0.821869356 | 0.89852789 | NS | 0.085197212 |
| GTSF1 | -0.045348622 | 0.744087523 | 0.850014588 | NS | 0.128375978 |
| NCKAP1L | -0.036004106 | 0.460197448 | 0.651531991 | NS | 0.337055794 |
| PDE1B | 0.354066774 | 0.02375813 | 0.174898328 | Up | 1.624187741 |
| PPP1R1A | 0.546013867 | 0.099144101 | 0.314049068 | NS | 1.003733119 |
| LACRT | 0.240657081 | 0.644079771 | NA | NS | 0.191060341 |
| DCD | 0.270146676 | 0.662375695 | NA | NS | 0.178895612 |
| MUCL1 | 0.352936719 | 0.357042012 | 0.570301029 | NS | 0.447280679 |
| TESPA1 | 0.1191827 | 0.166100003 | 0.394117516 | NS | 0.77963036 |
| NEUROD4 | 0.639127764 | 0.107425965 | NA | NS | 0.968890738 |
| OR9K2 | 0.420453045 | 0.483453467 | NA | NS | 0.315645321 |
| OR10A7 | 0.417129492 | 0.552777889 | NA | NS | 0.257449337 |
| OR6C74 | 0.675262132 | 0.13786865 | 0.362607036 | NS | 0.860534478 |
| OR6C6 | 0.383282648 | 0.584705109 | NA | NS | 0.233063111 |
| OR6C1 | 0.561256451 | 0.384144822 | NA | NS | 0.415505016 |
| OR6C3 | 0.691954893 | 0.210460162 | NA | NS | 0.676830099 |
| OR6C75 | 0.272485707 | 0.610429854 | NA | NS | 0.214364235 |
| OR6C65 | 0.391257735 | 0.529723261 | NA | NS | 0.275950956 |
| OR6C76 | 0.618191096 | 0.355241791 | NA | NS | 0.449475949 |
| OR6C2 | 0.696775298 | 0.145781291 | NA | NS | 0.836298207 |
| OR6C70 | 0.361663508 | 0.510608729 | NA | NS | 0.291911765 |
| OR6C68 | 0.20006827 | 0.709350583 | NA | NS | 0.14913907 |
| OR6C4 | -0.164813823 | 0.693858839 | NA | NS | 0.158728875 |
| OR2AP1 | 0.314730915 | 0.528177267 | NA | NS | 0.277220295 |
| OR10P1 | 0.007462714 | 0.988611437 | NA | NS | 0.00497437 |
| METTL7B | 0.465417975 | 0.20434708 | NA | NS | 0.689631563 |
| ITGA7 | 0.280707011 | 0.295532655 | 0.517431069 | NS | 0.529394525 |
| BLOC1S1 | 0.080251014 | 0.365746176 | 0.578075841 | NS | 0.436820205 |
| RDH5 | 0.286808836 | 0.013256933 | 0.138004676 | Up | 1.877556929 |
| CD63 | -0.01690639 | 0.872830666 | 0.928150063 | NS | 0.059070004 |
| GDF11 | 0.072761532 | 0.254426426 | 0.481360925 | NS | 0.594437783 |
| SARNP | 0.114876945 | 0.141051246 | 0.365424845 | NS | 0.850623074 |
| ORMDL2 | 0.012733853 | 0.812193974 | 0.8924275 | NS | 0.090340237 |
| DNAJC14 | -0.163943459 | 0.011731997 | 0.132684543 | Down | 1.930628051 |
| MMP19 | 0.557984813 | 0.083228431 | 0.29051936 | NS | 1.079728294 |
| PYM1 | -0.187117273 | 0.019730476 | 0.163246878 | Down | 1.704862439 |
| DGKA | -0.037889802 | 0.702643713 | 0.82383594 | NS | 0.153264836 |
| PMEL | -0.018195216 | 0.901989321 | 0.9454453 | NS | 0.044798604 |
| CDK2 | -0.069890029 | 0.246691963 | 0.47480291 | NS | 0.607844999 |
| RAB5B | 0.188934177 | 0.18896702 | 0.418420447 | NS | 0.723613987 |
| SUOX | 0.022078905 | 0.843956244 | 0.912443275 | NS | 0.073680069 |
| IKZF4 | 0.257310089 | 0.210060495 | 0.440111431 | NS | 0.677655615 |
| RPS26 | -0.283678753 | 0.214646909 | 0.443889264 | NS | 0.668275361 |
| ERBB3 | 0.184295676 | 0.506367642 | 0.686528045 | NS | 0.295534055 |
| PA2G4 | 0.01622073 | 0.874117804 | 0.929029107 | NS | 0.058430034 |
| RPL41 | -0.749480782 | 9.70E-04 | 0.051639949 | Down | 3.013419151 |
| ESYT1 | -0.21889527 | 0.014397215 | 0.143935548 | Down | 1.8417215 |
| ZC3H10 | 0.0451443 | 0.346976824 | 0.561433348 | NS | 0.459699533 |
| MYL6B | 0.180448943 | 0.121911002 | 0.342773783 | NS | 0.913957098 |
| MYL6 | -0.223428234 | 0.067516572 | 0.267434379 | NS | 1.170589618 |
| SMARCC2 | -0.082802735 | 0.419059213 | 0.619391222 | NS | 0.377724607 |
| RNF41 | -0.078851625 | 0.130009138 | 0.352095778 | NS | 0.88602612 |
| NABP2 | 0.100652561 | 0.242241708 | 0.47007453 | NS | 0.615751079 |
| SLC39A5 | 0.591832649 | 0.086903135 | NA | NS | 1.060964555 |
| ANKRD52 | 0.008904425 | 0.927612949 | 0.960131468 | NS | 0.032633198 |
| COQ10A | -0.122348418 | 0.061248202 | 0.257380382 | NS | 1.212906654 |
| CS | 0.139840073 | 0.050909994 | 0.238726596 | NS | 1.29319695 |
| CNPY2 | -0.16829923 | 0.039332252 | 0.214674614 | Down | 1.405251187 |
| PAN2 | -0.074053895 | 0.255136374 | 0.481973773 | NS | 0.59322762 |
| IL23A | 4.80E-04 | 0.996597549 | 0.998035641 | NS | 0.001480185 |
| STAT2 | 0.079764293 | 0.373803836 | 0.584439837 | NS | 0.427356246 |
| APOF | 0.137094555 | 0.449786289 | 0.643129524 | NS | 0.346993788 |
| TIMELESS | -0.105664014 | 0.175954388 | 0.405354744 | NS | 0.754599898 |
| MIP | -0.099502717 | 0.713727507 | NA | NS | 0.146467565 |
| SPRYD4 | 0.11859405 | 0.092956846 | 0.305431813 | NS | 1.031718619 |
| GLS2 | 0.394510626 | 0.070878788 | NA | NS | 1.149483717 |
| RBMS2 | 0.177718267 | 0.156163251 | 0.383245904 | NS | 0.806421159 |
| BAZ2A | -0.032268322 | 0.812029562 | 0.892289657 | NS | 0.09042816 |
| ATP5F1B | -0.055036624 | 0.422012805 | 0.621527536 | NS | 0.374674371 |
| PTGES3 | -0.102917629 | 0.457979134 | 0.649452133 | NS | 0.339154308 |
| NACA | -0.268922001 | 0.004085521 | 0.090695453 | Down | 2.388752506 |
| PRIM1 | -0.286027 | 0.008067837 | 0.115486104 | Down | 2.093242866 |
| HSD17B6 | -0.13509985 | 0.612635957 | 0.763002143 | NS | 0.212797517 |
| SDR9C7 | 0.43528277 | 0.451128162 | NA | NS | 0.345700061 |
| RDH16 | 0.180185201 | 0.200191694 | 0.429891249 | NS | 0.698553946 |
| GPR182 | 0.208000421 | 0.122588893 | 0.343398823 | NS | 0.911548876 |
| ZBTB39 | 0.131504222 | 0.0209601 | 0.167842555 | Up | 1.678606653 |
| TAC3 | -0.132208129 | 0.729536618 | NA | NS | 0.136952904 |
| MYO1A | 0.200787543 | 0.397834444 | 0.602951787 | NS | 0.400297618 |
| NEMP1 | -0.009574269 | 0.844414712 | 0.91272351 | NS | 0.073444209 |
| NAB2 | -0.160932382 | 0.023190354 | 0.173122445 | Down | 1.634692628 |
| STAT6 | -0.124036019 | 0.140877963 | 0.365265155 | NS | 0.851156935 |
| LRP1 | 0.045262638 | 0.764700167 | 0.863142594 | NS | 0.116508815 |
| NXPH4 | 0.63906254 | 0.135405195 | NA | NS | 0.868364674 |
| SHMT2 | -0.329631926 | 0.001021636 | 0.052784419 | Down | 2.990703652 |
| NDUFA4L2 | 0.216708592 | 0.346949764 | 0.561433348 | NS | 0.459733403 |
| STAC3 | -0.099988003 | 0.430755451 | 0.627828924 | NS | 0.365769218 |
| R3HDM2 | -0.001483038 | 0.989751424 | 0.994035648 | NS | 0.004473865 |
| INHBC | 0.392134664 | 0.403088896 | NA | NS | 0.394599166 |
| INHBE | 0.830669699 | 0.030139128 | NA | Up | 1.520869315 |
| GLI1 | 0.067628287 | 0.607648837 | 0.759420118 | NS | 0.216347329 |
| ARHGAP9 | 0.010602475 | 0.913710563 | 0.951994787 | NS | 0.039191354 |
| MARS1 | -0.061526597 | 0.165164497 | 0.392956296 | NS | 0.782083302 |
| DDIT3 | -0.06798366 | 0.441007149 | 0.635776515 | NS | 0.355554371 |
| MBD6 | -0.137237035 | 0.539616442 | 0.710206067 | NS | 0.267914826 |
| DCTN2 | 0.123776443 | 0.013287251 | 0.138194647 | Up | 1.876564876 |
| KIF5A | 0.209991709 | 0.472688513 | 0.6610873 | NS | 0.325424952 |
| PIP4K2C | -0.012485499 | 0.824555902 | 0.900078142 | NS | 0.083779895 |
| DTX3 | 0.348392373 | 0.018250269 | 0.157108372 | Up | 1.738730722 |
| ARHGEF25 | 0.782337281 | 0.069895245 | NA | NS | 1.155552371 |
| B4GALNT1 | 0.679898421 | 0.151973621 | NA | NS | 0.81823179 |
| OS9 | 0.047352327 | 0.476653736 | 0.664460977 | NS | 0.321796998 |
| AGAP2 | 0.032806535 | 0.706296614 | 0.826364601 | NS | 0.151012876 |
| TSPAN31 | -0.186534598 | 0.009907524 | 0.125229427 | Down | 2.004034864 |
| CDK4 | 0.033814797 | 0.605540057 | 0.758108478 | NS | 0.217857122 |
| MARCHF9 | 0.011701841 | 0.87010735 | 0.92673217 | NS | 0.060427163 |
| CYP27B1 | 0.418748136 | 0.077903629 | 0.282355958 | NS | 1.108442312 |
| METTL1 | -0.214085282 | 0.118859205 | 0.339500315 | NS | 0.924967177 |
| EEF1AKMT3 | -0.046901606 | 0.685194654 | 0.812880518 | NS | 0.164186034 |
| TSFM | 0.191387604 | 0.132463083 | 0.355399001 | NS | 0.877905142 |
| AVIL | 0.009646459 | 0.929062761 | 0.961212294 | NS | 0.031954947 |
| CTDSP2 | 0.026499324 | 0.789540471 | 0.878537356 | NS | 0.102625604 |
| ATP23 | -0.90985248 | 2.22E-05 | 0.005719286 | Down | 4.653141603 |
| LRIG3 | 0.476281869 | 0.247217643 | 0.475460066 | NS | 0.606920538 |
| SLC16A7 | -0.016434962 | 0.794942575 | 0.881507522 | NS | 0.099664243 |
| TAFA2 | 0.187254699 | 0.048327897 | 0.233310996 | Up | 1.315802106 |
| USP15 | -0.040664441 | 0.661938732 | 0.797292565 | NS | 0.179182206 |
| MON2 | 0.021040757 | 0.647435076 | 0.787108934 | NS | 0.188803776 |
| PPM1H | 0.183368965 | 0.168839146 | 0.396956588 | NS | 0.772526853 |
| AVPR1A | -0.039777974 | 0.798219756 | 0.883857702 | NS | 0.097877528 |
| DPY19L2 | 0.010026206 | 0.947146052 | 0.971541457 | NS | 0.023583047 |
| RXYLT1 | -0.04680534 | 0.521204469 | 0.696988482 | NS | 0.282991869 |
| SRGAP1 | 0.399136455 | 0.076079416 | 0.279719181 | NS | 1.118732832 |
| KICS2 | 0.009549119 | 0.877512029 | 0.93118249 | NS | 0.056746921 |
| C12orf56 | 0.660222789 | 0.065059368 | 0.264167619 | NS | 1.18669016 |
| XPOT | -0.125511454 | 0.021791603 | 0.169280311 | Down | 1.661710814 |
| TBK1 | -0.032032978 | 0.587086975 | 0.745603388 | NS | 0.231297555 |
| RASSF3 | 0.229330986 | 0.005035001 | 0.097887596 | Up | 2.298000456 |
| GNS | -0.059700583 | 0.571583715 | 0.733561796 | NS | 0.242920153 |
| TBC1D30 | 0.30388129 | 0.051935368 | 0.240676606 | NS | 1.284536791 |
| WIF1 | 0.690484217 | 0.193114733 | NA | NS | 0.714184593 |
| LEMD3 | -0.070956009 | 0.199279994 | 0.428926322 | NS | 0.700536299 |
| MSRB3 | 0.139210004 | 0.190448688 | 0.420036196 | NS | 0.720222014 |
| HMGA2 | 0.540320269 | 0.099480333 | 0.314541689 | NS | 1.002262771 |
| LLPH | -0.100172771 | 0.101998503 | 0.317905513 | NS | 0.991406203 |
| TMBIM4 | 0.057488626 | 0.329785882 | 0.547182226 | NS | 0.481767941 |
| IRAK3 | 0.292557111 | 0.015197536 | 0.147378943 | Up | 1.818226805 |
| HELB | 0.045983214 | 0.403358592 | 0.60738002 | NS | 0.394308688 |
| GRIP1 | 0.214767864 | 0.011454453 | 0.131355811 | Up | 1.941025649 |
| CAND1 | -0.135661218 | 0.031374205 | 0.196726942 | Down | 1.503427273 |
| DYRK2 | -0.050213597 | 0.46705372 | 0.656869584 | NS | 0.330633164 |
| IFNG | -0.158011515 | 0.187564429 | 0.417008111 | NS | 0.726849521 |
| IL26 | -0.075659027 | 0.640570465 | 0.782168094 | NS | 0.19343309 |
| IL22 | -0.012305788 | 0.966007412 | NA | NS | 0.015019541 |
| MDM1 | -0.090192343 | 0.03590264 | 0.207166105 | NS | 1.44487361 |
| RAP1B | -0.110827987 | 0.098134855 | 0.312881552 | NS | 1.008176717 |
| NUP107 | -0.224702367 | 0.00365957 | 0.08703165 | Down | 2.436569908 |
| SLC35E3 | 0.143620237 | 0.021715416 | 0.169115127 | Up | 1.663231849 |
| MDM2 | 0.064608407 | 0.35171473 | 0.565262486 | NS | 0.453809442 |
| CPM | 0.066206825 | 0.493721804 | 0.677407901 | NS | 0.306517693 |
| CPSF6 | -0.033034868 | 0.665005545 | 0.799449198 | NS | 0.177174734 |
| LYZ | 0.043519244 | 0.768921495 | 0.865305885 | NS | 0.114117998 |
| YEATS4 | -0.241039443 | 0.060834488 | 0.256782931 | NS | 1.215850144 |
| FRS2 | 0.006095402 | 0.887711722 | 0.936581465 | NS | 0.051728045 |
| CCT2 | -0.514854044 | 5.42E-04 | 0.040010004 | Down | 3.265864057 |
| LRRC10 | 0.553551037 | 0.149447297 | NA | NS | 0.825511934 |
| BEST3 | -0.159200971 | 0.576667202 | 0.737358732 | NS | 0.239074749 |
| RAB3IP | -0.136833256 | 0.147517289 | 0.373326202 | NS | 0.831157077 |
| MYRFL | 0.190057309 | 0.555507979 | 0.721922646 | NS | 0.255309698 |
| CNOT2 | 0.012915335 | 0.737537736 | 0.845484494 | NS | 0.132215754 |
| KCNMB4 | 0.054413644 | 0.693066114 | 0.817696744 | NS | 0.159225335 |
| PTPRB | 0.065147866 | 0.744082729 | 0.850014588 | NS | 0.128378776 |
| PTPRR | 0.543001949 | 0.172975304 | NA | NS | 0.762015898 |
| TSPAN8 | 0.351014682 | 0.4890817 | NA | NS | 0.310618587 |
| LGR5 | 0.555069103 | 0.178817297 | NA | NS | 0.747590473 |
| ZFC3H1 | 0.040307166 | 0.516653316 | 0.694202442 | NS | 0.286800779 |
| THAP2 | -0.101811256 | 0.244194169 | 0.472143086 | NS | 0.612264711 |
| TMEM19 | -0.13427909 | 0.107120033 | 0.323991416 | NS | 0.970129301 |
| RAB21 | -0.047057492 | 0.310184604 | 0.530296194 | NS | 0.508379762 |
| TBC1D15 | -0.019305458 | 0.726029989 | 0.838611255 | NS | 0.13904544 |
| TPH2 | 0.375105235 | 0.363375048 | 0.57563916 | NS | 0.439644898 |
| TRHDE | 0.302026402 | 0.201254923 | 0.431002455 | NS | 0.696253487 |
| ATXN7L3B | -0.010882258 | 0.846121761 | 0.91336164 | NS | 0.072567136 |
| KCNC2 | 0.563342209 | 0.07737199 | 0.281652078 | NS | 1.111416233 |
| CAPS2 | -0.27280361 | 0.017273227 | 0.154286833 | Down | 1.762626525 |
| GLIPR1L1 | -0.248326592 | 0.036340982 | 0.208236145 | Down | 1.439603345 |
| GLIPR1L2 | 0.383821079 | 0.0558718 | 0.249087638 | NS | 1.252807335 |
| GLIPR1 | -0.051853728 | 0.503996224 | 0.684785733 | NS | 0.297572717 |
| KRR1 | 0.015551203 | 0.777273645 | 0.870385342 | NS | 0.109426058 |
| PHLDA1 | 0.099050941 | 0.273831639 | 0.498830114 | NS | 0.562516374 |
| NAP1L1 | 0.099959565 | 0.114848615 | 0.333655677 | NS | 0.939874237 |
| BBS10 | -0.094053471 | 0.260761302 | 0.486910341 | NS | 0.583756859 |
| OSBPL8 | -0.084060881 | 0.373804678 | 0.584439837 | NS | 0.427355268 |
| ZDHHC17 | -0.039542519 | 0.487787969 | 0.672694188 | NS | 0.311768915 |
| CSRP2 | 0.360494263 | 0.002210232 | 0.072832159 | Up | 2.655562129 |
| E2F7 | 0.287100455 | 0.192235162 | 0.42102173 | NS | 0.716167173 |
| NAV3 | -0.054985487 | 0.831250456 | 0.904084055 | NS | 0.080268103 |
| SYT1 | 0.625510638 | 0.006953624 | 0.110119911 | Up | 2.157788816 |
| PAWR | 0.042738042 | 0.679152831 | 0.808796118 | NS | 0.168032485 |
| PPP1R12A | 0.108888098 | 0.069978541 | 0.270671937 | NS | 1.155035119 |
| OTOGL | 0.306454176 | 0.443801618 | 0.637917821 | NS | 0.352811119 |
| PTPRQ | 0.33566082 | 0.463465774 | 0.654113093 | NS | 0.333982332 |
| MYF6 | 0.766615695 | 0.173942074 | NA | NS | 0.759595357 |
| MYF5 | 1.174802781 | 0.035485974 | NA | Up | 1.449943266 |
| LIN7A | 0.165243094 | 0.238426258 | 0.466504755 | NS | 0.622645918 |
| ACSS3 | 0.187918607 | 0.299616474 | 0.520939606 | NS | 0.523434311 |
| PPFIA2 | 0.417278131 | 0.34986535 | 0.563990726 | NS | 0.456099067 |
| CCDC59 | -0.300362188 | 0.003354964 | 0.085084692 | Down | 2.474312074 |
| METTL25 | -0.23048007 | 0.014030117 | 0.14167449 | Down | 1.852938702 |
| TMTC2 | 0.098367306 | 0.135008531 | 0.358688835 | NS | 0.869638787 |
| SLC6A15 | 0.311642349 | 0.489660981 | 0.674255399 | NS | 0.310104501 |
| TSPAN19 | 0.194571515 | 0.692273427 | NA | NS | 0.159722339 |
| LRRIQ1 | 0.317246782 | 0.50824441 | 0.687821019 | NS | 0.293927389 |
| ALX1 | 1.070458449 | 0.076186552 | NA | NS | 1.11812168 |
| RASSF9 | 0.517925768 | 0.316766527 | NA | NS | 0.499260717 |
| NTS | 0.52663194 | 0.40423263 | NA | NS | 0.393368633 |
| MGAT4C | 0.344537032 | 0.42087596 | 0.620823215 | NS | 0.37584588 |
| C12orf50 | 0.220312502 | 0.633524772 | NA | NS | 0.198236399 |
| C12orf29 | -0.248704404 | 0.013231575 | 0.137928783 | Down | 1.878388448 |
| CEP290 | -0.097505876 | 0.13820091 | 0.362884344 | NS | 0.859489096 |
| TMTC3 | -0.10640896 | 0.119313922 | 0.340007095 | NS | 0.923308877 |
| KITLG | 0.010429119 | 0.961497965 | 0.978517825 | NS | 0.017051631 |
| DUSP6 | 0.069150949 | 0.427125077 | 0.625168947 | NS | 0.36944493 |
| POC1B | -0.063743921 | 0.579497427 | 0.739202967 | NS | 0.236948488 |
| POC1B-GALNT4 | 0.046928175 | 0.683095386 | 0.811353013 | NS | 0.165518648 |
| GALNT4 | 0.018247643 | 0.868676266 | 0.925923112 | NS | 0.061142044 |
| ATP2B1 | 8.44E-04 | 0.987819317 | 0.992892416 | NS | 0.005322485 |
| CCER1 | 0.71009091 | 0.170190125 | NA | NS | 0.769065644 |
| EPYC | -0.458139766 | 0.415923394 | NA | NS | 0.380986652 |
| KERA | 0.380486281 | 0.543812346 | NA | NS | 0.264550937 |
| LUM | 0.158466965 | 0.711369392 | NA | NS | 0.147904825 |
| DCN | 0.366277518 | 0.368356975 | 0.580230409 | NS | 0.433731102 |
| BTG1 | 0.14623163 | 0.006636175 | 0.108543277 | Up | 2.178082174 |
| PLEKHG7 | 0.054608373 | 0.741412508 | 0.848481332 | NS | 0.129940092 |
| EEA1 | -0.060591652 | 0.399954227 | 0.604986227 | NS | 0.397989709 |
| NUDT4 | 0.012629858 | 0.923885665 | 0.958323724 | NS | 0.034381771 |
| UBE2N | -0.107128193 | 0.197713628 | 0.427455633 | NS | 0.703963394 |
| MRPL42 | -0.105036407 | 0.160818389 | 0.388591588 | NS | 0.793664292 |
| SOCS2 | -0.086966173 | 0.304535769 | 0.525820127 | NS | 0.516361691 |
| CRADD | 0.080827481 | 0.101402601 | 0.317114382 | NS | 0.993950903 |
| PLXNC1 | 0.127412315 | 0.266788858 | 0.492821295 | NS | 0.573832313 |
| CEP83 | 0.044691514 | 0.497720081 | 0.679732613 | NS | 0.303014837 |
| TMCC3 | 0.047937222 | 0.682540058 | 0.810890305 | NS | 0.165871855 |
| NDUFA12 | -0.122176152 | 0.194252618 | 0.423733065 | NS | 0.71163312 |
| NR2C1 | -0.061618134 | 0.26935778 | 0.494693815 | NS | 0.569670476 |
| FGD6 | 0.14866084 | 0.113868631 | 0.332423088 | NS | 0.943595901 |
| VEZT | -0.082382064 | 0.11198002 | 0.330763937 | NS | 0.950859459 |
| METAP2 | -0.255454127 | 0.01490371 | 0.146349507 | Down | 1.826705622 |
| USP44 | -0.10841062 | 0.405398039 | 0.60905444 | NS | 0.392118357 |
| NTN4 | 0.204492525 | 0.196780084 | 0.426481571 | NS | 0.706018857 |
| SNRPF | -0.237978007 | 0.0261467 | 0.182899123 | Down | 1.582583112 |
| CCDC38 | 0.053650495 | 0.721108256 | 0.835389817 | NS | 0.141999532 |
| AMDHD1 | 0.348127172 | 0.008695259 | 0.118739663 | Up | 2.060717469 |
| HAL | 0.194835433 | 0.1341548 | 0.357714894 | NS | 0.872393784 |
| LTA4H | 0.027486026 | 0.759803576 | 0.859819226 | NS | 0.119298667 |
| ELK3 | 0.150703014 | 0.01145965 | 0.131355811 | Up | 1.940828636 |
| CDK17 | -0.017264676 | 0.660313491 | 0.795945129 | NS | 0.180249829 |
| CFAP54 | 0.257332628 | 0.122394112 | 0.343177027 | NS | 0.912239473 |
| NEDD1 | -0.142822745 | 0.030299866 | 0.193469884 | Down | 1.518559295 |
| TMPO | -0.216882339 | 0.004690459 | 0.095485232 | Down | 2.328784682 |
| SLC25A3 | -0.033683571 | 0.553050384 | 0.720106878 | NS | 0.257235302 |
| IKBIP | -0.183457064 | 0.074489993 | 0.277060776 | NS | 1.127902066 |
| APAF1 | 0.019153356 | 0.803943288 | 0.887406457 | NS | 0.094774586 |
| ANKS1B | 0.176989431 | 0.412833296 | 0.614932835 | NS | 0.384225283 |
| FAM71C | 0.822790064 | 0.13991524 | NA | NS | 0.854134979 |
| UHRF1BP1L | 0.038280137 | 0.586830162 | 0.745392188 | NS | 0.231487572 |
| ACTR6 | -0.21280186 | 0.104444756 | 0.320470766 | NS | 0.981113361 |
| DEPDC4 | 0.218790991 | 0.186999139 | 0.416438572 | NS | 0.728160394 |
| SCYL2 | -0.091177282 | 0.178627734 | 0.40855024 | NS | 0.748051111 |
| SLC17A8 | 0.494302722 | 0.277088698 | NA | NS | 0.557381187 |
| NR1H4 | 0.718559946 | 0.132359827 | 0.355335356 | NS | 0.87824381 |
| GAS2L3 | -0.166304637 | 0.292772664 | 0.515290877 | NS | 0.533469475 |
| ANO4 | 0.492899874 | 0.286562086 | 0.509714081 | NS | 0.542781271 |
| UTP20 | -0.007222356 | 0.894330186 | 0.940920154 | NS | 0.04850211 |
| ARL1 | -0.1684588 | 0.031355885 | 0.196726942 | Down | 1.50368094 |
| SPIC | 0.450908018 | 0.292550549 | NA | NS | 0.533799083 |
| MYBPC1 | 0.334399553 | 0.384730061 | 0.59325935 | NS | 0.414843878 |
| CHPT1 | 0.109473676 | 0.434763721 | 0.630833536 | NS | 0.361746703 |
| SYCP3 | 0.136268985 | 0.187725078 | 0.417152261 | NS | 0.726477706 |
| GNPTAB | -0.040964971 | 0.506589746 | 0.686666569 | NS | 0.295343605 |
| DRAM1 | 0.215215455 | 0.008444181 | 0.117703165 | Up | 2.073442475 |
| WASHC3 | -0.085949316 | 0.170379128 | 0.39869447 | NS | 0.76858361 |
| NUP37 | -0.142428958 | 0.067741786 | 0.267671219 | NS | 1.169143358 |
| PARPBP | -0.287829717 | 0.027680477 | 0.186639939 | Down | 1.557826434 |
| IGF1 | 0.444135982 | 0.278986773 | NA | NS | 0.554416386 |
| PAH | 0.27263505 | 0.467034592 | 0.656869584 | NS | 0.330650951 |
| ASCL1 | 0.987219093 | 0.054105113 | NA | NS | 1.266761694 |
| C12orf42 | 0.088127213 | 0.332695409 | 0.549382815 | NS | 0.477953192 |
| STAB2 | 0.363747992 | 0.195796297 | 0.425600493 | NS | 0.708195526 |
| NT5DC3 | 0.177130532 | 0.136700069 | 0.361144434 | NS | 0.864231267 |
| HSP90B1 | 0.074797196 | 0.240771804 | 0.468651695 | NS | 0.618394374 |
| C12orf73 | 0.182958502 | 0.015068755 | 0.147015649 | Up | 1.821922618 |
| TDG | -0.102838702 | 0.046072039 | 0.228777969 | Down | 1.336562566 |
| GLT8D2 | 0.451023279 | 0.003913661 | 0.089796534 | Up | 2.407416797 |
| HCFC2 | -0.090817992 | 0.089382147 | 0.299843258 | NS | 1.048749219 |
| NFYB | -0.190607482 | 0.080562388 | 0.286274601 | NS | 1.093867671 |
| TXNRD1 | -0.075530065 | 0.320896516 | 0.539507562 | NS | 0.493634998 |
| EID3 | -0.131423752 | 0.069663145 | 0.270202877 | NS | 1.156996925 |
| CHST11 | 0.043975251 | 0.516772076 | 0.694202442 | NS | 0.286700961 |
| SLC41A2 | -0.090636554 | 0.412932273 | 0.614960916 | NS | 0.384121173 |
| NOPCHAP1 | 0.103423898 | 0.145025024 | 0.37031258 | NS | 0.838557053 |
| ALDH1L2 | 0.337226362 | 0.054220248 | 0.246184004 | NS | 1.265838501 |
| WASHC4 | -0.031308931 | 0.644906016 | 0.785410134 | NS | 0.190503572 |
| APPL2 | 0.127036212 | 0.073238275 | 0.275328789 | NS | 1.135261891 |
| C12orf75 | -0.105312185 | 0.288566582 | 0.511830225 | NS | 0.539753964 |
| NUAK1 | 0.145371407 | 0.440288317 | 0.635140025 | NS | 0.356262838 |
| CKAP4 | -0.022790641 | 0.805177015 | 0.887901589 | NS | 0.094108631 |
| TCP11L2 | -0.028303201 | 0.836042388 | 0.907700899 | NS | 0.077771703 |
| POLR3B | -0.038594019 | 0.437739172 | 0.633367338 | NS | 0.358784588 |
| RFX4 | 0.496396733 | 0.115298155 | 0.334247891 | NS | 0.938177643 |
| RIC8B | -0.016811821 | 0.70869246 | 0.827410161 | NS | 0.149542188 |
| TMEM263 | -0.11167263 | 0.195537425 | 0.425199212 | NS | 0.708770109 |
| MTERF2 | -0.007195197 | 0.910956532 | 0.950725779 | NS | 0.040502346 |
| CRY1 | -0.184760868 | 0.002760507 | 0.079323886 | Down | 2.559011109 |
| BTBD11 | -0.056624963 | 0.44375604 | 0.637917821 | NS | 0.352855722 |
| PWP1 | -0.189899371 | 0.014544712 | 0.144454103 | Down | 1.837294876 |
| PRDM4 | 0.056996639 | 0.092991575 | 0.305431813 | NS | 1.031556395 |
| ASCL4 | 0.714812835 | 0.088984991 | NA | NS | 1.050683239 |
| WSCD2 | 0.347293566 | 0.268167481 | 0.493891886 | NS | 0.571593887 |
| CMKLR1 | -0.112901581 | 0.335590348 | 0.551756712 | NS | 0.474190539 |
| FICD | 0.004983283 | 0.909835713 | 0.950032257 | NS | 0.04103702 |
| SART3 | -0.178698867 | 6.39E-04 | 0.042319789 | Down | 3.194253818 |
| ISCU | -0.17545769 | 0.069580957 | 0.270000522 | NS | 1.157509602 |
| TMEM119 | 0.25612949 | 0.264301168 | 0.490441204 | NS | 0.577900918 |
| SELPLG | 0.105005396 | 0.197080729 | 0.426862716 | NS | 0.70535584 |
| CORO1C | 0.099560735 | 0.162372734 | 0.390041993 | NS | 0.789486895 |
| SSH1 | 0.165122667 | 0.092302408 | 0.304334833 | NS | 1.034786969 |
| DAO | 0.260606379 | 0.565982503 | 0.729849734 | NS | 0.247196995 |
| SVOP | 0.66394303 | 0.181964478 | 0.412022664 | NS | 0.740013384 |
| USP30 | -0.167140723 | 0.007233084 | 0.11179811 | Down | 2.14067651 |
| ALKBH2 | -0.257875474 | 0.008838785 | 0.119269279 | Down | 2.053607409 |
| UNG | -0.184341242 | 0.041111117 | 0.218382923 | Down | 1.386040726 |
| ACACB | 0.132197938 | 0.185559228 | 0.415463304 | NS | 0.731517442 |
| FOXN4 | 0.684651017 | 0.132362576 | NA | NS | 0.878234789 |
| MYO1H | 0.043638786 | 0.793242845 | 0.880637817 | NS | 0.100593837 |
| KCTD10 | 0.021485465 | 0.688357095 | 0.815165966 | NS | 0.162186207 |
| UBE3B | -0.067181465 | 0.368998433 | 0.580590828 | NS | 0.432975478 |
| MMAB | -0.116726525 | 0.105786846 | 0.321957522 | NS | 0.97556833 |
| MVK | -0.059075121 | 0.44218482 | 0.636699969 | NS | 0.354396171 |
| FAM222A | 0.567556302 | 0.121972444 | 0.342773783 | NS | 0.913738274 |
| TRPV4 | 0.661621165 | 0.066669656 | 0.266279415 | NS | 1.176071789 |
| GLTP | -0.254857146 | 0.003279025 | 0.084967003 | Down | 2.484255263 |
| TCHP | -0.106277911 | 0.065097276 | 0.264178946 | NS | 1.186437187 |
| GIT2 | 0.142620797 | 0.010762182 | 0.12826482 | Up | 1.968099683 |
| ANKRD13A | -0.001851776 | 0.978532987 | 0.987485532 | NS | 0.009424529 |
| C12orf76 | 0.060147485 | 0.238010731 | 0.466050082 | NS | 0.623403462 |
| IFT81 | -0.042996464 | 0.784742933 | 0.875675247 | NS | 0.105272587 |
| ATP2A2 | 0.014743406 | 0.810490542 | 0.891197158 | NS | 0.091252049 |
| ANAPC7 | -0.114572944 | 0.001553317 | 0.062836479 | Down | 2.808739877 |
| ARPC3 | -0.099418673 | 0.336644422 | 0.552913838 | NS | 0.472828577 |
| GPN3 | -0.202696732 | 0.053376041 | 0.243995625 | NS | 1.272653645 |
| FAM216A | -0.097448654 | 0.119082378 | 0.339629468 | NS | 0.9241525 |
| VPS29 | 0.009219443 | 0.89395057 | 0.940563971 | NS | 0.048686494 |
| RAD9B | 0.243890272 | 4.92E-05 | 0.009968332 | Up | 4.308172479 |
| PPTC7 | 0.112419095 | 0.049663885 | 0.235877703 | Up | 1.303959314 |
| TCTN1 | 0.076301702 | 0.258501747 | 0.485086532 | NS | 0.587536517 |
| HVCN1 | -0.154640374 | 0.021284239 | 0.168379028 | Down | 1.671941872 |
| PPP1CC | -0.19959835 | 0.008937282 | 0.11966441 | Down | 2.048794539 |
| CCDC63 | 0.00551517 | 0.991522129 | NA | NS | 0.003697589 |
| MYL2 | 0.476231096 | 0.40419775 | NA | NS | 0.393406108 |
| CUX2 | 0.656746769 | 0.031134202 | 0.1959427 | Up | 1.506762267 |
| PHETA1 | 0.041251396 | 0.694682797 | 0.818859716 | NS | 0.158213456 |
| SH2B3 | 0.002676299 | 0.967841966 | 0.982114953 | NS | 0.014195551 |
| ATXN2 | -0.058985778 | 0.611637303 | 0.762324763 | NS | 0.213506035 |
| BRAP | -0.195486518 | 0.005063464 | 0.098112508 | Down | 2.295552297 |
| ACAD10 | 0.029889536 | 0.553367454 | 0.720396853 | NS | 0.256986387 |
| ALDH2 | -0.159251002 | 0.165799473 | 0.393793812 | NS | 0.780416855 |
| MAPKAPK5 | -0.056274977 | 0.169206765 | 0.397453676 | NS | 0.771582277 |
| TMEM116 | -0.009944271 | 0.863271361 | 0.922858706 | NS | 0.063852667 |
| ERP29 | -0.143960741 | 0.109211805 | 0.327173527 | NS | 0.961730414 |
| NAA25 | 0.00262209 | 0.952639015 | 0.974466869 | NS | 0.021071636 |
| TRAFD1 | 0.159928904 | 0.015979571 | 0.149801122 | Up | 1.796434877 |
| HECTD4 | -0.071579853 | 0.559111149 | 0.724213069 | NS | 0.252501848 |
| RPL6 | -0.411301093 | 0.008585355 | 0.118335308 | Down | 2.066241737 |
| PTPN11 | -0.145004943 | 0.004602618 | 0.094750689 | Down | 2.336995087 |
| RPH3A | 0.630381569 | 0.041645797 | 0.22001662 | Up | 1.380428821 |
| OAS1 | 0.22940444 | 0.047242197 | 0.23095054 | Up | 1.325669916 |
| OAS3 | 0.730038905 | 8.74E-06 | 0.002819799 | Up | 5.058396974 |
| OAS2 | 0.232492665 | 0.007468748 | 0.112903808 | Up | 2.126752187 |
| DTX1 | 0.234390811 | 0.130124832 | 0.35213444 | NS | 0.885639817 |
| RASAL1 | 0.367959354 | 0.223690838 | 0.452319227 | NS | 0.650351804 |
| CFAP73 | 0.58200592 | 0.060210971 | NA | NS | 1.220324366 |
| DDX54 | -0.09844955 | 0.215725456 | 0.445134192 | NS | 0.666098604 |
| RITA1 | -0.089331894 | 0.240499439 | 0.468406361 | NS | 0.618885932 |
| IQCD | 0.034315042 | 0.879482479 | 0.931977963 | NS | 0.055772808 |
| TPCN1 | 0.112042216 | 0.411717506 | 0.614131594 | NS | 0.385400666 |
| SLC8B1 | 0.047089028 | 0.401778022 | 0.606121118 | NS | 0.396013823 |
| PLBD2 | -0.009350638 | 0.956765504 | 0.976333091 | NS | 0.019194492 |
| SDS | 0.046610209 | 0.842733757 | 0.911681082 | NS | 0.07430961 |
| SDSL | 0.122482814 | 0.491777161 | 0.675891158 | NS | 0.308231644 |
| LHX5 | 0.746792236 | 0.103696451 | NA | NS | 0.984236109 |
| RBM19 | -0.175984586 | 0.017010963 | 0.153426713 | Down | 1.769271102 |
| TBX5 | 0.558240534 | 0.200916471 | 0.430596428 | NS | 0.696984459 |
| TBX3 | 0.813560867 | 0.069699844 | 0.270202877 | NS | 1.156768193 |
| MED13L | 0.043656678 | 0.51536126 | 0.693278651 | NS | 0.287888231 |
| MAP1LC3B2 | 0.149880079 | 0.272723407 | 0.498012997 | NS | 0.564277586 |
| SPRING1 | -0.096706125 | 0.060396774 | 0.256244335 | NS | 1.218986257 |
| RNFT2 | 0.233120321 | 0.16404205 | 0.391953477 | NS | 0.785044813 |
| HRK | 0.362199613 | 0.081517516 | 0.287842422 | NS | 1.088749061 |
| FBXW8 | 0.131703927 | 0.127918927 | 0.349506689 | NS | 0.893065191 |
| TESC | -0.449996783 | 0.014020004 | 0.141634817 | Down | 1.853251866 |
| FBXO21 | -0.162141304 | 0.018866033 | 0.159320106 | Down | 1.724319422 |
| NOS1 | 0.579696804 | 0.163364821 | 0.391129522 | NS | 0.786841459 |
| KSR2 | 0.399925368 | 0.29560252 | 0.517469712 | NS | 0.529291868 |
| RFC5 | 0.002540054 | 0.955298988 | 0.975854235 | NS | 0.019860683 |
| WSB2 | 0.075559825 | 0.239229362 | 0.467477036 | NS | 0.621185517 |
| VSIG10 | 0.325024953 | 0.009885903 | 0.125229427 | Up | 2.004983664 |
| PEBP1 | -0.227000814 | 0.013954266 | 0.141407346 | Down | 1.855292999 |
| TAOK3 | -0.074540113 | 0.042567771 | 0.221206584 | NS | 1.370919086 |
| SUDS3 | -0.092530611 | 0.153607575 | 0.380233538 | NS | 0.813587366 |
| SRRM4 | 0.691414886 | 0.080684334 | 0.28635249 | NS | 1.093210782 |
| HSPB8 | 0.384710943 | 0.363687112 | NA | NS | 0.439272089 |
| CCDC60 | 0.286963471 | 0.479919936 | 0.666842078 | NS | 0.318831209 |
| TMEM233 | 0.377861493 | 0.352433021 | NA | NS | 0.452923407 |
| PRKAB1 | 0.076351837 | 0.066408915 | 0.265844603 | NS | 1.177773615 |
| CIT | 0.148317469 | 0.479938781 | 0.666842078 | NS | 0.318814156 |
| BICDL1 | 0.089211649 | 0.29236478 | 0.515175671 | NS | 0.534074946 |
| RAB35 | -0.021746312 | 0.664116284 | 0.798935311 | NS | 0.177755871 |
| GCN1 | -0.073051632 | 0.27379977 | 0.498811736 | NS | 0.562566921 |
| RPLP0 | -0.239432152 | 0.070710789 | 0.271623362 | NS | 1.150514318 |
| PXN | -0.137905029 | 0.464571498 | 0.654918093 | NS | 0.332947438 |
| SIRT4 | -0.10876879 | 0.416575096 | 0.617701707 | NS | 0.380306696 |
| PLA2G1B | 1.196091014 | 0.040745984 | NA | Up | 1.389915188 |
| MSI1 | 0.68070443 | 0.164637004 | NA | NS | 0.783472546 |
| COX6A1 | -0.237793907 | 0.030251953 | 0.193469884 | Down | 1.519246586 |
| TRIAP1 | -0.308706774 | 0.038319121 | 0.213420358 | Down | 1.416584461 |
| GATC | -0.087032727 | 0.108314091 | 0.326009898 | NS | 0.96531504 |
| SRSF9 | -0.122430313 | 0.0054158 | 0.100338483 | Down | 2.266337349 |
| DYNLL1 | -0.099784192 | 0.302430129 | 0.523922452 | NS | 0.519374945 |
| COQ5 | -0.228093832 | 0.043160191 | 0.222962086 | Down | 1.36491664 |
| RNF10 | -0.027913046 | 0.88130199 | 0.932827018 | NS | 0.054875249 |
| POP5 | -0.124197126 | 0.062377017 | 0.259501987 | NS | 1.2049754 |
| CABP1 | 0.464471808 | 0.293897221 | 0.515962844 | NS | 0.53180452 |
| MLEC | -0.013664797 | 0.72683255 | 0.838611255 | NS | 0.138565632 |
| UNC119B | 0.053289023 | 0.258919018 | 0.485499932 | NS | 0.586836049 |
| ACADS | -0.016421724 | 0.830525091 | 0.903626518 | NS | 0.080647243 |
| SPPL3 | 0.041339328 | 0.695730662 | 0.819630844 | NS | 0.157558856 |
| HNF1A | 0.216471376 | 0.50915268 | NA | NS | 0.293151966 |
| C12orf43 | -0.005461663 | 0.936330019 | 0.964721527 | NS | 0.028571053 |
| OASL | 0.145390401 | 0.299450762 | 0.520805117 | NS | 0.523674577 |
| P2RX7 | -0.014600351 | 0.872036533 | 0.927742865 | NS | 0.059465321 |
| P2RX4 | -0.112748129 | 0.06644127 | 0.265911706 | NS | 1.177562072 |
| CAMKK2 | 0.22970579 | 0.010244902 | 0.126233693 | Up | 1.9894922 |
| ANAPC5 | -0.151752071 | 1.29E-04 | 0.017955842 | Down | 3.887873767 |
| RNF34 | -0.085970891 | 0.026677299 | 0.184525374 | NS | 1.573858136 |
| KDM2B | -0.015433012 | 0.850729769 | 0.916176675 | NS | 0.07020837 |
| ORAI1 | -0.248788176 | 0.067614062 | 0.26752655 | NS | 1.169962974 |
| MORN3 | -0.079841222 | 0.414359355 | 0.616091538 | NS | 0.382622851 |
| TMEM120B | 0.102916637 | 0.259232294 | 0.485568555 | NS | 0.586310898 |
| RHOF | 0.36614961 | 0.004008958 | 0.090296145 | Up | 2.396968534 |
| SETD1B | 0.034455859 | 0.858599886 | 0.920152297 | NS | 0.066209174 |
| HPD | 0.077864801 | 0.631645929 | 0.775715902 | NS | 0.199526298 |
| PSMD9 | 0.018236932 | 0.754907031 | 0.85656564 | NS | 0.12210653 |
| CFAP251 | 0.165943875 | 0.432530514 | 0.629218386 | NS | 0.363983249 |
| BCL7A | -0.024032393 | 0.859044104 | 0.920338791 | NS | 0.065984539 |
| MLXIP | -0.243469163 | 0.166154195 | 0.394151195 | NS | 0.77948869 |
| LRRC43 | 0.650771447 | 0.019583836 | 0.162444405 | Up | 1.708102246 |
| IL31 | 0.136474797 | 0.795914525 | NA | NS | 0.09913357 |
| B3GNT4 | 0.037390425 | 0.694070782 | 0.818391034 | NS | 0.158596238 |
| DIABLO | -0.057258944 | 0.342640669 | 0.557881175 | NS | 0.46516109 |
| VPS33A | -0.135592348 | 0.008620224 | 0.118495713 | Down | 2.064481435 |
| CLIP1 | 0.069142835 | 0.263127876 | 0.489264288 | NS | 0.57983314 |
| ZCCHC8 | 0.029759174 | 0.599490063 | 0.753996454 | NS | 0.222218011 |
| RSRC2 | -0.135441932 | 0.002150313 | 0.072527942 | Down | 2.667498283 |
| KNTC1 | -0.14814209 | 0.029640449 | 0.191839895 | Down | 1.528115223 |
| HCAR2 | 0.352464384 | 0.030936353 | 0.195253594 | Up | 1.509530881 |
| HCAR3 | 0.278023709 | 0.076448401 | 0.280308141 | NS | 1.116631596 |
| HCAR1 | 0.567182316 | 0.167300297 | NA | NS | 0.776503289 |
| DENR | -0.13544293 | 0.052962446 | 0.243221565 | NS | 1.276031964 |
| CCDC62 | 0.044362393 | 0.759402007 | 0.859534683 | NS | 0.119528259 |
| HIP1R | -0.193988223 | 0.020960021 | 0.167842555 | Down | 1.678608285 |
| VPS37B | 0.227085284 | 0.016906044 | 0.153017917 | Up | 1.771957993 |
| ABCB9 | 0.349344793 | 0.103369268 | 0.31928753 | NS | 0.98560856 |
| OGFOD2 | -0.015002459 | 0.837222414 | 0.908571347 | NS | 0.077159153 |
| ARL6IP4 | -0.154539974 | 0.230941435 | 0.45931574 | NS | 0.63649814 |
| PITPNM2 | 0.074942853 | 0.495495942 | 0.678208303 | NS | 0.304959898 |
| MPHOSPH9 | -0.188324075 | 0.015863845 | 0.149445555 | Down | 1.799591537 |
| MTRFR | -0.117178249 | 0.061127953 | 0.257107876 | NS | 1.213760145 |
| CDK2AP1 | -0.061638237 | 0.51908361 | 0.69601515 | NS | 0.284762684 |
| SBNO1 | 0.04364589 | 0.276598297 | 0.500969068 | NS | 0.558150498 |
| KMT5A | -0.034788497 | 0.739073011 | 0.846651166 | NS | 0.131312656 |
| RILPL2 | -0.082600667 | 0.228613358 | 0.457186791 | NS | 0.640898396 |
| SNRNP35 | -0.129564502 | 0.018383128 | 0.157572037 | Down | 1.735580584 |
| RILPL1 | 0.107303401 | 0.279670152 | 0.503419463 | NS | 0.553353882 |
| TMED2 | -0.182290112 | 0.023671866 | 0.174639469 | Down | 1.625767511 |
| DDX55 | -0.041894734 | 0.419994885 | 0.62016266 | NS | 0.376755999 |
| EIF2B1 | -0.253958565 | 1.77E-04 | 0.021642631 | Down | 3.752721745 |
| GTF2H3 | -0.261830184 | 0.001489601 | 0.062253356 | Down | 2.826930085 |
| TCTN2 | 0.021973519 | 0.877322363 | 0.93118249 | NS | 0.0568408 |
| ATP6V0A2 | -0.008178559 | 0.833649976 | 0.905661579 | NS | 0.079016258 |
| DNAH10 | 0.312987413 | 0.123293341 | 0.344057977 | NS | 0.90906038 |
| CCDC92 | 0.0852258 | 0.389852341 | 0.59697769 | NS | 0.409099853 |
| ZNF664 | -0.084596283 | 0.059868428 | 0.25523208 | NS | 1.222802147 |
| RFLNA | 0.811798179 | 0.018290217 | NA | Up | 1.737781144 |
| NCOR2 | -0.100310566 | 0.559358286 | 0.724358091 | NS | 0.252309924 |
| SCARB1 | 0.356443509 | 0.031344277 | 0.196726942 | Up | 1.503841745 |
| UBC | -0.070149755 | 0.392348451 | 0.599004293 | NS | 0.406328057 |
| DHX37 | -0.034878142 | 0.67092555 | 0.803720941 | NS | 0.173325669 |
| BRI3BP | 0.001358163 | 0.982108643 | 0.989409763 | NS | 0.007840467 |
| AACS | -0.033955884 | 0.677358017 | 0.807456449 | NS | 0.169181725 |
| TMEM132B | 0.471126506 | 0.204517586 | 0.434697146 | NS | 0.689269343 |
| TMEM132C | 0.904848402 | 0.007270148 | 0.111927889 | Up | 2.138456722 |
| SLC15A4 | 0.035077558 | 0.681546076 | 0.810213767 | NS | 0.166504778 |
| GLT1D1 | 0.182234763 | 0.099689165 | 0.314777508 | NS | 1.00135204 |
| TMEM132D | 0.407501359 | 0.017979364 | 0.15641156 | Up | 1.745225663 |
| FZD10 | 0.596235978 | 0.279137389 | NA | NS | 0.554181988 |
| RIMBP2 | 0.4199813 | 0.21093434 | 0.440805655 | NS | 0.675852712 |
| STX2 | 0.006548517 | 0.953416361 | 0.974839407 | NS | 0.0207174 |
| RAN | -0.227080747 | 0.035247994 | 0.205555536 | Down | 1.452865588 |
| ADGRD1 | 0.158239997 | 0.270805016 | 0.496051178 | NS | 0.567343296 |
| SFSWAP | -0.035569353 | 0.50297185 | 0.684134317 | NS | 0.298456321 |
| MMP17 | 0.208266411 | 0.310015814 | 0.530293015 | NS | 0.508616153 |
| ULK1 | -0.09094527 | 0.631998185 | 0.775886882 | NS | 0.199284169 |
| PUS1 | -0.038984239 | 0.516787068 | 0.694202442 | NS | 0.286688363 |
| EP400 | 0.050004425 | 0.718170945 | 0.833717613 | NS | 0.143772169 |
| DDX51 | 0.070240621 | 0.613997276 | 0.763977269 | NS | 0.211833556 |
| NOC4L | -0.052679113 | 0.689162289 | 0.815289774 | NS | 0.161678495 |
| FBRSL1 | 0.112337968 | 0.564868324 | 0.728912205 | NS | 0.248052779 |
| LRCOL1 | 0.709061647 | 0.100967671 | NA | NS | 0.99581766 |
| P2RX2 | 0.568278708 | 0.070800281 | NA | NS | 1.149965017 |
| POLE | -0.044896044 | 0.577454943 | 0.737912794 | NS | 0.238481897 |
| PXMP2 | 0.142923701 | 0.310020661 | 0.530293015 | NS | 0.508609362 |
| PGAM5 | -0.099533323 | 0.057214045 | 0.250855835 | NS | 1.242497347 |
| ANKLE2 | -0.112147532 | 0.001452569 | 0.062064799 | Down | 2.837863265 |
| GOLGA3 | -0.143507303 | 0.065066617 | 0.264167619 | NS | 1.186641773 |
| CHFR | -0.013320127 | 0.776133419 | 0.869703863 | NS | 0.110063616 |
| ZNF605 | -0.114696149 | 0.098430459 | 0.313177879 | NS | 1.006870489 |
| ZNF26 | -0.021293184 | 0.663921555 | 0.798798605 | NS | 0.177883231 |
| ZNF84 | -0.079977965 | 0.22768286 | 0.45648191 | NS | 0.642669662 |
| ZNF140 | 0.030368351 | 0.555775 | 0.721941582 | NS | 0.255100992 |
| ZNF891 | 0.058223893 | 0.404829631 | 0.60860084 | NS | 0.392727708 |
| ZNF10 | 0.004320183 | 0.950114417 | 0.973014371 | NS | 0.022224092 |
| ZNF268 | -0.171525472 | 0.064540453 | 0.263569594 | NS | 1.190167993 |
| ANHX | 0.552440061 | 0.280907653 | NA | NS | 0.551436429 |
| TUBA3C | 1.424460694 | 0.006144332 | NA | Up | 2.211525344 |
| TPTE2 | 0.453030243 | 0.185709135 | 0.415463304 | NS | 0.731166733 |
| MPHOSPH8 | -0.021739402 | 0.590354084 | 0.747720896 | NS | 0.228887428 |
| PSPC1 | -0.091524934 | 0.018387748 | 0.157572037 | NS | 1.73547145 |
| ZMYM5 | -0.183135328 | 0.005493047 | 0.100721984 | Down | 2.260186701 |
| ZMYM2 | 0.02127947 | 0.587278135 | 0.745763452 | NS | 0.231156168 |
| GJA3 | 0.381349488 | 0.157431041 | 0.384872513 | NS | 0.802909633 |
| GJB2 | 0.465337134 | 0.360559037 | NA | NS | 0.443023615 |
| GJB6 | 0.078574741 | 0.745950554 | 0.850700109 | NS | 0.127289959 |
| CRYL1 | -0.175536833 | 0.032045279 | 0.198142899 | Down | 1.49423595 |
| IFT88 | -0.146396348 | 0.0297716 | 0.192227002 | Down | 1.526197829 |
| IL17D | -0.038611869 | 0.878838978 | NA | NS | 0.05609069 |
| EEF1AKMT1 | -0.212592204 | 0.063749008 | 0.262209014 | NS | 1.195526566 |
| XPO4 | -0.025012501 | 0.615729407 | 0.765059537 | NS | 0.210610104 |
| LATS2 | 0.127738194 | 0.070754482 | 0.271700057 | NS | 1.150246047 |
| SAP18 | -0.198971978 | 0.004070537 | 0.090508199 | Down | 2.390348264 |
| SKA3 | -0.576779236 | 0.006649057 | 0.108543277 | Down | 2.177239945 |
| MRPL57 | -0.236210361 | 0.022505896 | 0.171387603 | Down | 1.647703699 |
| ZDHHC20 | -0.227698908 | 0.003581711 | 0.086556724 | Down | 2.445909401 |
| MICU2 | -0.095556363 | 0.161196957 | 0.389034501 | NS | 0.792643161 |
| FGF9 | 0.132993446 | 0.363955846 | 0.576120873 | NS | 0.4389513 |
| SGCG | 0.187326573 | 0.505221343 | NA | NS | 0.296518311 |
| SACS | -0.145417923 | 0.066218836 | 0.2655108 | NS | 1.179018459 |
| TNFRSF19 | 0.279339734 | 0.248324903 | 0.476110795 | NS | 0.604979725 |
| MIPEP | -0.013502973 | 0.819642857 | 0.8971687 | NS | 0.086375341 |
| C1QTNF9B | 0.534692929 | 0.38469877 | NA | NS | 0.414879203 |
| SPATA13 | 0.022767677 | 0.648392251 | 0.787937988 | NS | 0.188162184 |
| C1QTNF9 | -0.037796914 | 0.825869652 | 0.900867904 | NS | 0.083088492 |
| PARP4 | 0.081885777 | 0.127338125 | 0.348830209 | NS | 0.895041549 |
| ATP12A | 0.521333633 | 0.244786334 | NA | NS | 0.611212832 |
| RNF17 | 0.085518967 | 0.820302148 | 0.897461425 | NS | 0.086026151 |
| CENPJ | -0.103045724 | 0.0463791 | 0.229315052 | Down | 1.333677679 |
| PABPC3 | 0.771951445 | 0.194252785 | NA | NS | 0.711632745 |
| AMER2 | 0.520419603 | 0.224104156 | 0.452515727 | NS | 0.64955009 |
| MTMR6 | -0.063744729 | 0.452646798 | 0.645312359 | NS | 0.344240548 |
| NUP58 | -0.025491354 | 0.62068873 | 0.767922494 | NS | 0.20712614 |
| ATP8A2 | 0.294179307 | 0.150688234 | 0.377206464 | NS | 0.821920657 |
| SHISA2 | 0.446437617 | 0.067515539 | NA | NS | 1.170596261 |
| RNF6 | -0.098577117 | 0.141536434 | 0.365923729 | NS | 0.849131752 |
| CDK8 | -0.064873048 | 0.602271449 | 0.75600004 | NS | 0.220207724 |
| WASF3 | 0.481424941 | 0.032495848 | 0.198993631 | Up | 1.488172119 |
| GPR12 | 0.80225377 | 0.074397102 | NA | NS | 1.128443982 |
| USP12 | -0.587857805 | 9.03E-04 | 0.050282353 | Down | 3.044354121 |
| RPL21 | -0.648885601 | 0.003974789 | 0.090040171 | Down | 2.400685945 |
| RASL11A | -0.016002911 | 0.895351733 | 0.941303039 | NS | 0.048006321 |
| GTF3A | -0.191286226 | 0.02222194 | 0.170639329 | Down | 1.65321802 |
| MTIF3 | -0.053388884 | 0.427373531 | 0.62541269 | NS | 0.369192379 |
| LNX2 | -0.055932623 | 0.588630132 | 0.746776384 | NS | 0.23015751 |
| POLR1D | -0.220979907 | 0.045920135 | 0.228673646 | Down | 1.337996843 |
| GSX1 | 0.658322192 | 0.174289941 | NA | NS | 0.758727678 |
| PDX1 | 0.881809431 | 0.049145793 | 0.234974405 | Up | 1.308513651 |
| CDX2 | 0.927896529 | 0.044273155 | NA | Up | 1.353859526 |
| URAD | 1.08069174 | 0.049975906 | NA | Up | 1.301239321 |
| FLT3 | -0.213143088 | 0.21554161 | 0.444834997 | NS | 0.666468877 |
| PAN3 | 0.041027451 | 0.505993277 | 0.686297541 | NS | 0.295855253 |
| FLT1 | 0.22330027 | 0.175783077 | 0.40521632 | NS | 0.755022936 |
| POMP | -0.133087773 | 0.180077372 | 0.410006074 | NS | 0.744540857 |
| SLC46A3 | 0.029551856 | 0.568836953 | 0.731594536 | NS | 0.245012199 |
| MTUS2 | 0.384840246 | 0.081148157 | 0.2872419 | NS | 1.090721339 |
| SLC7A1 | -0.055254218 | 0.678207195 | 0.808048132 | NS | 0.168637607 |
| UBL3 | -0.1078862 | 0.162423945 | 0.390045866 | NS | 0.789349945 |
| KATNAL1 | -0.145217281 | 0.084059733 | 0.292007269 | NS | 1.075411993 |
| UBE2L5 | 0.132904295 | 0.705931659 | NA | NS | 0.151237341 |
| HMGB1 | -0.258870633 | 0.006859581 | 0.109849098 | Down | 2.163702394 |
| USPL1 | -0.04904012 | 0.278440537 | 0.502868581 | NS | 0.555267538 |
| ALOX5AP | 0.280512039 | 0.059606153 | 0.254791869 | NS | 1.224708904 |
| MEDAG | 0.479642991 | 0.179404241 | NA | NS | 0.746167294 |
| TEX26 | 0.851984492 | 0.073643742 | NA | NS | 1.132864151 |
| HSPH1 | -0.1173581 | 0.128953692 | 0.35091462 | NS | 0.889566221 |
| B3GLCT | -0.238037295 | 4.09E-04 | 0.034543974 | Down | 3.388059502 |
| RXFP2 | 0.113786426 | 0.524298527 | 0.699765205 | NS | 0.280421363 |
| FRY | 0.130420899 | 0.189342162 | 0.418800923 | NS | 0.722752669 |
| ZAR1L | 0.456315875 | 0.181309195 | NA | NS | 0.741580171 |
| BRCA2 | -0.017328324 | 0.826637549 | 0.901249793 | NS | 0.082684871 |
| N4BP2L1 | -0.088785694 | 0.064473988 | 0.263488519 | NS | 1.190615467 |
| N4BP2L2 | -0.046756277 | 0.428977655 | 0.626678972 | NS | 0.367565329 |
| PDS5B | 0.046978968 | 0.28380835 | 0.506589562 | NS | 0.546974831 |
| KL | 0.369526458 | 0.046262773 | 0.229182357 | Up | 1.334768336 |
| STARD13 | 0.382515787 | 0.10648932 | 0.322999226 | NS | 0.972693945 |
| RFC3 | -0.06971057 | 0.237415265 | 0.46528191 | NS | 0.624491361 |
| NBEA | 0.033059337 | 0.780880885 | 0.873363014 | NS | 0.107415208 |
| MAB21L1 | -0.04781174 | 0.863775893 | NA | NS | 0.063598921 |
| DCLK1 | 0.555950256 | 0.190756954 | 0.420152441 | NS | 0.719519621 |
| SOHLH2 | 0.677570853 | 0.185613753 | NA | NS | 0.731389848 |
| CCDC169 | 0.528268866 | 0.169891204 | 0.398309642 | NS | 0.769829105 |
| SPART | -0.131197785 | 0.064907137 | 0.264064926 | NS | 1.187707549 |
| CCNA1 | 0.440664523 | 0.133680942 | 0.35690848 | NS | 0.873930502 |
| SERTM1 | 1.038314322 | 0.054584302 | NA | NS | 1.26293224 |
| RFXAP | 0.150783145 | 0.032751352 | 0.199752719 | Up | 1.484770768 |
| SMAD9 | 0.28680305 | 0.005468408 | 0.100673218 | Up | 2.262139079 |
| ALG5 | -0.032615274 | 0.56738421 | 0.730628798 | NS | 0.246122755 |
| EXOSC8 | 0.025813199 | 0.597401082 | 0.752238586 | NS | 0.223733995 |
| SUPT20H | 0.042664303 | 0.418550265 | 0.618901614 | NS | 0.378252379 |
| CSNK1A1L | 0.321070923 | 0.087174048 | 0.296703312 | NS | 1.059612785 |
| POSTN | 0.425195981 | 0.376020375 | 0.586177342 | NS | 0.424788622 |
| TRPC4 | 0.059940174 | 0.905473431 | 0.947680858 | NS | 0.043124288 |
| UFM1 | -0.262983065 | 0.006045001 | 0.103993354 | Down | 2.218603599 |
| FREM2 | 0.697294816 | 0.106330442 | 0.322754112 | NS | 0.973342382 |
| STOML3 | 0.4981091 | 0.360957808 | NA | NS | 0.44254356 |
| PROSER1 | 0.096964879 | 0.0946648 | 0.307912689 | NS | 1.023811479 |
| NHLRC3 | 0.08212715 | 0.078770763 | 0.283635871 | NS | 1.103634945 |
| LHFPL6 | 0.092546965 | 0.722176831 | 0.836035676 | NS | 0.141356449 |
| COG6 | -0.096810847 | 0.112490792 | 0.330929727 | NS | 0.948883026 |
| FOXO1 | 0.103972707 | 0.034941103 | 0.20492492 | Up | 1.456663385 |
| MRPS31 | -0.165110272 | 0.021863488 | 0.169347779 | Down | 1.660280542 |
| SLC25A15 | -0.07909704 | 0.200045773 | 0.429790861 | NS | 0.698870621 |
| ELF1 | -0.001518028 | 0.976493797 | 0.986397233 | NS | 0.010330511 |
| WBP4 | -0.26536971 | 0.004407913 | 0.093388262 | Down | 2.355766987 |
| KBTBD6 | -0.005204056 | 0.944895632 | 0.970055575 | NS | 0.024616159 |
| KBTBD7 | 0.008748939 | 0.938217734 | 0.966009763 | NS | 0.027696362 |
| MTRF1 | -0.135930016 | 0.149292652 | 0.375271684 | NS | 0.825961567 |
| NAA16 | -0.107775884 | 0.206967919 | 0.436943551 | NS | 0.684096967 |
| RGCC | -0.359726033 | 0.013249819 | 0.138004676 | Down | 1.87779006 |
| VWA8 | 0.056063304 | 0.47149946 | 0.660409087 | NS | 0.3265188 |
| DGKH | 0.094013783 | 0.183616637 | 0.413733592 | NS | 0.73608797 |
| AKAP11 | 0.098597536 | 0.074821987 | 0.277636607 | NS | 1.125970764 |
| TNFSF11 | 0.112274193 | 0.653969209 | 0.792109309 | NS | 0.184442699 |
| FAM216B | 0.363045702 | 0.447126891 | 0.64108809 | NS | 0.34956921 |
| EPSTI1 | 0.340179522 | 0.022838925 | 0.172237653 | Up | 1.641324333 |
| DNAJC15 | 0.005278863 | 0.951448052 | 0.97361348 | NS | 0.021614919 |
| ENOX1 | 0.351770645 | 0.363675749 | NA | NS | 0.439285658 |
| CCDC122 | 0.162789211 | 0.20345847 | 0.433444748 | NS | 0.691524225 |
| LACC1 | -0.109422474 | 0.205225875 | 0.435273475 | NS | 0.687767884 |
| SMIM2 | 0.478250349 | 0.374461601 | NA | NS | 0.42659271 |
| SERP2 | 0.738157038 | 0.002043513 | NA | Up | 2.689622685 |
| TSC22D1 | 0.117810712 | 0.157030447 | 0.384262345 | NS | 0.804016134 |
| NUFIP1 | 0.044353891 | 0.61272575 | 0.763016971 | NS | 0.212733868 |
| GPALPP1 | -0.107782782 | 0.08053305 | 0.286274601 | NS | 1.09402585 |
| GTF2F2 | -0.1025255 | 0.154079907 | 0.380990933 | NS | 0.812253994 |
| KCTD4 | 0.113091127 | 0.316661857 | 0.536245921 | NS | 0.499404246 |
| TPT1 | -0.416153704 | 0.049977165 | 0.236733572 | Down | 1.301228387 |
| SLC25A30 | 0.009359105 | 0.785980612 | 0.876321892 | NS | 0.104588166 |
| COG3 | -0.061042915 | 0.041743024 | 0.220293088 | NS | 1.379416088 |
| ERICH6B | 0.034237967 | 0.880969362 | 0.932756987 | NS | 0.055039195 |
| CBY2 | 0.655103385 | 0.112342575 | NA | NS | 0.949455626 |
| SIAH3 | 0.291662737 | 0.38632072 | 0.594454271 | NS | 0.413051998 |
| ZC3H13 | -0.149268303 | 0.020575677 | 0.166586724 | Down | 1.686645858 |
| CPB2 | 0.217055754 | 0.291876625 | 0.514593347 | NS | 0.534800685 |
| LCP1 | -0.120057047 | 0.21458838 | 0.443861438 | NS | 0.668393799 |
| LRRC63 | -0.007166494 | 0.957436098 | 0.976655159 | NS | 0.018890202 |
| RUBCNL | -0.064729031 | 0.468753671 | 0.6580408 | NS | 0.329055318 |
| LRCH1 | 0.129831904 | 0.005957572 | 0.103288853 | Up | 2.224930699 |
| ESD | -0.135494703 | 0.128904039 | 0.350821187 | NS | 0.889733476 |
| HTR2A | 0.039795725 | 0.894741205 | 0.941068205 | NS | 0.048302562 |
| SUCLA2 | -0.107533334 | 0.127809086 | 0.349398501 | NS | 0.89343827 |
| NUDT15 | -0.177079932 | 0.040419182 | 0.216879751 | Down | 1.393412481 |
| MED4 | -0.088989072 | 0.159550302 | 0.387098757 | NS | 0.797102369 |
| ITM2B | 0.094045216 | 0.348629257 | 0.562740382 | NS | 0.45763617 |
| RB1 | -0.04465572 | 0.468465952 | 0.657926488 | NS | 0.329321968 |
| LPAR6 | -0.249370828 | 0.038874211 | 0.214216116 | Down | 1.410338414 |
| RCBTB2 | -0.068202494 | 0.335810317 | 0.551900099 | NS | 0.473905965 |
| CYSLTR2 | 0.223320972 | 0.064106935 | 0.262789011 | NS | 1.193094986 |
| FNDC3A | 0.0314454 | 0.386227909 | 0.59436847 | NS | 0.413156347 |
| MLNR | 0.800726742 | 0.048494537 | NA | Up | 1.314307185 |
| CDADC1 | -0.089712963 | 0.088088954 | 0.29803361 | NS | 1.055078548 |
| CAB39L | 0.09199218 | 0.173759358 | 0.402859976 | NS | 0.760051798 |
| SETDB2 | -0.21135803 | 0.012093065 | 0.134107677 | Down | 1.917463597 |
| PHF11 | 0.030243486 | 0.52301644 | 0.698750737 | NS | 0.28148466 |
| RCBTB1 | -0.026337468 | 0.620729392 | 0.767931317 | NS | 0.20709769 |
| ARL11 | 0.185608983 | 0.051413476 | 0.239542615 | NS | 1.288923037 |
| EBPL | -0.114329256 | 0.227034186 | 0.455511653 | NS | 0.643908744 |
| KPNA3 | -0.052677305 | 0.265375 | 0.491568941 | NS | 0.576139992 |
| SPRYD7 | -0.133970028 | 0.1790924 | 0.409134578 | NS | 0.746922844 |
| TRIM13 | 0.048583436 | 0.351297191 | 0.565122842 | NS | 0.454325323 |
| KCNRG | 0.203835727 | 0.066391897 | 0.265822942 | NS | 1.177884921 |
| DLEU7 | 0.234333417 | 0.106616136 | 0.323123005 | NS | 0.972177062 |
| RNASEH2B | -0.016546756 | 0.744541877 | 0.850164936 | NS | 0.12811087 |
| C13orf42 | 0.424113035 | 0.427802543 | NA | NS | 0.368756638 |
| FAM124A | 0.539422913 | 0.069746616 | 0.270202877 | NS | 1.156476859 |
| SERPINE3 | 0.225203628 | 0.031058925 | 0.195845679 | Up | 1.507813577 |
| INTS6 | 0.051662979 | 0.248272615 | 0.476110795 | NS | 0.605071182 |
| WDFY2 | 0.155100865 | 0.023994456 | 0.175491741 | Up | 1.619889087 |
| DHRS12 | 0.207913864 | 0.036505529 | 0.208452907 | Up | 1.437641353 |
| TMEM272 | 0.243355097 | 0.084301146 | 0.29207932 | NS | 1.074166522 |
| CCDC70 | 0.208910991 | 0.631821295 | NA | NS | 0.199405741 |
| ATP7B | 0.165009809 | 0.234117985 | 0.46228643 | NS | 0.630565223 |
| ALG11 | 0.023776441 | 0.654956917 | 0.792662798 | NS | 0.183787267 |
| NEK5 | 0.242043874 | 0.175968774 | 0.405354744 | NS | 0.754564391 |
| NEK3 | -0.032343549 | 0.687162107 | 0.814178911 | NS | 0.162940797 |
| THSD1 | 0.184453267 | 0.224721396 | 0.453043082 | NS | 0.648355576 |
| VPS36 | -0.040814652 | 0.337634981 | 0.553706862 | NS | 0.471552565 |
| CKAP2 | -0.113704379 | 0.137684503 | 0.36251944 | NS | 0.861114937 |
| SUGT1 | -0.032039896 | 0.539496844 | 0.710206067 | NS | 0.268011091 |
| CNMD | 0.387693308 | 0.039323224 | 0.214674614 | Up | 1.405350883 |
| PCDH8 | 0.943541644 | 0.01384799 | NA | Up | 1.858613247 |
| OLFM4 | 0.589987258 | 0.032815795 | 0.200039216 | Up | 1.483917071 |
| PCDH17 | 0.678695175 | 0.059620541 | 0.254791869 | NS | 1.224604089 |
| DIAPH3 | -0.136106769 | 0.55729155 | 0.72308034 | NS | 0.253917542 |
| TDRD3 | -0.055779302 | 0.382932545 | 0.59214603 | NS | 0.416877722 |
| PCDH20 | 0.268775932 | 0.800644019 | NA | NS | 0.096560536 |
| PCDH9 | 0.086218554 | 0.548289435 | 0.716413556 | NS | 0.260990122 |
| KLHL1 | 0.263100989 | 0.601760246 | NA | NS | 0.220576507 |
| DACH1 | 0.35268465 | 0.009430519 | 0.122353766 | Up | 2.025464398 |
| MZT1 | -0.291627464 | 0.013394135 | 0.138927758 | Down | 1.873085314 |
| BORA | -0.057652925 | 0.36971224 | 0.581122148 | NS | 0.432136171 |
| DIS3 | -0.090560158 | 0.057411042 | 0.251059516 | NS | 1.241004569 |
| PIBF1 | -0.196707302 | 0.001305136 | 0.058839026 | Down | 2.884344225 |
| KLF5 | 0.208594242 | 0.066117609 | 0.265377158 | NS | 1.179682862 |
| KLF12 | -0.037717625 | 0.582335598 | 0.741501716 | NS | 0.234826661 |
| TBC1D4 | -0.163630262 | 0.076992715 | 0.281360643 | NS | 1.113550368 |
| COMMD6 | -0.811138484 | 0.001286837 | 0.058646647 | Down | 2.89047655 |
| UCHL3 | 0.078075679 | 0.382947989 | 0.59214603 | NS | 0.416860207 |
| LMO7 | 0.116076056 | 0.205557159 | 0.435492143 | NS | 0.687067393 |
| KCTD12 | 0.027230304 | 0.785553421 | 0.876229517 | NS | 0.104824276 |
| ACOD1 | -0.173780186 | 0.613572347 | NA | NS | 0.212134222 |
| CLN5 | 0.127324194 | 0.035048472 | 0.205289029 | Up | 1.455330911 |
| FBXL3 | -0.1334527 | 0.081389966 | 0.287719656 | NS | 1.089429132 |
| MYCBP2 | 0.013841838 | 0.814938833 | 0.89432791 | NS | 0.088874987 |
| SCEL | 0.65126034 | 0.139631844 | 0.363934068 | NS | 0.855015528 |
| SLAIN1 | -0.191489233 | 0.014906429 | 0.146349507 | Down | 1.826626388 |
| EDNRB | 0.59944079 | 0.034674932 | 0.204304637 | Up | 1.459984383 |
| POU4F1 | 0.572078146 | 0.134321243 | NA | NS | 0.871855299 |
| OBI1 | -0.397274289 | 0.00350253 | 0.085936504 | Down | 2.455618111 |
| RBM26 | 0.043018011 | 0.501964149 | 0.683308543 | NS | 0.2993273 |
| NDFIP2 | -0.010031912 | 0.927217791 | 0.959888272 | NS | 0.032818244 |
| SPRY2 | -0.117595262 | 0.34565033 | 0.560549771 | NS | 0.461363024 |
| SLITRK1 | 0.612652135 | 0.270258447 | NA | NS | 0.568220723 |
| SLITRK6 | 0.722718843 | 0.223041387 | NA | NS | 0.651614543 |
| SLITRK5 | -0.169730298 | 0.483392304 | 0.669406854 | NS | 0.315700268 |
| GPC5 | 0.348746277 | 0.22979023 | 0.458066753 | NS | 0.63866844 |
| GPC6 | 0.270470677 | 0.48374094 | NA | NS | 0.315387156 |
| DCT | 0.389420645 | 0.078506459 | 0.283187104 | NS | 1.105094612 |
| TGDS | -0.059078121 | 0.395764165 | 0.601183351 | NS | 0.402563532 |
| GPR180 | -0.028218245 | 0.710621794 | 0.828951459 | NS | 0.148361477 |
| SOX21 | 0.957710137 | 0.023684799 | NA | Up | 1.625530301 |
| ABCC4 | -0.255653225 | 0.08412091 | 0.292007269 | NS | 1.075096039 |
| CLDN10 | 0.715740802 | 0.035257729 | NA | Up | 1.452745667 |
| DZIP1 | 0.64045388 | 0.069472709 | 0.269939848 | NS | 1.158185768 |
| DNAJC3 | 0.071474834 | 0.429347295 | 0.627018987 | NS | 0.367191268 |
| UGGT2 | -0.107678483 | 0.466671236 | 0.656560381 | NS | 0.330988966 |
| HS6ST3 | 0.508142714 | 0.304792961 | 0.526026255 | NS | 0.515995066 |
| OXGR1 | 0.750498996 | 0.0481625 | NA | Up | 1.317290976 |
| MBNL2 | -0.090423802 | 0.079908813 | 0.285326242 | NS | 1.097405322 |
| RAP2A | -0.257959315 | 0.018175433 | 0.156913557 | Down | 1.740515226 |
| IPO5 | -0.149107862 | 0.02319908 | 0.173122445 | Down | 1.634529236 |
| FARP1 | 0.280163372 | 0.008227535 | 0.116684441 | Up | 2.084730278 |
| RNF113B | 0.964449654 | 0.033671173 | NA | Up | 1.472741755 |
| STK24 | -0.109465252 | 0.083892479 | 0.291681426 | NS | 1.076276972 |
| SLC15A1 | 0.64961805 | 0.193054279 | 0.422285492 | NS | 0.714320567 |
| DOCK9 | -0.009857643 | 0.881729425 | 0.933063824 | NS | 0.054664666 |
| UBAC2 | -0.04261462 | 0.281957555 | 0.504917658 | NS | 0.549816264 |
| GPR18 | 0.074663569 | 0.204941115 | 0.43495148 | NS | 0.688370904 |
| GPR183 | -0.137424998 | 0.194221438 | 0.423733065 | NS | 0.711702835 |
| TM9SF2 | 0.180924208 | 0.003479957 | 0.08569675 | Up | 2.458426109 |
| CLYBL | 0.097140928 | 0.247624302 | 0.475813479 | NS | 0.606206735 |
| ZIC5 | 1.037161643 | 0.042233143 | NA | Up | 1.374346595 |
| ZIC2 | 0.842685589 | 0.116376425 | NA | NS | 0.934134988 |
| PCCA | -0.024561057 | 0.725178988 | 0.838197698 | NS | 0.139554788 |
| GGACT | -0.100536416 | 0.213037324 | 0.44285929 | NS | 0.671544302 |
| TMTC4 | -0.00274096 | 0.964164015 | 0.979940943 | NS | 0.015849082 |
| NALCN | 0.531808835 | 0.196294295 | 0.426063609 | NS | 0.707092322 |
| ITGBL1 | 0.831179841 | 0.101909319 | NA | NS | 0.991786101 |
| FGF14 | 0.39231113 | 0.271298419 | 0.496530841 | NS | 0.566552738 |
| TPP2 | -0.062583068 | 0.195119579 | 0.424804449 | NS | 0.70969915 |
| METTL21C | 0.226469131 | 0.145218796 | 0.370531575 | NS | 0.837977169 |
| CCDC168 | 0.358678982 | 0.110483224 | 0.329041981 | NS | 0.956703662 |
| TEX30 | 0.071141298 | 0.472356696 | 0.660918442 | NS | 0.325729924 |
| POGLUT2 | 0.150716875 | 0.368263499 | 0.580219196 | NS | 0.433841324 |
| BIVM | 0.104628744 | 0.150594772 | 0.377054934 | NS | 0.822190103 |
| ERCC5 | 0.059125261 | 0.180025575 | 0.40999858 | NS | 0.744665793 |
| SLC10A2 | 0.284379108 | 0.59985894 | NA | NS | 0.221950864 |
| DAOA | 0.29089185 | 0.630115981 | NA | NS | 0.200579506 |
| EFNB2 | 0.568089739 | 0.044772931 | 0.226305376 | Up | 1.348984477 |
| ARGLU1 | -0.017500509 | 0.789026655 | 0.878322434 | NS | 0.102908325 |
| NALF1 | 0.375888275 | 0.430417374 | 0.627698475 | NS | 0.366110207 |
| LIG4 | -0.032558049 | 0.674791738 | 0.806117594 | NS | 0.170830243 |
| ABHD13 | -0.091412521 | 0.232519168 | 0.46097973 | NS | 0.63354124 |
| TNFSF13B | 0.14135558 | 0.268201085 | 0.493891886 | NS | 0.57153947 |
| MYO16 | 0.249582632 | 0.160285364 | 0.388015316 | NS | 0.795106133 |
| IRS2 | 0.136987994 | 0.233501851 | 0.46187597 | NS | 0.631709672 |
| COL4A1 | 0.423932014 | 0.167639099 | 0.395909177 | NS | 0.775624683 |
| COL4A2 | 0.529872954 | 0.120244491 | 0.340863885 | NS | 0.919934813 |
| RAB20 | -0.205349637 | 0.134133963 | 0.357714894 | NS | 0.872461243 |
| NAXD | -0.123324387 | 0.013890807 | 0.141394243 | Down | 1.857272532 |
| CARS2 | -0.017593455 | 0.731746484 | 0.84116137 | NS | 0.135639356 |
| ING1 | 0.018770658 | 0.696698432 | 0.820222459 | NS | 0.156955167 |
| ANKRD10 | 0.01006242 | 0.84927786 | 0.915233404 | NS | 0.070950198 |
| ARHGEF7 | 0.11819289 | 0.050682354 | 0.238391305 | NS | 1.295143225 |
| TEX29 | 0.016502381 | 0.936536916 | NA | NS | 0.028475099 |
| SOX1 | 0.750717748 | 0.08781383 | NA | NS | 1.05643708 |
| TUBGCP3 | 0.049911303 | 0.268187437 | 0.493891886 | NS | 0.57156157 |
| ATP11A | 0.107073021 | 0.297932171 | 0.519509867 | NS | 0.525882598 |
| MCF2L | 0.14185474 | 0.313629064 | 0.533319352 | NS | 0.503583698 |
| F7 | 0.700350955 | 0.061069293 | NA | NS | 1.214177106 |
| F10 | 0.965077787 | 0.029011392 | NA | Up | 1.537431438 |
| PROZ | 0.540515855 | 0.007965983 | 0.114752972 | Up | 2.098760607 |
| PCID2 | -0.091679151 | 0.084190239 | 0.292034469 | NS | 1.074738259 |
| CUL4A | -0.182585625 | 0.073864138 | 0.276013938 | NS | 1.131566369 |
| LAMP1 | -0.34355688 | 0.002295089 | 0.073928862 | Down | 2.639200415 |
| GRTP1 | -0.031910675 | 0.760517258 | 0.860168677 | NS | 0.118890926 |
| ADPRHL1 | 0.405489298 | 0.084901968 | 0.293013352 | NS | 1.071082243 |
| DCUN1D2 | 0.029253239 | 0.529553806 | 0.704071643 | NS | 0.276089906 |
| TMCO3 | 0.048242848 | 0.468587952 | 0.658017124 | NS | 0.329208882 |
| TFDP1 | -0.126266721 | 0.483112413 | 0.669197484 | NS | 0.315951803 |
| ATP4B | 0.752800534 | 0.052399595 | NA | NS | 1.280672071 |
| TMEM255B | 0.178256111 | 0.290105918 | 0.512990599 | NS | 0.537443412 |
| GAS6 | 0.030959599 | 0.781592485 | 0.873812361 | NS | 0.107019625 |
| C13orf46 | -0.712902811 | 0.033569803 | NA | Down | 1.474051205 |
| RASA3 | -0.148842333 | 0.059789481 | 0.25508876 | NS | 1.223375214 |
| CFAP97D2 | 0.113986714 | 0.656765622 | 0.793958901 | NS | 0.182589588 |
| CDC16 | -0.101385096 | 0.227600387 | 0.45635651 | NS | 0.642827004 |
| UPF3A | -0.072318456 | 0.219520009 | 0.448876282 | NS | 0.658525889 |
| CHAMP1 | 0.291661168 | 3.01E-04 | 0.029045993 | Up | 3.520958284 |
| POTEM | 0.440527591 | 0.420008953 | NA | NS | 0.376741452 |
| POTEG | 0.658870168 | 0.149515052 | NA | NS | 0.825315084 |
| OR4N2 | -0.268284948 | 0.614166603 | NA | NS | 0.211713803 |
| OR4Q3 | 0.099050298 | 0.863979151 | NA | NS | 0.063496738 |
| OR4M1 | 0.291663284 | 0.58466588 | NA | NS | 0.23309225 |
| OR4K2 | 0.032803441 | 0.942789231 | 0.968745091 | NS | 0.025585387 |
| OR4K5 | -0.169085307 | 0.796458248 | NA | NS | 0.098836986 |
| OR4K1 | -0.865070604 | 0.142762159 | NA | NS | 0.845386893 |
| OR4K15 | -0.062509488 | 0.918653367 | NA | NS | 0.036848329 |
| OR4K14 | 1.428533135 | 0.045000974 | NA | Up | 1.346778086 |
| OR4K13 | 0.540903757 | 0.264692221 | NA | NS | 0.577258821 |
| OR4L1 | -0.019215384 | 0.9749724 | NA | NS | 0.011007679 |
| OR4K17 | 0.787194238 | 0.108782547 | NA | NS | 0.963440777 |
| OR4N5 | 0.109716134 | 0.836080284 | NA | NS | 0.077752017 |
| OR11G2 | 0.544820829 | 0.281563191 | NA | NS | 0.550424121 |
| OR11H6 | -0.108582036 | 0.838629587 | NA | NS | 0.076429819 |
| OR11H4 | 0.628502846 | 0.052836209 | NA | NS | 1.277068347 |
| TTC5 | -0.031566431 | 0.468329097 | 0.657895662 | NS | 0.329448859 |
| CCNB1IP1 | -0.176739389 | 0.092053379 | 0.303924681 | NS | 1.035960263 |
| PARP2 | 0.033762367 | 0.578776831 | 0.738612894 | NS | 0.237488862 |
| TEP1 | 0.00229803 | 0.975950191 | 0.986155362 | NS | 0.010572346 |
| KLHL33 | 0.2898057 | 0.286569673 | 0.509714081 | NS | 0.542769771 |
| OSGEP | -0.120858227 | 0.015617144 | 0.148593197 | Down | 1.806398393 |
| APEX1 | -0.19048997 | 0.05661886 | 0.249939307 | NS | 1.247038881 |
| PIP4P1 | -0.100733555 | 0.013883457 | 0.141394243 | Down | 1.857502366 |
| PNP | -0.007873347 | 0.933697842 | 0.963391341 | NS | 0.029793645 |
| RNASE10 | 0.439125889 | 0.409708504 | NA | NS | 0.387525021 |
| RNASE9 | 0.592312665 | 0.270841075 | NA | NS | 0.567285471 |
| RNASE11 | 0.536689615 | 0.466212033 | NA | NS | 0.331416522 |
| OR6S1 | 0.669637939 | 0.238010517 | NA | NS | 0.623403853 |
| ANG | -0.124470725 | 0.412299076 | 0.614504324 | NS | 0.384787638 |
| RNASE4 | 1.084967213 | 0.0421517 | NA | Up | 1.375184907 |
| EDDM3A | 0.855332531 | 0.180649778 | NA | NS | 0.743162568 |
| EDDM3B | 0.774966004 | 0.215935522 | NA | NS | 0.665675909 |
| RNASE6 | 0.105217381 | 0.421846184 | 0.621413862 | NS | 0.374845875 |
| RNASE1 | 0.369248707 | 0.183015201 | NA | NS | 0.737512836 |
| RNASE3 | 0.378822074 | 0.145039902 | 0.37031258 | NS | 0.838512501 |
| RNASE2 | 0.304842209 | 0.125039379 | 0.34652128 | NS | 0.902953191 |
| METTL17 | -0.12474318 | 0.015752848 | 0.148894649 | Down | 1.802640905 |
| SLC39A2 | 0.651311088 | 0.206551094 | NA | NS | 0.684972501 |
| NDRG2 | -0.096559402 | 0.369159461 | 0.58066552 | NS | 0.432785997 |
| TPPP2 | 0.228911443 | 0.630555675 | NA | NS | 0.200276561 |
| RNASE13 | 0.291962904 | 0.545486704 | NA | NS | 0.26321583 |
| RNASE7 | 0.578497987 | 0.207519323 | NA | NS | 0.682941458 |
| RNASE8 | 0.994398228 | 0.004854398 | NA | Up | 2.313864621 |
| ARHGEF40 | 0.127672464 | 0.305706249 | 0.526808466 | NS | 0.514695683 |
| ZNF219 | 0.343173393 | 0.01154085 | 0.131824706 | Up | 1.937762221 |
| TMEM253 | 0.637014808 | 0.023781107 | NA | Up | 1.623767934 |
| OR5AU1 | 0.516987738 | 0.286783404 | 0.509965128 | NS | 0.542445984 |
| HNRNPC | -0.11248011 | 0.048404823 | 0.233382583 | Down | 1.315111359 |
| RPGRIP1 | -0.007290772 | 0.943228148 | 0.968908322 | NS | 0.025383248 |
| SUPT16H | -0.114841314 | 0.022642892 | 0.171563006 | Down | 1.645068105 |
| CHD8 | -0.046561921 | 0.377281357 | 0.587252069 | NS | 0.423334655 |
| RAB2B | -0.106310369 | 0.527149078 | 0.702079431 | NS | 0.278066549 |
| TOX4 | -0.152840686 | 0.004454215 | 0.093587547 | Down | 2.351228849 |
| METTL3 | -0.096569959 | 0.012582368 | 0.135545097 | NS | 1.900237621 |
| SALL2 | 0.504075676 | 0.027869495 | 0.187065309 | Up | 1.554870895 |
| OR10G3 | 0.605035792 | 0.26840106 | NA | NS | 0.571215773 |
| OR10G2 | 0.090901976 | 0.71185019 | NA | NS | 0.147611394 |
| OR4E2 | 0.205409271 | 0.597585861 | NA | NS | 0.223599686 |
| OR4E1 | 0.229279604 | 0.4661282 | NA | NS | 0.331494622 |
| DAD1 | -0.085115226 | 0.257994284 | 0.48474732 | NS | 0.588389916 |
| ABHD4 | 0.088102426 | 0.321606314 | 0.540057273 | NS | 0.492675434 |
| OR6J1 | 0.069211061 | 0.885786588 | NA | NS | 0.0526709 |
| OXA1L | -0.092007618 | 0.083311223 | 0.290675446 | NS | 1.07929649 |
| SLC7A7 | -0.116269947 | 0.248408344 | 0.476110795 | NS | 0.604833821 |
| MRPL52 | -0.079615572 | 0.218158783 | 0.447660331 | NS | 0.661227298 |
| MMP14 | 0.025193003 | 0.890218144 | 0.93792961 | NS | 0.050503558 |
| LRP10 | 0.065947458 | 0.73951564 | 0.847031117 | NS | 0.131052637 |
| REM2 | 0.140614986 | 0.273120183 | 0.49848569 | NS | 0.563646205 |
| RBM23 | -0.05130623 | 0.401813205 | 0.606133976 | NS | 0.395975795 |
| PRMT5 | -0.195103472 | 0.010497905 | 0.127748681 | Down | 1.978897368 |
| HAUS4 | 0.092817023 | 0.357453791 | 0.570623495 | NS | 0.446780093 |
| AJUBA | 0.34278441 | 0.099847179 | 0.314828493 | NS | 1.000664201 |
| C14orf93 | 0.062344877 | 0.220926587 | 0.449641695 | NS | 0.655752016 |
| PSMB5 | -0.123175544 | 0.137856301 | 0.362607036 | NS | 0.86057338 |
| PSMB11 | 0.62770275 | 0.212015536 | NA | NS | 0.673632313 |
| CDH24 | 0.802299407 | 0.005869954 | 0.102935441 | Up | 2.231365304 |
| ACIN1 | 0.022608297 | 0.67630925 | 0.806788244 | NS | 0.169854672 |
| C14orf119 | -0.133066281 | 0.068775523 | 0.268822456 | NS | 1.162566101 |
| LMLN2 | -0.029873687 | 0.864436613 | 0.923382803 | NS | 0.063266847 |
| CEBPE | 0.226196727 | 0.170150839 | 0.398485991 | NS | 0.769165906 |
| SLC7A8 | 0.35111282 | 0.196307013 | 0.426063609 | NS | 0.707064186 |
| RNF212B | 0.319326344 | 0.17385272 | 0.402960258 | NS | 0.75981851 |
| HOMEZ | 0.051411013 | 0.321213174 | 0.539828108 | NS | 0.493206651 |
| PPP1R3E | 0.127160002 | 0.104567041 | 0.320548581 | NS | 0.980605183 |
| BCL2L2 | 0.093206469 | 0.163690744 | 0.391401693 | NS | 0.785975878 |
| BCL2L2-PABPN1 | 0.118334458 | 0.657560278 | NA | NS | 0.18206443 |
| PABPN1 | 0.051213252 | 0.482507276 | 0.668666442 | NS | 0.316496134 |
| SLC22A17 | 0.237714317 | 0.248033195 | 0.476092812 | NS | 0.605490192 |
| EFS | 0.817054511 | 0.112837873 | NA | NS | 0.94754511 |
| IL25 | 1.208413004 | 0.032809312 | NA | Up | 1.484002881 |
| CMTM5 | 0.135767754 | 0.418075067 | 0.618884053 | NS | 0.378745732 |
| MYH6 | 0.71192615 | 0.121274964 | 0.341964938 | NS | 0.916228846 |
| MYH7 | 0.243207563 | 0.61142147 | 0.76220302 | NS | 0.213659315 |
| NGDN | -0.173938594 | 0.0434238 | 0.223481319 | Down | 1.362272174 |
| ZFHX2 | 0.368219036 | 0.157573018 | 0.385109899 | NS | 0.802518148 |
| THTPA | 0.110395839 | 0.284282647 | 0.507138276 | NS | 0.546249649 |
| AP1G2 | -0.020991675 | 0.782082557 | 0.874032361 | NS | 0.1067474 |
| JPH4 | 0.552763567 | 0.049368933 | 0.235159589 | Up | 1.30654626 |
| DHRS2 | 0.385944589 | 0.417137711 | 0.61813574 | NS | 0.379720546 |
| DHRS4 | -0.336071135 | 0.004302264 | 0.092915457 | Down | 2.366302915 |
| DHRS4L2 | -0.128237698 | 0.409840257 | 0.612913775 | NS | 0.387385385 |
| CARMIL3 | 0.120632212 | 0.52282509 | 0.698543855 | NS | 0.281643579 |
| NRL | -0.083567867 | 0.825417562 | NA | NS | 0.083326295 |
| PCK2 | 0.055327284 | 0.501364072 | 0.682856971 | NS | 0.299846791 |
| DCAF11 | -0.114884755 | 0.161555617 | 0.389222254 | NS | 0.791677937 |
| PSME1 | -0.090261606 | 0.366304374 | 0.578598716 | NS | 0.436157895 |
| EMC9 | -0.024421775 | 0.867462631 | 0.925183068 | NS | 0.061749225 |
| PSME2 | 0.105911869 | 0.179473418 | 0.409678085 | NS | 0.745999865 |
| IRF9 | -0.003052126 | 0.98808383 | NA | NS | 0.005206208 |
| REC8 | -0.080161038 | 0.676293982 | 0.806788244 | NS | 0.169864477 |
| TM9SF1 | 0.804503016 | 0.036891808 | NA | Up | 1.43307006 |
| NEDD8 | -0.135009844 | 0.194637683 | 0.424088309 | NS | 0.710773074 |
| GMPR2 | -0.097604613 | 0.240437013 | 0.468398135 | NS | 0.618998676 |
| TINF2 | -0.158718143 | 0.038561724 | 0.213464789 | Down | 1.413843553 |
| TGM1 | -0.119470798 | 0.617290401 | NA | NS | 0.209510476 |
| RABGGTA | 0.082248451 | 0.400067598 | 0.605015064 | NS | 0.397866621 |
| DHRS1 | 0.024813629 | 0.815109514 | 0.894472357 | NS | 0.088784038 |
| NOP9 | -0.21083412 | 0.005608235 | 0.101640588 | Down | 2.251173761 |
| LTB4R | -0.090545972 | 0.558875773 | 0.724151228 | NS | 0.252684716 |
| ADCY4 | 0.020293208 | 0.925841073 | 0.959365989 | NS | 0.033463556 |
| RIPK3 | 0.038219256 | 0.585166524 | 0.744113478 | NS | 0.232720526 |
| NYNRIN | 0.639759894 | 0.065928773 | 0.265127437 | NS | 1.180925008 |
| CBLN3 | 0.43448333 | 0.012481159 | 0.135470856 | Up | 1.90374508 |
| KHNYN | -0.100800686 | 0.049261423 | 0.235025767 | Down | 1.307493048 |
| SDR39U1 | -0.133774213 | 0.083261814 | 0.290591598 | NS | 1.079554131 |
| CMA1 | -0.439070296 | 0.366882409 | NA | NS | 0.435473111 |
| CTSG | 0.051475127 | 0.832147228 | 0.904628723 | NS | 0.079799829 |
| GZMH | -0.271955662 | 0.103431347 | 0.31928753 | NS | 0.985347819 |
| GZMB | -0.352510662 | 0.022539408 | 0.171443155 | Down | 1.647057491 |
| STXBP6 | 0.395401603 | 0.158049837 | 0.385419569 | NS | 0.801205947 |
| NOVA1 | 0.331131846 | 0.407470198 | 0.611075752 | NS | 0.389904149 |
| FOXG1 | 0.734660708 | 0.152866074 | NA | NS | 0.815688889 |
| PRKD1 | 0.249044472 | 0.387127643 | 0.595130169 | NS | 0.412145817 |
| G2E3 | -0.023013388 | 0.662368603 | 0.797602358 | NS | 0.178900262 |
| SCFD1 | -0.109257425 | 0.100284512 | 0.315396306 | NS | 0.998766133 |
| COCH | 0.039719271 | 0.720585539 | 0.835048159 | NS | 0.142314457 |
| STRN3 | -0.314192426 | 0.003165117 | 0.084307443 | Down | 2.499610287 |
| AP4S1 | -0.090109306 | 0.073281223 | 0.275354648 | NS | 1.135007289 |
| HECTD1 | -0.119272843 | 0.007064743 | 0.110895638 | Down | 2.150903656 |
| HEATR5A | 0.003144267 | 0.963777249 | 0.979831825 | NS | 0.01602333 |
| DTD2 | -0.166078484 | 0.14407365 | 0.369286484 | NS | 0.841415441 |
| GPR33 | 0.344802941 | 0.297596154 | NA | NS | 0.526372686 |
| NUBPL | -0.063388084 | 0.301560612 | 0.523092915 | NS | 0.520625385 |
| ARHGAP5 | -0.269231218 | 0.001048477 | 0.052878189 | Down | 2.979441208 |
| AKAP6 | 0.124792809 | 0.382673855 | 0.592041926 | NS | 0.417171209 |
| NPAS3 | 0.735180201 | 0.06832265 | 0.268476763 | NS | 1.165435295 |
| EGLN3 | 0.052179409 | 0.651188922 | 0.789660526 | NS | 0.186292996 |
| SPTSSA | -0.08380266 | 0.436978908 | 0.632837419 | NS | 0.359539525 |
| EAPP | -0.19350425 | 0.045642843 | 0.228134524 | Down | 1.340627312 |
| SNX6 | -0.181575963 | 0.02453471 | 0.177561993 | Down | 1.610219079 |
| CFL2 | -0.025289112 | 0.745562392 | 0.850639911 | NS | 0.127516007 |
| BAZ1A | -0.009034243 | 0.900445602 | 0.944718083 | NS | 0.045542519 |
| SRP54 | -0.142493093 | 0.004419313 | 0.093388262 | Down | 2.354645264 |
| FAM177A1 | 0.059945397 | 0.240104048 | 0.468184257 | NS | 0.619600517 |
| PPP2R3C | 0.007844563 | 0.92529618 | 0.959164117 | NS | 0.033719231 |
| PRORP | -0.081843269 | 0.147803452 | 0.373699795 | NS | 0.830315424 |
| PSMA6 | -0.070521967 | 0.338778214 | 0.554595725 | NS | 0.470084526 |
| NFKBIA | -0.233026957 | 0.061022735 | 0.256948462 | NS | 1.214508329 |
| INSM2 | 0.323975007 | 0.090027732 | 0.300533458 | NS | 1.04562369 |
| RALGAPA1 | 0.018767014 | 0.684778494 | 0.812580159 | NS | 0.164449888 |
| BRMS1L | 0.05159188 | 0.549194679 | 0.717084181 | NS | 0.260273679 |
| MBIP | -0.0462573 | 0.44926463 | 0.642780256 | NS | 0.347497771 |
| SFTA3 | 0.620904397 | 0.18451021 | NA | NS | 0.733979596 |
| NKX2-1 | 1.150138223 | 0.031761238 | NA | Up | 1.498102573 |
| NKX2-8 | 1.147515651 | 0.034581084 | NA | Up | 1.461161393 |
| PAX9 | 0.582501809 | 0.126654144 | 0.348597283 | NS | 0.897380598 |
| SLC25A21 | -0.597556212 | 0.028249885 | 0.188091738 | Down | 1.548983318 |
| MIPOL1 | 0.188098818 | 0.552589413 | 0.719711257 | NS | 0.25759744 |
| FOXA1 | 1.041503302 | 0.033170249 | NA | Up | 1.479251274 |
| TTC6 | 0.585449351 | 0.165597127 | 0.393591001 | NS | 0.780947202 |
| SSTR1 | 0.804467067 | 0.071120847 | NA | NS | 1.148003078 |
| CLEC14A | 1.020293468 | 0.028420955 | NA | Up | 1.54636133 |
| SEC23A | -0.108938794 | 0.106806769 | 0.323513903 | NS | 0.971401223 |
| GEMIN2 | -0.110924494 | 0.249823341 | 0.476907072 | NS | 0.602366989 |
| TRAPPC6B | -0.225215486 | 0.014423098 | 0.143992063 | Down | 1.840941443 |
| PNN | -0.273295648 | 6.55E-04 | 0.04259149 | Down | 3.183887511 |
| MIA2 | 0.012374071 | 0.751109648 | 0.853947238 | NS | 0.12429666 |
| FBXO33 | -0.087885046 | 0.149880866 | 0.376071037 | NS | 0.824253807 |
| LRFN5 | 0.723970353 | 0.130064199 | 0.352095778 | NS | 0.88584223 |
| FSCB | 0.379952696 | 0.552895656 | NA | NS | 0.257356822 |
| C14orf28 | -0.092138608 | 0.15275246 | 0.37932805 | NS | 0.816011786 |
| KLHL28 | -0.004111094 | 0.944409119 | 0.969643039 | NS | 0.024839828 |
| TOGARAM1 | -0.1271963 | 0.022589589 | 0.171534077 | Down | 1.646091664 |
| PRPF39 | -0.1464029 | 0.032970224 | 0.200155801 | Down | 1.481878097 |
| FKBP3 | -0.324412321 | 0.024663536 | 0.177903714 | Down | 1.607944665 |
| FANCM | -0.114853113 | 0.078797734 | 0.283635871 | NS | 1.103486274 |
| MIS18BP1 | -0.261980374 | 7.14E-04 | 0.044011002 | Down | 3.146288263 |
| MDGA2 | 0.486022628 | 0.256021367 | 0.482983139 | NS | 0.591723788 |
| RPS29 | -0.142951646 | 0.426400052 | 0.62458676 | NS | 0.370182751 |
| LRR1 | -0.262808527 | 0.006948696 | 0.110119911 | Down | 2.158096714 |
| RPL36AL | -0.232470656 | 0.037547894 | 0.210352706 | Down | 1.425414421 |
| MGAT2 | 0.459667071 | 0.013623004 | 0.140154175 | Up | 1.865727105 |
| DNAAF2 | -0.094437271 | 0.292865149 | 0.515298984 | NS | 0.533332307 |
| POLE2 | -0.39811689 | 0.013793135 | 0.141148514 | Down | 1.860337017 |
| KLHDC1 | -0.119207241 | 0.218760122 | 0.448245889 | NS | 0.660031844 |
| KLHDC2 | -0.196238903 | 0.012201299 | 0.134278781 | Down | 1.91359394 |
| NEMF | -0.132448893 | 0.141579345 | 0.365923729 | NS | 0.849000101 |
| ARF6 | -0.23364222 | 0.004290521 | 0.092915457 | Down | 2.367489958 |
| VCPKMT | 0.0828081 | 0.186965777 | 0.416429803 | NS | 0.728237882 |
| SOS2 | 0.088187787 | 0.250491716 | 0.477493271 | NS | 0.601206631 |
| L2HGDH | 0.019443847 | 0.776846406 | 0.870202787 | NS | 0.109664839 |
| DMAC2L | -0.021661554 | 0.814024611 | 0.89371005 | NS | 0.089362464 |
| CDKL1 | 0.035322872 | 0.743493011 | 0.849632101 | NS | 0.128723109 |
| MAP4K5 | -0.04475096 | 0.658346981 | 0.794555206 | NS | 0.181545152 |
| ATL1 | 0.024261847 | 0.789759487 | 0.878567629 | NS | 0.102505148 |
| SAV1 | -0.153133308 | 0.04582284 | 0.228442337 | Down | 1.338917998 |
| NIN | -0.031048404 | 0.576598403 | 0.737358732 | NS | 0.239126565 |
| PYGL | 0.041641263 | 0.738526978 | 0.846321984 | NS | 0.131633636 |
| ABHD12B | 0.459862172 | 0.003508177 | 0.085936504 | Up | 2.45491852 |
| TRIM9 | 0.437531055 | 0.007104334 | 0.110938851 | Up | 2.148476606 |
| TMX1 | -0.209332143 | 0.045258617 | 0.227404155 | Down | 1.344298726 |
| FRMD6 | 0.14843231 | 0.47032177 | 0.65935085 | NS | 0.327604918 |
| GNG2 | 0.066395884 | 0.239851305 | 0.467893917 | NS | 0.620057914 |
| RTRAF | -0.117388598 | 0.213419865 | 0.443180155 | NS | 0.67076516 |
| NID2 | 0.196810623 | 0.14193337 | 0.3663829 | NS | 0.847915486 |
| PTGDR | 0.015343607 | 0.886709493 | 0.935955238 | NS | 0.052218642 |
| PTGER2 | -0.201378766 | 0.004989365 | 0.097847413 | Down | 2.301954682 |
| TXNDC16 | -0.401508332 | 3.84E-04 | 0.033652826 | Down | 3.416211361 |
| GPR137C | 0.573642924 | 0.022541918 | 0.171443155 | Up | 1.647009136 |
| ERO1A | 0.122398974 | 0.108714188 | 0.326704032 | NS | 0.963713773 |
| PSMC6 | -0.084653889 | 0.410554786 | 0.613347918 | NS | 0.386628881 |
| STYX | -0.027165751 | 0.656965347 | 0.79397469 | NS | 0.182457538 |
| GNPNAT1 | -0.085636721 | 0.149159064 | 0.375265394 | NS | 0.826350351 |
| FERMT2 | 0.319137511 | 0.157639315 | 0.38513501 | NS | 0.802335462 |
| DDHD1 | 0.083433191 | 0.129082041 | 0.351110762 | NS | 0.889134177 |
| BMP4 | 0.538102105 | 0.178075896 | NA | NS | 0.749394863 |
| CDKN3 | -0.243601539 | 0.219436682 | 0.448847618 | NS | 0.658690771 |
| CNIH1 | 0.016394602 | 0.849069765 | 0.915158582 | NS | 0.071056624 |
| GMFB | -0.078015095 | 0.381447316 | 0.591038759 | NS | 0.418565437 |
| CGRRF1 | -0.080196109 | 0.371310179 | 0.58236492 | NS | 0.430263144 |
| SAMD4A | 0.221228217 | 0.024634022 | 0.177746811 | Up | 1.608464671 |
| GCH1 | -0.057366506 | 0.512302728 | 0.690853034 | NS | 0.290473332 |
| WDHD1 | -0.240838221 | 0.074556741 | 0.277209074 | NS | 1.127513081 |
| SOCS4 | -0.059654804 | 0.346003174 | 0.560904975 | NS | 0.460919917 |
| MAPK1IP1L | 0.149113415 | 0.005020524 | 0.097855352 | Up | 2.299250962 |
| LGALS3 | -0.255479503 | 0.148285821 | 0.374467073 | NS | 0.828900373 |
| DLGAP5 | -0.524768641 | 0.008056339 | 0.115486104 | Down | 2.09386225 |
| FBXO34 | -0.11819293 | 0.028501339 | 0.188709674 | Down | 1.545134734 |
| ATG14 | -0.189391725 | 0.005284276 | 0.099441645 | Down | 2.277014533 |
| TBPL2 | 0.547675739 | 0.245344475 | NA | NS | 0.610223717 |
| KTN1 | -0.085325923 | 0.174259941 | 0.403422181 | NS | 0.758802438 |
| PELI2 | 0.026837819 | 0.747756355 | 0.851783936 | NS | 0.126239887 |
| TMEM260 | 0.151580497 | 0.029895558 | 0.192538825 | Up | 1.52439334 |
| OTX2 | 0.258478069 | 0.589969127 | NA | NS | 0.229170715 |
| EXOC5 | -0.148924842 | 0.012994251 | 0.136762107 | Down | 1.886248737 |
| AP5M1 | -0.030388905 | 0.602482455 | 0.756016283 | NS | 0.220055596 |
| NAA30 | -0.071033348 | 0.19713922 | 0.42687873 | NS | 0.705226965 |
| CCDC198 | 0.443340272 | 0.019230432 | 0.161080521 | Up | 1.716010968 |
| SLC35F4 | 0.268325792 | 0.37523005 | 0.585819023 | NS | 0.425702389 |
| ARMH4 | 0.255841207 | 0.498107615 | 0.68003938 | NS | 0.302676819 |
| ACTR10 | -0.144955074 | 0.022755126 | 0.171982657 | Down | 1.642920764 |
| PSMA3 | 0.050840871 | 0.33741515 | 0.5535843 | NS | 0.471835422 |
| ARID4A | 0.017954749 | 0.743243818 | 0.84943776 | NS | 0.128868694 |
| TOMM20L | 0.316455564 | 0.068801002 | NA | NS | 1.162405239 |
| TIMM9 | 0.021705807 | 0.84603768 | 0.913313927 | NS | 0.072610294 |
| KIAA0586 | -0.080193149 | 0.14025513 | 0.364599659 | NS | 0.853081244 |
| DACT1 | -0.194525558 | 0.306284477 | 0.527408052 | NS | 0.513875014 |
| DAAM1 | 0.048389326 | 0.498473113 | 0.680295049 | NS | 0.302358262 |
| GPR135 | 0.151138714 | 0.181005676 | 0.410963814 | NS | 0.742307807 |
| L3HYPDH | 0.051151052 | 0.460214524 | 0.651531991 | NS | 0.33703968 |
| JKAMP | -0.058600778 | 0.412191163 | 0.614423458 | NS | 0.384901323 |
| CCDC175 | 0.090476312 | 0.773190086 | 0.86784941 | NS | 0.111713723 |
| RTN1 | -0.03382817 | 0.760136436 | 0.860025919 | NS | 0.11910845 |
| LRRC9 | 0.398593143 | 0.10752081 | 0.324805239 | NS | 0.968507471 |
| PCNX4 | 0.023629625 | 0.565733937 | 0.729604932 | NS | 0.247387768 |
| DHRS7 | -0.057822056 | 0.578337536 | 0.73833204 | NS | 0.23781862 |
| PPM1A | -0.043708061 | 0.717975948 | 0.833574225 | NS | 0.143890104 |
| C14orf39 | 0.291686961 | 0.530626569 | NA | NS | 0.275211008 |
| SIX6 | 0.820079915 | 0.071852579 | NA | NS | 1.143557641 |
| SIX1 | 0.854560035 | 0.088525532 | NA | NS | 1.052931457 |
| SIX4 | 0.555129584 | 0.180860063 | NA | NS | 0.742657323 |
| MNAT1 | -0.090754767 | 0.157321865 | 0.384687738 | NS | 0.803210914 |
| TRMT5 | -0.03595427 | 0.568533221 | 0.73140927 | NS | 0.245244154 |
| SLC38A6 | 0.087068817 | 0.226132823 | 0.454636958 | NS | 0.645636396 |
| PRKCH | -0.160333221 | 0.017099404 | 0.153661571 | Down | 1.767019034 |
| TMEM30B | -0.141266691 | 0.273311254 | 0.498634409 | NS | 0.563342485 |
| HIF1A | 0.009906936 | 0.889922367 | 0.937850629 | NS | 0.050647878 |
| SNAPC1 | -0.217669065 | 0.054031798 | 0.245669294 | NS | 1.267350582 |
| SYT16 | 0.393013011 | 0.320997174 | 0.539597569 | NS | 0.493498791 |
| KCNH5 | 0.477297075 | 0.322714665 | 0.541387141 | NS | 0.491181299 |
| RHOJ | 0.624653016 | 0.213814278 | NA | NS | 0.669963298 |
| GPHB5 | 0.871781688 | 0.093189591 | NA | NS | 1.030632596 |
| PPP2R5E | 0.096832148 | 0.063119449 | 0.261328765 | NS | 1.199836801 |
| WDR89 | -0.299963684 | 0.014633143 | 0.144888992 | Down | 1.834662385 |
| SGPP1 | -0.303569789 | 0.005758526 | 0.102313243 | Down | 2.239688641 |
| SYNE2 | 0.00114206 | 0.989435755 | 0.993949367 | NS | 0.004612399 |
| ESR2 | 0.250681341 | 0.0279238 | 0.187199945 | Up | 1.55402548 |
| MTHFD1 | -0.029738735 | 0.517763181 | 0.695146698 | NS | 0.285868836 |
| ZBTB25 | 0.027026229 | 0.633571177 | 0.776709642 | NS | 0.198204588 |
| AKAP5 | 0.043088865 | 0.567125427 | 0.730541221 | NS | 0.246320881 |
| ZBTB1 | -0.140372413 | 0.047135167 | 0.230808123 | Down | 1.326654951 |
| HSPA2 | 0.337999244 | 0.052990175 | 0.24325135 | NS | 1.275804646 |
| PPP1R36 | 0.548596498 | 0.086901989 | NA | NS | 1.060970282 |
| PLEKHG3 | -0.051108531 | 0.562943988 | 0.727118788 | NS | 0.249534814 |
| SPTB | -0.189230363 | 0.411387165 | 0.614050321 | NS | 0.385749263 |
| CHURC1 | -0.079502956 | 0.348332753 | 0.562612118 | NS | 0.458005688 |
| GPX2 | 0.196631617 | 0.146906436 | 0.372544701 | NS | 0.832959178 |
| RAB15 | 0.221357297 | 0.082541374 | 0.289685039 | NS | 1.083328305 |
| FNTB | 0.009687381 | 0.881900897 | 0.933159044 | NS | 0.054580216 |
| MAX | 0.014225877 | 0.829772084 | 0.903248266 | NS | 0.08104118 |
| FUT8 | -0.00937105 | 0.857086997 | 0.919521686 | NS | 0.066975093 |
| CCDC196 | 0.19814505 | 0.129218735 | 0.351188733 | NS | 0.888674516 |
| GPHN | 0.178032992 | 0.073750432 | 0.275940596 | NS | 1.132235433 |
| FAM71D | 0.461861593 | 0.076228144 | 0.279989714 | NS | 1.117884654 |
| PALS1 | 0.009529309 | 0.844791244 | 0.912958148 | NS | 0.073250596 |
| ATP6V1D | -0.12048111 | 0.168066955 | 0.396185854 | NS | 0.774517668 |
| EIF2S1 | -0.250112395 | 0.001510473 | 0.062591307 | Down | 2.820887061 |
| PLEK2 | -0.326945392 | 0.088952846 | 0.299279869 | NS | 1.050840154 |
| TMEM229B | 0.221979482 | 8.01E-05 | 0.013591828 | Up | 4.09626176 |
| PLEKHH1 | 0.109615565 | 0.408972106 | 0.612044462 | NS | 0.388306312 |
| PIGH | -0.114746354 | 0.066852432 | 0.266409327 | NS | 1.174882787 |
| ARG2 | 0.221653614 | 0.002682225 | 0.078552822 | Up | 2.571504868 |
| VTI1B | 0.015496964 | 0.86555068 | 0.924012571 | NS | 0.062707498 |
| RDH11 | -0.236752079 | 7.19E-04 | 0.044131873 | Down | 3.143412222 |
| RDH12 | -0.018672327 | 0.93980534 | NA | NS | 0.026962092 |
| ZFYVE26 | 0.092195487 | 0.157672455 | 0.385174886 | NS | 0.802244172 |
| RAD51B | 0.069902429 | 0.271367278 | 0.496559809 | NS | 0.566442522 |
| ZFP36L1 | 0.083490484 | 0.48623758 | 0.671641319 | NS | 0.313151479 |
| ACTN1 | 0.055490456 | 0.617491154 | 0.766154761 | NS | 0.20936926 |
| DCAF5 | 0.129038047 | 0.01523745 | 0.147394235 | Up | 1.817087717 |
| EXD2 | -0.052373834 | 0.379429461 | 0.589291572 | NS | 0.420868951 |
| GALNT16 | 0.69119363 | 0.051418719 | 0.239542615 | NS | 1.288878751 |
| ERH | -0.332276417 | 0.02160575 | 0.168842295 | Down | 1.665430662 |
| SLC39A9 | -0.065148552 | 0.220475428 | 0.449602156 | NS | 0.656639805 |
| PLEKHD1 | 0.521493836 | 0.079526014 | 0.284900136 | NS | 1.099490784 |
| CCDC177 | 1.203969888 | 0.016828491 | NA | Up | 1.773954836 |
| SUSD6 | 0.285269088 | 0.010640538 | 0.127927347 | Up | 1.973036409 |
| SRSF5 | -0.166873069 | 0.005818943 | 0.102762132 | Down | 2.235155866 |
| SLC10A1 | 0.087071581 | 0.234796712 | 0.462803538 | NS | 0.629307988 |
| SMOC1 | 0.274752175 | 0.453990309 | 0.646396397 | NS | 0.342953418 |
| SLC8A3 | 0.192373462 | 0.398705775 | 0.603654972 | NS | 0.399347474 |
| COX16 | -0.03426378 | 0.722067792 | 0.835993962 | NS | 0.141422026 |
| SYNJ2BP | -0.012693375 | 0.819538336 | 0.897114813 | NS | 0.086430727 |
| ADAM21 | 0.305620701 | 0.050873464 | 0.238726596 | NS | 1.293508689 |
| ADAM20 | 0.123263627 | 0.343677305 | 0.558655521 | NS | 0.463849146 |
| MED6 | -0.0882148 | 0.036236684 | 0.208201842 | NS | 1.440851549 |
| TTC9 | 0.142279379 | 0.107213663 | 0.324231785 | NS | 0.969749866 |
| MAP3K9 | 0.274210539 | 0.044358644 | 0.225362854 | Up | 1.353021736 |
| PCNX1 | 0.168908081 | 0.031682487 | 0.197493826 | Up | 1.499180736 |
| SIPA1L1 | 0.048069451 | 0.529729215 | 0.704071643 | NS | 0.275946075 |
| RGS6 | -0.491559022 | 0.007925386 | 0.11472857 | Down | 2.100979576 |
| DPF3 | -0.058461771 | 0.771254183 | 0.866554152 | NS | 0.112802468 |
| DCAF4 | -0.284780982 | 3.64E-04 | 0.032535375 | Down | 3.439277626 |
| ZFYVE1 | 0.083603437 | 0.315217882 | 0.534573252 | NS | 0.501389153 |
| RBM25 | -0.056118941 | 0.296125391 | 0.51789719 | NS | 0.528524353 |
| PSEN1 | -0.062401476 | 0.320013151 | 0.539024418 | NS | 0.494832173 |
| PAPLN | 0.209524619 | 0.076274284 | 0.279989714 | NS | 1.11762186 |
| NUMB | 0.208638829 | 0.022319305 | 0.17078407 | Up | 1.651319335 |
| HEATR4 | 0.00689735 | 0.939289513 | 0.966553041 | NS | 0.027200526 |
| RIOX1 | -0.226925478 | 0.001403145 | 0.060860925 | Down | 2.852897574 |
| ACOT1 | -0.232016547 | 0.209781324 | 0.439792418 | NS | 0.678233177 |
| ACOT2 | -0.126039643 | 0.107302665 | 0.324372443 | NS | 0.969389493 |
| ACOT4 | -0.149074399 | 0.539098128 | NA | NS | 0.268332176 |
| ACOT6 | 0.772784229 | 0.148013207 | NA | NS | 0.829699532 |
| DNAL1 | -0.071244759 | 0.266984013 | 0.492953536 | NS | 0.573514743 |
| PNMA1 | 0.083738751 | 0.205739089 | 0.435756646 | NS | 0.686683187 |
| MIDEAS | 0.022292614 | 0.82849136 | 0.902454656 | NS | 0.081712016 |
| PTGR2 | 0.129816859 | 0.3853489 | 0.593556772 | NS | 0.414145877 |
| ZNF410 | 0.134080173 | 0.02617136 | 0.182943431 | Up | 1.582173703 |
| FAM161B | 0.059257077 | 0.345293723 | 0.560209467 | NS | 0.461811317 |
| COQ6 | -0.088853668 | 0.15584216 | 0.383185333 | NS | 0.807315041 |
| ENTPD5 | -0.093813083 | 0.461856001 | 0.652766362 | NS | 0.335493409 |
| BBOF1 | -0.302303355 | 0.124487655 | 0.346117593 | NS | 0.904873713 |
| ALDH6A1 | -0.143930398 | 0.050045678 | 0.23675813 | NS | 1.300633427 |
| LIN52 | -0.161628761 | 0.017857194 | 0.156153671 | Down | 1.748186786 |
| VSX2 | 0.555024896 | 0.223258162 | NA | NS | 0.651192655 |
| ABCD4 | -0.081077417 | 0.236703121 | 0.464633596 | NS | 0.625796016 |
| VRTN | 0.858734198 | 0.092376584 | NA | NS | 1.034438102 |
| SYNDIG1L | 0.630098681 | 0.220299169 | NA | NS | 0.656987142 |
| NPC2 | -0.231668166 | 0.063714309 | 0.262207567 | NS | 1.195763021 |
| ISCA2 | -0.191192121 | 0.021715752 | 0.169115127 | Down | 1.663225133 |
| LTBP2 | -0.158432136 | 0.148972656 | 0.375043615 | NS | 0.82689344 |
| AREL1 | 0.015888858 | 0.802766342 | 0.886620118 | NS | 0.095410845 |
| FCF1 | -0.242625903 | 2.75E-04 | 0.027604457 | Down | 3.560959351 |
| YLPM1 | 0.012184918 | 0.877030943 | 0.931079927 | NS | 0.056985084 |
| PROX2 | 0.098562683 | 0.249603259 | 0.47668561 | NS | 0.602749748 |
| DLST | 0.0661125 | 0.052622457 | 0.242513843 | NS | 1.278828879 |
| RPS6KL1 | 0.19896524 | 0.029015823 | 0.190037745 | Up | 1.537365104 |
| PGF | 0.180207949 | 0.492215203 | 0.676245815 | NS | 0.307844977 |
| EIF2B2 | -0.051073943 | 0.345885911 | 0.560812682 | NS | 0.461067128 |
| MLH3 | -0.086487675 | 0.061040025 | 0.256954217 | NS | 1.214385301 |
| ACYP1 | 0.080814222 | 0.337532533 | 0.553636868 | NS | 0.471684361 |
| ZC2HC1C | 0.21439178 | 0.010084785 | 0.126199476 | Up | 1.996333345 |
| NEK9 | 0.034040356 | 0.28270317 | 0.505580821 | NS | 0.548669322 |
| TMED10 | -0.124947068 | 0.097016483 | 0.311260536 | NS | 1.013154474 |
| FOS | -0.210205849 | 0.188229098 | 0.417634451 | NS | 0.725313238 |
| JDP2 | 0.00920378 | 0.918280679 | 0.95488849 | NS | 0.037024553 |
| BATF | -0.010745963 | 0.905423471 | 0.947671876 | NS | 0.043148252 |
| FLVCR2 | 0.072834734 | 0.445295664 | 0.639222847 | NS | 0.351351534 |
| TTLL5 | 0.104740863 | 0.259779642 | 0.485843002 | NS | 0.585394886 |
| ERG28 | -0.140847649 | 0.033349052 | 0.201360398 | Down | 1.476916508 |
| IFT43 | -0.076408916 | 0.302108087 | 0.523602196 | NS | 0.519837649 |
| TGFB3 | 0.244840684 | 0.048148733 | 0.233024927 | Up | 1.317415138 |
| GPATCH2L | 0.075433619 | 0.079023921 | 0.283921514 | NS | 1.102241428 |
| ESRRB | 0.698646483 | 0.071326817 | 0.272521361 | NS | 1.146747158 |
| VASH1 | 0.126545591 | 0.334747102 | 0.55127581 | NS | 0.475283175 |
| ANGEL1 | 0.063277144 | 0.231489846 | 0.45979109 | NS | 0.635468054 |
| LRRC74A | 0.311032676 | 0.344440484 | 0.559498969 | NS | 0.462885809 |
| IRF2BPL | 0.246947972 | 0.023181948 | 0.173122445 | Up | 1.634850065 |
| CIPC | -0.105466982 | 0.117451909 | 0.33737409 | NS | 0.93013992 |
| TMEM63C | 0.232974967 | 0.259341356 | 0.485568555 | NS | 0.586128222 |
| ZDHHC22 | 0.622728903 | 0.20225359 | NA | NS | 0.694103762 |
| NGB | 1.419390777 | 0.01163224 | NA | Up | 1.934336639 |
| POMT2 | 0.129146116 | 0.360295531 | 0.573099615 | NS | 0.443341124 |
| GSTZ1 | -0.026546941 | 0.690707469 | 0.816318285 | NS | 0.160705848 |
| TMED8 | 0.194338001 | 0.010339332 | 0.126897847 | Up | 1.985507517 |
| SAMD15 | 0.347311504 | 0.043472403 | 0.223613314 | Up | 1.361786357 |
| NOXRED1 | 0.150600308 | 0.133633726 | 0.356834322 | NS | 0.874083922 |
| VIPAS39 | 0.024946054 | 0.49483585 | 0.677832837 | NS | 0.305538844 |
| AHSA1 | -0.170245451 | 0.012548755 | 0.135545097 | Down | 1.901399344 |
| ISM2 | 0.90841917 | 0.070788753 | NA | NS | 1.15003574 |
| SPTLC2 | 0.086756569 | 0.246771611 | 0.474843656 | NS | 0.607704803 |
| ALKBH1 | -0.065608476 | 0.172588126 | 0.401378778 | NS | 0.762989086 |
| SLIRP | -0.166949379 | 0.169504384 | 0.397826337 | NS | 0.770819065 |
| SNW1 | -0.117869949 | 0.027746477 | 0.186639939 | Down | 1.556792156 |
| ADCK1 | 0.118603443 | 0.357781414 | 0.570884829 | NS | 0.446382224 |
| NRXN3 | 0.095115505 | 0.679769582 | 0.809193979 | NS | 0.167638273 |
| DIO2 | 0.173019478 | 0.695294606 | 0.81928571 | NS | 0.15783114 |
| CEP128 | -0.032140341 | 0.654460354 | 0.792290225 | NS | 0.184116657 |
| TSHR | 0.262576227 | 0.075025613 | 0.278076808 | NS | 1.124790445 |
| GTF2A1 | 0.02605521 | 0.544928515 | 0.713725092 | NS | 0.263660466 |
| STON2 | -0.012391077 | 0.923370794 | 0.958009674 | NS | 0.034623866 |
| SEL1L | -0.0140238 | 0.838723647 | 0.909109618 | NS | 0.076381112 |
| FLRT2 | 0.563475907 | 0.18572053 | 0.415463304 | NS | 0.731140086 |
| GALC | 0.035965718 | 0.503875533 | 0.684730164 | NS | 0.297676729 |
| GPR65 | -0.075401987 | 0.450227363 | 0.643605891 | NS | 0.346568114 |
| KCNK10 | 0.22690978 | 0.430806547 | 0.627828924 | NS | 0.365717705 |
| SPATA7 | -0.020077939 | 0.828858631 | 0.902682979 | NS | 0.081519536 |
| PTPN21 | 0.48016135 | 0.083737488 | 0.29147602 | NS | 1.077080069 |
| ZC3H14 | -0.122680773 | 0.008878543 | 0.119469085 | Down | 2.051658316 |
| EML5 | -0.027332299 | 0.808764291 | 0.890068227 | NS | 0.092178032 |
| TTC8 | 0.059532224 | 0.562153681 | 0.726507737 | NS | 0.250144941 |
| FOXN3 | -0.032545575 | 0.658974001 | 0.794898116 | NS | 0.181131719 |
| EFCAB11 | 0.018205623 | 0.887660218 | 0.936581465 | NS | 0.051753243 |
| TDP1 | -0.089055647 | 0.105887395 | 0.322185601 | NS | 0.975155736 |
| KCNK13 | -0.030775096 | 0.824894322 | 0.900263116 | NS | 0.083601686 |
| PSMC1 | -0.043020152 | 0.471555868 | 0.660409087 | NS | 0.326466846 |
| NRDE2 | 0.071809444 | 0.157166411 | 0.384512887 | NS | 0.803640265 |
| CALM1 | -0.044271934 | 0.39455682 | 0.600288335 | NS | 0.403890445 |
| TTC7B | 0.316790623 | 0.06668497 | 0.266279415 | NS | 1.175972041 |
| RPS6KA5 | 0.045381675 | 0.534176959 | 0.706631279 | NS | 0.272314849 |
| DGLUCY | -0.046710832 | 0.530524101 | 0.704623132 | NS | 0.275294882 |
| GPR68 | -0.220969975 | 0.052606335 | 0.242513843 | NS | 1.278961957 |
| CCDC88C | -0.08634903 | 0.338552459 | 0.554372563 | NS | 0.470374027 |
| PPP4R3A | -0.121720185 | 0.007077159 | 0.110899425 | Down | 2.15014103 |
| CATSPERB | -0.374797848 | 0.004888679 | 0.096834757 | Down | 2.310808489 |
| TC2N | -0.072447452 | 0.348080491 | 0.562578646 | NS | 0.458320317 |
| FBLN5 | 0.076025545 | 0.614363268 | 0.764059058 | NS | 0.211574758 |
| TRIP11 | -0.182370669 | 0.004304572 | 0.092915457 | Down | 2.36607006 |
| ATXN3 | -0.198691181 | 3.23E-04 | 0.030244481 | Down | 3.491418098 |
| NDUFB1 | -0.084813253 | 0.464026038 | 0.654670332 | NS | 0.333457649 |
| CPSF2 | -0.137911031 | 0.012040521 | 0.13403215 | Down | 1.91935471 |
| SLC24A4 | 0.412843701 | 3.25E-04 | 0.030251999 | Up | 3.488184227 |
| RIN3 | 0.168678198 | 0.236828522 | 0.464700343 | NS | 0.625565995 |
| LGMN | 0.117693772 | 0.298728835 | 0.520146566 | NS | 0.524722855 |
| GOLGA5 | -0.059207071 | 0.154491738 | 0.381395414 | NS | 0.81109474 |
| ITPK1 | 0.13265664 | 0.186856192 | 0.416322648 | NS | 0.728492507 |
| MOAP1 | 0.143109991 | 0.32036349 | 0.539086307 | NS | 0.494356984 |
| TMEM251 | -0.318852281 | 0.273814746 | NA | NS | 0.562543167 |
| GON7 | -0.219146288 | 0.317527622 | 0.536957661 | NS | 0.498218489 |
| UBR7 | -0.139353988 | 0.113569809 | 0.332228782 | NS | 0.944737106 |
| BTBD7 | 0.05227733 | 0.485477158 | 0.671360296 | NS | 0.313831199 |
| ASB2 | 0.176884334 | 0.248405942 | 0.476110795 | NS | 0.604838021 |
| DDX24 | 0.031501429 | 0.592828898 | 0.749346361 | NS | 0.227070634 |
| IFI27L1 | -0.172487001 | 0.395059261 | 0.600626238 | NS | 0.403337754 |
| IFI27 | 0.286161711 | 0.284645837 | 0.507588489 | NS | 0.545695163 |
| IFI27L2 | -0.294803319 | 0.068180829 | 0.268476763 | NS | 1.166337726 |
| PPP4R4 | 0.022993055 | 0.902052851 | 0.9454453 | NS | 0.044768016 |
| SERPINA1 | 0.482711844 | 1.01E-04 | 0.015913 | Up | 3.996753347 |
| SERPINA11 | 0.626341238 | 0.044701488 | NA | Up | 1.349678022 |
| SERPINA9 | 0.569832135 | 0.147125578 | NA | NS | 0.832311818 |
| SERPINA12 | 0.48637797 | 0.338243494 | NA | NS | 0.470770548 |
| SERPINA4 | 0.972382002 | 0.082477237 | NA | NS | 1.083665897 |
| SERPINA5 | 0.503374811 | 0.287063998 | NA | NS | 0.542021271 |
| SERPINA3 | 0.266369565 | 0.617667936 | NA | NS | 0.209244943 |
| GSC | 0.80123547 | 0.045475605 | NA | Up | 1.34222151 |
| DICER1 | 0.011825284 | 0.869442187 | 0.926426506 | NS | 0.060759291 |
| CLMN | 0.209364395 | 0.019461917 | 0.161936818 | Up | 1.710814375 |
| SYNE3 | -0.072770485 | 0.328785898 | 0.546433799 | NS | 0.483086818 |
| GLRX5 | -0.09904865 | 0.628259549 | 0.773528315 | NS | 0.201860902 |
| TCL1B | 0.109361439 | 0.702137916 | NA | NS | 0.153577574 |
| TCL1A | -0.249376845 | 0.159351451 | 0.387047082 | NS | 0.797643976 |
| TUNAR | 0.586719144 | 0.063755286 | NA | NS | 1.195483802 |
| C14orf132 | 0.133311173 | 0.41133535 | 0.614050321 | NS | 0.385803965 |
| BDKRB2 | 0.95802556 | 0.026258549 | NA | Up | 1.580729273 |
| BDKRB1 | 0.679153427 | 0.222229407 | NA | NS | 0.653198473 |
| ATG2B | 0.046711524 | 0.399259655 | 0.604289507 | NS | 0.398744573 |
| GSKIP | -0.149388606 | 0.154475724 | 0.381395414 | NS | 0.811139762 |
| AK7 | 0.310997261 | 0.279339922 | 0.503278325 | NS | 0.553866992 |
| PAPOLA | -0.054310251 | 0.450808307 | 0.643924649 | NS | 0.34600809 |
| VRK1 | -0.345389704 | 0.001751855 | 0.065523229 | Down | 2.756501909 |
| BCL11B | -0.031317605 | 0.680418216 | 0.809629448 | NS | 0.167224068 |
| SETD3 | -0.403243756 | 2.62E-04 | 0.026772493 | Down | 3.58193658 |
| CCNK | 0.071201536 | 0.241989853 | 0.469918053 | NS | 0.616202844 |
| CCDC85C | 0.170076512 | 0.037777823 | 0.211382288 | Up | 1.422763071 |
| HHIPL1 | 0.542251705 | 0.075582064 | 0.278875532 | NS | 1.121581252 |
| CYP46A1 | 0.567179892 | 0.063302225 | 0.261404175 | NS | 1.198581025 |
| EML1 | 0.716947873 | 0.084892492 | 0.293013352 | NS | 1.071130719 |
| EVL | 0.01408425 | 0.839559039 | 0.909492508 | NS | 0.075948758 |
| DEGS2 | 0.151916036 | 0.184268924 | 0.414428646 | NS | 0.7345479 |
| YY1 | -0.1150506 | 0.081045131 | 0.287057145 | NS | 1.091273072 |
| SLC25A29 | -0.001750673 | 0.988183587 | 0.993039952 | NS | 0.005162364 |
| SLC25A47 | 1.242635376 | 0.003460704 | NA | Up | 2.460835597 |
| WARS1 | -0.187300372 | 0.123347901 | 0.34408205 | NS | 0.908868236 |
| WDR25 | 0.015866243 | 0.839479707 | 0.909492508 | NS | 0.075989798 |
| BEGAIN | 0.529242714 | 0.048217722 | 0.233118485 | Up | 1.316793313 |
| DLK1 | 0.617558348 | 0.1537599 | 0.380446113 | NS | 0.813156913 |
| RTL1 | 0.42136931 | 0.308689475 | NA | NS | 0.510478178 |
| DIO3 | 0.713740064 | 0.140530514 | NA | NS | 0.852229364 |
| PPP2R5C | -0.085568731 | 0.084953311 | 0.293144603 | NS | 1.07081969 |
| DYNC1H1 | 0.003245651 | 0.965045666 | 0.980465179 | NS | 0.015452135 |
| HSP90AA1 | -0.103003294 | 0.313753949 | 0.53333281 | NS | 0.503410799 |
| WDR20 | -0.136639102 | 0.002588778 | 0.077810428 | Down | 2.586905206 |
| MOK | 0.113290495 | 0.526624594 | 0.701573873 | NS | 0.278498863 |
| ZNF839 | -0.10748231 | 0.044463142 | 0.225635911 | Down | 1.351999855 |
| CINP | -0.082901302 | 0.112371581 | 0.330929727 | NS | 0.94934351 |
| TECPR2 | 0.104834956 | 0.372217492 | 0.583052983 | NS | 0.429203222 |
| ANKRD9 | 0.122749613 | 0.513069744 | 0.69139884 | NS | 0.289823595 |
| RCOR1 | 0.141086465 | 0.08461483 | 0.292637999 | NS | 1.072553516 |
| TRAF3 | 0.018210646 | 0.710841305 | 0.829073517 | NS | 0.148227344 |
| AMN | 0.53208491 | 0.01614897 | 0.150281882 | Up | 1.791855172 |
| CDC42BPB | 0.02328095 | 0.855952375 | 0.918905981 | NS | 0.067550399 |
| LBHD2 | 0.961120155 | 0.091361062 | NA | NS | 1.039238859 |
| EXOC3L4 | 1.026550023 | 0.020396519 | NA | Up | 1.690443951 |
| TNFAIP2 | 0.092836709 | 0.426112273 | 0.624284262 | NS | 0.370475957 |
| EIF5 | -0.111272471 | 0.044107565 | 0.224627854 | Down | 1.355486914 |
| MARK3 | -0.036183379 | 0.694255457 | 0.818482368 | NS | 0.158480698 |
| CKB | 0.254762487 | 0.085291304 | 0.293565942 | NS | 1.069095248 |
| TRMT61A | 5.72E-04 | 0.994512627 | 0.996819475 | NS | 0.002389698 |
| BAG5 | -0.104085251 | 0.050981715 | 0.238791409 | NS | 1.292585562 |
| KLC1 | 0.184754218 | 0.007935344 | 0.11472857 | Up | 2.100434236 |
| COA8 | -0.103154562 | 0.166215641 | 0.394228522 | NS | 0.779328111 |
| XRCC3 | 0.201416536 | 0.046459393 | 0.229461508 | Up | 1.33292647 |
| ZFYVE21 | -0.108290274 | 0.10337372 | 0.31928753 | NS | 0.985589857 |
| PPP1R13B | 0.138575631 | 0.142155911 | 0.366669071 | NS | 0.847235076 |
| ATP5MJ | -0.172445633 | 0.283365732 | 0.506330811 | NS | 0.547652671 |
| TDRD9 | -0.129659165 | 0.379295795 | 0.589203845 | NS | 0.421021972 |
| RD3L | 0.323747671 | 0.180440528 | NA | NS | 0.74366591 |
| ASPG | 0.747136276 | 0.048667549 | 0.233910643 | Up | 1.312760523 |
| KIF26A | 0.833524837 | 0.006182086 | 0.105138876 | Up | 2.208864934 |
| INF2 | 0.187330302 | 0.266561234 | 0.492480267 | NS | 0.574203009 |
| ADSS1 | 0.149984777 | 0.419796222 | 0.620029219 | NS | 0.376961474 |
| SIVA1 | -0.067241799 | 0.335729617 | 0.551832552 | NS | 0.474010345 |
| AKT1 | -0.08697627 | 0.150011723 | 0.376171504 | NS | 0.823874802 |
| ZBTB42 | 0.396169319 | 0.002644965 | 0.078363492 | Up | 2.577580138 |
| CEP170B | 0.729742643 | 0.02764898 | 0.186639939 | Up | 1.55832089 |
| PLD4 | 0.051877528 | 0.675542754 | 0.806509938 | NS | 0.17034716 |
| AHNAK2 | 0.340913398 | 0.043977575 | 0.224627854 | Up | 1.356768718 |
| CLBA1 | 0.351990362 | 0.015466093 | 0.147951473 | Up | 1.810619383 |
| CDCA4 | -0.125265483 | 0.117552578 | 0.337535326 | NS | 0.929767844 |
| GPR132 | 0.011762125 | 0.810667569 | 0.891306225 | NS | 0.0911572 |
| JAG2 | 0.542854918 | 0.042958201 | 0.222434711 | Up | 1.366953912 |
| NUDT14 | 1.84E-04 | 0.998779217 | 0.999521155 | NS | 5.31E-04 |
| BRF1 | 0.021750303 | 0.780258646 | 0.872874342 | NS | 0.10776141 |
| BTBD6 | -0.187180838 | 0.001870246 | 0.067487067 | Down | 2.728101163 |
| PACS2 | 0.088249069 | 0.411517631 | 0.614058952 | NS | 0.385611553 |
| TEX22 | 0.453468738 | 1.29E-04 | 0.017955842 | Up | 3.889757311 |
| MTA1 | 0.185745014 | 0.126161378 | 0.347987405 | NS | 0.899073575 |
| CRIP2 | 0.143999491 | 0.326816096 | 0.54501873 | NS | 0.485696563 |
| CRIP1 | 0.028302049 | 0.785964322 | 0.876321892 | NS | 0.104597168 |
| TEDC1 | 0.106005618 | 0.370531301 | 0.581645533 | NS | 0.431175098 |
| TMEM121 | 0.457797436 | 0.033395793 | NA | Up | 1.476308243 |
| GOLGA6L6 | 0.757227501 | 0.417235518 | NA | NS | 0.379618729 |
| POTEB3 | 1.000271524 | 0.089775242 | NA | NS | 1.046843414 |
| GOLGA6L22 | 0.71136134 | 0.183728472 | NA | NS | 0.735823537 |
| NIPA1 | -0.050592386 | 0.682382267 | 0.810825369 | NS | 0.165972268 |
| NIPA2 | -0.117791059 | 0.138001833 | 0.362607036 | NS | 0.860115144 |
| CYFIP1 | -0.104715832 | 0.359917668 | 0.572764191 | NS | 0.443796833 |
| TUBGCP5 | -0.274613313 | 0.001845888 | 0.066996088 | Down | 2.733794608 |
| GOLGA8S | 0.541743457 | 0.404128356 | NA | NS | 0.393480675 |
| GOLGA6L2 | -0.262281885 | 0.77109899 | NA | NS | 0.112889866 |
| NPAP1 | 0.379598378 | 0.439996055 | 0.635045158 | NS | 0.356551218 |
| SNRPN | 0.04749447 | 0.44502457 | 0.639053249 | NS | 0.35161601 |
| SNURF | 0.128990115 | 0.224893494 | 0.453236933 | NS | 0.648023108 |
| UBE3A | -0.096566254 | 0.041217027 | 0.218659333 | NS | 1.384923342 |
| ATP10A | 0.052646396 | 0.505649997 | 0.686001791 | NS | 0.296149991 |
| GABRB3 | 0.373181532 | 0.303793721 | 0.524974254 | NS | 0.517421207 |
| GABRA5 | 1.078207636 | 0.045424345 | NA | Up | 1.342711322 |
| GABRG3 | 0.542595493 | 0.257149087 | 0.484152285 | NS | 0.589815013 |
| OCA2 | -0.045782439 | 0.907011683 | NA | NS | 0.042387119 |
| HERC2 | -0.06672682 | 0.470925023 | 0.659963583 | NS | 0.327048232 |
| GOLGA8M | -0.305708064 | 0.312917807 | NA | NS | 0.504569722 |
| GOLGA6L7 | 0.365193924 | 0.353247611 | NA | NS | 0.451920766 |
| APBA2 | -0.067901231 | 0.523849403 | 0.699504346 | NS | 0.280793546 |
| FAM189A1 | 1.209868976 | 0.01825265 | NA | Up | 1.738674085 |
| NSMCE3 | 0.369954004 | 0.5478279 | NA | NS | 0.261355854 |
| CHRFAM7A | 0.525028753 | 0.217440393 | NA | NS | 0.662659776 |
| GOLGA8R | -0.279067434 | 0.232846419 | 0.461193892 | NS | 0.632930437 |
| GOLGA8Q | 0.245810927 | 0.442058235 | NA | NS | 0.354520514 |
| GOLGA8H | -0.013665896 | 0.942121933 | NA | NS | 0.025892885 |
| FAN1 | -0.281561224 | 2.62E-04 | 0.026772493 | Down | 3.58237812 |
| MTMR10 | -0.056201465 | 0.456793256 | 0.648411996 | NS | 0.340280316 |
| TRPM1 | 0.671397479 | 0.219788551 | NA | NS | 0.657994934 |
| KLF13 | 0.134645951 | 0.423091954 | 0.62248824 | NS | 0.373565233 |
| OTUD7A | 0.396002917 | 0.133057295 | NA | NS | 0.87596131 |
| CHRNA7 | 1.30622576 | 0.011035195 | NA | Up | 1.957219983 |
| GOLGA8K | 0.363368781 | 0.174264194 | NA | NS | 0.758791839 |
| GOLGA8O | 0.014374714 | 0.93963159 | 0.96670501 | NS | 0.027042391 |
| GOLGA8N | 0.18545534 | 0.415812345 | NA | NS | 0.381102621 |
| ARHGAP11A | -0.56224149 | 0.010909596 | 0.128723113 | Down | 1.96219134 |
| FMN1 | 0.178507944 | 0.367771197 | 0.579795963 | NS | 0.434422287 |
| RYR3 | 0.184588828 | 0.228213448 | 0.456877344 | NS | 0.641658768 |
| AVEN | -0.039860854 | 0.589150783 | 0.747064461 | NS | 0.229773541 |
| CHRM5 | 0.016769581 | 0.899704226 | 0.944275064 | NS | 0.04590024 |
| EMC7 | -0.138960037 | 0.092258018 | 0.304276084 | NS | 1.03499588 |
| PGBD4 | 0.049161104 | 0.588678641 | 0.746796557 | NS | 0.230121721 |
| KATNBL1 | 0.05600015 | 0.551260671 | 0.718687588 | NS | 0.258642991 |
| EMC4 | -0.179048587 | 0.021114961 | 0.168202484 | Down | 1.675409719 |
| SLC12A6 | 0.169065464 | 0.055649716 | 0.248695897 | NS | 1.254537047 |
| NOP10 | -0.084366514 | 0.454753046 | 0.647080104 | NS | 0.342224383 |
| NUTM1 | 0.389378574 | 0.132112606 | 0.355039063 | NS | 0.879055742 |
| LPCAT4 | -0.073957826 | 0.375214608 | 0.585819023 | NS | 0.425720262 |
| GOLGA8A | -0.005439212 | 0.9612814 | 0.978517825 | NS | 0.017149461 |
| GOLGA8B | -0.026784354 | 0.813734532 | 0.893529505 | NS | 0.089517254 |
| GJD2 | 0.72119441 | 0.107988127 | NA | NS | 0.966623991 |
| ACTC1 | 0.737483944 | 0.132414069 | NA | NS | 0.878065867 |
| AQR | -0.063295907 | 0.110787916 | 0.32927273 | NS | 0.955507605 |
| ZNF770 | -0.09853757 | 0.127226105 | 0.348830209 | NS | 0.895423767 |
| NANOGP8 | 1.013868253 | 0.071543007 | NA | NS | 1.14543281 |
| DPH6 | -0.004499725 | 0.946821218 | 0.971427419 | NS | 0.023732018 |
| CDIN1 | 0.079376634 | 0.30667644 | 0.527638781 | NS | 0.513319587 |
| MEIS2 | 0.460217257 | 0.136963612 | 0.361500594 | NS | 0.863394798 |
| TMCO5A | 0.557141637 | 0.210307671 | 0.440268138 | NS | 0.677144887 |
| SPRED1 | -0.164386264 | 0.087301737 | 0.296859543 | NS | 1.058977115 |
| FAM98B | 0.09735093 | 0.174121295 | 0.403371019 | NS | 0.759148112 |
| RASGRP1 | 0.020703076 | 0.725548003 | 0.838412653 | NS | 0.139333849 |
| THBS1 | 0.060464831 | 0.714335517 | 0.831075885 | NS | 0.146097756 |
| FSIP1 | 0.047540238 | 0.749117577 | 0.852613594 | NS | 0.125450013 |
| GPR176 | 0.61634103 | 0.047810959 | NA | Up | 1.320472544 |
| EIF2AK4 | -0.071400067 | 0.116905127 | 0.336721001 | NS | 0.932166442 |
| SRP14 | -0.112528994 | 0.353745144 | 0.567005458 | NS | 0.451309513 |
| BMF | 0.203745372 | 0.017377124 | 0.154692513 | Up | 1.760022092 |
| BUB1B | -0.012505811 | 0.939648076 | 0.96670501 | NS | 0.027034771 |
| PAK6 | 0.610646654 | 0.055528641 | 0.248604264 | NS | 1.255482957 |
| PLCB2 | -0.052788414 | 0.658184641 | 0.794520233 | NS | 0.181652256 |
| ANKRD63 | 0.696755816 | 0.055748287 | NA | NS | 1.253768473 |
| INAFM2 | -0.092471872 | 0.297654262 | 0.519257109 | NS | 0.526287895 |
| CCDC9B | 0.360491415 | 0.028695261 | 0.189026296 | Up | 1.542189813 |
| PHGR1 | 0.073743062 | 0.770991997 | NA | NS | 0.11295013 |
| DISP2 | 0.191450541 | 0.209291404 | 0.439218521 | NS | 0.679248609 |
| KNSTRN | -0.290918702 | 0.001408449 | 0.060860925 | Down | 2.851258991 |
| IVD | 0.075342831 | 0.202825101 | 0.432901167 | NS | 0.692878299 |
| BAHD1 | -0.038666311 | 0.57541093 | 0.736492546 | NS | 0.240021893 |
| CHST14 | -0.009088658 | 0.924698873 | 0.958933826 | NS | 0.033999672 |
| CCDC32 | 0.024738614 | 0.701573598 | 0.823126974 | NS | 0.153926763 |
| RPUSD2 | -0.228985152 | 0.012707472 | 0.135824642 | Down | 1.895940847 |
| KNL1 | -0.327666368 | 0.016629471 | 0.152056591 | Down | 1.779121565 |
| RAD51 | 0.093880403 | 0.47945104 | 0.666617505 | NS | 0.319255735 |
| RMDN3 | -0.134590158 | 0.027477591 | 0.186246285 | Down | 1.561021343 |
| GCHFR | 0.389105022 | 0.005311786 | 0.099529109 | Up | 2.274759396 |
| DNAJC17 | -0.129526994 | 0.034856128 | 0.204782731 | Down | 1.457720853 |
| C15orf62 | 0.236965657 | 0.028011286 | 0.187577424 | Up | 1.55266695 |
| ZFYVE19 | -0.053638192 | 0.321286234 | 0.539845732 | NS | 0.493107882 |
| PPP1R14D | 0.326148667 | 0.419598865 | NA | NS | 0.377165695 |
| SPINT1 | -0.146877484 | 0.212089843 | 0.441812042 | NS | 0.673480129 |
| RHOV | 0.816740198 | 0.087342916 | NA | NS | 1.058772315 |
| VPS18 | -0.204557421 | 0.008232351 | 0.116684441 | Down | 2.08447614 |
| DLL4 | 0.830778261 | 0.043264036 | 0.223075426 | Up | 1.36387297 |
| CHAC1 | 0.566958561 | 0.096216495 | NA | NS | 1.01675047 |
| INO80 | -0.027206841 | 0.581516044 | 0.740664058 | NS | 0.235438299 |
| EXD1 | 0.62433543 | 0.192843901 | NA | NS | 0.714794092 |
| CHP1 | -0.017055811 | 0.877049483 | 0.931079927 | NS | 0.056975903 |
| OIP5 | 0.198615995 | 0.220123076 | 0.449270199 | NS | 0.657334426 |
| NUSAP1 | -0.152441887 | 0.554626358 | 0.721447662 | NS | 0.255999495 |
| NDUFAF1 | -0.106054982 | 0.166833675 | 0.394803788 | NS | 0.777716282 |
| RTF1 | 0.022771189 | 0.601329851 | 0.755149216 | NS | 0.220887236 |
| ITPKA | 1.155996378 | 0.00157431 | NA | Up | 2.802909821 |
| LTK | -0.270052225 | 0.041467958 | 0.219431417 | Down | 1.382287351 |
| RPAP1 | -0.103226349 | 0.335397528 | 0.551701439 | NS | 0.474440143 |
| TYRO3 | 0.595066939 | 0.114044477 | 0.33250813 | NS | 0.942925742 |
| MGA | -0.108046858 | 0.02555827 | 0.180994279 | Down | 1.592468547 |
| MAPKBP1 | -0.049016688 | 0.573197264 | 0.734733139 | NS | 0.241695891 |
| JMJD7 | 0.166905203 | 0.262796324 | 0.488909438 | NS | 0.580380714 |
| JMJD7-PLA2G4B | 0.007226173 | 0.943020572 | 0.968789986 | NS | 0.025478833 |
| PLA2G4B | -0.312428856 | 0.093000133 | 0.305431813 | NS | 1.031516431 |
| SPTBN5 | 0.085044323 | 0.543506137 | 0.713114152 | NS | 0.264795548 |
| EHD4 | -0.105368922 | 0.17679889 | 0.406510431 | NS | 0.752520466 |
| PLA2G4E | 0.559163247 | 0.259441909 | NA | NS | 0.585959869 |
| PLA2G4D | 0.942736115 | 0.036015305 | NA | Up | 1.4435129 |
| PLA2G4F | 0.520912249 | 0.282879646 | NA | NS | 0.548398301 |
| VPS39 | 0.10839046 | 0.072507279 | 0.27406531 | NS | 1.13961839 |
| TMEM87A | -0.130388664 | 0.002703124 | 0.078862345 | Down | 2.568134065 |
| GANC | 0.092090899 | 0.042180807 | 0.220751111 | NS | 1.374885113 |
| CAPN3 | 0.167398497 | 0.103501251 | 0.319326135 | NS | 0.9850544 |
| ZNF106 | 0.001142313 | 0.987663031 | 0.992821785 | NS | 0.005391202 |
| SNAP23 | -0.006059015 | 0.932436093 | 0.962852453 | NS | 0.030380924 |
| LRRC57 | 0.004176848 | 0.929683299 | 0.961680529 | NS | 0.031664971 |
| HAUS2 | -0.236932499 | 0.003237386 | 0.084541185 | Down | 2.489805485 |
| STARD9 | 0.037923208 | 0.700838473 | 0.822549366 | NS | 0.154382065 |
| CDAN1 | -0.135884281 | 0.123689048 | 0.344659912 | NS | 0.907668754 |
| TTBK2 | 0.037742124 | 0.289915195 | 0.512990599 | NS | 0.537729022 |
| UBR1 | -0.086255604 | 0.039154593 | 0.214525956 | NS | 1.407217282 |
| TMEM62 | 0.024750793 | 0.545674155 | 0.714320063 | NS | 0.263066616 |
| CCNDBP1 | 0.034532836 | 0.726345373 | 0.838611255 | NS | 0.138856825 |
| EPB42 | -0.053446202 | 0.768173495 | 0.864804079 | NS | 0.114540682 |
| TGM5 | 0.460039929 | 0.304499468 | NA | NS | 0.516413461 |
| TGM7 | 0.578799384 | 0.275007991 | NA | NS | 0.560654687 |
| LCMT2 | -0.002371227 | 0.9781257 | 0.987221135 | NS | 0.00960533 |
| ADAL | -0.243344989 | 0.052375923 | 0.241847979 | NS | 1.280868314 |
| ZSCAN29 | 0.094907615 | 0.034663698 | 0.204304637 | NS | 1.460125103 |
| TUBGCP4 | 0.110902205 | 0.098734445 | 0.313587396 | NS | 1.00553131 |
| TP53BP1 | -0.036917056 | 0.418763066 | 0.619102172 | NS | 0.378031629 |
| MAP1A | 0.158525702 | 0.400450297 | 0.605341375 | NS | 0.39745138 |
| PPIP5K1 | -0.064819497 | 0.301919626 | 0.523434011 | NS | 0.520108654 |
| CKMT1B | 0.888783817 | 0.08956208 | NA | NS | 1.047875827 |
| STRC | 0.711411204 | 0.09778 | NA | NS | 1.009749965 |
| CATSPER2 | 0.141227471 | 0.296489193 | 0.518137772 | NS | 0.527991131 |
| CKMT1A | -0.04896983 | 0.93109212 | NA | NS | 0.031007349 |
| PDIA3 | 0.086526144 | 0.15445588 | 0.381395414 | NS | 0.811195554 |
| ELL3 | 0.529651366 | 0.063708671 | NA | NS | 1.195801454 |
| SERF2 | -0.299773835 | 0.004026226 | 0.090296145 | Down | 2.395101877 |
| HYPK | 0.173444666 | 0.048898306 | 0.23437492 | Up | 1.310706187 |
| MFAP1 | -0.085292454 | 0.124611004 | 0.346180975 | NS | 0.904443606 |
| WDR76 | -0.089175457 | 0.163273591 | 0.391057496 | NS | 0.787084056 |
| FRMD5 | 0.152129641 | 0.18315566 | 0.413453601 | NS | 0.737179656 |
| GOLM2 | -0.11484038 | 0.074006586 | 0.276267127 | NS | 1.130729632 |
| CTDSPL2 | -0.069788398 | 0.118890096 | 0.339503863 | NS | 0.924854322 |
| EIF3J | -0.138980131 | 0.10873016 | 0.326704032 | NS | 0.963649971 |
| SPG11 | 0.02785543 | 0.520583051 | 0.696688569 | NS | 0.283509976 |
| PATL2 | 0.149981625 | 0.125470845 | 0.347087003 | NS | 0.901457177 |
| B2M | -0.044613031 | 0.749237281 | 0.85265413 | NS | 0.125380621 |
| TRIM69 | 0.035211948 | 0.666976109 | 0.800832906 | NS | 0.175889722 |
| TERB2 | 0.56136806 | 0.020188689 | 0.164578764 | Up | 1.694891878 |
| SORD | 0.298969291 | 0.003792199 | 0.088644036 | Up | 2.421108905 |
| DUOX2 | 0.488611551 | 0.21975089 | 0.449082183 | NS | 0.658069357 |
| DUOXA2 | 1.267386261 | 0.012767438 | NA | Up | 1.893896235 |
| DUOXA1 | 0.542820687 | 0.172726655 | NA | NS | 0.762640636 |
| DUOX1 | 0.085265349 | 0.517396798 | 0.694817724 | NS | 0.286176263 |
| SHF | 0.035909977 | 0.773028465 | 0.867838133 | NS | 0.111804514 |
| SLC28A2 | 0.524144612 | 0.090616349 | 0.301422749 | NS | 1.04279344 |
| GATM | -0.071559063 | 0.654731381 | 0.79248518 | NS | 0.183936843 |
| SPATA5L1 | 0.013323641 | 0.835004363 | 0.906831844 | NS | 0.078311255 |
| C15orf48 | 0.269207278 | 0.369589884 | NA | NS | 0.432279924 |
| SLC30A4 | -0.062906908 | 0.440593581 | 0.635380278 | NS | 0.355961834 |
| BLOC1S6 | -0.26535165 | 0.009525757 | 0.122699033 | Down | 2.021100512 |
| SQOR | -0.028074192 | 0.769254487 | 0.865536277 | NS | 0.113929962 |
| SEMA6D | 0.770664254 | 0.079948886 | 0.285340058 | NS | 1.097187585 |
| SLC24A5 | 0.691636348 | 0.047061078 | NA | Up | 1.32733813 |
| MYEF2 | 0.243821777 | 0.009229257 | 0.12133665 | Up | 2.034833259 |
| CTXN2 | 1.247231953 | 0.001392895 | 0.060860925 | Up | 2.856081586 |
| SLC12A1 | 1.137570437 | 0.003181591 | 0.084307443 | Up | 2.497355597 |
| DUT | -0.149805348 | 0.098758973 | 0.313587396 | NS | 1.005423437 |
| FBN1 | 0.347925111 | 0.080467223 | 0.286202881 | NS | 1.094380984 |
| CEP152 | -0.02264008 | 0.648563811 | 0.787998723 | NS | 0.188047288 |
| SHC4 | 0.136728815 | 0.716739548 | 0.832645284 | NS | 0.144638631 |
| EID1 | -0.358431168 | 0.002681226 | 0.078552822 | Down | 2.571666562 |
| SECISBP2L | 0.019370397 | 0.666793701 | 0.800739833 | NS | 0.176008511 |
| COPS2 | -0.42887213 | 0.001611349 | 0.063905938 | Down | 2.792810433 |
| GALK2 | -0.009413522 | 0.79754389 | 0.883451036 | NS | 0.098245408 |
| FAM227B | 0.051977206 | 0.391791122 | 0.598576761 | NS | 0.406945409 |
| FGF7 | 0.411442736 | 0.116908043 | 0.336721001 | NS | 0.932155608 |
| DTWD1 | -0.043360263 | 0.390249034 | 0.59729185 | NS | 0.408658164 |
| ATP8B4 | 0.073186413 | 0.358525311 | 0.571528602 | NS | 0.445480179 |
| SLC27A2 | 0.04791838 | 0.758381839 | 0.858918632 | NS | 0.120112075 |
| HDC | -0.334829247 | 0.161351292 | 0.389034501 | NS | 0.792227553 |
| GABPB1 | -0.225005619 | 8.80E-04 | 0.04953684 | Down | 3.055350704 |
| USP8 | -0.018311381 | 0.620687911 | 0.767922494 | NS | 0.207126713 |
| USP50 | 0.19532588 | 0.002755092 | 0.079323886 | Up | 2.559863862 |
| TRPM7 | -0.18582961 | 0.012627945 | 0.135545097 | Down | 1.898667303 |
| SPPL2A | 0.073268098 | 0.156929213 | 0.384159619 | NS | 0.804296202 |
| AP4E1 | -0.097937049 | 0.03801596 | 0.212212115 | NS | 1.42003404 |
| TNFAIP8L3 | 0.608428376 | 0.153492786 | NA | NS | 0.813912032 |
| CYP19A1 | 0.505914395 | 0.150308685 | 0.376600453 | NS | 0.823015925 |
| GLDN | 0.457346814 | 0.212118068 | 0.441830649 | NS | 0.673422338 |
| DMXL2 | 0.125641185 | 0.106584325 | 0.32311031 | NS | 0.97230666 |
| SCG3 | 0.057332936 | 0.598410839 | 0.753135409 | NS | 0.223000549 |
| LYSMD2 | 0.067850276 | 0.487363615 | 0.672268351 | NS | 0.312146897 |
| TMOD2 | 0.001823716 | 0.981701708 | 0.989263416 | NS | 0.008020454 |
| TMOD3 | -0.065253294 | 0.118718169 | 0.339297335 | NS | 0.925482809 |
| LEO1 | -0.150384027 | 0.005601538 | 0.101640588 | Down | 2.251692723 |
| MAPK6 | -0.184787332 | 0.017047809 | 0.153490995 | Down | 1.768331423 |
| BCL2L10 | 0.709003476 | 0.10762715 | NA | NS | 0.968078159 |
| GNB5 | -0.006551728 | 0.905649316 | 0.947761281 | NS | 0.043039936 |
| MYO5C | 0.088392974 | 0.536720691 | 0.708512148 | NS | 0.270251662 |
| MYO5A | 0.068885143 | 0.219756529 | 0.449082183 | NS | 0.658058214 |
| ARPP19 | -0.067655634 | 0.310571328 | 0.530428918 | NS | 0.507838641 |
| FAM214A | 0.044126984 | 0.441496268 | 0.636081248 | NS | 0.355072963 |
| ONECUT1 | 0.840883105 | 0.057390235 | NA | NS | 1.241161995 |
| WDR72 | 0.316426519 | 0.462413828 | 0.653153652 | NS | 0.334969187 |
| UNC13C | 0.547815523 | 0.180288643 | 0.410149052 | NS | 0.74403163 |
| RSL24D1 | -0.415733082 | 0.019213984 | 0.161025962 | Down | 1.716382583 |
| RAB27A | 0.114691197 | 0.07564228 | 0.278917966 | NS | 1.121235391 |
| PIGBOS1 | -0.199638414 | 0.080517183 | 0.286274601 | NS | 1.094111426 |
| PIGB | 0.121610242 | 0.139229252 | 0.363473528 | NS | 0.85626951 |
| CCPG1 | 0.019519312 | 0.805983488 | 0.888459464 | NS | 0.093673856 |
| C15orf65 | 0.254200289 | 0.370724301 | NA | NS | 0.430948944 |
| DNAAF4 | 0.462532108 | 0.197728121 | NA | NS | 0.70393156 |
| PYGO1 | 0.21131081 | 0.520075718 | 0.696688569 | NS | 0.283933423 |
| PRTG | 0.389134892 | 0.305828807 | 0.526821462 | NS | 0.51452161 |
| NEDD4 | -0.196115788 | 0.029363665 | 0.190938857 | Down | 1.532189739 |
| RFX7 | 0.079116883 | 0.186340398 | 0.41588454 | NS | 0.729692981 |
| TEX9 | -0.555136505 | 0.002909389 | 0.080729745 | Down | 2.536198229 |
| MNS1 | -0.025694059 | 0.914590506 | 0.952305149 | NS | 0.038773311 |
| ZNF280D | -0.02190609 | 0.726477094 | 0.838611255 | NS | 0.138778075 |
| TCF12 | -0.050353303 | 0.224881004 | 0.453236933 | NS | 0.648047228 |
| CGNL1 | 0.745133277 | 0.070527609 | 0.2714661 | NS | 1.15164084 |
| MYZAP | -0.512205389 | 0.009249685 | 0.121396148 | Down | 2.033873072 |
| GCOM1 | -0.213831912 | 0.26943718 | 0.494799959 | NS | 0.569542476 |
| POLR2M | -0.200909449 | 0.038137878 | 0.212721309 | Down | 1.418643469 |
| ALDH1A2 | 0.173344237 | 0.235293021 | 0.463213084 | NS | 0.628390954 |
| AQP9 | 0.131376861 | 0.43212223 | 0.628985847 | NS | 0.364393391 |
| LIPC | 0.273143489 | 0.10365165 | 0.319492609 | NS | 0.98442378 |
| ADAM10 | 0.019904223 | 0.747376403 | 0.851665499 | NS | 0.126460618 |
| MINDY2 | 0.049757369 | 0.351534906 | 0.565262486 | NS | 0.454031545 |
| RNF111 | 0.104535213 | 0.057611719 | 0.251510407 | NS | 1.239489165 |
| SLTM | -0.276589974 | 0.002264805 | 0.07354844 | Down | 2.644969128 |
| CCNB2 | 0.222146282 | 0.116882838 | 0.336721001 | NS | 0.932249252 |
| MYO1E | 0.108951202 | 0.210757396 | 0.44059935 | NS | 0.676217176 |
| LDHAL6B | 0.423337418 | 0.008563299 | 0.118335308 | Up | 2.067358866 |
| FAM81A | 0.06720867 | 0.742272478 | 0.84888192 | NS | 0.129436642 |
| GCNT3 | 0.240495684 | 0.372782205 | 0.583458834 | NS | 0.428544828 |
| GTF2A2 | -0.143530909 | 0.075786702 | 0.279091018 | NS | 1.120406993 |
| BNIP2 | -0.105746004 | 0.158656697 | 0.386214013 | NS | 0.799541591 |
| FOXB1 | 0.70477469 | 0.049396079 | NA | Up | 1.306307526 |
| ANXA2 | -0.167834707 | 0.079497017 | 0.284900136 | NS | 1.099649167 |
| ICE2 | -0.085568725 | 0.077574141 | 0.28177684 | NS | 1.110283025 |
| RORA | 0.008576824 | 0.906747807 | 0.948236123 | NS | 0.042513486 |
| VPS13C | -0.011894686 | 0.840328889 | 0.909982137 | NS | 0.075550706 |
| C2CD4A | 0.854618138 | 0.038875335 | NA | Up | 1.410325854 |
| C2CD4B | 1.830480212 | 1.95E-04 | NA | Up | 3.710200326 |
| TLN2 | -0.285293852 | 0.168311332 | 0.396575496 | NS | 0.773886643 |
| TPM1 | -0.238879297 | 0.012144761 | 0.134107677 | Down | 1.915611019 |
| LACTB | -0.064907478 | 0.442976718 | 0.637332127 | NS | 0.353619099 |
| RPS27L | -0.219693037 | 0.056284489 | 0.249221499 | NS | 1.249611275 |
| RAB8B | -0.065220831 | 0.472677294 | 0.6610873 | NS | 0.325435259 |
| APH1B | 0.043191065 | 0.538173964 | 0.709204219 | NS | 0.269077317 |
| CA12 | 0.323260416 | 0.20940548 | 0.439377399 | NS | 0.679011956 |
| USP3 | 0.153904186 | 0.005236061 | 0.098879942 | Up | 2.280995309 |
| FBXL22 | 0.117916111 | 0.430435921 | 0.627698475 | NS | 0.366091492 |
| HERC1 | -0.145510984 | 0.08469675 | 0.292761741 | NS | 1.072133254 |
| DAPK2 | 0.109677879 | 0.384000068 | 0.592802518 | NS | 0.415668698 |
| CIAO2A | -0.308557248 | 0.013525761 | 0.139789052 | Down | 1.868838287 |
| SNX1 | -0.041894379 | 0.210952318 | 0.440805655 | NS | 0.675815699 |
| SNX22 | -0.211884792 | 0.030607264 | 0.194120065 | Down | 1.514175493 |
| PPIB | -0.190209805 | 0.023407909 | 0.173829337 | Down | 1.630637386 |
| CSNK1G1 | 0.16830423 | 0.001279333 | 0.058481609 | Up | 2.893016374 |
| PCLAF | -0.125060098 | 0.541111001 | 0.711314668 | NS | 0.266713637 |
| TRIP4 | 0.014704973 | 0.726032338 | 0.838611255 | NS | 0.139044035 |
| ZNF609 | -0.17925548 | 0.030590321 | 0.19406635 | Down | 1.514415971 |
| OAZ2 | -0.023334862 | 0.823547926 | 0.89959256 | NS | 0.084311122 |
| RBPMS2 | 0.502477527 | 0.147675983 | NA | NS | 0.830690129 |
| PIF1 | 0.345734532 | 0.043009585 | 0.222650435 | Up | 1.366434745 |
| PLEKHO2 | 0.283674949 | 0.003514999 | 0.085936504 | Up | 2.454074788 |
| ANKDD1A | 0.209531478 | 0.045940418 | 0.228673646 | Up | 1.337805061 |
| SPG21 | -0.036436529 | 0.537481954 | 0.708904153 | NS | 0.269636113 |
| MTFMT | 0.174395984 | 0.00307824 | 0.083174295 | Up | 2.511697452 |
| SLC51B | 0.783447884 | 0.222782676 | NA | NS | 0.652118584 |
| RASL12 | 0.721711598 | 0.231965974 | NA | NS | 0.634575716 |
| KBTBD13 | 0.821004037 | 0.109802633 | NA | NS | 0.959387247 |
| UBAP1L | 0.164804089 | 0.182735494 | 0.412926322 | NS | 0.738177089 |
| PDCD7 | -0.140654017 | 0.034231287 | 0.203152353 | Down | 1.465576775 |
| CLPX | -0.093473078 | 0.092865819 | 0.305313379 | NS | 1.032144105 |
| CILP | 0.278482054 | 0.112313052 | 0.330922582 | NS | 0.94956977 |
| PARP16 | 0.17034119 | 0.014519958 | 0.144329895 | Up | 1.838034639 |
| IGDCC3 | 0.860574534 | 0.055545717 | 0.248604264 | NS | 1.255349419 |
| IGDCC4 | 0.736425398 | 0.050294345 | 0.237303925 | NS | 1.298480845 |
| DPP8 | -0.0016694 | 0.959628765 | 0.977556178 | NS | 0.017896742 |
| HACD3 | -0.209368221 | 0.003438311 | 0.085194315 | Down | 2.463654811 |
| INTS14 | -0.089788615 | 0.125715878 | 0.347428564 | NS | 0.900609866 |
| SLC24A1 | 0.152807371 | 0.068035358 | 0.268137287 | NS | 1.167265324 |
| DENND4A | -0.045503773 | 0.603711707 | 0.756827305 | NS | 0.219170402 |
| RAB11A | 0.060604529 | 0.308628446 | 0.529171648 | NS | 0.510564047 |
| MEGF11 | 0.116051138 | 0.280112364 | 0.50348007 | NS | 0.552667721 |
| DIS3L | -0.117688288 | 0.021867702 | 0.169347779 | Down | 1.660196862 |
| TIPIN | -0.360010225 | 0.01636002 | 0.151112662 | Down | 1.786216176 |
| MAP2K1 | -0.124015365 | 0.021240902 | 0.168379028 | Down | 1.672827039 |
| SNAPC5 | -0.190818427 | 0.014061322 | 0.141927014 | Down | 1.851973847 |
| RPL4 | -0.265254771 | 0.031850256 | 0.197978516 | Down | 1.496887076 |
| ZWILCH | -0.129413157 | 0.329432414 | 0.546873089 | NS | 0.482233672 |
| LCTL | 0.423868316 | 0.110020951 | NA | NS | 0.958524604 |
| SMAD6 | 0.458911984 | 0.089307523 | 0.299768562 | NS | 1.049111955 |
| SMAD3 | 0.076485774 | 0.38802768 | 0.595953448 | NS | 0.411137292 |
| AAGAB | -0.146143832 | 0.006469917 | 0.1080547 | Down | 2.189101296 |
| IQCH | 0.282714561 | 0.032931792 | 0.200078102 | Up | 1.482384639 |
| C15orf61 | -0.025469049 | 0.760922199 | 0.86036239 | NS | 0.118659746 |
| MAP2K5 | 0.103059895 | 0.237555271 | 0.465476627 | NS | 0.624235329 |
| SKOR1 | 0.309524751 | 0.244784399 | 0.472685692 | NS | 0.611216265 |
| PIAS1 | 0.094417883 | 0.059359791 | 0.25415179 | NS | 1.226507639 |
| CALML4 | 0.283832392 | 0.048359414 | 0.233310996 | Up | 1.315518971 |
| CLN6 | 0.1760449 | 0.023109721 | 0.173073519 | Up | 1.636205306 |
| FEM1B | -0.195185739 | 0.010172943 | 0.126233693 | Down | 1.992553407 |
| ITGA11 | 0.172234736 | 0.127535033 | 0.349003147 | NS | 0.8943705 |
| CORO2B | 0.224374463 | 0.40334651 | 0.60738002 | NS | 0.394321696 |
| ANP32A | -0.052205156 | 0.35724587 | 0.570389167 | NS | 0.447032783 |
| SPESP1 | 0.535608926 | 0.002142375 | 0.072389226 | Up | 2.669104471 |
| NOX5 | 0.559956296 | 0.162469156 | 0.390076509 | NS | 0.789229075 |
| GLCE | -0.170635152 | 0.020899742 | 0.167651802 | Down | 1.679859072 |
| PAQR5 | 0.193274982 | 0.573866581 | NA | NS | 0.241189065 |
| KIF23 | -0.157452033 | 0.373200178 | 0.583828339 | NS | 0.428058158 |
| RPLP1 | -0.251560512 | 0.10442204 | 0.320470766 | NS | 0.981207827 |
| TLE3 | 0.137577593 | 0.242352671 | 0.470094569 | NS | 0.615552189 |
| UACA | 0.009610544 | 0.934606085 | 0.963896938 | NS | 0.029371396 |
| LARP6 | 0.076307534 | 0.865104701 | NA | NS | 0.062931328 |
| LRRC49 | 0.28991794 | 0.328786034 | 0.546433799 | NS | 0.483086638 |
| THAP10 | 0.222666012 | 0.414070108 | NA | NS | 0.38292612 |
| THSD4 | 0.441186958 | 0.259343052 | 0.485568555 | NS | 0.586125383 |
| NR2E3 | 0.749456037 | 0.02126283 | 0.168379028 | Up | 1.672378935 |
| MYO9A | -0.019030273 | 0.685667722 | 0.81308767 | NS | 0.163886295 |
| SENP8 | 0.225905971 | 0.026051492 | 0.182704458 | Up | 1.584167406 |
| GRAMD2A | 0.237040273 | 0.533786668 | 0.706389823 | NS | 0.272632277 |
| PKM | 0.213806758 | 0.019539257 | 0.162368674 | Up | 1.709091953 |
| PARP6 | 0.108158666 | 0.123720598 | 0.344659912 | NS | 0.907557989 |
| CELF6 | 0.192615092 | 0.728512065 | NA | NS | 0.137563252 |
| HEXA | 0.022978149 | 0.589700804 | 0.747377833 | NS | 0.22936828 |
| TMEM202 | 0.5513331 | 0.32878343 | NA | NS | 0.483090078 |
| ARIH1 | 0.034737305 | 0.415344565 | 0.616572671 | NS | 0.381591468 |
| GOLGA6B | 0.272067095 | 0.525303821 | NA | NS | 0.27958944 |
| HIGD2B | 0.105323891 | 0.728988546 | NA | NS | 0.137279295 |
| BBS4 | 0.085457869 | 0.056010381 | 0.249087638 | NS | 1.251731476 |
| ADPGK | -0.035551803 | 0.464258196 | 0.654881564 | NS | 0.333240421 |
| NEO1 | -0.10801994 | 0.308391643 | 0.52916651 | NS | 0.5108974 |
| HCN4 | 0.876488916 | 0.031681295 | 0.197493826 | Up | 1.499197081 |
| REC114 | 0.198359914 | 0.045732682 | 0.228356747 | Up | 1.33977333 |
| NPTN | -0.005438861 | 0.927055405 | 0.959872638 | NS | 0.03289431 |
| CD276 | 0.789524805 | 0.067249015 | 0.267139948 | NS | 1.172314074 |
| INSYN1 | 1.011226878 | 0.020610668 | NA | Up | 1.685907922 |
| TBC1D21 | 0.659267225 | 0.217678482 | NA | NS | 0.662184499 |
| LOXL1 | 0.70472568 | 1.97E-04 | 0.023046451 | Up | 3.705013276 |
| STOML1 | 0.14168726 | 0.123275792 | 0.344057977 | NS | 0.9091222 |
| PML | 0.308397944 | 0.021581332 | 0.168827377 | Up | 1.665921749 |
| GOLGA6A | 0.44817041 | 0.332772806 | NA | NS | 0.477852171 |
| ISLR2 | 0.953120845 | 0.044736069 | NA | Up | 1.349342178 |
| SEMA7A | -0.07958762 | 0.511843134 | 0.690515454 | NS | 0.290863118 |
| UBL7 | -0.255126458 | 0.036130467 | 0.207904514 | Down | 1.442126419 |
| ARID3B | 0.054320198 | 0.629804602 | 0.774680971 | NS | 0.20079417 |
| CLK3 | -0.106628003 | 0.179122452 | 0.409162417 | NS | 0.746849975 |
| EDC3 | 0.046191985 | 0.434952695 | 0.630940374 | NS | 0.361557974 |
| CYP1A1 | 0.305680258 | 0.498072982 | NA | NS | 0.302707016 |
| CYP1A2 | 0.63557878 | 0.211948906 | NA | NS | 0.673768821 |
| CSK | 0.087767474 | 0.374177435 | 0.584902847 | NS | 0.426922407 |
| LMAN1L | 0.440806909 | 0.299010669 | NA | NS | 0.524313316 |
| ULK3 | 0.019560559 | 0.793147941 | 0.880637817 | NS | 0.100645799 |
| SCAMP2 | -0.054918048 | 0.477519064 | 0.665296362 | NS | 0.321009286 |
| MPI | -0.012114063 | 0.80918052 | 0.890312384 | NS | 0.091954581 |
| FAM219B | -0.066260522 | 0.131436042 | 0.354132123 | NS | 0.881285526 |
| COX5A | 0.085029198 | 0.393945122 | 0.600038083 | NS | 0.404564273 |
| RPP25 | 0.257041448 | 0.067932027 | 0.267927444 | NS | 1.167925425 |
| SCAMP5 | 0.316079944 | 0.262835254 | 0.488909438 | NS | 0.580316383 |
| PPCDC | 0.040646703 | 0.609180568 | 0.760421506 | NS | 0.215253958 |
| C15orf39 | 0.093826113 | 0.619167426 | 0.767117793 | NS | 0.208191899 |
| GOLGA6C | 0.601333503 | 0.171271976 | NA | NS | 0.766313692 |
| GOLGA6D | 0.524302018 | 0.299529282 | NA | NS | 0.523560715 |
| COMMD4 | -0.177289312 | 0.023657199 | 0.174604313 | Down | 1.626036668 |
| NEIL1 | -0.028450276 | 0.775923449 | 0.869613999 | NS | 0.110181123 |
| MAN2C1 | -0.148218752 | 0.136744546 | 0.361172137 | NS | 0.864089986 |
| SIN3A | -0.003821794 | 0.959866914 | 0.97768896 | NS | 0.017788978 |
| PTPN9 | -0.021030174 | 0.695953215 | 0.819766525 | NS | 0.157419954 |
| SNUPN | -0.197557627 | 0.00466429 | 0.09505087 | Down | 2.33121449 |
| IMP3 | -0.232937142 | 0.011935127 | 0.133792603 | Down | 1.923172942 |
| SNX33 | 0.159118848 | 0.130122523 | 0.35213444 | NS | 0.885647523 |
| CSPG4 | 0.698815543 | 0.041312101 | 0.218960826 | Up | 1.383922722 |
| ODF3L1 | 0.497268947 | 0.458448557 | NA | NS | 0.33870939 |
| UBE2Q2 | -0.182922517 | 0.045042268 | 0.226915537 | Down | 1.346379746 |
| FBXO22 | -0.053568442 | 0.340361241 | 0.556026263 | NS | 0.468059901 |
| NRG4 | 0.095875899 | 0.46490378 | 0.655173612 | NS | 0.332636923 |
| TMEM266 | 0.236868616 | 0.044955364 | 0.226727096 | Up | 1.347218483 |
| ETFA | -0.159980268 | 0.016436561 | 0.151237494 | Down | 1.784189041 |
| ISL2 | 0.174011557 | 0.321560614 | 0.540057273 | NS | 0.49273715 |
| SCAPER | -0.028324238 | 0.635358653 | 0.777670027 | NS | 0.196981051 |
| RCN2 | -0.069314214 | 0.46233436 | 0.653120004 | NS | 0.33504383 |
| PSTPIP1 | -0.087344552 | 0.340576244 | 0.556086414 | NS | 0.467785648 |
| TSPAN3 | -0.160315896 | 0.036303437 | 0.208236145 | Down | 1.440052255 |
| PEAK1 | -0.087125493 | 0.443783085 | 0.637917821 | NS | 0.352829256 |
| HMG20A | -0.00731726 | 0.865950009 | 0.924223465 | NS | 0.062507179 |
| LINGO1 | 0.676033604 | 0.101563524 | 0.317326231 | NS | 0.993262239 |
| TBC1D2B | 0.041381048 | 0.357080319 | 0.570303392 | NS | 0.447234086 |
| SH2D7 | 1.052300471 | 0.006613754 | NA | Up | 2.179551967 |
| CIB2 | 0.399804946 | 0.17225185 | NA | NS | 0.763836105 |
| IDH3A | -0.186620684 | 0.00340159 | 0.085194315 | Down | 2.468318013 |
| ACSBG1 | 0.158957704 | 0.407623148 | 0.611075752 | NS | 0.389741161 |
| DNAJA4 | -0.09125781 | 0.559346082 | 0.724358091 | NS | 0.2523194 |
| WDR61 | -0.169509941 | 0.010685035 | 0.128167376 | Down | 1.971224068 |
| CRABP1 | 0.864074541 | 0.190320929 | NA | NS | 0.720513451 |
| IREB2 | 0.03999736 | 0.324923675 | 0.543445703 | NS | 0.488218643 |
| HYKK | 0.292495912 | 0.096889001 | 0.311037904 | NS | 1.013725524 |
| PSMA4 | -0.193767523 | 0.1706728 | 0.399000229 | NS | 0.767835687 |
| CHRNA5 | 0.149851758 | 0.577416239 | NA | NS | 0.238511006 |
| CHRNA3 | 0.567302614 | 0.115281092 | NA | NS | 0.938241919 |
| CHRNB4 | 0.357437959 | 0.447978107 | 0.641635461 | NS | 0.34874321 |
| ADAMTS7 | 0.966139642 | 0.015359202 | 0.147610764 | Up | 1.81363134 |
| MORF4L1 | -0.142666401 | 0.075007751 | 0.278055602 | NS | 1.124893857 |
| CTSH | -0.130790104 | 0.098601965 | 0.31333179 | NS | 1.006114429 |
| RASGRF1 | 0.521314671 | 0.092757698 | 0.305193805 | NS | 1.032650039 |
| ANKRD34C | 0.598605513 | 0.193754031 | NA | NS | 0.712749254 |
| TMED3 | -0.001485689 | 0.980070242 | 0.988182121 | NS | 0.008742797 |
| MINAR1 | 0.151458322 | 0.335602611 | 0.551756712 | NS | 0.474174669 |
| MTHFS | 0.10913853 | 0.172259127 | 0.401076443 | NS | 0.763817758 |
| ST20 | 0.165741095 | 0.268076246 | 0.493891886 | NS | 0.571741667 |
| BCL2A1 | -0.134398984 | 0.454811178 | 0.647122615 | NS | 0.34216887 |
| ZFAND6 | -0.165911342 | 0.0286574 | 0.189026296 | Down | 1.542763209 |
| FAH | -0.190972943 | 0.045788147 | 0.2283624 | Down | 1.339246933 |
| CTXND1 | 0.704757323 | 0.002432708 | 0.075642185 | Up | 2.613909969 |
| ARNT2 | 0.403378777 | 0.339301457 | 0.554994713 | NS | 0.469414275 |
| ABHD17C | 0.003660695 | 0.972405412 | 0.984309822 | NS | 0.012152633 |
| CEMIP | 0.287236693 | 0.023925186 | 0.175295276 | Up | 1.621144672 |
| MESD | -0.035430345 | 0.411853074 | 0.614159337 | NS | 0.385257688 |
| TLNRD1 | 0.284320356 | 2.33E-04 | 0.025213462 | Up | 3.631905075 |
| CFAP161 | -0.054682413 | 0.709599088 | 0.828172976 | NS | 0.148986951 |
| IL16 | 0.080573685 | 0.189259349 | 0.418800923 | NS | 0.722942657 |
| STARD5 | 0.123105291 | 0.207625552 | 0.437401068 | NS | 0.6827192 |
| TMC3 | 0.506368321 | 0.014879521 | 0.146316351 | Up | 1.827411061 |
| MEX3B | 0.368711139 | 0.111983542 | 0.330763937 | NS | 0.950845801 |
| EFL1 | 0.019693453 | 0.671690499 | 0.804303074 | NS | 0.172830795 |
| SAXO2 | -0.236192306 | 0.412003796 | 0.614310935 | NS | 0.385098783 |
| GOLGA6L10 | 0.430209171 | 0.220775893 | NA | NS | 0.656048351 |
| GOLGA6L9 | 0.162775185 | 0.619056054 | NA | NS | 0.208270025 |
| CPEB1 | -0.245755454 | 0.581888449 | NA | NS | 0.235160264 |
| AP3B2 | 0.267807518 | 0.351650261 | 0.565262486 | NS | 0.453889056 |
| FSD2 | 0.279172971 | 0.013801843 | 0.141174551 | Up | 1.860062901 |
| WHAMM | -0.026867199 | 0.786992923 | 0.877108955 | NS | 0.104029173 |
| HOMER2 | 0.128798453 | 0.08584195 | 0.294524696 | NS | 1.066300424 |
| RAMAC | -0.119054843 | 0.192653561 | 0.421709861 | NS | 0.715222959 |
| C15orf40 | -0.016140258 | 0.752335326 | 0.854731503 | NS | 0.123588546 |
| BTBD1 | -0.137103976 | 0.07733661 | 0.281652078 | NS | 1.111614871 |
| TM6SF1 | 0.139669995 | 0.129118217 | 0.351110762 | NS | 0.889012479 |
| HDGFL3 | 0.244331335 | 8.57E-04 | 0.048570579 | Up | 3.067118764 |
| BNC1 | 0.571010197 | 0.18894402 | NA | NS | 0.723666849 |
| SH3GL3 | 0.636585739 | 0.155280167 | NA | NS | 0.808884011 |
| ADAMTSL3 | 0.640749061 | 0.096639514 | 0.310804402 | NS | 1.014845263 |
| GOLGA6L4 | 1.164556656 | 0.039933675 | NA | Up | 1.398660717 |
| ZSCAN2 | -0.158450354 | 0.007339605 | 0.112311046 | Down | 2.134327321 |
| WDR73 | -0.011014471 | 0.81228834 | 0.892488368 | NS | 0.090289781 |
| NMB | -0.089258864 | 0.504644392 | 0.685165156 | NS | 0.297014549 |
| SEC11A | -0.074211169 | 0.393054846 | 0.599318562 | NS | 0.405546844 |
| ZNF592 | 0.077233162 | 0.456227118 | 0.64794489 | NS | 0.340818904 |
| ALPK3 | 0.2054707 | 0.335094163 | 0.551502437 | NS | 0.474833137 |
| SLC28A1 | 0.522998175 | 0.244096971 | NA | NS | 0.61243761 |
| PDE8A | -0.05295034 | 0.316344647 | 0.535897363 | NS | 0.49983951 |
| AKAP13 | -6.47E-04 | 0.992184282 | 0.995609559 | NS | 0.003407657 |
| KLHL25 | 0.142655537 | 0.18053678 | 0.410305979 | NS | 0.743434307 |
| AGBL1 | 0.292843483 | 0.474021621 | 0.662196118 | NS | 0.324201849 |
| NTRK3 | 0.387097429 | 0.331675212 | 0.54864219 | NS | 0.479286984 |
| MRPL46 | 0.026951687 | 0.735536914 | 0.843921385 | NS | 0.133395527 |
| MRPS11 | -5.71E-04 | 0.9904462 | 0.994440985 | NS | 0.00416911 |
| DET1 | 0.07988405 | 0.437657632 | 0.633299747 | NS | 0.358865494 |
| AEN | -0.161542982 | 0.008630288 | 0.118495713 | Down | 2.063974724 |
| ISG20 | -0.005720872 | 0.937461291 | 0.965583022 | NS | 0.028046656 |
| ACAN | 0.597107905 | 0.008904994 | 0.11966441 | Up | 2.050366368 |
| HAPLN3 | 0.020106047 | 0.834978793 | 0.906831844 | NS | 0.078324555 |
| MFGE8 | 0.230970942 | 0.060179194 | 0.255937585 | NS | 1.220553635 |
| ABHD2 | -0.007573839 | 0.932601862 | 0.962852453 | NS | 0.030303722 |
| RLBP1 | 0.172174171 | 0.442098321 | 0.636699969 | NS | 0.354481134 |
| FANCI | -0.025304006 | 0.749736793 | 0.853021612 | NS | 0.125091176 |
| POLG | -0.159843218 | 0.011065933 | 0.129467323 | Down | 1.956011954 |
| RHCG | 0.638407016 | 0.192279879 | NA | NS | 0.71606616 |
| TICRR | 0.382811682 | 0.131747501 | 0.354444963 | NS | 0.880257615 |
| KIF7 | 0.718406383 | 0.049020674 | 0.234672133 | Up | 1.309620719 |
| PLIN1 | 0.435692515 | 0.205911726 | 0.435885316 | NS | 0.686318921 |
| PEX11A | -0.074015613 | 0.472476853 | 0.661005796 | NS | 0.325619463 |
| WDR93 | 0.557333057 | 0.156086913 | 0.383229102 | NS | 0.80663351 |
| MESP1 | 0.784907456 | 0.016362931 | NA | Up | 1.786138913 |
| MESP2 | 0.922360364 | 0.055536396 | NA | NS | 1.255422306 |
| ANPEP | 0.168174022 | 0.297673853 | 0.519257109 | NS | 0.526259311 |
| AP3S2 | 0.133287035 | 0.030265341 | 0.193469884 | Up | 1.519054427 |
| ARPIN | 0.198259687 | 0.259316202 | 0.485568555 | NS | 0.586170348 |
| ZNF710 | 0.118639267 | 0.21104872 | 0.440966863 | NS | 0.675617277 |
| IDH2 | -0.204692131 | 0.010125145 | 0.126203819 | Down | 1.994598764 |
| SEMA4B | 0.100101927 | 0.344920413 | 0.559787079 | NS | 0.462281102 |
| CIB1 | -0.050957004 | 0.407908535 | 0.611112752 | NS | 0.389437208 |
| GDPGP1 | 0.214782676 | 0.02730344 | 0.185919029 | Up | 1.563782639 |
| TTLL13P | 0.330497989 | 0.209134881 | NA | NS | 0.679573525 |
| NGRN | -0.01059419 | 0.876193525 | 0.930516305 | NS | 0.057399961 |
| ZNF774 | 0.421086528 | 0.151145335 | 0.377654815 | NS | 0.820605252 |
| IQGAP1 | -0.034839721 | 0.710717328 | 0.828971135 | NS | 0.148303096 |
| CRTC3 | 0.127290082 | 0.063152172 | 0.261340232 | NS | 1.199611709 |
| BLM | -0.014951785 | 0.758909341 | 0.859274322 | NS | 0.119810102 |
| FURIN | 0.008890658 | 0.964927858 | 0.980424925 | NS | 0.015505155 |
| FES | -0.01451695 | 0.86817808 | 0.925714258 | NS | 0.061391183 |
| MAN2A2 | -0.133076821 | 0.258099681 | 0.484826024 | NS | 0.588212533 |
| HDDC3 | -0.140224312 | 0.06396798 | 0.262496805 | NS | 1.194037366 |
| UNC45A | -0.174329097 | 0.019340998 | 0.161365225 | Down | 1.713521115 |
| RCCD1 | 0.079001771 | 0.51167885 | 0.690336909 | NS | 0.291002534 |
| PRC1 | 0.113741981 | 0.37737335 | 0.587332957 | NS | 0.423228773 |
| VPS33B | -0.070416293 | 0.344295936 | 0.559422882 | NS | 0.463068104 |
| SV2B | 0.356607864 | 0.237198125 | 0.465015533 | NS | 0.624888748 |
| SLCO3A1 | 0.201268728 | 0.013011281 | 0.136878437 | Up | 1.885679944 |
| ST8SIA2 | 0.476123915 | 0.278734599 | 0.503079817 | NS | 0.554809119 |
| FAM174B | 0.197779442 | 0.044806385 | 0.226424497 | Up | 1.348660098 |
| CHD2 | -0.130697007 | 0.061681517 | 0.258215532 | NS | 1.209844955 |
| RGMA | 0.608335454 | 0.103806122 | 0.319643728 | NS | 0.983777035 |
| MCTP2 | 0.101168812 | 0.3615477 | 0.574174151 | NS | 0.441834397 |
| NR2F2 | 0.568448115 | 0.218819936 | 0.448248137 | NS | 0.659913114 |
| ARRDC4 | 0.200974545 | 0.080122962 | 0.285643847 | NS | 1.096243005 |
| IGF1R | 0.105357842 | 0.370074172 | 0.581306195 | NS | 0.431711224 |
| PGPEP1L | 0.282197227 | 0.249016693 | 0.476376043 | NS | 0.603771538 |
| SYNM | -0.135072846 | 0.32667332 | 0.54485998 | NS | 0.485886333 |
| TTC23 | 0.181817748 | 0.193341836 | 0.422666547 | NS | 0.713674161 |
| LRRC28 | 0.14380747 | 0.031816924 | 0.197978516 | Up | 1.497341813 |
| MEF2A | 0.225984143 | 6.22E-04 | 0.041621138 | Up | 3.206533359 |
| LYSMD4 | -0.053457195 | 0.382459598 | 0.591830386 | NS | 0.417414436 |
| ADAMTS17 | 0.153005478 | 0.208501303 | 0.438346984 | NS | 0.680891226 |
| CERS3 | 0.143630174 | 0.524696687 | 0.69998273 | NS | 0.280091678 |
| LINS1 | -0.060969498 | 0.358121567 | 0.571228592 | NS | 0.445969524 |
| ASB7 | 0.022236623 | 0.614999859 | 0.764477137 | NS | 0.211124984 |
| ALDH1A3 | 0.458033319 | 0.076792716 | 0.280881929 | NS | 1.114679971 |
| LRRK1 | 0.103727187 | 0.295092372 | 0.517117263 | NS | 0.530042017 |
| CHSY1 | 0.036696172 | 0.67168919 | 0.804303074 | NS | 0.172831641 |
| SELENOS | -0.144811406 | 0.057295698 | 0.250991981 | NS | 1.241877986 |
| SNRPA1 | -0.048107274 | 0.378624982 | 0.588481122 | NS | 0.421790735 |
| PCSK6 | 0.222479212 | 0.121204419 | 0.34187214 | NS | 0.916481546 |
| TM2D3 | 0.021155377 | 0.628121589 | 0.773528315 | NS | 0.201956279 |
| TARS3 | -0.128682982 | 0.008317099 | 0.117263361 | Down | 2.080028114 |
| OR4F6 | 0.757276691 | 0.117813601 | NA | NS | 0.92880457 |
| OR4F15 | 1.009865385 | 0.035054704 | NA | Up | 1.455253692 |
| POLR3K | 0.073242825 | 0.244836041 | 0.472745554 | NS | 0.611124652 |
| SNRNP25 | -0.258766878 | 0.029592897 | 0.191700154 | Down | 1.528812513 |
| RHBDF1 | 0.365280215 | 0.171820863 | 0.40051312 | NS | 0.764924104 |
| MPG | -0.051047996 | 0.581665675 | 0.740813441 | NS | 0.235326564 |
| NPRL3 | -0.0673547 | 0.772362908 | 0.867431116 | NS | 0.112178591 |
| HBZ | -0.217583341 | 0.595487324 | NA | NS | 0.225127479 |
| HBM | 0.222071662 | 0.369754255 | 0.581122148 | NS | 0.43208682 |
| HBA2 | 2.721003926 | 1.35E-09 | 1.23E-06 | Up | 8.870825474 |
| HBA1 | 2.207630164 | 3.75E-08 | 2.26E-05 | Up | 7.425960085 |
| HBQ1 | 0.107867431 | 0.664837071 | 0.799396157 | NS | 0.177284772 |
| LUC7L | -0.006502001 | 0.927216751 | 0.959888272 | NS | 0.032818731 |
| FAM234A | 0.118919955 | 0.341587654 | 0.55696544 | NS | 0.466497834 |
| RGS11 | 1.049461324 | 0.015215665 | NA | Up | 1.817709062 |
| ARHGDIG | 0.353973406 | 0.593661266 | NA | NS | 0.226461286 |
| PDIA2 | 0.416435328 | 0.10576931 | NA | NS | 0.975640331 |
| AXIN1 | 0.046446804 | 0.417858844 | 0.618692258 | NS | 0.378970401 |
| MRPL28 | -0.111387966 | 0.166777335 | 0.394802555 | NS | 0.77786297 |
| PGAP6 | 0.244339694 | 0.018833166 | 0.159320106 | Up | 1.725076665 |
| NME4 | -0.131416967 | 0.090173601 | 0.300826981 | NS | 1.044920586 |
| DECR2 | 0.043677251 | 0.845463189 | NA | NS | 0.072905297 |
| RAB11FIP3 | -0.111469142 | 0.328170601 | 0.545886338 | NS | 0.483900327 |
| CAPN15 | 0.139265204 | 0.387164631 | 0.595130169 | NS | 0.412104324 |
| PRR35 | 1.525340376 | 0.007528502 | NA | Up | 2.123291433 |
| PIGQ | 0.060204057 | 0.633877206 | 0.776736637 | NS | 0.197994865 |
| NHLRC4 | 0.030940918 | 0.790707728 | 0.878939396 | NS | 0.101984017 |
| RAB40C | -0.045546149 | 0.510800032 | 0.689720084 | NS | 0.291749084 |
| WFIKKN1 | 0.514392882 | 0.020839296 | 0.167577793 | Up | 1.681116958 |
| METTL26 | 0.120662041 | 0.217539675 | 0.447145363 | NS | 0.662461524 |
| MCRIP2 | 0.059054157 | 0.47283163 | 0.661181715 | NS | 0.325293479 |
| WDR90 | 0.289841435 | 0.078386515 | 0.28303577 | NS | 1.105758645 |
| RHOT2 | -0.03843572 | 0.617718948 | 0.766154761 | NS | 0.209209077 |
| RHBDL1 | 0.912243144 | 8.24E-05 | 0.01387957 | Up | 4.083958484 |
| STUB1 | -0.042211687 | 0.614166452 | 0.764054018 | NS | 0.21171391 |
| JMJD8 | 0.070479271 | 0.335745122 | 0.551832552 | NS | 0.473990289 |
| WDR24 | 0.320462109 | 0.011503731 | 0.131729229 | Up | 1.939161264 |
| FBXL16 | 0.273287478 | 0.079490095 | 0.284900136 | NS | 1.099686982 |
| METRN | 0.209676138 | 0.03036651 | 0.193611865 | Up | 1.517605114 |
| ANTKMT | 0.046504572 | 0.633282794 | 0.776665018 | NS | 0.198402312 |
| CCDC78 | -0.088030336 | 0.392678717 | 0.599318562 | NS | 0.405962637 |
| HAGHL | 0.051302123 | 0.616541313 | 0.765562196 | NS | 0.210037817 |
| CIAO3 | 0.052608269 | 0.456266144 | 0.64794489 | NS | 0.340781755 |
| MSLN | 1.120896252 | 0.007286773 | 0.11207634 | Up | 2.137464773 |
| MSLNL | 1.203353179 | 0.01147752 | NA | Up | 1.940151958 |
| RPUSD1 | 0.181481029 | 0.04687275 | 0.230315532 | Up | 1.329079563 |
| CHTF18 | -0.049318088 | 0.672259565 | 0.804729697 | NS | 0.17246301 |
| GNG13 | 0.20250773 | 0.695101901 | NA | NS | 0.157951523 |
| LMF1 | 0.013948661 | 0.905752912 | 0.947779517 | NS | 0.042990261 |
| SOX8 | 0.258804844 | 0.219484957 | 0.448847618 | NS | 0.658595241 |
| SSTR5 | 1.020260447 | 0.032456893 | NA | Up | 1.488693063 |
| C1QTNF8 | 0.836305622 | 0.080645963 | NA | NS | 1.09341737 |
| CACNA1H | 0.490697905 | 0.046188446 | 0.229024017 | Up | 1.335466647 |
| TPSG1 | 0.687127348 | 0.196242637 | NA | NS | 0.707206629 |
| TPSB2 | 0.41860974 | 0.479186811 | NA | NS | 0.319495144 |
| TPSAB1 | 1.137594015 | 0.028191559 | NA | Up | 1.54988091 |
| TPSD1 | 0.906060234 | 0.099885028 | NA | NS | 1.000499606 |
| UBE2I | -0.063490885 | 0.109381829 | 0.327424668 | NS | 0.96105482 |
| BAIAP3 | 0.014184427 | 0.929045835 | 0.961212294 | NS | 0.031962859 |
| TSR3 | -0.1672453 | 0.024826627 | 0.178586374 | Down | 1.60508228 |
| GNPTG | -0.209577028 | 0.003424257 | 0.085194315 | Down | 2.46543361 |
| UNKL | 0.036249199 | 0.780930893 | 0.873363014 | NS | 0.107387396 |
| C16orf91 | -0.24409707 | 0.020972944 | 0.167842555 | Down | 1.678340612 |
| PERCC1 | 0.655724973 | 0.019699591 | NA | Up | 1.705542795 |
| CCDC154 | 0.150599609 | 0.532040055 | 0.705123221 | NS | 0.27405567 |
| CLCN7 | -0.127955418 | 0.315916246 | 0.535418875 | NS | 0.50042804 |
| PTX4 | 0.908123572 | 9.28E-04 | NA | Up | 3.032443621 |
| TELO2 | 0.038578111 | 0.722129034 | 0.836022603 | NS | 0.141385193 |
| IFT140 | 0.102931911 | 0.367000861 | 0.579259389 | NS | 0.435332916 |
| TMEM204 | 0.041229521 | 0.705164938 | 0.825437639 | NS | 0.15170929 |
| CRAMP1 | 0.07509168 | 0.543036492 | 0.712919229 | NS | 0.265170985 |
| JPT2 | -0.081741484 | 0.113922443 | 0.332423088 | NS | 0.943390709 |
| MAPK8IP3 | -0.033568442 | 0.766514442 | 0.864083173 | NS | 0.115479658 |
| NME3 | -0.014126996 | 0.88184764 | 0.933145805 | NS | 0.054606443 |
| MRPS34 | -0.166982773 | 0.058068002 | 0.25208642 | NS | 1.236063115 |
| EME2 | 1.62E-04 | 0.998453682 | 0.999332818 | NS | 6.72E-04 |
| SPSB3 | 0.515828879 | 0.036704201 | NA | Up | 1.435284225 |
| NUBP2 | -0.139161453 | 0.035810076 | 0.206944832 | Down | 1.445994752 |
| IGFALS | 0.532677585 | 0.013060821 | 0.137118535 | Up | 1.884029531 |
| HAGH | -0.244007063 | 0.158250012 | 0.38565549 | NS | 0.800656248 |
| FAHD1 | -0.307822908 | 0.01074287 | 0.12826482 | Down | 1.968879666 |
| MEIOB | 0.235359057 | 0.574952213 | NA | NS | 0.24036825 |
| MSRB1 | 0.245469314 | 0.052661261 | 0.242513843 | NS | 1.278508749 |
| RPL3L | -0.13615379 | 0.62491749 | 0.771193538 | NS | 0.20417732 |
| NDUFB10 | -0.177117532 | 0.037464303 | 0.210180429 | Down | 1.426382347 |
| RPS2 | -0.018319503 | 0.85389152 | 0.917596827 | NS | 0.0685973 |
| RNF151 | 0.373013399 | 0.258619238 | NA | NS | 0.587339173 |
| TBL3 | -0.042029958 | 0.622866651 | 0.769743797 | NS | 0.205604921 |
| GFER | -0.167461513 | 0.098288165 | 0.313029559 | NS | 1.007498772 |
| SYNGR3 | 0.568136742 | 8.65E-04 | 0.04893071 | Up | 3.062836868 |
| ZNF598 | 0.097065976 | 0.369753911 | 0.581122148 | NS | 0.432087224 |
| NPW | 0.94013588 | 0.006247508 | NA | Up | 2.204293177 |
| SLC9A3R2 | 0.783960085 | 0.012552517 | 0.135545097 | Up | 1.901269174 |
| NTHL1 | -0.127892695 | 0.160275006 | 0.388015316 | NS | 0.795134197 |
| TSC2 | -0.006435556 | 0.965637475 | 0.980667351 | NS | 0.015185888 |
| PKD1 | 0.100314258 | 0.537332696 | 0.70878893 | NS | 0.269756732 |
| RAB26 | 0.27417769 | 0.046081249 | 0.228777969 | Up | 1.336475755 |
| TRAF7 | 0.015571775 | 0.825601563 | 0.900763518 | NS | 0.083229494 |
| CASKIN1 | 1.121300015 | 0.006845124 | 0.109849098 | Up | 2.164618693 |
| MLST8 | -0.097078724 | 0.249066532 | 0.476376043 | NS | 0.603684626 |
| BRICD5 | -0.071785404 | 0.564271411 | 0.728337377 | NS | 0.248511953 |
| PGP | -0.050040253 | 0.337073226 | 0.553332034 | NS | 0.472275743 |
| E4F1 | -0.031508475 | 0.592022773 | 0.748757762 | NS | 0.227661587 |
| DNASE1L2 | 0.138967973 | 0.372728876 | 0.583434947 | NS | 0.428606961 |
| ECI1 | -0.059840314 | 0.413289375 | 0.615219581 | NS | 0.38374576 |
| RNPS1 | 0.026792781 | 0.519531215 | 0.696295874 | NS | 0.284388353 |
| ABCA3 | 0.261195665 | 0.059880246 | 0.255234952 | NS | 1.222716422 |
| CCNF | -0.01897574 | 0.853042811 | 0.917099521 | NS | 0.069029173 |
| TEDC2 | 0.487924095 | 0.065787649 | NA | NS | 1.181855636 |
| NTN3 | 0.236561111 | 0.070583309 | 0.271500817 | NS | 1.151297984 |
| TBC1D24 | 0.373926348 | 0.01001594 | 0.125966533 | Up | 1.999308288 |
| ATP6V0C | -0.353067934 | 0.252971195 | 0.479852623 | NS | 0.596928927 |
| AMDHD2 | -0.054652849 | 0.494896531 | 0.677837471 | NS | 0.30548559 |
| PDPK1 | 0.177770375 | 0.022384494 | 0.171054283 | Up | 1.650052714 |
| KCTD5 | -0.087277069 | 0.131920269 | 0.354682132 | NS | 0.87968847 |
| PRSS27 | 0.561071471 | 0.031165142 | 0.196029682 | Up | 1.506330896 |
| SRRM2 | -0.116401401 | 0.507795797 | 0.687641579 | NS | 0.294310898 |
| ELOB | -0.200647001 | 0.061332156 | 0.257517688 | NS | 1.212311767 |
| PRSS33 | 0.754938848 | 0.001971388 | 0.069745459 | Up | 2.705227809 |
| PRSS41 | 0.537702485 | 0.077493068 | NA | NS | 1.110737143 |
| PRSS21 | 0.658028782 | 0.008047673 | NA | Up | 2.094329679 |
| ZG16B | -0.382559101 | 0.204215743 | NA | NS | 0.689910781 |
| FLYWCH2 | -0.112971917 | 0.187699763 | 0.417152261 | NS | 0.726536275 |
| FLYWCH1 | -0.070570241 | 0.501685122 | 0.683166599 | NS | 0.299568778 |
| KREMEN2 | 1.004222529 | 0.003236758 | NA | Up | 2.489889767 |
| PKMYT1 | 0.864069834 | 0.002355164 | 0.074602988 | Up | 2.627978889 |
| PAQR4 | 0.096675524 | 0.469301931 | 0.658454501 | NS | 0.328547659 |
| GREP1 | 0.213195203 | 0.388488443 | 0.596204672 | NS | 0.410621896 |
| CLDN9 | 0.59094324 | 0.068857427 | NA | NS | 1.162049209 |
| CLDN6 | 0.638368501 | 0.158751018 | NA | NS | 0.799283481 |
| TNFRSF12A | 0.028455167 | 0.815685191 | 0.89492705 | NS | 0.088477422 |
| HCFC1R1 | 0.377108873 | 0.006889156 | 0.109912638 | Up | 2.161833993 |
| THOC6 | -0.254184871 | 9.09E-04 | 0.050282353 | Down | 3.041457479 |
| BICDL2 | -0.259382263 | 0.346424851 | 0.561209256 | NS | 0.460390962 |
| MMP25 | 0.083180241 | 0.586569825 | 0.745196127 | NS | 0.231680283 |
| IL32 | 0.266811611 | 0.004572641 | 0.094685918 | Up | 2.339832887 |
| ZSCAN10 | 0.7276504 | 0.042276411 | 0.220751111 | Up | 1.373901884 |
| ZNF205 | 0.286805367 | 0.251535948 | 0.47838202 | NS | 0.599399939 |
| ZNF213 | 0.188751494 | 0.078797786 | 0.283635871 | NS | 1.103485982 |
| OR1F1 | 0.152653222 | 0.807683133 | NA | NS | 0.092758986 |
| ZNF200 | 0.060565247 | 0.376080687 | 0.586177342 | NS | 0.424718969 |
| MEFV | 0.007729273 | 0.937817233 | 0.965732476 | NS | 0.027881791 |
| ZNF263 | -0.167396952 | 0.001484542 | 0.062253356 | Down | 2.828407591 |
| TIGD7 | -0.082383479 | 0.426719745 | 0.624815267 | NS | 0.369857262 |
| ZNF75A | -0.04765201 | 0.3745981 | 0.585360649 | NS | 0.42643443 |
| OR2C1 | 0.218871891 | 0.490989208 | NA | NS | 0.308928053 |
| MTRNR2L4 | 0.055358861 | 0.806830404 | NA | NS | 0.093217744 |
| ZSCAN32 | -0.040186399 | 0.277010605 | 0.501351104 | NS | 0.557503604 |
| ZNF174 | -0.026558894 | 0.674338657 | 0.805989661 | NS | 0.171121943 |
| ZNF597 | 0.042145607 | 0.49532091 | 0.67813036 | NS | 0.305113338 |
| NAA60 | 0.171506349 | 0.006950101 | 0.110119911 | Up | 2.158008909 |
| C16orf90 | 0.862019324 | 0.013356585 | NA | Up | 1.87430457 |
| CLUAP1 | 0.032944604 | 0.636509372 | 0.778637849 | NS | 0.196195197 |
| NLRC3 | -0.10213088 | 0.094228954 | 0.307415197 | NS | 1.025815629 |
| SLX4 | 0.207377401 | 0.198277221 | 0.428189053 | NS | 0.702727177 |
| DNASE1 | -0.091705042 | 0.191306689 | 0.42070534 | NS | 0.718269844 |
| TRAP1 | -0.100152881 | 0.114506809 | 0.333261525 | NS | 0.941168688 |
| CREBBP | -0.002123525 | 0.985751639 | 0.991457393 | NS | 0.006232492 |
| ADCY9 | 0.136954401 | 0.185045723 | 0.415319204 | NS | 0.732720948 |
| SRL | 0.630270997 | 0.214527728 | NA | NS | 0.668516567 |
| TFAP4 | -0.026045648 | 0.789690916 | 0.878567629 | NS | 0.102542858 |
| GLIS2 | 1.342989679 | 0.020340754 | NA | Up | 1.691632944 |
| PAM16 | 0.201690977 | 0.555547781 | NA | NS | 0.255278583 |
| CORO7 | 0.006505491 | 0.95552272 | 0.975891069 | NS | 0.019758982 |
| DNAJA3 | -0.316994341 | 6.75E-05 | 0.012360094 | Down | 4.170941618 |
| NMRAL1 | -0.234607229 | 0.007771711 | 0.114529629 | Down | 2.109483349 |
| HMOX2 | -0.111689067 | 0.06776628 | 0.267721812 | NS | 1.168986354 |
| CDIP1 | 0.396312895 | 0.010145945 | 0.126203819 | Up | 1.993707493 |
| C16orf96 | 0.684133229 | 0.038656925 | 0.213650843 | Up | 1.4127727 |
| UBALD1 | -0.11551157 | 0.550163775 | 0.717735732 | NS | 0.259508009 |
| MGRN1 | 0.006346582 | 0.968497298 | 0.982455456 | NS | 0.013901586 |
| NUDT16L1 | -0.140699311 | 0.032646897 | 0.19959403 | Down | 1.486158091 |
| ANKS3 | 0.058182321 | 0.485693529 | 0.671376842 | NS | 0.313637683 |
| DNAAF8 | 0.191993086 | 0.26398297 | 0.490237234 | NS | 0.578424089 |
| ZNF500 | 0.169950063 | 0.06161864 | 0.258215532 | NS | 1.210287894 |
| SEPTIN12 | 0.779314507 | 0.151038051 | NA | NS | 0.820913626 |
| SMIM22 | 0.604622918 | 0.223860099 | NA | NS | 0.650023308 |
| ROGDI | -0.122044115 | 0.327902459 | 0.545717761 | NS | 0.484255327 |
| GLYR1 | -0.025142227 | 0.623551022 | 0.770308731 | NS | 0.205128005 |
| UBN1 | -0.097522607 | 0.36896433 | 0.580590828 | NS | 0.433015617 |
| PPL | -0.099253851 | 0.588582536 | 0.746757367 | NS | 0.230192628 |
| SEC14L5 | 0.236024386 | 0.24028585 | 0.468217208 | NS | 0.619271803 |
| NAGPA | -0.107424125 | 0.151068101 | 0.377620788 | NS | 0.820827229 |
| ALG1 | -0.146012641 | 0.010184323 | 0.126233693 | Down | 1.992067833 |
| C16orf89 | 0.216012406 | 0.499373675 | NA | NS | 0.301574356 |
| EEF2KMT | -0.134584098 | 0.11421427 | 0.332667558 | NS | 0.942279633 |
| RBFOX1 | 0.552470318 | 0.176905377 | 0.406662379 | NS | 0.752258966 |
| TMEM114 | 0.67126856 | 0.193111255 | NA | NS | 0.714192413 |
| METTL22 | 0.03535135 | 0.669845533 | 0.803057236 | NS | 0.174025335 |
| ABAT | 0.106556877 | 0.236675001 | 0.46462634 | NS | 0.625847612 |
| TMEM186 | -0.055279558 | 0.427346751 | 0.62541269 | NS | 0.369219593 |
| PMM2 | -0.041901485 | 0.508828291 | 0.688232771 | NS | 0.293428749 |
| CARHSP1 | -0.017856952 | 0.813679529 | 0.893529505 | NS | 0.08954661 |
| LITAFD | 0.696524298 | 0.086675005 | NA | NS | 1.062106124 |
| USP7 | -0.125632315 | 0.062287824 | 0.25927222 | NS | 1.205596838 |
| C16orf72 | 0.129330657 | 0.042200349 | 0.220751111 | Up | 1.374683953 |
| GRIN2A | 0.762250751 | 0.056007304 | 0.249087638 | NS | 1.251755331 |
| ATF7IP2 | -0.265616348 | 0.016847075 | 0.15292577 | Down | 1.773475483 |
| EMP2 | 0.494593782 | 0.275847975 | 0.500512623 | NS | 0.559330199 |
| TEKT5 | 0.295715837 | 0.502865087 | NA | NS | 0.298548516 |
| NUBP1 | -0.225508702 | 8.50E-04 | 0.048491302 | Down | 3.070339854 |
| TVP23A | -0.019587929 | 0.825435411 | 0.900673176 | NS | 0.083316904 |
| CIITA | 0.173555225 | 0.194031665 | 0.423452752 | NS | 0.712127391 |
| DEXI | 0.057817703 | 0.432403983 | 0.629116067 | NS | 0.364110315 |
| CLEC16A | -0.143931557 | 0.260318861 | 0.486480828 | NS | 0.584494365 |
| RMI2 | 0.038922634 | 0.782164149 | 0.874066719 | NS | 0.106702094 |
| SOCS1 | 0.752894227 | 3.75E-06 | 0.00138374 | Up | 5.42642722 |
| TNP2 | 0.073993916 | 0.865962179 | NA | NS | 0.062501075 |
| PRM3 | 0.864439794 | 0.148220979 | NA | NS | 0.829090323 |
| PRM2 | 0.144587893 | 0.820417035 | NA | NS | 0.085965331 |
| PRM1 | 0.104070058 | 0.906083227 | NA | NS | 0.042831909 |
| LITAF | 0.044132686 | 0.704224521 | 0.824734454 | NS | 0.152288857 |
| SNN | -0.1217419 | 0.14139899 | 0.365829154 | NS | 0.849553692 |
| TXNDC11 | 0.031539587 | 0.609974326 | 0.760957709 | NS | 0.214688444 |
| ZC3H7A | 0.027658371 | 0.53435708 | 0.706777121 | NS | 0.272168432 |
| RSL1D1 | -0.278513139 | 0.003403533 | 0.085194315 | Down | 2.468070064 |
| GSPT1 | -0.319683755 | 0.099490874 | 0.314541689 | NS | 1.002216754 |
| NPIPB2 | -0.05427784 | 0.806101453 | 0.888482827 | NS | 0.093610296 |
| TNFRSF17 | 0.176682885 | 0.409352101 | 0.612463698 | NS | 0.387902976 |
| SNX29 | -0.028920817 | 0.766993053 | 0.864326958 | NS | 0.11520857 |
| CPPED1 | 0.036724335 | 0.735228392 | 0.843792643 | NS | 0.13357773 |
| SHISA9 | 0.502365913 | 0.152792592 | 0.37932805 | NS | 0.815897702 |
| ERCC4 | 0.001783 | 0.968583948 | 0.982455456 | NS | 0.013862733 |
| MRTFB | 0.102782871 | 0.174674332 | 0.404024697 | NS | 0.75777091 |
| PARN | -0.155342375 | 0.03592157 | 0.207223125 | Down | 1.444644685 |
| BFAR | -0.023354931 | 0.700937623 | 0.822549366 | NS | 0.154320629 |
| NOMO1 | 0.020669756 | 0.85046223 | 0.916056274 | NS | 0.070344969 |
| NPIPA1 | -0.137326269 | 0.56280258 | NA | NS | 0.24964392 |
| PDXDC1 | -0.254256321 | 8.56E-04 | 0.048570579 | Down | 3.067382289 |
| NTAN1 | -0.164498755 | 0.076643694 | 0.280676284 | NS | 1.11552357 |
| RRN3 | -0.153276416 | 0.026356248 | 0.183162888 | Down | 1.579116421 |
| BMERB1 | -0.24201937 | 0.288225632 | NA | NS | 0.5402674 |
| MARF1 | 0.160345671 | 0.303823311 | 0.524985776 | NS | 0.517378907 |
| NDE1 | -0.217871807 | 0.065860104 | 0.265127437 | NS | 1.181377588 |
| MYH11 | 0.687770749 | 0.228531054 | NA | NS | 0.641054777 |
| CEP20 | -0.398545131 | 0.001360804 | 0.059956442 | Down | 2.86620445 |
| ABCC1 | 0.018142621 | 0.870775839 | 0.927057761 | NS | 0.06009363 |
| ABCC6 | -0.056998647 | 0.766510524 | 0.864083173 | NS | 0.115481878 |
| NOMO3 | 0.083042876 | 0.554758594 | 0.721469695 | NS | 0.255895961 |
| XYLT1 | -0.00396577 | 0.958340117 | 0.976977806 | NS | 0.018480332 |
| NOMO2 | -0.007501158 | 0.934960764 | 0.96397808 | NS | 0.029206614 |
| RPS15A | -0.43971047 | 0.032670807 | 0.199595539 | Down | 1.485840134 |
| ARL6IP1 | -0.266564244 | 0.035095382 | 0.205352924 | Down | 1.454750028 |
| SMG1 | 0.132457607 | 0.054599393 | 0.24676996 | NS | 1.262812184 |
| TMC7 | 0.770213082 | 0.082790978 | NA | NS | 1.082016989 |
| COQ7 | -0.172520312 | 0.007032551 | 0.110626998 | Down | 2.152887138 |
| ITPRIPL2 | 0.045361831 | 0.596251568 | 0.751450221 | NS | 0.224570466 |
| SYT17 | 0.182541537 | 0.165802716 | 0.393793812 | NS | 0.78040836 |
| CLEC19A | 0.625924053 | 0.198639693 | NA | NS | 0.701933964 |
| TMC5 | -0.116656869 | 0.52589552 | 0.701010372 | NS | 0.279100529 |
| GDE1 | -0.082432049 | 0.410156238 | 0.613073051 | NS | 0.387050679 |
| CCP110 | -0.013157431 | 0.782861931 | 0.874453587 | NS | 0.106314825 |
| VPS35L | -0.145139088 | 0.010481836 | 0.127685158 | Down | 1.979562654 |
| KNOP1 | -0.091190212 | 0.075600666 | 0.278899237 | NS | 1.121474376 |
| IQCK | 0.171202789 | 0.350663653 | 0.564808607 | NS | 0.455109248 |
| GPRC5B | 0.581505946 | 0.026413267 | 0.183252541 | Up | 1.578177882 |
| GPR139 | 0.220089383 | 0.62547793 | NA | NS | 0.20378801 |
| GP2 | 0.751016489 | 0.136962526 | NA | NS | 0.863398242 |
| UMOD | 0.741489702 | 0.119061579 | NA | NS | 0.924228363 |
| PDILT | 0.713275078 | 0.134227537 | NA | NS | 0.87215838 |
| ACSM5 | 0.378254324 | 0.365149634 | NA | NS | 0.437529131 |
| ACSM2A | 0.588721043 | 0.145137692 | 0.370479651 | NS | 0.838219787 |
| ACSM2B | 0.615391306 | 0.152890216 | 0.379463345 | NS | 0.815620305 |
| ACSM3 | 0.210042118 | 0.047550491 | 0.231617346 | Up | 1.322844997 |
| ACSM1 | 0.289624812 | 0.05787971 | 0.251883322 | NS | 1.23747365 |
| THUMPD1 | -0.111973443 | 0.107744483 | 0.325150106 | NS | 0.967604957 |
| ERI2 | -0.037273942 | 0.590344948 | 0.747720896 | NS | 0.228894149 |
| REXO5 | 0.106085528 | 0.37502006 | 0.585728929 | NS | 0.425945501 |
| DCUN1D3 | 0.179677899 | 0.003363326 | 0.085084692 | Up | 2.473231064 |
| LYRM1 | -0.008131369 | 0.912547242 | 0.951241312 | NS | 0.039744643 |
| DNAH3 | 0.396018136 | 0.120156325 | 0.340734879 | NS | 0.920253364 |
| LDAF1 | -0.090952672 | 0.370032318 | 0.581306195 | NS | 0.431760344 |
| CRYM | -0.357757313 | 0.275743796 | NA | NS | 0.55949425 |
| METTL9 | -0.124999399 | 0.253310157 | 0.480240829 | NS | 0.596347396 |
| IGSF6 | 0.05354893 | 0.691706838 | 0.816889674 | NS | 0.160077931 |
| NPIPB4 | -0.034663008 | 0.886372716 | 0.935950794 | NS | 0.052383621 |
| UQCRC2 | -0.194005071 | 0.029102947 | 0.190161395 | Down | 1.536063033 |
| MOSMO | -0.344068014 | 0.002021515 | 0.070790133 | Down | 2.694322935 |
| EEF2K | 0.110092448 | 0.434755844 | 0.630833536 | NS | 0.361754571 |
| POLR3E | -0.117219947 | 0.094802304 | 0.308053683 | NS | 1.023181109 |
| CDR2 | -0.166262233 | 0.11575774 | 0.33485964 | NS | 0.936449959 |
| NPIPB5 | -0.043995242 | 0.897338091 | NA | NS | 0.047043896 |
| HS3ST2 | 1.305266012 | 0.004688159 | NA | Up | 2.328997676 |
| USP31 | -0.032313568 | 0.579628336 | 0.739260992 | NS | 0.236850391 |
| SCNN1G | 0.707490872 | 0.156566617 | NA | NS | 0.805300832 |
| SCNN1B | 0.530310655 | 0.177183365 | 0.407027924 | NS | 0.751577056 |
| COG7 | 0.06456335 | 0.239396357 | 0.46763242 | NS | 0.620882463 |
| GGA2 | -0.096581756 | 0.079599226 | 0.284900136 | NS | 1.099091153 |
| EARS2 | -0.112125527 | 0.023628726 | 0.174506638 | Down | 1.626559692 |
| UBFD1 | -0.049398497 | 0.514120066 | 0.692243974 | NS | 0.288935445 |
| NDUFAB1 | -0.131861086 | 0.221966454 | 0.450738673 | NS | 0.653712656 |
| PALB2 | -0.039467438 | 0.481088179 | 0.667952803 | NS | 0.317775314 |
| DCTN5 | -0.003760708 | 0.947435163 | 0.97158694 | NS | 0.023450501 |
| PLK1 | 0.189521869 | 0.249132444 | 0.476376043 | NS | 0.603569711 |
| ERN2 | 0.737285105 | 0.070747593 | 0.271700057 | NS | 1.150288334 |
| CHP2 | 0.1475786 | 0.781163459 | NA | NS | 0.10725808 |
| PRKCB | 0.010754885 | 0.837677397 | 0.908700632 | NS | 0.076923203 |
| CACNG3 | 0.740317002 | 0.143952206 | NA | NS | 0.841781677 |
| RBBP6 | 0.091946107 | 0.017699086 | 0.155670827 | NS | 1.75204915 |
| TNRC6A | 0.005537226 | 0.953221724 | 0.974797443 | NS | 0.020806069 |
| SLC5A11 | -0.179510828 | 0.712512018 | NA | NS | 0.147207806 |
| ARHGAP17 | 0.190855303 | 0.094421877 | 0.307427263 | NS | 1.024927372 |
| LCMT1 | -0.027123667 | 0.667171754 | 0.800941842 | NS | 0.175762348 |
| AQP8 | 0.379717389 | 0.430118048 | NA | NS | 0.366412333 |
| ZKSCAN2 | 0.07780162 | 0.299470902 | 0.520805117 | NS | 0.52364537 |
| HS3ST4 | 0.667251246 | 0.198524126 | NA | NS | 0.702186707 |
| C16orf82 | 1.423848905 | 0.048449072 | NA | Up | 1.314714534 |
| KDM8 | -0.092857405 | 0.208327638 | 0.438079107 | NS | 0.681253111 |
| NSMCE1 | -0.104822847 | 0.226558148 | 0.455187048 | NS | 0.644820314 |
| IL4R | 0.172923041 | 0.045141626 | 0.227216158 | Up | 1.345422802 |
| IL21R | -0.157311997 | 0.017328482 | 0.154539288 | Down | 1.761239484 |
| GTF3C1 | 0.001348656 | 0.983924379 | 0.990411222 | NS | 0.007038279 |
| KATNIP | 0.172047088 | 0.060258511 | 0.256083601 | NS | 1.219981606 |
| GSG1L | 0.571604936 | 0.148416549 | 0.374564243 | NS | 0.82851767 |
| XPO6 | 0.162407698 | 0.139277929 | 0.36350349 | NS | 0.8561177 |
| SBK1 | 0.210841071 | 0.174301916 | 0.403422181 | NS | 0.758697839 |
| NPIPB6 | 0.155963632 | 0.626324023 | NA | NS | 0.203200931 |
| EIF3CL | 0.21429321 | 0.59377097 | NA | NS | 0.226381039 |
| NPIPB7 | 0.757588252 | 0.150052633 | NA | NS | 0.823756379 |
| CLN3 | 0.209790603 | 0.006186589 | 0.105138876 | Up | 2.208548759 |
| APOBR | -0.288723115 | 0.067925102 | 0.267927444 | NS | 1.167969702 |
| IL27 | -4.63E-04 | 0.99802553 | 0.999116173 | NS | 8.58E-04 |
| NUPR1 | 0.230586192 | 0.516137036 | 0.693776875 | NS | 0.287234977 |
| SGF29 | -0.177757293 | 0.021304257 | 0.168379028 | Down | 1.671533613 |
| SULT1A2 | 0.217733494 | 0.316253461 | NA | NS | 0.499964713 |
| SULT1A1 | -0.401480599 | 4.68E-04 | 0.036600787 | Down | 3.329676146 |
| NPIPB8 | 0.095597751 | 0.717640183 | NA | NS | 0.144093251 |
| EIF3C | -0.320624132 | 0.014606301 | 0.144746239 | Down | 1.83545975 |
| NPIPB9 | 0.251442149 | 0.684670812 | NA | NS | 0.164518186 |
| ATXN2L | -0.065890761 | 0.692902842 | 0.817687478 | NS | 0.159327658 |
| TUFM | -0.311776501 | 2.86E-04 | 0.028091276 | Down | 3.544158304 |
| SH2B1 | -0.008864923 | 0.932098654 | 0.96274403 | NS | 0.030538119 |
| ATP2A1 | 0.123799039 | 0.60179317 | 0.75556533 | NS | 0.220552746 |
| RABEP2 | -0.056579711 | 0.529927665 | 0.70413711 | NS | 0.275783407 |
| CD19 | 0.035938948 | 0.765013751 | 0.86331923 | NS | 0.116330758 |
| NFATC2IP | -0.095449698 | 0.103543033 | 0.319328319 | NS | 0.984879116 |
| SPNS1 | -0.042585246 | 0.840949234 | 0.91030956 | NS | 0.075230221 |
| LAT | 0.234056129 | 0.184962488 | 0.415213773 | NS | 0.73291634 |
| NPIPB11 | 0.0683311 | 0.624108875 | 0.770738283 | NS | 0.204739641 |
| BOLA2-SMG1P6 | 0.151832614 | 0.272700535 | 0.498012997 | NS | 0.56431401 |
| SLX1B | 0.275450645 | 0.674959385 | NA | NS | 0.170722359 |
| NPIPB12 | -0.085315511 | 0.486938227 | 0.67206682 | NS | 0.31252613 |
| SPN | -0.177618499 | 0.051005958 | 0.238791409 | NS | 1.292379089 |
| QPRT | -0.105359978 | 0.502832134 | 0.684042859 | NS | 0.298576976 |
| C16orf54 | 0.086715099 | 0.166742713 | 0.394802555 | NS | 0.777953137 |
| ZG16 | 0.444064502 | 0.385340397 | NA | NS | 0.414155459 |
| KIF22 | -0.125021722 | 0.108942852 | 0.327029744 | NS | 0.962801259 |
| MAZ | 0.218412719 | 0.045301585 | 0.227416493 | Up | 1.343886603 |
| PRRT2 | 0.344473089 | 0.17834451 | 0.408326093 | NS | 0.748740256 |
| PAGR1 | 0.028739084 | 0.612503914 | 0.762989646 | NS | 0.212891132 |
| MVP | -0.048230761 | 0.52216062 | 0.697843221 | NS | 0.282195885 |
| CDIPT | -0.052656377 | 0.593828083 | 0.749840148 | NS | 0.226339267 |
| SEZ6L2 | 0.80052953 | 0.108653384 | 0.326644764 | NS | 0.963956741 |
| ASPHD1 | 0.905152685 | 0.034315865 | NA | Up | 1.464505055 |
| KCTD13 | 0.164774294 | 0.097679674 | 0.312338632 | NS | 1.010195799 |
| TMEM219 | 0.076906393 | 0.186487154 | 0.415945539 | NS | 0.729351079 |
| TAOK2 | -0.070089233 | 0.313892056 | 0.533448787 | NS | 0.503219676 |
| HIRIP3 | -0.098001692 | 0.13896338 | 0.363222438 | NS | 0.857099633 |
| INO80E | -0.094403676 | 0.206574302 | 0.436556674 | NS | 0.684923705 |
| DOC2A | 0.505402143 | 0.038987049 | 0.214288889 | Up | 1.409079634 |
| C16orf92 | 0.573062335 | 0.397317864 | NA | NS | 0.400861908 |
| TLCD3B | 0.959251793 | 0.07619925 | NA | NS | 1.118049302 |
| ALDOA | 0.167660115 | 0.059760159 | 0.25508876 | NS | 1.223588254 |
| PPP4C | -0.082175099 | 0.273356949 | 0.498634409 | NS | 0.563269882 |
| TBX6 | 0.372695834 | 0.079854421 | 0.285263759 | NS | 1.097701032 |
| YPEL3 | 0.088417992 | 0.65208499 | 0.790412323 | NS | 0.185695797 |
| GDPD3 | -0.077897811 | 0.524980477 | 0.700198177 | NS | 0.279856847 |
| MAPK3 | -0.029234911 | 0.739829792 | 0.847306191 | NS | 0.130868184 |
| CORO1A | 0.022654674 | 0.711562121 | 0.829660711 | NS | 0.147787179 |
| NPIPB13 | 0.493072892 | 0.151106652 | NA | NS | 0.820716418 |
| CD2BP2 | -0.071407296 | 0.112141325 | 0.330787614 | NS | 0.950234318 |
| TBC1D10B | 0.17049023 | 0.157106656 | 0.38440776 | NS | 0.803805416 |
| MYLPF | 0.095186769 | 0.664111106 | 0.798935311 | NS | 0.177759257 |
| ZNF48 | -0.086390119 | 0.250911647 | 0.47774934 | NS | 0.600479179 |
| SEPTIN1 | -0.198129195 | 0.007229334 | 0.11179811 | Down | 2.140901734 |
| ZNF771 | 0.203589032 | 0.185630215 | 0.415463304 | NS | 0.731351333 |
| DCTPP1 | -0.222718472 | 0.040299211 | 0.216621104 | Down | 1.394703457 |
| SEPHS2 | 0.261518045 | 7.54E-04 | 0.045387814 | Up | 3.122561845 |
| ITGAL | -0.017084381 | 0.855618332 | 0.918797854 | NS | 0.067719919 |
| ZNF768 | -0.018118549 | 0.807527172 | 0.889134004 | NS | 0.092842856 |
| ZNF747 | -0.01752441 | 0.752697332 | 0.854945906 | NS | 0.123379623 |
| ZNF764 | 0.314333997 | 7.02E-04 | 0.043826722 | Up | 3.15394786 |
| ZNF688 | -0.065223261 | 0.3669711 | 0.579259389 | NS | 0.435368136 |
| ZNF785 | 0.204941305 | 0.036341294 | 0.208236145 | Up | 1.439599608 |
| ZNF689 | -0.076806674 | 0.269759136 | 0.495193461 | NS | 0.569023837 |
| PRR14 | 0.037150306 | 0.706104969 | 0.826242387 | NS | 0.151130732 |
| FBRS | -0.081602634 | 0.486877512 | 0.67206682 | NS | 0.312580284 |
| SRCAP | 0.112288423 | 0.354820608 | 0.568099942 | NS | 0.449991164 |
| PHKG2 | -0.110061893 | 0.0226065 | 0.171548727 | Down | 1.645766675 |
| CCDC189 | 0.09678155 | 0.554353394 | NA | NS | 0.256213289 |
| RNF40 | -0.182110183 | 0.052919314 | 0.243123408 | NS | 1.276385795 |
| ZNF629 | 0.157233061 | 0.078445743 | 0.283101859 | NS | 1.105430621 |
| BCL7C | -0.263834363 | 0.003054189 | 0.083025772 | Down | 2.515104068 |
| CTF1 | 0.896969029 | 0.015250789 | NA | Up | 1.816707687 |
| FBXL19 | -0.008166343 | 0.930681379 | 0.962064222 | NS | 0.031198976 |
| ORAI3 | 0.165488841 | 0.022296639 | 0.170667658 | Up | 1.651760591 |
| SETD1A | -0.039588735 | 0.839732069 | 0.909602551 | NS | 0.075859261 |
| HSD3B7 | 0.309149575 | 0.111801397 | 0.330426582 | NS | 0.951552772 |
| STX1B | 0.405295422 | 0.232071688 | 0.46031947 | NS | 0.634377839 |
| STX4 | -0.123957438 | 0.074896737 | 0.277823952 | NS | 1.1255371 |
| ZNF668 | 0.176803264 | 0.051454716 | 0.239579569 | NS | 1.288574817 |
| ZNF646 | 0.083431763 | 0.27211365 | 0.497465358 | NS | 0.565249673 |
| PRSS53 | 0.079546559 | 0.460873378 | 0.652105895 | NS | 0.336418378 |
| VKORC1 | -0.112367362 | 0.207776087 | 0.43757584 | NS | 0.682404437 |
| BCKDK | 0.073686863 | 0.298191931 | 0.519744165 | NS | 0.525504113 |
| KAT8 | -0.056450512 | 0.1638502 | 0.391619419 | NS | 0.785553025 |
| PRSS8 | 0.237317487 | 0.161617601 | 0.389330629 | NS | 0.791511345 |
| PRSS36 | 0.267135874 | 0.069204942 | 0.269500354 | NS | 1.159862891 |
| FUS | 0.134929179 | 0.129491618 | 0.351485827 | NS | 0.887758341 |
| PYCARD | 0.029001263 | 0.770785987 | 0.86621231 | NS | 0.11306619 |
| TRIM72 | 0.361420478 | 0.385363534 | 0.593556772 | NS | 0.414129384 |
| PYDC1 | 1.716267029 | 0.001032865 | NA | Up | 2.985956248 |
| ITGAM | 0.17043593 | 0.099017197 | 0.31391083 | NS | 1.004289373 |
| ITGAX | 0.001767703 | 0.990776684 | 0.994641383 | NS | 0.004024222 |
| ITGAD | 0.246195467 | 0.246278844 | 0.474507252 | NS | 0.608572894 |
| COX6A2 | 1.461879383 | 0.031300039 | NA | Up | 1.504455115 |
| ZNF843 | 0.647178979 | 0.107496526 | NA | NS | 0.968605571 |
| ARMC5 | 0.201873263 | 0.111620754 | 0.330105697 | NS | 0.952255047 |
| TGFB1I1 | 0.49358757 | 0.009953055 | 0.125403475 | Up | 2.002043618 |
| SLC5A2 | 0.522933029 | 0.022365996 | 0.170969976 | Up | 1.65041175 |
| RUSF1 | 0.056441602 | 0.384514124 | 0.59312605 | NS | 0.415087703 |
| AHSP | 0.162813879 | 0.481884437 | 0.668490967 | NS | 0.3170571 |
| ZNF720 | -0.093417705 | 0.122894297 | 0.343701955 | NS | 0.91046827 |
| ZNF267 | -0.010982117 | 0.908615569 | 0.949407508 | NS | 0.041619826 |
| TP53TG3D | -0.028571019 | 0.932178285 | NA | NS | 0.030501018 |
| SHCBP1 | 0.20485252 | 0.165654688 | 0.393591001 | NS | 0.78079627 |
| VPS35 | -0.125228042 | 0.031636808 | 0.197316498 | Down | 1.499807339 |
| ORC6 | 0.051994299 | 0.668772823 | 0.802193833 | NS | 0.174721384 |
| MYLK3 | 0.65037475 | 0.024314062 | 0.176718708 | Up | 1.614142479 |
| C16orf87 | -0.14306813 | 0.078665088 | 0.283536015 | NS | 1.104217968 |
| GPT2 | 0.319406772 | 0.082279373 | 0.289101289 | NS | 1.084709026 |
| DNAJA2 | -0.266574718 | 3.01E-04 | 0.029045993 | Down | 3.520906904 |
| NETO2 | -0.018646811 | 0.845217712 | 0.913061552 | NS | 0.073031411 |
| ITFG1 | -0.048279132 | 0.153530105 | 0.380233538 | NS | 0.813806453 |
| PHKB | 0.067622511 | 0.060940149 | 0.256832544 | NS | 1.215096486 |
| ABCC12 | 0.715664794 | 0.132319556 | 0.355335356 | NS | 0.878375966 |
| ABCC11 | 0.59145787 | 0.048544192 | 0.233710128 | Up | 1.313862726 |
| LONP2 | 0.012484445 | 0.74197754 | 0.84883209 | NS | 0.129609241 |
| SIAH1 | 0.062668627 | 0.159234438 | 0.386966794 | NS | 0.797963 |
| N4BP1 | 0.111505027 | 0.134922155 | 0.35858085 | NS | 0.869916731 |
| CBLN1 | 0.910462133 | 0.072130863 | NA | NS | 1.141878871 |
| C16orf78 | 0.160591886 | 0.765332279 | NA | NS | 0.116149969 |
| ZNF423 | 0.510894423 | 0.206644706 | 0.436615014 | NS | 0.684775716 |
| CNEP1R1 | 0.085560611 | 0.329393449 | 0.546848044 | NS | 0.482285042 |
| HEATR3 | -0.174139566 | 0.004136814 | 0.091185087 | Down | 2.383334045 |
| TENT4B | -0.039196086 | 0.301638976 | 0.523185082 | NS | 0.520512542 |
| ADCY7 | -0.1057338 | 0.200706208 | 0.430459005 | NS | 0.697439195 |
| BRD7 | -0.084311473 | 0.044530116 | 0.225674429 | NS | 1.351346172 |
| NKD1 | 0.284123415 | 0.042230848 | 0.220751111 | Up | 1.374370194 |
| SNX20 | 0.255419058 | 7.09E-05 | 0.012790004 | Up | 4.149198996 |
| NOD2 | 0.180728826 | 0.127136862 | 0.348830209 | NS | 0.895728513 |
| CYLD | -0.047034804 | 0.401235496 | 0.605820044 | NS | 0.396600653 |
| SALL1 | 0.400628488 | 0.403148297 | NA | NS | 0.39453517 |
| HNRNPA1P48 | -0.219284685 | 0.098764557 | 0.313587396 | NS | 1.00539888 |
| TOX3 | 0.497804668 | 0.319232604 | 0.53829076 | NS | 0.495892759 |
| CHD9 | -0.137872093 | 0.020858093 | 0.167611247 | Down | 1.680725393 |
| RBL2 | -0.006609557 | 0.883821417 | 0.934370959 | NS | 0.053635479 |
| AKTIP | 0.02168323 | 0.678530054 | 0.808348694 | NS | 0.168430911 |
| RPGRIP1L | -0.047751854 | 0.543920853 | 0.713285597 | NS | 0.264464291 |
| FTO | 0.042082089 | 0.494651181 | 0.677817745 | NS | 0.30570095 |
| IRX3 | 0.56460656 | 0.164272047 | NA | NS | 0.784436331 |
| IRX5 | 1.02503343 | 0.035022318 | NA | Up | 1.455655114 |
| IRX6 | 0.725964476 | 0.123341913 | NA | NS | 0.908889321 |
| MMP2 | 0.452155778 | 0.309740194 | 0.53013003 | NS | 0.509002433 |
| LPCAT2 | 0.196034759 | 0.128565312 | 0.350315619 | NS | 0.890876192 |
| CAPNS2 | 0.132617564 | 0.333367234 | 0.550016309 | NS | 0.477077089 |
| SLC6A2 | 0.678182542 | 0.135306147 | 0.359108389 | NS | 0.868682473 |
| CES1 | -0.45130563 | 0.03904336 | 0.214333278 | Down | 1.408452811 |
| CES5A | -0.542935306 | 0.400965121 | NA | NS | 0.396893404 |
| GNAO1 | 0.221240821 | 0.218752835 | 0.448245889 | NS | 0.66004631 |
| AMFR | -0.090954721 | 0.42456293 | 0.623251088 | NS | 0.372057928 |
| NUDT21 | -0.093475762 | 0.260614614 | 0.486795196 | NS | 0.584001234 |
| OGFOD1 | -0.038719 | 0.413543439 | 0.615486243 | NS | 0.383478865 |
| BBS2 | 0.093926984 | 0.135968883 | 0.360134579 | NS | 0.866560472 |
| MT3 | 1.1379456 | 0.02255208 | NA | Up | 1.646813393 |
| MT2A | -0.322887538 | 0.06801039 | 0.268131166 | NS | 1.167424736 |
| MT1E | -0.166674862 | 0.406558969 | 0.610158159 | NS | 0.390876454 |
| MT1M | 0.846973822 | 0.405974541 | NA | NS | 0.3915012 |
| MT1A | 0.776153029 | 0.382824953 | NA | NS | 0.416999762 |
| MT1F | -0.159507401 | 0.241311417 | 0.469262706 | NS | 0.617422131 |
| MT1G | 0.487288397 | 0.389828141 | NA | NS | 0.409126813 |
| MT1H | 0.648910444 | 0.289581578 | NA | NS | 0.53822907 |
| MT1X | -0.074804061 | 0.508583545 | 0.688025303 | NS | 0.293637695 |
| NUP93 | -0.081082558 | 0.146569318 | 0.372104233 | NS | 0.833956934 |
| SLC12A3 | 0.294651012 | 0.269345775 | 0.494693815 | NS | 0.569689832 |
| HERPUD1 | 0.017386488 | 0.701896691 | 0.823337329 | NS | 0.153726805 |
| CETP | 0.194664091 | 0.083040141 | 0.290260272 | NS | 1.080711924 |
| NLRC5 | 0.099393695 | 0.297251927 | 0.519153789 | NS | 0.526875321 |
| CPNE2 | -0.009135099 | 0.919498008 | 0.95533019 | NS | 0.036449207 |
| PSME3IP1 | -0.065148161 | 0.138281581 | 0.362971321 | NS | 0.859235662 |
| RSPRY1 | -6.81E-04 | 0.980368705 | 0.988352515 | NS | 0.00861056 |
| ARL2BP | 0.311549041 | 0.020326323 | 0.165368901 | Up | 1.691941171 |
| PLLP | -0.050464948 | 0.460938703 | 0.652111908 | NS | 0.336356825 |
| CCL22 | 0.024906818 | 0.96327962 | NA | NS | 0.016247628 |
| CX3CL1 | 0.724354847 | 0.100697679 | 0.316153692 | NS | 0.99698054 |
| CCL17 | 0.244922277 | 0.691104225 | NA | NS | 0.160456452 |
| CIAPIN1 | -0.099687118 | 0.162825131 | 0.390555211 | NS | 0.788278563 |
| COQ9 | -0.133196887 | 0.005488318 | 0.100721984 | Down | 2.260560705 |
| POLR2C | -0.044147247 | 0.535082906 | 0.70726901 | NS | 0.271578923 |
| DOK4 | 0.1193724 | 0.363511498 | 0.575717089 | NS | 0.439481848 |
| CCDC102A | -0.163188066 | 0.138526844 | 0.363014451 | NS | 0.85846606 |
| ADGRG5 | 0.025965071 | 0.809005688 | 0.890205558 | NS | 0.092048425 |
| ADGRG1 | 0.018966921 | 0.882102773 | 0.93320019 | NS | 0.054480813 |
| ADGRG3 | 0.213876622 | 0.207406935 | 0.437312072 | NS | 0.683176727 |
| DRC7 | 0.379889206 | 0.184315863 | 0.414457849 | NS | 0.734437286 |
| KATNB1 | 0.207087156 | 0.042360272 | 0.220932874 | Up | 1.373041258 |
| KIFC3 | 0.090906349 | 0.437067573 | 0.632879529 | NS | 0.359451413 |
| CNGB1 | 0.535495127 | 0.063027137 | 0.261116791 | NS | 1.20047242 |
| TEPP | 0.272038226 | 0.309648102 | 0.530051635 | NS | 0.509131578 |
| ZNF319 | 0.242093951 | 0.006956662 | 0.110119911 | Up | 2.15759907 |
| USB1 | 0.142547479 | 0.214811028 | 0.444126252 | NS | 0.667943427 |
| MMP15 | 0.658837745 | 0.079314879 | NA | NS | 1.100645336 |
| CFAP20 | -0.224021707 | 6.58E-04 | 0.04259149 | Down | 3.181547311 |
| CSNK2A2 | 0.140583788 | 0.026347459 | 0.183162888 | Up | 1.579261269 |
| CCDC113 | 0.303763581 | 0.354382023 | 0.567596132 | NS | 0.450528317 |
| PRSS54 | 0.67579883 | 0.189529813 | NA | NS | 0.722322467 |
| GINS3 | -0.154877131 | 0.132350332 | 0.355335356 | NS | 0.878274966 |
| NDRG4 | 0.385016166 | 0.027613284 | 0.186639939 | Up | 1.558881935 |
| SETD6 | 0.03694197 | 0.453366857 | 0.645910281 | NS | 0.343550231 |
| CNOT1 | 0.042712018 | 0.389007767 | 0.596625801 | NS | 0.410041727 |
| SLC38A7 | 0.157345681 | 0.185933474 | 0.415601057 | NS | 0.730642415 |
| GOT2 | -0.128831137 | 0.090882602 | 0.302001358 | NS | 1.041519246 |
| CDH8 | 0.459184904 | 0.289388121 | 0.512601366 | NS | 0.5385193 |
| CDH11 | 0.636427212 | 0.124224965 | 0.345644532 | NS | 0.905791118 |
| CDH5 | 0.814342262 | 0.058458383 | 0.252415889 | NS | 1.233153205 |
| BEAN1 | 0.080291835 | 0.703066869 | 0.824076843 | NS | 0.153003367 |
| TK2 | 0.10983923 | 0.157302013 | 0.384687738 | NS | 0.803265719 |
| CKLF | 0.138353858 | 0.129837078 | 0.351911357 | NS | 0.886601266 |
| CKLF-CMTM1 | 0.32927884 | 0.071890462 | 0.273508834 | NS | 1.143328723 |
| CMTM1 | 0.29701399 | 0.015216687 | 0.147378943 | Up | 1.817679894 |
| CMTM2 | 0.091625407 | 0.624774743 | 0.771138884 | NS | 0.204276536 |
| CMTM3 | -0.18005001 | 0.012635961 | 0.135545097 | Down | 1.89839172 |
| CMTM4 | 0.086012523 | 0.211439608 | 0.441234413 | NS | 0.674813656 |
| DYNC1LI2 | -0.039397464 | 0.338782385 | 0.554595725 | NS | 0.470079178 |
| TERB1 | 0.340340734 | 0.258327033 | 0.485014404 | NS | 0.587830144 |
| NAE1 | -0.266543238 | 0.002310711 | 0.074221468 | Down | 2.636254339 |
| CA7 | 0.901141599 | 0.031568567 | NA | Up | 1.500745127 |
| PDP2 | 0.096454411 | 0.138822652 | 0.363222438 | NS | 0.857539664 |
| CDH16 | 0.540989307 | 0.279749869 | NA | NS | 0.553230108 |
| RRAD | 0.743967347 | 0.040653887 | NA | Up | 1.390897925 |
| CIAO2B | -0.124571778 | 0.158124627 | 0.385419569 | NS | 0.801000485 |
| CES2 | 0.06125167 | 0.236319207 | 0.46408699 | NS | 0.62650098 |
| CES3 | 0.306847864 | 0.454188353 | NA | NS | 0.342764007 |
| CES4A | -0.047691186 | 0.696564246 | 0.820148803 | NS | 0.157038821 |
| CBFB | -0.190027224 | 0.016999704 | 0.153426713 | Down | 1.769558638 |
| PHAF1 | 0.233754796 | 0.005419993 | 0.100338483 | Up | 2.266001285 |
| B3GNT9 | 0.12589534 | 0.177279244 | 0.407185763 | NS | 0.751342109 |
| TRADD | -0.126629732 | 0.064872514 | 0.264064926 | NS | 1.187939269 |
| FBXL8 | 0.12072532 | 0.18841901 | 0.417733637 | NS | 0.724875284 |
| HSF4 | 0.245560537 | 0.308965474 | 0.529357918 | NS | 0.510090049 |
| NOL3 | 0.247208662 | 0.160867784 | 0.388628058 | NS | 0.79353092 |
| KIAA0895L | 0.192000063 | 0.159409431 | 0.387098757 | NS | 0.797485988 |
| EXOC3L1 | 0.657504065 | 0.001462518 | 0.062253356 | Up | 2.834898642 |
| E2F4 | -0.081815758 | 0.31252975 | 0.532199891 | NS | 0.505108636 |
| ELMO3 | 0.080673989 | 0.473946078 | 0.662171369 | NS | 0.324271066 |
| TMEM208 | -0.121991437 | 0.145192509 | 0.370531575 | NS | 0.838055789 |
| FHOD1 | -0.032386615 | 0.769619761 | 0.865739766 | NS | 0.11372379 |
| SLC9A5 | 0.306604115 | 0.067368234 | 0.267264446 | NS | 1.171544837 |
| PLEKHG4 | 0.01315775 | 0.888654942 | 0.93718804 | NS | 0.05126684 |
| KCTD19 | 0.170319387 | 0.631830752 | 0.775798192 | NS | 0.19939924 |
| LRRC36 | 0.158191456 | 0.571570793 | 0.733561796 | NS | 0.242929972 |
| TPPP3 | 0.230050029 | 0.088695366 | 0.298940583 | NS | 1.052099072 |
| ZDHHC1 | 0.569450108 | 0.0032076 | 0.084356647 | Up | 2.493819804 |
| HSD11B2 | 0.329715064 | 0.056503649 | 0.249864178 | NS | 1.247923501 |
| ATP6V0D1 | -0.085310121 | 0.331320745 | 0.548259227 | NS | 0.479751371 |
| AGRP | 0.132171343 | 0.287436017 | 0.510576256 | NS | 0.541458813 |
| RIPOR1 | -0.121389621 | 0.35127848 | 0.565122842 | NS | 0.454348455 |
| CTCF | 0.033867261 | 0.584272146 | 0.743221139 | NS | 0.233384817 |
| CARMIL2 | 0.050802827 | 0.692940771 | 0.817690104 | NS | 0.159303885 |
| ACD | 0.167400314 | 0.056339836 | 0.249336874 | NS | 1.249184422 |
| PARD6A | -0.096626098 | 0.407756232 | 0.611085087 | NS | 0.389599392 |
| ENKD1 | -0.013778839 | 0.865253876 | 0.92378711 | NS | 0.062856446 |
| C16orf86 | 0.035072818 | 0.722722618 | 0.836344784 | NS | 0.141028353 |
| GFOD2 | -0.182235005 | 0.064278924 | 0.263112765 | NS | 1.191931401 |
| RANBP10 | -0.252580626 | 0.237397364 | 0.46528191 | NS | 0.624524107 |
| TSNAXIP1 | 0.020281721 | 0.912543832 | 0.951241312 | NS | 0.039746266 |
| CENPT | 0.0618875 | 0.65003903 | 0.788976303 | NS | 0.187060567 |
| THAP11 | -0.082019278 | 0.283435516 | 0.506380563 | NS | 0.547545731 |
| NUTF2 | -0.147924546 | 0.138388077 | 0.363014451 | NS | 0.858901326 |
| EDC4 | -0.003767388 | 0.964674962 | 0.980341941 | NS | 0.015618993 |
| NRN1L | 0.196624071 | 0.694206666 | NA | NS | 0.15851122 |
| PSKH1 | 0.232800253 | 0.004768542 | 0.095682107 | Up | 2.3216144 |
| CTRL | 0.816123711 | 0.018273151 | NA | Up | 1.738186553 |
| PSMB10 | -0.082263709 | 0.293044078 | 0.515386052 | NS | 0.533067051 |
| LCAT | 0.146824823 | 0.252195322 | 0.47907912 | NS | 0.598262974 |
| SLC12A4 | -0.009923787 | 0.909205304 | 0.949569393 | NS | 0.041338039 |
| DPEP3 | -0.032641491 | 0.825963117 | 0.900894684 | NS | 0.083039345 |
| DPEP2 | 0.038638226 | 0.664174718 | 0.798935311 | NS | 0.17771766 |
| DUS2 | -0.096325198 | 0.049529503 | 0.235468059 | NS | 1.305136027 |
| DPEP2NB | 0.081883938 | 0.671727574 | 0.804303074 | NS | 0.172806824 |
| DDX28 | -0.063432927 | 0.429328204 | 0.627018987 | NS | 0.36721058 |
| NFATC3 | -0.112293556 | 0.13434146 | 0.357917311 | NS | 0.871789937 |
| ESRP2 | 0.059649133 | 0.58608937 | 0.744832071 | NS | 0.232036156 |
| PLA2G15 | 0.233166074 | 0.004709958 | 0.095487183 | Up | 2.326982924 |
| SLC7A6 | 0.074055076 | 0.249084051 | 0.476376043 | NS | 0.60365408 |
| SLC7A6OS | 0.028899414 | 0.503249576 | 0.684244941 | NS | 0.298216582 |
| PRMT7 | -0.131585505 | 0.030054529 | 0.193148154 | Down | 1.522090067 |
| SMPD3 | 0.425266477 | 0.014369216 | 0.143935548 | Up | 1.842566923 |
| ZFP90 | -0.089928525 | 0.129690616 | 0.351791365 | NS | 0.887091446 |
| CDH3 | 0.670810309 | 0.0774472 | 0.281659932 | NS | 1.110994278 |
| CDH1 | -0.327028565 | 0.111180868 | 0.32972609 | NS | 0.953969939 |
| TANGO6 | -0.114719746 | 0.008940084 | 0.11966441 | Down | 2.048658397 |
| HAS3 | 0.051480011 | 0.594354504 | 0.750256675 | NS | 0.225954442 |
| CHTF8 | -0.002458646 | 0.974335565 | 0.985403414 | NS | 0.011291445 |
| UTP4 | -0.030882798 | 0.715537254 | 0.831794184 | NS | 0.14536775 |
| SNTB2 | 0.111449314 | 0.021939681 | 0.169652654 | Up | 1.658769694 |
| VPS4A | -0.035894312 | 0.320844063 | 0.539490663 | NS | 0.493705993 |
| COG8 | -0.015381738 | 0.706774151 | 0.826560875 | NS | 0.150719342 |
| PDF | 0.171003639 | 0.493677558 | NA | NS | 0.306556615 |
| NIP7 | -0.244996227 | 0.002898857 | 0.080662453 | Down | 2.537773136 |
| TMED6 | 0.120401627 | 0.274601939 | 0.499348937 | NS | 0.561296401 |
| TERF2 | -0.188187973 | 0.16724043 | 0.395348919 | NS | 0.776658725 |
| CYB5B | -0.11432464 | 0.105088577 | 0.321069717 | NS | 0.978444488 |
| NFAT5 | 0.131149786 | 0.279941482 | 0.50348007 | NS | 0.552932743 |
| NQO1 | 0.018208833 | 0.830409291 | 0.903626518 | NS | 0.080707801 |
| NOB1 | 0.011385585 | 0.877357466 | 0.93118249 | NS | 0.056823424 |
| WWP2 | 0.013222994 | 0.875499 | 0.93012377 | NS | 0.057744346 |
| CLEC18A | 0.047825434 | 0.833648196 | 0.905661579 | NS | 0.079017185 |
| PDPR | 0.026683616 | 0.823303848 | 0.899413506 | NS | 0.084439855 |
| CLEC18C | 0.876954202 | 0.021364319 | NA | Up | 1.670310951 |
| EXOSC6 | -0.125184166 | 0.060112455 | 0.255813968 | NS | 1.221035533 |
| AARS1 | -0.220250131 | 0.007917741 | 0.11472857 | Down | 2.101398716 |
| DDX19B | -0.2114708 | 0.001859192 | 0.067265738 | Down | 2.730675791 |
| DDX19A | -0.083752702 | 0.112067261 | 0.330785464 | NS | 0.950521241 |
| ST3GAL2 | 0.062545889 | 0.429467032 | 0.627113872 | NS | 0.367070169 |
| FCSK | 0.132764225 | 0.157579544 | 0.385109899 | NS | 0.802500161 |
| COG4 | -0.182398446 | 6.52E-05 | 0.012037902 | Down | 4.185900914 |
| SF3B3 | -0.159088818 | 0.006997138 | 0.110516053 | Down | 2.155079568 |
| IL34 | 0.453260378 | 0.096175923 | 0.310315721 | NS | 1.016933637 |
| MTSS2 | 0.609489597 | 0.044377183 | NA | Up | 1.352840273 |
| VAC14 | 0.054952439 | 0.561951998 | 0.726288073 | NS | 0.25030078 |
| HYDIN | 0.448951661 | 0.249972937 | 0.477055781 | NS | 0.602107008 |
| CMTR2 | -0.297469405 | 0.007945136 | 0.11472857 | Down | 2.099898646 |
| TLE7 | 0.782445833 | 0.111410627 | NA | NS | 0.953073381 |
| ZNF23 | 0.04991345 | 0.604791424 | 0.757668245 | NS | 0.218394376 |
| ZNF19 | 0.092618934 | 0.421355052 | 0.621298082 | NS | 0.375351795 |
| CHST4 | 0.673104838 | 0.014895314 | NA | Up | 1.826950339 |
| TAT | 0.268055351 | 0.428394791 | 0.626186974 | NS | 0.368155818 |
| MARVELD3 | 0.498922698 | 0.062212254 | 0.259098936 | NS | 1.20612406 |
| PHLPP2 | -0.117820087 | 0.318255736 | 0.537481826 | NS | 0.497223761 |
| AP1G1 | 0.083338972 | 0.075315415 | 0.278385027 | NS | 1.123116129 |
| ATXN1L | 0.038433127 | 0.421569041 | 0.621325323 | NS | 0.37513129 |
| IST1 | -0.054397199 | 0.233519424 | 0.46187597 | NS | 0.631676989 |
| ZNF821 | 0.117560673 | 0.104858954 | 0.32076633 | NS | 0.979394479 |
| PKD1L3 | 0.036496978 | 0.798549869 | 0.884094996 | NS | 0.097697957 |
| DHODH | -0.03321026 | 0.548462542 | 0.716536745 | NS | 0.260853028 |
| TXNL4B | -0.026168506 | 0.650480966 | 0.789303543 | NS | 0.186765407 |
| HP | 0.275655695 | 0.08146699 | 0.287767953 | NS | 1.089018331 |
| HPR | 0.354450285 | 0.00772901 | 0.114126159 | Up | 2.111876155 |
| DHX38 | -0.199071148 | 0.004000562 | 0.090296145 | Down | 2.397878952 |
| PMFBP1 | -0.004311943 | 0.976818284 | 0.986489187 | NS | 0.01018622 |
| ZFHX3 | 0.156550302 | 0.225962254 | 0.454435762 | NS | 0.645964103 |
| PSMD7 | -0.153304692 | 0.005957758 | 0.103288853 | Down | 2.224917138 |
| NPIPB15 | 0.260846466 | 0.350371297 | 0.564607616 | NS | 0.455471479 |
| CLEC18B | 0.497541817 | 0.005201876 | 0.098827133 | Up | 2.283839971 |
| GLG1 | -0.093790025 | 0.189572118 | 0.419031044 | NS | 0.722225537 |
| RFWD3 | -0.041286326 | 0.286098014 | 0.509186875 | NS | 0.543485157 |
| MLKL | 0.02914187 | 0.675521699 | 0.806509938 | NS | 0.170360696 |
| FA2H | 0.803238497 | 0.044149321 | NA | Up | 1.355075971 |
| WDR59 | -0.137659491 | 0.131557693 | 0.354337796 | NS | 0.880883752 |
| ZNRF1 | 0.075569035 | 0.549063174 | 0.717035114 | NS | 0.260377684 |
| LDHD | 0.283625035 | 0.111052808 | 0.32960107 | NS | 0.954470455 |
| ZFP1 | -0.174722706 | 0.069332186 | 0.269766518 | NS | 1.159065105 |
| BCAR1 | 1.375836826 | 0.004514197 | NA | Up | 2.345419468 |
| CFDP1 | -0.203431455 | 0.042839577 | 0.222071523 | Down | 1.368154828 |
| TMEM170A | -0.097425455 | 0.034378843 | 0.203549976 | NS | 1.463708746 |
| CHST6 | 0.586299575 | 0.141783331 | 0.366244286 | NS | 0.848374826 |
| CHST5 | 0.748567874 | 0.108733535 | NA | NS | 0.963636494 |
| TMEM231 | 0.159313725 | 0.402676756 | 0.606877012 | NS | 0.395043438 |
| GABARAPL2 | 0.029682599 | 0.848552453 | 0.914821272 | NS | 0.071321307 |
| ADAT1 | -0.006710383 | 0.882285346 | 0.933307113 | NS | 0.054390934 |
| KARS1 | -0.211836514 | 0.003642681 | 0.08688637 | Down | 2.438578901 |
| TERF2IP | -0.155270631 | 0.013623189 | 0.140154175 | Down | 1.865721234 |
| DUXB | -0.033440286 | 0.884197862 | 0.934725786 | NS | 0.05345054 |
| CPHXL | 0.498472601 | 0.149522231 | NA | NS | 0.825294232 |
| CNTNAP4 | 0.474122862 | 0.325034956 | NA | NS | 0.48806993 |
| MON1B | 0.051747264 | 0.325643556 | 0.544093436 | NS | 0.487257511 |
| SYCE1L | 0.321507456 | 0.082113058 | 0.288607877 | NS | 1.085587771 |
| ADAMTS18 | 0.523812953 | 0.211305733 | 0.441234413 | NS | 0.67508872 |
| NUDT7 | -0.122922974 | 0.324322981 | 0.542854027 | NS | 0.489022278 |
| VAT1L | 0.696750936 | 0.111930127 | 0.330721684 | NS | 0.951053003 |
| CLEC3A | 0.564093398 | 0.266559866 | NA | NS | 0.574205239 |
| WWOX | 0.0564762 | 0.322747299 | 0.541387141 | NS | 0.491137384 |
| MAF | -0.047613317 | 0.483602837 | 0.669598929 | NS | 0.31551116 |
| DYNLRB2 | 0.317177236 | 0.304531292 | NA | NS | 0.516368075 |
| CDYL2 | 0.186766265 | 0.00475959 | 0.095617668 | Up | 2.322430414 |
| CMC2 | 0.136235227 | 0.022890391 | 0.17233675 | Up | 1.640346793 |
| CENPN | -0.183263398 | 0.125946763 | 0.347689581 | NS | 0.89981299 |
| ATMIN | -0.198990793 | 0.006955334 | 0.110119911 | Down | 2.157681994 |
| C16orf46 | 1.080321415 | 0.047444585 | NA | Up | 1.323813348 |
| GCSH | 0.235298095 | 0.173432215 | 0.402425996 | NS | 0.76087023 |
| BCO1 | 0.567949712 | 0.251712154 | NA | NS | 0.599095814 |
| GAN | 0.008396143 | 0.825148338 | 0.900483524 | NS | 0.083467971 |
| CMIP | 0.128771797 | 0.400803648 | 0.605608309 | NS | 0.397068334 |
| PLCG2 | 0.008620551 | 0.892600181 | 0.939686278 | NS | 0.049343029 |
| SDR42E1 | 0.16307418 | 0.073779389 | 0.275958773 | NS | 1.132064946 |
| HSD17B2 | 0.225059065 | 0.424355228 | 0.623190546 | NS | 0.372270443 |
| MPHOSPH6 | -0.070351257 | 0.333361011 | 0.550016309 | NS | 0.477085196 |
| CDH13 | 0.421569321 | 0.074568791 | 0.277209074 | NS | 1.127442896 |
| HSBP1 | -0.145522604 | 0.144410405 | 0.36967266 | NS | 0.840401516 |
| MLYCD | 0.111815411 | 0.312887981 | 0.532524774 | NS | 0.50461112 |
| OSGIN1 | 0.188720081 | 0.279035177 | 0.503208948 | NS | 0.554341043 |
| NECAB2 | 0.219218613 | 0.349503719 | 0.563606123 | NS | 0.456548199 |
| SLC38A8 | 0.760052865 | 0.134917378 | NA | NS | 0.869932107 |
| MBTPS1 | -0.097373567 | 0.021373689 | 0.168379028 | NS | 1.670120518 |
| HSDL1 | -0.091446774 | 0.069931988 | 0.270539117 | NS | 1.155324128 |
| DNAAF1 | 0.271317244 | 0.407274981 | 0.610912471 | NS | 0.390112268 |
| TAF1C | -0.004873436 | 0.977196316 | 0.986717959 | NS | 0.010018179 |
| ADAD2 | 0.462642869 | 0.307321045 | NA | NS | 0.512407699 |
| KCNG4 | 0.727187551 | 0.099195198 | NA | NS | 1.003509352 |
| WFDC1 | 0.431860031 | 0.283574488 | 0.506400289 | NS | 0.547332844 |
| ATP2C2 | 0.259068139 | 0.185197587 | 0.415384315 | NS | 0.732364676 |
| MEAK7 | -0.039675536 | 0.501745342 | 0.683166599 | NS | 0.29951665 |
| COTL1 | 0.151656204 | 0.028276409 | 0.188091738 | Up | 1.548575752 |
| KLHL36 | 0.030322918 | 0.4976816 | 0.679732613 | NS | 0.303048416 |
| USP10 | 0.07718857 | 0.415055067 | 0.616445599 | NS | 0.38189428 |
| CRISPLD2 | -0.052737805 | 0.734387537 | 0.843223873 | NS | 0.134074702 |
| ZDHHC7 | -0.21491059 | 0.008178221 | 0.116478613 | Down | 2.087341175 |
| KIAA0513 | 0.083220186 | 0.343461415 | 0.558502793 | NS | 0.464122045 |
| CIBAR2 | 0.522911705 | 0.274170075 | NA | NS | 0.561979949 |
| GSE1 | 0.173592005 | 0.257931601 | 0.484730205 | NS | 0.588495446 |
| GINS2 | -0.024572628 | 0.78281795 | 0.874453587 | NS | 0.106339225 |
| C16orf74 | -0.243163091 | 0.053694729 | 0.244821585 | NS | 1.270068343 |
| EMC8 | 0.157809712 | 0.011832837 | 0.133168118 | Up | 1.926911105 |
| COX4I1 | -0.246799813 | 0.021810745 | 0.169325318 | Down | 1.661329495 |
| IRF8 | -0.284714237 | 0.002342454 | 0.074602988 | Down | 2.630328886 |
| FOXF1 | 0.965548082 | 0.043520789 | NA | Up | 1.361303237 |
| MTHFSD | 0.052598639 | 0.216015436 | 0.4453714 | NS | 0.665515215 |
| FOXC2 | 1.080933207 | 0.068303906 | NA | NS | 1.165554461 |
| FOXL1 | 0.567559385 | 0.192188715 | 0.42102173 | NS | 0.716272118 |
| C16orf95 | 0.412137674 | 0.124520343 | 0.346117593 | NS | 0.90475969 |
| FBXO31 | -0.031509014 | 0.606726591 | 0.758838518 | NS | 0.217006971 |
| MAP1LC3B | 0.031814755 | 0.790214881 | 0.878678439 | NS | 0.102254796 |
| ZCCHC14 | 0.006388252 | 0.91892859 | 0.955085209 | NS | 0.036718236 |
| JPH3 | 0.531365734 | 0.023897887 | 0.175295276 | Up | 1.621640497 |
| KLHDC4 | -0.202696532 | 0.0500109 | 0.236733572 | NS | 1.300935329 |
| SLC7A5 | 0.034669434 | 0.880308721 | 0.932334579 | NS | 0.055364996 |
| CA5A | 0.336255617 | 0.496397731 | NA | NS | 0.304170213 |
| BANP | -0.122715339 | 0.027378555 | 0.186060433 | Down | 1.562589483 |
| ZNF469 | 0.32736944 | 0.015788649 | 0.149058805 | Up | 1.801655041 |
| ZFPM1 | 0.212113277 | 0.14204136 | 0.36646393 | NS | 0.847585178 |
| ZC3H18 | -0.027903045 | 0.813175294 | 0.893120112 | NS | 0.089815825 |
| IL17C | 0.968422375 | 0.010571582 | NA | Up | 1.975860021 |
| CYBA | 0.223699752 | 0.008688763 | 0.118739663 | Up | 2.061042068 |
| MVD | -0.029238416 | 0.692313274 | 0.817286526 | NS | 0.159697341 |
| SNAI3 | 0.088786957 | 0.255314667 | 0.48212537 | NS | 0.592924236 |
| RNF166 | 0.032930457 | 0.754052557 | 0.856060659 | NS | 0.122598383 |
| CTU2 | -0.010607878 | 0.899860411 | 0.944322115 | NS | 0.045824854 |
| PIEZO1 | -0.010203913 | 0.936749517 | 0.965023501 | NS | 0.028376522 |
| CDT1 | 0.330805313 | 0.011829223 | 0.133168118 | Up | 1.927043768 |
| APRT | -0.093777338 | 0.26789983 | 0.493837886 | NS | 0.572027562 |
| GALNS | 0.018352767 | 0.892136457 | 0.939388926 | NS | 0.049568713 |
| TRAPPC2L | -0.160201415 | 0.019215962 | 0.161025962 | Down | 1.716337875 |
| PABPN1L | 0.287528837 | 0.211744009 | 0.441352326 | NS | 0.674188868 |
| CBFA2T3 | 0.118961006 | 0.511256175 | 0.689969884 | NS | 0.291361433 |
| ACSF3 | -0.023889985 | 0.721763068 | 0.83580398 | NS | 0.141605344 |
| CDH15 | 1.102470913 | 0.016161099 | NA | Up | 1.791529122 |
| SLC22A31 | 0.943016927 | 0.036754679 | NA | Up | 1.434687365 |
| ZNF778 | 0.039808974 | 0.421344646 | 0.621298082 | NS | 0.37536252 |
| ANKRD11 | -0.167915314 | 0.234407236 | 0.46252024 | NS | 0.630028987 |
| SPG7 | -0.046837666 | 0.453109371 | 0.645704115 | NS | 0.343796955 |
| RPL13 | -0.184572264 | 0.114044684 | 0.33250813 | NS | 0.942924953 |
| CPNE7 | 0.554650549 | 0.052538945 | 0.242394626 | NS | 1.27951865 |
| DPEP1 | 1.092965302 | 0.0235371 | NA | Up | 1.628247041 |
| CHMP1A | 0.083054997 | 0.18592817 | 0.415601057 | NS | 0.730654805 |
| SPATA33 | 0.040983888 | 0.630298979 | 0.774958255 | NS | 0.200453397 |
| CDK10 | -0.121639409 | 0.183656512 | 0.413782729 | NS | 0.735993668 |
| SPATA2L | 0.224214213 | 0.02286274 | 0.172237653 | Up | 1.640871727 |
| VPS9D1 | 0.174156038 | 0.171494808 | 0.400078852 | NS | 0.765749024 |
| ZNF276 | -0.123914486 | 0.052396991 | 0.241847979 | NS | 1.280693655 |
| FANCA | 0.010269603 | 0.919497515 | 0.95533019 | NS | 0.03644944 |
| SPIRE2 | 0.455059364 | 0.018071361 | 0.156740993 | Up | 1.743009132 |
| TCF25 | -0.313587533 | 1.08E-04 | 0.016467302 | Down | 3.967159698 |
| MC1R | -0.017492688 | 0.884671688 | 0.934906348 | NS | 0.053217871 |
| TUBB3 | 0.792553091 | 0.067362948 | NA | NS | 1.171578913 |
| DEF8 | 0.060040038 | 0.392904793 | 0.599318562 | NS | 0.405712674 |
| DBNDD1 | 0.313482126 | 0.095571108 | 0.309119979 | NS | 1.01967338 |
| GAS8 | -0.135446137 | 0.260715117 | 0.486863797 | NS | 0.583833786 |
| PRDM7 | 0.398866478 | 0.329763466 | 0.547182226 | NS | 0.481797461 |
| DOC2B | 0.397123182 | 0.38941582 | NA | NS | 0.409586409 |
| RPH3AL | 0.123164109 | 0.593601962 | NA | NS | 0.226504673 |
| RFLNB | 0.173397832 | 0.159646727 | 0.387107395 | NS | 0.79683998 |
| VPS53 | -0.009453743 | 0.903094866 | 0.946056108 | NS | 0.044266627 |
| TLCD3A | 0.187706865 | 0.095465408 | 0.309119979 | NS | 1.020153968 |
| GEMIN4 | -0.132740565 | 0.156350393 | 0.383622959 | NS | 0.805901022 |
| GLOD4 | -0.220531772 | 0.001822399 | 0.066397006 | Down | 2.739356464 |
| MRM3 | -0.242398678 | 7.59E-04 | 0.045387814 | Down | 3.119952798 |
| NXN | 0.387605079 | 0.103425057 | 0.31928753 | NS | 0.985374233 |
| TIMM22 | -0.164763927 | 0.063779521 | 0.262240322 | NS | 1.195318746 |
| ABR | -0.040258154 | 0.767733803 | 0.864740339 | NS | 0.114789337 |
| BHLHA9 | 1.711990034 | 0.003006938 | NA | Up | 2.52187547 |
| TRARG1 | 0.906712369 | 0.09171459 | NA | NS | 1.037561571 |
| YWHAE | 0.068278262 | 0.603823159 | 0.756851459 | NS | 0.219090234 |
| CRK | 0.009017587 | 0.891144706 | 0.938568265 | NS | 0.050051768 |
| MYO1C | 0.146305899 | 0.217662811 | 0.447248377 | NS | 0.662215766 |
| INPP5K | -0.107868719 | 0.233020321 | 0.461411926 | NS | 0.632606205 |
| PITPNA | -0.038186663 | 0.613724412 | 0.763762238 | NS | 0.212026602 |
| SLC43A2 | -0.056865625 | 0.567156707 | 0.730541221 | NS | 0.246296928 |
| SCARF1 | 0.098297351 | 0.509057245 | 0.688340853 | NS | 0.293233377 |
| RILP | -0.110062873 | 0.523470657 | 0.699120888 | NS | 0.281107657 |
| PRPF8 | 0.022605216 | 0.771103536 | 0.866450216 | NS | 0.112887305 |
| WDR81 | -0.207236622 | 0.097471478 | 0.311937489 | NS | 1.011122447 |
| SERPINF2 | 0.295566792 | 0.241975382 | NA | NS | 0.616228817 |
| SERPINF1 | -0.02518326 | 0.827242622 | 0.901708524 | NS | 0.082367098 |
| SMYD4 | -0.135646732 | 0.003832279 | 0.08919396 | Down | 2.416542864 |
| RPA1 | -0.134947297 | 0.120262635 | 0.340873128 | NS | 0.919869284 |
| RTN4RL1 | 0.254628267 | 0.376543948 | NA | NS | 0.424184328 |
| DPH1 | -0.051341322 | 0.530655612 | 0.704648624 | NS | 0.275187239 |
| HIC1 | 0.555833316 | 5.69E-04 | 0.040214178 | Up | 3.244828638 |
| SMG6 | -0.046883482 | 0.607236875 | 0.759162499 | NS | 0.216641864 |
| SRR | -0.005430496 | 0.95507484 | 0.975781763 | NS | 0.019962596 |
| TSR1 | -0.257074172 | 7.96E-05 | 0.013591828 | Down | 4.099219119 |
| SGSM2 | -0.030662639 | 0.731919902 | 0.84131849 | NS | 0.135536444 |
| MNT | 0.063225885 | 0.599076368 | 0.753683091 | NS | 0.222517812 |
| METTL16 | 0.106788938 | 0.105143776 | 0.321152674 | NS | 0.978216431 |
| PAFAH1B1 | 0.126271919 | 0.01763119 | 0.155449116 | Up | 1.753718371 |
| CLUH | 0.098099606 | 0.350931772 | 0.56491456 | NS | 0.454777311 |
| CCDC92B | 0.514388255 | 0.201126285 | NA | NS | 0.696531169 |
| RAP1GAP2 | -0.004061071 | 0.970116989 | 0.983124 | NS | 0.01317589 |
| OR1D5 | 0.436475774 | 0.307659604 | NA | NS | 0.511929523 |
| OR1D2 | 0.147781195 | 0.738661149 | NA | NS | 0.131554743 |
| OR1G1 | 0.213282316 | 0.733617084 | NA | NS | 0.134530564 |
| OR1A2 | 0.980489754 | 0.113157943 | NA | NS | 0.946314957 |
| OR1A1 | 0.521485136 | 0.259895702 | 0.48591097 | NS | 0.585200902 |
| OR3A2 | 0.277718106 | 0.456754208 | NA | NS | 0.340317443 |
| OR3A1 | 0.401138176 | 0.354532337 | NA | NS | 0.450344147 |
| OR1R1P | 0.768552329 | 0.134041467 | NA | NS | 0.872760828 |
| OR1E1 | 0.280088393 | 0.680878926 | NA | NS | 0.166930108 |
| OR3A3 | 0.85071426 | 0.058935141 | NA | NS | 1.229625671 |
| OR1E2 | 1.215533052 | 0.029672826 | NA | Up | 1.527641088 |
| SPATA22 | 0.429351186 | 0.284442717 | NA | NS | 0.546005182 |
| ASPA | 0.499577148 | 0.27152594 | NA | NS | 0.566188675 |
| TRPV3 | 0.520798734 | 0.042390392 | 0.220932874 | Up | 1.372732572 |
| TRPV1 | 0.243667382 | 0.040011311 | 0.215862456 | Up | 1.397817217 |
| SHPK | -0.351653583 | 0.002175946 | 0.072832159 | Down | 2.662351933 |
| CTNS | -0.022737076 | 0.698815896 | 0.82172696 | NS | 0.155637225 |
| TAX1BP3 | 0.426208117 | 0.079307028 | NA | NS | 1.100688327 |
| P2RX5-TAX1BP3 | -0.051211935 | 0.512774666 | 0.691200693 | NS | 0.29007344 |
| EMC6 | 0.057454681 | 0.735795893 | 0.844121502 | NS | 0.133242641 |
| P2RX5 | -0.152168668 | 0.161485539 | 0.389156531 | NS | 0.791866363 |
| ITGAE | -0.007292167 | 0.920854782 | 0.956387874 | NS | 0.035808852 |
| HASPIN | 0.098356045 | 0.579892703 | 0.73941889 | NS | 0.236652356 |
| NCBP3 | -0.059108023 | 0.199001576 | 0.428784342 | NS | 0.701143484 |
| CAMKK1 | 0.019237236 | 0.864498572 | 0.923387051 | NS | 0.06323572 |
| P2RX1 | 0.095065141 | 0.329771699 | 0.547182226 | NS | 0.481786618 |
| ATP2A3 | 0.124930552 | 0.318283492 | 0.537481826 | NS | 0.497185886 |
| ZZEF1 | 0.079941361 | 0.300813398 | 0.522222199 | NS | 0.521702824 |
| CYB5D2 | -0.198751239 | 0.018757408 | 0.159116357 | Down | 1.726827185 |
| ANKFY1 | 0.006464742 | 0.917101105 | 0.954138441 | NS | 0.037582783 |
| UBE2G1 | -0.079909641 | 0.189381876 | 0.418800923 | NS | 0.722661587 |
| SPNS3 | 0.18504712 | 0.146341323 | 0.371811513 | NS | 0.834633024 |
| SPNS2 | 0.31250141 | 0.034072921 | 0.202909595 | Up | 1.467590639 |
| MYBBP1A | -0.045182294 | 0.648884426 | 0.788174637 | NS | 0.18783265 |
| GGT6 | 0.226078506 | 0.635968755 | NA | NS | 0.196564221 |
| SMTNL2 | 1.232681681 | 0.011501922 | NA | Up | 1.939229566 |
| ALOX15 | 0.748708927 | 0.003515324 | 0.085936504 | Up | 2.454034649 |
| PELP1 | 0.10014083 | 0.49721472 | 0.679630648 | NS | 0.303456023 |
| ARRB2 | -0.029703482 | 0.768741894 | 0.865188798 | NS | 0.114219451 |
| MED11 | -0.137549635 | 0.070877605 | 0.271922411 | NS | 1.149490964 |
| CXCL16 | 0.277600473 | 0.010457433 | 0.127471706 | Up | 1.980574929 |
| ZMYND15 | -0.043436444 | 0.83873204 | 0.909109618 | NS | 0.076376767 |
| TM4SF5 | 0.995728937 | 0.094946486 | NA | NS | 1.022521105 |
| VMO1 | -0.315115896 | 0.243023736 | NA | NS | 0.614351306 |
| GLTPD2 | 0.125531018 | 0.457400093 | 0.648911412 | NS | 0.339703751 |
| PSMB6 | -0.179799946 | 0.076487979 | 0.28036615 | NS | 1.116406811 |
| C17orf114 | 0.287395916 | 0.412836246 | NA | NS | 0.38422218 |
| PLD2 | 0.070723747 | 0.519620592 | 0.696374907 | NS | 0.284313647 |
| MINK1 | -0.042637437 | 0.81840658 | 0.896650914 | NS | 0.087030888 |
| CHRNE | 0.033846477 | 0.844229016 | 0.912609137 | NS | 0.073539725 |
| C17orf107 | 0.079824964 | 0.69619086 | 0.819962102 | NS | 0.157271683 |
| GP1BA | -0.059363668 | 0.742771517 | 0.849061806 | NS | 0.129144758 |
| SLC25A11 | -0.138264422 | 0.030358854 | 0.193611865 | Down | 1.517714625 |
| RNF167 | 0.201714711 | 0.004555441 | 0.094415114 | Up | 2.341469612 |
| PFN1 | 0.095585193 | 0.170023363 | 0.398309642 | NS | 0.769491398 |
| ENO3 | 0.10221094 | 0.247475691 | 0.475676734 | NS | 0.606467454 |
| SPAG7 | -0.162303152 | 0.093432732 | 0.306166324 | NS | 1.02950095 |
| CAMTA2 | -0.146428317 | 0.442223675 | 0.636699969 | NS | 0.354358011 |
| INCA1 | 0.238430216 | 0.102841406 | 0.318690856 | NS | 0.987831993 |
| KIF1C | -0.004274203 | 0.952574687 | 0.974466869 | NS | 0.021100964 |
| SLC52A1 | 0.538373567 | 0.005906451 | 0.10308115 | Up | 2.228673396 |
| ZFP3 | 0.198956633 | 0.016086496 | 0.150228934 | Up | 1.793538548 |
| ZNF232 | -0.126500878 | 0.098801568 | 0.313653655 | NS | 1.005236161 |
| USP6 | 0.136409496 | 0.326337351 | 0.544632642 | NS | 0.486333216 |
| ZNF594 | -0.111650079 | 0.274365665 | 0.499246977 | NS | 0.561670239 |
| SCIMP | -0.100069009 | 0.30252966 | 0.523977057 | NS | 0.519232041 |
| RABEP1 | -0.131298332 | 0.003184261 | 0.084307443 | Down | 2.496991387 |
| NUP88 | -0.073356686 | 0.400483729 | 0.605341375 | NS | 0.397415124 |
| RPAIN | -0.068220106 | 0.222393548 | 0.451089986 | NS | 0.652877817 |
| C1QBP | -0.281924741 | 0.001377008 | 0.060387091 | Down | 2.861063524 |
| DHX33 | 0.020058318 | 0.763590318 | 0.862314865 | NS | 0.117139587 |
| DERL2 | -0.01716502 | 0.715948365 | 0.832021589 | NS | 0.145118298 |
| MIS12 | -0.180422725 | 0.025480418 | 0.180610502 | Down | 1.593793453 |
| NLRP1 | -0.068627439 | 0.573404497 | 0.734910168 | NS | 0.241538905 |
| WSCD1 | 0.443229354 | 0.230701563 | 0.458998106 | NS | 0.636949463 |
| AIPL1 | 0.837003511 | 0.077822597 | NA | NS | 1.108894282 |
| PIMREG | 0.495529067 | 0.132384361 | NA | NS | 0.878163315 |
| PITPNM3 | 0.661110255 | 0.013478856 | 0.139553691 | Up | 1.870346959 |
| KIAA0753 | 0.043060842 | 0.411178638 | 0.613993165 | NS | 0.385969456 |
| TXNDC17 | -0.10026491 | 0.27272846 | 0.498012997 | NS | 0.564269541 |
| MED31 | 0.023855633 | 0.720014783 | 0.834756433 | NS | 0.142658587 |
| C17orf100 | 0.118239082 | 0.216435266 | 0.445875727 | NS | 0.664671974 |
| SLC13A5 | 0.543302559 | 0.095393213 | 0.309058959 | NS | 1.020482523 |
| XAF1 | 0.316070769 | 0.008093648 | 0.115705815 | Up | 2.091855714 |
| FBXO39 | 0.464235828 | 0.030365442 | 0.193611865 | Up | 1.517620387 |
| TEKT1 | 0.568174827 | 0.19779632 | NA | NS | 0.703781793 |
| ALOX12 | 0.233269754 | 0.005542004 | 0.101053331 | Up | 2.256333138 |
| RNASEK | 0.084690627 | 0.448687285 | 0.642279906 | NS | 0.348056237 |
| C17orf49 | -0.183823163 | 0.298056409 | 0.519647373 | NS | 0.525701535 |
| BCL6B | 0.631235444 | 0.053288277 | 0.243707535 | NS | 1.273368321 |
| SLC16A13 | 0.410977374 | 0.005127708 | 0.098436518 | Up | 2.290076724 |
| SLC16A11 | 0.434174355 | 0.03554053 | 0.206049964 | Up | 1.4492761 |
| CLEC10A | -0.252640689 | 0.070792384 | 0.27171348 | NS | 1.150013463 |
| ASGR2 | 0.100886362 | 0.475824398 | 0.663783295 | NS | 0.322553294 |
| ASGR1 | 0.058662461 | 0.617524727 | 0.766154761 | NS | 0.209345647 |
| DLG4 | 0.072543678 | 0.681106558 | 0.810027647 | NS | 0.166784938 |
| ACADVL | -0.16758665 | 0.012743033 | 0.135992984 | Down | 1.894727191 |
| DVL2 | 0.098800015 | 0.231855262 | 0.460014658 | NS | 0.634783044 |
| PHF23 | -0.209003032 | 0.001037797 | 0.052796552 | Down | 2.983887591 |
| GABARAP | -0.011430593 | 0.906883738 | 0.948290173 | NS | 0.042448386 |
| CTDNEP1 | -0.064155225 | 0.655735458 | 0.793223847 | NS | 0.183271332 |
| ELP5 | -0.256860633 | 0.007537316 | 0.11304493 | Down | 2.122783285 |
| CLDN7 | -0.344821482 | 0.2788385 | NA | NS | 0.554647262 |
| SLC2A4 | -0.0940001 | 0.757304853 | NA | NS | 0.12072926 |
| EIF5A | -0.125065517 | 0.155963189 | 0.383185333 | NS | 0.806977894 |
| NEURL4 | -0.229780019 | 0.004495914 | 0.094105548 | Down | 2.347181986 |
| ACAP1 | -0.141408769 | 0.02874585 | 0.189231806 | Down | 1.541424848 |
| TNK1 | -0.196393797 | 0.419572384 | NA | NS | 0.377193105 |
| TMEM256 | -0.404390392 | 0.069239195 | 0.269542077 | NS | 1.159647992 |
| TMEM102 | 0.111241603 | 0.658580956 | NA | NS | 0.181390832 |
| CHRNB1 | -0.094355629 | 0.542318133 | 0.712369939 | NS | 0.265745874 |
| ZBTB4 | 0.265923128 | 0.091229659 | 0.30254006 | NS | 1.039863947 |
| POLR2A | 0.071999491 | 0.662713591 | 0.797890162 | NS | 0.178674123 |
| TNFSF12 | 0.370270234 | 0.022789156 | 0.171982657 | Up | 1.642271755 |
| TNFSF13 | -0.205427394 | 0.072434878 | 0.27394225 | NS | 1.140052265 |
| SENP3 | -0.135608714 | 0.053685682 | 0.244821585 | NS | 1.270141523 |
| SENP3-EIF4A1 | 0.123699168 | 0.478832794 | NA | NS | 0.319816114 |
| EIF4A1 | -0.015645151 | 0.85866792 | 0.920182149 | NS | 0.066174762 |
| CD68 | 0.527936019 | 0.289510079 | NA | NS | 0.538336312 |
| MPDU1 | -0.075596129 | 0.331121057 | 0.548174874 | NS | 0.4800132 |
| SOX15 | 0.44005434 | 0.0337937 | 0.20229026 | Up | 1.471164256 |
| FXR2 | -0.219660939 | 3.95E-04 | 0.034192468 | Down | 3.403676138 |
| SHBG | 0.378403516 | 0.214219294 | 0.443419982 | NS | 0.669141417 |
| SAT2 | 0.1907963 | 0.036069531 | 0.207679449 | Up | 1.442859507 |
| ATP1B2 | -0.396859074 | 0.219969942 | 0.449237703 | NS | 0.65763666 |
| TP53 | 0.184424212 | 0.026175272 | 0.182943431 | Up | 1.582108794 |
| WRAP53 | -0.14462386 | 0.019969936 | 0.163807837 | Down | 1.69962332 |
| EFNB3 | 0.550770359 | 0.264085367 | NA | NS | 0.578255662 |
| DNAH2 | 0.317519995 | 0.350057881 | 0.564221661 | NS | 0.455860141 |
| KDM6B | -0.375523439 | 0.068234196 | 0.268476763 | NS | 1.165997922 |
| TMEM88 | -0.173556534 | 0.424239307 | 0.62309553 | NS | 0.372389096 |
| NAA38 | -0.221193792 | 0.047532128 | 0.231617346 | Down | 1.323012746 |
| CYB5D1 | 0.012613936 | 0.955634231 | NA | NS | 0.019708302 |
| CHD3 | -0.158606053 | 0.036477754 | 0.208452907 | Down | 1.437971907 |
| RNF227 | 0.019282207 | 0.811681349 | 0.892035427 | NS | 0.090614433 |
| KCNAB3 | 0.141697473 | 0.424562281 | 0.623251088 | NS | 0.372058592 |
| TRAPPC1 | 0.123173102 | 0.226896252 | 0.455463052 | NS | 0.644172678 |
| CNTROB | -0.169075985 | 0.039661547 | 0.215550248 | Down | 1.401630352 |
| GUCY2D | 0.576763442 | 0.097761026 | 0.312436926 | NS | 1.00983425 |
| ALOX15B | 0.098200764 | 0.703251292 | 0.824166467 | NS | 0.152889461 |
| ALOX12B | 0.294046174 | 0.453823694 | NA | NS | 0.343112833 |
| ALOXE3 | 0.740808545 | 0.115539182 | 0.334479283 | NS | 0.937270712 |
| HES7 | 0.707090723 | 0.099430739 | NA | NS | 1.002479334 |
| PER1 | 0.063791522 | 0.726620814 | 0.838611255 | NS | 0.138692166 |
| VAMP2 | 0.138997035 | 0.222418546 | 0.451100739 | NS | 0.652829002 |
| TMEM107 | -0.125894089 | 0.25780112 | 0.484730205 | NS | 0.588715201 |
| BORCS6 | 0.064130003 | 0.367958467 | 0.579931513 | NS | 0.434201199 |
| AURKB | 0.276553415 | 0.205450297 | 0.435426865 | NS | 0.687293226 |
| CTC1 | 0.021794041 | 0.808659873 | 0.890038851 | NS | 0.092234107 |
| PFAS | 0.003363514 | 0.972490539 | 0.984309822 | NS | 0.012114615 |
| SLC25A35 | 0.300402089 | 0.097806013 | 0.312493486 | NS | 1.009634445 |
| RANGRF | -0.049205693 | 0.676580554 | 0.806945644 | NS | 0.169680489 |
| ARHGEF15 | 0.665806832 | 0.169016722 | 0.397251742 | NS | 0.772070325 |
| ODF4 | 0.256197821 | 0.663037811 | NA | NS | 0.178461704 |
| KRBA2 | 0.089383558 | 0.190158931 | 0.419639606 | NS | 0.720883274 |
| RPL26 | -0.808999646 | 0.004871699 | 0.096717602 | Down | 2.312319571 |
| RNF222 | 0.357279632 | 0.088190606 | 0.298122562 | NS | 1.054577674 |
| NDEL1 | 0.048910401 | 0.596475058 | 0.751690517 | NS | 0.224407712 |
| MYH10 | 0.008250795 | 0.946327686 | 0.971046941 | NS | 0.023958454 |
| CCDC42 | 0.559392674 | 0.115394923 | NA | NS | 0.937813299 |
| SPDYE4 | 0.591345966 | 0.008844189 | 0.119269279 | Up | 2.053341975 |
| MFSD6L | 0.224246769 | 0.151188602 | 0.377654815 | NS | 0.82048095 |
| PIK3R6 | 0.299031527 | 0.054632776 | 0.24676996 | NS | 1.262546733 |
| PIK3R5 | 0.086032927 | 0.338512491 | 0.554352025 | NS | 0.470425301 |
| NTN1 | 0.384066501 | 0.030408737 | 0.193712955 | Up | 1.517001614 |
| STX8 | -0.125141585 | 0.110779306 | 0.32927273 | NS | 0.95554136 |
| CFAP52 | 0.517748757 | 0.216795205 | NA | NS | 0.663950327 |
| USP43 | 0.632553119 | 0.018326555 | 0.157466628 | Up | 1.736919176 |
| DHRS7C | 0.798722478 | 0.121016505 | NA | NS | 0.917155395 |
| GSG1L2 | 0.499799412 | 0.359951136 | NA | NS | 0.443756452 |
| GLP2R | 0.582573586 | 0.199685371 | 0.429206416 | NS | 0.699653751 |
| RCVRN | 0.364913414 | 0.180333669 | NA | NS | 0.743923182 |
| GAS7 | 0.197778269 | 0.09229409 | 0.304334833 | NS | 1.03482611 |
| MYH13 | 0.84662173 | 0.048345402 | 0.233310996 | Up | 1.315644821 |
| MYH8 | 0.593023851 | 0.181310357 | 0.411329352 | NS | 0.741577386 |
| MYH4 | 0.651253756 | 0.119475468 | 0.340007095 | NS | 0.922721261 |
| MYH1 | 0.331841422 | 0.439011267 | 0.634216618 | NS | 0.357524333 |
| MYH2 | 0.589090329 | 0.16559308 | 0.393591001 | NS | 0.780957815 |
| MYH3 | -0.031375783 | 0.74582335 | 0.850639911 | NS | 0.127364024 |
| SCO1 | 0.017798188 | 0.658417112 | 0.794555206 | NS | 0.18149889 |
| ADPRM | -0.062212194 | 0.503428319 | 0.684406706 | NS | 0.298062358 |
| TMEM220 | 0.060142455 | 0.521639961 | 0.697367252 | NS | 0.282629146 |
| TMEM238L | 0.992286338 | 0.081251746 | NA | NS | 1.090167296 |
| PIRT | 0.203592695 | 0.652533136 | NA | NS | 0.18539743 |
| SHISA6 | 0.641344885 | 0.138117489 | 0.362790084 | NS | 0.859751324 |
| DNAH9 | 0.34228485 | 0.256596975 | 0.483550064 | NS | 0.590748467 |
| ZNF18 | -0.039997704 | 0.368249922 | 0.580219196 | NS | 0.433857336 |
| MAP2K4 | -0.067854528 | 0.278656069 | 0.503057015 | NS | 0.554931493 |
| MYOCD | 0.382031297 | 0.350689337 | 0.564808607 | NS | 0.455077439 |
| ARHGAP44 | 0.201482202 | 0.207031369 | 0.436958106 | NS | 0.683963847 |
| ELAC2 | -0.133216776 | 0.015664358 | 0.148636863 | Down | 1.805087402 |
| HS3ST3A1 | 0.654995873 | 0.045706498 | 0.228303209 | Up | 1.340022054 |
| COX10 | -0.014215236 | 0.785872782 | 0.876321892 | NS | 0.104647752 |
| CDRT15 | 0.027579394 | 0.926884548 | NA | NS | 0.032974358 |
| HS3ST3B1 | 0.141251347 | 0.018774045 | 0.159116357 | Up | 1.726442157 |
| PMP22 | 0.506180753 | 0.029658302 | 0.191874131 | Up | 1.527853722 |
| TEKT3 | 0.298788515 | 0.399253606 | NA | NS | 0.398751153 |
| CDRT4 | 0.21582001 | 0.183048777 | 0.413359339 | NS | 0.737433168 |
| TVP23C | 0.152849401 | 0.093119127 | 0.305593248 | NS | 1.030961102 |
| CDRT1 | 0.192759152 | 0.409438992 | 0.612553684 | NS | 0.387810801 |
| TRIM16 | 0.152670643 | 0.030946237 | 0.195253594 | Up | 1.509392154 |
| ZNF286A-TBC1D26 | 0.561179812 | 0.255833397 | NA | NS | 0.592042762 |
| ZNF286A | -0.102478603 | 0.187917982 | 0.417308021 | NS | 0.72603166 |
| TBC1D26 | 0.477040093 | 0.311727576 | NA | NS | 0.506224777 |
| ADORA2B | -0.01128829 | 0.914363727 | 0.95220598 | NS | 0.038881011 |
| ZSWIM7 | -0.088748023 | 0.236264255 | 0.46408699 | NS | 0.626601979 |
| TTC19 | 0.043176933 | 0.264724216 | 0.490947769 | NS | 0.577206329 |
| NCOR1 | 0.055042599 | 0.406094597 | 0.609645411 | NS | 0.391372788 |
| PIGL | 0.029197889 | 0.64352983 | 0.784401521 | NS | 0.191431317 |
| CENPV | 0.055769535 | 0.477608873 | 0.665381009 | NS | 0.320927614 |
| UBB | -0.246176991 | 0.181114574 | 0.411088798 | NS | 0.742046602 |
| TRPV2 | -0.077739959 | 0.186289892 | 0.41588454 | NS | 0.72981071 |
| LRRC75A | -0.089272996 | 0.393040719 | 0.599318562 | NS | 0.405562454 |
| ZNF287 | -0.068049291 | 0.417752196 | 0.618692258 | NS | 0.379081258 |
| ZNF624 | -0.149486683 | 0.082955362 | 0.290260272 | NS | 1.081155539 |
| CCDC144A | -0.066704316 | 0.846654955 | 0.913721869 | NS | 0.072293545 |
| TNFRSF13B | 0.055623906 | 0.73807019 | 0.845925507 | NS | 0.131902335 |
| MPRIP | 0.073683631 | 0.278518755 | 0.502967712 | NS | 0.555145555 |
| PLD6 | -0.181080476 | 0.196724704 | 0.426481571 | NS | 0.7061411 |
| FLCN | -0.222679884 | 0.016771436 | 0.152541471 | Down | 1.775429754 |
| COPS3 | -0.276845852 | 0.00866419 | 0.118708759 | Down | 2.062272018 |
| NT5M | -0.154955999 | 0.438425727 | 0.633805873 | NS | 0.358103969 |
| MED9 | 0.099469958 | 0.060264938 | 0.256083601 | NS | 1.219935283 |
| RASD1 | 0.149595931 | 0.392013956 | 0.598738083 | NS | 0.406698472 |
| PEMT | -0.014217333 | 0.898291357 | 0.943418999 | NS | 0.046582779 |
| RAI1 | 0.05740047 | 0.677865191 | 0.807808753 | NS | 0.168856667 |
| SREBF1 | 0.044165686 | 0.751425067 | 0.854178632 | NS | 0.124114321 |
| TOM1L2 | 0.113469265 | 0.312393131 | 0.532085935 | NS | 0.505298525 |
| DRC3 | 0.221206861 | 0.086663865 | 0.295984568 | NS | 1.062161947 |
| ATPAF2 | 0.035498453 | 0.531433174 | 0.704868508 | NS | 0.274551339 |
| GID4 | -0.082331097 | 0.528442005 | 0.70325869 | NS | 0.277002668 |
| DRG2 | -0.02023082 | 0.677136871 | 0.807234846 | NS | 0.169323538 |
| MYO15A | 0.511097512 | 0.055525575 | 0.248604264 | NS | 1.255506935 |
| ALKBH5 | -0.108990901 | 0.142971183 | 0.367845183 | NS | 0.844751489 |
| LLGL1 | 0.014790217 | 0.837987408 | 0.908864729 | NS | 0.076762507 |
| FLII | -0.204042016 | 0.022741434 | 0.171982657 | Down | 1.64318215 |
| MIEF2 | -0.148027078 | 0.268459945 | 0.494168679 | NS | 0.571120503 |
| TOP3A | -0.100351363 | 0.117106332 | 0.33690942 | NS | 0.931419621 |
| SMCR8 | 0.299920186 | 0.058124806 | 0.252197542 | NS | 1.235638485 |
| SHMT1 | -0.307730453 | 0.004748489 | 0.095617668 | Down | 2.323444571 |
| LGALS9C | 0.499591681 | 0.075446876 | NA | NS | 1.122358741 |
| TBC1D28 | 0.898250468 | 0.081959711 | NA | NS | 1.086399581 |
| FOXO3B | -0.276838784 | 0.012447451 | 0.135361598 | Down | 1.90491957 |
| FBXW10 | 0.490329851 | 0.141200704 | NA | NS | 0.850163139 |
| TVP23B | -0.146618457 | 0.060709326 | 0.256526216 | NS | 1.216744589 |
| PRPSAP2 | -0.203256187 | 0.006667619 | 0.108607264 | Down | 2.176029215 |
| SLC5A10 | 0.09240164 | 0.390033741 | 0.597135686 | NS | 0.408897822 |
| FAM83G | 0.134106245 | 0.18409221 | 0.414354362 | NS | 0.734964588 |
| GRAP | -0.181082019 | 0.088052591 | 0.298002133 | NS | 1.055257861 |
| EPN2 | 0.045400799 | 0.723438329 | 0.836887361 | NS | 0.140598485 |
| B9D1 | 0.175989775 | 0.341511188 | 0.55696544 | NS | 0.466595064 |
| MAPK7 | 0.180027868 | 0.095601462 | 0.309159091 | NS | 1.019535466 |
| MFAP4 | 0.068742553 | 0.617689978 | 0.766154761 | NS | 0.209229445 |
| RNF112 | 0.341313754 | 0.161864464 | 0.38963426 | NS | 0.790848488 |
| SLC47A1 | 0.056349765 | 0.763422066 | 0.862228238 | NS | 0.117235291 |
| ALDH3A2 | -0.038691415 | 0.456974862 | 0.648589384 | NS | 0.340107689 |
| SLC47A2 | 0.551790966 | 0.001655612 | 0.064883573 | Up | 2.781041556 |
| ALDH3A1 | 0.958977057 | 0.055085749 | NA | NS | 1.258960739 |
| ULK2 | -0.027551796 | 0.658403647 | 0.794555206 | NS | 0.181507772 |
| AKAP10 | 0.017930703 | 0.62822783 | 0.773528315 | NS | 0.201882829 |
| SPECC1 | -0.347411851 | 0.032165707 | 0.198223945 | Down | 1.492606898 |
| LGALS9B | 0.197652839 | 0.492723423 | NA | NS | 0.307396792 |
| CDRT15L2 | 1.733094694 | 0.024295565 | NA | Up | 1.614472997 |
| USP22 | 0.143308955 | 0.027615896 | 0.186639939 | Up | 1.558840859 |
| DHRS7B | 0.120384061 | 0.037215586 | 0.209820891 | Up | 1.429275138 |
| TMEM11 | 0.102331249 | 0.159508324 | 0.387098757 | NS | 0.797216648 |
| NATD1 | 0.194854899 | 0.216934018 | 0.446581832 | NS | 0.66367234 |
| MAP2K3 | -0.172291769 | 0.467709687 | 0.657428788 | NS | 0.330023635 |
| KCNJ12 | 0.786316922 | 0.046205042 | 0.229024017 | Up | 1.335310634 |
| KCNJ18 | 0.813712173 | 0.07583249 | NA | NS | 1.120144685 |
| MTRNR2L1 | 1.541632298 | 0.092209042 | 0.304264722 | NS | 1.035226491 |
| WSB1 | 0.108689545 | 0.090908954 | 0.3020451 | NS | 1.041393337 |
| KSR1 | 0.214649693 | 0.110222274 | 0.328790965 | NS | 0.957730634 |
| LGALS9 | 0.049308361 | 0.661673381 | 0.79709872 | NS | 0.179356337 |
| NOS2 | 0.799672844 | 0.026764054 | NA | Up | 1.572448106 |
| LYRM9 | 0.10266186 | 0.184349484 | 0.414457849 | NS | 0.734358073 |
| NLK | -0.078189767 | 0.079515819 | 0.284900136 | NS | 1.099546463 |
| TMEM97 | -0.095743686 | 0.328658981 | 0.546356595 | NS | 0.483254495 |
| IFT20 | -0.074342655 | 0.341831339 | 0.557197247 | NS | 0.466188124 |
| TNFAIP1 | -0.015026357 | 0.722309824 | 0.836147372 | NS | 0.141276478 |
| POLDIP2 | -0.209841788 | 0.002103153 | 0.071967411 | Down | 2.677129113 |
| TMEM199 | -0.09682856 | 0.090606616 | 0.301422749 | NS | 1.042840091 |
| SEBOX | 0.204423391 | 0.172612854 | 0.401378778 | NS | 0.762926867 |
| SARM1 | 0.111747214 | 0.267602382 | 0.49359381 | NS | 0.572510024 |
| VTN | 0.146049028 | 0.287164424 | 0.510331314 | NS | 0.541869364 |
| SLC46A1 | 0.189991883 | 0.04468831 | 0.226077243 | Up | 1.349806067 |
| SLC13A2 | 0.646381779 | 0.19899209 | NA | NS | 0.701164186 |
| FOXN1 | 0.648990523 | 0.172064463 | NA | NS | 0.764308817 |
| UNC119 | 0.02165207 | 0.718322816 | 0.833717613 | NS | 0.143680339 |
| PIGS | 0.001609424 | 0.972453707 | 0.984309822 | NS | 0.012131064 |
| ALDOC | -0.024381534 | 0.732550032 | 0.841958287 | NS | 0.135162708 |
| SPAG5 | -0.146795293 | 0.108227275 | 0.325834237 | NS | 0.965663278 |
| RSKR | -0.007380875 | 0.951339416 | 0.97361348 | NS | 0.021664509 |
| KIAA0100 | -0.132415644 | 0.023567501 | 0.174335563 | Down | 1.627686469 |
| SDF2 | -0.06659702 | 0.402262639 | 0.606572225 | NS | 0.395490301 |
| SUPT6H | -0.038986384 | 0.642672892 | 0.783815879 | NS | 0.192010018 |
| PROCA1 | 0.162216349 | 0.192194232 | 0.42102173 | NS | 0.71625965 |
| RAB34 | -0.220946708 | 0.061703896 | 0.258215532 | NS | 1.209687411 |
| RPL23A | -0.178593631 | 0.087008068 | 0.29656937 | NS | 1.060440474 |
| TLCD1 | 9.00E-04 | 0.992305641 | 0.995609559 | NS | 0.00335454 |
| NEK8 | 0.279426819 | 0.042558685 | 0.221206584 | Up | 1.371011798 |
| TRAF4 | -0.133038979 | 0.093447138 | 0.306169721 | NS | 1.029433996 |
| FAM222B | 0.122502816 | 0.496369586 | 0.679085761 | NS | 0.304194837 |
| ERAL1 | -0.125223887 | 0.087478445 | 0.297087647 | NS | 1.058098945 |
| FLOT2 | 0.170455731 | 0.217079795 | 0.446641045 | NS | 0.663380596 |
| DHRS13 | 0.064979019 | 0.584202344 | 0.743210286 | NS | 0.233436705 |
| PHF12 | 0.09666133 | 0.353784542 | 0.567005458 | NS | 0.451261147 |
| PIPOX | 0.061826834 | 0.534093766 | 0.706592665 | NS | 0.272382491 |
| SEZ6 | 0.092324328 | 0.369609791 | 0.581054602 | NS | 0.432256533 |
| MYO18A | -0.125635238 | 0.256775554 | 0.483775638 | NS | 0.590446325 |
| TIAF1 | -0.032270175 | 0.817365061 | 0.896131678 | NS | 0.08758393 |
| CRYBA1 | 0.087486255 | 0.332665975 | 0.549373822 | NS | 0.477991616 |
| NUFIP2 | 0.06836604 | 0.238991827 | 0.467218862 | NS | 0.621616951 |
| TAOK1 | 0.007912089 | 0.872368214 | 0.92791681 | NS | 0.059300167 |
| ABHD15 | -0.145463528 | 0.026043334 | 0.182704458 | Down | 1.584303426 |
| TP53I13 | -0.072489617 | 0.290111535 | 0.512990599 | NS | 0.537435004 |
| GIT1 | 0.098063068 | 0.310409767 | 0.530362297 | NS | 0.508064622 |
| ANKRD13B | 0.545172157 | 0.036560906 | 0.208527349 | Up | 1.436983049 |
| CORO6 | 0.398169201 | 0.011097245 | 0.129535727 | Up | 1.954784842 |
| SSH2 | 0.232335919 | 0.027122823 | 0.185716026 | Up | 1.566665112 |
| EFCAB5 | 0.222995058 | 0.145445379 | 0.37059965 | NS | 0.837300072 |
| NSRP1 | 0.043303129 | 0.544638088 | 0.7135756 | NS | 0.26389199 |
| SLC6A4 | 0.15799232 | 0.198328077 | 0.428218122 | NS | 0.702615799 |
| BLMH | -0.040390168 | 0.492611794 | 0.676528862 | NS | 0.307495195 |
| TMIGD1 | 0.280707617 | 0.417002749 | NA | NS | 0.379861082 |
| CPD | 0.075176113 | 0.520372954 | 0.696688569 | NS | 0.283685284 |
| GOSR1 | -6.76E-05 | 0.999267954 | 0.999609713 | NS | 3.18E-04 |
| CRLF3 | -0.145299979 | 0.008086775 | 0.115679778 | Down | 2.092224631 |
| ATAD5 | -0.101242619 | 0.427608844 | 0.625597148 | NS | 0.368953321 |
| TEFM | 5.79E-04 | 0.992675486 | 0.995749068 | NS | 0.003192703 |
| ADAP2 | 0.178170215 | 0.10617577 | 0.322455574 | NS | 0.973974581 |
| RNF135 | -0.073690988 | 0.279911706 | 0.50348007 | NS | 0.55297894 |
| NF1 | 0.104999289 | 0.068564312 | 0.268602708 | NS | 1.163901875 |
| OMG | 0.144193876 | 0.16946246 | 0.397768704 | NS | 0.770926495 |
| EVI2B | 0.322275499 | 0.001546979 | 0.062836479 | Up | 2.810515645 |
| EVI2A | -0.152329414 | 0.370403899 | 0.581504668 | NS | 0.431324451 |
| RAB11FIP4 | -0.029290735 | 0.591068067 | 0.74812871 | NS | 0.228362503 |
| COPRS | -0.264949045 | 0.019968814 | 0.163807837 | Down | 1.699647722 |
| UTP6 | -0.085972719 | 0.120756407 | 0.341343277 | NS | 0.918089818 |
| SUZ12 | -0.120819856 | 0.187444039 | 0.416913012 | NS | 0.727128367 |
| LRRC37B | 0.11782771 | 0.056403413 | 0.249517281 | NS | 1.248694616 |
| RHOT1 | -0.172309463 | 0.081887999 | 0.288475384 | NS | 1.086779739 |
| RHBDL3 | 0.305798564 | 0.500490597 | NA | NS | 0.300604078 |
| C17orf75 | -0.083944357 | 0.203977722 | 0.434113724 | NS | 0.690417263 |
| ZNF207 | 0.023981605 | 0.532810225 | 0.705750131 | NS | 0.273427449 |
| PSMD11 | 0.035324328 | 0.357841665 | 0.570901415 | NS | 0.446309094 |
| CDK5R1 | 0.041717304 | 0.726778475 | 0.838611255 | NS | 0.138597944 |
| MYO1D | -0.069101697 | 0.512485549 | 0.690977514 | NS | 0.290318376 |
| TMEM98 | 0.595240307 | 0.076948649 | 0.281244486 | NS | 1.113799003 |
| SPACA3 | -0.050349703 | 0.920612867 | NA | NS | 0.035922959 |
| ASIC2 | 0.738464313 | 0.086107272 | NA | NS | 1.064960169 |
| CCL2 | 0.099233886 | 0.771021045 | NA | NS | 0.112933768 |
| CCL7 | 0.454618403 | 0.454050256 | NA | NS | 0.342896075 |
| CCL11 | 0.340917621 | 0.56787746 | NA | NS | 0.245745369 |
| CCL8 | 0.084003593 | 0.827694863 | NA | NS | 0.08212974 |
| CCL13 | 0.623407053 | 0.309599444 | NA | NS | 0.509199828 |
| CCL1 | 0.889805564 | 0.085332693 | NA | NS | 1.068884546 |
| TMEM132E | 0.67843842 | 0.013037531 | 0.137028702 | Up | 1.884804636 |
| CCT6B | 0.176569294 | 0.221780333 | 0.450609639 | NS | 0.654076969 |
| ZNF830 | -0.181982585 | 0.030075191 | 0.19315433 | Down | 1.52179161 |
| LIG3 | 0.02779341 | 0.576112971 | 0.737102752 | NS | 0.239492347 |
| RFFL | 0.15256561 | 0.093019678 | 0.305431813 | NS | 1.031425169 |
| RAD51D | -0.058317239 | 0.296539605 | 0.518186329 | NS | 0.527917296 |
| FNDC8 | 0.525087594 | 0.226646576 | NA | NS | 0.644650837 |
| NLE1 | -0.194365294 | 0.011049777 | 0.129467323 | Down | 1.956646475 |
| UNC45B | -0.331701636 | 0.021066159 | 0.168044994 | Down | 1.676414638 |
| SLC35G3 | 0.462365508 | 0.215744814 | NA | NS | 0.666059635 |
| SLFN5 | 0.014313455 | 0.819554455 | 0.897114813 | NS | 0.086422185 |
| SLFN11 | -0.036316828 | 0.539719761 | 0.710260413 | NS | 0.267831681 |
| SLFN12 | -0.050025672 | 0.439869662 | 0.635045158 | NS | 0.356675991 |
| SLFN13 | 0.191234187 | 0.012259694 | 0.134364821 | Up | 1.911520379 |
| SLFN12L | 0.028770339 | 0.716793412 | 0.832665621 | NS | 0.144605995 |
| SLFN14 | -0.183959331 | 0.22698581 | 0.455488754 | NS | 0.644001293 |
| PEX12 | -0.080095601 | 0.274710542 | 0.499348937 | NS | 0.561124675 |
| AP2B1 | -0.132287292 | 0.180332169 | 0.410166584 | NS | 0.743926793 |
| RASL10B | 0.790336733 | 0.026205857 | NA | Up | 1.581601637 |
| GAS2L2 | 0.430103286 | 0.273133246 | NA | NS | 0.563625434 |
| MMP28 | -0.108189401 | 0.673018167 | NA | NS | 0.171973212 |
| C17orf50 | 0.843609786 | 5.48E-04 | NA | Up | 3.260909826 |
| TAF15 | -0.015837107 | 0.830734478 | 0.903694533 | NS | 0.080537765 |
| HEATR9 | 0.468948994 | 0.185770356 | NA | NS | 0.731023588 |
| CCL5 | -0.230740203 | 0.052376202 | 0.241847979 | NS | 1.280865996 |
| CCL16 | 0.609635812 | 0.457058596 | NA | NS | 0.340028118 |
| CCL23 | 0.706531513 | 0.087746728 | NA | NS | 1.056769068 |
| CCL3 | -0.780662467 | 0.005324527 | NA | Down | 2.273718927 |
| CCL4 | -0.265527307 | 0.068028101 | 0.268137287 | NS | 1.167311653 |
| TBC1D3B | 0.553736428 | 0.147854578 | NA | NS | 0.830165224 |
| CCL3L3 | -0.667735863 | 0.202262538 | NA | NS | 0.694084547 |
| CCL4L2 | -0.13629235 | 0.552421959 | 0.719615932 | NS | 0.257729066 |
| TBC1D3F | -0.170609559 | 0.922514917 | NA | NS | 0.035026603 |
| ZNHIT3 | -0.257284921 | 0.105643393 | 0.321777494 | NS | 0.97615766 |
| MYO19 | -0.083488874 | 0.287002886 | 0.510123406 | NS | 0.542113737 |
| PIGW | 0.329275149 | 0.357322535 | NA | NS | 0.446939593 |
| GGNBP2 | -0.066665906 | 0.135756363 | 0.359806994 | NS | 0.867239804 |
| DHRS11 | 0.141223419 | 0.595718955 | NA | NS | 0.224958581 |
| MRM1 | 0.023935218 | 0.850989832 | 0.916327478 | NS | 0.070075629 |
| AATF | -0.213515817 | 8.51E-04 | 0.048491302 | Down | 3.069983515 |
| ACACA | 0.038600824 | 0.592677631 | 0.749296335 | NS | 0.227181464 |
| TADA2A | -0.200092609 | 0.016143626 | 0.150281882 | Down | 1.791998911 |
| DUSP14 | -0.019656149 | 0.932689212 | NA | NS | 0.030263047 |
| SYNRG | 0.005686471 | 0.917324429 | 0.954284082 | NS | 0.037477041 |
| DDX52 | -0.203378728 | 0.001105681 | 0.053762861 | Down | 2.956370135 |
| HNF1B | 0.813304619 | 0.230940448 | NA | NS | 0.636499995 |
| TBC1D3L | 0.436621229 | 0.035166935 | NA | Up | 1.453865482 |
| TBC1D3D | 0.89107393 | 0.373229585 | NA | NS | 0.428023938 |
| TBC1D3E | 0.237168831 | 0.429658592 | NA | NS | 0.366876499 |
| MRPL45 | -0.084117428 | 0.485075108 | 0.670966367 | NS | 0.314191011 |
| SOCS7 | -0.248806541 | 9.24E-04 | 0.050757919 | Down | 3.034233543 |
| ARHGAP23 | -0.357236835 | 0.209397366 | NA | NS | 0.679028786 |
| SRCIN1 | 0.609768129 | 0.258227873 | NA | NS | 0.587996882 |
| MLLT6 | -0.12380816 | 0.494109283 | 0.677463766 | NS | 0.306176987 |
| CISD3 | -0.141147289 | 0.346473569 | 0.561209256 | NS | 0.460329891 |
| PSMB3 | -0.036591847 | 0.747043069 | 0.851437263 | NS | 0.126654359 |
| PIP4K2B | -0.341143699 | 2.20E-04 | 0.024537741 | Down | 3.657309995 |
| CWC25 | -0.079526225 | 0.172412665 | 0.401144432 | NS | 0.763430836 |
| RPL23 | -0.330343647 | 0.160850386 | 0.388627022 | NS | 0.793577892 |
| LASP1 | 0.086870609 | 0.258632067 | 0.485189351 | NS | 0.587317629 |
| LINC00672 | 0.108770187 | 0.246244346 | 0.474504671 | NS | 0.608633732 |
| FBXO47 | 0.198283735 | 0.687962501 | NA | NS | 0.162435233 |
| PLXDC1 | 0.056319834 | 0.669268694 | 0.802573388 | NS | 0.174399489 |
| ARL5C | 0.553047127 | 0.172686635 | NA | NS | 0.762741274 |
| CACNB1 | 0.112114949 | 0.397186666 | 0.602351096 | NS | 0.40100534 |
| RPL19 | -0.28940727 | 0.013877144 | 0.141394243 | Down | 1.8576999 |
| STAC2 | 0.721769855 | 0.116457954 | NA | NS | 0.933830843 |
| FBXL20 | 0.172155429 | 0.027262918 | 0.185919029 | Up | 1.564427664 |
| MED1 | -0.070447616 | 0.137246816 | 0.361831079 | NS | 0.862497722 |
| CDK12 | 0.059846604 | 0.403393165 | 0.60738002 | NS | 0.394271465 |
| NEUROD2 | 0.71257406 | 0.059674081 | 0.254870821 | NS | 1.22421426 |
| PPP1R1B | 0.333581638 | 0.30772272 | 0.528573145 | NS | 0.511840437 |
| STARD3 | 0.039181486 | 0.518203523 | 0.69545251 | NS | 0.285499639 |
| TCAP | 0.036909283 | 0.881760907 | NA | NS | 0.05464916 |
| PNMT | 0.734268579 | 0.111717756 | NA | NS | 0.951877795 |
| PGAP3 | -0.028949157 | 0.688953772 | 0.815257617 | NS | 0.161809918 |
| ERBB2 | -0.02333802 | 0.832225245 | 0.904628723 | NS | 0.079759114 |
| MIEN1 | 0.305610695 | 0.002097039 | 0.071895774 | Up | 2.678393583 |
| GRB7 | 0.862716614 | 0.078130557 | NA | NS | 1.10717908 |
| IKZF3 | 0.014824191 | 0.827635176 | 0.901779381 | NS | 0.082161059 |
| ZPBP2 | 0.394714102 | 0.200823549 | NA | NS | 0.697185363 |
| GSDMB | -0.00833467 | 0.929893463 | 0.961767605 | NS | 0.031566805 |
| ORMDL3 | -0.282459712 | 5.76E-04 | 0.040235668 | Down | 3.239388354 |
| LRRC3C | 1.093460039 | 0.029869708 | NA | Up | 1.524769017 |
| GSDMA | 0.546195996 | 0.14199362 | NA | NS | 0.847731167 |
| PSMD3 | -0.283885401 | 0.001676389 | 0.064977566 | Down | 2.775625193 |
| CSF3 | 0.932432365 | 0.029473779 | NA | Up | 1.530564172 |
| MED24 | -0.044816333 | 0.533275218 | 0.705998557 | NS | 0.273048598 |
| THRA | 0.033651053 | 0.699480251 | 0.822044571 | NS | 0.155224543 |
| NR1D1 | 0.19610431 | 0.193600762 | 0.422794379 | NS | 0.713092937 |
| MSL1 | 0.113211368 | 0.187243401 | 0.416779897 | NS | 0.72759348 |
| CASC3 | -0.003941462 | 0.975156694 | 0.985701487 | NS | 0.010925594 |
| RAPGEFL1 | 0.133228721 | 0.460285619 | 0.651552088 | NS | 0.336972594 |
| WIPF2 | 0.085160285 | 0.350693319 | 0.564808607 | NS | 0.455072508 |
| CDC6 | 0.217151768 | 0.22316184 | 0.451870269 | NS | 0.651380066 |
| RARA | 0.104452174 | 0.446467328 | 0.640438462 | NS | 0.350210316 |
| GJD3 | 0.074508211 | 0.865920551 | NA | NS | 0.062521953 |
| TOP2A | -0.129031172 | 0.480329828 | 0.667102106 | NS | 0.318460443 |
| IGFBP4 | 0.046586361 | 0.699125747 | 0.821855116 | NS | 0.155444704 |
| TNS4 | 0.437365221 | 0.240317194 | 0.468217208 | NS | 0.619215155 |
| CCR7 | -0.033825999 | 0.788354393 | 0.878028124 | NS | 0.103278508 |
| SMARCE1 | 0.058558283 | 0.265716928 | 0.491781978 | NS | 0.575580778 |
| KRT222 | 0.421007018 | 0.262470717 | NA | NS | 0.580919143 |
| KRT24 | 0.471707752 | 0.301160329 | NA | NS | 0.521202237 |
| KRT25 | -0.062728326 | 0.926708771 | NA | NS | 0.033056726 |
| KRT26 | 0.805574977 | 0.158907593 | NA | NS | 0.798855351 |
| KRT27 | 0.79228325 | 0.151670224 | NA | NS | 0.819099671 |
| KRT28 | 0.506052808 | 0.322917308 | NA | NS | 0.490908677 |
| KRT10 | -0.051038063 | 0.599017362 | 0.753683091 | NS | 0.22256059 |
| KRT23 | -0.00707913 | 0.968855057 | 0.982627807 | NS | 0.013741189 |
| KRTAP4-8 | 0.223469289 | 0.764712256 | NA | NS | 0.116501949 |
| KRTAP4-12 | 0.182087685 | 0.823583698 | NA | NS | 0.084292259 |
| KRTAP4-6 | 0.306714507 | 0.453527952 | NA | NS | 0.343395942 |
| KRTAP4-5 | 0.728997308 | 0.205560398 | NA | NS | 0.687060551 |
| KRTAP4-4 | 0.032966948 | 0.935780081 | NA | NS | 0.028826204 |
| KRTAP4-3 | 0.864551655 | 0.070844686 | NA | NS | 1.149692721 |
| KRTAP4-2 | 0.830765227 | 0.120240762 | NA | NS | 0.919948279 |
| KRTAP4-1 | 0.573419625 | 0.305837931 | NA | NS | 0.514508652 |
| KRTAP9-1 | 0.553124525 | 0.608951471 | NA | NS | 0.215417316 |
| KRT31 | 0.630166199 | 0.385192312 | NA | NS | 0.414322389 |
| KRT38 | 1.078698624 | 0.043496029 | NA | Up | 1.361550386 |
| KRT32 | 0.702201219 | 0.258665575 | NA | NS | 0.587261366 |
| KRT35 | 0.649374635 | 0.280646561 | NA | NS | 0.551840275 |
| KRT36 | 0.172322772 | 0.724663275 | NA | NS | 0.139863748 |
| KRT13 | 0.086321323 | 0.793379004 | NA | NS | 0.100519297 |
| KRT15 | 0.209750753 | 0.596693382 | 0.75175978 | NS | 0.224248779 |
| KRT19 | 0.924238887 | 0.072501416 | NA | NS | 1.139653513 |
| KRT9 | 0.175003338 | 0.73686427 | 0.844971316 | NS | 0.132612501 |
| KRT14 | 0.670237861 | 0.218211102 | NA | NS | 0.661123157 |
| KRT16 | 1.053634674 | 0.053872968 | NA | NS | 1.268629097 |
| KRT17 | 0.569697976 | 0.2831791 | NA | NS | 0.547938803 |
| EIF1 | 0.018667929 | 0.890771919 | 0.938340394 | NS | 0.050233482 |
| GAST | 0.599635623 | 0.2444597 | NA | NS | 0.611792726 |
| HAP1 | 0.471811656 | 0.291068646 | 0.513839813 | NS | 0.536004575 |
| JUP | 0.184654425 | 0.325801071 | 0.544197807 | NS | 0.487047492 |
| P3H4 | 0.034705838 | 0.774394227 | 0.868392331 | NS | 0.111037893 |
| FKBP10 | 0.4026658 | 0.24977816 | 0.476907072 | NS | 0.602445538 |
| NT5C3B | -0.093579787 | 0.474074254 | 0.662229251 | NS | 0.32415363 |
| KLHL10 | 0.33444014 | 0.043909837 | 0.224569695 | Up | 1.357438172 |
| KLHL11 | -0.043739368 | 0.392916364 | 0.599318562 | NS | 0.405699884 |
| ACLY | -0.145330121 | 0.040022189 | 0.215862456 | Down | 1.397699164 |
| ODAD4 | -0.268101248 | 0.196761437 | 0.426481571 | NS | 0.706060015 |
| CNP | 0.030668396 | 0.568531291 | 0.73140927 | NS | 0.245245628 |
| DNAJC7 | -0.060615862 | 0.086694683 | 0.295990626 | NS | 1.062007536 |
| NKIRAS2 | -0.080928784 | 0.173388209 | 0.402364653 | NS | 0.760980439 |
| ZNF385C | 0.317892211 | 0.224959785 | 0.453236933 | NS | 0.647895111 |
| C17orf113 | 0.285373698 | 0.15760295 | 0.385109899 | NS | 0.802435659 |
| DHX58 | -0.054709104 | 0.524455314 | 0.699823783 | NS | 0.28029151 |
| KAT2A | -0.014083961 | 0.896504474 | 0.942008544 | NS | 0.047447539 |
| HSPB9 | -0.087095052 | 0.805524687 | NA | NS | 0.093921145 |
| RAB5C | 0.137204373 | 0.046592432 | 0.229846735 | Up | 1.331684624 |
| KCNH4 | 0.493976041 | 0.161848284 | 0.38963426 | NS | 0.7908919 |
| HCRT | 0.938850493 | 0.014794442 | NA | Up | 1.829901413 |
| GHDC | 0.1995773 | 0.014914292 | 0.146349507 | Up | 1.826397344 |
| STAT5B | 0.151401683 | 0.043133741 | 0.222949439 | Up | 1.365182878 |
| STAT5A | -0.172907881 | 0.059486653 | 0.254552191 | NS | 1.225580467 |
| STAT3 | 0.097534424 | 0.22930179 | 0.457791831 | NS | 0.639592556 |
| CAVIN1 | 0.190738373 | 0.445889745 | 0.639795791 | NS | 0.350772516 |
| ATP6V0A1 | -0.059375443 | 0.554045867 | 0.720989214 | NS | 0.25645428 |
| NAGLU | -0.071590901 | 0.283632889 | 0.506452552 | NS | 0.547243411 |
| HSD17B1 | 0.317815748 | 0.290058509 | NA | NS | 0.537514391 |
| COASY | 0.104136509 | 0.394814941 | 0.600481589 | NS | 0.403606421 |
| MLX | -0.018499148 | 0.744968548 | 0.850384773 | NS | 0.127862062 |
| PSMC3IP | 0.011945363 | 0.912436075 | 0.951241312 | NS | 0.039797552 |
| RETREG3 | -0.162636403 | 0.007954153 | 0.114752972 | Down | 2.099406078 |
| TUBG1 | -0.049929347 | 0.605005416 | 0.757720329 | NS | 0.218240738 |
| TUBG2 | 0.02636536 | 0.792021672 | 0.880056932 | NS | 0.101262935 |
| PLEKHH3 | 0.702155346 | 0.032054818 | 0.198142899 | Up | 1.494106686 |
| CCR10 | 0.541254671 | 0.015034178 | 0.146973997 | Up | 1.822920309 |
| CNTNAP1 | 0.068595636 | 0.596694144 | 0.75175978 | NS | 0.224248225 |
| EZH1 | -0.039805131 | 0.468877167 | 0.658100441 | NS | 0.328940915 |
| RAMP2 | 0.701585083 | 0.221273901 | NA | NS | 0.655069807 |
| VPS25 | -0.093091506 | 0.238596644 | 0.466720558 | NS | 0.622335669 |
| WNK4 | 0.321769105 | 0.103435229 | 0.31928753 | NS | 0.985331519 |
| COA3 | -0.247776489 | 0.026813775 | 0.185022315 | Down | 1.571642047 |
| CNTD1 | 0.303623802 | 0.007483354 | 0.112915927 | Up | 2.125903705 |
| BECN1 | -0.212196496 | 9.67E-04 | 0.051639949 | Down | 3.014531517 |
| PSME3 | 0.061146382 | 0.414349454 | 0.616091538 | NS | 0.382633229 |
| AOC2 | -0.013473689 | 0.930294917 | 0.961856154 | NS | 0.031379352 |
| AOC3 | 0.154467513 | 0.395678127 | 0.601155664 | NS | 0.402657956 |
| G6PC1 | 0.485994799 | 0.354075072 | NA | NS | 0.450904648 |
| AARSD1 | 0.172696472 | 0.193775757 | 0.423056963 | NS | 0.712700558 |
| PTGES3L | 0.202589247 | 0.338272323 | 0.554275792 | NS | 0.470733534 |
| RUNDC1 | 0.200739672 | 0.006533323 | 0.108189554 | Up | 2.184865898 |
| RPL27 | -0.456716489 | 0.027729829 | 0.186639939 | Down | 1.557052809 |
| IFI35 | 0.050484996 | 0.673693374 | 0.805795597 | NS | 0.171537724 |
| VAT1 | 0.092330605 | 0.213536203 | 0.443180155 | NS | 0.670528485 |
| RND2 | 0.638612983 | 0.034423432 | 0.203549976 | Up | 1.46314583 |
| BRCA1 | -0.242369044 | 0.042228152 | 0.220751111 | Down | 1.374397927 |
| NBR1 | -0.1735218 | 0.007876973 | 0.11472857 | Down | 2.103640652 |
| TMEM106A | 0.104294706 | 0.401007771 | 0.605756874 | NS | 0.396847211 |
| CCDC200 | 0.117634543 | 0.530827997 | 0.704673243 | NS | 0.27504618 |
| ARL4D | 0.081146387 | 0.676303928 | 0.806788244 | NS | 0.16985809 |
| DHX8 | -0.10700407 | 0.068541875 | 0.268602708 | NS | 1.164044022 |
| ETV4 | 0.236535231 | 0.132790417 | 0.356066752 | NS | 0.876833265 |
| MEOX1 | 0.164153404 | 0.303676038 | 0.524889707 | NS | 0.517589476 |
| SOST | 0.720885273 | 0.146533765 | NA | NS | 0.834062291 |
| DUSP3 | -0.011012111 | 0.895604607 | 0.941415199 | NS | 0.047883681 |
| CFAP97D1 | 0.505795413 | 0.17375997 | 0.402859976 | NS | 0.760050266 |
| MPP3 | 0.543965073 | 0.030145591 | 0.193469884 | Up | 1.520776195 |
| CD300LG | 0.582018479 | 0.227671028 | NA | NS | 0.642692232 |
| MPP2 | 0.239122165 | 0.359867853 | 0.572764191 | NS | 0.443856948 |
| PPY | 0.947311127 | 0.122742655 | NA | NS | 0.911004486 |
| PYY | 0.919291591 | 0.033706447 | NA | Up | 1.47228702 |
| NAGS | 0.025322613 | 0.781072498 | 0.873443474 | NS | 0.107308654 |
| TMEM101 | -0.141936411 | 0.074608774 | 0.277229083 | NS | 1.127210096 |
| LSM12 | 0.054081423 | 0.461309373 | 0.652436686 | NS | 0.336007721 |
| G6PC3 | 0.175832166 | 0.059768571 | 0.25508876 | NS | 1.223527129 |
| HDAC5 | 0.032639366 | 0.654094546 | 0.79213544 | NS | 0.184359472 |
| HROB | 0.063954447 | 0.753105864 | 0.855296001 | NS | 0.123143971 |
| ASB16 | -0.098539039 | 0.559044881 | 0.724213069 | NS | 0.252553325 |
| TMUB2 | 0.049776082 | 0.361687524 | 0.57427477 | NS | 0.441666471 |
| ATXN7L3 | 0.007982144 | 0.865258812 | 0.92378711 | NS | 0.062853969 |
| UBTF | -0.080498285 | 0.093806947 | 0.307041118 | NS | 1.027764997 |
| SLC4A1 | -0.174193446 | 0.461022789 | 0.652111908 | NS | 0.336277607 |
| RUNDC3A | 0.106697941 | 0.592610347 | 0.749252632 | NS | 0.22723077 |
| SLC25A39 | -0.287976232 | 0.172404692 | 0.401144432 | NS | 0.763450919 |
| GRN | 0.151237638 | 0.241623493 | 0.469551229 | NS | 0.616860842 |
| FAM171A2 | 0.509811952 | 0.089140352 | NA | NS | 1.049925654 |
| ITGA2B | 0.27872477 | 0.168601726 | 0.396805748 | NS | 0.773137983 |
| GPATCH8 | 0.035132423 | 0.59146181 | 0.748413779 | NS | 0.228073292 |
| FZD2 | 0.078206979 | 0.540554933 | 0.710828496 | NS | 0.267160165 |
| MEIOC | -0.08630569 | 0.429717786 | 0.627240072 | NS | 0.366816671 |
| CCDC43 | -0.152861109 | 0.052684871 | 0.242513843 | NS | 1.278314077 |
| DBF4B | 0.217883537 | 0.167314765 | 0.395361406 | NS | 0.776465733 |
| ADAM11 | 0.674688847 | 0.018670887 | 0.158900279 | Up | 1.728835052 |
| GJC1 | 0.470380191 | 0.31075943 | NA | NS | 0.507575684 |
| HIGD1B | 0.418563582 | 0.182911066 | NA | NS | 0.737760019 |
| EFTUD2 | -0.115916273 | 0.063763112 | 0.262219931 | NS | 1.195430493 |
| GFAP | 0.40002532 | 0.22653986 | 0.455187048 | NS | 0.644855372 |
| KIF18B | 0.130028402 | 0.639832827 | 0.781475729 | NS | 0.193933482 |
| NMT1 | -0.033935913 | 0.392935873 | 0.599318562 | NS | 0.405678321 |
| C1QL1 | 1.289986888 | 0.029269735 | NA | Up | 1.533581211 |
| DCAKD | -0.118072861 | 0.05607204 | 0.249087638 | NS | 1.251253647 |
| PLCD3 | 0.401075542 | 0.045187496 | 0.227297172 | Up | 1.344981728 |
| ACBD4 | 0.118419216 | 0.240896856 | 0.468815414 | NS | 0.618168868 |
| HEXIM1 | -0.105459251 | 0.239823355 | 0.467879246 | NS | 0.620108526 |
| HEXIM2 | 0.062465607 | 0.715562018 | NA | NS | 0.14535272 |
| FMNL1 | -0.048670029 | 0.784928019 | 0.875787854 | NS | 0.105170168 |
| SPATA32 | 0.130906927 | 0.541912476 | 0.711959588 | NS | 0.26607085 |
| MAP3K14 | 0.014720383 | 0.904992166 | 0.947388363 | NS | 0.04335518 |
| ARHGAP27 | 0.11883974 | 0.356130411 | 0.569162503 | NS | 0.448390939 |
| PLEKHM1 | 0.035958583 | 0.798985272 | 0.884435787 | NS | 0.097461226 |
| MAPT | 0.261891972 | 0.678800533 | NA | NS | 0.168257825 |
| KANSL1 | 0.0827902 | 0.329826814 | 0.547189614 | NS | 0.48171404 |
| ARL17B | 0.27839061 | 0.309508373 | 0.529970898 | NS | 0.509327598 |
| LRRC37A | -0.156942551 | 0.560185601 | 0.724761145 | NS | 0.251668059 |
| LRRC37A2 | 0.15652903 | 0.470101914 | 0.659132732 | NS | 0.32780798 |
| ARL17A | 0.350835324 | 0.110483166 | 0.329041981 | NS | 0.956703888 |
| NSF | -0.047021245 | 0.423278216 | 0.622593337 | NS | 0.373374082 |
| GOSR2 | -0.012277179 | 0.766108638 | 0.863854529 | NS | 0.115709641 |
| RPRML | 1.114368358 | 0.013141681 | NA | Up | 1.881349081 |
| CDC27 | -0.193341669 | 0.092008078 | 0.303889385 | NS | 1.036174043 |
| MYL4 | -0.172519194 | 0.496537785 | 0.679155378 | NS | 0.304047698 |
| ITGB3 | 0.021088221 | 0.912613488 | 0.951241312 | NS | 0.039713117 |
| EFCAB13 | -0.334974804 | 0.021416656 | 0.168379028 | Down | 1.669248331 |
| NPEPPS | 0.10586114 | 0.080599278 | 0.286282138 | NS | 1.093668847 |
| KPNB1 | 0.144762967 | 0.013179166 | 0.137758676 | Up | 1.880112074 |
| TBKBP1 | 0.020164859 | 0.878345557 | 0.931635325 | NS | 0.056334591 |
| TBX21 | -0.069428437 | 0.520184882 | 0.696688569 | NS | 0.283842274 |
| OSBPL7 | -0.081107088 | 0.410651779 | 0.613446418 | NS | 0.386526292 |
| MRPL10 | 0.078716509 | 0.253914413 | 0.480746129 | NS | 0.595312647 |
| LRRC46 | -0.006928564 | 0.948834829 | 0.972356151 | NS | 0.022809382 |
| SCRN2 | -0.205688663 | 0.003367173 | 0.085084692 | Down | 2.472734505 |
| SP6 | 0.381557972 | 0.32473631 | NA | NS | 0.488469148 |
| SP2 | -0.165058366 | 0.360773424 | 0.57346526 | NS | 0.442765461 |
| PNPO | -0.042550863 | 0.505276306 | 0.685819827 | NS | 0.296471067 |
| PRR15L | 0.184317124 | 0.399858194 | NA | NS | 0.398094 |
| CDK5RAP3 | -0.096305817 | 0.264095102 | 0.490257438 | NS | 0.578239654 |
| COPZ2 | -0.065754042 | 0.720065233 | 0.834772664 | NS | 0.142628158 |
| NFE2L1 | -0.126761797 | 0.076321769 | 0.280025819 | NS | 1.11735157 |
| CBX1 | -0.111138818 | 0.128363589 | 0.350057504 | NS | 0.891558149 |
| SNX11 | 0.13523355 | 0.017663955 | 0.155652138 | Up | 1.752912041 |
| SKAP1 | 0.172064915 | 0.050969757 | 0.238791409 | NS | 1.292687437 |
| HOXB1 | 0.633540312 | 0.161185649 | NA | NS | 0.792673627 |
| HOXB2 | 0.07806054 | 0.567130399 | 0.730541221 | NS | 0.246317073 |
| HOXB3 | 0.227274715 | 0.080794773 | 0.286611199 | NS | 1.092616733 |
| HOXB4 | 0.12794948 | 0.260502557 | 0.486665271 | NS | 0.58418801 |
| HOXB5 | 0.849978787 | 0.108710925 | NA | NS | 0.96372681 |
| HOXB6 | 0.702913914 | 0.097521379 | NA | NS | 1.010900166 |
| HOXB7 | 0.751397898 | 0.052736912 | NA | NS | 1.277885303 |
| HOXB8 | 0.675757533 | 0.184548349 | NA | NS | 0.733889837 |
| HOXB9 | 0.733637595 | 0.099152289 | NA | NS | 1.003697255 |
| PRAC1 | 0.258893832 | 0.776581854 | NA | NS | 0.109812761 |
| HOXB13 | 0.986171585 | 0.058052357 | NA | NS | 1.236180144 |
| TTLL6 | 0.539063302 | 0.227795937 | 0.456610283 | NS | 0.642454026 |
| CALCOCO2 | -0.038039553 | 0.627872419 | 0.773384292 | NS | 0.202128594 |
| ATP5MC1 | -0.114321479 | 0.166554725 | 0.394583254 | NS | 0.778443041 |
| UBE2Z | 0.111090812 | 0.027067173 | 0.185578254 | Up | 1.567557093 |
| SNF8 | -0.035898982 | 0.364816123 | 0.577163501 | NS | 0.437925977 |
| GIP | 0.734862696 | 0.198848168 | NA | NS | 0.701478405 |
| IGF2BP1 | 0.545772541 | 0.218342199 | 0.447821763 | NS | 0.66086232 |
| B4GALNT2 | 0.113311021 | 0.787225758 | 0.87719095 | NS | 0.103900704 |
| GNGT2 | -0.025776756 | 0.799948269 | 0.884720935 | NS | 0.096938097 |
| ABI3 | 0.148123149 | 0.097501071 | 0.311954391 | NS | 1.010990613 |
| PHOSPHO1 | 0.004973803 | 0.9778732 | 0.987153917 | NS | 0.009717456 |
| ZNF652 | 0.098359075 | 0.183605915 | 0.413733592 | NS | 0.736113332 |
| PHB | -0.374410994 | 8.70E-05 | 0.014340402 | Down | 4.060297286 |
| NGFR | 0.768148549 | 0.076600191 | NA | NS | 1.115770149 |
| NXPH3 | 0.627221817 | 0.026591748 | 0.184156096 | Up | 1.575253116 |
| SPOP | -0.175964774 | 1.29E-04 | 0.017955842 | Down | 3.889686614 |
| SLC35B1 | -0.020802837 | 0.692505413 | 0.817386949 | NS | 0.159576827 |
| FAM117A | 0.067954075 | 0.692405772 | 0.81735359 | NS | 0.15963932 |
| KAT7 | 0.0234452 | 0.556097914 | 0.722126301 | NS | 0.254848734 |
| TAC4 | 0.092780145 | 0.541856109 | 0.711926374 | NS | 0.266116026 |
| DLX4 | 0.506631517 | 0.244161933 | NA | NS | 0.612322045 |
| DLX3 | 0.77495069 | 0.161086237 | NA | NS | 0.792941565 |
| ITGA3 | 0.381119499 | 0.015397457 | 0.147618407 | Up | 1.812551011 |
| PDK2 | 0.033114189 | 0.805279171 | 0.887939507 | NS | 0.094053534 |
| SAMD14 | 0.420153471 | 0.034365032 | 0.203549976 | Up | 1.463883249 |
| PPP1R9B | -0.051103368 | 0.725416389 | 0.838334255 | NS | 0.139412637 |
| SGCA | 0.225800236 | 0.48161712 | 0.668380172 | NS | 0.317298084 |
| COL1A1 | 0.473999099 | 0.316798809 | 0.536323856 | NS | 0.49921646 |
| TMEM92 | 0.222653944 | 0.272048276 | 0.497401375 | NS | 0.565354022 |
| XYLT2 | -0.065936956 | 0.259958571 | 0.485926477 | NS | 0.585095859 |
| MRPL27 | -0.145878436 | 0.034281438 | 0.203291945 | Down | 1.46494097 |
| EME1 | 0.092124579 | 0.31114079 | 0.53090049 | NS | 0.50704305 |
| LRRC59 | -0.245208898 | 0.009059328 | 0.12055592 | Down | 2.042904 |
| ACSF2 | -0.031355858 | 0.676033536 | 0.806785925 | NS | 0.17003176 |
| CHAD | 0.378254273 | 0.06587235 | 0.265127437 | NS | 1.181296844 |
| RSAD1 | -0.142977642 | 0.016864651 | 0.152976731 | Down | 1.773022632 |
| MYCBPAP | 0.085383056 | 0.72720379 | 0.838717893 | NS | 0.138343866 |
| EPN3 | 0.419765601 | 0.081585027 | 0.287942716 | NS | 1.08838954 |
| SPATA20 | -0.055686666 | 0.617704717 | 0.766154761 | NS | 0.209219082 |
| CACNA1G | 0.845790078 | 0.036056044 | 0.207679449 | Up | 1.443021928 |
| ABCC3 | 0.125818186 | 0.455345579 | 0.64744052 | NS | 0.341658875 |
| ANKRD40 | 0.09428355 | 0.08377588 | 0.291497295 | NS | 1.076881003 |
| LUC7L3 | -0.08886991 | 0.17470337 | 0.404024697 | NS | 0.757698718 |
| ANKRD40CL | 0.434054246 | 0.111737192 | NA | NS | 0.951802247 |
| WFIKKN2 | 0.770175625 | 0.106422206 | NA | NS | 0.972967744 |
| TOB1 | 0.242558599 | 8.89E-05 | 0.014535159 | Up | 4.051325597 |
| SPAG9 | -0.028230491 | 0.691294868 | 0.816757897 | NS | 0.160336667 |
| NME1 | -0.088305606 | 0.48204146 | 0.668506329 | NS | 0.316915607 |
| NME2 | 0.237684302 | 0.232776463 | 0.461123308 | NS | 0.633060935 |
| MBTD1 | 0.031179171 | 0.320123393 | 0.539074418 | NS | 0.494682589 |
| UTP18 | -0.130140383 | 0.064165829 | 0.262840183 | NS | 1.19269619 |
| CA10 | 0.608316279 | 0.122698648 | 0.343568217 | NS | 0.911160221 |
| KIF2B | 0.044873516 | 0.929854056 | NA | NS | 0.03158521 |
| TOM1L1 | 0.171763827 | 0.405378244 | 0.60905444 | NS | 0.392139563 |
| COX11 | -0.143727378 | 0.044991277 | 0.226808328 | Down | 1.346871676 |
| STXBP4 | 0.175265726 | 0.027442065 | 0.186246285 | Up | 1.561583209 |
| HLF | 0.226607628 | 0.115565028 | 0.334479283 | NS | 0.937173571 |
| MMD | 0.088286585 | 0.41226798 | 0.614497968 | NS | 0.384820395 |
| SMIM36 | 0.396679228 | 0.357428526 | NA | NS | 0.446810789 |
| TMEM100 | 0.608652396 | 0.191460495 | NA | NS | 0.717920822 |
| PCTP | -0.200519626 | 0.084338509 | 0.29207932 | NS | 1.073974079 |
| ANKFN1 | 0.358387617 | 0.355811839 | 0.568903557 | NS | 0.448779605 |
| NOG | 0.042619299 | 0.841841389 | 0.910963576 | NS | 0.074769726 |
| C17orf67 | 0.010053648 | 0.900906231 | 0.94488709 | NS | 0.045320409 |
| DGKE | -0.056025141 | 0.32498151 | 0.543463056 | NS | 0.488141347 |
| TRIM25 | 0.242389492 | 0.026368354 | 0.183162888 | Up | 1.578916979 |
| COIL | -0.12488969 | 0.094101701 | 0.30725936 | NS | 1.026402527 |
| SCPEP1 | -0.224926687 | 0.028351324 | 0.188312653 | Down | 1.547426651 |
| AKAP1 | -0.062061501 | 0.341547819 | 0.55696544 | NS | 0.466548484 |
| MSI2 | -0.017685287 | 0.761039003 | 0.860451977 | NS | 0.118593085 |
| CCDC182 | 0.851657366 | 0.15079749 | NA | NS | 0.821605887 |
| MRPS23 | -0.197635622 | 0.056880196 | 0.250369065 | NS | 1.245038917 |
| CUEDC1 | 0.055755503 | 0.559458373 | 0.724373341 | NS | 0.252232222 |
| VEZF1 | -0.033619189 | 0.448150166 | 0.641635461 | NS | 0.348576438 |
| SRSF1 | -0.070690818 | 0.126086041 | 0.347906327 | NS | 0.899332991 |
| DYNLL2 | -0.15862335 | 0.019251659 | 0.161148205 | Down | 1.715531845 |
| OR4D1 | -0.077868694 | 0.513228628 | 0.691490884 | NS | 0.289689126 |
| OR4D2 | 0.146225664 | 0.549572007 | NA | NS | 0.259975397 |
| EPX | 0.420078754 | 0.150515876 | NA | NS | 0.822417688 |
| MKS1 | -0.051854685 | 0.522397204 | 0.698053606 | NS | 0.281999156 |
| LPO | 0.432787179 | 0.311238328 | NA | NS | 0.506906926 |
| MPO | 0.231616283 | 0.204227182 | 0.434295341 | NS | 0.689886455 |
| TSPOAP1 | 0.024374542 | 0.86865572 | 0.925923112 | NS | 0.061152316 |
| SUPT4H1 | 0.294782924 | 0.009267307 | 0.121488182 | Up | 2.033046472 |
| RNF43 | 0.054474333 | 0.697912663 | 0.821145431 | NS | 0.156198922 |
| HSF5 | -0.104013263 | 0.30881613 | 0.529238414 | NS | 0.510300024 |
| MTMR4 | -0.059314952 | 0.190896561 | 0.420174246 | NS | 0.719201896 |
| SEPTIN4 | 0.302331144 | 0.135867335 | 0.360059444 | NS | 0.866884942 |
| TEX14 | 0.21012357 | 0.380111795 | 0.590043886 | NS | 0.420088654 |
| IGBP1P2 | 0.914187821 | 0.114673861 | NA | NS | 0.940535565 |
| RAD51C | -0.236867223 | 0.061538322 | 0.258028313 | NS | 1.210854352 |
| PPM1E | 0.254370504 | 0.049237499 | 0.235025767 | Up | 1.307704013 |
| TRIM37 | -0.017674997 | 0.718922675 | 0.834077883 | NS | 0.143317819 |
| SKA2 | -0.215222178 | 0.032168492 | 0.198223945 | Down | 1.492569294 |
| PRR11 | 0.033467739 | 0.743288028 | 0.849440241 | NS | 0.128842862 |
| SMG8 | -0.090213822 | 0.236296769 | 0.46408699 | NS | 0.626542217 |
| GDPD1 | -0.109498946 | 0.296263314 | 0.517980183 | NS | 0.528322124 |
| YPEL2 | -0.175202279 | 0.04901199 | 0.234672133 | Down | 1.309697667 |
| DHX40 | -0.1237618 | 0.07534543 | 0.278406106 | NS | 1.122943083 |
| CLTC | 0.094552852 | 0.092250132 | 0.304276084 | NS | 1.035033004 |
| PTRH2 | 0.12582384 | 0.00196898 | 0.069745459 | Up | 2.705758597 |
| VMP1 | 0.10628131 | 0.196485909 | 0.426209538 | NS | 0.706668589 |
| TUBD1 | -0.048696776 | 0.396472507 | 0.601723881 | NS | 0.401786923 |
| RPS6KB1 | -0.067304891 | 0.193467335 | 0.422717401 | NS | 0.713392351 |
| RNFT1 | -0.027656899 | 0.634769963 | 0.77728181 | NS | 0.197383632 |
| HEATR6 | -0.119057194 | 0.058444031 | 0.252404602 | NS | 1.233259835 |
| CA4 | 0.203801197 | 0.263289761 | 0.48943686 | NS | 0.579566029 |
| USP32 | -0.094212868 | 0.326532883 | 0.544749339 | NS | 0.486073077 |
| C17orf64 | 0.247282143 | 0.49051981 | NA | NS | 0.309343449 |
| APPBP2 | -0.091055962 | 0.078686166 | 0.28356736 | NS | 1.104101615 |
| PPM1D | 0.045565865 | 0.311428719 | 0.531192767 | NS | 0.506641341 |
| BCAS3 | 0.069293561 | 0.273991697 | 0.498866181 | NS | 0.562262597 |
| TBX2 | 1.283372847 | 0.003633753 | NA | Up | 2.439644546 |
| TBX4 | 0.731551223 | 0.101818906 | NA | NS | 0.992171574 |
| NACA2 | 0.20035374 | 0.542069019 | NA | NS | 0.265945413 |
| BRIP1 | -0.30283943 | 0.012013432 | 0.134014426 | Down | 1.92033291 |
| INTS2 | -0.058795754 | 0.208926858 | 0.438906623 | NS | 0.680005728 |
| MED13 | -0.038186067 | 0.48836914 | 0.673043089 | NS | 0.311251786 |
| EFCAB3 | 0.5472881 | 0.207898666 | NA | NS | 0.682148297 |
| METTL2A | -0.168470679 | 0.005867715 | 0.102935441 | Down | 2.231530954 |
| TLK2 | -0.137018117 | 0.07492881 | 0.277897912 | NS | 1.125351164 |
| MRC2 | -0.401054154 | 0.023283352 | 0.173353487 | Down | 1.632954496 |
| MARCHF10 | 0.707857585 | 0.02214192 | 0.170412614 | Up | 1.654784728 |
| TANC2 | 0.1011163 | 0.310705627 | 0.530523732 | NS | 0.507650881 |
| CYB561 | 0.068233441 | 0.330143567 | 0.547441347 | NS | 0.48129716 |
| ACE | 0.295558711 | 0.169556066 | 0.397906857 | NS | 0.770686669 |
| KCNH6 | 0.962976051 | 0.022825851 | NA | Up | 1.641573024 |
| DCAF7 | 0.152688008 | 0.041051383 | 0.218237901 | Up | 1.38667221 |
| TACO1 | -0.022139076 | 0.850207724 | 0.915915955 | NS | 0.070474954 |
| MAP3K3 | 0.19111673 | 0.017634128 | 0.155449116 | Up | 1.753646006 |
| STRADA | 0.183115072 | 0.005968704 | 0.103371228 | Up | 2.224119968 |
| LIMD2 | 0.046124796 | 0.396944892 | 0.602161483 | NS | 0.401269783 |
| CCDC47 | -0.08972951 | 0.110706006 | 0.329185788 | NS | 0.955828819 |
| DDX42 | -0.040241417 | 0.395951334 | 0.601331396 | NS | 0.40235819 |
| FTSJ3 | -0.202165014 | 0.001361337 | 0.059956442 | Down | 2.866034214 |
| PSMC5 | -0.00139886 | 0.974775755 | 0.985403414 | NS | 0.011095281 |
| SMARCD2 | -0.263515224 | 0.001550362 | 0.062836479 | Down | 2.809566971 |
| CSH2 | 0.588302008 | 0.381711702 | NA | NS | 0.418264525 |
| GH2 | 0.368659564 | 0.580453136 | NA | NS | 0.236232838 |
| CSH1 | 0.512758959 | 0.491074638 | NA | NS | 0.308852495 |
| CSHL1 | 1.365753115 | 0.044187994 | NA | Up | 1.354695709 |
| GH1 | 1.183903532 | 0.095796776 | NA | NS | 1.018649106 |
| CD79B | -0.26952736 | 0.043154334 | 0.222962086 | Down | 1.364975579 |
| SCN4A | 0.722230852 | 0.016723931 | 0.152381397 | Up | 1.776661628 |
| PRR29 | -0.170401049 | 0.040244817 | 0.216510875 | Down | 1.395290045 |
| ICAM2 | -0.218813406 | 0.003171508 | 0.084307443 | Down | 2.49873416 |
| ERN1 | 0.11150216 | 0.217026808 | 0.446641045 | NS | 0.663486617 |
| TEX2 | -0.121193371 | 0.073601356 | 0.27580509 | NS | 1.133114186 |
| PECAM1 | 0.07432449 | 0.304182931 | 0.525408967 | NS | 0.51686516 |
| MILR1 | -0.160824061 | 0.126906259 | 0.348807379 | NS | 0.896516956 |
| POLG2 | -4.25E-04 | 0.994402405 | 0.996752621 | NS | 0.002437834 |
| DDX5 | -0.13173969 | 0.008856013 | 0.119278041 | Down | 2.052761743 |
| CEP95 | 0.007499886 | 0.888340601 | 0.937074009 | NS | 0.051420488 |
| SMURF2 | -0.029504814 | 0.374784425 | 0.585545419 | NS | 0.426218466 |
| LRRC37A3 | 0.148974138 | 0.318431424 | 0.537536335 | NS | 0.496984081 |
| GNA13 | -0.010365431 | 0.870121467 | 0.92673217 | NS | 0.060420116 |
| RGS9 | 0.191593409 | 0.194340459 | 0.423758145 | NS | 0.711436775 |
| AXIN2 | 0.091373816 | 0.360175825 | 0.572949 | NS | 0.44348544 |
| CEP112 | 0.199013445 | 0.270574153 | 0.495933513 | NS | 0.567713693 |
| APOH | 0.739147116 | 0.160680709 | NA | NS | 0.79403626 |
| PRKCA | 0.111503408 | 0.16439859 | 0.392193385 | NS | 0.784101913 |
| CACNG5 | 0.360915159 | 0.367576821 | 0.579649133 | NS | 0.434651883 |
| CACNG4 | 0.501488035 | 0.113111423 | NA | NS | 0.946493533 |
| CACNG1 | 0.758235784 | 0.002720229 | NA | Up | 2.565394504 |
| HELZ | 0.085283691 | 0.143669072 | 0.368744715 | NS | 0.842636713 |
| PSMD12 | -0.034379652 | 0.368274748 | 0.580219196 | NS | 0.433828058 |
| PITPNC1 | -0.02977979 | 0.518480041 | 0.695660549 | NS | 0.285267957 |
| NOL11 | -0.112776599 | 0.093840268 | 0.307106292 | NS | 1.027610758 |
| BPTF | 0.026293993 | 0.727011224 | 0.838611255 | NS | 0.138458884 |
| C17orf58 | 0.121673628 | 0.309638453 | 0.530051635 | NS | 0.509145112 |
| KPNA2 | 0.160756906 | 0.103126592 | 0.319138589 | NS | 0.986629333 |
| AMZ2 | -0.11476904 | 0.031577514 | 0.19723047 | Down | 1.500622058 |
| ARSG | 0.105433519 | 0.106152014 | 0.322426185 | NS | 0.97407176 |
| SLC16A6 | 0.068711783 | 0.334780542 | 0.55127581 | NS | 0.475239792 |
| WIPI1 | 0.066307491 | 0.447726592 | 0.641535901 | NS | 0.34898711 |
| PRKAR1A | -0.106641131 | 0.20354188 | 0.433508262 | NS | 0.691346219 |
| FAM20A | 0.083066257 | 0.46369503 | 0.654275373 | NS | 0.333767559 |
| ABCA8 | 0.477971832 | 0.24051393 | 0.468406361 | NS | 0.618859765 |
| ABCA9 | 0.224642136 | 0.343271678 | 0.558389993 | NS | 0.464362027 |
| ABCA6 | 0.272589694 | 0.12259317 | 0.343398823 | NS | 0.911533725 |
| ABCA10 | 0.120149797 | 0.290925191 | 0.513666543 | NS | 0.536218672 |
| ABCA5 | -0.0260817 | 0.707358972 | 0.826867416 | NS | 0.150360133 |
| MAP2K6 | 0.203878396 | 0.013532589 | 0.139789052 | Up | 1.868619098 |
| KCNJ16 | 0.428861461 | 0.241552665 | 0.469453424 | NS | 0.616988166 |
| KCNJ2 | 0.187933175 | 0.252684415 | 0.479607512 | NS | 0.597421543 |
| SOX9 | 0.830677164 | 0.057581224 | NA | NS | 1.239719104 |
| SLC39A11 | -0.052149048 | 0.53158222 | 0.704868508 | NS | 0.274429554 |
| SSTR2 | 0.501356655 | 0.027465403 | 0.186246285 | Up | 1.561214024 |
| COG1 | 0.092038378 | 0.089008801 | 0.299357611 | NS | 1.05056705 |
| FAM104A | -0.387539523 | 0.017718013 | 0.155687895 | Down | 1.75158499 |
| C17orf80 | -0.070199861 | 0.215508316 | 0.444806367 | NS | 0.666535967 |
| CPSF4L | 0.156503496 | 0.362123223 | 0.574723698 | NS | 0.441143623 |
| CDC42EP4 | 0.230516625 | 0.073070485 | 0.275014009 | NS | 1.136258011 |
| SDK2 | 0.313447886 | 0.131514252 | 0.354262456 | NS | 0.881027181 |
| RPL38 | -0.373948732 | 8.42E-04 | 0.048398623 | Down | 3.074457481 |
| TTYH2 | 0.127064169 | 0.153317885 | 0.38000933 | NS | 0.81440718 |
| DNAI2 | 0.214831304 | 0.23506237 | 0.463064159 | NS | 0.628816889 |
| KIF19 | 0.197860171 | 0.292532831 | 0.515264643 | NS | 0.533825386 |
| BTBD17 | 1.091861713 | 0.012493933 | NA | Up | 1.903300813 |
| GPR142 | 0.349602673 | 0.433851245 | NA | NS | 0.362659152 |
| GPRC5C | 0.554326277 | 0.0442011 | 0.224918162 | Up | 1.354566918 |
| CD300A | -0.021132153 | 0.786806136 | 0.876987431 | NS | 0.104132262 |
| CD300LB | 0.016317024 | 0.854200294 | 0.917756386 | NS | 0.068440283 |
| CD300C | -0.064491821 | 0.514252644 | 0.6923554 | NS | 0.288823467 |
| CD300LD | -0.108638729 | 0.708034887 | 0.82706671 | NS | 0.149945343 |
| CD300E | 0.180890039 | 0.072261163 | 0.273903534 | NS | 1.141095054 |
| RAB37 | 0.026527579 | 0.662370066 | 0.797602358 | NS | 0.178899303 |
| CD300LF | -0.00259285 | 0.969323429 | 0.982823994 | NS | 0.01353129 |
| SLC9A3R1 | -0.024783819 | 0.760204658 | 0.860029708 | NS | 0.119069474 |
| NAT9 | -0.128742904 | 0.015159242 | 0.147333918 | Down | 1.819322507 |
| TMEM104 | -0.003928762 | 0.968387197 | 0.982455456 | NS | 0.013950961 |
| GRIN2C | 0.366183752 | 0.213151866 | 0.442976773 | NS | 0.671310861 |
| FDXR | 0.056781662 | 0.591523121 | 0.748415144 | NS | 0.228028275 |
| FADS6 | 0.891782579 | 0.10908152 | NA | NS | 0.962248821 |
| USH1G | 1.073471132 | 0.025935171 | NA | Up | 1.58611089 |
| OTOP2 | 0.985146192 | 0.04566261 | NA | Up | 1.340439273 |
| OTOP3 | 0.914526791 | 0.094942517 | NA | NS | 1.022539257 |
| HID1 | 0.392700603 | 0.119006297 | 0.339550081 | NS | 0.924430058 |
| CDR2L | 0.90839841 | 0.004286905 | NA | Up | 2.367856154 |
| MRPL58 | -0.165844084 | 0.116245567 | 0.335719542 | NS | 0.934623601 |
| KCTD2 | -0.092708554 | 0.08446788 | 0.292350519 | NS | 1.073308406 |
| ATP5PD | -0.247864258 | 0.019129172 | 0.160710306 | Down | 1.718303832 |
| SLC16A5 | 0.162914051 | 0.110102192 | 0.328627707 | NS | 0.958204035 |
| ARMC7 | 0.063609124 | 0.404499832 | 0.608303576 | NS | 0.393081654 |
| NT5C | -0.169667982 | 0.026974093 | 0.185246086 | Down | 1.569053147 |
| JPT1 | -0.01377572 | 0.893665659 | 0.940364021 | NS | 0.04882493 |
| SUMO2 | -0.249182855 | 0.012167981 | 0.134186405 | Down | 1.914781472 |
| NUP85 | -0.047680947 | 0.482036453 | 0.668506329 | NS | 0.316920118 |
| GGA3 | -0.070268187 | 0.500148478 | 0.681890727 | NS | 0.300901049 |
| MRPS7 | -0.194441152 | 0.031875391 | 0.197995713 | Down | 1.496544486 |
| MIF4GD | 0.029943489 | 0.64357954 | 0.784420364 | NS | 0.191397771 |
| SLC25A19 | 0.024219332 | 0.723632293 | 0.836984289 | NS | 0.14048206 |
| GRB2 | -0.055609256 | 0.428155624 | 0.625957236 | NS | 0.368398347 |
| TMEM94 | 0.042020094 | 0.766615595 | 0.864133669 | NS | 0.11542235 |
| CASKIN2 | 0.908899278 | 0.002393499 | 0.075090296 | Up | 2.620966733 |
| TSEN54 | -0.030058925 | 0.602821671 | 0.756151929 | NS | 0.219811143 |
| LLGL2 | -0.026427384 | 0.726646212 | 0.838611255 | NS | 0.138676986 |
| MYO15B | 0.05156935 | 0.68205624 | 0.810483683 | NS | 0.166179813 |
| RECQL5 | -0.058548478 | 0.400498239 | 0.605341375 | NS | 0.397399389 |
| SMIM5 | -0.036646779 | 0.812402462 | 0.892507557 | NS | 0.090228769 |
| SMIM6 | -0.024098957 | 0.878079337 | 0.93143923 | NS | 0.056466242 |
| SAP30BP | -0.227346589 | 5.37E-06 | 0.001892282 | Down | 5.269974251 |
| ITGB4 | 0.364215564 | 0.092810128 | 0.305242354 | NS | 1.032404628 |
| GALK1 | 0.141089897 | 0.090947494 | 0.30205689 | NS | 1.041209265 |
| H3-3B | -0.057276856 | 0.576071529 | 0.737090907 | NS | 0.239523588 |
| UNK | 0.047935636 | 0.518530212 | 0.695687107 | NS | 0.285225934 |
| UNC13D | -0.043027415 | 0.68142639 | 0.810207571 | NS | 0.166581051 |
| WBP2 | -0.104173942 | 0.568799558 | 0.731587525 | NS | 0.24504075 |
| TRIM47 | -0.040534131 | 0.729900027 | 0.840372591 | NS | 0.136736621 |
| TRIM65 | 0.028199752 | 0.634682123 | 0.777257365 | NS | 0.197443734 |
| MRPL38 | 0.399576108 | 0.005513717 | 0.100822429 | Up | 2.258555499 |
| FBF1 | 0.00316901 | 0.971438892 | 0.98368013 | NS | 0.012584514 |
| ACOX1 | -0.027533399 | 0.793417952 | 0.880651596 | NS | 0.100497977 |
| TEN1-CDK3 | 0.214632281 | 0.315138524 | NA | NS | 0.501498504 |
| TEN1 | 0.287846412 | 0.42379086 | NA | NS | 0.372848414 |
| CDK3 | 0.099401391 | 0.545545441 | NA | NS | 0.263169069 |
| EVPL | -0.284155336 | 0.311959645 | 0.531703489 | NS | 0.505901583 |
| SRP68 | -0.054036757 | 0.212707387 | 0.442550518 | NS | 0.672217428 |
| ZACN | 0.130717238 | 0.204185648 | 0.434281871 | NS | 0.689974787 |
| GALR2 | 0.48488895 | 0.222514424 | NA | NS | 0.652641831 |
| EXOC7 | -0.1098308 | 0.053442721 | 0.244251686 | NS | 1.272111437 |
| FOXJ1 | 0.602597665 | 0.051856281 | NA | NS | 1.285198631 |
| RNF157 | 0.080162062 | 0.451176545 | 0.644192346 | NS | 0.345653486 |
| UBALD2 | 0.145732186 | 0.304790063 | 0.526026255 | NS | 0.515999197 |
| QRICH2 | -0.038233915 | 0.733000498 | 0.842188847 | NS | 0.13489573 |
| PRPSAP1 | 0.009010286 | 0.840283518 | 0.909982137 | NS | 0.075574155 |
| SPHK1 | -0.118387644 | 0.262284178 | 0.488519214 | NS | 0.581227906 |
| UBE2O | -0.024867278 | 0.89529465 | 0.941286236 | NS | 0.04803401 |
| AANAT | 0.148986936 | 0.446187009 | 0.640141257 | NS | 0.350483078 |
| RHBDF2 | 0.075222083 | 0.585425425 | 0.7442774 | NS | 0.23252842 |
| CYGB | 0.504921846 | 0.05621629 | 0.249221499 | NS | 1.250137819 |
| PRCD | 0.222973061 | 0.052293142 | 0.241796393 | NS | 1.281555264 |
| ST6GALNAC2 | 0.014241185 | 0.9137627 | 0.951994787 | NS | 0.039166574 |
| ST6GALNAC1 | -0.033456242 | 0.819013899 | 0.896951814 | NS | 0.086708728 |
| MXRA7 | -0.452126489 | 0.00209261 | 0.071851492 | Down | 2.679311784 |
| JMJD6 | -0.113912132 | 0.033319924 | 0.201295037 | Down | 1.477296002 |
| METTL23 | -0.086335694 | 0.276488225 | 0.500969068 | NS | 0.55832336 |
| SRSF2 | -0.057134932 | 0.289025017 | 0.512127733 | NS | 0.539064564 |
| MFSD11 | -0.071093427 | 0.123830718 | 0.344840845 | NS | 0.907171608 |
| MGAT5B | 0.993849805 | 0.016945827 | 0.153154431 | Up | 1.770937233 |
| SEC14L1 | -0.178666561 | 0.235661585 | 0.463448831 | NS | 0.627711205 |
| SEPTIN9 | 0.011750326 | 0.880085708 | 0.932281518 | NS | 0.055475032 |
| TNRC6C | -0.00145885 | 0.987079824 | 0.992295615 | NS | 0.005647725 |
| TMC6 | -0.077482236 | 0.353068463 | 0.566681193 | NS | 0.452141073 |
| TMC8 | -0.113126875 | 0.360941561 | 0.573615336 | NS | 0.442563108 |
| C17orf99 | -0.013586358 | 0.953809491 | 0.975032942 | NS | 0.02053836 |
| SYNGR2 | -0.039878166 | 0.602146544 | 0.755884683 | NS | 0.220297802 |
| TK1 | 0.48715501 | 5.24E-04 | 0.039237697 | Up | 3.280308897 |
| AFMID | -0.129463496 | 0.235257227 | 0.463195007 | NS | 0.628457026 |
| BIRC5 | 0.06586916 | 0.722681857 | 0.836344784 | NS | 0.141052848 |
| TMEM235 | 1.233551724 | 0.017708808 | NA | Up | 1.75181066 |
| SOCS3 | 0.043859602 | 0.762369197 | 0.861501199 | NS | 0.11783466 |
| PGS1 | 0.057880707 | 0.580313889 | 0.739791187 | NS | 0.236337035 |
| DNAH17 | 0.213027862 | 0.139497043 | 0.36380609 | NS | 0.855434998 |
| CYTH1 | -0.029690003 | 0.479129761 | 0.666445782 | NS | 0.319546852 |
| USP36 | 0.068898331 | 0.473855262 | 0.662084877 | NS | 0.324354292 |
| TIMP2 | 0.34132678 | 0.008926257 | 0.11966441 | Up | 2.049330633 |
| CEP295NL | -0.396865371 | 0.071560555 | 0.27278293 | NS | 1.145326297 |
| LGALS3BP | 0.239519267 | 0.034596222 | 0.204206875 | Up | 1.460971325 |
| CANT1 | 0.070916303 | 0.390343357 | 0.59729185 | NS | 0.408553208 |
| C1QTNF1 | 0.581885194 | 0.16242544 | 0.390045866 | NS | 0.789345948 |
| ENGASE | -0.086454237 | 0.301562997 | 0.523092915 | NS | 0.520621949 |
| RBFOX3 | 0.269881727 | 0.277600639 | 0.501982616 | NS | 0.556579539 |
| ENPP7 | 0.44521142 | 0.12871075 | 0.350545083 | NS | 0.890385178 |
| CBX2 | 0.517170702 | 0.058262044 | 0.252197542 | NS | 1.234614282 |
| CBX8 | -0.004766873 | 0.956754304 | 0.976333091 | NS | 0.019199576 |
| CBX4 | 0.216299961 | 0.002579079 | 0.077799913 | Up | 2.58853536 |
| TBC1D16 | 0.184272975 | 0.234407059 | 0.46252024 | NS | 0.630029313 |
| CCDC40 | 0.643620323 | 0.039044557 | 0.214333278 | Up | 1.408439505 |
| GAA | 0.01264086 | 0.908310765 | 0.949280874 | NS | 0.041765538 |
| EIF4A3 | -0.194241601 | 0.020866514 | 0.167612586 | Down | 1.680550098 |
| CARD14 | 0.461277488 | 0.010231594 | 0.126233693 | Up | 1.990056707 |
| SGSH | 0.139976747 | 0.358347276 | 0.571412376 | NS | 0.445695893 |
| SLC26A11 | -0.142368431 | 0.092624984 | 0.304959372 | NS | 1.033271853 |
| RNF213 | 0.07744216 | 0.469750211 | 0.658841285 | NS | 0.328133016 |
| ENDOV | 0.065980484 | 0.398412103 | 0.603427946 | NS | 0.399667477 |
| NPTX1 | 0.697118981 | 0.034715471 | 0.204367256 | Up | 1.459476939 |
| RPTOR | 0.120187824 | 0.192253563 | 0.42102173 | NS | 0.716125602 |
| CHMP6 | 0.157663398 | 0.07601742 | 0.279614057 | NS | 1.119086872 |
| BAIAP2 | 0.278910847 | 0.145580658 | 0.370771051 | NS | 0.836896323 |
| AATK | -0.115576277 | 0.545771758 | 0.714406996 | NS | 0.262988941 |
| PVALEF | 0.996447125 | 0.022421524 | NA | Up | 1.649334873 |
| CEP131 | 0.016985626 | 0.877448203 | 0.93118249 | NS | 0.056778511 |
| TEPSIN | 0.143872928 | 0.079399054 | 0.284881269 | NS | 1.100184671 |
| NDUFAF8 | -0.034225829 | 0.739357114 | 0.846891898 | NS | 0.131145744 |
| SLC38A10 | -0.176201746 | 0.124735185 | 0.346235762 | NS | 0.904011023 |
| BAHCC1 | 0.533081976 | 0.044091551 | 0.224627854 | Up | 1.355644621 |
| ACTG1 | -0.18307529 | 0.035676242 | 0.206431857 | Down | 1.447620901 |
| FSCN2 | 0.543063381 | 0.003602959 | 0.086766518 | Up | 2.443340724 |
| FAAP100 | 0.188121839 | 0.029716499 | 0.192180034 | Up | 1.52700236 |
| NPLOC4 | -0.049403315 | 0.407617674 | 0.611075752 | NS | 0.389746994 |
| TSPAN10 | 0.784098585 | 0.006592788 | 0.108312793 | Up | 2.18093088 |
| PDE6G | 0.177806532 | 0.399384178 | NA | NS | 0.398609144 |
| OXLD1 | -0.14416804 | 0.064995808 | 0.264064926 | NS | 1.187114655 |
| CCDC137 | -0.227905379 | 5.46E-05 | 0.010772251 | Down | 4.26310895 |
| ARL16 | -0.047369017 | 0.572486538 | 0.734073901 | NS | 0.242234721 |
| HGS | -0.022508866 | 0.818258733 | 0.896553512 | NS | 0.087109351 |
| MRPL12 | 0.774673087 | 3.77E-05 | 0.00829096 | Up | 4.423742307 |
| SLC25A10 | 0.394645502 | 0.123815125 | NA | NS | 0.907226299 |
| MCRIP1 | -0.063281561 | 0.506403428 | 0.686535921 | NS | 0.295503363 |
| P4HB | -0.236499252 | 0.003162662 | 0.084307443 | Down | 2.499947278 |
| ARHGDIA | -0.063176779 | 0.683480327 | 0.811586377 | NS | 0.165273981 |
| ALYREF | -0.292459203 | 0.001885796 | 0.067799826 | Down | 2.724505402 |
| ANAPC11 | -0.11070611 | 0.212016673 | 0.441739979 | NS | 0.673629985 |
| PCYT2 | -0.163574035 | 0.018203398 | 0.157022309 | Down | 1.739847524 |
| SIRT7 | -0.115338395 | 0.049286646 | 0.235025767 | Down | 1.307270736 |
| MAFG | -0.008641225 | 0.933175926 | 0.963088299 | NS | 0.030036474 |
| PYCR1 | 0.302089218 | 0.235686403 | NA | NS | 0.627665472 |
| MYADML2 | 1.173663049 | 0.045638538 | NA | Up | 1.340668277 |
| NOTUM | 1.115311996 | 0.017201425 | NA | Up | 1.76443558 |
| ASPSCR1 | 0.224632123 | 0.07653854 | 0.280416761 | NS | 1.116119824 |
| CENPX | -0.148658262 | 0.134139259 | 0.357714894 | NS | 0.872444097 |
| LRRC45 | 0.013623907 | 0.853213058 | 0.917168996 | NS | 0.068942506 |
| RAC3 | 0.268217244 | 0.253962882 | NA | NS | 0.595229753 |
| DCXR | -0.216767739 | 0.028406097 | 0.18856708 | Down | 1.54658844 |
| RFNG | 0.206348384 | 0.071944947 | 0.273508834 | NS | 1.142999705 |
| GPS1 | -0.144610816 | 0.065642689 | 0.265047401 | NS | 1.182813638 |
| DUS1L | -0.140886568 | 0.041438689 | 0.219327214 | Down | 1.382593994 |
| FASN | 0.13863141 | 0.432270125 | 0.629114552 | NS | 0.364244778 |
| CCDC57 | -0.083364922 | 0.405674172 | 0.609189554 | NS | 0.391822642 |
| SLC16A3 | 0.122987899 | 0.191926912 | 0.420934764 | NS | 0.716864124 |
| CSNK1D | -0.012783048 | 0.9186527 | 0.954971819 | NS | 0.036848644 |
| CD7 | 0.277824401 | 0.113315291 | 0.332115589 | NS | 0.94571148 |
| SECTM1 | 0.172855137 | 0.117597191 | 0.337537394 | NS | 0.929603053 |
| TEX19 | 0.729636595 | 0.331241826 | NA | NS | 0.47985483 |
| UTS2R | 1.108730817 | 0.020980191 | NA | Up | 1.678190562 |
| OGFOD3 | -0.113578036 | 0.129642725 | 0.351703113 | NS | 0.88725185 |
| HEXD | 0.075035754 | 0.465980449 | 0.656166026 | NS | 0.331632304 |
| CYBC1 | 0.264758037 | 0.005255491 | 0.099062758 | Up | 2.279386718 |
| NARF | 0.109348762 | 0.220123173 | 0.449270199 | NS | 0.657334236 |
| FOXK2 | 0.043147899 | 0.389623203 | 0.59694612 | NS | 0.409355187 |
| WDR45B | -0.17537339 | 5.51E-04 | 0.040103819 | Down | 3.259163929 |
| RAB40B | 0.232923129 | 0.115356918 | 0.334375919 | NS | 0.937956355 |
| FN3KRP | -0.133641845 | 0.056256439 | 0.249221499 | NS | 1.249827765 |
| FN3K | 0.03895566 | 0.871270419 | 0.927222822 | NS | 0.059847031 |
| TBCD | -0.010720062 | 0.88381051 | 0.934370959 | NS | 0.053640838 |
| ZNF750 | 0.117034657 | 0.543953977 | 0.713285597 | NS | 0.264437843 |
| B3GNTL1 | 0.011398449 | 0.912444522 | 0.951241312 | NS | 0.039793532 |
| METRNL | 0.073648804 | 0.525713581 | 0.700849434 | NS | 0.279250803 |
| TUBB8B | 0.614649626 | 0.141173606 | NA | NS | 0.850246492 |
| USP14 | -0.083772307 | 0.38177147 | 0.591316309 | NS | 0.41819653 |
| THOC1 | -0.092148503 | 0.086722512 | 0.296018897 | NS | 1.061868152 |
| COLEC12 | 0.35906903 | 0.160895635 | 0.388654343 | NS | 0.793455739 |
| CETN1 | 0.931332279 | 0.143518209 | NA | NS | 0.843092994 |
| CLUL1 | 0.410311592 | 0.351580339 | NA | NS | 0.45397542 |
| TYMS | 0.149233843 | 0.449754189 | 0.643129524 | NS | 0.347024783 |
| ENOSF1 | -0.029972899 | 0.746792458 | 0.851314627 | NS | 0.126800077 |
| YES1 | -0.047630521 | 0.51187149 | 0.690515454 | NS | 0.290839059 |
| ADCYAP1 | 0.963201252 | 0.062350018 | NA | NS | 1.205163419 |
| METTL4 | 0.042946948 | 0.370074418 | 0.581306195 | NS | 0.431710935 |
| NDC80 | -0.291903097 | 0.032878901 | 0.200078102 | Down | 1.483082713 |
| SMCHD1 | 0.008308787 | 0.932604628 | 0.962852453 | NS | 0.030302434 |
| EMILIN2 | -0.049353732 | 0.618245972 | 0.766432243 | NS | 0.208838704 |
| LPIN2 | 0.024734735 | 0.802080402 | 0.886076183 | NS | 0.095782095 |
| MYOM1 | 0.024300425 | 0.761803897 | 0.861061733 | NS | 0.11815681 |
| MYL12A | -0.109591852 | 0.198447371 | 0.428314173 | NS | 0.70235465 |
| MYL12B | -0.116846346 | 0.295079472 | 0.517117263 | NS | 0.530061002 |
| TGIF1 | -0.0827098 | 0.110549977 | 0.329041981 | NS | 0.956441345 |
| DLGAP1 | 0.482215901 | 0.094352071 | 0.307427263 | NS | 1.025248565 |
| AKAIN1 | 0.247558947 | 0.643492881 | NA | NS | 0.191456253 |
| ZBTB14 | -0.127335492 | 0.048863668 | 0.234288536 | Down | 1.311013936 |
| EPB41L3 | -0.101986887 | 0.309177055 | 0.529536849 | NS | 0.509792744 |
| TMEM200C | 0.419919249 | 0.367250866 | 0.579396578 | NS | 0.435037172 |
| L3MBTL4 | 0.068903259 | 0.466246379 | 0.656254399 | NS | 0.331384528 |
| ARHGAP28 | 0.457356113 | 0.277995365 | 0.502260165 | NS | 0.555962445 |
| LAMA1 | 0.384705413 | 0.338744052 | 0.554595725 | NS | 0.470128322 |
| LRRC30 | 0.761632359 | 0.255402252 | NA | NS | 0.592775278 |
| PTPRM | -0.028717764 | 0.77595393 | 0.869613999 | NS | 0.110164063 |
| RAB12 | -0.172101627 | 0.010451443 | 0.127471706 | Down | 1.980823736 |
| MTCL1 | 0.266299313 | 0.225973982 | 0.454435762 | NS | 0.645941561 |
| NDUFV2 | 0.276637745 | 8.41E-06 | 0.002751291 | Up | 5.075238896 |
| ANKRD12 | -0.0884349 | 0.184327139 | 0.414457849 | NS | 0.734410717 |
| TWSG1 | -0.17813672 | 0.03069895 | 0.194491094 | Down | 1.512876482 |
| RALBP1 | -0.178146152 | 0.15152569 | 0.377980759 | NS | 0.81951373 |
| PPP4R1 | 0.071326073 | 0.455511556 | 0.647513534 | NS | 0.341500601 |
| RAB31 | 0.006485265 | 0.949641878 | 0.972791443 | NS | 0.022440142 |
| TXNDC2 | 0.176613082 | 0.615366076 | NA | NS | 0.210866449 |
| VAPA | -0.044733468 | 0.490061813 | 0.674615197 | NS | 0.309749138 |
| APCDD1 | 0.49973637 | 0.005743252 | 0.102208347 | Up | 2.240842101 |
| NAPG | -0.026781585 | 0.560015356 | 0.724761145 | NS | 0.251800064 |
| PIEZO2 | 0.425754369 | 0.267791518 | 0.493757454 | NS | 0.572203183 |
| SLC35G4 | 0.867841823 | 0.16226594 | NA | NS | 0.78977263 |
| GNAL | 0.173885129 | 0.023791776 | 0.174977279 | Up | 1.623573142 |
| CHMP1B | -0.050003161 | 0.497682541 | 0.679732613 | NS | 0.303047595 |
| MPPE1 | 0.013566017 | 0.854693267 | 0.918113753 | NS | 0.068189717 |
| IMPA2 | 0.050063561 | 0.617815991 | 0.766154761 | NS | 0.209140855 |
| ANKRD62 | 0.538177553 | 0.175560832 | NA | NS | 0.755572369 |
| CIDEA | 0.960957886 | 0.033439036 | NA | Up | 1.475746254 |
| TUBB6 | -0.027499134 | 0.79543948 | 0.881887737 | NS | 0.099392857 |
| AFG3L2 | -0.208385198 | 3.07E-04 | 0.029282103 | Down | 3.513059897 |
| PRELID3A | 0.625804966 | 0.03640098 | NA | Up | 1.438886922 |
| SPIRE1 | -0.002838049 | 0.979126998 | 0.9877094 | NS | 0.009160974 |
| PSMG2 | -0.127769769 | 0.140966419 | 0.365370409 | NS | 0.850884331 |
| CEP76 | -0.173741717 | 0.04620041 | 0.229024017 | Down | 1.335354173 |
| PTPN2 | -0.035590343 | 0.481992884 | 0.668506329 | NS | 0.316959373 |
| SEH1L | -0.081413339 | 0.114177149 | 0.332664071 | NS | 0.942420805 |
| CEP192 | -0.183284063 | 0.024575853 | 0.17769403 | Down | 1.609491399 |
| LDLRAD4 | 0.083585584 | 0.204048078 | 0.434170494 | NS | 0.690267493 |
| FAM210A | -0.182786442 | 0.034075432 | 0.202909595 | Down | 1.467558632 |
| RNMT | -0.044434644 | 0.332083128 | 0.548846634 | NS | 0.478753189 |
| MC5R | 0.941029578 | 0.129685451 | NA | NS | 0.887108744 |
| MC2R | 0.22784879 | 0.645912171 | NA | NS | 0.189826532 |
| ZNF519 | 0.03439516 | 0.659056551 | 0.794908002 | NS | 0.181077319 |
| POTEC | 0.525488747 | 0.277474301 | NA | NS | 0.556777234 |
| ANKRD30B | 0.365937734 | 0.303148095 | 0.524458861 | NS | 0.518345157 |
| ROCK1 | 0.046672358 | 0.37225573 | 0.583052983 | NS | 0.429158609 |
| GREB1L | 0.097850234 | 0.280509668 | 0.503714282 | NS | 0.552052166 |
| ESCO1 | -0.181470752 | 0.00774647 | 0.114236734 | Down | 2.110896178 |
| SNRPD1 | -0.290987968 | 0.00572104 | 0.102180893 | Down | 2.242525034 |
| ABHD3 | 0.002513066 | 0.975869073 | 0.986116904 | NS | 0.010608445 |
| MIB1 | -0.11318097 | 0.01780915 | 0.156031046 | Down | 1.749356807 |
| GATA6 | 0.543805483 | 0.072008328 | NA | NS | 1.142617275 |
| RBBP8 | -0.266381621 | 0.032869431 | 0.200078102 | Down | 1.48320781 |
| CABLES1 | 0.491516886 | 0.039833522 | 0.215796828 | Up | 1.399751296 |
| TMEM241 | 0.281541046 | 0.013210245 | 0.137913995 | Up | 1.87908912 |
| RIOK3 | -0.088077778 | 0.613892299 | 0.763888152 | NS | 0.211907814 |
| RMC1 | -0.131063059 | 0.042088365 | 0.220751111 | Down | 1.375837943 |
| NPC1 | 0.067020048 | 0.395787448 | 0.601183351 | NS | 0.402537983 |
| ANKRD29 | 0.645842941 | 0.037401001 | 0.210115856 | Up | 1.427116776 |
| LAMA3 | 0.66168956 | 0.072256401 | 0.273903534 | NS | 1.141123675 |
| TTC39C | -0.033646015 | 0.595759507 | 0.75107807 | NS | 0.224929019 |
| CABYR | 0.655658377 | 2.82E-04 | 0.028091276 | Up | 3.549830606 |
| OSBPL1A | 0.092651389 | 0.346056484 | 0.560904975 | NS | 0.460853008 |
| IMPACT | -0.110590335 | 0.14783513 | 0.373699795 | NS | 0.830222353 |
| HRH4 | 0.226888668 | 0.182228604 | 0.412275698 | NS | 0.739383451 |
| ZNF521 | 0.076879918 | 0.702766455 | 0.82383594 | NS | 0.153188977 |
| SS18 | -0.042943197 | 0.279048899 | 0.503208948 | NS | 0.554319686 |
| PSMA8 | 0.083202626 | 0.727074713 | 0.838611255 | NS | 0.13842096 |
| TAF4B | -0.11609957 | 0.273966475 | 0.498866181 | NS | 0.562302579 |
| KCTD1 | 0.104045491 | 0.460876169 | 0.652105895 | NS | 0.336415748 |
| AQP4 | 0.407256315 | 0.425117067 | NA | NS | 0.371491459 |
| CHST9 | 0.503069162 | 0.248498258 | 0.476119605 | NS | 0.604676651 |
| CDH2 | 0.209512501 | 0.32437336 | 0.542854027 | NS | 0.488954821 |
| DSC3 | 0.610390935 | 0.137984124 | 0.362607036 | NS | 0.860170878 |
| DSC2 | 0.30153455 | 0.06752058 | 0.267434379 | NS | 1.170563838 |
| DSC1 | -0.208042997 | 0.273008158 | 0.49836066 | NS | 0.563824375 |
| DSG1 | 0.393659818 | 0.397357204 | 0.602512142 | NS | 0.400818908 |
| DSG4 | 0.440533796 | 0.33934452 | NA | NS | 0.469359159 |
| DSG3 | 0.304200392 | 0.428858948 | 0.626665452 | NS | 0.367685524 |
| DSG2 | 0.204753976 | 0.470899654 | 0.659963583 | NS | 0.327071628 |
| TTR | 0.223220809 | 0.684921329 | NA | NS | 0.164359309 |
| B4GALT6 | -0.114806188 | 0.297821632 | 0.519442518 | NS | 0.52604376 |
| SLC25A52 | 0.352840787 | 0.19270336 | NA | NS | 0.715110713 |
| TRAPPC8 | -0.068758988 | 0.180160673 | 0.410020841 | NS | 0.744340005 |
| RNF125 | -0.204843304 | 3.20E-04 | 0.030136408 | Down | 3.495175656 |
| RNF138 | -0.051167103 | 0.532448664 | 0.705393608 | NS | 0.273722258 |
| GAREM1 | 0.143387424 | 0.354084985 | 0.567279071 | NS | 0.450892489 |
| MEP1B | 0.125955941 | 0.708499296 | NA | NS | 0.149660577 |
| KLHL14 | -0.075532196 | 0.52624422 | 0.7012711 | NS | 0.278812661 |
| CCDC178 | 0.48002783 | 0.362878104 | NA | NS | 0.440239237 |
| ASXL3 | 0.33826136 | 0.342452629 | 0.557733617 | NS | 0.465399495 |
| NOL4 | 0.760730205 | 0.103927851 | 0.319840889 | NS | 0.983268053 |
| DTNA | 0.287138951 | 0.281589372 | 0.504671794 | NS | 0.550383741 |
| MAPRE2 | 0.075796271 | 0.112122361 | 0.330787614 | NS | 0.950307767 |
| ZNF397 | 0.011535033 | 0.832200707 | 0.904628723 | NS | 0.079771919 |
| ZSCAN30 | 0.024700193 | 0.664448356 | 0.799096584 | NS | 0.177538769 |
| ZNF24 | -0.100079442 | 0.128563234 | 0.350315619 | NS | 0.890883212 |
| ZNF396 | 0.184696814 | 0.089167483 | 0.299549938 | NS | 1.049793494 |
| INO80C | 0.148568082 | 0.032098926 | 0.198223945 | Up | 1.493509492 |
| GALNT1 | -0.193917428 | 0.022117349 | 0.170377235 | Down | 1.655266936 |
| C18orf21 | -0.143770602 | 0.074775554 | 0.277611674 | NS | 1.126240363 |
| RPRD1A | -0.042783572 | 0.20860692 | 0.438465095 | NS | 0.68067129 |
| SLC39A6 | -0.127358145 | 0.113985987 | 0.332506314 | NS | 0.943148536 |
| ELP2 | -0.088582504 | 0.076459914 | 0.280308141 | NS | 1.116566192 |
| MOCOS | 0.287325446 | 0.410032981 | 0.613001849 | NS | 0.38718121 |
| FHOD3 | 0.361405884 | 0.256409868 | 0.483396346 | NS | 0.591065264 |
| TPGS2 | -0.229978732 | 0.046385107 | 0.229315052 | Down | 1.333621435 |
| KIAA1328 | -0.034713869 | 0.700754926 | 0.822549366 | NS | 0.15443384 |
| CELF4 | 0.675122735 | 0.063145982 | 0.261340232 | NS | 1.199654278 |
| PIK3C3 | -0.05881254 | 0.205203732 | 0.435266821 | NS | 0.687814746 |
| RIT2 | 0.213349101 | 0.719825778 | NA | NS | 0.142772604 |
| SYT4 | 0.689183267 | 0.165837109 | NA | NS | 0.780318282 |
| SETBP1 | 0.139940763 | 0.079950535 | 0.285340058 | NS | 1.097178626 |
| SLC14A2 | 0.278261539 | 0.237145352 | 0.464991683 | NS | 0.624985382 |
| SLC14A1 | 0.122852664 | 0.539890097 | 0.710280503 | NS | 0.267694639 |
| SIGLEC15 | 0.302101456 | 0.003059722 | 0.083025772 | Up | 2.514318042 |
| EPG5 | 0.136403827 | 0.033123578 | 0.200739925 | Up | 1.479862761 |
| PSTPIP2 | 0.218092676 | 0.015485397 | 0.147954346 | Up | 1.81007765 |
| ATP5F1A | -0.216211504 | 0.016633948 | 0.152056591 | Down | 1.779004667 |
| HAUS1 | -0.453383359 | 0.00783001 | 0.11472857 | Down | 2.106237672 |
| C18orf25 | 0.133149974 | 0.011669337 | 0.132368086 | Up | 1.932953803 |
| RNF165 | 0.31234882 | 0.109787241 | 0.328012756 | NS | 0.959448128 |
| LOXHD1 | -0.042340418 | 0.790790551 | 0.878951525 | NS | 0.101938529 |
| ST8SIA5 | 0.462173864 | 0.233976552 | 0.46228643 | NS | 0.630827664 |
| PIAS2 | -0.008134117 | 0.867785889 | 0.925449706 | NS | 0.061587416 |
| KATNAL2 | 0.109242073 | 0.224540786 | 0.452878298 | NS | 0.648704762 |
| ELOA3CP | 0.519256829 | 0.406768409 | NA | NS | 0.390652783 |
| ELOA2 | 0.799677311 | 0.021790779 | NA | Up | 1.661727245 |
| HDHD2 | 0.102834886 | 0.126592657 | 0.348597283 | NS | 0.897591483 |
| IER3IP1 | -0.239396813 | 0.042482261 | 0.221095683 | Down | 1.371792379 |
| SKOR2 | 0.724492957 | 0.130635817 | NA | NS | 0.883937736 |
| SMAD2 | 0.017537047 | 0.60293731 | 0.756214144 | NS | 0.219727841 |
| ZBTB7C | 0.650145896 | 0.060280617 | 0.256083601 | NS | 1.219822311 |
| CTIF | -0.40213956 | 0.121444801 | 0.342148251 | NS | 0.915621072 |
| SMAD7 | 0.171305999 | 0.026690826 | 0.184563192 | Up | 1.573637988 |
| DYM | 0.022865978 | 0.49486355 | 0.677832837 | NS | 0.305514533 |
| C18orf32 | 0.170723021 | 0.015637911 | 0.148636863 | Up | 1.805821263 |
| RPL17 | -0.197005518 | 0.051225264 | 0.239227656 | NS | 1.290515794 |
| LIPG | 0.476463909 | 0.244544161 | 0.472420951 | NS | 0.611642703 |
| ACAA2 | -0.071015503 | 0.318660961 | 0.53773033 | NS | 0.496671139 |
| MYO5B | 0.051371611 | 0.606119087 | 0.758543133 | NS | 0.217442039 |
| CFAP53 | 0.076848275 | 0.685209082 | 0.812880518 | NS | 0.164176889 |
| MBD1 | -0.011201907 | 0.777458231 | 0.870549474 | NS | 0.109322934 |
| CXXC1 | -0.148214575 | 0.015292064 | 0.147398508 | Down | 1.815533887 |
| SKA1 | -0.254009157 | 0.321664146 | NA | NS | 0.492597344 |
| MAPK4 | 0.665600249 | 0.148558418 | 0.374706999 | NS | 0.828102733 |
| MRO | 0.48663661 | 0.27377667 | 0.498811736 | NS | 0.562603563 |
| ME2 | 0.007233649 | 0.88955162 | 0.937615575 | NS | 0.050828845 |
| ELAC1 | -0.116102736 | 0.206427484 | 0.436355477 | NS | 0.685232481 |
| SMAD4 | 0.035484942 | 0.284085606 | 0.506905224 | NS | 0.54655077 |
| MEX3C | -0.16586767 | 0.027583425 | 0.186639939 | Down | 1.559351815 |
| DCC | 0.457471888 | 0.28185118 | 0.504917658 | NS | 0.549980143 |
| MBD2 | -0.186302296 | 0.01008533 | 0.126199476 | Down | 1.99630987 |
| POLI | -0.008823762 | 0.872656587 | 0.928087369 | NS | 0.059156629 |
| STARD6 | 0.526824739 | 0.081146462 | NA | NS | 1.090730411 |
| C18orf54 | -0.036928104 | 0.594266119 | 0.750186454 | NS | 0.22601903 |
| DYNAP | 0.427144292 | 0.378303597 | NA | NS | 0.422159529 |
| RAB27B | -0.17348964 | 0.117689085 | 0.337758824 | NS | 0.929263815 |
| CCDC68 | -0.573094032 | 0.026363865 | 0.183162888 | Down | 1.578990928 |
| TCF4 | 0.030491571 | 0.694428937 | 0.81860261 | NS | 0.15837219 |
| TXNL1 | -0.173190058 | 0.015646349 | 0.148636863 | Down | 1.805586973 |
| WDR7 | 0.013821337 | 0.742686377 | 0.849009739 | NS | 0.129194542 |
| BOD1L2 | 0.559883069 | 0.356223961 | NA | NS | 0.448276871 |
| ST8SIA3 | 0.730826968 | 0.088105528 | 0.29803361 | NS | 1.054996841 |
| ONECUT2 | 0.789165314 | 0.061430015 | 0.257725124 | NS | 1.211619377 |
| FECH | -0.583988523 | 0.019844596 | 0.163600051 | Down | 1.702357735 |
| NARS1 | -0.242016242 | 1.96E-04 | 0.023041407 | Down | 3.707329812 |
| ATP8B1 | 0.272970214 | 0.049466197 | 0.235329216 | Up | 1.305691476 |
| NEDD4L | 0.08447986 | 0.559383338 | 0.724358091 | NS | 0.252290474 |
| ALPK2 | 0.176544586 | 0.479841637 | 0.666828469 | NS | 0.31890207 |
| MALT1 | -0.134185903 | 0.038308194 | 0.213411396 | Down | 1.416708326 |
| ZNF532 | 0.015210807 | 0.858306952 | 0.920010569 | NS | 0.06635737 |
| SEC11C | -0.094029275 | 0.41450495 | 0.616134093 | NS | 0.382470279 |
| GRP | 1.303981558 | 0.041984877 | NA | Up | 1.376907112 |
| RAX | 0.996881161 | 0.044986239 | NA | Up | 1.346920313 |
| CPLX4 | 0.788979811 | 0.078808981 | NA | NS | 1.103424286 |
| LMAN1 | -0.124190645 | 0.095669447 | 0.309291599 | NS | 1.019226735 |
| CCBE1 | 0.512494605 | 0.222081707 | 0.45081681 | NS | 0.653487214 |
| PMAIP1 | -0.130785066 | 0.251432056 | 0.478279044 | NS | 0.599579354 |
| MC4R | 0.731930417 | 0.191460476 | NA | NS | 0.717920865 |
| CDH20 | 0.333579214 | 0.267376525 | 0.493349756 | NS | 0.572876726 |
| RNF152 | 0.079563449 | 0.728092258 | 0.839150968 | NS | 0.137813587 |
| PIGN | -0.010570342 | 0.797269676 | 0.883232725 | NS | 0.098394754 |
| RELCH | 0.027639234 | 0.551529116 | 0.718943583 | NS | 0.258431556 |
| TNFRSF11A | 0.384406764 | 0.042053285 | 0.220751111 | Up | 1.376200077 |
| ZCCHC2 | 0.033307113 | 0.757327913 | 0.858374931 | NS | 0.120716036 |
| PHLPP1 | 0.097381075 | 0.264998663 | 0.49119273 | NS | 0.576756317 |
| BCL2 | -0.053747254 | 0.494378814 | 0.677614675 | NS | 0.305940149 |
| KDSR | -0.078414453 | 0.196372439 | 0.426159043 | NS | 0.706919465 |
| VPS4B | -0.098237752 | 0.156566931 | 0.383636723 | NS | 0.805299961 |
| SERPINB5 | 0.861449448 | 0.097647214 | NA | NS | 1.010340143 |
| SERPINB12 | 0.467828644 | 0.333685651 | NA | NS | 0.476662468 |
| SERPINB13 | 0.041966225 | 0.934215165 | NA | NS | 0.029553087 |
| SERPINB4 | 0.412854541 | 0.421184064 | NA | NS | 0.37552807 |
| SERPINB11 | 0.238176658 | 0.66004067 | NA | NS | 0.180429304 |
| SERPINB3 | 1.294972424 | 0.020584794 | NA | Up | 1.686453466 |
| SERPINB7 | 0.717648917 | 0.138087232 | NA | NS | 0.859846477 |
| SERPINB2 | -0.054082426 | 0.744454769 | 0.850164936 | NS | 0.128161683 |
| SERPINB10 | 0.185521835 | 0.420758861 | 0.620717816 | NS | 0.375966729 |
| HMSD | 0.365786126 | 0.361940313 | NA | NS | 0.441363042 |
| SERPINB8 | 0.006710195 | 0.919360071 | 0.955316894 | NS | 0.036514362 |
| CDH7 | 0.586226217 | 0.199674881 | 0.429206416 | NS | 0.699676566 |
| CDH19 | 0.437708594 | 0.412494448 | 0.614635514 | NS | 0.384581893 |
| DSEL | 0.068863237 | 0.828572834 | NA | NS | 0.08166931 |
| TMX3 | -0.153296225 | 0.019742619 | 0.163256521 | Down | 1.704595245 |
| CCDC102B | -0.095652389 | 0.369426625 | 0.580846393 | NS | 0.432471808 |
| DOK6 | 0.185786091 | 0.157364344 | 0.384750529 | NS | 0.803093665 |
| CD226 | -0.067667101 | 0.295435213 | 0.5174029 | NS | 0.529537743 |
| RTTN | 0.030117568 | 0.594711861 | 0.750384214 | NS | 0.2256934 |
| SOCS6 | -0.150348186 | 0.094884612 | 0.308146253 | NS | 1.022804215 |
| CBLN2 | 0.683447788 | 0.146523923 | 0.372104233 | NS | 0.834091462 |
| NETO1 | 0.442243051 | 0.017603905 | 0.155449116 | Up | 1.754390979 |
| FBXO15 | 0.387782833 | 0.069577116 | 0.270000522 | NS | 1.157533577 |
| TIMM21 | -0.101683497 | 0.079562229 | 0.284900136 | NS | 1.099293058 |
| CYB5A | -0.084491117 | 0.375945607 | 0.586177342 | NS | 0.424874986 |
| C18orf63 | 0.480199383 | 0.316579403 | NA | NS | 0.499517344 |
| DIPK1C | 0.539912295 | 0.29935677 | NA | NS | 0.523810915 |
| CNDP2 | -0.099638157 | 0.170701434 | 0.399000229 | NS | 0.767762831 |
| CNDP1 | 0.260822638 | 0.42442144 | 0.623190546 | NS | 0.372202685 |
| ZNF407 | 0.045212664 | 0.464314654 | 0.654907569 | NS | 0.33318761 |
| ZADH2 | -0.013453206 | 0.805680546 | 0.888253784 | NS | 0.093837123 |
| TSHZ1 | 0.115702703 | 0.080583924 | 0.286282138 | NS | 1.093751587 |
| SMIM21 | 0.51349022 | 0.319675623 | NA | NS | 0.49529048 |
| ZNF516 | 0.1810805 | 0.06854785 | 0.268602708 | NS | 1.164006163 |
| ZNF236 | -0.161179948 | 0.072944217 | 0.274846078 | NS | 1.137009131 |
| MBP | -0.015711431 | 0.852764145 | 0.91698772 | NS | 0.069171068 |
| GALR1 | 0.332350984 | 0.394180717 | 0.600085288 | NS | 0.404304625 |
| ATP9B | -0.10343812 | 0.042133008 | 0.220751111 | Down | 1.375377539 |
| NFATC1 | 0.198873625 | 0.032130886 | 0.198223945 | Up | 1.493077299 |
| CTDP1 | -0.153215916 | 0.065222581 | 0.26442336 | NS | 1.185602016 |
| KCNG2 | 0.333032059 | 0.031719607 | 0.197517887 | Up | 1.498672199 |
| SLC66A2 | 0.040291618 | 0.745676044 | 0.850639911 | NS | 0.127449809 |
| HSBP1L1 | -0.05876825 | 0.419208118 | 0.619480212 | NS | 0.377570316 |
| TXNL4A | -0.113246122 | 0.059640932 | 0.25483146 | NS | 1.224455578 |
| RBFA | -0.026740782 | 0.691605989 | 0.81688906 | NS | 0.160141254 |
| ADNP2 | 0.017274339 | 0.633568639 | 0.776709642 | NS | 0.198206328 |
| PARD6G | 0.195916239 | 0.031983527 | 0.198142899 | Up | 1.49507365 |
| OR4F17 | 0.909020184 | 0.111730779 | NA | NS | 0.951827173 |
| PLPP2 | 0.492736532 | 0.166876947 | 0.394815686 | NS | 0.777603655 |
| MIER2 | 0.295364061 | 0.030458284 | 0.193793197 | Up | 1.516294563 |
| THEG | 0.915283274 | 0.064065197 | NA | NS | 1.193377831 |
| C2CD4C | 0.874594729 | 0.078327466 | NA | NS | 1.106085921 |
| SHC2 | 0.542973476 | 0.141514388 | 0.365923729 | NS | 0.849199402 |
| ODF3L2 | 0.886441186 | 0.057999754 | NA | NS | 1.236573847 |
| MADCAM1 | 0.397423365 | 0.098378989 | 0.313080008 | NS | 1.007097643 |
| TPGS1 | -0.005169256 | 0.961290588 | 0.978517825 | NS | 0.01714531 |
| CDC34 | -0.155412523 | 0.348305666 | 0.562612118 | NS | 0.458039461 |
| GZMM | -0.188673857 | 0.117827916 | 0.337950518 | NS | 0.928751803 |
| BSG | -0.064891594 | 0.70054718 | 0.822549366 | NS | 0.154562611 |
| HCN2 | 0.297366387 | 0.19277389 | 0.421806458 | NS | 0.71495179 |
| POLRMT | -0.052291361 | 0.504046757 | 0.684785733 | NS | 0.297529175 |
| FGF22 | 0.921864242 | 0.008912872 | NA | Up | 2.049982335 |
| RNF126 | 0.064465687 | 0.524846204 | 0.700100632 | NS | 0.27996794 |
| FSTL3 | 0.295956316 | 0.011837864 | 0.133168118 | Up | 1.92672665 |
| PRSS57 | -0.159269124 | 0.497734517 | 0.679732613 | NS | 0.30300224 |
| PALM | 0.943652748 | 0.038695601 | 0.213650843 | Up | 1.412338404 |
| MISP | 1.081614294 | 0.058527448 | NA | NS | 1.23264041 |
| PTBP1 | -0.091801182 | 0.219354889 | 0.448781997 | NS | 0.658852681 |
| PLPPR3 | 0.748638076 | 0.059582001 | NA | NS | 1.224884913 |
| AZU1 | 0.314270359 | 0.300493146 | NA | NS | 0.522165429 |
| PRTN3 | 0.885616342 | 0.13045774 | NA | NS | 0.884530148 |
| ELANE | 0.484926679 | 0.118473657 | NA | NS | 0.926378206 |
| CFD | 0.623959091 | 0.002556084 | 0.077575819 | Up | 2.592424848 |
| MED16 | 0.056710457 | 0.715881211 | 0.832021589 | NS | 0.145159036 |
| R3HDM4 | -0.029863119 | 0.880690512 | 0.932527873 | NS | 0.055176682 |
| KISS1R | 0.970160338 | 7.24E-05 | NA | Up | 4.139978095 |
| ARID3A | -0.016154381 | 0.872896387 | 0.928176853 | NS | 0.059037304 |
| WDR18 | -0.066356917 | 0.534094211 | 0.706592665 | NS | 0.272382129 |
| GRIN3B | 0.408745287 | 0.114534913 | 0.333276311 | NS | 0.941062108 |
| TMEM259 | 0.013999502 | 0.892877413 | 0.939752341 | NS | 0.049208163 |
| CNN2 | 0.2117093 | 0.042590802 | 0.221206584 | Up | 1.370684179 |
| ABCA7 | 0.047536789 | 0.714590967 | 0.831203328 | NS | 0.145942478 |
| ARHGAP45 | -0.078044447 | 0.369017531 | 0.580590828 | NS | 0.432953001 |
| POLR2E | 4.34E-05 | 0.999495898 | 0.999687252 | NS | 2.19E-04 |
| GPX4 | -0.084136229 | 0.430418157 | 0.627698475 | NS | 0.366109417 |
| SBNO2 | 0.042128418 | 0.757443162 | 0.858378114 | NS | 0.120649951 |
| STK11 | -0.043724546 | 0.720116748 | 0.834790127 | NS | 0.142597089 |
| CBARP | 1.336171335 | 0.003600847 | NA | Up | 2.443595379 |
| ATP5F1D | 0.183112309 | 0.216339074 | 0.445797865 | NS | 0.664865034 |
| MIDN | -0.037507812 | 0.817594951 | 0.896255005 | NS | 0.087461799 |
| CIRBP | -0.246719816 | 2.92E-05 | 0.006982755 | Down | 4.533988283 |
| FAM174C | 0.095403497 | 0.236293493 | 0.46408699 | NS | 0.626548238 |
| EFNA2 | 1.126373229 | 0.049629767 | NA | Up | 1.304257767 |
| PWWP3A | -0.013243725 | 0.805166552 | 0.887901589 | NS | 0.094114275 |
| NDUFS7 | 0.175478939 | 0.082976612 | 0.290260272 | NS | 1.081044302 |
| GAMT | 0.012043504 | 0.919434129 | 0.95533019 | NS | 0.03647938 |
| DAZAP1 | -0.073448784 | 0.328391535 | 0.546122207 | NS | 0.483608046 |
| RPS15 | -0.073509084 | 0.455622555 | 0.647513534 | NS | 0.341394785 |
| APC2 | 0.360949883 | 0.004300585 | 0.092915457 | Up | 2.366472466 |
| C19orf25 | 0.027960262 | 0.620166735 | 0.767649906 | NS | 0.207491533 |
| PCSK4 | 0.51294039 | 0.126976576 | 0.348807379 | NS | 0.896276387 |
| REEP6 | 0.599045769 | 0.015605428 | 0.148543437 | Up | 1.806724319 |
| ADAMTSL5 | 0.272622127 | 0.123811462 | 0.34482915 | NS | 0.907239149 |
| PLK5 | 0.594279779 | 0.175163334 | 0.404501491 | NS | 0.756556798 |
| MEX3D | 0.040478029 | 0.718253564 | 0.833717613 | NS | 0.143722211 |
| MBD3 | 0.042622393 | 0.537153853 | 0.708735809 | NS | 0.269901305 |
| UQCR11 | -0.104507687 | 0.190648771 | 0.420149522 | NS | 0.719765989 |
| TCF3 | 0.122299676 | 0.396721721 | 0.601902673 | NS | 0.401514021 |
| ONECUT3 | 0.465221963 | 0.334944231 | 0.551386772 | NS | 0.475027499 |
| ATP8B3 | 0.504766939 | 0.005204153 | 0.098827133 | Up | 2.283649907 |
| REXO1 | 0.171387619 | 0.387164215 | 0.595130169 | NS | 0.41210479 |
| KLF16 | 0.035393925 | 0.736500275 | 0.844718014 | NS | 0.132827087 |
| ABHD17A | -0.039953503 | 0.598918351 | 0.753608487 | NS | 0.22263238 |
| SCAMP4 | 0.380039006 | 0.025905917 | 0.182272603 | Up | 1.586601022 |
| ADAT3 | 0.779978707 | 0.003404525 | NA | Up | 2.467943455 |
| CSNK1G2 | -0.066940317 | 0.381589079 | 0.591122367 | NS | 0.418404064 |
| BTBD2 | 0.01418345 | 0.892891928 | 0.939752341 | NS | 0.049201103 |
| MKNK2 | -0.104517022 | 0.453666617 | 0.646056016 | NS | 0.343263177 |
| MOB3A | 0.012975934 | 0.933193708 | 0.963088299 | NS | 0.030028198 |
| IZUMO4 | -0.002548279 | 0.98399357 | 0.990437337 | NS | 0.00700774 |
| AP3D1 | -0.159191836 | 0.080384764 | 0.286107685 | NS | 1.09482626 |
| DOT1L | 0.017674518 | 0.915826852 | 0.953375752 | NS | 0.038186627 |
| PLEKHJ1 | -0.108807177 | 0.093000517 | 0.305431813 | NS | 1.031514635 |
| SF3A2 | -0.117404123 | 0.497851308 | 0.679810772 | NS | 0.302900348 |
| AMH | 0.722756544 | 0.012955735 | NA | Up | 1.887537931 |
| JSRP1 | 0.713330791 | 0.003909348 | NA | Up | 2.407895706 |
| OAZ1 | -0.17488349 | 0.078949 | 0.283755492 | NS | 1.102653367 |
| PEAK3 | 0.23633875 | 0.176563678 | 0.406141882 | NS | 0.753098633 |
| LINGO3 | 0.327928823 | 0.017838901 | 0.1561459 | Up | 1.748631895 |
| LSM7 | -0.133609155 | 0.223647286 | 0.452317462 | NS | 0.650436368 |
| SPPL2B | 0.058814763 | 0.611050633 | 0.76197946 | NS | 0.213922801 |
| TMPRSS9 | 0.223807414 | 0.399949773 | 0.604986227 | NS | 0.397994546 |
| TIMM13 | -0.125311821 | 0.151325575 | 0.377750911 | NS | 0.820087668 |
| LMNB2 | -0.029212292 | 0.707820564 | 0.82706671 | NS | 0.150076824 |
| GADD45B | 0.024789857 | 0.772039682 | 0.867153153 | NS | 0.112360377 |
| GNG7 | 0.056357286 | 0.531308082 | 0.704804549 | NS | 0.274653577 |
| DIRAS1 | 0.515038696 | 0.089639277 | 0.299979972 | NS | 1.047501656 |
| SLC39A3 | 0.171060778 | 0.049280059 | 0.235025767 | Up | 1.307328783 |
| SGTA | 0.086649992 | 0.577698641 | 0.737977147 | NS | 0.238298654 |
| THOP1 | -0.035641604 | 0.700868334 | 0.822549366 | NS | 0.154363561 |
| ZNF554 | 0.03471522 | 0.629937725 | 0.774734714 | NS | 0.200702382 |
| ZNF555 | -0.106497786 | 0.107894229 | 0.325473344 | NS | 0.967001785 |
| ZNF556 | 0.615619877 | 0.065420962 | NA | NS | 1.184283072 |
| ZNF57 | 0.232936924 | 0.150986979 | 0.37754163 | NS | 0.821060505 |
| ZNF77 | -0.088032054 | 0.248848332 | 0.476376043 | NS | 0.604065266 |
| TLE6 | 0.326639759 | 0.346796182 | NA | NS | 0.459925693 |
| TLE2 | 0.423253532 | 0.058954685 | 0.25340841 | NS | 1.229481677 |
| TLE5 | 0.131539251 | 0.120169226 | 0.340734879 | NS | 0.920206736 |
| GNA11 | 0.334827695 | 0.045424538 | 0.227515368 | Up | 1.342709478 |
| GNA15 | -0.025950219 | 0.787671532 | 0.877523638 | NS | 0.10365485 |
| S1PR4 | 0.188361844 | 0.022363607 | 0.170969976 | Up | 1.650458152 |
| NCLN | 0.113353917 | 0.313685009 | 0.533319352 | NS | 0.503506236 |
| CELF5 | 0.718352156 | 0.056272928 | 0.249221499 | NS | 1.249700488 |
| NFIC | 0.064150287 | 0.740709813 | 0.847890045 | NS | 0.130351902 |
| SMIM24 | 0.172749643 | 0.462524113 | 0.653226889 | NS | 0.334865621 |
| DOHH | 0.020750977 | 0.853816142 | 0.917558879 | NS | 0.068635639 |
| FZR1 | -0.172465094 | 0.189592092 | 0.419034751 | NS | 0.722179782 |
| MFSD12 | -0.021076511 | 0.837173444 | 0.908571347 | NS | 0.077184556 |
| C19orf71 | 0.103751113 | 0.447744472 | 0.641535901 | NS | 0.348969768 |
| HMG20B | -0.0808912 | 0.258916831 | 0.485499932 | NS | 0.586839717 |
| GIPC3 | 0.297300092 | 0.279051472 | 0.503208948 | NS | 0.554315682 |
| TBXA2R | 0.430992318 | 0.044847295 | 0.226531265 | Up | 1.348263745 |
| CACTIN | 0.02137225 | 0.798366376 | 0.883977313 | NS | 0.097797762 |
| PIP5K1C | -0.048090903 | 0.753236222 | 0.855345892 | NS | 0.123068803 |
| TJP3 | 0.105541416 | 0.341964521 | 0.557326624 | NS | 0.46601895 |
| APBA3 | 0.062945818 | 0.299341068 | 0.520658398 | NS | 0.523833697 |
| MRPL54 | -0.314917511 | 0.013539071 | 0.139789052 | Down | 1.868411126 |
| RAX2 | 0.626187305 | 0.047446709 | NA | Up | 1.323793906 |
| MATK | -0.147907735 | 0.125310721 | 0.346840099 | NS | 0.90201177 |
| ZFR2 | 0.530957635 | 0.118309519 | 0.338732916 | NS | 0.926980312 |
| ATCAY | 0.75213978 | 0.125321306 | 0.346840099 | NS | 0.901975089 |
| NMRK2 | 0.87920291 | 0.066716905 | NA | NS | 1.175764108 |
| DAPK3 | -0.110389117 | 0.121383974 | 0.342066258 | NS | 0.91583865 |
| EEF2 | -0.214049377 | 0.005841725 | 0.102935441 | Down | 2.233458857 |
| PIAS4 | -0.100592965 | 0.289662371 | 0.512751809 | NS | 0.538107919 |
| ZBTB7A | 0.10638931 | 0.546031494 | 0.714624452 | NS | 0.262782307 |
| MAP2K2 | -0.006166976 | 0.955204176 | 0.975854235 | NS | 0.019903787 |
| CREB3L3 | 0.306576573 | 0.471115833 | NA | NS | 0.3268723 |
| SIRT6 | 0.004023616 | 0.956367246 | 0.976165401 | NS | 0.019375306 |
| ANKRD24 | 0.304987264 | 0.271545056 | 0.496798599 | NS | 0.5661581 |
| EBI3 | 1.289534281 | 2.65E-04 | NA | Up | 3.576391747 |
| YJU2 | -0.068964995 | 0.344832048 | 0.559698339 | NS | 0.462392379 |
| SHD | 0.61838443 | 0.088234505 | NA | NS | 1.054361544 |
| TMIGD2 | -0.423208923 | 0.004375011 | 0.093367359 | Down | 2.359020893 |
| FSD1 | 0.159813679 | 0.536001294 | 0.708067984 | NS | 0.270834162 |
| STAP2 | 0.301733085 | 0.224524698 | 0.452878298 | NS | 0.648735878 |
| MPND | -0.248890011 | 0.002682093 | 0.078552822 | Down | 2.57152616 |
| SH3GL1 | -0.042359658 | 0.739045775 | 0.846651166 | NS | 0.131328662 |
| CHAF1A | -0.14738551 | 0.153862478 | 0.380576571 | NS | 0.812867278 |
| UBXN6 | 0.134153946 | 0.41617354 | 0.617266137 | NS | 0.380725535 |
| HDGFL2 | -0.22776717 | 0.013816452 | 0.141260881 | Down | 1.859603481 |
| PLIN4 | -0.047764606 | 0.786592802 | 0.876842327 | NS | 0.104250032 |
| PLIN5 | 0.081600369 | 0.639133402 | 0.78082968 | NS | 0.194408485 |
| LRG1 | 0.125360445 | 0.416957893 | 0.617989235 | NS | 0.379907801 |
| SEMA6B | 0.528961252 | 0.038433989 | 0.213464789 | Up | 1.415284544 |
| TNFAIP8L1 | -0.190114693 | 0.002468788 | 0.076302532 | Down | 2.60751625 |
| MYDGF | -0.233009891 | 0.032197759 | 0.198223945 | Down | 1.492174355 |
| DPP9 | -0.009060522 | 0.915655853 | 0.953241072 | NS | 0.038267724 |
| FEM1A | 0.047367503 | 0.693014171 | 0.817696744 | NS | 0.159257885 |
| TICAM1 | 0.309777163 | 0.140791901 | 0.365248766 | NS | 0.851422328 |
| PLIN3 | 0.04033418 | 0.699953578 | 0.822195048 | NS | 0.154930762 |
| ARRDC5 | -0.02024529 | 0.852285328 | 0.916677841 | NS | 0.069414988 |
| UHRF1 | 0.147138035 | 0.100624012 | 0.316073396 | NS | 0.997298369 |
| KDM4B | -0.063563665 | 0.613172148 | 0.763365325 | NS | 0.212417581 |
| PTPRS | 0.31811617 | 0.075853865 | 0.279203667 | NS | 1.120022286 |
| ZNRF4 | 0.942799765 | 0.081941381 | NA | NS | 1.086496718 |
| TINCR | 0.451942054 | 0.130201486 | 0.35213444 | NS | 0.885384059 |
| SAFB2 | -0.011324368 | 0.856164297 | 0.919047327 | NS | 0.067442887 |
| SAFB | -0.07556241 | 0.253533139 | 0.480385204 | NS | 0.595965266 |
| RPL36 | -0.281664731 | 0.02292684 | 0.172380335 | Down | 1.639655791 |
| MICOS13 | -0.244610515 | 0.029582962 | 0.191700154 | Down | 1.528958346 |
| HSD11B1L | 0.384867708 | 0.119845112 | 0.340405897 | NS | 0.921379675 |
| LONP1 | -0.228713653 | 0.002953893 | 0.081359678 | Down | 2.529605178 |
| CATSPERD | 0.331396363 | 0.420052565 | NA | NS | 0.376696359 |
| PRR22 | 0.444368568 | 0.070064794 | NA | NS | 1.154500153 |
| DUS3L | -0.156195544 | 0.061445787 | 0.257734691 | NS | 1.21150789 |
| NRTN | 0.986104469 | 0.02297608 | NA | Up | 1.638724068 |
| FUT6 | 0.388233806 | 0.369045025 | NA | NS | 0.432920645 |
| FUT3 | 0.692397368 | 0.139712475 | NA | NS | 0.854764814 |
| NDUFA11 | -0.044502622 | 0.555675772 | 0.721924582 | NS | 0.255178539 |
| VMAC | 0.051469076 | 0.37565342 | 0.586154335 | NS | 0.425212653 |
| CAPS | 0.070562299 | 0.375669083 | 0.586154335 | NS | 0.425194545 |
| RANBP3 | 0.063213502 | 0.288937219 | 0.512011775 | NS | 0.539196512 |
| RFX2 | 0.086010537 | 0.588391928 | 0.746745381 | NS | 0.230333294 |
| ACSBG2 | 0.108274109 | 0.572799619 | 0.734422622 | NS | 0.24199728 |
| MLLT1 | 0.108834781 | 0.456576345 | 0.648144266 | NS | 0.340486593 |
| ACER1 | 0.544934457 | 0.051887031 | NA | NS | 1.284941181 |
| CLPP | -0.093243207 | 0.234049607 | 0.46228643 | NS | 0.630692083 |
| ALKBH7 | -0.102751585 | 0.333748946 | 0.550289298 | NS | 0.476580098 |
| PSPN | 0.033754798 | 0.707601221 | 0.826979137 | NS | 0.150211426 |
| GTF2F1 | -0.195488975 | 0.005483465 | 0.100721984 | Down | 2.260944951 |
| KHSRP | -0.08634692 | 0.401993807 | 0.606366473 | NS | 0.395780638 |
| SLC25A41 | 0.617426969 | 0.169087276 | NA | NS | 0.771889071 |
| SLC25A23 | 0.332003355 | 0.037058657 | 0.209518227 | Up | 1.431110328 |
| CRB3 | 0.212894445 | 0.297321296 | 0.51918646 | NS | 0.526773983 |
| DENND1C | -0.019537741 | 0.884295634 | 0.934742851 | NS | 0.053402519 |
| TUBB4A | 0.510220453 | 0.132479315 | 0.355399001 | NS | 0.877851927 |
| TNFSF9 | 0.209972236 | 0.426527672 | NA | NS | 0.370052788 |
| CD70 | 0.101055318 | 0.455306946 | 0.64744052 | NS | 0.341695724 |
| TNFSF14 | 0.068444607 | 0.531658788 | 0.704918055 | NS | 0.274367003 |
| C3 | 0.301183954 | 0.186469933 | 0.415945539 | NS | 0.729391186 |
| GPR108 | -0.137725496 | 0.056059675 | 0.249087638 | NS | 1.251349427 |
| TRIP10 | -0.033352118 | 0.756799988 | 0.857946407 | NS | 0.121018884 |
| SH2D3A | 0.122144098 | 0.322730724 | 0.541387141 | NS | 0.491159687 |
| VAV1 | -0.203044987 | 0.0061161 | 0.104530544 | Down | 2.213525401 |
| ADGRE1 | 0.245155665 | 0.044936914 | 0.226683964 | Up | 1.347396759 |
| MBD3L2B | 0.632065206 | 0.260627076 | NA | NS | 0.583980468 |
| MBD3L2 | 0.657177348 | 0.395425118 | NA | NS | 0.402935747 |
| MBD3L3 | 0.088560688 | 0.719168025 | NA | NS | 0.14316963 |
| ZNF557 | -0.150553713 | 0.01389761 | 0.141396297 | Down | 1.85705988 |
| INSR | -0.034202052 | 0.741186476 | 0.84830848 | NS | 0.130072513 |
| ARHGEF18 | -0.091994076 | 0.21086578 | 0.440705246 | NS | 0.675993894 |
| PEX11G | 0.201300735 | 0.177105877 | 0.406950818 | NS | 0.751767027 |
| TEX45 | 0.574799107 | 0.226868209 | NA | NS | 0.644226358 |
| ZNF358 | 0.597660128 | 0.028490179 | NA | Up | 1.545304829 |
| MCOLN1 | 0.122627401 | 0.507035449 | 0.687026733 | NS | 0.294961676 |
| PNPLA6 | 0.047569954 | 0.682729352 | 0.81107312 | NS | 0.165751426 |
| CAMSAP3 | 0.786270153 | 0.012353256 | 0.134785264 | Up | 1.908218557 |
| XAB2 | 0.044051582 | 0.577966731 | 0.738214279 | NS | 0.23809716 |
| PET100 | -0.105510962 | 0.373297083 | 0.58384551 | NS | 0.427945403 |
| PCP2 | 0.128115358 | 0.268832092 | 0.494425689 | NS | 0.570518889 |
| STXBP2 | 0.026051501 | 0.86279756 | 0.922704331 | NS | 0.064091092 |
| RETN | 0.169039683 | 0.358361538 | 0.571412376 | NS | 0.445678608 |
| MCEMP1 | 0.517341365 | 6.58E-04 | 0.04259149 | Up | 3.181875097 |
| TRAPPC5 | 0.047624991 | 0.60706209 | 0.759059619 | NS | 0.216766887 |
| FCER2 | 0.063426822 | 0.660352098 | 0.795945129 | NS | 0.180224438 |
| CD209 | 0.522487399 | 0.186716367 | NA | NS | 0.728817611 |
| CLEC4M | 0.641479927 | 0.23485363 | NA | NS | 0.629202724 |
| EVI5L | 0.122461162 | 0.301237482 | 0.522647032 | NS | 0.521090991 |
| PRR36 | 0.913424397 | 0.030116104 | 0.193361091 | Up | 1.521201214 |
| LRRC8E | 0.787394672 | 0.076270342 | NA | NS | 1.117644306 |
| MAP2K7 | -0.152635172 | 0.257144339 | 0.484152285 | NS | 0.589823032 |
| TGFBR3L | 0.520893208 | 0.095179923 | NA | NS | 1.02145465 |
| SNAPC2 | 0.247923789 | 0.013829739 | 0.141333638 | Up | 1.859186015 |
| CTXN1 | 1.389735987 | 0.06097664 | NA | NS | 1.214836507 |
| TIMM44 | -0.121227801 | 0.057033334 | 0.250513504 | NS | 1.243871242 |
| ELAVL1 | 0.006996942 | 0.891152167 | 0.938568265 | NS | 0.050048133 |
| CCL25 | 0.729070814 | 0.085415636 | NA | NS | 1.068462621 |
| FBN3 | 0.573641723 | 0.154969769 | 0.382283245 | NS | 0.809753015 |
| CERS4 | -0.028177934 | 0.713148441 | 0.830448491 | NS | 0.146820063 |
| CD320 | -0.013820621 | 0.878525304 | 0.931684267 | NS | 0.056245725 |
| NDUFA7 | 0.121289563 | 0.40271847 | 0.606899941 | NS | 0.394998451 |
| RPS28 | -0.246034614 | 0.01812845 | 0.156789181 | Down | 1.741639338 |
| KANK3 | 0.140224501 | 0.309932296 | 0.530293015 | NS | 0.508733167 |
| ANGPTL4 | 0.692320316 | 0.012044283 | 0.13403215 | Up | 1.919219055 |
| RAB11B | -0.178105969 | 0.387518324 | 0.595550181 | NS | 0.411707757 |
| MARCHF2 | -0.102227506 | 0.397402893 | 0.602512142 | NS | 0.400768975 |
| HNRNPM | -0.139525751 | 0.133143448 | 0.356387477 | NS | 0.8756802 |
| PRAM1 | 0.066053892 | 0.708496443 | 0.82730768 | NS | 0.149662326 |
| ZNF414 | 0.452450007 | 0.023068288 | 0.172916604 | Up | 1.636984634 |
| MYO1F | -0.043259483 | 0.650951933 | 0.789620058 | NS | 0.186451079 |
| ADAMTS10 | 0.03857989 | 0.771355032 | 0.866554152 | NS | 0.112745683 |
| NFILZ | 0.327082743 | 0.194908074 | 0.424596662 | NS | 0.710170171 |
| ACTL9 | 0.987558012 | 0.018532427 | NA | Up | 1.732067696 |
| OR2Z1 | 0.355647864 | 0.606515529 | NA | NS | 0.217158075 |
| ZNF558 | -0.02220008 | 0.770196484 | 0.86597476 | NS | 0.113398468 |
| MBD3L1 | 1.256227188 | 0.031162823 | NA | Up | 1.506363214 |
| MUC16 | 0.505459964 | 0.147923483 | 0.373799361 | NS | 0.829962875 |
| OR1M1 | 0.352021194 | 0.49923264 | NA | NS | 0.301697028 |
| OR7G2 | 0.436463485 | 0.368004176 | NA | NS | 0.434147253 |
| OR7G1 | -0.07874281 | 0.892638783 | NA | NS | 0.049324248 |
| OR7G3 | 0.831606619 | 0.145892607 | NA | NS | 0.835966714 |
| ZNF317 | -0.033969524 | 0.431955715 | 0.628863377 | NS | 0.364560776 |
| OR7D2 | 0.405647076 | 0.363354838 | 0.57563916 | NS | 0.439669053 |
| OR7D4 | 0.697849603 | 0.167937609 | NA | NS | 0.774852036 |
| OR7E24 | 0.396668903 | 0.534195055 | NA | NS | 0.272300137 |
| ZNF699 | -0.044777652 | 0.403454257 | 0.607391207 | NS | 0.394205698 |
| ZNF559 | -0.049259773 | 0.51110992 | 0.689903595 | NS | 0.29148569 |
| ZNF559-ZNF177 | 0.369145863 | 0.002776967 | 0.079430699 | Up | 2.556429332 |
| ZNF177 | -0.030253665 | 0.939300563 | NA | NS | 0.027195417 |
| ZNF266 | -0.182873704 | 0.002944783 | 0.081254729 | Down | 2.530946672 |
| ZNF560 | 0.436241558 | 0.299439473 | NA | NS | 0.52369095 |
| ZNF426 | -0.111853122 | 0.058437948 | 0.252404602 | NS | 1.233305042 |
| ZNF121 | -0.118443936 | 0.059356014 | 0.25415179 | NS | 1.226535272 |
| ZNF561 | 0.00796542 | 0.881240293 | 0.932827018 | NS | 0.054905654 |
| ZNF562 | 0.034746645 | 0.498472158 | 0.680295049 | NS | 0.302359094 |
| ZNF846 | 0.035466483 | 0.564958888 | 0.728925412 | NS | 0.247983155 |
| FBXL12 | 0.055815209 | 0.323178825 | 0.541912538 | NS | 0.490557103 |
| UBL5 | -0.248039413 | 0.030051901 | 0.193148154 | Down | 1.522128044 |
| PIN1 | 0.042519175 | 0.43623253 | 0.632036275 | NS | 0.360281952 |
| OLFM2 | 0.242936834 | 0.068509679 | 0.268602708 | NS | 1.164248068 |
| COL5A3 | 0.077728205 | 0.746257277 | 0.850880422 | NS | 0.127111421 |
| RDH8 | 0.98353223 | 0.088797966 | NA | NS | 1.05159698 |
| SHFL | 0.059822512 | 0.214864265 | 0.444145438 | NS | 0.667835808 |
| ANGPTL6 | 0.577157082 | 0.008800873 | 0.119193046 | Up | 2.055474251 |
| PPAN | -0.097953705 | 0.245605187 | 0.473904262 | NS | 0.609762466 |
| P2RY11 | 0.029607597 | 0.758629482 | 0.859039816 | NS | 0.119970284 |
| EIF3G | -0.295698234 | 0.001610753 | 0.063905938 | Down | 2.79297097 |
| DNMT1 | -0.180235793 | 0.115849158 | 0.334954857 | NS | 0.936107117 |
| S1PR2 | 0.304912655 | 0.003154089 | 0.084307443 | Up | 2.501126113 |
| MRPL4 | 0.129613727 | 0.295044635 | 0.517117263 | NS | 0.530112278 |
| ICAM1 | 0.055437876 | 0.542976773 | 0.712907989 | NS | 0.265218748 |
| ICAM5 | 0.669263272 | 9.08E-04 | 0.050282353 | Up | 3.041780058 |
| ZGLP1 | -0.125511457 | 0.459488282 | 0.650865947 | NS | 0.33772556 |
| FDX2 | 0.049884033 | 0.911880031 | NA | NS | 0.040062295 |
| RAVER1 | -0.120856497 | 0.456564289 | 0.648144266 | NS | 0.34049806 |
| ICAM3 | 0.040231199 | 0.672590015 | 0.804914904 | NS | 0.172249584 |
| TYK2 | -0.057545613 | 0.441003668 | 0.635776515 | NS | 0.355557799 |
| CDC37 | -0.242231295 | 2.57E-04 | 0.026605481 | Down | 3.590789156 |
| PDE4A | 0.440636682 | 0.022637098 | 0.171563006 | Up | 1.64517925 |
| KEAP1 | -0.103265922 | 0.123278654 | 0.344057977 | NS | 0.909112118 |
| S1PR5 | -0.296322894 | 0.017700063 | 0.155670827 | Down | 1.752025198 |
| ATG4D | 0.103282992 | 0.246802005 | 0.474860078 | NS | 0.607651317 |
| KRI1 | -0.048998644 | 0.507829401 | 0.687641579 | NS | 0.294282159 |
| CDKN2D | -0.19015321 | 0.083707919 | 0.29147602 | NS | 1.077233453 |
| AP1M2 | 0.252813197 | 0.19495589 | 0.424620048 | NS | 0.710063639 |
| SLC44A2 | 0.132540684 | 0.231325663 | 0.459789912 | NS | 0.635776184 |
| ILF3 | -0.110442846 | 0.076666825 | 0.280691678 | NS | 1.115392521 |
| QTRT1 | -0.150506483 | 0.08500548 | 0.293236255 | NS | 1.070553077 |
| DNM2 | 0.004335011 | 0.946474492 | 0.971154069 | NS | 0.023891086 |
| TMED1 | -0.106112149 | 0.178346254 | 0.408326093 | NS | 0.748736008 |
| C19orf38 | -0.006225237 | 0.943024887 | 0.968789986 | NS | 0.025476846 |
| CARM1 | -0.118980816 | 0.527744508 | 0.702589043 | NS | 0.277576277 |
| YIPF2 | -0.066986436 | 0.408244875 | 0.61140312 | NS | 0.389079259 |
| TIMM29 | -0.096892948 | 0.101703456 | 0.317425743 | NS | 0.992664289 |
| SMARCA4 | -0.079096473 | 0.470991695 | 0.659998409 | NS | 0.326986751 |
| LDLR | 0.200652047 | 0.014653927 | 0.145032082 | Up | 1.834045975 |
| SPC24 | 0.136074759 | 0.41957323 | 0.619739833 | NS | 0.377192229 |
| KANK2 | -0.48726285 | 0.070274251 | 0.271077991 | NS | 1.153203774 |
| DOCK6 | 0.356664463 | 0.196446445 | 0.426204669 | NS | 0.706755826 |
| ANGPTL8 | 0.306025561 | 0.589831863 | NA | NS | 0.22927177 |
| TSPAN16 | 0.331940667 | 0.032717211 | 0.199704044 | Up | 1.485223721 |
| RAB3D | 0.252764957 | 0.028865372 | 0.189677769 | Up | 1.539622834 |
| TMEM205 | -0.247243414 | 4.80E-04 | 0.037141101 | Down | 3.318784692 |
| CCDC159 | -0.164515866 | 0.146199054 | 0.371713762 | NS | 0.835055438 |
| PLPPR2 | 0.119350089 | 0.424874038 | 0.623547915 | NS | 0.371739806 |
| SWSAP1 | 0.109018432 | 0.179713462 | 0.409908267 | NS | 0.74541939 |
| EPOR | -0.137384165 | 0.395919268 | 0.601322573 | NS | 0.402393362 |
| RGL3 | 0.348530257 | 0.298202704 | 0.519744165 | NS | 0.525488423 |
| ODAD3 | 0.621872728 | 0.046418708 | 0.229359492 | Up | 1.333306954 |
| PRKCSH | -0.150355095 | 0.084999823 | 0.293236255 | NS | 1.070581979 |
| ELAVL3 | 1.035461234 | 0.012537152 | 0.135545097 | Up | 1.901801102 |
| ZNF653 | -0.066797778 | 0.58660458 | 0.745197364 | NS | 0.231654551 |
| ECSIT | 0.09366176 | 0.502988981 | 0.684134317 | NS | 0.298441529 |
| CNN1 | 0.472154209 | 0.121367899 | 0.342066258 | NS | 0.915896165 |
| ELOF1 | -0.194683172 | 0.23138061 | 0.459789912 | NS | 0.635673038 |
| ZNF627 | -0.218714401 | 0.016239866 | 0.150699117 | Down | 1.789417546 |
| ACP5 | -0.042899579 | 0.749168586 | 0.852629278 | NS | 0.125420442 |
| ZNF823 | -0.197837257 | 0.034159607 | 0.203008627 | Down | 1.466487132 |
| ZNF441 | -0.203754 | 0.038999043 | 0.214288889 | Down | 1.408946046 |
| ZNF491 | -0.122512847 | 0.194348873 | 0.423758145 | NS | 0.711417973 |
| ZNF440 | -0.064457744 | 0.231191208 | 0.459654528 | NS | 0.636028686 |
| ZNF439 | -0.022846099 | 0.771509898 | 0.866685617 | NS | 0.112658498 |
| ZNF69 | 0.057640818 | 0.522207185 | 0.697843221 | NS | 0.282157157 |
| ZNF700 | -0.066347407 | 0.213953123 | 0.443234232 | NS | 0.669681369 |
| ZNF763 | 0.015898559 | 0.821389578 | 0.898309164 | NS | 0.085450812 |
| ZNF433 | 0.085772105 | 0.383945699 | 0.592802518 | NS | 0.415730193 |
| ZNF878 | 0.136271262 | 0.530249081 | NA | NS | 0.275520075 |
| ZNF844 | -0.104325673 | 0.138609642 | 0.363039337 | NS | 0.858206557 |
| ZNF20 | 0.11538412 | 0.334493269 | 0.551080127 | NS | 0.475612617 |
| ZNF625 | -0.176393468 | 0.485357671 | NA | NS | 0.313938102 |
| ZNF136 | -0.182016757 | 0.002435697 | 0.075642185 | Down | 2.613376814 |
| ZNF44 | -0.018926866 | 0.677628232 | 0.807610417 | NS | 0.169008508 |
| ZNF563 | -0.086367541 | 0.426529165 | 0.624735925 | NS | 0.370051268 |
| ZNF442 | 0.010533706 | 0.934466487 | 0.963842168 | NS | 0.029436269 |
| ZNF799 | -0.119734104 | 0.081583989 | 0.287942716 | NS | 1.088395066 |
| ZNF443 | 0.041253515 | 0.638313183 | 0.780077296 | NS | 0.194966186 |
| ZNF709 | 0.119313003 | 0.531293605 | 0.704804549 | NS | 0.274665411 |
| ZNF564 | 0.103809635 | 0.231746011 | 0.459837736 | NS | 0.634987732 |
| ZNF490 | 0.094630205 | 0.074972588 | 0.277970247 | NS | 1.125097496 |
| ZNF791 | -0.10910623 | 0.052621941 | 0.242513843 | NS | 1.278833138 |
| MAN2B1 | -0.193437325 | 0.024027133 | 0.175592792 | Down | 1.619298049 |
| WDR83 | 0.098524364 | 0.295080796 | 0.517117263 | NS | 0.530059054 |
| DHPS | -0.017904665 | 0.851484309 | 0.916355684 | NS | 0.06982335 |
| FBXW9 | 0.103596136 | 0.453316418 | 0.645878599 | NS | 0.343598552 |
| TNPO2 | 0.115318527 | 0.232053711 | 0.46031947 | NS | 0.634411481 |
| TRIR | 0.033201972 | 0.666493627 | 0.800463424 | NS | 0.176203999 |
| GET3 | -0.354105519 | 1.48E-04 | 0.019564513 | Down | 3.830358269 |
| BEST2 | 0.647211183 | 0.123290807 | NA | NS | 0.909069305 |
| HOOK2 | -0.036117515 | 0.750182745 | 0.853282895 | NS | 0.124832929 |
| JUNB | -0.011187573 | 0.922888141 | 0.957636003 | NS | 0.034850934 |
| PRDX2 | 0.002038573 | 0.989721018 | 0.994035648 | NS | 0.004487207 |
| THSD8 | 1.028324293 | 0.050434452 | NA | NS | 1.297272694 |
| RNASEH2A | 0.31211889 | 0.305357338 | NA | NS | 0.51519164 |
| RTBDN | 0.48360094 | 0.329463416 | NA | NS | 0.482192804 |
| MAST1 | 0.571447593 | 0.052276518 | 0.241796393 | NS | 1.281693349 |
| DNASE2 | -0.179579859 | 0.025089232 | 0.179152549 | Down | 1.600512639 |
| KLF1 | -0.02184119 | 0.907378515 | 0.948677597 | NS | 0.042211508 |
| GCDH | -0.267152232 | 3.88E-05 | 0.008377992 | Down | 4.411427617 |
| SYCE2 | 0.195978304 | 0.295155014 | 0.517147895 | NS | 0.529949834 |
| FARSA | -0.181945843 | 0.037880431 | 0.211801177 | Down | 1.421585093 |
| CALR | 0.048046913 | 0.523966069 | 0.699578553 | NS | 0.280696836 |
| RAD23A | -0.117168418 | 0.564174122 | 0.728337377 | NS | 0.248586839 |
| GADD45GIP1 | -0.097006298 | 0.365737623 | 0.578075841 | NS | 0.436830362 |
| DAND5 | 0.948139734 | 0.118322941 | NA | NS | 0.926931045 |
| NFIX | 0.42339632 | 0.027749061 | 0.186639939 | Up | 1.556751711 |
| LYL1 | -0.077226402 | 0.644017405 | 0.78470348 | NS | 0.191102395 |
| TRMT1 | -0.15854 | 0.028757642 | 0.189255035 | Down | 1.541246723 |
| NACC1 | 0.21392783 | 0.043849789 | 0.224412931 | Up | 1.358032492 |
| STX10 | -0.129282468 | 0.063740552 | 0.262209014 | NS | 1.195584178 |
| IER2 | -0.23612775 | 0.004955427 | 0.097499295 | Down | 2.304918955 |
| CACNA1A | 0.394196872 | 0.141576886 | 0.365923729 | NS | 0.849007644 |
| YJU2B | 0.02603067 | 0.767704073 | 0.864740339 | NS | 0.114806155 |
| MRI1 | -0.052770886 | 0.502665578 | 0.683938158 | NS | 0.298720854 |
| C19orf53 | -0.197975493 | 0.05472042 | 0.246888703 | NS | 1.261850579 |
| ZSWIM4 | -0.014733378 | 0.94241221 | NA | NS | 0.025759096 |
| CC2D1A | -0.101139113 | 0.618575371 | 0.766716087 | NS | 0.208607376 |
| DCAF15 | 0.057202318 | 0.556114819 | 0.722126301 | NS | 0.254835532 |
| RFX1 | -0.165031029 | 0.520427149 | 0.696688569 | NS | 0.283640056 |
| IL27RA | -0.226143913 | 0.013837432 | 0.141349184 | Down | 1.858944495 |
| MISP3 | 0.249173816 | 0.436392692 | NA | NS | 0.360122531 |
| SAMD1 | 0.012655244 | 0.935027451 | 0.96397808 | NS | 0.029175639 |
| PRKACA | -0.077283731 | 0.534065569 | 0.706592665 | NS | 0.27240542 |
| ASF1B | 0.230439691 | 0.145475978 | 0.37059965 | NS | 0.837208714 |
| ADGRL1 | 0.130282822 | 0.493665619 | 0.67740743 | NS | 0.306567118 |
| ADGRE5 | -0.037316658 | 0.73337301 | 0.842396864 | NS | 0.134675077 |
| DDX39A | -0.12367987 | 0.13850624 | 0.363014451 | NS | 0.85853066 |
| PKN1 | 0.146180209 | 0.170443225 | 0.398762972 | NS | 0.768420258 |
| PTGER1 | 1.140104888 | 0.033626276 | NA | Up | 1.473321231 |
| GIPC1 | -0.039081669 | 0.6227631 | 0.769743797 | NS | 0.205677128 |
| DNAJB1 | -0.171492349 | 0.019605416 | 0.162564529 | Down | 1.707623947 |
| TECR | 0.325258994 | 0.001234649 | 0.057117621 | Up | 2.908456386 |
| NDUFB7 | -0.028488765 | 0.787782992 | 0.877605121 | NS | 0.1035934 |
| CLEC17A | -0.137078087 | 0.085605414 | 0.294065576 | NS | 1.067498767 |
| ADGRE3 | 0.131752656 | 0.304264195 | 0.52547007 | NS | 0.516749151 |
| ZNF333 | 0.052131887 | 0.329185992 | 0.546701782 | NS | 0.482558654 |
| ADGRE2 | 0.066450908 | 0.602710595 | 0.756146026 | NS | 0.219891174 |
| OR7C1 | 0.272002834 | 0.209693255 | 0.439792418 | NS | 0.678415538 |
| OR7A5 | 0.449833574 | 0.41404073 | NA | NS | 0.382956934 |
| OR7A10 | 0.558273471 | 0.397904109 | NA | NS | 0.400221576 |
| OR7A17 | 0.540947627 | 0.282561604 | NA | NS | 0.548886852 |
| OR7C2 | 0.343830632 | 0.557947505 | NA | NS | 0.25340666 |
| SLC1A6 | 0.361175531 | 0.451789366 | 0.6445048 | NS | 0.345063995 |
| CCDC105 | 1.038997069 | 0.077134058 | NA | NS | 1.112753821 |
| CASP14 | 0.401770467 | 0.387045915 | 0.595067505 | NS | 0.412237512 |
| OR1I1 | 0.378849855 | 0.469120578 | NA | NS | 0.328715517 |
| SYDE1 | 0.821547459 | 0.015215809 | 0.147378943 | Up | 1.817704963 |
| ILVBL | -0.20062462 | 0.007240766 | 0.11179811 | Down | 2.140215511 |
| NOTCH3 | 0.833130459 | 0.024519004 | 0.177561993 | Up | 1.610497175 |
| EPHX3 | 1.053892331 | 0.015028548 | NA | Up | 1.823082964 |
| BRD4 | 0.131734193 | 0.41149166 | 0.614058952 | NS | 0.385638962 |
| AKAP8 | -0.061832639 | 0.156685652 | 0.383828732 | NS | 0.80497077 |
| AKAP8L | -0.22665537 | 0.001053723 | 0.052878189 | Down | 2.977273629 |
| WIZ | 0.067403542 | 0.699361039 | 0.822044571 | NS | 0.155298566 |
| RASAL3 | -0.099620307 | 0.342630409 | 0.557881175 | NS | 0.465174095 |
| PGLYRP2 | 0.45251217 | 0.085050868 | NA | NS | 1.07032125 |
| CYP4F22 | 0.624259524 | 0.005186515 | 0.098827133 | Up | 2.285124322 |
| CYP4F8 | 0.558001627 | 0.221861597 | 0.450609639 | NS | 0.653917865 |
| CYP4F3 | 0.029906963 | 0.851496926 | 0.916355684 | NS | 0.069816915 |
| CYP4F12 | 0.257937512 | 0.101657224 | 0.317403374 | NS | 0.992861753 |
| OR10H2 | 1.066295301 | 0.137947171 | NA | NS | 0.860287201 |
| OR10H3 | 1.57441227 | 0.017130524 | NA | Up | 1.766229361 |
| OR10H5 | 0.542112688 | 0.266061797 | 0.491994128 | NS | 0.57501748 |
| OR10H1 | 0.621876854 | 0.199207806 | 0.428926322 | NS | 0.700693647 |
| CYP4F2 | 0.789475199 | 0.059704653 | NA | NS | 1.22399182 |
| CYP4F11 | 0.773789779 | 0.059400843 | 0.254232499 | NS | 1.226207393 |
| OR10H4 | 0.301160796 | 0.580093761 | NA | NS | 0.236501806 |
| TPM4 | -0.045994484 | 0.492795328 | 0.676692631 | NS | 0.307333418 |
| RAB8A | 0.135472903 | 0.008655094 | 0.118708759 | Up | 2.062728202 |
| HSH2D | 0.167279625 | 0.09938011 | 0.314387008 | NS | 1.002700529 |
| CIB3 | 0.350288603 | 0.152466857 | 0.37908978 | NS | 0.816824552 |
| FAM32A | -0.169562714 | 0.060465064 | 0.256344114 | NS | 1.218495481 |
| AP1M1 | -0.006162561 | 0.90754905 | 0.948812579 | NS | 0.042129893 |
| KLF2 | 0.125234193 | 0.171691954 | 0.400253372 | NS | 0.765250057 |
| EPS15L1 | 0.102767081 | 0.339239948 | 0.554994713 | NS | 0.469493013 |
| CALR3 | -0.215268631 | 0.628061317 | NA | NS | 0.201997954 |
| C19orf44 | 0.350117282 | 0.002642882 | 0.078363492 | Up | 2.577922301 |
| CHERP | 0.103433051 | 0.485455708 | 0.671360296 | NS | 0.313850388 |
| SLC35E1 | -0.024724391 | 0.727435323 | 0.838753198 | NS | 0.138205614 |
| MED26 | 0.1061385 | 0.078721016 | 0.283584921 | NS | 1.10390931 |
| SMIM7 | -0.096325265 | 0.061771202 | 0.258295429 | NS | 1.20921395 |
| TMEM38A | -0.032597763 | 0.760014182 | 0.86001506 | NS | 0.119178303 |
| NWD1 | 0.389612476 | 0.255150206 | 0.481973773 | NS | 0.593204076 |
| SIN3B | 0.130497654 | 0.354261654 | 0.567443028 | NS | 0.450675854 |
| F2RL3 | 0.246238195 | 0.144239922 | 0.369629932 | NS | 0.84091452 |
| CPAMD8 | 0.360705904 | 0.010134919 | 0.126203819 | Up | 1.994179733 |
| HAUS8 | -0.036194002 | 0.598753319 | 0.753483627 | NS | 0.222752066 |
| MYO9B | -0.112989271 | 0.329040238 | 0.546618275 | NS | 0.482750989 |
| USE1 | 0.226341119 | 0.017196985 | 0.154054413 | Up | 1.764547692 |
| OCEL1 | 0.343248911 | 0.010133903 | 0.126203819 | Up | 1.994223259 |
| NR2F6 | 0.32582981 | 0.034162748 | 0.203008627 | Up | 1.466447206 |
| USHBP1 | 0.368725874 | 0.209723519 | 0.439792418 | NS | 0.678352863 |
| BABAM1 | -0.282459333 | 0.005708263 | 0.102180893 | Down | 2.243496052 |
| ANKLE1 | 0.141622688 | 0.351307916 | 0.565122842 | NS | 0.454312064 |
| ABHD8 | 0.182599408 | 0.045522899 | 0.227848562 | Up | 1.34177009 |
| MRPL34 | -0.002030692 | 0.984693443 | 0.990727732 | NS | 0.006698954 |
| DDA1 | 0.158072807 | 0.051765533 | 0.240198165 | NS | 1.285959307 |
| ANO8 | 0.058601189 | 0.657262206 | 0.794202854 | NS | 0.18226134 |
| GTPBP3 | 0.119054916 | 0.331298625 | 0.548259227 | NS | 0.479780367 |
| PLVAP | -0.332249409 | 0.201030135 | 0.430601586 | NS | 0.696738835 |
| CCDC194 | 0.919385812 | 0.002387349 | NA | Up | 2.622084009 |
| BST2 | -0.149746692 | 0.164075539 | 0.391953477 | NS | 0.784956162 |
| MVB12A | 0.303869135 | 0.007915746 | 0.11472857 | Up | 2.101508144 |
| TMEM221 | 0.696753298 | 0.074182015 | NA | NS | 1.129701377 |
| NXNL1 | 0.377046644 | 0.725721064 | NA | NS | 0.139230271 |
| SLC27A1 | 0.248711219 | 0.090299973 | 0.300982388 | NS | 1.044312378 |
| PGLS | 0.194811238 | 0.005106775 | 0.09835814 | Up | 2.291853249 |
| NIBAN3 | -0.101608919 | 0.383496798 | 0.592439708 | NS | 0.416238258 |
| COLGALT1 | -0.08720685 | 0.195090344 | 0.424791696 | NS | 0.709764225 |
| UNC13A | 0.438450618 | 0.24189794 | 0.46988521 | NS | 0.61636783 |
| MAP1S | 0.112692355 | 0.39267885 | 0.599318562 | NS | 0.405962489 |
| FCHO1 | -0.044032981 | 0.656325683 | 0.793578522 | NS | 0.182880601 |
| B3GNT3 | 0.559033277 | 0.071961913 | NA | NS | 1.142897299 |
| INSL3 | 0.341127273 | 0.071705659 | 0.272972574 | NS | 1.144446568 |
| JAK3 | 0.10012315 | 0.325974005 | 0.544407253 | NS | 0.486817032 |
| RPL18A | -0.162433195 | 0.149625173 | 0.375764013 | NS | 0.824995335 |
| SLC5A5 | 0.309953112 | 0.160645414 | 0.388500662 | NS | 0.794131668 |
| CCDC124 | -0.159111222 | 0.401423363 | 0.605905085 | NS | 0.396397355 |
| KCNN1 | 0.935005953 | 0.013929362 | 0.141407346 | Up | 1.856068784 |
| ARRDC2 | -0.098257184 | 0.090440732 | 0.301169683 | NS | 1.043635929 |
| IL12RB1 | -0.037214684 | 0.531288093 | 0.704804549 | NS | 0.274669917 |
| MAST3 | -0.157443338 | 0.25970303 | 0.485843002 | NS | 0.585522983 |
| PIK3R2 | 0.287436994 | 0.348774511 | NA | NS | 0.457455261 |
| IFI30 | -0.062040006 | 0.546082668 | 0.714650587 | NS | 0.262741607 |
| MPV17L2 | -0.145332873 | 0.175945127 | 0.405354744 | NS | 0.754622758 |
| RAB3A | 0.370784974 | 0.082071846 | 0.288607877 | NS | 1.0858058 |
| PDE4C | 0.592594453 | 0.292105805 | NA | NS | 0.534459813 |
| IQCN | 0.040696666 | 0.837321692 | 0.908571347 | NS | 0.077107658 |
| JUND | 0.049975777 | 0.788198463 | 0.87789715 | NS | 0.103364416 |
| LSM4 | -0.204582382 | 0.005055047 | 0.098110742 | Down | 2.296274842 |
| PGPEP1 | -0.047807306 | 0.306810132 | 0.527638781 | NS | 0.513130302 |
| GDF15 | 0.147594637 | 0.456449094 | 0.648084139 | NS | 0.34060765 |
| LRRC25 | 0.165033844 | 0.087017282 | 0.29656937 | NS | 1.060394487 |
| SSBP4 | 0.034557214 | 0.748098931 | 0.851877572 | NS | 0.126040966 |
| ISYNA1 | 0.068499665 | 0.592336433 | 0.749026006 | NS | 0.227431554 |
| ELL | 0.023711611 | 0.813743117 | 0.893529505 | NS | 0.089512672 |
| FKBP8 | -0.105843101 | 0.594734859 | 0.750384214 | NS | 0.225676605 |
| KXD1 | -0.022253102 | 0.766808386 | 0.864244373 | NS | 0.115313146 |
| UBA52 | -0.244457761 | 0.049786591 | 0.236268112 | Down | 1.302887606 |
| CRLF1 | 0.119040933 | 0.424935981 | 0.623595614 | NS | 0.371676494 |
| REX1BD | 0.047599055 | 0.60664366 | 0.758788758 | NS | 0.217066336 |
| TMEM59L | 0.545186112 | 0.330356958 | NA | NS | 0.481016541 |
| KLHL26 | 0.342633912 | 0.040427022 | 0.216879751 | Up | 1.393328249 |
| CRTC1 | 0.130012149 | 0.34117879 | 0.556807287 | NS | 0.467017975 |
| COMP | 0.964134613 | 0.018687716 | 0.158925385 | Up | 1.728443784 |
| UPF1 | -0.047423279 | 0.593146511 | 0.74951674 | NS | 0.22683802 |
| CERS1 | 0.702748282 | 0.099038377 | NA | NS | 1.004196487 |
| COPE | 0.082389984 | 0.183534795 | 0.413733592 | NS | 0.736281588 |
| DDX49 | -0.173421259 | 0.009041463 | 0.120388133 | Down | 2.043761284 |
| HOMER3 | -0.211195569 | 0.081772471 | 0.288293278 | NS | 1.087392878 |
| SUGP2 | -0.070623895 | 0.258273727 | 0.484975865 | NS | 0.587919771 |
| ARMC6 | -0.183901626 | 0.0045034 | 0.094105548 | Down | 2.346459483 |
| SLC25A42 | -0.136696931 | 0.133001125 | 0.356364174 | NS | 0.876144686 |
| TMEM161A | 0.077530606 | 0.424508393 | 0.623250928 | NS | 0.372113718 |
| MEF2B | 0.181139283 | 0.590725745 | NA | NS | 0.228614101 |
| BORCS8 | 0.144351398 | 0.13502124 | 0.358688835 | NS | 0.869597907 |
| RFXANK | -0.148969306 | 0.036619617 | 0.20873154 | Down | 1.436286201 |
| NR2C2AP | -0.254043791 | 0.002137951 | 0.072389226 | Down | 2.670002302 |
| NCAN | 0.358235439 | 0.442537589 | 0.636980445 | NS | 0.354049835 |
| TM6SF2 | 0.361260773 | 0.567263936 | NA | NS | 0.246214826 |
| SUGP1 | 0.108574282 | 0.050066106 | 0.236805858 | NS | 1.300456184 |
| MAU2 | 0.159475974 | 0.077133929 | 0.281472473 | NS | 1.112754547 |
| GATAD2A | -0.262396207 | 0.077369839 | 0.281652078 | NS | 1.111428309 |
| TSSK6 | 0.190626241 | 0.112173733 | 0.330787614 | NS | 0.950108826 |
| NDUFA13 | -0.099298346 | 0.270740749 | 0.496040372 | NS | 0.567446373 |
| YJEFN3 | 0.118518508 | 0.687183663 | 0.814178911 | NS | 0.162927174 |
| CILP2 | 0.994693989 | 0.012538965 | 0.135545097 | Up | 1.901738298 |
| PBX4 | 0.148461534 | 0.191442265 | 0.420728886 | NS | 0.717962176 |
| LPAR2 | 0.141558213 | 0.259338164 | 0.485568555 | NS | 0.586133568 |
| GMIP | 0.005663566 | 0.967793451 | 0.982114953 | NS | 0.014217321 |
| ATP13A1 | 0.085305589 | 0.504939702 | 0.685516059 | NS | 0.29676048 |
| ZNF101 | -0.084717041 | 0.174684234 | 0.404024697 | NS | 0.757746291 |
| ZNF14 | -0.18454466 | 0.056606674 | 0.249939307 | NS | 1.247132361 |
| ZNF506 | 0.035074964 | 0.490323065 | 0.674680296 | NS | 0.309517676 |
| ZNF253 | -0.136001179 | 0.09450512 | 0.307524334 | NS | 1.024544661 |
| ZNF93 | -0.044008034 | 0.533169126 | 0.705980534 | NS | 0.273135007 |
| ZNF682 | 0.001862655 | 0.981453669 | 0.989191488 | NS | 0.008130197 |
| ZNF90 | -0.137680455 | 0.210795271 | 0.440610543 | NS | 0.676139136 |
| ZNF486 | -0.195448689 | 0.044203853 | 0.224918162 | Down | 1.354539872 |
| ZNF737 | -0.116108332 | 0.102794513 | 0.318649084 | NS | 0.988030065 |
| ZNF626 | -0.183102239 | 0.044416403 | 0.225484138 | Down | 1.352456612 |
| ZNF66 | 0.031611596 | 0.781809383 | 0.873857226 | NS | 0.106899122 |
| ZNF85 | -0.162258816 | 0.304957241 | 0.526190818 | NS | 0.51576105 |
| ZNF430 | -0.202469574 | 0.008506605 | 0.118000168 | Down | 2.070243711 |
| ZNF714 | 0.036664799 | 0.600809295 | 0.754785215 | NS | 0.221263357 |
| ZNF431 | -0.068572294 | 0.177979282 | 0.407982383 | NS | 0.749630549 |
| ZNF708 | -0.034815531 | 0.664431181 | 0.799096584 | NS | 0.177549994 |
| ZNF738 | -0.056752258 | 0.434055989 | 0.63047823 | NS | 0.362454247 |
| ZNF493 | 0.023113731 | 0.707910571 | 0.82706671 | NS | 0.150021602 |
| ZNF429 | 0.210583589 | 0.046532368 | 0.229687036 | Up | 1.332244849 |
| ZNF100 | 0.095854112 | 0.460437199 | 0.651723712 | NS | 0.336829597 |
| ZNF43 | 0.074551695 | 0.165642998 | 0.393591001 | NS | 0.780826917 |
| ZNF208 | -0.038227627 | 0.79000536 | 0.878627756 | NS | 0.102369962 |
| ZNF257 | -0.080422231 | 0.493355528 | 0.677207384 | NS | 0.306840001 |
| ZNF676 | 0.424222723 | 0.589332941 | NA | NS | 0.229639283 |
| ZNF729 | 0.561801103 | 0.346780974 | NA | NS | 0.459944737 |
| ZNF98 | 0.434738372 | 0.334607658 | NA | NS | 0.475464123 |
| ZNF492 | -0.005681367 | 0.971003626 | 0.983510672 | NS | 0.012779148 |
| ZNF99 | 0.486686561 | 0.219415344 | NA | NS | 0.658733005 |
| ZNF723 | 0.278560617 | 0.529156491 | NA | NS | 0.276415872 |
| ZNF728 | 0.676521613 | 0.105410283 | NA | NS | 0.97711702 |
| ZNF730 | -0.284588201 | 0.240177847 | 0.468217208 | NS | 0.619467053 |
| ZNF724 | -0.225023754 | 0.016872728 | 0.152976731 | Down | 1.772814689 |
| ZNF91 | -0.275199474 | 0.002783154 | 0.079430699 | Down | 2.5554628 |
| ZNF675 | -0.135624161 | 0.110376689 | 0.32888979 | NS | 0.957122639 |
| ZNF681 | -0.127888518 | 0.25105348 | 0.477900988 | NS | 0.600233755 |
| RPSAP58 | -0.481632845 | 0.008191935 | 0.116591208 | Down | 2.086613492 |
| ZNF726 | -0.040340376 | 0.722364858 | 0.836151808 | NS | 0.14124339 |
| ZNF254 | -0.091166043 | 0.298434293 | 0.51995396 | NS | 0.525151273 |
| UQCRFS1 | -0.131619845 | 0.129898651 | 0.351980467 | NS | 0.886395359 |
| VSTM2B | 0.838048893 | 0.039411543 | NA | Up | 1.404376559 |
| POP4 | -0.12001602 | 0.132515568 | 0.35545462 | NS | 0.877733096 |
| PLEKHF1 | -0.061739525 | 0.516355621 | 0.693953766 | NS | 0.287051091 |
| C19orf12 | -0.058697672 | 0.265867196 | 0.491832837 | NS | 0.575335245 |
| CCNE1 | -0.128387143 | 0.330667793 | 0.547740264 | NS | 0.480608103 |
| URI1 | -0.154962228 | 0.021187533 | 0.168202484 | Down | 1.673919605 |
| ZNF536 | 0.491421681 | 0.264611947 | 0.49081905 | NS | 0.577390551 |
| TSHZ3 | 0.031887813 | 0.792439943 | 0.880394857 | NS | 0.101033642 |
| ZNF507 | 0.070365045 | 0.252104159 | 0.479033151 | NS | 0.598419989 |
| DPY19L3 | 0.188659436 | 0.016391286 | 0.151221722 | Up | 1.785386967 |
| PDCD5 | -0.092670445 | 0.238335882 | 0.466447476 | NS | 0.622810569 |
| ANKRD27 | -0.08283586 | 0.02153045 | 0.168635557 | NS | 1.66694689 |
| RGS9BP | 0.509008392 | 0.069904242 | NA | NS | 1.155496471 |
| NUDT19 | -0.132024158 | 0.06122272 | 0.257380382 | NS | 1.213087377 |
| TDRD12 | 0.292565199 | 0.233075493 | 0.461424095 | NS | 0.632503388 |
| SLC7A9 | 0.494122698 | 0.076557172 | NA | NS | 1.116014116 |
| CEP89 | 0.014932299 | 0.901365207 | 0.945230163 | NS | 0.04509921 |
| FAAP24 | -0.066656342 | 0.560186341 | 0.724761145 | NS | 0.251667485 |
| RHPN2 | 0.15180463 | 0.450796971 | 0.643924649 | NS | 0.346019011 |
| GPATCH1 | -0.121778471 | 0.16794558 | 0.396034769 | NS | 0.774831421 |
| WDR88 | 0.149810974 | 0.131327907 | 0.354010326 | NS | 0.881642977 |
| LRP3 | 0.358037762 | 0.021471024 | 0.168494559 | Up | 1.668147247 |
| SLC7A10 | 1.147433846 | 0.005756637 | NA | Up | 2.239831118 |
| CEBPA | -0.123257552 | 0.220577913 | 0.449602156 | NS | 0.656437977 |
| CEBPG | -0.129792938 | 0.087716846 | 0.297393222 | NS | 1.056916991 |
| PEPD | -0.151458139 | 0.044621598 | 0.225889772 | Down | 1.350454879 |
| CHST8 | 0.723404698 | 0.013752157 | 0.140981156 | Up | 1.861629174 |
| KCTD15 | 0.037789967 | 0.604862496 | 0.757685768 | NS | 0.218343343 |
| LSM14A | -0.389331445 | 3.75E-05 | 0.00829096 | Down | 4.426101903 |
| GARRE1 | 0.014807796 | 0.806367469 | 0.888578902 | NS | 0.093467001 |
| GPI | 0.223367315 | 0.010040134 | 0.126199476 | Up | 1.998260492 |
| PDCD2L | -0.142132255 | 0.223981373 | 0.452443504 | NS | 0.649788097 |
| UBA2 | -0.277789612 | 0.001017996 | 0.05274695 | Down | 2.99225379 |
| WTIP | 0.673875046 | 0.028170671 | 0.187820875 | Up | 1.55020281 |
| SCGB2B2 | 0.516682548 | 0.065725855 | 0.265127437 | NS | 1.182263754 |
| ZNF302 | -0.145402404 | 0.079032384 | 0.283921514 | NS | 1.10219492 |
| ZNF181 | -0.20054721 | 0.060504404 | 0.256368522 | NS | 1.218213012 |
| ZNF599 | 0.14326477 | 0.065376779 | 0.264481527 | NS | 1.184576479 |
| ZNF30 | -0.052783487 | 0.512345138 | 0.690869544 | NS | 0.290437381 |
| ZNF792 | 0.007610579 | 0.89687119 | 0.942251663 | NS | 0.047269926 |
| GRAMD1A | 0.044673548 | 0.719950925 | 0.834756433 | NS | 0.142697106 |
| SCN1B | 0.16269403 | 0.335495452 | 0.551756712 | NS | 0.474313363 |
| HPN | 0.837771206 | 0.019121077 | NA | Up | 1.718487653 |
| FXYD3 | 0.344814024 | 0.523131294 | NA | NS | 0.2813893 |
| LGI4 | 0.789578896 | 0.018095502 | 0.156740993 | Up | 1.742429365 |
| FXYD1 | 0.54167993 | 0.187175537 | NA | NS | 0.727750912 |
| FXYD7 | 0.326201996 | 0.114230097 | 0.332667558 | NS | 0.942219456 |
| FXYD5 | 0.213189911 | 0.032046448 | 0.198142899 | Up | 1.494220102 |
| FAM187B | 0.853096271 | 0.114688939 | NA | NS | 0.940478464 |
| LSR | 0.151213602 | 0.064907392 | 0.264064926 | NS | 1.18770584 |
| USF2 | 0.038705859 | 0.670740871 | 0.803625814 | NS | 0.173445229 |
| HAMP | 0.58193942 | 0.05722987 | NA | NS | 1.24237724 |
| MAG | 0.798127391 | 0.066932438 | NA | NS | 1.174363354 |
| CD22 | 0.073578215 | 0.562688012 | 0.72691877 | NS | 0.249732337 |
| FFAR1 | 0.685223244 | 0.077482255 | NA | NS | 1.110797748 |
| FFAR3 | -0.048927957 | 0.821943905 | 0.89852789 | NS | 0.085157821 |
| GPR42 | 0.388130656 | 0.267104923 | NA | NS | 0.573318108 |
| FFAR2 | 0.333497413 | 0.021770795 | 0.16924465 | Up | 1.662125719 |
| KRTDAP | 0.267975481 | 0.652028169 | NA | NS | 0.185733641 |
| DMKN | 0.396458288 | 0.151780924 | 0.378328987 | NS | 0.818782806 |
| SBSN | 0.805841923 | 0.165626646 | NA | NS | 0.780869794 |
| GAPDHS | -0.004572001 | 0.987223424 | NA | NS | 0.005584549 |
| TMEM147 | -0.110776773 | 0.233290594 | 0.461566743 | NS | 0.632102772 |
| ATP4A | 0.345489892 | 0.296189748 | 0.517930641 | NS | 0.528429978 |
| PMIS2 | 0.08159249 | 0.901897392 | NA | NS | 0.044842869 |
| HAUS5 | -0.124612842 | 0.247101448 | 0.475332521 | NS | 0.60712471 |
| RBM42 | 0.184046333 | 0.037577116 | 0.210464935 | Up | 1.425076549 |
| ETV2 | 0.408877155 | 0.068435599 | NA | NS | 1.164717928 |
| COX6B1 | -0.129755226 | 0.086119543 | 0.29485869 | NS | 1.064898283 |
| UPK1A | 1.239181418 | 0.005633322 | NA | Up | 2.249235401 |
| ZBTB32 | 0.14148246 | 0.104206575 | 0.320245132 | NS | 0.98210488 |
| KMT2B | -0.24868116 | 0.244119966 | 0.472039467 | NS | 0.6123967 |
| IGFLR1 | 0.13908373 | 0.14565553 | 0.370809577 | NS | 0.836673021 |
| U2AF1L4 | 0.310616146 | 0.009097693 | 0.120659125 | Up | 2.041068711 |
| PSENEN | 0.003930874 | 0.968752745 | 0.982567554 | NS | 0.013787054 |
| LIN37 | -0.210607028 | 0.028462604 | 0.188709674 | Down | 1.54572537 |
| HSPB6 | 0.775928309 | 0.035196127 | NA | Up | 1.453505125 |
| PROSER3 | 0.045331133 | 0.806190824 | 0.888516951 | NS | 0.093562149 |
| ARHGAP33 | 0.06900896 | 0.753569416 | 0.855639404 | NS | 0.122876736 |
| PRODH2 | 1.110246116 | 0.04574901 | NA | Up | 1.339618304 |
| NPHS1 | 0.573381981 | 0.191951319 | 0.420934764 | NS | 0.716808899 |
| KIRREL2 | 0.684110562 | 0.134357605 | 0.357917311 | NS | 0.871737747 |
| APLP1 | 0.60761853 | 0.167279046 | 0.395361076 | NS | 0.776558457 |
| NFKBID | 0.010468262 | 0.912583856 | 0.951241312 | NS | 0.039727218 |
| HCST | 0.034272218 | 0.715839073 | 0.832021237 | NS | 0.1451846 |
| TYROBP | 0.20449448 | 0.054648821 | 0.24676996 | NS | 1.262419203 |
| LRFN3 | 0.286851299 | 0.128532694 | 0.350310099 | NS | 0.890986389 |
| SDHAF1 | -0.094201306 | 0.327836628 | 0.545647854 | NS | 0.484342525 |
| SYNE4 | 0.126592588 | 0.558803168 | 0.724139091 | NS | 0.25274114 |
| ALKBH6 | -0.049302623 | 0.620593539 | 0.767922494 | NS | 0.20719275 |
| CLIP3 | 0.275742996 | 0.250042522 | 0.47708706 | NS | 0.601986129 |
| THAP8 | 0.365207242 | 0.049258642 | 0.235025767 | Up | 1.307517569 |
| WDR62 | 0.276742926 | 0.191036421 | 0.420401279 | NS | 0.718883827 |
| OVOL3 | 0.390167837 | 0.071287869 | NA | NS | 1.146984365 |
| POLR2I | -0.222193262 | 0.0391983 | 0.214561777 | Down | 1.406732766 |
| TBCB | -0.228447663 | 0.003789217 | 0.088644036 | Down | 2.421450497 |
| CAPNS1 | 0.147357057 | 0.101276964 | 0.31699399 | NS | 0.994489327 |
| COX7A1 | 0.017631849 | 0.945015026 | NA | NS | 0.024561286 |
| ZNF565 | -0.146747621 | 0.054384639 | 0.246520549 | NS | 1.264523746 |
| ZNF146 | -0.226063205 | 0.005048969 | 0.098075907 | Down | 2.296797273 |
| ZFP14 | -0.123299301 | 0.066261478 | 0.2655108 | NS | 1.178738879 |
| ZFP82 | -0.066727414 | 0.339293623 | 0.554994713 | NS | 0.469424303 |
| ZNF566 | -0.294840535 | 0.00935483 | 0.12207653 | Down | 2.028964115 |
| ZNF260 | -0.076505659 | 0.341486393 | 0.55696544 | NS | 0.466626597 |
| ZNF529 | 0.006060435 | 0.908072554 | 0.949229889 | NS | 0.04187945 |
| ZNF382 | 0.020860709 | 0.745718058 | 0.850639911 | NS | 0.12742534 |
| ZNF461 | -0.083265696 | 0.242109212 | 0.469918053 | NS | 0.615988685 |
| ZNF567 | -0.191882391 | 0.011214858 | 0.13003023 | Down | 1.950206221 |
| ZNF850 | -0.123448685 | 0.058312346 | 0.252212565 | NS | 1.234239484 |
| ZNF790 | -0.240524943 | 0.001954841 | 0.069734836 | Down | 2.7088886 |
| ZNF345 | -0.077401323 | 0.283581508 | 0.506400289 | NS | 0.547322092 |
| ZNF829 | -0.121176677 | 0.146064521 | 0.371561664 | NS | 0.835455262 |
| ZNF568 | -0.133712519 | 0.056701407 | 0.250023562 | NS | 1.246406166 |
| ZNF420 | -0.192303027 | 0.032556964 | 0.199310234 | Down | 1.487356104 |
| ZNF585A | 0.165186228 | 0.016320945 | 0.151094105 | Up | 1.787254694 |
| ZNF585B | 0.042082414 | 0.457930318 | 0.649452133 | NS | 0.339200602 |
| ZNF383 | -0.129881987 | 0.022652232 | 0.171563006 | Down | 1.64488899 |
| ZNF875 | 0.009872643 | 0.855526588 | 0.918750347 | NS | 0.067766489 |
| ZNF527 | -0.183746138 | 0.013203914 | 0.137913995 | Down | 1.879297316 |
| ZNF569 | -0.180191566 | 0.063800882 | 0.262281064 | NS | 1.195173316 |
| ZNF570 | -0.149047884 | 0.041771965 | 0.220293088 | Down | 1.379115096 |
| ZNF793 | 0.021721582 | 0.789491019 | 0.878537356 | NS | 0.102652806 |
| ZNF540 | -0.071170901 | 0.424157374 | 0.623055108 | NS | 0.372472978 |
| ZNF571 | -0.080822204 | 0.353682891 | 0.567005458 | NS | 0.451385948 |
| ZFP30 | -0.074705246 | 0.408811063 | 0.611866175 | NS | 0.38847736 |
| ZNF781 | -0.092586377 | 0.411684773 | 0.614131594 | NS | 0.385435196 |
| ZNF607 | -0.069995365 | 0.331200987 | 0.54817988 | NS | 0.479908378 |
| ZNF573 | -0.019168248 | 0.742210485 | 0.84888192 | NS | 0.129472915 |
| WDR87 | 0.437760111 | 0.272207228 | 0.497465358 | NS | 0.565100346 |
| SIPA1L3 | 0.111206695 | 0.407011935 | 0.610557893 | NS | 0.390392855 |
| DPF1 | 0.557631887 | 0.171620003 | 0.400205856 | NS | 0.765432096 |
| SPINT2 | -0.043906075 | 0.593137113 | 0.74951674 | NS | 0.226844901 |
| PPP1R14A | 0.36032149 | 0.263048625 | NA | NS | 0.579963964 |
| YIF1B | 0.145490628 | 0.106930092 | 0.323758986 | NS | 0.970900059 |
| KCNK6 | -0.020834458 | 0.709462779 | 0.828056089 | NS | 0.149070384 |
| CATSPERG | 0.194822925 | 0.114691918 | 0.333461256 | NS | 0.940467183 |
| PSMD8 | -0.083504629 | 0.190572816 | 0.420149522 | NS | 0.719939048 |
| GGN | 0.463919252 | 0.01481647 | 0.14588426 | Up | 1.829255264 |
| SPRED3 | 0.433911089 | 0.1724105 | 0.401144432 | NS | 0.763436289 |
| FAM98C | 0.035156521 | 0.520667748 | 0.696688569 | NS | 0.283439323 |
| RASGRP4 | 0.053298507 | 0.749801734 | 0.853053121 | NS | 0.125053559 |
| RYR1 | 0.312347167 | 0.231617011 | 0.459829325 | NS | 0.635229547 |
| MAP4K1 | -0.015073573 | 0.899225442 | 0.943953719 | NS | 0.046131414 |
| EIF3K | -0.374758244 | 2.73E-04 | 0.027591492 | Down | 3.563072367 |
| ACTN4 | -0.21236469 | 0.122716672 | 0.343571896 | NS | 0.911096431 |
| CAPN12 | 0.149584721 | 0.423388358 | 0.622593337 | NS | 0.373261088 |
| LGALS4 | -0.069310453 | 0.790590882 | NA | NS | 0.102048199 |
| ECH1 | -0.045747491 | 0.540718619 | 0.710921282 | NS | 0.267028676 |
| HNRNPL | -0.167616256 | 0.036830686 | 0.209195494 | Down | 1.433790194 |
| RINL | -0.046126431 | 0.460483777 | 0.651723712 | NS | 0.336785666 |
| SIRT2 | 0.017066669 | 0.850586169 | 0.916084519 | NS | 0.070281683 |
| NFKBIB | 0.020503805 | 0.844496532 | 0.912766905 | NS | 0.07340213 |
| SARS2 | 0.183422247 | 0.587861672 | NA | NS | 0.230724855 |
| MRPS12 | -0.017387193 | 0.903387691 | 0.946189742 | NS | 0.044125831 |
| FBXO27 | 0.577348843 | 0.133084856 | NA | NS | 0.875871362 |
| ACP7 | 0.787233186 | 0.089407929 | NA | NS | 1.048623963 |
| PAK4 | 0.413899449 | 0.034430666 | 0.203549976 | Up | 1.463054576 |
| NCCRP1 | 0.707089155 | 0.164142 | NA | NS | 0.784780279 |
| SYCN | 1.364729255 | 0.015058295 | NA | Up | 1.8222242 |
| IFNL3 | 1.00520128 | 0.060719436 | NA | NS | 1.216672268 |
| IFNL2 | 0.648152109 | 0.260304898 | NA | NS | 0.58451766 |
| IFNL1 | 0.287173378 | 0.369301331 | NA | NS | 0.432619127 |
| LRFN1 | 0.081037701 | 0.700927003 | 0.822549366 | NS | 0.154327208 |
| GMFG | -0.050885904 | 0.637247584 | 0.779190826 | NS | 0.195691802 |
| SAMD4B | 0.126345015 | 0.368377724 | 0.580230409 | NS | 0.43370664 |
| PAF1 | -0.118319898 | 0.098595502 | 0.31333179 | NS | 1.006142899 |
| MED29 | -0.057826764 | 0.213111047 | 0.442932136 | NS | 0.671394036 |
| ZFP36 | 0.079011795 | 0.53533499 | 0.707471561 | NS | 0.27137437 |
| PLEKHG2 | 0.027794499 | 0.835000757 | 0.906831844 | NS | 0.078313131 |
| RPS16 | 0.036675413 | 0.845529261 | 0.913184055 | NS | 0.072871358 |
| SUPT5H | -0.228234469 | 0.018339347 | 0.157470359 | Down | 1.73661613 |
| TIMM50 | -0.168413008 | 0.009902676 | 0.125229427 | Down | 2.004247415 |
| DLL3 | 0.889618682 | 0.026650633 | NA | Up | 1.574292464 |
| SELENOV | 0.628767897 | 0.197185093 | NA | NS | 0.705125921 |
| EID2B | -0.071987727 | 0.492281036 | 0.676245815 | NS | 0.307786894 |
| EID2 | -0.197961314 | 0.083925826 | 0.291753077 | NS | 1.076104374 |
| LGALS13 | 0.18612927 | 0.769678445 | NA | NS | 0.113690676 |
| LGALS16 | 0.148239466 | 0.780727102 | NA | NS | 0.107500744 |
| LGALS14 | 0.486501242 | 0.300980866 | NA | NS | 0.521461113 |
| CLC | 0.419177548 | 0.095531461 | 0.309119979 | NS | 1.019853579 |
| DYRK1B | -0.108193869 | 0.487811269 | 0.672694188 | NS | 0.311748171 |
| FBL | -0.104078288 | 0.344383982 | 0.559498969 | NS | 0.462957057 |
| FCGBP | -0.268249627 | 0.109693364 | 0.327886489 | NS | 0.959819643 |
| PSMC4 | -0.264767069 | 0.001315828 | 0.058972784 | Down | 2.880800879 |
| ZNF546 | -0.041734327 | 0.748356726 | 0.852044034 | NS | 0.125891334 |
| ZNF780B | -0.201180032 | 0.008561903 | 0.118335308 | Down | 2.067429697 |
| ZNF780A | -0.199487084 | 0.064771584 | 0.26405139 | NS | 1.188615485 |
| MAP3K10 | 0.075351986 | 0.537199585 | 0.708735809 | NS | 0.269864331 |
| TTC9B | 0.929801625 | 0.063743834 | NA | NS | 1.19556182 |
| CCNP | 1.118601245 | 0.010199696 | NA | Up | 1.991412756 |
| AKT2 | -0.086224919 | 0.14292902 | 0.367793306 | NS | 0.844879583 |
| C19orf47 | 0.238310107 | 0.112836377 | 0.331432435 | NS | 0.947550869 |
| PLD3 | 0.002891547 | 0.977324843 | 0.986760726 | NS | 0.009961062 |
| HIPK4 | 0.9748762 | 0.006078673 | NA | Up | 2.216191221 |
| PRX | 0.067567336 | 0.5626867 | 0.72691877 | NS | 0.24973335 |
| SERTAD1 | -0.056919507 | 0.543859624 | 0.713285597 | NS | 0.264513182 |
| SERTAD3 | 0.192144489 | 0.015131706 | 0.147278508 | Up | 1.820112106 |
| BLVRB | -0.114654954 | 0.429846449 | 0.627282309 | NS | 0.366686657 |
| SPTBN4 | 0.399720217 | 0.230511959 | 0.458820172 | NS | 0.637306537 |
| SHKBP1 | -0.047309339 | 0.67517604 | 0.806240246 | NS | 0.170582978 |
| LTBP4 | 0.11289791 | 0.455443894 | 0.647499911 | NS | 0.341565116 |
| NUMBL | 0.324542199 | 0.121114084 | 0.341745092 | NS | 0.916805349 |
| COQ8B | 0.030518018 | 0.53798999 | 0.709108966 | NS | 0.269225805 |
| ITPKC | 0.028470814 | 0.680050887 | 0.809402631 | NS | 0.167458588 |
| C19orf54 | 0.251095859 | 0.12198089 | 0.342773783 | NS | 0.913708204 |
| SNRPA | 0.135822975 | 0.121562532 | 0.342312197 | NS | 0.915200264 |
| MIA | 0.001384127 | 0.995860492 | NA | NS | 0.001801497 |
| RAB4B | 0.14149367 | 0.263161196 | 0.489277294 | NS | 0.579778149 |
| EGLN2 | 0.061719936 | 0.461008819 | 0.652111908 | NS | 0.336290767 |
| CYP2A6 | 0.274700119 | 0.266452749 | 0.492363509 | NS | 0.574379795 |
| CYP2A7 | 0.350900782 | 0.317496209 | 0.536944186 | NS | 0.498261456 |
| CYP2B6 | 0.56271762 | 0.279414936 | 0.503278325 | NS | 0.553750382 |
| CYP2A13 | 0.496000601 | 0.348779947 | NA | NS | 0.457448493 |
| CYP2F1 | 0.570297035 | 0.157936926 | 0.385290564 | NS | 0.80151632 |
| CYP2S1 | 0.085799644 | 0.771298144 | 0.866554152 | NS | 0.112777714 |
| AXL | 0.273166696 | 0.189055086 | 0.41857498 | NS | 0.723411634 |
| HNRNPUL1 | -0.092209009 | 0.436290084 | 0.632079675 | NS | 0.360224658 |
| TGFB1 | 0.183657017 | 0.302919823 | 0.524311995 | NS | 0.518672306 |
| CCDC97 | 0.054635273 | 0.624805068 | 0.771138884 | NS | 0.204255457 |
| TMEM91 | 0.002667934 | 0.976417425 | 0.986397233 | NS | 0.010364479 |
| B9D2 | -0.059051788 | 0.449669761 | 0.643129524 | NS | 0.347106317 |
| EXOSC5 | -0.073892312 | 0.243944686 | 0.471820048 | NS | 0.612708638 |
| BCKDHA | -0.019225118 | 0.87319682 | 0.928388473 | NS | 0.058887855 |
| B3GNT8 | 0.254249439 | 0.025046415 | 0.179152549 | Up | 1.601254428 |
| DMAC2 | 0.180629029 | 0.038357017 | 0.213464789 | Up | 1.41615517 |
| ERICH4 | 0.692077482 | 0.214554557 | NA | NS | 0.668462256 |
| CEACAM21 | -0.184550395 | 0.030699734 | 0.194491094 | Down | 1.51286539 |
| CEACAM4 | 0.166524267 | 0.214256007 | 0.443420485 | NS | 0.669066992 |
| CEACAM7 | 0.660894721 | 0.225815641 | NA | NS | 0.646245979 |
| CEACAM5 | 0.566123876 | 0.136658365 | 0.361144434 | NS | 0.864363779 |
| CEACAM6 | 0.200296354 | 0.330482591 | 0.54753725 | NS | 0.480851413 |
| CEACAM3 | 0.188459325 | 0.198852784 | 0.428784342 | NS | 0.701468325 |
| LYPD4 | 0.164856911 | 0.707609254 | NA | NS | 0.150206496 |
| DMRTC2 | 0.446782083 | 0.235120657 | 0.463083359 | NS | 0.628709213 |
| RPS19 | -0.220170081 | 0.053762926 | 0.245040443 | NS | 1.269517099 |
| CD79A | 0.385838133 | 0.004021242 | 0.090296145 | Up | 2.39563976 |
| ARHGEF1 | -0.300881921 | 0.025356822 | 0.180159958 | Down | 1.595905181 |
| ERFL | -0.01865568 | 0.935042501 | 0.96397808 | NS | 0.029168648 |
| RABAC1 | 0.097000478 | 0.251103131 | 0.477955776 | NS | 0.600147873 |
| ATP1A3 | 0.462884474 | 0.276298015 | 0.50091575 | NS | 0.558622235 |
| GRIK5 | 0.56013334 | 0.156573769 | 0.383636723 | NS | 0.805280995 |
| ZNF574 | -0.158418785 | 0.029951324 | 0.192843748 | Down | 1.523583981 |
| POU2F2 | 0.075202913 | 0.624948951 | 0.771193538 | NS | 0.204155457 |
| DEDD2 | -3.47E-04 | 0.995811814 | 0.997641389 | NS | 0.001822726 |
| ZNF526 | 0.211284429 | 0.03491305 | 0.20492492 | Up | 1.457012211 |
| GSK3A | -0.074151311 | 0.556508208 | 0.722432319 | NS | 0.254528426 |
| ERF | 0.186083908 | 0.09304259 | 0.305431813 | NS | 1.031318207 |
| CIC | -0.081783586 | 0.65992005 | 0.795656172 | NS | 0.180508677 |
| PAFAH1B3 | -0.119021348 | 0.218210003 | 0.447679431 | NS | 0.661125345 |
| PRR19 | 0.433913197 | 0.323565994 | NA | NS | 0.490037127 |
| TMEM145 | 0.632703938 | 0.199270187 | NA | NS | 0.700557672 |
| MEGF8 | 0.137483826 | 0.308586559 | 0.529171648 | NS | 0.510622994 |
| CNFN | 0.719642753 | 0.030600539 | NA | Up | 1.514270925 |
| LIPE | 0.117608053 | 0.313089896 | 0.53275762 | NS | 0.504330947 |
| CXCL17 | 0.346306755 | 0.347872952 | NA | NS | 0.458579338 |
| CEACAM1 | 0.361249378 | 0.012181187 | 0.134186405 | Up | 1.914310402 |
| CEACAM8 | 0.296808052 | 0.159630546 | 0.387107395 | NS | 0.796884002 |
| PSG3 | 0.408429123 | 0.412671007 | NA | NS | 0.384396042 |
| PSG8 | 0.375938025 | 0.413619167 | NA | NS | 0.383399344 |
| PSG1 | 0.542825129 | 0.219004209 | 0.448465163 | NS | 0.659547539 |
| PSG6 | 0.808510432 | 0.111707155 | NA | NS | 0.951919008 |
| PSG7 | 0.72259117 | 0.162694349 | NA | NS | 0.788627532 |
| PSG11 | 0.621093137 | 0.157921767 | 0.385290564 | NS | 0.801558005 |
| PSG2 | 0.762868868 | 0.145266806 | NA | NS | 0.837833613 |
| PSG5 | 0.508849062 | 0.327530772 | NA | NS | 0.484747891 |
| PSG4 | 0.744050502 | 0.090731046 | NA | NS | 1.04224408 |
| PSG9 | 0.627369404 | 0.243118505 | NA | NS | 0.614181984 |
| CD177 | 0.212043782 | 0.462234859 | 0.653019724 | NS | 0.335137306 |
| TEX101 | 0.298880681 | 0.458864619 | NA | NS | 0.338315428 |
| LYPD3 | 0.489424072 | 0.059261491 | NA | NS | 1.227227427 |
| PHLDB3 | 0.028138666 | 0.806412438 | 0.888578902 | NS | 0.093442782 |
| ETHE1 | -0.112735327 | 0.227270962 | 0.455855629 | NS | 0.64345605 |
| ZNF575 | -0.00223902 | 0.987083933 | 0.992295615 | NS | 0.005645917 |
| XRCC1 | 0.001128353 | 0.989815749 | 0.994035648 | NS | 0.004445641 |
| PINLYP | 0.1052238 | 0.681018933 | 0.809988404 | NS | 0.166840814 |
| IRGQ | 0.146056019 | 0.007083422 | 0.110899425 | Up | 2.149756875 |
| ZNF576 | 0.063289918 | 0.283331882 | 0.506309829 | NS | 0.547704553 |
| SRRM5 | 0.138216919 | 0.367348529 | 0.579488635 | NS | 0.434921695 |
| ZNF428 | -0.167800624 | 0.122568988 | 0.343398823 | NS | 0.911619399 |
| CADM4 | -0.006819233 | 0.955811697 | 0.976012104 | NS | 0.019627659 |
| PLAUR | -0.081365917 | 0.374242879 | 0.58496522 | NS | 0.426846455 |
| IRGC | 1.263412889 | 0.010812825 | NA | Up | 1.966060816 |
| SMG9 | 0.110644074 | 0.223650465 | 0.452317462 | NS | 0.650430195 |
| KCNN4 | -0.184617142 | 0.025731786 | 0.181829487 | Down | 1.589530066 |
| LYPD5 | 0.406445536 | 0.332519121 | 0.549210508 | NS | 0.478183377 |
| ZNF283 | -0.071169412 | 0.24022526 | 0.468217208 | NS | 0.619381329 |
| ZNF404 | -0.208617284 | 0.05050455 | 0.237848078 | NS | 1.296669495 |
| ZNF45 | -0.146582744 | 0.001038868 | 0.052796552 | Down | 2.983439657 |
| ZNF221 | -0.078248337 | 0.425252149 | 0.623663211 | NS | 0.371353483 |
| ZNF155 | -0.15184982 | 0.037078743 | 0.209518227 | Down | 1.430874995 |
| ZNF230 | -0.061003051 | 0.506093376 | 0.68631843 | NS | 0.295769347 |
| ZNF222 | -0.178031366 | 0.057376866 | 0.251040799 | NS | 1.241263176 |
| ZNF223 | -0.001371521 | 0.991285676 | 0.9949349 | NS | 0.003801169 |
| ZNF284 | 0.104716411 | 0.232032492 | 0.46031947 | NS | 0.634451195 |
| ZNF224 | -0.020237049 | 0.710515709 | 0.828947059 | NS | 0.148426316 |
| ZNF225 | -0.12106305 | 0.028629051 | 0.189018044 | Down | 1.543193047 |
| ZNF234 | -0.074865573 | 0.159981753 | 0.387550465 | NS | 0.79592955 |
| ZNF226 | -0.113939513 | 0.101506 | 0.317281274 | NS | 0.993508287 |
| ZNF227 | -0.115973686 | 0.029548398 | 0.191596098 | Down | 1.529466054 |
| ZNF235 | -0.049910415 | 0.383437317 | 0.592439708 | NS | 0.416305623 |
| ZNF233 | -0.005085439 | 0.966092382 | 0.980911852 | NS | 0.014981342 |
| ZNF112 | 0.064212992 | 0.705052355 | 0.825367242 | NS | 0.151778633 |
| ZNF285 | -0.023619884 | 0.856740447 | 0.919321075 | NS | 0.06715073 |
| ZNF229 | 0.089828408 | 0.497505282 | 0.679709754 | NS | 0.303202304 |
| ZNF180 | -0.204106003 | 0.006793173 | 0.109684865 | Down | 2.16792731 |
| CEACAM20 | 0.739578431 | 0.120591971 | NA | NS | 0.918681606 |
| IGSF23 | -0.043020729 | 0.872928519 | NA | NS | 0.059021318 |
| PVR | -0.017841199 | 0.839838196 | 0.909641309 | NS | 0.075804377 |
| CEACAM19 | 0.351377617 | 0.157225253 | 0.384615759 | NS | 0.803477698 |
| CEACAM16 | 1.120693233 | 0.0058349 | NA | Up | 2.233966577 |
| BCL3 | 0.120235824 | 0.43238032 | 0.629116067 | NS | 0.364134081 |
| CBLC | 1.105238781 | 0.040292094 | NA | Up | 1.394780156 |
| BCAM | -0.127877224 | 0.704326461 | 0.82477644 | NS | 0.152225995 |
| NECTIN2 | -0.118431725 | 0.629047939 | 0.774041259 | NS | 0.201316256 |
| TOMM40 | -0.188642919 | 0.005310755 | 0.099529109 | Down | 2.274843715 |
| APOE | 1.020600814 | 0.024238895 | NA | Up | 1.615487183 |
| APOC1 | 1.010105039 | 0.097580567 | NA | NS | 1.010636661 |
| APOC4 | 0.475153041 | 0.513638475 | NA | NS | 0.289342452 |
| APOC2 | 0.387171418 | 0.380354924 | NA | NS | 0.419810957 |
| CLPTM1 | 0.080171025 | 0.509284617 | 0.688497544 | NS | 0.293039442 |
| RELB | 0.053821415 | 0.500309583 | 0.682049966 | NS | 0.300761178 |
| CLASRP | -0.152876463 | 0.293277168 | 0.515386052 | NS | 0.532721746 |
| ZNF296 | 0.280576039 | 0.032585414 | 0.199377814 | Up | 1.486976753 |
| GEMIN7 | 0.07390953 | 0.451099648 | 0.644162872 | NS | 0.345727512 |
| MARK4 | -0.058762188 | 0.65446751 | 0.792290225 | NS | 0.184111908 |
| PPP1R37 | 0.131248411 | 0.196028155 | 0.425983188 | NS | 0.707681547 |
| NKPD1 | 0.637868566 | 0.003515959 | 0.085936504 | Up | 2.45395614 |
| TRAPPC6A | -0.274718209 | 0.004715571 | 0.095487183 | Down | 2.326465726 |
| BLOC1S3 | 0.286862807 | 0.026938459 | 0.185246086 | Up | 1.569627253 |
| EXOC3L2 | 1.101438244 | 0.002252727 | 0.073492826 | Up | 2.64729135 |
| CKM | 0.282180815 | 0.257862261 | NA | NS | 0.588612213 |
| KLC3 | 0.187819733 | 0.371679954 | 0.582748977 | NS | 0.429830861 |
| ERCC2 | -0.018319509 | 0.859727804 | 0.920697331 | NS | 0.065639028 |
| PPP1R13L | 0.620279771 | 0.008449219 | 0.117703165 | Up | 2.073183456 |
| POLR1G | 0.051804304 | 0.773318656 | 0.867908648 | NS | 0.111641512 |
| ERCC1 | -0.162979368 | 0.005489504 | 0.100721984 | Down | 2.260466902 |
| FOSB | -0.364484696 | 0.214236403 | 0.443419982 | NS | 0.669106732 |
| RTN2 | 0.182039632 | 0.1342835 | 0.357887095 | NS | 0.871977346 |
| PPM1N | -0.041336292 | 0.709830742 | 0.828316705 | NS | 0.148845196 |
| VASP | 0.024917555 | 0.766063734 | 0.863854529 | NS | 0.115735097 |
| OPA3 | -0.024976999 | 0.668102373 | 0.801680842 | NS | 0.175156985 |
| GPR4 | 0.764015327 | 0.091624616 | NA | NS | 1.037987832 |
| EML2 | -0.15123594 | 0.012174094 | 0.134186405 | Down | 1.914563339 |
| GIPR | 0.13638939 | 0.368865257 | 0.580544833 | NS | 0.433132249 |
| SNRPD2 | -0.327594223 | 0.058570918 | 0.252711222 | NS | 1.232317969 |
| QPCTL | 0.125631873 | 0.275500649 | 0.500040883 | NS | 0.559877374 |
| FBXO46 | 0.191840723 | 0.143718564 | 0.368818142 | NS | 0.84248713 |
| MEIOSIN | 0.716290649 | 0.149380578 | NA | NS | 0.825705865 |
| SIX5 | 0.924512923 | 0.015486333 | NA | Up | 1.810051417 |
| DMPK | 0.092981562 | 0.599901422 | 0.75439633 | NS | 0.221920109 |
| DMWD | 0.118855798 | 0.257065557 | 0.48410524 | NS | 0.589956108 |
| RSPH6A | 0.614146775 | 0.108207592 | NA | NS | 0.965742267 |
| SYMPK | 0.024236239 | 0.845444522 | 0.913147068 | NS | 0.072914886 |
| FOXA3 | 0.996232668 | 0.016419636 | NA | Up | 1.784636479 |
| IRF2BP1 | -0.022593601 | 0.79816565 | 0.883857702 | NS | 0.097906966 |
| MYPOP | 0.302076632 | 0.094303149 | 0.30742606 | NS | 1.025473807 |
| NANOS2 | 0.863959516 | 0.157057629 | NA | NS | 0.803940964 |
| NOVA2 | 0.566857785 | 0.191344416 | 0.42070534 | NS | 0.718184208 |
| CCDC61 | 0.080821035 | 0.307889288 | 0.528756098 | NS | 0.511605421 |
| PGLYRP1 | 0.449006881 | 0.020975273 | 0.167842555 | Up | 1.678292384 |
| IGFL4 | 0.416376631 | 0.114970031 | 0.333658762 | NS | 0.939415353 |
| IGFL3 | 0.638892915 | 0.229065711 | NA | NS | 0.640039916 |
| IGFL2 | 0.277196999 | 0.486210599 | NA | NS | 0.313175578 |
| IGFL1 | 0.505508506 | 0.474939813 | NA | NS | 0.323361424 |
| HIF3A | 0.508030386 | 0.206053349 | 0.43596473 | NS | 0.686020322 |
| PPP5C | -0.157656633 | 0.034101783 | 0.202909595 | Down | 1.467222915 |
| CCDC8 | 0.487055611 | 0.386826587 | NA | NS | 0.412483684 |
| PNMA8C | 0.766931234 | 0.112208733 | NA | NS | 0.94997334 |
| PNMA8A | 0.531206102 | 0.214972627 | NA | NS | 0.667616836 |
| PNMA8B | 0.823471189 | 0.002543524 | NA | Up | 2.594564118 |
| CALM3 | 0.099424922 | 0.221999597 | 0.450738673 | NS | 0.653647814 |
| PTGIR | 0.057022847 | 0.638031019 | 0.779898932 | NS | 0.195158207 |
| GNG8 | 0.506064452 | 0.18365218 | NA | NS | 0.736003911 |
| DACT3 | 0.669774632 | 0.10459124 | 0.320548581 | NS | 0.980504686 |
| PRKD2 | -0.04712688 | 0.6702368 | 0.803358098 | NS | 0.173771731 |
| STRN4 | -0.008369128 | 0.950620451 | 0.973306371 | NS | 0.021992847 |
| FKRP | 0.048499744 | 0.536265002 | 0.708237189 | NS | 0.270620545 |
| SLC1A5 | -0.019881202 | 0.913208816 | 0.951687128 | NS | 0.039429905 |
| AP2S1 | -0.181130684 | 0.037206625 | 0.209820891 | Down | 1.429379719 |
| ARHGAP35 | 0.021586769 | 0.719733467 | 0.834641545 | NS | 0.142828302 |
| NPAS1 | 0.461342166 | 0.165647274 | 0.393591001 | NS | 0.780815707 |
| TMEM160 | 0.606679376 | 2.64E-05 | 0.006431912 | Up | 4.578405493 |
| ZC3H4 | -0.085510201 | 0.511108478 | 0.689903595 | NS | 0.291486915 |
| SAE1 | -0.228867049 | 0.002121182 | 0.072290641 | Down | 2.673422065 |
| BBC3 | -0.136772101 | 0.122014746 | 0.342826857 | NS | 0.913587681 |
| CCDC9 | -0.194263573 | 0.133278336 | 0.35644448 | NS | 0.875240438 |
| INAFM1 | -0.045977175 | 0.726459181 | 0.838611255 | NS | 0.138788783 |
| C5AR1 | 0.29186219 | 0.029561717 | 0.191628203 | Up | 1.529270347 |
| C5AR2 | -0.006254048 | 0.956694015 | 0.976333091 | NS | 0.019226943 |
| DHX34 | 0.046233427 | 0.675736339 | 0.806656198 | NS | 0.170222725 |
| MEIS3 | 0.440389462 | 0.297432948 | 0.519192788 | NS | 0.526610924 |
| SLC8A2 | 0.667828842 | 0.163142178 | 0.390872794 | NS | 0.787433744 |
| KPTN | 0.16398599 | 0.178448295 | 0.408396408 | NS | 0.748487597 |
| NAPA | -0.209399033 | 0.162693164 | 0.390361324 | NS | 0.788630695 |
| ZNF541 | 0.685288801 | 0.052946335 | 0.243196344 | NS | 1.276164094 |
| BICRA | 0.185501624 | 0.389711982 | 0.596947855 | NS | 0.409256242 |
| EHD2 | 0.591436911 | 0.107796037 | 0.325262824 | NS | 0.967397206 |
| NOP53 | -0.036792178 | 0.730613817 | 0.840555934 | NS | 0.136312119 |
| SELENOW | -0.029972414 | 0.744872856 | 0.850360308 | NS | 0.127917852 |
| TPRX1 | 0.84212511 | 0.074095155 | NA | NS | 1.130210188 |
| CRX | 0.9214617 | 0.041745605 | 0.220293088 | Up | 1.379389243 |
| SULT2A1 | 0.706574006 | 0.1937629 | NA | NS | 0.712729375 |
| BSPH1 | 0.835038917 | 0.136836641 | NA | NS | 0.863797596 |
| ELSPBP1 | 0.604999868 | 0.269264555 | NA | NS | 0.569820812 |
| CABP5 | -0.336646788 | 0.068659049 | 0.268653601 | NS | 1.163302219 |
| PLA2G4C | 0.149653244 | 0.379817479 | 0.589694251 | NS | 0.420425053 |
| LIG1 | -0.020567321 | 0.806679882 | 0.888684563 | NS | 0.093298774 |
| ZSWIM9 | 0.064109171 | 0.711613093 | 0.829677903 | NS | 0.14775607 |
| CARD8 | 0.10462564 | 0.094260594 | 0.307415197 | NS | 1.025669826 |
| ZNF114 | 0.187507535 | 0.599762245 | NA | NS | 0.222020877 |
| ODAD1 | 0.400783797 | 0.18974279 | 0.419084713 | NS | 0.721834717 |
| EMP3 | -0.070766989 | 0.413991598 | 0.615784606 | NS | 0.383008473 |
| TMEM143 | 0.103987091 | 0.274226999 | 0.499113624 | NS | 0.561889789 |
| SYNGR4 | 1.107838686 | 0.046910258 | NA | Up | 1.328732181 |
| KDELR1 | 0.180402449 | 0.118089559 | 0.338399285 | NS | 0.927788498 |
| GRIN2D | 0.75052931 | 0.007512233 | 0.113019428 | Up | 2.124230924 |
| GRWD1 | -0.141434105 | 0.043057931 | 0.222799985 | Down | 1.365946843 |
| KCNJ14 | 0.466150196 | 0.038301551 | NA | Up | 1.416783644 |
| CYTH2 | -0.065256646 | 0.286814367 | 0.509965128 | NS | 0.542399098 |
| LMTK3 | 0.347640703 | 0.08683852 | 0.296188519 | NS | 1.061287589 |
| SULT2B1 | 0.124003069 | 0.727360481 | 0.838753198 | NS | 0.138250299 |
| FAM83E | 0.546554907 | 0.010200946 | 0.126233693 | Up | 1.991359541 |
| SPACA4 | 1.218376127 | 0.006179023 | NA | Up | 2.209080158 |
| RPL18 | -0.23499 | 0.032350018 | 0.19862172 | Down | 1.490125467 |
| SPHK2 | 0.17260871 | 0.10292911 | 0.318730365 | NS | 0.987461782 |
| DBP | 0.053849217 | 0.379129383 | 0.589025245 | NS | 0.421212556 |
| CA11 | 0.015092138 | 0.930236766 | 0.961856154 | NS | 0.0314065 |
| NTN5 | 0.474930196 | 0.141842435 | NA | NS | 0.848193822 |
| FUT2 | 0.042879879 | 0.773715324 | 0.868056059 | NS | 0.111418802 |
| MAMSTR | 0.294237382 | 0.198973863 | 0.428784342 | NS | 0.701203968 |
| RASIP1 | 0.649904066 | 0.055497749 | 0.248604264 | NS | 1.255724633 |
| IZUMO1 | 0.18987536 | 0.656950903 | 0.79397469 | NS | 0.182467086 |
| FUT1 | 0.4745941 | 0.196699141 | NA | NS | 0.706197536 |
| FGF21 | 0.680302899 | 0.217328499 | NA | NS | 0.66288332 |
| BCAT2 | 0.064818242 | 0.384952208 | 0.59332226 | NS | 0.414593185 |
| HSD17B14 | 0.269262216 | 0.542295401 | NA | NS | 0.265764079 |
| PLEKHA4 | 0.563569026 | 0.067992459 | 0.268106627 | NS | 1.167539254 |
| PPP1R15A | -0.19674575 | 0.116350181 | 0.335809937 | NS | 0.934232936 |
| TULP2 | 0.533739837 | 0.05827567 | 0.252197542 | NS | 1.234512726 |
| NUCB1 | -0.073318975 | 0.630300734 | 0.774958255 | NS | 0.200452187 |
| DHDH | 0.422698916 | 0.138881852 | NA | NS | 0.857354499 |
| BAX | -0.102340479 | 0.052399339 | 0.241847979 | NS | 1.280674189 |
| FTL | -0.24456165 | 0.043807094 | 0.224344829 | Down | 1.358455551 |
| GYS1 | -0.004720483 | 0.955051142 | 0.975781763 | NS | 0.019973372 |
| RUVBL2 | -0.189122003 | 0.017547633 | 0.15540444 | Down | 1.755781467 |
| LHB | 0.210640771 | 0.432433048 | NA | NS | 0.364081123 |
| CGB2 | 1.679871932 | 0.031129023 | NA | Up | 1.506834503 |
| CGB1 | 0.342750491 | 0.796394089 | NA | NS | 0.098871972 |
| CGB5 | 1.26274273 | 0.095511307 | NA | NS | 1.01994521 |
| CGB8 | 0.300337162 | 0.67674233 | NA | NS | 0.169576658 |
| CGB7 | 0.780320918 | 0.148299951 | NA | NS | 0.828858993 |
| NTF4 | 0.536460498 | 0.416555616 | NA | NS | 0.380327006 |
| KCNA7 | 0.635184587 | 0.171526074 | NA | NS | 0.765669854 |
| SNRNP70 | -0.027448738 | 0.799657514 | 0.884702387 | NS | 0.097095978 |
| LIN7B | 0.094481702 | 0.506153099 | 0.68631843 | NS | 0.295718099 |
| C19orf73 | -0.018358611 | 0.939551361 | NA | NS | 0.027079474 |
| PPFIA3 | 0.32909394 | 0.137007683 | 0.361533582 | NS | 0.863255078 |
| HRC | 0.508436921 | 0.339007102 | NA | NS | 0.469791203 |
| TRPM4 | 0.41853576 | 0.083057671 | 0.290277244 | NS | 1.080620251 |
| SLC6A16 | 0.079591045 | 0.493481281 | 0.677316811 | NS | 0.306729316 |
| CD37 | 0.114596891 | 0.122732696 | 0.343571896 | NS | 0.911039726 |
| TEAD2 | 0.141730685 | 0.5036862 | 0.684554113 | NS | 0.297839948 |
| DKKL1 | 0.353141832 | 0.486726075 | NA | NS | 0.312715387 |
| KASH5 | 0.523966507 | 0.184611952 | 0.414734144 | NS | 0.733740185 |
| PTH2 | 1.528460716 | 0.018671453 | NA | Up | 1.728821873 |
| GFY | 0.452621749 | 0.344734827 | 0.559649007 | NS | 0.462514839 |
| SLC17A7 | 0.713315183 | 0.094424873 | 0.307427263 | NS | 1.024913593 |
| PIH1D1 | -0.206710567 | 0.002688275 | 0.078629458 | Down | 2.570526368 |
| ALDH16A1 | -0.143707406 | 0.214117506 | 0.443414335 | NS | 0.669347823 |
| FLT3LG | -0.064956233 | 0.699821181 | 0.822143007 | NS | 0.155012917 |
| RPL13A | -0.326417841 | 0.013948386 | 0.141407346 | Down | 1.855476044 |
| RPS11 | -0.367941036 | 0.002667267 | 0.078516379 | Down | 2.573933548 |
| FCGRT | -0.047087674 | 0.57710322 | 0.737669138 | NS | 0.238746502 |
| RCN3 | -0.119609461 | 0.603516241 | 0.756695228 | NS | 0.219311038 |
| NOSIP | 0.002733073 | 0.973732436 | 0.985087916 | NS | 0.011560363 |
| PRRG2 | -0.023417844 | 0.910529466 | 0.950414811 | NS | 0.040705995 |
| PRR12 | -0.125760807 | 0.619334622 | 0.767200428 | NS | 0.208074641 |
| RRAS | 0.017888596 | 0.873743872 | 0.928779593 | NS | 0.058615857 |
| SCAF1 | -0.105136723 | 0.573565482 | 0.735034227 | NS | 0.241416993 |
| IRF3 | -0.096432452 | 0.174915045 | 0.404269285 | NS | 0.757172835 |
| BCL2L12 | 0.137689353 | 0.277248336 | 0.50158659 | NS | 0.557131052 |
| PRMT1 | -0.101943162 | 0.166802442 | 0.394802555 | NS | 0.777797596 |
| ADM5 | 0.333442772 | 0.346540083 | NA | NS | 0.460246525 |
| CPT1C | 0.338538702 | 0.39165241 | 0.598413736 | NS | 0.407099197 |
| TSKS | 0.178449715 | 0.541318477 | 0.711383242 | NS | 0.266547149 |
| AP2A1 | -0.21077865 | 0.297609686 | 0.519257109 | NS | 0.526352938 |
| FUZ | 0.002645907 | 0.975770435 | 0.98610425 | NS | 0.010652345 |
| MED25 | -0.108959319 | 0.52676487 | 0.701719931 | NS | 0.278383196 |
| PTOV1 | -0.085934137 | 0.329343833 | 0.546844955 | NS | 0.482350464 |
| PNKP | -0.051422891 | 0.602387069 | 0.756016283 | NS | 0.220124359 |
| AKT1S1 | 0.026482618 | 0.81276969 | 0.892717445 | NS | 0.090032501 |
| TBC1D17 | -0.071009164 | 0.614605019 | 0.764203112 | NS | 0.211403898 |
| IL4I1 | 0.107139475 | 0.581228479 | 0.740503706 | NS | 0.235653115 |
| NUP62 | -0.117903569 | 0.188683603 | 0.417994959 | NS | 0.724265839 |
| ATF5 | 0.132758291 | 0.433737113 | 0.630135929 | NS | 0.362773415 |
| SIGLEC11 | 0.099127544 | 0.667602133 | 0.80123442 | NS | 0.175482285 |
| VRK3 | -0.012464275 | 0.8010844 | 0.885488437 | NS | 0.096321726 |
| ZNF473 | -0.076309578 | 0.100277848 | 0.315396306 | NS | 0.998794994 |
| IZUMO2 | 0.639618521 | 0.168372922 | NA | NS | 0.773727752 |
| MYH14 | 0.680868172 | 0.089294408 | 0.299768474 | NS | 1.049175739 |
| KCNC3 | 0.343550977 | 0.13286264 | 0.356202467 | NS | 0.876597121 |
| NR1H2 | 0.172464087 | 0.048812815 | 0.234117506 | Up | 1.311466146 |
| NAPSA | 0.215091189 | 0.178568219 | 0.408483 | NS | 0.748195832 |
| POLD1 | -0.133821574 | 0.108633865 | 0.326644764 | NS | 0.964034771 |
| SPIB | -0.177790737 | 0.123629945 | 0.344575027 | NS | 0.907876325 |
| MYBPC2 | 0.611278102 | 0.080845701 | 0.286747443 | NS | 1.09234307 |
| FAM71E1 | 0.693361052 | 0.088013079 | NA | NS | 1.055452788 |
| EMC10 | -0.031432246 | 0.605296691 | 0.758010981 | NS | 0.2180317 |
| JOSD2 | 0.327029683 | 0.001298336 | 0.058839026 | Up | 2.886613045 |
| ASPDH | 0.377812157 | 0.017327845 | 0.154539288 | Up | 1.761255435 |
| LRRC4B | 0.86865481 | 0.002184982 | 0.072832159 | Up | 2.660552062 |
| SYT3 | 0.549296341 | 0.198222533 | 0.428143472 | NS | 0.702846978 |
| C19orf81 | 0.604771996 | 0.233860465 | NA | NS | 0.631043192 |
| SHANK1 | 0.474829379 | 0.197699787 | 0.427455633 | NS | 0.703993798 |
| CLEC11A | -0.073326728 | 0.704442542 | 0.824844987 | NS | 0.152154425 |
| GPR32 | 0.685941039 | 0.23866615 | NA | NS | 0.622209173 |
| ACP4 | 0.215812168 | 0.222873921 | 0.451584405 | NS | 0.651940747 |
| KLK1 | 0.14167865 | 0.740066532 | 0.847450185 | NS | 0.130729236 |
| KLK15 | 0.391498546 | 0.392154467 | 0.598861212 | NS | 0.406542834 |
| KLK3 | 0.437379812 | 0.314244008 | 0.53361135 | NS | 0.502732995 |
| KLK2 | 0.598604844 | 0.159176044 | 0.386908274 | NS | 0.798122293 |
| KLK4 | 0.404078799 | 0.424001872 | NA | NS | 0.372632226 |
| KLK5 | 0.901677811 | 0.151299335 | NA | NS | 0.820162981 |
| KLK6 | 0.551240266 | 0.266832344 | NA | NS | 0.573761528 |
| KLK7 | 0.912723527 | 0.071249213 | NA | NS | 1.147219931 |
| KLK8 | 0.982536241 | 0.111148704 | NA | NS | 0.954095596 |
| KLK10 | 0.564470176 | 0.201802172 | 0.43177068 | NS | 0.695074163 |
| KLK11 | 0.29560468 | 0.546764563 | 0.715216017 | NS | 0.262199641 |
| KLK12 | 0.665778602 | 0.227916881 | NA | NS | 0.642223507 |
| KLK13 | 0.280590951 | 0.588032995 | NA | NS | 0.230598304 |
| KLK14 | 0.52973099 | 0.22067275 | NA | NS | 0.656251292 |
| CTU1 | 0.085090437 | 0.405064219 | 0.608752592 | NS | 0.392476118 |
| SIGLEC9 | 0.056242322 | 0.526351983 | 0.701317887 | NS | 0.278723736 |
| SIGLEC7 | -0.165666781 | 0.082974447 | 0.290260272 | NS | 1.081055635 |
| CD33 | -0.019032429 | 0.836459925 | 0.908025084 | NS | 0.077554862 |
| SIGLECL1 | 0.854206008 | 0.048222019 | NA | Up | 1.316754615 |
| IGLON5 | 0.632594304 | 0.212612668 | NA | NS | 0.672410862 |
| VSIG10L | 0.357844758 | 0.24859425 | 0.476227981 | NS | 0.604508921 |
| ETFB | -0.056622166 | 0.406649636 | 0.610228172 | NS | 0.390779612 |
| CLDND2 | -0.042604597 | 0.725225068 | 0.838202171 | NS | 0.139527193 |
| NKG7 | 0.383150266 | 0.002662243 | 0.078469363 | Up | 2.574752262 |
| LIM2 | 0.1177395 | 0.517527999 | 0.694871679 | NS | 0.286066149 |
| C19orf84 | 0.115282627 | 0.623281482 | 0.770131778 | NS | 0.205315776 |
| SIGLEC10 | -0.070262308 | 0.435797674 | 0.631685971 | NS | 0.360715092 |
| SIGLEC8 | 0.51264309 | 0.035201512 | 0.205507211 | Up | 1.453438687 |
| CEACAM18 | 0.969463332 | 0.059295868 | NA | NS | 1.226975569 |
| SIGLEC12 | 0.110173741 | 0.760013204 | NA | NS | 0.119178862 |
| SIGLEC6 | 0.031209804 | 0.837359856 | 0.908571347 | NS | 0.077087864 |
| ZNF175 | -0.073438309 | 0.161715983 | 0.389444735 | NS | 0.791247056 |
| SIGLEC5 | 0.194580861 | 0.153087074 | 0.379704589 | NS | 0.815061477 |
| SIGLEC14 | 0.502022495 | 0.010374835 | 0.127182759 | Up | 1.984018787 |
| SPACA6 | 0.062687578 | 0.644958912 | 0.785432787 | NS | 0.190467952 |
| HAS1 | 0.347664216 | 0.121122387 | 0.341745092 | NS | 0.916775579 |
| FPR1 | 0.22866047 | 0.129842414 | 0.351911357 | NS | 0.886583418 |
| FPR2 | 0.111615246 | 0.479574697 | 0.666669523 | NS | 0.319143739 |
| FPR3 | -0.18782232 | 0.213478869 | 0.443180155 | NS | 0.670645108 |
| ZNF577 | -0.075825406 | 0.310811436 | 0.530654802 | NS | 0.50750301 |
| ZNF649 | -0.062540232 | 0.299151491 | 0.520433287 | NS | 0.524108829 |
| ZNF613 | -0.134306396 | 0.097025979 | 0.311260536 | NS | 1.013111967 |
| ZNF350 | -0.101710376 | 0.049206936 | 0.234974405 | Down | 1.307973677 |
| ZNF615 | -0.144871472 | 0.024629654 | 0.177746811 | Down | 1.608541684 |
| ZNF614 | -0.096632666 | 0.207345006 | 0.437297664 | NS | 0.68330642 |
| ZNF432 | -0.205043218 | 0.018183939 | 0.156913557 | Down | 1.740312023 |
| ZNF841 | -0.103808461 | 0.037389615 | 0.210115856 | Down | 1.427249011 |
| ZNF616 | -0.092588957 | 0.151946859 | 0.378507048 | NS | 0.818308273 |
| ZNF836 | -0.140842325 | 0.024754526 | 0.178279295 | Down | 1.606345381 |
| PPP2R1A | -0.04616794 | 0.384316257 | 0.593034804 | NS | 0.415311244 |
| ZNF766 | -0.219801216 | 0.003349563 | 0.085084692 | Down | 2.475011855 |
| ZNF480 | -0.243754665 | 0.010258062 | 0.126284714 | Down | 1.988934689 |
| ZNF610 | 0.064472654 | 0.708248333 | 0.827160771 | NS | 0.149814439 |
| ZNF880 | -0.181366248 | 0.044627469 | 0.225889772 | Down | 1.350397747 |
| ZNF528 | 0.121850456 | 0.105007572 | 0.320907849 | NS | 0.978779382 |
| ZNF534 | 0.13738815 | 0.729175597 | 0.840103608 | NS | 0.137167874 |
| ZNF578 | 0.28927018 | 0.224061593 | 0.452509578 | NS | 0.64963258 |
| ZNF808 | -0.048666033 | 0.3084841 | 0.529171648 | NS | 0.510767216 |
| ZNF701 | -0.038205917 | 0.544854087 | 0.713725092 | NS | 0.263719787 |
| ZNF83 | -0.002767943 | 0.974536712 | 0.985403414 | NS | 0.011201796 |
| ZNF611 | -0.045144918 | 0.312521236 | 0.532199891 | NS | 0.505120467 |
| ZNF600 | -0.133069113 | 0.188639184 | 0.417936983 | NS | 0.724368091 |
| ZNF28 | -0.161985038 | 0.062000117 | 0.258700409 | NS | 1.207607492 |
| ZNF468 | -0.188607492 | 0.021260138 | 0.168379028 | Down | 1.67243393 |
| ZNF320 | -0.073460212 | 0.270293758 | 0.495776682 | NS | 0.568163983 |
| ZNF888 | -0.155641059 | 0.044732573 | 0.226151301 | Down | 1.349376118 |
| ZNF816 | -0.211693655 | 0.043982242 | 0.224627854 | Down | 1.356722639 |
| ERVV-1 | 0.55403851 | 0.269198134 | NA | NS | 0.569927955 |
| ERVV-2 | 0.392316945 | 0.44583941 | NA | NS | 0.350821545 |
| ZNF160 | -0.085523282 | 0.018650235 | 0.158871289 | NS | 1.729315697 |
| ZNF415 | 0.068347956 | 0.699417485 | 0.822044571 | NS | 0.155263515 |
| ZNF347 | -0.021268471 | 0.756350245 | 0.857479002 | NS | 0.121277048 |
| ZNF665 | 0.112772817 | 0.262786988 | 0.488909438 | NS | 0.580396143 |
| ZNF677 | 0.091502803 | 0.439370958 | 0.634536112 | NS | 0.357168653 |
| VN1R2 | 0.231144488 | 0.687418699 | NA | NS | 0.162778658 |
| VN1R4 | 0.631677664 | 0.318621314 | NA | NS | 0.496725176 |
| ZNF845 | -0.289373032 | 0.010883338 | 0.12856202 | Down | 1.96323787 |
| ZNF525 | -0.340268385 | 0.002222774 | 0.072832159 | Down | 2.653104604 |
| ZNF765 | -0.254404326 | 0.012328 | 0.134659948 | Down | 1.909107365 |
| ZNF761 | -0.244947927 | 0.004195657 | 0.091951138 | Down | 2.377200011 |
| ZNF813 | -0.204949174 | 0.026359646 | 0.183162888 | Down | 1.579060427 |
| ZNF331 | 0.013665788 | 0.822662993 | 0.899047461 | NS | 0.084778039 |
| DPRX | 0.542012081 | 0.368598267 | NA | NS | 0.43344671 |
| NLRP12 | -0.022366351 | 0.815555221 | 0.894875703 | NS | 0.088546628 |
| MYADM | 0.245672739 | 0.05776524 | 0.251746134 | NS | 1.238333421 |
| PRKCG | 0.714083558 | 0.111084148 | 0.329625441 | NS | 0.954347912 |
| CACNG7 | 0.954064279 | 0.060656979 | NA | NS | 1.217119221 |
| CACNG8 | 0.587523524 | 0.019454428 | 0.161936818 | Up | 1.710981528 |
| CACNG6 | 0.424727006 | 0.045172439 | 0.227271351 | Up | 1.345126463 |
| VSTM1 | 0.15169198 | 0.394030237 | 0.600042784 | NS | 0.40447045 |
| TARM1 | 0.750283725 | 0.079727769 | NA | NS | 1.098390387 |
| OSCAR | 0.104475682 | 0.445121711 | 0.639053249 | NS | 0.351521222 |
| NDUFA3 | -0.20013182 | 0.025238287 | 0.17961692 | Down | 1.597940125 |
| TFPT | -0.202028269 | 0.093195609 | 0.305607938 | NS | 1.030604549 |
| PRPF31 | -0.380377798 | 1.14E-04 | 0.016924534 | Down | 3.943835958 |
| CNOT3 | -0.314217626 | 0.201009985 | 0.430601586 | NS | 0.696782369 |
| LENG1 | -0.382373053 | 4.10E-04 | 0.034543974 | Down | 3.386932212 |
| TMC4 | -0.04113364 | 0.890242561 | NA | NS | 0.050491647 |
| MBOAT7 | 0.316106742 | 0.030862354 | 0.195143464 | Up | 1.510570954 |
| TSEN34 | 0.096766466 | 0.347915168 | 0.562390822 | NS | 0.458526637 |
| RPS9 | -0.246724726 | 0.055581483 | 0.248667148 | NS | 1.255069868 |
| LILRB3 | 0.221478783 | 0.159940767 | 0.387533163 | NS | 0.796040825 |
| LILRA6 | 0.462242073 | 0.007907034 | 0.11472857 | Up | 2.101986403 |
| LILRB2 | 0.179341042 | 0.248871276 | 0.476376043 | NS | 0.604025225 |
| LILRA5 | 0.0269107 | 0.851376393 | 0.916355684 | NS | 0.069878396 |
| LILRA4 | -0.171949976 | 0.470744114 | NA | NS | 0.327215102 |
| LAIR1 | -0.01849219 | 0.827628757 | 0.901779381 | NS | 0.082164428 |
| LENG8 | -0.152337192 | 0.407666467 | 0.611075752 | NS | 0.38969501 |
| LAIR2 | -0.701116124 | 0.003180773 | 0.084307443 | Down | 2.497467265 |
| LILRA2 | 0.229324717 | 0.041011339 | 0.218135377 | Up | 1.387096053 |
| LILRA1 | 0.078674711 | 0.372176935 | 0.583052983 | NS | 0.429250545 |
| LILRB1 | 0.231674333 | 0.086359734 | 0.295291694 | NS | 1.063688706 |
| LILRB4 | 0.290582408 | 0.034540967 | 0.204038493 | Up | 1.461665503 |
| KIR2DL3 | 0.749025167 | 0.041907248 | NA | Up | 1.377710858 |
| KIR2DL1 | 0.843939908 | 0.021797904 | NA | Up | 1.661585259 |
| KIR3DL1 | 0.374507892 | 0.268067535 | NA | NS | 0.571755779 |
| KIR3DL2 | -0.231873903 | 0.459156626 | NA | NS | 0.338039144 |
| FCAR | 0.288154858 | 0.023711817 | 0.174782118 | Up | 1.625035159 |
| NCR1 | -0.180925061 | 0.177771938 | 0.407680235 | NS | 0.750136794 |
| NLRP7 | -0.460212955 | 0.155934701 | NA | NS | 0.807057227 |
| NLRP2 | 0.12274233 | 0.493337135 | 0.677207384 | NS | 0.306856192 |
| GP6 | 0.19838398 | 0.403415603 | NA | NS | 0.394247308 |
| RDH13 | 0.0321485 | 0.838236922 | 0.90890645 | NS | 0.076633213 |
| EPS8L1 | 0.219872164 | 0.218471502 | 0.447886776 | NS | 0.660605206 |
| PPP1R12C | -0.040837612 | 0.734653869 | 0.843487387 | NS | 0.13391723 |
| TNNT1 | 0.20363013 | 0.332870282 | 0.549631954 | NS | 0.477724976 |
| TNNI3 | 0.825831439 | 0.077528984 | NA | NS | 1.110535905 |
| DNAAF3 | 1.096269645 | 0.013752082 | NA | Up | 1.861631562 |
| SYT5 | 1.018791518 | 0.02189829 | 0.169487881 | Up | 1.659589796 |
| PTPRH | 0.245609579 | 0.487452289 | 0.67234596 | NS | 0.312067886 |
| TMEM86B | -0.055424222 | 0.760439136 | 0.860155929 | NS | 0.11893554 |
| PPP6R1 | -0.058825459 | 0.716037557 | 0.832083019 | NS | 0.145064198 |
| HSPBP1 | -0.065682491 | 0.546317291 | 0.714753418 | NS | 0.262555054 |
| BRSK1 | 0.140564887 | 0.543165724 | 0.712992572 | NS | 0.265067644 |
| TMEM150B | 0.162874183 | 0.272752468 | 0.498012997 | NS | 0.564231311 |
| KMT5C | 0.201930687 | 0.130285232 | 0.352156503 | NS | 0.885104808 |
| COX6B2 | 0.557792546 | 0.104148667 | NA | NS | 0.982346284 |
| FAM71E2 | 0.687179992 | 0.17350806 | NA | NS | 0.760680347 |
| IL11 | 0.743988484 | 0.088060246 | NA | NS | 1.055220105 |
| TMEM190 | 0.716792843 | 0.083321752 | NA | NS | 1.079241609 |
| TMEM238 | 0.23874725 | 0.322972548 | NA | NS | 0.49083439 |
| RPL28 | 0.36353245 | 3.80E-04 | 0.03343235 | Up | 3.420733157 |
| UBE2S | 0.010205292 | 0.905816002 | 0.947779517 | NS | 0.042960011 |
| SHISA7 | 0.058003641 | 0.870202177 | 0.926775031 | NS | 0.060379834 |
| ISOC2 | 0.087065326 | 0.477210446 | 0.664987747 | NS | 0.321290059 |
| C19orf85 | 1.008271417 | 0.030087057 | NA | Up | 1.521620294 |
| ZNF628 | 0.47799723 | 0.022457589 | 0.171252981 | Up | 1.648636879 |
| NAT14 | 0.371514307 | 0.052693984 | 0.242513843 | NS | 1.278238965 |
| SSC5D | 0.825723528 | 0.022152826 | 0.170421238 | Up | 1.654570859 |
| SBK2 | 0.742239337 | 0.193763808 | NA | NS | 0.712727339 |
| SBK3 | 0.844156666 | 0.107024881 | NA | NS | 0.970515247 |
| ZNF579 | 0.657428273 | 0.002799944 | 0.079460125 | Up | 2.552850634 |
| FIZ1 | 0.086288169 | 0.250932093 | 0.47774934 | NS | 0.60044379 |
| ZNF524 | 0.099574613 | 0.189714586 | 0.419084713 | NS | 0.721899279 |
| ZNF865 | 0.093315855 | 0.742112774 | 0.848841757 | NS | 0.129530093 |
| ZNF784 | 0.038108549 | 0.664241564 | 0.798973755 | NS | 0.177673952 |
| ZNF580 | 0.034150962 | 0.725920416 | 0.838611255 | NS | 0.139110989 |
| ZNF581 | 0.007828651 | 0.932879556 | 0.963029416 | NS | 0.030174424 |
| CCDC106 | 0.181293431 | 0.179920259 | 0.409996481 | NS | 0.744919932 |
| U2AF2 | -0.08575073 | 0.175541504 | 0.404940726 | NS | 0.755620185 |
| EPN1 | -0.046996284 | 0.781856225 | 0.873857226 | NS | 0.106873102 |
| NLRP9 | 0.254419969 | 0.500093379 | NA | NS | 0.300948895 |
| RFPL4A | -0.025817368 | 0.960380362 | NA | NS | 0.017556729 |
| RFPL4AL1 | 0.263064862 | 0.630708226 | NA | NS | 0.200171505 |
| NLRP11 | 0.437546553 | 0.297592965 | NA | NS | 0.52637734 |
| NLRP4 | 0.22021408 | 0.45677664 | NA | NS | 0.340296114 |
| NLRP13 | 0.635974369 | 0.197606507 | NA | NS | 0.704198758 |
| NLRP8 | 0.757849981 | 0.089253431 | NA | NS | 1.049375081 |
| NLRP5 | 0.733345444 | 0.134143655 | NA | NS | 0.872429865 |
| ZNF787 | 0.035880081 | 0.665267859 | 0.799541399 | NS | 0.177003458 |
| ZNF444 | 0.241881277 | 0.065297408 | 0.264481527 | NS | 1.185104059 |
| GALP | 0.378772351 | 0.516954465 | NA | NS | 0.286547709 |
| ZSCAN5B | 0.305669441 | 0.530090737 | NA | NS | 0.275649785 |
| ZSCAN5C | 0.338254419 | 0.479353039 | NA | NS | 0.319344515 |
| ZSCAN5A | -0.125647416 | 0.103430042 | 0.31928753 | NS | 0.9853533 |
| EDDM13 | 0.197559716 | 0.165606001 | 0.393591001 | NS | 0.780923929 |
| ZNF582 | -0.209110351 | 0.015016672 | 0.146973997 | Down | 1.823426303 |
| ZNF583 | -0.11066221 | 0.245888461 | 0.474098126 | NS | 0.60926185 |
| ZNF667 | 0.037060315 | 0.824794186 | 0.900225728 | NS | 0.083654409 |
| ZNF471 | -0.033957903 | 0.821390804 | 0.898309164 | NS | 0.085450163 |
| ZFP28 | 0.094639853 | 0.265381119 | 0.491568941 | NS | 0.576129979 |
| ZNF470 | -0.02923515 | 0.712251829 | 0.830016761 | NS | 0.147366427 |
| ZNF71 | 0.023651685 | 0.712060382 | 0.829945894 | NS | 0.147483177 |
| SMIM17 | 0.359921361 | 0.290659757 | NA | NS | 0.536615093 |
| ZNF835 | 0.099932085 | 0.467202023 | 0.656916793 | NS | 0.330495286 |
| ZIM2 | 0.533580206 | 0.297402435 | NA | NS | 0.52665548 |
| PEG3 | 0.312201464 | 0.393013897 | NA | NS | 0.405592092 |
| USP29 | 0.711351816 | 0.151394324 | NA | NS | 0.819890408 |
| ZIM3 | 0.070593527 | 0.894085253 | NA | NS | 0.048621068 |
| DUXA | 0.430644013 | 0.44671954 | NA | NS | 0.349965051 |
| ZNF264 | -0.015935537 | 0.624361364 | 0.770862645 | NS | 0.204563979 |
| AURKC | 0.391605357 | 0.185992511 | NA | NS | 0.730504542 |
| ZNF805 | -0.024695634 | 0.586571067 | 0.745196127 | NS | 0.231679362 |
| ZNF460 | -0.015245793 | 0.780718027 | 0.873306158 | NS | 0.107505793 |
| ZNF543 | -0.092621838 | 0.10079586 | 0.316216401 | NS | 0.996557307 |
| ZNF304 | -0.213943752 | 0.002915182 | 0.080729745 | Down | 2.535334322 |
| ZNF547 | 0.028840725 | 0.773489615 | 0.867972912 | NS | 0.111545513 |
| TRAPPC2B | -0.163887159 | 0.220992355 | 0.449641695 | NS | 0.65562275 |
| ZNF548 | -0.090651411 | 0.087358045 | 0.296859543 | NS | 1.058697094 |
| ZNF17 | -0.129364344 | 0.075395335 | 0.278545567 | NS | 1.122655523 |
| ZNF749 | 0.023062901 | 0.779018131 | 0.871827269 | NS | 0.108452435 |
| VN1R1 | 0.133467824 | 0.404480489 | 0.608303576 | NS | 0.393102423 |
| ZNF772 | -0.230682759 | 0.007121534 | 0.111026118 | Down | 2.147426458 |
| ZNF419 | -0.049887786 | 0.456251316 | 0.64794489 | NS | 0.34079587 |
| ZNF773 | -0.034077534 | 0.622189572 | 0.769280656 | NS | 0.206077272 |
| ZNF549 | -0.01668422 | 0.75222267 | 0.85466151 | NS | 0.123653582 |
| ZNF550 | 0.002683621 | 0.966828107 | 0.98148481 | NS | 0.014650733 |
| ZNF416 | -0.073229955 | 0.354670723 | 0.567939373 | NS | 0.450174659 |
| ZIK1 | -0.122507437 | 0.174194241 | 0.403422181 | NS | 0.758966208 |
| ZNF530 | -0.117594826 | 0.058272105 | 0.252197542 | NS | 1.234539291 |
| ZNF134 | -0.092186312 | 0.120001775 | 0.340700614 | NS | 0.920812331 |
| ZNF211 | -0.148583123 | 0.002748361 | 0.079323886 | Down | 2.560926298 |
| ZSCAN4 | 0.375212147 | 0.367041394 | NA | NS | 0.435284954 |
| ZNF551 | -0.105589772 | 0.109217349 | 0.327173527 | NS | 0.961708367 |
| ZNF154 | 5.38E-04 | 0.996116941 | 0.997710407 | NS | 0.001689674 |
| ZNF671 | -0.072713759 | 0.206440992 | 0.436355477 | NS | 0.685204064 |
| ZNF776 | -0.04984802 | 0.384659073 | 0.59325935 | NS | 0.414924019 |
| ZNF586 | -0.013545555 | 0.772785684 | 0.867693177 | NS | 0.111940932 |
| ZNF552 | -0.055707067 | 0.348708407 | 0.562790769 | NS | 0.457537582 |
| ZNF587B | 0.012548147 | 0.782995088 | 0.874459109 | NS | 0.106240962 |
| ZNF814 | 0.079395417 | 0.189740819 | 0.419084713 | NS | 0.721839229 |
| ZNF587 | 0.131286304 | 0.004783961 | 0.095771218 | Up | 2.320212376 |
| ZNF417 | -0.062378402 | 0.197415035 | 0.427334512 | NS | 0.704619775 |
| ZNF418 | -0.140808134 | 0.231561891 | 0.45979109 | NS | 0.635332912 |
| ZNF256 | -0.074583287 | 0.377295736 | 0.587252069 | NS | 0.423318103 |
| C19orf18 | 0.064266852 | 0.768618456 | NA | NS | 0.114289191 |
| ZNF606 | -0.08875887 | 0.113432401 | 0.33220521 | NS | 0.945262877 |
| ZSCAN1 | 0.706340475 | 0.179525256 | NA | NS | 0.745874446 |
| ZNF135 | -0.04715289 | 0.710608273 | 0.828951459 | NS | 0.14836974 |
| ZSCAN18 | -0.039542512 | 0.630763608 | 0.775188655 | NS | 0.200133371 |
| ZNF329 | -0.186587384 | 0.01169272 | 0.132526766 | Down | 1.932084435 |
| ZNF274 | -0.076142572 | 0.051020198 | 0.238791409 | NS | 1.292257858 |
| ZNF544 | -0.092231158 | 0.128049923 | 0.349785226 | NS | 0.892620678 |
| ZNF8 | 0.127894503 | 0.051322507 | 0.239386568 | NS | 1.28969214 |
| ERVK3-1 | 0.213944284 | 0.003436498 | 0.085194315 | Up | 2.463883932 |
| ZSCAN22 | 0.069499221 | 0.269759567 | 0.495193461 | NS | 0.569023143 |
| A1BG | 0.47366189 | 0.017514898 | NA | Up | 1.7565924 |
| ZNF497 | 0.509342905 | 0.019330563 | NA | Up | 1.713755508 |
| ZNF837 | 0.414786348 | 0.004052265 | 0.090453197 | Up | 2.392302128 |
| RPS5 | -0.289832098 | 0.033800965 | 0.20229026 | Down | 1.471070901 |
| RNF225 | 0.712215887 | 0.026651233 | NA | Up | 1.574282699 |
| ZNF584 | -0.179276488 | 0.026394169 | 0.183204185 | Down | 1.578492005 |
| ZNF132 | -0.142800181 | 0.102310661 | 0.31812203 | NS | 0.990079108 |
| ZNF324B | -0.017657694 | 0.784884082 | 0.875781498 | NS | 0.105194478 |
| ZNF324 | 0.031661947 | 0.608429774 | 0.759898494 | NS | 0.215789542 |
| ZNF446 | 0.114193764 | 0.143736116 | 0.368818142 | NS | 0.842434094 |
| SLC27A5 | 0.085127885 | 0.42777737 | 0.625790589 | NS | 0.368782194 |
| ZBTB45 | 0.14034589 | 0.078832111 | 0.283646978 | NS | 1.103296841 |
| TRIM28 | 0.119703166 | 0.135987131 | 0.360134579 | NS | 0.86650219 |
| CHMP2A | -0.029583479 | 0.705989046 | 0.826187668 | NS | 0.151202038 |
| UBE2M | -0.011938368 | 0.89949979 | 0.944103767 | NS | 0.045998933 |
| MZF1 | 0.099044502 | 0.244886972 | 0.472764177 | NS | 0.611034319 |
| DEFB125 | 0.570451576 | 0.352455539 | NA | NS | 0.45289566 |
| DEFB126 | 0.323517141 | 0.679482174 | NA | NS | 0.167821932 |
| DEFB127 | 0.697123672 | 0.422328339 | NA | NS | 0.374349776 |
| DEFB128 | 0.067163345 | 0.915465991 | NA | NS | 0.038357785 |
| DEFB129 | 0.192251164 | 0.749156191 | NA | NS | 0.125427627 |
| DEFB132 | 0.47999068 | 0.346679756 | NA | NS | 0.460071518 |
| C20orf96 | -0.035240636 | 0.857938852 | 0.919794085 | NS | 0.066543664 |
| ZCCHC3 | -0.067049864 | 0.283809272 | 0.506589562 | NS | 0.54697342 |
| SOX12 | 0.262333669 | 0.138662375 | 0.363055415 | NS | 0.858041365 |
| NRSN2 | 0.48847062 | 0.100718308 | NA | NS | 0.99689158 |
| TRIB3 | 0.063271371 | 0.53153196 | 0.704868508 | NS | 0.274470617 |
| RBCK1 | 0.008904499 | 0.914145187 | 0.952101191 | NS | 0.038984823 |
| TBC1D20 | 0.004161291 | 0.920996101 | 0.956452759 | NS | 0.035742208 |
| CSNK2A1 | -0.118220444 | 0.007513117 | 0.113019428 | Down | 2.124179826 |
| TCF15 | 1.137781506 | 0.038643962 | NA | Up | 1.412918349 |
| SRXN1 | 0.478944932 | 0.042314873 | 0.220851235 | Up | 1.37350696 |
| SCRT2 | 0.146574671 | 0.383791905 | 0.592650678 | NS | 0.41590419 |
| SLC52A3 | 0.590462335 | 0.136391173 | NA | NS | 0.865213735 |
| FAM110A | 0.343720391 | 0.01524657 | 0.147394235 | Up | 1.816827852 |
| ANGPT4 | 0.071375839 | 0.692047877 | 0.817141704 | NS | 0.159863859 |
| RSPO4 | 1.005180939 | 0.023862592 | NA | Up | 1.622282389 |
| PSMF1 | 0.152048274 | 0.398079128 | 0.603162963 | NS | 0.400030593 |
| TMEM74B | 0.407848505 | 0.072033006 | NA | NS | 1.14246846 |
| C20orf202 | 0.743426912 | 0.027039028 | NA | Up | 1.568008924 |
| RAD21L1 | 0.751051385 | 0.04830133 | NA | Up | 1.316040911 |
| SNPH | -0.055518931 | 0.543445414 | 0.713114152 | NS | 0.264844072 |
| SDCBP2 | 0.08158785 | 0.482305303 | 0.66853282 | NS | 0.316677963 |
| FKBP1A | 0.220475136 | 5.89E-04 | 0.04060164 | Up | 3.229914326 |
| NSFL1C | 0.002857717 | 0.969467217 | 0.982823994 | NS | 0.013466872 |
| SIRPB2 | -0.034445467 | 0.786807298 | 0.876987431 | NS | 0.10413162 |
| SIRPD | 0.352362452 | 0.002848828 | 0.080030557 | Up | 2.545333708 |
| SIRPB1 | 0.18442197 | 0.146586516 | 0.372104233 | NS | 0.833905977 |
| SIRPG | 0.231182782 | 0.043299133 | 0.223140581 | Up | 1.3635208 |
| SIRPA | 0.258053142 | 0.023407985 | 0.173829337 | Up | 1.630635968 |
| PDYN | 0.484948066 | 0.306866594 | NA | NS | 0.513050388 |
| STK35 | 0.175525776 | 0.009509735 | 0.122561596 | Up | 2.021831575 |
| TGM3 | 0.424895236 | 0.320158643 | NA | NS | 0.494634769 |
| TGM6 | 0.419184291 | 0.393337552 | NA | NS | 0.40523459 |
| SNRPB | -0.104459161 | 0.10596905 | 0.322255103 | NS | 0.974820959 |
| ZNF343 | -0.105510087 | 0.057463771 | 0.251193798 | NS | 1.24060588 |
| TMC2 | 0.239479641 | 0.167663398 | 0.395909177 | NS | 0.775561736 |
| NOP56 | -0.147562066 | 0.017143881 | 0.153970026 | Down | 1.76589086 |
| IDH3B | -0.234631268 | 0.002578521 | 0.077799913 | Down | 2.588629304 |
| EBF4 | 0.555435942 | 0.024594872 | 0.17769403 | Up | 1.609155427 |
| CPXM1 | 0.55442904 | 0.141542914 | NA | NS | 0.849111867 |
| C20orf141 | 1.029991555 | 0.18970203 | NA | NS | 0.721928022 |
| TMEM239 | 0.776091641 | 0.158084211 | NA | NS | 0.801111503 |
| PCED1A | 0.157201076 | 0.082081207 | 0.288607877 | NS | 1.085756267 |
| VPS16 | -0.121978622 | 0.021114209 | 0.168202484 | Down | 1.675425183 |
| PTPRA | -0.363340253 | 0.003255553 | 0.084906991 | Down | 2.48737519 |
| GNRH2 | 0.278951877 | 0.236826646 | NA | NS | 0.625569435 |
| MRPS26 | -0.052266282 | 0.542380713 | 0.712411281 | NS | 0.265695762 |
| OXT | 0.806021164 | 0.001116498 | NA | Up | 2.952142212 |
| AVP | 1.364110224 | 0.059064794 | NA | NS | 1.228671307 |
| UBOX5 | 0.181282393 | 0.024380833 | 0.176923269 | Up | 1.612951461 |
| FASTKD5 | -0.023723575 | 0.578202263 | 0.738275713 | NS | 0.237920213 |
| LZTS3 | 0.342351368 | 0.136915954 | 0.361416456 | NS | 0.863545945 |
| DDRGK1 | -0.197454806 | 0.004822429 | 0.096288809 | Down | 2.316734177 |
| ITPA | -0.207970386 | 0.002063071 | 0.071264622 | Down | 2.685485897 |
| SLC4A11 | 0.857489076 | 0.019358669 | 0.161396883 | Up | 1.713124508 |
| DNAAF9 | 0.146207435 | 0.052379828 | 0.241847979 | NS | 1.28083593 |
| ATRN | -0.075179055 | 0.200274863 | 0.429960996 | NS | 0.698373557 |
| GFRA4 | 0.649302169 | 0.284146716 | NA | NS | 0.546457359 |
| ADAM33 | 0.621679574 | 0.150247905 | 0.376545633 | NS | 0.823191574 |
| SIGLEC1 | 1.0806218 | 8.97E-06 | 0.002832126 | Up | 5.047335653 |
| HSPA12B | 0.478861066 | 0.087013452 | 0.29656937 | NS | 1.060413602 |
| C20orf27 | 0.004014987 | 0.971635492 | 0.983723402 | NS | 0.01249663 |
| SPEF1 | 1.140751773 | 0.003493905 | NA | Up | 2.456688955 |
| CENPB | -0.171667625 | 0.072561534 | 0.274170249 | NS | 1.139293547 |
| CDC25B | -0.088530981 | 0.447320829 | 0.641295423 | NS | 0.349380879 |
| AP5S1 | 0.043519696 | 0.540344501 | 0.710599212 | NS | 0.267329264 |
| MAVS | 0.009288536 | 0.916293646 | 0.953437582 | NS | 0.037965325 |
| PANK2 | -0.015243902 | 0.75078327 | 0.853703314 | NS | 0.124485413 |
| RNF24 | 0.381680337 | 0.005824089 | 0.102762132 | Up | 2.234772024 |
| SMOX | -0.297311374 | 0.12203552 | 0.342843165 | NS | 0.913513743 |
| ADRA1D | 1.196960429 | 0.016365823 | NA | Up | 1.786062147 |
| PRNP | -0.281771774 | 0.004371321 | 0.093367359 | Down | 2.359387314 |
| PRND | 0.264826643 | 0.549768447 | NA | NS | 0.25982019 |
| RASSF2 | 0.065221931 | 0.49905012 | 0.680757987 | NS | 0.301855836 |
| SLC23A2 | 0.042243773 | 0.520908647 | 0.696755627 | NS | 0.283238433 |
| TMEM230 | -0.199013672 | 0.007484341 | 0.112915927 | Down | 2.125846436 |
| PCNA | -0.175767377 | 0.110997147 | 0.329580794 | NS | 0.954688183 |
| CDS2 | 0.116568985 | 0.062026196 | 0.258700409 | NS | 1.207424852 |
| PROKR2 | 0.068368754 | 0.832071069 | 0.904614627 | NS | 0.079839578 |
| GPCPD1 | -0.054190963 | 0.505379228 | 0.685915662 | NS | 0.296382612 |
| SHLD1 | -0.066788027 | 0.549332037 | 0.717222639 | NS | 0.260165072 |
| CHGB | 0.354492946 | 0.390592054 | 0.59752734 | NS | 0.408276596 |
| TRMT6 | -0.137089888 | 0.074340726 | 0.276837611 | NS | 1.128773202 |
| MCM8 | -0.159617388 | 0.011087589 | 0.129489019 | Down | 1.955162875 |
| CRLS1 | 0.134890972 | 0.094582176 | 0.307721221 | NS | 1.024190699 |
| LRRN4 | 0.339061349 | 0.051018237 | 0.238791409 | NS | 1.292274551 |
| FERMT1 | 0.413212752 | 0.257336758 | 0.484204915 | NS | 0.589498175 |
| BMP2 | 0.357804683 | 0.204336442 | 0.43446401 | NS | 0.689654172 |
| HAO1 | 0.484337882 | 0.426192954 | NA | NS | 0.370393734 |
| TMX4 | -0.038126402 | 0.679295567 | 0.808797872 | NS | 0.16794122 |
| PLCB1 | 0.034369033 | 0.660028746 | 0.795745333 | NS | 0.180437149 |
| PLCB4 | 0.363206662 | 0.22846401 | 0.457089362 | NS | 0.641182204 |
| LAMP5 | 0.027136427 | 0.870802852 | 0.927057761 | NS | 0.060080157 |
| PAK5 | 0.756611318 | 0.085694737 | NA | NS | 1.067045851 |
| ANKEF1 | -0.225075507 | 0.008859137 | 0.119278041 | Down | 2.052608598 |
| SNAP25 | 0.261409724 | 0.470520385 | 0.659517587 | NS | 0.327421556 |
| MKKS | 0.022508594 | 0.76340381 | 0.862228238 | NS | 0.117245677 |
| SLX4IP | 0.120788223 | 0.037223015 | 0.209820891 | Up | 1.429188451 |
| JAG1 | 0.157944803 | 0.056086595 | 0.249087638 | NS | 1.251140924 |
| BTBD3 | 0.033389959 | 0.656132746 | 0.793470913 | NS | 0.183008287 |
| SPTLC3 | 0.013602367 | 0.950499675 | 0.973278349 | NS | 0.022048027 |
| ISM1 | -0.302748047 | 0.068784368 | 0.268822456 | NS | 1.16251025 |
| TASP1 | -0.071587561 | 0.190273745 | 0.419771632 | NS | 0.720621134 |
| ESF1 | -0.194837931 | 0.0337845 | 0.20229026 | Down | 1.47128251 |
| NDUFAF5 | 0.013593451 | 0.809667096 | 0.89059103 | NS | 0.091693509 |
| SEL1L2 | 0.727166358 | 0.111987755 | 0.330763937 | NS | 0.950829461 |
| MACROD2 | -0.095197321 | 0.564238252 | 0.728337377 | NS | 0.248537475 |
| FLRT3 | 0.923557836 | 0.021090704 | NA | Up | 1.675908918 |
| KIF16B | -0.070948192 | 0.39514617 | 0.600626375 | NS | 0.403242223 |
| SNRPB2 | -0.202933776 | 0.060801161 | 0.256770828 | NS | 1.216088125 |
| OTOR | 0.165714921 | 0.724554978 | NA | NS | 0.139928655 |
| PCSK2 | 0.632031948 | 0.11523103 | 0.334095589 | NS | 0.938430555 |
| BFSP1 | 0.091850302 | 0.572137909 | 0.733952836 | NS | 0.242499276 |
| DSTN | -0.013985054 | 0.841164959 | 0.910500042 | NS | 0.075118827 |
| RRBP1 | -0.156040328 | 0.122385041 | 0.343177027 | NS | 0.912271662 |
| BANF2 | 0.53465176 | 0.377679921 | NA | NS | 0.422876104 |
| SNX5 | -0.051624899 | 0.242085745 | 0.469918053 | NS | 0.616030783 |
| OVOL2 | 0.891136681 | 0.069795858 | NA | NS | 1.15617035 |
| MGME1 | 0.008889368 | 0.867281163 | 0.92508375 | NS | 0.061840086 |
| KAT14 | -0.16663677 | 0.020565237 | 0.166586724 | Down | 1.686866271 |
| PET117 | -0.169732754 | 0.293306515 | 0.515386052 | NS | 0.532678291 |
| ZNF133 | -0.12764178 | 0.009546792 | 0.122752564 | Down | 2.02014256 |
| DZANK1 | 0.115653132 | 0.285344901 | 0.508518319 | NS | 0.544629884 |
| POLR3F | -0.110593418 | 0.120493401 | 0.341231589 | NS | 0.919036738 |
| RBBP9 | -0.170104765 | 0.025369992 | 0.180162342 | Down | 1.595679673 |
| SEC23B | -0.191145622 | 0.007478217 | 0.112915927 | Down | 2.126201958 |
| SMIM26 | -0.3549604 | 0.011167652 | 0.129586317 | Down | 1.95203812 |
| DTD1 | -0.076091778 | 0.217178849 | 0.446804707 | NS | 0.663182473 |
| SLC24A3 | -0.044357807 | 0.749264884 | 0.85265413 | NS | 0.125364621 |
| RIN2 | -0.025317937 | 0.789866035 | 0.878600803 | NS | 0.102446561 |
| NAA20 | -0.042063389 | 0.592790089 | 0.749346361 | NS | 0.227099066 |
| CRNKL1 | -0.067321337 | 0.227827513 | 0.456610283 | NS | 0.64239383 |
| CFAP61 | 0.537552587 | 0.133902344 | 0.35729133 | NS | 0.87321182 |
| INSM1 | 1.161379285 | 0.03466831 | NA | Up | 1.460067332 |
| RALGAPA2 | 0.209941613 | 0.033983929 | 0.202672427 | Up | 1.468726413 |
| KIZ | 0.053955627 | 0.248857736 | 0.476376043 | NS | 0.604048854 |
| XRN2 | -0.044870123 | 0.454083637 | 0.646489087 | NS | 0.342864148 |
| NKX2-4 | 0.808625789 | 0.111976612 | NA | NS | 0.950872678 |
| NKX2-2 | 1.565157508 | 0.001341999 | NA | Up | 2.872247911 |
| PAX1 | 0.939613249 | 0.038783517 | 0.213874334 | Up | 1.411352809 |
| FOXA2 | 0.763582723 | 0.108756353 | NA | NS | 0.963545364 |
| SSTR4 | 0.682289241 | 0.053764832 | NA | NS | 1.269501708 |
| THBD | -0.017295487 | 0.901495796 | 0.945288985 | NS | 0.045036294 |
| CD93 | -0.072157601 | 0.519976491 | 0.696688569 | NS | 0.284016291 |
| NXT1 | -0.138218567 | 0.17028717 | 0.398682965 | NS | 0.768818073 |
| GZF1 | -0.085594964 | 0.070652879 | 0.271537547 | NS | 1.150870135 |
| NAPB | 0.031310859 | 0.528031213 | 0.702828416 | NS | 0.277340405 |
| CSTL1 | 0.856665042 | 0.102812945 | NA | NS | 0.987952202 |
| CST8 | 1.359334285 | 0.011235641 | NA | Up | 1.94940214 |
| CST9 | 0.819791558 | 0.136912232 | NA | NS | 0.863557749 |
| CST3 | 0.128970959 | 0.269128863 | 0.49444608 | NS | 0.570039723 |
| CST4 | 0.618214155 | 0.265672979 | NA | NS | 0.575652614 |
| CST1 | 0.844899323 | 0.11770124 | NA | NS | 0.929218963 |
| CST2 | 0.874727391 | 0.130554019 | NA | NS | 0.884209753 |
| CST5 | 1.121169579 | 0.07567569 | NA | NS | 1.12104361 |
| GGTLC1 | 1.078291316 | 0.046381689 | NA | Up | 1.333653443 |
| SYNDIG1 | 0.580782404 | 0.195001366 | NA | NS | 0.709962347 |
| CST7 | 0.429133528 | 3.56E-04 | 0.032222893 | Up | 3.448039338 |
| APMAP | -0.022148385 | 0.830845771 | 0.903772673 | NS | 0.080479586 |
| ACSS1 | -0.005645958 | 0.922297794 | 0.957283666 | NS | 0.03512883 |
| VSX1 | 0.380226038 | 0.349748086 | NA | NS | 0.456244654 |
| ENTPD6 | 0.007264469 | 0.917507495 | 0.954431171 | NS | 0.037390379 |
| PYGB | -0.150384529 | 0.059817612 | 0.255146205 | NS | 1.223170926 |
| ABHD12 | 0.132656358 | 0.06851659 | 0.268602708 | NS | 1.164204262 |
| GINS1 | 0.090219166 | 0.605785699 | 0.758254148 | NS | 0.217680984 |
| NINL | 0.354731838 | 0.023542994 | 0.17423553 | Up | 1.628138312 |
| NANP | -0.129628529 | 0.147446318 | 0.373211269 | NS | 0.831366069 |
| ZNF337 | 0.16125888 | 0.022724929 | 0.171934696 | Up | 1.643497465 |
| DEFB115 | 0.731513594 | 0.295994313 | NA | NS | 0.528716634 |
| DEFB116 | 0.809017445 | 0.283034911 | NA | NS | 0.548159992 |
| DEFB118 | 0.47590899 | 0.390798146 | NA | NS | 0.408047506 |
| DEFB119 | 1.073358868 | 0.068157535 | NA | NS | 1.166486124 |
| DEFB121 | 0.904818243 | 0.128848468 | NA | NS | 0.889920739 |
| DEFB123 | 0.223538185 | 0.876274635 | NA | NS | 0.057359759 |
| DEFB124 | 0.804873054 | 0.156283567 | NA | NS | 0.806086684 |
| REM1 | 1.018017801 | 0.033509193 | NA | Up | 1.474836031 |
| HM13 | 0.215311854 | 0.00530887 | 0.099529109 | Up | 2.274997869 |
| ID1 | 0.282142971 | 0.451195345 | NA | NS | 0.345635389 |
| COX4I2 | -0.052012568 | 0.920630599 | NA | NS | 0.035914594 |
| BCL2L1 | -0.320433374 | 0.169119886 | 0.397331107 | NS | 0.771805323 |
| TPX2 | -0.242598358 | 0.147042332 | 0.37264175 | NS | 0.832557617 |
| MYLK2 | 0.682289598 | 0.097521405 | NA | NS | 1.010900052 |
| FOXS1 | 1.048428297 | 0.058048461 | NA | NS | 1.236209291 |
| DUSP15 | 0.699325364 | 0.154914122 | NA | NS | 0.809908991 |
| TTLL9 | 0.224423005 | 0.257715585 | 0.484700856 | NS | 0.588859318 |
| PDRG1 | -0.268770493 | 0.001035309 | 0.052796552 | Down | 2.984930161 |
| XKR7 | 0.434570719 | 0.17065035 | 0.399000229 | NS | 0.767892817 |
| CCM2L | 0.566421608 | 0.154998168 | NA | NS | 0.809673434 |
| HCK | -0.054126801 | 0.58385052 | 0.742976308 | NS | 0.233698329 |
| TM9SF4 | -0.048484665 | 0.526109131 | 0.701218204 | NS | 0.27892416 |
| PLAGL2 | 0.1824384 | 0.08986245 | 0.30028957 | NS | 1.046421747 |
| POFUT1 | 0.007004818 | 0.904601425 | 0.947195487 | NS | 0.043542732 |
| KIF3B | -0.139893149 | 0.00291254 | 0.080729745 | Down | 2.535728154 |
| ASXL1 | 0.017420297 | 0.796865178 | 0.883073092 | NS | 0.098615151 |
| NOL4L | 0.035260912 | 0.658505809 | 0.794578505 | NS | 0.18144039 |
| C20orf203 | 0.789698255 | 0.069760782 | NA | NS | 1.156388661 |
| COMMD7 | -0.047164998 | 0.558182362 | 0.723836631 | NS | 0.253223891 |
| DNMT3B | 0.224364798 | 0.291846375 | 0.514593347 | NS | 0.534845697 |
| MAPRE1 | -0.046435717 | 0.416738325 | 0.617816026 | NS | 0.380136558 |
| EFCAB8 | 0.179498604 | 0.276354489 | 0.50091575 | NS | 0.558533477 |
| SUN5 | 0.899019293 | 0.06251832 | NA | NS | 1.203992704 |
| BPIFB2 | 0.839125833 | 0.059707383 | NA | NS | 1.223971961 |
| BPIFB6 | 0.576755989 | 0.240464475 | NA | NS | 0.618949076 |
| BPIFB3 | 0.336171217 | 0.414149181 | NA | NS | 0.382843193 |
| BPIFB4 | 0.67190287 | 0.189719426 | NA | NS | 0.721888198 |
| BPIFA2 | 0.387960182 | 0.51394699 | NA | NS | 0.289081673 |
| BPIFA3 | 0.311284556 | 0.542050133 | NA | NS | 0.265960545 |
| BPIFA1 | 0.681506039 | 0.250537602 | NA | NS | 0.601127084 |
| BPIFB1 | 0.709649245 | 0.148423052 | NA | NS | 0.828498643 |
| CDK5RAP1 | -0.145127951 | 0.003232373 | 0.084506638 | Down | 2.490478479 |
| SNTA1 | 0.29630005 | 0.011436455 | 0.131302419 | Up | 1.941708584 |
| CBFA2T2 | 0.077762848 | 0.340713424 | 0.556206346 | NS | 0.467610755 |
| NECAB3 | 0.146761579 | 0.07029005 | 0.271077991 | NS | 1.153106149 |
| C20orf144 | 0.260029281 | 0.11145633 | 0.329942771 | NS | 0.952895262 |
| ACTL10 | 0.211302621 | 0.032018751 | 0.198142899 | Up | 1.494595613 |
| E2F1 | 0.198026411 | 0.144541199 | 0.369690386 | NS | 0.840008346 |
| PXMP4 | -0.087342204 | 0.096478563 | 0.310542373 | NS | 1.015569173 |
| ZNF341 | 0.214745129 | 0.090978448 | 0.30205689 | NS | 1.041061474 |
| CHMP4B | -0.159102503 | 0.130255121 | 0.352156503 | NS | 0.885205194 |
| RALY | 0.034243482 | 0.448652462 | 0.642270202 | NS | 0.348089945 |
| EIF2S2 | -0.239684379 | 0.001718924 | 0.065336585 | Down | 2.764743431 |
| ASIP | 0.558352231 | 0.139811234 | NA | NS | 0.854457932 |
| AHCY | 0.161965493 | 0.048868934 | 0.234288536 | Up | 1.310967131 |
| ITCH | -0.128222701 | 0.005090894 | 0.09830662 | Down | 2.293205959 |
| DYNLRB1 | 0.143647316 | 0.072895384 | 0.274846078 | NS | 1.137299974 |
| MAP1LC3A | 0.101831985 | 0.399790011 | 0.604874866 | NS | 0.398168061 |
| PIGU | -0.072897524 | 0.204189468 | 0.434281871 | NS | 0.689966663 |
| NCOA6 | 0.125615857 | 0.181065625 | 0.411018431 | NS | 0.742163991 |
| TP53INP2 | 0.066362369 | 0.592351689 | 0.749026006 | NS | 0.227420369 |
| GGT7 | 0.126442067 | 0.174162455 | 0.403422181 | NS | 0.759045462 |
| ACSS2 | 0.038250006 | 0.522209288 | 0.697843221 | NS | 0.282155408 |
| GSS | -0.077607738 | 0.228597495 | 0.457186791 | NS | 0.640928532 |
| MYH7B | 0.098313104 | 0.597597189 | 0.752400814 | NS | 0.223591454 |
| TRPC4AP | -0.030421106 | 0.4863893 | 0.671762851 | NS | 0.313015987 |
| EDEM2 | -0.083497147 | 0.1919384 | 0.420934764 | NS | 0.716838129 |
| PROCR | -0.16059272 | 0.201001816 | 0.430601586 | NS | 0.696800019 |
| MMP24OS | 0.00827185 | 0.932353307 | 0.96283336 | NS | 0.030419484 |
| MMP24 | 0.102331452 | 0.41280362 | 0.614932835 | NS | 0.384256503 |
| EIF6 | -0.189400127 | 0.043614243 | 0.224086881 | Down | 1.360371663 |
| FAM83C | 0.762136698 | 0.121650623 | NA | NS | 0.914885664 |
| UQCC1 | 0.035365275 | 0.490427221 | 0.67478307 | NS | 0.309425433 |
| GDF5 | 0.646027498 | 0.156494344 | NA | NS | 0.805501354 |
| CEP250 | -0.086287076 | 0.114643798 | 0.33336367 | NS | 0.940649435 |
| C20orf173 | 0.346735387 | 0.320701301 | NA | NS | 0.493899278 |
| ERGIC3 | 0.009550482 | 0.89778919 | 0.943043068 | NS | 0.046825628 |
| SPAG4 | 0.344086597 | 0.036275015 | 0.208236145 | Up | 1.440392394 |
| CPNE1 | -0.109169393 | 0.081904677 | 0.288475384 | NS | 1.086691296 |
| RBM12 | 0.038588342 | 0.423615838 | 0.622710628 | NS | 0.373027811 |
| NFS1 | -0.060589195 | 0.220816768 | 0.449641695 | NS | 0.655967952 |
| ROMO1 | -0.226234812 | 0.081435383 | 0.287767953 | NS | 1.089186855 |
| RBM39 | -0.125723469 | 0.006654212 | 0.108543277 | Down | 2.176903372 |
| PHF20 | -0.073361181 | 0.048964897 | 0.234552202 | NS | 1.310115153 |
| SCAND1 | 0.115832077 | 0.327791123 | 0.545611767 | NS | 0.484402811 |
| CNBD2 | 0.604804253 | 0.066633895 | 0.266279415 | NS | 1.176304803 |
| EPB41L1 | 0.601527704 | 0.138190188 | 0.362884344 | NS | 0.859522791 |
| AAR2 | 0.056139654 | 0.247853932 | 0.475964343 | NS | 0.605804187 |
| DLGAP4 | 0.185104264 | 0.289576515 | 0.512711385 | NS | 0.538236663 |
| MYL9 | 0.149068775 | 0.540075941 | 0.710424351 | NS | 0.267545169 |
| TGIF2 | -0.042461251 | 0.38334603 | 0.592439708 | NS | 0.416409029 |
| RAB5IF | 0.396678856 | 2.46E-04 | 0.026225737 | Up | 3.608707243 |
| SLA2 | 0.003296117 | 0.957988441 | 0.976710648 | NS | 0.018639731 |
| NDRG3 | -0.111997189 | 0.07002003 | 0.270674772 | NS | 1.154777707 |
| DSN1 | -0.175890819 | 0.006264091 | 0.105952889 | Down | 2.203141945 |
| SOGA1 | 0.009434105 | 0.955446541 | 0.975856785 | NS | 0.019793608 |
| TLDC2 | 0.118657361 | 0.356167422 | 0.569181934 | NS | 0.448345807 |
| SAMHD1 | -0.016192872 | 0.807585036 | 0.889154969 | NS | 0.092811737 |
| RBL1 | -0.238834104 | 0.037152701 | 0.209820891 | Down | 1.430009612 |
| MROH8 | 0.230165811 | 0.124186801 | 0.345580329 | NS | 0.905924561 |
| RPN2 | -0.206803067 | 0.008425027 | 0.117652418 | Down | 2.074428704 |
| GHRH | 1.359310469 | 0.011639022 | NA | Up | 1.934083508 |
| MANBAL | 0.162708392 | 0.007534686 | 0.11304493 | Up | 2.122934835 |
| SRC | 0.209356302 | 0.217989553 | 0.44751176 | NS | 0.661564319 |
| BLCAP | 0.05515058 | 0.466274148 | 0.656254399 | NS | 0.331358662 |
| NNAT | 0.161123759 | 0.099115178 | 0.314049068 | NS | 1.003859835 |
| CTNNBL1 | -0.071844582 | 0.115008509 | 0.333661624 | NS | 0.939270028 |
| VSTM2L | 0.432487977 | 0.31924438 | NA | NS | 0.49587674 |
| TTI1 | 0.142220255 | 0.001724009 | 0.065336585 | Up | 2.763460528 |
| RPRD1B | 0.132795212 | 0.084247552 | 0.292034469 | NS | 1.07444271 |
| TGM2 | -0.149494388 | 0.478255797 | 0.665917695 | NS | 0.320339758 |
| KIAA1755 | 0.706101646 | 0.115714662 | 0.334819606 | NS | 0.93661161 |
| BPI | 0.28497291 | 0.156940722 | 0.384159619 | NS | 0.804264353 |
| LBP | 0.475797395 | 0.291081632 | NA | NS | 0.535985199 |
| RALGAPB | 0.064323527 | 0.181417176 | 0.411367937 | NS | 0.741321598 |
| ADIG | 0.923547072 | 0.059056658 | NA | NS | 1.228731131 |
| ARHGAP40 | 1.121611926 | 0.010058874 | NA | Up | 1.997450643 |
| SLC32A1 | 1.11740762 | 0.030849946 | NA | Up | 1.510745592 |
| ACTR5 | -0.179706975 | 4.99E-04 | 0.038126146 | Down | 3.301666498 |
| PPP1R16B | 0.05984799 | 0.19948637 | 0.429060559 | NS | 0.700086773 |
| FAM83D | -0.028872473 | 0.837898534 | 0.908811377 | NS | 0.076808569 |
| DHX35 | -0.118531279 | 0.005712055 | 0.102180893 | Down | 2.243207638 |
| MAFB | 0.174294627 | 0.120380961 | 0.341081873 | NS | 0.919442193 |
| TOP1 | -0.054740718 | 0.672777286 | 0.805012822 | NS | 0.172128679 |
| PLCG1 | -0.190421043 | 0.03029552 | 0.193469884 | Down | 1.518621592 |
| ZHX3 | 0.093866079 | 0.229093324 | 0.457706996 | NS | 0.639987567 |
| LPIN3 | 0.628647045 | 0.108022194 | NA | NS | 0.966487005 |
| EMILIN3 | 0.728349155 | 0.119232151 | NA | NS | 0.92360662 |
| CHD6 | -0.059834511 | 0.241787505 | 0.469750377 | NS | 0.616566146 |
| PTPRT | 0.440436465 | 0.242875197 | 0.470546296 | NS | 0.614616834 |
| SRSF6 | -0.089361209 | 0.130330305 | 0.35223326 | NS | 0.884954588 |
| L3MBTL1 | 0.135516759 | 0.165238558 | 0.393073717 | NS | 0.781888603 |
| SGK2 | 0.44182368 | 0.096983811 | 0.311212448 | NS | 1.013300754 |
| IFT52 | -0.270771023 | 0.007126384 | 0.111026155 | Down | 2.147130775 |
| MYBL2 | -0.083059985 | 0.623029788 | 0.769862318 | NS | 0.205491189 |
| GTSF1L | 0.511271336 | 0.053801804 | NA | NS | 1.269203163 |
| TOX2 | 0.307369524 | 0.122091439 | 0.342932892 | NS | 0.913314789 |
| JPH2 | 0.810451215 | 0.047800729 | 0.232279289 | Up | 1.320565483 |
| OSER1 | -0.016261373 | 0.797944523 | 0.88374371 | NS | 0.098027302 |
| GDAP1L1 | 0.362895498 | 0.122953558 | 0.343735642 | NS | 0.9102589 |
| FITM2 | 0.110398012 | 0.120730869 | 0.341313216 | NS | 0.918181673 |
| R3HDML | 0.954151248 | 0.117680444 | NA | NS | 0.929295702 |
| HNF4A | 0.309546753 | 0.451469977 | 0.644308496 | NS | 0.345371125 |
| TTPAL | -0.025956171 | 0.700664718 | 0.822549366 | NS | 0.154489751 |
| SERINC3 | -0.087069944 | 0.126557768 | 0.348577992 | NS | 0.897711193 |
| PKIG | -0.295149049 | 0.015738638 | 0.148841337 | Down | 1.803032859 |
| ADA | -0.181976186 | 0.024326909 | 0.176755986 | Down | 1.613913071 |
| CCN5 | 0.583328967 | 0.132699003 | NA | NS | 0.877132339 |
| KCNK15 | 0.458830195 | 0.361147713 | NA | NS | 0.442315131 |
| RIMS4 | 0.748298272 | 0.130497257 | NA | NS | 0.884398616 |
| YWHAB | -0.048618476 | 0.417620496 | 0.61865101 | NS | 0.379218195 |
| PABPC1L | 0.118972379 | 0.381047885 | 0.590883517 | NS | 0.419020445 |
| TOMM34 | 0.052451862 | 0.503641098 | 0.684533438 | NS | 0.297878838 |
| STK4 | 0.01037963 | 0.841213643 | 0.910509705 | NS | 0.075093692 |
| KCNS1 | 0.171062624 | 0.090376891 | 0.301065195 | NS | 1.043942602 |
| WFDC5 | 0.071321089 | 0.811080621 | NA | NS | 0.090935975 |
| WFDC12 | 0.125871206 | 0.717567362 | NA | NS | 0.144137323 |
| PI3 | 0.219842191 | 0.342118116 | 0.557387002 | NS | 0.465823928 |
| SEMG1 | 0.190044156 | 0.394512717 | 0.600265002 | NS | 0.403938993 |
| SEMG2 | 0.748289715 | 0.199290493 | NA | NS | 0.700513418 |
| SLPI | 0.445122011 | 0.038346197 | 0.213464789 | Up | 1.416277705 |
| MATN4 | 0.723057005 | 0.129730708 | NA | NS | 0.88695721 |
| RBPJL | 0.685402124 | 0.119590197 | 0.34007656 | NS | 0.922304418 |
| SDC4 | 0.073719616 | 0.572495588 | 0.734073901 | NS | 0.242227856 |
| SYS1 | 0.135497619 | 0.005114663 | 0.09835814 | Up | 2.291182993 |
| SYS1-DBNDD2 | -0.151153147 | 0.611053386 | NA | NS | 0.213920845 |
| TP53TG5 | 0.257085505 | 0.00982784 | 0.124834823 | Up | 2.007541934 |
| DBNDD2 | 0.426893797 | 0.019074627 | 0.160487545 | Up | 1.719543955 |
| PIGT | -0.030055308 | 0.570946731 | 0.732990752 | NS | 0.24340441 |
| WFDC2 | 0.671781765 | 0.203840949 | NA | NS | 0.690708567 |
| SPINT3 | 0.978348007 | 0.222725759 | NA | NS | 0.652229552 |
| WFDC6 | 0.750879577 | 0.289614014 | NA | NS | 0.538180426 |
| EPPIN-WFDC6 | 0.638822339 | 0.346166467 | NA | NS | 0.460715004 |
| EPPIN | 0.511535683 | 0.234755962 | 0.462791997 | NS | 0.62938337 |
| WFDC8 | 0.883072246 | 0.107814977 | NA | NS | 0.967320903 |
| WFDC9 | 0.403980756 | 0.456409191 | NA | NS | 0.340645618 |
| WFDC10A | 1.056682785 | 0.095678097 | NA | NS | 1.01918747 |
| WFDC11 | 0.593770849 | 0.27982317 | NA | NS | 0.553116327 |
| WFDC10B | 0.130763538 | 0.814633292 | NA | NS | 0.089037845 |
| WFDC13 | 0.710687787 | 0.132575733 | NA | NS | 0.877535962 |
| SPINT4 | -0.009259617 | 0.988196649 | NA | NS | 0.005156623 |
| WFDC3 | 0.335072509 | 0.346124605 | NA | NS | 0.460767527 |
| DNTTIP1 | 0.160861044 | 0.030215013 | 0.193469884 | Up | 1.519777211 |
| UBE2C | 0.305874452 | 0.054590036 | 0.24676996 | NS | 1.262886621 |
| TNNC2 | 0.04542565 | 0.849733492 | 0.915577343 | NS | 0.070717264 |
| SNX21 | 0.061600333 | 0.420437503 | 0.620581631 | NS | 0.376298552 |
| ACOT8 | 0.004333384 | 0.92589691 | 0.959365989 | NS | 0.033437365 |
| ZSWIM3 | 0.165711462 | 0.015109729 | 0.147189717 | Up | 1.820743311 |
| ZSWIM1 | 0.175431972 | 0.035098473 | 0.205352924 | Up | 1.45471178 |
| SPATA25 | 0.046716321 | 0.771864966 | 0.866999433 | NS | 0.112458671 |
| NEURL2 | -0.219969309 | 0.289759242 | NA | NS | 0.537962703 |
| CTSA | 0.202612764 | 0.026849838 | 0.185036235 | Up | 1.571058331 |
| PLTP | 0.36688336 | 0.056351745 | 0.249336874 | NS | 1.249092633 |
| PCIF1 | 0.012541656 | 0.87122238 | 0.927222822 | NS | 0.059870977 |
| ZNF335 | 0.095335066 | 0.27175074 | 0.497095483 | NS | 0.565829265 |
| MMP9 | 0.134299013 | 0.537030616 | 0.708735809 | NS | 0.270000955 |
| SLC12A5 | 0.252964149 | 0.218116297 | 0.447660331 | NS | 0.661311885 |
| NCOA5 | 0.143382188 | 0.045423715 | 0.227515368 | Up | 1.342717354 |
| CD40 | -0.100351894 | 0.270658645 | 0.495997715 | NS | 0.567578097 |
| CDH22 | 0.830757223 | 0.072358131 | 0.27394225 | NS | 1.140512659 |
| SLC35C2 | -0.090473874 | 0.090339807 | 0.301065195 | NS | 1.044120844 |
| ELMO2 | -0.027709641 | 0.624035651 | 0.770689413 | NS | 0.204790599 |
| ZNF334 | 0.295422748 | 0.252757626 | 0.479630056 | NS | 0.597295733 |
| OCSTAMP | 1.060355609 | 0.092618844 | NA | NS | 1.033300642 |
| SLC13A3 | 0.221261827 | 0.377736767 | 0.587778735 | NS | 0.422810742 |
| TP53RK | -0.11551988 | 0.162274466 | 0.389928636 | NS | 0.78974981 |
| SLC2A10 | 0.522957936 | 0.155908363 | 0.383185333 | NS | 0.807130589 |
| EYA2 | 0.602132633 | 0.131799597 | 0.354447438 | NS | 0.880085916 |
| ZMYND8 | -0.092736823 | 0.345579119 | 0.560513667 | NS | 0.461452507 |
| NCOA3 | 0.040354124 | 0.375904015 | 0.586177342 | NS | 0.424923035 |
| SULF2 | -0.087730591 | 0.356320446 | 0.569347021 | NS | 0.448159257 |
| PREX1 | -0.11720431 | 0.281587854 | 0.504671794 | NS | 0.550386083 |
| ARFGEF2 | -0.119689712 | 0.011389268 | 0.131149124 | Down | 1.943504195 |
| CSE1L | -0.274916431 | 4.10E-04 | 0.034543974 | Down | 3.387198337 |
| STAU1 | -0.063581906 | 0.646855377 | 0.786738282 | NS | 0.189192807 |
| DDX27 | -0.242578832 | 2.32E-05 | 0.005830479 | Down | 4.635127755 |
| ZNFX1 | 0.025557173 | 0.721001986 | 0.835351216 | NS | 0.142063539 |
| KCNB1 | 0.32676609 | 0.070573058 | 0.271500817 | NS | 1.151361063 |
| PTGIS | 0.229113721 | 0.627945655 | NA | NS | 0.20207794 |
| B4GALT5 | 0.095554986 | 0.386110289 | 0.594227394 | NS | 0.413288625 |
| SLC9A8 | 0.202337256 | 0.036771591 | 0.209023715 | Up | 1.43448758 |
| SPATA2 | 0.004863932 | 0.969853528 | 0.98307451 | NS | 0.01329385 |
| RNF114 | -0.120203399 | 0.048229532 | 0.233126372 | Down | 1.316686948 |
| SNAI1 | 1.047426943 | 0.030531758 | NA | Up | 1.515248193 |
| UBE2V1 | 0.173717705 | 0.053617052 | 0.244657844 | NS | 1.27069707 |
| PEDS1 | 0.073370975 | 0.286070358 | 0.509186875 | NS | 0.543527141 |
| CEBPB | 0.12363241 | 0.18550604 | 0.415463304 | NS | 0.731641946 |
| PTPN1 | 0.024426851 | 0.528538567 | 0.703278992 | NS | 0.276923317 |
| RIPOR3 | 0.121297078 | 0.311278566 | 0.530987325 | NS | 0.506850782 |
| PARD6B | -0.074085105 | 0.291673018 | 0.514510934 | NS | 0.535103744 |
| BCAS4 | 0.156584432 | 0.23838718 | 0.466504755 | NS | 0.622717104 |
| ADNP | 0.030926531 | 0.575048587 | 0.736193345 | NS | 0.240295459 |
| DPM1 | -0.027775646 | 0.68899194 | 0.815257617 | NS | 0.161785859 |
| MOCS3 | -0.031628898 | 0.658396113 | 0.794555206 | NS | 0.181512742 |
| KCNG1 | 0.663952515 | 1.06E-04 | 0.016409885 | Up | 3.974506167 |
| NFATC2 | 0.115008296 | 0.151311243 | 0.377750911 | NS | 0.820128801 |
| ATP9A | 0.368291103 | 0.001097142 | 0.05356245 | Up | 2.959737249 |
| SALL4 | 0.814801612 | 0.006473115 | 0.1080547 | Up | 2.188886667 |
| ZFP64 | 0.236237785 | 0.001034112 | 0.052796552 | Up | 2.985432406 |
| TSHZ2 | 0.132411185 | 0.252703862 | 0.479607512 | NS | 0.597388121 |
| ZNF217 | -7.01E-04 | 0.990053174 | 0.994124998 | NS | 0.00434148 |
| BCAS1 | 0.137519307 | 0.455009275 | 0.647163236 | NS | 0.34197975 |
| CYP24A1 | 0.365821132 | 0.105583886 | 0.321707479 | NS | 0.976402358 |
| PFDN4 | -0.493796501 | 0.019896918 | 0.163677883 | Down | 1.701214181 |
| DOK5 | 0.123203643 | 0.782186476 | NA | NS | 0.106689697 |
| CBLN4 | 0.810968423 | 0.085976068 | NA | NS | 1.065622421 |
| MC3R | 1.164252972 | 0.112547465 | NA | NS | 0.948664283 |
| FAM210B | -0.038368757 | 0.842826959 | 0.911738842 | NS | 0.074261581 |
| AURKA | -0.580474145 | 4.04E-04 | 0.034514738 | Down | 3.393792368 |
| CSTF1 | -0.081153255 | 0.090963305 | 0.30205689 | NS | 1.04113377 |
| CASS4 | 0.042657746 | 0.66692566 | 0.800814316 | NS | 0.175922573 |
| RTF2 | -0.125719183 | 0.091297501 | 0.302721204 | NS | 1.039541112 |
| GCNT7 | 0.19751631 | 0.049129512 | 0.234974405 | Up | 1.308657551 |
| FAM209A | 0.42892625 | 0.009604876 | 0.123095064 | Up | 2.017508218 |
| FAM209B | 0.354308474 | 0.023637704 | NA | Up | 1.626394719 |
| TFAP2C | 0.692244929 | 0.135377843 | NA | NS | 0.868452411 |
| BMP7 | 1.092416564 | 0.021203181 | 0.168258925 | Up | 1.673598973 |
| SPO11 | 0.927976872 | 0.058672316 | NA | NS | 1.231566767 |
| RAE1 | -0.086780284 | 0.048075919 | 0.23287536 | NS | 1.318072403 |
| MTRNR2L3 | 0.197598737 | 0.103366965 | 0.31928753 | NS | 0.985618234 |
| RBM38 | -0.322407653 | 0.102426868 | 0.318159125 | NS | 0.989586108 |
| HMGB1P1 | 0.527996411 | 0.15566769 | NA | NS | 0.80780152 |
| CTCFL | 0.57249631 | 0.166314343 | 0.394295091 | NS | 0.779070296 |
| PCK1 | 0.767164791 | 0.08548409 | NA | NS | 1.068114706 |
| ZBP1 | 0.122204849 | 0.153832393 | 0.380543257 | NS | 0.812952203 |
| PMEPA1 | 0.151429649 | 0.219557773 | 0.448876282 | NS | 0.658451184 |
| C20orf85 | 1.24258708 | 0.03480048 | NA | Up | 1.458414769 |
| ANKRD60 | 1.112261244 | 0.046184019 | NA | Up | 1.33550828 |
| RAB22A | 0.031924541 | 0.429623618 | 0.627190497 | NS | 0.366911852 |
| VAPB | -0.020831826 | 0.787190451 | 0.87719095 | NS | 0.103920183 |
| APCDD1L | 0.592621967 | 0.00146162 | 0.062253356 | Up | 2.835165582 |
| STX16 | 0.196070793 | 0.020670491 | 0.166806057 | Up | 1.684649203 |
| STX16-NPEPL1 | 0.031043731 | 0.78703947 | 0.877118148 | NS | 0.104003487 |
| NPEPL1 | 0.223128111 | 0.037024131 | 0.209468043 | Up | 1.431515132 |
| GNAS | -0.117567158 | 0.308511875 | 0.529171648 | NS | 0.510728115 |
| CTSZ | -0.134465288 | 0.081957622 | 0.28847931 | NS | 1.086410652 |
| TUBB1 | 0.161584687 | 0.254510058 | 0.48140404 | NS | 0.59429505 |
| ATP5F1E | -0.253821163 | 0.055854513 | 0.249087638 | NS | 1.25294173 |
| PRELID3B | -0.224661155 | 0.007238544 | 0.11179811 | Down | 2.140348786 |
| ZNF831 | 0.07233843 | 0.308302745 | 0.529093254 | NS | 0.511022608 |
| EDN3 | 0.528411985 | 0.094314685 | NA | NS | 1.025420682 |
| PHACTR3 | 0.352273439 | 0.072168391 | 0.273778447 | NS | 1.141652979 |
| SYCP2 | 0.067650271 | 0.376208857 | 0.586277575 | NS | 0.424570984 |
| FAM217B | 0.035375106 | 0.564213323 | 0.728337377 | NS | 0.248556663 |
| PPP1R3D | 0.093944378 | 0.29698131 | 0.518839421 | NS | 0.527270881 |
| CDH26 | 0.115896988 | 0.23549692 | 0.463333673 | NS | 0.628014769 |
| CDH4 | 0.708072098 | 0.080881483 | NA | NS | 1.092150897 |
| TAF4 | 0.067908324 | 0.663781944 | 0.798689271 | NS | 0.177974566 |
| LSM14B | -0.09286007 | 0.149095547 | 0.375188026 | NS | 0.826535326 |
| PSMA7 | -0.217540699 | 0.006063699 | 0.104179163 | Down | 2.217262397 |
| SS18L1 | 0.082362708 | 0.159507694 | 0.387098757 | NS | 0.797218364 |
| MTG2 | 0.094936007 | 0.10991155 | 0.328242412 | NS | 0.958956666 |
| HRH3 | 1.192437408 | 0.009862949 | NA | Up | 2.005993196 |
| OSBPL2 | -0.061321799 | 0.385050322 | 0.593353685 | NS | 0.414482509 |
| ADRM1 | 0.083478905 | 0.247650715 | 0.475813479 | NS | 0.606160414 |
| LAMA5 | 0.637643796 | 0.007616034 | 0.113408591 | Up | 2.118271118 |
| RPS21 | -0.312800541 | 0.107233311 | 0.324248389 | NS | 0.969670283 |
| CABLES2 | 0.054247205 | 0.537065438 | 0.708735809 | NS | 0.269972795 |
| RBBP8NL | 1.06968821 | 0.014893327 | NA | Up | 1.827008275 |
| GATA5 | 1.275943597 | 0.006561375 | NA | Up | 2.183005134 |
| SLCO4A1 | 0.594195721 | 0.006485841 | 0.1080547 | Up | 2.188033685 |
| NTSR1 | 0.35255059 | 0.076191649 | 0.279935021 | NS | 1.118092627 |
| MRGBP | -0.190428221 | 0.012624432 | 0.135545097 | Down | 1.898788152 |
| OGFR | -0.059297249 | 0.424654507 | 0.623345566 | NS | 0.371964262 |
| COL9A3 | 0.417199768 | 0.010656112 | 0.127927347 | Up | 1.972401228 |
| TCFL5 | -0.121453326 | 0.092228002 | 0.304264722 | NS | 1.0351372 |
| DIDO1 | 0.028610418 | 0.435496646 | 0.631369512 | NS | 0.361015186 |
| GID8 | -0.093002865 | 0.263692975 | 0.490023249 | NS | 0.57890144 |
| SLC17A9 | 0.238427362 | 0.095554332 | 0.309119979 | NS | 1.019749617 |
| BHLHE23 | 1.490524823 | 0.036198934 | NA | Up | 1.441304219 |
| YTHDF1 | -0.045643185 | 0.147883605 | 0.373742695 | NS | 0.830079972 |
| BIRC7 | 0.848406411 | 0.047318862 | NA | Up | 1.324965711 |
| NKAIN4 | 0.661781076 | 0.184388613 | NA | NS | 0.734265903 |
| ARFGAP1 | -0.017823814 | 0.85165822 | 0.916355684 | NS | 0.069734657 |
| COL20A1 | 0.948043568 | 0.017962493 | 0.15641156 | Up | 1.745633384 |
| CHRNA4 | 0.756826647 | 0.076299703 | 0.279989714 | NS | 1.117477151 |
| KCNQ2 | 0.756332058 | 0.072908547 | 0.274846078 | NS | 1.137221557 |
| EEF1A2 | 0.853654023 | 0.024694046 | 0.178011662 | Up | 1.607407742 |
| PPDPF | -0.399941234 | 0.104043312 | 0.320096712 | NS | 0.982785833 |
| PTK6 | 0.015004413 | 0.92472507 | 0.958933826 | NS | 0.033987368 |
| SRMS | 0.69517079 | 0.033443069 | 0.201567051 | Up | 1.47569388 |
| FNDC11 | 0.364866471 | 0.019336103 | 0.161365225 | Up | 1.713631042 |
| HELZ2 | 0.253917612 | 0.043844771 | 0.224412931 | Up | 1.358082193 |
| GMEB2 | 0.086854118 | 0.16977217 | 0.398291563 | NS | 0.7701335 |
| STMN3 | -0.100006471 | 0.213739584 | 0.443234232 | NS | 0.670115039 |
| RTEL1 | 0.133876519 | 0.381391612 | 0.591038759 | NS | 0.418628863 |
| RTEL1-TNFRSF6B | -0.048079344 | 0.769685066 | 0.865739766 | NS | 0.11368694 |
| ARFRP1 | -0.085917142 | 0.102646259 | 0.318537213 | NS | 0.988656873 |
| ZGPAT | -0.213862693 | 0.025437179 | 0.180429975 | Down | 1.594531062 |
| LIME1 | -0.067669573 | 0.466949439 | 0.65680359 | NS | 0.330730142 |
| SLC2A4RG | 0.178369398 | 0.056855317 | 0.250307665 | NS | 1.245228914 |
| ZBTB46 | 0.285192522 | 0.055508603 | 0.248604264 | NS | 1.255639706 |
| TPD52L2 | -0.068743075 | 0.292724061 | 0.515290877 | NS | 0.533541578 |
| DNAJC5 | -0.024054403 | 0.697418727 | 0.820690766 | NS | 0.156506395 |
| UCKL1 | -0.155777597 | 0.05355986 | 0.244494302 | NS | 1.271160567 |
| ZNF512B | 0.087784743 | 0.53660711 | 0.708403046 | NS | 0.270343577 |
| SAMD10 | 0.243665672 | 0.140366418 | 0.36472336 | NS | 0.852736784 |
| PRPF6 | -0.258591367 | 2.05E-04 | 0.023784694 | Down | 3.688343693 |
| C20orf204 | 0.292458543 | 0.028337219 | 0.188273565 | Up | 1.54764278 |
| SOX18 | 1.006227847 | 0.073910861 | NA | NS | 1.131291736 |
| TCEA2 | 0.009924161 | 0.883743642 | 0.934370959 | NS | 0.053673698 |
| RGS19 | 0.180825255 | 0.010657819 | 0.127927347 | Up | 1.972331647 |
| OPRL1 | 0.313894427 | 0.012118982 | 0.134107677 | Up | 1.916533868 |
| LKAAEAR1 | 0.371047478 | 0.114615676 | NA | NS | 0.94075598 |
| PCMTD2 | -0.194145713 | 0.021185477 | 0.168202484 | Down | 1.673961743 |
| GATD3B | 0.196206763 | 0.27404643 | 0.498866181 | NS | 0.562175851 |
| SIK1B | 0.430873211 | 0.030741351 | 0.194647058 | Up | 1.512277057 |
| CBSL | 0.996320738 | 0.038237371 | NA | Up | 1.417511973 |
| U2AF1L5 | 0.385739732 | 0.21238664 | 0.442188984 | NS | 0.672872806 |
| KCNE1B | 0.333413756 | 0.244854194 | NA | NS | 0.611092452 |
| TPTE | 0.389013762 | 0.463210903 | NA | NS | 0.334221227 |
| POTED | 0.025832289 | 0.96260285 | NA | NS | 0.016552857 |
| LIPI | 1.033507388 | 0.001647329 | NA | Up | 2.783219733 |
| RBM11 | 0.052883676 | 0.738855689 | 0.846571592 | NS | 0.131440379 |
| HSPA13 | -0.250444069 | 0.010896296 | 0.128632465 | Down | 1.962721089 |
| SAMSN1 | 0.013011725 | 0.904591745 | 0.947195487 | NS | 0.04354738 |
| NRIP1 | -0.160341313 | 0.048763509 | 0.234036095 | Down | 1.311905052 |
| USP25 | -0.07849125 | 0.153139992 | 0.379723802 | NS | 0.814911379 |
| CXADR | 0.128334392 | 0.498759797 | 0.680564637 | NS | 0.30210856 |
| BTG3 | 0.001915186 | 0.982817506 | 0.989819109 | NS | 0.007527116 |
| C21orf91 | -0.028731996 | 0.725695703 | 0.838541018 | NS | 0.139245449 |
| CHODL | 0.089124923 | 0.707042719 | 0.826665892 | NS | 0.150554346 |
| TMPRSS15 | 0.486471447 | 0.236281186 | 0.46408699 | NS | 0.626570859 |
| NCAM2 | 0.496893925 | 0.19336436 | 0.422666547 | NS | 0.71362357 |
| MRPL39 | -0.136954177 | 0.145285791 | 0.37054456 | NS | 0.837776858 |
| JAM2 | 0.248440274 | 0.150409212 | 0.376796299 | NS | 0.822725565 |
| ATP5PF | 0.050988923 | 0.697324246 | 0.82062175 | NS | 0.156565234 |
| GABPA | -0.143781458 | 0.055489835 | 0.248604264 | NS | 1.255786566 |
| APP | -0.137041609 | 0.1793351 | 0.409484791 | NS | 0.746334701 |
| CYYR1 | 0.560909146 | 0.02387642 | NA | Up | 1.622030786 |
| ADAMTS1 | -0.351697421 | 0.018007464 | 0.15641156 | Down | 1.744547443 |
| ADAMTS5 | -0.058666001 | 0.688381355 | 0.815165966 | NS | 0.162170901 |
| N6AMT1 | -0.067800456 | 0.416450657 | 0.61755717 | NS | 0.380436449 |
| LTN1 | -0.065228175 | 0.164757307 | 0.392395158 | NS | 0.783155316 |
| RWDD2B | 0.052108822 | 0.538716932 | 0.709653107 | NS | 0.268639374 |
| USP16 | -0.167808969 | 0.018979681 | 0.159923717 | Down | 1.721711084 |
| CCT8 | -0.167224505 | 0.037071681 | 0.209518227 | Down | 1.430957716 |
| MAP3K7CL | -0.352308012 | 0.006048343 | 0.103993354 | Down | 2.218363601 |
| BACH1 | -0.016715778 | 0.838413593 | 0.908983201 | NS | 0.076541689 |
| GRIK1 | 0.202402478 | 0.095623086 | 0.309185362 | NS | 1.019437244 |
| CLDN17 | 0.539913084 | 0.429016854 | NA | NS | 0.367525646 |
| CLDN8 | 0.991412515 | 0.173323379 | NA | NS | 0.761142853 |
| KRTAP24-1 | 0.963167876 | 0.20601761 | NA | NS | 0.686095655 |
| KRTAP26-1 | 1.285154267 | 0.054027988 | NA | NS | 1.267381208 |
| KRTAP27-1 | 0.426087123 | 0.594517956 | NA | NS | 0.225835024 |
| KRTAP13-2 | 0.515886426 | 0.49072534 | NA | NS | 0.309161516 |
| KRTAP13-1 | 0.261433702 | 0.685313113 | NA | NS | 0.164110958 |
| KRTAP13-3 | 0.273903569 | 0.698352988 | NA | NS | 0.155925004 |
| KRTAP13-4 | 0.426209391 | 0.463707027 | NA | NS | 0.333756323 |
| KRTAP15-1 | 0.27567024 | 0.726813061 | NA | NS | 0.138577277 |
| KRTAP19-1 | 0.882219542 | 0.384512493 | NA | NS | 0.415089545 |
| KRTAP19-2 | 0.412456569 | 0.684988552 | NA | NS | 0.164316687 |
| KRTAP19-3 | 0.391312678 | 0.575947242 | NA | NS | 0.239617297 |
| KRTAP19-4 | 1.210886847 | 0.297311486 | NA | NS | 0.526788312 |
| KRTAP19-5 | 0.91473423 | 0.248198406 | NA | NS | 0.605201011 |
| KRTAP19-6 | 0.661971999 | 0.542339076 | NA | NS | 0.265729103 |
| KRTAP19-7 | 0.915767753 | 0.250524521 | NA | NS | 0.60114976 |
| KRTAP6-3 | 0.623275948 | 0.390857905 | NA | NS | 0.4079811 |
| KRTAP6-2 | 0.144499742 | 0.851885975 | NA | NS | 0.069618532 |
| KRTAP22-1 | 0.420826737 | 0.597427396 | NA | NS | 0.223714866 |
| KRTAP20-1 | 0.77903594 | 0.396710657 | NA | NS | 0.401526132 |
| KRTAP20-3 | 0.783994122 | 0.347496028 | NA | NS | 0.459050155 |
| KRTAP21-3 | 0.29677919 | 0.710034517 | NA | NS | 0.148720539 |
| KRTAP21-2 | 0.728570932 | 0.346068236 | NA | NS | 0.460838261 |
| KRTAP21-1 | 1.275333482 | 0.100643121 | NA | NS | 0.997215905 |
| KRTAP8-1 | 0.812302381 | 0.200464444 | NA | NS | 0.697962646 |
| KRTAP7-1 | 0.645930418 | 0.379400119 | NA | NS | 0.420902537 |
| KRTAP11-1 | 0.551172684 | 0.386187879 | NA | NS | 0.413201361 |
| KRTAP19-8 | -0.309883245 | 0.668720532 | NA | NS | 0.174755342 |
| TIAM1 | 0.034933561 | 0.634028041 | 0.776753334 | NS | 0.197891534 |
| SOD1 | -0.147615859 | 0.156038181 | 0.383185333 | NS | 0.806769122 |
| SCAF4 | 0.084362245 | 0.389789755 | 0.596958206 | NS | 0.409169579 |
| HUNK | 0.557195189 | 0.109009915 | 0.327029744 | NS | 0.962534001 |
| MIS18A | 0.086628459 | 0.458184105 | 0.649581055 | NS | 0.338959982 |
| MRAP | 1.062397414 | 0.002936458 | NA | Up | 2.532176241 |
| URB1 | -0.043092782 | 0.479090817 | 0.666432088 | NS | 0.319582153 |
| CFAP298-TCP10L | -0.083428464 | 0.485015852 | 0.670924924 | NS | 0.314244067 |
| EVA1C | -0.017189048 | 0.846242401 | 0.913405762 | NS | 0.072505218 |
| TCP10L | 0.350169589 | 0.164276275 | 0.392064948 | NS | 0.784425154 |
| CFAP298 | -0.205064356 | 0.001090043 | 0.05356245 | Down | 2.96255654 |
| SYNJ1 | 0.127560567 | 0.065974905 | 0.265129038 | NS | 1.180621224 |
| PAXBP1 | -0.063517515 | 0.19225537 | 0.42102173 | NS | 0.716121522 |
| OLIG2 | 0.369072689 | 0.049853904 | 0.236485936 | Up | 1.30230083 |
| OLIG1 | 0.472069261 | 0.001076124 | 0.053345026 | Up | 2.9681375 |
| IFNAR2 | 0.12746233 | 0.078892936 | 0.283714663 | NS | 1.102961883 |
| IL10RB | 0.090474681 | 0.448153036 | 0.641635461 | NS | 0.348573657 |
| IFNAR1 | 0.174028104 | 0.017077578 | 0.153632509 | Up | 1.767573728 |
| IFNGR2 | -0.00541657 | 0.957898874 | 0.976662809 | NS | 0.018680337 |
| TMEM50B | -0.234482337 | 0.015333123 | 0.147421994 | Down | 1.814369371 |
| GART | -0.119366828 | 0.028525796 | 0.188728833 | Down | 1.544762229 |
| SON | 0.050040837 | 0.357776805 | 0.570884829 | NS | 0.446387819 |
| DONSON | 0.091626232 | 0.096402708 | 0.310522478 | NS | 1.015910766 |
| CRYZL1 | 0.017157582 | 0.695273022 | 0.81928571 | NS | 0.157844622 |
| ITSN1 | -0.090872201 | 0.52317706 | 0.698851036 | NS | 0.281351307 |
| ATP5PO | -0.17247775 | 0.151191492 | 0.377654815 | NS | 0.820472646 |
| MRPS6 | 0.097341685 | 0.087380502 | 0.296868159 | NS | 1.058585464 |
| SLC5A3 | 0.031147754 | 0.688087374 | 0.814944254 | NS | 0.162356411 |
| KCNE2 | 0.274523862 | 0.63225025 | NA | NS | 0.19911099 |
| KCNE1 | 0.406334162 | 0.011808336 | 0.133153378 | Up | 1.927811297 |
| RCAN1 | -0.042426772 | 0.573202209 | 0.734733139 | NS | 0.241692144 |
| CLIC6 | 0.37822596 | 0.232724056 | 0.461082819 | NS | 0.633158723 |
| RUNX1 | 0.201195648 | 0.010772784 | 0.12826482 | Up | 1.967672055 |
| SETD4 | -0.039012967 | 0.420441259 | 0.620581631 | NS | 0.376294672 |
| CBR1 | -0.320912812 | 0.006521214 | 0.108176159 | Down | 2.185671541 |
| CBR3 | -0.252797027 | 0.140921952 | 0.365337848 | NS | 0.851021349 |
| DOP1B | 0.049426612 | 0.482292967 | 0.66853282 | NS | 0.316689071 |
| MORC3 | -0.55628089 | 4.26E-05 | 0.008952073 | Down | 4.370523299 |
| CHAF1B | 0.041012995 | 0.793053198 | 0.880637817 | NS | 0.100697679 |
| CLDN14 | 0.814449333 | 0.100653085 | NA | NS | 0.997172911 |
| SIM2 | 1.104557298 | 4.68E-04 | 0.036600787 | Up | 3.329515372 |
| HLCS | -0.018259482 | 0.755927025 | 0.857296263 | NS | 0.121520128 |
| RIPPLY3 | 0.623052357 | 0.235493836 | NA | NS | 0.628020456 |
| PIGP | -0.147757101 | 0.070578803 | 0.271500817 | NS | 1.151325714 |
| TTC3 | -0.041551388 | 0.47904876 | 0.666414062 | NS | 0.31962028 |
| VPS26C | 0.054534649 | 0.087824487 | 0.297581949 | NS | 1.056384379 |
| DYRK1A | -0.03502037 | 0.433050176 | 0.62957628 | NS | 0.36346178 |
| KCNJ6 | 0.68750823 | 0.069074237 | 0.269259661 | NS | 1.160683906 |
| KCNJ15 | 0.078976693 | 0.650848817 | 0.789620058 | NS | 0.18651988 |
| ERG | 0.392080767 | 0.13063314 | 0.352635569 | NS | 0.883946635 |
| ETS2 | -0.068168926 | 0.485551488 | 0.671376828 | NS | 0.313764711 |
| PSMG1 | -0.217017564 | 0.00884807 | 0.119269279 | Down | 2.053151439 |
| BRWD1 | -0.104159333 | 0.03089443 | 0.195241361 | Down | 1.51011982 |
| HMGN1 | -0.099386083 | 0.128199188 | 0.350012309 | NS | 0.892114727 |
| GET1 | 0.013817083 | 0.857220041 | 0.919577187 | NS | 0.066907684 |
| LCA5L | 0.364775557 | 0.019745898 | 0.163256521 | Up | 1.704523105 |
| SH3BGR | 0.327402284 | 0.446087945 | NA | NS | 0.350579513 |
| B3GALT5 | 0.458017604 | 0.278725348 | 0.503079817 | NS | 0.554823534 |
| IGSF5 | 0.557171877 | 0.27701337 | NA | NS | 0.557499269 |
| PCP4 | 0.815110435 | 0.208718149 | NA | NS | 0.680439786 |
| DSCAM | 0.485976099 | 0.23024118 | 0.458600061 | NS | 0.637816998 |
| BACE2 | 0.184427132 | 0.078575926 | 0.283348427 | NS | 1.104710492 |
| FAM3B | 0.837188367 | 0.010594347 | 0.127927347 | Up | 1.974925788 |
| MX2 | 0.156098571 | 0.081466373 | 0.287767953 | NS | 1.089021618 |
| MX1 | 0.233896754 | 0.140030746 | 0.364288325 | NS | 0.853776599 |
| TMPRSS2 | 0.612392495 | 0.089435248 | 0.299870767 | NS | 1.048491286 |
| RIPK4 | 0.77723061 | 0.006670057 | NA | Up | 2.175870451 |
| PRDM15 | 0.076160713 | 0.275391041 | 0.499985735 | NS | 0.560050192 |
| C2CD2 | 0.136968486 | 0.073906758 | 0.276119505 | NS | 1.131315849 |
| ZBTB21 | -0.031330963 | 0.491596928 | 0.675742923 | NS | 0.30839084 |
| UMODL1 | 0.466135903 | 0.037922888 | 0.211935087 | Up | 1.421098594 |
| ABCG1 | -0.277114361 | 0.012903995 | 0.136242016 | Down | 1.889275827 |
| TFF3 | 0.58226745 | 0.025570606 | NA | Up | 1.592258987 |
| TFF2 | 0.708856816 | 0.279955993 | NA | NS | 0.55291023 |
| TMPRSS3 | 0.327443689 | 0.082018645 | 0.288503668 | NS | 1.08608741 |
| UBASH3A | 0.009419425 | 0.911249371 | 0.950841372 | NS | 0.040362758 |
| RSPH1 | 0.470625676 | 0.167765644 | NA | NS | 0.775296972 |
| SLC37A1 | -0.045130677 | 0.462956537 | 0.653595772 | NS | 0.334459779 |
| PDE9A | 0.162229301 | 0.394095978 | 0.60006556 | NS | 0.404397998 |
| WDR4 | 0.101940017 | 0.253673902 | 0.480532648 | NS | 0.59572421 |
| NDUFV3 | -0.161434128 | 0.005926138 | 0.103131013 | Down | 2.227228237 |
| ERVH48-1 | 0.058828026 | 0.646021196 | 0.786192259 | NS | 0.189753233 |
| PKNOX1 | 0.05201888 | 0.357147622 | 0.570310614 | NS | 0.447152238 |
| CBS | 0.548653204 | 0.059624235 | NA | NS | 1.224577177 |
| U2AF1 | 0.060247064 | 0.840542366 | 0.910084212 | NS | 0.075440392 |
| SIK1 | 0.479856302 | 0.438587582 | NA | NS | 0.357943669 |
| HSF2BP | -0.111867363 | 0.652739576 | NA | NS | 0.185260055 |
| RRP1B | 0.084668174 | 0.161375764 | 0.389034501 | NS | 0.792161688 |
| PDXK | 0.076040832 | 0.234129992 | 0.46228643 | NS | 0.63054295 |
| CSTB | 0.039276949 | 0.614908445 | 0.764404994 | NS | 0.211189542 |
| RRP1 | -0.116758381 | 0.213521401 | 0.443180155 | NS | 0.670558589 |
| AGPAT3 | 0.048973075 | 0.548200486 | 0.716398718 | NS | 0.261060584 |
| TRAPPC10 | -0.131099514 | 0.011760531 | 0.132876014 | Down | 1.929573071 |
| PWP2 | 0.384540866 | 0.31230994 | 0.532023375 | NS | 0.505414193 |
| GATD3A | 0.667152681 | 0.075209016 | 0.278206739 | NS | 1.123730092 |
| ICOSLG | 0.484172553 | 0.146761547 | NA | NS | 0.83338772 |
| DNMT3L | 1.023761412 | 0.100286547 | NA | NS | 0.99875732 |
| AIRE | 0.288355402 | 0.042258425 | 0.220751111 | Up | 1.374086693 |
| PFKL | -0.204552184 | 0.034465665 | 0.203698751 | Down | 1.462613337 |
| CFAP410 | -0.070149015 | 0.476658344 | 0.664460977 | NS | 0.3217928 |
| TRPM2 | 0.028490092 | 0.857344529 | 0.91958151 | NS | 0.066844619 |
| LRRC3 | 0.484909282 | 0.052131472 | 0.241262687 | NS | 1.282900016 |
| TSPEAR | 0.486806971 | 0.06026674 | 0.256083601 | NS | 1.219922301 |
| KRTAP10-1 | 1.535548339 | 0.004843282 | NA | Up | 2.314860284 |
| KRTAP10-2 | 0.627321447 | 0.04170962 | NA | Up | 1.37976377 |
| KRTAP10-3 | 0.754386115 | 0.092816777 | NA | NS | 1.032373517 |
| KRTAP10-4 | 1.267687891 | 0.009998038 | NA | Up | 2.000085196 |
| KRTAP10-5 | 0.649184652 | 0.203264759 | NA | NS | 0.691937911 |
| KRTAP10-6 | 0.159124101 | 0.762672937 | NA | NS | 0.117661664 |
| KRTAP10-7 | 0.45616267 | 0.360000428 | NA | NS | 0.443696983 |
| KRTAP10-8 | 0.908492576 | 0.104424496 | NA | NS | 0.981197614 |
| KRTAP10-9 | 0.767015425 | 0.108273915 | NA | NS | 0.965476161 |
| KRTAP10-10 | 0.783221281 | 0.157370562 | NA | NS | 0.803076504 |
| KRTAP10-11 | 0.318242602 | 0.442778555 | NA | NS | 0.353813421 |
| KRTAP12-4 | 0.345581306 | 0.427363743 | NA | NS | 0.369202326 |
| KRTAP12-3 | 0.089676635 | 0.794962004 | NA | NS | 0.099653628 |
| KRTAP12-2 | 1.133722125 | 0.134100832 | NA | NS | 0.872568528 |
| KRTAP12-1 | 1.278259811 | 0.034524554 | NA | Up | 1.461871928 |
| KRTAP10-12 | 0.958918348 | 0.140316948 | NA | NS | 0.852889869 |
| UBE2G2 | -0.069200343 | 0.089347223 | 0.299813934 | NS | 1.048918941 |
| SUMO3 | 0.16166675 | 0.030770366 | 0.194776928 | Up | 1.511867335 |
| PTTG1IP | -0.053393167 | 0.381532394 | 0.591109727 | NS | 0.418468582 |
| ITGB2 | -0.249875518 | 0.059399103 | 0.254232499 | NS | 1.226220112 |
| SLX9 | -0.141345992 | 0.149906563 | 0.376071871 | NS | 0.824179353 |
| ADARB1 | 0.012114575 | 0.874655192 | 0.929380875 | NS | 0.058163121 |
| POFUT2 | -0.001205714 | 0.989499855 | 0.993955951 | NS | 0.004584265 |
| COL18A1 | -0.053500638 | 0.712151768 | 0.829996153 | NS | 0.147427443 |
| SLC19A1 | -0.013274928 | 0.921749867 | 0.956852823 | NS | 0.035386916 |
| PCBP3 | 0.222246888 | 0.266451349 | 0.492363509 | NS | 0.574382076 |
| COL6A1 | 0.445776002 | 0.056093855 | 0.249087638 | NS | 1.251084716 |
| COL6A2 | -0.225554154 | 0.054618647 | 0.24676996 | NS | 1.262659065 |
| FTCD | 0.701593573 | 0.092236869 | NA | NS | 1.03509545 |
| SPATC1L | 0.552863888 | 0.086829086 | NA | NS | 1.061334768 |
| LSS | -0.197666371 | 0.037363411 | 0.210115856 | Down | 1.427553487 |
| MCM3AP | 0.033923212 | 0.566693697 | 0.730251058 | NS | 0.246651618 |
| YBEY | -0.080781431 | 0.361991265 | 0.574559841 | NS | 0.441301909 |
| C21orf58 | 0.15761072 | 0.273468224 | 0.498634409 | NS | 0.56309313 |
| PCNT | -0.317963519 | 0.003351879 | 0.085084692 | Down | 2.474711619 |
| DIP2A | 0.03663323 | 0.677574627 | 0.807588556 | NS | 0.169042865 |
| S100B | 0.211057029 | 0.47196635 | 0.660614433 | NS | 0.326088964 |
| PRMT2 | 0.004695223 | 0.911824271 | 0.951111288 | NS | 0.040088852 |
| POTEH | -0.060149646 | 0.908982783 | NA | NS | 0.041444343 |
| CCT8L2 | 1.10480077 | 0.029192246 | NA | Up | 1.534732486 |
| XKR3 | 0.265075296 | 0.393401653 | 0.599726064 | NS | 0.405163819 |
| GAB4 | 0.636616857 | 0.081392841 | NA | NS | 1.089413794 |
| IL17RA | 0.206110807 | 0.053135606 | 0.243367273 | NS | 1.274614364 |
| TMEM121B | 0.107636299 | 0.380696567 | 0.59065868 | NS | 0.419421039 |
| HDHD5 | -0.129798828 | 0.055929095 | 0.249087638 | NS | 1.252362206 |
| ADA2 | 0.085763033 | 0.290012711 | 0.512990599 | NS | 0.537582967 |
| CECR2 | 0.390098982 | 0.203448281 | 0.433444748 | NS | 0.691545975 |
| SLC25A18 | 0.162548602 | 0.186021177 | 0.41563483 | NS | 0.730437611 |
| ATP6V1E1 | -0.115701197 | 0.171914254 | 0.400641833 | NS | 0.764688113 |
| BCL2L13 | -0.119161205 | 0.235072933 | 0.463064159 | NS | 0.628797374 |
| BID | -0.025309746 | 0.725256057 | 0.838202171 | NS | 0.139508636 |
| MICAL3 | -0.042286745 | 0.698221036 | 0.821297286 | NS | 0.156007071 |
| PEX26 | 0.009112416 | 0.869504458 | 0.926426506 | NS | 0.060728187 |
| TUBA8 | 0.25500645 | 0.118195165 | 0.338583901 | NS | 0.927400288 |
| USP18 | 0.098255127 | 0.486898553 | 0.67206682 | NS | 0.312561516 |
| TMEM191B | -0.504687394 | 0.128722714 | NA | NS | 0.890344812 |
| RIMBP3 | 0.177815646 | 0.150491941 | 0.376962314 | NS | 0.822486756 |
| DGCR6 | -0.055920982 | 0.86535486 | NA | NS | 0.062805763 |
| DGCR2 | 0.048530225 | 0.552674427 | 0.719781048 | NS | 0.25753063 |
| ESS2 | -0.068727071 | 0.253640522 | 0.480509161 | NS | 0.595781361 |
| GSC2 | 0.717650176 | 0.156007553 | NA | NS | 0.806854376 |
| SLC25A1 | 0.015017033 | 0.851576012 | 0.916355684 | NS | 0.069776581 |
| CLTCL1 | 0.129903125 | 0.191686494 | 0.420934764 | NS | 0.717408485 |
| HIRA | -0.089540807 | 0.143230169 | 0.368154582 | NS | 0.843965496 |
| C22orf39 | -0.067289857 | 0.214013599 | 0.443279319 | NS | 0.669558631 |
| MRPL40 | -0.178047033 | 0.161087781 | 0.38887239 | NS | 0.7929374 |
| UFD1 | 0.071871099 | 0.063376484 | 0.261443316 | NS | 1.198071857 |
| CDC45 | 0.579970613 | 0.005318302 | 0.099529109 | Up | 2.274226987 |
| CLDN5 | 0.07751367 | 0.635411773 | 0.777679224 | NS | 0.196944743 |
| SEPTIN5 | 0.327582361 | 0.313936248 | NA | NS | 0.503158537 |
| TBX1 | 0.898432902 | 0.001586763 | 0.063754474 | Up | 2.799487887 |
| GNB1L | 0.097267757 | 0.291459425 | 0.514252986 | NS | 0.535421896 |
| RTL10 | -0.020906624 | 0.749390039 | 0.852754183 | NS | 0.125292084 |
| TXNRD2 | -0.201908987 | 0.053908134 | 0.245399341 | NS | 1.268345701 |
| COMT | -0.150136694 | 0.047098658 | 0.230730823 | Down | 1.326991464 |
| ARVCF | 0.203773761 | 0.161830311 | 0.38963426 | NS | 0.790940131 |
| TANGO2 | -0.046366948 | 0.744436535 | 0.850164936 | NS | 0.128172321 |
| DGCR8 | -0.019370265 | 0.807772004 | 0.889232573 | NS | 0.092711203 |
| TRMT2A | -0.064716113 | 0.279098733 | 0.503208948 | NS | 0.554242135 |
| RANBP1 | -0.142120729 | 0.09202508 | 0.303901713 | NS | 1.036093797 |
| ZDHHC8 | 0.215458336 | 0.069138521 | 0.269424945 | NS | 1.160279917 |
| CCDC188 | 0.45723442 | 0.123091311 | NA | NS | 0.909772601 |
| RTN4R | 0.474144636 | 0.008389655 | 0.117652418 | Up | 2.076255905 |
| DGCR6L | -0.120919902 | 0.210054026 | 0.440111431 | NS | 0.67766899 |
| USP41 | 0.507378049 | 0.1945047 | NA | NS | 0.711069899 |
| ZNF74 | -0.096416023 | 0.179687617 | 0.409908267 | NS | 0.74548185 |
| SCARF2 | 0.587601591 | 0.005551125 | 0.101139119 | Up | 2.255618966 |
| KLHL22 | 0.025986335 | 0.527441522 | 0.702304788 | NS | 0.277825684 |
| MED15 | 0.038783076 | 0.710413871 | 0.828870471 | NS | 0.148488568 |
| PI4KA | -0.035357576 | 0.722534393 | 0.836280521 | NS | 0.141141475 |
| SERPIND1 | 0.173083693 | 0.155731777 | 0.383185333 | NS | 0.807622761 |
| SNAP29 | 0.083565033 | 0.085183669 | 0.293490206 | NS | 1.069643657 |
| CRKL | -0.171637669 | 0.015314085 | 0.147421994 | Down | 1.814908947 |
| AIFM3 | -0.031106023 | 0.79932654 | 0.884559442 | NS | 0.097275767 |
| LZTR1 | 0.076355962 | 0.280039808 | 0.50348007 | NS | 0.552780229 |
| THAP7 | -0.180496035 | 0.017897897 | 0.156228986 | Down | 1.747197998 |
| P2RX6 | 0.545302609 | 0.020412648 | 0.165714619 | Up | 1.690100649 |
| SLC7A4 | 0.510021366 | 0.12337307 | NA | NS | 0.908779628 |
| LRRC74B | 0.701008042 | 0.122533113 | 0.343398823 | NS | 0.911746535 |
| GGT2 | 0.579574888 | 0.3497146 | NA | NS | 0.456286237 |
| RIMBP3B | -0.149815667 | 0.573558164 | NA | NS | 0.241422534 |
| HIC2 | 0.032381814 | 0.568063724 | 0.731174923 | NS | 0.245602943 |
| TMEM191C | 0.081256092 | 0.688146528 | NA | NS | 0.162319077 |
| UBE2L3 | -0.07195022 | 0.279468316 | 0.503278325 | NS | 0.553667422 |
| YDJC | 0.136325102 | 0.123550108 | 0.344394423 | NS | 0.90815687 |
| CCDC116 | 0.624044284 | 0.125472052 | NA | NS | 0.901453 |
| SDF2L1 | -0.075646703 | 0.493652447 | 0.67740743 | NS | 0.306578705 |
| PPIL2 | -0.098901599 | 0.174851805 | 0.404252655 | NS | 0.757329881 |
| YPEL1 | -0.085441873 | 0.286684694 | 0.509874023 | NS | 0.542595493 |
| MAPK1 | -0.029223418 | 0.651936638 | 0.790274329 | NS | 0.185794611 |
| PPM1F | 0.060934202 | 0.467138853 | 0.656899202 | NS | 0.33055401 |
| TOP3B | -0.197123638 | 0.096759574 | 0.310921075 | NS | 1.01430605 |
| VPREB1 | 0.959738683 | 0.028253393 | NA | Up | 1.548929389 |
| ZNF280B | 0.20477954 | 0.476864352 | NA | NS | 0.321605142 |
| GGTLC2 | 1.537766063 | 0.005042197 | NA | Up | 2.297380231 |
| IGLL5 | 0.242209138 | 0.151318217 | 0.377750911 | NS | 0.820108784 |
| RSPH14 | 0.326370765 | 0.113225441 | 0.332115589 | NS | 0.946055977 |
| GNAZ | -0.173230614 | 0.244496348 | 0.472368429 | NS | 0.611727624 |
| RAB36 | 0.337223488 | 0.041419099 | 0.219324903 | Up | 1.382799352 |
| BCR | -0.003664504 | 0.961922619 | 0.978825093 | NS | 0.016859863 |
| IGLL1 | 0.674756952 | 0.09647527 | NA | NS | 1.015583997 |
| DRICH1 | -0.085769089 | 0.587752254 | 0.746241386 | NS | 0.230805697 |
| RGL4 | -0.087119861 | 0.388462456 | 0.596204672 | NS | 0.410650949 |
| ZNF70 | 0.18765886 | 0.07711198 | 0.281454506 | NS | 1.112878145 |
| VPREB3 | 0.043984445 | 0.80669214 | 0.888684563 | NS | 0.093292175 |
| C22orf15 | 0.619857046 | 0.034204464 | NA | Up | 1.465917206 |
| CHCHD10 | 0.044192795 | 0.689666603 | 0.815465152 | NS | 0.161360804 |
| MMP11 | -0.360258768 | 0.183089334 | NA | NS | 0.737336956 |
| SMARCB1 | -0.236678338 | 0.025704248 | 0.181747049 | Down | 1.589995094 |
| DERL3 | 0.379003848 | 0.157072262 | NA | NS | 0.803900501 |
| SLC2A11 | -0.143154652 | 0.319822251 | 0.538985786 | NS | 0.495091324 |
| CABIN1 | 0.117593425 | 0.219794292 | 0.449119279 | NS | 0.65798359 |
| SUSD2 | 0.311346876 | 0.354508654 | 0.567759244 | NS | 0.450373159 |
| GGT5 | 0.244896079 | 0.368293007 | 0.580219196 | NS | 0.433806527 |
| SPECC1L | -0.069310809 | 0.254885917 | 0.481752683 | NS | 0.59365416 |
| SPECC1L-ADORA2A | 0.161052155 | 0.627912265 | NA | NS | 0.202101034 |
| ADORA2A | -0.029240978 | 0.747733201 | 0.851783936 | NS | 0.126253335 |
| UPB1 | 0.108760274 | 0.411733463 | 0.614131594 | NS | 0.385383835 |
| GUCD1 | -0.237835376 | 0.098283449 | 0.313029559 | NS | 1.007519614 |
| SNRPD3 | -0.297743361 | 5.64E-07 | 2.58E-04 | Down | 6.248946514 |
| LRRC75B | 0.452419448 | 0.045924872 | 0.228673646 | Up | 1.337952043 |
| GGT1 | 0.142352301 | 0.418388514 | 0.618901466 | NS | 0.378420247 |
| PIWIL3 | 0.272403087 | 0.523752708 | NA | NS | 0.280873719 |
| SGSM1 | 0.120120584 | 0.307459296 | 0.52835843 | NS | 0.512212371 |
| TMEM211 | 0.435934383 | 0.365452113 | NA | NS | 0.437169522 |
| KIAA1671 | 0.231147413 | 0.11637816 | 0.335848363 | NS | 0.934128515 |
| CRYBB3 | 0.789944691 | 0.009783818 | NA | Up | 2.009491624 |
| CRYBB2 | 0.433208675 | 0.492632546 | NA | NS | 0.3074769 |
| GRK3 | -0.091983376 | 0.244449028 | 0.472356703 | NS | 0.611811685 |
| MYO18B | 0.509151291 | 0.0595819 | 0.254791869 | NS | 1.224885653 |
| SEZ6L | 0.26981749 | 0.175929654 | 0.405354744 | NS | 0.754660952 |
| ASPHD2 | 0.07536043 | 0.298728094 | 0.520146566 | NS | 0.524723933 |
| HPS4 | -2.97E-04 | 0.995882366 | 0.997668429 | NS | 0.001791958 |
| SRRD | -0.302265815 | 0.036203128 | 0.208165711 | Down | 1.441253909 |
| TFIP11 | -0.230660848 | 1.62E-04 | 0.020530906 | Down | 3.791806701 |
| TPST2 | -0.162224235 | 0.021923044 | 0.169622143 | Down | 1.659099154 |
| CRYBB1 | 0.216077671 | 0.56689908 | NA | NS | 0.246494248 |
| CRYBA4 | 1.220540277 | 0.007120065 | NA | Up | 2.147516055 |
| MN1 | 0.882059875 | 0.002661458 | 0.078469363 | Up | 2.574880309 |
| PITPNB | 0.112070035 | 0.162350803 | 0.390030221 | NS | 0.789545558 |
| TTC28 | 0.106247235 | 0.368525357 | 0.580345715 | NS | 0.433532624 |
| CHEK2 | -0.40811239 | 0.001133818 | 0.054594994 | Down | 2.94545669 |
| HSCB | -0.237660121 | 0.052642309 | 0.242513843 | NS | 1.278665072 |
| CCDC117 | -0.10801368 | 0.098624273 | 0.313359199 | NS | 1.006016184 |
| XBP1 | -0.178197229 | 0.057383573 | 0.251040799 | NS | 1.241212416 |
| ZNRF3 | 0.369735769 | 0.051713145 | 0.24015089 | NS | 1.286399048 |
| C22orf31 | 0.68377471 | 0.231126523 | NA | NS | 0.636150214 |
| KREMEN1 | 0.164298157 | 0.387474745 | 0.595536879 | NS | 0.411756598 |
| EMID1 | 0.106696176 | 0.609296408 | 0.760524653 | NS | 0.215171382 |
| RHBDD3 | 0.133622931 | 0.078112721 | 0.282567926 | NS | 1.107278234 |
| EWSR1 | -0.131096701 | 0.016345362 | 0.151112662 | Down | 1.786605454 |
| GAS2L1 | 0.598621878 | 3.32E-04 | 0.03067151 | Up | 3.478686578 |
| RASL10A | 0.145127648 | 0.626060513 | NA | NS | 0.203383688 |
| AP1B1 | 0.09902877 | 0.279450946 | 0.503278325 | NS | 0.553694416 |
| RFPL1 | 0.786188635 | 0.306995201 | NA | NS | 0.512868413 |
| NEFH | 0.30928 | 0.073673062 | 0.275940596 | NS | 1.132691282 |
| THOC5 | 0.06262827 | 0.344599177 | 0.559597983 | NS | 0.462685764 |
| NIPSNAP1 | -0.010307221 | 0.847456419 | 0.914210023 | NS | 0.071882626 |
| NF2 | -0.01048097 | 0.852997455 | 0.917099521 | NS | 0.069052265 |
| CABP7 | 0.528027806 | 0.002639968 | 0.078363492 | Up | 2.578401319 |
| ZMAT5 | -0.085869399 | 0.235641229 | 0.463448831 | NS | 0.627748721 |
| UQCR10 | -0.184392741 | 0.125746021 | 0.347428564 | NS | 0.900505749 |
| ASCC2 | -0.378566562 | 0.049137772 | 0.234974405 | Down | 1.308584536 |
| MTMR3 | 0.054925028 | 0.555448132 | 0.721922646 | NS | 0.25535649 |
| HORMAD2 | 0.729271253 | 0.07405473 | NA | NS | 1.130447197 |
| LIF | 0.276963645 | 0.408087383 | NA | NS | 0.389246832 |
| OSM | 0.244752991 | 0.070021634 | 0.270674772 | NS | 1.154767758 |
| CASTOR1 | 7.99E-05 | 0.99951265 | 0.999687252 | NS | 2.12E-04 |
| TBC1D10A | 0.147870483 | 0.077569541 | 0.28177684 | NS | 1.110308779 |
| SF3A1 | 0.011917918 | 0.91696946 | 0.95404482 | NS | 0.037645128 |
| CCDC157 | 0.158019885 | 0.17219497 | 0.400977042 | NS | 0.763979539 |
| RNF215 | 0.03178059 | 0.686679599 | 0.813982316 | NS | 0.163245855 |
| SEC14L2 | 0.134391732 | 0.185815867 | 0.415463304 | NS | 0.730917203 |
| MTFP1 | 0.021941816 | 0.924081689 | NA | NS | 0.034289636 |
| SEC14L3 | -0.244733802 | 0.311530572 | 0.531239846 | NS | 0.506499327 |
| SEC14L4 | -0.504927381 | 0.020815959 | 0.167470045 | Down | 1.681603573 |
| SEC14L6 | 0.252381413 | 0.577377942 | NA | NS | 0.238539812 |
| GAL3ST1 | 0.518303803 | 0.153787933 | NA | NS | 0.813077741 |
| PES1 | -0.352588986 | 1.74E-04 | 0.02157417 | Down | 3.758767586 |
| TCN2 | -0.03155603 | 0.843953472 | 0.912443275 | NS | 0.073681496 |
| SLC35E4 | 0.168626148 | 0.235690706 | 0.463448831 | NS | 0.627657543 |
| DUSP18 | 0.204082592 | 0.005291293 | 0.099491956 | Up | 2.276438153 |
| OSBP2 | -0.180322474 | 0.417290943 | 0.618297974 | NS | 0.379561041 |
| MORC2 | -0.108077881 | 0.069217978 | 0.269505291 | NS | 1.159781095 |
| TUG1 | 0.125459516 | 0.019930339 | 0.163751071 | Up | 1.700485322 |
| SMTN | 0.453790234 | 0.040768994 | 0.217865963 | Up | 1.389670001 |
| SELENOM | 0.005391927 | 0.962616255 | 0.979032249 | NS | 0.016546809 |
| INPP5J | 0.863573154 | 0.038453911 | 0.213464789 | Up | 1.415059485 |
| PLA2G3 | 0.651858951 | 0.26245936 | NA | NS | 0.580937935 |
| RNF185 | -0.037517869 | 0.55473959 | 0.721469695 | NS | 0.255910839 |
| LIMK2 | 0.08114425 | 0.469992591 | 0.659019796 | NS | 0.327908988 |
| PIK3IP1 | -0.045600718 | 0.545229048 | 0.713941433 | NS | 0.263421014 |
| PATZ1 | 0.040992991 | 0.444540058 | 0.638698645 | NS | 0.352089098 |
| DRG1 | -0.232141163 | 0.00409876 | 0.090695453 | Down | 2.387347544 |
| EIF4ENIF1 | -0.080293159 | 0.144770914 | 0.369914478 | NS | 0.839318685 |
| SFI1 | -0.102025704 | 0.229959444 | 0.458158433 | NS | 0.63834875 |
| PISD | -0.048659328 | 0.611999034 | 0.762684698 | NS | 0.213249264 |
| PRR14L | -0.013765275 | 0.750737951 | 0.853694168 | NS | 0.12451163 |
| DEPDC5 | 0.081668433 | 0.276593744 | 0.500969068 | NS | 0.558157647 |
| YWHAH | 0.324296633 | 1.42E-04 | 0.019026652 | Up | 3.847514914 |
| SLC5A1 | 0.452933343 | 0.280201615 | 0.503488416 | NS | 0.552529366 |
| C22orf42 | 0.343986291 | 0.339814708 | NA | NS | 0.468757827 |
| RFPL2 | 0.051233219 | 0.757413116 | 0.858378114 | NS | 0.120667178 |
| SLC5A4 | 0.326932921 | 0.203161208 | 0.433163396 | NS | 0.692159215 |
| RFPL3 | 0.59327307 | 0.328822829 | NA | NS | 0.483038039 |
| RTCB | -0.192556353 | 0.024197215 | 0.176346063 | Down | 1.616234612 |
| BPIFC | 0.432853383 | 0.317104295 | NA | NS | 0.498797876 |
| FBXO7 | -0.266436416 | 0.127418514 | 0.348830209 | NS | 0.894767463 |
| SYN3 | 0.412185902 | 0.054642443 | 0.24676996 | NS | 1.262469894 |
| TIMP3 | 0.268706629 | 0.251206272 | 0.478040002 | NS | 0.599969522 |
| LARGE1 | 0.287201957 | 0.02723163 | 0.185833969 | Up | 1.564926356 |
| ISX | 1.010512098 | 0.020339315 | NA | Up | 1.691663677 |
| HMGXB4 | 0.076632891 | 0.074335943 | 0.276837611 | NS | 1.128801143 |
| TOM1 | -0.017525007 | 0.825893918 | 0.900867904 | NS | 0.083075732 |
| HMOX1 | -0.140388234 | 0.248304354 | 0.476110795 | NS | 0.605015665 |
| MCM5 | -0.065497434 | 0.181967213 | 0.412022664 | NS | 0.740006857 |
| RASD2 | 1.182446159 | 0.015451446 | NA | Up | 1.811030885 |
| MB | 0.552157287 | 0.256760202 | NA | NS | 0.590472291 |
| APOL6 | 0.070724192 | 0.474740114 | 0.662876362 | NS | 0.32354407 |
| RBFOX2 | 0.293429581 | 0.303504327 | NA | NS | 0.517835114 |
| APOL3 | -0.178118824 | 0.021424176 | 0.168379028 | Down | 1.669095865 |
| APOL4 | 0.314978669 | 0.12716567 | 0.348830209 | NS | 0.895630116 |
| APOL2 | 0.040157353 | 0.608316034 | 0.759851448 | NS | 0.215870736 |
| APOL1 | 0.089638194 | 0.367264873 | 0.579396578 | NS | 0.435020608 |
| MYH9 | -0.127039519 | 0.335567303 | 0.551756712 | NS | 0.474220362 |
| TXN2 | 0.092950676 | 0.43248715 | 0.629197097 | NS | 0.364026792 |
| FOXRED2 | 0.450627945 | 0.068287602 | 0.268476763 | NS | 1.16565814 |
| EIF3D | -0.210717239 | 0.006954362 | 0.110119911 | Down | 2.157742712 |
| CACNG2 | 0.879065012 | 0.084789713 | NA | NS | 1.071656834 |
| IFT27 | 0.121020229 | 0.073354932 | 0.275499766 | NS | 1.134570678 |
| NCF4 | 0.139677871 | 0.216214317 | 0.445580877 | NS | 0.665115552 |
| CSF2RB | 0.122091968 | 0.301152263 | 0.522578355 | NS | 0.521213869 |
| TEX33 | -0.227841023 | 0.686634839 | NA | NS | 0.163274165 |
| TST | 0.03414981 | 0.737362341 | 0.845368058 | NS | 0.132319047 |
| MPST | 0.023455746 | 0.776467689 | 0.869932305 | NS | 0.109876612 |
| KCTD17 | -0.161626353 | 0.091125973 | 0.302283754 | NS | 1.04035782 |
| TMPRSS6 | 0.404163471 | 0.228864197 | 0.457408835 | NS | 0.640422142 |
| IL2RB | 0.08521747 | 0.481340683 | 0.668181859 | NS | 0.317547431 |
| C1QTNF6 | 0.081686272 | 0.314395587 | 0.533741944 | NS | 0.502523558 |
| SSTR3 | 0.037149724 | 0.782847546 | 0.874453587 | NS | 0.106322806 |
| RAC2 | 0.280920071 | 9.62E-04 | 0.051639949 | Up | 3.01667328 |
| CYTH4 | -0.053688115 | 0.464472319 | 0.654918093 | NS | 0.333040163 |
| MFNG | 0.110293795 | 0.03868398 | 0.213650843 | Up | 1.412468845 |
| CARD10 | 1.014822788 | 0.012041945 | 0.13403215 | Up | 1.919303349 |
| CDC42EP1 | 0.349150952 | 0.200933719 | 0.430596428 | NS | 0.696947178 |
| LGALS2 | -0.118260853 | 0.572243331 | 0.733955912 | NS | 0.24241926 |
| GGA1 | -0.009505298 | 0.910757597 | 0.950585116 | NS | 0.040597197 |
| SH3BP1 | 0.144579978 | 0.204925275 | 0.43495148 | NS | 0.688404473 |
| PDXP | 0.308247811 | 0.187203659 | NA | NS | 0.727685668 |
| LGALS1 | -0.185001616 | 0.119301618 | 0.340007095 | NS | 0.923353666 |
| NOL12 | -0.025301384 | 0.717848255 | 0.833468225 | NS | 0.143967351 |
| TRIOBP | -0.003196922 | 0.966254418 | 0.980989391 | NS | 0.014908507 |
| H1-0 | -0.222749166 | 0.250919278 | 0.47774934 | NS | 0.60046597 |
| GCAT | -0.453181248 | 0.031955058 | 0.198142899 | Down | 1.495460395 |
| GALR3 | 1.310371009 | 0.039820564 | NA | Up | 1.399892598 |
| ANKRD54 | 0.070038191 | 0.259141143 | 0.485568555 | NS | 0.586463629 |
| EIF3L | -0.253735686 | 0.007175463 | 0.1114299 | Down | 2.144150071 |
| MICALL1 | 0.223376031 | 0.034094894 | 0.202909595 | Up | 1.467310659 |
| C22orf23 | 0.14743624 | 0.43780674 | NA | NS | 0.358717557 |
| POLR2F | -0.189360238 | 0.008476587 | 0.11782982 | Down | 2.071778999 |
| SOX10 | 0.226173511 | 0.174625988 | 0.404024697 | NS | 0.757891125 |
| PICK1 | -0.093086171 | 0.144466435 | 0.36967266 | NS | 0.840233043 |
| SLC16A8 | 0.690331989 | 0.039031444 | NA | Up | 1.408585381 |
| BAIAP2L2 | 0.174970167 | 0.574482374 | 0.735797513 | NS | 0.240723292 |
| PLA2G6 | 0.081034289 | 0.417328184 | 0.618297974 | NS | 0.379522284 |
| MAFF | -0.069046879 | 0.429661673 | 0.627198141 | NS | 0.366873385 |
| TMEM184B | 0.120463256 | 0.269158121 | 0.49444608 | NS | 0.569992512 |
| CSNK1E | -0.050349887 | 0.399746885 | 0.604849574 | NS | 0.398214912 |
| TPTEP2-CSNK1E | 1.084182089 | 0.033363515 | NA | Up | 1.476728204 |
| KCNJ4 | 1.316435809 | 0.006617449 | NA | Up | 2.179309401 |
| KDELR3 | 0.150600596 | 0.047020321 | 0.230640263 | Up | 1.327714412 |
| DDX17 | 0.059251425 | 0.144441922 | 0.36967266 | NS | 0.840306742 |
| DMC1 | -0.454358977 | 0.108551316 | 0.326591248 | NS | 0.964364907 |
| FAM227A | 0.72613441 | 0.021318304 | 0.168379028 | Up | 1.671247356 |
| CBY1 | -0.071697688 | 0.33447331 | 0.551080127 | NS | 0.475638532 |
| TOMM22 | -0.305561801 | 0.003647313 | 0.08688637 | Down | 2.438026971 |
| JOSD1 | -0.190994913 | 0.004029882 | 0.090296145 | Down | 2.394707626 |
| GTPBP1 | -0.050348501 | 0.616744481 | 0.765715595 | NS | 0.209894728 |
| SUN2 | -0.219779671 | 0.015539037 | 0.148281257 | Down | 1.808575908 |
| DNAL4 | -0.128763395 | 0.092113815 | 0.304063214 | NS | 1.035675233 |
| NPTXR | 0.322399046 | 0.186370605 | 0.41588454 | NS | 0.729622584 |
| CBX6 | 0.173372832 | 0.012612053 | 0.135545097 | Up | 1.899214203 |
| APOBEC3A | 0.089930942 | 0.447992885 | 0.641635461 | NS | 0.348728884 |
| APOBEC3B | -0.104218622 | 0.788044545 | 0.877811098 | NS | 0.103449233 |
| APOBEC3C | -0.011839846 | 0.940188756 | 0.966870359 | NS | 0.026784947 |
| APOBEC3D | 0.155645686 | 0.261049343 | 0.487090765 | NS | 0.583277396 |
| APOBEC3F | 0.179856669 | 0.106485422 | 0.322999226 | NS | 0.972709845 |
| APOBEC3G | -0.203184027 | 0.017536612 | 0.15536692 | Down | 1.756054306 |
| APOBEC3H | -0.137765937 | 0.280989301 | 0.504167626 | NS | 0.551310215 |
| CBX7 | 0.033042266 | 0.525345522 | 0.700562661 | NS | 0.279554966 |
| PDGFB | 0.096259823 | 0.395320249 | 0.600786059 | NS | 0.40305094 |
| RPL3 | -0.14198794 | 0.245747308 | 0.474025507 | NS | 0.609511232 |
| SYNGR1 | 0.382396756 | 5.66E-04 | 0.040211156 | Up | 3.247372247 |
| TAB1 | 0.033142476 | 0.67912011 | 0.808796118 | NS | 0.168053409 |
| MGAT3 | -0.091211421 | 0.490253427 | 0.674680296 | NS | 0.309579361 |
| MIEF1 | 0.122905427 | 0.008724243 | 0.118782974 | Up | 2.059272225 |
| ATF4 | -0.063881278 | 0.264253417 | 0.49043207 | NS | 0.577979389 |
| RPS19BP1 | -0.104897345 | 0.158883527 | 0.386607578 | NS | 0.798921129 |
| CACNA1I | 0.19190684 | 0.17895125 | 0.408934497 | NS | 0.747265264 |
| ENTHD1 | 0.185976302 | 0.352584799 | 0.566142961 | NS | 0.452736416 |
| GRAP2 | -0.109011757 | 0.106217985 | 0.322498249 | NS | 0.973801942 |
| FAM83F | 0.207294902 | 0.412757429 | 0.614932835 | NS | 0.384305101 |
| TNRC6B | 0.060343114 | 0.46215015 | 0.652999544 | NS | 0.335216901 |
| ADSL | -0.074353385 | 0.120928118 | 0.341491462 | NS | 0.917472706 |
| SGSM3 | 0.065271832 | 0.538757929 | 0.709653107 | NS | 0.268606325 |
| MRTFA | 0.058781372 | 0.604615773 | 0.75753107 | NS | 0.218520527 |
| MCHR1 | 0.36961617 | 0.271222277 | NA | NS | 0.566674642 |
| SLC25A17 | -0.015250597 | 0.754329097 | 0.856204836 | NS | 0.12243914 |
| ST13 | -0.179419216 | 0.278080865 | 0.502375007 | NS | 0.555828894 |
| XPNPEP3 | 0.073256373 | 0.197523134 | 0.427410856 | NS | 0.704382033 |
| DNAJB7 | 0.259158205 | 0.084744141 | 0.29286409 | NS | 1.071890317 |
| RBX1 | -0.201484918 | 0.156435395 | 0.383636723 | NS | 0.805664976 |
| EP300 | -0.034690771 | 0.77068493 | 0.86621231 | NS | 0.113123133 |
| L3MBTL2 | -0.102958003 | 0.058743653 | 0.253109387 | NS | 1.231039053 |
| CHADL | 0.819228157 | 0.001954327 | NA | Up | 2.709002723 |
| RANGAP1 | -0.13053112 | 0.094270235 | 0.307415197 | NS | 1.025625409 |
| ZC3H7B | 0.024577487 | 0.762268966 | 0.86145985 | NS | 0.117891761 |
| TEF | -0.173554948 | 0.025136258 | 0.179224966 | Down | 1.599699366 |
| TOB2 | 0.103414044 | 0.249117756 | 0.476376043 | NS | 0.603595317 |
| ACO2 | 0.012903963 | 0.862955378 | 0.922704331 | NS | 0.06401166 |
| PHF5A | -0.096098712 | 0.443552954 | 0.637880676 | NS | 0.353054524 |
| POLR3H | -0.040971048 | 0.51789446 | 0.69521796 | NS | 0.285758735 |
| CSDC2 | 0.502388513 | 0.062106242 | 0.258798607 | NS | 1.206864752 |
| PMM1 | 0.076864924 | 0.253613012 | 0.48049679 | NS | 0.595828469 |
| DESI1 | 0.090556259 | 0.210228163 | 0.440176028 | NS | 0.677309105 |
| XRCC6 | -0.274879991 | 0.002875533 | 0.080409563 | Down | 2.541281599 |
| SNU13 | -0.127704491 | 0.112039166 | 0.330785464 | NS | 0.950630134 |
| MEI1 | 0.032776276 | 0.634266494 | 0.776956101 | NS | 0.19772823 |
| CCDC134 | 0.224855679 | 0.008676415 | 0.118708759 | Up | 2.061659705 |
| SREBF2 | 0.215390506 | 0.059018012 | 0.253419931 | NS | 1.229015424 |
| SHISA8 | 0.527239803 | 0.123074961 | NA | NS | 0.909830294 |
| TNFRSF13C | 0.159351551 | 0.298374254 | 0.519924459 | NS | 0.525238654 |
| CENPM | 0.36238489 | 0.032127971 | 0.198223945 | Up | 1.493116698 |
| LINC00634 | 1.324052997 | 0.027792422 | NA | Up | 1.556073606 |
| SEPTIN3 | 0.215128201 | 0.600987111 | 0.75493123 | NS | 0.221134842 |
| WBP2NL | 0.171746327 | 0.12959795 | 0.351639928 | NS | 0.887401869 |
| NAGA | -0.124279148 | 0.209198113 | 0.439145985 | NS | 0.679442237 |
| PHETA2 | 0.005217203 | 0.973159003 | 0.984855832 | NS | 0.011816195 |
| SMDT1 | -0.015127935 | 0.899003885 | 0.943886076 | NS | 0.046238432 |
| NDUFA6 | -0.288685535 | 0.097047516 | 0.311286025 | NS | 1.013015574 |
| CYP2D6 | 0.055968394 | 0.828997678 | NA | NS | 0.081446686 |
| TCF20 | 0.051037182 | 0.394765299 | 0.600481589 | NS | 0.40366103 |
| NFAM1 | 0.200149143 | 0.094733331 | 0.308004364 | NS | 1.023497193 |
| RRP7A | -0.279474701 | 0.00225831 | 0.07354844 | Down | 2.646216375 |
| SERHL2 | 0.089227 | 0.499122862 | 0.680816664 | NS | 0.301792537 |
| POLDIP3 | 0.25366967 | 0.039589195 | 0.215363569 | Up | 1.402423328 |
| CYB5R3 | 0.041555323 | 0.806405791 | 0.888578902 | NS | 0.093446362 |
| ATP5MGL | 0.203907755 | 0.232609057 | NA | NS | 0.633373379 |
| A4GALT | 0.267310811 | 0.507436424 | 0.687285409 | NS | 0.294618362 |
| ARFGAP3 | -0.07069119 | 0.285698528 | 0.508831767 | NS | 0.544091997 |
| PACSIN2 | -0.125924006 | 0.139076926 | 0.363310112 | NS | 0.856744918 |
| TTLL1 | -0.27132318 | 0.005847522 | 0.102935441 | Down | 2.23302816 |
| BIK | 0.188170839 | 0.136679439 | 0.361144434 | NS | 0.864296813 |
| MCAT | 6.82E-04 | 0.992718398 | 0.995749068 | NS | 0.003173929 |
| TSPO | 0.030936315 | 0.767882851 | 0.864743324 | NS | 0.114705031 |
| TTLL12 | -0.072660955 | 0.308529681 | 0.529171648 | NS | 0.510703049 |
| SCUBE1 | 0.643529556 | 0.068078652 | 0.26826175 | NS | 1.166989052 |
| MPPED1 | 0.709952531 | 0.070450833 | NA | NS | 1.152113867 |
| EFCAB6 | 0.437087376 | 0.086695709 | 0.295990626 | NS | 1.0620024 |
| SULT4A1 | 0.960150013 | 0.06219642 | NA | NS | 1.206234614 |
| PNPLA5 | 0.976464038 | 0.067613133 | NA | NS | 1.169968937 |
| PNPLA3 | 0.53960488 | 0.177965683 | NA | NS | 0.749663734 |
| SAMM50 | -0.080355262 | 0.134905391 | 0.35858085 | NS | 0.869970695 |
| PARVB | 0.386153549 | 8.45E-04 | 0.048398623 | Up | 3.072980417 |
| PARVG | -0.097336594 | 0.084887769 | 0.293013352 | NS | 1.07115488 |
| SHISAL1 | 0.581035614 | 0.216075165 | NA | NS | 0.665395146 |
| RTL6 | 0.092632683 | 0.136520916 | 0.361003586 | NS | 0.864800805 |
| PRR5 | -0.021752998 | 0.875940416 | 0.930333785 | NS | 0.057525435 |
| PRR5-ARHGAP8 | 0.594306347 | 0.313081091 | NA | NS | 0.504343161 |
| ARHGAP8 | 0.286885327 | 0.286203809 | NA | NS | 0.54332459 |
| PHF21B | 0.773736507 | 0.070774294 | 0.27171348 | NS | 1.150124454 |
| NUP50 | 0.00316274 | 0.95088671 | 0.973413528 | NS | 0.021871222 |
| KIAA0930 | 0.230627308 | 0.012261919 | 0.134364821 | Up | 1.911441546 |
| UPK3A | -0.172312729 | 0.338379577 | 0.554290685 | NS | 0.470595856 |
| FAM118A | -0.102795621 | 0.48129982 | 0.668165635 | NS | 0.3175843 |
| SMC1B | 0.103089324 | 0.542933038 | 0.712891437 | NS | 0.26525373 |
| RIBC2 | 0.875603809 | 0.004395714 | NA | Up | 2.356970556 |
| FBLN1 | 0.72492651 | 0.065943904 | 0.265129038 | NS | 1.180825347 |
| ATXN10 | -0.048699286 | 0.478701224 | 0.666135874 | NS | 0.319935462 |
| WNT7B | 1.258715983 | 0.004322297 | NA | Up | 2.364285384 |
| PPARA | 0.138080721 | 0.0372581 | 0.209852065 | Up | 1.428779295 |
| CDPF1 | -0.033022992 | 0.616832024 | 0.765715595 | NS | 0.209833087 |
| PKDREJ | 0.264814975 | 0.02770421 | 0.186639939 | Up | 1.557454226 |
| TTC38 | -0.19792136 | 0.027877648 | 0.187065309 | Down | 1.554743867 |
| GTSE1 | 0.384342328 | 0.04409735 | 0.224627854 | Up | 1.355587513 |
| TRMU | -0.072578585 | 0.108992253 | 0.327029744 | NS | 0.96260437 |
| CELSR1 | 0.173852369 | 0.238306037 | 0.466428925 | NS | 0.622864956 |
| GRAMD4 | -0.221805251 | 0.011944331 | 0.133830267 | Down | 1.922838174 |
| CERK | -0.120645344 | 0.004659702 | 0.09505087 | Down | 2.331641851 |
| TBC1D22A | -0.122857378 | 0.087943781 | 0.297765891 | NS | 1.055794864 |
| TAFA5 | 0.946079268 | 0.017771577 | NA | Up | 1.750274042 |
| BRD1 | 0.024066638 | 0.706721464 | 0.826541465 | NS | 0.150751718 |
| ZBED4 | 0.037018608 | 0.540704903 | 0.710921282 | NS | 0.267039692 |
| ALG12 | 0.043533364 | 0.478492333 | 0.666071166 | NS | 0.320125017 |
| CRELD2 | -0.159896909 | 0.029992861 | 0.193002671 | Down | 1.522982104 |
| PIM3 | 0.032960541 | 0.728175596 | 0.839178888 | NS | 0.13776388 |
| IL17REL | 0.512868258 | 0.066678951 | 0.266279415 | NS | 1.176011238 |
| TTLL8 | 1.037631694 | 0.033785121 | NA | Up | 1.471274516 |
| MLC1 | -0.119718227 | 0.342292615 | 0.55755231 | NS | 0.46560247 |
| MOV10L1 | 0.392141617 | 0.281795262 | 0.504863889 | NS | 0.550066313 |
| PANX2 | 0.046134253 | 0.831338675 | 0.904137066 | NS | 0.080222015 |
| TRABD | 0.143564095 | 0.219861327 | 0.449178818 | NS | 0.657851155 |
| SELENOO | 0.01701126 | 0.816868893 | 0.895844719 | NS | 0.087847642 |
| TUBGCP6 | -0.086898024 | 0.257684463 | 0.484682126 | NS | 0.588911767 |
| HDAC10 | 0.110606609 | 0.339157049 | 0.554994713 | NS | 0.469599152 |
| MAPK12 | 0.221497108 | 0.104338298 | 0.320354348 | NS | 0.981556253 |
| MAPK11 | -0.223828085 | 0.040981807 | 0.218105862 | Down | 1.387408896 |
| PLXNB2 | 0.101133886 | 0.528057312 | 0.702828416 | NS | 0.27731894 |
| DENND6B | 0.453018782 | 0.00265471 | 0.078469363 | Up | 2.575982944 |
| PPP6R2 | -0.133092144 | 0.099801471 | 0.314828276 | NS | 1.000863058 |
| SBF1 | 0.028351109 | 0.7730022 | 0.867838133 | NS | 0.11181927 |
| ADM2 | 0.347668255 | 0.197875022 | 0.427645311 | NS | 0.703609023 |
| MIOX | 0.465570811 | 0.221824935 | NA | NS | 0.653989636 |
| LMF2 | -0.036159131 | 0.656226237 | 0.793542071 | NS | 0.18294641 |
| NCAPH2 | -0.169940456 | 0.007446786 | 0.112720615 | Down | 2.128031132 |
| SCO2 | 0.516108051 | 0.0683263 | 0.268476763 | NS | 1.165412094 |
| TYMP | -0.019725806 | 0.856509642 | 0.919245692 | NS | 0.067267743 |
| ODF3B | 0.22554749 | 0.073001864 | 0.274846078 | NS | 1.136666049 |
| KLHDC7B | 0.519770619 | 2.12E-04 | 0.023858165 | Up | 3.673482572 |
| SYCE3 | 0.184163059 | 0.657987226 | NA | NS | 0.181782538 |
| CPT1B | 0.058966572 | 0.703546565 | 0.824428133 | NS | 0.152707153 |
| CHKB-CPT1B | -0.240407543 | 0.192249274 | 0.42102173 | NS | 0.716135291 |
| CHKB | 0.227850235 | 0.078783122 | 0.283635871 | NS | 1.103566812 |
| MAPK8IP2 | 0.775085278 | 0.031361623 | 0.196726942 | Up | 1.503601476 |
| ARSA | 0.029558719 | 0.831955471 | 0.904571606 | NS | 0.079899918 |
| SHANK3 | 0.80141907 | 0.163307001 | NA | NS | 0.786995197 |
| RABL2B | 0.04258353 | 0.707863249 | 0.82706671 | NS | 0.150050635 |
| XG | 0.077858273 | 0.764391869 | 0.862964734 | NS | 0.116683941 |
| GYG2 | 0.14477055 | 0.727129165 | NA | NS | 0.138388435 |
| ARSD | -0.140297988 | 0.099605774 | 0.314750917 | NS | 1.001715487 |
| ARSL | 0.328937389 | 0.390813183 | NA | NS | 0.408030795 |
| ARSH | 0.41766664 | 0.320196179 | NA | NS | 0.494583855 |
| ARSF | 0.428683279 | 0.3813767 | NA | NS | 0.418645843 |
| MXRA5 | 0.293408653 | 0.479166252 | 0.666456062 | NS | 0.319513777 |
| PRKX | -0.155509299 | 0.015285268 | 0.147395039 | Down | 1.81572693 |
| NLGN4X | 0.384025197 | 0.409784322 | 0.612870146 | NS | 0.387444661 |
| VCX3A | 0.98394059 | 0.072193919 | NA | NS | 1.141499383 |
| PUDP | 0.049875296 | 0.404751277 | 0.60860084 | NS | 0.392811772 |
| STS | 0.052463666 | 0.572007103 | 0.733899534 | NS | 0.242598578 |
| VCX | 0.325042303 | 0.570506327 | NA | NS | 0.243739535 |
| PNPLA4 | -0.044842514 | 0.625254445 | 0.771404261 | NS | 0.203943212 |
| VCX3B | 1.44672571 | 0.012177006 | NA | Up | 1.914459466 |
| ANOS1 | 0.461698696 | 0.081041462 | 0.287057145 | NS | 1.091292731 |
| FAM9A | 0.681087033 | 0.223743656 | NA | NS | 0.65024927 |
| FAM9B | 0.130245772 | 0.698032273 | 0.821159601 | NS | 0.156124498 |
| TBL1X | 0.109883727 | 0.278921869 | 0.503208948 | NS | 0.554517434 |
| GPR143 | 0.114857416 | 0.471440534 | 0.66040315 | NS | 0.32657308 |
| SHROOM2 | 0.356492502 | 0.33332382 | 0.550016309 | NS | 0.47713365 |
| CLDN34 | 0.808883162 | 0.274886237 | NA | NS | 0.560847004 |
| WWC3 | 0.019270037 | 0.882065591 | 0.93320019 | NS | 0.054499119 |
| CLCN4 | 0.060604917 | 0.529661089 | 0.704071643 | NS | 0.276001931 |
| MID1 | 0.093736678 | 0.759172197 | 0.859401989 | NS | 0.119659706 |
| HCCS | -0.155803187 | 0.004606542 | 0.094750689 | Down | 2.336624931 |
| ARHGAP6 | -0.033388054 | 0.774049137 | 0.868209356 | NS | 0.111231469 |
| AMELX | 0.623610068 | 0.128929341 | NA | NS | 0.889648238 |
| MSL3 | 0.160799387 | 0.005151428 | 0.0985614 | Up | 2.288072398 |
| FRMPD4 | 0.074164972 | 0.860162869 | 0.921008463 | NS | 0.065419309 |
| PRPS2 | -0.061222286 | 0.432577295 | 0.629218386 | NS | 0.36393628 |
| TLR7 | 0.14745485 | 0.116314638 | 0.335809937 | NS | 0.934365627 |
| TLR8 | 0.082203355 | 0.530404931 | 0.704519993 | NS | 0.275392447 |
| TMSB4X | -0.162322866 | 0.082796789 | 0.290110921 | NS | 1.081986508 |
| FAM9C | 0.521456002 | 0.047404998 | 0.231338008 | Up | 1.324175865 |
| ATXN3L | 0.132855786 | 0.861122465 | NA | NS | 0.064935081 |
| EGFL6 | 0.308454169 | 0.46598841 | NA | NS | 0.331624885 |
| TCEANC | 0.019494084 | 0.862262109 | 0.922350622 | NS | 0.064360698 |
| RAB9A | -0.200072227 | 0.007434931 | 0.112720615 | Down | 2.128723046 |
| TRAPPC2 | -0.045417654 | 0.376847558 | 0.586981039 | NS | 0.423834295 |
| OFD1 | -0.079914677 | 0.151911784 | 0.37849039 | NS | 0.818408537 |
| GPM6B | -0.024533877 | 0.77650737 | 0.869932305 | NS | 0.109854418 |
| GEMIN8 | 0.070581077 | 0.288644375 | 0.511830295 | NS | 0.539636901 |
| GLRA2 | 0.365826824 | 0.31328359 | NA | NS | 0.504062353 |
| FANCB | -0.136869285 | 0.143051303 | 0.367983932 | NS | 0.844508182 |
| MOSPD2 | 0.138652474 | 0.162627062 | 0.390243605 | NS | 0.788807184 |
| ASB9 | 0.134905651 | 0.571825098 | NA | NS | 0.242736787 |
| ASB11 | 0.517996958 | 0.238588832 | NA | NS | 0.62234989 |
| PIGA | -0.044988432 | 0.363481478 | 0.575717089 | NS | 0.439517714 |
| VEGFD | 0.548506224 | 0.283748842 | NA | NS | 0.547065902 |
| PIR | 0.215610123 | 0.519271948 | NA | NS | 0.284605138 |
| BMX | 0.238008698 | 0.257930187 | 0.484730205 | NS | 0.588497828 |
| ACE2 | 0.490361519 | 0.035625109 | 0.206396722 | Up | 1.448243801 |
| CLTRN | 0.608313348 | 0.045459171 | NA | Up | 1.342378489 |
| CA5B | -0.031429512 | 0.597556043 | 0.752390373 | NS | 0.223621357 |
| ZRSR2 | 0.021919531 | 0.656930739 | 0.79397469 | NS | 0.182480416 |
| AP1S2 | -0.232405185 | 0.071954009 | 0.273508834 | NS | 1.142945001 |
| GRPR | 0.287989412 | 0.269484424 | NA | NS | 0.569466332 |
| MAGEB17 | 0.582722344 | 0.196408266 | NA | NS | 0.706840237 |
| CTPS2 | -0.085034548 | 0.168465322 | 0.396682192 | NS | 0.773489483 |
| S100G | 0.156607734 | 0.769285679 | NA | NS | 0.113912352 |
| SYAP1 | -0.174783613 | 0.001994478 | 0.070057586 | Down | 2.700170649 |
| TXLNG | -0.174891891 | 0.009382112 | 0.122151764 | Down | 2.027699408 |
| RBBP7 | -0.174397362 | 0.00474199 | 0.095617668 | Down | 2.324039363 |
| REPS2 | 0.196064171 | 0.166745955 | 0.394802555 | NS | 0.777944692 |
| NHS | 0.028640418 | 0.800966985 | 0.885420215 | NS | 0.096385385 |
| SCML1 | 0.146847899 | 0.217462662 | 0.447107449 | NS | 0.662615301 |
| RAI2 | 0.319193818 | 0.421338557 | NA | NS | 0.375368796 |
| BEND2 | -0.016559559 | 0.91153744 | 0.950985353 | NS | 0.040225488 |
| SCML2 | 0.00384795 | 0.976436819 | 0.986397233 | NS | 0.010355853 |
| CDKL5 | 0.107241881 | 0.259203948 | 0.485568555 | NS | 0.586358388 |
| RS1 | 0.531007535 | 0.130985741 | NA | NS | 0.882775977 |
| PPEF1 | 0.188209915 | 0.334138976 | 0.55076549 | NS | 0.476072863 |
| PHKA2 | 0.104885193 | 0.033320648 | 0.201295037 | Up | 1.477286559 |
| ADGRG2 | 0.274802327 | 0.063839288 | 0.262358156 | NS | 1.194911966 |
| PDHA1 | -0.075814418 | 0.075101195 | 0.278122419 | NS | 1.124353151 |
| MAP3K15 | 0.24486922 | 0.29001239 | 0.512990599 | NS | 0.537583447 |
| SH3KBP1 | 0.122786128 | 0.02264832 | 0.171563006 | Up | 1.644964001 |
| BCLAF3 | 0.036638612 | 0.487267789 | 0.672268351 | NS | 0.312232297 |
| MAP7D2 | -0.218767336 | 0.330877592 | 0.548001056 | NS | 0.480332644 |
| EIF1AX | -0.119740155 | 0.208861504 | 0.438906623 | NS | 0.680141599 |
| RPS6KA3 | 0.016753125 | 0.760382924 | 0.860134828 | NS | 0.118967645 |
| CNKSR2 | 0.126773457 | 0.261934286 | 0.488145416 | NS | 0.581807651 |
| KLHL34 | 0.45136542 | 0.028277 | 0.188091738 | Up | 1.548566669 |
| SMPX | -0.046035697 | 0.939855398 | NA | NS | 0.02693896 |
| MBTPS2 | 0.151754798 | 0.016018849 | 0.149977606 | Up | 1.7953687 |
| YY2 | -0.037681504 | 0.792795618 | 0.880533717 | NS | 0.100838759 |
| SMS | -0.196700285 | 0.012413213 | 0.13511759 | Down | 1.906115783 |
| PHEX | 0.06332877 | 0.67816386 | 0.808038537 | NS | 0.168665358 |
| CBLL2 | 0.379384765 | 0.450601401 | NA | NS | 0.346207462 |
| DDX53 | 0.660971206 | 0.170760644 | NA | NS | 0.767612217 |
| PTCHD1 | 0.497266971 | 0.135633623 | 0.3596977 | NS | 0.867632637 |
| PRDX4 | -0.175393767 | 0.113183962 | 0.332112632 | NS | 0.946215109 |
| ACOT9 | -0.061238116 | 0.338466504 | 0.554316353 | NS | 0.470484304 |
| SAT1 | 0.041981348 | 0.627079511 | 0.772725276 | NS | 0.202677389 |
| APOO | -0.19677761 | 0.14774154 | 0.373669436 | NS | 0.830497379 |
| CXorf58 | 0.4561263 | 0.005002676 | 0.097847413 | Up | 2.300797641 |
| KLHL15 | -0.109272865 | 0.04177508 | 0.220293088 | Down | 1.37908271 |
| EIF2S3 | -0.282154946 | 0.001536195 | 0.062836479 | Down | 2.813553704 |
| ZFX | -0.048762557 | 0.40489051 | 0.60860084 | NS | 0.392662402 |
| SUPT20HL2 | 0.373352194 | 0.225877449 | 0.454435762 | NS | 0.646127126 |
| SUPT20HL1 | 0.517750846 | 0.157354987 | NA | NS | 0.803119487 |
| PDK3 | 0.086618314 | 0.270542881 | 0.495915885 | NS | 0.567763889 |
| PCYT1B | 0.279641189 | 0.129765556 | 0.351821639 | NS | 0.886840568 |
| POLA1 | -0.076056847 | 0.413576011 | 0.615486243 | NS | 0.38344466 |
| ARX | 0.853713988 | 0.05850926 | NA | NS | 1.232775397 |
| MAGEB18 | 0.423340148 | 0.472207725 | NA | NS | 0.325866912 |
| MAGEB6B | -0.10352987 | 0.907673386 | NA | NS | 0.042070398 |
| MAGEB6 | 0.174649514 | 0.774135774 | NA | NS | 0.111182862 |
| MAGEB5 | 0.913001817 | 0.139811344 | NA | NS | 0.854457588 |
| PPP4R3C | 0.3298747 | 0.563571329 | NA | NS | 0.249051109 |
| DCAF8L2 | 0.535336074 | 0.310410661 | NA | NS | 0.508063371 |
| MAGEB10 | 0.165128845 | 0.785787421 | NA | NS | 0.104694928 |
| DCAF8L1 | -0.033623856 | 0.958583699 | NA | NS | 0.01836996 |
| IL1RAPL1 | 0.46896385 | 0.359972587 | NA | NS | 0.443730571 |
| MAGEB2 | 0.364483314 | 0.568172651 | NA | NS | 0.245519675 |
| MAGEB3 | 0.587373497 | 0.323014338 | NA | NS | 0.4907782 |
| MAGEB4 | 1.176512772 | 0.024822489 | NA | Up | 1.60515467 |
| MAGEB1 | 0.64903127 | 0.322769735 | NA | NS | 0.491107194 |
| NR0B1 | 0.323248593 | 0.54610421 | NA | NS | 0.262724476 |
| TASL | -0.060772147 | 0.537720922 | 0.709095159 | NS | 0.269443065 |
| GK | 0.245910996 | 0.064983185 | 0.264064926 | NS | 1.187199004 |
| TAB3 | 0.047260738 | 0.465064673 | 0.655294755 | NS | 0.332486649 |
| FTHL17 | 1.001924953 | 0.1365117 | NA | NS | 0.864830124 |
| DMD | 0.188341528 | 0.501039497 | 0.682709639 | NS | 0.300128038 |
| FAM47A | 0.149561707 | 0.796423772 | NA | NS | 0.098855785 |
| TMEM47 | 0.642898036 | 0.207387412 | NA | NS | 0.683217608 |
| FAM47B | 0.859021891 | 0.114285648 | NA | NS | 0.942008305 |
| MAGEB16 | 0.507708849 | 0.333835741 | NA | NS | 0.476467169 |
| CFAP47 | 0.383564393 | 0.372204173 | 0.583052983 | NS | 0.429218762 |
| FAM47C | 0.499125785 | 0.350797373 | NA | NS | 0.454943667 |
| PRRG1 | 0.384793495 | 0.11098918 | 0.329580794 | NS | 0.954719358 |
| LANCL3 | 0.047885258 | 0.67201062 | 0.804515798 | NS | 0.172623863 |
| XK | -0.213790286 | 0.318983192 | 0.538089997 | NS | 0.4962322 |
| CYBB | -0.061734306 | 0.557238764 | 0.723076042 | NS | 0.253958679 |
| DYNLT3 | -0.290326263 | 0.022600377 | 0.171548727 | Down | 1.645884319 |
| H2AP | 0.240450023 | 0.791096876 | NA | NS | 0.10177033 |
| H2AL3 | 0.527874309 | 0.325713479 | NA | NS | 0.487164268 |
| SYTL5 | 0.104295553 | 0.742134246 | 0.848841757 | NS | 0.129517527 |
| SRPX | 0.225878374 | 0.587530689 | NA | NS | 0.230969444 |
| RPGR | 0.09627362 | 0.200314813 | 0.429960996 | NS | 0.698286935 |
| OTC | 0.503945922 | 0.318128726 | NA | NS | 0.497397114 |
| TSPAN7 | 0.163950854 | 0.558494699 | 0.724008899 | NS | 0.252980945 |
| MID1IP1 | -0.075841321 | 0.412818876 | 0.614932835 | NS | 0.384240452 |
| BCOR | -0.005335944 | 0.940373747 | 0.966973758 | NS | 0.026699504 |
| ATP6AP2 | -0.274372297 | 2.42E-04 | 0.026067045 | Down | 3.615402013 |
| MPC1L | 0.407934293 | 0.066641236 | NA | NS | 1.176256955 |
| CXorf38 | -0.070957603 | 0.196166244 | 0.425986774 | NS | 0.707375724 |
| MED14 | 0.098344423 | 0.0737195 | 0.275940596 | NS | 1.132417618 |
| USP9X | -0.074095878 | 0.362169785 | 0.574723698 | NS | 0.441087785 |
| DDX3X | -0.179192703 | 0.009261841 | 0.121486068 | Down | 2.033302695 |
| NYX | 0.76206689 | 0.035877541 | NA | Up | 1.445177337 |
| CASK | 0.101810484 | 0.06204723 | 0.258716142 | NS | 1.207277605 |
| GPR34 | 0.04065383 | 0.679236153 | 0.808797872 | NS | 0.167979206 |
| GPR82 | 0.069414063 | 0.432077541 | 0.628973186 | NS | 0.364438308 |
| PPP1R2C | -0.414305379 | 0.545113103 | NA | NS | 0.263513379 |
| MAOA | -0.096642437 | 0.740864451 | 0.848024673 | NS | 0.130261244 |
| MAOB | 0.073133383 | 0.790566402 | 0.878910279 | NS | 0.102061646 |
| NDP | -0.00372982 | 0.994329605 | NA | NS | 0.00246963 |
| EFHC2 | 0.089651765 | 0.347611855 | 0.562229849 | NS | 0.458905421 |
| FUNDC1 | -0.437768236 | 1.66E-04 | 0.0208614 | Down | 3.780458564 |
| DUSP21 | 0.079693313 | 0.854972632 | NA | NS | 0.068047787 |
| KDM6A | 0.120164869 | 0.102760795 | 0.318649084 | NS | 0.988172546 |
| DIPK2B | 0.14677063 | 0.592352128 | 0.749026006 | NS | 0.227420047 |
| KRBOX4 | -0.104495173 | 0.179284158 | 0.409409291 | NS | 0.746458085 |
| ZNF674 | 0.023984514 | 0.694130604 | 0.818419434 | NS | 0.158558807 |
| CHST7 | -0.018974908 | 0.846424404 | 0.913516103 | NS | 0.072411823 |
| SLC9A7 | -0.080677195 | 0.220272945 | 0.449439828 | NS | 0.657038842 |
| RP2 | -0.024785229 | 0.823709499 | 0.899684994 | NS | 0.084225926 |
| JADE3 | -0.025856885 | 0.805848861 | 0.88835382 | NS | 0.093746404 |
| RGN | 0.770400192 | 0.052291667 | NA | NS | 1.281567514 |
| NDUFB11 | -0.186261893 | 0.064988514 | 0.264064926 | NS | 1.187163392 |
| RBM10 | -0.237945312 | 0.003342084 | 0.085084692 | Down | 2.47598267 |
| UBA1 | -0.184769875 | 0.073532653 | 0.275712399 | NS | 1.133519763 |
| CDK16 | -0.039800337 | 0.581809855 | 0.740914664 | NS | 0.235218926 |
| USP11 | -0.008338344 | 0.915131124 | 0.952824741 | NS | 0.038516674 |
| ZNF157 | 0.02129326 | 0.878901492 | 0.931811593 | NS | 0.056059798 |
| ZNF41 | -0.021695025 | 0.596665447 | 0.75175978 | NS | 0.224269111 |
| ARAF | -0.083569253 | 0.46664174 | 0.656560381 | NS | 0.331016417 |
| SYN1 | 0.306250944 | 0.267848566 | 0.493783133 | NS | 0.572110674 |
| TIMP1 | 0.2048481 | 0.038436831 | 0.213464789 | Up | 1.415252431 |
| CFP | -0.120701789 | 0.208209767 | 0.438032344 | NS | 0.681498901 |
| ELK1 | 0.067304529 | 0.290103345 | 0.512990599 | NS | 0.537447263 |
| UXT | -0.205049034 | 0.055988678 | 0.249087638 | NS | 1.251899787 |
| ZNF81 | 0.061862883 | 0.241390742 | 0.469262706 | NS | 0.61727939 |
| ZNF182 | 0.020058431 | 0.671720084 | 0.804303074 | NS | 0.172811667 |
| ZNF630 | 0.097223466 | 0.261897499 | 0.488134281 | NS | 0.58186865 |
| SSX5 | 0.388633932 | 0.432528235 | NA | NS | 0.363985537 |
| SSX1 | 0.136206694 | 0.792133411 | NA | NS | 0.101201669 |
| SSX3 | 0.054348713 | 0.920044432 | NA | NS | 0.036191199 |
| SLC38A5 | 0.041171479 | 0.799739763 | 0.884702387 | NS | 0.09705131 |
| FTSJ1 | -0.036178892 | 0.563880032 | 0.728081439 | NS | 0.248813285 |
| PORCN | 0.058727507 | 0.570242583 | 0.732374139 | NS | 0.243940354 |
| EBP | -0.247188682 | 0.013928166 | 0.141407346 | Down | 1.856106064 |
| TBC1D25 | -0.163119071 | 0.290218013 | 0.513109384 | NS | 0.537275635 |
| RBM3 | -0.334617888 | 0.001514085 | 0.062591307 | Down | 2.819849627 |
| WDR13 | -0.089208735 | 0.262557332 | 0.488752892 | NS | 0.58077585 |
| WAS | -0.083823578 | 0.637528203 | 0.779367508 | NS | 0.195500598 |
| SUV39H1 | -0.169600596 | 0.030947201 | 0.195253594 | Down | 1.509378618 |
| GLOD5 | 0.708696794 | 0.029457551 | NA | Up | 1.530803364 |
| GATA1 | -0.09101731 | 0.59283095 | 0.749346361 | NS | 0.227069131 |
| HDAC6 | -0.140102229 | 0.104246958 | 0.320245132 | NS | 0.98193661 |
| ERAS | 0.697044485 | 0.209866759 | NA | NS | 0.678056345 |
| PCSK1N | -0.206949872 | 0.578608063 | 0.738460241 | NS | 0.237615519 |
| PQBP1 | -0.371308681 | 3.15E-04 | 0.029830842 | Down | 3.501392529 |
| TIMM17B | -0.118911631 | 0.128895264 | 0.350821187 | NS | 0.88976304 |
| SLC35A2 | 0.006334653 | 0.90181063 | 0.9454453 | NS | 0.04488465 |
| PIM2 | 0.198973951 | 0.004124052 | 0.090991368 | Up | 2.384675863 |
| OTUD5 | -0.121896847 | 0.247325389 | 0.475507879 | NS | 0.606731299 |
| KCND1 | 0.167999866 | 0.533264322 | 0.705998557 | NS | 0.273057472 |
| GRIPAP1 | -0.106686285 | 0.254563466 | 0.48140404 | NS | 0.594203925 |
| TFE3 | 0.105839165 | 0.369024164 | 0.580590828 | NS | 0.432945195 |
| CCDC120 | 0.076793225 | 0.68424371 | 0.812198064 | NS | 0.164789186 |
| PRAF2 | 0.335777398 | 0.023266466 | 0.173340472 | Up | 1.633269572 |
| WDR45 | -0.174017053 | 0.144852346 | 0.370039981 | NS | 0.839074467 |
| GPKOW | -0.313670711 | 1.91E-05 | 0.005139728 | Down | 4.718912326 |
| MAGIX | 0.18250083 | 0.184109248 | 0.414354362 | NS | 0.734924396 |
| PLP2 | 0.113302063 | 0.221654116 | 0.450507861 | NS | 0.654324199 |
| PRICKLE3 | 0.230913311 | 0.064189378 | 0.262840183 | NS | 1.19253683 |
| SYP | 0.394976347 | 0.044171696 | 0.224854455 | Up | 1.354855921 |
| CACNA1F | 0.175994396 | 0.471306686 | 0.660256039 | NS | 0.326696399 |
| CCDC22 | -0.171367617 | 0.042400775 | 0.220932874 | Down | 1.372626209 |
| FOXP3 | 0.206781961 | 0.324698546 | 0.543227855 | NS | 0.488519656 |
| PPP1R3F | 0.094842151 | 0.245869001 | 0.474098126 | NS | 0.609296224 |
| GAGE10 | 0.356480104 | 0.293114706 | NA | NS | 0.532962392 |
| GAGE12J | 0.542726828 | 0.452417456 | NA | NS | 0.344460647 |
| GAGE2E | -0.148299567 | 0.843749021 | NA | NS | 0.073786718 |
| GAGE2A | 0.437721273 | 0.562924852 | NA | NS | 0.249549577 |
| GAGE1 | 0.948676104 | 0.10062247 | NA | NS | 0.997305027 |
| PAGE1 | 0.556560093 | 0.343588105 | NA | NS | 0.46396188 |
| PAGE4 | 0.157490575 | 0.745367476 | NA | NS | 0.127629562 |
| USP27X | -0.193021648 | 0.00544425 | 0.100470755 | Down | 2.264061964 |
| CLCN5 | 0.041924615 | 0.589923893 | 0.747465395 | NS | 0.229204013 |
| AKAP4 | 0.768585246 | 0.15786365 | NA | NS | 0.80171786 |
| CCNB3 | 0.250219782 | 0.250214669 | 0.477256296 | NS | 0.601687234 |
| DGKK | 0.309382351 | 0.156953727 | 0.384159619 | NS | 0.804228366 |
| SHROOM4 | 0.232172906 | 0.2820608 | 0.505014315 | NS | 0.549657267 |
| BMP15 | 0.400502595 | 0.519682478 | NA | NS | 0.284261926 |
| NUDT10 | 0.32723216 | 0.057359796 | 0.251032686 | NS | 1.241392403 |
| EZHIP | 0.640308648 | 0.04454747 | NA | Up | 1.35117696 |
| NUDT11 | -0.031660803 | 0.856933264 | 0.919484895 | NS | 0.067052999 |
| CENPVL3 | 0.98890835 | 0.052061254 | NA | NS | 1.283485374 |
| GSPT2 | -0.152435483 | 0.126343522 | 0.348239179 | NS | 0.898447022 |
| MAGED1 | 0.155144132 | 0.060362758 | 0.256205353 | NS | 1.219230928 |
| XAGE2 | 0.576162044 | 0.334374713 | NA | NS | 0.475766574 |
| SSX7 | 0.496866378 | 0.333117572 | NA | NS | 0.477402458 |
| SSX2 | 0.539642774 | 0.476763747 | NA | NS | 0.321696776 |
| SPANXN5 | 0.488191461 | 0.435011349 | NA | NS | 0.361499412 |
| XAGE5 | 0.352751132 | 0.521806149 | NA | NS | 0.282490808 |
| XAGE3 | -0.180408562 | 0.773763206 | NA | NS | 0.111391926 |
| FAM156A | 0.008744213 | 0.960653826 | NA | NS | 0.017433083 |
| GPR173 | 0.333237346 | 0.316494639 | 0.536042023 | NS | 0.499633641 |
| TSPYL2 | -0.073004731 | 0.403962824 | 0.607775364 | NS | 0.393658601 |
| KANTR | 0.051479504 | 0.554419913 | 0.72131755 | NS | 0.25616118 |
| KDM5C | -0.065993389 | 0.400452299 | 0.605341375 | NS | 0.397449208 |
| IQSEC2 | 0.319726415 | 0.068213101 | 0.268476763 | NS | 1.166132208 |
| SMC1A | -0.051951949 | 0.57732535 | 0.737788358 | NS | 0.238579373 |
| RIBC1 | 0.127629464 | 0.488883237 | 0.673467903 | NS | 0.310794854 |
| HSD17B10 | -0.271376706 | 0.004049003 | 0.090453197 | Down | 2.392651949 |
| HUWE1 | -0.070540676 | 0.518713832 | 0.695808062 | NS | 0.285072171 |
| PHF8 | 0.01037947 | 0.877895758 | 0.93141706 | NS | 0.056557049 |
| FAM120C | 0.090076389 | 0.174790183 | 0.40418465 | NS | 0.757482963 |
| WNK3 | 0.230292642 | 0.114944748 | 0.333658762 | NS | 0.939510869 |
| TSR2 | -0.244163808 | 5.84E-04 | 0.040600896 | Down | 3.23342334 |
| FGD1 | 0.367226563 | 0.203040198 | 0.432986484 | NS | 0.692417971 |
| GNL3L | -0.14708119 | 0.117566994 | 0.337535326 | NS | 0.929714586 |
| ITIH6 | 0.218966343 | 0.608778822 | 0.76003769 | NS | 0.215540464 |
| MAGED2 | -0.191018024 | 0.015450398 | 0.147928516 | Down | 1.811060335 |
| TRO | 0.201489431 | 0.4081501 | 0.61130427 | NS | 0.389180093 |
| PFKFB1 | 0.020278466 | 0.910780246 | 0.950585116 | NS | 0.040586397 |
| APEX2 | 0.068478789 | 0.233251455 | 0.461545259 | NS | 0.632175638 |
| ALAS2 | 0.16001621 | 0.479396668 | 0.666617505 | NS | 0.319304989 |
| PAGE2B | -0.719498308 | 0.009575147 | 0.122920416 | Down | 2.018854541 |
| PAGE2 | -0.056206086 | 0.873792922 | NA | NS | 0.058591477 |
| FAM104B | -0.357817591 | 0.026960007 | 0.185246086 | Down | 1.56928 |
| MTRNR2L10 | 1.354999348 | 0.107941851 | NA | NS | 0.966810139 |
| PAGE5 | -0.307426187 | 0.536628113 | NA | NS | 0.270326579 |
| PAGE3 | 0.290832469 | 0.635974997 | NA | NS | 0.196559958 |
| MAGEH1 | -0.288393636 | 0.018419978 | 0.157584735 | Down | 1.734710893 |
| USP51 | -0.011429637 | 0.92544972 | 0.959164117 | NS | 0.033647172 |
| FOXR2 | 0.891948349 | 0.087811289 | NA | NS | 1.056449647 |
| RRAGB | -0.072298786 | 0.100099797 | 0.315204109 | NS | 0.999566804 |
| KLF8 | 0.169754297 | 0.14046665 | 0.364826818 | NS | 0.852426776 |
| UBQLN2 | 0.166043564 | 0.00577473 | 0.102521601 | Up | 2.238468319 |
| NBDY | 0.084921064 | 0.302872245 | 0.524291773 | NS | 0.518740523 |
| SPIN3 | 0.00148553 | 0.983550981 | 0.990165923 | NS | 0.007203124 |
| SPIN2B | 0.072681025 | 0.426139021 | 0.624284262 | NS | 0.370448696 |
| SPIN2A | -0.270877957 | 0.426948704 | 0.625067227 | NS | 0.3696243 |
| FAAH2 | -0.072198358 | 0.464556415 | 0.654918093 | NS | 0.332961538 |
| ZXDB | 0.20420104 | 0.002553963 | 0.077575819 | Up | 2.592785346 |
| NLRP2B | 0.608398873 | 0.128661206 | NA | NS | 0.890552381 |
| ZXDA | 0.015381004 | 0.816282281 | 0.895330307 | NS | 0.08815963 |
| SPIN4 | 0.002352418 | 0.974505634 | 0.985403414 | NS | 0.011215646 |
| ARHGEF9 | 0.008872226 | 0.878519455 | 0.931684267 | NS | 0.056248616 |
| AMER1 | 0.078201278 | 0.275635586 | 0.500246153 | NS | 0.559664713 |
| ASB12 | 0.315079277 | 0.468779377 | NA | NS | 0.329031503 |
| MTMR8 | 0.01068861 | 0.873395855 | 0.928535506 | NS | 0.058788873 |
| ZC4H2 | -7.49E-04 | 0.993630881 | 0.996240892 | NS | 0.002774919 |
| ZC3H12B | 0.051940243 | 0.538125075 | 0.709180599 | NS | 0.269116771 |
| LAS1L | -0.208754193 | 3.02E-04 | 0.029045993 | Down | 3.519601729 |
| MSN | 0.234931381 | 0.020614644 | 0.166707831 | Up | 1.685824161 |
| VSIG4 | 0.132191278 | 0.295612463 | 0.517469712 | NS | 0.529277259 |
| HEPH | 0.06922177 | 0.775041766 | 0.86894828 | NS | 0.110674893 |
| EDA2R | 0.031318056 | 0.879261921 | 0.931881903 | NS | 0.055881735 |
| AR | 0.012381655 | 0.959887183 | 0.97768896 | NS | 0.017779807 |
| OPHN1 | 0.205826996 | 0.203662744 | 0.433564246 | NS | 0.69108841 |
| YIPF6 | -0.112601472 | 0.341583332 | 0.55696544 | NS | 0.46650333 |
| STARD8 | 0.036781538 | 0.730244806 | 0.840464443 | NS | 0.136531523 |
| EFNB1 | 0.019300823 | 0.858037687 | 0.919794085 | NS | 0.066493637 |
| PJA1 | -0.312394677 | 7.22E-04 | 0.044238055 | Down | 3.141205781 |
| NALF2 | 0.592203941 | 0.178030226 | NA | NS | 0.749506256 |
| EDA | 0.215919018 | 0.191169472 | 0.420492053 | NS | 0.718581459 |
| AWAT2 | 0.045315411 | 0.917631236 | NA | NS | 0.037331811 |
| OTUD6A | 1.08549845 | 0.034889494 | NA | Up | 1.457305327 |
| IGBP1 | -0.204953406 | 0.0371071 | 0.209626738 | Down | 1.430542989 |
| DGAT2L6 | 0.196828584 | 0.641060966 | NA | NS | 0.193100666 |
| AWAT1 | -0.254274561 | 0.447987116 | NA | NS | 0.348734476 |
| P2RY4 | 0.313010419 | 0.304051866 | NA | NS | 0.517052328 |
| ARR3 | 0.2069867 | 0.296396447 | 0.518054753 | NS | 0.528127007 |
| RAB41 | 0.112956205 | 0.555516671 | 0.721922646 | NS | 0.255302904 |
| PDZD11 | -0.038551822 | 0.624025695 | 0.770689413 | NS | 0.204797528 |
| KIF4A | -0.073331752 | 0.696611359 | 0.820162109 | NS | 0.157009448 |
| GDPD2 | 0.545156154 | 0.149329614 | NA | NS | 0.825854057 |
| DLG3 | -0.043872143 | 0.49142598 | 0.675629595 | NS | 0.308541887 |
| TEX11 | 0.430279854 | 0.281160984 | 0.504319303 | NS | 0.551044945 |
| SLC7A3 | 0.119967476 | 0.768737579 | NA | NS | 0.114221888 |
| SNX12 | -0.111301673 | 0.069150602 | 0.269426182 | NS | 1.160204032 |
| FOXO4 | -0.494180613 | 0.003152183 | 0.084307443 | Down | 2.501388623 |
| CXorf65 | 0.088512983 | 0.781175617 | NA | NS | 0.107251321 |
| IL2RG | 0.069766577 | 0.331960248 | 0.548813376 | NS | 0.47891392 |
| MED12 | -0.057783261 | 0.672755919 | 0.805012822 | NS | 0.172142473 |
| NLGN3 | 0.075512146 | 0.593514589 | 0.749772128 | NS | 0.226568601 |
| GJB1 | 0.373825273 | 0.42974909 | NA | NS | 0.366785034 |
| ZMYM3 | 0.001331912 | 0.988851239 | 0.993516162 | NS | 0.004869038 |
| NONO | 0.058477379 | 0.113459852 | 0.332206431 | NS | 0.945157789 |
| ITGB1BP2 | 0.332509209 | 0.039195181 | 0.214561777 | Up | 1.406767329 |
| TAF1 | -0.095296693 | 0.102799183 | 0.318649084 | NS | 0.988010338 |
| OGT | 0.048455736 | 0.284231799 | 0.507087065 | NS | 0.546327337 |
| GCNA | -0.154094109 | 0.052817197 | 0.242846705 | NS | 1.277224653 |
| CXCR3 | 0.32171477 | 0.033064604 | 0.200435564 | Up | 1.480636674 |
| NHSL2 | 0.197031695 | 0.08412645 | 0.292007269 | NS | 1.075067436 |
| RTL5 | 0.16586956 | 0.223741172 | 0.452337751 | NS | 0.650254091 |
| PIN4 | -0.069634366 | 0.497950355 | 0.67986521 | NS | 0.302813953 |
| ERCC6L | 0.132218548 | 0.097282599 | 0.311603648 | NS | 1.011964835 |
| RPS4X | -0.315736097 | 0.009851913 | 0.125018575 | Down | 2.006479441 |
| CITED1 | 0.835702599 | 0.095286614 | NA | NS | 1.020968108 |
| HDAC8 | 0.16807057 | 0.044360428 | 0.225362854 | Up | 1.353004274 |
| PHKA1 | 0.303835074 | 0.330270902 | 0.547441347 | NS | 0.481129687 |
| DMRTC1 | 0.347836345 | 0.628540303 | NA | NS | 0.20166687 |
| PABPC1L2B | 0.799803517 | 0.164600192 | NA | NS | 0.783569663 |
| PABPC1L2A | 1.101319096 | 0.110169949 | NA | NS | 0.957936851 |
| NAP1L2 | -0.26940377 | 0.039043212 | 0.214333278 | Down | 1.408454456 |
| CDX4 | 0.659018744 | 0.190282188 | NA | NS | 0.720601864 |
| CHIC1 | -0.086969389 | 0.206329507 | 0.436307756 | NS | 0.68543866 |
| ZCCHC13 | 0.762382898 | 0.208366644 | NA | NS | 0.681171802 |
| SLC16A2 | 0.218817648 | 0.300832927 | 0.522222199 | NS | 0.521674631 |
| RLIM | 0.011755717 | 0.838784493 | 0.909109618 | NS | 0.076349607 |
| NEXMIF | 0.146059053 | 0.345357404 | 0.560273094 | NS | 0.461731229 |
| ABCB7 | -0.131604764 | 0.021483019 | 0.168494559 | Down | 1.667904679 |
| UPRT | -0.168299891 | 0.006675443 | 0.108657422 | Down | 2.175519916 |
| ZDHHC15 | 0.167707734 | 0.22871072 | 0.457341561 | NS | 0.640713479 |
| MAGEE2 | 0.310709187 | 0.543086436 | NA | NS | 0.265131044 |
| PBDC1 | -0.011720564 | 0.860359109 | 0.921132453 | NS | 0.065320239 |
| MAGEE1 | 0.226312508 | 0.067239234 | 0.267139948 | NS | 1.172377244 |
| FGF16 | 0.295032925 | 0.625438666 | NA | NS | 0.203815273 |
| ATRX | -0.064559706 | 0.229049034 | 0.457671413 | NS | 0.640071535 |
| MAGT1 | -0.190264984 | 0.012302603 | 0.134659948 | Down | 1.910002979 |
| COX7B | -0.378251148 | 0.039758747 | 0.215730166 | Down | 1.400567308 |
| ATP7A | -0.125668467 | 0.140736508 | 0.365211163 | NS | 0.851593229 |
| PGK1 | -0.033267812 | 0.71280028 | 0.830393544 | NS | 0.147032138 |
| PGAM4 | 0.201850418 | 0.311686015 | NA | NS | 0.506282683 |
| TAF9B | 0.019995449 | 0.707592895 | 0.826979137 | NS | 0.150216537 |
| CYSLTR1 | -0.059253844 | 0.497644895 | 0.679732613 | NS | 0.303080447 |
| RTL3 | 0.740658653 | 0.161444309 | NA | NS | 0.79197726 |
| LPAR4 | 0.240556742 | 0.386665096 | 0.594681622 | NS | 0.412665029 |
| P2RY10 | -0.161808583 | 0.101374475 | 0.317114382 | NS | 0.994071382 |
| GPR174 | 0.020123777 | 0.79596368 | 0.882340764 | NS | 0.099106749 |
| ITM2A | -0.206138803 | 0.101570843 | 0.317326231 | NS | 0.993230943 |
| TENT5D | 0.594648286 | 0.209294352 | NA | NS | 0.679242492 |
| BRWD3 | 0.022431747 | 0.75557538 | 0.857024781 | NS | 0.121722202 |
| HMGN5 | 0.028388865 | 0.880991459 | 0.932756987 | NS | 0.055028302 |
| SH3BGRL | -0.219916425 | 0.010862098 | 0.12856202 | Down | 1.964086294 |
| POU3F4 | 0.564906231 | 0.263271353 | NA | NS | 0.579596394 |
| CYLC1 | 0.064484828 | 0.90747471 | NA | NS | 0.042165469 |
| RPS6KA6 | 0.131414275 | 0.660737226 | 0.796222255 | NS | 0.179971224 |
| HDX | -0.011972054 | 0.906763143 | 0.948236123 | NS | 0.042506141 |
| APOOL | -0.129881563 | 0.271975676 | 0.497340928 | NS | 0.565469936 |
| SATL1 | 0.37783115 | 0.342850186 | 0.558038995 | NS | 0.464895611 |
| ZNF711 | -0.219876622 | 0.036032524 | 0.207679449 | Down | 1.443305321 |
| POF1B | -0.058059471 | 0.718544348 | 0.833853695 | NS | 0.143546422 |
| CHM | -0.004393545 | 0.937870616 | 0.965744024 | NS | 0.027857071 |
| DACH2 | 0.073704024 | 0.868316366 | NA | NS | 0.061322013 |
| KLHL4 | 0.094337722 | 0.724855848 | 0.838061435 | NS | 0.139748353 |
| CPXCR1 | 0.425816403 | 0.456781089 | NA | NS | 0.340291884 |
| TGIF2LX | 0.280818687 | 0.655422591 | NA | NS | 0.183478593 |
| PABPC5 | -0.095031698 | 0.850390518 | NA | NS | 0.070381591 |
| PCDH11X | 0.084652438 | 0.860205732 | 0.921011299 | NS | 0.065397668 |
| NAP1L3 | -0.054178192 | 0.67453055 | 0.806015792 | NS | 0.170998376 |
| FAM133A | 0.156056382 | 0.717616389 | NA | NS | 0.144107651 |
| DIAPH2 | -0.082094757 | 0.119010065 | 0.339550081 | NS | 0.924416309 |
| RPA4 | 0.084841888 | 0.456834236 | 0.648429977 | NS | 0.340241357 |
| PCDH19 | 0.633587531 | 0.147035088 | 0.37264175 | NS | 0.832579014 |
| TNMD | 0.32382448 | 0.512232545 | NA | NS | 0.290532832 |
| TSPAN6 | 0.023082216 | 0.915869779 | 0.953377105 | NS | 0.038166271 |
| SRPX2 | 0.593531343 | 0.169285691 | NA | NS | 0.771379749 |
| SYTL4 | -0.068061178 | 0.65506176 | 0.792678031 | NS | 0.183717752 |
| CSTF2 | -0.159659649 | 0.008749356 | 0.118782974 | Down | 2.058023906 |
| NOX1 | 0.530049858 | 0.047219636 | NA | Up | 1.325877364 |
| XKRX | 0.131290881 | 0.372517994 | 0.583264312 | NS | 0.428852744 |
| ARL13A | 0.21603667 | 0.104259353 | 0.320245132 | NS | 0.981884974 |
| TRMT2B | -0.135967047 | 0.019284552 | 0.16124674 | Down | 1.714790435 |
| TMEM35A | 0.239894932 | 0.613537713 | NA | NS | 0.212158737 |
| CENPI | -0.108837252 | 0.471290295 | 0.660256039 | NS | 0.326711503 |
| DRP2 | 0.172004492 | 0.620681362 | 0.767922494 | NS | 0.207131295 |
| TAF7L | 0.204839261 | 0.56055783 | NA | NS | 0.251379577 |
| TIMM8A | 0.011143862 | 0.893282457 | 0.940033765 | NS | 0.049011195 |
| BTK | -0.120931006 | 0.0888991 | 0.299209951 | NS | 1.051102635 |
| RPL36A | -0.039809109 | 0.57308488 | 0.734664983 | NS | 0.24178105 |
| GLA | -0.075085548 | 0.335378775 | 0.551701439 | NS | 0.474464426 |
| HNRNPH2 | -0.070920583 | 0.516736162 | 0.694202442 | NS | 0.286731145 |
| ARMCX4 | 0.104089444 | 0.340536548 | 0.556086414 | NS | 0.46783627 |
| ARMCX1 | -0.321540563 | 0.021867197 | 0.169347779 | Down | 1.66020688 |
| ARMCX6 | -0.19878756 | 0.010239245 | 0.126233693 | Down | 1.989732073 |
| ARMCX3 | -0.13234472 | 0.016806627 | 0.15280086 | Down | 1.774519445 |
| ARMCX2 | -0.265529885 | 0.01271973 | 0.135824642 | Down | 1.895522104 |
| ZMAT1 | 0.025067532 | 0.763763515 | 0.862433673 | NS | 0.117041092 |
| TCEAL2 | 0.224926037 | 0.4297405 | NA | NS | 0.366793716 |
| TCEAL6 | 0.945048059 | 0.083480145 | NA | NS | 1.078416805 |
| BEX5 | -0.176320229 | 0.288306054 | 0.511526592 | NS | 0.540146238 |
| TMSB15A | 0.150817252 | 0.70235999 | NA | NS | 0.153440236 |
| ARMCX5 | -0.139229782 | 0.029818378 | 0.192312163 | Down | 1.525515985 |
| GPRASP1 | -0.092353119 | 0.295961926 | 0.517745305 | NS | 0.528764155 |
| GPRASP2 | -0.255934871 | 0.004701213 | 0.095487183 | Down | 2.327790098 |
| BHLHB9 | 0.352965746 | 0.151242827 | NA | NS | 0.820325215 |
| RAB40AL | -0.256276321 | 0.447556984 | NA | NS | 0.349151661 |
| BEX1 | -0.08153454 | 0.774905477 | NA | NS | 0.110751269 |
| NXF3 | 0.458487825 | 0.287806328 | 0.51095663 | NS | 0.540899661 |
| BEX4 | -0.330808927 | 0.001396742 | 0.060860925 | Down | 2.854883801 |
| TCEAL8 | -0.356130343 | 0.011648929 | 0.132276213 | Down | 1.933714018 |
| TCEAL5 | 0.394313764 | 0.478264546 | NA | NS | 0.320331813 |
| BEX2 | -0.226867174 | 0.211717673 | 0.441352326 | NS | 0.674242887 |
| TCEAL7 | 0.838011313 | 0.168623552 | NA | NS | 0.773081766 |
| TCEAL9 | 0.005148269 | 0.979941272 | 0.988095585 | NS | 0.008799951 |
| BEX3 | -0.10140218 | 0.385747975 | 0.593890885 | NS | 0.413696345 |
| RAB40A | 0.205159858 | 0.479550613 | NA | NS | 0.319165549 |
| TCEAL4 | -0.307513697 | 0.002995608 | 0.082074431 | Down | 2.523515019 |
| TCEAL3 | -0.150325378 | 0.110342793 | 0.328874367 | NS | 0.957256029 |
| TCEAL1 | -0.306758849 | 0.03538283 | 0.205857644 | Down | 1.451207432 |
| MORF4L2 | -0.160640632 | 0.03960839 | 0.215363569 | Down | 1.402212808 |
| TMEM31 | 0.319755378 | 0.520470229 | NA | NS | 0.283604107 |
| PLP1 | 0.4630125 | 0.128230121 | 0.350012309 | NS | 0.892009946 |
| RAB9B | -0.078885251 | 0.45272265 | 0.645353539 | NS | 0.344167777 |
| TMSB15B | 0.233544113 | 0.094766657 | 0.308053683 | NS | 1.023344439 |
| H2BW1 | 0.912503348 | 0.115679061 | NA | NS | 0.936745245 |
| H2BW2 | 1.187958 | 0.064211697 | NA | NS | 1.192385851 |
| SLC25A53 | 0.025712594 | 0.633035879 | 0.776534704 | NS | 0.198571674 |
| ZCCHC18 | -0.184571569 | 0.033697898 | 0.202081501 | Down | 1.472397192 |
| FAM199X | -0.018292788 | 0.721794215 | 0.83580398 | NS | 0.141586603 |
| ESX1 | 1.077240382 | 0.050298121 | NA | NS | 1.298448239 |
| IL1RAPL2 | 0.354690447 | 0.51037555 | NA | NS | 0.292110139 |
| TEX13A | -0.256989962 | 0.646884145 | NA | NS | 0.189173494 |
| NRK | 0.475246707 | 0.280581804 | 0.503778541 | NS | 0.551940497 |
| SERPINA7 | 0.215736871 | 0.698789561 | NA | NS | 0.155653591 |
| PWWP3B | 0.385580924 | 0.474893495 | NA | NS | 0.32340378 |
| RADX | -0.023258761 | 0.727537522 | 0.838765421 | NS | 0.138144604 |
| RNF128 | 0.366429806 | 0.403715221 | NA | NS | 0.393924877 |
| TBC1D8B | 0.080768235 | 0.507250226 | 0.687180693 | NS | 0.29477775 |
| MORC4 | -0.158678752 | 0.030799741 | 0.194908998 | Down | 1.511452939 |
| RIPPLY1 | 0.46532592 | 0.46564713 | NA | NS | 0.33194307 |
| CLDN2 | 0.542128031 | 0.149079564 | NA | NS | 0.826581886 |
| RBM41 | -0.09457399 | 0.202234103 | 0.432437343 | NS | 0.694145608 |
| NUP62CL | -0.087475853 | 0.642000256 | 0.783287511 | NS | 0.192464799 |
| DNAAF6 | -0.374708798 | 0.501922275 | NA | NS | 0.29936353 |
| FRMPD3 | 0.02800076 | 0.86289235 | 0.922704331 | NS | 0.064043381 |
| PRPS1 | -0.104784555 | 0.09743225 | 0.311908496 | NS | 1.01129727 |
| TSC22D3 | 0.258219495 | 0.011372428 | 0.131077679 | Up | 1.944146799 |
| NCBP2L | 0.35143406 | 0.147082648 | NA | NS | 0.83243856 |
| MID2 | -0.014829847 | 0.867906862 | 0.925535619 | NS | 0.061526878 |
| TEX13B | 0.247475506 | 0.66613744 | NA | NS | 0.176436156 |
| VSIG1 | 0.023323783 | 0.783751718 | 0.874944277 | NS | 0.105821494 |
| PSMD10 | -0.233192368 | 0.017620347 | 0.155449116 | Down | 1.753985535 |
| ATG4A | -0.154557415 | 0.036332608 | 0.208236145 | Down | 1.439703429 |
| COL4A6 | 0.240749126 | 0.436142116 | 0.631945257 | NS | 0.360371974 |
| COL4A5 | 0.199115407 | 0.631050222 | 0.775295013 | NS | 0.199936076 |
| IRS4 | 0.408046956 | 0.3342572 | 0.550791033 | NS | 0.475919229 |
| GUCY2F | 0.326817195 | 0.437567888 | 0.633279267 | NS | 0.358954557 |
| NXT2 | -0.289508861 | 0.016455293 | 0.151349041 | Down | 1.783694391 |
| KCNE5 | 0.335110228 | 0.132405185 | NA | NS | 0.878095006 |
| ACSL4 | 0.033166359 | 0.744943625 | 0.850384773 | NS | 0.127876592 |
| TMEM164 | 0.06136532 | 0.551519819 | 0.718943583 | NS | 0.258438877 |
| AMMECR1 | 0.024361209 | 0.670899935 | 0.803720941 | NS | 0.17334225 |
| GNG5P2 | -0.144541409 | 0.715117797 | NA | NS | 0.145622414 |
| RTL9 | 0.318876061 | 0.513140975 | NA | NS | 0.289763305 |
| TDGF1P3 | -0.062938904 | 0.916938427 | NA | NS | 0.037659826 |
| CHRDL1 | 0.437067388 | 0.293771871 | NA | NS | 0.53198979 |
| PAK3 | 0.200277687 | 0.314226377 | 0.53361135 | NS | 0.502757362 |
| CAPN6 | 0.377627607 | 0.418494701 | NA | NS | 0.378310037 |
| DCX | 0.283483566 | 0.529855479 | 0.70413711 | NS | 0.275842571 |
| SERTM2 | 0.720713411 | 0.177376099 | NA | NS | 0.7511049 |
| ALG13 | 0.133015253 | 0.032188982 | 0.198223945 | Up | 1.492292761 |
| TRPC5 | 0.246220585 | 0.029175026 | 0.190306592 | Up | 1.534988749 |
| TRPC5OS | 0.359382074 | 0.403826698 | NA | NS | 0.393804972 |
| RTL4 | 0.323560807 | 0.56713638 | NA | NS | 0.246312493 |
| LHFPL1 | 0.037998278 | 0.869236224 | 0.926306268 | NS | 0.060862184 |
| AMOT | 8.04E-04 | 0.992749038 | 0.995749068 | NS | 0.003160525 |
| HTR2C | 0.124700517 | 0.798990203 | NA | NS | 0.097458546 |
| IL13RA2 | 0.100475024 | 0.848965932 | NA | NS | 0.071109737 |
| LRCH2 | 0.092551331 | 0.842403769 | NA | NS | 0.074479699 |
| RBMXL3 | 0.753920172 | 0.104388145 | NA | NS | 0.981348818 |
| LUZP4 | 0.279957382 | 0.678253443 | NA | NS | 0.168607993 |
| PLS3 | 0.102006836 | 0.600441929 | 0.754648056 | NS | 0.221528988 |
| AGTR2 | 0.156266683 | 0.787015941 | NA | NS | 0.104016471 |
| SLC6A14 | 0.214785377 | 0.649383241 | NA | NS | 0.187498924 |
| CT83 | 0.463168307 | 0.574185144 | NA | NS | 0.240948049 |
| KLHL13 | 0.408273713 | 0.01864557 | 0.158871289 | Up | 1.729424337 |
| WDR44 | 0.027525505 | 0.726495074 | 0.838611255 | NS | 0.138767326 |
| DOCK11 | -0.047485382 | 0.390341898 | 0.59729185 | NS | 0.408554831 |
| IL13RA1 | 0.116148459 | 0.335110816 | 0.551502437 | NS | 0.474811554 |
| ZCCHC12 | 0.292032982 | 0.471389341 | NA | NS | 0.326620242 |
| LONRF3 | 0.112426811 | 0.088195958 | 0.298122562 | NS | 1.05455132 |
| KIAA1210 | 0.178024346 | 0.695772505 | 0.819637976 | NS | 0.157532738 |
| PGRMC1 | -0.163394978 | 0.125336156 | 0.346840099 | NS | 0.901923627 |
| SLC25A43 | -0.183496636 | 0.033631057 | 0.201786342 | Down | 1.473259483 |
| SLC25A5 | -0.23786145 | 0.024367918 | 0.176894283 | Down | 1.613181577 |
| STEEP1 | -0.25601185 | 3.03E-04 | 0.029045993 | Down | 3.518389285 |
| UBE2A | 0.154433758 | 0.004873466 | 0.096717602 | Up | 2.312162083 |
| NKRF | 0.013948817 | 0.807331338 | 0.889015228 | NS | 0.092948189 |
| SEPTIN6 | 0.064736959 | 0.342204113 | 0.557467049 | NS | 0.465714774 |
| SOWAHD | 0.037944271 | 0.666188946 | 0.800269084 | NS | 0.176402578 |
| RPL39 | -0.261279953 | 0.256348745 | 0.483320899 | NS | 0.591168805 |
| UPF3B | 0.001969262 | 0.977068972 | 0.986632875 | NS | 0.010074778 |
| RNF113A | -0.232361187 | 0.01554966 | 0.148318589 | Down | 1.808279101 |
| NDUFA1 | -0.277335387 | 0.061710019 | 0.258215532 | NS | 1.209644322 |
| AKAP14 | 0.236644011 | 0.540033441 | NA | NS | 0.267579346 |
| NKAP | -0.016388505 | 0.726915793 | 0.838611255 | NS | 0.138515896 |
| RHOXF1 | 0.686807435 | 0.030398532 | NA | Up | 1.517147388 |
| RHOXF2 | 0.001946573 | 0.993521883 | NA | NS | 0.002822563 |
| ZBTB33 | -0.118275727 | 0.163959125 | 0.391802691 | NS | 0.785264407 |
| TMEM255A | 0.088218863 | 0.471870258 | 0.660546067 | NS | 0.326177396 |
| ATP1B4 | 0.321463081 | 0.467763384 | 0.657432551 | NS | 0.329973777 |
| LAMP2 | -0.024670829 | 0.823293612 | 0.899413506 | NS | 0.084445254 |
| CUL4B | 0.063483658 | 0.186328099 | 0.41588454 | NS | 0.729721647 |
| MCTS1 | -0.042212011 | 0.70698154 | 0.826665892 | NS | 0.150591926 |
| C1GALT1C1 | -0.02173339 | 0.884243072 | 0.934730433 | NS | 0.053428334 |
| CT47B1 | 0.23483194 | 0.73842491 | NA | NS | 0.131693661 |
| GRIA3 | 0.360799057 | 0.186395118 | 0.41588454 | NS | 0.729565467 |
| THOC2 | -0.095866574 | 0.034856277 | 0.204782731 | NS | 1.457718999 |
| XIAP | -0.09848347 | 0.041585717 | 0.219800623 | NS | 1.381055801 |
| STAG2 | -0.139870364 | 0.029018593 | 0.190037745 | Down | 1.537323647 |
| SH2D1A | -0.237161748 | 0.023264013 | 0.173340472 | Down | 1.633315366 |
| TEX13D | 0.369465321 | 0.405196489 | NA | NS | 0.392334326 |
| TENM1 | 0.094944956 | 0.345016341 | 0.559878427 | NS | 0.462160335 |
| TEX13C | 0.374796709 | 0.391591023 | NA | NS | 0.407167273 |
| DCAF12L2 | 0.464642494 | 0.347870323 | NA | NS | 0.45858262 |
| DCAF12L1 | 0.979973421 | 0.083099298 | NA | NS | 1.080402647 |
| PRR32 | 0.283363 | 0.647747308 | NA | NS | 0.188594383 |
| ACTRT1 | 0.823088206 | 0.269990555 | NA | NS | 0.568651428 |
| SMARCA1 | 0.050808977 | 0.80440752 | 0.887662187 | NS | 0.094523878 |
| OCRL | -0.032929393 | 0.699776937 | 0.822143007 | NS | 0.155040375 |
| APLN | 0.499792193 | 0.333508312 | NA | NS | 0.476893338 |
| XPNPEP2 | 0.150034045 | 0.588491415 | 0.746745381 | NS | 0.230259869 |
| SASH3 | 0.045989664 | 0.384892366 | 0.593309885 | NS | 0.414660703 |
| ZDHHC9 | 0.162476332 | 0.17292668 | 0.401822933 | NS | 0.762137997 |
| UTP14A | -0.180075941 | 0.005414529 | 0.100338483 | Down | 2.266439335 |
| BCORL1 | -0.024612128 | 0.891625167 | 0.938936894 | NS | 0.049817682 |
| ELF4 | 0.297475437 | 0.017484214 | 0.155202892 | Up | 1.757353895 |
| AIFM1 | -0.031880366 | 0.403304374 | 0.60738002 | NS | 0.394367068 |
| RAB33A | 0.042290346 | 0.655302683 | 0.792843893 | NS | 0.183558054 |
| ZNF280C | 0.061809626 | 0.33228236 | 0.549073569 | NS | 0.478492713 |
| SLC25A14 | 0.087071737 | 0.104548243 | 0.320548581 | NS | 0.980683263 |
| GPR119 | 0.407808095 | 0.253726056 | NA | NS | 0.595634931 |
| RBMX2 | -0.224607327 | 0.008929806 | 0.11966441 | Down | 2.049157987 |
| ENOX2 | -0.204123162 | 0.001487019 | 0.062253356 | Down | 2.827683406 |
| ARHGAP36 | 0.343645842 | 0.461111706 | NA | NS | 0.336193852 |
| IGSF1 | 0.315055288 | 0.457109088 | 0.648659294 | NS | 0.339980144 |
| OR13H1 | 0.170946136 | 0.743964562 | NA | NS | 0.128447751 |
| STK26 | -0.231283798 | 0.004624633 | 0.094890448 | Down | 2.334922732 |
| FRMD7 | -0.036075522 | 0.790281132 | 0.878678439 | NS | 0.102218387 |
| RAP2C | 0.022563071 | 0.762674842 | 0.861748445 | NS | 0.117660579 |
| MBNL3 | -0.142228789 | 0.431948194 | 0.628863377 | NS | 0.364568337 |
| HS6ST2 | 0.731100378 | 0.105956875 | NA | NS | 0.97487086 |
| USP26 | 0.519924559 | 0.283810293 | NA | NS | 0.546971858 |
| TFDP3 | 0.433663527 | 0.430545772 | NA | NS | 0.365980671 |
| GPC4 | 0.012351152 | 0.962616723 | 0.979032249 | NS | 0.016546598 |
| GPC3 | 0.441209591 | 0.179452089 | 0.409670229 | NS | 0.746051482 |
| CCDC160 | 0.810708424 | 0.150965014 | NA | NS | 0.821123687 |
| PHF6 | -0.112818759 | 0.048593241 | 0.233749717 | Down | 1.313424135 |
| HPRT1 | -0.083553125 | 0.511154868 | 0.689903595 | NS | 0.291447499 |
| PLAC1 | -0.044645327 | 0.935270591 | NA | NS | 0.029062722 |
| PABIR2 | 0.017629636 | 0.826240521 | 0.901031399 | NS | 0.08289351 |
| PABIR3 | 0.177115406 | 0.02852436 | 0.188728833 | Up | 1.544784099 |
| MOSPD1 | -0.25124845 | 0.077779143 | 0.282118772 | NS | 1.109136849 |
| SMIM10 | -0.135494242 | 0.628007437 | NA | NS | 0.202035213 |
| RTL8B | 0.398812983 | 0.311780205 | NA | NS | 0.506151462 |
| RTL8C | 0.205384748 | 0.112826178 | 0.331432435 | NS | 0.947590124 |
| RTL8A | 0.372673844 | 0.045763213 | 0.228356747 | Up | 1.339483496 |
| SMIM10L2B | 0.565283236 | 0.17678985 | NA | NS | 0.752542672 |
| ETDB | 0.7155967 | 0.329022801 | NA | NS | 0.482774004 |
| CT55 | 0.871508124 | 0.113634164 | NA | NS | 0.944491079 |
| ZNF75D | 0.006025993 | 0.893163784 | 0.939952072 | NS | 0.049068895 |
| ETDA | -0.348687214 | 0.492694288 | NA | NS | 0.307422472 |
| ETDC | 0.006592714 | 0.970822358 | 0.983447171 | NS | 0.012860231 |
| ZNF449 | -0.015753363 | 0.791631688 | 0.879667585 | NS | 0.10147683 |
| SMIM10L2A | 0.277605544 | 0.139584547 | 0.363934068 | NS | 0.855162659 |
| INTS6L | -0.101202905 | 0.05431556 | 0.246476974 | NS | 1.265075739 |
| CT45A1 | 0.716742418 | 0.285105708 | NA | NS | 0.544994088 |
| CT45A3 | 0.530817918 | 0.664060474 | NA | NS | 0.177792369 |
| CT45A10 | 0.485315671 | 0.461764655 | NA | NS | 0.335579313 |
| SAGE1 | 0.426403212 | 0.355939886 | NA | NS | 0.448623343 |
| MMGT1 | 0.07822864 | 0.155493003 | 0.38287289 | NS | 0.808289149 |
| SLC9A6 | -0.050442473 | 0.407763614 | 0.611085087 | NS | 0.38959153 |
| FHL1 | -0.093626301 | 0.381791733 | 0.591316309 | NS | 0.41817348 |
| MAP7D3 | -0.194850874 | 6.49E-04 | 0.04259149 | Down | 3.187616016 |
| ADGRG4 | 0.400395556 | 0.342800708 | 0.558038995 | NS | 0.46495829 |
| BRS3 | 0.214017563 | 0.489660166 | NA | NS | 0.310105224 |
| HTATSF1 | -0.241599408 | 0.00112043 | 0.05424964 | Down | 2.950615412 |
| VGLL1 | 0.365216233 | 0.438765451 | NA | NS | 0.357767577 |
| CD40LG | 0.005441334 | 0.94716431 | 0.971541457 | NS | 0.023574675 |
| ARHGEF6 | -0.051489107 | 0.288645282 | 0.511830295 | NS | 0.539635537 |
| RBMX | -0.064533083 | 0.226968425 | 0.455488754 | NS | 0.644034556 |
| GPR101 | 0.233431302 | 0.60230756 | NA | NS | 0.220181685 |
| ZIC3 | 0.596691474 | 0.208178244 | NA | NS | 0.681564659 |
| FGF13 | 0.236157847 | 0.389583314 | 0.59694612 | NS | 0.409399652 |
| F9 | 0.200223253 | 0.680697056 | NA | NS | 0.167046128 |
| MCF2 | 0.281274744 | 0.011282116 | 0.130167764 | Up | 1.947609454 |
| ATP11C | -0.058740355 | 0.189418281 | 0.418812268 | NS | 0.722578109 |
| CXorf66 | 0.228262523 | 0.711161942 | NA | NS | 0.148031493 |
| SOX3 | 0.777611417 | 0.13187344 | NA | NS | 0.879842664 |
| SPANXB1 | 0.621279535 | 0.307695431 | NA | NS | 0.511878952 |
| LDOC1 | 0.068228143 | 0.731566065 | 0.841122848 | NS | 0.135746448 |
| SPANXD | 0.447455672 | 0.498676269 | NA | NS | 0.302181298 |
| MAGEC3 | 0.116494822 | 0.822073944 | NA | NS | 0.085089117 |
| MAGEC1 | 0.507311359 | 0.326561622 | NA | NS | 0.486034856 |
| MAGEC2 | 0.23627057 | 0.702064576 | NA | NS | 0.15362294 |
| SPANXN4 | 0.741355921 | 0.181924462 | NA | NS | 0.7401089 |
| SPANXN3 | -0.032245594 | 0.962924324 | NA | NS | 0.016407843 |
| SLITRK4 | 0.09449558 | 0.39703104 | 0.602252276 | NS | 0.401175539 |
| SPANXN2 | 0.43085123 | 0.567967038 | NA | NS | 0.245676868 |
| SPANXN1 | 0.973874586 | 0.20167443 | NA | NS | 0.695349161 |
| SLITRK2 | 0.573876503 | 0.231233572 | 0.459697158 | NS | 0.635949112 |
| FMR1 | -0.036578798 | 0.424107287 | 0.623021494 | NS | 0.372524265 |
| FMR1NB | 0.528757653 | 0.220582291 | NA | NS | 0.656429356 |
| AFF2 | 0.09502903 | 0.45702921 | 0.648626322 | NS | 0.340056042 |
| IDS | 0.250406657 | 0.01586172 | 0.149445555 | Up | 1.799649724 |
| EOLA1 | -0.094061011 | 0.10244107 | 0.318159125 | NS | 0.989525894 |
| HSFX3 | -0.102829388 | 0.687163421 | NA | NS | 0.162939967 |
| MAGEA9B | 0.821057676 | 0.474240979 | NA | NS | 0.324000921 |
| HSFX2 | 0.700745675 | 0.206428758 | NA | NS | 0.6852298 |
| TMEM185A | -0.030178349 | 0.616466318 | 0.765552089 | NS | 0.210090647 |
| MAGEA11 | 0.077668178 | 0.874100495 | NA | NS | 0.058438634 |
| HSFX1 | 0.292321554 | 0.423993976 | NA | NS | 0.372640314 |
| MAGEA8 | 0.506544302 | 0.184492783 | NA | NS | 0.734020617 |
| EOLA2 | -0.05037185 | 0.307540605 | 0.528458503 | NS | 0.512097536 |
| HSFX4 | 0.295869505 | 0.165565926 | NA | NS | 0.781029037 |
| MAMLD1 | -0.077695771 | 0.751184557 | 0.853990008 | NS | 0.124253349 |
| MTM1 | 0.018256335 | 0.663795808 | 0.798689271 | NS | 0.177965495 |
| MTMR1 | 0.003255243 | 0.934976658 | 0.96397808 | NS | 0.029199231 |
| CD99L2 | -0.165145907 | 0.007246781 | 0.11179811 | Down | 2.139854841 |
| HMGB3 | -0.039658406 | 0.785070468 | 0.875872417 | NS | 0.105091359 |
| GPR50 | 0.28742533 | 0.580539832 | NA | NS | 0.236167977 |
| VMA21 | -0.2139522 | 0.010404541 | 0.127356918 | Down | 1.982777082 |
| PASD1 | -0.155984115 | 0.749758319 | NA | NS | 0.125078707 |
| PRRG3 | 0.581043178 | 0.228239288 | NA | NS | 0.641609596 |
| FATE1 | 1.420465292 | 0.006366161 | NA | Up | 2.196122362 |
| CNGA2 | 0.226486216 | 0.653871027 | NA | NS | 0.184507906 |
| MAGEA4 | 0.669895329 | 0.280789784 | NA | NS | 0.551618698 |
| GABRE | 0.357562991 | 0.359322379 | 0.572255251 | NS | 0.444515734 |
| MAGEA10 | 0.333745675 | 0.593555948 | NA | NS | 0.226538338 |
| GABRA3 | 0.421872414 | 0.340355419 | 0.556026263 | NS | 0.46806733 |
| GABRQ | 0.434959488 | 0.295783025 | 0.517575095 | NS | 0.529026753 |
| MAGEA3 | 0.69871298 | 0.230276068 | NA | NS | 0.637751194 |
| CSAG2 | 0.268971667 | 0.826832788 | NA | NS | 0.08258231 |
| CSAG1 | 1.334441061 | 0.061792121 | NA | NS | 1.209066894 |
| MAGEA12 | 0.027753302 | 0.966134918 | NA | NS | 0.014962222 |
| CSAG3 | 0.477010244 | 0.486928332 | NA | NS | 0.312534955 |
| MAGEA6 | 0.641308421 | 0.254439395 | NA | NS | 0.594415645 |
| CETN2 | -0.152566122 | 0.113477029 | 0.332206431 | NS | 0.945092044 |
| NSDHL | -0.26689563 | 0.001664487 | 0.064977566 | Down | 2.77871969 |
| ZNF185 | 0.015703531 | 0.879416457 | 0.931951122 | NS | 0.055805412 |
| PNMA5 | -0.119194427 | 0.675582941 | NA | NS | 0.170321325 |
| PNMA3 | 0.01066648 | 0.934228992 | 0.963770828 | NS | 0.029546659 |
| PNMA6A | 0.074821874 | 0.797062834 | NA | NS | 0.098507441 |
| MAGEA1 | 0.181659304 | 0.788765829 | NA | NS | 0.103051912 |
| PWWP4 | 0.927870251 | 0.098575803 | NA | NS | 1.006229678 |
| PNMA6F | 1.229104456 | 0.028464065 | NA | Up | 1.545703079 |
| ZNF275 | 0.208834336 | 0.00557016 | 0.101332034 | Up | 2.254132308 |
| PNMA6E | 0.602810917 | 0.233528684 | NA | NS | 0.631659768 |
| ZFP92 | 0.289664986 | 0.081345928 | 0.287675175 | NS | 1.089664184 |
| TREX2 | 0.295109016 | 0.065856709 | 0.265127437 | NS | 1.181399975 |
| HAUS7 | 0.182457762 | 0.099309675 | 0.314301595 | NS | 1.003008438 |
| BGN | 0.609610853 | 0.229401234 | NA | NS | 0.639404251 |
| ATP2B3 | 0.528727369 | 0.209742719 | 0.439792418 | NS | 0.678313106 |
| CCNQ | -0.187159719 | 0.053003511 | 0.243263808 | NS | 1.275695363 |
| DUSP9 | 0.719144391 | 0.138762698 | NA | NS | 0.857727264 |
| PNCK | 0.435413948 | 0.361675872 | 0.57427477 | NS | 0.441680463 |
| SLC6A8 | -0.192492707 | 0.411774842 | 0.614131594 | NS | 0.385340191 |
| BCAP31 | -0.037166508 | 0.593500356 | 0.749772128 | NS | 0.226579016 |
| ABCD1 | 0.314107082 | 0.0128051 | 0.136078062 | Up | 1.89261703 |
| PLXNB3 | 0.488403708 | 0.145967514 | 0.371438666 | NS | 0.835743789 |
| SRPK3 | 0.193123556 | 0.207521776 | 0.437401068 | NS | 0.682936325 |
| IDH3G | -0.191429525 | 0.011223059 | 0.13003023 | Down | 1.949888769 |
| SSR4 | -0.145965123 | 0.094592498 | 0.307721221 | NS | 1.024143304 |
| PDZD4 | -0.272755462 | 0.007878983 | 0.11472857 | Down | 2.103529809 |
| L1CAM | 0.503411585 | 0.148861932 | 0.374840808 | NS | 0.827216349 |
| AVPR2 | -0.077539917 | 0.625211235 | 0.771392506 | NS | 0.203973226 |
| ARHGAP4 | -0.071505667 | 0.433377075 | 0.629801069 | NS | 0.363134065 |
| NAA10 | -0.092701283 | 0.269193898 | 0.494472141 | NS | 0.569934789 |
| RENBP | -0.043502338 | 0.625693872 | 0.77176194 | NS | 0.203638098 |
| HCFC1 | -0.027880435 | 0.857391757 | 0.919589098 | NS | 0.066820696 |
| TMEM187 | -0.110825655 | 0.267011118 | 0.492953536 | NS | 0.573470655 |
| IRAK1 | 0.051505325 | 0.438191231 | 0.633591488 | NS | 0.358336318 |
| MECP2 | 0.030629816 | 0.7657731 | 0.863717375 | NS | 0.115899894 |
| OPN1LW | 0.284644444 | 0.619599905 | NA | NS | 0.207888658 |
| TEX28 | 1.1061261 | 0.052923692 | NA | NS | 1.276349867 |
| TKTL1 | -0.026631767 | 0.83775617 | 0.908709855 | NS | 0.076882365 |
| FLNA | -0.091237109 | 0.465958923 | 0.656166026 | NS | 0.331652368 |
| EMD | -0.050438573 | 0.505475736 | 0.685927908 | NS | 0.296299687 |
| RPL10 | -0.037617537 | 0.737346962 | 0.845368058 | NS | 0.132328105 |
| DNASE1L1 | -0.167998225 | 0.01424958 | 0.143336863 | Down | 1.846197926 |
| TAFAZZIN | -0.08325107 | 0.145451558 | 0.37059965 | NS | 0.837281622 |
| ATP6AP1 | 0.104822312 | 0.144285245 | 0.36967266 | NS | 0.840778078 |
| GDI1 | -0.047708306 | 0.537853167 | 0.709095159 | NS | 0.26933627 |
| FAM50A | -0.316818232 | 1.02E-04 | 0.015965905 | Down | 3.992327008 |
| PLXNA3 | -0.086284175 | 0.572176838 | 0.733952836 | NS | 0.242469727 |
| LAGE3 | -0.233850762 | 0.022259258 | 0.170666732 | Down | 1.652489316 |
| UBL4A | -0.079730011 | 0.231655404 | 0.459829325 | NS | 0.635157564 |
| SLC10A3 | -0.03788754 | 0.619230843 | 0.767141109 | NS | 0.20814742 |
| FAM3A | 0.007883782 | 0.923851695 | 0.958323724 | NS | 0.03439774 |
| G6PD | 0.059564748 | 0.614178081 | 0.764054018 | NS | 0.211705687 |
| IKBKG | 0.13761743 | 0.14459741 | 0.369718643 | NS | 0.839839485 |
| CTAG2 | 0.987316186 | 0.152219092 | NA | NS | 0.817530874 |
| GAB3 | 0.047221356 | 0.47963928 | 0.666709079 | NS | 0.319085258 |
| DKC1 | -0.161079279 | 0.00341056 | 0.085194315 | Down | 2.467174296 |
| MPP1 | -0.174032024 | 0.327014376 | 0.545190598 | NS | 0.485433155 |
| SMIM9 | 0.414994053 | 0.312116725 | NA | NS | 0.505682958 |
| F8 | 0.059129821 | 0.589574206 | 0.747377833 | NS | 0.229461525 |
| F8A1 | 0.203642444 | 0.086056131 | 0.29485869 | NS | 1.065218183 |
| FUNDC2 | -0.262754358 | 0.020189565 | 0.164578764 | Down | 1.69487304 |
| CMC4 | -0.266217902 | 0.45962428 | NA | NS | 0.337597038 |
| MTCP1 | 0.050270564 | 0.580000737 | 0.739515471 | NS | 0.236571454 |
| BRCC3 | -0.057433124 | 0.231692954 | 0.459829325 | NS | 0.635087172 |
| VBP1 | -0.263722899 | 0.032031742 | 0.198142899 | Down | 1.494419438 |
| RAB39B | -0.170366676 | 0.019880333 | 0.163677883 | Down | 1.701576335 |
| CLIC2 | -0.329709288 | 0.064863059 | 0.264064926 | NS | 1.188002575 |
| TMLHE | 0.111351291 | 0.024836154 | 0.178586374 | Up | 1.604915654 |
| SPRY3 | 0.443605644 | 0.009955053 | NA | Up | 2.00195643 |
| RPS4Y1 | 0.037040643 | 0.97793434 | 0.987153917 | NS | 0.009690303 |
| ZFY | 0.123099546 | 0.93373494 | 0.963391341 | NS | 0.02977639 |
| PCDH11Y | 0.774097337 | 0.510633186 | NA | NS | 0.291890964 |
| AMELY | 0.816091961 | 0.673758694 | NA | NS | 0.171495618 |
| TBL1Y | 1.351843591 | 0.360362747 | NA | NS | 0.443260111 |
| TSPY8 | 1.390685926 | 0.461462158 | NA | NS | 0.335863907 |
| TSPY1 | 1.747911205 | 0.307480241 | NA | NS | 0.512182788 |
| TSPY10 | 0.718039482 | 0.591779484 | NA | NS | 0.227840095 |
| USP9Y | 0.470583283 | 0.743823714 | 0.849882793 | NS | 0.12852998 |
| DDX3Y | 0.20648929 | 0.883299495 | 0.934077898 | NS | 0.053892018 |
| UTY | 0.520009701 | 0.69503769 | 0.819109411 | NS | 0.157991644 |
| TMSB4Y | 0.262454585 | 0.824690241 | NA | NS | 0.083709145 |
| NLGN4Y | 0.989247562 | 0.402278747 | NA | NS | 0.395472911 |
| KDM5D | 0.437531781 | 0.744507674 | 0.850164936 | NS | 0.128130821 |
| EIF1AY | 0.184986982 | 0.89847628 | 0.943474965 | NS | 0.046493384 |
| RPS4Y2 | 0.741399304 | 0.453329774 | NA | NS | 0.343585756 |
| MT-ND1 | 0.386308067 | 0.185201696 | 0.415384315 | NS | 0.73235504 |
| MT-ND2 | 0.576103572 | 0.036680394 | 0.208886406 | Up | 1.435566005 |
| MT-CO1 | -0.02977752 | 0.851875574 | 0.916433852 | NS | 0.069623834 |
| MT-CO2 | 0.134213298 | 0.485606373 | 0.671376828 | NS | 0.313715622 |
| MT-ATP8 | 0.524263952 | 0.076867508 | 0.281082497 | NS | 1.114257196 |
| MT-ATP6 | 0.397821651 | 0.138915024 | 0.363222438 | NS | 0.857250781 |
| MT-CO3 | 0.053906226 | 0.708109513 | 0.82706671 | NS | 0.149899571 |
| MT-ND3 | 0.095043906 | 0.612167466 | 0.762736484 | NS | 0.213129755 |
| MT-ND4L | 0.138820863 | 0.590944223 | 0.748054643 | NS | 0.228453508 |
| MT-ND4 | 0.44961696 | 0.113284503 | 0.332115589 | NS | 0.945829498 |
| MT-ND5 | 0.296519056 | 0.302226068 | 0.523727407 | NS | 0.519668079 |
| MT-ND6 | 0.534513024 | 0.088017329 | 0.29792682 | NS | 1.055431816 |
| MT-CYB | 0.198626937 | 0.425999922 | 0.624240208 | NS | 0.370590481 |

**Supplementary Table 2.** Lists of the differentially expressed lncRNAs (DELs) between T2D and normal samples in GSE184050.

| symbol | logfc | p.value | adj.p.val | direction | log10pvalue |
| --- | --- | --- | --- | --- | --- |
| LINC01409 | 0.154366886 | 0.124435255 | 0.34606147 | NS | 0.905056559 |
| FAM87B | 0.116663277 | 0.250526351 | 0.477493271 | NS | 0.601146587 |
| LINC01128 | -0.022514431 | 0.773156066 | 0.86784941 | NS | 0.111732832 |
| LINC00115 | 0.428308123 | 0.00121059 | 0.056242293 | Up | 2.917002739 |
| FAM41C | -0.147155477 | 0.707815325 | NA | NS | 0.150080039 |
| LINC02593 | 0.620371485 | 0.1889056 | NA | NS | 0.723755166 |
| LINC01342 | 0.54882165 | 0.195838072 | NA | NS | 0.708102875 |
| TTLL10-AS1 | 0.838011851 | 0.103126554 | NA | NS | 0.986629493 |
| LINC01786 | -0.001541656 | 0.994254289 | NA | NS | 0.002502527 |
| MRPL20-AS1 | -0.25228187 | 8.00E-05 | 0.013591828 | Down | 4.096851072 |
| MRPL20-DT | -0.135263795 | 0.200914299 | 0.430596428 | NS | 0.696989154 |
| LINC01770 | 1.241739888 | 0.061122269 | NA | NS | 1.213800534 |
| PRKCZ-AS1 | 0.036949107 | 0.82924254 | 0.902929332 | NS | 0.081318427 |
| TNFRSF14-AS1 | 0.217989518 | 0.479110213 | NA | NS | 0.319564571 |
| PRDM16-DT | 1.073555289 | 0.01772041 | NA | Up | 1.751526242 |
| TP73-AS3 | 0.64304083 | 0.167420715 | NA | NS | 0.776190807 |
| TP73-AS2 | 1.179821498 | 0.006901266 | NA | Up | 2.161071221 |
| LINC01134 | 0.166124688 | 0.421815537 | 0.621413862 | NS | 0.374877427 |
| LINC01346 | 0.674483961 | 0.165972463 | NA | NS | 0.779963961 |
| LINC01345 | 0.688305096 | 0.201294376 | NA | NS | 0.696168359 |
| LINC02780 | 0.919185083 | 0.104359757 | NA | NS | 0.98146694 |
| LINC01777 | 0.454283849 | 0.332523176 | NA | NS | 0.47817808 |
| LINC01646 | 0.398063952 | 0.425746117 | NA | NS | 0.370849304 |
| LINC02781 | 0.179086056 | 0.73394243 | NA | NS | 0.134338005 |
| LINC02782 | 0.405433477 | 0.506958893 | NA | NS | 0.295027254 |
| RNF207-AS1 | -0.054849031 | 0.887041659 | NA | NS | 0.052055984 |
| LINC00337 | 0.19590324 | 0.370935133 | NA | NS | 0.43070203 |
| LINC01672 | 0.684818838 | 0.213195998 | NA | NS | 0.671220952 |
| CAMTA1-DT | 0.06148667 | 0.723559608 | NA | NS | 0.140525685 |
| CAMTA1-IT1 | 0.182167853 | 0.802327216 | NA | NS | 0.095648476 |
| CAMTA1-AS2 | 0.221786521 | 0.662486631 | NA | NS | 0.178822882 |
| LINC01714 | 0.615320305 | 0.281465762 | NA | NS | 0.550574426 |
| RERE-AS1 | 0.193629536 | 0.06592819 | 0.265127437 | NS | 1.180928845 |
| ENO1-AS1 | 0.289697296 | 0.208757055 | NA | NS | 0.680358839 |
| MIR34AHG | 0.362950517 | 0.182011759 | 0.412022664 | NS | 0.739900554 |
| LNCTAM34A | 0.006622531 | 0.987237606 | NA | NS | 0.00557831 |
| LINC02606 | 0.73295701 | 0.041384964 | NA | Up | 1.383157419 |
| PIK3CD-AS1 | 0.123855911 | 0.242985575 | 0.470680507 | NS | 0.614419509 |
| PIK3CD-AS2 | -0.022368739 | 0.90783762 | 0.949070944 | NS | 0.041991824 |
| EXOSC10-AS1 | 0.391697418 | 0.17424574 | NA | NS | 0.75883783 |
| MTOR-AS1 | 0.471918566 | 0.010092477 | 0.126199476 | Up | 1.996002221 |
| LINC01647 | 0.181990064 | 0.743770775 | NA | NS | 0.12856089 |
| LINC02766 | 0.360660073 | 0.144821115 | 0.37000147 | NS | 0.839168113 |
| KAZN-AS1 | 0.557777537 | 0.184037329 | NA | NS | 0.735094079 |
| TMEM51-AS1 | 0.565976092 | 0.145520882 | NA | NS | 0.837074682 |
| TMEM51-AS2 | 0.635822347 | 0.183769715 | NA | NS | 0.735726058 |
| FHAD1-AS1 | 1.194441048 | 0.002180178 | NA | Up | 2.661508037 |
| SLC25A34-AS1 | 0.172074042 | 0.817755894 | NA | NS | 0.087376317 |
| FLJ37453 | 0.010700208 | 0.88859927 | 0.937172484 | NS | 0.051294048 |
| LINC01772 | 0.153539158 | 0.113462941 | 0.332206431 | NS | 0.945145965 |
| LINC01783 | 1.160448685 | 2.45E-04 | NA | Up | 3.610993812 |
| LINC02810 | 0.245795195 | 0.664227994 | NA | NS | 0.177682825 |
| LINC01654 | 1.128724984 | 0.040477487 | NA | Up | 1.392786458 |
| IGSF21-AS1 | 0.458321386 | 0.425332715 | NA | NS | 0.371271212 |
| EMC1-AS1 | 0.122762635 | 0.259762294 | 0.485843002 | NS | 0.585423889 |
| LINC01757 | 0.916410961 | 0.128456618 | NA | NS | 0.891243517 |
| LINC01141 | 0.64245186 | 0.114840426 | 0.333655677 | NS | 0.939905205 |
| PINK1-AS | 0.11196311 | 0.089600983 | 0.299962245 | NS | 1.047687224 |
| ECE1-AS1 | 0.072661978 | 0.481755663 | 0.668433794 | NS | 0.317173171 |
| LINC02596 | 0.166142215 | 0.371723854 | 0.582777909 | NS | 0.429779569 |
| LINC01635 | 0.413545171 | 0.234257665 | NA | NS | 0.63030619 |
| LINC00339 | 0.315412179 | 0.001413761 | 0.060975423 | Up | 2.849624064 |
| CDC42-AS1 | 0.287716417 | 0.02859867 | 0.188990708 | Up | 1.543654169 |
| CDC42-IT1 | 0.060513217 | 0.560446195 | 0.724996541 | NS | 0.251466075 |
| ZBTB40-IT1 | 0.139078005 | 0.445081463 | 0.639053249 | NS | 0.351560493 |
| LINC01355 | 0.03301398 | 0.903695254 | NA | NS | 0.043977998 |
| MDS2 | 0.1521456 | 0.252050064 | 0.479033151 | NS | 0.598513188 |
| ELOA-AS1 | 0.190889788 | 0.077044619 | 0.28141561 | NS | 1.113257686 |
| LINC02800 | 0.126795749 | 0.830822633 | NA | NS | 0.080491681 |
| GRHL3-AS1 | 0.38253574 | 0.300706264 | NA | NS | 0.521857525 |
| RCAN3AS | 0.115364024 | 0.423644861 | 0.622710628 | NS | 0.372998057 |
| RUNX3-AS1 | 0.084411431 | 0.666775851 | NA | NS | 0.176020137 |
| LINC02793 | 0.523476392 | 0.100094829 | NA | NS | 0.999588356 |
| DHDDS-AS1 | 0.0679012 | 0.415801548 | 0.61707407 | NS | 0.381113899 |
| LINC02574 | 0.295113692 | 0.264968122 | NA | NS | 0.576806372 |
| SNHG3 | 0.078792323 | 0.225654342 | 0.454284084 | NS | 0.646556306 |
| SNHG12 | -0.019504675 | 0.782095239 | 0.874032361 | NS | 0.106740358 |
| LINC01715 | 0.076251447 | 0.627813924 | 0.773353834 | NS | 0.202169057 |
| LINC01756 | 0.908519036 | 0.09418368 | NA | NS | 1.026024344 |
| LINC01648 | -0.036468938 | 0.949210317 | NA | NS | 0.02263755 |
| MATN1-AS1 | 0.326916951 | 0.006556327 | 0.108312793 | Up | 2.183339383 |
| LINC01778 | 0.604283699 | 0.252325793 | NA | NS | 0.598038353 |
| LINC01226 | 0.446363342 | 0.137645901 | 0.36251944 | NS | 0.861236717 |
| PEF1-AS1 | 0.222300723 | 0.267120805 | 0.493076543 | NS | 0.573292285 |
| PHC2-AS1 | 0.366587016 | 0.120491739 | NA | NS | 0.919042728 |
| CSMD2-AS1 | 0.580740763 | 0.259162998 | NA | NS | 0.586427005 |
| ZMYM4-AS1 | 0.121821994 | 0.480284339 | NA | NS | 0.318501575 |
| TFAP2E-AS1 | 0.086560706 | 0.399973562 | 0.604986227 | NS | 0.397968714 |
| LINC01137 | -0.040018182 | 0.655209099 | 0.792772547 | NS | 0.18362008 |
| MIR3659HG | 0.78303877 | 0.149503041 | NA | NS | 0.825349973 |
| LINC02786 | 0.72319468 | 0.153618825 | NA | NS | 0.81355556 |
| LINC01343 | 0.521145421 | 0.315771096 | NA | NS | 0.500627625 |
| LINC01685 | 0.365798276 | 0.12950124 | 0.351485827 | NS | 0.887726072 |
| PABPC4-AS1 | 0.412664792 | 0.004634989 | 0.0949468 | Up | 2.333951273 |
| BMP8B-AS1 | 0.435427065 | 0.242352018 | NA | NS | 0.61555336 |
| LINC02811 | 0.301077099 | 0.491570287 | NA | NS | 0.308414376 |
| MYCL-AS1 | 0.761506169 | 0.006697444 | NA | Up | 2.174090899 |
| ZMPSTE24-DT | -0.144279213 | 0.201890996 | 0.431920372 | NS | 0.69488305 |
| NFYC-AS1 | 0.074235451 | 0.351149076 | 0.565113052 | NS | 0.45450847 |
| SLFNL1-AS1 | 0.285261802 | 0.058964868 | 0.25340841 | NS | 1.229406667 |
| SLC2A1-AS1 | 0.103973382 | 0.63511863 | 0.777459344 | NS | 0.197145148 |
| MED8-AS1 | 0.162203246 | 0.142543287 | 0.36732856 | NS | 0.846053231 |
| KDM4A-AS1 | 0.141929526 | 0.071438892 | 0.272521361 | NS | 1.146065288 |
| ST3GAL3-AS1 | 0.307427734 | 0.192324673 | NA | NS | 0.715964998 |
| ERI3-IT1 | -0.076416631 | 0.687615016 | 0.81446905 | NS | 0.162654648 |
| LINC01144 | 0.46331645 | 0.017825278 | NA | Up | 1.748963677 |
| LINC01398 | 0.382656922 | 0.130604297 | NA | NS | 0.884042534 |
| MKNK1-AS1 | 0.144264735 | 0.275472023 | 0.500040883 | NS | 0.559922501 |
| EFCAB14-AS1 | 0.121249063 | 0.672644122 | NA | NS | 0.172214649 |
| CYP4A22-AS1 | -0.257400788 | 0.375951809 | 0.586177342 | NS | 0.424867821 |
| LINC00853 | 0.220297351 | 0.406234609 | NA | NS | 0.391223079 |
| LINC01389 | 0.158053575 | 0.202412127 | 0.43255039 | NS | 0.693763472 |
| FOXD2-AS1 | 0.345889091 | 0.102014537 | 0.317910259 | NS | 0.991337935 |
| LINC01738 | 0.41507691 | 0.483637228 | NA | NS | 0.315480277 |
| LINC02794 | 0.728340488 | 0.216467563 | NA | NS | 0.664607172 |
| AGBL4-AS1 | 0.429920354 | 0.475241628 | NA | NS | 0.323085525 |
| AGBL4-IT1 | 0.286729666 | 0.557743187 | NA | NS | 0.253565726 |
| ELAVL4-AS1 | 0.446014277 | 0.498440266 | NA | NS | 0.302386881 |
| LINC02808 | 0.622444723 | 0.186735775 | NA | NS | 0.728772472 |
| FAF1-AS1 | 0.24637915 | 0.138456131 | 0.363014451 | NS | 0.858687808 |
| LINC01562 | 0.353385114 | 0.308224961 | NA | NS | 0.511132194 |
| TTC39A-AS1 | -0.328518936 | 0.584576789 | NA | NS | 0.233158433 |
| EPS15-AS1 | -0.068618856 | 0.764274423 | NA | NS | 0.116750674 |
| TXNDC12-AS1 | 0.139628953 | 0.1776129 | 0.407521884 | NS | 0.750525494 |
| CZIB-DT | 0.295716062 | 0.14783584 | NA | NS | 0.830220268 |
| LINC01771 | 0.315378546 | 0.172905233 | NA | NS | 0.762191862 |
| LINC02812 | -0.041878715 | 0.730632564 | 0.840555934 | NS | 0.136300976 |
| SSBP3-AS1 | 0.164238131 | 0.128312174 | 0.350046063 | NS | 0.891732135 |
| LINC02784 | 0.533212296 | 0.513929198 | NA | NS | 0.289096708 |
| DHCR24-DT | 0.923957116 | 0.199181288 | NA | NS | 0.700751465 |
| MIR4422HG | 0.230156976 | 0.361589761 | NA | NS | 0.441783875 |
| LINC01755 | 0.591706601 | 0.213652218 | NA | NS | 0.670292595 |
| LINC01753 | 1.191446642 | 0.084583795 | NA | NS | 1.072712831 |
| LINC01767 | 0.71080866 | 0.147812938 | NA | NS | 0.830287551 |
| DAB1-AS1 | 0.339233517 | 0.143154059 | 0.368107192 | NS | 0.844196333 |
| LINC01135 | 0.085289369 | 0.458625653 | 0.650086323 | NS | 0.338541657 |
| LINC02777 | 0.033942086 | 0.825997143 | 0.900894684 | NS | 0.083021455 |
| LINC01358 | 0.327793194 | 0.116242723 | 0.335719542 | NS | 0.934634225 |
| FGGY-DT | 0.332276327 | 0.202955281 | 0.432986484 | NS | 0.692599643 |
| LINC02778 | 0.1423712 | 0.764104993 | NA | NS | 0.116846962 |
| LINC01748 | 0.463363804 | 0.245652558 | 0.473922574 | NS | 0.60967871 |
| NFIA-AS2 | 0.407981249 | 0.365004228 | 0.577421208 | NS | 0.437702105 |
| NFIA-AS1 | 0.137772981 | 0.455912827 | 0.647804664 | NS | 0.341118188 |
| PATJ-DT | -0.03868302 | 0.894609131 | NA | NS | 0.048366674 |
| LINC01739 | 0.859187035 | 0.155057904 | NA | NS | 0.809506091 |
| LINC00466 | 0.684232113 | 0.117012672 | 0.336830596 | NS | 0.931767102 |
| FOXD3-AS1 | 1.044503903 | 0.053747054 | NA | NS | 1.269645334 |
| DLEU2L | 0.144867598 | 0.106197916 | 0.322480068 | NS | 0.973884006 |
| ROR1-AS1 | 0.586953731 | 0.141953394 | NA | NS | 0.847854219 |
| LINC01359 | 0.282346377 | 0.031626542 | 0.197306204 | Up | 1.499948285 |
| PDE4B-AS1 | 0.470732554 | 0.155171301 | NA | NS | 0.809188599 |
| LINC01702 | 0.520439964 | 0.358805505 | NA | NS | 0.445140903 |
| GNG12-AS1 | 0.299863146 | 0.087488154 | 0.297087647 | NS | 1.058050747 |
| DEPDC1-AS1 | 0.770245525 | 0.126399946 | NA | NS | 0.898253111 |
| LINC01707 | 0.776310232 | 0.117354736 | NA | NS | 0.930499378 |
| LINC02791 | -0.033445174 | 0.957129021 | NA | NS | 0.019029515 |
| LINC01758 | 1.081070413 | 0.091887561 | NA | NS | 1.036743275 |
| LRRC7-AS1 | 0.23355609 | 0.539648687 | NA | NS | 0.267888875 |
| HHLA3 | 0.174880767 | 0.363371376 | 0.57563916 | NS | 0.439649286 |
| HHLA3-AS1 | 0.525036561 | 0.029421436 | NA | Up | 1.53133614 |
| LINC01788 | 0.499972485 | 0.398517397 | NA | NS | 0.399552715 |
| ZRANB2-AS1 | 0.081965567 | 0.384994306 | 0.593347214 | NS | 0.414545693 |
| ZRANB2-AS2 | 0.228463054 | 0.277962079 | 0.502239648 | NS | 0.556014448 |
| NEGR1-IT1 | -0.260419545 | 0.674503136 | NA | NS | 0.171016027 |
| LINC02796 | 0.639829827 | 0.353321846 | NA | NS | 0.451829509 |
| LINC02797 | 0.696047836 | 0.284908384 | NA | NS | 0.54529477 |
| LINC01360 | 0.015535773 | 0.977936375 | NA | NS | 0.0096894 |
| LINC02238 | 0.251492405 | 0.699470819 | NA | NS | 0.155230399 |
| ERICH3-AS1 | 0.396901571 | 0.397476304 | NA | NS | 0.400688757 |
| LINC02567 | 0.869881399 | 0.152831172 | NA | NS | 0.815788056 |
| NEXN-AS1 | 0.212974457 | 0.14169785 | 0.366106066 | NS | 0.848636738 |
| MGC27382 | 0.368149499 | 0.464956557 | NA | NS | 0.332587623 |
| LINC02792 | 0.76863721 | 0.20747656 | NA | NS | 0.683030961 |
| LINC01781 | -0.222287707 | 0.307449223 | 0.52835843 | NS | 0.512226601 |
| LINC01362 | 0.262053801 | 0.220618189 | 0.449602156 | NS | 0.656358685 |
| LINC01361 | 0.189696842 | 0.590209893 | NA | NS | 0.228993515 |
| LINC01712 | 0.184578345 | 0.362357001 | 0.574901284 | NS | 0.440863344 |
| LINC01725 | 0.268390449 | 0.058205657 | 0.252197542 | NS | 1.235034806 |
| TTLL7-IT1 | 0.614689703 | 0.069950003 | NA | NS | 1.155212261 |
| PRKACB-DT | 0.138681599 | 0.42015197 | NA | NS | 0.376593596 |
| LINC01555 | 0.380948079 | 0.408596118 | NA | NS | 0.388705764 |
| LINC02795 | 0.337550895 | 0.03467523 | 0.204304637 | Up | 1.459980646 |
| CLCA4-AS1 | -0.06682262 | 0.605514671 | 0.758108478 | NS | 0.21787533 |
| LINC01140 | 0.368111724 | 0.208288727 | 0.438046853 | NS | 0.681334234 |
| LINC02801 | 0.698814198 | 0.139914092 | 0.364126424 | NS | 0.854138542 |
| LINC01364 | 0.170546616 | 0.185599179 | 0.415463304 | NS | 0.73142395 |
| PKN2-AS1 | 0.146727938 | 0.54424828 | 0.713350547 | NS | 0.264202935 |
| LRRC8C-DT | 0.180216222 | 0.022474189 | 0.171252981 | Up | 1.648315972 |
| LRRC8D-DT | -0.124299022 | 0.397412821 | 0.602512142 | NS | 0.400758126 |
| LINC02787 | 0.554952082 | 0.654446495 | NA | NS | 0.184125854 |
| LINC02609 | 0.384432095 | 0.297373595 | 0.51918646 | NS | 0.526697597 |
| LINC02788 | 0.577986652 | 0.201862878 | NA | NS | 0.694943539 |
| LINC01763 | 1.082658931 | 0.01139655 | NA | Up | 1.943226604 |
| CCDC18-AS1 | 0.004961069 | 0.940313452 | 0.966955174 | NS | 0.026727351 |
| BCAR3-AS1 | 0.400226331 | 0.039886934 | 0.215796828 | Up | 1.399169349 |
| ARHGAP29-AS1 | 0.511268472 | 0.249815535 | NA | NS | 0.602380558 |
| CNN3-DT | 0.330607198 | 0.154771835 | 0.382000708 | NS | 0.810308068 |
| ALG14-AS1 | 0.184809405 | 0.439548577 | NA | NS | 0.356993122 |
| LINC01760 | 0.950450417 | 0.141352564 | NA | NS | 0.84969631 |
| LINC01650 | 0.296688937 | 0.647631048 | NA | NS | 0.188672339 |
| LINC01761 | 0.400163498 | 0.481064853 | NA | NS | 0.317796371 |
| LINC02607 | 0.432613417 | 0.352811781 | 0.566410233 | NS | 0.452456922 |
| LINC02790 | 0.693279564 | 0.166003141 | NA | NS | 0.779883694 |
| LINC01787 | 0.403291816 | 0.488509778 | NA | NS | 0.311126739 |
| DPYD-AS1 | 0.274783332 | 0.016418225 | 0.151221722 | Up | 1.784673797 |
| DPYD-IT1 | 0.112306652 | 0.303740047 | 0.524936328 | NS | 0.517497944 |
| DPYD-AS2 | 0.190515059 | 0.086098798 | 0.29485869 | NS | 1.065002914 |
| MIR137HG | 0.288645541 | 0.530764833 | 0.704671084 | NS | 0.27509786 |
| LINC01776 | 0.559484556 | 0.345619153 | NA | NS | 0.461402198 |
| LINC01708 | 0.760603406 | 0.161069528 | NA | NS | 0.792986614 |
| RTCA-AS1 | -0.080129544 | 0.40548464 | 0.609064684 | NS | 0.392025592 |
| LINC01307 | 0.266628138 | 0.56229332 | NA | NS | 0.250037076 |
| LINC01709 | 0.573970672 | 0.281661944 | NA | NS | 0.550271828 |
| LINC01676 | 0.344644862 | 0.539585871 | NA | NS | 0.267939431 |
| LINC01677 | 0.156580534 | 0.753575414 | NA | NS | 0.122873279 |
| LINC01661 | 0.322002891 | 0.541657478 | NA | NS | 0.266275256 |
| VAV3-AS1 | 0.139990442 | 0.20006096 | 0.429790861 | NS | 0.698837652 |
| LINC02785 | -0.248147176 | 0.349562218 | NA | NS | 0.456475514 |
| TMEM167B-DT | 0.226450147 | 0.067162663 | 0.266995195 | NS | 1.172872089 |
| LINC01768 | 0.583891843 | 0.270366214 | NA | NS | 0.568047581 |
| LINC01397 | 0.653796272 | 0.204475072 | NA | NS | 0.689359629 |
| SLC6A17-AS1 | 0.85935023 | 0.111918071 | NA | NS | 0.951099783 |
| LINC02586 | 0.27076806 | 0.336491558 | NA | NS | 0.473025826 |
| RBM15-AS1 | -0.008055731 | 0.957660334 | 0.976655159 | NS | 0.0187885 |
| LAMTOR5-AS1 | 0.03568911 | 0.622629833 | 0.769700336 | NS | 0.205770074 |
| CYMP-AS1 | 1.160221737 | 0.032181818 | NA | Up | 1.492389428 |
| LINC01160 | 0.605099638 | 0.043263521 | NA | Up | 1.363878141 |
| INKA2-AS1 | 0.215694756 | 0.163145273 | 0.390872794 | NS | 0.787425506 |
| KCND3-IT1 | 0.467861019 | 0.533125734 | NA | NS | 0.273170354 |
| KCND3-AS1 | 0.09393161 | 0.864132757 | NA | NS | 0.063419532 |
| LINC01750 | 0.339949571 | 0.234075538 | 0.46228643 | NS | 0.63064397 |
| LINC02884 | -0.25078864 | 0.120471755 | 0.341212484 | NS | 0.919114761 |
| PPM1J-DT | 0.76045347 | 0.15564705 | NA | NS | 0.807859105 |
| LINC01356 | 0.953292959 | 0.050733207 | NA | NS | 1.294707681 |
| LINC01357 | 1.270757534 | 0.014582457 | NA | Up | 1.836169303 |
| SLC16A1-AS1 | -0.120320569 | 0.516967629 | NA | NS | 0.28653665 |
| LRIG2-DT | 0.873081337 | 0.038599283 | NA | Up | 1.413420766 |
| AP4B1-AS1 | 0.058328925 | 0.465415916 | 0.655653276 | NS | 0.332158769 |
| HIPK1-AS1 | 0.094206786 | 0.390333548 | 0.59729185 | NS | 0.408564121 |
| LINC01765 | 0.217793353 | 0.172388067 | 0.401144432 | NS | 0.763492801 |
| NGF-AS1 | 0.562558565 | 0.273723277 | NA | NS | 0.562688269 |
| LINC01649 | 0.366019371 | 0.40838147 | NA | NS | 0.388933971 |
| LINC01779 | 0.573907592 | 0.282147005 | NA | NS | 0.549524555 |
| ATP1A1-AS1 | 0.049298787 | 0.359934601 | 0.572764191 | NS | 0.443776402 |
| LINC01762 | 0.094202398 | 0.469332738 | 0.658457386 | NS | 0.328519151 |
| LINC02868 | 0.0835298 | 0.899401446 | NA | NS | 0.046046418 |
| LINC01525 | 0.769562093 | 0.166166051 | NA | NS | 0.779457701 |
| WARS2-IT1 | 0.282754643 | 0.155393787 | 0.382714082 | NS | 0.808566348 |
| WARS2-AS1 | 0.094109567 | 0.190831124 | 0.420152441 | NS | 0.719350791 |
| LINC01780 | 0.2652383 | 0.595325612 | NA | NS | 0.225245433 |
| HAO2-IT1 | 0.534920455 | 0.320181657 | NA | NS | 0.494603552 |
| LINC00622 | 0.320651272 | 0.100056098 | NA | NS | 0.999756439 |
| LINC00623 | 0.025448019 | 0.78140459 | 0.873772175 | NS | 0.107124042 |
| SRGAP2-AS1 | 0.097502597 | 0.611602548 | NA | NS | 0.213530714 |
| LINC02798 | 0.268092396 | 0.282963781 | 0.505888876 | NS | 0.54826915 |
| LINC01691 | -0.040975226 | 0.854537409 | NA | NS | 0.06826892 |
| LINC02591 | 0.160081604 | 0.824470492 | NA | NS | 0.083824883 |
| LIX1L-AS1 | 0.074043564 | 0.539950403 | 0.710319037 | NS | 0.267646131 |
| LINC01719 | 0.322320594 | 0.144462115 | 0.36967266 | NS | 0.84024603 |
| LINC00624 | 0.266227147 | 0.007148974 | 0.111200182 | Up | 2.145756288 |
| LINC02804 | 0.306357317 | 0.172409577 | NA | NS | 0.763438614 |
| LINC02805 | 0.380422668 | 0.175015909 | 0.404321539 | NS | 0.756922471 |
| LINC01731 | 0.737196542 | 0.183542951 | NA | NS | 0.736262289 |
| LINC01138 | 0.163107806 | 0.029122572 | 0.190232923 | Up | 1.535770275 |
| LINC02806 | 0.739442315 | 0.164564612 | NA | NS | 0.783663551 |
| FALEC | 0.213736657 | 0.348859278 | NA | NS | 0.457349722 |
| ADAMTSL4-AS2 | 0.039818228 | 0.809413911 | 0.890440969 | NS | 0.091829336 |
| ADAMTSL4-AS1 | 0.180840063 | 0.210133743 | 0.440111431 | NS | 0.677504204 |
| ZNF687-AS1 | 0.252719589 | 0.171856287 | NA | NS | 0.764834576 |
| RFX5-AS1 | 0.046463143 | 0.850389434 | NA | NS | 0.070382145 |
| TDRKH-AS1 | 0.187939127 | 0.267419492 | NA | NS | 0.57280694 |
| C2CD4D-AS1 | -0.134767427 | 0.155298698 | 0.382558975 | NS | 0.808832185 |
| FLG-AS1 | 0.340809592 | 0.311201867 | 0.530925593 | NS | 0.506957806 |
| LINC01527 | 0.885885814 | 0.135142278 | NA | NS | 0.869208764 |
| IL6R-AS1 | 0.026770375 | 0.799546318 | 0.884650476 | NS | 0.097156372 |
| UBE2Q1-AS1 | -0.022243373 | 0.835505091 | 0.907203565 | NS | 0.0780509 |
| DCST1-AS1 | 0.174980005 | 0.61850122 | NA | NS | 0.208659439 |
| THBS3-AS1 | 0.08390552 | 0.646072724 | 0.786192259 | NS | 0.189718594 |
| RUSC1-AS1 | 0.097101608 | 0.218793061 | 0.448248137 | NS | 0.659966456 |
| ASH1L-IT1 | 0.015334025 | 0.934645852 | 0.963896938 | NS | 0.029352917 |
| ASH1L-AS1 | -0.108427066 | 0.138580566 | 0.363039337 | NS | 0.858297669 |
| ARHGEF2-AS2 | 0.165747405 | 0.162861959 | 0.390602638 | NS | 0.788180345 |
| ARHGEF2-AS1 | 0.671521351 | 0.104709367 | NA | NS | 0.980014466 |
| MIR9-1HG | 0.311662526 | 0.181690532 | 0.411702441 | NS | 0.740667703 |
| BCAN-AS1 | 0.917277427 | 0.125244432 | NA | NS | 0.902241571 |
| LINC02772 | -0.233090632 | 0.29100233 | 0.513763133 | NS | 0.536103534 |
| KIRREL1-IT1 | 0.616124733 | 0.25905727 | NA | NS | 0.586604215 |
| LINC01704 | 0.456310894 | 0.300227881 | NA | NS | 0.522548979 |
| CADM3-AS1 | 0.689853191 | 0.200757293 | NA | NS | 0.697328669 |
| LINC02819 | 0.063206338 | 0.918602276 | NA | NS | 0.036872483 |
| SNHG28 | 0.756280802 | 0.038682329 | NA | Up | 1.412487388 |
| LINC01133 | 0.52706852 | 0.180100476 | 0.410006074 | NS | 0.74448514 |
| NECTIN4-AS1 | 0.670630517 | 0.278787786 | NA | NS | 0.554726257 |
| UAP1-DT | -0.072030252 | 0.789308227 | NA | NS | 0.102753371 |
| RGS5-AS1 | 0.2513784 | 0.41857336 | 0.618901614 | NS | 0.378228415 |
| PBX1-AS1 | 0.45762894 | 0.355623066 | NA | NS | 0.449010078 |
| LMX1A-AS2 | 0.692750126 | 0.3128109 | NA | NS | 0.504718122 |
| LMX1A-AS1 | 0.719221437 | 0.164247769 | NA | NS | 0.78450052 |
| LRRC52-AS1 | 0.65564095 | 0.168791447 | 0.396950027 | NS | 0.772649564 |
| TMCO1-AS1 | -0.087898193 | 0.410518134 | 0.613347918 | NS | 0.386667653 |
| FAM78B-AS1 | 0.835968324 | 0.101657238 | NA | NS | 0.992861693 |
| LINC01675 | -0.112884187 | 0.509387085 | 0.688583581 | NS | 0.29295207 |
| LINC01363 | 0.687318641 | 0.18820038 | NA | NS | 0.725379505 |
| POU2F1-DT | 0.720770315 | 0.086160913 | NA | NS | 1.064689709 |
| LINC00626 | -0.107518166 | 0.816184368 | NA | NS | 0.088211727 |
| LINC00970 | 1.04293883 | 0.034065813 | NA | Up | 1.467681245 |
| LINC01681 | 0.373873853 | 0.414016478 | NA | NS | 0.382982373 |
| LINC01142 | 0.453999366 | 0.407488075 | NA | NS | 0.389885096 |
| GORAB-AS1 | 0.030211392 | 0.918844641 | 0.955043049 | NS | 0.036757913 |
| DNM3-IT1 | 0.456646617 | 0.169554329 | NA | NS | 0.770691118 |
| DNM3OS | 0.108167392 | 0.598073701 | 0.752876596 | NS | 0.223245294 |
| PRDX6-AS1 | 0.129439337 | 0.122753832 | 0.343571896 | NS | 0.91096494 |
| GAS5 | -0.270987185 | 0.102359673 | 0.31812203 | NS | 0.989871109 |
| GAS5-AS1 | -0.053452809 | 0.527160452 | 0.702079431 | NS | 0.278057178 |
| RC3H1-IT1 | 0.160487033 | 0.073544976 | 0.275712399 | NS | 1.133446988 |
| LINC02776 | 0.537148923 | 0.018047456 | NA | Up | 1.743584001 |
| RABGAP1L-DT | -0.012304545 | 0.949123046 | 0.972477451 | NS | 0.022677481 |
| RABGAP1L-IT1 | 0.450765989 | 0.016081243 | 0.150228934 | Up | 1.793680377 |
| RABGAP1L-AS1 | 0.330512442 | 0.064840566 | 0.264064926 | NS | 1.188153205 |
| TNR-IT1 | 0.601266775 | 0.280322356 | NA | NS | 0.552342266 |
| LINC01657 | 0.340526143 | 0.179785423 | NA | NS | 0.745245524 |
| LINC02803 | 0.345532751 | 0.018560394 | 0.158608258 | Up | 1.73141282 |
| LINC01645 | 0.562172611 | 0.341051008 | NA | NS | 0.467180663 |
| LINC01741 | 0.384293989 | 0.48634535 | NA | NS | 0.313055232 |
| CRYZL2P-SEC16B | 0.764770448 | 0.013575309 | NA | Up | 1.867250273 |
| RASAL2-AS1 | 0.683569395 | 0.135471553 | NA | NS | 0.868151891 |
| C1orf220 | 0.134715146 | 0.236772546 | 0.464698333 | NS | 0.625668656 |
| RALGPS2-AS1 | 0.248903571 | 0.041871608 | 0.220548198 | Up | 1.378080362 |
| LINC02818 | 0.368133652 | 0.111860148 | NA | NS | 0.951324612 |
| OVAAL | 0.581602325 | 0.305118025 | NA | NS | 0.515532136 |
| LINC02816 | 0.678077397 | 0.088999667 | NA | NS | 1.05061162 |
| KIAA1614-AS1 | 0.387596688 | 0.164831428 | NA | NS | 0.782959979 |
| LINC01732 | 0.452723475 | 0.184914039 | NA | NS | 0.733030114 |
| LINC01699 | 0.810591504 | 0.13051548 | NA | NS | 0.884337977 |
| LINC01344 | 0.387157532 | 0.113847879 | 0.332423088 | NS | 0.943675058 |
| LINC00272 | 0.245435468 | 0.40470907 | NA | NS | 0.392857062 |
| LINC01686 | 0.571757519 | 0.111577112 | NA | NS | 0.952424885 |
| LINC01688 | 0.654049819 | 0.459032902 | NA | NS | 0.338156185 |
| LAMC1-AS1 | 0.118238294 | 0.66546213 | NA | NS | 0.176876654 |
| SMG7-AS1 | 0.281867351 | 0.108671993 | 0.326657827 | NS | 0.963882369 |
| C1orf21-DT | 0.765108689 | 0.029797542 | NA | Up | 1.525819565 |
| LINC01633 | 1.020239039 | 0.143781105 | NA | NS | 0.842298182 |
| LINC01350 | 0.439534039 | 0.288516928 | NA | NS | 0.5398287 |
| PDC-AS1 | 0.153427068 | 0.088411257 | 0.298422198 | NS | 1.053492433 |
| PACERR | -0.015847418 | 0.913248974 | 0.951687128 | NS | 0.039410807 |
| LINC01036 | -0.090254088 | 0.66348351 | 0.798539598 | NS | 0.178169867 |
| LINC01035 | 0.1497566 | 0.83058503 | NA | NS | 0.080615901 |
| LINC01701 | 1.200985185 | 0.132234657 | NA | NS | 0.878654707 |
| LINC01351 | 0.89376594 | 0.135258354 | NA | NS | 0.868835901 |
| LINC01720 | 0.264074821 | 0.658271056 | NA | NS | 0.18159524 |
| LINC01680 | 0.889491616 | 0.202241834 | NA | NS | 0.694129005 |
| LINC02770 | 0.2336258 | 0.454704875 | NA | NS | 0.342270389 |
| LINC01031 | 0.519125597 | 0.034207842 | 0.203152353 | Up | 1.465874326 |
| LINC01724 | 0.112008893 | 0.80276924 | NA | NS | 0.095409277 |
| MIR181A1HG | 0.046294704 | 0.546653273 | 0.715111285 | NS | 0.262288047 |
| LINC01222 | 0.009982354 | 0.977950569 | NA | NS | 0.009683096 |
| LINC01221 | 0.487622851 | 0.372917789 | NA | NS | 0.428386899 |
| LINC02789 | 0.142853894 | 0.809833453 | NA | NS | 0.091604287 |
| LINC00862 | 0.287923213 | 0.030276356 | 0.193469884 | Up | 1.518896402 |
| DDX59-AS1 | 1.403960128 | 0.005732417 | NA | Up | 2.241662199 |
| IPO9-AS1 | 0.268525725 | 0.32000769 | NA | NS | 0.494839586 |
| ELF3-AS1 | 0.040647354 | 0.807736425 | 0.889232573 | NS | 0.092730332 |
| PCAT6 | 0.79859258 | 0.080801187 | NA | NS | 1.092582261 |
| LINC01353 | 0.037290784 | 0.821907651 | 0.89852789 | NS | 0.085176977 |
| LINC01136 | 0.194067161 | 0.193575994 | 0.422794379 | NS | 0.713148502 |
| LINC00303 | 0.626547493 | 0.335236712 | NA | NS | 0.474648427 |
| ERLNC1 | 1.007318435 | 0.039135637 | NA | Up | 1.407427597 |
| LINC00628 | 0.981496521 | 0.057600146 | NA | NS | 1.239576418 |
| TMCC2-AS1 | 0.322239692 | 0.427744046 | NA | NS | 0.368816027 |
| LEMD1-AS1 | 0.747407813 | 0.169188762 | NA | NS | 0.771628487 |
| LEMD1-DT | 0.70860444 | 0.125404871 | NA | NS | 0.901685593 |
| C1orf147 | 0.08861601 | 0.377803551 | 0.58780278 | NS | 0.422733964 |
| DYRK3-AS1 | 0.713055155 | 0.152853004 | NA | NS | 0.815726023 |
| MIR29B2CHG | 0.10504601 | 0.35069115 | 0.564808607 | NS | 0.455075194 |
| LINC02767 | 0.496676665 | 0.170512596 | 0.398884521 | NS | 0.768243534 |
| LINC01735 | 0.33242685 | 0.186238082 | 0.415835484 | NS | 0.729931509 |
| LINC02769 | 0.023016496 | 0.948453994 | NA | NS | 0.02298373 |
| LINC01717 | 0.455407814 | 0.33744274 | NA | NS | 0.471799911 |
| LINC01774 | -0.089855925 | 0.886389449 | NA | NS | 0.052375422 |
| LINC01696 | 0.367508723 | 0.546390789 | NA | NS | 0.262496631 |
| LINC01698 | 0.320453111 | 0.455739417 | NA | NS | 0.341283407 |
| MIR205HG | 0.533503333 | 0.321701783 | NA | NS | 0.492546533 |
| HSD11B1-AS1 | 0.118318243 | 0.521729306 | NA | NS | 0.282554768 |
| KCNH1-IT1 | 0.865644329 | 0.142576812 | NA | NS | 0.845951101 |
| LINC00467 | -0.004543119 | 0.961478455 | 0.978517825 | NS | 0.017060443 |
| LINC01693 | 0.546477204 | 0.343593654 | NA | NS | 0.463954866 |
| LPGAT1-AS1 | 0.631075487 | 0.060038909 | NA | NS | 1.221567207 |
| LINC02608 | 0.10711361 | 0.474135186 | 0.662273971 | NS | 0.324097814 |
| LINC02771 | 0.023187286 | 0.974719798 | NA | NS | 0.011120213 |
| LINC01740 | 0.515186136 | 0.233154686 | NA | NS | 0.632355852 |
| LINC02773 | 0.225950145 | 0.322033338 | NA | NS | 0.492099166 |
| FLVCR1-DT | 0.061208571 | 0.674001248 | 0.805887887 | NS | 0.171339299 |
| PROX1-AS1 | 0.43857333 | 0.30958468 | 0.530022302 | NS | 0.509220539 |
| LINC00538 | 0.702624045 | 0.176426472 | NA | NS | 0.753436249 |
| LINC02775 | 0.276017687 | 0.572432082 | NA | NS | 0.242276034 |
| USH2A-AS2 | 0.369583779 | 0.621024219 | NA | NS | 0.206891462 |
| USH2A-AS1 | -0.003861051 | 0.995469501 | NA | NS | 0.001972041 |
| SPATA17-AS1 | 0.245504522 | 0.751570395 | NA | NS | 0.124030336 |
| LINC00210 | 0.355964782 | 0.574571723 | NA | NS | 0.240655751 |
| LINC01653 | 0.784018569 | 0.222448548 | NA | NS | 0.652770425 |
| TGFB2-AS1 | 0.145755527 | 0.524294763 | NA | NS | 0.28042448 |
| LINC02869 | 0.405384385 | 0.414922254 | NA | NS | 0.382033272 |
| LINC01710 | 0.170453636 | 0.755252645 | NA | NS | 0.121907745 |
| LYPLAL1-DT | 0.004007197 | 0.978901297 | 0.98756872 | NS | 0.009261096 |
| LYPLAL1-AS1 | 0.343520148 | 0.217790236 | NA | NS | 0.661961595 |
| LINC02779 | 0.793917703 | 0.275223023 | NA | NS | 0.560315239 |
| RNU6ATAC35P | 0.507519153 | 0.091007916 | NA | NS | 1.040920828 |
| LINC01352 | 0.378470772 | 0.089188998 | 0.299549938 | NS | 1.049688714 |
| HLX-AS1 | 0.171162981 | 0.589642101 | NA | NS | 0.229411515 |
| LINC02817 | 0.980552904 | 0.072779583 | NA | NS | 1.137990438 |
| LINC01655 | 0.32150938 | 0.378555208 | NA | NS | 0.421870774 |
| LINC02257 | 0.103042988 | 0.704629812 | NA | NS | 0.152038986 |
| LINC02474 | 0.131987405 | 0.682432425 | NA | NS | 0.165940346 |
| LINC01705 | 0.692962584 | 0.076196466 | NA | NS | 1.118065173 |
| TAF1A-AS1 | -0.072588829 | 0.689536839 | 0.81541388 | NS | 0.161442526 |
| CNIH3-AS2 | 0.571298936 | 0.093820891 | NA | NS | 1.027700448 |
| CNIH3-AS1 | 0.540104594 | 0.349952183 | NA | NS | 0.455991293 |
| LINC02813 | 0.602569481 | 0.236798778 | NA | NS | 0.625620543 |
| LINC02765 | 0.538069103 | 0.061367866 | NA | NS | 1.212058982 |
| H3-3A-DT | 0.25516619 | 0.007515876 | 0.113019428 | Up | 2.124020404 |
| LINC01703 | 0.522953278 | 0.149565639 | NA | NS | 0.825168168 |
| ACBD3-AS1 | 0.064833805 | 0.593741478 | 0.749772128 | NS | 0.226402611 |
| ITPKB-IT1 | 0.057969532 | 0.527754751 | 0.702589043 | NS | 0.277567849 |
| ITPKB-AS1 | 0.149389431 | 0.230543164 | 0.458835215 | NS | 0.63724775 |
| LINC01641 | 0.289165004 | 0.601602806 | NA | NS | 0.220690147 |
| SNAP47-AS1 | 0.534842068 | 0.010945124 | NA | Up | 1.96077932 |
| LINC02809 | 0.127293177 | 0.248656259 | 0.476232204 | NS | 0.604400605 |
| IBA57-DT | 0.293092161 | 0.121988794 | NA | NS | 0.913680062 |
| OBSCN-AS1 | 0.007941522 | 0.968549969 | 0.982455456 | NS | 0.013877968 |
| LINC02815 | 0.830741557 | 0.038705135 | NA | Up | 1.41223141 |
| LINC02814 | 0.349745345 | 0.403354821 | 0.60738002 | NS | 0.394312747 |
| TMEM78 | 0.42886395 | 0.373743266 | NA | NS | 0.427426624 |
| LINC01682 | 0.416134784 | 0.379056227 | NA | NS | 0.421296365 |
| LINC01736 | 0.050668354 | 0.871248008 | NA | NS | 0.059858202 |
| LINC01737 | 1.062024542 | 0.021162766 | NA | Up | 1.67442758 |
| TRIM67-AS1 | 1.077873577 | 0.104058267 | NA | NS | 0.982723411 |
| LINC00582 | 0.16422235 | 0.636072406 | NA | NS | 0.196493445 |
| DISC1-IT1 | 0.075621824 | 0.706993073 | 0.826665892 | NS | 0.150584841 |
| LINC01745 | 1.090824995 | 0.156554714 | NA | NS | 0.805333851 |
| LINC01744 | 0.294777044 | 0.563513621 | NA | NS | 0.249095582 |
| SLC35F3-AS1 | 0.388792713 | 0.381818689 | NA | NS | 0.418142818 |
| COA6-AS1 | -0.158394372 | 0.286061123 | 0.509186875 | NS | 0.543541161 |
| LINC01354 | 0.660869782 | 0.236226783 | NA | NS | 0.626670864 |
| LINC00184 | -0.077527846 | 0.861651499 | NA | NS | 0.064668352 |
| LINC01132 | 0.064067337 | 0.830373788 | 0.903626518 | NS | 0.080726368 |
| PP2672 | 0.412497424 | 0.323336191 | NA | NS | 0.490345682 |
| LINC01348 | 0.444502034 | 0.082987848 | 0.290260272 | NS | 1.080985496 |
| LYST-AS1 | 0.116465444 | 0.44057295 | 0.635380278 | NS | 0.355982171 |
| LINC02768 | -0.087083515 | 0.824698644 | NA | NS | 0.08370472 |
| LGALS8-AS1 | -0.027113566 | 0.842722167 | 0.911681082 | NS | 0.074315582 |
| LINC01139 | 0.228799526 | 0.659671157 | NA | NS | 0.180672504 |
| CHRM3-AS2 | 0.129654951 | 0.313488351 | 0.533197936 | NS | 0.503778593 |
| CHRM3-AS1 | 0.566969064 | 0.477428325 | NA | NS | 0.321091819 |
| LINC02774 | 0.193697298 | 0.183520665 | 0.413733592 | NS | 0.736315026 |
| KIF26B-AS1 | 1.216179523 | 0.041923738 | NA | Up | 1.37754 |
| SMYD3-AS1 | 0.250610529 | 0.060693521 | 0.256526216 | NS | 1.216857665 |
| SMYD3-IT1 | 0.226660915 | 0.176978913 | 0.406740698 | NS | 0.752078477 |
| LINC01743 | 0.080767104 | 0.89337645 | NA | NS | 0.0489655 |
| LINC01341 | 0.143457713 | 0.496100656 | 0.678854657 | NS | 0.304430198 |
| LINC02897 | 0.600895249 | 0.24127867 | NA | NS | 0.617481069 |
| LINC01865 | 0.155665514 | 0.671883911 | NA | NS | 0.172705759 |
| LINC01874 | 0.323684592 | 0.631630325 | NA | NS | 0.199537027 |
| LINC01875 | 0.849973879 | 0.28158676 | NA | NS | 0.550387769 |
| TMEM18-DT | 0.245672946 | 0.398518788 | 0.603470335 | NS | 0.399551199 |
| LINC01115 | 0.178102764 | 0.642562235 | NA | NS | 0.192084803 |
| LINC01939 | -0.084124248 | 0.804328987 | NA | NS | 0.094566279 |
| SNTG2-AS1 | 0.413957235 | 0.121741132 | NA | NS | 0.914562663 |
| MYT1L-AS1 | 0.677662653 | 0.170908493 | NA | NS | 0.767236355 |
| LINC01250 | 0.453858878 | 0.134015161 | NA | NS | 0.872846069 |
| EIPR1-IT1 | 0.073690975 | 0.667735758 | 0.801324897 | NS | 0.175395366 |
| RNASEH1-AS1 | -0.016365974 | 0.870956344 | 0.927147076 | NS | 0.060003613 |
| LINC01304 | 0.554316116 | 0.278051419 | NA | NS | 0.555874884 |
| LINC01249 | 0.601029984 | 0.314266973 | NA | NS | 0.502701257 |
| LINC01248 | 1.00775771 | 0.163638177 | NA | NS | 0.786115369 |
| LINC01810 | 0.851436917 | 0.245559342 | NA | NS | 0.609843539 |
| SILC1 | 0.477491093 | 0.242637775 | 0.470404666 | NS | 0.615041586 |
| LINC01247 | 0.633787434 | 0.178448673 | NA | NS | 0.748486676 |
| LINC01824 | 0.67271878 | 0.20535521 | NA | NS | 0.687494274 |
| MIR7515HG | 0.213503371 | 0.636497036 | 0.778637849 | NS | 0.196203614 |
| LINC00487 | 0.837534236 | 0.011024619 | NA | Up | 1.957636424 |
| NRIR | 0.385335849 | 0.036753382 | 0.209023715 | Up | 1.434702686 |
| GRASLND | 0.406809437 | 0.008921221 | 0.11966441 | Up | 2.049575688 |
| LINC01871 | 0.025798503 | 0.821781526 | 0.898521938 | NS | 0.085243626 |
| LINC00298 | 0.322752969 | 0.024046433 | 0.175665523 | Up | 1.618949333 |
| LINC00299 | 0.164897495 | 0.129746108 | 0.351821639 | NS | 0.88690566 |
| LINC01814 | 0.169207901 | 0.218967339 | 0.448429759 | NS | 0.659620659 |
| ID2-AS1 | 0.399395381 | 0.120429274 | 0.341134353 | NS | 0.919267931 |
| ODC1-DT | 0.098031187 | 0.354709754 | 0.567962161 | NS | 0.450126868 |
| RN7SL832P | 0.590918141 | 0.007280687 | NA | Up | 2.137827655 |
| LINC01954 | 0.570162966 | 0.187498878 | NA | NS | 0.727001328 |
| LINC00570 | -0.421892031 | 0.046597607 | 0.229846735 | Down | 1.331636388 |
| MIR3681HG | 0.22474245 | 0.305804636 | 0.526819451 | NS | 0.514555935 |
| LINC00276 | 0.395136674 | 0.452309153 | NA | NS | 0.344564624 |
| LINC01804 | 0.905486003 | 0.064179019 | NA | NS | 1.192606923 |
| MYCNOS | 0.380393708 | 0.407246545 | NA | NS | 0.390142591 |
| MYCNUT | 0.443321255 | 0.473875028 | NA | NS | 0.324336177 |
| GACAT3 | 1.058727853 | 0.039571182 | NA | Up | 1.40262098 |
| LINC01866 | 0.099326571 | 0.891461231 | NA | NS | 0.049897539 |
| LINC01376 | 0.138454956 | 0.184640718 | 0.414735359 | NS | 0.73367252 |
| LINC01808 | 0.188364534 | 0.659708212 | NA | NS | 0.18064811 |
| LINC00954 | 0.300195524 | 0.003621063 | 0.08688637 | Up | 2.441163965 |
| TTC32-DT | -0.190224578 | 0.428083994 | NA | NS | 0.36847101 |
| LAPTM4A-DT | -0.079719806 | 0.860133626 | NA | NS | 0.065434074 |
| HS1BP3-IT1 | 0.001349475 | 0.995943768 | NA | NS | 0.001765182 |
| LINC02850 | -0.165962001 | 0.754804448 | NA | NS | 0.122165549 |
| LINC01822 | 0.64333705 | 0.141273683 | NA | NS | 0.849938734 |
| LINC01830 | 0.686220232 | 0.292117842 | NA | NS | 0.534441917 |
| LINC01884 | 0.648568109 | 0.239371088 | NA | NS | 0.620928307 |
| DNAJC27-AS1 | 0.183376447 | 0.16298232 | 0.390737932 | NS | 0.787859505 |
| ARNILA | 0.153974511 | 0.275321292 | 0.499985735 | NS | 0.560160201 |
| DTNB-AS1 | 0.284387557 | 0.258557181 | NA | NS | 0.587443396 |
| MAPRE3-AS1 | 0.482358242 | 0.028700979 | 0.189026296 | Up | 1.542103283 |
| AGBL5-AS1 | 0.265059454 | 0.397412538 | NA | NS | 0.400758436 |
| AGBL5-IT1 | -0.284735213 | 0.029861083 | 0.192425018 | Down | 1.524894452 |
| GTF3C2-AS1 | 0.276623342 | 0.068855471 | 0.268899965 | NS | 1.162061547 |
| LINC01460 | 0.770494157 | 0.060593255 | NA | NS | 1.217575717 |
| FLJ31356 | -0.049697177 | 0.770032421 | 0.865925463 | NS | 0.113490989 |
| LINC01936 | 0.439701602 | 0.345156705 | NA | NS | 0.461983686 |
| LINC01946 | 0.342244289 | 0.404568262 | NA | NS | 0.39300819 |
| SLC30A6-DT | -0.235177313 | 0.213502175 | NA | NS | 0.670597697 |
| BIRC6-AS1 | 0.357803233 | 0.005008286 | 0.097847413 | Up | 2.300310922 |
| LINC00486 | 0.423182418 | 0.19714856 | 0.42687873 | NS | 0.70520639 |
| LINC01320 | 0.468356536 | 0.25136146 | 0.478209017 | NS | 0.59970131 |
| LINC01318 | 1.119557125 | 0.069377898 | NA | NS | 1.158778863 |
| LINC00211 | 0.495752973 | 0.003808143 | 0.088772476 | Up | 2.419286699 |
| RMDN2-AS1 | 0.359157309 | 0.017783228 | 0.155923237 | Up | 1.749989396 |
| CYP1B1-AS1 | 0.141950596 | 0.327150557 | 0.545260243 | NS | 0.485252336 |
| LINC02613 | 0.67603544 | 0.124654219 | 0.346185853 | NS | 0.904293019 |
| MAP4K3-DT | 0.324327911 | 0.055931886 | 0.249087638 | NS | 1.252340534 |
| SLC8A1-AS1 | 0.462167795 | 0.006483352 | 0.1080547 | Up | 2.188200384 |
| LINC01794 | 0.686221471 | 0.284265301 | NA | NS | 0.54627615 |
| LINC01913 | 0.422012291 | 0.367501016 | 0.579609412 | NS | 0.434741456 |
| LINC01914 | 0.573342206 | 0.340906895 | NA | NS | 0.467364215 |
| LINC02898 | 0.431668575 | 0.299598643 | NA | NS | 0.523460157 |
| EML4-AS1 | 0.110322043 | 0.126932222 | 0.348807379 | NS | 0.896428118 |
| LINC01819 | 0.098693926 | 0.679852287 | 0.809250368 | NS | 0.167585437 |
| LINC02590 | 0.749493285 | 0.139781446 | NA | NS | 0.854550473 |
| LINC02580 | -0.181446389 | 0.10270768 | 0.318624114 | NS | 0.988397079 |
| LINC01126 | 0.211634343 | 0.004998083 | 0.097847413 | Up | 2.301196527 |
| PPM1B-DT | 0.234489482 | 0.505427159 | NA | NS | 0.296341425 |
| LINC01833 | 0.41733153 | 0.336330119 | NA | NS | 0.473234239 |
| SIX3-AS1 | 0.178387221 | 0.776972514 | NA | NS | 0.109594344 |
| LINC01121 | 0.32852374 | 0.42639361 | 0.62458676 | NS | 0.370189312 |
| PRKCE-AS1 | 0.340026638 | 0.036856202 | 0.209195494 | Up | 1.433489419 |
| LINC01820 | 0.537506082 | 0.295431001 | NA | NS | 0.529543934 |
| LINC02583 | 0.101788521 | 0.833738557 | NA | NS | 0.078970114 |
| RHOQ-AS1 | 0.27037948 | 0.013431716 | 0.13912852 | Up | 1.871868503 |
| LINC01118 | 0.488506721 | 0.091996326 | 0.303889385 | NS | 1.036229518 |
| LINC01119 | 0.36909126 | 0.330528133 | NA | NS | 0.480791569 |
| EPCAM-DT | 0.495689408 | 0.202194973 | NA | NS | 0.694229647 |
| MSH2-OT1 | 0.086357887 | 0.531326982 | 0.704804549 | NS | 0.274638129 |
| PPP1R21-DT | 0.251837149 | 0.344547921 | NA | NS | 0.462750366 |
| LINC01867 | 0.411727621 | 0.60331498 | NA | NS | 0.219455891 |
| SPTBN1-AS1 | 0.46199794 | 0.443698946 | NA | NS | 0.352911603 |
| EML6-AS1 | 0.248345252 | 0.419754024 | NA | NS | 0.377005132 |
| MIR217HG | 0.682791637 | 0.206461217 | NA | NS | 0.685161517 |
| LINC01813 | 0.477869189 | 0.490194836 | NA | NS | 0.309631268 |
| LINC01795 | 0.91183641 | 0.10650798 | NA | NS | 0.972617853 |
| LINC01122 | 0.359810038 | 0.327534412 | 0.545461976 | NS | 0.484743065 |
| LINC01793 | 0.585561971 | 0.272131133 | NA | NS | 0.565221769 |
| MIR4432HG | -0.029975131 | 0.808898367 | 0.890130232 | NS | 0.092106041 |
| LINC01185 | 0.709650286 | 0.047117995 | NA | Up | 1.3268132 |
| USP34-DT | 0.119259272 | 0.112904414 | 0.331563123 | NS | 0.947289077 |
| LINC00309 | 0.225815978 | 0.441232799 | 0.635901677 | NS | 0.355332212 |
| LGALSL-DT | -0.087395598 | 0.663497075 | 0.798539598 | NS | 0.178160988 |
| LINC01805 | 0.690849631 | 0.289965725 | NA | NS | 0.537653334 |
| AFTPH-DT | -0.09754526 | 0.514602334 | 0.692659911 | NS | 0.288528248 |
| LINC02579 | 0.508090417 | 0.226924219 | NA | NS | 0.644119151 |
| LINC01800 | -0.07162432 | 0.702877802 | 0.823897401 | NS | 0.153120172 |
| LINC02245 | 0.023303572 | 0.82490706 | 0.900263116 | NS | 0.08359498 |
| LINC02576 | 0.119983704 | 0.365407638 | 0.577700243 | NS | 0.437222379 |
| LINC01873 | 0.468862747 | 0.332429194 | NA | NS | 0.478300843 |
| MEIS1-AS3 | 0.711085903 | 0.207667459 | NA | NS | 0.682631551 |
| MEIS1-AS2 | 0.211217451 | 0.485135569 | NA | NS | 0.314136883 |
| LINC01798 | 0.812725902 | 0.068026105 | NA | NS | 1.167324397 |
| LINC01797 | 1.169051562 | 0.023709719 | NA | Up | 1.625073594 |
| LINC01799 | 0.546577415 | 0.266881238 | NA | NS | 0.573681956 |
| LINC01628 | 0.377074131 | 0.624156724 | NA | NS | 0.204706347 |
| LINC01828 | 0.421378214 | 0.196797539 | 0.426481571 | NS | 0.705980336 |
| LINC01829 | 0.108713438 | 0.557256665 | 0.723076042 | NS | 0.253944728 |
| LINC02831 | 0.814213992 | 0.07907548 | NA | NS | 1.101958162 |
| LINC01812 | 0.692770964 | 0.207075743 | NA | NS | 0.683870771 |
| LINC01890 | 0.034627688 | 0.675120815 | 0.806240246 | NS | 0.170618502 |
| LINC01888 | 0.132158016 | 0.260405312 | 0.486523288 | NS | 0.584350161 |
| PCBP1-AS1 | 0.117035006 | 0.239333582 | 0.467600896 | NS | 0.620996359 |
| LINC01816 | 0.338987653 | 0.017953315 | 0.15641156 | Up | 1.745855337 |
| TGFA-IT1 | 0.412138311 | 0.12133463 | NA | NS | 0.916015231 |
| LINC01143 | 0.836675422 | 0.185122763 | NA | NS | 0.732540176 |
| ALMS1-IT1 | -0.015369287 | 0.8664664 | 0.924516097 | NS | 0.062248274 |
| DGUOK-AS1 | 0.290055898 | 0.001191873 | 0.056242293 | Up | 2.92377016 |
| BOLA3-AS1 | 0.259392211 | 3.75E-04 | 0.033152046 | Up | 3.426063306 |
| DCTN1-AS1 | 0.322715008 | 0.006573636 | 0.108312793 | Up | 2.182194367 |
| LBX2-AS1 | 0.038024583 | 0.852368838 | 0.916691765 | NS | 0.069372436 |
| HK2-DT | -0.356738051 | 0.015677131 | 0.148636863 | Down | 1.80473341 |
| LINC01291 | 0.166392159 | 0.664153248 | 0.798935311 | NS | 0.177731699 |
| LINC01293 | 0.313012312 | 0.419045553 | 0.619391222 | NS | 0.377738764 |
| EVA1A-AS | 0.325605712 | 0.538000446 | NA | NS | 0.269217365 |
| LRRTM4-AS1 | -0.125024528 | 0.842127274 | NA | NS | 0.074622267 |
| LINC01851 | 0.993464641 | 0.161172623 | NA | NS | 0.792708727 |
| CTNNA2-AS1 | 0.676986102 | 0.243104163 | NA | NS | 0.614207604 |
| LINC01815 | 0.494615966 | 0.402935205 | NA | NS | 0.394764786 |
| LINC01809 | 1.217991534 | 0.104299373 | NA | NS | 0.981718301 |
| LINC01964 | 0.579580898 | 0.06031223 | NA | NS | 1.219594615 |
| TCF7L1-IT1 | 1.589164314 | 0.036181141 | NA | Up | 1.441517746 |
| PARTICL | 0.378646003 | 0.011717721 | 0.132588561 | Up | 1.931156846 |
| ST3GAL5-AS1 | -0.044388493 | 0.688808614 | 0.815257617 | NS | 0.161901431 |
| ANAPC1P2 | 0.007140066 | 0.927475495 | 0.960047177 | NS | 0.032697556 |
| LINC01943 | 0.045675458 | 0.655920145 | 0.793339486 | NS | 0.18314903 |
| CYTOR | -0.039826365 | 0.593959483 | 0.749923374 | NS | 0.226243179 |
| EIF2AK3-DT | -0.040153233 | 0.773409188 | 0.867925187 | NS | 0.111590673 |
| LINC00342 | 0.029492442 | 0.770378736 | 0.866009611 | NS | 0.113295713 |
| STARD7-AS1 | 0.020512574 | 0.80466086 | 0.887737559 | NS | 0.094387123 |
| CNNM3-DT | -0.02094614 | 0.829682542 | 0.903193724 | NS | 0.081088048 |
| LINC02611 | 0.053731901 | 0.485887309 | 0.671513443 | NS | 0.313464444 |
| LINC01104 | 0.85766113 | 0.091893872 | NA | NS | 1.036713449 |
| LINC01849 | 0.694962267 | 0.160672597 | NA | NS | 0.794058186 |
| LINC01868 | 0.377288492 | 0.743822433 | NA | NS | 0.128530728 |
| NPAS2-AS1 | 0.614316896 | 0.176426722 | NA | NS | 0.753435634 |
| TBC1D8-AS1 | 0.182742046 | 0.424131543 | NA | NS | 0.372499427 |
| LINC01870 | 0.318049391 | 0.535664775 | NA | NS | 0.271106911 |
| LINC01127 | -0.030187728 | 0.857590111 | 0.91967171 | NS | 0.066720236 |
| IL1R1-AS1 | 0.160519361 | 0.664072466 | NA | NS | 0.177784526 |
| LINC01796 | 0.522180485 | 0.030897495 | 0.195241361 | Up | 1.510076731 |
| LINC01935 | 0.257874685 | 0.594472942 | NA | NS | 0.225867908 |
| LINC01965 | 0.567234245 | 0.236412982 | 0.46419154 | NS | 0.626328678 |
| LINC01831 | 0.655570417 | 0.315760611 | NA | NS | 0.500642046 |
| LINC01102 | 0.345111797 | 0.451294834 | 0.644288327 | NS | 0.345539638 |
| LINC01103 | 0.820914563 | 0.172470217 | NA | NS | 0.763285889 |
| LINC01114 | 0.630045187 | 0.220893888 | NA | NS | 0.655816301 |
| PANTR1 | 0.633991615 | 0.146054647 | 0.371561664 | NS | 0.835484621 |
| LINC01159 | 0.603531111 | 0.184945547 | NA | NS | 0.732956122 |
| MRPS9-AS2 | -0.035130502 | 0.860311566 | NA | NS | 0.065344238 |
| MRPS9-AS1 | 0.026519011 | 0.801702476 | 0.885829599 | NS | 0.095986775 |
| LINC01918 | 0.346176588 | 0.092694336 | NA | NS | 1.032946803 |
| C2orf49-DT | -0.005696436 | 0.946011901 | 0.970854819 | NS | 0.0241034 |
| ST6GAL2-IT1 | 0.813188239 | 0.426303577 | NA | NS | 0.370281024 |
| LINC01789 | 0.499150537 | 0.3490993 | NA | NS | 0.457051022 |
| LINC01885 | 0.51518211 | 0.313061489 | NA | NS | 0.504370354 |
| LINC01886 | 0.324718433 | 0.589247581 | NA | NS | 0.229702192 |
| GACAT1 | 0.562574251 | 0.372449672 | NA | NS | 0.428932403 |
| RGPD4-AS1 | 0.528967001 | 0.270892709 | NA | NS | 0.567202684 |
| LINC01593 | 1.323106932 | 0.136576846 | NA | NS | 0.864622922 |
| LINC01594 | 0.320174315 | 0.514651508 | NA | NS | 0.28848675 |
| GCC2-AS1 | 0.160304385 | 0.4735155 | NA | NS | 0.324665801 |
| LIMS1-AS1 | 0.146861268 | 0.21387887 | 0.443234232 | NS | 0.66983212 |
| SH3RF3-AS1 | 0.42182872 | 0.028029321 | 0.187577424 | Up | 1.552387416 |
| LINC01123 | 0.57984659 | 0.266888986 | NA | NS | 0.573669349 |
| LINC01106 | 0.544028188 | 0.352882889 | NA | NS | 0.4523694 |
| MIR4435-2HG | 0.232775613 | 0.013944592 | 0.141407346 | Up | 1.855594181 |
| ACOXL-AS1 | 0.356066313 | 0.068497483 | 0.268602708 | NS | 1.164325389 |
| SOCAR | 0.471466777 | 0.110086537 | NA | NS | 0.958265789 |
| PAX8-AS1 | 0.400519283 | 0.069195862 | 0.269500354 | NS | 1.159919879 |
| LINC01961 | 0.503631366 | 0.304734075 | NA | NS | 0.516078981 |
| PGM5P4-AS1 | 0.143052771 | 0.763143631 | NA | NS | 0.117393716 |
| FAM138B | 0.182545933 | 0.719249021 | NA | NS | 0.143120721 |
| ACTR3-AS1 | 0.113320958 | 0.065902053 | 0.265127437 | NS | 1.181101059 |
| LINC01191 | 0.261662353 | 0.016207489 | 0.150606807 | Up | 1.790284252 |
| DPP10-AS3 | 0.725954635 | 0.423704713 | NA | NS | 0.372936706 |
| DPP10-AS1 | 0.246737138 | 0.597021045 | 0.752047521 | NS | 0.22401036 |
| THORLNC | 0.268907731 | 0.349624731 | 0.563682195 | NS | 0.456397855 |
| LINC01956 | 0.537164192 | 0.307137253 | NA | NS | 0.512667504 |
| STEAP3-AS1 | 0.108133366 | 0.66461257 | 0.799245525 | NS | 0.177431449 |
| SCTR-AS1 | 0.345857367 | 0.486095144 | NA | NS | 0.313278717 |
| CLASP1-AS1 | 0.613506027 | 9.33E-04 | 0.050989731 | Up | 3.030248516 |
| NIFK-AS1 | 0.041211941 | 0.471165645 | 0.660178881 | NS | 0.326826383 |
| LINC01823 | 0.325181285 | 0.492875944 | NA | NS | 0.307262379 |
| LINC01826 | 0.597431063 | 0.265561337 | NA | NS | 0.575835154 |
| LINC01889 | 0.011541394 | 0.987927597 | NA | NS | 0.005274883 |
| LINC01941 | 0.815551511 | 0.140046936 | NA | NS | 0.853726389 |
| MAP3K2-DT | 0.062846772 | 0.576352173 | 0.737285229 | NS | 0.239312065 |
| LINC01854 | 0.56781085 | 0.292264048 | NA | NS | 0.534224604 |
| LINC02572 | 0.435847412 | 0.189514102 | 0.41894324 | NS | 0.722358468 |
| LINC01856 | 1.207598608 | 0.019837008 | NA | Up | 1.702523834 |
| LINC01120 | 0.380635152 | 0.454316521 | NA | NS | 0.34264147 |
| LINC01087 | 0.742847565 | 0.162377079 | NA | NS | 0.789475274 |
| C2orf27A | 0.380568943 | 0.01086349 | 0.12856202 | Up | 1.964030651 |
| LINC01945 | 0.66511083 | 0.466013642 | NA | NS | 0.331601369 |
| NCKAP5-AS1 | 0.594458089 | 0.254665748 | NA | NS | 0.594029462 |
| NCKAP5-AS2 | 0.821058672 | 0.097149431 | NA | NS | 1.01255974 |
| CCNT2-AS1 | 0.1709004 | 0.046369822 | 0.229315052 | Up | 1.333764571 |
| LCT-AS1 | 0.493827444 | 0.14919896 | NA | NS | 0.826234205 |
| DARS1-AS1 | 0.167191419 | 0.088215711 | 0.298122562 | NS | 1.054454059 |
| LINC01832 | 0.231703271 | 0.309898276 | NA | NS | 0.508780839 |
| LINC02631 | 0.19998458 | 0.1059365 | 0.322198901 | NS | 0.974954379 |
| LINC01853 | 0.396006199 | 0.625589348 | NA | NS | 0.203710654 |
| ARHGAP15-AS1 | 0.250920489 | 0.046326535 | 0.229299829 | Up | 1.334170181 |
| ZEB2-AS1 | -0.085645665 | 0.419298074 | 0.619573166 | NS | 0.377477132 |
| LINC01412 | -0.113162299 | 0.728608614 | NA | NS | 0.137505698 |
| TEX41 | 0.398001867 | 0.195479492 | 0.425153971 | NS | 0.708898799 |
| LINC01966 | 0.566987093 | 0.290933795 | NA | NS | 0.536205828 |
| LINC01911 | 0.719569589 | 0.152764788 | NA | NS | 0.815976739 |
| KIF5C-AS1 | 0.314381639 | 0.086116494 | 0.29485869 | NS | 1.064913661 |
| MMADHC-DT | 0.326973776 | 0.22115139 | 0.449806618 | NS | 0.655310326 |
| LINC01931 | 0.06088623 | 0.903847774 | NA | NS | 0.043904707 |
| LINC01818 | 0.252703294 | 0.685134854 | NA | NS | 0.164223938 |
| LINC01817 | 0.013819787 | 0.981080427 | NA | NS | 0.008295388 |
| LINC01920 | -0.082180168 | 0.874195905 | NA | NS | 0.058391232 |
| LINC02612 | 0.459240203 | 0.458894182 | NA | NS | 0.338287449 |
| LINC01850 | 0.486022969 | 0.406980176 | NA | NS | 0.390426744 |
| LINC01876 | 0.078513417 | 0.650695229 | 0.789521702 | NS | 0.186622378 |
| LINC01958 | 0.069776244 | 0.913904094 | NA | NS | 0.039099377 |
| UPP2-IT1 | -0.130325723 | 0.851376457 | NA | NS | 0.069878363 |
| CCDC148-AS1 | 0.350619057 | 0.470790227 | NA | NS | 0.327172561 |
| PKP4-AS1 | -0.007535143 | 0.962799145 | 0.979174299 | NS | 0.016464304 |
| LINC02478 | 0.183155458 | 0.13493362 | 0.35858085 | NS | 0.869879828 |
| TANK-AS1 | 0.382367688 | 0.010764773 | 0.12826482 | Up | 1.967995108 |
| LINC01806 | 0.018339955 | 0.879020136 | 0.931832955 | NS | 0.056001176 |
| DPP4-DT | 0.022778366 | 0.879411905 | 0.931951122 | NS | 0.05580766 |
| KCNH7-AS1 | 0.526554123 | 0.314845915 | NA | NS | 0.501901937 |
| TTC21B-AS1 | -0.017813335 | 0.87926976 | 0.931881903 | NS | 0.055877863 |
| SCN1A-AS1 | 0.330398497 | 0.256138077 | 0.483082784 | NS | 0.591525855 |
| XIRP2-AS1 | 0.626534259 | 0.444747359 | NA | NS | 0.351886622 |
| B3GALT1-AS1 | 0.157485677 | 0.654556249 | NA | NS | 0.184053027 |
| CERS6-AS1 | 0.583284591 | 0.033678608 | NA | Up | 1.472645861 |
| MYO3B-AS1 | 0.38725041 | 0.266879513 | 0.492869495 | NS | 0.573684763 |
| LINC01124 | 1.115605886 | 0.062588749 | NA | NS | 1.203503729 |
| DLX2-DT | 0.214793446 | 0.70648364 | NA | NS | 0.15089789 |
| ITGA6-AS1 | 0.13290438 | 0.124723212 | 0.346235762 | NS | 0.904052712 |
| RAPGEF4-AS1 | 0.637412675 | 0.017971727 | 0.15641156 | Up | 1.745410191 |
| MAP3K20-AS1 | 0.331563649 | 0.01616021 | 0.150325396 | Up | 1.791553004 |
| LINC01960 | 0.206713205 | 0.42673187 | NA | NS | 0.369844921 |
| LINC01305 | 0.393393357 | 0.319759104 | NA | NS | 0.495177082 |
| HOXD-AS2 | 0.628140616 | 0.330180326 | NA | NS | 0.481248809 |
| HAGLR | 0.493491185 | 0.305989685 | NA | NS | 0.514293214 |
| HAGLROS | 0.551556638 | 0.416971642 | NA | NS | 0.37989348 |
| LINC01117 | 0.377184443 | 0.383948452 | NA | NS | 0.415727079 |
| LINC01116 | 1.443383944 | 0.007519198 | NA | Up | 2.123828504 |
| PDE11A-AS1 | 0.152253051 | 0.719269769 | NA | NS | 0.143108193 |
| CHROMR | 0.083876562 | 0.154264821 | 0.381242357 | NS | 0.811733101 |
| TTN-AS1 | 0.066769804 | 0.405974563 | 0.609560706 | NS | 0.391501177 |
| SCHLAP1 | 0.694518624 | 0.23702059 | NA | NS | 0.625213924 |
| LINC01934 | 0.121942195 | 0.164734954 | 0.392382726 | NS | 0.783214241 |
| ITPRID2-DT | 0.238836139 | 0.229489928 | 0.457820413 | NS | 0.63923637 |
| FSIP2-AS1 | 0.219724069 | 0.374706808 | NA | NS | 0.426308416 |
| FSIP2-AS2 | 0.276139993 | 0.509672174 | NA | NS | 0.292709077 |
| LINC01473 | 0.089887589 | 0.389002815 | 0.596625801 | NS | 0.410047256 |
| LINC01090 | 0.314271843 | 0.550995414 | NA | NS | 0.258852016 |
| DIRC1 | 0.15985595 | 0.784827514 | NA | NS | 0.10522578 |
| OSGEPL1-AS1 | -0.043046041 | 0.711706067 | 0.829701824 | NS | 0.147699332 |
| NABP1-OT1 | 0.456611558 | 0.116954681 | NA | NS | 0.931982393 |
| CAVIN2-AS1 | 0.272836345 | 0.472091893 | 0.660669022 | NS | 0.325973457 |
| PCGEM1 | 0.09337411 | 0.893673759 | NA | NS | 0.048820994 |
| LINC01821 | 0.255722844 | 0.477976735 | 0.665691023 | NS | 0.320593242 |
| LINC01790 | 0.178988953 | 0.502838943 | NA | NS | 0.298571095 |
| LINC01825 | 0.216534054 | 0.11422641 | 0.332667558 | NS | 0.942233471 |
| LINC01827 | 0.734425626 | 0.155955181 | NA | NS | 0.807000193 |
| HECW2-AS1 | -0.190962779 | 0.250885875 | 0.47774934 | NS | 0.600523789 |
| ANKRD44-AS1 | 0.069310032 | 0.488732257 | 0.673300418 | NS | 0.310928996 |
| ANKRD44-IT1 | 0.177768153 | 0.120124276 | 0.340734879 | NS | 0.920369218 |
| LINC01923 | 0.35698022 | 0.321557244 | NA | NS | 0.492741703 |
| SATB2-AS1 | 0.598978081 | 0.076087724 | NA | NS | 1.118685404 |
| LINC01877 | 0.074202919 | 0.82552033 | 0.900717799 | NS | 0.083272227 |
| LINC01792 | 0.648253165 | 0.095162478 | NA | NS | 1.021534255 |
| BZW1-AS1 | 1.304864386 | 8.39E-04 | NA | Up | 3.076128554 |
| CFLAR-AS1 | 0.255093502 | 0.008722633 | 0.118782974 | Up | 2.059352397 |
| KIAA2012-AS1 | 0.560433471 | 0.266425088 | NA | NS | 0.574424882 |
| KLF7-IT1 | 0.108071016 | 0.306742752 | 0.527638781 | NS | 0.513225691 |
| MYOSLID | 0.047037102 | 0.790795397 | 0.878951525 | NS | 0.101935867 |
| LINC01802 | 0.311429399 | 0.438778756 | NA | NS | 0.357754408 |
| LINC01857 | -0.236881592 | 0.1454606 | 0.37059965 | NS | 0.837254624 |
| IDH1-AS1 | 0.610687057 | 0.147943622 | NA | NS | 0.829903753 |
| KANSL1L-AS1 | 0.153393155 | 0.063230637 | 0.261404175 | NS | 1.199072443 |
| LANCL1-AS1 | 0.067987605 | 0.482382581 | 0.668574551 | NS | 0.316608383 |
| CPS1-IT1 | 0.228969859 | 0.645514004 | NA | NS | 0.190094331 |
| LINC01878 | 0.280441924 | 0.210165899 | 0.440111431 | NS | 0.67743775 |
| LINC01953 | -0.017685176 | 0.963111875 | NA | NS | 0.016323262 |
| SPAG16-DT | 0.56821041 | 0.010911106 | NA | Up | 1.962131219 |
| VWC2L-IT1 | 0.348932283 | 0.29421084 | NA | NS | 0.531341329 |
| SNHG31 | 0.650728068 | 0.009574352 | NA | Up | 2.018890622 |
| LINC02862 | 1.09861049 | 0.156782013 | NA | NS | 0.804703763 |
| LINC00607 | 0.478977776 | 0.118233995 | 0.338601345 | NS | 0.927257635 |
| LINC01614 | 0.281156615 | 0.558883657 | NA | NS | 0.25267859 |
| LINC01963 | 0.157788317 | 0.055901295 | 0.249087638 | NS | 1.252578129 |
| SMARCAL1-AS1 | 0.806667048 | 0.089705656 | NA | NS | 1.047180176 |
| RPL37A-DT | 0.204559486 | 0.107041261 | 0.323901028 | NS | 0.970448785 |
| LINC01280 | 0.339723186 | 0.567874652 | NA | NS | 0.245747516 |
| DIRC3-AS1 | 0.179553344 | 0.327079252 | 0.545259065 | NS | 0.485347004 |
| DIRC3 | 0.055388111 | 0.648579859 | 0.787998723 | NS | 0.188036542 |
| TNS1-AS1 | 0.787610444 | 0.149565142 | NA | NS | 0.825169611 |
| CATIP-AS2 | 0.852386508 | 0.063192011 | NA | NS | 1.199337825 |
| CATIP-AS1 | 0.110600694 | 0.677284139 | NA | NS | 0.169229095 |
| LINC01494 | 0.585445796 | 0.171144218 | NA | NS | 0.76663777 |
| LINC00608 | 0.593760156 | 0.216097478 | NA | NS | 0.665350301 |
| ASIC4-AS1 | 0.53156309 | 0.263541364 | NA | NS | 0.57915121 |
| LINC02832 | 0.542662582 | 0.324844152 | NA | NS | 0.488324947 |
| LINC01803 | 0.966944515 | 0.154749806 | NA | NS | 0.810369886 |
| CCDC140 | 0.694085602 | 0.126630859 | NA | NS | 0.897460449 |
| ACSL3-AS1 | 0.280297171 | 0.287338699 | NA | NS | 0.541605879 |
| MFF-DT | 0.207427538 | 0.196176658 | 0.425986774 | NS | 0.707352669 |
| LINC01807 | 0.437655894 | 0.418791553 | NA | NS | 0.378002087 |
| FBXO36-IT1 | 0.351827659 | 0.43591698 | NA | NS | 0.360596214 |
| LINC01907 | 0.213520323 | 0.382570842 | NA | NS | 0.417288134 |
| GCSIR | 0.272460903 | 0.223152278 | 0.451870269 | NS | 0.651398675 |
| SPATA3-AS1 | 0.313971685 | 0.283262625 | 0.506225567 | NS | 0.547810724 |
| LINC00471 | 0.309228009 | 0.141930941 | NA | NS | 0.847922918 |
| LINC01891 | 0.070177934 | 0.608806716 | 0.76003769 | NS | 0.215520565 |
| LINC01173 | 0.587518252 | 0.404988774 | NA | NS | 0.392557015 |
| AGAP1-IT1 | 0.401725941 | 0.252435346 | NA | NS | 0.597849835 |
| KLHL30-AS1 | 0.785963734 | 0.114304794 | NA | NS | 0.941935555 |
| LINC02610 | 0.495865576 | 0.028879714 | 0.189677769 | Up | 1.539407107 |
| LINC01107 | 0.752905899 | 0.027504074 | NA | Up | 1.560602974 |
| LINC01937 | 0.71179037 | 0.390742029 | NA | NS | 0.408109872 |
| LINC01940 | 0.825476453 | 0.108058077 | NA | NS | 0.966342765 |
| HDAC4-AS1 | 0.142074906 | 0.20597347 | 0.43596473 | NS | 0.686188715 |
| CAPN10-DT | 0.004287759 | 0.956413206 | 0.976165401 | NS | 0.019354436 |
| UICLM | 0.263807582 | 0.20792702 | 0.437678733 | NS | 0.682089071 |
| SNED1-AS1 | 0.636879063 | 0.022638798 | NA | Up | 1.645146629 |
| BOK-AS1 | 0.10010218 | 0.87453311 | NA | NS | 0.058223743 |
| LINC01237 | 0.02883913 | 0.862678295 | 0.922649089 | NS | 0.064151129 |
| LINC01238 | -0.139490855 | 0.705031293 | NA | NS | 0.151791606 |
| LINC01986 | 0.525226322 | 0.292549872 | NA | NS | 0.533800087 |
| CHL1-AS2 | 0.293511361 | 0.561435949 | NA | NS | 0.250699783 |
| CHL1-AS1 | 0.686585012 | 0.079826998 | NA | NS | 1.097850203 |
| LINC01266 | 0.167450313 | 0.659619012 | 0.795335085 | NS | 0.180706835 |
| CNTN4-AS1 | 0.672198058 | 0.010480664 | NA | Up | 1.979611217 |
| ITPR1-DT | 0.039812689 | 0.767674508 | 0.864740339 | NS | 0.11482288 |
| EGOT | 0.218074267 | 0.114933369 | 0.333658762 | NS | 0.939553864 |
| BHLHE40-AS1 | 0.324126561 | 0.007694779 | 0.113974683 | Up | 2.113803845 |
| GRM7-AS3 | 0.524920484 | 0.215989976 | 0.445361714 | NS | 0.665566404 |
| GRM7-AS2 | 0.236125188 | 0.71039344 | NA | NS | 0.148501058 |
| GRM7-AS1 | 0.041049246 | 0.945272512 | NA | NS | 0.024442971 |
| LMCD1-AS1 | 0.404606519 | 0.292626311 | 0.515290877 | NS | 0.533686628 |
| SRGAP3-AS1 | -0.042800838 | 0.922094296 | NA | NS | 0.035224665 |
| SRGAP3-AS2 | 0.528735478 | 0.06757757 | NA | NS | 1.170197432 |
| SRGAP3-AS3 | 0.751809795 | 0.005516491 | NA | Up | 2.258337103 |
| SRGAP3-AS4 | 0.582889322 | 0.341779724 | NA | NS | 0.466253705 |
| THUMPD3-AS1 | 0.089845279 | 0.145389115 | 0.37059965 | NS | 0.837468108 |
| PRRT3-AS1 | 0.123327839 | 0.483182674 | 0.669197484 | NS | 0.315888647 |
| LINC00852 | 0.14652735 | 0.179150488 | 0.409182106 | NS | 0.746782005 |
| GHRLOS | 0.056130942 | 0.583746003 | 0.742884583 | NS | 0.23377608 |
| ATP2B2-IT1 | 0.40186035 | 0.483629722 | NA | NS | 0.315487017 |
| ATP2B2-IT2 | 0.690422932 | 0.222989897 | NA | NS | 0.651714813 |
| LINC00606 | 0.608067907 | 0.237730927 | NA | NS | 0.623914317 |
| SLC6A1-AS1 | 0.532477335 | 0.492956639 | NA | NS | 0.30719128 |
| LINC02022 | 1.023799881 | 0.136598026 | NA | NS | 0.864555576 |
| LINC00620 | 0.567460204 | 0.305125702 | NA | NS | 0.515521208 |
| XPC-AS1 | 0.16062525 | 0.164788744 | 0.392429221 | NS | 0.783072457 |
| LINC01267 | 0.627588506 | 0.271319824 | NA | NS | 0.566518474 |
| LINC02011 | 0.47826496 | 0.402249621 | NA | NS | 0.395504357 |
| FGD5-AS1 | -0.11224457 | 0.04464139 | 0.225889772 | Down | 1.350262294 |
| SH3BP5-AS1 | 0.175555215 | 0.00736794 | 0.112456655 | Up | 2.132653934 |
| EAF1-AS1 | 0.173689773 | 0.057404928 | 0.251059516 | NS | 1.241050821 |
| LINC00690 | 0.359001123 | 0.445281813 | NA | NS | 0.351365043 |
| SATB1-AS1 | 0.014618329 | 0.879061264 | 0.931833421 | NS | 0.055980857 |
| SGO1-AS1 | 0.314815665 | 0.011472255 | 0.131434507 | Up | 1.940351215 |
| ZNF385D-AS1 | 0.359044425 | 0.431212903 | NA | NS | 0.365308253 |
| ZNF385D-AS2 | 0.806719088 | 0.10245447 | NA | NS | 0.989469091 |
| UBE2E2-AS1 | 0.356324879 | 0.509856767 | NA | NS | 0.292551812 |
| UBE2E1-AS1 | 0.819324061 | 0.051384701 | NA | NS | 1.289166164 |
| LINC00691 | 0.46509014 | 0.218576272 | NA | NS | 0.660396985 |
| THRB-IT1 | 0.264218819 | 0.398180628 | NA | NS | 0.399919873 |
| THRB-AS1 | 0.416822569 | 0.268553985 | 0.494168679 | NS | 0.570968398 |
| LINC00692 | 0.531175737 | 0.08014427 | NA | NS | 1.096127523 |
| LINC02084 | -0.32800732 | 0.004532456 | 0.094211252 | Down | 2.343666433 |
| LINC01980 | 0.393629855 | 0.154454597 | 0.381395414 | NS | 0.811199162 |
| LINC01981 | 0.656509752 | 0.215327892 | NA | NS | 0.66689971 |
| LINC01967 | 0.489447745 | 0.084107647 | 0.292007269 | NS | 1.075164515 |
| RBMS3-AS3 | 0.454652903 | 0.292893862 | 0.515298984 | NS | 0.533289729 |
| RBMS3-AS2 | 0.606707729 | 0.078555206 | NA | NS | 1.104825025 |
| RBMS3-AS1 | 0.758305044 | 0.165867048 | NA | NS | 0.780239886 |
| LINC01985 | 0.676926476 | 0.184365639 | NA | NS | 0.734320016 |
| OSBPL10-AS1 | 0.231584587 | 0.255303659 | 0.48212537 | NS | 0.592942961 |
| CNOT10-AS1 | 0.298007965 | 0.080425268 | NA | NS | 1.094607481 |
| PDCD6IP-DT | 0.052311988 | 0.674480059 | 0.806015792 | NS | 0.171030886 |
| LINC01811 | 0.185336123 | 0.380621763 | 0.590622646 | NS | 0.419506383 |
| ARPP21-AS1 | 0.676226926 | 0.310407846 | NA | NS | 0.50806731 |
| LINC02033 | 0.253774423 | 0.029960203 | 0.192846704 | Up | 1.523455245 |
| GOLGA4-AS1 | 0.193092345 | 0.008067588 | 0.115486104 | Up | 2.093256266 |
| C3orf35 | 0.125636592 | 0.124112549 | 0.345415676 | NS | 0.906184305 |
| ITGA9-AS1 | 0.194101061 | 0.335990242 | 0.552156181 | NS | 0.473673335 |
| ACVR2B-AS1 | 0.26746248 | 0.261428738 | NA | NS | 0.582646674 |
| EIF1B-AS1 | 0.23259977 | 0.013070027 | 0.137118535 | Up | 1.883723511 |
| ENTPD3-AS1 | 0.109847929 | 0.198137063 | 0.428007454 | NS | 0.703034278 |
| VIPR1-AS1 | 0.258790096 | 0.068215221 | 0.268476763 | NS | 1.16611871 |
| HHATL-AS1 | 0.898101962 | 0.095795805 | NA | NS | 1.018653508 |
| CCDC13-AS1 | 0.293444825 | 0.138927831 | 0.363222438 | NS | 0.857210746 |
| LINC02158 | -0.075711778 | 0.599271225 | 0.753845413 | NS | 0.222376576 |
| KRBOX1-AS1 | 0.770668234 | 0.159986174 | NA | NS | 0.795917547 |
| SNRK-AS1 | 0.27590676 | 0.005717304 | 0.102180893 | Up | 2.242808697 |
| LINC01988 | 0.336797184 | 0.060614337 | 0.256526216 | NS | 1.217424641 |
| LARS2-AS1 | 0.39572502 | 0.05791802 | NA | NS | 1.237186294 |
| LIMD1-AS1 | 0.144191649 | 0.133182118 | 0.356407673 | NS | 0.875554083 |
| CCR5AS | -0.263012408 | 0.076260599 | 0.279989714 | NS | 1.11769979 |
| LINC02009 | 0.396052221 | 0.072013163 | 0.273667821 | NS | 1.142588113 |
| LRRC2-AS1 | 0.256886776 | 0.474617423 | NA | NS | 0.323656323 |
| KIF9-AS1 | 0.10226371 | 0.36439799 | 0.576661331 | NS | 0.438424027 |
| PTPN23-DT | -0.003056908 | 0.976774429 | 0.986489187 | NS | 0.010205718 |
| LINC02585 | 0.663758249 | 0.099612484 | NA | NS | 1.00168623 |
| PRKAR2A-AS1 | 0.07157438 | 0.508262522 | 0.687821019 | NS | 0.293911913 |
| ARIH2OS | -0.192902078 | 0.13678638 | 0.361199317 | NS | 0.863957144 |
| RHOA-IT1 | 0.215270923 | 0.110306874 | 0.328810103 | NS | 0.957397424 |
| BSN-DT | 0.50038532 | 0.224368033 | NA | NS | 0.64903902 |
| BSN-AS1 | 0.499908442 | 0.451419708 | NA | NS | 0.345419484 |
| RBM5-AS1 | 0.130902534 | 0.276712879 | 0.501010226 | NS | 0.557970627 |
| SEMA3F-AS1 | 0.18073739 | 0.01594426 | 0.149715225 | Up | 1.797395643 |
| SEMA3B-AS1 | 0.529838028 | 0.032696697 | NA | Up | 1.485496119 |
| RASSF1-AS1 | -0.076231182 | 0.690865992 | NA | NS | 0.160606185 |
| LINC02019 | 0.149078456 | 0.148746961 | 0.374763795 | NS | 0.827551899 |
| IQCF5-AS1 | 0.005461585 | 0.992522071 | NA | NS | 0.003259827 |
| GLYCTK-AS1 | 0.149575612 | 0.100932448 | 0.316391311 | NS | 0.995969194 |
| ITIH4-AS1 | 0.467474938 | 0.253821629 | NA | NS | 0.595471372 |
| ESRG | 0.02133329 | 0.954517454 | 0.975516923 | NS | 0.020216126 |
| CACNA2D3-AS1 | 0.882071747 | 0.13280089 | NA | NS | 0.876799016 |
| LINC02017 | 0.375150554 | 0.464162231 | NA | NS | 0.333330201 |
| LINC02030 | 0.317690617 | 0.542665391 | NA | NS | 0.265467875 |
| WNT5A-AS1 | 0.207235811 | 0.754684204 | NA | NS | 0.12223474 |
| ERC2-IT1 | 0.654349316 | 0.158139948 | NA | NS | 0.800958409 |
| ARHGEF3-AS1 | 0.788641882 | 0.174114782 | NA | NS | 0.759164357 |
| ARF4-AS1 | 0.233889271 | 0.268577088 | NA | NS | 0.570931039 |
| DENND6A-AS1 | 0.500531829 | 0.004771154 | 0.095682107 | Up | 2.321376575 |
| DENND6A-DT | 0.606503835 | 0.136383051 | NA | NS | 0.865239598 |
| FLNB-AS1 | 0.147625018 | 0.36910137 | 0.580614023 | NS | 0.432854343 |
| FAM3D-AS1 | 0.246044936 | 0.609777019 | NA | NS | 0.214828947 |
| CFAP20DC-AS1 | 0.417164385 | 0.29819912 | NA | NS | 0.525493643 |
| PTPRG-AS1 | 0.265077217 | 0.459788101 | 0.651210087 | NS | 0.337442272 |
| LINC00698 | 0.235103871 | 0.648724673 | NA | NS | 0.187939584 |
| SYNPR-AS1 | 0.269869308 | 0.748855333 | NA | NS | 0.125602073 |
| THOC7-AS1 | 0.117367258 | 0.309897983 | 0.530293015 | NS | 0.508781251 |
| SCAANT1 | 0.211365198 | 0.055472251 | 0.248604264 | NS | 1.25592421 |
| PSMD6-AS2 | 0.105780907 | 0.284644722 | 0.507588489 | NS | 0.545696864 |
| PRICKLE2-AS1 | 0.712511249 | 0.140023274 | NA | NS | 0.853799773 |
| LINC00994 | 0.68052304 | 0.201097069 | NA | NS | 0.696594259 |
| PRICKLE2-AS2 | 0.597762288 | 0.337607242 | NA | NS | 0.471588246 |
| PRICKLE2-AS3 | 0.454356813 | 0.313857514 | NA | NS | 0.50326747 |
| PRICKLE2-DT | 0.161895353 | 0.766994899 | NA | NS | 0.115207525 |
| ADAMTS9-AS1 | 0.612276849 | 0.157533488 | NA | NS | 0.802627111 |
| ADAMTS9-AS2 | 0.599806978 | 0.186179555 | 0.415835484 | NS | 0.730068012 |
| LINC02040 | 1.17658823 | 0.050806526 | NA | NS | 1.294080496 |
| MAGI1-IT1 | 0.831830255 | 0.150520684 | NA | NS | 0.822403816 |
| MAGI1-AS1 | 0.317328672 | 0.607582055 | NA | NS | 0.216395062 |
| SUCLG2-AS1 | 0.068291492 | 0.595032125 | 0.750601864 | NS | 0.225459586 |
| SAMMSON | 0.523529851 | 0.206840855 | 0.436757262 | NS | 0.684363676 |
| FOXP1-AS1 | 0.156140244 | 0.161326024 | 0.389034501 | NS | 0.792295569 |
| FOXP1-IT1 | 0.166882547 | 0.148261917 | 0.374447996 | NS | 0.828970388 |
| LINC00877 | 0.018443829 | 0.845143021 | 0.913061552 | NS | 0.073069791 |
| LINC00870 | 0.392984714 | 0.402970821 | 0.607160377 | NS | 0.3947264 |
| PDZRN3-AS1 | 0.447531328 | 0.525399511 | NA | NS | 0.279510336 |
| LINC02005 | 0.314654281 | 0.640658381 | NA | NS | 0.193373488 |
| LINC02047 | 0.250231288 | 0.736949624 | NA | NS | 0.132562199 |
| LINC02018 | -0.59945613 | 0.013733366 | NA | Down | 1.862223013 |
| LINC00960 | 0.256442965 | 0.162002355 | 0.389682175 | NS | 0.790478672 |
| LINC02077 | 1.035194585 | 0.149990649 | NA | NS | 0.823935815 |
| LINC02050 | 0.69538325 | 0.052566495 | NA | NS | 1.279290983 |
| LINC02027 | 0.38541814 | 0.414029232 | 0.615800589 | NS | 0.382968995 |
| LINC02008 | 0.271977203 | 0.557644281 | NA | NS | 0.253642748 |
| LINC00971 | 0.356767805 | 0.476169518 | NA | NS | 0.322238409 |
| CADM2-AS2 | 0.215940966 | 0.733961149 | NA | NS | 0.134326928 |
| CADM2-AS1 | 0.161573924 | 0.726057628 | NA | NS | 0.139028908 |
| LINC02070 | -0.306541948 | 0.660703643 | NA | NS | 0.179993299 |
| LINC00506 | 0.515821811 | 0.156971693 | 0.384159619 | NS | 0.804178657 |
| LINC00879 | 0.408014031 | 0.480269363 | NA | NS | 0.318515117 |
| ST3GAL6-AS1 | 0.311049984 | 0.339132931 | 0.554994713 | NS | 0.469630037 |
| LINC00973 | 0.960248085 | 0.127153902 | NA | NS | 0.89567031 |
| ZBTB11-AS1 | 0.062471529 | 0.286524415 | 0.509714081 | NS | 0.542838365 |
| LINC02085 | -0.599073907 | 0.14596471 | NA | NS | 0.835752133 |
| LINC00882 | 0.327532944 | 0.396565285 | 0.601745058 | NS | 0.401685306 |
| DUBR | 0.130848122 | 0.375968793 | 0.586177342 | NS | 0.424848202 |
| LINC01990 | 0.210694109 | 0.585965088 | NA | NS | 0.232128259 |
| LINC00636 | 0.719991427 | 0.125238451 | NA | NS | 0.902262312 |
| LINC00635 | 0.586464065 | 0.159844848 | 0.38738272 | NS | 0.796301357 |
| LINC01215 | 0.029800553 | 0.721787405 | 0.83580398 | NS | 0.1415907 |
| MORC1-AS1 | 0.355834043 | 0.627874282 | NA | NS | 0.202127306 |
| LINC00488 | 0.065729644 | 0.909247982 | NA | NS | 0.041317654 |
| LINC01205 | 0.463886707 | 0.231732994 | 0.459837736 | NS | 0.635012127 |
| NECTIN3-AS1 | 0.517616217 | 0.171070821 | NA | NS | 0.766824061 |
| PLCXD2-AS1 | -0.014305055 | 0.918551126 | 0.954909572 | NS | 0.036896666 |
| LINC02042 | 0.127504744 | 0.699968425 | NA | NS | 0.15492155 |
| CD200R1L-AS1 | 0.14856096 | 0.644245571 | NA | NS | 0.190948559 |
| LINC02044 | 0.400892034 | 0.15197434 | 0.378507048 | NS | 0.818229734 |
| CFAP44-AS1 | 0.185158502 | 0.249375924 | 0.47650024 | NS | 0.603145478 |
| SIDT1-AS1 | 0.093460517 | 0.457104941 | 0.648659294 | NS | 0.339984085 |
| ZBTB20-AS1 | 0.184105963 | 0.050892166 | 0.238726596 | NS | 1.293349066 |
| ZBTB20-AS5 | 0.192557083 | 0.136005715 | 0.360134579 | NS | 0.866442842 |
| ZBTB20-AS2 | 0.186756822 | 0.302990903 | 0.524319378 | NS | 0.518570411 |
| ZBTB20-AS3 | 0.214199668 | 0.048331257 | 0.233310996 | Up | 1.315771907 |
| ZBTB20-AS4 | 0.303549146 | 0.020999872 | 0.167842555 | Up | 1.677783353 |
| LSAMP-AS1 | 0.884891078 | 0.102733 | NA | NS | 0.988290028 |
| TUSC7 | 0.532335019 | 0.300919841 | NA | NS | 0.521549177 |
| LINC00901 | 0.781142797 | 0.201427491 | NA | NS | 0.695881257 |
| LINC02024 | 0.229573726 | 0.698763307 | NA | NS | 0.155669909 |
| IGSF11-AS1 | 0.746444251 | 0.086853511 | NA | NS | 1.061212619 |
| B4GALT4-AS1 | 0.242125609 | 0.052702445 | 0.242513843 | NS | 1.278169235 |
| ARHGAP31-AS1 | 0.337986555 | 0.100169686 | 0.315294963 | NS | 0.999263687 |
| TIMMDC1-DT | 0.490192455 | 0.042333299 | NA | Up | 1.37331789 |
| LINC02049 | 0.744710535 | 0.178396894 | NA | NS | 0.748612711 |
| WDR5B-DT | 0.059938959 | 0.34157693 | 0.55696544 | NS | 0.466511468 |
| LINC02035 | 0.332166324 | 0.001767793 | 0.065790668 | Up | 2.75256848 |
| MYLK-AS1 | 0.067224233 | 0.405370109 | 0.60905444 | NS | 0.392148278 |
| MYLK-AS2 | 0.32322093 | 0.475134675 | NA | NS | 0.323183274 |
| ITGB5-AS1 | 1.065532541 | 0.120243907 | NA | NS | 0.919936919 |
| LINC02614 | 0.098732069 | 0.51899987 | 0.69601515 | NS | 0.284832751 |
| SLC41A3-AS1 | -0.025308344 | 0.870831963 | 0.927057761 | NS | 0.060065639 |
| ALDH1L1-AS2 | 0.621051648 | 0.237302767 | NA | NS | 0.624697197 |
| CFAP100-DT | 0.866384009 | 0.200250489 | NA | NS | 0.698426416 |
| LINC02016 | 0.821216189 | 0.087775991 | NA | NS | 1.056624256 |
| LINC01471 | 0.387036132 | 0.346228601 | 0.561050551 | NS | 0.460637059 |
| LINC02034 | 0.526988875 | 0.280414966 | NA | NS | 0.552198811 |
| RUVBL1-AS1 | 0.112173569 | 0.549572719 | NA | NS | 0.259974834 |
| DNAJB8-AS1 | 0.131977981 | 0.833291205 | NA | NS | 0.079203202 |
| GATA2-AS1 | 0.762263697 | 0.014251071 | 0.143336863 | Up | 1.846152485 |
| LINC01565 | 0.952066971 | 0.070210922 | NA | NS | 1.153595324 |
| H1-10-AS1 | 0.232376954 | 0.222667988 | 0.451412976 | NS | 0.652342216 |
| TMCC1-DT | -0.035354853 | 0.685392778 | 0.813023826 | NS | 0.164060476 |
| LINC02014 | 0.116660783 | 0.722077531 | NA | NS | 0.141416169 |
| LINC02021 | 0.272872548 | 0.072391674 | 0.27394225 | NS | 1.14031138 |
| NUDT16-DT | 0.155858315 | 0.178338863 | 0.408326093 | NS | 0.748754007 |
| NPHP3-AS1 | 0.45230353 | 0.276754584 | NA | NS | 0.557905177 |
| TMEM108-AS1 | 0.93894908 | 0.07950493 | NA | NS | 1.09960594 |
| BFSP2-AS1 | 0.208576433 | 0.385795463 | NA | NS | 0.413642884 |
| C3orf36 | 0.868688456 | 0.079895989 | NA | NS | 1.097475022 |
| LINC02004 | 0.808513584 | 0.33008494 | NA | NS | 0.48137429 |
| STAG1-DT | 0.360067594 | 0.099145356 | 0.314049068 | NS | 1.003727624 |
| NCK1-DT | 0.17385708 | 0.026531575 | 0.183906212 | Up | 1.576236973 |
| IL20RB-AS1 | 0.593290995 | 0.006190209 | NA | Up | 2.20829467 |
| LINC01210 | 0.272913542 | 0.614157828 | NA | NS | 0.211720008 |
| LINC01391 | 0.730772658 | 0.120386599 | NA | NS | 0.919421853 |
| BPESC1 | 0.418186342 | 0.438051618 | NA | NS | 0.358474711 |
| PISRT1 | 0.677896034 | 0.363439848 | NA | NS | 0.439567458 |
| COPB2-DT | 0.193060075 | 0.05960892 | 0.254791869 | NS | 1.224688745 |
| CLSTN2-AS1 | 0.733376603 | 0.176909458 | NA | NS | 0.752248948 |
| RASA2-IT1 | 0.179037081 | 0.243700783 | 0.471587269 | NS | 0.613143076 |
| ATP1B3-AS1 | 0.321582118 | 0.043181864 | 0.222962086 | Up | 1.36469862 |
| PLS1-AS1 | 0.691191995 | 0.208174462 | NA | NS | 0.68157255 |
| PAQR9-AS1 | 0.402056109 | 0.41653725 | NA | NS | 0.380346154 |
| SLC9A9-AS1 | 0.306848515 | 0.026238123 | 0.183092929 | Up | 1.581067236 |
| SLC9A9-AS2 | 0.146307446 | 0.34986383 | 0.563990726 | NS | 0.456100954 |
| LNCSRLR | 0.624380363 | 0.306760741 | NA | NS | 0.513200222 |
| LINC02010 | 0.132575025 | 0.81167603 | NA | NS | 0.090617279 |
| LINC02032 | 0.519815439 | 0.392259835 | NA | NS | 0.406426158 |
| LINC02045 | 0.2499936 | 0.671130569 | NA | NS | 0.173192979 |
| LINC02046 | 0.278526128 | 0.46811993 | 0.657722861 | NS | 0.329642868 |
| HLTF-AS1 | 0.692374604 | 0.393172307 | NA | NS | 0.405417079 |
| TM4SF1-AS1 | 0.834108029 | 0.166402923 | NA | NS | 0.77883905 |
| WWTR1-IT1 | 0.4558136 | 0.408932791 | NA | NS | 0.388348063 |
| WWTR1-AS1 | 0.982628639 | 0.028202559 | NA | Up | 1.549711487 |
| LINC01998 | 0.264601259 | 0.628765555 | NA | NS | 0.201511258 |
| LINC01213 | 0.287497374 | 0.545581884 | NA | NS | 0.263140059 |
| LINC01214 | 0.224236495 | 0.663967789 | NA | NS | 0.177852989 |
| ERICH6-AS1 | -0.053853287 | 0.608325749 | 0.759851448 | NS | 0.2158638 |
| SIAH2-AS1 | 0.11724816 | 0.364079888 | 0.576277393 | NS | 0.438803311 |
| CLRN1-AS1 | 0.651580431 | 0.060983991 | 0.256832544 | NS | 1.214784154 |
| AADACL2-AS1 | 0.75236639 | 0.051647753 | NA | NS | 1.286948564 |
| MBNL1-AS1 | 0.172012774 | 0.078804255 | 0.283635871 | NS | 1.103450332 |
| LINC02006 | 0.099528327 | 0.652125157 | 0.790419176 | NS | 0.185669046 |
| LINC02877 | 0.204185767 | 0.725768809 | NA | NS | 0.1392017 |
| ARHGEF26-AS1 | 0.32010887 | 0.441097601 | 0.635784938 | NS | 0.355465304 |
| MME-AS1 | 0.240275409 | 0.246706178 | 0.47480291 | NS | 0.607819974 |
| LINC01487 | 0.509009904 | 0.31635006 | NA | NS | 0.499832079 |
| PLCH1-AS1 | 0.182957995 | 0.720453753 | NA | NS | 0.142393892 |
| PLCH1-AS2 | 0.215890299 | 0.753008701 | NA | NS | 0.123200006 |
| KCNAB1-AS2 | -0.430557135 | 0.466059881 | NA | NS | 0.33155828 |
| KCNAB1-AS1 | 0.253063778 | 0.706023116 | NA | NS | 0.151181079 |
| TIPARP-AS1 | -0.171626732 | 0.269997803 | 0.495511633 | NS | 0.568639771 |
| LINC00886 | 0.016060942 | 0.909226583 | 0.949569393 | NS | 0.041327875 |
| LINC00880 | 0.117054176 | 0.583752718 | NA | NS | 0.233771085 |
| LINC02029 | 0.244705021 | 0.419136294 | NA | NS | 0.377644731 |
| LINC00881 | 0.19302845 | 0.185593185 | 0.415463304 | NS | 0.731437975 |
| IQCJ-SCHIP1-AS1 | 0.419225542 | 0.462490739 | NA | NS | 0.33489696 |
| IL12A-AS1 | 0.509229745 | 0.18289511 | 0.413083217 | NS | 0.737797905 |
| LINC01100 | 0.634331069 | 0.305934944 | NA | NS | 0.514370915 |
| PPM1L-DT | 0.05892684 | 0.579639754 | 0.739260992 | NS | 0.236841837 |
| LINC02067 | 0.16216797 | 0.723399435 | NA | NS | 0.140621835 |
| LINC01192 | 0.273184658 | 0.567657793 | 0.730855676 | NS | 0.245913396 |
| LINC01323 | 0.696450323 | 0.346187889 | NA | NS | 0.460688129 |
| LINC01324 | 0.657616586 | 0.308911889 | NA | NS | 0.510165377 |
| LINC02023 | 0.717955312 | 0.243786076 | NA | NS | 0.612991103 |
| LINC01322 | 0.296295478 | 0.502804654 | 0.684042859 | NS | 0.298600711 |
| LINC01326 | 0.359076271 | 0.585187769 | NA | NS | 0.23270476 |
| LINC01327 | 0.357009148 | 0.58549451 | NA | NS | 0.232477173 |
| LINC02082 | 0.42400417 | 0.446804393 | NA | NS | 0.349882566 |
| LINC01997 | 0.238419012 | 0.698876249 | NA | NS | 0.155599718 |
| MECOM-AS1 | 0.100092103 | 0.838313372 | NA | NS | 0.076593606 |
| TERC | -0.263949586 | 0.385786666 | NA | NS | 0.413652787 |
| SEC62-AS1 | -0.076274784 | 0.513305906 | 0.691554319 | NS | 0.289623738 |
| SLC7A14-AS1 | 0.443174773 | 0.222197301 | 0.450931554 | NS | 0.653261221 |
| TMEM212-AS1 | 0.319025867 | 0.707508286 | NA | NS | 0.150268469 |
| TMEM212-IT1 | 1.465734939 | 0.01984607 | NA | Up | 1.702325492 |
| LINC02068 | 0.279339701 | 0.220981501 | 0.449641695 | NS | 0.655644081 |
| NLGN1-AS1 | 0.744575794 | 0.231317781 | NA | NS | 0.635790983 |
| NAALADL2-AS3 | 0.45608588 | 0.405060845 | NA | NS | 0.392479735 |
| NAALADL2-AS2 | 0.558974142 | 0.271059772 | NA | NS | 0.566934932 |
| NAALADL2-AS1 | 0.383448611 | 0.567757717 | NA | NS | 0.245836954 |
| LINC01208 | 0.489178638 | 0.259688314 | 0.485843002 | NS | 0.585547594 |
| LINC01209 | 0.253840258 | 0.61850114 | NA | NS | 0.208659496 |
| TBL1XR1-AS1 | 0.269474055 | 0.011776852 | 0.132971584 | Up | 1.928970777 |
| LINC00501 | 0.536008346 | 0.2621997 | NA | NS | 0.58136781 |
| LINC00578 | 0.650378196 | 0.099547848 | 0.314678374 | NS | 1.001968123 |
| LINC02015 | 0.07234249 | 0.898125042 | NA | NS | 0.046663194 |
| KCNMB2-AS1 | 0.223051193 | 0.407541455 | 0.611075752 | NS | 0.389828208 |
| PEX5L-AS1 | 0.276261289 | 0.62822379 | NA | NS | 0.201885621 |
| PEX5L-AS2 | 0.460340096 | 0.460209999 | NA | NS | 0.337043949 |
| LINC02053 | 0.299878196 | 0.563793091 | NA | NS | 0.24888025 |
| CCDC39-AS1 | 0.523194723 | 0.585647394 | NA | NS | 0.232363785 |
| SOX2-OT | 0.38369182 | 0.161285219 | 0.389034501 | NS | 0.792405432 |
| LINC01206 | 0.232974823 | 0.605934411 | 0.7583949 | NS | 0.217574383 |
| LINC01994 | 0.499228799 | 0.27239747 | NA | NS | 0.56479693 |
| LINC01995 | -0.306232459 | 0.565395686 | NA | NS | 0.24764751 |
| LINC02031 | -0.215842461 | 0.659288685 | NA | NS | 0.180924378 |
| MCCC1-AS1 | 0.237982448 | 0.041964279 | 0.220731721 | Up | 1.377120237 |
| KLHL6-AS1 | 0.429705965 | 0.00275698 | 0.079323886 | Up | 2.55956633 |
| YEATS2-AS1 | 0.139599365 | 0.152113555 | 0.378745884 | NS | 0.817832084 |
| ABCC5-AS1 | -0.220455586 | 0.224292437 | NA | NS | 0.64918537 |
| HTR3E-AS1 | 0.598715312 | 0.432028099 | NA | NS | 0.364488006 |
| EIF2B5-DT | -0.386192004 | 0.054309641 | NA | NS | 1.265123071 |
| LINC02054 | 0.915426996 | 0.084512785 | NA | NS | 1.073077588 |
| LINC01840 | 0.737395686 | 0.234495567 | NA | NS | 0.629865363 |
| LINC02069 | 0.662832632 | 0.173701835 | NA | NS | 0.760195594 |
| EHHADH-AS1 | 0.24089171 | 0.667748136 | NA | NS | 0.175387316 |
| IGF2BP2-AS1 | 0.65573706 | 0.049747861 | NA | Up | 1.303225591 |
| ETV5-AS1 | 0.314599587 | 0.648485616 | NA | NS | 0.188099652 |
| LINC02020 | 0.547385147 | 0.281161425 | NA | NS | 0.551044264 |
| LINC02052 | 0.746572088 | 0.124376321 | NA | NS | 0.905262294 |
| LINC02051 | 0.819380994 | 0.208168954 | NA | NS | 0.681584039 |
| LINC02043 | 0.790287884 | 0.006041981 | NA | Up | 2.218820634 |
| ADIPOQ-AS1 | 0.42526915 | 0.439205677 | NA | NS | 0.357332055 |
| LINC02041 | 0.416166717 | 0.609196595 | NA | NS | 0.215242533 |
| LINC01991 | 0.438982184 | 0.168970894 | NA | NS | 0.772188098 |
| LPP-AS2 | 0.346765546 | 0.004004123 | 0.090296145 | Up | 2.397492611 |
| LPP-AS1 | 0.224011282 | 0.070392427 | 0.271192883 | NS | 1.152474063 |
| TPRG1-AS1 | 0.056659693 | 0.738399477 | 0.846218215 | NS | 0.13170862 |
| TPRG1-AS2 | 0.1619078 | 0.595216143 | NA | NS | 0.225325298 |
| P3H2-AS1 | 0.652733934 | 0.163617896 | NA | NS | 0.786169197 |
| LINC02013 | 0.34917402 | 0.441392023 | NA | NS | 0.35517552 |
| OSTN-AS1 | 0.238120431 | 0.386227995 | NA | NS | 0.41315625 |
| FGF12-AS1 | 0.225326495 | 0.7214177 | NA | NS | 0.141813206 |
| FGF12-AS2 | 1.384261524 | 0.009338015 | NA | Up | 2.029745434 |
| ATP13A5-AS1 | 0.827109922 | 0.085164785 | NA | NS | 1.069739945 |
| ATP13A4-AS1 | 0.305313394 | 0.228272154 | NA | NS | 0.641547064 |
| OPA1-AS1 | 0.048351899 | 0.610486996 | 0.761429756 | NS | 0.214323582 |
| LINC02038 | 0.668333037 | 0.170647878 | NA | NS | 0.767899108 |
| LINC02026 | 0.455403627 | 0.084919519 | NA | NS | 1.070992473 |
| LINC02028 | 0.659656949 | 0.102098836 | 0.317962583 | NS | 0.990979209 |
| LINC02036 | 0.649932689 | 0.136673454 | NA | NS | 0.864315831 |
| LINC02037 | 0.56864906 | 0.276335482 | NA | NS | 0.558563347 |
| LINC02048 | 0.802142434 | 0.093715141 | NA | NS | 1.028190236 |
| LINC00887 | 0.626273281 | 0.161925685 | NA | NS | 0.790684257 |
| LINC00884 | 0.232580986 | 0.212786798 | 0.442579534 | NS | 0.67205532 |
| TMEM44-AS1 | 0.019534905 | 0.837619616 | 0.908686606 | NS | 0.07695316 |
| LINC01968 | 0.323344119 | 0.433000536 | 0.629544076 | NS | 0.363511566 |
| LINC01972 | 0.535780167 | 0.307197619 | NA | NS | 0.512582155 |
| XXYLT1-AS1 | 0.532335533 | 0.141246076 | NA | NS | 0.850023608 |
| XXYLT1-AS2 | -0.093216344 | 0.530747691 | 0.704671084 | NS | 0.275111887 |
| ACAP2-IT1 | 0.271349269 | 0.009292567 | 0.121621961 | Up | 2.031864294 |
| MUC20-OT1 | -0.314521232 | 0.11214982 | 0.330787614 | NS | 0.950201419 |
| LINC01983 | 0.454790838 | 0.432293538 | NA | NS | 0.364221256 |
| TNK2-AS1 | -0.036937207 | 0.838520635 | 0.908983201 | NS | 0.076486246 |
| LINC00885 | 0.139589146 | 0.753911468 | NA | NS | 0.122679651 |
| TM4SF19-AS1 | 0.084070391 | 0.6547178 | 0.79248518 | NS | 0.183945852 |
| UBXN7-AS1 | 0.351215417 | 0.004264886 | 0.092751043 | Up | 2.370092602 |
| LINC01063 | -0.170424585 | 0.434029433 | NA | NS | 0.362480819 |
| NCBP2-AS1 | 0.027637866 | 0.800665976 | 0.885325037 | NS | 0.096548626 |
| MELTF-AS1 | 0.232457431 | 0.457406278 | NA | NS | 0.339697879 |
| DLG1-AS1 | 0.126512393 | 0.795849727 | NA | NS | 0.099168928 |
| LMLN-AS1 | 0.44194073 | 0.387501363 | NA | NS | 0.411726765 |
| PDE6B-AS1 | 0.693695853 | 0.151500198 | NA | NS | 0.819586799 |
| CTBP1-AS | 0.089780945 | 0.497768862 | 0.679738983 | NS | 0.302972274 |
| CTBP1-DT | 0.108596022 | 0.101507287 | 0.317281274 | NS | 0.993502778 |
| NOP14-AS1 | 0.165050034 | 0.001520742 | 0.062682592 | Up | 2.817944365 |
| HTT-AS | 0.16897874 | 0.435163663 | 0.631046553 | NS | 0.361347376 |
| LINC00955 | 1.147457094 | 0.027753177 | NA | Up | 1.556687287 |
| LINC02171 | 1.512848914 | 0.027268479 | NA | Up | 1.564339092 |
| LINC02600 | 0.8986629 | 0.065555316 | NA | NS | 1.183392083 |
| STX18-IT1 | 0.383829135 | 0.02174793 | 0.16918176 | Up | 1.662582066 |
| STX18-AS1 | 0.056363895 | 0.543229335 | 0.713032722 | NS | 0.265016786 |
| LINC01396 | 0.758068916 | 0.126265876 | NA | NS | 0.898714003 |
| LINC01587 | 0.140193625 | 0.709462798 | NA | NS | 0.149070373 |
| LINC02482 | 0.313283602 | 0.067699286 | 0.267671219 | NS | 1.169415911 |
| LINC02481 | -0.075235549 | 0.466838428 | 0.656687776 | NS | 0.330833402 |
| LINC02447 | 0.680916967 | 0.001351542 | 0.059779209 | Up | 2.869170451 |
| AFAP1-AS1 | 0.121504838 | 0.524358199 | 0.69977574 | NS | 0.280371937 |
| LINC02517 | 1.283766156 | 0.020560881 | NA | Up | 1.686958273 |
| SLC2A9-AS1 | -0.051124153 | 0.821489818 | NA | NS | 0.085397815 |
| LINC02498 | 0.383362967 | 0.505808149 | NA | NS | 0.296014178 |
| LINC02360 | 0.45109642 | 0.412180853 | NA | NS | 0.384912186 |
| LINC02270 | 0.847875278 | 0.134024061 | NA | NS | 0.872817228 |
| LINC01097 | 1.112458269 | 0.040075425 | NA | Up | 1.39712186 |
| LINC01096 | 0.645186008 | 0.1841672 | NA | NS | 0.734787716 |
| LINC01182 | 0.434557405 | 0.161914189 | 0.389676204 | NS | 0.790715091 |
| LINC00504 | 0.329773462 | 0.222690761 | 0.451412976 | NS | 0.652297801 |
| CPEB2-DT | 0.637687616 | 0.138252377 | NA | NS | 0.859327394 |
| C1QTNF7-AS1 | 0.307478866 | 0.102318333 | NA | NS | 0.990046543 |
| TAPT1-AS1 | 0.264176722 | 0.004356266 | 0.093367359 | Up | 2.360885579 |
| LINC02493 | 0.900346753 | 0.119401161 | NA | NS | 0.922991451 |
| MED28-DT | 0.249245028 | 0.018653606 | 0.158871289 | Up | 1.729237189 |
| LINC02438 | 1.031840704 | 0.079876779 | NA | NS | 1.097579456 |
| KCNIP4-IT1 | 0.400911092 | 0.259571034 | 0.485843002 | NS | 0.585743773 |
| ERVH-1 | 0.518572017 | 0.331652628 | NA | NS | 0.479316557 |
| LINC02473 | 0.512577693 | 0.371204142 | NA | NS | 0.430387186 |
| SEPSECS-AS1 | -0.032588052 | 0.735224916 | 0.843792643 | NS | 0.133579784 |
| LINC02357 | 0.011898467 | 0.97949937 | NA | NS | 0.008995839 |
| STIM2-AS1 | 0.016110198 | 0.901398426 | 0.945230163 | NS | 0.045083205 |
| LINC02261 | 0.495613183 | 0.262014468 | NA | NS | 0.581674727 |
| LINC02364 | 0.458876387 | 0.384945102 | NA | NS | 0.414601202 |
| LINC02472 | 0.450318062 | 0.421159494 | NA | NS | 0.375553405 |
| LINC02497 | 0.744279377 | 0.21840632 | NA | NS | 0.660734799 |
| LINC02501 | 0.822775759 | 0.250135643 | NA | NS | 0.60182442 |
| LINC02506 | -0.902249649 | 0.029803284 | 0.192297294 | Down | 1.525735886 |
| LINC02353 | 0.78120395 | 0.306506406 | NA | NS | 0.513560444 |
| LINC02484 | 0.380227614 | 0.429418222 | NA | NS | 0.36711953 |
| LINC02505 | 0.940198663 | 0.089715206 | NA | NS | 1.04713394 |
| LINC02616 | -0.096887079 | 0.859085665 | NA | NS | 0.065963528 |
| LINC02513 | 0.438141022 | 0.329911871 | NA | NS | 0.481602057 |
| LINC01258 | 0.391342537 | 0.352908159 | NA | NS | 0.452338301 |
| LINC01259 | 0.02434189 | 0.839483761 | 0.909492508 | NS | 0.0759877 |
| LINC02278 | 0.86269595 | 0.114417097 | NA | NS | 0.941509074 |
| KLF3-AS1 | 0.068665237 | 0.506019339 | 0.686297541 | NS | 0.295832885 |
| UGDH-AS1 | 0.313470096 | 0.001174079 | 0.055901767 | Up | 2.930302798 |
| SMIM14-DT | 0.393648451 | 0.139553399 | NA | NS | 0.855259581 |
| LINC02265 | 0.312546221 | 0.413471254 | NA | NS | 0.383554679 |
| UCHL1-DT | 0.21444558 | 0.692466405 | NA | NS | 0.159601291 |
| PHOX2B-AS1 | 0.598892893 | 0.334435516 | NA | NS | 0.475687609 |
| LINC00682 | 0.937193094 | 0.054728173 | NA | NS | 1.261789053 |
| ATP8A1-DT | 0.12874518 | 0.611370939 | NA | NS | 0.213695209 |
| LINC02383 | 0.297469849 | 0.692944056 | NA | NS | 0.159301826 |
| LINC02475 | 0.516404595 | 0.348384492 | NA | NS | 0.457941185 |
| OCIAD1-AS1 | -0.036479868 | 0.811004812 | 0.89154861 | NS | 0.090976569 |
| LINC02480 | 0.820081511 | 0.241415851 | NA | NS | 0.617234219 |
| USP46-DT | 0.049787334 | 0.68457178 | 0.812461155 | NS | 0.164581007 |
| DANCR | 0.02501348 | 0.696525631 | 0.820148803 | NS | 0.157062897 |
| LINC01618 | 0.623861759 | 0.163123182 | NA | NS | 0.787484316 |
| LNX1-AS1 | 0.328574975 | 0.534688897 | NA | NS | 0.271898834 |
| LNX1-AS2 | 0.239253566 | 0.661308319 | NA | NS | 0.179596014 |
| LINC02283 | 0.245043157 | 0.684780888 | NA | NS | 0.164448369 |
| LINC02260 | 0.259780152 | 0.650802887 | NA | NS | 0.186550529 |
| LINC02358 | 0.702692111 | 0.406183683 | NA | NS | 0.391277527 |
| SRD5A3-AS1 | 0.350172699 | 0.112272119 | 0.330913109 | NS | 0.949728082 |
| IGFBP7-AS1 | 0.634820609 | 0.116835505 | NA | NS | 0.93242516 |
| LINC02380 | 0.437175337 | 0.402947087 | NA | NS | 0.39475198 |
| LINC02494 | 0.195357877 | 0.752698942 | NA | NS | 0.123378695 |
| LINC02619 | -0.018703146 | 0.979105659 | NA | NS | 0.009170439 |
| LINC02429 | -0.086604426 | 0.902067951 | NA | NS | 0.044760747 |
| LINC02496 | 0.208383902 | 0.744964878 | NA | NS | 0.127864202 |
| LINC02271 | 1.052194039 | 0.082060343 | NA | NS | 1.085866674 |
| ADGRL3-AS1 | 0.201687742 | 0.716638887 | NA | NS | 0.144699629 |
| LINC02232 | 0.52757215 | 0.229772101 | 0.458066753 | NS | 0.638702705 |
| LINC02835 | 0.765715434 | 0.230083675 | NA | NS | 0.638114194 |
| EPHA5-AS1 | 1.368516716 | 0.059827222 | NA | NS | 1.223101163 |
| UBA6-DT | 0.119592227 | 0.11094623 | 0.329557789 | NS | 0.954887452 |
| ANKRD17-DT | -0.015841604 | 0.889136191 | 0.93735026 | NS | 0.051031712 |
| LINC02499 | 0.515821503 | 0.363521051 | NA | NS | 0.439470435 |
| UMLILO | 0.822036891 | 0.131453824 | NA | NS | 0.881226777 |
| PARM1-AS1 | -0.005859785 | 0.97165042 | 0.983723402 | NS | 0.012489957 |
| LINC02562 | 0.391326378 | 0.376617009 | NA | NS | 0.42410007 |
| LINC02483 | 0.567386459 | 0.327203754 | NA | NS | 0.485181722 |
| SDAD1-AS1 | 0.329003281 | 0.066804473 | 0.266334178 | NS | 1.175194457 |
| SHROOM3-AS1 | 0.704227829 | 0.099266356 | NA | NS | 1.003197922 |
| LINC01094 | -0.082716892 | 0.565204125 | 0.729102337 | NS | 0.247794678 |
| BMP2K-DT | 0.067607551 | 0.735288175 | 0.843792643 | NS | 0.133542418 |
| LINC01088 | 0.435595397 | 0.070616612 | 0.271500817 | NS | 1.151093124 |
| LINC00989 | -0.113935253 | 0.382600352 | 0.591968198 | NS | 0.417254635 |
| LINC02469 | 0.357468411 | 0.502290174 | NA | NS | 0.299045318 |
| PCAT4 | 0.507555791 | 0.196893621 | NA | NS | 0.705768353 |
| PRDM8-AS1 | 0.119633788 | 0.324016391 | 0.542570549 | NS | 0.489433019 |
| PRKG2-AS1 | 0.526874477 | 0.269346065 | NA | NS | 0.569689366 |
| HNRNPD-DT | 0.141647583 | 0.32019184 | 0.539074418 | NS | 0.494589741 |
| LINC00575 | 0.483664621 | 0.375414103 | NA | NS | 0.425489416 |
| THAP9-AS1 | -0.183147683 | 0.011696752 | 0.132526766 | Down | 1.931934717 |
| WDFY3-AS1 | 0.639988239 | 0.009020635 | NA | Up | 2.044762872 |
| WDFY3-AS2 | 0.358866816 | 0.023753481 | 0.174898328 | Up | 1.624272738 |
| MAPK10-AS1 | 1.045928422 | 0.071112598 | NA | NS | 1.148053452 |
| PPM1K-DT | 0.722458915 | 0.03178424 | NA | Up | 1.497788174 |
| PIGY-DT | 0.223844479 | 0.588496047 | NA | NS | 0.23025645 |
| FAM13A-AS1 | 0.169604808 | 0.081314849 | 0.287654103 | NS | 1.089830142 |
| SNCA-AS1 | 0.060394052 | 0.862230149 | NA | NS | 0.064376795 |
| LNCPRESS2 | 0.009131789 | 0.988372202 | NA | NS | 0.005079478 |
| SMARCAD1-DT | 0.200718184 | 0.739591025 | NA | NS | 0.131008368 |
| BMPR1B-DT | 0.044261174 | 0.924437563 | NA | NS | 0.034122416 |
| UNC5C-AS1 | 0.812130763 | 0.092836699 | NA | NS | 1.03228031 |
| LINC02267 | 0.391838087 | 0.434558778 | NA | NS | 0.361951473 |
| STPG2-AS1 | 0.381557407 | 0.343188655 | 0.55833676 | NS | 0.464467078 |
| TSPAN5-DT | 0.123325067 | 0.505267352 | 0.685819827 | NS | 0.296478763 |
| H2AZ1-DT | 0.195134792 | 0.183481677 | 0.413733592 | NS | 0.736407298 |
| DDIT4L-AS1 | 0.391776515 | 0.523388811 | NA | NS | 0.281175566 |
| LINC01216 | -5.15E-04 | 0.999326109 | NA | NS | 2.93E-04 |
| LINC01217 | 0.683186629 | 0.161399389 | NA | NS | 0.792098115 |
| LINC01218 | 0.024756607 | 0.944318492 | NA | NS | 0.024881506 |
| FLJ20021 | 0.275894903 | 0.04178534 | 0.220296466 | Up | 1.378976063 |
| UBE2D3-AS1 | 0.056592827 | 0.617938313 | 0.766202615 | NS | 0.209054877 |
| LINC02428 | 0.302675126 | 0.562819035 | NA | NS | 0.249631222 |
| TACR3-AS1 | 1.118936157 | 0.070900741 | NA | NS | 1.149349229 |
| LINC02503 | -0.095139264 | 0.890249323 | NA | NS | 0.050488348 |
| CXXC4-AS1 | 0.15320755 | 0.62917385 | 0.774113006 | NS | 0.201229336 |
| TET2-AS1 | 0.201192498 | 0.073678561 | 0.275940596 | NS | 1.132658863 |
| ARHGEF38-IT1 | 1.100103198 | 0.104790535 | NA | NS | 0.979677941 |
| GSTCD-AS1 | 0.328890569 | 0.072462761 | 0.27394225 | NS | 1.139885123 |
| LINC02173 | 1.000672849 | 0.157156772 | NA | NS | 0.8036669 |
| CYP2U1-AS1 | 0.369265862 | 0.006738468 | 0.109217554 | Up | 2.171438803 |
| LEF1-AS1 | 0.09708533 | 0.537853184 | 0.709095159 | NS | 0.269336256 |
| RPL34-DT | 0.076273473 | 0.555652987 | 0.721924582 | NS | 0.255196347 |
| COL25A1-DT | 0.228093771 | 0.615669265 | NA | NS | 0.210652526 |
| SEC24B-AS1 | 0.05921465 | 0.519327141 | 0.696103839 | NS | 0.284558979 |
| PANCR | 0.791896593 | 0.123193197 | NA | NS | 0.909413275 |
| LINC01438 | 0.705104251 | 0.30459726 | NA | NS | 0.516274008 |
| AP1AR-DT | -0.043603114 | 0.761334271 | 0.860657491 | NS | 0.11842462 |
| NEUROG2-AS1 | 0.655657108 | 0.137582931 | NA | NS | 0.861435444 |
| MIR302CHG | -0.009224998 | 0.933230963 | 0.963088299 | NS | 0.03001086 |
| ANK2-AS1 | 0.647602368 | 0.101454043 | NA | NS | 0.993730643 |
| LINC02262 | 1.09869929 | 0.048444692 | NA | Up | 1.314753801 |
| LINC02263 | 0.493027318 | 0.283445991 | 0.506380563 | NS | 0.547529681 |
| LINC01378 | 0.306276828 | 0.497396824 | 0.679709754 | NS | 0.303296992 |
| LINC02264 | 0.246832143 | 0.489572911 | NA | NS | 0.31018262 |
| SNHG8 | -0.421569315 | 0.029759291 | 0.192220466 | Down | 1.526377427 |
| METTL14-DT | 0.35774171 | 0.124739816 | 0.346235762 | NS | 0.903994899 |
| LINC01365 | 1.04098531 | 0.090886634 | NA | NS | 1.041499978 |
| LINC02502 | 0.503222405 | 0.623838515 | NA | NS | 0.204927816 |
| MAD2L1-DT | 0.098922649 | 0.448956308 | 0.642504366 | NS | 0.347795922 |
| NDNF-AS1 | 1.226720022 | 0.078144405 | NA | NS | 1.107102113 |
| PP12613 | 0.738621821 | 0.119863054 | NA | NS | 0.921314663 |
| BBS7-DT | 0.451327233 | 0.300651325 | NA | NS | 0.521936878 |
| IL21-AS1 | 0.212246956 | 0.402895208 | 0.607111252 | NS | 0.394807898 |
| LINC02435 | 0.589026579 | 0.325447472 | NA | NS | 0.487519098 |
| LINC01091 | 0.5612402 | 0.167683926 | 0.395909177 | NS | 0.775508565 |
| LINC02516 | 0.502365014 | 0.219389582 | NA | NS | 0.658783999 |
| LINC02379 | 0.329968904 | 0.571013575 | NA | NS | 0.243353567 |
| LINC02615 | -0.129667998 | 0.351508115 | 0.565262486 | NS | 0.454064644 |
| JADRR | 0.398202922 | 0.471439663 | NA | NS | 0.326573882 |
| LINC02466 | 0.499757057 | 0.384868546 | NA | NS | 0.414687581 |
| LINC02465 | 0.474828594 | 0.310088403 | 0.530296194 | NS | 0.508514476 |
| LINC02479 | 1.396775023 | 0.02271213 | NA | Up | 1.643742137 |
| LINC02377 | 0.708446579 | 0.219140775 | NA | NS | 0.659276807 |
| SNHG27 | 0.526659791 | 0.233338268 | NA | NS | 0.63201403 |
| LINC01256 | 1.554598245 | 0.037533233 | NA | Up | 1.42558403 |
| PCDH10-DT | 0.568708865 | 0.244381025 | 0.472332633 | NS | 0.611932518 |
| LINC02462 | 0.545777846 | 0.310773573 | NA | NS | 0.507555918 |
| LINC02485 | 0.630612098 | 0.33747203 | NA | NS | 0.471762216 |
| LINC00613 | 0.458478534 | 0.464651893 | NA | NS | 0.332872289 |
| LINC02511 | 0.315993055 | 0.554728651 | NA | NS | 0.255919403 |
| LINC02510 | 0.546991648 | 0.352389664 | NA | NS | 0.452976839 |
| LINC02172 | 1.085212559 | 0.113116638 | NA | NS | 0.946473512 |
| LINC00616 | 0.22080981 | 0.64947686 | NA | NS | 0.187436317 |
| SLC7A11-AS1 | 0.449600964 | 0.089882325 | 0.30028957 | NS | 1.046325703 |
| LINC00498 | -0.037586649 | 0.951491204 | NA | NS | 0.021595222 |
| LINC00499 | 0.524110586 | 0.135922479 | 0.360104507 | NS | 0.866708713 |
| LINC00500 | 1.181864375 | 0.080179843 | NA | NS | 1.095934801 |
| RAB33B-AS1 | 0.184620341 | 0.01251224 | 0.135545097 | Up | 1.902664944 |
| SCOC-AS1 | 0.229837622 | 0.332652256 | 0.549373822 | NS | 0.478009527 |
| LINC02432 | 0.301386435 | 0.040924847 | 0.218105862 | Up | 1.388012941 |
| LINC02276 | 0.438038439 | 0.206153616 | NA | NS | 0.685809042 |
| USP38-DT | 0.153089863 | 0.150581748 | 0.377054934 | NS | 0.822227667 |
| SMARCA5-AS1 | -0.309266239 | 0.005905963 | 0.10308115 | Down | 2.228709245 |
| HHIP-AS1 | 0.533991215 | 0.220132392 | NA | NS | 0.657316048 |
| LINC02266 | 0.415770487 | 0.47638132 | NA | NS | 0.322045277 |
| SMAD1-AS2 | 0.230782232 | 0.304026372 | NA | NS | 0.517088743 |
| SMAD1-AS1 | -0.068838506 | 0.868412153 | NA | NS | 0.061274107 |
| LINC01095 | 0.443724877 | 0.276448756 | NA | NS | 0.55838536 |
| LINC02507 | 0.537310377 | 0.214372598 | NA | NS | 0.66883073 |
| TMEM184C-DT | 0.39446034 | 0.319649438 | NA | NS | 0.495326055 |
| LINC02430 | -0.098270521 | 0.865389874 | NA | NS | 0.062788191 |
| LINC02355 | 0.895796156 | 0.064162654 | NA | NS | 1.192717683 |
| FHIP1A-DT | 0.086758023 | 0.79636667 | NA | NS | 0.098886925 |
| LINC02273 | 0.170901467 | 0.31117902 | 0.530925593 | NS | 0.506989691 |
| FBXW7-AS1 | 0.092228238 | 0.403602594 | 0.60743385 | NS | 0.394046051 |
| MIR4453HG | -0.031796763 | 0.606767513 | 0.758838518 | NS | 0.21697768 |
| NPY2R-AS1 | 1.234866077 | 0.016507765 | NA | Up | 1.782311721 |
| LINC02272 | 0.828324246 | 0.36487065 | NA | NS | 0.43786107 |
| LINC02433 | 0.781731028 | 0.293040057 | NA | NS | 0.53307301 |
| GASK1B-AS1 | 0.3402288 | 0.0039319 | 0.089958412 | Up | 2.405397554 |
| LINC02233 | 0.439333526 | 0.478110102 | NA | NS | 0.32047208 |
| LINC02477 | 0.872525926 | 0.202721546 | NA | NS | 0.69310009 |
| FAM218A | -0.118728598 | 0.411759257 | 0.614131594 | NS | 0.385356628 |
| LINC01179 | 0.894942842 | 0.149273855 | NA | NS | 0.826016251 |
| PALLD-AS1 | 0.907789529 | 0.089879486 | NA | NS | 1.046339419 |
| CBR4-DT | 0.771010859 | 0.119257442 | NA | NS | 0.923514512 |
| LINC02275 | 0.535795726 | 0.25090503 | 0.47774934 | NS | 0.600490633 |
| LINC01612 | 0.409871168 | 0.466039119 | NA | NS | 0.331577628 |
| LINC02512 | 0.590343755 | 0.294187868 | NA | NS | 0.531375241 |
| LINC02382 | 0.899084314 | 0.273300974 | NA | NS | 0.563358821 |
| LINC02431 | 0.696498522 | 0.24952447 | NA | NS | 0.602886858 |
| LINC02504 | 0.67415971 | 0.198822031 | NA | NS | 0.701535494 |
| LINC02174 | 0.45470994 | 0.368705153 | NA | NS | 0.433320793 |
| SAP30-DT | 0.185595607 | 0.250868818 | 0.47774934 | NS | 0.600553316 |
| HAND2-AS1 | 0.534111875 | 0.185427783 | 0.415463304 | NS | 0.731825194 |
| LINC02269 | 0.471503408 | 0.308593876 | NA | NS | 0.510612697 |
| LINC02268 | 0.5173022 | 0.233285929 | NA | NS | 0.632111456 |
| GPM6A-DT | -0.171477389 | 0.48710082 | NA | NS | 0.31238114 |
| SPCS3-AS1 | 0.091032151 | 0.635464141 | NA | NS | 0.196908952 |
| LINC02509 | 0.773414588 | 0.277541621 | NA | NS | 0.55667188 |
| AGA-DT | 0.052998546 | 0.72607496 | 0.838611255 | NS | 0.13901854 |
| LINC01098 | 0.90010275 | 0.099221707 | NA | NS | 1.003393305 |
| LINC01099 | -0.13688043 | 0.826147585 | NA | NS | 0.082942362 |
| LINC00290 | 0.344523148 | 0.578764389 | NA | NS | 0.237498198 |
| LINC02500 | 0.578093751 | 0.440265579 | NA | NS | 0.356285268 |
| TEMN3-AS1 | 0.722975242 | 0.376052579 | NA | NS | 0.424751429 |
| TENM3-AS1 | 0.914209415 | 0.062910489 | NA | NS | 1.201276941 |
| WWC2-AS2 | 0.815380724 | 0.006727368 | NA | Up | 2.172154835 |
| WWC2-AS1 | 0.200520297 | 0.562144916 | NA | NS | 0.250151713 |
| LINC02363 | 0.049113001 | 0.75215562 | 0.85466151 | NS | 0.123692295 |
| LINC02362 | 0.290182075 | 0.015911792 | 0.149655792 | Up | 1.798280916 |
| IRF2-DT | 0.119233771 | 0.249338396 | 0.476497659 | NS | 0.603210838 |
| LINC02427 | 0.400576807 | 0.01665826 | 0.152116213 | Up | 1.778370373 |
| LINC02365 | 0.210959075 | 0.295542564 | 0.517431069 | NS | 0.529379963 |
| MIR3945HG | 0.045981943 | 0.792241237 | 0.880216796 | NS | 0.101142556 |
| LINC01093 | 0.307449792 | 0.160247025 | 0.388015316 | NS | 0.795210025 |
| LINC02437 | 0.961849188 | 0.103771089 | NA | NS | 0.983923627 |
| LINC02436 | 0.59031983 | 0.156438045 | 0.383636723 | NS | 0.80565762 |
| F11-AS1 | 0.76930467 | 0.104664497 | NA | NS | 0.980200609 |
| LINC02374 | 0.82669897 | 0.175430811 | NA | NS | 0.755894129 |
| LINC02514 | 0.651652177 | 0.237321207 | NA | NS | 0.624663451 |
| LINC02515 | 0.641342334 | 0.145425611 | NA | NS | 0.837359104 |
| LINC02492 | 0.430866408 | 0.440204364 | NA | NS | 0.356345657 |
| LINC02434 | 0.982959286 | 0.152588346 | NA | NS | 0.816478635 |
| LINC01060 | 1.001004666 | 0.070353926 | NA | NS | 1.152711663 |
| LINC02508 | 0.509379249 | 0.367476692 | NA | NS | 0.434770202 |
| LINC01262 | -0.115782033 | 0.899379895 | NA | NS | 0.046056825 |
| DBET | 0.23271337 | 0.799446855 | NA | NS | 0.097210401 |
| PDCD6-DT | 0.024723817 | 0.842567799 | 0.911630734 | NS | 0.074395142 |
| EXOC3-AS1 | 0.112654288 | 0.418274709 | 0.618901466 | NS | 0.378538395 |
| PP7080 | 0.276810922 | 0.230247003 | NA | NS | 0.637806015 |
| SLC9A3-AS1 | 0.331354467 | 0.031600351 | 0.197250269 | Up | 1.500308094 |
| CTD-3080P12.3 | 0.724200081 | 0.15037733 | NA | NS | 0.822817629 |
| LINC02116 | 1.541928976 | 0.008829306 | NA | Up | 2.054073433 |
| IRX4-AS1 | 1.248063286 | 0.03646505 | NA | Up | 1.438123191 |
| CTD-2194D22.4 | 0.766767796 | 0.111821888 | NA | NS | 0.951473179 |
| LSINCT5 | 0.801964344 | 0.179372938 | NA | NS | 0.746243078 |
| C5orf38 | 0.923332974 | 0.093906303 | NA | NS | 1.027305256 |
| LINC01377 | 0.264909375 | 0.591560751 | NA | NS | 0.228000649 |
| LINC01019 | 0.699467312 | 0.135449263 | NA | NS | 0.868223353 |
| LINC02162 | 1.680142171 | 0.005442994 | NA | Up | 2.264162173 |
| LINC01017 | 0.370905293 | 0.569774961 | NA | NS | 0.24429664 |
| LINC02063 | 0.342835338 | 0.533799451 | NA | NS | 0.272621877 |
| LINC02114 | 0.888094148 | 0.256280718 | NA | NS | 0.591284068 |
| LINC01020 | 0.479698267 | 0.328832183 | 0.54647084 | NS | 0.483025685 |
| ADAMTS16-DT | 0.59121473 | 0.195138854 | 0.424804449 | NS | 0.70965625 |
| LINC02145 | 0.265744062 | 0.307710035 | NA | NS | 0.51185834 |
| LINC01018 | 0.461284447 | 0.111551256 | 0.329942771 | NS | 0.952525534 |
| LINC02102 | 0.179621222 | 0.399271191 | 0.604289507 | NS | 0.398732025 |
| LINC02236 | 0.423199572 | 0.162488818 | 0.390076509 | NS | 0.78917652 |
| LINC02196 | 0.048527099 | 0.870613957 | NA | NS | 0.060174375 |
| LINC02123 | 1.242642661 | 0.048912163 | NA | Up | 1.310583135 |
| LINC02142 | 0.987169936 | 0.107639103 | NA | NS | 0.968029928 |
| LINC02226 | 0.725589885 | 0.212011125 | NA | NS | 0.673641349 |
| MIR4458HG | 0.190580174 | 0.248761732 | 0.476309772 | NS | 0.604216428 |
| LINC02199 | 0.649816356 | 0.168458148 | 0.396682192 | NS | 0.773507978 |
| SEMA5A-AS1 | 0.259456284 | 0.224146859 | NA | NS | 0.649467342 |
| SNHG18 | 0.241325755 | 0.499451442 | NA | NS | 0.301506729 |
| LINC02112 | 0.607723609 | 0.226009304 | NA | NS | 0.645873682 |
| LINC02221 | 0.883745269 | 0.132456812 | NA | NS | 0.877925703 |
| ROPN1L-AS1 | 0.238492859 | 0.502992918 | NA | NS | 0.29843813 |
| LINC01513 | 0.188417696 | 0.259168363 | 0.485568555 | NS | 0.586418015 |
| LINC02212 | 0.262380328 | 0.183926037 | 0.414267712 | NS | 0.735356787 |
| LINC02213 | 0.107619345 | 0.570352984 | 0.732392713 | NS | 0.243856282 |
| ANKRD33B-AS1 | 0.832248436 | 0.001268809 | NA | Up | 2.896603874 |
| LINC01194 | 1.004654643 | 0.056074411 | NA | NS | 1.251235279 |
| LINC02220 | 0.458427253 | 0.498988129 | NA | NS | 0.301909786 |
| OTULIN-DT | -0.007096355 | 0.955268849 | 0.975854235 | NS | 0.019874384 |
| ANKH-DT | -0.181681939 | 0.624311613 | NA | NS | 0.204598586 |
| LINC02149 | 0.66235875 | 0.036884904 | NA | Up | 1.433151348 |
| CTD-2350J17.1 | 0.054986338 | 0.931303507 | NA | NS | 0.030908762 |
| MARCHF11-AS1 | 0.689191347 | 0.341197454 | NA | NS | 0.466994218 |
| MARCHF11-DT | 0.286322311 | 0.617628932 | NA | NS | 0.209272368 |
| LINC02150 | 0.220928488 | 0.307621131 | 0.52847792 | NS | 0.511983835 |
| RETREG1-AS1 | 8.99E-04 | 0.997452359 | NA | NS | 0.001107838 |
| BASP1-AS1 | 0.328631138 | 0.140500491 | 0.364826818 | NS | 0.852322158 |
| LINC02111 | 0.735736694 | 0.199622829 | NA | NS | 0.699789794 |
| LINC02217 | 0.358569608 | 0.081045826 | 0.287057145 | NS | 1.091269348 |
| LINC02223 | 0.228233048 | 0.499622714 | 0.681417305 | NS | 0.301357826 |
| LINC02100 | 0.031792768 | 0.745757326 | 0.850639911 | NS | 0.127402471 |
| CDH18-AS1 | 0.512454873 | 0.36049329 | NA | NS | 0.443102815 |
| LINC02146 | 0.239364151 | 0.686880466 | NA | NS | 0.163118834 |
| LINC02241 | 0.288533674 | 0.430497771 | 0.627698475 | NS | 0.366029093 |
| LINC02899 | 0.12732092 | 0.60678515 | NA | NS | 0.216965057 |
| LINC02239 | 0.386948053 | 0.529769178 | NA | NS | 0.275913312 |
| LINC02228 | 0.374232131 | 0.470822713 | NA | NS | 0.327142595 |
| LINC02211 | 0.212495264 | 0.601272034 | 0.755118014 | NS | 0.220928996 |
| PURPL | 0.271280533 | 0.495522641 | 0.678208303 | NS | 0.304936497 |
| LINC02103 | 0.386931535 | 0.540858418 | NA | NS | 0.266916407 |
| LINC02109 | 1.281766018 | 0.031965914 | NA | Up | 1.495312871 |
| LINC02064 | 0.495236681 | 0.349935336 | NA | NS | 0.456012201 |
| GOLPH3-DT | -0.069921581 | 0.691619896 | 0.81688906 | NS | 0.160132521 |
| LINC02061 | 0.494249404 | 0.331483161 | NA | NS | 0.479538528 |
| LINC02120 | 0.71888621 | 0.161303795 | NA | NS | 0.792355415 |
| LINC02160 | 0.312783121 | 0.486524092 | NA | NS | 0.31289565 |
| TARS1-DT | 0.138835836 | 0.340384605 | 0.556026263 | NS | 0.468030091 |
| TTC23L-AS1 | 0.115520665 | 0.566485355 | NA | NS | 0.246811313 |
| NADK2-AS1 | 0.26053994 | 0.055513139 | 0.248604264 | NS | 1.255604213 |
| NIPBL-DT | 0.055296101 | 0.327151384 | 0.545260243 | NS | 0.485251238 |
| GDNF-AS1 | 0.673093799 | 0.148396539 | 0.374564243 | NS | 0.828576227 |
| LINC02117 | 0.353289785 | 0.55137734 | NA | NS | 0.258551086 |
| LINC02119 | 0.514395243 | 0.254039866 | NA | NS | 0.595098125 |
| EGFLAM-AS4 | 0.655888292 | 0.248707385 | NA | NS | 0.604311318 |
| EGFLAM-AS3 | 0.846396652 | 0.150748287 | NA | NS | 0.821747615 |
| EGFLAM-AS2 | -0.158156196 | 0.783452044 | NA | NS | 0.105987582 |
| LIFR-AS1 | 0.373367901 | 0.392584384 | 0.599277982 | NS | 0.40606698 |
| OSMR-DT | 0.596879822 | 0.220920577 | 0.449641695 | NS | 0.655763832 |
| LINC02104 | 0.753944098 | 0.23957424 | NA | NS | 0.620559881 |
| LINC00603 | 0.555764565 | 0.109326513 | NA | NS | 0.961274503 |
| LINC00604 | 0.672742724 | 0.222290734 | NA | NS | 0.65307864 |
| OXCT1-AS1 | 0.332701636 | 0.114188727 | NA | NS | 0.942376769 |
| C5orf34-AS1 | 0.383831784 | 0.197424011 | NA | NS | 0.704600029 |
| NNT-AS1 | -0.020759792 | 0.773762406 | 0.868066358 | NS | 0.111392375 |
| FGF10-AS1 | 1.450762217 | 0.008235604 | NA | Up | 2.084304559 |
| LINC02224 | -0.034857975 | 0.945001717 | NA | NS | 0.024567402 |
| MRPS30-DT | 0.074706312 | 0.756197504 | 0.857433173 | NS | 0.121364761 |
| ISL1-DT | 0.510034154 | 0.302197309 | 0.523717198 | NS | 0.519709407 |
| LINC02118 | 0.242761064 | 0.720864485 | NA | NS | 0.142146371 |
| ITGA2-AS1 | 0.075223176 | 0.773189363 | NA | NS | 0.11171413 |
| MOCS2-DT | 0.071300924 | 0.54393581 | 0.713285597 | NS | 0.264452348 |
| LINC02105 | 0.573687949 | 0.28823511 | NA | NS | 0.540253119 |
| LINC01033 | 0.266153458 | 0.384820405 | 0.593278823 | NS | 0.414741908 |
| CCNO-DT | 0.249248557 | 0.070935178 | 0.272029043 | NS | 1.149138338 |
| FLJ31104 | 0.388813065 | 0.053611537 | 0.244657844 | NS | 1.270741741 |
| LINC01948 | 0.192746195 | 0.063281487 | 0.261404175 | NS | 1.198723323 |
| C5orf67 | 0.003926856 | 0.984095834 | 0.990467313 | NS | 0.006962607 |
| LINCR-0003 | 0.535543126 | 0.242342517 | 0.470094569 | NS | 0.615570385 |
| LINC02225 | 0.380347491 | 0.442635145 | NA | NS | 0.353954106 |
| LINC02101 | 0.275250785 | 0.614859121 | NA | NS | 0.21122438 |
| LINC02108 | 0.146803519 | 0.682536716 | NA | NS | 0.165873982 |
| PART1 | 0.29273039 | 0.113017987 | 0.331759797 | NS | 0.946852431 |
| ERCC8-AS1 | 0.85689305 | 3.40E-04 | NA | Up | 3.468352349 |
| SMIM15-AS1 | -0.078995388 | 0.378576242 | 0.588481122 | NS | 0.421846644 |
| LINC02057 | 0.413293626 | 0.46301923 | NA | NS | 0.334400971 |
| C5orf64 | 0.400193467 | 0.236719273 | 0.464633596 | NS | 0.625766381 |
| C5orf64-AS1 | 0.919895262 | 0.113462431 | NA | NS | 0.945147917 |
| ERBIN-DT | 0.065670612 | 0.624747906 | 0.771138884 | NS | 0.204295191 |
| LINC02065 | 0.466181097 | 0.0724029 | NA | NS | 1.140244041 |
| LINC02229 | 0.742186433 | 0.186950777 | NA | NS | 0.728272726 |
| MAST4-IT1 | -0.126884768 | 0.799662506 | NA | NS | 0.097093267 |
| MAST4-AS1 | 0.165753009 | 0.370110942 | NA | NS | 0.431668075 |
| LINC02242 | 1.762175203 | 0.012845911 | NA | Up | 1.891235086 |
| LINC02219 | 0.368334845 | 0.496146712 | NA | NS | 0.304389883 |
| LINC02198 | -0.027589746 | 0.960952537 | NA | NS | 0.017298062 |
| LINC02056 | 0.043005694 | 0.942074125 | NA | NS | 0.025914924 |
| FCHO2-DT | 0.064923131 | 0.804881879 | NA | NS | 0.09426785 |
| LINC01385 | 1.302322756 | 0.054012981 | NA | NS | 1.267501856 |
| LINC01386 | 0.486226988 | 0.474238309 | NA | NS | 0.324003367 |
| BTF3-DT | -0.025046819 | 0.89427542 | NA | NS | 0.048528706 |
| LINC02122 | 0.456369032 | 0.655570316 | NA | NS | 0.18338072 |
| LINC01331 | 0.256639796 | 0.346151286 | 0.561004652 | NS | 0.460734051 |
| LINC01335 | 0.570438215 | 0.208689079 | NA | NS | 0.680500278 |
| LINC01333 | 0.434992171 | 0.38959411 | NA | NS | 0.409387617 |
| LINC01332 | 0.956256506 | 0.143034635 | NA | NS | 0.844558788 |
| SV2C-AS1 | 1.196317334 | 0.043764613 | NA | Up | 1.358876908 |
| ZBED3-AS1 | 0.152291688 | 0.393532528 | 0.5998058 | NS | 0.405019365 |
| SCAMP1-AS1 | -0.033442758 | 0.629392845 | 0.77434085 | NS | 0.201078199 |
| LINC01455 | 1.072943417 | 0.09993542 | NA | NS | 1.000280557 |
| THBS4-AS1 | 0.281567291 | 0.014558142 | 0.144521266 | Up | 1.83689406 |
| FAM151B-DT | 0.31955698 | 0.011231242 | 0.13003023 | Up | 1.949572199 |
| LINC01337 | -0.163426949 | 0.538733871 | NA | NS | 0.268625719 |
| RASGRF2-AS1 | 0.225290038 | 0.224446885 | 0.452848432 | NS | 0.648886418 |
| CKMT2-AS1 | 0.05335775 | 0.465104194 | 0.655294755 | NS | 0.332449744 |
| ATG10-IT1 | 0.070905916 | 0.794169741 | NA | NS | 0.100086664 |
| ATG10-AS1 | 0.332331487 | 0.037169797 | 0.209820891 | Up | 1.429809814 |
| LINC01338 | 0.660885271 | 0.122283053 | NA | NS | 0.912633726 |
| VCAN-AS1 | 0.24364687 | 0.042247767 | 0.220751111 | Up | 1.374196239 |
| EDIL3-DT | 0.397931659 | 0.368401466 | 0.580230409 | NS | 0.43367865 |
| LINC02059 | 0.197782932 | 0.470971271 | NA | NS | 0.327005584 |
| MIR4280HG | 0.220112667 | 0.437154582 | 0.632879529 | NS | 0.359364965 |
| LINC01949 | 0.628965267 | 0.081473577 | NA | NS | 1.088983217 |
| LINC02488 | 0.193597495 | 0.764084855 | NA | NS | 0.116858409 |
| LINC02144 | 0.284703725 | 0.640259698 | NA | NS | 0.193643835 |
| TMEM161B-DT | 0.0661053 | 0.318130424 | 0.537421477 | NS | 0.497394796 |
| LINC02060 | 0.396723126 | 0.462126358 | NA | NS | 0.33523926 |
| LINC00461 | 0.564426757 | 0.157895814 | 0.385290564 | NS | 0.801629384 |
| MEF2C-AS2 | 0.153489059 | 0.146384441 | 0.371879808 | NS | 0.834505081 |
| MEF2C-AS1 | 0.060114229 | 0.48170052 | 0.668397777 | NS | 0.317222885 |
| LINC02161 | -0.315520721 | 0.389583481 | NA | NS | 0.409399467 |
| LINC01339 | 0.756890052 | 0.141068844 | NA | NS | 0.850568893 |
| LUCAT1 | 0.176895513 | 0.225979301 | 0.454435762 | NS | 0.645931339 |
| ARRDC3-AS1 | 0.119355932 | 0.044423388 | 0.225484138 | Up | 1.352388323 |
| LINC02058 | 0.390696829 | 0.559128225 | NA | NS | 0.252488584 |
| NR2F1-AS1 | 0.401109874 | 0.315007027 | 0.534426923 | NS | 0.501679757 |
| MCTP1-AS1 | 0.554281998 | 1.96E-04 | 0.023041407 | Up | 3.707889992 |
| LINC01554 | 0.38248032 | 0.190796427 | NA | NS | 0.719429763 |
| MIR583HG | 0.505771203 | 0.16826775 | NA | NS | 0.773999113 |
| LIX1-AS1 | -0.252189854 | 0.145837895 | 0.371191316 | NS | 0.836129614 |
| LINC01340 | 0.192288837 | 0.380424521 | 0.590393369 | NS | 0.419731498 |
| LINC02234 | 0.308432299 | 0.337159389 | NA | NS | 0.472164742 |
| LINC01846 | 0.056646096 | 0.92639087 | NA | NS | 0.033205734 |
| RGMB-AS1 | 0.416389159 | 0.115903976 | 0.33507105 | NS | 0.935901666 |
| CHD1-DT | 0.033386845 | 0.680930065 | 0.809943926 | NS | 0.16689749 |
| FAM174A-DT | 0.265181763 | 0.289263857 | 0.512431997 | NS | 0.538705827 |
| LINC00492 | 0.57559507 | 0.369600436 | NA | NS | 0.432267526 |
| LINC00491 | 0.428408332 | 0.378758295 | NA | NS | 0.421637847 |
| LINC02115 | 1.360980565 | 0.004154942 | NA | Up | 2.381435006 |
| LINC02163 | 0.506338966 | 0.371280208 | NA | NS | 0.430298201 |
| LINC01950 | 0.192274657 | 0.739980107 | NA | NS | 0.130779955 |
| LINC01023 | 0.650533389 | 0.140236823 | NA | NS | 0.853137935 |
| MAN2A1-DT | 0.158139616 | 0.597832062 | NA | NS | 0.223420797 |
| LINC01848 | 0.695461059 | 0.114106345 | NA | NS | 0.942690207 |
| STARD4-AS1 | 0.077587646 | 0.323974621 | 0.542570549 | NS | 0.48948901 |
| NREP-AS1 | -0.226325747 | 0.725444632 | NA | NS | 0.139395729 |
| EPB41L4A-AS1 | -0.141226608 | 0.14522275 | 0.370531575 | NS | 0.837965344 |
| EPB41L4A-DT | 0.754805776 | 0.00223413 | NA | Up | 2.650891516 |
| LINC02200 | 0.556373743 | 0.211193809 | NA | NS | 0.675318818 |
| LINC01957 | 0.669221444 | 0.472890761 | NA | NS | 0.32523917 |
| TRIM36-IT1 | 0.92398137 | 0.050479073 | NA | NS | 1.29688863 |
| TICAM2-AS1 | 0.032736559 | 0.602717915 | 0.756146026 | NS | 0.219885899 |
| LINCADL | 0.336494922 | 0.457770461 | NA | NS | 0.339352235 |
| SEMA6A-AS1 | 0.071574337 | 0.480198373 | 0.667051596 | NS | 0.318579316 |
| SEMA6A-AS2 | 0.434649353 | 0.344815092 | NA | NS | 0.462413734 |
| LINC02214 | 0.05277093 | 0.949824864 | NA | NS | 0.022356466 |
| LINC00992 | -0.079511182 | 0.898294776 | NA | NS | 0.046581126 |
| LINC02147 | 0.382019395 | 0.421958644 | NA | NS | 0.374730112 |
| LINC02208 | 0.343137491 | 0.366171852 | 0.57858521 | NS | 0.436315043 |
| LINC02148 | 0.160972223 | 0.790466276 | NA | NS | 0.102116654 |
| LINC02216 | 0.377601802 | 0.569321003 | NA | NS | 0.244642794 |
| LINC02215 | 0.638843942 | 0.30809602 | NA | NS | 0.511313912 |
| DMXL1-DT | 0.367112285 | 0.152794571 | 0.37932805 | NS | 0.815892077 |
| MGC32805 | 0.483046825 | 0.358818801 | NA | NS | 0.44512481 |
| LINC02201 | 0.354784083 | 0.360954702 | 0.573615336 | NS | 0.442547296 |
| PPIC-AS1 | 1.451934122 | 0.033071622 | NA | Up | 1.48054451 |
| LINC01170 | 0.476428946 | 0.357254546 | NA | NS | 0.447022237 |
| LINC02240 | 0.296376873 | 0.478974522 | 0.666351263 | NS | 0.319687588 |
| LINC02039 | -0.004791823 | 0.992203972 | NA | NS | 0.003399039 |
| LMNB1-DT | 0.254321999 | 0.40653379 | NA | NS | 0.390903351 |
| LINC01184 | 0.0977064 | 0.134453068 | 0.358010017 | NS | 0.871429284 |
| ADAMTS19-AS1 | 0.681963769 | 0.195777797 | NA | NS | 0.708236563 |
| ACSL6-AS1 | 0.524803106 | 0.31098088 | NA | NS | 0.507266312 |
| P4HA2-AS1 | 1.172914838 | 0.13414831 | NA | NS | 0.872414794 |
| MIR3936HG | 0.359754068 | 2.63E-04 | 0.026788981 | Up | 3.579734695 |
| IRF1-AS1 | 0.093959229 | 0.123869267 | 0.344887752 | NS | 0.907036433 |
| LINC02863 | 0.149384104 | 0.198544288 | 0.428432978 | NS | 0.702142603 |
| TH2LCRR | 0.431555141 | 0.008564776 | 0.118335308 | Up | 2.067283982 |
| AFF4-DT | 0.244779412 | 0.446487953 | NA | NS | 0.350190255 |
| WSPAR | 0.427252866 | 0.46633826 | NA | NS | 0.331298952 |
| PPP2CA-DT | -0.144098006 | 0.290512203 | 0.513491586 | NS | 0.53683562 |
| LINC01843 | 0.322364984 | 0.678598172 | NA | NS | 0.168387315 |
| PITX1-AS1 | 0.427523663 | 0.001682717 | 0.064977566 | Up | 2.77398896 |
| EPIST | 1.12289643 | 0.02933594 | NA | Up | 1.532599998 |
| LINC02900 | 0.802548874 | 0.10822666 | NA | NS | 0.965665745 |
| SLC25A48-AS1 | 0.602533409 | 0.206110683 | NA | NS | 0.685899497 |
| SMAD5-AS1 | 0.205961204 | 0.095444075 | 0.309119979 | NS | 1.020251026 |
| TRPC7-AS1 | 0.440659966 | 0.43855416 | NA | NS | 0.357976765 |
| TRPC7-AS2 | 0.520942278 | 0.471839233 | NA | NS | 0.326205951 |
| PKD2L2-DT | 0.09394536 | 0.318415948 | 0.537536335 | NS | 0.497005189 |
| FAM13B-AS1 | 0.284697545 | 0.088627148 | 0.298798608 | NS | 1.052433226 |
| CTNNA1-AS1 | 1.136025588 | 0.030930292 | NA | Up | 1.509615983 |
| SNHG4 | -0.016713356 | 0.832829047 | 0.905027323 | NS | 0.079444136 |
| CXXC5-AS1 | 0.503463311 | 0.019263537 | NA | Up | 1.715263963 |
| PSD2-AS1 | 0.761726476 | 0.013998017 | NA | Up | 1.853933479 |
| MALINC1 | 0.235529376 | 0.023427599 | 0.173918597 | Up | 1.630272218 |
| ANKHD1-DT | 0.134453714 | 0.248267669 | 0.476110795 | NS | 0.605079833 |
| DIAPH1-AS1 | 0.257575365 | 0.079618851 | 0.284911083 | NS | 1.098984093 |
| SPRY4-AS1 | 0.478485542 | 0.276394358 | 0.500948369 | NS | 0.558470826 |
| LINC01844 | 0.169627521 | 0.731366849 | 0.84106463 | NS | 0.135864729 |
| ARHGAP26-AS1 | 0.262626729 | 0.17570255 | 0.405108206 | NS | 0.755221935 |
| ARHGAP26-IT1 | 0.233948528 | 0.111143017 | 0.329714687 | NS | 0.954117817 |
| PPP2R2B-IT1 | 1.168113249 | 0.178926604 | NA | NS | 0.74732508 |
| STK32A-AS1 | 0.593233323 | 0.293545149 | NA | NS | 0.532325092 |
| JAKMIP2-AS1 | 0.022973058 | 0.871223204 | 0.927222822 | NS | 0.059870566 |
| FBXO38-DT | 0.173642504 | 0.167557583 | 0.395812663 | NS | 0.775835912 |
| SH3TC2-DT | 0.479536704 | 0.247109816 | 0.475332521 | NS | 0.607110003 |
| GRPEL2-AS1 | 0.204536907 | 0.216050381 | NA | NS | 0.665444964 |
| CARMN | 0.638684076 | 0.10802029 | 0.3257678 | NS | 0.966494662 |
| CLMAT3 | 0.247542012 | 0.231550823 | 0.45979109 | NS | 0.635353671 |
| ATOX1-AS1 | -0.033465166 | 0.840854521 | NA | NS | 0.075279136 |
| LINC01933 | 0.518193309 | 0.083850617 | 0.291624423 | NS | 1.076493739 |
| LINC01470 | 0.581660282 | 0.214840264 | NA | NS | 0.667884323 |
| LINC01861 | 0.900607921 | 0.083378326 | NA | NS | 1.078946827 |
| SAP30L-AS1 | 0.11166936 | 0.217529667 | 0.447145363 | NS | 0.662481506 |
| NIPAL4-DT | 0.067552078 | 0.558476065 | 0.724008899 | NS | 0.252995435 |
| LINC02227 | 0.635032182 | 0.100809679 | NA | NS | 0.99649777 |
| LINC02202 | 0.235730425 | 0.257431004 | 0.48432065 | NS | 0.58933915 |
| LINC01932 | 0.924152676 | 0.09203225 | NA | NS | 1.036059959 |
| LINC01845 | 0.179245146 | 0.430869114 | 0.62788015 | NS | 0.365654637 |
| LINC01847 | 0.514332741 | 0.246710097 | 0.47480291 | NS | 0.607813077 |
| FABP6-AS1 | 0.507625483 | 0.334188571 | NA | NS | 0.476008407 |
| MIR3142HG | 0.099121134 | 0.267567558 | 0.493587441 | NS | 0.572566545 |
| LINC02159 | 0.601174687 | 0.280164113 | NA | NS | 0.552587495 |
| LINC01202 | 0.837442881 | 0.093953856 | NA | NS | 1.027085391 |
| HMMR-AS1 | 0.986870237 | 0.094513632 | NA | NS | 1.024505546 |
| LINC02143 | 0.397585157 | 0.57482421 | NA | NS | 0.240464949 |
| LINC01938 | 0.20754709 | 0.690080427 | NA | NS | 0.16110029 |
| LINC01947 | 0.837754401 | 0.307717231 | NA | NS | 0.511848185 |
| CTB-178M22.2 | 0.740340341 | 0.187385147 | NA | NS | 0.727264837 |
| SLIT3-AS2 | 0.339245349 | 0.485773359 | NA | NS | 0.313566306 |
| SLIT3-AS1 | 0.438310408 | 0.378227198 | NA | NS | 0.422247245 |
| LINC01187 | 0.453583361 | 0.073546012 | NA | NS | 1.133440874 |
| LINC01366 | 0.070346411 | 0.646374386 | 0.786387676 | NS | 0.189515862 |
| KCNIP1-OT1 | -0.071844828 | 0.906584363 | NA | NS | 0.042591776 |
| KCNIP1-AS1 | 0.51260826 | 0.261052321 | NA | NS | 0.58327244 |
| LINC01944 | 0.614014649 | 0.280720305 | NA | NS | 0.551726173 |
| LINC01863 | 0.770938746 | 0.260584223 | NA | NS | 0.584051882 |
| LINC01942 | 1.020280695 | 0.083517919 | NA | NS | 1.078220334 |
| LINC01484 | 0.448105761 | 0.29859219 | 0.52004303 | NS | 0.524921555 |
| LINC01485 | 0.327683318 | 0.123336262 | 0.34408205 | NS | 0.908909217 |
| LINC01411 | 0.388280142 | 0.373105233 | 0.583824546 | NS | 0.428168659 |
| LINC01951 | 0.372262248 | 0.484069504 | NA | NS | 0.315092276 |
| THOC3-AS1 | 0.277448601 | 0.432671743 | NA | NS | 0.363841467 |
| LINC01574 | 0.428166611 | 0.577837051 | NA | NS | 0.238194615 |
| ZNF346-IT1 | 0.321522668 | 0.038698083 | 0.213650843 | Up | 1.412310551 |
| PRR7-AS1 | 0.1084143 | 0.304599267 | 0.525850476 | NS | 0.516271146 |
| PDLIM7-AS1 | 0.536346712 | 0.021306614 | NA | Up | 1.671485555 |
| FAM193B-DT | 0.385319959 | 0.180308898 | NA | NS | 0.743982841 |
| FAM153CP | 0.401335578 | 0.05452521 | 0.24676996 | NS | 1.26340265 |
| ZNF454-DT | 0.806425292 | 0.175449605 | NA | NS | 0.755847605 |
| LINC02222 | 0.141462289 | 0.787662924 | NA | NS | 0.103659596 |
| HEIH | -0.290775151 | 8.84E-04 | 0.049640343 | Down | 3.053378478 |
| LINC00847 | 0.081495859 | 0.143443904 | 0.36857986 | NS | 0.843317903 |
| LINC01962 | 0.136557888 | 0.308064094 | 0.528889667 | NS | 0.511358918 |
| TRIM7-AS1 | 0.390446936 | 0.23543227 | NA | NS | 0.62813401 |
| CTC-338M12.4 | 0.289924552 | 0.015841494 | 0.149362655 | Up | 1.800203869 |
| TRIM52-AS1 | -0.047312442 | 0.567959602 | 0.731081992 | NS | 0.245682554 |
| LINC01622 | 0.405245992 | 0.389707138 | 0.596947855 | NS | 0.409261639 |
| LINC01394 | 1.493137903 | 0.034454852 | NA | Up | 1.462749612 |
| FOXF2-DT | 0.762484893 | 0.169097761 | NA | NS | 0.771862142 |
| FOXCUT | 0.508792682 | 0.248522254 | NA | NS | 0.604634717 |
| GMDS-DT | 0.194773795 | 0.005136449 | 0.098521731 | Up | 2.289337051 |
| LINC02521 | 0.687592719 | 0.161477148 | NA | NS | 0.791888929 |
| LINC01600 | 0.763859654 | 0.076249644 | NA | NS | 1.117762178 |
| SERPINB9P1 | 0.047301251 | 0.519293019 | 0.696098842 | NS | 0.284587516 |
| LINC01011 | 0.024606139 | 0.822828626 | 0.899065897 | NS | 0.084690608 |
| NQO2-AS1 | -0.062543528 | 0.812591972 | NA | NS | 0.090127472 |
| LINC02525 | 0.913583143 | 0.095511829 | NA | NS | 1.01994284 |
| ECI2-DT | -0.004075697 | 0.984274139 | NA | NS | 0.006883925 |
| LINC02533 | 0.653706386 | 0.119513998 | NA | NS | 0.922581224 |
| KU-MEL-3 | 0.704947855 | 0.244232849 | NA | NS | 0.612195924 |
| CDYL-AS1 | 0.521190539 | 0.291304281 | NA | NS | 0.535653132 |
| LYRM4-AS1 | 0.406027226 | 0.088528357 | 0.298649128 | NS | 1.052917595 |
| LY86-AS1 | 0.051841316 | 0.611850414 | 0.762548878 | NS | 0.213354742 |
| HULC | 0.352704925 | 0.210438626 | 0.440341584 | NS | 0.676874543 |
| TFAP2A-AS2 | 0.619901326 | 0.168251051 | NA | NS | 0.774042213 |
| TFAP2A-AS1 | 0.140462513 | 0.779221439 | NA | NS | 0.108339107 |
| LINC00518 | 0.659699511 | 0.174806729 | NA | NS | 0.757441855 |
| MIR5689HG | 1.016384379 | 0.097204685 | NA | NS | 1.012312803 |
| LINC02522 | 0.687412844 | 0.497382677 | NA | NS | 0.303309344 |
| TMEM14B-DT | 0.310276023 | 0.110838347 | 0.329289192 | NS | 0.955309958 |
| ELOVL2-AS1 | 0.698318517 | 0.14658868 | NA | NS | 0.833899567 |
| LINC02530 | 0.533707071 | 0.428554803 | NA | NS | 0.367993633 |
| GFOD1-AS1 | 0.486047678 | 0.049071899 | NA | Up | 1.309167132 |
| LINC01108 | 0.266073812 | 0.27612997 | 0.500746581 | NS | 0.558886454 |
| JARID2-DT | 0.074655776 | 0.674921682 | NA | NS | 0.17074662 |
| JARID2-AS1 | 0.159982185 | 0.179999255 | 0.409996481 | NS | 0.744729293 |
| LINC02543 | 0.555540049 | 0.392599911 | NA | NS | 0.406049803 |
| ATXN1-AS1 | 0.21181995 | 0.021630569 | 0.168842295 | Up | 1.664932053 |
| NUP153-AS1 | 0.267955408 | 0.216172594 | NA | NS | 0.665199366 |
| MIR548A1HG | 0.282282885 | 0.485888265 | NA | NS | 0.313463589 |
| LNC-LBCS | 0.542530991 | 0.188050319 | 0.417520932 | NS | 0.725725924 |
| E2F3-IT1 | 0.228582865 | 0.13709053 | 0.361585547 | NS | 0.862992546 |
| LINC00581 | 0.376654973 | 0.518457812 | NA | NS | 0.285286577 |
| CASC15 | 0.267599092 | 0.370841997 | 0.581904895 | NS | 0.430811089 |
| NBAT1 | 0.266375047 | 0.436360705 | NA | NS | 0.360154365 |
| LINC02828 | -0.118327106 | 0.740983849 | NA | NS | 0.130191258 |
| HCG11 | -0.147467184 | 0.072139455 | 0.273763446 | NS | 1.141827144 |
| LINC00240 | 0.27005335 | 0.409871852 | 0.612921003 | NS | 0.387351906 |
| LINC01012 | 0.040051017 | 0.748067422 | 0.851877572 | NS | 0.126059258 |
| ZSCAN16-AS1 | 0.08870968 | 0.287360106 | 0.510520606 | NS | 0.541573526 |
| HLA-F-AS1 | 0.060725802 | 0.815494139 | 0.894851553 | NS | 0.088579156 |
| HCP5B | 0.290269125 | 0.484594504 | NA | NS | 0.314621516 |
| HCG18 | -0.161170983 | 0.114952969 | 0.333658762 | NS | 0.939479807 |
| LINC00243 | -0.283046222 | 0.329654665 | NA | NS | 0.481940774 |
| HCG27 | 0.075438083 | 0.864810599 | 0.923566908 | NS | 0.063078996 |
| LINC01149 | 0.732167959 | 0.4468593 | NA | NS | 0.3498292 |
| HCP5 | 0.632659366 | 0.146852248 | 0.372448525 | NS | 0.833119401 |
| SNHG32 | -0.243317278 | 0.094371168 | 0.307427263 | NS | 1.025160668 |
| TSBP1-AS1 | -0.480555425 | 0.133535108 | NA | NS | 0.874404537 |
| HLA-DQB1-AS1 | 1.051091288 | 0.115043323 | NA | NS | 0.939138581 |
| PSMB8-AS1 | -0.146552787 | 0.491851917 | NA | NS | 0.308165631 |
| LINC00336 | 1.128854386 | 0.003440806 | NA | Up | 2.463339754 |
| LINC01016 | 0.464285076 | 0.364501829 | NA | NS | 0.438300288 |
| ILRUN-AS1 | 0.08955412 | 0.520997408 | 0.696811896 | NS | 0.283164438 |
| BRPF3-AS1 | 0.241129854 | 0.166773535 | 0.394802555 | NS | 0.777872864 |
| ETV7-AS1 | 0.851150189 | 0.066995455 | NA | NS | 1.17395466 |
| DINOL | 1.345931834 | 0.02667388 | NA | Up | 1.573913815 |
| LINC02520 | -0.209978014 | 0.33722987 | 0.553386045 | NS | 0.472073965 |
| BTBD9-AS1 | 0.095720365 | 0.486086576 | 0.671572908 | NS | 0.313286372 |
| DNAH8-AS1 | 0.29163882 | 0.488988887 | NA | NS | 0.310701011 |
| DAAM2-AS1 | 0.087074871 | 0.805282702 | NA | NS | 0.09405163 |
| TDRG1 | 0.2646006 | 0.369405543 | 0.580846393 | NS | 0.432496592 |
| LINC00951 | 0.557838191 | 0.267720802 | NA | NS | 0.572317882 |
| FOXP4-AS1 | 0.049909712 | 0.77462081 | 0.868603887 | NS | 0.11091084 |
| LINC01276 | 0.287398848 | 0.539708101 | NA | NS | 0.267841063 |
| KLC4-AS1 | 0.55600245 | 0.25508294 | NA | NS | 0.593318587 |
| POLH-AS1 | 0.3578501 | 0.018609129 | 0.158787728 | Up | 1.730273964 |
| LINC02537 | 0.293906573 | 0.528109825 | NA | NS | 0.277275753 |
| LINC01512 | 0.709937826 | 0.212246987 | NA | NS | 0.673158466 |
| SCIRT | 0.817854892 | 0.144366836 | NA | NS | 0.84053256 |
| C6orf223 | 0.780394467 | 0.129917856 | NA | NS | 0.886331155 |
| RUNX2-AS1 | 0.221910522 | 0.317689417 | 0.537032996 | NS | 0.497997252 |
| ADGRF5-AS1 | 0.454679157 | 0.506372389 | NA | NS | 0.295529983 |
| CD2AP-DT | -0.212394153 | 0.541601004 | NA | NS | 0.266320539 |
| LINCMD1 | 0.428169393 | 0.524794877 | NA | NS | 0.280010413 |
| TRAM2-AS1 | 0.069233491 | 0.60099145 | 0.75493123 | NS | 0.221131707 |
| LINC01564 | 0.557044523 | 0.26488794 | NA | NS | 0.576937815 |
| MLIP-AS1 | 0.670451352 | 0.14963618 | 0.375764013 | NS | 0.824963386 |
| MLIP-IT1 | 0.416988077 | 0.474229395 | NA | NS | 0.32401153 |
| TINAG-AS1 | 0.792875142 | 0.209137847 | NA | NS | 0.679567368 |
| DST-AS1 | 1.105969587 | 0.015161102 | NA | Up | 1.819269244 |
| ZNF451-AS1 | 0.126630565 | 0.081334953 | 0.287675175 | NS | 1.089722781 |
| KHDRBS2-OT1 | 0.470864143 | 0.096807253 | NA | NS | 1.014092105 |
| SCAT8 | -0.044305886 | 0.733649548 | 0.842629987 | NS | 0.134511346 |
| FAM135A-AS1 | -0.111433475 | 0.787376632 | NA | NS | 0.103817479 |
| LINC00472 | 0.498945418 | 0.167296956 | 0.395361076 | NS | 0.776511962 |
| LINC01626 | 0.763176025 | 0.198552992 | NA | NS | 0.702123564 |
| KCNQ5-DT | 0.550739286 | 0.042588948 | 0.221206584 | Up | 1.37070309 |
| KCNQ5-AS1 | 0.354674591 | 0.02536179 | 0.180159958 | Up | 1.595820106 |
| KHDC1-AS1 | 0.152352188 | 0.153602527 | 0.380233538 | NS | 0.813601639 |
| TMEM30A-DT | 0.187435842 | 0.043759438 | 0.2243014 | Up | 1.358928261 |
| LINC02540 | 0.574531369 | 0.330520697 | NA | NS | 0.480801341 |
| HMGN3-AS1 | 0.055753504 | 0.477961102 | 0.665691023 | NS | 0.320607446 |
| LCAL1 | 0.219704699 | 0.639335047 | NA | NS | 0.194271488 |
| LINC01621 | 0.886134886 | 0.132612448 | NA | NS | 0.877415707 |
| LINC02542 | 0.273739007 | 0.553872799 | NA | NS | 0.256589963 |
| LINC02857 | 0.344630557 | 0.503192226 | NA | NS | 0.298266077 |
| LINC01611 | 0.118640255 | 0.851059088 | NA | NS | 0.070040286 |
| TBX18-AS1 | 0.233784598 | 0.646453941 | NA | NS | 0.189462413 |
| LINC02535 | 0.442686167 | 0.354195485 | NA | NS | 0.450756979 |
| SNHG5 | -0.085687581 | 0.164716134 | 0.392382726 | NS | 0.78326386 |
| PNRC1-DT | -0.946084667 | 0.008129561 | NA | Down | 2.089932921 |
| CASC6 | 0.474102848 | 0.344555037 | 0.559565981 | NS | 0.462741396 |
| LINC02531 | 0.927306009 | 0.020904345 | NA | Up | 1.67976343 |
| MANEA-DT | 0.04979485 | 0.568945544 | 0.731688258 | NS | 0.2449293 |
| UFL1-AS1 | 0.112779284 | 0.469181758 | 0.658326224 | NS | 0.328658882 |
| MCHR2-AS1 | 0.111316061 | 0.832890758 | NA | NS | 0.079411957 |
| SIM1-AS1 | 0.266330612 | 0.768551 | NA | NS | 0.114327308 |
| LIN28B-AS1 | 0.735127991 | 0.116175656 | NA | NS | 0.934884867 |
| BVES-AS1 | 0.644401195 | 0.16168501 | 0.38941477 | NS | 0.791330243 |
| LINC02836 | 0.615882151 | 0.096167437 | NA | NS | 1.01697196 |
| LINC02526 | 1.244214182 | 0.040646473 | NA | Up | 1.390977129 |
| LINC02532 | 0.4035248 | 0.248066349 | 0.476092812 | NS | 0.605432145 |
| OSTM1-AS1 | 0.636217602 | 0.241233297 | NA | NS | 0.617562748 |
| LINC00222 | 0.320139588 | 0.187342464 | 0.416819945 | NS | 0.727363771 |
| ARMC2-AS1 | 0.338502701 | 0.115021419 | NA | NS | 0.939221278 |
| REV3L-IT1 | 0.218580758 | 0.179568094 | 0.409812504 | NS | 0.745770828 |
| TRAF3IP2-AS1 | 0.103203976 | 0.088905302 | 0.299209951 | NS | 1.051072341 |
| LINC02527 | -0.162453082 | 0.766323659 | NA | NS | 0.115587766 |
| LAMA4-AS1 | 0.533770404 | 0.309065252 | NA | NS | 0.50994982 |
| LINC02518 | 0.041508043 | 0.910356845 | NA | NS | 0.040788338 |
| LINC02541 | -0.420479688 | 0.10706648 | 0.323901028 | NS | 0.970346477 |
| MROCKI | 0.187529727 | 0.179844316 | 0.409993482 | NS | 0.745103284 |
| LINC02880 | 0.269240868 | 0.569979456 | NA | NS | 0.244140797 |
| HDAC2-AS2 | 0.338548878 | 0.118526086 | 0.339042355 | NS | 0.926186058 |
| LINC02534 | 0.500120872 | 0.447476544 | NA | NS | 0.349229725 |
| CALHM6-AS1 | 0.185210515 | 0.640809548 | NA | NS | 0.193271026 |
| TRDN-AS1 | 0.296943062 | 0.547136053 | 0.715538481 | NS | 0.261904667 |
| RNF217-AS1 | 0.266114269 | 0.299139942 | 0.520433287 | NS | 0.524125595 |
| LINC02523 | 0.551016685 | 0.352591195 | NA | NS | 0.452728537 |
| NCOA7-AS1 | 0.240233137 | 0.079601597 | 0.284900136 | NS | 1.099078218 |
| LINC01013 | 0.281916593 | 0.131031223 | 0.35337695 | NS | 0.882625207 |
| LINC00326 | 0.386114301 | 0.409892301 | NA | NS | 0.387330239 |
| TARID | 0.289829738 | 0.538308294 | 0.709299611 | NS | 0.268968929 |
| LINC01312 | 0.857145827 | 0.102554697 | NA | NS | 0.989044445 |
| LINC01010 | 0.296266161 | 0.421230251 | NA | NS | 0.375480447 |
| CT69 | 0.392768287 | 0.456179179 | NA | NS | 0.340864541 |
| MYB-AS1 | 0.348671402 | 0.212010561 | NA | NS | 0.673642504 |
| LINC00271 | 0.434241156 | 0.112631558 | 0.331203909 | NS | 0.94833991 |
| MAP3K5-AS1 | 0.216122622 | 0.038679901 | 0.213650843 | Up | 1.412514649 |
| NHEG1 | 0.595184104 | 0.33391691 | NA | NS | 0.476361588 |
| LINC02539 | 0.333565099 | 0.527390826 | NA | NS | 0.277867429 |
| WAKMAR2 | -0.084801528 | 0.202667595 | 0.432824035 | NS | 0.693215687 |
| LINC02528 | 0.292186391 | 0.247834762 | NA | NS | 0.605837778 |
| LINC02865 | 0.373196594 | 0.421950141 | NA | NS | 0.374738863 |
| CCDC28A-AS1 | 0.200570451 | 0.351950664 | 0.565521232 | NS | 0.453518211 |
| LINC01625 | 0.312995375 | 0.268676787 | 0.494315213 | NS | 0.570769854 |
| FILNC1 | 0.132127729 | 0.738982373 | NA | NS | 0.131365921 |
| LINC01277 | 0.025462905 | 0.904977824 | 0.947388363 | NS | 0.043362063 |
| PHACTR2-AS1 | 0.173749232 | 0.233919068 | 0.46228643 | NS | 0.630934375 |
| HYMAI | 0.253467172 | 0.08110537 | 0.28717991 | NS | 1.090950388 |
| FBXO30-DT | 0.101074753 | 0.163268221 | 0.391057496 | NS | 0.787098339 |
| STXBP5-AS1 | 0.146836803 | 0.167751593 | 0.395942971 | NS | 0.775333346 |
| LUADT1 | 0.405211588 | 0.196280524 | NA | NS | 0.707122791 |
| UST-AS1 | 0.143646836 | 0.351767142 | 0.565305669 | NS | 0.45374473 |
| UST-AS2 | 0.427454116 | 0.114488491 | NA | NS | 0.941238168 |
| TAB2-AS1 | 0.710079757 | 0.059784812 | NA | NS | 1.223409132 |
| SYNE1-AS1 | 0.349905147 | 0.081972286 | NA | NS | 1.086332952 |
| NANOGP11 | 0.61863562 | 0.001379026 | 0.060387091 | Up | 2.860427615 |
| LINC02840 | 0.432688301 | 0.32983812 | 0.547189614 | NS | 0.481699154 |
| TMEM242-DT | 0.191052724 | 0.589052276 | NA | NS | 0.229846162 |
| SNX9-AS1 | 0.222470211 | 0.12662658 | 0.348597283 | NS | 0.897475124 |
| SYNJ2-IT1 | 0.253457738 | 0.553283033 | NA | NS | 0.257052648 |
| EZR-AS1 | 0.370470709 | 0.056118395 | NA | NS | 1.250894761 |
| LINC02901 | 0.107836395 | 0.751315183 | NA | NS | 0.124177834 |
| TAGAP-AS1 | 0.060129203 | 0.202280217 | 0.43247027 | NS | 0.694046589 |
| FNDC1-IT1 | 0.621680291 | 0.423897296 | NA | NS | 0.372739353 |
| LINC02529 | 0.645401072 | 0.355756461 | NA | NS | 0.448847204 |
| SOD2-OT1 | 0.015552851 | 0.916260294 | 0.953437582 | NS | 0.037981133 |
| AIRN | 0.231253115 | 0.094100391 | 0.30725936 | NS | 1.026408572 |
| MAP3K4-AS1 | -0.073935851 | 0.674223653 | 0.805943426 | NS | 0.171196016 |
| PACRG-AS2 | 0.338376089 | 0.532975925 | NA | NS | 0.273292408 |
| PACRG-AS3 | 0.267659148 | 0.448793039 | 0.642391137 | NS | 0.347953888 |
| PACRG-AS1 | 0.111470317 | 0.793031612 | 0.880637817 | NS | 0.1007095 |
| CAHM | 0.006443801 | 0.954112545 | 0.975190142 | NS | 0.020400394 |
| LINC00602 | 0.768186124 | 0.130831984 | NA | NS | 0.883286074 |
| LNCDAT | 0.721113031 | 0.135311113 | NA | NS | 0.868666534 |
| MPC1-DT | 0.129866088 | 0.531921059 | NA | NS | 0.274152816 |
| RPS6KA2-IT1 | 0.248394924 | 0.245839803 | NA | NS | 0.6093478 |
| RPS6KA2-AS1 | 0.007556322 | 0.974764258 | 0.985403414 | NS | 0.011100404 |
| HPAT5 | 1.14963053 | 0.017659389 | NA | Up | 1.753024329 |
| LINC02538 | 0.478659699 | 0.310207092 | NA | NS | 0.508348278 |
| LINC02487 | 0.399595801 | 0.413921893 | NA | NS | 0.383081603 |
| LINC01558 | 0.600424916 | 0.261590226 | NA | NS | 0.582378487 |
| AFDN-DT | 0.76915883 | 0.058658837 | NA | NS | 1.231666553 |
| KIF25-AS1 | 1.088429131 | 0.016357163 | NA | Up | 1.78629203 |
| LINC01615 | 0.920185823 | 0.091584019 | NA | NS | 1.038180302 |
| LINC02544 | 0.176418284 | 0.806207246 | NA | NS | 0.093553303 |
| LINC02519 | 0.487018669 | 0.306621908 | NA | NS | 0.513396819 |
| LINC01624 | 0.004786977 | 0.978142257 | NA | NS | 0.009597979 |
| HRAT92 | -0.046966788 | 0.768449295 | 0.865072048 | NS | 0.114384784 |
| PRKAR1B-AS2 | 0.009018528 | 0.98133853 | NA | NS | 0.008181149 |
| PRKAR1B-AS1 | -0.0446404 | 0.658462854 | 0.794568538 | NS | 0.18146872 |
| ZFAND2A-DT | 0.069651614 | 0.497192549 | 0.679630648 | NS | 0.303475388 |
| PSMG3-AS1 | 0.412747617 | 5.03E-04 | 0.038248966 | Up | 3.298687217 |
| ELFN1-AS1 | 0.633708123 | 0.230922088 | NA | NS | 0.636534525 |
| CARD11-AS1 | -0.077852942 | 0.559063043 | 0.724213069 | NS | 0.252539216 |
| SDK1-AS1 | 0.11856194 | 0.835182908 | NA | NS | 0.078218402 |
| RNF216P1 | 0.160245587 | 0.070958733 | 0.27207252 | NS | 1.14899415 |
| RBAKDN | 1.037647224 | 0.048133135 | NA | Up | 1.317555855 |
| RNF216-IT1 | 0.350805421 | 0.13656436 | NA | NS | 0.864662625 |
| MIOS-DT | 0.076089864 | 0.675706538 | 0.806656198 | NS | 0.170241879 |
| GLCCI1-DT | 0.514507323 | 0.003721882 | 0.087784297 | Up | 2.429237347 |
| MGC4859 | 0.553331703 | 0.307886401 | NA | NS | 0.511609493 |
| LINC02587 | 0.644437646 | 0.288739793 | NA | NS | 0.53949336 |
| CRPPA-AS1 | 0.179175132 | 0.308381354 | 0.52916651 | NS | 0.510911888 |
| LINC02888 | 0.640298649 | 0.192696088 | NA | NS | 0.715127103 |
| LINC02889 | 0.16269486 | 0.503571741 | 0.684479791 | NS | 0.297938649 |
| HDAC9-AS1 | 0.275154321 | 0.248061715 | NA | NS | 0.605440258 |
| MACC1-AS1 | -0.190770597 | 0.554295865 | NA | NS | 0.256258362 |
| ITGB8-AS1 | 0.38571645 | 0.205300817 | NA | NS | 0.687609323 |
| LINC01162 | 0.445822931 | 0.49467938 | NA | NS | 0.305676192 |
| IL6-AS1 | 0.195642294 | 0.415011069 | NA | NS | 0.38194032 |
| SNHG26 | 0.146417502 | 0.069289141 | 0.269644846 | NS | 1.15933482 |
| KLHL7-DT | 0.438780312 | 0.021414985 | 0.168379028 | Up | 1.669282229 |
| KIAA0087 | 0.329008962 | 0.411363861 | NA | NS | 0.385773864 |
| LINC02860 | 0.055189415 | 0.686471135 | 0.813787564 | NS | 0.163377719 |
| HOTAIRM1 | -0.201533548 | 0.083004839 | 0.290260272 | NS | 1.080896591 |
| HOXA-AS2 | 0.112278552 | 0.663695068 | 0.798689271 | NS | 0.178031409 |
| HOXA-AS3 | 0.745824536 | 0.075293279 | 0.278348131 | NS | 1.12324379 |
| HOXA10-AS | 0.658742363 | 0.173485552 | NA | NS | 0.760736689 |
| HOXA11-AS | 0.839468782 | 0.134253262 | NA | NS | 0.872075153 |
| HOTTIP | 0.639075699 | 0.164389519 | NA | NS | 0.784125875 |
| EVX1-AS | 0.967989568 | 0.146729146 | NA | NS | 0.83348361 |
| TAX1BP1-AS1 | 0.090322312 | 0.584745312 | 0.743660436 | NS | 0.233033252 |
| JAZF1-AS1 | 0.369168029 | 0.21154033 | 0.441234413 | NS | 0.674606822 |
| CPVL-AS1 | 0.02062357 | 0.909073565 | 0.949563038 | NS | 0.041400971 |
| CHN2-AS1 | 0.300121859 | 0.118656205 | 0.339258978 | NS | 0.925709545 |
| FKBP14-AS1 | 0.199739755 | 0.069932389 | 0.270539117 | NS | 1.155321635 |
| LINC01176 | 0.207293107 | 0.06971999 | 0.270202877 | NS | 1.156642685 |
| GARS1-DT | 0.104814209 | 0.083479932 | 0.290969351 | NS | 1.078417914 |
| LINC00997 | 0.434548257 | 0.203011354 | 0.432986484 | NS | 0.692479672 |
| NPSR1-AS1 | 0.420184774 | 0.358442132 | 0.571461309 | NS | 0.445580948 |
| HERPUD2-AS1 | 0.0048519 | 0.957001739 | 0.976356639 | NS | 0.019087273 |
| SEPTIN7-DT | 0.017277457 | 0.828983067 | 0.902732643 | NS | 0.08145434 |
| AOAH-IT1 | 0.060816631 | 0.593701583 | 0.749772128 | NS | 0.226431793 |
| ELMO1-AS1 | 0.416387666 | 0.016910678 | 0.153017917 | Up | 1.77183898 |
| TRG-AS1 | -0.008011496 | 0.922231093 | 0.957257818 | NS | 0.03516024 |
| POU6F2-AS2 | 0.92334488 | 0.043027486 | NA | Up | 1.366254027 |
| POU6F2-AS1 | 0.043417779 | 0.94769474 | NA | NS | 0.02333153 |
| LINC00265 | 0.124909388 | 0.326845306 | 0.545027756 | NS | 0.485657747 |
| CDK13-DT | 0.100108602 | 0.292575209 | 0.515268951 | NS | 0.533762476 |
| SUGCT-AS1 | 0.297023485 | 0.244822242 | NA | NS | 0.611149129 |
| LINC01450 | 0.636827276 | 0.172934546 | NA | NS | 0.762118242 |
| LINC01449 | 0.358146567 | 0.470535446 | NA | NS | 0.327407655 |
| INHBA-AS1 | 0.505308025 | 0.14005165 | 0.364288325 | NS | 0.853711771 |
| LINC01448 | 0.621043788 | 0.301856273 | NA | NS | 0.520199794 |
| HECW1-IT1 | 0.435025335 | 0.393819846 | NA | NS | 0.404702402 |
| LUARIS | 0.363921304 | 0.45689895 | NA | NS | 0.34017984 |
| POLR2J4 | -0.063630806 | 0.409706866 | 0.612834345 | NS | 0.387526757 |
| LINC00957 | 0.005238719 | 0.943813724 | 0.969292462 | NS | 0.025113712 |
| LINC01952 | -0.083882783 | 0.785792186 | NA | NS | 0.104692294 |
| SNHG15 | -0.179946657 | 0.023910223 | 0.175295276 | Down | 1.621416364 |
| LINC01447 | 0.502576139 | 0.22502214 | NA | NS | 0.64777475 |
| LINC02902 | 0.626806043 | 0.222550132 | NA | NS | 0.652572143 |
| LINC00525 | 0.719797195 | 0.023758502 | NA | Up | 1.624180937 |
| PKD1L1-AS1 | 0.24216773 | 0.688205862 | NA | NS | 0.162281633 |
| LINC02838 | 0.167523578 | 0.76880396 | NA | NS | 0.114184388 |
| DDC-AS1 | 0.432221038 | 0.525737319 | NA | NS | 0.279231194 |
| LINC01446 | 0.264750876 | 0.520348713 | 0.696688569 | NS | 0.283705515 |
| LINC02854 | 0.922532806 | 0.180625839 | NA | NS | 0.743220122 |
| LINC01445 | 0.298569059 | 0.529207721 | 0.70382202 | NS | 0.276373828 |
| VSTM2A-OT1 | 0.085934518 | 0.892308396 | NA | NS | 0.04948502 |
| SEC61G-DT | 0.413753416 | 0.180214903 | NA | NS | 0.744209297 |
| EGFR-AS1 | 0.346369623 | 0.552294081 | NA | NS | 0.257829611 |
| ELDR | 0.984848733 | 0.079966955 | NA | NS | 1.09708944 |
| LINC02848 | 0.009660362 | 0.955401435 | 0.975854235 | NS | 0.019814111 |
| LINC01005 | 0.529875066 | 0.305551057 | NA | NS | 0.51491621 |
| LINC00174 | 0.129363948 | 0.25517917 | 0.481988729 | NS | 0.59315478 |
| GS1-124K5.4 | 0.061613785 | 0.670416758 | 0.803363573 | NS | 0.173655138 |
| LINC02604 | 0.583848697 | 0.099765089 | NA | NS | 1.001021405 |
| CT66 | 0.792540785 | 0.1027944 | NA | NS | 0.988030543 |
| GTF2I-AS1 | 0.193237401 | 0.132165639 | 0.355111874 | NS | 0.878881441 |
| DTX2P1-UPK3BP1-PMS2P11 | -0.164622716 | 0.422143252 | 0.62165154 | NS | 0.374540149 |
| APTR | 0.166720716 | 0.034762651 | 0.204451526 | Up | 1.458887106 |
| DDX3ILA1 | 0.587392116 | 0.167525639 | NA | NS | 0.775918718 |
| MAGI2-AS1 | 0.43143444 | 0.608858913 | NA | NS | 0.215483332 |
| MAGI2-AS2 | 0.651048056 | 0.451908182 | NA | NS | 0.344949796 |
| MAGI2-AS3 | 0.004641051 | 0.980357718 | 0.988352515 | NS | 0.008615428 |
| CACNA2D1-AS1 | 0.147615614 | 0.754424079 | NA | NS | 0.122384459 |
| LINC00972 | -0.047712039 | 0.935915232 | NA | NS | 0.028763485 |
| GRM3-AS1 | 0.241241643 | 0.437014775 | NA | NS | 0.35950388 |
| TP53TG1 | -0.07491914 | 0.300795741 | 0.522222199 | NS | 0.521728317 |
| SRI-AS1 | -0.010625555 | 0.934159074 | 0.963746654 | NS | 0.029579163 |
| STEAP2-AS1 | 0.285051461 | 0.61060384 | NA | NS | 0.214240468 |
| CYP51A1-AS1 | 0.041211717 | 0.855965387 | NA | NS | 0.067543797 |
| CDK6-AS1 | 0.613277251 | 0.186458566 | NA | NS | 0.72941766 |
| TFPI2-DT | 0.430369961 | 0.52123112 | NA | NS | 0.282969663 |
| BET1-AS1 | -6.50E-04 | 0.994961293 | 0.997066567 | NS | 0.002193814 |
| PPP1R9A-AS1 | 0.522714348 | 0.080588393 | NA | NS | 1.093727506 |
| DLX6-AS1 | 0.361271853 | 0.363409468 | 0.575653869 | NS | 0.439603762 |
| STAG3L5P-PVRIG2P-PILRB | 0.269836671 | 0.275500904 | NA | NS | 0.559876971 |
| PPP1R35-AS1 | -0.04342031 | 0.891643775 | NA | NS | 0.049808618 |
| PCOLCE-AS1 | 0.29212973 | 0.358700097 | NA | NS | 0.445268506 |
| SLC12A9-AS1 | 0.409914498 | 0.139324581 | NA | NS | 0.855972254 |
| MUC12-AS1 | -0.301151585 | 0.344697279 | NA | NS | 0.462562145 |
| LNCPRESS1 | 0.894452886 | 0.12523546 | NA | NS | 0.902272686 |
| EMSLR | 0.119319705 | 0.758532169 | NA | NS | 0.120025996 |
| LINC01007 | 0.627251139 | 0.215618245 | NA | NS | 0.666314494 |
| NFE4 | 0.140627747 | 0.390947411 | 0.597849461 | NS | 0.407881659 |
| LHFPL3-AS1 | 0.23330904 | 0.581720386 | 0.740841922 | NS | 0.235285717 |
| LHFPL3-AS2 | 0.402906809 | 0.294610069 | NA | NS | 0.530752414 |
| LINC01004 | 0.089666762 | 0.049157513 | 0.234974405 | NS | 1.308410101 |
| KMT2E-AS1 | -0.015724318 | 0.858064825 | 0.919794085 | NS | 0.066479901 |
| LINC02577 | 0.272805366 | 0.568154527 | 0.731225533 | NS | 0.245533529 |
| PRKAR2B-AS1 | 0.25212616 | 0.408584705 | NA | NS | 0.388717895 |
| SLC26A4-AS1 | 0.489901517 | 0.121062043 | 0.341743241 | NS | 0.916992 |
| CBLL1-AS1 | 1.04E-04 | 0.999234965 | 0.999609713 | NS | 3.32E-04 |
| DOCK4-AS1 | 0.403513457 | 0.020785791 | 0.167382627 | Up | 1.682233438 |
| HRAT17 | 0.332895333 | 0.521982335 | NA | NS | 0.282344194 |
| LINC01393 | 0.19419569 | 0.430463556 | 0.627698475 | NS | 0.366063611 |
| LINC01392 | 0.489071251 | 0.232097008 | NA | NS | 0.634330458 |
| COMETT | 0.672417629 | 0.223589153 | NA | NS | 0.650549269 |
| ST7-AS1 | 0.262898872 | 0.353931456 | NA | NS | 0.451080837 |
| ST7-OT4 | 0.260424125 | 0.102354945 | 0.31812203 | NS | 0.989891171 |
| ST7-AS2 | 0.211799101 | 0.234291919 | 0.462445361 | NS | 0.630242691 |
| CFTR-AS1 | 0.433720271 | 0.615960064 | NA | NS | 0.210447445 |
| LINC02476 | 0.304025219 | 0.555192175 | NA | NS | 0.255556663 |
| FEZF1-AS1 | 0.606995905 | 0.244646023 | NA | NS | 0.611461841 |
| ASB15-AS1 | 0.226149449 | 0.709156497 | NA | NS | 0.149257914 |
| WASL-DT | -0.33345995 | 0.012233324 | 0.134364821 | Down | 1.91245553 |
| C7orf77 | 1.157604972 | 0.109952739 | NA | NS | 0.958793949 |
| POT1-AS1 | 0.255392918 | 0.23134419 | 0.459789912 | NS | 0.635741402 |
| LINC02830 | 0.240593947 | 0.416260428 | NA | NS | 0.380634873 |
| GRM8-AS1 | 0.561982214 | 0.300275947 | NA | NS | 0.522479455 |
| SND1-IT1 | 0.181276124 | 0.186853958 | 0.416322648 | NS | 0.728497698 |
| MESTIT1 | 0.721629814 | 0.007142642 | NA | Up | 2.146141103 |
| COPG2IT1 | 0.24046909 | 0.27540477 | 0.499985735 | NS | 0.560028541 |
| LINC00513 | 0.122463449 | 0.308070909 | 0.528889667 | NS | 0.51134931 |
| LINC-PINT | 0.125726399 | 0.12572974 | 0.347428564 | NS | 0.900561982 |
| MKLN1-AS | 0.210895997 | 0.056552345 | 0.249901284 | NS | 1.24754938 |
| FLJ40288 | 0.87170681 | 0.103635047 | NA | NS | 0.984493351 |
| CREB3L2-AS1 | 0.188483588 | 0.283767177 | 0.506589562 | NS | 0.54703784 |
| KDM7A-DT | -0.357399962 | 0.09988402 | 0.314828493 | NS | 1.000503986 |
| NDUFB2-AS1 | 0.556579491 | 0.010012165 | NA | Up | 1.999471996 |
| AGK-DT | 0.221258262 | 0.54585267 | NA | NS | 0.262924561 |
| TRBV11-2 | 0.204592234 | 0.216561958 | 0.446056476 | NS | 0.664417831 |
| TMEM139-AS1 | 0.527312676 | 0.208785429 | NA | NS | 0.680299814 |
| FAM131B-AS1 | 0.102460551 | 0.884073824 | NA | NS | 0.053511468 |
| EPHA1-AS1 | 0.018539896 | 0.931257261 | NA | NS | 0.030930328 |
| ARHGEF35-AS1 | 0.211762812 | 0.388479165 | 0.596204672 | NS | 0.410632269 |
| OR2A1-AS1 | -0.029464673 | 0.921143371 | NA | NS | 0.035672769 |
| CNTNAP2-AS1 | 0.821438303 | 0.19214538 | NA | NS | 0.716370053 |
| GHET1 | 0.265819031 | 0.264434452 | NA | NS | 0.577681963 |
| ATP6V0E2-AS1 | 0.045479892 | 0.663476528 | 0.798539598 | NS | 0.178174437 |
| REPIN1-AS1 | -0.034711697 | 0.802253228 | 0.88622436 | NS | 0.095688527 |
| ZNF775-AS1 | -0.043599708 | 0.776894955 | 0.870202787 | NS | 0.109637699 |
| LINC00996 | -0.091635758 | 0.536092462 | 0.708090984 | NS | 0.270760299 |
| WDR86-AS1 | -0.082614105 | 0.409238192 | 0.612333272 | NS | 0.388023843 |
| PRKAG2-AS1 | 0.44389765 | 0.007709155 | 0.114053667 | Up | 2.1129932 |
| LINC01003 | -0.081440741 | 0.383975792 | 0.592802518 | NS | 0.415696155 |
| LINC01287 | 0.28725963 | 0.422742325 | 0.622205725 | NS | 0.373924268 |
| PAXIP1-AS2 | -0.13766203 | 0.003582162 | 0.086556724 | Down | 2.445854783 |
| PAXIP1-DT | -0.014337266 | 0.874144102 | 0.929029107 | NS | 0.058416968 |
| HTR5A-AS1 | 0.534740354 | 0.361682347 | NA | NS | 0.441672688 |
| INSIG1-DT | -0.09373938 | 0.440880217 | 0.635713577 | NS | 0.355679388 |
| BLACE | 0.829438002 | 0.116588768 | NA | NS | 0.933343285 |
| LINC01006 | 0.258763597 | 0.128728826 | 0.350552625 | NS | 0.890324191 |
| LINC00244 | 0.482994611 | 0.434147926 | NA | NS | 0.362362269 |
| RNF32-AS1 | -0.007748727 | 0.944987211 | 0.970106106 | NS | 0.024574069 |
| MNX1-AS1 | 0.989264013 | 0.114960056 | NA | NS | 0.939453032 |
| PTPRN2-AS1 | 0.053848582 | 0.662336662 | 0.797602358 | NS | 0.178921205 |
| LINC01022 | 0.88653939 | 0.185919303 | NA | NS | 0.730675517 |
| LINC00689 | 0.31148453 | 0.217221321 | 0.446851943 | NS | 0.66309755 |
| FAM87A | 0.378880378 | 0.127874018 | 0.349506689 | NS | 0.89321769 |
| CLN8-AS1 | -0.352026699 | 0.068815778 | 0.268884683 | NS | 1.162311975 |
| MCPH1-DT | 0.161687016 | 0.09157784 | 0.303343316 | NS | 1.038209605 |
| MCPH1-AS1 | -0.178658584 | 0.567120173 | NA | NS | 0.246324904 |
| GS1-24F4.2 | 0.31907709 | 0.576049376 | NA | NS | 0.23954029 |
| FAM66B | 0.222231765 | 0.240295994 | NA | NS | 0.619253469 |
| MSRA-DT | -0.115055865 | 0.680344712 | NA | NS | 0.167270986 |
| LINCR-0001 | 0.007725684 | 0.978641156 | NA | NS | 0.009376524 |
| FAM167A-AS1 | 0.79030813 | 0.152815491 | NA | NS | 0.815832618 |
| LINC00208 | 0.437317757 | 0.505418732 | NA | NS | 0.296348666 |
| FAM66D | 0.635721149 | 0.01388695 | 0.141394243 | Up | 1.857393113 |
| FAM66A | -0.178171499 | 0.583613453 | NA | NS | 0.233874706 |
| MTUS1-DT | 0.949345976 | 0.13197055 | NA | NS | 0.879522973 |
| LZTS1-AS1 | 0.349213843 | 0.492386013 | NA | NS | 0.307694293 |
| LINC02153 | 0.49816275 | 0.076155538 | NA | NS | 1.118298509 |
| PIWIL2-DT | 0.117232502 | 0.552872987 | NA | NS | 0.257374629 |
| TNFRSF10A-AS1 | -0.020269101 | 0.818201573 | 0.896550239 | NS | 0.08713969 |
| TNFRSF10A-DT | 0.043695284 | 0.704347987 | 0.82477644 | NS | 0.152212722 |
| ENTPD4-DT | 0.221318379 | 0.528916694 | NA | NS | 0.276612725 |
| EXTL3-AS1 | 0.362993059 | 0.079582274 | 0.284900136 | NS | 1.099183656 |
| INTS9-AS1 | 0.206913723 | 0.195245396 | 0.424946251 | NS | 0.709419199 |
| HMBOX1-IT1 | 0.209091796 | 0.264045397 | 0.490244644 | NS | 0.578321399 |
| LINC00589 | 0.174596336 | 0.696642558 | NA | NS | 0.156989998 |
| LINC02099 | 0.398312099 | 0.338402426 | 0.554290685 | NS | 0.470566532 |
| LINC02209 | 0.585835272 | 0.187051994 | NA | NS | 0.728037658 |
| DCTN6-DT | 0.035542005 | 0.868795815 | 0.925923112 | NS | 0.06108228 |
| RBPMS-AS1 | 0.403767417 | 0.476766634 | NA | NS | 0.321694146 |
| NRG1-IT1 | 0.606586386 | 0.263512905 | NA | NS | 0.579198112 |
| NRG1-IT3 | 0.410298369 | 0.603210965 | NA | NS | 0.219530772 |
| LINC01288 | 0.542696021 | 0.295783678 | NA | NS | 0.529025795 |
| LINC01605 | 0.758570348 | 0.059954667 | NA | NS | 1.222177005 |
| C8orf86 | 0.569129022 | 0.230847967 | NA | NS | 0.636673946 |
| LINC02866 | 0.701765546 | 0.300440042 | NA | NS | 0.522242186 |
| SIRLNT | 0.21001735 | 0.694489235 | NA | NS | 0.158334482 |
| GPAT4-AS1 | 0.138736622 | 0.49288079 | NA | NS | 0.307258108 |
| IKBKB-DT | 0.411447884 | 0.366434705 | NA | NS | 0.436003401 |
| LINC00293 | 0.319202681 | 0.420203955 | 0.620351382 | NS | 0.376539864 |
| LINC02599 | 0.393935957 | 0.456053764 | NA | NS | 0.340983955 |
| LINC02847 | 1.144023575 | 0.09125154 | NA | NS | 1.039759799 |
| PENK-AS1 | 0.296579679 | 0.016645091 | 0.152056591 | Up | 1.778713827 |
| LINC00968 | -0.022492166 | 0.856362209 | 0.919130533 | NS | 0.067342506 |
| LINC01602 | 0.708445983 | 0.143053022 | NA | NS | 0.844502964 |
| LINC01301 | 0.303606019 | 0.366445674 | 0.578781989 | NS | 0.4359904 |
| LINC02842 | 0.243447848 | 0.505457001 | NA | NS | 0.296315784 |
| LINC02155 | -0.104683742 | 0.887224445 | NA | NS | 0.051966501 |
| YTHDF3-DT | -0.056574189 | 0.805673362 | NA | NS | 0.093840995 |
| LINC01414 | 0.48471281 | 0.27957839 | NA | NS | 0.5534964 |
| LINC01289 | 0.66150595 | 0.163680247 | NA | NS | 0.786003728 |
| MIR124-2HG | 0.504296982 | 0.320405742 | NA | NS | 0.49429971 |
| LINC00251 | 0.632368517 | 0.225948392 | NA | NS | 0.645990745 |
| LINC01299 | 0.516276843 | 0.279688908 | NA | NS | 0.553324756 |
| PDE7A-DT | 0.194231771 | 0.600120153 | NA | NS | 0.221761789 |
| LINC00967 | 0.490390455 | 0.326708654 | NA | NS | 0.485839361 |
| RRS1-DT | 0.279958992 | 0.035767586 | 0.206803647 | Up | 1.446510375 |
| C8orf44 | 0.149554192 | 0.01828746 | 0.157273532 | Up | 1.737846602 |
| SNHG6 | -0.173588212 | 0.167047477 | 0.395096697 | NS | 0.77716008 |
| ARFGEF1-DT | -0.012327236 | 0.913927375 | 0.952090827 | NS | 0.039088314 |
| C8orf34-AS1 | 0.625109371 | 0.199026101 | NA | NS | 0.701089964 |
| LINC01592 | 0.769230934 | 0.137401014 | NA | NS | 0.862010062 |
| LINC01603 | -0.196731881 | 0.762910267 | NA | NS | 0.11752654 |
| SLCO5A1-AS1 | 0.575551888 | 0.019865746 | 0.163656588 | Up | 1.701895121 |
| LACTB2-AS1 | 0.096135396 | 0.233991217 | 0.46228643 | NS | 0.630800444 |
| MSC-AS1 | 0.127023285 | 0.368590737 | 0.580406285 | NS | 0.433455583 |
| RDH10-AS1 | 0.21768961 | 0.140745512 | 0.365211163 | NS | 0.851565444 |
| STAU2-AS1 | 0.557570111 | 0.032944482 | 0.200078102 | Up | 1.482217318 |
| MIR2052HG | 0.859876911 | 0.018009662 | 0.15641156 | Up | 1.744494433 |
| CASC9 | 0.5600182 | 0.204439022 | NA | NS | 0.689436205 |
| LINC01109 | 0.738369137 | 0.216392463 | NA | NS | 0.66475787 |
| LINC01111 | 1.203706942 | 0.026528933 | NA | Up | 1.576280215 |
| ZFHX4-AS1 | 0.542396255 | 0.229455612 | 0.457791831 | NS | 0.639301316 |
| PKIA-AS1 | 0.13885551 | 0.275255493 | 0.499918696 | NS | 0.560264006 |
| LINC02605 | 0.116303879 | 0.44281771 | 0.637263491 | NS | 0.353775018 |
| LINC01607 | 0.392549934 | 0.469439665 | NA | NS | 0.328420218 |
| LINC02235 | 0.334085935 | 0.330944251 | NA | NS | 0.480245159 |
| LINC02839 | 0.841539925 | 0.125904645 | NA | NS | 0.899958246 |
| LINC01419 | -0.078368679 | 0.912895045 | NA | NS | 0.03957915 |
| E2F5-DT | 0.38028578 | 0.101840268 | NA | NS | 0.992080466 |
| CA3-AS1 | -0.114770739 | 0.544155212 | 0.713285597 | NS | 0.264277206 |
| LINC02849 | 0.300587262 | 0.496824839 | NA | NS | 0.3037967 |
| WWP1-AS1 | 0.007875906 | 0.939331545 | 0.966553041 | NS | 0.027181093 |
| RIPK2-DT | 0.066943908 | 0.332019571 | 0.548820802 | NS | 0.478836316 |
| LINC00534 | 0.283027492 | 0.197583701 | 0.427410856 | NS | 0.704248885 |
| LINC01030 | 0.673599969 | 0.021276142 | NA | Up | 1.672107129 |
| OTUD6B-AS1 | -0.205348505 | 0.040171234 | 0.216267417 | Down | 1.396084832 |
| FLJ46284 | 0.183177689 | 0.233149927 | 0.461424095 | NS | 0.632364716 |
| LINC02906 | -0.049889783 | 0.939473298 | NA | NS | 0.027115559 |
| CIBAR1-DT | 0.54900404 | 0.150184995 | NA | NS | 0.823373454 |
| RBM12B-DT | 0.476455121 | 0.272503437 | NA | NS | 0.564628016 |
| MIR378D2HG | 0.171032534 | 0.29594104 | 0.517745305 | NS | 0.528794805 |
| VIRMA-DT | 0.103319646 | 0.462163549 | 0.652999544 | NS | 0.33520431 |
| LINC02894 | 0.628718609 | 0.22819433 | NA | NS | 0.64169515 |
| MIR3150BHG | 0.203482389 | 0.141980973 | 0.3663829 | NS | 0.847769853 |
| CFAP418-AS1 | 0.052898859 | 0.719023388 | 0.834113749 | NS | 0.143256983 |
| LINC01298 | 0.274506321 | 0.591453878 | NA | NS | 0.228079116 |
| UQCRB-AS1 | 0.276729373 | 0.166671184 | NA | NS | 0.77813948 |
| VPS13B-DT | 0.208233848 | 0.033477483 | 0.201567051 | Up | 1.475247203 |
| FLJ42969 | 0.363692202 | 0.439246509 | NA | NS | 0.357291681 |
| ZNNT1 | 0.207802011 | 0.028642102 | 0.189026296 | Up | 1.542995112 |
| LINC02844 | 0.524902339 | 0.296210614 | NA | NS | 0.528399384 |
| LINC02845 | 0.257320126 | 0.67436299 | NA | NS | 0.171106273 |
| UBR5-DT | 0.190420391 | 0.121385577 | 0.342066258 | NS | 0.915832914 |
| GASAL1 | -0.093920932 | 0.330226287 | 0.547441347 | NS | 0.481188358 |
| MAILR | 0.055760071 | 0.562873897 | 0.727105118 | NS | 0.249588891 |
| LINC01181 | 0.684670076 | 0.424798843 | NA | NS | 0.371816675 |
| BAALC-AS2 | 0.180209681 | 0.745880774 | NA | NS | 0.127330587 |
| BAALC-AS1 | 0.333499478 | 0.166385606 | 0.394386581 | NS | 0.778884247 |
| ZFPM2-AS1 | 0.554722678 | 0.244753596 | NA | NS | 0.611270918 |
| OXR1-AS1 | 0.048848637 | 0.788317115 | NA | NS | 0.103299045 |
| LINC01608 | 0.547008874 | 0.239531084 | 0.46763242 | NS | 0.62063812 |
| LINC01609 | 0.570765216 | 0.267606752 | NA | NS | 0.572502934 |
| LINC02237 | 0.383735256 | 0.450259959 | 0.643605891 | NS | 0.346536672 |
| LINC00536 | 0.353921613 | 0.457656799 | NA | NS | 0.339460082 |
| RAD21-AS1 | 0.316148038 | 0.415144021 | NA | NS | 0.381801212 |
| SAMD12-AS1 | 0.206392661 | 0.404884358 | 0.60860084 | NS | 0.392669001 |
| MAL2-AS1 | 0.448209902 | 0.353888716 | NA | NS | 0.451133285 |
| HAS2-AS1 | 0.175571076 | 0.695163875 | 0.819215965 | NS | 0.157912804 |
| LINC02855 | 0.288795507 | 0.619641688 | NA | NS | 0.207859372 |
| SMILR | -0.125327878 | 0.619814401 | 0.767500028 | NS | 0.207738338 |
| LINC01151 | 0.265443331 | 0.300511921 | 0.521981344 | NS | 0.522138295 |
| FAM83A-AS1 | 0.291958582 | 0.533565561 | NA | NS | 0.27281221 |
| FER1L6-AS1 | 0.339470358 | 0.516557288 | NA | NS | 0.286881507 |
| FER1L6-AS2 | 0.180120556 | 0.692274941 | NA | NS | 0.159721389 |
| RNF139-DT | 0.06824999 | 0.327672354 | 0.545572683 | NS | 0.484560199 |
| LINC00964 | 0.567503783 | 0.162074502 | 0.389682175 | NS | 0.790285304 |
| SQLE-DT | 0.915197273 | 0.101266259 | NA | NS | 0.994535234 |
| WASHC5-AS1 | 0.277143828 | 0.055906082 | 0.249087638 | NS | 1.252540947 |
| LINC00861 | 0.173621711 | 0.072725044 | 0.274345075 | NS | 1.138316009 |
| PCAT1 | 0.178734702 | 0.113282224 | 0.332115589 | NS | 0.945838233 |
| CASC19 | 0.266697812 | 0.050208195 | 0.236988475 | NS | 1.299225392 |
| PRNCR1 | 0.237812912 | 0.096216851 | 0.310402489 | NS | 1.016748861 |
| CASC8 | 0.018792311 | 0.921571721 | 0.956746852 | NS | 0.03547086 |
| CCAT2 | -0.010134783 | 0.952201331 | 0.974149686 | NS | 0.021271216 |
| CASC11 | 0.581376263 | 0.119809206 | 0.340379194 | NS | 0.921509811 |
| PVT1 | 0.163025515 | 0.112551926 | 0.331021474 | NS | 0.948647068 |
| LINC02912 | 0.327887483 | 0.039341077 | 0.214674614 | Up | 1.405153751 |
| LINC00824 | 0.369481481 | 0.123333302 | 0.34408205 | NS | 0.908919639 |
| CCDC26 | 0.30553817 | 0.205148851 | 0.435206069 | NS | 0.68793091 |
| ASAP1-IT2 | 0.211259989 | 0.242420634 | 0.470182703 | NS | 0.615430418 |
| HPYR1 | 0.188640124 | 0.462013803 | 0.652934428 | NS | 0.33534505 |
| PTCSC1 | 0.222910177 | 0.05782717 | 0.251831441 | NS | 1.23786806 |
| ST3GAL1-DT | 0.244435188 | 0.027814539 | 0.18691566 | Up | 1.555728136 |
| NCRNA00250 | 0.69035205 | 0.177129458 | NA | NS | 0.751709207 |
| LINC01591 | 1.19983307 | 0.033110343 | NA | Up | 1.480036315 |
| LINC02055 | 0.344346012 | 0.373260612 | 0.583828339 | NS | 0.427987836 |
| PEG13 | 0.086765789 | 0.550287036 | 0.717855645 | NS | 0.259410718 |
| ERICD | 0.689463996 | 0.009382754 | NA | Up | 2.027669688 |
| DENND3-AS1 | 0.036597402 | 0.695486146 | 0.819469245 | NS | 0.157711517 |
| LINC00051 | 1.46942588 | 0.005223868 | NA | Up | 2.28200781 |
| LNCOC1 | 0.343742588 | 0.273405297 | 0.498634409 | NS | 0.563193075 |
| LY6E-DT | 0.307968659 | 0.229113274 | NA | NS | 0.639949748 |
| LINC02904 | 0.376602146 | 0.221165869 | NA | NS | 0.655281894 |
| MINCR | 0.059212673 | 0.526400066 | 0.701317887 | NS | 0.278684065 |
| RHPN1-AS1 | 0.818440366 | 0.08181226 | NA | NS | 1.087181611 |
| TONSL-AS1 | 0.556136543 | 0.129997335 | NA | NS | 0.88606555 |
| ZNF252P-AS1 | 0.25005411 | 0.023928172 | 0.175295276 | Up | 1.62109047 |
| MIR1302-9HG | 0.03941543 | 0.795942699 | 0.882340764 | NS | 0.099118196 |
| FAM138C | 0.563501959 | 0.408814214 | NA | NS | 0.388474013 |
| PGM5P3-AS1 | 0.751119427 | 0.088314292 | NA | NS | 1.053969011 |
| LINC01388 | 0.796267044 | 0.206458179 | NA | NS | 0.685167908 |
| DOCK8-AS1 | 0.141846893 | 0.176475964 | 0.406073799 | NS | 0.753314438 |
| LINC01230 | 0.646601702 | 0.183369183 | NA | NS | 0.73667365 |
| VLDLR-AS1 | 0.522788589 | 0.182665328 | 0.412849239 | NS | 0.738343879 |
| LINC01231 | 0.667398851 | 0.143968677 | 0.369130894 | NS | 0.841731985 |
| RFX3-AS1 | -0.009989137 | 0.927238156 | 0.959888272 | NS | 0.032808706 |
| GLIS3-AS1 | 0.191700532 | 0.720618498 | NA | NS | 0.142294594 |
| CDC37L1-DT | -0.266413739 | 0.102701787 | 0.318624114 | NS | 0.988421999 |
| LINC02851 | -0.098226069 | 0.799688784 | NA | NS | 0.097078995 |
| PTPRD-AS1 | 0.400554035 | 0.445231611 | NA | NS | 0.351414008 |
| PTPRD-AS2 | 0.685470841 | 0.180879484 | NA | NS | 0.74261069 |
| LURAP1L-AS1 | 0.248115731 | 0.626073761 | NA | NS | 0.203374497 |
| LINC01235 | 0.500366787 | 0.391719078 | NA | NS | 0.407025277 |
| C9orf92 | 0.289694811 | 0.617766165 | NA | NS | 0.209175882 |
| BNC2-AS1 | 0.389057497 | 0.192769031 | NA | NS | 0.714962735 |
| FOCAD-AS1 | 0.384616069 | 0.024964941 | 0.178950571 | Up | 1.602669458 |
| MIR31HG | 0.220335795 | 0.387537241 | NA | NS | 0.411686557 |
| CDKN2A-DT | 0.736849012 | 0.314152545 | NA | NS | 0.502859417 |
| CDKN2B-AS1 | 0.20549827 | 0.067326805 | 0.26723024 | NS | 1.171811995 |
| LINC01239 | 0.248275215 | 0.626179369 | NA | NS | 0.203301245 |
| LINC01241 | 0.73304765 | 0.159716981 | NA | NS | 0.796648908 |
| IFT74-AS1 | -0.152912066 | 0.664030868 | NA | NS | 0.177811732 |
| LINC01242 | 0.602780402 | 0.256506988 | NA | NS | 0.5909008 |
| LINC01243 | 0.728178148 | 0.291423895 | NA | NS | 0.535474841 |
| B4GALT1-AS1 | 0.126477982 | 0.533334992 | 0.706036878 | NS | 0.272999921 |
| CYP4F26P | 0.767987759 | 0.121462692 | NA | NS | 0.915557099 |
| PTENP1-AS | 0.209582007 | 0.276983859 | 0.501342318 | NS | 0.557545538 |
| LINC01251 | -0.265914387 | 0.542658588 | NA | NS | 0.26547332 |
| UBE2R2-AS1 | 0.155520138 | 0.195084353 | 0.424791696 | NS | 0.709777561 |
| CNTFR-AS1 | 0.599155329 | 0.254977899 | NA | NS | 0.593497461 |
| DNAJB5-DT | 0.786675559 | 0.081655708 | NA | NS | 1.088013453 |
| RMRP | -0.075684217 | 0.616325435 | 0.765484597 | NS | 0.210189909 |
| LINC01400 | 0.459254546 | 0.343011171 | NA | NS | 0.464691736 |
| EBLN3P | -0.003244749 | 0.942815445 | 0.968745091 | NS | 0.025573311 |
| LINC01627 | 0.635191789 | 0.370481471 | NA | NS | 0.431233508 |
| FAM201A | 0.233120385 | 0.052687055 | 0.242513843 | NS | 1.278296075 |
| GLIDR | -0.288635999 | 0.033845822 | 0.202385645 | Down | 1.470494932 |
| PTGER4P2-CDK2AP2P2 | 0.691107397 | 0.120287682 | NA | NS | 0.919778845 |
| FAM242F | 0.611189808 | 0.190019501 | NA | NS | 0.721201828 |
| FAM242D | 0.170335388 | 0.802725184 | NA | NS | 0.095433112 |
| FAM27C | 0.485844534 | 0.092148419 | 0.304133605 | NS | 1.035512113 |
| LINC01189 | 0.19788792 | 0.568466623 | NA | NS | 0.24529503 |
| LINC01410 | -0.014191418 | 0.908492638 | 0.949322371 | NS | 0.041678588 |
| LERFS | 0.786875231 | 0.063932041 | 0.262496805 | NS | 1.194281431 |
| FAM27E3 | 0.052413298 | 0.851233917 | NA | NS | 0.06995108 |
| PGM5-AS1 | 0.327029622 | 0.45838794 | NA | NS | 0.338766817 |
| TMEM252-DT | 0.125810611 | 0.449944227 | 0.643315188 | NS | 0.346841316 |
| LINC01506 | 0.109888225 | 0.495221714 | 0.678080097 | NS | 0.305200321 |
| BANCR | 0.547975957 | 0.294724474 | NA | NS | 0.530583799 |
| C9orf135-DT | 0.823386276 | 0.113599141 | NA | NS | 0.944624951 |
| MAMDC2-AS1 | 0.601084085 | 0.206277985 | NA | NS | 0.68554712 |
| SMC5-DT | 0.211082821 | 0.104501454 | 0.320548581 | NS | 0.980877667 |
| LINC01504 | 0.06654068 | 0.258837238 | 0.485454952 | NS | 0.586973244 |
| LINC01474 | 0.529371151 | 0.134130742 | NA | NS | 0.872471672 |
| RORB-AS1 | 0.799103748 | 0.077811956 | NA | NS | 1.10895367 |
| CARNMT1-AS1 | 0.029072357 | 0.772635274 | 0.867602583 | NS | 0.112025468 |
| PCA3 | 0.46269481 | 0.266191713 | 0.492079345 | NS | 0.57480547 |
| VPS13A-AS1 | 0.576285737 | 0.090290897 | NA | NS | 1.044356033 |
| GNA14-AS1 | 0.778116297 | 0.108215392 | NA | NS | 0.965710965 |
| LNCARSR | 0.64090306 | 0.213706904 | NA | NS | 0.670181448 |
| LINC01507 | 0.759939669 | 0.213630343 | NA | NS | 0.670337062 |
| UBQLN1-AS1 | -0.2524572 | 0.248256963 | 0.476110795 | NS | 0.605098561 |
| LINC02834 | 0.389124483 | 0.302530899 | NA | NS | 0.519230262 |
| GAS1RR | 0.301338494 | 0.356633604 | NA | NS | 0.447777738 |
| LINC02893 | 0.592012206 | 0.271193735 | NA | NS | 0.566720348 |
| LINC02872 | 0.681661106 | 0.130197896 | NA | NS | 0.885396034 |
| DAPK1-IT1 | 0.061247387 | 0.797912465 | 0.88374371 | NS | 0.09804475 |
| LINC02843 | 0.424954827 | 0.426256511 | NA | NS | 0.370328974 |
| MIR4290HG | 0.134407483 | 0.781854215 | NA | NS | 0.106874218 |
| LINC01508 | 0.23438046 | 0.664458635 | NA | NS | 0.17753205 |
| LINC01501 | 0.300597674 | 0.543165122 | NA | NS | 0.265068125 |
| LINC00484 | 0.172044449 | 0.296161463 | 0.517920726 | NS | 0.528471453 |
| BARX1-DT | 0.671866515 | 0.249023857 | NA | NS | 0.603759044 |
| MIRLET7A1HG | 0.101603598 | 0.103204161 | 0.319144166 | NS | 0.986302793 |
| LINC02603 | 0.135370809 | 0.124927257 | 0.346400199 | NS | 0.903342794 |
| PCAT7 | 0.414833389 | 0.265427057 | 0.491585012 | NS | 0.576054809 |
| LINC00476 | -0.10786918 | 0.158905644 | 0.386620319 | NS | 0.798860677 |
| LINC00092 | 0.338902026 | 0.020081805 | 0.164372226 | Up | 1.697197262 |
| SUGT1P4-STRA6LP | -0.045912135 | 0.633063646 | 0.776534704 | NS | 0.198552626 |
| SUGT1P4-STRA6LP-CCDC180 | -0.168382124 | 0.52393365 | NA | NS | 0.280723708 |
| PTCSC2 | 0.614515633 | 0.136973185 | NA | NS | 0.863364447 |
| NAMA | 0.428965034 | 0.305198131 | 0.526527126 | NS | 0.51541813 |
| STX17-DT | 0.159603537 | 0.387627027 | 0.595601085 | NS | 0.411585949 |
| TMEM246-AS1 | 0.493628205 | 0.326155374 | NA | NS | 0.486575461 |
| LINC00587 | 0.662579018 | 0.275027676 | NA | NS | 0.5606236 |
| LINC01492 | 0.408618765 | 0.458836094 | NA | NS | 0.338342426 |
| SMC2-DT | 0.081191997 | 0.708064658 | 0.82706671 | NS | 0.149927082 |
| CT70 | 0.586268624 | 0.104776103 | NA | NS | 0.97973776 |
| LINC01505 | 0.1613561 | 0.469467575 | 0.658565871 | NS | 0.328394398 |
| LINC01509 | 0.345339271 | 0.453915591 | NA | NS | 0.3430249 |
| HSDL2-AS1 | 0.16633185 | 0.138504971 | 0.363014451 | NS | 0.85853464 |
| FAM225B | 0.329409562 | 0.398212124 | NA | NS | 0.399885522 |
| FAM225A | 0.504501326 | 0.109580106 | NA | NS | 0.960268284 |
| DELEC1 | 0.376230019 | 0.060457858 | 0.256344114 | NS | 1.218547245 |
| LINC00474 | 0.509965662 | 0.469584038 | NA | NS | 0.328286674 |
| PAPPA-AS2 | 0.557771053 | 0.294874966 | NA | NS | 0.530362097 |
| ASTN2-AS1 | 0.288766566 | 0.10966573 | 0.327880488 | NS | 0.959929067 |
| LINC01613 | 0.78830573 | 0.094621094 | NA | NS | 1.024012037 |
| GSN-AS1 | 0.181188609 | 0.190865056 | 0.420152441 | NS | 0.719273577 |
| MIR600HG | 0.166248618 | 0.122298168 | 0.34308586 | NS | 0.912580049 |
| MIR181A2HG | -0.004589447 | 0.971199714 | 0.983568403 | NS | 0.012691454 |
| SLC25A25-AS1 | 0.065409713 | 0.697962345 | 0.821159601 | NS | 0.156168007 |
| PTGES2-AS1 | 0.200834501 | 0.209709086 | 0.439792418 | NS | 0.678382752 |
| ODF2-AS1 | 0.214572639 | 0.430468372 | NA | NS | 0.366058752 |
| HMGA1P4 | 0.045351955 | 0.69982873 | 0.822143007 | NS | 0.155008233 |
| LINC02913 | 0.237477375 | 0.067174825 | 0.266997195 | NS | 1.172793457 |
| LINC01503 | 0.065750871 | 0.599972699 | 0.75439633 | NS | 0.221868511 |
| LINC00963 | 0.088008167 | 0.273746892 | 0.498811736 | NS | 0.562650803 |
| GPRACR | 0.521015408 | 0.13238129 | NA | NS | 0.87817339 |
| DBH-AS1 | 0.387052156 | 0.116715548 | 0.336518862 | NS | 0.932871288 |
| WDR5-DT | 0.367295326 | 0.239044961 | NA | NS | 0.621520407 |
| LINC02247 | 0.564411164 | 0.311368592 | NA | NS | 0.506725197 |
| COL5A1-AS1 | 0.316174901 | 0.625939947 | NA | NS | 0.203467332 |
| LINC02907 | 0.655086084 | 0.215051126 | NA | NS | 0.667458279 |
| PPP1R26-AS1 | 0.385155403 | 0.075654463 | 0.278917983 | NS | 1.121165447 |
| LINC01502 | 0.92992001 | 0.072277279 | NA | NS | 1.140998209 |
| LINC02846 | 0.269892678 | 0.045274686 | 0.227404155 | Up | 1.344144549 |
| DKFZP434A062 | 1.008435679 | 0.04756873 | NA | Up | 1.322678448 |
| C9orf163 | 0.311644131 | 0.135831469 | NA | NS | 0.866999602 |
| NALT1 | 0.045914354 | 0.691727424 | 0.816889674 | NS | 0.160065006 |
| LINC01451 | 0.59490301 | 0.004252732 | 0.092669893 | Up | 2.371332031 |
| SNHG7 | -0.069561405 | 0.323873726 | 0.542561339 | NS | 0.489624282 |
| CCDC183-AS1 | 0.197107919 | 0.339178175 | 0.554994713 | NS | 0.469572101 |
| LINC02692 | 0.77752389 | 0.031191477 | NA | Up | 1.505964055 |
| LINC02908 | 0.108403464 | 0.293053247 | 0.515386052 | NS | 0.533053463 |
| MAN1B1-DT | 0.357510395 | 0.070619613 | 0.271500817 | NS | 1.15107467 |
| STPG3-AS1 | 0.447109413 | 0.223308248 | NA | NS | 0.651095236 |
| ARRDC1-AS1 | 0.047416138 | 0.448606439 | 0.642244462 | NS | 0.348134497 |
| DIP2C-AS1 | 0.435525843 | 0.237338732 | 0.465211541 | NS | 0.624631381 |
| IDI2-AS1 | 0.186097097 | 0.039875907 | 0.215796828 | Up | 1.399289429 |
| LINC00200 | 0.173926796 | 0.571174253 | 0.733159552 | NS | 0.243231378 |
| ADARB2-AS1 | 0.305525522 | 0.572735641 | NA | NS | 0.24204579 |
| LINC00700 | 0.234294505 | 0.603070509 | 0.756298368 | NS | 0.219631909 |
| LINC02662 | 0.287613457 | 0.569985616 | NA | NS | 0.244136104 |
| LINC00701 | 0.371262446 | 0.54157066 | NA | NS | 0.266344872 |
| LINC02645 | 0.525262289 | 0.291314716 | NA | NS | 0.535637576 |
| PFKP-DT | 0.211468039 | 0.524648455 | NA | NS | 0.280131601 |
| PITRM1-AS1 | 0.035946813 | 0.657508201 | 0.794202854 | NS | 0.182098826 |
| LINC02668 | 0.459830479 | 0.311078684 | NA | NS | 0.507129747 |
| LINC02669 | 0.078284391 | 0.684850477 | 0.812623472 | NS | 0.164404237 |
| LINC02639 | 0.320424707 | 0.533224306 | NA | NS | 0.273090062 |
| LINC02660 | 0.2294056 | 0.656974118 | NA | NS | 0.18245174 |
| LINC00702 | 0.482224327 | 0.287977621 | 0.511141864 | NS | 0.54064126 |
| LINC00703 | 0.282466643 | 0.569722184 | NA | NS | 0.24433687 |
| MANCR | 0.79644757 | 0.136362946 | NA | NS | 0.865303626 |
| LINC00705 | 0.338119848 | 0.554367206 | NA | NS | 0.256202468 |
| LINC02561 | -0.09022098 | 0.526243088 | 0.7012711 | NS | 0.278813595 |
| CALML3-AS1 | 0.694827618 | 0.098586851 | 0.31333179 | NS | 1.006181003 |
| LASTR | 0.744454815 | 0.113356959 | 0.332115589 | NS | 0.945551813 |
| LINC02649 | 0.275215408 | 0.075465519 | 0.278643389 | NS | 1.122251434 |
| LINC02656 | 0.127517272 | 0.428935146 | 0.626678972 | NS | 0.367608367 |
| PRKCQ-AS1 | -0.089711117 | 0.336262485 | 0.552487676 | NS | 0.473321582 |
| LINC02648 | -0.109880245 | 0.392352782 | 0.599004293 | NS | 0.406323264 |
| LINP1 | 0.311793676 | 0.55554147 | NA | NS | 0.255283516 |
| LINC00706 | 0.503793796 | 0.372734462 | NA | NS | 0.428600451 |
| LINC00707 | 0.472151116 | 0.017729195 | 0.155687895 | Up | 1.751310977 |
| LINC02665 | 0.57770409 | 0.358194308 | NA | NS | 0.44588132 |
| LINC02642 | 0.143845431 | 0.738170693 | NA | NS | 0.131843201 |
| GATA3-AS1 | 0.964733838 | 0.005243537 | NA | Up | 2.280375652 |
| LINC00708 | 0.896435594 | 0.095426648 | NA | NS | 1.02033033 |
| LINC02676 | 0.439813752 | 0.007564439 | 0.113104804 | Up | 2.121223286 |
| LINC02663 | 0.409254242 | 0.470215131 | NA | NS | 0.3277034 |
| LINC02670 | 0.61355484 | 0.280908531 | NA | NS | 0.551435072 |
| CELF2-DT | 0.293108004 | 0.520181236 | 0.696688569 | NS | 0.283845318 |
| SFTA1P | 0.36979001 | 0.431324682 | NA | NS | 0.365195689 |
| LINC00710 | 0.464235881 | 0.312803141 | 0.5324666 | NS | 0.504728895 |
| CELF2-AS2 | 0.361721578 | 0.173932037 | 0.403014115 | NS | 0.759620417 |
| CELF2-AS1 | 0.222028235 | 0.054688938 | 0.246795282 | NS | 1.262100511 |
| USP6NL-AS1 | 0.40484356 | 0.429353558 | NA | NS | 0.367184933 |
| PROSER2-AS1 | 0.499518304 | 0.187398503 | NA | NS | 0.727233883 |
| SUV39H2-DT | 0.019190446 | 0.898353049 | 0.943418999 | NS | 0.046552954 |
| RPP38-DT | 0.294944539 | 0.420287933 | NA | NS | 0.376453079 |
| LINC02654 | 0.412990628 | 0.398585363 | NA | NS | 0.399478654 |
| VIM-AS1 | -0.110940364 | 0.08982286 | 0.30028957 | NS | 1.046613121 |
| ST8SIA6-AS1 | -0.058004193 | 0.708565923 | 0.827346628 | NS | 0.149619738 |
| STAM-DT | 0.052045101 | 0.702485744 | 0.823782045 | NS | 0.153362485 |
| SLC39A12-AS1 | 0.395912694 | 0.387607334 | NA | NS | 0.411608014 |
| NEBL-AS1 | 1.735230356 | 0.008802343 | NA | Up | 2.055401698 |
| LINC02643 | 0.660659378 | 0.335281566 | NA | NS | 0.474590323 |
| MIR1915HG | 0.978605602 | 0.025294285 | NA | Up | 1.596977591 |
| LINC01516 | 0.801605832 | 0.161301533 | NA | NS | 0.792361506 |
| GPR158-AS1 | 1.060039258 | 0.10214453 | NA | NS | 0.990784886 |
| LINC00836 | 0.598174552 | 0.131217069 | 0.353836491 | NS | 0.882009666 |
| LINC02680 | 0.213469521 | 0.096867685 | 0.311014121 | NS | 1.013821078 |
| MKX-AS1 | 1.355748751 | 0.020267795 | NA | Up | 1.69319349 |
| MPP7-DT | 0.202239886 | 0.155877656 | 0.383185333 | NS | 0.807216134 |
| WAC-AS1 | -0.096703109 | 0.164169016 | 0.391978395 | NS | 0.784708805 |
| LINC01517 | 0.512443884 | 0.229334784 | 0.457791831 | NS | 0.639530069 |
| LINC00837 | 0.356182028 | 0.528398258 | NA | NS | 0.277038623 |
| LINC02664 | 0.68081146 | 0.021630811 | NA | Up | 1.664927207 |
| ZEB1-AS1 | 0.281304447 | 0.002177597 | 0.072832159 | Up | 2.662022396 |
| MACORIS | -0.299030832 | 0.329849738 | NA | NS | 0.481683857 |
| ITGB1-DT | 0.275132812 | 0.040449884 | 0.216951581 | Up | 1.393082716 |
| IATPR | 0.023588241 | 0.811146194 | 0.891618437 | NS | 0.090900865 |
| LINC02628 | 0.93089395 | 0.083350952 | NA | NS | 1.079089434 |
| LINC00838 | 0.554470053 | 0.20456204 | NA | NS | 0.689174955 |
| LINC02629 | 0.670779964 | 0.243102452 | NA | NS | 0.614210661 |
| PARD3-AS1 | 0.716927037 | 0.318792389 | NA | NS | 0.496492056 |
| LINC02635 | 0.292694346 | 0.618489351 | NA | NS | 0.208667774 |
| LINC02634 | 0.898173818 | 0.13293273 | NA | NS | 0.876368078 |
| CCNY-AS1 | 0.050185961 | 0.651078151 | 0.789632901 | NS | 0.186366878 |
| PCAT5 | 0.394896974 | 0.42439471 | NA | NS | 0.372230039 |
| LINC02630 | 1.141933714 | 0.073556386 | NA | NS | 1.133379619 |
| ZNF25-DT | 0.243475668 | 0.674497282 | NA | NS | 0.171019796 |
| LINC00839 | 0.532316255 | 0.216508334 | 0.445986136 | NS | 0.664525382 |
| LINC01518 | 0.62501369 | 0.078269603 | NA | NS | 1.10640687 |
| LINC02632 | 0.363540892 | 0.470550737 | NA | NS | 0.327393542 |
| LINC02623 | 0.922012354 | 0.081323967 | NA | NS | 1.089781442 |
| LINC01264 | 0.852215855 | 0.25063426 | NA | NS | 0.600959565 |
| CSGALNACT2-DT | 0.346550222 | 0.005298325 | 0.099529109 | Up | 2.275861424 |
| LINC02633 | 0.73988388 | 0.235043899 | NA | NS | 0.628851017 |
| LINC02916 | 0.174594227 | 0.350225623 | NA | NS | 0.455652084 |
| ZNF32-AS3 | 0.441895516 | 0.373999948 | NA | NS | 0.427128459 |
| ZNF32-AS1 | 0.111675579 | 0.425195924 | 0.623663211 | NS | 0.371410907 |
| ZNF32-AS2 | -0.038387948 | 0.74145966 | 0.848481332 | NS | 0.129912472 |
| LINC02658 | 0.732382776 | 0.40362405 | NA | NS | 0.394022964 |
| LINC00840 | 0.212548143 | 0.601243201 | 0.755118014 | NS | 0.220949821 |
| LINC02659 | 0.359562144 | 0.535952981 | NA | NS | 0.27087331 |
| LINC00841 | 0.30304181 | 0.497175284 | NA | NS | 0.303490469 |
| LINC02881 | 1.02228572 | 0.090627527 | NA | NS | 1.042739872 |
| TMEM72-AS1 | 0.374056759 | 0.185764838 | 0.415463304 | NS | 0.731036486 |
| ZNF22-AS1 | 0.160072682 | 0.275900505 | 0.500528626 | NS | 0.559247504 |
| LINC00842 | 0.429636778 | 0.42914495 | NA | NS | 0.367395994 |
| LINC02637 | 0.164079678 | 0.597238405 | NA | NS | 0.223852273 |
| FAM245B | 0.557342425 | 0.243480009 | NA | NS | 0.61353669 |
| LINC02675 | 1.117766884 | 0.05153355 | NA | NS | 1.28790994 |
| ARHGAP22-IT1 | 0.023659453 | 0.950616324 | NA | NS | 0.021994732 |
| FAM170B-AS1 | 0.399954682 | 0.036937385 | 0.209371187 | Up | 1.432533856 |
| C10orf71-AS1 | 0.809576253 | 0.223703936 | NA | NS | 0.650326375 |
| SGMS1-AS1 | 0.097817925 | 0.113785644 | 0.332423088 | NS | 0.94391253 |
| PRKG1-AS1 | 0.180529746 | 0.651857476 | 0.790244421 | NS | 0.185847349 |
| LNCAROD | 0.248003913 | 0.382850616 | 0.59214603 | NS | 0.41697065 |
| LINC02672 | 1.509008274 | 0.015612399 | NA | Up | 1.806530349 |
| LINC00844 | 0.331010981 | 0.499715207 | 0.681462288 | NS | 0.301277434 |
| LINC01553 | 0.646333479 | 0.171777647 | NA | NS | 0.76503335 |
| ANK3-DT | 0.272062544 | 0.273602489 | NA | NS | 0.562879956 |
| LINC00845 | 0.786132087 | 0.113548615 | NA | NS | 0.944818159 |
| LINC02625 | 0.232022018 | 0.577046942 | NA | NS | 0.238788856 |
| LINC02621 | 0.461461375 | 0.484274466 | NA | NS | 0.314908429 |
| JMJD1C-AS1 | 0.432855924 | 0.017501286 | 0.155294248 | Up | 1.756930036 |
| LINC02671 | 0.736291425 | 0.192454144 | NA | NS | 0.715672734 |
| LINC01515 | 0.599231118 | 0.209809545 | 0.439792418 | NS | 0.678174758 |
| LINC02640 | 0.398501648 | 0.465631203 | NA | NS | 0.331957924 |
| LINC02651 | 0.854582874 | 0.126712606 | NA | NS | 0.897180176 |
| LINC02636 | 0.306767833 | 0.526081604 | NA | NS | 0.278946885 |
| LINC02622 | 0.916751543 | 0.080223411 | NA | NS | 1.095698877 |
| UNC5B-AS1 | 0.631042448 | 0.212146966 | NA | NS | 0.673363176 |
| CDH23-AS1 | 0.144038204 | 0.862330883 | NA | NS | 0.06432606 |
| DNAJC9-AS1 | -0.088895072 | 0.355875679 | 0.568903557 | NS | 0.448701691 |
| PPP3CB-AS1 | 0.047361188 | 0.389779429 | 0.596958206 | NS | 0.409181084 |
| SYNPO2L-AS1 | 0.48968797 | 0.249983855 | NA | NS | 0.602088039 |
| BMS1P4 | 0.050054013 | 0.60436485 | 0.757299545 | NS | 0.218700802 |
| C10orf55 | 0.37965179 | 0.352730487 | NA | NS | 0.452557001 |
| ZNF503-AS1 | 0.509397233 | 0.213319001 | 0.443180155 | NS | 0.670970458 |
| ZNF503-AS2 | 0.606767111 | 0.014404639 | 0.143935548 | Up | 1.841497612 |
| KCNMA1-AS1 | 0.316184801 | 0.389115492 | 0.596625801 | NS | 0.409921478 |
| KCNMA1-AS2 | 0.88178602 | 0.152078337 | NA | NS | 0.817932644 |
| KCNMA1-AS3 | 0.805916657 | 0.035426192 | NA | Up | 1.450675524 |
| LINC00595 | 0.321784964 | 0.327318515 | 0.545360849 | NS | 0.485029427 |
| ZMIZ1-AS1 | 0.28020328 | 0.268207021 | 0.493891886 | NS | 0.571529858 |
| LINC02679 | 1.209867283 | 0.006707136 | NA | Up | 2.173462878 |
| NUTM2B-AS1 | 0.166584134 | 0.122187947 | 0.342932892 | NS | 0.912971632 |
| SFTPD-AS1 | 0.359620727 | 0.280219023 | 0.503488416 | NS | 0.552502385 |
| TMEM254-AS1 | 0.173022145 | 0.303146143 | 0.524458861 | NS | 0.518347952 |
| LINC00857 | 0.813335478 | 0.083880459 | NA | NS | 1.076339202 |
| LINC02655 | 0.926036665 | 0.090216521 | NA | NS | 1.044713923 |
| NRG3-AS1 | 0.975042438 | 0.122361946 | NA | NS | 0.912353625 |
| LINC02650 | 0.705945028 | 0.28413605 | NA | NS | 0.546473662 |
| CERNA2 | 0.285613473 | 0.504018751 | NA | NS | 0.297553306 |
| LINC00858 | 0.287640847 | 0.426776534 | 0.62485847 | NS | 0.369799468 |
| LINC01519 | 1.205022363 | 0.028361915 | NA | Up | 1.547264456 |
| LINC02647 | 0.597409046 | 0.303873976 | NA | NS | 0.517306492 |
| LINC01520 | 0.209210668 | 0.744119993 | NA | NS | 0.128357027 |
| GRID1-AS1 | 0.333294486 | 0.539096249 | NA | NS | 0.26833369 |
| ADIRF-AS1 | 0.153720582 | 0.202994129 | 0.432986484 | NS | 0.692516522 |
| NUTM2A-AS1 | 0.068586351 | 0.482152102 | 0.66853282 | NS | 0.316815936 |
| LINC00863 | 0.016848599 | 0.849861628 | 0.915672328 | NS | 0.070651779 |
| FAM245A | 0.630900866 | 0.11616367 | NA | NS | 0.934929676 |
| ACTA2-AS1 | 0.146844089 | 0.17958678 | 0.409814313 | NS | 0.745725636 |
| LINC00865 | 0.34034534 | 0.093268007 | 0.305740971 | NS | 1.030267304 |
| LINC01374 | -0.063326083 | 0.730108799 | 0.840420069 | NS | 0.136612418 |
| LINC01375 | 0.336920463 | 0.071314137 | 0.272521361 | NS | 1.14682437 |
| LINC02653 | 0.506628511 | 0.30880748 | NA | NS | 0.510312189 |
| LINC00502 | 0.619730018 | 0.286857996 | NA | NS | 0.54233304 |
| TNKS2-AS1 | 0.025622139 | 0.898748036 | NA | NS | 0.046362046 |
| PLCE1-AS2 | 0.45524252 | 0.314231663 | 0.53361135 | NS | 0.502750056 |
| PLCE1-AS1 | 0.739397647 | 0.203113778 | NA | NS | 0.692260616 |
| ENTPD1-AS1 | 0.173613894 | 0.086817305 | 0.296188519 | NS | 1.0613937 |
| SLIT1-AS1 | 0.591220314 | 0.281280752 | NA | NS | 0.550859986 |
| LINC00866 | 0.381211125 | 0.243980199 | NA | NS | 0.612645419 |
| LINC01475 | 0.805527843 | 0.108194424 | NA | NS | 0.965795121 |
| DNMBP-AS1 | 0.298486609 | 0.155577089 | 0.38303138 | NS | 0.808054358 |
| OLMALINC | 0.330447975 | 0.223640507 | 0.452317462 | NS | 0.650449531 |
| TLX1NB | 0.261120359 | 0.601554196 | NA | NS | 0.220725239 |
| LINC01514 | 0.26659321 | 0.6426929 | NA | NS | 0.191996498 |
| LBX1-AS1 | 0.679352829 | 0.103513982 | 0.319326135 | NS | 0.985000982 |
| LINC02681 | 0.583570937 | 0.303489492 | NA | NS | 0.517856341 |
| KCNIP2-AS1 | 0.028086357 | 0.894461266 | NA | NS | 0.048438462 |
| C10orf95-AS1 | 0.024421831 | 0.72594898 | 0.838611255 | NS | 0.1390939 |
| TRIM8-DT | 0.414920315 | 0.002890873 | 0.080543529 | Up | 2.538970939 |
| NEURL1-AS1 | 0.852855536 | 0.132949024 | NA | NS | 0.876314845 |
| SH3PXD2A-AS1 | 0.426181242 | 0.195856849 | NA | NS | 0.708061236 |
| ITPRIP-AS1 | 0.205322456 | 0.080224043 | 0.285870705 | NS | 1.095695457 |
| CFAP58-DT | 0.095306859 | 0.490542313 | 0.67490088 | NS | 0.309323526 |
| LINC02620 | 0.213895546 | 0.741912841 | NA | NS | 0.129647112 |
| SORCS3-AS1 | 0.667485551 | 0.229745692 | NA | NS | 0.638752623 |
| LINC02627 | 0.628793719 | 0.273189118 | NA | NS | 0.563536604 |
| LINC02624 | 0.217063474 | 0.66459172 | NA | NS | 0.177445074 |
| LINC01435 | 0.235948322 | 0.410031016 | 0.613001849 | NS | 0.387183291 |
| LINC02661 | -0.443992154 | 0.544265572 | NA | NS | 0.264189136 |
| ADD3-AS1 | 0.298675836 | 0.165836192 | 0.393793812 | NS | 0.780320684 |
| DUSP5-DT | 0.160327924 | 0.401696711 | 0.606078008 | NS | 0.396101724 |
| PDCD4-AS1 | 0.243709771 | 0.005695453 | 0.102180893 | Up | 2.24447173 |
| LINC02626 | 0.355334396 | 0.230883461 | NA | NS | 0.636607175 |
| MIR3663HG | 1.250019444 | 0.029682268 | NA | Up | 1.527502919 |
| EMX2OS | 0.682285138 | 0.064919245 | 0.264064926 | NS | 1.18762654 |
| LINC02674 | 0.835003985 | 0.110125596 | NA | NS | 0.958111729 |
| CASC2 | 0.504864396 | 0.118571524 | 0.339101527 | NS | 0.9260196 |
| LINC00867 | 0.165996617 | 0.212934742 | 0.442766611 | NS | 0.671753475 |
| GRK5-IT1 | 0.143303144 | 0.175425546 | 0.404762595 | NS | 0.755907164 |
| LINC01561 | 0.055950319 | 0.915638226 | NA | NS | 0.038276085 |
| WDR11-AS1 | 0.121347519 | 0.31898305 | 0.538089997 | NS | 0.496232393 |
| LINC01153 | 0.902851295 | 0.06282203 | NA | NS | 1.201888035 |
| LINC02641 | 0.198048383 | 0.372559888 | 0.583290029 | NS | 0.428803906 |
| FAM53B-AS1 | 0.106801176 | 0.521474387 | 0.697234983 | NS | 0.282767017 |
| TEX36-AS1 | 0.672852602 | 0.233702542 | NA | NS | 0.631336564 |
| EDRF1-DT | 0.172656364 | 0.491841854 | NA | NS | 0.308174517 |
| EDRF1-AS1 | 0.038491813 | 0.656817862 | 0.793964038 | NS | 0.182555045 |
| FANK1-AS1 | 0.639842463 | 0.391352475 | NA | NS | 0.407431915 |
| LINC00601 | 0.581107548 | 0.278092373 | NA | NS | 0.555810921 |
| LINC01163 | -0.079253784 | 0.628097853 | 0.773528315 | NS | 0.201972691 |
| LINC02667 | 0.61912608 | 0.249775335 | NA | NS | 0.602450449 |
| LINC02666 | -0.165173237 | 0.327499833 | 0.545444053 | NS | 0.484788917 |
| LINC02646 | 0.721920948 | 0.020638671 | NA | Up | 1.685318278 |
| TCERG1L-AS1 | 1.172422741 | 0.030956057 | NA | Up | 1.509254356 |
| LINC01164 | 0.775480793 | 0.137797932 | NA | NS | 0.860757301 |
| LINC02870 | 0.010744548 | 0.96882119 | NA | NS | 0.013756371 |
| LINC01165 | 1.592994045 | 0.006538115 | NA | Up | 2.184547414 |
| LINC01166 | 0.729982386 | 0.161300662 | NA | NS | 0.792363851 |
| LINC01167 | 0.956107391 | 0.166487028 | NA | NS | 0.778619598 |
| LINC01168 | 0.629768209 | 0.146695022 | 0.372132189 | NS | 0.833584622 |
| ADGRA1-AS1 | 0.64401049 | 0.160101308 | NA | NS | 0.795605121 |
| MIR202HG | 0.62917504 | 0.291201103 | NA | NS | 0.535806984 |
| LINC01001 | -0.178617744 | 0.465883842 | 0.656110672 | NS | 0.331722352 |
| LMNTD2-AS1 | -0.033177975 | 0.922190777 | NA | NS | 0.035179226 |
| MIR210HG | 0.436277379 | 0.232355045 | NA | NS | 0.633847893 |
| TOLLIP-DT | 0.105899088 | 0.50305295 | 0.684180691 | NS | 0.2983863 |
| LINC02689 | 0.601481368 | 0.346787898 | NA | NS | 0.459936067 |
| H19 | -0.35743903 | 0.429624188 | NA | NS | 0.366911276 |
| IGF2-AS | 1.271572414 | 0.060191557 | NA | NS | 1.220464424 |
| CD81-AS1 | 0.185942884 | 0.414471744 | NA | NS | 0.382505072 |
| KCNQ1OT1 | 0.159609916 | 0.179354492 | 0.409488244 | NS | 0.746287741 |
| CARS1-AS1 | -0.27438954 | 0.45275521 | NA | NS | 0.344136544 |
| MRGPRG-AS1 | 1.152804468 | 0.032078765 | NA | Up | 1.493782358 |
| STIM1-AS1 | -0.265590146 | 0.312022355 | NA | NS | 0.50581429 |
| RRM1-AS1 | 0.799155928 | 0.01187723 | NA | Up | 1.925284849 |
| LINC02749 | 0.53979423 | 0.200279034 | NA | NS | 0.698364512 |
| C11orf40 | 0.551323896 | 0.350761405 | NA | NS | 0.454988199 |
| BGLT3 | 0.184702997 | 0.765385059 | NA | NS | 0.11612002 |
| CASC23 | 0.579699507 | 0.346565156 | NA | NS | 0.460215104 |
| TUB-AS1 | 0.377237126 | 0.408306869 | NA | NS | 0.389013314 |
| RIC3-DT | 0.710404454 | 0.086573062 | NA | NS | 1.062617223 |
| TMEM9B-AS1 | 0.126847185 | 0.193794072 | 0.423056963 | NS | 0.712659511 |
| NRIP3-DT | 0.400563053 | 0.357192917 | NA | NS | 0.447097162 |
| LINC02709 | 0.445079278 | 0.013383795 | 0.138883402 | Up | 1.873420732 |
| SBF2-AS1 | -0.083765068 | 0.377777471 | 0.587802137 | NS | 0.422763945 |
| ADM-DT | 0.270158775 | 0.047507092 | 0.23161048 | Up | 1.323241552 |
| IRAG1-AS1 | 0.212176865 | 0.133364726 | 0.356521415 | NS | 0.874959021 |
| ZBED5-AS1 | 0.022620735 | 0.785501846 | 0.876214665 | NS | 0.10485279 |
| LINC02752 | 0.799850725 | 0.116177937 | NA | NS | 0.934876339 |
| LINC02547 | 0.594999285 | 0.29318277 | NA | NS | 0.532861556 |
| LINC00958 | 0.238539669 | 0.547432413 | 0.715885178 | NS | 0.261669492 |
| RASSF10-DT | 1.609926163 | 0.025830962 | NA | Up | 1.587859421 |
| FAR1-IT1 | 0.251069175 | 0.067887822 | 0.267924676 | NS | 1.168208126 |
| LINC02548 | 0.620374016 | 0.180738738 | NA | NS | 0.742948755 |
| LINC02545 | 0.961560503 | 0.038938286 | NA | Up | 1.409623171 |
| LINC02683 | 1.302163512 | 0.024531297 | NA | Up | 1.610279488 |
| SPON1-AS1 | 0.416577145 | 0.17919385 | NA | NS | 0.746676899 |
| LINC02751 | 0.444849985 | 0.047648026 | 0.231881658 | Up | 1.321955091 |
| LINC02682 | 0.382853853 | 0.482315876 | NA | NS | 0.316668443 |
| LINC02729 | 0.505751849 | 0.330851832 | NA | NS | 0.480366456 |
| IGSF22-AS1 | 0.634769038 | 0.083662733 | NA | NS | 1.077467954 |
| CSRP3-AS1 | -0.156868891 | 0.717884314 | NA | NS | 0.143945536 |
| NAV2-IT1 | 0.380605041 | 0.671596351 | NA | NS | 0.172891672 |
| NAV2-AS5 | 0.633793728 | 0.131062525 | NA | NS | 0.88252147 |
| NAV2-AS4 | 0.63572669 | 0.221565817 | NA | NS | 0.654497242 |
| NAV2-AS6 | 0.703784624 | 0.107882878 | NA | NS | 0.967047476 |
| NAV2-AS3 | 0.828861036 | 0.070145824 | NA | NS | 1.15399818 |
| SLC17A6-DT | 0.797901143 | 0.133651664 | NA | NS | 0.874025631 |
| LINC01495 | 0.262506876 | 0.68664212 | NA | NS | 0.163269559 |
| LINC02718 | -0.019592878 | 0.933395355 | 0.963171146 | NS | 0.029934365 |
| LINC02726 | 0.572135826 | 0.255171655 | NA | NS | 0.593167569 |
| LINC02686 | 0.170120566 | 0.809117074 | NA | NS | 0.091988634 |
| LINC02699 | 0.478128086 | 0.391221036 | NA | NS | 0.407577801 |
| ANO3-AS1 | 0.57668224 | 0.306895078 | NA | NS | 0.513010077 |
| BBOX1-AS1 | 1.066728476 | 0.038679529 | NA | Up | 1.412518821 |
| LGR4-AS1 | 0.389880974 | 0.173524357 | NA | NS | 0.760639556 |
| BDNF-AS | 0.103301646 | 0.406500382 | 0.610110213 | NS | 0.390939042 |
| LINC00678 | 0.652031261 | 0.059540776 | NA | NS | 1.225185508 |
| LINC02758 | -0.0178666 | 0.980352646 | NA | NS | 0.008617675 |
| LINC02742 | 0.228440121 | 0.691792765 | NA | NS | 0.160023984 |
| LINC02755 | 0.480810933 | 0.369091223 | NA | NS | 0.432866282 |
| LINC02546 | 0.686653549 | 0.205823432 | NA | NS | 0.686505185 |
| LINC01616 | 1.465636341 | 0.01372547 | NA | Up | 1.862472779 |
| ARL14EP-DT | 0.702437542 | 0.141050947 | NA | NS | 0.850623994 |
| MPPED2-AS1 | 0.432798168 | 0.361649432 | NA | NS | 0.441712213 |
| PAUPAR | 0.74735393 | 0.083744311 | 0.29147602 | NS | 1.077044687 |
| WT1-AS | 0.686429157 | 0.078193505 | 0.282681555 | NS | 1.10682932 |
| LINC00294 | 0.289469834 | 0.001788481 | 0.066048296 | Up | 2.747515656 |
| CSTF3-DT | -0.03952385 | 0.874677993 | 0.929380875 | NS | 0.0581518 |
| FBXO3-DT | 0.598142927 | 0.308841505 | NA | NS | 0.51026434 |
| LINC02722 | 0.530868794 | 0.396943153 | NA | NS | 0.401271685 |
| LINC02721 | 0.478934417 | 0.295156858 | NA | NS | 0.529947121 |
| LINC02707 | 0.57798118 | 0.286548687 | NA | NS | 0.542801577 |
| CD44-AS1 | 0.270680807 | 0.002345007 | 0.074602988 | Up | 2.629855947 |
| SLC1A2-AS1 | 0.194292552 | 0.739081123 | NA | NS | 0.13130789 |
| LINC02760 | 0.595796788 | 0.55436487 | NA | NS | 0.256204299 |
| LINC02759 | 0.387844304 | 0.611308608 | NA | NS | 0.213739489 |
| LINC01493 | 0.308298002 | 0.538181659 | NA | NS | 0.269071107 |
| LINC02741 | 0.778120591 | 0.145437947 | NA | NS | 0.837322263 |
| LINC01499 | 0.296377689 | 0.555300363 | NA | NS | 0.255472043 |
| LINC02745 | 0.546363675 | 0.345440185 | NA | NS | 0.461627142 |
| LINC02740 | 0.268650024 | 0.615865062 | NA | NS | 0.210514432 |
| MIR670HG | 0.681159762 | 0.249099024 | NA | NS | 0.603627974 |
| ALKBH3-AS1 | 0.173520713 | 0.146039049 | 0.371561664 | NS | 0.835531004 |
| LINC02704 | -0.426774327 | 0.271528647 | NA | NS | 0.566184344 |
| TSPAN18-AS1 | 0.780998937 | 0.019915925 | NA | Up | 1.700799519 |
| LINC02685 | 0.637768435 | 0.272465052 | NA | NS | 0.564689195 |
| LINC02696 | 0.211830445 | 0.245784032 | 0.474056417 | NS | 0.609446336 |
| LINC02687 | 0.29770615 | 0.547265567 | NA | NS | 0.261801876 |
| LINC02690 | 0.579364941 | 0.260964488 | NA | NS | 0.583418588 |
| LINC02716 | 0.48393446 | 0.141949367 | NA | NS | 0.847866541 |
| LINC02710 | 0.577574907 | 0.310103674 | NA | NS | 0.508493088 |
| LINC02489 | 0.560642927 | 0.32280741 | NA | NS | 0.491056505 |
| LRP4-AS1 | 0.148767991 | 0.777730847 | NA | NS | 0.109170675 |
| MADD-AS1 | -0.063806656 | 0.501316247 | 0.682850513 | NS | 0.29988822 |
| PTPRJ-AS1 | 0.213348675 | 0.170951464 | 0.399340109 | NS | 0.767127176 |
| LINC02750 | 1.002950214 | 0.087196475 | NA | NS | 1.059501071 |
| LINC02735 | 0.174892522 | 0.757078798 | NA | NS | 0.120858916 |
| FAM111A-DT | -0.024409565 | 0.717368444 | 0.833080072 | NS | 0.144257731 |
| LINC02739 | 0.421971092 | 0.173937451 | NA | NS | 0.7596069 |
| PATL1-DT | 0.198023123 | 0.363471448 | NA | NS | 0.439529698 |
| LINC02705 | -0.0389422 | 0.779086184 | 0.871860825 | NS | 0.108414497 |
| PRPF19-DT | -0.058132831 | 0.804906022 | 0.887888965 | NS | 0.094254823 |
| TMEM109-DT | -0.649622297 | 0.051290052 | NA | NS | 1.289966856 |
| MYRF-AS1 | 0.444819899 | 0.333048084 | NA | NS | 0.47749306 |
| LINC02733 | 0.851770871 | 0.089388407 | NA | NS | 1.0487188 |
| STX5-DT | 0.5753057 | 0.033358575 | NA | Up | 1.476792504 |
| SNHG1 | -0.154186749 | 0.038495018 | 0.213464789 | Down | 1.414595474 |
| PPP1R14B-AS1 | 0.419251285 | 0.318716444 | NA | NS | 0.496595529 |
| KCNK4-TEX40 | 0.99507643 | 0.266079563 | NA | NS | 0.574988481 |
| LINC02723 | -0.351601535 | 0.03849963 | 0.213464789 | Down | 1.414543441 |
| LINC02724 | 0.06148041 | 0.696761649 | 0.820254718 | NS | 0.156915762 |
| SF1-DT | 0.285847023 | 0.074457624 | 0.277058381 | NS | 1.128090826 |
| MIR194-2HG | 0.709900119 | 0.01993197 | NA | Up | 1.700449781 |
| NEAT1 | 0.132515558 | 0.42518636 | 0.623663211 | NS | 0.371420676 |
| LINC02736 | -0.010266713 | 0.965010977 | NA | NS | 0.015467747 |
| MALAT1 | 0.038914017 | 0.627784057 | 0.773353834 | NS | 0.202189718 |
| ZNRD2-DT | 0.169789973 | 0.103969582 | 0.319912852 | NS | 0.983093703 |
| RELA-DT | 0.109286795 | 0.166756218 | 0.394802555 | NS | 0.777917962 |
| OVOL1-AS1 | 0.500118879 | 0.442526321 | NA | NS | 0.354060892 |
| DPP3-DT | 0.028950616 | 0.688974748 | 0.815257617 | NS | 0.161796695 |
| NDUFV1-DT | 0.417657306 | 0.443858892 | NA | NS | 0.352755075 |
| OR7E11P | 0.27968982 | 0.708034455 | NA | NS | 0.149945608 |
| LINC02754 | 0.159040012 | 0.302812246 | 0.524267164 | NS | 0.518826566 |
| CHKA-DT | 0.701129834 | 0.099784951 | NA | NS | 1.00093495 |
| LINC02701 | 0.138463273 | 0.37101091 | 0.581978895 | NS | 0.430613319 |
| MRGPRF-AS1 | 1.002789973 | 0.041244781 | NA | Up | 1.384631003 |
| LINC02747 | 1.035894175 | 0.064723925 | NA | NS | 1.188935153 |
| LINC01488 | 0.652556779 | 0.169129471 | NA | NS | 0.771780708 |
| LINC02753 | 0.868976199 | 0.078482221 | NA | NS | 1.105228715 |
| LINC02584 | 0.287928534 | 0.71293779 | NA | NS | 0.146948364 |
| SHANK2-AS1 | 0.518561156 | 0.314412761 | NA | NS | 0.502499836 |
| SHANK2-AS3 | 0.644181071 | 0.202326624 | NA | NS | 0.693946965 |
| ALG1L9P | 0.209236826 | 0.082787668 | 0.290110921 | NS | 1.082034348 |
| LINC01537 | 0.835928772 | 0.070147729 | NA | NS | 1.153986382 |
| PDE2A-AS2 | 0.385369982 | 0.186033967 | NA | NS | 0.730407752 |
| PDE2A-AS1 | 0.618257167 | 0.179406449 | NA | NS | 0.746161949 |
| ARAP1-AS2 | 0.195688811 | 0.270730068 | 0.496040372 | NS | 0.567463507 |
| ARHGEF17-AS1 | 0.523104312 | 0.410965645 | NA | NS | 0.386194482 |
| P4HA3-AS1 | 0.659479264 | 0.142397149 | NA | NS | 0.846498707 |
| TPBGL-AS1 | 0.570072261 | 0.214554818 | 0.443838357 | NS | 0.668461728 |
| DGAT2-DT | 1.060069217 | 0.136595948 | NA | NS | 0.864562182 |
| UVRAG-DT | 0.19124083 | 0.14842506 | 0.374564243 | NS | 0.828492767 |
| LINC02761 | 0.556621357 | 0.096144459 | NA | NS | 1.017075739 |
| EMSY-DT | -0.1811596 | 0.140822456 | 0.365265155 | NS | 0.851328087 |
| LINC02757 | -0.081796701 | 0.78624483 | NA | NS | 0.104442197 |
| TSKU-AS1 | 0.546648072 | 0.481023014 | NA | NS | 0.317834145 |
| RSF1-IT2 | 0.174330746 | 0.07048987 | 0.271366448 | NS | 1.151873291 |
| RSF1-IT1 | 0.121415997 | 0.381283487 | 0.59100876 | NS | 0.418752003 |
| KCTD21-AS1 | -0.042574469 | 0.793756255 | 0.880874437 | NS | 0.100312839 |
| LINC02728 | 0.078186961 | 0.396550657 | 0.601745058 | NS | 0.401701326 |
| LINC02720 | 0.634755666 | 0.263504452 | NA | NS | 0.579212042 |
| MIR4300HG | 0.315089072 | 0.455454487 | NA | NS | 0.341555015 |
| LINC02734 | 0.492129048 | 0.026218099 | NA | Up | 1.581398804 |
| RAB30-DT | -0.035214697 | 0.629670005 | 0.774589928 | NS | 0.200886995 |
| ANKRD42-DT | 0.063834681 | 0.346718043 | 0.561331191 | NS | 0.460023558 |
| CCDC90B-AS1 | 0.263880575 | 0.471756244 | 0.660508711 | NS | 0.326282342 |
| DLG2-AS2 | 0.493599271 | 0.351497306 | NA | NS | 0.454077999 |
| LINC02695 | 0.653674415 | 0.118088434 | NA | NS | 0.927792636 |
| FZD4-DT | 1.324816714 | 0.004939585 | NA | Up | 2.306309515 |
| LINC02711 | 0.090620012 | 0.942096834 | NA | NS | 0.025904456 |
| GRM5-AS1 | 0.948946408 | 0.163005707 | NA | NS | 0.78779719 |
| DISC1FP1 | 0.101360756 | 0.590573464 | 0.747833322 | NS | 0.228726071 |
| LINC02748 | 0.569509974 | 0.34872368 | NA | NS | 0.457518561 |
| LINC02756 | 0.703218845 | 0.195456814 | NA | NS | 0.708949184 |
| LINC02746 | 0.487302999 | 0.422454619 | NA | NS | 0.374219937 |
| SCARNA9 | 0.20731433 | 0.064580598 | 0.263640796 | NS | 1.189897935 |
| PIWIL4-AS1 | 0.442288706 | 0.500694054 | NA | NS | 0.300427566 |
| LINC02700 | 0.357373239 | 0.171609274 | NA | NS | 0.765459246 |
| JRKL-AS1 | 0.089430007 | 0.886859664 | NA | NS | 0.052145097 |
| LINC02737 | 0.361172256 | 0.596481738 | NA | NS | 0.224402848 |
| LINC02553 | 0.459960398 | 0.56910744 | NA | NS | 0.244805737 |
| LINC02713 | 0.128911033 | 0.782660399 | NA | NS | 0.10642664 |
| ARHGAP42-AS1 | 0.520894272 | 0.237480682 | NA | NS | 0.624371713 |
| PGR-AS1 | 0.058554376 | 0.919181435 | NA | NS | 0.036598756 |
| TMEM123-DT | 0.462651628 | 0.098145924 | NA | NS | 1.008127734 |
| LINC02552 | 0.402379008 | 0.364230687 | 0.57647624 | NS | 0.438623467 |
| LINC02719 | 0.103251908 | 0.87968157 | NA | NS | 0.055674507 |
| LINC02715 | -0.25163947 | 0.590556206 | NA | NS | 0.228738762 |
| LINC02732 | 0.111244183 | 0.768670902 | NA | NS | 0.114259559 |
| LINC02550 | 0.093192008 | 0.807831648 | NA | NS | 0.092679137 |
| COLCA1 | 0.457840176 | 0.048704524 | 0.233958392 | Up | 1.3124307 |
| ALG9-IT1 | 0.164365534 | 0.53175796 | NA | NS | 0.274286 |
| LINC02762 | 0.100627829 | 0.255871945 | 0.482898993 | NS | 0.59197733 |
| LINC02763 | 0.274774751 | 0.314210171 | 0.53361135 | NS | 0.502779761 |
| LINC02764 | -0.041814514 | 0.88616128 | NA | NS | 0.05248723 |
| NCAM1-AS1 | 0.063141159 | 0.767147228 | 0.864377708 | NS | 0.11512128 |
| TTC12-DT | 0.173838347 | 0.560590991 | NA | NS | 0.251353886 |
| LINC00900 | 0.430532432 | 0.140467492 | 0.364826818 | NS | 0.85242417 |
| LINC02703 | 0.448780733 | 0.340794475 | NA | NS | 0.467507455 |
| LINC02151 | 0.410695571 | 0.48557471 | NA | NS | 0.31374394 |
| LINC02702 | 0.340956824 | 0.579329835 | NA | NS | 0.237074105 |
| APOA1-AS | 0.288576253 | 0.058806232 | 0.253109387 | NS | 1.230576644 |
| BACE1-AS | 0.301422599 | 0.033641248 | 0.201794618 | Up | 1.473127906 |
| TTC36-AS1 | 0.020146659 | 0.857049989 | 0.919521686 | NS | 0.066993846 |
| USP2-AS1 | 0.393711213 | 0.381216513 | 0.59100876 | NS | 0.418828296 |
| NECTIN1-AS1 | 0.209533988 | 0.60497085 | NA | NS | 0.218265551 |
| NECTIN1-DT | 0.606172913 | 0.129247573 | NA | NS | 0.888577604 |
| LINC02744 | 0.262590401 | 0.642090389 | NA | NS | 0.192403831 |
| SORL1-AS1 | 0.304771945 | 3.57E-04 | 0.032222893 | Up | 3.447371381 |
| MIR100HG | 0.506649352 | 0.165352752 | 0.393199952 | NS | 0.781588571 |
| LINC02727 | 0.518863715 | 0.334919993 | NA | NS | 0.475058926 |
| ESAM-AS1 | 0.04781243 | 0.722772508 | 0.836344784 | NS | 0.140998375 |
| CCDC15-DT | 0.381537694 | 0.150474841 | NA | NS | 0.822536107 |
| PKNOX2-DT | 1.383369894 | 0.028837257 | NA | Up | 1.540046049 |
| GSEC | 0.357388797 | 0.014455303 | 0.144187871 | Up | 1.839972798 |
| KIRREL3-AS1 | 0.594694435 | 0.236782221 | NA | NS | 0.62565091 |
| KIRREL3-AS2 | 0.925267867 | 0.12653063 | NA | NS | 0.897804331 |
| KIRREL3-AS3 | 0.824287544 | 0.082170693 | NA | NS | 1.085283052 |
| LINC02712 | 0.553654143 | 0.301618359 | NA | NS | 0.520542228 |
| LINC02725 | 0.065838856 | 0.665446982 | 0.799583776 | NS | 0.17688654 |
| LINC02098 | 0.492655348 | 0.352104769 | NA | NS | 0.453328093 |
| ETS1-AS1 | 0.95812583 | 0.031013498 | NA | Up | 1.508449252 |
| SENCR | 0.073701982 | 0.381869964 | 0.591317506 | NS | 0.4180845 |
| LINC01395 | 0.014056431 | 0.945146039 | 0.970182178 | NS | 0.024501082 |
| LINC00167 | -0.018113489 | 0.911168507 | 0.950817021 | NS | 0.040401299 |
| ZBTB44-DT | 0.177914902 | 0.232531562 | 0.46097973 | NS | 0.633518091 |
| LINC02873 | 0.540976145 | 0.270881103 | NA | NS | 0.56722129 |
| LINC02551 | 0.152411829 | 0.260900827 | 0.487051739 | NS | 0.583524544 |
| NTM-AS1 | 0.630320316 | 0.187053357 | 0.416478319 | NS | 0.728034492 |
| OPCML-IT1 | 0.760745098 | 0.10111784 | NA | NS | 0.995172214 |
| LINC02743 | 0.486583076 | 0.434250871 | NA | NS | 0.362259301 |
| LINC02730 | 0.821169439 | 0.048061397 | NA | Up | 1.318203614 |
| LINC02731 | 0.325273428 | 0.078375105 | 0.28303577 | NS | 1.105821866 |
| B3GAT1-DT | 0.406576902 | 0.056689589 | 0.250023562 | NS | 1.246496691 |
| LINC02706 | 0.485831594 | 0.375642706 | NA | NS | 0.42522504 |
| LINC02714 | 0.597225543 | 0.136424322 | NA | NS | 0.865108195 |
| LINC02717 | 0.336482495 | 0.474118851 | NA | NS | 0.324112777 |
| LINC02684 | 0.671873218 | 0.212371342 | NA | NS | 0.672904089 |
| SLC6A12-AS1 | 0.334641196 | 0.391670173 | NA | NS | 0.4070795 |
| NINJ2-AS1 | 0.016781075 | 0.808740505 | 0.890068227 | NS | 0.092190805 |
| LINC02455 | 0.888398181 | 0.08930041 | NA | NS | 1.049146547 |
| LINC00942 | 0.71042289 | 0.083877256 | NA | NS | 1.076355785 |
| ITFG2-AS1 | 0.091884153 | 0.502812943 | 0.684042859 | NS | 0.298593552 |
| LINC02371 | 0.155782015 | 0.389813552 | 0.596958206 | NS | 0.409143067 |
| TSPAN9-IT1 | 0.284678206 | 0.579125938 | NA | NS | 0.237226983 |
| LINC02827 | 0.776121805 | 0.186774175 | NA | NS | 0.728683173 |
| LINC02417 | 0.876766697 | 0.131743042 | NA | NS | 0.880272312 |
| THCAT155 | 0.458522591 | 0.326132795 | NA | NS | 0.486605528 |
| PARP11-AS1 | 0.036003316 | 0.607725343 | 0.759474289 | NS | 0.216292652 |
| CCND2-AS1 | 0.403642385 | 0.11883343 | 0.33946903 | NS | 0.925061367 |
| GAU1 | 0.092406503 | 0.535563698 | 0.707637444 | NS | 0.271188868 |
| LINC02443 | 0.491102233 | 0.308201849 | NA | NS | 0.511164761 |
| CD27-AS1 | -0.022685893 | 0.767892978 | 0.864743324 | NS | 0.114699304 |
| GAPDH-DT | 0.02591541 | 0.916824883 | NA | NS | 0.037713608 |
| MIR200CHG | 0.565448601 | 0.18325206 | NA | NS | 0.736951136 |
| C1RL-AS1 | 0.166282547 | 0.224514819 | 0.452878298 | NS | 0.648754988 |
| FAM66C | 0.1357659 | 0.163597137 | 0.391341302 | NS | 0.786224302 |
| LINC02449 | 0.470976129 | 0.006163444 | 0.105104392 | Up | 2.210176534 |
| LINC00937 | 0.060550824 | 0.720738377 | 0.835096644 | NS | 0.142222352 |
| A2ML1-AS1 | 0.711952559 | 0.193114379 | NA | NS | 0.714185388 |
| A2ML1-AS2 | 0.70191364 | 0.336897299 | NA | NS | 0.472502471 |
| LINC00612 | 0.025287692 | 0.864418006 | 0.923382803 | NS | 0.063276195 |
| A2M-AS1 | -0.006875795 | 0.965115357 | 0.980465179 | NS | 0.015420774 |
| LINC00987 | 0.144149147 | 0.39851914 | 0.603470335 | NS | 0.399550815 |
| LINC02367 | 0.536633978 | 0.017379436 | 0.154692513 | Up | 1.75996433 |
| LINC02390 | 0.03862087 | 0.774070027 | 0.868209356 | NS | 0.111219749 |
| LINC02470 | -0.276797902 | 0.425246966 | 0.623663211 | NS | 0.371358776 |
| CLEC12A-AS1 | 0.410826554 | 0.183326649 | 0.41356845 | NS | 0.736774401 |
| LINC02617 | 0.565904718 | 0.070744216 | NA | NS | 1.150309063 |
| LINC02598 | 0.215068147 | 0.206750079 | 0.436666684 | NS | 0.684554316 |
| KLRK1-AS1 | 0.030647846 | 0.76495758 | 0.863305499 | NS | 0.116362648 |
| LINC02446 | -0.093538925 | 0.515432696 | 0.693278651 | NS | 0.287828036 |
| LINC02366 | 0.393001664 | 0.474109285 | NA | NS | 0.32412154 |
| LINC01252 | 0.126646852 | 0.494863082 | 0.677832837 | NS | 0.305514944 |
| GPRC5D-AS1 | 0.08700917 | 0.69078021 | 0.816318285 | NS | 0.160660113 |
| LINC01559 | 0.397215487 | 0.40349016 | NA | NS | 0.394167052 |
| PLBD1-AS1 | 0.240089194 | 0.058908487 | 0.253261154 | NS | 1.229822133 |
| LINC01489 | 0.743334427 | 0.218754106 | NA | NS | 0.660043787 |
| RERG-IT1 | 0.008298634 | 0.989592129 | NA | NS | 0.004543768 |
| RERG-AS1 | -0.031135965 | 0.974049706 | NA | NS | 0.01141888 |
| LINC02378 | 0.046003724 | 0.933039821 | NA | NS | 0.030099821 |
| LINC02398 | 0.699378132 | 0.187528001 | NA | NS | 0.726933875 |
| LINC02468 | 0.355778942 | 0.564522675 | NA | NS | 0.248318609 |
| PDE3A-AS1 | 0.246530026 | 0.725124008 | NA | NS | 0.139587715 |
| ETNK1-DT | 0.808939056 | 0.084816121 | NA | NS | 1.071521592 |
| SOX5-AS1 | 0.27886688 | 0.256570707 | 0.48354035 | NS | 0.590792929 |
| LINC00477 | 1.245373264 | 0.007321881 | NA | Up | 2.13537733 |
| LINC02909 | 0.552145948 | 0.284063993 | NA | NS | 0.546583813 |
| RASSF8-AS1 | 0.555959248 | 0.148851669 | 0.374840808 | NS | 0.827246292 |
| ARNTL2-AS1 | 0.758445116 | 0.064063031 | NA | NS | 1.193392514 |
| MRPS35-DT | -0.108267932 | 0.802335412 | NA | NS | 0.095644039 |
| OVCH1-AS1 | -0.630044283 | 0.050987696 | 0.238791409 | NS | 1.29253461 |
| LINC02386 | 0.265715386 | 0.620949668 | NA | NS | 0.206943601 |
| DDX11-AS1 | 0.276332465 | 0.300004368 | 0.521534845 | NS | 0.522872422 |
| LINC02387 | 0.876684489 | 0.095027285 | NA | NS | 1.022151677 |
| DENND5B-AS1 | 0.334377274 | 0.132992963 | NA | NS | 0.876171339 |
| LINC02422 | 0.159436809 | 0.058851197 | 0.253109882 | NS | 1.230244698 |
| BICD1-AS1 | 0.457725718 | 0.397466495 | NA | NS | 0.400699475 |
| CPNE8-AS1 | 0.279996316 | 0.336920223 | NA | NS | 0.47247292 |
| LINC02406 | 0.575811013 | 0.267297467 | NA | NS | 0.573005156 |
| LINC02555 | 0.243447502 | 0.217070177 | 0.446641045 | NS | 0.66339984 |
| LINC02471 | 0.208383477 | 0.234685367 | 0.462791997 | NS | 0.629513988 |
| LRRK2-DT | 0.098021528 | 0.481956261 | 0.668506329 | NS | 0.316992373 |
| LINC02400 | 0.573032137 | 0.191078008 | 0.420452397 | NS | 0.718789294 |
| LINC02402 | 0.850266942 | 0.206437552 | NA | NS | 0.685211299 |
| LINC02451 | 0.847666105 | 0.173706072 | NA | NS | 0.760185 |
| LINC02450 | 0.452495319 | 0.336031814 | NA | NS | 0.473619604 |
| LINC02461 | -0.110354218 | 0.850793309 | NA | NS | 0.070175934 |
| DBX2-AS1 | 0.449264899 | 0.519466555 | NA | NS | 0.284442409 |
| SLC38A2-AS1 | 0.14289451 | 0.24199377 | 0.469918053 | NS | 0.616195815 |
| PCED1B-AS1 | -0.021115771 | 0.774663616 | 0.868609358 | NS | 0.110886841 |
| LINC02416 | -0.350354924 | 0.182921719 | NA | NS | 0.737734725 |
| LINC02156 | 0.291840524 | 0.090738813 | 0.301698648 | NS | 1.042206908 |
| RPAP3-DT | 0.253581266 | 0.008404178 | 0.117652418 | Up | 2.075504748 |
| LINC02354 | 0.841240091 | 0.025399353 | NA | Up | 1.595177345 |
| ADCY6-DT | 1.412388079 | 0.040791427 | NA | Up | 1.389431098 |
| DDN-AS1 | 0.140358093 | 0.155998489 | 0.383185333 | NS | 0.806879608 |
| TROAP-AS1 | -0.480630108 | 0.041411835 | 0.219324903 | Down | 1.382875526 |
| BCDIN3D-AS1 | 0.142766896 | 0.211538878 | 0.441234413 | NS | 0.674609804 |
| LINC02395 | 0.615422413 | 0.130936341 | 0.35320425 | NS | 0.8829398 |
| LINC02396 | 0.525451673 | 0.352895148 | NA | NS | 0.452354313 |
| AQP5-AS1 | 0.725565846 | 0.265293746 | NA | NS | 0.576272988 |
| SLC4A8-AS1 | 0.809791733 | 0.004972963 | NA | Up | 2.303384814 |
| FIGNL2-DT | 1.585283674 | 0.003349569 | NA | Up | 2.475011058 |
| LINC00592 | 0.554562329 | 0.295246258 | NA | NS | 0.529815598 |
| LINC02874 | 0.622816246 | 0.198158501 | NA | NS | 0.702987292 |
| KRT7-AS | 0.491566206 | 0.286788388 | NA | NS | 0.542438437 |
| KRT73-AS1 | 0.263211643 | 0.158103498 | 0.385419569 | NS | 0.801058522 |
| MYG1-AS1 | 0.647376793 | 0.2484786 | NA | NS | 0.604711008 |
| PCBP2-OT1 | 0.60728079 | 0.005300953 | NA | Up | 2.275646067 |
| CISTR | 0.868136271 | 0.07584522 | NA | NS | 1.120071787 |
| HOXC13-AS | 0.238612671 | 0.687975023 | NA | NS | 0.162427328 |
| HOTAIR | 0.917901526 | 0.047119886 | NA | Up | 1.326795768 |
| HOXC-AS3 | 0.639465087 | 0.26245964 | NA | NS | 0.58093747 |
| HOXC-AS2 | 0.860792738 | 0.092474809 | NA | NS | 1.033976558 |
| HOXC-AS1 | 1.063756066 | 0.134326381 | NA | NS | 0.871838686 |
| FLJ12825 | 0.287012583 | 0.416090639 | 0.617223125 | NS | 0.380812054 |
| FAM242C | -0.041817486 | 0.91586328 | NA | NS | 0.038169353 |
| LINC02381 | 0.31067042 | 0.253138062 | NA | NS | 0.59664255 |
| SCAT2 | 0.080206735 | 0.770771811 | NA | NS | 0.113074177 |
| LINC01154 | 0.193564256 | 0.715296601 | NA | NS | 0.145513839 |
| CD63-AS1 | -0.119226275 | 0.19338333 | 0.422666547 | NS | 0.713580965 |
| MYL6B-AS1 | 0.183661028 | 0.275234159 | NA | NS | 0.560297667 |
| CNPY2-AS1 | 0.101021266 | 0.321888068 | 0.540341606 | NS | 0.492295122 |
| LRP1-AS | 0.02412793 | 0.939793353 | NA | NS | 0.026967631 |
| R3HDM2-DT | 0.614174731 | 0.146718606 | NA | NS | 0.833514809 |
| AGAP2-AS1 | 0.382580193 | 0.324115653 | NA | NS | 0.489299994 |
| GIHCG | -0.178635188 | 0.256979811 | 0.484042931 | NS | 0.590100995 |
| LINC02388 | 0.610847771 | 0.194634869 | 0.424088309 | NS | 0.710779353 |
| LRIG3-DT | 0.861458382 | 0.123769913 | NA | NS | 0.907384915 |
| LINC02448 | 0.508484643 | 0.369837052 | NA | NS | 0.431989582 |
| LINC01465 | 0.09874872 | 0.140254718 | 0.364599659 | NS | 0.85308252 |
| MIRLET7IHG | 0.116041628 | 0.12929639 | 0.351262861 | NS | 0.888413602 |
| RXYLT1-AS1 | 0.752771475 | 0.171821484 | NA | NS | 0.764922536 |
| RASSF3-DT | 0.351221297 | 0.083346832 | NA | NS | 1.079110905 |
| LINC02389 | 0.32612301 | 0.431616676 | NA | NS | 0.364901784 |
| LINC02231 | 1.012965084 | 0.063686779 | NA | NS | 1.195950714 |
| LINC02454 | 0.603555809 | 0.166424813 | 0.39443869 | NS | 0.778781924 |
| HMGA2-AS1 | 0.199218764 | 0.208472237 | 0.438342929 | NS | 0.680951773 |
| LINC02425 | 0.488207793 | 0.506147468 | NA | NS | 0.295722931 |
| LLPH-DT | 0.451188218 | 0.003215586 | 0.084356647 | Up | 2.492739858 |
| LINC02420 | 0.977554983 | 0.091731838 | NA | NS | 1.037479906 |
| LINC02408 | 0.429665174 | 0.381557664 | NA | NS | 0.418439818 |
| LINC02442 | 0.829098401 | 0.105191986 | NA | NS | 0.978017343 |
| LINC02421 | 0.340199746 | 0.013569636 | 0.139924271 | Up | 1.8674318 |
| LINC01479 | 0.008107551 | 0.97380873 | NA | NS | 0.011526337 |
| IFNG-AS1 | -0.040208203 | 0.675097579 | 0.806240246 | NS | 0.170633449 |
| LINC02384 | 0.122944945 | 0.388879351 | 0.596604696 | NS | 0.410185117 |
| NUP107-DT | 0.127715821 | 0.192662225 | 0.421709861 | NS | 0.715203429 |
| LINC02373 | 0.392485349 | 0.331568848 | NA | NS | 0.47942628 |
| PRANCR | 0.079899822 | 0.213864011 | 0.443234232 | NS | 0.669862293 |
| LINC02821 | 0.634486738 | 0.080562297 | NA | NS | 1.093868159 |
| TRHDE-AS1 | 0.014181815 | 0.962165206 | 0.97896114 | NS | 0.016750352 |
| LINC02444 | 0.430373012 | 0.42370437 | NA | NS | 0.372937056 |
| LINC02445 | 1.03047948 | 0.111433131 | NA | NS | 0.952985669 |
| LINC02882 | 0.571761635 | 0.160556658 | 0.388327022 | NS | 0.794371681 |
| LINC02394 | 0.834910242 | 0.118239736 | NA | NS | 0.92723655 |
| CAPS2-AS1 | 0.532625663 | 0.205032448 | NA | NS | 0.688177402 |
| PHLDA1-DT | 0.220528648 | 0.066280047 | 0.265514368 | NS | 1.178617191 |
| LNCOG | 0.652600515 | 0.171390548 | NA | NS | 0.766013132 |
| LINC02464 | 0.633978692 | 0.208492219 | NA | NS | 0.680910148 |
| LINC02424 | 0.288431939 | 0.639379684 | NA | NS | 0.194241167 |
| PPP1R12A-AS2 | 0.226640492 | 0.048370095 | 0.233313377 | Up | 1.315423061 |
| PPP1R12A-AS1 | 0.176189881 | 0.233034916 | 0.461411926 | NS | 0.632579003 |
| LINC01490 | 0.4689451 | 0.282060433 | NA | NS | 0.549657832 |
| PPFIA2-AS1 | 0.01184949 | 0.982836686 | NA | NS | 0.007518641 |
| LINC02426 | 0.175358649 | 0.774552675 | NA | NS | 0.110949042 |
| LINC02820 | 0.505667515 | 0.377249658 | NA | NS | 0.423371146 |
| LINC02258 | 0.872723391 | 0.249561802 | NA | NS | 0.602821888 |
| LINC02458 | 0.04045018 | 0.846795555 | 0.913820481 | NS | 0.07222143 |
| POC1B-AS1 | 0.133057827 | 0.083430506 | 0.290969351 | NS | 1.078675124 |
| ATP2B1-AS1 | -0.095288276 | 0.317864327 | 0.537121802 | NS | 0.497758209 |
| LINC02399 | 0.430887036 | 0.129718314 | NA | NS | 0.886998703 |
| LINC02392 | 0.751793725 | 0.17933418 | NA | NS | 0.746336929 |
| LINC02822 | 0.36344872 | 0.411661981 | 0.614131594 | NS | 0.38545924 |
| LINC00615 | 0.392223263 | 0.491060789 | NA | NS | 0.308864743 |
| LINC02823 | 0.760313333 | 0.129812385 | NA | NS | 0.886683872 |
| LINC02404 | 0.25619848 | 0.253704473 | NA | NS | 0.595671875 |
| LINC01619 | 0.122329448 | 0.134423196 | 0.357972097 | NS | 0.871525784 |
| BTG1-DT | 0.166962411 | 0.049202611 | 0.234974405 | Up | 1.308011848 |
| LINC02391 | 0.087813236 | 0.531991766 | 0.705114614 | NS | 0.27409509 |
| CLLU1-AS1 | 0.021815735 | 0.968022622 | NA | NS | 0.014114493 |
| CLLU1 | 0.222844422 | 0.55313369 | NA | NS | 0.257169889 |
| LINC02397 | 0.204709856 | 0.123294202 | 0.344057977 | NS | 0.909057346 |
| LINC02413 | 0.162885317 | 0.578085244 | NA | NS | 0.238008116 |
| LINC02412 | 0.13133696 | 0.800808127 | NA | NS | 0.096471528 |
| SOCS2-AS1 | 0.147436626 | 0.355366661 | 0.568576726 | NS | 0.449323319 |
| CEP83-DT | 0.58665773 | 0.008831212 | NA | Up | 2.053979696 |
| SNRPF-DT | 0.617258551 | 0.40923839 | NA | NS | 0.388023633 |
| LINC02410 | 0.170352225 | 0.874787992 | NA | NS | 0.058097187 |
| LINC02452 | 0.650445856 | 0.162758907 | NA | NS | 0.788455235 |
| LINC02409 | 1.328767742 | 0.05528346 | NA | NS | 1.257404783 |
| RMST | 0.308891501 | 0.443679695 | 0.637917821 | NS | 0.352930447 |
| LINC02453 | 0.065244457 | 0.704570425 | 0.824910377 | NS | 0.152075591 |
| TMPO-AS1 | -0.019679888 | 0.848075232 | 0.914522034 | NS | 0.07156562 |
| UHRF1BP1L-DT | 0.135232297 | 0.499189207 | NA | NS | 0.301734813 |
| HELLPAR | 0.335905441 | 0.186436363 | 0.415913265 | NS | 0.729469379 |
| LINC02456 | 1.421271354 | 0.019722505 | NA | Up | 1.705037931 |
| LINC00485 | 0.473641721 | 0.337707906 | NA | NS | 0.471458772 |
| LINC02401 | 0.304576337 | 0.543258578 | 0.713032722 | NS | 0.264993407 |
| KCCAT198 | -0.0644463 | 0.782861405 | NA | NS | 0.106315117 |
| CASC18 | 0.692298644 | 0.16308857 | NA | NS | 0.787576475 |
| TMEM263-DT | -0.067937503 | 0.687298965 | 0.814178911 | NS | 0.16285431 |
| LINC01498 | 0.621588987 | 0.157707866 | NA | NS | 0.802146645 |
| USP30-AS1 | -0.084851274 | 0.49010714 | 0.674626381 | NS | 0.30970897 |
| LINC01486 | 1.497256264 | 0.00893188 | NA | Up | 2.049057125 |
| FAM222A-AS1 | 0.807234465 | 0.145566318 | NA | NS | 0.836939102 |
| LINC01405 | 0.609692159 | 0.232975028 | NA | NS | 0.632690628 |
| LINC02356 | 0.241930093 | 0.332063692 | NA | NS | 0.478778608 |
| ATXN2-AS | 0.125084142 | 0.62980658 | NA | NS | 0.200792806 |
| MAPKAPK5-AS1 | -0.157662702 | 0.083552769 | 0.29111905 | NS | 1.078039153 |
| LHX5-AS1 | 0.81077104 | 0.095191206 | NA | NS | 1.021403172 |
| LINC01234 | 0.491691631 | 0.220507218 | 0.449602156 | NS | 0.656577189 |
| LINC02459 | 0.313874125 | 0.612535999 | NA | NS | 0.212868382 |
| TBX5-AS1 | 0.536071781 | 0.246185425 | NA | NS | 0.608737662 |
| TBX3-AS1 | 0.672237817 | 0.169025522 | NA | NS | 0.772047715 |
| LINC02463 | 0.510250892 | 0.468437269 | NA | NS | 0.32934856 |
| LINC02457 | 0.380415848 | 0.405159196 | NA | NS | 0.3923743 |
| LINC00173 | 0.439061338 | 0.002832581 | 0.079855432 | Up | 2.547817589 |
| TESC-AS1 | 0.078300281 | 0.735705549 | NA | NS | 0.133295968 |
| LINC02460 | -0.104514316 | 0.864138501 | NA | NS | 0.063416645 |
| LINC02423 | 0.437873666 | 0.480010306 | NA | NS | 0.318749438 |
| LINC02440 | 1.129735843 | 0.050975727 | NA | NS | 1.292636572 |
| LINC02439 | 0.585792571 | 0.281990023 | NA | NS | 0.549766257 |
| LINC00934 | 0.452282946 | 0.412363168 | NA | NS | 0.384720133 |
| PXN-AS1 | 0.161952731 | 0.135669814 | 0.3596977 | NS | 0.867516769 |
| NRAV | -0.016640449 | 0.85309015 | 0.917099521 | NS | 0.069005073 |
| CABP1-DT | 0.726544996 | 0.137159851 | NA | NS | 0.862772996 |
| HNF1A-AS1 | 0.659106284 | 0.160325622 | 0.388015316 | NS | 0.794997067 |
| KDM2B-DT | -0.084988292 | 0.543630479 | 0.713193861 | NS | 0.264696202 |
| LINC01089 | 0.177735845 | 0.040500052 | 0.217017358 | Up | 1.39254442 |
| CLIP1-AS1 | 0.132503996 | 0.334754747 | 0.55127581 | NS | 0.475273256 |
| PITPNM2-AS1 | 0.177962998 | 0.407876636 | 0.611112752 | NS | 0.389471171 |
| SBNO1-AS1 | 0.277551423 | 0.003279662 | 0.084967003 | Up | 2.484170847 |
| TMED2-DT | 0.133034682 | 0.16077086 | 0.388591588 | NS | 0.793792666 |
| LINC00939 | 0.415778699 | 0.381275189 | 0.59100876 | NS | 0.418761455 |
| LINC02826 | 0.365373342 | 0.403638329 | NA | NS | 0.3940076 |
| LINC02359 | 0.456019832 | 0.290740135 | 0.513652722 | NS | 0.536495013 |
| LINC02347 | 0.465323729 | 0.271058157 | NA | NS | 0.566937519 |
| LINC02824 | 0.202774935 | 0.34154587 | 0.55696544 | NS | 0.466550962 |
| LINC00943 | 0.01552436 | 0.914455223 | 0.952222667 | NS | 0.038837555 |
| LINC00944 | 0.012868517 | 0.926077511 | 0.959397324 | NS | 0.033352662 |
| LINC02372 | 0.484958861 | 0.372284149 | NA | NS | 0.429125455 |
| LINC02405 | 0.58977888 | 0.168589833 | 0.396805748 | NS | 0.77316862 |
| LINC02376 | 0.901909784 | 0.086098916 | NA | NS | 1.065002317 |
| LINC02375 | 0.550956226 | 0.365184168 | NA | NS | 0.437488059 |
| LINC02411 | 0.276366787 | 0.581952125 | NA | NS | 0.235112742 |
| LINC02393 | 0.665600364 | 0.180239333 | NA | NS | 0.744150428 |
| LINC00507 | 0.375117733 | 0.431287016 | 0.628209327 | NS | 0.365233616 |
| LINC00508 | 1.276477232 | 0.014196853 | NA | Up | 1.847807918 |
| LINC02441 | 0.382196416 | 0.486034554 | NA | NS | 0.313332854 |
| LINC02369 | 0.718839395 | 0.140888798 | NA | NS | 0.851123536 |
| LINC02368 | 0.658531465 | 0.171331296 | NA | NS | 0.766163299 |
| TMEM132D-AS1 | 0.683474819 | 0.203843162 | NA | NS | 0.690703853 |
| TMEM132D-AS2 | 0.706405977 | 0.189585671 | NA | NS | 0.72219449 |
| LINC02418 | 0.618364108 | 0.211503763 | NA | NS | 0.674681902 |
| LINC02419 | 1.303327587 | 0.043727027 | NA | Up | 1.359250051 |
| FZD10-AS1 | 0.718601905 | 0.12166668 | 0.34234916 | NS | 0.914828344 |
| ADGRD1-AS1 | 0.323497503 | 0.249762123 | NA | NS | 0.602473422 |
| LINC01257 | 0.381817905 | 0.391832883 | NA | NS | 0.406899121 |
| LINC02370 | 1.080067206 | 0.056979027 | NA | NS | 1.244284975 |
| LINC02414 | 0.61425699 | 0.139411954 | NA | NS | 0.855699986 |
| LINC02361 | -0.127817162 | 0.609980446 | NA | NS | 0.214684087 |
| ZNF84-DT | 0.403355118 | 0.480528664 | NA | NS | 0.318280701 |
| LINC00388 | 0.818419154 | 0.174735546 | NA | NS | 0.757618739 |
| LINC00387 | 0.535903676 | 0.423568895 | NA | NS | 0.37307594 |
| LINC00408 | 0.799548919 | 0.095123482 | NA | NS | 1.021712262 |
| LINC00442 | 0.708123782 | 0.176901973 | NA | NS | 0.752267323 |
| LINC00421 | 0.812749418 | 0.194593477 | NA | NS | 0.710871722 |
| LINC00350 | 0.753330189 | 0.046751734 | NA | Up | 1.330202277 |
| PSPC1-AS2 | 0.171250375 | 0.051769197 | 0.240198165 | NS | 1.285928575 |
| LINC01072 | 1.037410681 | 0.090879999 | NA | NS | 1.041531686 |
| LATS2-AS1 | 0.316574795 | 0.302600059 | NA | NS | 0.519130991 |
| LINC01046 | 0.374120533 | 0.537531255 | NA | NS | 0.269596279 |
| LINC00539 | 0.073994307 | 0.398720145 | 0.603654972 | NS | 0.399331822 |
| ZDHHC20-IT1 | 0.28219052 | 0.321402284 | NA | NS | 0.492951041 |
| LINC00424 | 0.404960915 | 0.569964299 | NA | NS | 0.244152346 |
| LINC00540 | 0.184084115 | 0.248471732 | 0.476119605 | NS | 0.604723013 |
| LINC00621 | 0.688651922 | 0.143919743 | 0.369098539 | NS | 0.841879626 |
| LINC00362 | 0.343792054 | 0.677915708 | NA | NS | 0.168824303 |
| SACS-AS1 | 0.898843507 | 0.097303959 | NA | NS | 1.011869487 |
| LINC00327 | 0.169227132 | 0.645613589 | 0.786062861 | NS | 0.190027337 |
| LINC00352 | 0.54838416 | 0.358387276 | NA | NS | 0.445647418 |
| SPATA13-AS1 | -0.06808236 | 0.414942307 | 0.616431019 | NS | 0.382012283 |
| LINC00566 | 1.205930659 | 0.081200079 | NA | NS | 1.090443547 |
| LINC01053 | 0.087418995 | 0.885527849 | NA | NS | 0.052797776 |
| LINC00463 | 1.084273965 | 0.033615159 | NA | Up | 1.473464834 |
| LINC01076 | 0.4563314 | 0.509315451 | NA | NS | 0.293013148 |
| LINC00415 | 0.810620625 | 0.309986028 | NA | NS | 0.508657881 |
| WASF3-AS1 | 0.770895937 | 0.093048544 | 0.305431813 | NS | 1.031290417 |
| USP12-AS1 | 0.420006375 | 0.001025634 | 0.052784419 | Up | 2.989007655 |
| USP12-AS2 | -0.364995586 | 0.194964199 | NA | NS | 0.710045129 |
| LINC02340 | 0.092326314 | 0.547741883 | 0.7160589 | NS | 0.261424049 |
| LINC00412 | 0.241846556 | 0.023437912 | 0.173925996 | Up | 1.630081082 |
| LINC01079 | 0.284756326 | 0.765258579 | NA | NS | 0.116191793 |
| PLUT | 0.881469059 | 0.120650998 | NA | NS | 0.91846908 |
| LINC00543 | 1.673928629 | 0.005414523 | NA | Up | 2.266439796 |
| PAN3-AS1 | 0.100244808 | 0.284339441 | 0.507200085 | NS | 0.546162894 |
| MTUS2-AS2 | 0.583499174 | 0.242472836 | NA | NS | 0.615336907 |
| MTUS2-AS1 | 0.477915763 | 0.303493678 | NA | NS | 0.517850351 |
| LINC00297 | 0.814297579 | 0.1976819 | NA | NS | 0.704033094 |
| LINC00572 | 0.64826402 | 0.304846506 | NA | NS | 0.515918778 |
| LINC00544 | 0.24445438 | 0.023281247 | 0.173353487 | Up | 1.632993755 |
| LINC00365 | 0.57352565 | 0.208998686 | NA | NS | 0.679856445 |
| LINC00384 | 0.370076935 | 0.136639871 | 0.361144434 | NS | 0.864422558 |
| LINC00427 | 0.21301397 | 0.184547603 | 0.414734144 | NS | 0.733891591 |
| LINC00426 | 0.024009341 | 0.709123209 | 0.827744125 | NS | 0.1492783 |
| LINC01058 | 0.088194361 | 0.510627985 | 0.689569085 | NS | 0.291895387 |
| LINC00398 | 0.427239871 | 0.047762485 | NA | Up | 1.320913084 |
| LINC00545 | 0.494046585 | 0.16973699 | NA | NS | 0.770223503 |
| TEX26-AS1 | 0.395432686 | 0.257910368 | 0.484730205 | NS | 0.588531198 |
| FRY-AS1 | 0.199675711 | 0.106588874 | 0.32311031 | NS | 0.972288126 |
| N4BP2L2-IT2 | 0.094592645 | 0.304903313 | 0.526137408 | NS | 0.515837857 |
| LINC00423 | 0.841314821 | 0.135336202 | NA | NS | 0.868586017 |
| STARD13-IT1 | -0.152029924 | 0.801926174 | NA | NS | 0.095865612 |
| STARD13-AS | 0.444148442 | 0.072683318 | 0.274345075 | NS | 1.138565252 |
| LINC02344 | 1.236936533 | 0.009621281 | NA | Up | 2.016767104 |
| LINC02343 | 0.375091872 | 0.446204628 | NA | NS | 0.350465929 |
| LINC00457 | 0.343749862 | 0.500414948 | NA | NS | 0.300669726 |
| LINC00445 | 0.717563109 | 0.197787515 | NA | NS | 0.703801126 |
| SPART-AS1 | 0.425822988 | 0.204754777 | 0.434924698 | NS | 0.688765957 |
| SMAD9-IT1 | 1.092917354 | 0.008473369 | NA | Up | 2.071943884 |
| LINC01048 | 0.925246188 | 0.139863517 | NA | NS | 0.854295554 |
| LINC00547 | 0.562158918 | 0.324346055 | NA | NS | 0.48899138 |
| LINC02334 | 0.551210605 | 0.058184151 | 0.252197542 | NS | 1.235195302 |
| LINC00571 | 0.156112187 | 0.415418195 | NA | NS | 0.381514485 |
| LINC00437 | 0.237409023 | 0.68503496 | NA | NS | 0.164287264 |
| LINC00366 | 0.481181623 | 0.475635342 | NA | NS | 0.322725882 |
| LINC00598 | 0.27781098 | 0.119001407 | 0.339550081 | NS | 0.924447905 |
| LINC00332 | 0.388779638 | 0.102801432 | NA | NS | 0.988000837 |
| VWA8-AS1 | 0.665732244 | 0.014290011 | NA | Up | 1.844967437 |
| LINC02341 | 0.367779134 | 0.194915348 | NA | NS | 0.710153961 |
| LINC01050 | 0.547388035 | 0.255901119 | NA | NS | 0.591927816 |
| LINC00400 | 0.984413825 | 0.096946917 | NA | NS | 1.013465999 |
| ENOX1-AS2 | 0.339156405 | 0.663415206 | NA | NS | 0.178214578 |
| ENOX1-AS1 | 0.471686727 | 0.372883753 | NA | NS | 0.428426539 |
| NRAD1 | 0.381798264 | 0.359488185 | NA | NS | 0.444315379 |
| LINC00390 | 0.409242197 | 0.054819932 | 0.247094287 | NS | 1.261061511 |
| SMIM2-AS1 | 0.365828018 | 0.277438067 | 0.501886778 | NS | 0.556833951 |
| TUSC8 | 0.756080179 | 0.028194239 | NA | Up | 1.549839629 |
| TSC22D1-AS1 | 0.201519449 | 0.055856609 | 0.249087638 | NS | 1.252925436 |
| LINC00407 | -0.037096462 | 0.955210655 | NA | NS | 0.019900842 |
| LINC00330 | 0.478658563 | 0.351351175 | NA | NS | 0.45425859 |
| TPT1-AS1 | 0.108853682 | 0.229274322 | 0.457791831 | NS | 0.639644581 |
| SLC25A30-AS1 | 0.251696538 | 0.31126784 | NA | NS | 0.506865749 |
| LINC01055 | 0.792391954 | 0.180198578 | NA | NS | 0.744248639 |
| CPB2-AS1 | 0.126383241 | 0.225525546 | 0.454064708 | NS | 0.646804257 |
| LINC00563 | 0.28184003 | 0.34637498 | NA | NS | 0.460453486 |
| LINC01198 | 0.410048914 | 0.41709907 | NA | NS | 0.379760778 |
| HTR2A-AS1 | 0.917889689 | 0.109128043 | NA | NS | 0.962063634 |
| LINC00562 | 0.249270654 | 0.243176846 | 0.470891699 | NS | 0.614077779 |
| SUCLA2-AS1 | 0.181029175 | 0.616333515 | NA | NS | 0.210184215 |
| MED4-AS1 | 0.10214805 | 0.432584206 | 0.629218386 | NS | 0.363929341 |
| RB1-DT | 0.121284018 | 0.411657666 | 0.614131594 | NS | 0.385463793 |
| LINC01077 | -0.101389995 | 0.791330764 | NA | NS | 0.10164195 |
| LINC00462 | 0.408030954 | 0.254893635 | NA | NS | 0.593641009 |
| DLEU2 | 0.016396021 | 0.86205069 | 0.922196766 | NS | 0.064467196 |
| DLEU1 | 0.133853258 | 0.089507333 | 0.299870767 | NS | 1.048141383 |
| RNASEH2B-AS1 | 0.101519511 | 0.397821326 | 0.602951787 | NS | 0.400311939 |
| INTS6-AS1 | 0.069384966 | 0.382436293 | 0.591830386 | NS | 0.4174409 |
| LINC02333 | 0.649825988 | 0.299168938 | NA | NS | 0.524083501 |
| LINC00345 | 0.398412673 | 0.456211814 | NA | NS | 0.340833473 |
| SUGT1-DT | -0.058986798 | 0.601726252 | 0.755522732 | NS | 0.220601041 |
| LINC01065 | 0.390372748 | 0.373461629 | NA | NS | 0.427754012 |
| LINC00558 | 0.530972965 | 0.318031743 | NA | NS | 0.497529531 |
| LINC00458 | 0.320916791 | 0.532345102 | NA | NS | 0.273806737 |
| LINC02335 | -0.18453474 | 0.777018866 | NA | NS | 0.109568437 |
| LINC02338 | 0.320546852 | 0.538665151 | NA | NS | 0.26868112 |
| LINC00374 | 0.232230733 | 0.677231529 | NA | NS | 0.169262831 |
| DIAPH3-AS1 | 0.243634914 | 0.609801204 | NA | NS | 0.214811722 |
| DIAPH3-AS2 | 0.490165394 | 0.321524257 | NA | NS | 0.492786257 |
| LINC00434 | 0.67745542 | 0.290861125 | NA | NS | 0.53631432 |
| LINC00378 | -0.152958307 | 0.725820853 | NA | NS | 0.139170558 |
| LINC01442 | 0.135508671 | 0.779218345 | NA | NS | 0.108340831 |
| LINC00358 | 0.535719481 | 0.409599751 | NA | NS | 0.387640316 |
| LINC01075 | 0.31770841 | 0.581235125 | NA | NS | 0.235648149 |
| LINC00448 | -0.054130106 | 0.92393195 | NA | NS | 0.034360015 |
| LINC00376 | -0.142264021 | 0.826728071 | NA | NS | 0.082637316 |
| LINC00395 | 0.802884529 | 0.294465001 | NA | NS | 0.530966317 |
| LINC00355 | 0.673945775 | 0.17285836 | NA | NS | 0.762309613 |
| LINC01052 | 0.153807796 | 0.789083361 | NA | NS | 0.102877114 |
| PCDH9-AS2 | 0.486594076 | 0.125697079 | NA | NS | 0.900674815 |
| PCDH9-AS3 | 0.091423444 | 0.789752311 | NA | NS | 0.102509095 |
| PCDH9-AS4 | 0.484525245 | 0.181837038 | NA | NS | 0.740317652 |
| LINC00364 | 0.737160001 | 0.127247195 | NA | NS | 0.895351782 |
| LINC00550 | 0.346174749 | 0.550179174 | NA | NS | 0.259495853 |
| LINC02342 | 0.173667107 | 0.800465205 | NA | NS | 0.096657541 |
| LINC00383 | 0.732506281 | 0.159417906 | NA | NS | 0.797462899 |
| ATXN8OS | 0.2409929 | 0.652077946 | NA | NS | 0.185700488 |
| LINC00348 | 0.311351484 | 0.596383305 | NA | NS | 0.224474523 |
| LINC00393 | 0.243974878 | 0.548731364 | 0.716765283 | NS | 0.260640216 |
| LINC00392 | 0.871463392 | 0.120234588 | NA | NS | 0.919970581 |
| LINC00402 | 0.068233122 | 0.570717385 | 0.732737389 | NS | 0.243578898 |
| LINC00381 | 0.256808205 | 0.07547048 | 0.278643389 | NS | 1.122222885 |
| LINC00347 | 0.117003571 | 0.837773046 | NA | NS | 0.076873616 |
| LMO7-AS1 | 0.189351621 | 0.072644887 | 0.274345075 | NS | 1.138794949 |
| LMO7DN | 0.501999479 | 0.291223762 | NA | NS | 0.535773192 |
| LMO7DN-IT1 | 0.077072553 | 0.917812388 | NA | NS | 0.037246085 |
| LINC00561 | 0.598280797 | 0.293619515 | NA | NS | 0.532215083 |
| LINC01034 | 0.095675341 | 0.907772507 | NA | NS | 0.042022975 |
| MYCBP2-AS1 | 0.146888804 | 0.120670315 | 0.341313216 | NS | 0.918399555 |
| MYCBP2-AS2 | 0.192149755 | 0.077116716 | 0.281454506 | NS | 1.112851474 |
| SCEL-AS1 | 0.268681849 | 0.789370906 | NA | NS | 0.102718885 |
| EDNRB-AS1 | 0.298367622 | 0.036890122 | 0.20933042 | Up | 1.433089909 |
| OBI1-AS1 | 0.405349294 | 0.131690317 | 0.354444963 | NS | 0.880446158 |
| LINC01069 | 0.004466023 | 0.992760373 | NA | NS | 0.003155567 |
| LINC00446 | 0.255292155 | 0.672175048 | NA | NS | 0.172517613 |
| LINC00331 | 0.656393354 | 0.199812636 | NA | NS | 0.699377051 |
| RBM26-AS1 | 0.016800524 | 0.880385255 | 0.932334579 | NS | 0.05532724 |
| NDFIP2-AS1 | 0.409131039 | 0.707488058 | NA | NS | 0.150280886 |
| LINC01068 | 0.430466838 | 0.343419595 | NA | NS | 0.464174928 |
| LINC01038 | 0.375869436 | 0.5422944 | NA | NS | 0.26576488 |
| LINC00382 | 0.467927934 | 0.45810673 | NA | NS | 0.339033328 |
| LINC01080 | 0.500219534 | 0.296560343 | NA | NS | 0.527886924 |
| LINC00377 | 0.178060901 | 0.603318656 | NA | NS | 0.219453245 |
| LINC00564 | 0.474183207 | 0.494443674 | NA | NS | 0.305883176 |
| LINC00333 | 0.578553617 | 0.357242232 | NA | NS | 0.447037205 |
| LINC00375 | 0.202043602 | 0.780570602 | NA | NS | 0.107587809 |
| LINC00351 | 0.530450221 | 0.38337231 | NA | NS | 0.416379258 |
| LINC00430 | 0.689903536 | 0.287159132 | NA | NS | 0.541877368 |
| MIR4500HG | 0.55533588 | 0.226626263 | 0.455213225 | NS | 0.644689763 |
| LINC00397 | 1.06802461 | 0.066718719 | NA | NS | 1.175752301 |
| LINC00373 | 0.321032922 | 0.391797395 | NA | NS | 0.406938456 |
| LINC00433 | 0.754369995 | 0.122386644 | NA | NS | 0.912265976 |
| LINC00560 | 0.357938878 | 0.517408572 | NA | NS | 0.28616638 |
| LINC00440 | 0.511501787 | 0.462880219 | NA | NS | 0.334531378 |
| LINC01047 | 0.589114931 | 0.426714984 | NA | NS | 0.369862106 |
| LINC02336 | 0.659699999 | 0.251028107 | NA | NS | 0.600277649 |
| LINC01040 | 0.926941243 | 0.134937454 | NA | NS | 0.869867487 |
| LINC00353 | 0.679782292 | 0.295155071 | NA | NS | 0.529949752 |
| LINC00559 | 0.524686212 | 0.276648879 | NA | NS | 0.558071085 |
| LINC01049 | -0.389889002 | 0.554935401 | NA | NS | 0.25575757 |
| LINC00410 | 0.64823949 | 0.268423327 | NA | NS | 0.571179744 |
| LINC00380 | 0.513006189 | 0.441890338 | NA | NS | 0.354685494 |
| LINC00379 | 0.455253286 | 0.426138762 | NA | NS | 0.37044896 |
| MIR17HG | 9.19E-04 | 0.992197539 | 0.995609559 | NS | 0.003401854 |
| GPC5-AS2 | 0.177420151 | 0.781120189 | NA | NS | 0.107282137 |
| GPC5-IT1 | 0.197403531 | 0.779574968 | NA | NS | 0.108142114 |
| LINC00363 | 0.791732208 | 0.294878448 | NA | NS | 0.530356969 |
| GPC6-AS2 | -0.361446558 | 0.541146379 | NA | NS | 0.266685243 |
| GPC6-AS1 | 0.326693883 | 0.561186147 | NA | NS | 0.250893058 |
| LINC00391 | 0.622216595 | 0.215181196 | NA | NS | 0.667195683 |
| SOX21-AS1 | 1.042756972 | 0.012927897 | NA | Up | 1.888472116 |
| LINC00557 | 0.616683704 | 0.597407758 | NA | NS | 0.223729142 |
| CLDN10-AS1 | 0.805179678 | 0.118654047 | NA | NS | 0.925717443 |
| DNAJC3-DT | 0.227753552 | 0.068983134 | 0.269048319 | NS | 1.16125708 |
| LINC00456 | 0.539724888 | 0.273839969 | NA | NS | 0.562503164 |
| STK24-AS1 | -0.027286232 | 0.813293282 | 0.893206865 | NS | 0.089752815 |
| DOCK9-AS1 | 0.076048932 | 0.571039196 | 0.733068367 | NS | 0.243334081 |
| DOCK9-DT | 0.075997863 | 0.572466997 | 0.734073901 | NS | 0.242249546 |
| UBAC2-AS1 | 0.119230842 | 0.271450202 | 0.496664738 | NS | 0.566309831 |
| LINC01232 | 0.251918417 | 0.001975754 | 0.069745459 | Up | 2.704267228 |
| LINC00449 | 0.499704551 | 0.094861133 | NA | NS | 1.022911695 |
| LINC01039 | 0.693491023 | 0.207486943 | NA | NS | 0.683009227 |
| CLYBL-AS2 | 0.608636334 | 0.023713243 | NA | Up | 1.625009041 |
| CLYBL-AS1 | 0.163057941 | 0.64049993 | NA | NS | 0.193480913 |
| LINC00554 | 1.010899714 | 0.048474106 | NA | Up | 1.314490188 |
| PCCA-DT | 0.046887205 | 0.822060084 | NA | NS | 0.085096439 |
| PCCA-AS1 | 0.196091458 | 0.168346 | 0.396575496 | NS | 0.773797199 |
| NALCN-AS1 | 0.835287764 | 0.122128611 | NA | NS | 0.913182582 |
| FGF14-IT1 | 0.776622741 | 0.037346805 | NA | Up | 1.427746541 |
| FGF14-AS1 | 0.104393701 | 0.809070406 | NA | NS | 0.092013684 |
| FGF14-AS2 | 0.114147804 | 0.600922801 | NA | NS | 0.221181317 |
| LINC00555 | 1.068518907 | 0.026001028 | NA | Up | 1.585009475 |
| LINC01309 | 0.243117021 | 0.703454371 | NA | NS | 0.152764068 |
| DAOA-AS1 | 0.229524182 | 0.715067713 | NA | NS | 0.145652831 |
| LINC00343 | 0.366742911 | 0.399544254 | 0.604622869 | NS | 0.398435111 |
| LINC00460 | 0.616806235 | 0.238354732 | NA | NS | 0.622776221 |
| LINC00551 | 0.427338397 | 0.124477305 | 0.346117593 | NS | 0.904909822 |
| LINC00443 | 0.358626311 | 0.529080365 | NA | NS | 0.276478355 |
| NALF1-IT1 | 0.470015615 | 0.523083524 | NA | NS | 0.281428959 |
| MYO16-AS2 | 0.095383237 | 0.830256976 | NA | NS | 0.080787467 |
| MYO16-AS1 | 0.015442351 | 0.951009256 | NA | NS | 0.021815256 |
| LINC00370 | 0.374048902 | 0.440746858 | NA | NS | 0.355810775 |
| LINC01067 | 0.648211893 | 0.24304619 | NA | NS | 0.614311183 |
| LINC00399 | 0.881210453 | 0.004565901 | NA | Up | 2.340473541 |
| LINC00676 | 0.654618485 | 0.049379223 | 0.235159694 | Up | 1.306455747 |
| COL4A2-AS2 | 0.546794824 | 0.255310402 | NA | NS | 0.592931491 |
| COL4A2-AS1 | 0.679591994 | 0.320909575 | NA | NS | 0.493617325 |
| LINC00567 | 0.733242857 | 0.092443161 | NA | NS | 1.034125214 |
| PRECSIT | 0.245152755 | 0.363719759 | 0.575906382 | NS | 0.439233106 |
| ANKRD10-IT1 | 0.033428611 | 0.683523369 | 0.811595406 | NS | 0.165246633 |
| LINC00368 | 0.872726881 | 0.174668496 | NA | NS | 0.75778542 |
| ARHGEF7-IT1 | 0.827903456 | 0.153394844 | NA | NS | 0.814189237 |
| LINC00354 | 0.597923279 | 0.189736809 | NA | NS | 0.721848407 |
| SOX1-OT | 0.525241161 | 0.264911737 | NA | NS | 0.5768988 |
| LINC00404 | 0.985549821 | 0.071247295 | NA | NS | 1.147231617 |
| LINC01043 | 0.543904671 | 0.437931951 | NA | NS | 0.358593368 |
| ATP11AUN | 0.327010318 | 0.372654814 | NA | NS | 0.428693265 |
| ATP11A-AS1 | 0.058456254 | 0.770708761 | 0.86621231 | NS | 0.113109704 |
| MCF2L-AS1 | 0.384164123 | 0.217163514 | NA | NS | 0.66321314 |
| F10-AS1 | 0.501483031 | 0.383191871 | NA | NS | 0.416583712 |
| GRTP1-AS1 | -0.254515981 | 0.612938964 | NA | NS | 0.21258277 |
| DCUN1D2-AS | -0.049856642 | 0.687436874 | 0.814300159 | NS | 0.162767176 |
| GAS6-AS1 | 0.105755774 | 0.518741893 | 0.695808062 | NS | 0.285048677 |
| GAS6-DT | 0.892301937 | 0.086141792 | NA | NS | 1.0647861 |
| LINC00454 | 0.844257185 | 0.153045282 | NA | NS | 0.815180054 |
| LINC00452 | 0.559323051 | 0.078361264 | NA | NS | 1.105898567 |
| LINC00565 | -0.020821101 | 0.886009968 | 0.935826321 | NS | 0.052561392 |
| LINC01054 | 0.072526507 | 0.761900146 | NA | NS | 0.118101943 |
| LINC01297 | 0.696475527 | 0.161669495 | NA | NS | 0.791371918 |
| EGILA | 0.486863943 | 0.214227371 | NA | NS | 0.669125042 |
| RNASE2CP | 0.311220014 | 0.116182497 | 0.335622042 | NS | 0.934859293 |
| LINC00641 | 0.170349408 | 0.003161399 | 0.084307443 | Up | 2.500120694 |
| LINC02332 | 0.430869284 | 0.186635558 | NA | NS | 0.72900561 |
| OXA1L-DT | 0.273002307 | 0.147086558 | 0.372664177 | NS | 0.832427014 |
| PRMT5-AS1 | -0.036926765 | 0.741130469 | 0.848286771 | NS | 0.130105332 |
| PRMT5-DT | 0.23547618 | 0.043778744 | 0.224344829 | Up | 1.358736702 |
| AJUBA-DT | 0.42988596 | 0.098346996 | NA | NS | 1.007238902 |
| ZFHX2-AS1 | 0.123810344 | 0.482323281 | 0.66853282 | NS | 0.316661775 |
| AP1G2-AS1 | 0.219788461 | 0.078926937 | 0.283720719 | NS | 1.102774754 |
| DHRS4-AS1 | 0.09525965 | 0.6313043 | NA | NS | 0.199761253 |
| LINC02286 | 0.334940436 | 0.567966491 | NA | NS | 0.245677286 |
| LINC02306 | 0.188502165 | 0.716860367 | NA | NS | 0.14456543 |
| NOVA1-DT | 0.56894893 | 0.244225293 | NA | NS | 0.61220936 |
| LINC02294 | 0.551969939 | 0.399539641 | NA | NS | 0.398440125 |
| LINC02293 | 0.57812166 | 0.220985394 | 0.449641695 | NS | 0.65563643 |
| MIR4307HG | 0.496723921 | 0.390632019 | NA | NS | 0.408232162 |
| LINC00645 | 0.29530673 | 0.589743648 | NA | NS | 0.229336728 |
| LINC02300 | 0.734389962 | 0.310892853 | NA | NS | 0.507389261 |
| FOXG1-AS1 | 0.167157838 | 0.735821675 | NA | NS | 0.133227423 |
| LINC01551 | 0.524707571 | 0.262711971 | 0.488909438 | NS | 0.580520136 |
| LINC02282 | 0.423311708 | 0.470730625 | NA | NS | 0.327227546 |
| LINC02281 | 0.259737709 | 0.760659521 | NA | NS | 0.118809695 |
| LINC02327 | 0.674636941 | 0.130217997 | NA | NS | 0.885328989 |
| LINC02326 | 0.606926981 | 0.194356531 | NA | NS | 0.711400861 |
| G2E3-AS1 | 0.606014117 | 0.003439195 | 0.085194315 | Up | 2.46354322 |
| HEATR5A-DT | 0.176631287 | 0.116023448 | 0.335331777 | NS | 0.935454233 |
| LINC02313 | 0.471557091 | 0.233220481 | NA | NS | 0.632233312 |
| ARHGAP5-AS1 | 0.118866328 | 0.686650536 | NA | NS | 0.163264237 |
| EGLN3-AS1 | 0.450512675 | 0.499916064 | NA | NS | 0.301102907 |
| BAZ1A-AS1 | 0.130988085 | 0.210349248 | 0.44030879 | NS | 0.677059036 |
| LINC00609 | 0.645533475 | 0.379639481 | NA | NS | 0.42062863 |
| PTCSC3 | 0.62711996 | 0.292673857 | NA | NS | 0.533616069 |
| NKX2-1-AS1 | 0.762645562 | 0.1771182 | NA | NS | 0.751736809 |
| SLC25A21-AS1 | 0.959492385 | 0.114603342 | NA | NS | 0.940802716 |
| LINC00517 | 0.274553029 | 0.597235095 | NA | NS | 0.22385468 |
| LINC00639 | 0.223607713 | 0.348174053 | 0.562612118 | NS | 0.458203597 |
| MIA2-AS1 | 0.373169348 | 0.038429241 | NA | Up | 1.415338189 |
| LINC02315 | -0.47671577 | 0.169960578 | NA | NS | 0.7696518 |
| LRFN5-DT | 0.558671038 | 0.261835865 | NA | NS | 0.581970865 |
| LINC02307 | 1.007966652 | 0.071641121 | NA | NS | 1.144837625 |
| LINC02277 | 0.421763716 | 0.48007803 | NA | NS | 0.318688168 |
| LINC02302 | -0.072053046 | 0.875328684 | NA | NS | 0.057828839 |
| RRAGAP1-AS1 | 0.28140535 | 0.58850606 | NA | NS | 0.230249061 |
| PRPF39-DT | 0.628149009 | 0.242161922 | NA | NS | 0.615894144 |
| LINC02303 | 0.140904992 | 0.838934492 | NA | NS | 0.07627195 |
| LINC00871 | 1.540114861 | 0.009411649 | NA | Up | 2.026334299 |
| LINC00648 | 0.551556821 | 0.254636723 | NA | NS | 0.594078964 |
| LINC01588 | 0.102807569 | 0.153578763 | 0.380233538 | NS | 0.813668835 |
| LINC00519 | 0.924956811 | 0.024124311 | NA | Up | 1.61754509 |
| LINC00640 | 0.415758355 | 0.080703722 | 0.28637692 | NS | 1.093106436 |
| LINC02310 | 0.312902935 | 0.580764258 | NA | NS | 0.236000119 |
| FRMD6-AS2 | 0.848221689 | 0.15992489 | NA | NS | 0.79608394 |
| FRMD6-AS1 | 0.214042568 | 0.238633256 | 0.466720558 | NS | 0.622269033 |
| LINC02319 | 0.497136761 | 0.237937043 | NA | NS | 0.623537939 |
| DDHD1-DT | -0.181951635 | 0.290862772 | 0.513652722 | NS | 0.536311862 |
| LINC02331 | 0.574527538 | 0.221820765 | NA | NS | 0.6539978 |
| FBXO34-AS1 | -0.027440934 | 0.849105088 | 0.915158582 | NS | 0.071038557 |
| KTN1-AS1 | 0.331337787 | 0.043727539 | 0.224236694 | Up | 1.35924496 |
| LINC00520 | 0.248921787 | 0.475627058 | 0.663630967 | NS | 0.322733447 |
| LINC02284 | 0.091375337 | 0.4023988 | 0.606636604 | NS | 0.395343323 |
| OTX2-AS1 | 0.462441349 | 0.303659023 | NA | NS | 0.51761381 |
| PSMA3-AS1 | -0.013135446 | 0.750189987 | 0.853282895 | NS | 0.124828737 |
| LINC00216 | -0.09891679 | 0.197351038 | 0.427236362 | NS | 0.704760585 |
| TOMM20L-DT | 0.134835201 | 0.232653468 | 0.461081062 | NS | 0.633290469 |
| LINC01500 | 0.56394517 | 0.082526488 | 0.289685039 | NS | 1.083406636 |
| PCNX4-DT | 0.164766052 | 0.57626233 | NA | NS | 0.239379769 |
| LINC02322 | 0.194713931 | 0.726134103 | NA | NS | 0.138983166 |
| SALRNA1 | 0.781246021 | 0.131030395 | NA | NS | 0.882627951 |
| PRKCH-AS1 | 0.024822715 | 0.730605661 | 0.840555934 | NS | 0.136316967 |
| LINC01303 | 0.229664888 | 0.034340171 | 0.20348217 | Up | 1.464197551 |
| HIF1A-AS1 | 0.373735456 | 0.126165193 | NA | NS | 0.899060444 |
| HIF1A-AS3 | 0.375901212 | 2.97E-04 | 0.028988656 | Up | 3.526577466 |
| HSPA2-AS1 | 0.125078651 | 0.183985518 | 0.414320191 | NS | 0.73521636 |
| LINC02324 | 0.072324032 | 0.778251008 | 0.871266783 | NS | 0.108880308 |
| FUT8-AS1 | 0.197434216 | 0.191856918 | 0.420934764 | NS | 0.717022535 |
| LINC02290 | 0.262963806 | 0.343555317 | 0.558604157 | NS | 0.464003326 |
| ACTN1-DT | 0.252944602 | 0.1367107 | 0.361144434 | NS | 0.864197494 |
| GALNT16-AS1 | 0.536607363 | 0.06503994 | 0.264167619 | NS | 1.18681987 |
| TTC9-DT | 0.102838642 | 0.620652504 | 0.767922494 | NS | 0.207151488 |
| LINC01269 | -0.085167761 | 0.759152657 | NA | NS | 0.119670884 |
| MAP3K9-DT | 0.315394066 | 0.21426725 | NA | NS | 0.669044204 |
| SIPA1L1-AS1 | 0.078238959 | 0.418194774 | 0.618901466 | NS | 0.378621399 |
| RBM25-AS1 | -0.028317483 | 0.816946859 | 0.895844719 | NS | 0.087806193 |
| PAPLN-AS1 | 0.100410453 | 0.68314209 | 0.811353013 | NS | 0.165488956 |
| LINC02274 | -0.201475081 | 0.685135298 | NA | NS | 0.164223657 |
| LINC01220 | 0.03901567 | 0.794721156 | 0.88134734 | NS | 0.099785226 |
| JDP2-AS1 | 0.142120305 | 0.340591395 | 0.556086414 | NS | 0.467766328 |
| TGFB3-AS1 | 0.909250694 | 0.015689199 | NA | Up | 1.804399238 |
| VASH1-DT | 0.598116904 | 0.296707998 | NA | NS | 0.527670747 |
| VASH1-AS1 | 0.041286933 | 0.844605213 | NA | NS | 0.073346242 |
| LINC01629 | 0.017146283 | 0.965129929 | NA | NS | 0.015414216 |
| LINC02288 | 0.124382394 | 0.531033692 | 0.704804549 | NS | 0.274877924 |
| LINC02289 | 0.203801776 | 0.294696979 | 0.516780261 | NS | 0.530624316 |
| DIO2-AS1 | 0.433461006 | 0.253331929 | NA | NS | 0.59631007 |
| LINC02308 | 0.967079963 | 0.112430246 | NA | NS | 0.94911684 |
| LINC01467 | 0.683543098 | 0.210147618 | NA | NS | 0.677475528 |
| LINC02311 | 0.287367697 | 0.582130304 | NA | NS | 0.234979792 |
| LINC02301 | 0.173020014 | 0.657566985 | 0.794202854 | NS | 0.18206 |
| LINC02305 | 0.625368959 | 0.442016186 | NA | NS | 0.354561827 |
| LINC02329 | 0.578057906 | 0.365847216 | NA | NS | 0.436700246 |
| LINC00911 | 0.412179171 | 0.426839035 | NA | NS | 0.369735871 |
| LINC02328 | 0.020699764 | 0.789032099 | 0.878322434 | NS | 0.102905329 |
| LINC02316 | 0.142796509 | 0.18417517 | 0.414362912 | NS | 0.73476892 |
| LINC02309 | 0.057721524 | 0.925337236 | NA | NS | 0.033699961 |
| LINC01148 | 0.628693813 | 0.314442614 | NA | NS | 0.502458602 |
| LINC02296 | 0.685237091 | 0.246410117 | NA | NS | 0.608341466 |
| LINC02330 | -0.232043234 | 0.750645295 | NA | NS | 0.124565233 |
| LINC01147 | 0.106316921 | 0.389575314 | 0.59694612 | NS | 0.40940857 |
| LINC01146 | 0.185780485 | 0.145302854 | 0.37054456 | NS | 0.837725855 |
| FOXN3-AS1 | -0.050127077 | 0.616341336 | 0.765484597 | NS | 0.210178704 |
| FOXN3-AS2 | -0.002422934 | 0.986611427 | 0.992025943 | NS | 0.005853859 |
| LINC02317 | 0.577352504 | 0.31905839 | NA | NS | 0.496129831 |
| LINC00642 | 0.704710172 | 0.113087517 | NA | NS | 0.946585333 |
| LINC02321 | 0.822887043 | 0.061235138 | NA | NS | 1.212999299 |
| CCDC88C-DT | 0.078245355 | 0.873196365 | NA | NS | 0.058888081 |
| LINC02279 | 0.390488775 | 0.497084338 | NA | NS | 0.30356992 |
| DICER1-AS1 | 0.403095128 | 4.65E-04 | 0.036600787 | Up | 3.332210761 |
| LINC02292 | 0.617205059 | 0.067781243 | NA | NS | 1.168890469 |
| SNHG10 | -0.085248452 | 0.234717522 | 0.462791997 | NS | 0.629454489 |
| LINC02318 | 0.741833747 | 0.069764483 | NA | NS | 1.156365621 |
| TCL6 | 0.045660174 | 0.777730791 | 0.870769522 | NS | 0.109170707 |
| PAPOLA-DT | 0.182487161 | 0.317610157 | 0.537032996 | NS | 0.498105617 |
| LINC02299 | 0.552798789 | 0.3665013 | NA | NS | 0.43592448 |
| LINC02304 | 0.869620177 | 0.092767516 | NA | NS | 1.032604071 |
| LINC02325 | 0.106400568 | 0.227228949 | 0.455851208 | NS | 0.64353634 |
| LINC02291 | 0.174915665 | 0.642583359 | NA | NS | 0.192070525 |
| LINC02312 | 0.051802534 | 0.769828281 | 0.865858007 | NS | 0.113606138 |
| LINC01550 | 0.156674467 | 0.138535774 | 0.363014451 | NS | 0.858438063 |
| LINC02295 | 0.122610585 | 0.489173765 | 0.673825496 | NS | 0.310536843 |
| LINC02914 | 0.402170484 | 0.489059665 | NA | NS | 0.310638154 |
| LINC00523 | 0.535960231 | 0.281641788 | NA | NS | 0.550302908 |
| MEG3 | 0.543325225 | 0.105606359 | 0.321707479 | NS | 0.976309932 |
| MEG8 | 0.389585684 | 0.226147246 | 0.454636958 | NS | 0.645608697 |
| MEG9 | 1.094278697 | 0.054639647 | NA | NS | 1.262492118 |
| LINC02285 | -0.123725948 | 0.510186434 | NA | NS | 0.292271094 |
| LINC00524 | 1.10241664 | 0.062885105 | NA | NS | 1.201452211 |
| LINC02314 | 0.420993516 | 0.496989192 | NA | NS | 0.303653055 |
| DIO3OS | 0.755167258 | 0.07763169 | 0.281861236 | NS | 1.109960962 |
| LINC02320 | 0.570076937 | 0.064012082 | NA | NS | 1.193738044 |
| LINC00239 | -0.150371514 | 0.343165642 | 0.55833676 | NS | 0.4644962 |
| LINC02323 | 0.329472151 | 0.402401473 | NA | NS | 0.395340438 |
| LINC00677 | 0.102766637 | 0.385478424 | 0.593613964 | NS | 0.413999925 |
| LINC00605 | 0.368657475 | 0.522656344 | NA | NS | 0.281783773 |
| PPP1R13B-DT | 0.807130151 | 0.11392853 | NA | NS | 0.943367505 |
| KIF26A-DT | 1.386556525 | 0.012528424 | NA | Up | 1.90210354 |
| LINC02691 | 1.012628089 | 0.053497183 | NA | NS | 1.27166909 |
| LINC02280 | 0.481960586 | 0.181905999 | 0.412022664 | NS | 0.740152978 |
| LINC00638 | 0.254202297 | 0.140501814 | 0.364826818 | NS | 0.852318069 |
| LINC02298 | 0.115053333 | 0.484950151 | 0.670907239 | NS | 0.314302901 |
| FAM30A | -0.236305994 | 0.10981718 | 0.328033526 | NS | 0.959329715 |
| PWRN1 | 0.203224313 | 0.654489996 | NA | NS | 0.184096987 |
| PWRN2 | 0.523922815 | 0.460099361 | NA | NS | 0.33714837 |
| SNHG14 | 0.024276841 | 0.69991637 | 0.822193511 | NS | 0.154953849 |
| LINC02250 | 0.422147861 | 0.085085168 | 0.293307083 | NS | 1.07014614 |
| ATP10A-DT | 0.241901125 | 0.653517573 | NA | NS | 0.18474273 |
| LINC02346 | 0.229598827 | 0.577680962 | 0.737977147 | NS | 0.238311945 |
| LINC00929 | 0.478928656 | 0.312488541 | NA | NS | 0.505165904 |
| LINC02248 | 0.868164751 | 0.068039535 | NA | NS | 1.167238666 |
| GABRG3-AS1 | 0.766570821 | 0.130076211 | NA | NS | 0.885802122 |
| LINC02256 | 0.102834827 | 0.499992507 | 0.681759252 | NS | 0.301036504 |
| LINC02352 | 1.208306717 | 1.03E-05 | 0.003069231 | Up | 4.986353153 |
| RYR3-DT | 0.363424502 | 0.463422102 | NA | NS | 0.334023257 |
| LINC02252 | 0.599178959 | 0.295293389 | NA | NS | 0.529746275 |
| GJD2-DT | 0.726549795 | 0.127254783 | NA | NS | 0.895325886 |
| DPH6-DT | 0.356743377 | 0.175165975 | 0.404501491 | NS | 0.756550249 |
| LINC02853 | 1.000946374 | 0.060286236 | NA | NS | 1.219781833 |
| LINC02345 | 0.168985549 | 0.38128075 | 0.59100876 | NS | 0.418755121 |
| LINC01852 | 0.164423137 | 0.523204791 | NA | NS | 0.281328287 |
| LINC02895 | 0.359485389 | 0.404596107 | 0.608408408 | NS | 0.3929783 |
| LINC02694 | 0.115441592 | 0.386402182 | 0.594516814 | NS | 0.41296043 |
| LINC02915 | -0.484278368 | 0.00250969 | 0.076943659 | Down | 2.600379975 |
| THBS1-IT1 | -0.384658982 | 0.198111355 | NA | NS | 0.703090631 |
| GPR176-DT | 0.650344529 | 0.038684255 | NA | Up | 1.412465765 |
| SRP14-DT | 0.02735463 | 0.764775997 | 0.863185644 | NS | 0.116465751 |
| PAK6-AS1 | 0.284589911 | 0.535180431 | NA | NS | 0.271499775 |
| PLCB2-AS1 | -0.140849256 | 0.383500073 | 0.592439708 | NS | 0.416234549 |
| RAD51-AS1 | 0.013569957 | 0.822183257 | 0.898660858 | NS | 0.085031371 |
| SPINT1-AS1 | 0.118178652 | 0.663682103 | NA | NS | 0.178039894 |
| INO80-AS1 | 0.115133199 | 0.576845086 | NA | NS | 0.238940802 |
| OIP5-AS1 | -0.156649869 | 0.005618852 | 0.101725651 | Down | 2.250352405 |
| EHD4-AS1 | 0.117643603 | 0.339443745 | 0.555162511 | NS | 0.46923219 |
| PLA2G4E-AS1 | 1.085195022 | 0.026248827 | NA | Up | 1.580890106 |
| VPS39-DT | 0.101728859 | 0.280041269 | 0.50348007 | NS | 0.552777963 |
| CTDSPL2-DT | 0.311208744 | 0.105288049 | 0.32124631 | NS | 0.97762092 |
| EIF3J-DT | -0.005967087 | 0.932624049 | 0.962852453 | NS | 0.03029339 |
| SLC28A2-AS1 | 0.159826737 | 0.635984517 | NA | NS | 0.196553457 |
| LINC01491 | 0.697547833 | 0.232420759 | NA | NS | 0.633725085 |
| CTXN2-AS1 | 1.201863813 | 0.002577694 | 0.077799913 | Up | 2.588768674 |
| DUT-AS1 | 0.705087868 | 0.0011967 | 0.056242293 | Up | 2.922014806 |
| FBN1-DT | 0.582039412 | 0.206082008 | NA | NS | 0.685959923 |
| GABPB1-IT1 | 0.04981982 | 0.237106282 | 0.464954881 | NS | 0.62505694 |
| GABPB1-AS1 | 0.170414442 | 0.140145531 | 0.364423226 | NS | 0.853420747 |
| MIR4713HG | 0.477006156 | 0.292155656 | NA | NS | 0.534385702 |
| MAPK6-DT | 0.578066364 | 0.237157405 | NA | NS | 0.62496331 |
| CERNA1 | -0.024978961 | 0.853108383 | 0.917099521 | NS | 0.06899579 |
| LINC02490 | 0.769092159 | 0.176344104 | NA | NS | 0.753639057 |
| LINC00926 | 0.081070041 | 0.51465947 | 0.69268519 | NS | 0.288480031 |
| LINC01413 | 0.894460993 | 0.050137567 | NA | NS | 1.299836744 |
| ALDH1A2-AS1 | 0.183756705 | 0.59452924 | NA | NS | 0.225826781 |
| LIPC-AS1 | 0.479947258 | 0.012003702 | 0.134014426 | Up | 1.920684812 |
| RORA-AS1 | 0.182344692 | 0.055628198 | 0.248695897 | NS | 1.254705011 |
| RORA-AS2 | 0.397260278 | 0.135134721 | NA | NS | 0.869233052 |
| LINC02349 | 0.879622943 | 0.125877243 | NA | NS | 0.900052778 |
| VPS13C-DT | 0.514206845 | 0.098036466 | NA | NS | 1.008612352 |
| MGC15885 | 0.204398963 | 0.660316528 | NA | NS | 0.180247832 |
| TPM1-AS | 0.488265663 | 0.41012733 | NA | NS | 0.387081289 |
| LINC02568 | 0.013195283 | 0.975563221 | NA | NS | 0.010744581 |
| USP3-AS1 | 0.163590467 | 0.044474019 | 0.225641113 | Up | 1.351893622 |
| LINC01169 | 0.401054756 | 0.421230893 | NA | NS | 0.375479785 |
| LINC02206 | 0.945923581 | 0.149527809 | NA | NS | 0.825278031 |
| IQCH-AS1 | 0.088585606 | 0.183848226 | 0.414133182 | NS | 0.735540557 |
| MAP2K5-DT | 0.336352432 | 0.050854008 | 0.238708444 | NS | 1.293674811 |
| EWSAT1 | 0.339352543 | 0.477472135 | NA | NS | 0.321051969 |
| PAQR5-DT | 0.644975194 | 0.122212088 | NA | NS | 0.912885836 |
| KIF23-AS1 | 0.409611137 | 0.342006443 | NA | NS | 0.465965713 |
| LINC02896 | 0.356831098 | 0.084294583 | NA | NS | 1.074200335 |
| DRAIC | 0.20627795 | 0.188148036 | 0.417575959 | NS | 0.725500311 |
| LINC02205 | 0.648880402 | 0.160539963 | NA | NS | 0.794416843 |
| LINC02204 | 0.158004338 | 0.778841029 | NA | NS | 0.108551178 |
| SALRNA3 | 0.902696047 | 0.086714685 | NA | NS | 1.061907349 |
| SALRNA2 | 0.058559852 | 0.901469967 | NA | NS | 0.045048737 |
| CT62 | 0.865114286 | 0.088668741 | NA | NS | 1.052229459 |
| THSD4-AS1 | 0.531142036 | 0.308674961 | NA | NS | 0.510498598 |
| HEXA-AS1 | 0.079112611 | 0.561824831 | 0.726164697 | NS | 0.250399071 |
| TMEM202-AS1 | 0.091269086 | 0.348752362 | 0.562790769 | NS | 0.457482843 |
| LINC02259 | 1.02410479 | 0.024689901 | NA | Up | 1.607480649 |
| ADPGK-AS1 | 0.034620448 | 0.687018888 | 0.814158315 | NS | 0.163031323 |
| NPTN-IT1 | 0.213210698 | 0.036512127 | 0.208452907 | Up | 1.437562868 |
| INSYN1-AS1 | 0.338941211 | 0.522451286 | NA | NS | 0.281954197 |
| LOXL1-AS1 | 0.066892869 | 0.526216551 | 0.7012711 | NS | 0.278835496 |
| UBL7-DT | -0.163052808 | 0.433369138 | NA | NS | 0.36314202 |
| DNM1P35 | 0.551545446 | 0.076281643 | 0.279989714 | NS | 1.117579963 |
| LINGO1-AS1 | 1.229661641 | 0.021322479 | NA | Up | 1.671162311 |
| LINGO1-AS2 | 0.951325913 | 0.1387099 | NA | NS | 0.857892542 |
| DNAJA4-DT | 0.802400573 | 0.152383134 | NA | NS | 0.817063098 |
| ANKRD34C-AS1 | 0.516051858 | 0.222023044 | 0.450738673 | NS | 0.653601947 |
| ST20-AS1 | 0.1490245 | 0.081765894 | 0.288293278 | NS | 1.087427808 |
| LINC00927 | 0.318704014 | 0.560527407 | NA | NS | 0.251403148 |
| TMC3-AS1 | 0.128203902 | 0.332316337 | 0.549073569 | NS | 0.478448308 |
| LINC01583 | 0.426506645 | 0.355183943 | NA | NS | 0.449546675 |
| CPEB1-AS1 | 0.72148809 | 0.083896346 | NA | NS | 1.076256954 |
| SNHG21 | -0.21221548 | 0.117528905 | 0.337510592 | NS | 0.929855312 |
| LINC02883 | 0.26343837 | 0.293804172 | NA | NS | 0.531942041 |
| LINC01584 | 0.230915732 | 0.657287674 | NA | NS | 0.182244512 |
| AGBL1-AS1 | 0.530126278 | 0.347216211 | NA | NS | 0.459400007 |
| LINC00052 | 0.639344881 | 0.168913865 | NA | NS | 0.7723347 |
| NTRK3-AS1 | 0.796718149 | 0.110041908 | NA | NS | 0.958441886 |
| LINC01586 | 0.739183187 | 0.009058268 | NA | Up | 2.042954829 |
| CARMAL | 0.408236021 | 0.300120978 | NA | NS | 0.522703647 |
| POLG-DT | 0.385174827 | 0.251910469 | NA | NS | 0.598753784 |
| MIR9-3HG | 0.297231329 | 0.077699935 | 0.281965444 | NS | 1.109579343 |
| LINC00928 | 0.810845759 | 0.121672043 | NA | NS | 0.914809199 |
| ZNF710-AS1 | 0.205311268 | 0.037497747 | 0.210180429 | Up | 1.42599483 |
| IDH2-DT | 0.929748254 | 0.067353494 | NA | NS | 1.17163987 |
| CRTC3-AS1 | 0.084887017 | 0.646395424 | 0.786387676 | NS | 0.189501727 |
| LINC01585 | 0.294497648 | 0.382790637 | NA | NS | 0.417038693 |
| RCCD1-AS1 | 0.158846062 | 0.403735921 | NA | NS | 0.393902609 |
| PRC1-AS1 | 0.325934559 | 0.21589518 | 0.445323914 | NS | 0.665757053 |
| VPS33B-DT | 0.655340945 | 0.090288211 | NA | NS | 1.044368954 |
| CRAT37 | -0.177652489 | 0.382995869 | 0.592180083 | NS | 0.416805911 |
| C15orf32 | 1.356958503 | 0.03240152 | NA | Up | 1.489434615 |
| LINC00930 | 0.343199602 | 0.413802481 | NA | NS | 0.38320691 |
| CHASERR | -0.014849985 | 0.794603455 | 0.881302161 | NS | 0.099849551 |
| LINC01579 | 0.381007152 | 0.143972758 | 0.369130894 | NS | 0.841719674 |
| LINC02207 | -0.021673049 | 0.904838307 | 0.947351639 | NS | 0.043429021 |
| LINC01580 | 0.226622714 | 0.539449761 | NA | NS | 0.268048995 |
| LINC01581 | 0.440807984 | 0.196971523 | NA | NS | 0.705596558 |
| LINC02852 | 0.034366572 | 0.957915001 | NA | NS | 0.018673026 |
| LETR1 | 0.788642188 | 0.073822832 | 0.276013938 | NS | 1.1318093 |
| LINC00924 | 0.383867883 | 0.409323689 | NA | NS | 0.38793312 |
| NR2F2-AS1 | 0.507118606 | 0.228579484 | 0.457186791 | NS | 0.640962752 |
| LINC02157 | 0.173385081 | 0.772271056 | NA | NS | 0.112230242 |
| SPATA8-AS1 | 0.658365626 | 0.27924859 | NA | NS | 0.554009011 |
| SPATA8 | 0.550476935 | 0.377053008 | NA | NS | 0.42359759 |
| LINC02253 | 0.692158652 | 0.169132772 | NA | NS | 0.771772233 |
| LINC02254 | 0.377482864 | 0.423391845 | 0.622593337 | NS | 0.373257511 |
| LINC00923 | 0.325492768 | 0.328218991 | 0.545927179 | NS | 0.483836294 |
| LINC02251 | 0.599820072 | 0.298198402 | NA | NS | 0.525494689 |
| LINC01582 | 1.277334298 | 0.007326333 | NA | Up | 2.135113344 |
| LINC02351 | 0.618784464 | 0.277641748 | NA | NS | 0.55651523 |
| IRAIN | 0.414323886 | 0.027487226 | 0.186246285 | Up | 1.560869092 |
| SYNM-AS1 | 0.271291063 | 0.123135688 | 0.343867031 | NS | 0.909616059 |
| LUNAR1 | 0.119317284 | 0.473003431 | 0.661257757 | NS | 0.325135709 |
| LINC02244 | 0.3661029 | 0.097794587 | NA | NS | 1.009685184 |
| SPATA41 | 0.109814269 | 0.3480663 | 0.562578646 | NS | 0.458338023 |
| CERS3-AS1 | 0.247103416 | 0.540131641 | NA | NS | 0.267500381 |
| GCAWKR | 0.50272664 | 0.192653718 | 0.421709861 | NS | 0.715222604 |
| ALDH1A3-AS1 | 0.268195673 | 0.138279375 | 0.362971321 | NS | 0.859242593 |
| SNRPA1-DT | 0.308127036 | 0.193937315 | NA | NS | 0.712338621 |
| PCSK6-AS1 | 0.964660699 | 0.118301654 | NA | NS | 0.927009184 |
| LINC02348 | 0.295038973 | 0.294125389 | 0.51621271 | NS | 0.531467485 |
| WASIR2 | 0.086477742 | 0.740964476 | NA | NS | 0.130202613 |
| STUB1-DT | 0.818679006 | 0.046425069 | NA | Up | 1.33324744 |
| LMF1-AS1 | 0.253924618 | 0.258617248 | 0.485189351 | NS | 0.587342514 |
| CEROX1 | 0.043274012 | 0.820057419 | 0.897279394 | NS | 0.086155738 |
| SSTR5-AS1 | 0.781216942 | 0.125896236 | NA | NS | 0.899987255 |
| SNHG9 | -0.063840332 | 0.590863784 | 0.748022567 | NS | 0.228512629 |
| SNHG19 | -0.008127289 | 0.948181946 | 0.971776348 | NS | 0.023108318 |
| MIR3677HG | 0.03050502 | 0.80330448 | 0.886991739 | NS | 0.095119811 |
| TEDC2-AS1 | 0.486431039 | 0.019847043 | NA | Up | 1.702304197 |
| ERVK13-1 | 0.119401115 | 0.086752446 | 0.296051934 | NS | 1.061718269 |
| SRRM2-AS1 | 0.214904656 | 0.223743445 | 0.452337751 | NS | 0.650249679 |
| PRSS30P | -0.117390054 | 0.630303532 | NA | NS | 0.200450259 |
| MMP25-AS1 | 0.153562353 | 0.34857974 | 0.562710453 | NS | 0.457697859 |
| ZNF213-AS1 | 0.093696773 | 0.197934485 | 0.42773123 | NS | 0.703478535 |
| LINC00921 | 0.344031524 | 4.27E-04 | 0.035144156 | Up | 3.369576382 |
| LINC02861 | 0.622128498 | 0.075327123 | NA | NS | 1.12304862 |
| LINC01569 | 0.223292351 | 0.148438786 | 0.374564243 | NS | 0.828452607 |
| LINC02164 | 1.217229627 | 0.019984297 | NA | Up | 1.699311126 |
| LINC01570 | 0.844001005 | 0.072674476 | NA | NS | 1.138618093 |
| LINC02152 | 0.102570712 | 0.858902275 | NA | NS | 0.066056247 |
| CARHSP1-DT | 0.559429421 | 0.406258933 | NA | NS | 0.391197076 |
| USP7-AS1 | 0.029533529 | 0.866953539 | 0.924863516 | NS | 0.062004176 |
| LINC02177 | 0.381571858 | 0.381429047 | 0.591038759 | NS | 0.418586237 |
| LINC01177 | 0.708374592 | 0.238623239 | NA | NS | 0.622287264 |
| LINC01195 | 1.364204405 | 0.017171471 | NA | Up | 1.765192488 |
| BCAR4 | 0.22550782 | 0.675840405 | NA | NS | 0.170155847 |
| RSL1D1-DT | 0.369462964 | 0.001142375 | 0.054782928 | Up | 2.942191167 |
| LINC02185 | 0.093561098 | 0.650359404 | 0.789197853 | NS | 0.186846575 |
| LINC02186 | 0.520585165 | 0.482320558 | NA | NS | 0.316664226 |
| MIR193BHG | 0.666560943 | 0.047049798 | 0.230692912 | Up | 1.327442233 |
| LINC02130 | 1.381353788 | 0.024302506 | NA | Up | 1.614348941 |
| SMG1-DT | 0.100735171 | 0.67282412 | NA | NS | 0.172098448 |
| COQ7-DT | 0.779747479 | 0.180227293 | NA | NS | 0.74417944 |
| LINC02858 | 0.867665446 | 0.133283147 | NA | NS | 0.875224763 |
| LINC02194 | 0.747677884 | 0.159543459 | NA | NS | 0.797120997 |
| LINC01567 | 1.003617981 | 0.071826853 | NA | NS | 1.143713162 |
| LINC02175 | 0.109471213 | 0.267254513 | 0.493205152 | NS | 0.573074952 |
| LCMT1-AS1 | 0.125783972 | 0.444532831 | 0.638698645 | NS | 0.352096158 |
| LCMT1-AS2 | 0.090455994 | 0.346943636 | 0.561433348 | NS | 0.459741075 |
| ZKSCAN2-DT | 0.023186058 | 0.742619222 | 0.849009739 | NS | 0.129233813 |
| LINC02191 | 0.270833629 | 0.608398148 | NA | NS | 0.215812116 |
| LINC02195 | 0.083189829 | 0.845699616 | NA | NS | 0.072783867 |
| LINC02129 | 0.474498066 | 0.463196685 | NA | NS | 0.334234558 |
| NSMCE1-DT | 0.281288683 | 0.018110423 | 0.156751663 | Up | 1.742071415 |
| IL21R-AS1 | 0.048918834 | 0.708475617 | 0.82730768 | NS | 0.149675092 |
| ATP2A1-AS1 | 0.802301253 | 0.086094957 | NA | NS | 1.065022286 |
| RRN3P2 | -0.032790208 | 0.783645943 | 0.87491149 | NS | 0.10588011 |
| KCTD13-DT | -0.059709217 | 0.763971327 | NA | NS | 0.116922941 |
| YPEL3-DT | 0.03786163 | 0.800027355 | 0.884747042 | NS | 0.096895163 |
| CORO1A-AS1 | 0.023452751 | 0.792707698 | 0.880521472 | NS | 0.100886924 |
| CD2BP2-DT | 0.251198603 | 0.022477795 | 0.171252981 | Up | 1.648246295 |
| MIR762HG | -0.072219683 | 0.51028987 | 0.689437709 | NS | 0.292183053 |
| FBXL19-AS1 | 0.326523992 | 0.012869081 | 0.136134668 | Up | 1.890452457 |
| LINC02190 | 0.494620417 | 0.376353348 | NA | NS | 0.424404217 |
| LINC00273 | 1.106432377 | 0.074510526 | NA | NS | 1.127782372 |
| LINC01566 | 0.745198998 | 0.190726777 | NA | NS | 0.71958833 |
| LINC02167 | 0.617379284 | 0.281500377 | NA | NS | 0.550521019 |
| DNAJA2-DT | -0.12341317 | 0.33776872 | 0.553781471 | NS | 0.471380572 |
| ITFG1-AS1 | 0.100350803 | 0.453660547 | 0.646056016 | NS | 0.343268988 |
| LINC02133 | 0.734465421 | 0.098689024 | NA | NS | 1.005731147 |
| LINC02192 | 0.485310032 | 0.448437014 | NA | NS | 0.348298548 |
| LINC02134 | 0.946499753 | 0.061695059 | NA | NS | 1.209749617 |
| LINC02179 | 0.385970217 | 0.364002202 | NA | NS | 0.438895989 |
| HEATR3-AS1 | 0.622527469 | 0.366468703 | NA | NS | 0.435963109 |
| LINC02178 | 0.750792163 | 0.207692759 | NA | NS | 0.682578644 |
| CYLD-AS1 | 0.123497829 | 0.129148418 | 0.351110762 | NS | 0.888910911 |
| LINC02168 | 0.605305491 | 0.006897889 | NA | Up | 2.161283798 |
| LINC02128 | 0.698606209 | 0.122145619 | 0.342932892 | NS | 0.913122105 |
| LINC02127 | 0.240344025 | 0.643830223 | NA | NS | 0.191228641 |
| LINC01571 | 0.784333646 | 0.119574803 | NA | NS | 0.922360328 |
| LINC02911 | 0.667422644 | 0.144282608 | NA | NS | 0.840786015 |
| LINC00919 | 0.463971201 | 0.337630557 | NA | NS | 0.471558255 |
| LINC02180 | 0.036013402 | 0.949877495 | NA | NS | 0.022332402 |
| CASC22 | 0.293270404 | 0.512638707 | NA | NS | 0.290188605 |
| CASC16 | 0.747350074 | 0.138472207 | NA | NS | 0.858637386 |
| LINC02169 | 0.768165085 | 0.145252848 | NA | NS | 0.837875343 |
| LINC02140 | 0.301127477 | 0.502318265 | NA | NS | 0.29902103 |
| LINC02183 | 0.646198945 | 0.225260267 | NA | NS | 0.647315406 |
| CRNDE | 0.306201207 | 0.266728877 | 0.492750241 | NS | 0.573929963 |
| MMP2-AS1 | 0.11131208 | 0.736324751 | NA | NS | 0.132930601 |
| GNAO1-DT | 0.269870127 | 0.293123344 | 0.515386052 | NS | 0.532949593 |
| GNAO1-AS1 | -0.01261094 | 0.965709793 | NA | NS | 0.015153364 |
| NUP93-DT | 0.350703734 | 0.054563882 | 0.24676996 | NS | 1.263094741 |
| CPNE2-DT | 0.218645715 | 0.437366371 | NA | NS | 0.359154613 |
| LINC02137 | 0.707195958 | 0.096090457 | NA | NS | 1.017319739 |
| LINC02141 | 0.413960124 | 0.332937319 | 0.549703012 | NS | 0.477637522 |
| CDH8-AS1 | 0.75786643 | 0.335220146 | NA | NS | 0.474669889 |
| LINC02165 | 0.536292087 | 0.315741509 | NA | NS | 0.50066832 |
| LINC02126 | 0.489625173 | 0.351115079 | NA | NS | 0.454550518 |
| LINC00922 | 0.794292455 | 0.078204382 | NA | NS | 1.106768909 |
| LINC00920 | -0.236911765 | 0.09171953 | 0.303537202 | NS | 1.03753818 |
| BEAN1-AS1 | 0.363453142 | 0.104218825 | 0.320245132 | NS | 0.98205383 |
| DYNC1LI2-DT | 0.56190905 | 0.225744853 | NA | NS | 0.646382142 |
| ATP6V0D1-DT | 0.110758911 | 0.18771142 | 0.417152261 | NS | 0.726509304 |
| CTCF-DT | -0.010340542 | 0.921086069 | 0.956459449 | NS | 0.035699786 |
| NQO1-DT | 0.292320516 | 0.317072781 | NA | NS | 0.498841038 |
| PDXDC2P-NPIPB14P | 0.216985876 | 0.096930682 | 0.311085548 | NS | 1.013538733 |
| DDX19A-DT | 0.201210219 | 0.224975874 | 0.453236933 | NS | 0.647864053 |
| VAC14-AS1 | 0.231438666 | 0.025697645 | 0.181747049 | Up | 1.590106678 |
| LINC02136 | 1.151342421 | 0.019494322 | NA | Up | 1.710091862 |
| TAT-AS1 | 0.420132503 | 0.164741317 | NA | NS | 0.783197465 |
| LINC01572 | -0.085621717 | 0.491592599 | 0.675742923 | NS | 0.308394664 |
| HCCAT5 | 0.410311357 | 0.468107713 | NA | NS | 0.329654203 |
| LINC01568 | 0.325449404 | 0.449652501 | 0.643129524 | NS | 0.347122986 |
| PSMD7-DT | 0.2327098 | 0.415871216 | 0.617097486 | NS | 0.381041137 |
| LINC02125 | 0.150110135 | 0.773847617 | NA | NS | 0.11134455 |
| LINC02131 | 0.636196033 | 0.305380332 | NA | NS | 0.515158937 |
| WWOX-AS1 | 0.33540672 | 0.128428574 | 0.350159792 | NS | 0.89133834 |
| LINC01229 | 0.363014324 | 0.207631867 | 0.437401068 | NS | 0.682705992 |
| MAFTRR | 0.612335198 | 0.014612405 | 0.144746239 | Up | 1.835278292 |
| LINC01228 | 0.422310353 | 0.466088198 | NA | NS | 0.331531894 |
| DYNLRB2-AS1 | 0.398806956 | 0.251285229 | 0.478143429 | NS | 0.599833039 |
| LINC01227 | 0.514487936 | 0.218252142 | 0.447725775 | NS | 0.661041485 |
| ARLNC1 | 0.567416949 | 0.157888262 | 0.385290564 | NS | 0.801650156 |
| CENPN-AS1 | 0.384153915 | 0.197565504 | NA | NS | 0.704288883 |
| MPHOSPH6-DT | 0.779860777 | 0.148584674 | NA | NS | 0.828025985 |
| MBTPS1-DT | 0.882173847 | 0.018152376 | NA | Up | 1.741066527 |
| ATP2C2-AS1 | 0.334131955 | 0.063895949 | NA | NS | 1.194526677 |
| LINC02176 | 1.27446673 | 0.047129821 | NA | Up | 1.326704213 |
| LINC02139 | 0.484371646 | 0.141580903 | NA | NS | 0.848995323 |
| LINC00311 | 0.669926839 | 0.191108723 | NA | NS | 0.71871949 |
| LINC02132 | 0.616616708 | 0.312041734 | NA | NS | 0.505787318 |
| LINC01082 | 0.566243931 | 0.293446717 | NA | NS | 0.532470745 |
| LINC01081 | 0.463472765 | 0.379163958 | NA | NS | 0.421172952 |
| LINC02135 | 0.320225452 | 0.596828731 | NA | NS | 0.224150278 |
| LINC00917 | 0.341354335 | 0.483928521 | NA | NS | 0.315218781 |
| FENDRR | 0.63880739 | 0.135968252 | NA | NS | 0.866562486 |
| LINC02188 | 0.79556159 | 0.063953994 | 0.262496805 | NS | 1.194132329 |
| LINC02189 | 0.091044157 | 0.905457265 | NA | NS | 0.043132042 |
| LINC02181 | 0.316883283 | 0.392279393 | NA | NS | 0.406404506 |
| C16orf95-DT | 0.127597631 | 0.478202571 | NA | NS | 0.320388093 |
| ZCCHC14-DT | 0.534244619 | 0.097387376 | NA | NS | 1.011497336 |
| LINC02182 | 0.584089291 | 0.400913059 | NA | NS | 0.396949798 |
| ZFPM1-AS1 | 0.355897394 | 0.08043889 | NA | NS | 1.094533928 |
| SNAI3-AS1 | 0.154607716 | 0.041117246 | 0.218382923 | Up | 1.385975984 |
| LINC00304 | 0.912580374 | 0.014899407 | NA | Up | 1.826831003 |
| LINC02138 | 1.336034325 | 0.005387169 | NA | Up | 2.268639372 |
| LINC02166 | 0.247438553 | 0.493291334 | NA | NS | 0.306896514 |
| VPS9D1-AS1 | 0.197030704 | 0.225236966 | 0.453643215 | NS | 0.647360331 |
| GAS8-AS1 | 0.527737894 | 0.133521946 | NA | NS | 0.874447345 |
| FAM157C | 0.136450751 | 0.314387473 | 0.533741944 | NS | 0.502534767 |
| LINC02193 | 0.142417578 | 0.468295204 | 0.657888405 | NS | 0.32948029 |
| LINC02887 | 0.046090204 | 0.883628782 | NA | NS | 0.053730147 |
| PITPNA-AS1 | 0.048848097 | 0.76850868 | 0.865096376 | NS | 0.114351223 |
| MIR22HG | -0.49739167 | 0.034872616 | 0.204782731 | Down | 1.457515473 |
| LINC01975 | 1.208707673 | 0.038439939 | NA | Up | 1.415217314 |
| LINC01996 | 0.653018378 | 0.108768884 | NA | NS | 0.963495327 |
| CAMTA2-AS1 | 0.593123894 | 0.039126327 | NA | Up | 1.407530921 |
| KIF1C-AS1 | 0.075778 | 0.754846942 | NA | NS | 0.1221411 |
| ZFP3-DT | 0.008283869 | 0.982787924 | NA | NS | 0.007540189 |
| ZNF232-AS1 | 0.168095231 | 0.261277565 | 0.487278404 | NS | 0.582897879 |
| ZNF594-DT | 0.055227996 | 0.616866087 | 0.765716375 | NS | 0.209809105 |
| DHX33-DT | 0.227802677 | 0.405227677 | NA | NS | 0.3923009 |
| ALOX12-AS1 | -6.00E-04 | 0.991178823 | 0.994893433 | NS | 0.003847985 |
| MIR497HG | 0.396627653 | 0.34586669 | NA | NS | 0.461091262 |
| LINC00324 | -0.061540432 | 0.389032249 | 0.596625801 | NS | 0.410014396 |
| PIK3R5-DT | 0.429998985 | 0.003381625 | 0.085105461 | Up | 2.470874589 |
| MYHAS | 0.294072199 | 0.643923124 | NA | NS | 0.191165979 |
| TMEM220-AS1 | 0.345903999 | 0.176346159 | NA | NS | 0.753633995 |
| LINC00670 | -0.034220849 | 0.942285197 | 0.968413333 | NS | 0.025817631 |
| MYOCD-AS1 | 0.259182923 | 0.646371275 | NA | NS | 0.189517952 |
| ARHGAP44-AS1 | 0.48797528 | 0.273713001 | NA | NS | 0.562704573 |
| LINC02093 | 0.16582519 | 0.786569971 | NA | NS | 0.104262638 |
| COX10-AS1 | 0.084291257 | 0.151892305 | 0.37849039 | NS | 0.818464226 |
| LINC02096 | 0.547947446 | 0.257959437 | NA | NS | 0.58844858 |
| CDRT7 | 0.034544486 | 0.94934042 | NA | NS | 0.022578028 |
| CDRT8 | 0.778137317 | 0.231505847 | NA | NS | 0.635438035 |
| TBC1D26-AS1 | 0.777307531 | 0.291334887 | NA | NS | 0.535607506 |
| LINC02087 | 0.928859235 | 0.069192605 | NA | NS | 1.159940316 |
| SNHG29 | -0.331861762 | 0.012330011 | 0.134659948 | Down | 1.909036537 |
| FAM106C | 0.108174163 | 0.787210338 | NA | NS | 0.103909211 |
| LINC02090 | 0.164170332 | 0.60475819 | NA | NS | 0.218418241 |
| MPRIP-AS1 | 0.212698546 | 0.163664804 | 0.391382977 | NS | 0.786044705 |
| SMCR2 | 0.295005068 | 0.661200205 | NA | NS | 0.17966702 |
| RAI1-AS1 | -0.133271192 | 0.360687443 | 0.573444403 | NS | 0.442868976 |
| SMCR5 | 0.455107682 | 0.046261247 | 0.229182357 | Up | 1.334782666 |
| FAM106A | -0.010217754 | 0.965338538 | 0.980581176 | NS | 0.015320356 |
| EPN2-AS1 | 0.508451803 | 0.021812945 | NA | Up | 1.661285689 |
| LINC02094 | 1.102903345 | 0.074535514 | NA | NS | 1.127636749 |
| SPECC1-DT | 0.699896027 | 0.116858461 | NA | NS | 0.932339837 |
| LINC02088 | 0.850867929 | 0.184903628 | NA | NS | 0.733054567 |
| CCDC144NL-AS1 | 0.241656055 | 0.394755733 | 0.600481589 | NS | 0.403671554 |
| LINC01563 | 0.394667317 | 0.432827122 | NA | NS | 0.363685533 |
| LINC02693 | 0.202555199 | 0.079277379 | 0.284578454 | NS | 1.100850715 |
| LINC02002 | 0.600718575 | 0.324944176 | NA | NS | 0.488191242 |
| FAM27E5 | 0.609686816 | 0.331947065 | NA | NS | 0.478931167 |
| FLJ36000 | 0.473114974 | 0.317321392 | NA | NS | 0.498500649 |
| LINC01992 | 0.301976351 | 0.443020006 | NA | NS | 0.353576661 |
| ERVE-1 | 0.256101286 | 0.608826074 | NA | NS | 0.215506757 |
| SPAG5-AS1 | -0.03810918 | 0.755350812 | 0.85689345 | NS | 0.1218513 |
| ABHD15-AS1 | 0.279723264 | 0.161254146 | 0.389034501 | NS | 0.792489112 |
| MIR4733HG | 0.788720277 | 0.082740903 | NA | NS | 1.082279741 |
| AA06 | -0.015456474 | 0.981901306 | NA | NS | 0.007932162 |
| LINC01989 | 0.84816802 | 0.086038636 | NA | NS | 1.065306481 |
| TMEM132E-DT | 0.790333939 | 0.087338757 | NA | NS | 1.058792991 |
| SNHG30 | -0.14893664 | 0.048356305 | 0.233310996 | Down | 1.315546894 |
| CCL3-AS1 | 0.080942054 | 0.820173853 | NA | NS | 0.08609408 |
| LINC02079 | 0.304645666 | 0.645218783 | NA | NS | 0.190292998 |
| RARA-AS1 | 0.182919613 | 0.08490248 | 0.293013352 | NS | 1.071079622 |
| KRT10-AS1 | -0.045006781 | 0.657937139 | 0.794394578 | NS | 0.181815598 |
| LINC00974 | -0.260911518 | 0.660939968 | NA | NS | 0.179837985 |
| RAB5C-AS1 | 0.265368165 | 0.25782857 | NA | NS | 0.588668961 |
| HSD17B1-AS1 | 0.377522537 | 0.004149315 | 0.091333846 | Up | 2.382023599 |
| RAMP2-AS1 | 0.666962729 | 0.197809774 | NA | NS | 0.703752253 |
| LINC00671 | 0.27455573 | 0.104746255 | 0.320720021 | NS | 0.979861494 |
| NBR2 | 0.100104994 | 0.410104253 | 0.613068381 | NS | 0.387105727 |
| LINC00910 | 0.087939714 | 0.385896838 | 0.594058572 | NS | 0.41352878 |
| MIR2117HG | 1.125729976 | 0.061419424 | NA | NS | 1.211694263 |
| LINC02594 | 0.5523513 | 0.110675998 | NA | NS | 0.955946553 |
| FAM215A | 0.545118676 | 0.148659264 | NA | NS | 0.827808022 |
| LINC01976 | 1.493780127 | 0.011438366 | NA | Up | 1.941636012 |
| ASB16-AS1 | 0.15357482 | 0.10112392 | 0.316688091 | NS | 0.995146105 |
| ATXN7L3-AS1 | 0.066659295 | 0.415095567 | 0.616465773 | NS | 0.381851904 |
| RUNDC3A-AS1 | 0.14181551 | 0.576896194 | 0.737567537 | NS | 0.238902326 |
| LINC01180 | 1.12081443 | 0.042907994 | NA | Up | 1.367461784 |
| KIF18B-DT | 0.555264559 | 0.0564142 | NA | NS | 1.248611569 |
| FMNL1-DT | 0.072792075 | 0.362156502 | 0.574723698 | NS | 0.441103713 |
| MAP3K14-AS1 | -0.046219851 | 0.619607318 | 0.767330709 | NS | 0.207883461 |
| MAPT-IT1 | 1.072567252 | 0.110226762 | NA | NS | 0.957712949 |
| KANSL1-AS1 | 0.024253922 | 0.929950672 | NA | NS | 0.031540087 |
| FAM215B | 0.651282861 | 0.11216445 | NA | NS | 0.950144768 |
| LINC01974 | 0.845486398 | 0.157857005 | NA | NS | 0.801736142 |
| GOSR2-DT | 0.505593332 | 0.245795804 | NA | NS | 0.609425535 |
| EFCAB13-DT | 0.41529373 | 0.056475859 | NA | NS | 1.248137151 |
| KPNB1-DT | 0.20604355 | 0.027680121 | 0.186639939 | Up | 1.557832011 |
| SP2-DT | 0.103009649 | 0.469984852 | 0.659019796 | NS | 0.32791614 |
| SP2-AS1 | 0.116239353 | 0.025166163 | 0.179326528 | Up | 1.599182999 |
| NFE2L1-DT | -0.038996591 | 0.766123017 | 0.863854529 | NS | 0.11570149 |
| SKAP1-AS1 | 0.263247636 | 0.196104907 | 0.425986774 | NS | 0.707511539 |
| SKAP1-AS2 | 0.161229094 | 0.188469813 | 0.417733637 | NS | 0.7247582 |
| HOXB-AS1 | -0.129709779 | 0.39612 | 0.601442813 | NS | 0.40217323 |
| HOXB-AS3 | 1.241182442 | 0.013044434 | NA | Up | 1.884574761 |
| HOXB-AS2 | 0.486218204 | 0.175835204 | NA | NS | 0.754894169 |
| HOXB-AS4 | 0.69080571 | 0.042923785 | NA | Up | 1.367301991 |
| LINC02086 | 0.395185882 | 0.178000173 | 0.407982383 | NS | 0.749579574 |
| FLJ40194 | 0.077406994 | 0.511320741 | 0.690016358 | NS | 0.29130659 |
| ZNF652-AS1 | 0.171677114 | 0.094017135 | 0.30725936 | NS | 1.026792986 |
| LINC02075 | 0.176427222 | 0.758707026 | NA | NS | 0.119925894 |
| NGFR-AS1 | 0.014358814 | 0.981355364 | NA | NS | 0.0081737 |
| PICART1 | 0.315092732 | 0.306457947 | NA | NS | 0.513629112 |
| TMEM92-AS1 | 0.225807912 | 0.280294483 | 0.503553832 | NS | 0.55238545 |
| CACNA1G-AS1 | 0.312460541 | 0.399474805 | NA | NS | 0.398510607 |
| TOB1-AS1 | 0.271909007 | 0.02382098 | 0.175023449 | Up | 1.623040371 |
| LINC02071 | 0.167652995 | 0.753890765 | NA | NS | 0.122691576 |
| LINC02073 | 0.498323059 | 0.094264917 | 0.307415197 | NS | 1.02564991 |
| LINC02089 | 0.178219311 | 0.7707366 | NA | NS | 0.113094017 |
| LINC02876 | 0.7974379 | 0.18387983 | NA | NS | 0.735465906 |
| AKAP1-DT | -0.051940096 | 0.736868706 | 0.844971316 | NS | 0.132609887 |
| DYNLL2-DT | 0.423969402 | 0.325485489 | 0.54398808 | NS | 0.487468369 |
| TSPOAP1-AS1 | 0.154480107 | 0.003555549 | 0.086397822 | Up | 2.449093309 |
| SEPTIN4-AS1 | -0.011974456 | 0.973549565 | NA | NS | 0.011641933 |
| LINC01476 | 0.218427297 | 0.44709613 | 0.641084121 | NS | 0.349599089 |
| RNFT1-DT | 0.024965929 | 0.919180608 | NA | NS | 0.036599147 |
| HEATR6-DT | 0.216230314 | 0.303052302 | NA | NS | 0.518482413 |
| APPBP2-DT | 0.074627272 | 0.603011471 | 0.756265742 | NS | 0.219674426 |
| LINC01999 | 0.823118588 | 0.341454211 | NA | NS | 0.466667527 |
| TBX2-AS1 | 0.516892516 | 0.042526507 | NA | Up | 1.371340289 |
| LINC02875 | 0.952364881 | 0.059060681 | NA | NS | 1.228701546 |
| MARCHF10-DT | 0.674859133 | 0.267738888 | NA | NS | 0.572288545 |
| PRR29-AS1 | 0.228202531 | 0.448647587 | NA | NS | 0.348094664 |
| SNHG25 | -0.161614737 | 0.376865304 | 0.586981039 | NS | 0.423813844 |
| ARHGAP27P1-BPTFP1-KPNA2P3 | 0.028002732 | 0.765581717 | 0.863648023 | NS | 0.116008446 |
| LINC02563 | -0.439414419 | 0.485002242 | NA | NS | 0.314256254 |
| PRKCA-AS1 | 0.071551566 | 0.668775079 | 0.802193833 | NS | 0.174719919 |
| HELZ-AS1 | 0.871667352 | 3.95E-05 | NA | Up | 4.403753923 |
| LINC01482 | 0.260519486 | 0.514899907 | NA | NS | 0.288277187 |
| ABCA9-AS1 | 0.879965598 | 0.022500102 | NA | Up | 1.647815521 |
| LINC01483 | 0.312609398 | 0.193855692 | 0.423149357 | NS | 0.712521444 |
| LINC01497 | 0.807904899 | 0.092379039 | NA | NS | 1.034426561 |
| LINC01028 | 0.892091011 | 0.110116329 | NA | NS | 0.958148276 |
| KCNJ2-AS1 | 0.350222762 | 0.010814602 | 0.12856202 | Up | 1.965989462 |
| CASC17 | 0.567015535 | 0.309955287 | NA | NS | 0.508700952 |
| ROCR | 0.602756786 | 0.210895924 | NA | NS | 0.675931814 |
| LINC01152 | 0.61010615 | 0.196868637 | NA | NS | 0.705823465 |
| SOX9-AS1 | 0.512284271 | 0.22423998 | 0.452670254 | NS | 0.649286953 |
| LINC02097 | 0.396408865 | 0.37461011 | NA | NS | 0.426420506 |
| LINC00511 | 0.490834962 | 0.085085494 | 0.293307083 | NS | 1.070144477 |
| LINC02003 | 0.654090907 | 0.16861274 | NA | NS | 0.773109613 |
| LINC00469 | -0.281552998 | 0.310076489 | NA | NS | 0.508531162 |
| LINC02092 | 0.08691993 | 0.777378627 | NA | NS | 0.109367404 |
| LINC02074 | 0.222604733 | 0.634930333 | NA | NS | 0.197273925 |
| MGC16275 | 0.019474999 | 0.809543975 | 0.890541146 | NS | 0.091759555 |
| CD300LD-AS1 | 0.306092603 | 0.406670911 | NA | NS | 0.390756891 |
| SLC9A3R1-AS1 | 0.67137611 | 0.018891823 | NA | Up | 1.723726133 |
| HID1-AS1 | 0.597128882 | 0.268718252 | NA | NS | 0.570702835 |
| MIF4GD-DT | 0.172976482 | 0.096503802 | 0.310542373 | NS | 1.015455577 |
| RNF157-AS1 | 0.090703292 | 0.450820358 | 0.643924649 | NS | 0.345996481 |
| SNHG16 | -0.077758872 | 0.159266979 | 0.386966794 | NS | 0.797874258 |
| LINC02080 | 0.66239675 | 0.065194972 | NA | NS | 1.185785898 |
| LINC00868 | 0.296195403 | 0.613214861 | NA | NS | 0.212387329 |
| SNHG20 | 0.024982317 | 0.720594567 | 0.835048159 | NS | 0.142309017 |
| LINC01987 | 0.991432546 | 0.075385735 | NA | NS | 1.122710826 |
| LINC01973 | 0.319709097 | 0.149550412 | 0.37567221 | NS | 0.825212386 |
| LINC01993 | 0.858531549 | 0.003900511 | 0.089796534 | Up | 2.408878437 |
| SOCS3-DT | 0.585100336 | 0.030221303 | NA | Up | 1.519686816 |
| DNAH17-AS1 | 0.494709239 | 0.293317458 | NA | NS | 0.532662088 |
| SCAT1 | 0.414339427 | 0.038558062 | 0.213464789 | Up | 1.413884808 |
| C1QTNF1-AS1 | 0.43757075 | 0.358130991 | NA | NS | 0.445958096 |
| LINC02078 | 0.441382542 | 0.141358093 | 0.365805995 | NS | 0.849679321 |
| LINC01977 | 0.473917281 | 0.090554753 | NA | NS | 1.043088752 |
| LINC01979 | 0.608945901 | 0.218487127 | NA | NS | 0.660574147 |
| LINC01978 | 0.862017676 | 0.093635932 | NA | NS | 1.028557463 |
| RNF213-AS1 | 0.071985125 | 0.626022948 | 0.77193655 | NS | 0.203409747 |
| BAIAP2-DT | -0.258309678 | 0.005032938 | 0.097887596 | Down | 2.298178422 |
| LINC00482 | 4.02E-05 | 0.999800256 | 0.999802152 | NS | 8.68E-05 |
| TMEM105 | 0.697351387 | 0.117541699 | NA | NS | 0.929808035 |
| RENO1 | 0.39347424 | 0.027620096 | 0.186639939 | Up | 1.558774824 |
| LINC01971 | 0.853551261 | 0.183139476 | NA | NS | 0.737218033 |
| MAFG-DT | 1.019406811 | 0.045236716 | NA | Up | 1.344508927 |
| DCXR-DT | 0.812240245 | 0.074017682 | NA | NS | 1.130664522 |
| LINC01970 | 0.730588288 | 0.016971227 | NA | Up | 1.770286764 |
| HEXD-IT1 | 0.134141674 | 0.49062811 | 0.674954298 | NS | 0.309247573 |
| NARF-AS2 | -0.024367224 | 0.828561447 | 0.902488075 | NS | 0.081675278 |
| NARF-AS1 | 0.147202927 | 0.379080615 | 0.589025245 | NS | 0.421268424 |
| NARF-IT1 | 0.147099445 | 0.246313193 | 0.474517559 | NS | 0.608512326 |
| THOC1-DT | 0.056527786 | 0.668229055 | 0.801748837 | NS | 0.175074645 |
| LINC01925 | 1.35734937 | 0.004441499 | NA | Up | 2.352470395 |
| TYMSOS | 0.641243504 | 0.075940673 | NA | NS | 1.11952556 |
| LINC01904 | -0.08324844 | 0.899819303 | NA | NS | 0.045844694 |
| LINC00470 | 0.378656933 | 0.258017447 | 0.484751072 | NS | 0.588350927 |
| MYL12-AS1 | -0.066218682 | 0.442164242 | 0.636699969 | NS | 0.354416381 |
| LINC01895 | 0.611938125 | 0.269627723 | NA | NS | 0.569235456 |
| GAPLINC | 0.617045247 | 0.155061526 | NA | NS | 0.809495947 |
| DLGAP1-AS1 | 0.157363794 | 0.033993924 | 0.202672427 | Up | 1.4685987 |
| DLGAP1-AS2 | 0.085728669 | 0.413039829 | 0.614968026 | NS | 0.384008067 |
| DLGAP1-AS3 | 0.402379896 | 0.189287553 | 0.418800923 | NS | 0.722877943 |
| DLGAP1-AS4 | 0.195863186 | 0.610023813 | NA | NS | 0.214653211 |
| DLGAP1-AS5 | 0.348753093 | 0.278353805 | NA | NS | 0.555402837 |
| LINC01892 | 0.464146686 | 0.358863052 | NA | NS | 0.445071253 |
| LINC00526 | -0.392752269 | 0.012802604 | 0.136078062 | Down | 1.892701677 |
| LINC00667 | -0.053466077 | 0.464531432 | 0.654918093 | NS | 0.332984895 |
| MIR3976HG | 0.220856931 | 0.580624204 | 0.740040997 | NS | 0.236104864 |
| L3MBTL4-AS1 | 0.282347048 | 0.269134691 | 0.49444608 | NS | 0.570030319 |
| LINC01387 | 0.76589537 | 0.287168452 | NA | NS | 0.541863273 |
| LINC00668 | 0.517139557 | 0.24611688 | 0.47433887 | NS | 0.608858599 |
| GACAT2 | 0.970454068 | 0.077092637 | NA | NS | 1.112987099 |
| NDUFV2-AS1 | 0.094229142 | 0.102351591 | 0.31812203 | NS | 0.989905402 |
| TWSG1-DT | 0.351747789 | 0.088939261 | 0.299278129 | NS | 1.050906485 |
| PPP4R1-AS1 | 0.375576484 | 0.027078491 | 0.185578254 | Up | 1.567375533 |
| LINC02856 | 0.09698982 | 0.748489879 | NA | NS | 0.125814068 |
| LINC01254 | 0.495578213 | 0.301670452 | NA | NS | 0.520467226 |
| LINC01887 | 0.030928646 | 0.896587716 | NA | NS | 0.047407216 |
| LINC01928 | 0.379068845 | 0.496801215 | NA | NS | 0.30381735 |
| LINC01255 | 0.287930943 | 0.515732958 | 0.693487378 | NS | 0.287575114 |
| LINC01882 | 0.025515129 | 0.848218231 | 0.91459011 | NS | 0.071492397 |
| CEP192-DT | 0.149254626 | 0.463500154 | NA | NS | 0.333950117 |
| LDLRAD4-AS1 | 0.385628588 | 0.051130866 | 0.239125809 | NS | 1.291316854 |
| LINC01906 | 1.037332684 | 0.185326259 | NA | NS | 0.732063041 |
| LINC01443 | 0.098238078 | 0.854907531 | NA | NS | 0.068080857 |
| LINC01444 | 0.043116631 | 0.950145694 | NA | NS | 0.022209795 |
| GREB1L-DT | 0.513382081 | 0.409919237 | NA | NS | 0.3873017 |
| MIR133A1HG | 0.065741065 | 0.515784091 | 0.693487378 | NS | 0.287532058 |
| LINC01900 | 0.520420815 | 0.363037693 | NA | NS | 0.440048282 |
| GATA6-AS1 | 0.722590004 | 0.099313723 | NA | NS | 1.002990736 |
| TTC39C-AS1 | 0.061134346 | 0.63212229 | 0.775886882 | NS | 0.199198895 |
| LINC01915 | 0.558826085 | 0.149328468 | 0.375279334 | NS | 0.825857391 |
| LINC01894 | 0.365271637 | 0.454305746 | NA | NS | 0.34265177 |
| LINC01543 | 0.751269991 | 0.086017976 | NA | NS | 1.065410783 |
| AQP4-AS1 | 0.442161317 | 0.281062704 | 0.504182506 | NS | 0.55119678 |
| PCAT18 | 0.852457653 | 0.103997266 | NA | NS | 0.982978079 |
| LINC01908 | 0.848440188 | 0.209086125 | NA | NS | 0.679674785 |
| DSCAS | 0.477700024 | 0.124572813 | NA | NS | 0.904576727 |
| DSG1-AS1 | 0.622437369 | 0.261740277 | NA | NS | 0.582129442 |
| DSG2-AS1 | 0.234367447 | 0.644700918 | NA | NS | 0.190641711 |
| ASXL3-DT | 0.888748863 | 0.166719592 | NA | NS | 0.77801336 |
| COSMOC | 1.243512361 | 0.013225854 | NA | Up | 1.878576267 |
| MIR924HG | 0.054161743 | 0.847506462 | 0.914210023 | NS | 0.071856982 |
| LINC01901 | 0.787097444 | 0.164344791 | NA | NS | 0.784244057 |
| LINC01902 | 0.295036246 | 0.48676074 | 0.67206682 | NS | 0.312684457 |
| LINC01477 | 0.467606371 | 0.532460997 | NA | NS | 0.273712199 |
| KC6 | 0.297462065 | 0.503247724 | 0.684244941 | NS | 0.29821818 |
| LINC00907 | 0.353739608 | 0.111218847 | 0.32972609 | NS | 0.95382161 |
| LINC01478 | -0.022771429 | 0.937941311 | 0.965773397 | NS | 0.027824335 |
| SETBP1-DT | 0.394047405 | 0.0255916 | 0.181174286 | Up | 1.591902566 |
| ST8SIA5-DT | 0.242247425 | 0.148560805 | 0.374706999 | NS | 0.828095757 |
| MIR4527HG | 0.081749673 | 0.878105868 | NA | NS | 0.056453121 |
| LINC02837 | 0.728336223 | 0.223339583 | NA | NS | 0.651034299 |
| SNHG22 | -0.315748251 | 9.76E-04 | 0.051747224 | Down | 3.010620818 |
| LINC01630 | 0.441926019 | 0.26157514 | 0.487753937 | NS | 0.582403533 |
| LINC01917 | 1.003273864 | 0.068825519 | NA | NS | 1.162250508 |
| LINC01919 | 0.234897666 | 0.619250707 | NA | NS | 0.208133489 |
| LINC01929 | 0.546062252 | 0.247003726 | NA | NS | 0.607296496 |
| TCF4-AS1 | 0.205105348 | 0.559260506 | NA | NS | 0.252385848 |
| TCF4-AS2 | 0.168310304 | 0.55721542 | NA | NS | 0.253976873 |
| LINC01415 | 0.12446829 | 0.688529534 | 0.815257617 | NS | 0.162077426 |
| LINC01416 | 0.580953325 | 0.240108222 | NA | NS | 0.619592968 |
| LINC01905 | 0.656126843 | 0.154484899 | NA | NS | 0.811113968 |
| LINC01539 | 0.472943234 | 0.281442194 | 0.504586593 | NS | 0.550610792 |
| WDR7-OT1 | 0.148351947 | 0.504730805 | NA | NS | 0.296940188 |
| LINC-ROR | 0.57259674 | 0.141224245 | 0.365740533 | NS | 0.850090737 |
| LINC01897 | 0.802300257 | 0.190874587 | NA | NS | 0.719251889 |
| MIR122HG | 0.587686764 | 0.175864925 | NA | NS | 0.754820768 |
| MALT1-AS1 | 0.442353355 | 0.03240229 | NA | Up | 1.489424297 |
| LINC01926 | 0.863257778 | 0.074157996 | NA | NS | 1.129842013 |
| LINC01544 | 0.639765332 | 0.162086822 | 0.389682175 | NS | 0.790252293 |
| KDSR-DT | 0.35960715 | 0.034459658 | NA | Up | 1.462689032 |
| LINC01924 | 0.310696182 | 0.482843728 | NA | NS | 0.316193405 |
| LINC00305 | 0.801154819 | 0.071925978 | NA | NS | 1.143114227 |
| LINC01538 | 0.266086484 | 0.628040534 | NA | NS | 0.202012326 |
| LINC01916 | 0.500983234 | 0.48917147 | NA | NS | 0.31053888 |
| DSEL-AS1 | 0.128578817 | 0.819441089 | NA | NS | 0.086482263 |
| LINC01903 | 0.231660167 | 0.693447683 | NA | NS | 0.158986299 |
| LINC01912 | 0.513526165 | 0.375998085 | NA | NS | 0.424814367 |
| LINC01909 | 0.04605378 | 0.888136381 | NA | NS | 0.05152034 |
| LIVAR | 0.30059569 | 0.45534279 | NA | NS | 0.341661536 |
| LINC01910 | -0.06800125 | 0.887524413 | NA | NS | 0.051819692 |
| GTSCR1 | 0.227825058 | 0.275701318 | 0.500286156 | NS | 0.559561158 |
| LINC01541 | 0.446671317 | 0.440709357 | NA | NS | 0.355847728 |
| LINC01899 | 0.320473977 | 0.602524721 | NA | NS | 0.22002513 |
| NETO1-DT | 0.736335778 | 0.016712557 | NA | Up | 1.776957103 |
| LINC02864 | 0.517715783 | 0.2812022 | 0.504325448 | NS | 0.550981286 |
| LINC02582 | 0.646490613 | 0.212514666 | NA | NS | 0.672611092 |
| LINC01922 | 0.43582293 | 0.376340733 | NA | NS | 0.424418773 |
| ZNF407-AS1 | -0.047050817 | 0.601937067 | 0.755704567 | NS | 0.220448912 |
| LINC01898 | 0.823189029 | 0.13143026 | NA | NS | 0.881304633 |
| LINC01893 | 0.409693864 | 0.426056706 | NA | NS | 0.370532595 |
| ZNF516-AS1 | 0.139518662 | 0.213391413 | 0.443180155 | NS | 0.670823062 |
| ZNF516-DT | 0.143187838 | 0.442231526 | 0.636699969 | NS | 0.354350301 |
| LINC00683 | 0.180663711 | 0.478424274 | 0.666030801 | NS | 0.320186794 |
| LINC01927 | 0.357359771 | 0.404545723 | NA | NS | 0.393032386 |
| LINC01879 | 0.372264848 | 0.496319147 | NA | NS | 0.30423897 |
| ZNF236-DT | 0.236022004 | 0.064644241 | 0.26380656 | NS | 1.189470157 |
| LINC01029 | 0.216164041 | 0.647198461 | NA | NS | 0.188962524 |
| RBFADN | 0.422723519 | 0.095139673 | 0.308711929 | NS | 1.021638345 |
| PARD6G-AS1 | 0.385656829 | 0.002352106 | 0.074602988 | Up | 2.628543195 |
| LINC01002 | 0.00604118 | 0.973205574 | 0.984859443 | NS | 0.011795412 |
| MADCAM1-AS1 | 0.624098538 | 0.018122989 | NA | Up | 1.741770183 |
| BSG-AS1 | -0.084042397 | 0.544910706 | 0.713725092 | NS | 0.26367466 |
| LINC01836 | 0.830024922 | 0.137511137 | NA | NS | 0.861662129 |
| CBARP-DT | 1.276404235 | 0.057871394 | NA | NS | 1.237536058 |
| CIRBP-AS1 | 0.034654898 | 0.673893877 | 0.805843649 | NS | 0.17140849 |
| CSNK1G2-AS1 | 0.049331319 | 0.777189679 | 0.870361812 | NS | 0.109472976 |
| LINC01775 | 0.699359416 | 0.247693016 | NA | NS | 0.606086239 |
| GNA15-DT | 0.205467916 | 0.310495286 | 0.530391814 | NS | 0.507944988 |
| CACTIN-AS1 | 0.114630179 | 0.653696963 | NA | NS | 0.184623533 |
| DPP9-AS1 | 0.403399734 | 0.015332556 | 0.147421994 | Up | 1.814385428 |
| MIR7-3HG | 0.800873047 | 0.076223737 | NA | NS | 1.117909763 |
| RANBP3-DT | -0.344922074 | 0.212748927 | 0.442579534 | NS | 0.672132622 |
| ARHGEF18-AS1 | -0.401391569 | 0.221889491 | 0.45062633 | NS | 0.653863266 |
| RAB11B-AS1 | -0.137654678 | 0.316878589 | 0.536370252 | NS | 0.499107104 |
| ZNF426-DT | -0.094676504 | 0.806759599 | NA | NS | 0.093255858 |
| ZNF561-AS1 | 0.070369318 | 0.458127861 | 0.649541527 | NS | 0.339013295 |
| PIN1-DT | 0.454042368 | 0.058056076 | NA | NS | 1.236152322 |
| ILF3-DT | -0.040430469 | 0.642883143 | 0.783905321 | NS | 0.191867962 |
| ZNF433-AS1 | 0.069622981 | 0.295088321 | 0.517117263 | NS | 0.530047978 |
| FARSA-AS1 | -0.263213893 | 0.146195378 | NA | NS | 0.835066357 |
| ADGRL1-AS1 | -0.012496161 | 0.952996693 | NA | NS | 0.020908606 |
| LINC01841 | 0.334974388 | 0.021188724 | 0.168202484 | Up | 1.673895187 |
| LINC01842 | 0.426265304 | 0.340696595 | NA | NS | 0.467632206 |
| ILVBL-AS1 | 0.620061508 | 0.070862014 | NA | NS | 1.149586511 |
| LINC01764 | 0.468756316 | 0.321830192 | NA | NS | 0.492373216 |
| UCA1 | 0.557457199 | 0.051243846 | 0.239263314 | NS | 1.290358285 |
| LINC00661 | 0.602247945 | 0.284320444 | NA | NS | 0.546191912 |
| LINC00905 | 0.149966117 | 0.773940351 | NA | NS | 0.11129251 |
| LINC01855 | 1.104358893 | 0.130140314 | NA | NS | 0.885588149 |
| KLF2-DT | 0.104172588 | 0.513212802 | 0.691490884 | NS | 0.289702519 |
| BISPR | -0.020890092 | 0.791484909 | 0.879547158 | NS | 0.101557362 |
| PGLS-DT | 0.022445301 | 0.824462738 | 0.900078142 | NS | 0.083828968 |
| HOMER3-AS1 | 0.306136958 | 0.47234602 | NA | NS | 0.32573974 |
| LINC00663 | 0.041793789 | 0.721101928 | 0.835389817 | NS | 0.142003343 |
| LINC00664 | 0.375069716 | 0.121368295 | 0.342066258 | NS | 0.915894748 |
| LINC01233 | 0.445192781 | 0.417861891 | NA | NS | 0.378967235 |
| LINC01785 | 0.871066235 | 0.072956944 | NA | NS | 1.136933367 |
| LINC01859 | 0.531771697 | 0.317048309 | NA | NS | 0.498874558 |
| LINC01858 | 0.458241165 | 0.477401377 | NA | NS | 0.321116333 |
| LINC01224 | 0.013400794 | 0.97897535 | NA | NS | 0.009228243 |
| ERVK-28 | 1.000769869 | 0.185724304 | NA | NS | 0.73113126 |
| LINC00662 | -0.036475804 | 0.549165511 | 0.717084181 | NS | 0.260296745 |
| LINC00906 | 0.390209691 | 0.162405806 | 0.390045866 | NS | 0.789398449 |
| LINC01532 | 0.684287293 | 0.131060566 | NA | NS | 0.882527962 |
| UQCRFS1-DT | 0.397519552 | 0.256485957 | NA | NS | 0.590936408 |
| VSTM2B-DT | 0.590270693 | 0.211863482 | NA | NS | 0.673943893 |
| LINC01834 | 0.140979787 | 0.8283701 | NA | NS | 0.081775585 |
| LINC01791 | 0.458994898 | 0.115141647 | 0.333963272 | NS | 0.938767563 |
| TSHZ3-AS1 | 0.50249291 | 0.097083331 | NA | NS | 1.012855329 |
| LINC02841 | 0.329300569 | 0.527252042 | NA | NS | 0.277981729 |
| LINC01837 | 0.557803056 | 0.183239632 | 0.413453601 | NS | 0.736980589 |
| LINC01533 | 0.363126558 | 0.415464326 | NA | NS | 0.381466261 |
| LINC01782 | 0.518371116 | 0.416858745 | NA | NS | 0.380011083 |
| DPY19L3-DT | 0.207102214 | 0.098386028 | 0.313080008 | NS | 1.007066571 |
| NUDT19-DT | -0.247781976 | 0.106262515 | 0.322590685 | NS | 0.973619908 |
| CEBPA-DT | 0.049517264 | 0.701209645 | 0.822826422 | NS | 0.154152119 |
| SUNO1 | 1.149661264 | 0.021913981 | NA | Up | 1.659278711 |
| SCGB1B2P | 0.830938801 | 0.109521687 | NA | NS | 0.960499876 |
| LINC01801 | -0.102804335 | 0.348647423 | 0.562740382 | NS | 0.45761354 |
| LINC00904 | 0.859884003 | 0.108649083 | NA | NS | 0.963973933 |
| LINC01838 | 0.340033857 | 0.54482604 | NA | NS | 0.263742143 |
| ZNF30-AS1 | 0.182817298 | 0.698722089 | NA | NS | 0.155695527 |
| GRAMD1A-AS1 | 0.457189669 | 0.223162638 | NA | NS | 0.651378513 |
| HPN-AS1 | 0.377358309 | 0.355886253 | 0.568903557 | NS | 0.448688787 |
| LINC01531 | -0.03422726 | 0.861290254 | 0.921741561 | NS | 0.064850467 |
| TMEM147-AS1 | 0.268374172 | 0.005650694 | 0.101899364 | Up | 2.247898215 |
| LINC01766 | 0.452985456 | 0.393915111 | NA | NS | 0.404597359 |
| UPK1A-AS1 | 0.435131935 | 0.349209231 | NA | NS | 0.456914285 |
| LINC00665 | 0.283885919 | 0.113909535 | 0.332423088 | NS | 0.943439922 |
| ZNF566-AS1 | 0.204077004 | 0.356291387 | NA | NS | 0.448194677 |
| ZNF529-AS1 | 0.156915734 | 0.030289789 | 0.193469884 | Up | 1.518703747 |
| LINC01534 | 0.120015551 | 0.044090608 | 0.224627854 | Up | 1.355653911 |
| ZNF790-AS1 | 0.153443682 | 0.047244264 | 0.23095054 | Up | 1.325650914 |
| LINC01535 | 0.22122024 | 0.441549884 | 0.636118478 | NS | 0.355020225 |
| ZNF793-AS1 | 0.085468289 | 0.641405031 | 0.782894954 | NS | 0.192867638 |
| ZNF571-AS1 | 0.12995787 | 0.169414175 | 0.397736896 | NS | 0.771050256 |
| SERTAD3-AS1 | 0.229301644 | 0.133226751 | 0.356443815 | NS | 0.875408563 |
| PCAT19 | -0.16272752 | 0.682934326 | NA | NS | 0.165621058 |
| LINC01480 | -0.243704122 | 0.193274612 | NA | NS | 0.71382519 |
| POU2F2-AS1 | -0.108757808 | 0.219298654 | 0.448726211 | NS | 0.658964034 |
| LIPE-AS1 | 0.254326474 | 0.050741477 | 0.238451278 | NS | 1.294636892 |
| PSG8-AS1 | 0.782641537 | 0.106727798 | NA | NS | 0.971722451 |
| ZNF45-AS1 | 0.154678585 | 0.270488095 | 0.495915885 | NS | 0.567851844 |
| ZNF230-DT | -0.039180021 | 0.823585591 | 0.89959256 | NS | 0.08429126 |
| ZNF222-DT | -0.038632623 | 0.948628914 | NA | NS | 0.022903642 |
| CEACAM16-AS1 | 0.814874232 | 0.019333259 | NA | Up | 1.713694933 |
| GEMIN7-AS1 | 0.005679122 | 0.955401286 | 0.975854235 | NS | 0.019814178 |
| DM1-AS | 0.297472852 | 0.404096449 | NA | NS | 0.393514966 |
| IGFL2-AS1 | 0.589802919 | 0.204479088 | NA | NS | 0.6893511 |
| DACT3-AS1 | 0.182074406 | 0.157612221 | 0.385109899 | NS | 0.802410111 |
| NAPA-AS1 | 0.06822132 | 0.633074817 | 0.776534704 | NS | 0.198544962 |
| BICRA-AS1 | 0.152822528 | 0.451398485 | 0.644308389 | NS | 0.345439903 |
| NOP53-AS1 | -0.059568506 | 0.771974003 | NA | NS | 0.112397325 |
| LINC01595 | 0.942454192 | 0.136008062 | NA | NS | 0.866435346 |
| PLA2G4C-AS1 | 0.671958586 | 0.225432274 | NA | NS | 0.646983909 |
| CARD8-AS1 | -0.1255584 | 0.221821292 | 0.450609639 | NS | 0.653996769 |
| ZNF114-AS1 | 0.873831849 | 0.0676533 | NA | NS | 1.169711016 |
| NUCB1-AS1 | 0.424909196 | 0.116438513 | NA | NS | 0.933903351 |
| PTOV1-AS1 | 0.256726299 | 0.012428077 | 0.135215121 | Up | 1.90559605 |
| PTOV1-AS2 | -0.013829649 | 0.932214744 | 0.962763487 | NS | 0.030484033 |
| LINC01872 | 0.358772004 | 0.597151874 | NA | NS | 0.2239152 |
| VSIG10L-AS1 | 0.167148786 | 0.636443203 | NA | NS | 0.196240347 |
| SIGLEC10-AS1 | -0.084925502 | 0.633694073 | 0.776736637 | NS | 0.198120355 |
| LINC01530 | 0.166008404 | 0.376995587 | 0.587104103 | NS | 0.423663733 |
| SPACA6P-AS | 0.3872069 | 0.18456431 | 0.414734144 | NS | 0.733852276 |
| ZNF649-AS1 | 0.088780458 | 0.616546475 | NA | NS | 0.210034181 |
| ZNF350-AS1 | 0.068724624 | 0.329083763 | 0.546650927 | NS | 0.482693546 |
| ZNF528-AS1 | 0.291016854 | 0.008662104 | 0.118708759 | Up | 2.062376616 |
| MYADM-AS1 | 0.415329792 | 0.00685143 | 0.109849098 | Up | 2.164218805 |
| LENG8-AS1 | 0.793766629 | 0.029781437 | NA | Up | 1.526054352 |
| GP6-AS1 | 0.193218691 | 0.481153133 | NA | NS | 0.317716682 |
| DNAAF3-AS1 | 0.437845065 | 0.521586499 | NA | NS | 0.282673659 |
| ZNF628-DT | 0.480809649 | 0.271415869 | NA | NS | 0.566364764 |
| LINC01864 | 0.920600639 | 0.046968874 | NA | Up | 1.328189854 |
| ZSCAN5A-AS1 | 0.316648075 | 0.121924758 | 0.342773783 | NS | 0.913908097 |
| ZNF582-DT | -0.054570878 | 0.616345121 | 0.765484597 | NS | 0.210176037 |
| ZNF667-AS1 | 0.301449316 | 0.102432281 | 0.318159125 | NS | 0.989563155 |
| ZFP28-DT | 0.320392359 | 0.347314582 | NA | NS | 0.459276982 |
| ZNF470-DT | 0.18579161 | 0.091964144 | 0.303838495 | NS | 1.036381465 |
| MIMT1 | 0.476291803 | 0.406462634 | NA | NS | 0.390979373 |
| ZNF460-AS1 | 0.066014206 | 0.46532926 | 0.655571525 | NS | 0.332239638 |
| ZNF8-DT | 0.06801389 | 0.450253332 | 0.643605891 | NS | 0.346543064 |
| ZNF8-ERVK3-1 | -0.090566799 | 0.220843322 | 0.449641695 | NS | 0.655915728 |
| A1BG-AS1 | 0.18768892 | 0.215470053 | 0.444767477 | NS | 0.666613081 |
| ZNF497-AS1 | -0.06561833 | 0.716737164 | NA | NS | 0.144640076 |
| LINC02560 | 1.186306244 | 0.012859972 | NA | Up | 1.89075998 |
| ZNF132-DT | 0.456761687 | 0.167920232 | 0.39601577 | NS | 0.774896975 |
| MZF1-AS1 | -0.030519483 | 0.707800588 | 0.82706671 | NS | 0.150089081 |
| NRSN2-AS1 | 0.131899434 | 0.465878809 | 0.656110672 | NS | 0.331727043 |
| SDCBP2-AS1 | 0.146795419 | 0.069815474 | 0.270315129 | NS | 1.156048309 |
| SIRPG-AS1 | 0.21155912 | 0.123802736 | 0.34482915 | NS | 0.907269756 |
| PDYN-AS1 | 0.333665623 | 0.402998876 | NA | NS | 0.394696165 |
| UBOX5-AS1 | 0.236186359 | 0.232703357 | 0.461082819 | NS | 0.633197352 |
| LINC01730 | 0.154255137 | 0.322687618 | 0.541387141 | NS | 0.491217699 |
| PANK2-AS1 | 0.362019712 | 0.039947914 | NA | Up | 1.398505893 |
| LINC01433 | -0.204462311 | 0.616860325 | NA | NS | 0.209813162 |
| PRNT | 0.374783091 | 0.481092218 | NA | NS | 0.317771668 |
| LINC00658 | 0.255170486 | 0.540231365 | NA | NS | 0.267420205 |
| LINC00654 | 0.102900967 | 0.472939703 | 0.661209028 | NS | 0.325194225 |
| LINC01729 | 0.032450245 | 0.930045011 | NA | NS | 0.031496033 |
| MCM8-AS1 | 0.047853482 | 0.816680059 | NA | NS | 0.087948048 |
| CASC20 | 0.691842313 | 0.106549803 | NA | NS | 0.972447349 |
| LINC01713 | 0.509275583 | 0.337973476 | NA | NS | 0.471117382 |
| LINC01428 | 0.103377172 | 0.847226014 | NA | NS | 0.072000718 |
| LINC01751 | 0.496664295 | 0.394075122 | NA | NS | 0.404420981 |
| LINC01706 | 0.495657578 | 0.394443294 | NA | NS | 0.404015423 |
| PLCB1-IT1 | 0.045032931 | 0.764650278 | 0.863142594 | NS | 0.116537149 |
| LAMP5-AS1 | 0.57008765 | 0.218552116 | NA | NS | 0.660444985 |
| SNAP25-AS1 | 0.48756211 | 0.21737629 | 0.447016923 | NS | 0.662787828 |
| LINC01752 | 0.268691126 | 0.346484413 | NA | NS | 0.460316298 |
| LINC02871 | 0.368251381 | 0.439425668 | NA | NS | 0.357114578 |
| LINC00687 | 0.281827837 | 0.586479974 | NA | NS | 0.231746813 |
| LINC01722 | 0.676970388 | 0.136511747 | NA | NS | 0.864829974 |
| LINC01723 | 0.361216154 | 0.438546464 | NA | NS | 0.357984387 |
| ISM1-AS1 | -0.362547133 | 0.057497611 | 0.251193798 | NS | 1.240350198 |
| MACROD2-IT1 | 0.360054444 | 0.508453117 | NA | NS | 0.293749086 |
| MACROD2-AS1 | 0.492327146 | 0.406562948 | NA | NS | 0.390872203 |
| LINC00851 | 0.447308717 | 0.443572727 | NA | NS | 0.353035165 |
| DTD1-AS1 | 0.14224898 | 0.333644504 | 0.550196316 | NS | 0.476716025 |
| LINC00652 | 0.074356994 | 0.634872264 | 0.777365515 | NS | 0.197313646 |
| LINC00653 | -0.129926066 | 0.178909869 | 0.40892154 | NS | 0.747365701 |
| SCP2D1-AS1 | 0.266997102 | 0.661879592 | NA | NS | 0.179221009 |
| SLC24A3-AS1 | 0.137292889 | 0.508857109 | 0.688232771 | NS | 0.293404154 |
| CFAP61-AS1 | 0.994239177 | 0.155838422 | NA | NS | 0.807325457 |
| LINC00237 | 0.28839493 | 0.253450468 | NA | NS | 0.596106903 |
| KIZ-AS1 | 0.119176042 | 0.302732487 | 0.524208333 | NS | 0.518940971 |
| LINC01727 | 0.096359207 | 0.497491697 | 0.679709754 | NS | 0.303214163 |
| LINC01726 | 0.158652508 | 0.533516752 | NA | NS | 0.27285194 |
| LINC01432 | 0.714456866 | 0.321866808 | NA | NS | 0.492323807 |
| LINC01427 | 0.515771711 | 0.31420732 | NA | NS | 0.502783701 |
| LINC00261 | 0.417940259 | 0.388822181 | NA | NS | 0.410248968 |
| LNCNEF | 0.020455845 | 0.970772718 | NA | NS | 0.012882437 |
| LINC01747 | 0.558238943 | 0.203885133 | NA | NS | 0.690614441 |
| LINC00656 | 0.02132865 | 0.928039601 | NA | NS | 0.032433491 |
| NXT1-AS1 | -0.190045673 | 0.259992402 | NA | NS | 0.585039344 |
| LINC01431 | 0.291998218 | 0.108223083 | 0.325834237 | NS | 0.9656801 |
| LINC01721 | 0.312316828 | 0.483086495 | 0.669197484 | NS | 0.315975103 |
| ZNF337-AS1 | 0.014072262 | 0.871882389 | 0.927658559 | NS | 0.059542094 |
| FAM182B | 0.529295609 | 0.022104197 | 0.170333216 | Up | 1.655525258 |
| LINC01733 | 0.149774862 | 0.75933012 | NA | NS | 0.119569373 |
| FAM182A | 0.696481876 | 0.021973734 | NA | Up | 1.658096133 |
| MIR663AHG | 0.422307508 | 0.088405107 | 0.298422198 | NS | 1.053522647 |
| FAM242B | 0.991018806 | 0.104177287 | NA | NS | 0.982226956 |
| LINC01597 | 0.391220295 | 0.12876299 | 0.350578636 | NS | 0.890208948 |
| FAM242A | 0.576302865 | 0.243582837 | NA | NS | 0.613353316 |
| LINC00028 | 0.952439262 | 0.121060977 | NA | NS | 0.916995826 |
| HM13-IT1 | 0.335742184 | 0.055038492 | 0.247689435 | NS | 1.259333477 |
| HM13-AS1 | 0.246621122 | 0.283720047 | NA | NS | 0.547109977 |
| BCL2L1-AS1 | 0.015181186 | 0.90650222 | 0.948194284 | NS | 0.042631128 |
| ABALON | 0.079921391 | 0.51286421 | 0.691208607 | NS | 0.289997607 |
| NOL4L-DT | 0.616285896 | 0.072805942 | NA | NS | 1.137833177 |
| ZNF341-AS1 | 0.518920624 | 0.148649989 | NA | NS | 0.827835119 |
| RALY-AS1 | 0.25051728 | 0.008754941 | 0.118782974 | Up | 2.057746775 |
| ITCH-AS1 | 0.141786921 | 0.388378502 | NA | NS | 0.410744818 |
| ITCH-IT1 | 0.136217168 | 0.306936874 | 0.527718843 | NS | 0.512950935 |
| FAM83C-AS1 | 0.383471924 | 0.497242589 | NA | NS | 0.303431681 |
| GDF5-AS1 | 0.240972705 | 0.691506374 | NA | NS | 0.160203813 |
| CEP250-AS1 | 0.135386888 | 0.192255928 | 0.42102173 | NS | 0.716120261 |
| NORAD | -0.106683388 | 0.116460403 | 0.336001027 | NS | 0.93382171 |
| DLGAP4-AS1 | 0.098658104 | 0.508198233 | 0.687821019 | NS | 0.293966849 |
| LINC01746 | 1.282062652 | 0.040025781 | NA | Up | 1.397660182 |
| LINC00489 | 0.326539635 | 0.6355685 | NA | NS | 0.196837635 |
| SNHG17 | 0.213372555 | 0.03623523 | 0.208201842 | Up | 1.440868973 |
| SNHG11 | 0.07089076 | 0.426591428 | 0.624754992 | NS | 0.369987875 |
| LINC01734 | 0.29715596 | 0.408235711 | NA | NS | 0.389089008 |
| LINC01370 | 0.521742085 | 0.301700282 | NA | NS | 0.520424284 |
| PLCG1-AS1 | 0.356701427 | 1.33E-04 | 0.018210843 | Up | 3.876826889 |
| PTPRT-AS1 | 0.713459052 | 0.177630213 | NA | NS | 0.750483163 |
| LINC01728 | 0.270802499 | 0.739952808 | NA | NS | 0.130795977 |
| OSER1-DT | 0.037050019 | 0.614331017 | 0.764059058 | NS | 0.211597557 |
| HNF4A-AS1 | 0.781045938 | 0.106973938 | NA | NS | 0.970722014 |
| LINC01620 | 0.344654038 | 0.425131749 | NA | NS | 0.37147646 |
| KCNK15-AS1 | 0.589505974 | 0.158323291 | NA | NS | 0.80045519 |
| STK4-AS1 | 0.134284637 | 0.608757972 | NA | NS | 0.215555339 |
| SLC12A5-AS1 | 0.785727292 | 0.038060729 | NA | Up | 1.419522898 |
| TP53RK-DT | 0.939953667 | 0.009010288 | NA | Up | 2.045261321 |
| LINC01754 | 0.264602642 | 0.310580188 | NA | NS | 0.507826251 |
| LINC01522 | 0.640958809 | 0.13676758 | NA | NS | 0.864016837 |
| LINC01523 | 0.600729623 | 0.350503172 | NA | NS | 0.455308048 |
| LINC00494 | 0.052783985 | 0.707166536 | 0.826724247 | NS | 0.150478299 |
| CSE1L-AS1 | 0.392961443 | 0.274592885 | NA | NS | 0.561310719 |
| ZFAS1 | 0.151333153 | 0.08720774 | 0.296703312 | NS | 1.059444967 |
| TRERNA1 | 0.888118812 | 0.07660892 | NA | NS | 1.115720661 |
| LINC01275 | 0.60988862 | 0.163129179 | NA | NS | 0.78746835 |
| LINC01273 | 0.657221441 | 0.191776609 | NA | NS | 0.717204366 |
| CEBPB-AS1 | 0.216486522 | 0.094856776 | 0.308099543 | NS | 1.022931641 |
| PELATON | 0.142099878 | 0.305486499 | 0.526667648 | NS | 0.515007978 |
| LINC01270 | 0.018853881 | 0.921205352 | 0.956539942 | NS | 0.035643548 |
| LINC01271 | 0.206969771 | 0.389943727 | 0.597077704 | NS | 0.408998062 |
| RIPOR3-AS1 | 0.311517067 | 0.59060298 | NA | NS | 0.228704366 |
| ADNP-AS1 | 0.047183209 | 0.575006536 | 0.736180663 | NS | 0.240327219 |
| LINC01429 | 0.731818245 | 0.142797783 | NA | NS | 0.845278534 |
| LINC01524 | 0.342597225 | 0.30249729 | 0.523977057 | NS | 0.519278512 |
| LINC01440 | 0.276880192 | 0.519139411 | 0.69601515 | NS | 0.284716 |
| LINC01441 | 0.757113093 | 0.144164233 | NA | NS | 0.841142474 |
| LINC01716 | 0.574998692 | 0.383405251 | NA | NS | 0.416341943 |
| BMP7-AS1 | 0.453708955 | 0.600677913 | NA | NS | 0.221358336 |
| RBM38-AS1 | 0.014613494 | 0.846826132 | 0.913820481 | NS | 0.072205749 |
| NKILA | 0.484769243 | 0.019228493 | NA | Up | 1.716054759 |
| LINC01742 | 1.18853983 | 0.19614422 | NA | NS | 0.707424484 |
| APCDD1L-DT | 0.497034544 | 0.100916472 | 0.316384536 | NS | 0.996037942 |
| LINC01711 | 0.871360647 | 0.260586271 | NA | NS | 0.584048469 |
| GNAS-AS1 | 0.20772883 | 0.401368502 | NA | NS | 0.396456712 |
| PHACTR3-AS1 | 0.349880454 | 0.531665513 | NA | NS | 0.27436151 |
| LINC02910 | 0.128268292 | 0.173284238 | 0.40231957 | NS | 0.761240939 |
| MIR646HG | 0.256307114 | 0.028093757 | 0.187669577 | Up | 1.551390177 |
| LINC01718 | 0.374365121 | 0.531789593 | NA | NS | 0.274260166 |
| LAMA5-AS1 | 1.270648106 | 0.005053671 | NA | Up | 2.296393018 |
| MIR1-1HG-AS1 | 0.800333371 | 0.114363829 | NA | NS | 0.941711312 |
| MIR1-1HG | 1.020781028 | 0.091886784 | NA | NS | 1.036746946 |
| SLCO4A1-AS1 | 0.859194649 | 0.120473865 | NA | NS | 0.919107157 |
| LINC00686 | 0.870350054 | 0.262232078 | NA | NS | 0.581314183 |
| LINC00659 | 0.069617708 | 0.643681263 | 0.784502596 | NS | 0.191329133 |
| OGFR-AS1 | 0.100080095 | 0.496185144 | 0.678912185 | NS | 0.304356243 |
| LINC01749 | 0.839291274 | 0.095444509 | NA | NS | 1.020249051 |
| LINC00029 | 1.092540794 | 0.041899616 | NA | Up | 1.377789959 |
| LINC01056 | 1.046207294 | 0.106572749 | NA | NS | 0.972353832 |
| HAR1B | 0.770065417 | 0.010476264 | NA | Up | 1.979793551 |
| HAR1A | -0.160943953 | 0.282372425 | 0.5053027 | NS | 0.549177716 |
| FLJ16779 | 0.661298566 | 0.14262013 | 0.36732856 | NS | 0.845819174 |
| MHENCR | -0.047789226 | 0.694950183 | 0.819090581 | NS | 0.158046327 |
| ZBTB46-AS1 | 0.923314074 | 0.221979144 | NA | NS | 0.653687829 |
| UCKL1-AS1 | 0.02386785 | 0.878654436 | 0.931684267 | NS | 0.056181894 |
| LINC00266-1 | 0.112484849 | 0.568747423 | NA | NS | 0.245080558 |
| LINC01666 | 0.280945664 | 0.542870565 | NA | NS | 0.265303705 |
| LINC01674 | 0.492011347 | 0.38037732 | NA | NS | 0.419785385 |
| SAMSN1-AS1 | 0.601811275 | 0.262901257 | NA | NS | 0.580207337 |
| LINC02246 | 0.144242206 | 0.565290738 | NA | NS | 0.24772813 |
| MIR99AHG | 0.366937883 | 0.205860509 | 0.435885316 | NS | 0.686426957 |
| LINC01549 | 0.854857523 | 0.118916782 | NA | NS | 0.92475685 |
| C21orf91-OT1 | 0.272483405 | 0.022233332 | 0.170639329 | Up | 1.652995456 |
| CHODL-AS1 | 0.130041742 | 0.515260432 | 0.693278651 | NS | 0.287973207 |
| MIR548XHG | 0.686493295 | 0.187301474 | NA | NS | 0.727458805 |
| LINC01683 | 0.477512323 | 0.458767567 | NA | NS | 0.338407292 |
| LINC02573 | 0.383025793 | 0.277197928 | NA | NS | 0.557210021 |
| LINC00320 | 0.413465169 | 0.368445645 | 0.580260086 | NS | 0.433626572 |
| LINC00317 | 0.781971728 | 0.272942339 | NA | NS | 0.563929092 |
| LINC01425 | 0.92865185 | 0.162230136 | NA | NS | 0.789868469 |
| LINC01687 | 0.207468817 | 0.690083586 | NA | NS | 0.161098302 |
| D21S2088E | 0.011601853 | 0.98042065 | 0.988361376 | NS | 0.00858755 |
| LINC01689 | 0.316358814 | 0.597303092 | NA | NS | 0.223805237 |
| LINC01684 | 0.049894744 | 0.576260539 | 0.737250369 | NS | 0.239381119 |
| LINC01692 | 0.719677513 | 0.219072105 | NA | NS | 0.659412918 |
| LINC00158 | 0.270401736 | 0.385581916 | 0.593733412 | NS | 0.413883343 |
| MIR155HG | -0.103255018 | 0.355490969 | 0.568656434 | NS | 0.449171428 |
| APP-DT | 0.597359959 | 0.250061332 | NA | NS | 0.60195346 |
| CYYR1-AS1 | 0.299109627 | 0.322551353 | NA | NS | 0.491401132 |
| LINC01673 | 0.252867719 | 0.672276388 | NA | NS | 0.172452142 |
| LINC00113 | -0.055643395 | 0.939715111 | NA | NS | 0.027003789 |
| LINC01697 | 0.419691944 | 0.339800665 | NA | NS | 0.468775775 |
| LINC01695 | 0.601704211 | 0.149187039 | 0.375271684 | NS | 0.826268906 |
| LINC00161 | 0.081565352 | 0.649372413 | 0.78854332 | NS | 0.187506165 |
| LINC00189 | 0.177996029 | 0.344777217 | 0.559649007 | NS | 0.46246144 |
| BACH1-IT1 | 0.268971602 | 0.009802802 | 0.124655065 | Up | 2.008649769 |
| BACH1-AS1 | 0.265014941 | 0.06672877 | 0.266279415 | NS | 1.175686881 |
| BACH1-IT2 | 0.161127428 | 0.207406695 | 0.437312072 | NS | 0.683177228 |
| BACH1-IT3 | 0.180482997 | 0.261906997 | 0.488134281 | NS | 0.581852899 |
| GRIK1-AS1 | 0.660443477 | 0.023925045 | 0.175295276 | Up | 1.621147231 |
| LINC00307 | 0.903190555 | 0.142051359 | NA | NS | 0.847554606 |
| LINC00159 | 0.573604073 | 0.172449903 | NA | NS | 0.763337046 |
| MIS18A-AS1 | 0.145188394 | 0.435020043 | NA | NS | 0.361490733 |
| MRAP-AS1 | 0.296878848 | 0.302009894 | NA | NS | 0.519978829 |
| URB1-AS1 | -0.278507058 | 0.083484395 | 0.290969351 | NS | 1.078394697 |
| PAXBP1-AS1 | 0.136716361 | 0.031922182 | 0.198125154 | Up | 1.495907427 |
| C21orf62-AS1 | 0.228301312 | 0.098569073 | 0.31333179 | NS | 1.006259327 |
| LINC01690 | 0.439152382 | 0.226510605 | NA | NS | 0.64491146 |
| LINC00945 | 0.282377636 | 0.099962293 | 0.314939208 | NS | 1.000163792 |
| LINC01548 | 0.401214536 | 0.410437761 | NA | NS | 0.386752691 |
| IL10RB-DT | 0.055926191 | 0.507843395 | 0.687641579 | NS | 0.294270192 |
| LINC00649 | 0.083905093 | 0.343214281 | 0.558338789 | NS | 0.464434649 |
| LINC00310 | 0.093061727 | 0.553835332 | 0.720760131 | NS | 0.256619341 |
| LINC00160 | 0.886634234 | 0.096627412 | NA | NS | 1.014899653 |
| LINC01426 | 0.251022478 | 0.361020498 | 0.573615336 | NS | 0.442468139 |
| LINC01436 | 0.318743441 | 0.647947919 | NA | NS | 0.1884599 |
| SETD4-AS1 | 0.820326772 | 0.104302687 | NA | NS | 0.981704502 |
| CBR3-AS1 | 0.134439261 | 0.276640097 | 0.500969068 | NS | 0.558084871 |
| HLCS-IT1 | 0.059196522 | 0.76627083 | 0.863936127 | NS | 0.115617707 |
| TTC3-AS1 | 0.231168897 | 0.087047742 | 0.296617673 | NS | 1.060242491 |
| DSCR9 | 0.086132873 | 0.46896254 | 0.658139597 | NS | 0.328861847 |
| KCNJ6-AS1 | 0.738570063 | 0.333919926 | NA | NS | 0.476357664 |
| DSCR4 | 0.393615654 | 0.444738775 | NA | NS | 0.351895005 |
| DSCR8 | 0.297696523 | 0.645788551 | NA | NS | 0.189909659 |
| DSCR10 | 0.398072059 | 0.591068063 | NA | NS | 0.228362506 |
| LINC01423 | 0.661732215 | 0.099219309 | NA | NS | 1.003403803 |
| LINC00114 | 0.612412002 | 0.182456335 | NA | NS | 0.738841053 |
| LINC01700 | 0.108405643 | 0.819208188 | NA | NS | 0.086605715 |
| BRWD1-IT1 | 0.293814946 | 0.062303301 | NA | NS | 1.205488944 |
| BRWD1-AS2 | 0.04556107 | 0.781808583 | 0.873857226 | NS | 0.106899566 |
| BRWD1-AS1 | 0.271161835 | 0.275764121 | NA | NS | 0.55946224 |
| B3GALT5-AS1 | 0.747843651 | 0.079077221 | NA | NS | 1.101948602 |
| DSCAM-AS1 | 0.834187072 | 0.12610035 | NA | NS | 0.899283708 |
| DSCAM-IT1 | 0.853335736 | 0.165927846 | NA | NS | 0.780080723 |
| LINC00323 | 0.531966176 | 0.167675224 | NA | NS | 0.775531105 |
| PLAC4 | 0.411812916 | 0.018301019 | 0.157331058 | Up | 1.737524727 |
| PCSEAT | 0.177001799 | 0.721346971 | NA | NS | 0.141855788 |
| LINC00111 | 0.709010368 | 0.33323569 | NA | NS | 0.477248491 |
| LINC00479 | 0.718696828 | 0.085233521 | NA | NS | 1.069389572 |
| LINC00112 | 0.708153248 | 0.213975258 | NA | NS | 0.669636442 |
| ZNF295-AS1 | 0.22358981 | 0.152506235 | 0.379146526 | NS | 0.8167124 |
| UMODL1-AS1 | -0.208570538 | 0.496699155 | NA | NS | 0.303906579 |
| LINC01671 | 0.23154238 | 0.528766266 | NA | NS | 0.27673626 |
| PDE9A-AS1 | 0.490903853 | 0.11872573 | NA | NS | 0.92545515 |
| LINC01668 | 0.686023256 | 0.207619827 | NA | NS | 0.682731174 |
| LINC00322 | 0.446978569 | 0.442333051 | NA | NS | 0.354250609 |
| LINC01679 | 0.375249768 | 0.152303757 | 0.378947167 | NS | 0.817289383 |
| LINC00319 | 1.074397949 | 0.328889625 | NA | NS | 0.482949826 |
| LINC00313 | 0.108779109 | 0.85984751 | NA | NS | 0.065578562 |
| AATBC | -0.066677082 | 0.54626899 | 0.714753418 | NS | 0.262593452 |
| LINC01678 | 0.309603802 | 0.199131134 | NA | NS | 0.700860834 |
| TRPM2-AS | 0.644182465 | 0.04599991 | NA | Up | 1.337243022 |
| LRRC3-DT | 0.027047904 | 0.968402722 | NA | NS | 0.013943998 |
| LINC02575 | 0.355625827 | 0.295283878 | NA | NS | 0.529760264 |
| TSPEAR-AS1 | -0.230295552 | 0.398192499 | 0.603175118 | NS | 0.399906925 |
| TSPEAR-AS2 | 0.052218631 | 0.836864212 | 0.908377847 | NS | 0.077345004 |
| LINC01424 | 0.019666255 | 0.86043167 | 0.921167077 | NS | 0.065283613 |
| ITGB2-AS1 | 0.04526563 | 0.562443958 | 0.726800854 | NS | 0.249920744 |
| LINC01547 | 0.06910629 | 0.417879973 | 0.618692258 | NS | 0.378948442 |
| LINC00163 | 0.984477818 | 0.048362196 | NA | Up | 1.31549399 |
| LINC00165 | 1.169977782 | 0.051062905 | NA | NS | 1.291894484 |
| PICSAR | 0.810772571 | 0.188011617 | NA | NS | 0.725815316 |
| LINC00334 | 0.0410829 | 0.860580998 | 0.921283879 | NS | 0.065208247 |
| LINC00205 | -0.022536845 | 0.693476972 | 0.817901411 | NS | 0.158967956 |
| LINC00315 | 0.369073732 | 0.230519366 | NA | NS | 0.637292584 |
| LINC00316 | 0.213342346 | 0.774789452 | NA | NS | 0.1108163 |
| COL18A1-AS2 | 1.304409974 | 0.03895928 | NA | Up | 1.409389083 |
| COL18A1-AS1 | 1.052429272 | 0.036038989 | NA | Up | 1.4432274 |
| LINC01694 | 0.623657125 | 0.04230109 | 0.220829623 | Up | 1.373648444 |
| PCBP3-AS1 | 0.495240794 | 0.110194274 | NA | NS | 0.957840971 |
| MCM3AP-AS1 | 0.138122896 | 0.070996087 | 0.272080385 | NS | 1.148765588 |
| DIP2A-IT1 | 0.397109895 | 0.027445229 | 0.186246285 | Up | 1.561533135 |
| POTEH-AS1 | 0.262606684 | 0.64820413 | NA | NS | 0.188288206 |
| PSLNR | 0.221612469 | 0.707354411 | NA | NS | 0.150362934 |
| DUXAP8 | 0.493621128 | 0.182687378 | 0.412858331 | NS | 0.738291457 |
| ANKRD62P1-PARP4P3 | 0.542544229 | 0.227494463 | NA | NS | 0.64302917 |
| LINC01665 | 0.981903362 | 0.112466036 | NA | NS | 0.948978614 |
| LINC01664 | -0.06645434 | 0.764472182 | NA | NS | 0.116638313 |
| HDHD5-AS1 | 0.522062845 | 0.045063931 | NA | Up | 1.346170929 |
| CECR3 | 0.642439504 | 0.137572462 | NA | NS | 0.861468489 |
| LINC00528 | 0.007051856 | 0.930526235 | 0.962031051 | NS | 0.031271378 |
| LINC01634 | 0.421413202 | 0.447511009 | NA | NS | 0.349196276 |
| FAM230D | 0.825259306 | 0.186254924 | NA | NS | 0.729892237 |
| FAM230J | 1.219237365 | 0.047146497 | NA | Up | 1.326550569 |
| FAM230A | 0.815932748 | 0.324346125 | NA | NS | 0.488991286 |
| DGCR5 | 0.466661621 | 0.045282771 | 0.227404155 | Up | 1.344067006 |
| DGCR11 | 0.106366356 | 0.230406264 | 0.458716715 | NS | 0.637505717 |
| LINC01311 | 0.085239941 | 0.493862657 | 0.677407901 | NS | 0.306393812 |
| LINC00895 | 0.680938792 | 0.237587196 | NA | NS | 0.624176969 |
| LINC02891 | 0.581387795 | 0.217613015 | NA | NS | 0.662315133 |
| LINC00896 | 0.311772378 | 0.014719505 | 0.145429726 | Up | 1.832106793 |
| FAM230G | 0.921673352 | 0.04811991 | NA | Up | 1.317675189 |
| LINC01637 | 0.06340545 | 0.749387358 | NA | NS | 0.125293638 |
| THAP7-AS1 | 0.23710856 | 0.073766906 | 0.275957151 | NS | 1.132138432 |
| TUBA3FP | 0.319565878 | 0.194377764 | NA | NS | 0.711353418 |
| FAM230B | 0.484211692 | 0.322923863 | NA | NS | 0.490899862 |
| FAM230H | 1.676885193 | 0.005777933 | NA | Up | 2.238227499 |
| PPM1F-AS1 | 0.140038704 | 0.12285999 | 0.343683826 | NS | 0.910589525 |
| LINC02556 | 0.273641021 | 0.584200265 | NA | NS | 0.23343825 |
| PCAT14 | 0.581736136 | 0.260086691 | NA | NS | 0.584881871 |
| GUSBP11 | -0.031115555 | 0.753154941 | 0.855296001 | NS | 0.123115671 |
| ADORA2A-AS1 | 0.111873011 | 0.196296372 | 0.426063609 | NS | 0.707087728 |
| BCRP3 | 0.100743447 | 0.805302651 | NA | NS | 0.094040871 |
| KIAA1671-AS1 | 0.15041516 | 0.614666471 | NA | NS | 0.211360476 |
| MYO18B-AS1 | 0.355264349 | 0.36002326 | 0.572765303 | NS | 0.443669439 |
| SEZ6L-AS1 | 1.106310436 | 0.024593809 | NA | Up | 1.60917421 |
| TFIP11-DT | -0.023614237 | 0.859311926 | 0.920441595 | NS | 0.065849161 |
| MIAT | -0.2299989 | 0.039248956 | 0.214616748 | Down | 1.406171886 |
| MIATNB | 0.037313547 | 0.590094496 | 0.747557481 | NS | 0.229078437 |
| LINC01422 | 0.155452364 | 0.19175457 | 0.420934764 | NS | 0.717254276 |
| LINC01638 | 0.54131638 | 0.29403505 | NA | NS | 0.531600897 |
| LINC02554 | 0.514486397 | 0.283086972 | 0.505990621 | NS | 0.548080117 |
| TTC28-AS1 | 0.022935469 | 0.693674057 | 0.818049601 | NS | 0.158844548 |
| ZNRF3-IT1 | 0.444425411 | 0.309077116 | NA | NS | 0.509933148 |
| ZNRF3-AS1 | 0.376993976 | 0.321999635 | NA | NS | 0.492144621 |
| RFPL1S | 0.306789588 | 0.197876053 | 0.427645311 | NS | 0.703606761 |
| HORMAD2-AS1 | 0.583874807 | 0.014179576 | NA | Up | 1.848336755 |
| LIF-AS1 | 0.57187037 | 0.401359662 | NA | NS | 0.396466277 |
| LIF-AS2 | 1.139900837 | 0.0650379 | NA | NS | 1.186833489 |
| MORC2-AS1 | 0.098064873 | 0.429912726 | 0.627284739 | NS | 0.366619699 |
| PIK3IP1-DT | 0.115757515 | 0.273592872 | 0.498712509 | NS | 0.562895221 |
| LINC01521 | 0.168423895 | 0.140790191 | 0.365248766 | NS | 0.851427603 |
| YWHAH-AS1 | 0.267844588 | 0.207551676 | 0.437401068 | NS | 0.682873755 |
| LINC02558 | 0.717088703 | 0.186554479 | NA | NS | 0.729194319 |
| SLC5A4-AS1 | -0.169164662 | 0.456073658 | 0.647872405 | NS | 0.340965011 |
| RFPL3S | 0.436932532 | 0.077092635 | 0.281454506 | NS | 1.112987111 |
| SYN3-AS1 | -0.298135968 | 0.553573565 | NA | NS | 0.256824657 |
| LINC01640 | 0.742241166 | 0.058548517 | NA | NS | 1.2324841 |
| LARGE-IT1 | 0.292621587 | 0.619535106 | NA | NS | 0.207934079 |
| LARGE-AS1 | 0.396886511 | 0.059045007 | 0.253419931 | NS | 1.228816821 |
| LINC01643 | 0.269322606 | 0.57565914 | NA | NS | 0.239834596 |
| LINC02885 | 0.373410847 | 0.414532502 | NA | NS | 0.382441413 |
| NCF4-AS1 | 0.393929681 | 0.060580342 | NA | NS | 1.217668279 |
| LL22NC01-81G9.3 | 0.678576992 | 0.183827798 | NA | NS | 0.735588815 |
| ELFN2 | 0.805452651 | 0.027314413 | 0.185919029 | Up | 1.563608135 |
| PDXP-DT | 0.071373762 | 0.497207226 | 0.679630648 | NS | 0.303462568 |
| MGAT3-AS1 | 0.050967131 | 0.873240928 | NA | NS | 0.058865917 |
| TNRC6B-DT | -0.403313109 | 0.078731236 | NA | NS | 1.103852932 |
| SGSM3-AS1 | 0.257653701 | 0.04576411 | 0.228356747 | Up | 1.33947498 |
| MRTFA-AS1 | 0.243673236 | 0.116793861 | 0.336666205 | NS | 0.932579985 |
| EP300-AS1 | 0.465017909 | 0.002043539 | 0.071126352 | Up | 2.689616984 |
| L3MBTL2-AS1 | 0.082455812 | 0.508217287 | 0.687821019 | NS | 0.293950567 |
| SREBF2-AS1 | 0.134752157 | 0.222664004 | 0.451412976 | NS | 0.652349985 |
| NDUFA6-DT | -0.333860983 | 0.046706648 | 0.2299699 | Down | 1.3306213 |
| OGFRP1 | 0.129063943 | 0.141265927 | 0.365773968 | NS | 0.849962576 |
| LINC01315 | 0.997427986 | 0.008102584 | NA | Up | 2.091376432 |
| TTLL1-AS1 | 0.077995815 | 0.720090261 | NA | NS | 0.142613063 |
| SCUBE1-AS2 | 0.190377774 | 0.771581336 | NA | NS | 0.112618286 |
| SCUBE1-AS1 | 0.587653669 | 0.320814769 | NA | NS | 0.493745646 |
| LINC01639 | 0.860258256 | 0.189156439 | NA | NS | 0.72317887 |
| EFCAB6-AS1 | 0.76964228 | 0.094514613 | NA | NS | 1.024501038 |
| EFCAB6-DT | 0.125744163 | 0.335179768 | 0.551576276 | NS | 0.474722205 |
| LINC01656 | 0.483477715 | 0.374752021 | NA | NS | 0.426256016 |
| LINC00207 | 1.047581479 | 0.084461062 | NA | NS | 1.073343465 |
| LINC00229 | 1.066564737 | 0.039995202 | NA | Up | 1.397992101 |
| NUP50-DT | -0.066429002 | 0.397693404 | 0.602817813 | NS | 0.400451612 |
| LINC01589 | 0.372543391 | 0.576399643 | NA | NS | 0.239276296 |
| LINC00899 | 0.028669344 | 0.715926333 | 0.832021589 | NS | 0.145131663 |
| PRR34 | 0.027392212 | 0.786600542 | 0.876842327 | NS | 0.104245759 |
| PRR34-AS1 | -0.12551193 | 0.095550949 | 0.309119979 | NS | 1.019764995 |
| MIRLET7BHG | 0.126331411 | 0.249019214 | 0.476376043 | NS | 0.603767142 |
| GTSE1-DT | 0.104376523 | 0.385993917 | 0.594088219 | NS | 0.413419539 |
| TBC1D22A-DT | -0.027812356 | 0.871536015 | 0.927462377 | NS | 0.059714661 |
| TBC1D22A-AS1 | -0.139108238 | 0.306714422 | 0.527638781 | NS | 0.513265803 |
| LINC01644 | 0.111915041 | 0.667061248 | 0.800851158 | NS | 0.175834289 |
| LINC00898 | 0.773220609 | 0.086027259 | NA | NS | 1.065363914 |
| LINC01310 | 0.674940179 | 0.148933577 | NA | NS | 0.827007379 |
| MIR3667HG | -0.231207767 | 0.030030129 | 0.193133954 | Down | 1.522442805 |
| KLHDC7B-DT | 0.057913843 | 0.729496424 | 0.840233084 | NS | 0.136976833 |
| CHKB-DT | 0.036844716 | 0.679332817 | 0.808800175 | NS | 0.167917405 |
| LINC01546 | 0.597048596 | 0.258146329 | NA | NS | 0.588134047 |
| PRKX-AS1 | 0.007212889 | 0.977744357 | NA | NS | 0.009774682 |
| WWC3-AS1 | 0.211089621 | 0.239365041 | 0.467622466 | NS | 0.620939277 |
| TLR8-AS1 | 0.296580699 | 0.040354873 | 0.216644936 | Up | 1.394104017 |
| LINC02154 | 0.057157463 | 0.918126069 | NA | NS | 0.037097681 |
| GS1-600G8.3 | 0.668971317 | 0.140893785 | NA | NS | 0.851108164 |
| LINC01203 | 0.226407445 | 0.538649773 | 0.709653107 | NS | 0.268693519 |
| GS1-594A7.3 | 0.358864968 | 0.575139038 | NA | NS | 0.240227153 |
| NHS-AS1 | 0.246251971 | 0.372181609 | NA | NS | 0.429245091 |
| LINC01456 | 0.037740312 | 0.954560426 | NA | NS | 0.020196575 |
| PPEF1-AS1 | 0.595949264 | 0.288016989 | NA | NS | 0.540581894 |
| PHKA2-AS1 | 0.374001865 | 0.115839428 | NA | NS | 0.936143596 |
| EIF1AX-AS1 | 0.065508686 | 0.619076185 | 0.767087744 | NS | 0.208255903 |
| PHEX-AS1 | 0.633412976 | 0.111453153 | NA | NS | 0.952907641 |
| PTCHD1-AS | 0.88225421 | 0.027662417 | NA | Up | 1.558109876 |
| ZFX-AS1 | 0.070355889 | 0.761639235 | NA | NS | 0.118250692 |
| PCYT1B-AS1 | 0.340952274 | 0.631432701 | NA | NS | 0.199672931 |
| GK-IT1 | 0.276225269 | 0.074870667 | 0.277772235 | NS | 1.125688301 |
| GK-AS1 | 0.2466896 | 0.087325396 | 0.296859543 | NS | 1.058859437 |
| DMD-AS3 | 0.477011827 | 0.533958078 | NA | NS | 0.272492839 |
| LINC01281 | 0.097122565 | 0.62140595 | 0.768560732 | NS | 0.206624593 |
| LINC01282 | 0.425234576 | 0.468976022 | NA | NS | 0.328849361 |
| LINC01283 | 1.062559353 | 0.055821209 | NA | NS | 1.253200758 |
| MED14OS | -0.023333515 | 0.92540617 | NA | NS | 0.033667609 |
| LINC02601 | 0.491749048 | 0.251211084 | NA | NS | 0.599961202 |
| CASK-AS1 | 0.050197909 | 0.79570403 | NA | NS | 0.099248442 |
| PINCR | 0.396323684 | 0.393263726 | NA | NS | 0.40531611 |
| NDP-AS1 | 0.542407609 | 0.325835812 | NA | NS | 0.487001185 |
| LINC01204 | 0.400785241 | 0.198401657 | 0.428291956 | NS | 0.702454705 |
| MIR222HG | -0.082308938 | 0.363549932 | 0.575717089 | NS | 0.439435932 |
| LINC02595 | 0.225891897 | 0.203950541 | 0.434096216 | NS | 0.690475139 |
| LINC01186 | 0.344316484 | 0.487410514 | NA | NS | 0.312105107 |
| ZNF674-AS1 | 0.030724267 | 0.713460372 | 0.830478881 | NS | 0.146630144 |
| LINC01545 | 0.211712009 | 0.115842741 | 0.334954857 | NS | 0.936131174 |
| INE1 | 0.022569262 | 0.861023668 | 0.921547579 | NS | 0.06498491 |
| LINC01560 | -0.062394058 | 0.504461214 | 0.685038287 | NS | 0.297172219 |
| UXT-AS1 | 0.152063429 | 0.519718717 | 0.696465656 | NS | 0.284231642 |
| ZNF630-AS1 | 0.222441979 | 0.424940199 | NA | NS | 0.371672183 |
| SYP-AS1 | -0.1452452 | 0.777159826 | NA | NS | 0.109489657 |
| USP27X-DT | -0.046506947 | 0.728222003 | 0.839178888 | NS | 0.137736203 |
| LINC01284 | 0.238827927 | 0.212844215 | 0.442658754 | NS | 0.67193815 |
| LINC01496 | 0.174903372 | 0.717477277 | NA | NS | 0.144191849 |
| KDM5C-IT1 | -0.004894193 | 0.97331676 | 0.984884922 | NS | 0.011745798 |
| LINC01278 | 0.125484634 | 0.29330918 | 0.515386052 | NS | 0.532674344 |
| SPIN4-AS1 | 0.631046919 | 0.119773158 | NA | NS | 0.921640498 |
| ARHGEF9-IT1 | 0.020549547 | 0.920895051 | NA | NS | 0.035789861 |
| MIR223HG | 0.167347659 | 0.211410439 | 0.441234413 | NS | 0.674873571 |
| LINC00269 | 0.338644991 | 0.562651441 | NA | NS | 0.249760564 |
| IGBP1-AS2 | 0.473798709 | 0.019921849 | NA | Up | 1.700670348 |
| IGBP1-AS1 | 0.09382097 | 0.566874142 | NA | NS | 0.246513353 |
| DLG3-AS1 | 0.091100651 | 0.745203661 | NA | NS | 0.12772502 |
| LINC00891 | 0.060701986 | 0.723654087 | 0.836984289 | NS | 0.140468981 |
| PHKA1-AS1 | 0.182738834 | 0.742914153 | NA | NS | 0.129061368 |
| TSIX | 0.138743478 | 0.578109907 | 0.738214279 | NS | 0.237989588 |
| XIST | 0.313286091 | 0.381096135 | 0.590918327 | NS | 0.418965456 |
| FTX | 0.12246784 | 0.154315825 | 0.381327257 | NS | 0.811589535 |
| JPX | 0.171504021 | 0.0354083 | 0.205922013 | Up | 1.450894924 |
| MIR325HG | 0.346382049 | 0.438542214 | NA | NS | 0.357988596 |
| PABPC5-AS1 | 0.83721066 | 0.165375136 | NA | NS | 0.781529786 |
| DIAPH2-AS1 | 0.284918008 | 0.036358732 | 0.208256151 | Up | 1.439391271 |
| TRMT2B-AS1 | 0.245922789 | 0.478252769 | NA | NS | 0.320342507 |
| ARMCX5-GPRASP2 | 0.009669155 | 0.896184998 | 0.941833188 | NS | 0.04760233 |
| LINC00630 | 0.027846147 | 0.730793924 | 0.840657112 | NS | 0.136205072 |
| TCEAL3-AS1 | 0.290424444 | 0.286561123 | NA | NS | 0.542782729 |
| MORF4L2-AS1 | 0.200685946 | 0.131743614 | 0.354444963 | NS | 0.880270426 |
| TMSB15B-AS1 | 0.093137783 | 0.367237078 | 0.579396578 | NS | 0.435053477 |
| AMMECR1-IT1 | 0.013937134 | 0.92247335 | 0.95733572 | NS | 0.035046171 |
| ALG13-AS1 | 0.101788803 | 0.507921024 | 0.687657088 | NS | 0.29420381 |
| XACT | 0.232797468 | 0.423211035 | 0.622593337 | NS | 0.373443017 |
| PLS3-AS1 | 0.276040778 | 0.601667404 | NA | NS | 0.220643516 |
| DANT2 | 0.274724535 | 0.45605392 | 0.647872405 | NS | 0.340983807 |
| DANT1 | 0.238093811 | 0.54766957 | NA | NS | 0.261481389 |
| LINC01285 | 0.367849917 | 0.453509685 | 0.646034128 | NS | 0.343413433 |
| SLC25A5-AS1 | -0.109841649 | 0.284570692 | 0.507573052 | NS | 0.54580983 |
| RHOXF1-AS1 | -0.146693336 | 0.530714146 | 0.704671084 | NS | 0.275139336 |
| LINC01402 | 0.42739265 | 0.239100991 | NA | NS | 0.621418623 |
| XIAP-AS1 | 0.00925343 | 0.959135427 | 0.977314449 | NS | 0.018120067 |
| LINC01201 | 0.555517235 | 0.328453695 | NA | NS | 0.483525848 |
| FIRRE | 0.101194943 | 0.50033049 | 0.682049966 | NS | 0.300743031 |
| RAP2C-AS1 | 0.158942233 | 0.055636111 | 0.248695897 | NS | 1.254643234 |
| HS6ST2-AS1 | 0.540314752 | 0.429369555 | NA | NS | 0.367168753 |
| MIR503HG | 0.470437233 | 0.048651591 | 0.233910643 | Up | 1.312902953 |
| LINC00629 | 0.37340095 | 0.337219365 | NA | NS | 0.472087494 |
| LINC02243 | 0.747902097 | 0.242926488 | NA | NS | 0.614525128 |
| SMIM10L2B-AS1 | -0.090147518 | 0.893897766 | NA | NS | 0.048712148 |
| INTS6L-AS1 | 0.930023179 | 0.054042046 | NA | NS | 1.267268217 |
| LINC00892 | 9.73E-04 | 0.993280368 | 0.996063884 | NS | 0.002928148 |
| FGF13-AS1 | 1.044054911 | 0.019139344 | NA | Up | 1.718072946 |
| LINC00632 | 0.488650202 | 0.217652577 | 0.447248377 | NS | 0.662236187 |
| SPANXA2-OT1 | 0.221232013 | 0.509463047 | 0.688645618 | NS | 0.292887311 |
| FMR1-AS1 | -0.062140709 | 0.837312281 | NA | NS | 0.077112539 |
| FMR1-IT1 | 0.141979036 | 0.272257043 | 0.497465358 | NS | 0.565020877 |
| AFF2-IT1 | 0.474206584 | 0.214330688 | NA | NS | 0.668915642 |
| LINC00893 | 0.126515182 | 0.305637412 | 0.526741519 | NS | 0.514793486 |
| LINC00850 | 0.953879933 | 0.1142278 | NA | NS | 0.942228188 |
| MAGEA8-AS1 | 0.354247985 | 0.435367046 | NA | NS | 0.361144447 |
| LINC00894 | 0.038562387 | 0.733081594 | 0.842188847 | NS | 0.134847685 |
| GPR50-AS1 | 0.749980108 | 0.234780197 | NA | NS | 0.629338537 |
| MAGEA4-AS1 | -0.057316876 | 0.920679447 | NA | NS | 0.035891552 |
| MAGEA10-MAGEA5 | 0.881245145 | 0.146886716 | NA | NS | 0.833017477 |
| MAGEA6-DT | 0.710270999 | 0.221456104 | NA | NS | 0.654712345 |
| LCA10 | 0.484217715 | 0.346392594 | NA | NS | 0.460431402 |
| HCFC1-AS1 | 0.126343856 | 0.539443576 | NA | NS | 0.268053974 |
| CH17-340M24.3 | -0.148226928 | 0.440430766 | NA | NS | 0.356122351 |
| TMLHE-AS1 | 0.149884661 | 0.192798446 | 0.421806458 | NS | 0.714896472 |
| ZFY-AS1 | 0.475656169 | 0.620923858 | NA | NS | 0.206961652 |
| LINC00278 | 0.661270407 | 0.594901953 | 0.750492703 | NS | 0.225554605 |
| LINC00279 | 1.521887794 | 0.36787249 | NA | NS | 0.434302688 |
| TTTY14 | 1.061009743 | 0.427945644 | 0.625834892 | NS | 0.36861139 |
| TTTY10 | 0.598769852 | 0.52075218 | 0.696709103 | NS | 0.283368904 |

**Supplementary Table 3.** Lists of the 33 hypoxia-related DEGs in GSE184050.

hypoxia-related DEGs

ENO1

JUN

PGM1

PDK1

ANKZF1

ACKR3

GPC1

HDLBP

VHL

CP

IER3

CDKN1A

IGFBP1

TES

CAV1

BNIP3L

GRHPR

KLF6

CHST3

P4HA1

DPYSL4

AMPD3

PRDX5

SLC37A4

TPI1

CSRP2

BTG1

SCARB1

TGFB3

JMJD6

ANGPTL4

ILVBL

GPI

PFKL

TPST2

LARGE1

KDELR3

IDS
